# Expanding the Knowledge on Lignocellulolytic and Redox Enzymes of Worker and Soldier Castes from the Lower Termite *Coptotermes gestroi*

**DOI:** 10.3389/fmicb.2016.01518

**Published:** 2016-10-13

**Authors:** João P. L. Franco Cairo, Marcelo F. Carazzolle, Flávia C. Leonardo, Luciana S. Mofatto, Lívia B. Brenelli, Thiago A. Gonçalves, Cristiane A. Uchima, Romênia R. Domingues, Thabata M. Alvarez, Robson Tramontina, Ramon O. Vidal, Fernando F. Costa, Ana M. Costa-Leonardo, Adriana F. Paes Leme, Gonçalo A. G. Pereira, Fabio M. Squina

**Affiliations:** ^1^Laboratório Nacional de Ciência e Tecnologia do Bioetanol (CTBE), Centro Nacional de Pesquisa em Energia e Materiais (CNPEM)Campinas, Brazil; ^2^Departamento de Bioquímica e Biologia Tecidual, Universidade Estadual de Campinas (UNICAMP)Campinas, Brazil; ^3^Laboratório de Genômica e Expressão, Universidade Estadual de Campinas (UNICAMP)Campinas, Brazil; ^4^Centro de Hematologia e Hemoterapia (Hemocentro), Universidade Estadual de Campinas (UNICAMP)Campinas, Brazil; ^5^Laboratório de Espectrometria de Massas, Laboratório Nacional de Biociências (LNBIO), Centro Nacional de Pesquisa em Energia e Materiais (CNPEM)Campinas, Brazil; ^6^Departamento de Biologia, Instituto de Biociências, Universidade Estadual Paulista (UNESP)Rio Claro, Brazil

**Keywords:** termites, carbohydrate-active enzymes, CAZy, auxiliary activity enzymes, second-generation biofuels, termite digestomes

## Abstract

Termites are considered one of the most efficient decomposers of lignocelluloses on Earth due to their ability to produce, along with its microbial symbionts, a repertoire of carbohydrate-active enzymes (CAZymes). Recently, a set of Pro-oxidant, Antioxidant, and Detoxification enzymes (PAD) were also correlated with the metabolism of carbohydrates and lignin in termites. The lower termite *Coptotermes gestroi* is considered the main urban pest in Brazil, causing damage to wood constructions. Recently, analysis of the enzymatic repertoire of *C. gestroi* unveiled the presence of different CAZymes. Because the gene profile of CAZy/PAD enzymes endogenously synthesized by *C. gestroi* and also by their symbiotic protists remains unclear, the aim of this study was to explore the eukaryotic repertoire of these enzymes in worker and soldier castes of *C. gestroi*. Our findings showed that worker and soldier castes present similar repertoires of CAZy/PAD enzymes, and also confirmed that endo-glucanases (GH9) and beta-glucosidases (GH1) were the most important glycoside hydrolase families related to lignocellulose degradation in both castes. Classical cellulases such as exo-glucanases (GH7) and endo-glucanases (GH5 and GH45), as well as classical xylanases (GH10 and GH11), were found in both castes only taxonomically related to protists, highlighting the importance of symbiosis in *C. gestroi*. Moreover, our analysis revealed the presence of Auxiliary Activity enzyme families (AAs), which could be related to lignin modifications in termite digestomes. In conclusion, this report expanded the knowledge on genes and proteins related to CAZy/PAD enzymes from worker and soldier castes of lower termites, revealing new potential enzyme candidates for second-generation biofuel processes.

## Introduction

Termites are social insects that play fundamental roles in carbon cycling in tropical forests, display characteristic labor division among castes and are highly efficient at lignocellulose degradation (Ohkuma, 2003; Hongoh, 2011). These insects infest cities worldwide, causing damage to wood structures and buildings as well as several billion dollars' worth of damages annually in the U.S.A. (Korb, 2007). Conversely, such efficient decomposers can be considered a good model to overcome challenges in the development of biotechnologies for the conversion of lignified plant biomass into feedstock sugars, which is now a main focus in the biofuels research field.

Termites live in colonies (self-organized systems) and are typically distributed into castes including workers, soldiers, a king, a queen and alates reproductives (Barsotti and Costa-Leonardo, 2005). The worker caste is responsible for feeding the colony and distributes the food, which is originally from lignocellulolytic materials, to other castes by stomodeal or proctodeal trophallaxis process. The soldier caste is accountable for colony defense against invaders and this caste is fed by worker caste via trophallaxis, thus they receive the food partially digested (Kitade, 2004). All these castes have a mutually beneficial symbiotic relationship (in their guts) with bacteria species and, in the case of lower termites, also with protists (Hongoh, 2011).

Termites are also considered one of the most efficient decomposers of lignocelluloses on Earth (Katsumata et al., 2007) and a better understanding of these organisms could lead to important technological improvements necessary to make the lignocellulose-to-biofuel conversion route more profitable (Warnecke et al., 2007; Franco Cairo et al., 2011; Sethi et al., 2013b; Scharf, 2015). Termites ingest plant biomass particles, reduce their sizes (20–10 μm; Fujita et al., 2010) and expose them to a repertoire of endogenous and symbiotic carbohydrate-active enzymes (CAZymes). This set of genes, enzymes and co-factors that termites and their gut symbionts produce to degrade lignocelluloses biomass is named the digestome (Scharf and Tartar, 2008).

The termite gut is generally divided into three main compartments: the foregut, midgut and hindgut. In the foregut, termites can secrete endogenous glycoside hydrolases, laccases, and putative esterases (Coy et al., 2010; Watanabe and Tokuda, 2010; Wheeler et al., 2010). The endogenous enzymes act in biomass depolymerization together with the milling action of a structure called the pro-ventricle or gizzard. In the midgut, some termite species are able to secrete specialized enzymes for biomass deconstruction (Fujita et al., 2010). The hindgut or fermentative chamber is the location of the symbionts. The great biodiversity in the hindgut is reflected in the wide taxa range of the resident microorganisms that includes mainly protists (in lower termites) and bacteria from the Bacteroidetes, Spirochaetes, and Firmicutes phyla (Warnecke et al., 2007), which play an important role in carbohydrate and nitrogen metabolism. The protist species can phagocytose and degrade lignocellulosic materials, while the endo- and ecto-symbiotic bacteria supply the protists and termites with nitrogen metabolites and acetate (Brune, 2014).

Recent studies on termite digestomes revealed several CAZymes, such as those of the glycoside hydrolase families (GH), including cellulases and hemicellulases, carbohydrate esterase families (CE) and auxiliary activities (AA), such as Laccases (Tartar et al., 2009; Coy et al., 2010; Wheeler et al., 2010). Moreover, the release of *Zootermopsis nevadensis* and *Macrotermes natalensis* genomes expanded the knowledge on CAZy genes on lower and higher termites (Poulsen et al., 2014; Terrapon et al., 2014). In spite of this large diversity of CAZymes, several studies have shown that termite endogenous glycosidases (GH9 and GH1) and symbiotic glycosidases (GH5, GH7, and GH45) had low activity against recalcitrant lignocellulose biomass (Fujita et al., 2010; Franco Cairo et al., 2013; Otagiri et al., 2013). Moreover, the enzymatic degradation and modifications of lignin that occur in termites have not been fully elucidated (Katsumata et al., 2007; Geib et al., 2008; Ke et al., 2012). Previous reports showed evidences that several enzymes related to Pro-oxidant, Antioxidant, and Detoxification process (herein abbreviated as PAD) could be related to termite digestome. Sethi et al. (2013a) reported that endogenous PAD enzymes, such as catalase and an aldo-keto reductase, could act in synergism with endogenous and symbiotic carbohydrate-active enzymes from lower termite *Coptotermes formosanus*. Thus, enzymes related to these processes could be a key to understand the high efficiency of termites in the degradation of lignocellulosic materials.

*Coptotermes gestroi* was previously classified as a lower termite, belonging to the Rhinotermitidae family, which was introduced in Brazil in the early years of the past century (Kirton and Brown, 2003). Nowadays, this specie is considered the main urban pest in Brazil, causing damage to buildings and wood constructions (Barsotti and Costa-Leonardo, 2005; Chouvenc et al., 2015). *C. gestroi* has protists and bacteria in its hindgut and it is capable to produce several carbohydrate-active enzymes, however, the occurrences of these enzymes were only reported throughout biochemical assays and proteomic data (Franco Cairo et al., 2011; Lucena et al., 2011). Moreover, CAZy genes in this termite specie were only described for endogenous endo-glucanase (*Cg*EG1-GH9) and β-glucosidase (*Cg*BG1-GH1) (Leonardo et al., 2011; Franco Cairo et al., 2013). Recently, a metagenomic approach in *C. gestroi*'s gut provided only insights in the CAZy gene repertoire for the free-living bacteria (Do et al., 2014), thus, the metatranscriptomic and the metaproteomic profile of CAZy and PAD enzymes endogenously synthesized by *C. gestroi* as well as by their symbiotic protists remains unclear.

Therefore, the aim of this work was to explore the repertoire of CAZy-PAD genes and enzymes in workers and soldier castes of the lower termite *C. gestroi*, focused on the transcripts and peptides produced by *C. gestroi* and its protists, both eukaryotic organisms. Thus, we expected that our report could provide knowledge to termite biology as well as targeting new enzymes for the development of second-generation biofuels process.

## Methods

### Termites

Specimens of *C. gestroi* (Wasmanm) were collected from field colonies with traps of corrugated cardboard and maintained in the Termite Laboratory of the Biology Department, UNESP, Rio Claro, São Paulo, Brazil (22° 23′S, 47°31′W). Termites were kept at 25 ± 2°C and fed with pine wood chips with 10% of humidity.

### RNA isolation and sequencing

Prior RNA extraction, termites were washed in saline solution (1% NaCl) to remove possible microorganism occurring in its exoskeleton. After, total RNA (10 μg) was extracted from the whole bodies of 50 workers and 50 soldiers using TRIZol reagent (Invitrogen) and purified using RNeasy Plant Mini Kit (Qiagen) under manufacture instructions. The quality of RNA was verified using RNAnano chip Bioanalyzer 2100 (Agilent). The cDNA from workers and soldiers were synthesized using Oligo-dT for mRNA enrichment (kit Superscript III RT™–Invitrogen) and sequenced using high throughput sequencing platform, under manufacture instructions, (GS FLX Titanium/Roche) generating single-end reads.

### Metatranscriptome assembly

The pyrograms from workers and soldiers were processed using the sff_extract program (MIRA Package) to convert the sff file into a fasta file and remove low-quality or adaptor sequence ends. The cdhit-454 program was used to eliminate identical and nearly identical (>98% identical) duplicates reads (Niu et al., 2010). Finally, the BDtrimmer program (Baudet and Dias, 2006) was used to perform the sequence trimming (poly A/T, adaptor and low-quality regions). The trimmed non-ribosomal reads larger than 100 bp were assembled into contigs and singlets using the MIRA EST sequence assembler version 3.0.3 (Chevreux et al., 1999) with the default parameters for 454 data.

Sequence data of 454 reads from soldier and worker libraries was submitted to SRA/NCBI under the accession number SRR1774237 and SRR1774239, respectively. This Transcriptome Shotgun Assembly project was deposited at DDBJ/EMBL/GenBank under the accession GCET00000000. The version described in this paper is the first version, GCET01000000.

### ESTs annotation and discovery of CAZy and PAD genes

The EST unigenes were BLASTed against the protein sequence database (NCBI/NR) using an *e*-value threshold of 1e^−5^ and classified into four taxonomic groups (insect, fungi, bacteria, and protist). The taxonomic classification was performed by comparing homologous organisms (identified in the first hit of the BLASTx/NR output for each unisequence) with a list of all organisms described in the insect, fungi, and bacteria groups extracted from the NCBI/taxonomy database. The protist unigenes were defined as containing one homologous organism in the taxonomic group of Parabasalia (taxid 5719) that was represented by more than 121,414 entries.

For the discovery of genes related to CAZy and PAD enzymes, HMM-based methodology was applied to identify these genes among the EST unigenes from both castes of *C. gestroi*. The sequences related to the carbohydrate-active enzymes were downloaded from the CAZy database. Since this database has been organized into proteins families, these CAZy protein families were aligned using clustalW (Larkin et al., 2007) with the default settings, and the HMM models were calculated and calibrated using hmmbuild and hmmcalibrate from the HMMER package (Finn et al., 2011). For PAD enzymes identification, the HMM models related to a specific Pfam of interest were downloaded from Pfam database and used to searches. The list of Pfams used to categorize the classes of PAD genes was previously described in the literature (Tartar et al., 2009; Sethi et al., 2013b). Thus, the identifications of unigenes, correlated with CAZy and PAD enzymes, were performed by comparing the total EST unigenes and HMM models using hmm-search with an *e*-value cut-off of 1e^−5^, and a configuration appropriate for use with parameters of local searches. Additionally, the EST unigenes identified as CAZy and PAD enzymes were compared to the Pfam database using the RPS-BLAST program with an *e*-value threshold of 1e^−5^ to confirm our methodology. The dbCAN or CAT databases (Park et al., 2010; Yin et al., 2012) could be used for the identification of CAZymes, however both lack the PAD sequences. After the identification and annotation of CAZy and PAD unigenes, a read counting for each Pfam domain was performed and also classified based on their taxonomy origins in both castes.

### Phylogenetic analysis of symbiotic CAZymes

The proteins from GH5, GH7, GH10, and GH11 families that contain sequence similarities with non-insect organisms were submitted for phylogenetic analysis. Firstly, these protein sequences were aligned against non-protein redundant (NR) database from NCBI using BLASTp program and top 5 protein hits for every query were downloaded. For each protein family, a FASTA file containing non-insect proteins and all blast hits were submitted for global alignments among amino acid sequences using MUSCLE (Edgar, 2004). The selection of amino acid substitution models was done using Akaike criteria implemented in maximum likelihood analysis on MEGA version 6 (Tamura et al., 2013). The phylogeny was reconstructed using Maximum Likelihood analysis implemented on RAxML (Stamatakis, 2014) with branch support estimated by 1000 bootstraps.

### Mass spectrometry analyses

The protein was extracted from the whole bodies of 50 workers and 50 soldiers as previously described (Franco Cairo et al., 2011). The protein extract (75 μg) from each caste was loaded into a 12% SDS-PAGE gel and bands at 19, 26, 34, 50, and 90 kDa and above 90 kDa were excised, reduced (5 mM dithiothreitol, 25 min at 56°C), alkylated (14 mM iodoacetamide, 30 min at room temperature in the dark), and digested with trypsin (Promega). The samples were dried in a vacuum concentrator and reconstituted in 50 μL of 0.1% formic acid to extract the peptides from the gel. The supernatant was transferred to new tubes and 4.5 μL of the resulting peptide mixture was analyzed on an ETD-enabled LTQ Velos Orbitrap mass spectrometer (Thermo Fisher Scientific) coupled with LC-MS/MS by an EASY-nLC system (Proxeon Biosystems) through a Proxeon nanoelectrospray ion source. The peptides were separated by a 2–90% acetonitrile gradient in 0.1% formic acid using a PicoFrit Column analytical column (20 cm × ID75 μm, 5 μm particle size, New objective) at a flow rate of 300 nL/min over 27 min. The nanoelectrospray voltage was set to 2.5 kV, and the source temperature was 200°C. All instrument methods for the LTQ Velos Orbitrap were set up in the data-dependent acquisition mode. The full scan MS spectra (m/z 300–1600) were acquired in the Orbitrap analyzer after accumulation to a target value of 1e^6^. The resolution in the Orbitrap was set to *r* = 60,000, and the 20 most intense peptide ions with charge states ≥2 were sequentially isolated to a target value of 5000 and fragmented in the linear ion trap by low-energy Collision-Induced Dissociation–CID (normalized collision energy of 35%). The signal threshold for triggering an MS/MS event was set to 1000 counts. Dynamic exclusion was enabled with an exclusion size list of 500, exclusion duration of 60 s, and repeat count of 1. An activation q of 0.25 and an activation time of 10 ms were used.

The spectra were acquired using the software MassLynx v.4.1 (Waters - Milford, MA, USA), and the raw data files were converted to a peak list format (mgf) without summing the scans using the Mascot Distiller v.2.3.2.0 software (Matrix Science Ltd.). These spectra were searched against the *C. gestroi* database (181,554 unigenes; 42,520,001 residues—generated by the unigenes identified in the metatranscriptomic analysis described above) using the Mascot v.2.3.01 engine (Matrix Science Ltd.) with carbamidomethylation as the fixed modification, oxidation of methionine as a variable modification, one trypsin missed cleavage and a tolerance of 10 ppm for precursor ions and 1 Da for fragment ions.

All datasets processed using the workflow feature in the Mascot software were further analyzed in the software ScaffoldQ+ to validate the MS/MS-based peptide and protein identifications. Peptide identifications were accepted if they could be established at greater than 60.0% probability as specified by the Peptide Prophet algorithm (Keller et al., 2002). Peptide identifications were also required to exceed specific database search engine thresholds. Mascot identifications required at least both the associated identity scores and ion scores to be greater than 31. Protein identifications were accepted if they could be established at greater than 80.0% probability for peptide identification. Protein probabilities were assigned using the Protein Prophet algorithm (Nesvizhskii and Aebersold, 2004). Proteins that contained similar peptides and could not be differentiated based on the MS/MS analysis alone were grouped to satisfy the principles of parsimony. The scoring parameter (Peptide Probability) in the ScaffoldQ+ software was set to obtain a false discovery rate (FDR) of less than 2%. Using the number of total spectra output from the ScaffoldQ+ software, we identified the differentially expressed proteins using spectral counting. A normalization criterion, the “quantitative value,” was applied to normalize the spectral counts. All mass spectrometric raw file associated with this study is available for download via FTP from the PeptideAtlas data repository by accessing the following link: http://www.peptideatlas.org/PASS/00574.

### Biochemical analysis

Biochemical assays using the whole worker and soldier crude extracts were performed to evaluate the ability of this extract to hydrolyze natural polysaccharides and synthetics oligosaccharides. Total protein extractions for biochemical characterization was prepared from 100 whole bodies of worker and soldier. They were homogenized using Harvest-Potter with 2 ml of 50 mM sodium acetate buffer, pH 5.5. After extraction, the mixture was centrifuged at 20.100 × *g* for 20 min at 4°C, followed by addition of 1 μl of Cocktail Protease Inhibitor (Amresco) per ml of crude extract produced. The supernatant was collected and hereafter referred such as crude enzyme extract. For all assays, the protein concentration used was of 1 μg/μl and the concentration was determined for Bradford method (Bradford, 1976). All procedures were performed on ice. The assays were performed as previously described by Franco Cairo et al. (2011) with slight modifications. Enzymatic reactions consisted of 10 μl of crude enzyme extract incubated with 40 μl of 50 mM sodium acetate buffer pH 5.5 and 50 μl of 0.5% specific substrate (in water), in triplicate, at 37°C, for 60 min. Enzymatic assays were stopped after the addition of 100 μl of dinitrosalicylic acid–DNS (Miller, 1959) and heated at 99°C for 5 min. The measurement of color change was performed at 540 nm using a micro plate reader. The enzymatic activity assays results were expressed in mM of glucose equivalents produced. The enzymatic assays with *p*-nitrophenyl-carbohydrates (*p*NP) were performed as follows: 10 μl of crude extracts were incubated with 50 μl of 5 mM *p*NP substrates and 40 μl of 50 mM sodium acetate buffer pH 5.5. Assays were stopped after addition of 100 μl of 1 M Sodium Carbonate_._(Na_2_Co_3_). The measurement of color change was performed at 400 nm using a micro plate reader. The enzymatic assays were done in triplicates and results were expressed in terms mM of *p*-nitrophenyl released. Glucose and *p*-nitrophenyl were used for standard curve construction. Substrates were purchased from Megazyme and Sigma Aldrich: CMC (carboxymethyl cellulose); β-glucan from barley, lichenan from moss, laminarin from *Laminaria digitata*, xyloglucan from tamarind, xylan from oat spelt, rye arabinoxylan, mannan, pectin from *Citrus*; 4-nitrophenyl β-D-glucopyranoside (*p*NP-G); 4-nitrophenyl β-D-cellobioside (*p*NP-C); 4-nitrophenyl β-D-xylopyranoside (*p*NP-X); 4-nitrophenyl β-D-galactopyranoside (*p*NP-Gal); 4-nitrophenyl α-L-arabinofuranoside (*p*NP-A); 4-nitrophenyl β-D-mannopyranoside (*p*NP-M); 4-nitrophenyl-α-L-fucopyranoside (*p*NP-F).

## Results

### Metatranscriptomic sequencing and overview analyses of *C. gestroi* ESTs

**Figure 1:**
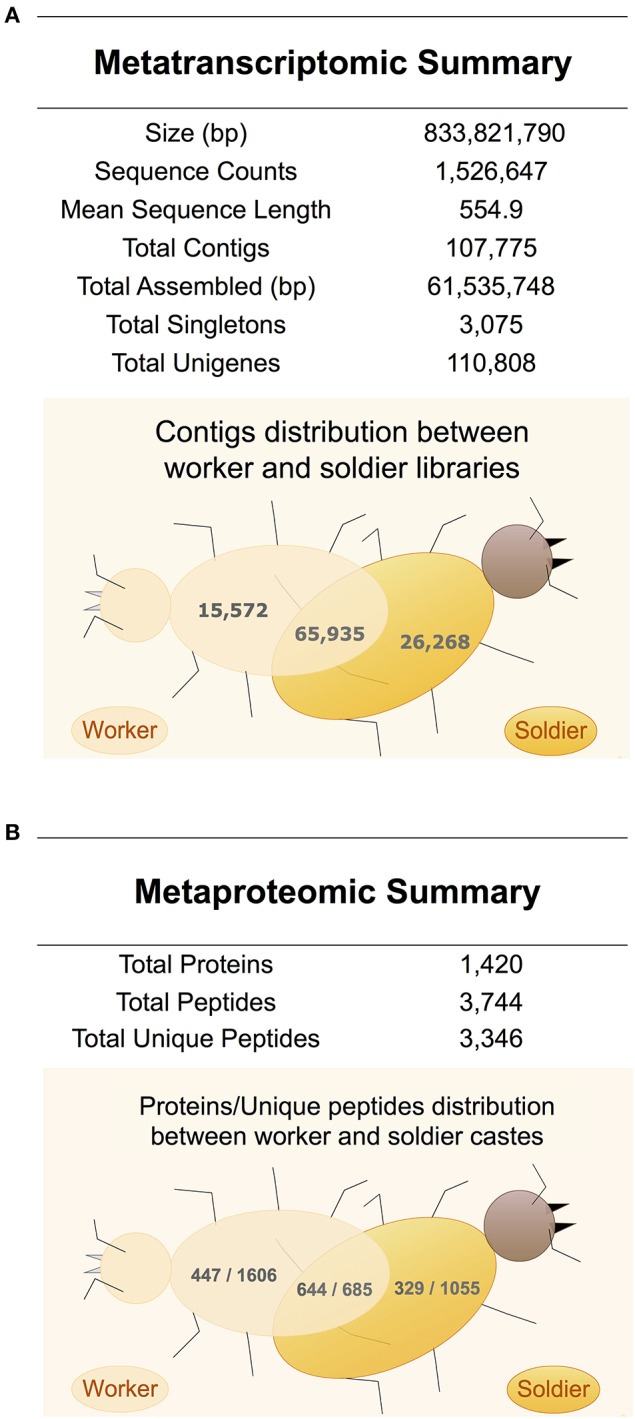
**Metaproteotranscriptomic overview of ***C. gestroi*** castes**. **(A)** Metatranscriptomic summary and Venn diagram showing contigs distribution between worker and soldier castes. It was generated 1,526,647 reads and a total of 833,821,790 base pairs were sequenced. After trimming low-quality sequences, adaptors and removing the ribosomal RNA sequences, a total of 335,965 and 393,961 high quality expressed sequence tags (ESTs) were obtained from the worker and soldier libraries, respectively. MIRA package was applied for assembling, which it generated 107,775 contigs and 3,045 singlets grouped into 110,881 unigenes with a total of 61,535,748 base pairs. **(B)** Metaproteomic summary and Venn diagram showing proteins/unique peptides distribution between worker and soldier castes.

Two cDNA libraries using oligo-dT were constructed from whole bodies of specimens of worker and soldier castes of *C. gestroi* without replicates aiming to perform only gene discovery of CAZy and PAD enzymes. The libraries were sequenced using the GS FLX Titanium system from Roche, producing approximately 800,000 single-ends reads for each library. All sequence data is summarized in Figure 1A. After trimming, high-quality expressed sequence tags (ESTs) were obtained from worker and soldier libraries. The MIRA EST sequence assembler configured with the default parameters for 454 data was used with success to perform the transcriptome assembly. The majority of contigs were shared by both castes (Figure 1A and Table S1). Moreover, the workflow applied in sequencing data analysis was also described (Figure S1).

**Figure 2:**
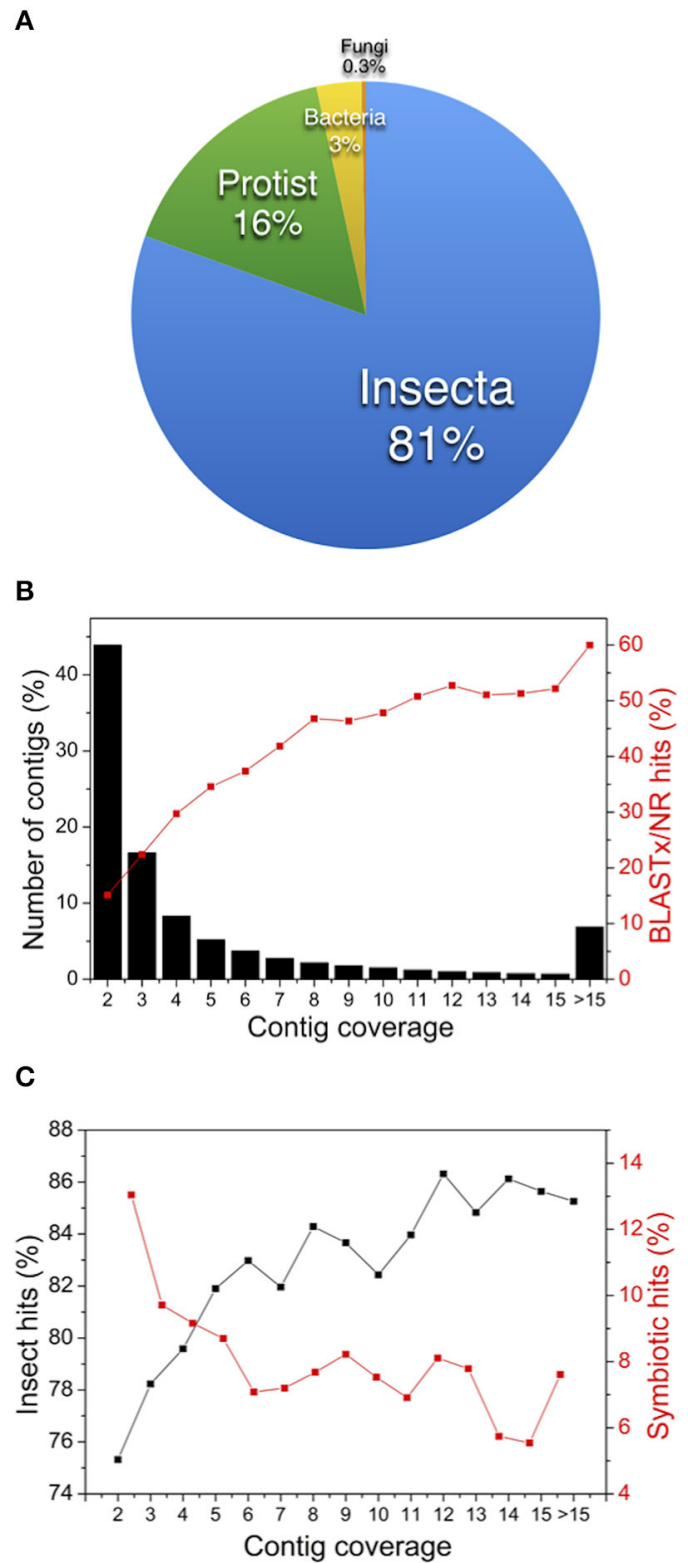
**Analyses of ***C. gestroi*** metatranscriptome. (A)** The taxonomic distribution of *C. gestroi* unigenes. **(B)** The distribution of the number of contigs (black bars) and BLASTx/NR hits (red line) as a function of the contig coverage. **(C)** The distribution of insect hits (black line) and symbiont hits (red line) as a function of the contig coverage.

The unigene annotation using BLASTx against NCBI/NR was performed and could assign protein hits for only 28.8% of the unigenes (Table S1). Based on homology searches using BLASTx against NR, the unigenes could be classified into insect, protist, bacteria, and fungi taxonomic groups (Figure 2A). As expected, most of the proteins were of insect group, but there was a contribution of protists and fungi organisms, as it was expected due to symbiotic organisms that inhabit the termite gut. Although, poly-A enrichment step was used, unigenes classified into bacteria group were also identified (around 3%). In addition, other taxonomic groups were identified, including *Branchiostoma, Hydra, Strongylocentrotus, Ixodes*, and some other genera (data no shown).

Although, a high number of unigenes were identified based on comparisons with other insect transcriptome projects available at the Gene Index Project website (Quackenbush et al., 2001; approximately 25,000 and 55,000 unique sequences from *Apis mellifera* and *Drosophila melanogaster* were identified, respectively), more detailed analyses of the sequence data were carried out. We further evaluated the number of contigs with BLASTx/NR hits as a function of the contig coverage and analyzed the ratios of the insect and symbiont hits found in these contigs. The distribution of reads/contigs revealed a high number of contigs that were assembled based only on a few reads, e.g., 48% of contigs were assembled based only on two reads (Figure 2B). In the same figure, the red line shows that the number of unigenes of symbiotic origin that showed similarity to known proteins (BLASTx/NR) increased as a function of the contig coverage, and only 15% of the unigenes assembled based on two reads resulted in hits to known proteins.

Another interesting result was observed when the percentage of insect and symbiotic protein hits was plotted as a function of the contig coverage (Figure 2C). For instance, almost 27% of the contigs assembled and based on two reads were from symbiotic organisms, and this number decreased as a function of the contig coverage. These results suggested that contigs assembled based on a few reads (2, 3, or 4 reads) mostly represent unknown proteins or non-coding RNAs that were probably highly expressed in symbiotic organisms, which were likely represented at low cell densities.

### Metatranscriptomic-driven identification of CAZy and PAD unigenes from castes of *C. gestroi*

The metatranscriptomic analysis of workers and soldiers from the lower termite *C. gestroi* has revealed a total of 778 unigenes containing Pfam domains assigned to CAZy and PAD enzymes. The results revealed unigenes from 32 different glycoside hydrolases families (GH), 8 Auxiliary Activities enzymes subfamilies (AA), 8 carbohydrate esterase families (CE), 2 polysaccharide lyase families (PL), and 5 classes of PAD.

**Figure 3:**
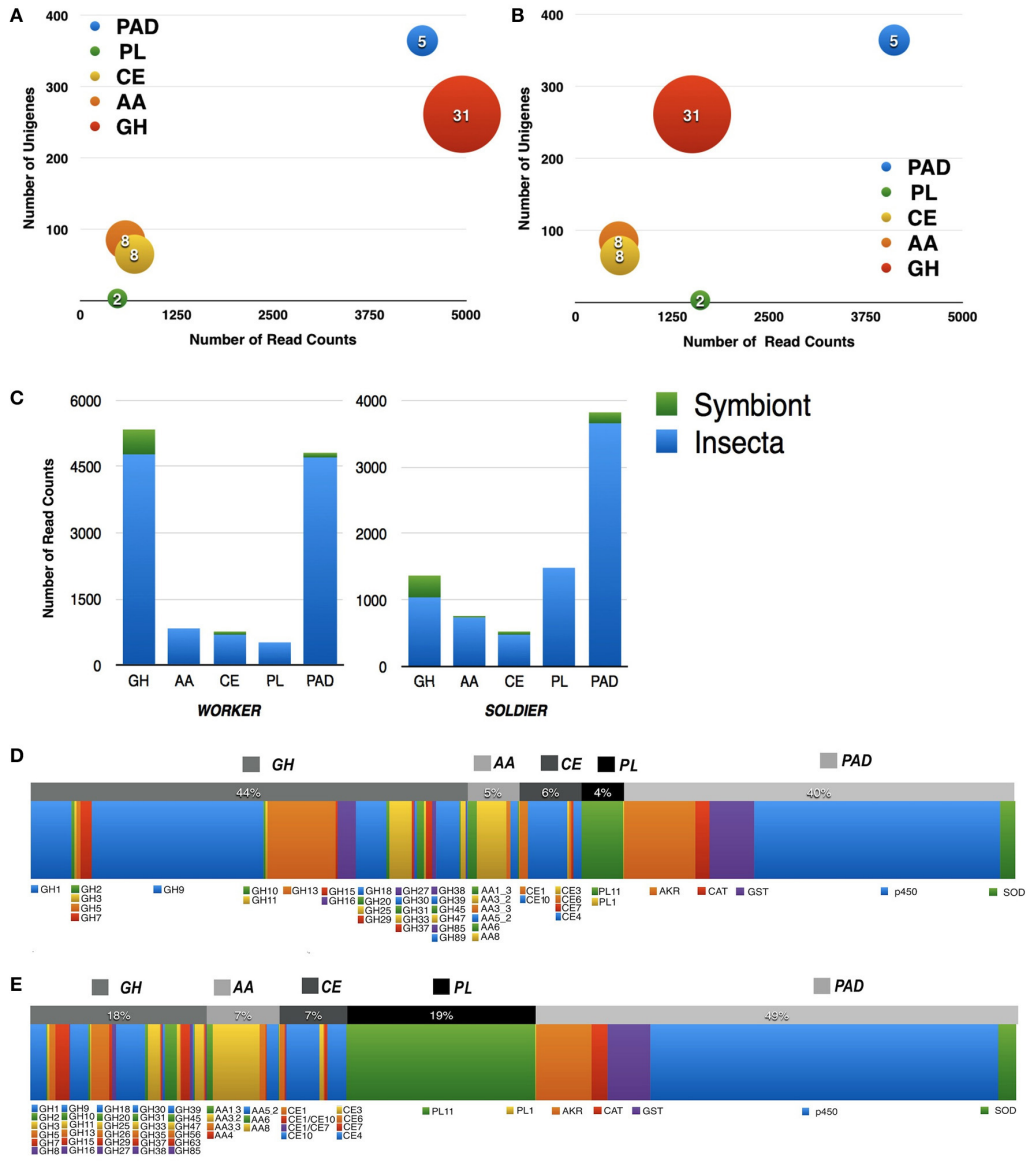
**Summary of CAZy and PAD enzymes identified in ***C. gestroi*** metatranscriptome**. Bubble chart from worker **(A)** and soldier **(B)** castes showing the number of unigenes and the number of reads identified as CAZy and PAD components. The size of the bubbles is proportional to the number of different families assigned to each group. GHF, Glycoside Hydrolase Families; AA, Auxiliary Activity Families; CE, Carbohydrate Esterase Families; and PL, Polysaccharide Lyase Families; PAD, Pro-oxidant/Antioxidant and Detoxification enzymes. **(C)** Taxonomic distribution of Cazy and PAD reads from worker and soldier. Symbiont = protist. The distribution of reads with similarity to CAZy families and PAD enzymes of worker **(D)** and soldier **(E)** metatranscriptome is shown.

The glycoside hydrolases and PADs were the most represented unigenes in both castes (Figures 3A,B). The majority of these unigenes were homologs to insect sequences, however, there were a counterpart of sequences with homology to symbionts (Figure 3C), mainly from protists. Figures 3D,E show the distribution of reads throughout the CAZy families and classes of PAD for worker and soldier, respectively. Table 1 describes in detail the distribution of CAZy and PADs enzymes identified by our analysis, along with their respective Pfams and taxonomic origin, such as insect, protist and bacteria. Although, the analysis was based on sequences derived from poly-A (for eukaryotic organisms), the bacterial sequences discovered in this analysis were maintained in the results due to their representative information for the main objective of this work, which was the discovery of CAZy and PAD genes in *C. gestroi* castes. No fungi sequences related to CAZy and PAD enzymes were found.

**Table 1:** **CAZy and PAD enzymes identified in metatranscriptomic from worker and soldier castes of ***C. gestroi*****.

	**Pfam Domains**	**Unigene Counts for Insect**	**Unigene Counts for Protists**	**Unigene Counts for Bacteria**	**Unigenes with Secretion Signals**	**Number of Worker Reads**			**Number of Soldier Reads**		
						**Insect**	**Protist**	**Bacteria**	**Insect**	**Protist**	**Bacteria**
**GLYCOSIDE HYDROLASES**											
GH1	Glyco_hydro_1	19	5		11	435	30		110	30	
GH2	Glyco_hydro_2	4			4	39			8		
GH3	Glyco_hydro_3			4	nd			23			12
GH5	Cellulase		7	4	1		22	18		38	14
GH7	Glyco_hydro_7		18		nd		125			119	
GH8	Glyco_hydro_8			1	nd			7			9
GH9	Glyco_hydro_9	41			4	1941			153		
GH10	Glyco_hydro_10		3		nd		26			19	
GH11	Glyco_hydro_11		5		nd		24			12	
GH13	Alpha-amylase	20			10	770			151		
GH15	Glyco_hydro_15	7			3	23			24		
GH16	Glyco_hydro_16	6			5	200			33		
GH18	Glyco_hydro_18	23	3		14	333	18		232	14	
GH20	Glyco_hydro_20	5	1		3	29	2		23	2	
GH25	Lys	9	8		6	59	194		30	79	
GH26	Glyco_hydro_26			2	1			5			7
GH29	Alpha_L_fucos	1			1	21			6		
GH27	Melibiase	2			2	11			10		
GH30	Glyco_hydro_30	3			1	16			12		
GH31	Glyco_hydro_31	8	2		2	78	7		97	5	
GH31	Alpha-amylase/Cellulase	2			nd	2			2		
GH33	BNR	1			1	20			31		
GH35	Glyco_hydro_35	2			nd	6			5		
GH37	Trehalase	9			4	69			78		
GH38	Glyco_hydro_38/Alpha-mann_mid	6			2	41			27		
GH39	Glyco_hydro_39	1	1		1	267	2		9	2	
GH45	Glyco_hydro_45		1		nd		5			5	
GH47	Glyco_hydro_47	14	1		2	44	11		60	10	
GH56	Glyco_hydro_56	1			nd	2			3		
GH63	Glyco_hydro_63	3			nd	5			10		
GH85	Glyco_hydro_85	1	2		1	2	9		3	4	
GH89	NAGLU	3			1	6			7		
	TOTAL (Ins+Prot+Bact)	261			80	4419	475	53	1124	339	42
**AUXILIARY ACTIVITIES**											
AA1_3	Cu_Oxidase_2 - PF007731	14			9	105			57		
AA2	Peroxidase - PF00141	nd			−						
AA3_1	GMC_oxred_N - PF00732	nd			−						
AA3_2	GMC_oxred_C - PF05199	40	3		20	323	8		390	11	
AA3_3	GMC_oxred_C - PF05199	15			7	45			50		
AA4	FAD-oxidase_C - PF02913	1			nd	6			6		
AA5_1	Glyoxal_oxid_N /Ald_Xan_dh_C		1		nd		3			3	
AA5_2	F5_F8_type_C - PF00754	7	1		1	73	10		96	2	
AA6	FMN_red - PF03358	2			nd	10			8		
AA8	GMC_oxred_C - PF05199	1			1	2			2		
	TOTAL (Ins+Prot+Bact)	85			38	564	21		552	5	
**CARBOHYDRATE ESTERASE**											
CE1	Abhydrolase_2	6	1		2	74	15		35	11	
CE1/CE10	Abhydrolase_2/Coesterase	2			nd	3			5		
CE1/CE7	Abhydrolase_2/ XE1	4			nd	3			7		
CE10	COesterase	37			20	448			287		
CE13	PectinaCEtylesterase	1			1	6			1		
CE3	PAF_aCEtylesterase_like	1	2	1	nd	1	18	2	3	23	2
CE6	AXE1/Glyco_hydro_10		2		nd		19			13	
CE7	AXE1	2		1	1	21		2	14		7
CE14	PIG-L	1			nd	3			2		
CE4	Polysacc_deac_1	4			2	88			162		
	TOTAL (Ins+Prot+Bact)	65			26	647	52	4	516	47	9
**POLYSACCHARIDE LYASE**											
PL11	fn3_3	2			2	475			1608		
PL1	Pec_Lyase_C			1	nd			6			2
	TOTAL (Ins+Prot+Bact)	3			2	475		6	1608		2
**PAD ENZYMES**											
Aldo-Keto Reductase	Ald_Ket_Red	24	2		6	796	11		482	1	
Catalase	Catalase	10	5		3	144	21		113	27	
Glutathione S-Transferase	GST_N	39	3		13	457	42		335	31	
p450	p450	244	12		72	2756	31		2889	80	
Superoxide Dismutase	Sod_Cu	18	7		5	154	22		129	28	
	TOTAL (Ins+Prot+Bact)	364			99	4307	127		3948	167	

Among the GHs, the predominant family in the worker caste was GH9, followed by GH13, GH1, GH18, GH39, GH25, GH16, and GH7. In soldier caste, the most abundant family was GH18, followed by GH9, GH1, GH13, GH7, and GH25. From these abundant families, GH9, GH39, GH16, and GH7 harbor typical members displaying enzymatic activity related to carbohydrates from plant cell wall. Regarding the unigenes related to cellulose degradation, endo-glucanases (GH5, GH9, and GH45), and beta-glucosidases (GH1) sequences were reported to insect, protist and bacteria taxonomic groups. Exo-glucanases (GH7) were reported only of protist origin.

The GH families found in both worker and soldier libraries involved with hemicellulose degradation were GH3, GH8, GH10, GH11, GH16, GH26, GH29, and GH31. Classical xylanases (GH10 and GH11) were classified only with protist origin. Beta-glucosidases/xylosidases (GH3), lichenases (GH8), and endo-mannanases (GH26) were classified with bacteria origin. Laminarases (GH16) and alpha-fucosidases (GH29) were only of insect origin and alpha-glucosidase/xylosidase (GH31) was assigned to insect and protist taxonomic origin. However, for instance, a termite enzyme classified as GH16 was previous described as Gram-negative bacteria-bind protein (DGNBP) in *C. formosanus* (Hussain et al., 2013).

Regarding the auxiliary activities enzymes, members of the families/subfamilies AA1_3, AA3_2, AA3_3, AA4, AA5_1, AA5_2, AA6, and AA8 were found in worker and soldier libraries. AA3_2 was the most abundant subfamily in both workers and soldiers, followed by AA3_3 and AA1_3. The sequences of AA1_3, AA3_3, and AA4 were only of insect origin, while AA3_2 sequences were both of insect and protist origin. The AA3 family contains *GMC_ oxidoreductase* domain (PF00732), which catalyzes the oxidation/reduction of glucose/alcohol/pyranose and generation of hydrogen peroxide as product. The literature has suggested this reactive oxygen specie as a co-factor for lignin peroxidases and Fenton reaction applied for lignocellulose breakdown (Levasseur et al., 2013). On the other hand, the ecdysone oxidases from insect *Bombyx mori* play a role in cuticle formation rather than lignocellulose degradation and this enzyme contains *GMC* domain (Sun et al., 2012).

The AA1_3 subfamily contains a *Cu2_oxidase* pfam domain (PF00394), which is also found in multi-copper laccases. AA5_1 and AA5_2 family members were also found from both castes, but sequences of protist origin were only identified for AA5_1 subfamily and for AA5_2 both insect and protist sequences were detected. The sequences from AA3_2, AA3_3, AA4, AA5_1, and AA5_2 are part of a family that contains glucose, alcohol, vanillyl-alcohol, glyoxal, and aldehyde oxidases also involved in hydrogen peroxide generation and lignin oxidation (Levasseur et al., 2013). Interestingly, AA6 and AA8 reads were identified of worker and soldier only with insect origin. These AAs feature protein domain are known to be involved in the generation of Fenton components and iron reduction, respectively (Arantes et al., 2012; Levasseur et al., 2013). Sequences related to the AA2 and AA3_1 families, which contains lignin peroxidases and cellobiose dehydrogenases respectively as well as for the families AA9, AA10, AA11, and AA13 classified as Lytic Polysaccharide Monoxygenases-LPMOs (Levasseur et al., 2013) were not identified in this study.

The predominant CE family found in our analysis was CE10, followed by CE4 and CE1. The identified CE10 and CE4 reads were only of insect origin, whilst CE1 reads were of both insect and protist origins. The unigenes from CE3 and CE6 families were only of protist origin. CE10 family is reported to contain only genes/proteins that are not involved with carbohydrate modifications. Wheeler et al. (2010) suggested that proteins with *COesterase* domain (PF00135), for instance members of CE10, could act as feruloyl esterases in termites. Otherwise, the families CE1, CE3, CE4, and CE6 are described as acetyl xylan esterases, directly involved in hemicelluloses modifications. Another class of CAZymes found in our analysis was PL (polysaccharide lyases), but only two families of this class were identified in this study, the PL11, a rhamnogalacturonan lyase, and PL1, a pectate lyase (Cantarel et al., 2009). The most abundant family was PL11, which had only sequences of insect origin.

Among the PAD components, the predominant class identified was p450, followed by aldo-keto reductase (AKR), glutathione transferase (GST), superoxide dismutase (SOD), and catalase (CAT). The reads classified as p450 members were predicted to be of insect and protist origin. The AKR reads in both castes also assigned with unigenes from insect and protist. In the case of the GST class, the results were similar to those for AKR. For the SOD and CAT classes, the results showed that both classes had sequences of insect and protist origins.

Considering that whole termite bodies were used for RNA extraction, it is important to mention that not all enzymes should be considered as components of the termite digestome. In order to investigate potential involvement in the termite digestome, further analysis to identify secretion signals was performed on all the unigenes classified as CAZy and PAD. As a result, our bioinformatic analysis suggested that the majority of unigenes from CAZy families and all PAD classes identified by metatranscriptomics display unigenes predicted with secretion signals (Table 1, Tables S2, S3).

Secretion signals were identified in glycoside hydrolase unigenes related to cellulose degradation (such as GH1, GH9, GH16), mannan degradation (such as GH2) and AA members (families AA1_3, AA3_2, AA3_3, AA5_2, and AA8) related to cellulose and lignin oxidation. Unigenes from CE families, which are directly involved in biomass degradation, such as CE1, CE4, and CE13, as well as PL11, were predicted with secretion signals (Table 1). As expected, all unigenes from protists indicated as cellulolytic enzymes, such as those from families GH5, GH7, and GH45 and the hemicellulolytic enzymes from GH10 and GH11, did not present secretion signals (Table 1, Table S3), since the degradation of lignocelluloses by protists has been reported to occur in phagocytic vesicles in the cytoplasm of these microorganisms.

### Phylogenetic analyses of symbiotic CAZymes

To get further insights into the diversity and taxonomic origin of symbiotic CAZymes, phylogenetic analyses were performed with enzymes classified as members of GH5, GH7, GH10, and GH11 families (Figures S2, S3). The sequences coding endo-glucanases from family GH5 were grouped into three different clades (Figure S2A). The first clade is represented by protist organisms, grouping uncultured protists from different termite species and the protist *Spirotrichonympha leidyi*, suggesting the occurrence of this protist specie in *C. gestroi* guts. Bacterial organisms from Firmicutes and Bacteriodetes phylum represent the second and third clades, respectivelly. Collectively, these results revealed the substantial diversity of GH5 endo-cellulases in *C. gestroi*.

For the exo-glucanases from GH7 family, the phylogenetic tree for GH7 enzymes generated two distinct clades highly supported by bootstrapping values bigger than 90% (Figure S2B). Protist organisms represent the first clade that grouped a subset of GH7 sequences identified in this work with GH7 enzymes from *Holomastigotoides mirabile* and *Pseudotrichonympha grassi*, two major protist species described for *Coptotemes* genus. The second clade grouped set of GH7 sequences with fungal organisms.

The xylanases from GH10 (Figure S3A) and GH11 (Figure S3B) families identified in this work were grouped with xylanases from protist origin, separating them from GH10 and GH11 sequences of bacterial origin. Interestingly, protist's proteins from GH10 family clustered in a specific clade only containing sequences from a uncultured protist from *C. gestroi* (Figure S3A), while the other GH10 sequences clustered in individual clades, belonging to other termite species such as *C. formosanus* and *Neotermes koshunensis*. In the case of protist's proteins from family GH11, they grouped together with sequences of xylanases GH11 from the protist *Holomastigotoides mirabilis*, suggesting the origin of these sequences, as well, as the occurrence of this specie in *C. gestroi* guts.

### Metaproteomic-driven identification of CAZy and PAD enzymes from *C. gestroi* castes

Metaproteomic analysis was also performed to elucidate the profile of carbohydrate-active and PAD enzymes in the lower termite *C. gestroi*. Mass spectrometry-based proteomics was used, which is a consolidated method applied for metaproteomics (Burnum et al., 2011; Franco Cairo et al., 2011). The summary of the metaproteomic results of worker and soldier castes is provided in Figure 1B. In total, 1,420 proteins were identified in the metaproteome of *C. gestroi* (identified from 3,744 total peptides in both castes), applying the criterion of one unique peptide with a 2.0% False Discovery Rate (FDR) (Table S4). The use of one unique peptide in the metaproteomic analysis is acceptable in literature only if the FDR is below 5.0% (Tanca et al., 2013; Tang et al., 2014). Moreover, the number of identified proteins represented 1.3% of the predicted unigenes in the metatranscriptomic analysis.

**Figure 4:**
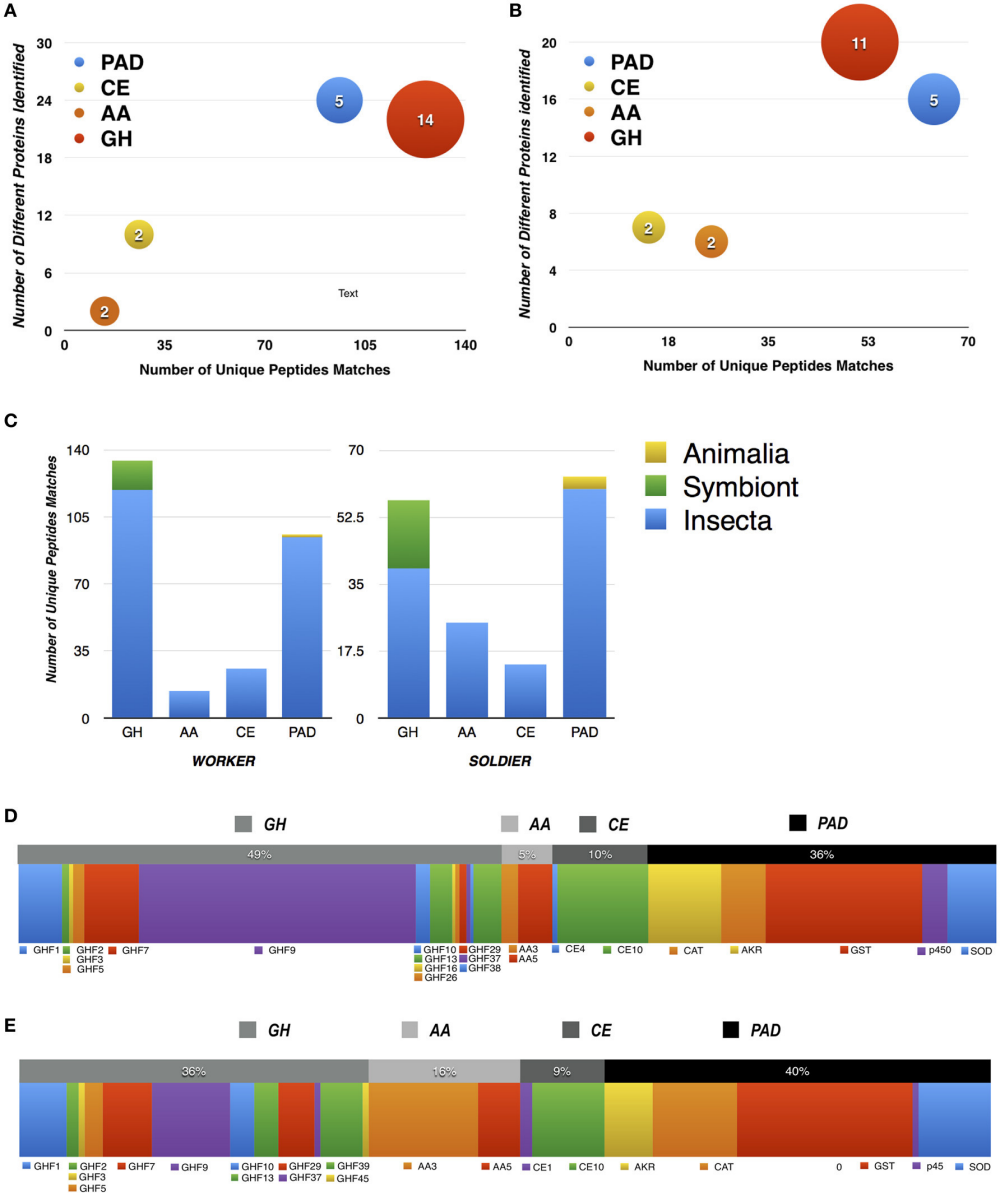
**Summary of CAZy and PAD enzymes identified in ***C. gestroi*** metaproteome**. Bubble chart from worker **(A)** and soldier **(B)** castes showing the number of proteins and the number of peptides identified as CAZy and PAD components. The number inside and the size of the bubble represent the number of different families (Cazy) and domains (Pfam) found in metaproteomic analysis, respectively. GHF, Glycoside Hydrolase Families; AA, Auxiliary Activities Families; CE, Carbohydrate Esterase Families and PL, Polysaccharide Lyase Families; PAD, Pro-oxidant/Antioxidant and Detoxification enzymes. **(C)** Taxonomic distribution of Cazy and PAD peptides in worker and soldier - symbiont = protist and bacteria. The distribution of peptides with similarity to CAZy and PAD enzymes of worker **(D)** and soldier **(E)** metaproteome is shown.

Performing the analysis based on the criterion of one unique peptide, 433 peptide matches for CAZy and PAD enzymes were identified. These peptide matches were distributed across 73 different proteins: 28 classified as GH families, 27 as PAD, 6 as AA families, and 12 as CE (Figures 4A,B), moreover, the majority of these peptides were of insect origin (Figure 4C). The distribution of the peptides throughout taxonomic origin as well as the CAZymes families and PAD enzymes are also described in Table 2. These results are in agreement with the metatranscriptomic data, confirming that the GHs and PAD enzymes were highly abundant (based on spectrum counts) in both castes of *C. gestroi*, as well as, that the majority of the protein matches are of insect origin (Figures 3C, 4C).

**Table 2:** **CAZy and PAD enzymes identified by LC-MS/MS from worker and soldier castes of *C. gestroi***.

	**Identified Proteins**	**Species**	**Taxonomic Group**	**Accession Number**	**Pfam domain**	**Secretion Signal**	**Normalized Spectrum Counts (Peptides)**	
							**Worker**	**Soldier**
**GLYCOSIDE HYDROLASES**								
GH1	β-glucosidase	*Coptotermes formosanus*	Insect	gALL_v3_rep_c96194_2	glyco_hydro_1	Yes	11	5
GH1	β-glucosidase	*Coptotermes formosanus*	Insect	gALL_v3_c53472_2	glyco_hydro_1	Yes	0	1
GH1	β-glucosidase	*Odontotermes formosanus*	Insect	gALL_v3_c8970_4	glyco_hydro_1	nd	1	1
GH2	PREDICTED: similar to mannosidase, β A, lysosomal	*Acyrthosiphon pisum*	Insect	gALL_v3_c19245_5	glyco_hydro_2	Yes	0	1
GH2	β-mannosidase precursor, putative	*Pediculus humanus corporis*	Insect	gALL_v3_c1701_2	glyco_hydro_2	nd	2	1
GH3	β-glucosidase	*Zunongwangia profunda* SM-A87	Bacteria	gALL_v3_c14740_6	glyco_hydro_3	nd	0	1
GH3	b-glucosidase, glycoside hydrolase family 3 protein	*Pedobacter* sp. BAL39	Bacteria	gALL_v3_c13414_4	glyco_hydro_3	nd	1	1
GH5	putative glycosyl hydrolase family5	uncultured symbiotic protist of *Cryptocercus punctulatus*	Protist	gALL_v3_c25216_2	cellulase	nd	2	1
GH5	putative glycosyl hydrolase family5	uncultured symbiotic protist of *Reticulitermes speratus*	Protist	gALL_v3_c2080_6	cellulase	nd	1	2
GH7	putative glycosyl hydrolase family7	uncultured symbiotic protist of *Neotermes koshunensis*	Protist	gALL_v3_c12313_5	glyco_hydro_7	nd	0	1
GH7	cellulase	*Pseudotrichonympha grassii*	Protist	gALL_v3_rep_c96913_5	glyco_hydro_7	nd	6	7
GH9	endo-β-1,4-glucanase	*Coptotermes formosanus*	Insect	gALL_v3_rep_c100427_4	glyco_hydro_9	nd	9	1
GH9	endo-β-1,4-glucanase	*Coptotermes formosanus*	Insect	gALL_v3_rep_c98165_4	glyco_hydro_9	nd	17	0
GH9	endo-β-1,4-glucanase	*Coptotermes formosanus*	Insect	gALL_v3_rep_c96171_2	glyco_hydro_9	nd	23	0
GH9	endo-β-1,4-glucanase	*Coptotermes formosanus*	Insect	gALL_v3_rep_c96486_1	glyco_hydro_9	Yes	17	0
GH9	endo-β-1,4-glucanase	*Coptotermes formosanus*	Insect	gALL_v3_rep_c96106_2	glyco_hydro_9	nd	19	12
GH10	putative glycosyl hydrolase family10	uncultured symbiotic protist of *Cryptocercus punctulatus*	Protist	gALL_v3_c47557_6	glyco_hydro_10	nd	4	4
GH13	α-amylase	*Blattella germanica*	Insect	gALL_v3_c56260_1	α-amylase	Yes	2	2
GH13	α-amylase	*Blattella germanica*	Insect	gALL_v3_rep_c96185_6	α-amylase	Yes	2	0
GH13	PREDICTED: similar to glycogen debranching enzyme	*Nasonia vitripennis*	Insect	gALL_v3_c6469_5	α-amylase	nd	2	2
GH16	gram negative bacteria-binding protein 2	*Reticulitermes flavipes*	Insect	gALL_v3_rep_c96609_5	glyco_hydro_16	Yes	1	0
GH26	Mannan endo-1,4-β-mannosidase	*Fibrobacter succinogenes* subsp. succinogenes	Bacteria	gALL_v3_c37810_4	glyco_hydro_26	Yes	1	0
GH29	PREDICTED: similar to fucosidase, α-L- 2, plasma	*Apis mellifera*	Insect	gALL_v3_c472_5	α_l_fucos	Yes	0	1
GH29	PREDICTED: similar to fucosidase, α-L- 2, plasma	*Apis mellifera*	Insect	gALL_v3_c472_4	α_l_fucos	Yes	2	5
GH37	Trehalase precursor, putative	*Zootermopsis nevadensis*	Insect	gALL_v3_c11076_6	trehalase	nd	1	1
GH38	lysosomal α-mannosidase	*Culex quinquefasciatus*	Insect	gALL_v3_c95732_3	α-mann_mid	Yes	1	0
GH39	Bla g 1.02 variant allergen	*Blattella germanica*	Insect	gALL_v3_rep_c96321_4	glyco_hydro_39	Yes	1	0
GH45	putative glycosyl hydrolase family45	uncultured symbiotic protist of *Hodotermopsis sjoestedti*	Protist	gALL_v3_c17434_3	glyco_hydro_45	nd	0	1
					Total spectra		126	51
**AUXILIARY ACTIVITIES**								
AA3	glucose dehydrogenase precursor, putative	*Pediculus humanus corporis*	Insect	gALL_v3_rep_c97150_5	gmc_oxred_n	Yes	0	4
AA3	PREDICTED: similar to glucose dehydrogenase	*Acyrthosiphon pisum*	Insect	gALL_v3_rep_c97604_6	gmc_oxred_n	Nd	3	12
AA3	PREDICTED: similar to glucose dehydrogenase	*Acyrthosiphon pisum*	Insect	gALL_v3_rep_c99896_6	gmc_oxred_n	Nd	1	1
AA3	PREDICTED: similar to glucose dehydrogenase	*Tribolium castaneum*	Insect	gALL_v3_c10164_5	gmc_oxred_n	Yes	0	1
AA5	PREDICTED: similar to aldehyde oxidase	*Nasonia vitripennis*	Insect	gALL_v3_c4092_3	glyoxal_oxid_n /ald_xan_dh_c	Nd	10	5
AA5	PREDICTED: similar to aldehyde oxidase	*Nasonia vitripennis*	Insect	gALL_v3_c2305_4	glyoxal_oxid_n /ald_xan_dh_c	Nd	0	2
					Total spectra		14	25
**CARBOHYDRATE ESTERASES**								
CE1	juvenile hormone esterase-like protein Est1	*Reticulitermes flavipes*	Insect	gALL_v3_c20639_4	abhydrolase_2	nd	0	2
CE4	predicted xylanase/chitin deacetylase	*Reticulitermes speratus*	Insect	gALL_v3_c26010_1	polysacc_deac_1	nd	1	0
CE10	carboxylesterase clade E, member 11	*Nasonia vitripennis*	Insect	gALL_v3_rep_c97115_5	coesterase	Yes	8	3
CE10	juvenile hormone esterase	*Apis mellifera*	Insect	gALL_v3_rep_c96274_1	coesterase	Yes	7	4
CE10	juvenile hormone esterase-like protein Est1	*Reticulitermes flavipes*	Insect	gALL_v3_c350_2	coesterase	nd	2	0
CE10	juvenile hormone esterase-like protein Est1	*Reticulitermes flavipes*	Insect	gALL_v3_c2097_4	coesterase	nd	1	0
CE10	PREDICTED: similar to juvenile hormone esterase	*Acyrthosiphon pisum*	Insect	gALL_v3_c14436_2	coesterase	Yes	1	1
CE10	PREDICTED: similar to juvenile hormone esterase	*Acyrthosiphon pisum*	Insect	gALL_v3_c13039_2	coesterase	nd	0	1
CE10	PREDICTED: similar to juvenile hormone esterase	*Nasonia vitripennis*	Insect	gALL_v3_c9362_2	coesterase	Yes	2	0
CE10	PREDICTED: similar to juvenile hormone esterase	*Tribolium castaneum*	Insect	gALL_v3_c26448_5	coesterase	nd	2	2
CE10	PREDICTED: similar to juvenile hormone esterase	*Tribolium castaneum*	Insect	gALL_v3_c5472_5	coesterase	nd	1	0
CE10	PREDICTED: similar to putative esterase	*Tribolium castaneum*	Insect	gALL_v3_c21767_2	coesterase	Yes	1	1
					Total spectra		26	14
**PAD ENZYMES**								
AKR	aldose reductase, putative	*Pediculus humanus corporis*	Insect	gALL_v3_rep_c98481_5	aldo_ket_red	nd	1	0
AKR	aldo-keto reductase	*Aedes aegypti*	Insect	gALL_v3_rep_c96584_4	aldo_ket_red	nd	3	2
AKR	PREDICTED: similar to aldo-keto reductase isoform 1	*Tribolium castaneum*	Insect	gALL_v3_rep_c96328_4	aldo_ket_red	Yes	8	6
AKR	PREDICTED: similar to aldo-keto reductase isoform 1	*Tribolium castaneum*	Insect	gALL_v3_rep_c97134_3	aldo_ket_red	nd	5	0
AKR	PREDICTED: similar to aldo-keto reductase isoform 1	*Tribolium castaneum*	Insect	gALL_v3_c15066_3	aldo_ket_red	nd	3	0
CAT	catalase	*Stylochus sp*. KJP-2004	Platyhelminthes	gALL_v3_c48664_4	catalase	nd	0	1
CAT	Catalase, putative	*Pediculus humanus corporis*	Insect	gALL_v3_rep_c96312_6	catalase	nd	12	13
GST	glutathione S-transferase	*Blattella germanica*	Insect	gALL_v3_rep_c96283_4	gst_n	nd	18	5
GST	glutathione S-transferase	*Blattella germanica*	Insect	gALL_v3_rep_c96354_6	gst_n	nd	10	7
GST	glutathione S-transferase	*Blattella germanica*	Insect	gALL_v3_c6961_2	gst_n	nd	3	6
GST	glutathione S-transferase O1	*Nasonia vitripennis*	Insect	gALL_v3_rep_c96308_6	gst_n	nd	5	3
GST	glutathione S-transferase O1	*Nasonia vitripennis*	Insect	gALL_v3_c2087_5	gst_n	nd	1	1
GST	glutathione S-transferase O1	*Nasonia vitripennis*	Insect	gALL_v3_c37119_2	gst_n	nd	2	0
GST	glutathione S-transferase T3	*Nasonia vitripennis*	Insect	gALL_v3_c4709_3	gst_n	Yes	4	2
GST	RecName: Full = Glutathione S-transferase;	*Blattella germanica*	Insect	gALL_v3_c57001_4	gst_n	nd	0	5
p450	cytochrome P-450, putative	*Pediculus humanus corporis*	Insect	gALL_v3_c2488_6	p450	nd	1	0
p450	cytochrome P450	*Hodotermopsis sjoestedti*	Insect	gALL_v3_rep_c96479_3	p450	nd	1	1
p450	cytochrome P450	*Hodotermopsis sjoestedti*	Insect	gALL_v3_c46657_4	p450	nd	1	0
p450	cytochrome P450	*Hodotermopsis sjoestedti*	Insect	gALL_v3_c11806_5	p450	nd	1	0
p450	cytochrome p450	*Hodotermopsis sjoestedti*	Insect	gALL_v3_rep_c96280_1	p450	nd	1	0
p450	NADPH–cytochrome P450, putative	*Pediculus humanus corporis*	Insect	gALL_v3_c17236_6	p450	nd	1	0
p450	p450	*Reticulitermes flavipes*	Insect	gALL_v3_rep_c104501_6	p450	Yes	1	0
SOD	superoxide dismutase	*Triatoma infestans*	Insect	gALL_v3_rep_c96668_1	sod_cu	nd	9	8
SOD	superoxide dismutase 1	*Tritrichomonas foetus*	Insect	gALL_v3_c59228_1	sod_cu	nd	0	1
SOD	Mn superoxide dismutase	*Bombyx mori*	Insect	gALL_v3_rep_c97252_3	sod_cu	nd	3	0
SOD	extracellular superoxide dismutase precursor	*Pacifastacus leniusculus*	Crustacea	gALL_v3_c25166_5	sod_cu	Yes	1	1
SOD	Cu/Zn superoxide dismutase	*Rachycentron canadum*	Actinopterygii	gALL_v3_c6342_6	sod_cu	Yes	1	2
					Total spectra		96	64

The distribution of the peptides throughout the CAZy families and domains of PAD enzymes are described in Figures 4D,E, Table 2. The most abundant glycoside hydrolase family in both castes was GH9 (Figures 4D,E). The second most abundant family was GH7, followed by GH1. Several previous studies have reported that these GH families are directly involved in cellulose breakdown (Cantarel et al., 2009; Franco Cairo et al., 2013; Scharf, 2015). All GH9 and GH1 peptide matches, for both workers and soldiers, were of insect origin, and conversely, GH7 peptide matches were of protist origin. Our results also identified peptide matches for proteins involved in hemicellulose degradation in both castes, such as peptides from proteins of GH2 and GH29 families that were of insect origin, the GH3 and GH26 families that were of bacterial origin and the GH5 and GH10 families of protist origin.

The AAs identified in our metaproteomics data were from families AA3 and AA5 for both castes (Table 2), in which the most abundant in soldier was AA3 and AA5 in workers. All auxiliary activity enzymes identified were of insect origin. No AA1 (laccases) 1and AA4 family members were found in our metaproteomic analysis. Concerning the CE members, our analysis resulted in the identification of 4 families in the workers and soldiers, all of insect origin. CE1 family members were only identified in the soldiers. Likewise, CE4 protein was identified only in the workers. CE10 was the most abundant family identified in both castes.

The PAD enzymes were also assigned in the metaproteomic analysis of *C. gestroi*. Five different Pfam domains were identified in the workers and soldiers. Based on spectrum counts, GST was the most abundant PAD enzyme in both castes, followed by CAT. SOD and AKR was the third most abundant enzyme in workers and soldier, respectively. Regarding the taxonomy of the PAD enzymes, our results indicated that insect was the major contributor of these components. However, two SODs were of Crustacea and Actinopterygii origin, and one CAT was of Platyhelminthes origin.

Several proteins identified by proteomics contained secretion signals (Table 1), thus confirming our metatranscriptomics findings. Among them, proteins correlated to lignocellulose breakdown were found from insect and bacteria origin, such as GH1, GH2, GH9, GH16, GH26, and GH29. All proteins from protists, assigned as cellulolytic enzymes, did not present secretion signals, as expected. In addition, putative secretion signals were identified in proteins from families AA3 and AA5, as well as from PAD enzymes, such as SOD and AKR. Regarding carbohydrate esterases, one CE10 was identified as containing a secretion signal (Table 2).

### Enzymatic assays using polysaccharides and oligosaccharides support the repertoire of glycoside hydrolases in *C. gestroi*

**Figure 5:**
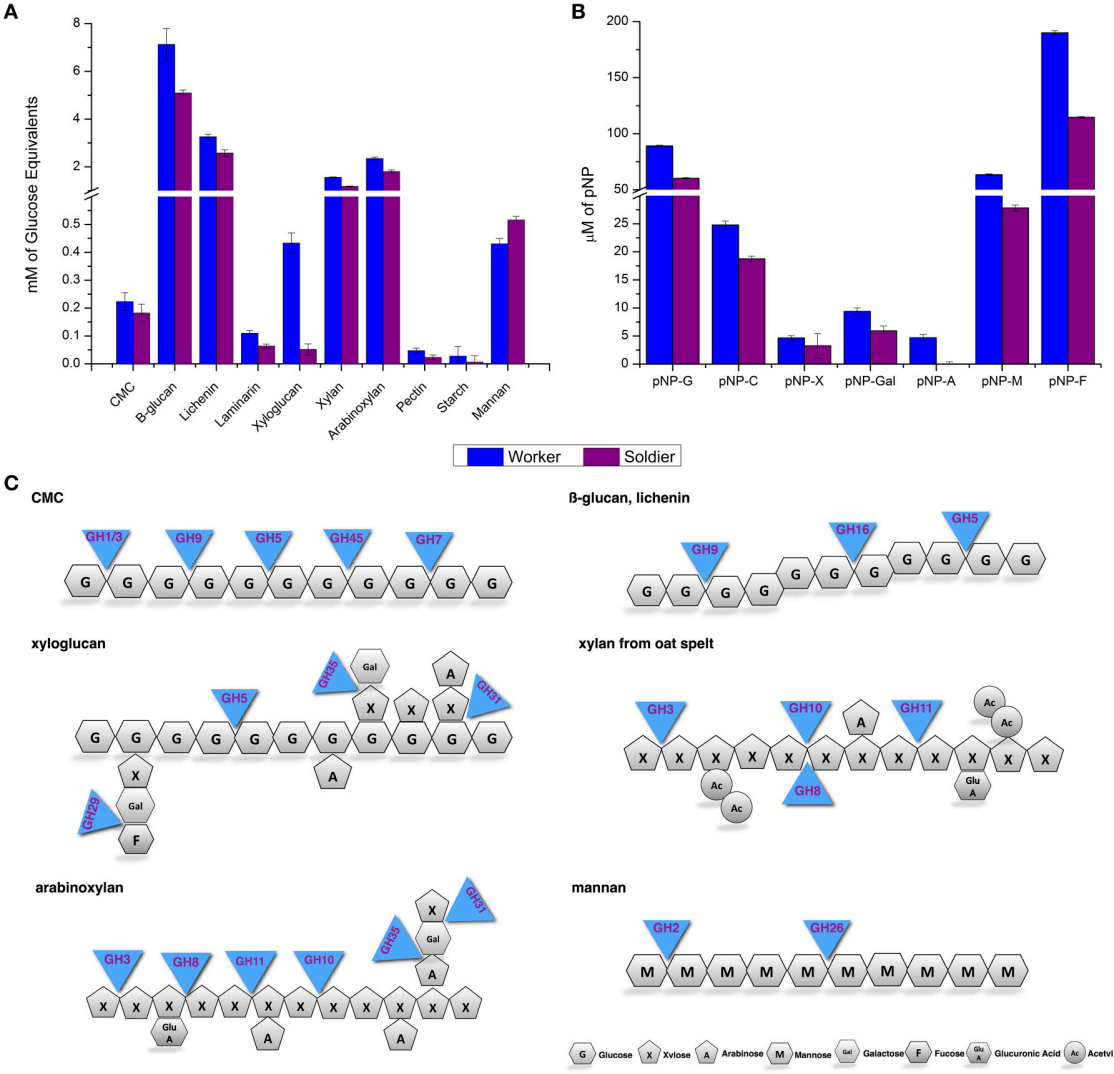
**Biochemical assays using worker and soldier crude extract from ***C. gestroi***. (A)** Evaluation of the enzymatic activities of worker and soldier's crude extracts against natural polysaccharides at pH 5.5 using DNS reagent. CMC, carboxymethylcellulose; β-glucan from barley, lichenan from moss, laminarin from *Laminaria digitata*, xyloglucan from tamarind, xylan from oat spelt, rye arabinoxylan, mannan, pectin from *Citrus*. **(B)** Evaluation of the enzymatic activities of worker and soldier's crude extracts on synthetic oligosaccharides at pH 5.5. *p*NP-G, 4-nitrophenyl β-D-glucopyranoside; *p*NP-C, 4-nitrophenyl β-D-cellobioside; *p*NP-X, 4-nitrophenyl β-D-xylopyranoside; *p*NP-Gal, 4-nitrophenyl β-D-galactopyranoside; *p*NP-A, 4-nitrophenyl α-L-arabinofuranoside; *p*NP-M, 4-nitrophenyl β-D-mannopyranoside; *p*NP-F, 4-nitrophenyl-α-L-fucopyranoside. **(C)** Scheme describing the deconstructive hydrolytic enzymes interactions in *C. gestroi* for natural polysaccharides breakdown. The reported enzymes were chosen based on the number of transcripts and/or peptides taken in consideration the presence of secretion signal for unigenes with insect origin (GH1, GH2, GH9, and GH16) and bacteria origin (GH5 and GH26). However, for the families GH3, GH5, GH7, GH10, GH11, and GH45 with protist origin, the secretion signal was not taken in consideration, since these microorganisms perform the digestion of lignocelluloses in their cytoplasm. The polysaccharide structures were drawn based on the current literature (van den Brink and de Vries, 2011; Segato et al., 2014).

The crude protein extracts of *C. gestroi* workers and soldiers were tested for their ability to breakdown natural polysaccharides and synthetic oligosaccharides. All these assays were performed with biological and technical triplicates (represented in the error bars). The biochemical assays using polysaccharides were performed using the same amount of protein source from both castes. In general, the results showed that the worker crude extract could breakdown all the natural substrates tested, as well as, the worker extract was more efficient than the soldier crude extract. According to Figure 5A, the crude extracts from both castes exhibited the highest activity toward β-glucan, followed by lichenin, rye arabinoxylan, xylan, mannan, xyloglucan, CMC, laminarin, and pectin. For the *p*-nitrophenyl-derivatives (*p*NP), the results showed *p*NP-F as the most hydrolyzed substrate by crude extracts from both castes, followed by *p*NP-G, *p*NP-M, *p*NP-C, and *p*NP-Gal. The activities against *p*NP-X and *p*NP-A were not conclusive (Figure 5B).

In Figure 5C, a scheme showing the deconstructive hydrolytic enzymes interactions in *C. gestroi* was generated based on the assessments for GHs found in our metatranscriptomic and metaproteomic data. In this scheme only GHs correlated with lignocellulose degradation were considered. The main enzymes from GH families identified in this work, which could be involved in the hydrolysis of glucose-polymers such as CMC, β-glucan, Laminarin, Xyloglucan, and Lichenin, were GH1, GH5, GH7, GH9, GH16, and GH45 families. For xylose polymers degradation such as xylan and arabinoxylan, the main families were GH3, GH8, GH10, and GH11. In the case of the mannose-based polymer, such as mannan, the putative GH families found in our data correlated with the hydrolysis of this substrate were GH2, GH5, and GH26. The activities against *p*NP-G (substrate for β-glucosidases) corroborated our findings for enzymes from families GH1 and GH3, *p*NP-C (substrate for cellobiohydrolases) to GH7 family, and *p*NP-M (β-mannosidases substrate) for GH2 (not exhibited in figure). Collectively, our biochemical assays agreed with our metatranscriptomic and metaproteomic data, thus, validating our overall results.

## Discussion

In this study, the comprehensive characterization of the CAZy and PAD enzymes profile from worker and soldier castes of the lower termite *C. gestroi* was performed using metatranscriptomic, metaproteomic, and biochemical approaches. Moreover, the data reported in the “omics” analysis was further correlated with the enzymatic assays. Therefore, the dataset expanded the knowledge for gene sequences related to lignocellulose-active enzymes from *C. gestroi*, supporting previously information of endogenous and symbiotic CAZymes identified by Franco Cairo et al. (2011), throughout proteomic and biochemical approaches.

Metagenomics and metatranscriptomics in worker caste of lower and high termites were previously described targeting CAZymes discovery (Xie et al., 2012; Sethi et al., 2013b; Do et al., 2014). However, the CAZy and PAD enzymes repertoire of soldiers from lower termites has not yet been reported in literature: thus, our study provides support that soldiers can express CAZy-PAD genes and enzymes, mainly endogenous glycoside hydrolases, which was further confirmed by enzymatic activities toward polysaccharides in this caste.

Our metatranscriptomic and metaproteomic results showed that worker and soldiers shared similar repertoires of GHs, AAs, and PADs. Moreover, the majority of GH and AA families, as well as the PAD classes identified in this work, contained unigenes predicted with secretion signals, suggesting a putative role in the *C. gestroi*'s digestome. Another interesting result is that GH9 is the most abundant glycoside hydrolase family in workers, and it is also expressed in soldier, along with other GHs. GH1 and GH9 are considered the main cellulases in lower termite digestomes and present high degree of synergism regarding lignocellulose degradation (Scharf et al., 2011; Franco Cairo et al., 2013). The high enzymatic activities against β-glucan, lichenin and *p*NP-G substrates exhibited by *C. gestroi* crude extract, in both castes, further corroborated the high abundance of both enzymes in *C. gestroi*. However, some GH1 unigenes identified in our analysis could be related to caste regulation rather than lignocellulose degradation, such as the gene *Neofem2* (GH1) from *Cryptotermes secundus* (displaying homology with pfam PF00232; Weil et al., 2009).

The present work also showed that genes and enzymes for classical endo-cellulases activities from families GH5 and GH45; exo-cellulases from family GH7; and xylanases from GH10 and GH11 families were expressed by protists in both castes. These results were also confirmed by the enzymatic activities against CMC and β-glucan for GH5 and GH45, *p*NP-C for GH7 and xylan and arabinoxylan for GH10 and GH11. Since protists degrade the lignocellulosic material in their cytoplasm (Ohkuma, 2008), the majority of the unigenes from these GH families were not predicted to contain secretion signals. Moreover, according to studies in *R. flavipes*, pine wood hydrolysis by GH1 and GH9 from termites was enhanced by GH7 and GH10 from protists, whereas the efficiency of these enzymes on recalcitrant substrates was considered very low when compared with commercial celullases and fungi secretomes (Watanabe and Tokuda, 2010; Scharf et al., 2011; Sethi et al., 2013a).

The phylogenetic analysis of symbiotic CAZymes from families GH5, GH7, GH10, and GH11 highlighted the extensive diversity of *C. gestroi* symbiotic enzymes. The occurrence of GH5 enzymes grouped with bacterial GH5 supported previous works, exposing the bacterial CAZymes role in the digestome of lower termites (Franco Cairo et al., 2011; Do et al., 2014). The symbiotic enzymes from family GH7 found in this work, which were annotated as protist origin based on the first Blast hit organism, were grouped together with fungi sequences, corroborating with previous phylogenetic studies of termite symbiotic GH7 enzymes (Todaka et al., 2010).

The AA is the new class of oxidative enzymes from the CAZy database. An endogenous multi-copper laccase, classified as AA1_3, was identified in our metatranscriptomic analyses, which shows similarities to an endogenous laccase from *R. flavipes*. Previously, this enzyme was functionally characterized and classified as a hydrogen peroxide-dependent enzyme involved in lignin oxidation and modifications (Coy et al., 2010). Other AAs identified in this study are known as hydrogen peroxide-generating enzymes, such as AA3_2, AA3_3, AA5_1, and AA5_2 (Arantes et al., 2012; Levasseur et al., 2013). Our data is supported by previous studies on the lower termite *R. flavipes*, which identified AA1_3 and AA3_3 based on metatranscriptomic analysis (Tartar et al., 2009) as well as in agreement with the generation of hydrogen peroxide in the midgut of another lower termite *C. formosanus* (Ke and Chen, 2013). However, the last two termite genomes published recently did not report the presence of AAs in their genomes, only from gut bacteria (Poulsen et al., 2014; Terrapon et al., 2014).

Several previous studies reported that termites could perform lignin modifications. For example, Ke et al. (2012) reported the increase of hydroxyl content and side chain oxidation in the lignin-rich feces of the lower termite *C. formosanus*. Geib et al. (2008) described significant levels of propyl side-chain oxidation, demethylation of the ring methoxyl group and ring hydroxylation in lignin-rich feces of the termite *Z. angusticollis*. In our study, the identification of laccases (AA1_3) and enzymes belonging to the families AA4 and AA5, composed mainly by vanillyl-alchool and glyoxal oxidases (Levasseur et al., 2008, 2013), corroborate the occurrence of lignin modifications in the termite digestome. Interestingly, lignin peroxidases and manganese peroxidases (AA2) were not identified in our data, as showed by previous studies on the termite digestome (Tartar et al., 2009).

According to our results, PAD components (in this study it was considered only SOD, CAT, GST, AKR, and p450) were identified as abundant constituents in metaproteogenomic data when compared with the number of reads and peptides from glycoside hydrolases GH1 and GH9. Tartar et al. (2009) raised the hypothesis that PAD enzymes from *R. flavipes* could play a role in either lignin degradation or the scavenging of free radicals and other toxic metabolites derived from lignin. Sethi et al. (2013a) reported that these enzymes were up regulated when termites were fed on wood (complex lignocellulosic substrate) and filter paper impregnated with alkali lignin. According to Sethi et al. (2013a,b), AKR and CAT enzymes were reported to work in synergy with cellulases, such as GH9 and GH1 from termite, and GH7 from protist. However, the supplementation of CAT in enzymatic cocktails containing AKR and those cellulases resulted in lower hydrolysis yields (Sethi et al., 2013b).

Furthermore, GST enzymes from bacteria have been described to cleave β-Aryl-Ether linkages from lignin (Masai et al., 2003). This class of enzyme was also described as a major constituent of the secretome of *Enterobacter lignolyticus* SCF, a bacteria with high capacity to degrade and assimilate low molecular weight lignin (DeAngelis et al., 2013). Recently, two superoxide dismutases from the bacteria *Sphingobacterium* sp. T2 were reported with high oxidative activity against Organosolv and Kraft lignins (Rashid et al., 2015). Thus, the GST and SOD enzymes found in our analysis could also be related to lignin oxidation and modifications. Unigenes and peptides matches for p450 were also identified in our analyses, but their contributions to lignin modification and detoxification have not been described. Moreover, saprotrophic Agaricomycetes mushrooms and their relatives have evolved and expanded the number of gene copies that encode oxidoreductases enzymes related to lignin degradation and detoxification, mainly from white-rot fungi, which included peroxidases class II (AA2), laccases (AA1_1), and p450 (Floudas et al., 2012).

Several *in-vitro* studies have shown that endogenous (GH9 and GH1) and symbiotic glycosidases (GH5, GH7, and GH45) have low activity against recalcitrant lignocellulose biomass (Fujita et al., 2010; Franco Cairo et al., 2011; Otagiri et al., 2013). Moreover, the enzymatic degradation and modifications of lignin that occur in termites have not been fully elucidated (Geib et al., 2008; Ke and Chen, 2013). Brune (2014) hypothesized that auxiliary oxido-reduction mechanisms could play a role in the termite digestome to degrade cellulose, hemicellulose and lignin. Although, these redox mechanisms promoted by oxidative enzymes (e.g., Lytic Polysaccharide Mono-oxygenases, cellobiose dehydrogenase and glucose oxidases/dehydrogenases) and Fenton chemistry (Fe^2+^ + H_2_O_2_ → OH• + OH) have already been reported for other lignocellulolytic organisms, such as bacteria and fungi (Jensen et al., 2001; Arantes et al., 2012), they have not yet been reported in termites. Fenton chemistry was reported in brown-rot fungi as the main reaction to depolymerize lignin and cellulose. In this reaction, enzymes from families AA3, AA5, and AA8 played a crucial role to generate hydrogen peroxide aiming to initiate the reaction in the presence of Fe^2+^ (Levasseur et al., 2013). Fenton-type reaction was previously reported by Barbehenn et al. (2005) in the midgut of the leaf–feeding caterpillars. In termites, hydrogen peroxide generation and iron reduction capacity were already described in the guts of *C. formosanus* and *Z. nevadensis*, respectively (Vu et al., 2004; Ke and Chen, 2013).

Thus, to aid in explaining the degradation of recalcitrant lignocellulosic biomass displayed by termites, the AAs and PAD enzymes described in this study indicate that redox mechanisms should be further investigated in this biological systems. Nevertheless, it is important to emphasize here that although the idea of pro-oxidant enzymes (such as AA3, AA5, AA8, AKR, and SOD) working together with antioxidant enzymes (such as CAT and GST) seems contradictory, reports in literature have indicated the occurrence of a fine tuning between the generation and scavenging of ROS in insects (Barbehenn et al., 2005; Ke and Chen, 2013) and fungi (Arantes et al., 2012).

In conclusion, our “omics” data indicate that enzymes such as GH, PAD, CE, and AA are highly abundant in *C. gestroi* and contribute to further understand the potential enzymatic arsenal in the degradation of lignocellulosic biomass in this biorecycling system. Our findings provide molecular basis to support that worker and soldier castes have similar repertoire of expressed CAZy and PAD enzymes, as well as, confirmed that GH9 and GH1, are the most important glycoside hydrolases related to lignocellulose degradation in both castes. Furthermore, gene sequences and peptides from classical cellulases such as exo-glucanases (GH7) and endo-glucanases (GH5 and GH45) as well as for classical xylanases (GH10 and GH11) were found in worker and soldier castes only taxonomically related to protists, highlighting the importance of the symbiosis relationships in both castes. Moreover, our analysis revealed the presence of oxidoreductases from Auxiliary Activity enzyme families (AAs) and PAD classes in both castes, which could be related to lignin modification and degradation in termite digestomes.

It is important to emphasize that this study was based on the whole termite bodies and not all the CAZy and PAD enzymes described herein may play a role in the digestive physiology of *C. gestroi*. For instance, the peroxidases described herein can be involved in the immune defense or cuticle formation, as well as many of the detected enzymes in the present study should have roles other than lignocellulose degradation. To gain conclusive evidence about which GHs, CEs, PADs, and AAs enzymes are occurring in termites, specifically in the their gut, further studies are necessary, such as *in situ* localization and functional characterization of the enzymes described in this work.

## Author contributions

JF performed the experimental design, biochemical assays, molecular biology experiments; mass spectrometer sample preparation, transcriptome, proteomic, and biochemical data analyses and drafted the manuscript. MC performed the bioinformatics analyses and drafted the manuscript. FL performed the termite cultivation for RNA extraction, libraries preparation for pyrosequence and transcriptome data analysis. LM performed the bioinformatics analyses. LB performed biochemical assays and drafted the manuscript. TG performed the experimental design for biochemical activities and analyzed data. RD performed LC-MS/MS runs, processed MS/MS data and reviewed the draft manuscript. CU performed experimental designed of biochemical assays, analyzed data and reviewed the manuscript. TA analyzed data and drafted the manuscript. RT performed the biochemical analyses and data interpretation. RV performed the bioinformatics analyses. FC helped with the experimental design and coordinated the metatranscriptomic study. AC performed the experimental design of termite cultivation and reviewed the draft manuscript. AP performed experimental designed of metaproteomic experiments, helped in data analyses and drafted the manuscript. GP helped with the experimental design and coordinated the metatranscriptomic study. FS conceived, designed and coordinated the study, performed the data analyses, provided financial support and wrote the final manuscript.

## Funding

We are grateful to FAPESP (The State of São Paulo Research Foundation) for scholarships (11/20977-3 and 15/06971-3 to JF, 12/19040-0 and 14/10351-8 to CU, 06/59086-8 to FL, 14/20576-7 to RT, 13/03061-0 to LB, and 10/11469-1 to TA) and financial support (08/58037-9 and 14/50371-8 to FS and 08/50114-4 to FC). We are grateful to the National Council for Scientific and Technological Development (CNPq) for the Ph.D. scholarship (140796/2013-4 to TG) and financial support (310186/2014-5, 442333/2014-5).

### Conflict of interest statement

The authors declare that the research was conducted in the absence of any commercial or financial relationships that could be construed as a potential conflict of interest.

## Supplementary material

Figure S1 **Pyrosequence and bioinformatic workflow performed in this present study**. Photo from Ana Maria Costa-Leonardo.

Figure S2 **Phylogenetic tree for putative symbiotic cellulases. (A)** endo-glucanases from GH5 family and **(B)** exo-glucanases from GH7 family.

Figure S3 **Phylogenetic tree for putative symbiotic xylanases. (A)** xylanases from family GH10 and **(B)** xylanases from family GH11.

Table S1 **Total unigenes identified in worker and soldier castes of ***Coptotermes gestroi*****.

Table S2 **Genome overview**.

Table S3 **Secretion signal predictions of CAZy and PAD enzymes identified in metatranscriptomic from worker and soldier castes of ***C. gestroi*****. The analysis was performed using *C. gestroi* (Method 1) and *Z. nevadensis* (Method 2) genomes as references, SignalP and WolF-PSORT were used for signal peptide prediction.

Table S4 **Total proteins identified in worker and soldier castes of ***Coptotermes gestroi*****.
