# Supplementary material for: Expanding the Knowledge on Lignocellulolytic and Redox Enzymes of Worker and Soldier Castes from the Lower Termite Coptotermes gestroi
Source: Front Microbiol. 2016 Oct 13;7:1518. doi: 10.3389/fmicb.2016.01518 (PMC5061848; doi:10.3389/fmicb.2016.01518)
Supplement: Figure S1 — Pyrosequence and bioinformatic workflow performed in this present study. Photo from Ana Maria Costa-Leonardo. [file DataSheet1.DOCX]

**Expanding the knowledge on lignocellulolytic and redox enzymes of worker and soldier castes from the lower termite *Coptotermes gestroi***

João Paulo Lourenço Franco Cairo^1,2^; Marcelo Falsarella Carazzolle^2,5^; Flávia Costa Leonardo^2,3^; Luciana Souto Mofatto^2^; Lívia Beatriz Brenelli^1^; Thiago Augusto Gonçalves^1^; Cristiane Akemi Uchima^1^; Romênia Ramos Domingues^5^, Thabata Maria Alvarez ^1^; Robson Tramontina^1^; Ramon Oliveira Vidal ^2,5^; Fernando Ferreira Costa^3^; Ana Maria Costa-Leonardo^4^; Adriana Franco Paes Leme^5^; Gonçalo Amarante Guimarães Pereira^2^ & Fabio Marcio Squina^1*^

^1^ Laboratório Nacional de Ciência e Tecnologia do Bioetanol (CTBE), Centro Nacional de Pesquisa em Energia e Materiais (CNPEM), Campinas, Brazil

^2^ Laboratório de Genômica e Expressão (LGE), Universidade Estadual de Campinas, Campinas, Brazil.

^3^ Centro de Hematologia e Hemoterapia (Hemocentro), Universidade Estadual de Campinas, Campinas, Brazil

^4^ Departamento de Biologia, Instituto de Biociências, Universidade Estadual Paulista (UNESP), Rio Claro, Brazil

^5^ Laboratório de Espectrometria de Massas, Laboratório Nacional de Biociências (LNBio), Centro Nacional de Pesquisa em Energia e Materiais (CNPEM), Campinas, Brazil

^*^ **Corresponding author:**

**Address:** Centro Nacional de Pesquisa em Energia e Materiais (CNPEM) Laboratório Nacional de Ciência e Tecnologia do Bioetanol (CTBE) Caixa Postal 6170, 13083-970, Campinas (SP), Brazil. Tel.: +55 19 3518 3111; fax: +55 19 35183104.

***E-mail address:*** fabio.squina@bioetanol.org.br

# Supporting Figures


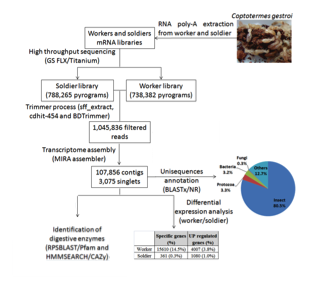


**Figure S1.** Pyrosequence and bioinformatic workflow performed in this present study. Photo from Ana Maria Costa-Leonardo.


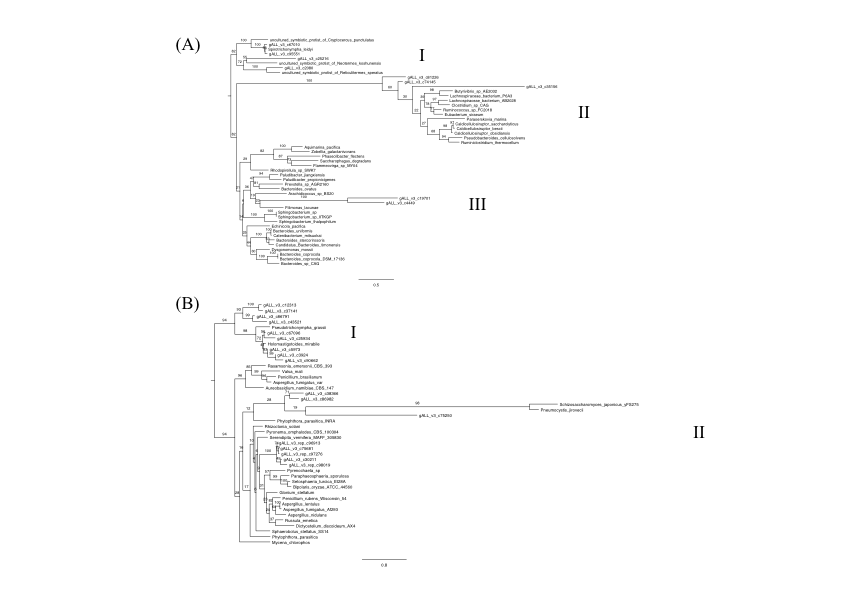


**Figure S2.** Phylogenetic tree for putative symbiotic cellulases. (A) endo-glucanases from GH5 family and (B) exo-glucanases from GH7 family.


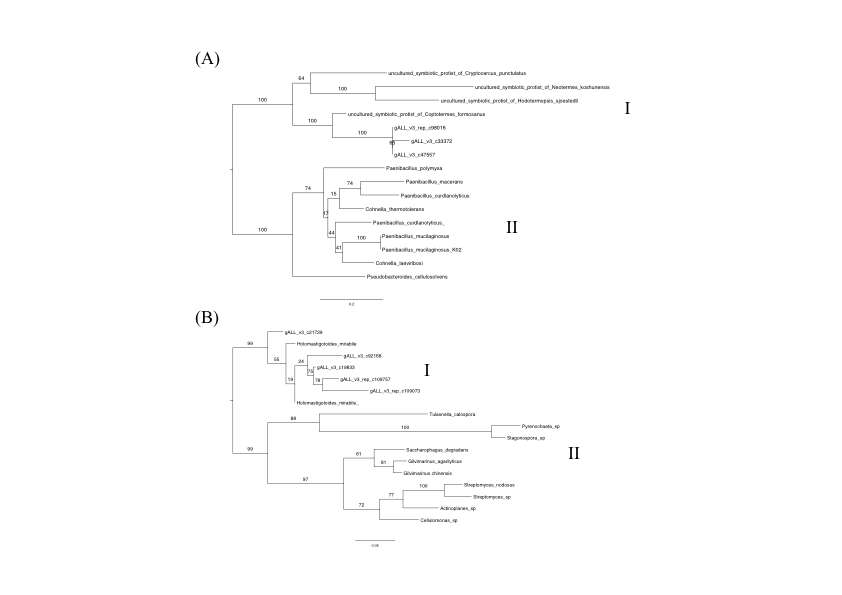


**Figure S3.** Phylogenetic tree for putative symbiotic xylanases. (A) xylanases from family GH10 and (B) xylanases from family GH11.

# Supporting Material and Methods

## Genome assembly

The *Coptotermes gestroi* DNA was extracted from 50 specimens using the MPBio DNA extraction kit according to the manufacturer’s guidelines. Then, the DNA was sequenced by Illumina technology, resulting in 1 billion paired-reads. The 100-bp paired-end reads were assembled into scaffolds using SOAPdenovo assembler (Luo et al., 2012) with k-mer size optimized to maximize the N50 length. The protein-coding prediction was performed using AUGUSTUS gene prediction previously trained for the *Camponotus floridanus* genome.

## Secretion Signal Analysis

Because of sub-representation of full-length ESTs in the unisequences dataset, we identified the secreted unisequences indirectly by alignment of EST unisequences against predicted secreted proteins from the *C. gestroi* draft genome (Table S2) and *Zootermopsis nevandensis* (Terrapon et al., 2014) genome. Firstly, the unisequences were aligned into the *C. gestroi* and *Z. nevandensis* genomes using the Exonerate program (Slater and Birney, 2005) configured with settings for est2genome (nucleotide alignment) and coding2genome (protein alignment), respectively. The list of secreted proteins in these two genomes was identified using the SignalP version 4.1 (Petersen et al., 2011) and WolF PSORT (Horton et al., 2007) programs configured with settings for eukaryote and animal sequence data, respectively. The protein was considered secreted if it was identified in at least one of the two programs. The identification of secreted unisequences was performed by computing the overlap between the genomic regions extracted from aligned unisequences and secreted protein coordinates.

**Table S2. Genome Overview**

| **Genome assembly - overall data** | |
| --- | --- |
| Assembly size (bp) | 848.548.110 |
| Number of scaffolds (> 1,000 bp) | 108.868 |
| N50 (bp) | 16.129 |
| Mean scaffold size (bp) | 7.794 |
| Largest scaffold (bp) | 205.555 |
| Genome coverage | 102x |

**Table S3. Secretion signal predictions of CAZy and PAD enzymes identified in metatranscriptomic from worker and soldier castes of *C. gestroi*.** The analysis was performed using *C. gestroi* (Method 1) and *Z. nevadensis* (Method 2) genomes as references, SignalP and WolF-PSORT were used for signal peptide prediction.

| **Unigenes** | **Cazy/PAD** | **Worker reads** | **Soldier reads** | **Taxonomy** | ***Signal Peptide prediction using C. gestroi* genome as reference** | ***Signal Peptide prediction using***  ***Z. nevandensis* genome as reference** |
| --- | --- | --- | --- | --- | --- | --- |
| gALL_v3_c103004 | AA1.3 | 3 | 1 | Insect |  | ✔ |
| gALL_v3_c5665 | AA1.3 | 5 | 6 | Insect |  |  |
| gALL_v3_c25111 | AA1.3 | 4 | 1 | Insect |  |  |
| gALL_v3_c1281 | AA1.3 | 23 | 2 | Insect |  | ✔ |
| gALL_v3_c1922 | AA1.3 | 4 | 18 | Insect |  |  |
| gALL_v3_c3217 | AA1.3 | 9 | 7 | Insect |  | ✔ |
| gALL_v3_c21180 | AA1.3 | 5 | 1 | Insect |  | ✔ |
| gALL_v3_rep_c99048 | AA1.3 | 3 | 3 | Insect |  | ✔ |
| gALL_v3_c11736 | AA1.3 | 12 | 3 | Insect | ✔ | ✔ |
| gALL_v3_c23176 | AA1.3 | 6 | 4 | Insect |  |  |
| gALL_v3_c13121 | AA1.3 | 9 | 3 | Insect |  | ✔ |
| gALL_v3_c87116 | AA1.3 | 2 | 2 | Insect | ✔ | ✔ |
| gALL_v3_c42628 | AA1.3 | 3 | 1 | Insect |  |  |
| gALL_v3_c7748 | AA1.3 | 17 | 5 | Insect |  | ✔ |
| gALL_v3_c57467 | AA3.2 | 6 | 22 | Insect | ✔ |  |
| gALL_v3_rep_c100069 | AA3.2 | 3 | 1 | Insect |  |  |
| gALL_v3_c17874 | AA3.2 | 3 | 13 | Insect | ✔ |  |
| gALL_v3_c10538 | AA3.2 | 9 | 7 | Insect |  |  |
| gALL_v3_c7596 | AA3.2 | 13 | 30 | Insect |  |  |
| gALL_v3_c2122 | AA3.2 | 22 | 10 | Insect | ✔ | ✔ |
| gALL_v3_c23184 | AA3.2 | 2 | 4 | Insect |  | ✔ |
| gALL_v3_c31002 | AA3.2 | 2 | 4 | Insect |  |  |
| gALL_v3_rep_c97604 | AA3.2 | 14 | 4 | Insect |  |  |
| gALL_v3_c5528 | AA3.2 | 9 | 17 | Insect |  |  |
| gALL_v3_rep_c109276 | AA3.2 | 3 | 1 | Insect |  |  |
| gALL_v3_c849 | AA3.2 | 19 | 4 | Insect |  |  |
| gALL_v3_c6368 | AA3.2 | 17 | 35 | Insect | ✔ |  |
| gALL_v3_c17646 | AA3.2 | 5 | 1 | Insect |  |  |
| gALL_v3_c17510 | AA3.2 | 3 | 4 | Protist |  |  |
| gALL_v3_c42370 | AA3.2 | 4 | 1 | Insect | ✔ |  |
| gALL_v3_c68242 | AA3.2 | 1 | 3 | Insect | ✔ |  |
| gALL_v3_rep_c98582 | AA3.2 | 3 | 5 | Protist |  |  |
| gALL_v3_c15863 | AA3.2 | 3 | 6 | Insect |  |  |
| gALL_v3_c4853 | AA3.2 | 1 | 12 | Insect |  |  |
| gALL_v3_c10164 | AA3.2 | 25 | 5 | Insect | ✔ |  |
| gALL_v3_c2472 | AA3.2 | 4 | 20 | Insect | ✔ |  |
| gALL_v3_rep_c105954 | AA3.2 | 1 | 4 | Insect | ✔ |  |
| gALL_v3_c103808 | AA3.2 | 3 | 3 | Insect | ✔ | ✔ |
| gALL_v3_c861 | AA3.2 | 15 | 18 | Insect |  |  |
| gALL_v3_c231 | AA3.2 | 7 | 10 | Insect |  |  |
| gALL_v3_c1659 | AA3.2 | 7 | 18 | Insect | ✔ |  |
| gALL_v3_c13704 | AA3.2 | 5 | 7 | Insect |  |  |
| gALL_v3_c1119 | AA3.2 | 25 | 20 | Insect | ✔ |  |
| gALL_v3_rep_c109053 | AA3.2 | 5 | 1 | Insect | ✔ |  |
| gALL_v3_rep_c97150 | AA3.2 | 10 | 27 | Insect | ✔ |  |
| gALL_v3_c49894 | AA3.2 | 4 | 8 | Insect | ✔ |  |
| gALL_v3_rep_c109537 | AA3.2 | 4 | 1 | Insect | ✔ |  |
| gALL_v3_c68421 | AA3.2 | 2 | 2 | Protist |  |  |
| gALL_v3_c1316 | AA3.2 | 6 | 36 | Insect |  |  |
| gALL_v3_rep_c99896 | AA3.2 | 1 | 4 | Insect |  |  |
| gALL_v3_c25769 | AA3.2 | 3 | 2 | Insect |  |  |
| gALL_v3_c68315 | AA3.2 | 4 | 2 | Insect |  |  |
| gALL_v3_c52860 | AA3.2 | 15 | 7 | Insect |  |  |
| gALL_v3_rep_c104195 | AA3.2 | 1 | 11 | Insect | ✔ |  |
| gALL_v3_c14523 | AA3.2 | 31 | 3 | Insect |  |  |
| gALL_v3_c24421 | AA3.2 | 2 | 4 | Insect | ✔ |  |
| gALL_v3_c17943 | AA3.2 | 9 | 4 | Insect | ✔ |  |
| gALL_v3_rep_c104823 | AA3.3 | 1 | 3 | Insect |  |  |
| gALL_v3_rep_c109773 | AA3.3 | 4 | 1 | Insect |  |  |
| gALL_v3_c1905 | AA3.3 | 3 | 7 | Insect | ✔ |  |
| gALL_v3_c62193 | AA3.3 | 3 | 2 | Insect |  |  |
| gALL_v3_c40346 | AA3.3 | 1 | 4 | Insect |  | ✔ |
| gALL_v3_rep_c99924 | AA3.3 | 2 | 3 | Insect | ✔ |  |
| gALL_v3_rep_c110445 | AA3.3 | 3 | 1 | Insect |  |  |
| gALL_v3_c19007 | AA3.3 | 3 | 3 | Insect |  | ✔ |
| gALL_v3_c55474 | AA3.3 | 2 | 3 | Insect |  |  |
| gALL_v3_c59013 | AA3.3 | 3 | 1 | Insect |  | ✔ |
| gALL_v3_c56078 | AA3.3 | 6 | 12 | Insect |  |  |
| gALL_v3_c29156 | AA3.3 | 5 | 3 | Insect |  | ✔ |
| gALL_v3_c89837 | AA3.3 | 3 | 3 | Insect |  |  |
| gALL_v3_rep_c99356 | AA3.3 | 3 | 3 | Insect |  |  |
| gALL_v3_rep_c109570 | AA3.3 | 3 | 1 | Insect | ✔ |  |
| gALL_v3_c10141 | AA4 | 6 | 6 | Insect |  |  |
| gALL_v3_c37298 | AA5.1 | 3 | 3 | Protist |  |  |
| gALL_v3_c1034 | AA5.2 | 10 | 2 | Protist |  |  |
| gALL_v3_c2305_4 | AA5.2 | 4 | 28 | Insect |  |  |
| gALL_v3_c4092_3 | AA5.2 | 43 | 28 | Insect |  |  |
| gALL_v3_c6234 | AA5.2 | 9 | 29 | Insect |  |  |
| gALL_v3_c41686 | AA5.2 | 2 | 3 | Insect | ✔ |  |
| gALL_v3_c16710 | AA5.2 | 7 | 2 | Insect |  |  |
| gALL_v3_c9519 | AA5.2 | 6 | 3 | Insect |  |  |
| gALL_v3_c52405 | AA5.2 | 2 | 3 | Insect |  |  |
| gALL_v3_rep_c98488 | AA6 | 6 | 1 | Insect |  |  |
| gALL_v3_c43994 | AA6 | 4 | 7 | Insect |  |  |
| gALL_v3_c78151 | AA8 | 2 | 2 | Insect | ✔ |  |
| gALL_v3_c17406 | AKR | 14 | 2 | Insect |  |  |
| gALL_v3_rep_c96865 | AKR | 44 | 13 | Insect |  |  |
| gALL_v3_c51060 | AKR | 2 | 5 | Insect |  |  |
| gALL_v3_rep_c104834 | AKR | 9 | 2 | Insect |  |  |
| gALL_v3_rep_c100464 | AKR | 2 | 0 | Insect |  |  |
| gALL_v3_rep_c109468 | AKR | 2 | 0 | Insect | ✔ |  |
| gALL_v3_rep_c98568 | AKR | 10 | 3 | Insect |  |  |
| gALL_v3_rep_c98481 | AKR | 7 | 2 | Insect |  |  |
| gALL_v3_c15542 | AKR | 4 | 4 | Insect |  |  |
| gALL_v3_rep_c99911 | AKR | 3 | 0 | Insect |  |  |
| gALL_v3_rep_c96328 | AKR | 98 | 58 | Insect | ✔ |  |
| gALL_v3_rep_c102252 | AKR | 1 | 1 | Insect |  |  |
| gALL_v3_rep_c97134 | AKR | 60 | 19 | Insect |  |  |
| gALL_v3_c50001 | AKR | 3 | 3 | Insect |  |  |
| gALL_v3_c57171 | AKR | 2 | 0 | Protist |  |  |
| gALL_v3_c62537 | AKR | 2 | 0 | Insect |  |  |
| gALL_v3_c45115 | AKR | 9 | 1 | Protist | ✔ |  |
| gALL_v3_rep_c100425 | AKR | 3 | 2 | Insect |  |  |
| gALL_v3_rep_c98155 | AKR | 6 | 5 | Insect | ✔ |  |
| gALL_v3_rep_c96122 | AKR | 479 | 323 | Insect |  |  |
| gALL_v3_c73469 | AKR | 1 | 1 | Insect | ✔ |  |
| gALL_v3_rep_c96584 | AKR | 22 | 23 | Insect |  |  |
| gALL_v3_c8209 | AKR | 6 | 2 | Insect |  | ✔ |
| gALL_v3_c15066 | AKR | 7 | 2 | Insect |  |  |
| gALL_v3_c1357 | AKR | 8 | 10 | Insect |  |  |
| gALL_v3_c30877 | AKR | 3 | 2 | Insect |  |  |
| gALL_v3_c6913 | CAT | 15 | 11 | Insect |  |  |
| gALL_v3_c10461 | CAT | 7 | 3 | Insect |  |  |
| gALL_v3_c6289 | CAT | 7 | 2 | Insect | ✔ | ✔ |
| gALL_v3_c25326 | CAT | 0 | 3 | Insect |  |  |
| gALL_v3_c24578 | CAT | 9 | 7 | Insect |  |  |
| gALL_v3_c46948 | CAT | 0 | 3 | Protist |  | ✔ |
| gALL_v3_rep_c98296 | CAT | 5 | 2 | Protist |  |  |
| gALL_v3_c16572 | CAT | 2 | 2 | Insect | ✔ |  |
| gALL_v3_c34612 | CAT | 4 | 7 | Protist |  |  |
| gALL_v3_rep_c96312 | CAT | 83 | 73 | Insect |  |  |
| gALL_v3_c28734 | CAT | 6 | 4 | Insect |  |  |
| gALL_v3_rep_c102491 | CAT | 1 | 1 | Protist |  |  |
| gALL_v3_c48664 | CAT | 10 | 13 | Protist |  |  |
| gALL_v3_rep_c102529 | CAT | 1 | 1 | Protist |  |  |
| gALL_v3_c50453 | CAT | 15 | 8 | Insect |  |  |
| gALL_v3_c12194 | CE1 | 6 | 9 | Insect |  |  |
| gALL_v3_c21716 | CE1 | 12 | 7 | Insect | ✔ |  |
| gALL_v3_c7182 | CE1 | 17 | 5 | Insect |  | ✔ |
| gALL_v3_c13199 | CE1 | 5 | 3 | Insect |  |  |
| gALL_v3_rep_c97360 | CE1 | 15 | 8 | Insect |  |  |
| gALL_v3_c4383 | CE1 | 15 | 11 | Protist |  |  |
| gALL_v3_c2091 | CE1 | 19 | 3 | Insect |  |  |
| gALL_v3_c20639 | CE1/CE10 | 3 | 5 | Insect |  |  |
| gALL_v3_c64020 | CE1/CE7 | 1 | 3 | Insect |  |  |
| gALL_v3_c26792 | CE1/CE7 | 2 | 4 | Insect |  |  |
| gALL_v3_c13039_2 | CE10 | 5 | 7 | Insect |  |  |
| gALL_v3_c35440 | CE10 | 2 | 4 | Insect |  | ✔ |
| gALL_v3_c3796 | CE10 | 10 | 33 | Insect | ✔ |  |
| gALL_v3_c26448 | CE10 | 12 | 9 | Insect |  |  |
| gALL_v3_c27938 | CE10 | 2 | 6 | Insect |  |  |
| gALL_v3_c87585 | CE10 | 2 | 2 | Insect | ✔ | ✔ |
| gALL_v3_c95933 | CE10 | 1 | 3 | Insect |  |  |
| gALL_v3_c17364 | CE10 | 5 | 2 | Insect | ✔ |  |
| gALL_v3_rep_c97555 | CE10 | 7 | 9 | Insect |  |  |
| gALL_v3_rep_c97115 | CE10 | 24 | 26 | Insect | ✔ | ✔ |
| gALL_v3_c39881 | CE10 | 3 | 3 | Insect |  |  |
| gALL_v3_c2143 | CE10 | 10 | 5 | Insect | ✔ |  |
| gALL_v3_c5239 | CE10 | 3 | 7 | Insect |  | ✔ |
| gALL_v3_c5328 | CE10 | 15 | 7 | Insect |  |  |
| gALL_v3_c9616 | CE10 | 6 | 2 | Insect |  | ✔ |
| gALL_v3_c6017 | CE10 | 16 | 9 | Insect |  | ✔ |
| gALL_v3_c31393 | CE10 | 3 | 2 | Insect | ✔ |  |
| gALL_v3_c4875 | CE10 | 14 | 6 | Insect | ✔ |  |
| gALL_v3_c15365 | CE10 | 3 | 8 | Insect |  |  |
| gALL_v3_c39235 | CE10 | 2 | 3 | Insect | ✔ |  |
| gALL_v3_c350 | CE10 | 22 | 12 | Insect |  |  |
| gALL_v3_c35839 | CE10 | 4 | 1 | Insect |  |  |
| gALL_v3_c94 | CE10 | 22 | 16 | Insect | ✔ |  |
| gALL_v3_rep_c97489 | CE10 | 13 | 2 | Insect |  | ✔ |
| gALL_v3_c44488 | CE10 | 3 | 1 | Insect |  |  |
| gALL_v3_c5925 | CE10 | 17 | 5 | Insect |  |  |
| gALL_v3_c2097 | CE10 | 16 | 5 | Insect |  |  |
| gALL_v3_c21767 | CE10 | 10 | 8 | Insect |  | ✔ |
| gALL_v3_c5472 | CE10 | 17 | 5 | Insect |  |  |
| gALL_v3_c50547 | CE10 | 3 | 1 | Insect |  | ✔ |
| gALL_v3_c23416 | CE10 | 4 | 2 | Insect |  |  |
| gALL_v3_rep_c96274 | CE10 | 150 | 47 | Insect | ✔ | ✔ |
| gALL_v3_c16609 | CE10 | 5 | 11 | Insect | ✔ |  |
| gALL_v3_c9362 | CE10 | 11 | 7 | Insect | ✔ | ✔ |
| gALL_v3_c31298 | CE10 | 3 | 3 | Insect |  |  |
| gALL_v3_c30320 | CE10 | 2 | 3 | Insect |  |  |
| gALL_v3_c14436 | CE10 | 6 | 5 | Insect |  | ✔ |
| gALL_v3_c19481 | CE13 | 6 | 1 | Insect | ✔ |  |
| gALL_v3_c32558 | CE14 | 3 | 2 | Insect |  |  |
| gALL_v3_c71738 | CE3 | 2 | 2 | Bacteria |  |  |
| gALL_v3_c7829 | CE3 | 14 | 20 | Protist |  |  |
| gALL_v3_c30003 | CE3 | 4 | 3 | Protist |  |  |
| gALL_v3_c82023 | CE3 | 1 | 3 | Insect |  |  |
| gALL_v3_rep_c96765 | CE4 | 15 | 25 | Insect |  | ✔ |
| gALL_v3_c26010 | CE4 | 3 | 3 | Insect |  |  |
| gALL_v3_c22564 | CE4 | 6 | 5 | Insect |  |  |
| gALL_v3_rep_c96427 | CE4 | 64 | 129 | Insect |  | ✔ |
| gALL_v3_c47557 | CE6 | 9 | 8 | Protist |  |  |
| gALL_v3_c12053 | CE7 | 2 | 7 | Bacteria |  |  |
| gALL_v3_c16734 | CE7 | 5 | 1 | Insect | ✔ |  |
| gALL_v3_c71 | CE7 | 16 | 13 | Insect |  |  |
| gALL_v3_rep_c109326 | GH1 | 12 | 1 | Insect |  |  |
| gALL_v3_c8970 | GH1 | 8 | 12 | Insect |  |  |
| gALL_v3_c47361 | GH1 | 1 | 6 | Insect |  |  |
| gALL_v3_c73139 | GH1 | 3 | 1 | Protist | ✔ | ✔ |
| gALL_v3_c406 | GH1 | 14 | 5 | Protist | ✔ | ✔ |
| gALL_v3_c22139 | GH1 | 3 | 4 | Insect |  |  |
| gALL_v3_c53472 | GH1 | 4 | 1 | Insect | ✔ |  |
| gALL_v3_c19427 | GH1 | 3 | 4 | Insect |  |  |
| gALL_v3_c6859 | GH1 | 5 | 8 | Insect |  | ✔ |
| gALL_v3_c6837 | GH1 | 7 | 12 | Insect |  | ✔ |
| gALL_v3_c4408 | GH1 | 7 | 6 | Insect | ✔ | ✔ |
| gALL_v3_c31043 | GH1 | 53 | 2 | Insect |  |  |
| gALL_v3_rep_c96194 | GH1 | 308 | 9 | Insect | ✔ |  |
| gALL_v3_rep_c101273 | GH1 | 1 | 3 | Protist |  |  |
| gALL_v3_c39216 | GH1 | 5 | 2 | Protist |  |  |
| gALL_v3_c3524 | GH1 | 3 | 12 | Insect | ✔ |  |
| gALL_v3_rep_c106998 | GH1 | 2 | 4 | Insect |  |  |
| gALL_v3_c34334 | GH1 | 2 | 4 | Insect |  |  |
| gALL_v3_c14689 | GH1 | 5 | 10 | Insect |  | ✔ |
| gALL_v3_c5824 | GH1 | 7 | 19 | Protist | ✔ |  |
| gALL_v3_c5263 | GH1 | 1 | 8 | Insect |  |  |
| gALL_v3_rep_c99830 | GH1 | 4 | 1 | Insect |  |  |
| gALL_v3_c73998 | GH1 | 2 | 2 | Insect | ✔ |  |
| gALL_v3_c27966 | GH1 | 5 | 4 | Insect |  |  |
| gALL_v3_rep_c98016 | GH10 | 10 | 5 | Protist |  |  |
| gALL_v3_c47557 | GH10 | 9 | 8 | Protist |  |  |
| gALL_v3_c33372 | GH10 | 7 | 6 | Protist |  |  |
| gALL_v3_rep_c98016 | CE6 | 10 | 5 | Protist |  |  |
| gALL_v3_rep_c109757 | GH11 | 6 | 1 | Protist |  |  |
| gALL_v3_c19833 | GH11 | 11 | 2 | Protist |  |  |
| gALL_v3_c21729 | GH11 | 2 | 4 | Protist |  |  |
| gALL_v3_c92168 | GH11 | 1 | 4 | Protist |  |  |
| gALL_v3_rep_c109073 | GH11 | 4 | 1 | Protist |  |  |
| gALL_v3_c6469_5 | GH13 | 12 | 13 | Insect |  | ✔ |
| gALL_v3_c23340 | GH13 | 17 | 5 | Insect |  |  |
| gALL_v3_c19061 | GH13 | 5 | 5 | Insect |  |  |
| gALL_v3_c14749 | GH13 | 3 | 5 | Insect |  |  |
| gALL_v3_c724 | GH13 | 8 | 12 | Insect |  |  |
| gALL_v3_rep_c96296 | GH13 | 148 | 36 | Insect |  | ✔ |
| gALL_v3_rep_c102219 | GH13 | 3 | 1 | Insect | ✔ |  |
| gALL_v3_rep_c97181 | GH13 | 47 | 2 | Insect | ✔ | ✔ |
| gALL_v3_c1869 | GH13 | 18 | 14 | Insect |  |  |
| gALL_v3_c86258 | GH13 | 3 | 1 | Insect |  |  |
| gALL_v3_c13984 | GH13 | 2 | 6 | Insect |  |  |
| gALL_v3_rep_c101825 | GH13 | 26 | 3 | Insect |  | ✔ |
| gALL_v3_c56260 | GH13 | 27 | 10 | Insect |  | ✔ |
| gALL_v3_rep_c101694 | GH13 | 2 | 2 | Insect | ✔ |  |
| gALL_v3_c11756 | GH13 | 3 | 8 | Insect |  |  |
| gALL_v3_rep_c96185 | GH13 | 358 | 8 | Insect | ✔ | ✔ |
| gALL_v3_rep_c110181 | GH13 | 3 | 1 | Insect |  |  |
| gALL_v3_rep_c97314 | GH13 | 18 | 7 | Insect |  | ✔ |
| gALL_v3_c15583 | GH13 | 3 | 7 | Insect |  |  |
| gALL_v3_c40990 | GH13 | 64 | 5 | Insect |  | ✔ |
| gALL_v3_c40979 | GH15 | 3 | 2 | Insect | ✔ |  |
| gALL_v3_c3655 | GH15 | 1 | 6 | Insect |  |  |
| gALL_v3_c45851 | GH15 | 2 | 3 | Insect |  |  |
| gALL_v3_c36633 | GH15 | 2 | 4 | Insect | ✔ |  |
| gALL_v3_c10857 | GH15 | 5 | 3 | Insect |  |  |
| gALL_v3_c39685 | GH15 | 3 | 1 | Insect |  |  |
| gALL_v3_c6757 | GH15 | 7 | 5 | Insect | ✔ |  |
| gALL_v3_rep_c108776 | GH16 | 6 | 1 | Insect |  | ✔ |
| gALL_v3_c80518 | GH16 | 16 | 3 | Insect |  | ✔ |
| gALL_v3_c20706 | GH16 | 5 | 1 | Insect |  |  |
| gALL_v3_rep_c96609 | GH16 | 64 | 2 | Insect |  | ✔ |
| gALL_v3_c12165 | GH16 | 3 | 14 | Insect |  | ✔ |
| gALL_v3_rep_c96441 | GH16 | 106 | 12 | Insect |  | ✔ |
| gALL_v3_c23332 | GH18 | 1 | 5 | Insect |  |  |
| gALL_v3_c30438 | GH18 | 2 | 6 | Insect | ✔ | ✔ |
| gALL_v3_c72188 | GH18 | 2 | 2 | Insect | ✔ |  |
| gALL_v3_rep_c96360 | GH18 | 112 | 95 | Insect | ✔ | ✔ |
| gALL_v3_c51574 | GH18 | 1 | 3 | Protist |  |  |
| gALL_v3_c19611 | GH18 | 7 | 2 | Insect | ✔ |  |
| gALL_v3_c44141 | GH18 | 3 | 1 | Insect |  |  |
| gALL_v3_c68281 | GH18 | 3 | 1 | Insect |  |  |
| gALL_v3_rep_c98342 | GH18 | 12 | 7 | Insect | ✔ | ✔ |
| gALL_v3_c15084 | GH18 | 10 | 6 | Protist | ✔ |  |
| gALL_v3_rep_c98101 | GH18 | 7 | 5 | Protist |  |  |
| gALL_v3_c40115 | GH18 | 9 | 21 | Insect | ✔ | ✔ |
| gALL_v3_c6396 | GH18 | 8 | 1 | Insect |  | ✔ |
| gALL_v3_c86593 | GH18 | 2 | 2 | Insect | ✔ |  |
| gALL_v3_c27174 | GH18 | 4 | 1 | Insect | ✔ | ✔ |
| gALL_v3_c55651 | GH18 | 2 | 2 | Insect |  |  |
| gALL_v3_c22830 | GH18 | 4 | 4 | Insect |  |  |
| gALL_v3_rep_c101289 | GH18 | 2 | 2 | Insect |  |  |
| gALL_v3_c12864 | GH18 | 5 | 2 | Insect |  |  |
| gALL_v3_c1310 | GH18 | 11 | 6 | Insect |  |  |
| gALL_v3_c8804 | GH18 | 6 | 5 | Insect | ✔ |  |
| gALL_v3_rep_c96475 | GH18 | 66 | 37 | Insect | ✔ | ✔ |
| gALL_v3_c5060 | GH18 | 14 | 4 | Insect |  |  |
| gALL_v3_c2491 | GH18 | 8 | 11 | Insect |  | ✔ |
| gALL_v3_rep_c96691 | GH18 | 50 | 15 | Insect | ✔ |  |
| >gALL_v3_c1701 | GH2 | 21 | 0 | Insect |  | ✔ |
| >gALL_v3_c19245 | GH2 | 4 | 0 | Insect |  | ✔ |
| gALL_v3_c7521 | GH2 | 11 | 6 | Insect |  | ✔ |
| gALL_v3_c44055 | GH2 | 3 | 2 | Insect |  | ✔ |
| gALL_v3_c495 | GH20 | 15 | 13 | Insect | ✔ | ✔ |
| gALL_v3_c12729 | GH20 | 4 | 5 | Insect |  | ✔ |
| gALL_v3_c55557 | GH20 | 3 | 2 | Insect |  | ✔ |
| gALL_v3_c41506 | GH20 | 3 | 2 | Insect |  |  |
| gALL_v3_c25910 | GH20 | 4 | 1 | Insect |  |  |
| gALL_v3_c87355 | GH20 | 2 | 2 | Protist |  |  |
| gALL_v3_rep_c96935 | GH25 | 18 | 4 | Insect | ✔ |  |
| gALL_v3_rep_c98435 | GH25 | 36 | 20 | Protist |  |  |
| gALL_v3_c9899 | GH25 | 6 | 4 | Insect |  |  |
| gALL_v3_c22719 | GH25 | 13 | 3 | Insect | ✔ |  |
| gALL_v3_c76498 | GH25 | 3 | 1 | Insect | ✔ |  |
| gALL_v3_rep_c106212 | GH25 | 8 | 3 | Protist |  |  |
| gALL_v3_rep_c99233 | GH25 | 4 | 2 | Insect |  |  |
| gALL_v3_c14010 | GH25 | 6 | 4 | Insect |  |  |
| gALL_v3_rep_c97307 | GH25 | 15 | 3 | Protist | ✔ |  |
| gALL_v3_c12188 | GH25 | 3 | 9 | Insect |  | ✔ |
| gALL_v3_rep_c104529 | GH25 | 4 | 9 | Protist |  |  |
| gALL_v3_rep_c96991 | GH25 | 25 | 8 | Protist |  |  |
| gALL_v3_rep_c96246 | GH25 | 95 | 27 | Protist |  |  |
| gALL_v3_rep_c104469 | GH25 | 6 | 6 | Protist |  |  |
| gALL_v3_rep_c109272 | GH25 | 3 | 1 | Insect | ✔ |  |
| gALL_v3_c22174 | GH25 | 5 | 3 | Protist |  |  |
| gALL_v3_rep_c107576 | GH25 | 3 | 2 | Insect |  |  |
| gALL_v3_c37810_4 | GH26 | 3 | 0 | Bacteria | ✔ |  |
| gALL_v3_c12053 | GH26 | 2 | 7 | Bacteria |  |  |
| gALL_v3_c6812 | GH27 | 8 | 9 | Insect | ✔ |  |
| gALL_v3_c64338 | GH27 | 3 | 1 | Insect | ✔ | ✔ |
| gALL_v3_c472 | GH29 | 21 | 6 | Insect | ✔ | ✔ |
| gALL_v3_c13414 | GH3 | 5 | 5 | Bacteria |  |  |
| gALL_v3_c84856 | GH3 | 2 | 2 | Bacteria |  |  |
| gALL_v3_c17124 | GH3 | 12 | 1 | Bacteria |  |  |
| >gALL_v3_c14740 | GH3 |  |  | Bacteria |  |  |
| gALL_v3_c18055 | GH30 | 5 | 2 | Insect |  |  |
| gALL_v3_c2568 | GH30 | 9 | 7 | Insect |  |  |
| gALL_v3_c33925 | GH30 | 2 | 3 | Insect |  | ✔ |
| gALL_v3_c15261 | GH31 | 3 | 5 | Insect |  |  |
| gALL_v3_c804 | GH31 | 12 | 13 | Insect | ✔ | ✔ |
| gALL_v3_c49985 | GH31 | 3 | 1 | Protist |  |  |
| gALL_v3_c35845 | GH31 | 4 | 4 | Protist |  |  |
| gALL_v3_c26313 | GH31 | 4 | 2 | Insect |  |  |
| gALL_v3_c4930 | GH31 | 6 | 5 | Insect |  |  |
| gALL_v3_c2845 | GH31 | 18 | 26 | Insect |  |  |
| gALL_v3_c518 | GH31 | 17 | 25 | Insect |  |  |
| gALL_v3_rep_c98398 | GH31 | 4 | 5 | Insect |  |  |
| gALL_v3_c2190 | GH31 | 14 | 16 | Insect | ✔ | ✔ |
| gALL_v3_c48303 | GH31 | 2 | 2 | Insect |  |  |
| gALL_v3_c2770 | GH33 | 20 | 31 | Insect |  | ✔ |
| gALL_v3_c92922 | GH35 | 2 | 2 | Insect |  |  |
| gALL_v3_c32728 | GH35 | 4 | 3 | Insect |  |  |
| gALL_v3_c11076_6 | GH37 | 4 | 9 | Insect |  | ✔ |
| gALL_v3_c76932 | GH37 | 4 | 1 | Insect |  | ✔ |
| gALL_v3_c2612 | GH37 | 5 | 8 | Insect |  | ✔ |
| gALL_v3_c54076 | GH37 | 2 | 5 | Insect |  |  |
| gALL_v3_c2954 | GH37 | 8 | 13 | Insect |  | ✔ |
| gALL_v3_c9090 | GH37 | 14 | 13 | Insect |  |  |
| gALL_v3_c47251 | GH37 | 3 | 2 | Insect |  |  |
| gALL_v3_rep_c96866 | GH37 | 28 | 24 | Insect |  |  |
| gALL_v3_c45304 | GH37 | 1 | 3 | Insect |  |  |
| gALL_v3_c95732 | GH38 | 2 | 0 | Insect |  | ✔ |
| gALL_v3_c91363 | GH38 | 1 | 3 | Insect |  |  |
| gALL_v3_c30683 | GH38 | 1 | 7 | Insect |  |  |
| gALL_v3_c53516 | GH38 | 3 | 4 | Insect |  |  |
| gALL_v3_c51008 | GH38 | 1 | 5 | Insect |  |  |
| gALL_v3_c698 | GH38 | 35 | 8 | Insect | ✔ | ✔ |
| gALL_v3_c95947 | GH39 | 2 | 2 | Protist |  |  |
| gALL_v3_rep_c96321 | GH39 | 267 | 9 | Insect |  | ✔ |
| gALL_v3_c17434 | GH45 | 5 | 5 | Protist |  |  |
| gALL_v3_c9718 | GH47 | 4 | 10 | Insect |  |  |
| gALL_v3_c26144 | GH47 | 4 | 3 | Insect |  |  |
| gALL_v3_c45995 | GH47 | 3 | 3 | Insect |  |  |
| gALL_v3_c42503 | GH47 | 1 | 3 | Insect |  |  |
| gALL_v3_c9787 | GH47 | 4 | 4 | Insect |  |  |
| gALL_v3_c23225 | GH47 | 4 | 2 | Insect |  |  |
| gALL_v3_c16854 | GH47 | 5 | 5 | Insect |  |  |
| gALL_v3_c5927 | GH47 | 4 | 10 | Insect | ✔ |  |
| gALL_v3_c93333 | GH47 | 1 | 3 | Insect |  |  |
| gALL_v3_c7295 | GH47 | 6 | 5 | Insect |  |  |
| gALL_v3_c91382 | GH47 | 1 | 3 | Insect |  |  |
| gALL_v3_c9520 | GH47 | 11 | 10 | Protist |  | ✔ |
| gALL_v3_c47730 | GH47 | 3 | 2 | Insect |  |  |
| gALL_v3_c30054 | GH47 | 2 | 3 | Insect |  |  |
| gALL_v3_c34652 | GH47 | 2 | 4 | Insect |  |  |
| gALL_v3_c67010 | GH5 | 2 | 2 | Protist |  |  |
| gALL_v3_c25216 | GH5 | 2 | 7 | Protist |  |  |
| gALL_v3_c74145 | GH5 | 1 | 3 | Bacteria |  |  |
| gALL_v3_c2080 | GH5 | 5 | 9 | Protist |  |  |
| gALL_v3_c35156 | GH5 | 3 | 1 | Protist |  |  |
| gALL_v3_c19701 | GH5 | 3 | 3 | Bacteria |  |  |
| gALL_v3_c95551 | GH5 | 2 | 2 | Protist |  |  |
| gALL_v3_c4449 | GH5 | 11 | 7 | Bacteria |  |  |
| gALL_v3_c81226 | GH5 | 3 | 1 | Bacteria |  |  |
| gALL_v3_c1472 | GH5 | 6 | 13 | Protist | ✔ |  |
| gALL_v3_c15620 | GH5 | 2 | 4 | Protist |  |  |
| gALL_v3_c52718 | GH56 | 2 | 3 | Insect |  |  |
| gALL_v3_c58055 | GH63 | 1 | 5 | Insect |  |  |
| gALL_v3_c39558 | GH63 | 2 | 3 | Insect |  |  |
| gALL_v3_c84791 | GH63 | 2 | 2 | Insect |  |  |
| gALL_v3_c75681 | GH7 | 9 | 9 | Protist |  |  |
| gALL_v3_c86791 | GH7 | 3 | 1 | Protist |  |  |
| gALL_v3_rep_c98019 | GH7 | 4 | 7 | Protist |  |  |
| gALL_v3_c43521 | GH7 | 3 | 1 | Protist |  |  |
| gALL_v3_c25934 | GH7 | 1 | 8 | Protist |  |  |
| gALL_v3_c30211 | GH7 | 6 | 9 | Protist |  |  |
| gALL_v3_c37141 | GH7 | 1 | 3 | Protist |  |  |
| gALL_v3_rep_c96913 | GH7 | 31 | 27 | Protist |  |  |
| gALL_v3_c67096 | GH7 | 2 | 2 | Protist |  |  |
| gALL_v3_rep_c97033 | GH7 | 16 | 26 | Insect |  |  |
| gALL_v3_c5973 | GH7 | 16 | 2 | Protist |  |  |
| gALL_v3_c3924 | GH7 | 7 | 1 | Protist |  |  |
| gALL_v3_rep_c97276 | GH7 | 9 | 14 | Protist |  |  |
| gALL_v3_c38366 | GH7 | 3 | 1 | Protist |  |  |
| gALL_v3_c75250 | GH7 | 1 | 3 | Protist |  |  |
| gALL_v3_c86982 | GH7 | 1 | 3 | Protist |  |  |
| gALL_v3_c12313 | GH7 | 9 | 1 | Protist |  |  |
| gALL_v3_c90662 | GH7 | 3 | 1 | Protist |  |  |
| gALL_v3_c14652 | GH8 | 7 | 9 | Bacteria |  |  |
| gALL_v3_c32329 | GH85 | 7 | 2 | Protist | ✔ |  |
| gALL_v3_c42131 | GH85 | 2 | 3 | Insect |  |  |
| gALL_v3_c74246 | GH85 | 2 | 2 | Protist |  |  |
| gALL_v3_c82461 | GH89 | 1 | 3 | Insect |  |  |
| gALL_v3_c77849 | GH89 | 2 | 3 | Insect |  | ✔ |
| gALL_v3_c82449 | GH89 | 3 | 1 | Insect |  |  |
| gALL_v3_rep_c96569 | GH9 | 53 | 1 | Insect |  |  |
| gALL_v3_rep_c100937 | GH9 | 3 | 1 | Insect |  |  |
| gALL_v3_c31840 | GH9 | 3 | 3 | Insect |  |  |
| gALL_v3_c103451 | GH9 | 2 | 2 | Insect |  |  |
| gALL_v3_c2977 | GH9 | 10 | 4 | Insect |  |  |
| gALL_v3_rep_c100194 | GH9 | 3 | 1 | Insect |  |  |
| gALL_v3_rep_c100791 | GH9 | 3 | 1 | Insect |  |  |
| gALL_v3_rep_c100884 | GH9 | 3 | 1 | Insect |  |  |
| gALL_v3_c8280 | GH9 | 2 | 10 | Insect |  |  |
| gALL_v3_rep_c99594 | GH9 | 5 | 1 | Insect |  |  |
| gALL_v3_rep_c99388 | GH9 | 4 | 1 | Insect |  |  |
| gALL_v3_rep_c108719 | GH9 | 56 | 1 | Insect |  |  |
| gALL_v3_rep_c100549 | GH9 | 3 | 1 | Insect |  |  |
| gALL_v3_rep_c100333 | GH9 | 3 | 1 | Insect |  |  |
| gALL_v3_rep_c99712 | GH9 | 3 | 1 | Insect |  |  |
| gALL_v3_rep_c101498 | GH9 | 3 | 1 | Insect |  |  |
| gALL_v3_c18687 | GH9 | 2 | 4 | Insect | ✔ | ✔ |
| gALL_v3_c7492 | GH9 | 97 | 2 | Insect |  |  |
| gALL_v3_rep_c100427 | GH9 | 3 | 1 | Insect |  |  |
| gALL_v3_rep_c99799 | GH9 | 3 | 1 | Insect |  |  |
| gALL_v3_rep_c98165 | GH9 | 6 | 1 | Insect |  |  |
| gALL_v3_rep_c100368 | GH9 | 3 | 1 | Insect |  |  |
| gALL_v3_rep_c100779 | GH9 | 3 | 1 | Insect |  |  |
| gALL_v3_c92849 | GH9 | 1 | 3 | Insect |  |  |
| gALL_v3_rep_c100536 | GH9 | 3 | 1 | Insect |  |  |
| gALL_v3_rep_c99814 | GH9 | 4 | 1 | Insect |  |  |
| gALL_v3_c31035 | GH9 | 5 | 2 | Insect |  |  |
| gALL_v3_rep_c101386 | GH9 | 3 | 1 | Insect | ✔ |  |
| gALL_v3_c27166 | GH9 | 1 | 5 | Insect |  | ✔ |
| gALL_v3_rep_c101118 | GH9 | 3 | 1 | Insect |  |  |
| gALL_v3_c38489 | GH9 | 5 | 2 | Insect |  |  |
| gALL_v3_rep_c99326 | GH9 | 37 | 2 | Insect |  |  |
| gALL_v3_rep_c98622 | GH9 | 4 | 2 | Insect |  |  |
| gALL_v3_rep_c100258 | GH9 | 3 | 1 | Insect |  |  |
| gALL_v3_rep_c100083 | GH9 | 3 | 1 | Insect |  |  |
| gALL_v3_rep_c96171 | GH9 | 321 | 4 | Insect |  |  |
| gALL_v3_rep_c99644 | GH9 | 4 | 1 | Insect |  |  |
| gALL_v3_c100877 | GH9 | 3 | 1 | Insect |  |  |
| gALL_v3_rep_c100234 | GH9 | 3 | 1 | Insect |  |  |
| gALL_v3_rep_c96486 | GH9 | 60 | 8 | Insect |  | ✔ |
| gALL_v3_rep_c96106 | GH9 | 1202 | 74 | Insect |  |  |
| gALL_v3_c21060 | GST | 6 | 18 | Insect |  |  |
| gALL_v3_rep_c97370 | GST | 13 | 14 | Insect | ✔ |  |
| gALL_v3_rep_c97447 | GST | 12 | 13 | Insect | ✔ |  |
| gALL_v3_c37644 | GST | 5 | 7 | Insect |  |  |
| gALL_v3_rep_c96354 | GST | 59 | 3 | Insect |  |  |
| gALL_v3_c4114 | GST | 13 | 24 | Insect |  |  |
| gALL_v3_c55797 | GST | 0 | 3 | Insect |  |  |
| gALL_v3_c4492 | GST | 6 | 2 | Insect |  |  |
| gALL_v3_c57001 | GST | 4 | 5 | Insect |  |  |
| gALL_v3_c38851 | GST | 6 | 7 | Protist |  |  |
| gALL_v3_rep_c99593 | GST | 3 | 1 | Insect | ✔ |  |
| gALL_v3_rep_c96308 | GST | 101 | 58 | Insect |  |  |
| gALL_v3_c51608 | GST | 1 | 1 | Insect |  |  |
| gALL_v3_c6961 | GST | 5 | 8 | Insect |  |  |
| gALL_v3_c7780 | GST | 8 | 8 | Insect | ✔ |  |
| gALL_v3_c95079 | GST | 13 | 7 | Insect |  |  |
| gALL_v3_c12300 | GST | 11 | 10 | Insect | ✔ |  |
| gALL_v3_rep_c96638 | GST | 18 | 17 | Insect |  | ✔ |
| gALL_v3_c26849 | GST | 2 | 3 | Insect | ✔ |  |
| gALL_v3_rep_c96540 | GST | 35 | 17 | Protist |  | ✔ |
| gALL_v3_c37119 | GST | 24 | 17 | Insect |  |  |
| gALL_v3_c40875 | GST | 1 | 2 | Insect |  |  |
| gALL_v3_rep_c96283 | GST | 73 | 34 | Insect |  |  |
| gALL_v3_c28753 | GST | 6 | 15 | Insect |  |  |
| gALL_v3_c50201 | GST | 1 | 1 | Insect | ✔ |  |
| gALL_v3_c4709 | GST | 7 | 11 | Insect | ✔ |  |
| gALL_v3_c421 | GST | 10 | 13 | Insect |  |  |
| gALL_v3_c37348 | GST | 1 | 5 | Insect |  |  |
| gALL_v3_rep_c97567 | GST | 13 | 3 | Insect |  |  |
| gALL_v3_rep_c98225 | GST | 2 | 5 | Insect |  |  |
| gALL_v3_c8401 | GST | 5 | 2 | Insect | ✔ |  |
| gALL_v3_rep_c103082 | GST | 2 | 0 | Insect |  |  |
| gALL_v3_c95130 | GST | 3 | 0 | Insect |  |  |
| gALL_v3_c80050 | GST | 1 | 1 | Insect |  |  |
| gALL_v3_rep_c97975 | GST | 5 | 6 | Insect |  |  |
| gALL_v3_rep_c102382 | GST | 1 | 1 | Insect |  |  |
| gALL_v3_c2087 | GST | 7 | 5 | Insect |  |  |
| gALL_v3_c44137 | GST | 1 | 1 | Insect |  |  |
| gALL_v3_c9932 | GST | 8 | 3 | Insect |  | ✔ |
| gALL_v3_c20803 | GST | 1 | 7 | Protist |  |  |
| gALL_v3_c77365 | GST | 5 | 6 | Insect | ✔ |  |
| gALL_v3_rep_c100108 | GST | 1 | 2 | Insect |  |  |
| gALL_v3_rep_c100815 | p450 | 0 | 19 | Insect |  |  |
| gALL_v3_rep_c98367 | p450 | 19 | 22 | Insect |  |  |
| gALL_v3_c13210 | p450 | 4 | 2 | Insect |  |  |
| gALL_v3_rep_c102646 | p450 | 2 | 0 | Insect |  |  |
| gALL_v3_c12037 | p450 | 12 | 7 | Insect |  |  |
| gALL_v3_c12721 | p450 | 1 | 12 | Insect |  |  |
| gALL_v3_c12515 | p450 | 14 | 8 | Insect |  |  |
| gALL_v3_c20090 | p450 | 3 | 6 | Insect |  |  |
| gALL_v3_c11806 | p450 | 4 | 5 | Insect |  |  |
| gALL_v3_c10091 | p450 | 31 | 12 | Insect |  |  |
| gALL_v3_c49001 | p450 | 3 | 2 | Insect | ✔ |  |
| gALL_v3_c16218 | p450 | 7 | 8 | Insect |  |  |
| gALL_v3_c29520 | p450 | 3 | 6 | Insect | ✔ |  |
| gALL_v3_rep_c105597 | p450 | 1 | 2 | Insect |  |  |
| gALL_v3_rep_c96463 | p450 | 105 | 66 | Insect |  |  |
| gALL_v3_rep_c96955 | p450 | 29 | 134 | Insect | ✔ |  |
| gALL_v3_c12547 | p450 | 6 | 12 | Insect |  | ✔ |
| gALL_v3_c21353 | p450 | 3 | 0 | Insect |  |  |
| gALL_v3_rep_c104091 | p450 | 2 | 6 | Protist |  |  |
| gALL_v3_rep_c109150 | p450 | 2 | 0 | Insect |  |  |
| gALL_v3_rep_c100783 | p450 | 2 | 2 | Insect |  |  |
| gALL_v3_c2761 | p450 | 11 | 5 | Insect |  |  |
| gALL_v3_rep_c97280 | p450 | 7 | 11 | Insect | ✔ |  |
| gALL_v3_c22892 | p450 | 2 | 1 | Insect |  |  |
| gALL_v3_c20547 | p450 | 2 | 4 | Insect | ✔ |  |
| gALL_v3_c51039 | p450 | 3 | 8 | Insect |  |  |
| gALL_v3_c31541 | p450 | 3 | 2 | Insect |  |  |
| gALL_v3_rep_c104343 | p450 | 2 | 4 | Insect |  |  |
| gALL_v3_c67914 | p450 | 3 | 7 | Insect |  |  |
| gALL_v3_c21848 | p450 | 4 | 9 | Insect | ✔ |  |
| gALL_v3_c49146 | p450 | 0 | 3 | Insect |  |  |
| gALL_v3_c10760 | p450 | 5 | 1 | Insect |  | ✔ |
| gALL_v3_c81640 | p450 | 1 | 4 | Insect |  |  |
| gALL_v3_rep_c97444 | p450 | 14 | 24 | Insect |  |  |
| gALL_v3_rep_c97898 | p450 | 7 | 12 | Insect |  |  |
| gALL_v3_c104389 | p450 | 13 | 11 | Insect |  |  |
| gALL_v3_c75867 | p450 | 0 | 8 | Insect | ✔ |  |
| gALL_v3_rep_c106784 | p450 | 4 | 1 | Insect |  |  |
| gALL_v3_c25292 | p450 | 7 | 8 | Insect |  |  |
| gALL_v3_rep_c99247 | p450 | 10 | 26 | Insect |  |  |
| gALL_v3_c21785 | p450 | 3 | 1 | Insect |  |  |
| gALL_v3_rep_c98105 | p450 | 9 | 42 | Insect |  |  |
| gALL_v3_rep_c109804 | p450 | 2 | 0 | Insect |  |  |
| gALL_v3_rep_c107452 | p450 | 3 | 2 | Insect |  |  |
| gALL_v3_c71631 | p450 | 0 | 2 | Insect |  |  |
| gALL_v3_c4199 | p450 | 5 | 6 | Insect |  |  |
| gALL_v3_c45838 | p450 | 9 | 4 | Insect | ✔ |  |
| gALL_v3_rep_c105108 | p450 | 1 | 3 | Insect |  |  |
| gALL_v3_c77209 | p450 | 5 | 5 | Insect |  |  |
| gALL_v3_c2488 | p450 | 19 | 14 | Insect |  |  |
| gALL_v3_rep_c104736 | p450 | 1 | 2 | Insect | ✔ |  |
| gALL_v3_c62802 | p450 | 7 | 4 | Insect |  |  |
| gALL_v3_rep_c98345 | p450 | 5 | 4 | Insect |  |  |
| gALL_v3_rep_c104108 | p450 | 10 | 6 | Insect |  |  |
| gALL_v3_rep_c96280 | p450 | 220 | 121 | Insect |  |  |
| gALL_v3_c5200 | p450 | 12 | 2 | Insect |  |  |
| gALL_v3_c2709 | p450 | 6 | 14 | Insect |  | ✔ |
| gALL_v3_rep_c109308 | p450 | 2 | 0 | Insect |  |  |
| gALL_v3_c13533 | p450 | 10 | 4 | Insect |  |  |
| gALL_v3_rep_c97749 | p450 | 0 | 12 | Insect |  |  |
| gALL_v3_rep_c97456 | p450 | 9 | 29 | Protist |  |  |
| gALL_v3_c88712 | p450 | 0 | 2 | Insect | ✔ |  |
| gALL_v3_c633 | p450 | 13 | 3 | Insect | ✔ |  |
| gALL_v3_c46598 | p450 | 3 | 0 | Insect |  |  |
| gALL_v3_rep_c109014 | p450 | 5 | 0 | Insect |  |  |
| gALL_v3_rep_c96176 | p450 | 210 | 445 | Insect |  |  |
| gALL_v3_c50788 | p450 | 0 | 3 | Insect |  |  |
| gALL_v3_rep_c97509 | p450 | 9 | 7 | Insect |  |  |
| gALL_v3_rep_c96439 | p450 | 92 | 42 | Insect | ✔ |  |
| gALL_v3_rep_c98721 | p450 | 4 | 1 | Insect | ✔ |  |
| gALL_v3_rep_c105767 | p450 | 6 | 2 | Insect | ✔ |  |
| gALL_v3_rep_c104501 | p450 | 2 | 5 | Insect | ✔ |  |
| gALL_v3_rep_c104228 | p450 | 0 | 2 | Insect |  |  |
| gALL_v3_rep_c103103 | p450 | 0 | 2 | Insect |  |  |
| gALL_v3_c63773 | p450 | 3 | 6 | Insect |  |  |
| gALL_v3_c21290 | p450 | 4 | 1 | Insect |  |  |
| gALL_v3_c19791 | p450 | 9 | 0 | Insect |  |  |
| gALL_v3_rep_c104020 | p450 | 1 | 3 | Insect |  |  |
| gALL_v3_c76268 | p450 | 4 | 11 | Insect |  |  |
| gALL_v3_rep_c97956 | p450 | 10 | 5 | Insect | ✔ |  |
| gALL_v3_c52492 | p450 | 4 | 2 | Insect |  | ✔ |
| gALL_v3_c9294 | p450 | 0 | 23 | Insect | ✔ |  |
| gALL_v3_c40802 | p450 | 7 | 1 | Insect | ✔ |  |
| gALL_v3_rep_c107806 | p450 | 1 | 1 | Insect | ✔ |  |
| gALL_v3_rep_c104130 | p450 | 0 | 3 | Protist |  |  |
| gALL_v3_c19121 | p450 | 3 | 5 | Insect | ✔ |  |
| gALL_v3_rep_c107507 | p450 | 1 | 1 | Insect |  |  |
| gALL_v3_c11102 | p450 | 7 | 9 | Insect |  |  |
| gALL_v3_c46657 | p450 | 17 | 4 | Insect |  |  |
| gALL_v3_c22588 | p450 | 5 | 2 | Insect | ✔ |  |
| gALL_v3_c20076 | p450 | 0 | 4 | Insect |  |  |
| gALL_v3_c2294 | p450 | 2 | 18 | Insect |  |  |
| gALL_v3_c13784 | p450 | 5 | 3 | Insect |  | ✔ |
| gALL_v3_rep_c96832 | p450 | 19 | 16 | Insect |  | ✔ |
| gALL_v3_rep_c109226 | p450 | 2 | 0 | Protist |  |  |
| gALL_v3_c8209 | p450 | 6 | 2 | Insect |  | ✔ |
| gALL_v3_c91520 | p450 | 1 | 1 | Insect |  |  |
| gALL_v3_c3054 | p450 | 14 | 7 | Insect | ✔ |  |
| gALL_v3_c82528 | p450 | 0 | 2 | Insect |  |  |
| gALL_v3_rep_c97498 | p450 | 33 | 19 | Insect |  |  |
| gALL_v3_c70716 | p450 | 4 | 4 | Insect |  | ✔ |
| gALL_v3_rep_c97350 | p450 | 6 | 17 | Protist | ✔ |  |
| gALL_v3_rep_c104316 | p450 | 8 | 6 | Insect |  |  |
| gALL_v3_c6227 | p450 | 2 | 29 | Insect |  | ✔ |
| gALL_v3_c29187 | p450 | 2 | 1 | Protist |  |  |
| gALL_v3_c5836 | p450 | 9 | 15 | Insect | ✔ |  |
| gALL_v3_rep_c104402 | p450 | 0 | 3 | Insect |  |  |
| gALL_v3_c94098 | p450 | 1 | 1 | Insect |  |  |
| gALL_v3_c82401 | p450 | 7 | 7 | Insect | ✔ |  |
| gALL_v3_rep_c96479 | p450 | 131 | 41 | Insect |  |  |
| gALL_v3_c34782 | p450 | 4 | 3 | Insect |  |  |
| gALL_v3_c6645 | p450 | 8 | 5 | Insect |  |  |
| gALL_v3_rep_c96302 | p450 | 305 | 162 | Insect | ✔ |  |
| gALL_v3_c4879 | p450 | 22 | 28 | Insect |  |  |
| gALL_v3_c23757 | p450 | 5 | 4 | Insect |  |  |
| gALL_v3_c6997 | p450 | 7 | 0 | Insect |  |  |
| gALL_v3_c34861 | p450 | 2 | 2 | Insect |  |  |
| gALL_v3_c32779 | p450 | 2 | 2 | Insect |  |  |
| gALL_v3_rep_c96715 | p450 | 14 | 88 | Insect |  |  |
| gALL_v3_c13535 | p450 | 3 | 3 | Insect |  | ✔ |
| gALL_v3_rep_c96695 | p450 | 57 | 16 | Insect |  |  |
| gALL_v3_c44828 | p450 | 0 | 4 | Protist |  |  |
| gALL_v3_c17236 | p450 | 15 | 4 | Insect |  |  |
| gALL_v3_c91708 | p450 | 4 | 2 | Insect |  |  |
| gALL_v3_c17683 | p450 | 2 | 2 | Insect | ✔ |  |
| gALL_v3_c22306 | p450 | 0 | 13 | Insect |  |  |
| gALL_v3_c51552 | p450 | 2 | 11 | Insect |  |  |
| gALL_v3_c24754 | p450 | 7 | 5 | Insect |  |  |
| gALL_v3_rep_c100396 | p450 | 0 | 3 | Insect |  |  |
| gALL_v3_c8974 | p450 | 9 | 12 | Insect | ✔ |  |
| gALL_v3_c10387 | p450 | 4 | 3 | Insect | ✔ |  |
| gALL_v3_c87195 | p450 | 1 | 2 | Insect |  |  |
| gALL_v3_c17007 | p450 | 3 | 2 | Protist |  |  |
| gALL_v3_c12261 | p450 | 3 | 16 | Insect |  |  |
| gALL_v3_rep_c105468 | p450 | 3 | 9 | Insect |  |  |
| gALL_v3_c15065 | p450 | 4 | 3 | Insect |  |  |
| gALL_v3_c8761 | p450 | 7 | 5 | Insect | ✔ |  |
| gALL_v3_rep_c97365 | p450 | 8 | 7 | Insect |  |  |
| gALL_v3_c27872 | p450 | 3 | 4 | Insect |  |  |
| gALL_v3_c28701 | p450 | 5 | 3 | Insect | ✔ |  |
| gALL_v3_rep_c110182 | p450 | 2 | 0 | Insect |  | ✔ |
| gALL_v3_c4497 | p450 | 0 | 19 | Insect |  |  |
| gALL_v3_c72832 | p450 | 8 | 16 | Insect |  |  |
| gALL_v3_c21401 | p450 | 0 | 10 | Insect |  |  |
| gALL_v3_c11937 | p450 | 0 | 19 | Insect |  |  |
| gALL_v3_c77636 | p450 | 1 | 1 | Insect |  |  |
| gALL_v3_rep_c96511 | p450 | 0 | 79 | Insect |  |  |
| gALL_v3_c17878 | p450 | 7 | 12 | Insect |  |  |
| gALL_v3_rep_c97521 | p450 | 25 | 18 | Insect | ✔ | ✔ |
| gALL_v3_c15420 | p450 | 6 | 3 | Insect |  |  |
| gALL_v3_rep_c98232 | p450 | 5 | 5 | Insect |  |  |
| gALL_v3_c2418 | p450 | 21 | 1 | Insect | ✔ |  |
| gALL_v3_c19761 | p450 | 3 | 2 | Insect |  |  |
| gALL_v3_rep_c98683 | p450 | 6 | 1 | Insect |  |  |
| gALL_v3_rep_c108102 | p450 | 2 | 1 | Insect |  |  |
| gALL_v3_rep_c99777 | p450 | 10 | 22 | Insect |  |  |
| gALL_v3_rep_c97141 | p450 | 21 | 68 | Insect |  |  |
| gALL_v3_rep_c105528 | p450 | 3 | 1 | Insect |  |  |
| gALL_v3_c38424 | p450 | 2 | 1 | Insect | ✔ |  |
| gALL_v3_c2002 | p450 | 5 | 8 | Insect |  |  |
| gALL_v3_rep_c108126 | p450 | 6 | 1 | Insect | ✔ |  |
| gALL_v3_c57680 | p450 | 0 | 3 | Insect |  |  |
| gALL_v3_c89304 | p450 | 2 | 0 | Insect | ✔ |  |
| gALL_v3_c12861 | p450 | 10 | 5 | Insect |  |  |
| gALL_v3_c1180 | p450 | 22 | 7 | Insect |  | ✔ |
| gALL_v3_c14963 | p450 | 7 | 12 | Insect |  |  |
| gALL_v3_c134 | p450 | 40 | 14 | Insect |  |  |
| gALL_v3_rep_c97210 | p450 | 0 | 21 | Insect |  |  |
| gALL_v3_c13132 | p450 | 5 | 8 | Insect |  |  |
| gALL_v3_rep_c109594 | p450 | 2 | 0 | Insect |  |  |
| gALL_v3_rep_c97343 | p450 | 31 | 13 | Insect |  |  |
| gALL_v3_rep_c105439 | p450 | 1 | 1 | Insect |  |  |
| gALL_v3_c32279 | p450 | 0 | 8 | Insect |  |  |
| gALL_v3_c91064 | p450 | 1 | 1 | Insect |  |  |
| gALL_v3_c56233 | p450 | 2 | 0 | Insect |  |  |
| gALL_v3_rep_c96792 | p450 | 20 | 17 | Insect |  |  |
| gALL_v3_rep_c105179 | p450 | 0 | 4 | Insect | ✔ |  |
| gALL_v3_rep_c106490 | p450 | 3 | 1 | Insect |  |  |
| gALL_v3_c41111 | p450 | 1 | 2 | Protist |  |  |
| gALL_v3_c52654 | p450 | 3 | 1 | Insect |  | ✔ |
| gALL_v3_rep_c97239 | p450 | 0 | 68 | Insect |  |  |
| gALL_v3_c10599 | p450 | 3 | 6 | Insect |  |  |
| gALL_v3_rep_c97596 | p450 | 4 | 10 | Insect |  |  |
| gALL_v3_rep_c98719 | p450 | 3 | 5 | Insect |  |  |
| gALL_v3_c15706 | p450 | 5 | 4 | Insect |  |  |
| gALL_v3_c15 | p450 | 8 | 11 | Insect | ✔ |  |
| gALL_v3_rep_c104965 | p450 | 11 | 4 | Insect |  |  |
| gALL_v3_c10461 | p450 | 7 | 3 | Insect |  |  |
| gALL_v3_rep_c104498 | p450 | 2 | 11 | Protist |  |  |
| gALL_v3_c82008 | p450 | 12 | 1 | Insect | ✔ |  |
| gALL_v3_c76082 | p450 | 2 | 0 | Insect |  |  |
| gALL_v3_rep_c108293 | p450 | 3 | 1 | Insect | ✔ |  |
| gALL_v3_c21565 | p450 | 4 | 0 | Insect |  |  |
| gALL_v3_rep_c97492 | p450 | 1 | 13 | Insect |  |  |
| gALL_v3_c37249 | p450 | 2 | 2 | Insect |  |  |
| gALL_v3_rep_c106603 | p450 | 1 | 1 | Insect |  |  |
| gALL_v3_rep_c105542 | p450 | 1 | 3 | Insect | ✔ |  |
| gALL_v3_c1831 | p450 | 7 | 18 | Insect | ✔ |  |
| gALL_v3_rep_c99964 | p450 | 1 | 2 | Insect |  | ✔ |
| gALL_v3_c5802 | p450 | 9 | 7 | Insect |  | ✔ |
| gALL_v3_rep_c97214 | p450 | 15 | 15 | Insect |  |  |
| gALL_v3_c81323 | p450 | 2 | 0 | Insect |  |  |
| gALL_v3_c5846 | p450 | 19 | 18 | Insect |  |  |
| gALL_v3_rep_c104615 | p450 | 9 | 9 | Insect | ✔ |  |
| gALL_v3_c14555 | p450 | 4 | 1 | Insect |  |  |
| gALL_v3_rep_c108922 | p450 | 2 | 0 | Insect |  |  |
| gALL_v3_rep_c106186 | p450 | 0 | 4 | Insect |  |  |
| gALL_v3_rep_c96914 | p450 | 15 | 5 | Insect |  |  |
| gALL_v3_rep_c106600 | p450 | 2 | 2 | Insect |  |  |
| gALL_v3_rep_c98076 | p450 | 16 | 8 | Insect |  |  |
| gALL_v3_c57043 | p450 | 1 | 1 | Insect |  |  |
| gALL_v3_c6913 | p450 | 15 | 11 | Insect |  |  |
| gALL_v3_rep_c106883 | p450 | 1 | 1 | Protist |  |  |
| gALL_v3_rep_c101394 | p450 | 0 | 2 | Insect |  |  |
| gALL_v3_c8425 | p450 | 5 | 5 | Insect |  |  |
| gALL_v3_rep_c97623 | p450 | 8 | 7 | Insect | ✔ |  |
| gALL_v3_rep_c109606 | p450 | 3 | 0 | Insect |  |  |
| gALL_v3_rep_c98365 | p450 | 23 | 11 | Insect |  |  |
| gALL_v3_rep_c106319 | p450 | 3 | 4 | Protist |  |  |
| gALL_v3_c70328 | p450 | 2 | 0 | Insect | ✔ |  |
| gALL_v3_c5570 | p450 | 0 | 14 | Insect |  |  |
| gALL_v3_c27091 | p450 | 3 | 3 | Insect |  |  |
| gALL_v3_rep_c97322 | p450 | 11 | 7 | Insect |  |  |
| gALL_v3_c65457 | p450 | 1 | 1 | Insect |  | ✔ |
| gALL_v3_c69303 | p450 | 1 | 1 | Insect |  |  |
| gALL_v3_c30245 | p450 | 3 | 3 | Insect |  |  |
| gALL_v3_c19568 | p450 | 7 | 1 | Insect |  |  |
| gALL_v3_rep_c105039 | p450 | 4 | 4 | Insect | ✔ |  |
| gALL_v3_rep_c97366 | p450 | 14 | 6 | Insect |  |  |
| gALL_v3_rep_c97216 | p450 | 93 | 17 | Insect |  |  |
| gALL_v3_rep_c104668 | p450 | 4 | 3 | Insect |  |  |
| gALL_v3_c70602 | p450 | 8 | 2 | Insect | ✔ |  |
| gALL_v3_c58496 | p450 | 12 | 16 | Insect |  |  |
| gALL_v3_c3717 | p450 | 17 | 10 | Insect |  |  |
| gALL_v3_c5675 | p450 | 4 | 37 | Insect |  |  |
| gALL_v3_rep_c96492 | p450 | 33 | 52 | Insect |  |  |
| gALL_v3_c1485 | p450 | 24 | 10 | Insect |  |  |
| gALL_v3_rep_c99323 | p450 | 20 | 10 | Insect | ✔ |  |
| gALL_v3_c56166 | p450 | 8 | 4 | Insect | ✔ |  |
| gALL_v3_c6541 | p450 | 6 | 7 | Insect |  |  |
| gALL_v3_c17232 | p450 | 7 | 4 | Insect |  |  |
| gALL_v3_rep_c96922 | p450 | 8 | 22 | Insect | ✔ |  |
| gALL_v3_rep_c101256 | p450 | 17 | 7 | Insect |  |  |
| gALL_v3_rep_c106099 | p450 | 3 | 2 | Insect |  |  |
| gALL_v3_c6341 | p450 | 5 | 8 | Insect |  |  |
| gALL_v3_rep_c97117 | p450 | 21 | 11 | Insect |  | ✔ |
| gALL_v3_c7900 | p450 | 12 | 10 | Insect |  |  |
| gALL_v3_rep_c100044 | p450 | 14 | 4 | Insect |  |  |
| gALL_v3_c9841 | p450 | 12 | 20 | Insect | ✔ |  |
| gALL_v3_c7756 | p450 | 5 | 6 | Insect |  |  |
| gALL_v3_c40107 | p450 | 1 | 2 | Insect |  | ✔ |
| gALL_v3_rep_c105009 | p450 | 0 | 4 | Insect | ✔ |  |
| gALL_v3_c86468 | p450 | 2 | 0 | Insect | ✔ |  |
| gALL_v3_c2765 | p450 | 5 | 6 | Insect | ✔ |  |
| gALL_v3_rep_c97451 | p450 | 15 | 3 | Insect |  |  |
| gALL_v3_c58341 | p450 | 3 | 5 | Insect |  |  |
| gALL_v3_c9353 | PL-1 | 6 | 2 | Bacteria |  |  |
| gALL_v3_rep_c96365 | PL-11 | 209 | 594 | Insect | ✔ |  |
| gALL_v3_rep_c96383 | PL-11 | 266 | 1014 | Insect | ✔ |  |
| gALL_v3_c25166_5 | SOD | 3 | 4 | Insect |  | ✔ |
| gALL_v3_c18281 | SOD | 8 | 6 | Insect |  |  |
| gALL_v3_c95234 | SOD | 1 | 1 | Insect |  |  |
| gALL_v3_rep_c96668 | SOD | 21 | 16 | Insect |  |  |
| gALL_v3_c7756 | SOD | 5 | 6 | Insect |  |  |
| gALL_v3_c6342 | SOD | 5 | 6 | Protist | ✔ |  |
| gALL_v3_c18892 | SOD | 4 | 1 | Insect | ✔ |  |
| gALL_v3_c59228 | SOD | 0 | 2 | Protist |  |  |
| gALL_v3_c12721 | SOD | 1 | 12 | Insect |  |  |
| gALL_v3_rep_c97252 | SOD | 8 | 12 | Insect |  |  |
| gALL_v3_c39361 | SOD | 0 | 7 | Protist |  |  |
| gALL_v3_c2878 | SOD | 8 | 7 | Protist |  |  |
| gALL_v3_c9841 | SOD | 12 | 20 | Insect | ✔ |  |
| gALL_v3_c68194 | SOD | 2 | 4 | Insect |  |  |
| gALL_v3_c18303 | SOD | 3 | 1 | Protist |  |  |
| gALL_v3_c12515 | SOD | 14 | 8 | Insect |  |  |
| gALL_v3_c10091 | SOD | 31 | 12 | Insect |  |  |
| gALL_v3_rep_c98051 | SOD | 4 | 5 | Protist | ✔ | ✔ |
| gALL_v3_c12037 | SOD | 12 | 7 | Insect |  |  |
| gALL_v3_c64579 | SOD | 12 | 1 | Insect |  |  |
| gALL_v3_rep_c98154 | SOD | 6 | 3 | Insect |  |  |
| gALL_v3_c13210 | SOD | 4 | 2 | Insect |  |  |
| gALL_v3_c58346 | SOD | 2 | 0 | Protist |  |  |
| gALL_v3_c11806 | SOD | 4 | 5 | Insect |  |  |
| gALL_v3_c39260 | SOD | 6 | 9 | Insect |  |  |
|  |  |  |  |  |  |  |
| Total Unigenes |  | 778 | Total Secreted Proteins | | 163 | 111 |
|  |  |  |  |  |  |  |
|  | | | Total Secreted Proteins => 245 | | | |

**References**

HORTON, P., PARK, K. J., OBAYASHI, T., FUJITA, N., HARADA, H., ADAMS-COLLIER, C. J. & NAKAI, K. 2007. WoLF PSORT: protein localization predictor. *Nucleic Acids Res,* 35**,** W585-7.

LUO, R., LIU, B., XIE, Y., LI, Z., HUANG, W., YUAN, J., HE, G., CHEN, Y., PAN, Q., LIU, Y., TANG, J., WU, G., ZHANG, H., SHI, Y., YU, C., WANG, B., LU, Y., HAN, C., CHEUNG, D. W., YIU, S. M., PENG, S., XIAOQIAN, Z., LIU, G., LIAO, X., LI, Y., YANG, H., WANG, J. & LAM, T. W. 2012. SOAPdenovo2: an empirically improved memory-efficient short-read de novo assembler. *Gigascience,* 1**,** 18.

PETERSEN, T. N., BRUNAK, S., VON HEIJNE, G. & NIELSEN, H. 2011. SignalP 4.0: discriminating signal peptides from transmembrane regions. *Nat Methods,* 8**,** 785-6.

SLATER, G. S. & BIRNEY, E. 2005. Automated generation of heuristics for biological sequence comparison. *BMC Bioinformatics,* 6**,** 31.

TERRAPON, N., LI, C., ROBERTSON, H. M., JI, L., MENG, X., BOOTH, W., CHEN, Z., CHILDERS, C. P., GLASTAD, K. M., GOKHALE, K., GOWIN, J., GRONENBERG, W., HERMANSEN, R. A., HU, H., HUNT, B. G., HUYLMANS, A. K., KHALIL, S. M., MITCHELL, R. D., MUNOZ-TORRES, M. C., MUSTARD, J. A., PAN, H., REESE, J. T., SCHARF, M. E., SUN, F., VOGEL, H., XIAO, J., YANG, W., YANG, Z., ZHOU, J., ZHU, J., BRENT, C. S., ELSIK, C. G., GOODISMAN, M. A., LIBERLES, D. A., ROE, R. M., VARGO, E. L., VILCINSKAS, A., WANG, J., BORNBERG-BAUER, E., KORB, J., ZHANG, G. & LIEBIG, J. 2014. Molecular traces of alternative social organization in a termite genome. *Nat Commun,* 5**,** 3636.
