# Supplementary material for: Expanding the Knowledge on Lignocellulolytic and Redox Enzymes of Worker and Soldier Castes from the Lower Termite Coptotermes gestroi
Source: Front Microbiol. 2016 Oct 13;7:1518. doi: 10.3389/fmicb.2016.01518 (PMC5061848; doi:10.3389/fmicb.2016.01518)
Supplement: Table S4 — Total proteins identified in worker and soldier castes of Coptotermes gestroi. [file Table4.PDF]

Cupim Orbitrap 454, Samples report created on 07/05/2012
Experiment: Cupim Orbitrap 454
Peak List Generator: unknown
Version: unknown
Charge States Calculated: unknown
Deisotoped: unknown
Textual Annotation: unknown
Database Set: 1 Database
Database Name: the cupim454 database
Version: unknown
Taxonomy: All Entries
Number of Proteins: 181554
Does database contain common contaminants?: unknown
Search Engine Set: 1 Search Engine
Search Engine: Mascot
Version: 2.3.02
Samples: All Samples
Fragment Tolerance: 1.00 Da (Monoisotopic)
Parent Tolerance: 10,0 PPM (Monoisotopic)
Fixed Modifications: +57 on C (Carbamidomethyl)
Variable Modifications: +16 on M (Oxidation)
Database: the cupim454 database (unknown version, 181554 entries)
Digestion Enzyme: Trypsin
Max Missed Cleavages: 1
Scaffold Version: Scaffold 3.5.2
Peptide Thresholds: 60,0% minimum and Mascot: ion scores must be greater than both the associated identity scores and 31, Mascot: ion scores must be greater than both the associated identity scores and 31
Protein Thresholds: 80,0% minimum and 1 peptides minimum

Cupim Orbitra	Displaying:Quantitative Value,Protein Identification Probability								Soldier	Worker	Soldier	Worker
#	Visible?	Starred?	Identified Proteins (1420)	Accession Number	Molecular Weight	Protein Grouping Amb	Quantitative Variance	Taxonomy	Cupim Soldado	Cupim Operario	Cupim Soldad	Cupim Operario
1	TRUE	FALSE	RecName: Full=Apolipophorins; Contains: RecName: Full=Apolipoi	gALL_v3_rep_c96290_5	273 kDa	FALSE		unknown	89.587	176.42	100%	100%
2	TRUE	FALSE	arginine kinase [Periplaneta americana]	gALL_v3_rep_c96129_1	89 kDa			unknown	66,443	39,789	100%	100%
3	TRUE	FALSE	actin 5C, isoform B [Drosophila melanogaster]	gALL_v3_rep_c96107_4	66 kDa	TRUE		unknown	100.15	39.071	100%	100%
4	TRUE	FALSE	putative glyceraldehyde-3-phosphate dehydrogenase [Oncometopi	gALL_v3_rep_c96157_1	47 kDa			unknown	48,272	54,605	100%	100%
5	TRUE	FALSE	hexamerin II [Reticulitermes flavipes]	gALL_v3_rep_c96113_4	101 kDa	FALSE		unknown	3.3656	47.972	100%	100%
6	TRUE	FALSE	RecName: Full=Apolipophorins; Contains: RecName: Full=Apolipoi	gALL_v3_rep_c96169_4	130 kDa	FALSE		unknown	30,773	53,27	100%	100%
7	TRUE	FALSE	hexamerin I [Reticulitermes flavipes]	gALL_v3_rep_c96143_6	48 kDa	TRUE		unknown	1.371	39.72	93%	100%
8	TRUE	FALSE	putative fructose 1,6-bisphosphate aldolase [Oncometopia nigricar	gALL_v3_rep_c96348_6	67 kDa			unknown	26,715	27,185	100%	100%
9	TRUE	FALSE	calponin [Triatoma infestans]	gALL_v3_rep_c96115_4	53 kDa			unknown	56.615	26.537	100%	100%
10	TRUE	FALSE	transferrin [Mastotermes darwiniensis]	gALL_v3_rep_c96108_1	144 kDa			unknown	10,721	25,451	100%	100%
11	TRUE	FALSE	PREDICTED: similar to CG3937-PD [Nasonia vitripennis]	gALL_v3_rep_c96471_4	146 kDa			unknown	37.277	7.1604	100%	100%
12	TRUE	FALSE	PREDICTED: similar to aconitase, mitochondrial [Nasonia vitripenn	gALL_v3_rep_c96318_5	112 kDa			unknown	19,073	17,484	100%	100%
13	TRUE	FALSE	enolase [Blattella germanica]	gALL_v3_rep_c96293_3	76 kDa			unknown	21.119	12.322	100%	100%
14	TRUE	FALSE	hexamerin I [Reticulitermes flavipes]	gALL_v3_rep_c99316_1	18 kDa	TRUE		unknown	2,742	26,203	100%	100%
15	TRUE	FALSE	PREDICTED: similar to mitochondrial malate dehydrogenase [Naso	gALL_v3_rep_c96214_1	43 kDa			unknown	12.321	22.752	100%	100%
16	TRUE	FALSE	hypothetical protein BRAFLDRAFT_290739 [Branchiostoma floridae	gALL_v3_rep_c96372_1	58 kDa	TRUE		unknown	9,4349	20,983	100%	100%
17	TRUE	FALSE	soldier-caste specific protein 1 [Hodotermopsis japonica]	gALL_v3_rep_c96965_3	32 kDa			unknown	45,248	0,68834	100%	90%
18	TRUE	FALSE	hexamerin I [Reticulitermes flavipes]	gALL_v3_rep_c96145_1 (+1)	28 kDa	TRUE		unknown	0	28,428	0	100%
19	TRUE	FALSE	endo-beta-1,4-glucanase [Coptotermes formosanus]	gALL_v3_rep_c96106_2	76 kDa	TRUE		unknown	11,788	14,747	100%	100%
20	TRUE	FALSE	heat shock protein 20.6 [Locusta migratoria]	gALL_v3_rep_c96384_4	139 kDa			unknown	21,753	16,819	100%	100%
21	TRUE	FALSE	PREDICTED: similar to 3-oxoacyl-[acyl-carrier-protein] reductase [	gALL_v3_rep_c98208_2	26 kDa	TRUE		unknown	6,55	17,907	100%	100%
22	TRUE	FALSE	hypothetical protein TcasGA2_TC012671 [Tribolium castaneum]	gALL_v3_rep_c96168_2	214 kDa			unknown	25,211	4,8183	100%	100%
23	TRUE	FALSE	Catalase, putative [Pediculus humanus corporis]	gALL_v3_rep_c96312_6	78 kDa			unknown	13,125	7,9153	100%	100%
24	TRUE	FALSE	heat shock cognate protein 70 [Nilaparvata lugens]	gALL_v3_rep_c96230_6	83 kDa	TRUE		unknown	11,968	7,5309	100%	100%
25	TRUE	FALSE	glutathione S-transferase [Blattella germanica]	gALL_v3_rep_c96283_4	38 kDa	TRUE		unknown	6,6272	17,889	100%	100%
26	TRUE	FALSE	PREDICTED: similar to alcohol dehydrogenase [Nasonia vitripennis	gALL_v3_rep_c96403_3	51 kDa	TRUE		unknown	7.7264	14.418	100%	100%
27	TRUE	FALSE	PREDICTED: similar to 14-3-3-like protein (Leonardo protein) (14-	gALL_v3_rep_c96226_6	112 kDa	TRUE		unknown	18,546	14,239	100%	100%
28	TRUE	FALSE	PREDICTED: similar to ATP synthase- CG11154-PA, isoform A [Api	gALL_v3_rep_c96234_6	65 kDa			unknown	17.31	8.9862	100%	100%
29	TRUE	FALSE	malate dehydrogenase, putative [Pediculus humanus corporis]	gALL_v3_rep_c96861_1	38 kDa			unknown	8,2611	11,451	100%	100%
30	TRUE	FALSE	Tropomyosin 2 [Lethocerus indicus]	gALL_v3_rep_c96521_4	65 kDa	TRUE		unknown	11.822	11.223	100%	100%
31	TRUE	FALSE	PREDICTED: similar to ENSANGP00000012072 [Nasonia vitripennis	gALL_v3_rep_c96386_2	40 kDa	TRUE		unknown	7,9718	7,9274	100%	100%
32	TRUE	FALSE	tubulin alpha-1 chain [Pediculus humanus corporis]	gALL_v3_rep_c96125_4	62 kDa	TRUE		unknown	14.72	8.9603	100%	100%
33	TRUE	FALSE	PREDICTED: similar to cyclohex-1-ene-1-carboxyl-CoA hydratase,	gALL_v3_rep_c96943_1	35 kDa			unknown	5,6941	12,267	100%	100%
34	TRUE	FALSE	tropomyosin [Periplaneta americana]	gALL_v3_rep_c96164_5	50 kDa	TRUE		unknown	10.932	3.6533	100%	100%
35	TRUE	FALSE	PREDICTED: similar to CG9517-PB, isoform B [Apis mellifera]	gALL_v3_c2122_6	58 kDa	TRUE		unknown	12,825	5,0962	100%	100%
36	TRUE	FALSE	AGAP010640-PA [Anopheles gambiae str. PEST]	gALL_v3_rep_c96402_6 (+1)	44 kDa			unknown	19.369	10.641	100%	100%
37	TRUE	FALSE	endo-b-1,4-glucanase [Coptotermes formosanus]	gALL_v3_rep_c98165_4	18 kDa			unknown	0	15,819	0	100%
38	TRUE	FALSE	endo-b-1,4-glucanase [Coptotermes formosanus]	gALL_v3_rep_c96171_2	27 kDa	TRUE		unknown	0	15.867	0	100%
39	TRUE	FALSE	EIP28, putative [Pediculus humanus corporis]	gALL_v3_c9190_5	33 kDa			unknown	2,2777	12,332	100%	100%
40	TRUE	FALSE	heat shock protein 90 [Locusta migratoria]	gALL_v3_rep_c96392_6	58 kDa	TRUE		unknown	11.218	6.1391	100%	100%
41	TRUE	FALSE	PREDICTED: similar to pyruvate kinase isoform 1 [Tribolium castar	gALL_v3_c19000_3	28 kDa			unknown	12,235	3,7654	100%	100%
42	TRUE	FALSE	PREDICTED: similar to putative elongation factor 1-alpha isoform 1	gALL_v3_rep_c96116_6	76 kDa			unknown	3.5611	8.5033	100%	100%
43	TRUE	FALSE	PREDICTED: similar to translation elongation factor 1-gamma isofo	gALL_v3_rep_c96203_6	63 kDa			unknown	5,3417	7,7064	100%	100%
44	TRUE	FALSE	PREDICTED: similar to AGAP012407-PA [Tribolium castaneum]	gALL_v3_rep_c96565_6	78 kDa			unknown	2,992	9,5713	100%	100%
45	TRUE	FALSE	conserved hypothetical protein [Pediculus humanus corporis]	gALL_v3_rep_c96282_5	196 kDa			unknown	18,99	10,953	100%	100%
46	TRUE	FALSE	PREDICTED: similar to estradiol 17 beta-dehydrogenase [Acyrthos	gALL_v3_rep_c96670_6	102 kDa			unknown	4,9866	13,695	100%	100%
47	TRUE	FALSE	beta tubulin [Pseudotrichonympha grassii]	gALL_v3_rep_c96476_1 (+1)	49 kDa	TRUE		unknown	2,7779	10,685	100%	100%
48	TRUE	FALSE	hypothetical protein CTRG_04948 [Candida tropicalis MYA-3404]	gALL_v3_c1406_5	73 kDa			unknown	16,205	6,5248	100%	100%
49	TRUE	FALSE	superoxide dismutase [Triatoma infestans]	gALL_v3_rep_c96668_1 (+1)	25 kDa			unknown	14,875	9,9487	100%	100%
50	TRUE	FALSE	PREDICTED: similar to AGAP008909-PA [Tribolium castaneum]	gALL_v3_rep_c96529_4	34 kDa			unknown	17,193	7,0157	100%	100%
51	TRUE	FALSE	PREDICTED: similar to CG6084 CG6084-PA [Tribolium castaneum]	gALL_v3_rep_c96122_4	74 kDa			unknown	4,9485	5,307	100%	100%
52	TRUE	FALSE	PREDICTED: similar to Synaptic vesicle membrane protein VAT-1 h	gALL_v3_rep_c96763_6	61 kDa			unknown	5,8769	6,7257	100%	100%
53	TRUE	FALSE	Selenium-binding protein, putative [Pediculus humanus corporis]	gALL_v3_rep_c96344_4	62 kDa			unknown	7.1228	5.9014	100%	100%
54	TRUE	FALSE	PREDICTED: similar to beta-tubulin [Acyrthosiphon pisum]	gALL_v3_rep_c96141_1	94 kDa	TRUE		unknown	8,6775	10,851	100%	100%
55	TRUE	FALSE	ATP synthase [Bombyx mori]	gALL_v3_rep_c96229_2	67 kDa			unknown	2.9413	8.9243	100%	100%
56	TRUE	FALSE	PREDICTED: similar to aldo-keto reductase isoform 1 [Tribolium ca	gALL_v3_rep_c96328_4	65 kDa	TRUE		unknown	5,5902	8,595	100%	100%
57	TRUE	FALSE	triosephosphate isomerase [Blattella germanica]	gALL_v3_rep_c96937_6	25 kDa	TRUE		unknown	11.183	6.6017	100%	100%
58	TRUE	FALSE	glutathione S-transferase [Blattella germanica]	gALL_v3_rep_c96354_6	33 kDa	TRUE		unknown	10,771	10,116	100%	100%
59	TRUE	FALSE	endo-b-1,4-glucanase [Coptotermes formosanus]	gALL_v3_rep_c96486_1 (+4)	32 kDa			unknown	0	11.512	0	91%
60	TRUE	FALSE	beta-glucosidase [Coptotermes formosanus]	gALL_v3_rep_c96194_2	60 kDa			unknown	4,9866	7,2071	100%	100%
61	TRUE	FALSE	glutathione S-transferase O1 [Nasonia vitripennis]	gALL_v3_rep_c96308_6	60 kDa	TRUE		unknown	3.4165	4.5494	100%	100%
62	TRUE	FALSE	PREDICTED: similar to protease m1 zinc metalloprotease [Nasonia	gALL_v3_rep_c96341_2	51 kDa	TRUE		unknown	16,452	2,7533	100%	100%
63	TRUE	FALSE	muscle lim protein, putative [Pediculus humanus corporis]	gALL_v3_rep_c96188_5	178 kDa			unknown	3.9387	12.356	100%	100%
64	TRUE	FALSE	ATPase [Homo sapiens]	gALL_v3_rep_c96657_5	66 kDa			unknown	10,24	2,9088	100%	100%
65	TRUE	FALSE	PREDICTED: similar to Calreticulin CG9429-PA isoform 1 [Apis mel	gALL_v3_rep_c96304_6	87 kDa			unknown	5.8769	6.2202	100%	100%
66	TRUE	FALSE	allergen Bla g 8 [Blattella germanica]	gALL_v3_rep_c96142_6 (+1)	28 kDa	TRUE		unknown	11,915	10,953	100%	100%
67	TRUE	FALSE	heat shock protein 20.5 [Locusta migratoria]	gALL_v3_c7207_5	27 kDa	TRUE		unknown	3.2108	11.834	100%	100%
68	TRUE	FALSE	Ves G 5 allergen precursor, putative [Pediculus humanus corporis]	gALL_v3_rep_c96110_2	43 kDa			unknown	10,267	10,168	100%	100%
69	TRUE	FALSE	Transposable element Tc3 transposase [Lepeophtheirus salmonis]	gALL_v3_c47310_6	14 kDa			unknown	1.371	0	93%	55%
70	TRUE	FALSE	actin [Trichomonas vaginalis G3]	gALL_v3_rep_c96614_6	61 kDa			unknown	0,89028	12,86	91%	100%
71	TRUE	FALSE	transketolase [Aedes aegypti]	gALL_v3_rep_c96522_3	166 kDa	TRUE		unknown	1,9947	5,4191	100%	100%
72	TRUE	FALSE	glutathione S-transferase [Blattella germanica]	gALL_v3_c6961_2	32 kDa	TRUE		unknown	8,6992	3,0784	100%	100%
73	TRUE	FALSE	AGAP011516-PA [Anopheles gambiae str. PEST]	gALL_v3_rep_c98174_5	18 kDa	TRUE		unknown	16,129	12,618	92%	94%
74	TRUE	FALSE	PREDICTED: similar to glucose dehydrogenase [Acyrthosiphon pisu	gALL_v3_rep_c97604_6	25 kDa	TRUE		unknown	18,726	2,6798	100%	100%
75	TRUE	FALSE	predicted protein [Pediculus humanus corporis]	gALL_v3_rep_c96210_5	34 kDa			unknown	3,4165	11,587	100%	100%
76	TRUE	FALSE	putative mitochondrial ATP synthase gamma-subunit [Graphoceph	gALL_v3_rep_c96346_1	46 kDa			unknown	4,5553	8,3633	100%	100%
77	TRUE	FALSE	phosphoglyceromutase [Bombyx mori]	gALL_v3_rep_c96809_5	24 kDa	TRUE		unknown	5,6941	5,285	100%	100%
78	TRUE	FALSE	PREDICTED: similar to Puromycin sensitive aminopeptidase CG100	gALL_v3_c2185_2	77 kDa			unknown	5,4839	5,5067	100%	100%
79	TRUE	FALSE	chymotrypsin [Teleogryllus emma]	gALL_v3_rep_c96147_5 (+1)	30 kDa			unknown	2,7442	9,4923	99%	100%
80	TRUE	FALSE	acyl-CoA dehydrogenase [Heliothis virescens]	gALL_v3_rep_c96643_5	51 kDa			unknown	3.3859	3.9863	100%	100%
81	TRUE	FALSE	PREDICTED: similar to aldehyde oxidase [Nasonia vitripennis]	gALL_v3_c4092_3	146 kDa	TRUE		unknown	3,7393	5,266	100%	100%
82	TRUE	FALSE	ubiquitin carboxyl-terminal esterase L3 [Scylla paramamosain]	gALL_v3_c1488_2	50 kDa			unknown	7.0938	4.84	100%	100%
83	TRUE	FALSE	carboxylesterase clade E, member 11 [Nasonia vitripennis]	gALL_v3_rep_c97115_5	59 kDa	FALSE		unknown	2,992	5,0206	100%	100%
84	TRUE	FALSE	RecName: Full=Peptidyl-prolyl cis-trans isomerase; Short=PPIase;	gALL_v3_rep_c96154_4	49 kDa			unknown	1.944	11.645	100%	100%
85	TRUE	FALSE	PREDICTED: similar to AGAP001884-PA [Tribolium castaneum]	gALL_v3_c8149_4	56 kDa			unknown	4,4514	3,2034	100%	100%
86	TRUE	FALSE	juvenile hormone esterase [Apis mellifera]	gALL_v3_rep_c96274_1	76 kDa			unknown	3.9893	4.393	100%	100%
87	TRUE	FALSE	heat shock protein 90 [Gryllus firmus]	gALL_v3_rep_c96791_4	24 kDa	TRUE		unknown	7,1049	1,9435	100%	100%
88	TRUE	FALSE	tubulin beta-1 chain [Pediculus humanus corporis]	gALL_v3_c10868_6	39 kDa	TRUE		unknown	8.0131	3.602	100%	100%
89	TRUE	FALSE	Probable adenylate kinase isoenzyme F38B2.4 [Caligus clemensi]	gALL_v3_c9222_5	27 kDa			unknown	11,448	5,8224	100%	100%
90	TRUE	FALSE	1 [Reticulitermes speratus]	gALL_v3_rep_c100471_6hexamer	?	TRUE		unknown	0	10.174	73%	100%
91	TRUE	FALSE	chymotrypsin [Teleogryllus emma]	gALL_v3_rep_c96228_4	37 kDa			unknown	5,955	9,0679	100%	100%
92	TRUE	FALSE	cellulase [Pseudotrichonympha grassii]	gALL_v3_rep_c96913_5	48 kDa			unknown	6.6946	3.7654	100%	100%
93	TRUE	FALSE	glycogen phosphorylase [Aedes aegypti]	gALL_v3_c5751_6	23 kDa	TRUE		unknown	7,4786	4,0693	100%	100%
94	TRUE	FALSE	PREDICTED: similar to Neural conserved at 73EF CG11661-PF, isof	gALL_v3_rep_c96694_5	86 kDa			unknown	6.4813	4.13	100%	100%
95	TRUE	FALSE	heat shock protein 20.7 [Locusta migratoria]	gALL_v3_c4660_2	47 kDa	TRUE		unknown	9,6323	7,1828	100%	100%
96	TRUE	FALSE	PREDICTED: similar to conserved hypothetical protein [Acyrthosipl	gALL_v3_rep_c96220_3	63 kDa			unknown	7.3591	4.2319	100%	99%
97	TRUE	FALSE	vesicle amine transport protein [Bombyx mori]	gALL_v3_c665_5	21 kDa			unknown	1,9947	5,5271	100%	100%
98	TRUE	FALSE	putative alcohol dehydrogenase [Gryllotalpa orientalis]	gALL_v3_c4238_3	37 kDa			unknown	3,275	6,1658	92%	100%
99	TRUE	FALSE	chickadee [Tribolium castaneum]	gALL_v3_c15102_4 (+1)	21 kDa			unknown	13,035	7,5404	100%	100%
100	TRUE	FALSE	PREDICTED: similar to aspartate aminotransferase [Tribolium casta	gALL_v3_rep_c96745_1	45 kDa	TRUE		unknown	4,9485	2,7725	100%	100%
101	TRUE	FALSE	protein lethal, putative [Pediculus humanus corporis]	gALL_v3_rep_c96449_5	31 kDa			unknown	1,6054	7,0157	100%	100%
102	TRUE	FALSE	hypothetical protein TcasGA2_TC014887 [Tribolium castaneum]	gALL_v3_rep_c96355_6	30 kDa	TRUE		unknown	0	8,218	72%	100%
103	TRUE	FALSE	beta actin [Liposcelis bostrychophila]	gALL_v3_rep_c96131_2	22 kDa	TRUE		unknown	35,351	4,1588	100%	97%
104	TRUE	FALSE	PREDICTED: similar to apolipophorin-III isoform 2 [Tribolium casta	gALL_v3_rep_c96133_4	33 kDa			unknown	5,3469	7,7512	100%	100%
105	TRUE	FALSE	beta-actin [Liposcelis entomophila]	gALL_v3_c25134_5	20 kDa	TRUE		unknown	13,487	2,707	100%	99%
106	TRUE	FALSE	putative alcohol dehydrogenase [Gryllotalpa orientalis]	gALL_v3_c4684_5	24 kDa	TRUE		unknown	2,2777	6,4564	100%	100%
107	TRUE	FALSE	L-3-hydroxyacyl-Coenzyme A dehydrogenase [Acyrthosiphon pisur	gALL_v3_c2372_5	63 kDa			unknown	1.1388	5.0973	99%	100%
108	TRUE	FALSE	HsjCib isoform2 [Hodotermopsis sjoestedti]	gALL_v3_rep_c96726_5	48 kDa			unknown	3,2108	2,9112	100%	100%
109	TRUE	FALSE	PREDICTED: similar to ENSANGP00000022132 [Nasonia vitripennis	gALL_v3_rep_c97815_6	22 kDa	TRUE		unknown	6.833	3.6686	100%	100%
110	TRUE	FALSE	PREDICTED: similar to Elongation factor 2 (EF-2) isoform 1 [Apis r	gALL_v3_rep_c96149_5	109 kDa			unknown	4,1129	4,8231	100%	100%
111	TRUE	FALSE	PREDICTED: similar to ENSANGP00000012893 [Nasonia vitripennis	gALL_v3_rep_c97083_5	32 kDa	TRUE		unknown	0.99733	4.393	91%	100%
112	TRUE	FALSE	PREDICTED: similar to Contactin CG1084-PA [Apis mellifera]	gALL_v3_c1594_5	142 kDa			unknown	6,8549	4,13	100%	100%
113	TRUE	FALSE	phosphoserine aminotransferase, putative [Pediculus humanus cor	gALL_v3_c4017_4	51 kDa			unknown	1.7806	5.545	100%	100%
114	TRUE	FALSE	PREDICTED: similar to luciferin-regenerating enzyme [Tribolium ca	gALL_v3_rep_c96612_3	40 kDa	TRUE		unknown	0,89028	6,2382	87%	100%
115	TRUE	FALSE	PREDICTED: similar to CG30084-PC, isoform C [Apis mellifera]	gALL_v3_rep_c96358_4	71 kDa			unknown	7.8267	4.7963	100%	100%
116	TRUE	FALSE	PREDICTED: similar to CAP, adenylate cyclase-associated protein 1	gALL_v3_c16317_6	37 kDa	TRUE		unknown	5,3417	3,6262	100%	100%
117	TRUE	FALSE	hypothetical protein LOC100168726 [Acyrthosiphon pisum]	gALL_v3_rep_c96140_3	59 kDa			unknown	6.2936	4.1045	100%	100%
118	TRUE	FALSE	PREDICTED: similar to CG1674 CG1674-PA [Acyrthosiphon pisum]	gALL_v3_rep_c96307_6	99 kDa			unknown	14,64	1,0261	100%	92%
119	TRUE	FALSE	similar to calponin/transgelin [Nasonia vitripennis]	gALL_v3_rep_c105204_2PREDICT	?			unknown	5.832	7.8747	100%	100%
120	TRUE	FALSE	PREDICTED: similar to cathepsin D isoform 1 [Tribolium castaneun	gALL_v3_rep_c96753_4	59 kDa			unknown	5,8388	4,1588	100%	100%
121	TRUE	FALSE	hypothetical protein TcasGA2_TC002492 [Tribolium castaneum]	gALL_v3_c48792_2 (+1)	14 kDa			unknown	9.7201	6.8486	100%	100%
122	TRUE	FALSE	RecName: Full=Glutathione S-transferase; AltName: Full=GST clas	gALL_v3_c57001_4	22 kDa			unknown	7,0938	0	100%	100%
123	TRUE	FALSE	PREDICTED: similar to phosphoribosylaminoimidazole carboxylase	gALL_v3_c3061_6	71 kDa			unknown	0.89028	5.1849	86%	100%
124	TRUE	FALSE	MPA13 allergen [Periplaneta americana]	gALL_v3_c24587_4	14 kDa	TRUE		unknown	9,7201	5,6553	100%	100%
125	TRUE	FALSE	adenosine deaminase, putative [Pediculus humanus corporis]	gALL_v3_rep_c96838_2	50 kDa			unknown	1,7806	3,4656	100%	100%
126	TRUE	FALSE	PREDICTED: similar to CG6084 CG6084-PA [Tribolium castaneum]	gALL_v3_rep_c96865_2	59 kDa	TRUE		unknown	1,7806	5,545	100%	100%
127	TRUE	FALSE	GH14297 [Drosophila grimshawi]	gALL_v3_c8566_1	27 kDa	TRUE		unknown	7,9718	2,6425	100%	100%
128	TRUE	FALSE	4-aminobutyrate aminotransferase [Aedes aegypti]	gALL_v3_c2605_6	70 kDa			unknown	0,99733	3,7654	99%	100%
129	TRUE	FALSE	Polyadenylation factor subunit, putative [Pediculus humanus corpo	gALL_v3_c2976_6	32 kDa			unknown	5,6941	0,88083	100%	98%
130	TRUE	FALSE	actin [Histomonas meleagridis]	gALL_v3_rep_c104375_4putative	?	TRUE		unknown	5,3299	5,6837	94%	100%
131	TRUE	FALSE	PREDICTED: similar to Chd64 CG14996-PB [Apis mellifera]	gALL_v3_rep_c96480_4	33 kDa	TRUE		unknown	5,4934	7,8747	100%	100%
132	TRUE	FALSE	ETHE1 protein, putative [Pediculus humanus corporis]	gALL_v3_c6499_3	38 kDa			unknown	1,1388	9,9578	92%	100%
133	TRUE	FALSE	PREDICTED: similar to Sarcoplasmic calcium-binding protein 2 CG	gALL_v3_rep_c96649_4	38 kDa			unknown	4,3496	4,1045	99%	100%
134	TRUE	FALSE	NADPH-specific isocitrate dehydrogenase [Bombyx mori]	gALL_v3_c2184_1	61 kDa			unknown	1.7806	5.0396	100%	100%
135	TRUE	FALSE	hypothetical protein TcasGA2_TC001169 [Tribolium castaneum]	gALL_v3_rep_c96755_4	45 kDa			unknown	9,5772	1,0261	100%	92%
136	TRUE	FALSE	hypothetical protein BRAFLDRAFT_79044 [Branchiostoma floridae]	gALL_v3_c4383_5	59 kDa			unknown	6.833	3.5233	100%	100%
137	TRUE	FALSE	hypothetical protein TcasGA2_TC008620 [Tribolium castaneum]	gALL_v3_rep_c96405_3	218 kDa			unknown	8,2259	1,3767	100%	100%
138	TRUE	FALSE	Dihydrolipoyl dehydrogenase, putative [Pediculus humanus corpori	gALL_v3_c1119_5	81 kDa			unknown	1.9947	3.1379	100%	100%
139	TRUE	FALSE	PREDICTED: similar to Spectrin beta chain [Apis mellifera]	gALL_v3_rep_c96855_1	97 kDa			unknown	1,371	3,4417	100%	100%
140	TRUE	FALSE	Spectrin beta chain, putative [Pediculus humanus corporis]	gALL_v3_rep_c96912_6	62 kDa	TRUE		unknown	1.371	4.13	99%	100%
141	TRUE	FALSE	PREDICTED: similar to CG11502-PA [Nasonia vitripennis]	gALL_v3_s104891_5	11 kDa			unknown	2,6027	2,6001	89%	92%
142	TRUE	FALSE	chymotrypsin [Teleogryllus emma]	gALL_v3_rep_c96775_5	43 kDa			unknown	4.3496	3.4809	100%	100%
143	TRUE	FALSE	hypothetical protein TcasGA2_TC012421 [Tribolium castaneum]	gALL_v3_c3257_4	50 kDa			unknown	1,7806	4,5265	100%	100%
144	TRUE	FALSE	cuticular protein [Tribolium castaneum]	gALL_v3_c161_1	49 kDa			unknown	10.049	1.8851	100%	94%
145	TRUE	FALSE	RecName: Full=Troponin T; Short=TnT	gALL_v3_rep_c96222_4	111 kDa			unknown	6,267	0,62757	100%	98%
146	TRUE	FALSE	putative glycosyl hydrolase family10 [uncultured symbiotic protist	gALL_v3_c47557_6	21 kDa			unknown	4.5553	3.6686	100%	100%
147	TRUE	FALSE	venom protein 3 [Microctonus hyperodae]	gALL_v3_rep_c96184_5	33 kDa			unknown	0	8,9445	81%	100%
148	TRUE	FALSE	glycogen/starch/alpha-glucan phosphorylase [Gallionella ferrugine	gALL_v3_c415_3	57 kDa			unknown	4.1129	1.3767	100%	100%
149	TRUE	FALSE	PREDICTED: similar to LRP16 protein isoform 1 [Apis mellifera]	gALL_v3_c1687_4	46 kDa			unknown	2,2777	4,4041	100%	100%
150	TRUE	FALSE	PREDICTED: similar to moesin/ezrin/radixin [Acyrthosiphon pisum	gALL_v3_rep_c96877_1	91 kDa	TRUE		unknown	3.9893	0.62757	100%	99%
151	TRUE	FALSE	[Culex quinquefasciatus]	gALL_v3_rep_c104216_2fixR	?	TRUE		unknown	4,5553	12,332	100%	100%

152	TRUE	FALSE	isocitrate dehydrogenase [Gryllus firmus]	gALL v3 c9969 6	33 kDa	TRUE	unknown	3,5611	2,0794	100%	100%
153	TRUE	FALSE	PREDICTED: similar to Rab-protein 10 CG17060-PA [Apis mellifera	gALL v3 c2020 6	79 kDa	TRUE	unknown	6.4215	3.0784	100%	100%
154	TRUE	FALSE	PREDICTED: similar to pyruvate kinase [Nasonia vitripennis]	gALL v3 rep c97529 4	31 kDa	TRUE	unknown	3,9893	1,8827	100%	100%
155	TRUE	FALSE	aspartate aminotransferase [Culex quinquefasciatus]	gALL v3 c8991 3	25 kDa	TRUE	unknown	3.5611	2.7725	100%	100%
156	TRUE	FALSE	PREDICTED: similar to carnitine o-acetyltransferase [Tribolium cas	gALL v3 c536 1	75 kDa		unknown	2,992	2,5103	100%	100%
157	TRUE	FALSE	PREDICTED: similar to AGAP002355-PA [Tribolium castaneum]	gALL v3 rep c96545 5	65 kDa		unknown	2.6708	2.0794	100%	100%
158	TRUE	FALSE	PREDICTED: similar to citrate synthase [Tribolium castaneum]	gALL v3 rep c97436 3	34 kDa		unknown	3,5611	2,0794	100%	100%
159	TRUE	FALSE	Aminopeptidase N precursor, putative [Pediculus humanus corporis	gALL v3 rep c96368 3	84 kDa	TRUE	unknown	10.968	3.4417	100%	100%
160	TRUE	FALSE	transaldolase [Aedes aegypti]	gALL v3 rep c96830 2	34 kDa	TRUE	unknown	2,6708	2,7725	100%	100%
161	TRUE	FALSE	PREDICTED: similar to putative phosphoglycerate kinase [Tribolium	gALL v3 rep c97887 2	17 kDa	TRUE	unknown	4.4514	2.0794	100%	100%
162	TRUE	FALSE	PREDICTED: similar to inorganic pyrophosphatase [Nasonia vitripe	gALL v3 c9025 5	36 kDa		unknown	1,7806	4,1588	100%	100%
163	TRUE	FALSE	PREDICTED: similar to aminoacylase, putative [Tribolium castaneu	gALL v3 rep c96850 4	66 kDa		unknown	2.6708	4.1588	100%	100%
164	TRUE	FALSE	macrophage migration inhibitory factor-like protein [Maconellicocc	gALL v3 rep c96714 2	25 kDa		unknown	3,888	1,8851	100%	100%
165	TRUE	FALSE	heat shock protein 90 [Gryllus firmus]	gALL v3 c39656 2	34 kDa	TRUE	unknown	11.842	6.0223	100%	100%
166	TRUE	FALSE	[Coptotermes formosanus]	gALL v3 rep c100427 4endo-b-1	?	TRUE	unknown	0,89028	6,7499	91%	100%
167	TRUE	FALSE	[Pyrrhocoris apterus]	gALL v3 rep c100281 5actin	?	TRUE	unknown	30.611	12.476	99%	100%
168	TRUE	FALSE	D-3-phosphoglycerate dehydrogenase, putative [Pediculus human	gALL v3 rep c97219 6	29 kDa		unknown	0,99733	4,3954	91%	100%
169	TRUE	FALSE	hexamerin I [Reticulitermes flavipes]	gALL v3 rep c99934 1	17 kDa	TRUE	unknown	0	2.6798	0	100%
170	TRUE	FALSE	thioredoxin-dependent peroxide reductase [Ixodes scapularis]	gALL v3 c1054 4	32 kDa	TRUE	unknown	4,8161	2,9112	100%	100%
171	TRUE	FALSE	PREDICTED: similar to n-myc downstream regulated [Acyrthosipho	gALL v3 rep c96662 1	65 kDa		unknown	3,8097	2,7725	100%	100%
172	TRUE	FALSE	hypothetical protein TcasGA2 TC011512 [Tribolium castaneum]	gALL v3 c5979 6	36 kDa		unknown	1,371	4,7576	99%	100%
173	TRUE	FALSE	Vinculin, putative [Pediculus humanus corporis]	gALL v3 c9830 4	34 kDa		unknown	1,371	3,3809	99%	100%
174	TRUE	FALSE	iron-responsive element-binding protein 1 [Culex quinquefasciatus	gALL v3 c3602 5	42 kDa		unknown	0	3,4417	95%	100%
175	TRUE	FALSE	PREDICTED: similar to Cytochrome c proximal CG17903-PA isofor	gALL v3 rep c96202 4	24 kDa		unknown	3,888	7,5404	100%	100%
176	TRUE	FALSE	PREDICTED: similar to glutamate carboxypeptidase [Nasonia vitrip	gALL v3 rep c96654 4	50 kDa	TRUE	unknown	1,9947	2,5103	100%	100%
177	TRUE	FALSE	phenoloxidase subunit A3 [Apis mellifera]	gALL v3 c7165 2	25 kDa		unknown	3,9893	1,8827	100%	100%
178	TRUE	FALSE	PREDICTED: similar to voltage-dependent anion-selective channel	gALL v3 rep c96247 2	66 kDa		unknown	3,4165	4,4041	100%	100%
179	TRUE	FALSE	similar to putative ferritin 2 isoform 2 [Acyrthosiphon pisum]	gALL v3 rep c100102 6PREDICT	?		unknown	6,4215	3,0784	100%	100%
180	TRUE	FALSE	PREDICTED: similar to Prolyl endopeptidase [Nasonia vitripennis]	gALL v3 c6935 3	83 kDa		unknown	3.9893	1.2551	100%	100%
181	TRUE	FALSE	PREDICTED: similar to alpha actinin CG4376-PB [Tribolium castane	gALL v3 rep c96251 5	124 kDa		unknown	2,742	3,4417	100%	100%
182	TRUE	FALSE	PREDICTED: similar to CG2852-PA [Nasonia vitripennis]	gALL v3 c3344 5	37 kDa		unknown	6.4215	1.0261	100%	100%
183	TRUE	FALSE	p10 [Periplaneta americana]	gALL v3 rep c96159 5 (+2)	24 kDa		unknown	3,888	11,311	100%	100%
184	TRUE	FALSE	PREDICTED: similar to CG1707-PA [Apis mellifera]	gALL v3 c2719 4	39 kDa		unknown	1.6054	4.1045	98%	100%
185	TRUE	FALSE	aliphatic nitrilase [Culex quinquefasciatus]	gALL v3 rep c97934 2	33 kDa	TRUE	unknown	0,89028	4,8519	91%	100%
186	TRUE	FALSE	PREDICTED: similar to lysosomal alpha-mannosidase (mannosidas	gALL v3 c698 5	69 kDa		unknown	4.3123	5.2532	94%	100%
187	TRUE	FALSE	putative polyubiquitin [Arabidopsis thaliana]	gALL v3 c11464 4 (+3)	43 kDa		unknown	2,3683	7,2245	93%	100%
188	TRUE	FALSE	lipid storage droplet surface-binding protein [Glossina morsitans m	gALL v3 rep c96856 5	34 kDa		unknown	2.0291	3.2932	91%	100%
189	TRUE	FALSE	imaginal disk growth factor [Bombyx mori]	gALL v3 rep c96360 3	81 kDa		unknown	6,9995	0	100%	0
190	TRUE	FALSE	cxpwmw03 [Periplaneta americana]	gALL v3 c10229 4	37 kDa		unknown	1.1388	3.4591	98%	100%
191	TRUE	FALSE	MPA3 allergen [Periplaneta americana]	gALL v3 c63752 1 (+3)	17 kDa		unknown	2,7442	3,6686	93%	100%
192	TRUE	FALSE	PREDICTED: similar to transitional endoplasmic reticulum ATPase T	gALL v3 c4464 4	39 kDa		unknown	2.3683	2.6926	99%	100%
193	TRUE	FALSE	PREDICTED: acyl-Coenzyme A dehydrogenase, C-4 to C-12 straigh	gALL v3 c11380 1 (+2)	24 kDa		unknown	1,944	0,68834	94%	89%
194	TRUE	FALSE	PREDICTED: similar to succinyl-CoA synthetase small subunit, puta	gALL v3 rep c97704 6	19 kDa	TRUE	unknown	2.2777	2.7878	100%	100%
195	TRUE	FALSE	PREDICTED: similar to AGAP007393-PB [Tribolium castaneum]	gALL v3 rep c96647 5	30 kDa		unknown	1,9947	2,9088	91%	100%
196	TRUE	FALSE	hypothetical protein TcasGA2 TC004962 [Tribolium castaneum]	gALL v3 c5293 6	19 kDa		unknown	4.6999	1.3863	100%	100%
197	TRUE	FALSE	AGAP001053-PC [Anopheles gambiae str. PEST]	gALL v3 rep c96305 5	39 kDa	TRUE	unknown	3,4165	3,6686	100%	100%
198	TRUE	FALSE	PREDICTED: similar to AGAP006821-PA [Acyrthosiphon pisum]	gALL v3 rep c96993 6	48 kDa		unknown	0,89028	6,0439	91%	100%
199	TRUE	FALSE	hexokinase [Locusta migratoria]	gALL v3 c5311 1	55 kDa		unknown	2,6708	0,62757	100%	90%
200	TRUE	FALSE	hypothetical protein TcasGA2 TC006935 [Tribolium castaneum]	gALL v3 c59230 2 (+1)	17 kDa		unknown	3,888	1,8851	100%	100%
201	TRUE	FALSE	alcohol dehydrogenase [Aedes aegypti]	gALL v3 rep c97005 5	23 kDa		unknown	3,5611	0,69313	100%	99%
202	TRUE	FALSE	muscle myosin heavy chain [Papilio xuthus]	gALL v3 rep c96114 5	249 kDa		unknown	4,1129	2,065	100%	100%
203	TRUE	FALSE	PREDICTED: similar to phosphoglycerate kinase [Nasonia vitripenn	gALL v3 c1419 5	66 kDa	TRUE	unknown	4,4514	1,3863	100%	100%
204	TRUE	FALSE	glucose-6-phosphate isomerase [Nasonia vitripennis]	gALL v3 c2443 4	33 kDa	TRUE	unknown	1,9947	1,2551	100%	100%
205	TRUE	FALSE	PREDICTED: similar to Hsc70Cb CG6603-PA, isoform A isoform 1 [	gALL v3 c9405 2	33 kDa		unknown	6,8549	0,68834	100%	100%
206	TRUE	FALSE	conserved hypothetical protein [Culex quinquefasciatus]	gALL v3 c183 3	44 kDa		unknown	1,7806	3,4656	100%	100%
207	TRUE	FALSE	PREDICTED: similar to predicted protein [Tribolium castaneum]	gALL v3 c9061 1	21 kDa		unknown	1.6054	1.0261	100%	100%
208	TRUE	FALSE	PREDICTED: similar to utp-glucose-1-phosphate uridylyltransferas	gALL v3 rep c97232 2	45 kDa		unknown	0,99733	3,7654	91%	100%
209	TRUE	FALSE	PREDICTED: similar to wd-repeat protein [Nasonia vitripennis]	gALL v3 c21099 6	22 kDa		unknown	4.9866	0	100%	0
210	TRUE	FALSE	PREDICTED: similar to pyruvate carboxylase [Nasonia vitripennis]	gALL v3 c693 6	61 kDa		unknown	0	4,8183	0	100%
211	TRUE	FALSE	Spectrin alpha chain, putative [Pediculus humanus corporis]	gALL v3 c5764 2	43 kDa	TRUE	unknown	0	2.065	0	100%
212	TRUE	FALSE	alpha tubulin [Spirotrichonympha leidyi]	gALL v3 rep c98259 5 (+1)	47 kDa	TRUE	unknown	0,99733	6,3461	91%	100%
213	TRUE	FALSE	AGAP007939-PA [Anopheles gambiae str. PEST]	gALL v3 rep c97364 4	19 kDa	TRUE	unknown	9.8313	4.0693	100%	90%
214	TRUE	FALSE	PREDICTED: similar to fucosidase, alpha-L- 2, plasma [Apis mellife	gALL v3 c472 4	51 kDa		unknown	4,7725	2,5127	100%	94%
215	TRUE	FALSE	luciferin regenerating enzyme [Lampyris turkestanicus]	gALL v3 c5956 4	20 kDa	TRUE	unknown	0.89028	6.3987	91%	100%
216	TRUE	FALSE	calmodulin [Culex quinquefasciatus]	gALL v3 rep c96252 5	43 kDa	TRUE	unknown	5,832	5,314	100%	94%
217	TRUE	FALSE	PREDICTED: similar to Rab-protein 1 CG3320-PA [Tribolium castar	gALL v3 rep c96799 3	31 kDa	TRUE	unknown	5.1548	4.1045	99%	100%
218	TRUE	FALSE	PREDICTED: similar to Legumain [Monodelphis domestica]	gALL v3 c4244 5	30 kDa		unknown	0,89028	0,69313	98%	86%
219	TRUE	FALSE	ADP/ATP translocase [Nylanderia nr. pubens LZ-2010]	gALL v3 rep c96139 1 (+1)	33 kDa		unknown	5.2518	0.88083	100%	91%
220	TRUE	FALSE	beta-NAC-like protein [Reticulitermes flavipes]	gALL v3 rep c96814 5	30 kDa		unknown	2,2777	1,9069	100%	99%
221	TRUE	FALSE	hypothetical protein TcasGA2 TC004962 [Tribolium castaneum]	gALL v3 rep c97178 3	31 kDa		unknown	1.7806	1.3863	100%	100%
222	TRUE	FALSE	PREDICTED: similar to dihydrolipoamide succinyltransferase compo	gALL v3 c620 4	97 kDa		unknown	4,1308	0	100%	98%
223	TRUE	FALSE	Rsp60 [Periplaneta americana]	gALL v3 rep c96306 5	71 kDa		unknown	1.9947	3.269	100%	100%
224	TRUE	FALSE	ribosomal protein P2 [Manduca sexta]	gALL v3 rep c96669 1	17 kDa		unknown	3,888	7,7075	100%	100%
225	TRUE	FALSE	PREDICTED: similar to AGAP001353-PA [Tribolium castaneum]	gALL v3 rep c96120 1	35 kDa	TRUE	unknown	1,944	2,9112	98%	100%
226	TRUE	FALSE	GK18009 [Drosophila willistoni]	gALL v3 c4251 3	22 kDa		unknown	1,1388	0,88083	98%	99%
227	TRUE	FALSE	PREDICTED: similar to CG12918-PA [Apis mellifera]	gALL v3 c13456 5	37 kDa		unknown	10,31	1,0261	100%	92%
228	TRUE	FALSE	PREDICTED: similar to CG17838-PE, isoform E [Apis mellifera]	gALL v3 c10486 6	80 kDa		unknown	0,99733	2,5759	91%	100%
229	TRUE	FALSE	PREDICTED: similar to Glycoprotein 93 CG5520-PA [Tribolium cast	gALL v3 rep c97453 5	36 kDa		unknown	0	2,0042	100%	100%
230	TRUE	FALSE	hexamerin I [Reticulitermes flavipes]	gALL v3 rep c98725 2 (+2)	16 kDa		unknown	0	2,136	0	99%
231	TRUE	FALSE	PREDICTED: similar to antdh CG1386-PA [Apis mellifera]	gALL v3 c11784 3	17 kDa		unknown	5,6941	0,88083	100%	91%
232	TRUE	FALSE	cytosolic juvenile hormone binding protein 36 kDa subunit [Bomby	gALL v3 c8737 4	40 kDa		unknown	2,2777	3,5233	100%	100%
233	TRUE	FALSE	PREDICTED: similar to malic enzyme [Acyrthosiphon pisum]	gALL v3 c12082 2	39 kDa	TRUE	unknown	0	2,5103	91%	100%
234	TRUE	FALSE	2-cys peroxiredoxin [Bombus ignitus]	gALL v3 c58100 1	21 kDa	TRUE	unknown	8.0269	2.0522	100%	92%
235	TRUE	FALSE	[Periplaneta americana]	gALL v3 rep c104223 4Parcxpwi	?	TRUE	unknown	0	2,6425	0	100%
236	TRUE	FALSE	restnal dehydrogenase, putative [Pediculus humanus corporis]	gALL v3 c4214 1	25 kDa		unknown	2.992	1.8827	100%	100%
237	TRUE	FALSE	PREDICTED: similar to CG3609-PA isoform 2 [Apis mellifera]	gALL v3 c2618 5	84 kDa		unknown	2,6708	2,7725	100%	100%
238	TRUE	FALSE	cuticular protein RR-1 motif 32 [Bombyx mori]	gALL v3 c1704 5	51 kDa		unknown	7.776	5.6553	100%	100%
239	TRUE	FALSE	pro-phenol oxidase subunit 2 [Tribolium castaneum]	gALL v3 rep c96537 5	77 kDa	TRUE	unknown	1,9947	1,8827	100%	100%
240	TRUE	FALSE	hydroxyacylglutathione hydrolase, mitochondrial [Gallus gallus]	gALL v3 c1127 5	27 kDa		unknown	2.2777	3.5233	100%	100%
241	TRUE	FALSE	PREDICTED: similar to Phosphogluconate mutase CG5165-PA [Apis	gALL v3 c1965 2	84 kDa		unknown	3,9893	1,2551	100%	100%
242	TRUE	FALSE	PREDICTED: similar to 60 kDa heat shock protein, mitochondrial p	gALL v3 c8001 6	45 kDa		unknown	1.9947	3.1379	100%	100%
243	TRUE	FALSE	PREDICTED: similar to aldehyde oxidase [Nasonia vitripennis]	gALL v3 c4092 2	144 kDa	TRUE	unknown	2,742	2,7533	100%	100%
244	TRUE	FALSE	PREDICTED: similar to CG9674 CG9674-PA [Acyrthosiphon pisum]	gALL v3 c9288 6	48 kDa		unknown	2.742	1.3767	100%	100%
245	TRUE	FALSE	Spectrin alpha chain, putative [Pediculus humanus corporis]	gALL v3 rep c97135 2	48 kDa	TRUE	unknown	1,371	2,065	99%	100%
246	TRUE	FALSE	aminopeptidase-like protein [Tribolium castaneum]	gALL v3 rep c96737 1	116 kDa		unknown	4.1129	0.68834	100%	99%
247	TRUE	FALSE	Muscle-specific protein, putative [Pediculus humanus corporis]	gALL v3 rep c97632 5	22 kDa		unknown	3,888	1,0261	100%	99%
248	TRUE	FALSE	PREDICTED: similar to serine 3-dehydrogenase [Tribolium castane	gALL v3 rep c96751 2	22 kDa	TRUE	unknown	2.2777	2.6425	100%	98%
249	TRUE	FALSE	PREDICTED: similar to AGAP002401-PA [Tribolium castaneum]	gALL v3 rep c96622 4	35 kDa		unknown	1,1388	3,5233	98%	100%
250	TRUE	FALSE	PREDICTED: similar to Spectrin alpha chain [Apis mellifera]	gALL v3 rep c96539 4	99 kDa		unknown	1.371	3.4417	93%	100%
251	TRUE	FALSE	PREDICTED: similar to glycerol-3-phosphate dehydrogenase [Tribo	gALL v3 c6700 4	28 kDa	TRUE	unknown	0,89028	2,7725	91%	100%
252	TRUE	FALSE	PREDICTED: similar to CG5525-PA isoform 1 [Apis mellifera]	gALL v3 c1790 2	78 kDa		unknown	1,9947	3,1379	91%	100%
253	TRUE	FALSE	PREDICTED: similar to chaperonin [Tribolium castaneum]	gALL v3 c712 4	70 kDa		unknown	2,992	0,62757	100%	89%
254	TRUE	FALSE	methylmalonate-semialdehyde dehydrogenase [Aedes aegypti]	gALL v3 c7599 5	69 kDa		unknown	0	3,7654	0	100%
255	TRUE	FALSE	PREDICTED: similar to ubiquilin-1 isoform 1 [Tribolium castaneum]	gALL v3 c4004 4	38 kDa		unknown	4,6882	2,7659	93%	98%
256	TRUE	FALSE	PREDICTED: similar to Hsc70Cb CG6603-PA, isoform A isoform 1 [	gALL v3 c1098 5	70 kDa		unknown	1,371	0,68834	98%	90%
257	TRUE	FALSE	enolase [Pseudotrichonympha grassii]	gALL v3 c2726 5	37 kDa		unknown	0,89028	3,602	91%	100%
258	TRUE	FALSE	RecName: Full=Putative defense protein; AltName: Full=Basic 19		gALL v3 rep c96513 1 (+1)	25 kDa		unknown	1,6054	0	99%	95%
259	TRUE	FALSE	Thymidine phosphorylase [Salmo salar]	gALL v3 rep c97156 1	24 kDa	TRUE	unknown	0,89028	1,2551	91%	100%
260	TRUE	FALSE	PREDICTED: similar to Type III alcohol dehydrogenase CG3425-PA	gALL v3 c17409 6	62 kDa		unknown	0,89028	1,3863	91%	100%
261	TRUE	FALSE	PREDICTED: similar to 3-oxoacyl-[acyl-carrier-protein] reductase [	gALL v3 c61087 1	14 kDa	TRUE	unknown	2.2777	6.6017	100%	100%
262	TRUE	FALSE	NADH dehydrogenase subunit 5 [Reticulitermes hageni]	gALL v3 c32468 4 (+9)	27 kDa		unknown	1,371	0	91%	0
263	TRUE	FALSE	Advillin, putative [Pediculus humanus corporis]	gALL v3 rep c96468 5	90 kDa		unknown	1.371	2.9458	98%	100%
264	TRUE	FALSE	conserved hypothetical protein [Culex quinquefasciatus]	gALL v3 c2110 5	34 kDa		unknown	4,5553	1,9069	100%	92%
265	TRUE	FALSE	PREDICTED: similar to yellow protein precursor, putative [Acyrthos	gALL v3 rep c96551 5	65 kDa		unknown	2.992	1.2551	100%	100%
266	TRUE	FALSE	adhesin AP65-1 precursor [Trichomonas vaginalis G3]	gALL v3 c2210 6 (+1)	61 kDa		unknown	0,99733	1,8827	91%	100%
267	TRUE	FALSE	PREDICTED: similar to Spectrin beta chain [Apis mellifera]	gALL v3 rep c96627 2	122 kDa	TRUE	unknown	0	3.0911	49%	100%
268	TRUE	FALSE	AGAP001325-PA [Anopheles gambiae str. PEST]	gALL v3 c16031 3	38 kDa	TRUE	unknown	3,888	0	100%	87%
269	TRUE	FALSE	electron-transfer-flavoprotein beta polypeptide [Bombyx mori]	gALL v3 c11993 6	41 kDa		unknown	0	2.6425	0	100%
270	TRUE	FALSE	PREDICTED: similar to F-actin capping protein subunit beta [Apis r	gALL v3 c6458 4	40 kDa		unknown	3,4165	1,7617	100%	100%
271	TRUE	FALSE	PREDICTED: similar to 14.5 kDa translational inhibitor protein, p14	gALL v3 c14869 6	25 kDa		unknown	3.888	3.7702	100%	100%
272	TRUE	FALSE	glutathione S-transferase T3 [Nasonia vitripennis]	gALL v3 c4709 3	56 kDa		unknown	2,2777	3,5233	100%	100%
273	TRUE	FALSE	hypothetical protein TcasGA2 TC015465 [Tribolium castaneum]	gALL v3 c14836 1	30 kDa	TRUE	unknown	1,9947	2.065	100%	100%
274	TRUE	FALSE	Heat shock 70 kDa protein cognate, putative [Pediculus humanus c	gALL v3 c593 3	44 kDa		unknown	3,9893	1,2551	100%	100%
275	TRUE	FALSE	PREDICTED: similar to glycogen debranching enzyme [Nasonia vit	gALL v3 c6469 5	60 kDa		unknown	2.742	1.3767	100%	100%
276	TRUE	FALSE	PREDICTED: es1 protein [Taeniopygia guttata]	gALL v3 c9412 6	35 kDa		unknown	3,2108	3,0784	100%	100%
277	TRUE	FALSE	PREDICTED: similar to 6-phosphogluconate dehydrogenase, decarl	gALL v3 rep c96685 1	30 kDa		unknown	2.6708	1.3863	100%	100%
278	TRUE	FALSE	proteasome subunit beta 1 [Helicoverpa armigera]	gALL v3 c41737 4	27 kDa		unknown	3,2108	2,0522	100%	100%
279	TRUE	FALSE	PREDICTED: similar to proteasome zeta subunit [Tribolium castane	gALL v3 c4271 4	53 kDa		unknown	2,2777	1,7617	100%	100%
280	TRUE	FALSE	PREDICTED: similar to serine 3-dehydrogenase [Tribolium castane	gALL v3 c237 4	41 kDa	TRUE	unknown	2,2777	1,7617	100%	99%
281	TRUE	FALSE	PREDICTED: similar to juvenile hormone esterase [Tribolium casta	gALL v3 c26448 5	30 kDa		unknown	1,9947	1,2551	98%	100%
282	TRUE	FALSE	PREDICTED: similar to Neural conserved at 73EF CG11661-PA, iso	gALL v3 c1613 6	49 kDa		unknown	2,742	2,065	93%	100%
283	TRUE	FALSE	PREDICTED: similar to fibroinase [Nasonia vitripennis]	gALL v3 rep c96740 2	80 kDa		unknown	1,1388	3,5233	92%	100%
284	TRUE	FALSE	hypothetical protein TcasGA2 TC004676 [Tribolium castaneum]	gALL v3 c7788 5	34 kDa		unknown	6,8549	0,68834	100%	90%
285	TRUE	FALSE	Helicase, putative [Pediculus humanus corporis]	gALL v3 c1955 4	96 kDa		unknown	0	9,4255	0	94%
286	TRUE	FALSE	PREDICTED: similar to fatty acid synthase [Tribolium castaneum]	gALL v3 c3809 3	46 kDa		unknown	0	1,3767	52%	100%
287	TRUE	FALSE	bifunctional purine biosynthesis protein [Aedes aegypti]	gALL v3 c14525 4	52 kDa		unknown	0	1,8827	0	100%
288	TRUE	FALSE	receptor for activated protein kinase C-like [Blattella germanica]	gALL v3 rep c97171 5	45 kDa		unknown	2.2777	0	100%	0
289	TRUE	FALSE	PREDICTED: similar to Type III alcohol dehydrogenase CG3425-PA	gALL v3 c17409 4	60 kDa		unknown	0,89028	3,8334	91%	100%
290	TRUE	FALSE	PREDICTED: similar to CG6330-PA, isoform A [Apis mellifera]	gALL v3 rep c97426 2	38 kDa		unknown	0.89028	2.6001	91%	99%
291	TRUE	FALSE	actin [Pyrrhocoris apterus]	gALL v3 rep c96134 4	31 kDa	TRUE	unknown	13,354	17,273	91%	94%
292	TRUE	FALSE	major royal jelly protein 4 precursor, putative [Pediculus humanus	gALL v3 rep c96949 6	41 kDa		unknown	1.8876	1.3207	99%	89%
293	TRUE	FALSE	Thymidine phosphorylase precursor, putative [Pediculus humanus c	gALL v3 c18752 6	15 kDa		unknown	0,89028	1,9483	91%	100%
294	TRUE	FALSE	Endocuticle structural glycoprotein SgAbd-8, putative [Pediculus hu	gALL v3 c3036 2	55 kDa		unknown	3.2108	4.7963	100%	100%
295	TRUE	FALSE	PREDICTED: similar to High mobility group protein DSP1 (Protein c	gALL v3 c26474 1	26 kDa		unknown	2,7442	0	99%	92%
296	TRUE	FALSE	PREDICTED: similar to Glycogenin CG9480-PB, isoform B [Apis me	gALL v3 c16413 3 (+1)	23 kDa		unknown	3.7393	0.68834	100%	90%
297	TRUE	FALSE	beta-hexosaminidase beta chain precursor, putative [Pediculus hur	gALL v3 c495 6	44 kDa		unknown	2,8849	0,62757	100%	89%
298	TRUE	FALSE	PREDICTED: similar to ran CG1404-PA, isoform A [Apis mellifera]	gALL v3 rep c96454 6	29 kDa		unknown	2.7442	0	93%	97%
299	TRUE	FALSE	PREDICTED: similar to transitional endoplasmic reticulum ATPase T	gALL v3 c12160 4	30 kDa	TRUE	unknown	1,371	2,6926	93%	100%
300	TRUE	FALSE	PREDICTED: similar to Protein CREG1 precursor (Cellular represso	gALL v3 rep c97407 4	27 kDa	TRUE	unknown	1.6054	2.0522	93%	100%
301	TRUE	FALSE	PREDICTED: hypothetical protein [Apis mellifera]	gALL v3 c21159 6	23 kDa		unknown	1,944	3,7702	97%	100%
302	TRUE	FALSE	hypothetical protein TcasGA2 TC013859 [Tribolium castaneum]	gALL v3 c7085 2	20 kDa		unknown	0	0.69313	36%	100%
303	TRUE	FALSE	PREDICTED: similar to RhoGDI CG7823-PA isoform 1 [Apis mellife	gALL v3 c29387 2	14 kDa	TRUE	unknown	3,4165	0,88083	100%	91%
304	TRUE	FALSE	similar to 3-oxoacyl-[acyl-carrier-protein] reductase [Tribolium cas	gALL v3 rep c100190 6PREDICT	?	TRUE	unknown	4.5553	13.212	92%	100%
305	TRUE	FALSE	aspartate aminotransferase, cytoplasmic, putative [Pediculus huma	gALL v3 c5888 2	20 kDa	TRUE	unknown	0,89028	2,0794	91%	100%
306	TRUE	FALSE	squid, variant G [Blattella germanica]	gALL v3 rep c96191 5	56 kDa		unknown	0	2,2671	54%	100%
307	TRUE	FALSE	ribosomal protein S8e [Cimex lectularius]	gALL v3 c9652 4	31 kDa		unknown	2,2777	0,88083	100%	98%
308	TRUE	FALSE	PREDICTED: similar to calcium/calmodulin-dependent protein kina	gALL v3 c3406 1	52 kDa		unknown	0	3,9645	50%	100%
309	TRUE	FALSE	aspartate aminotransferase, putative [Pediculus humanus corporis	gALL v3 c62136 1	25 kDa	TRUE	unknown	0,89028	1,3863	91%	100%
310	TRUE	FALSE	GL26144 [Drosophila persimilis]	gALL v3 c20862 5	19 kDa		unknown	0	1,8827	44%	100%
311	TRUE	FALSE	ADP-ribosylation factor 1 [Marsupenaeus japonicus]	gALL v3 rep c96442 4	92 kDa	TRUE	unknown	3,888	3,0784	100%	100%
312	TRUE	FALSE	RecName: Full=V-type proton ATPase subunit B; Short=V-ATPase s	gALL v3 rep c96772 5	65 kDa		unknown	1,9947	1,8827	100%	100%
313	TRUE	FALSE	aldose 1-epimerase, putative [Pediculus humanus corporis]	gALL v3 rep c96996 1	40 kDa		unknown	2,6708	1,3863	100%	100%
314	TRUE	FALSE	putative translationally controlled tumor protein [Graphocephala at	gALL v3 c76762 2 (+1)	20 kDa		unknown	3,2108	2,0522	100%	100%
315	TRUE	FALSE	PREDICTED: similar to 60 kDa heat shock protein, mitochondrial p	gALL v3 c8001 4	45 kDa	TRUE	unknown	1.9947	0.62757	100%	100%
316	TRUE	FALSE	aldo-keto reductase [Aedes aegypti]	gALL v3 rep c96584 4	27 kDa	TRUE	unknown	1,7806	2,0794	100%	100%
317	TRUE	FALSE	PREDICTED: similar to conserved hypothetical protein [Tribolium c	gALL v3 c1597 6	62 kDa		unknown	2.992	0.62757	100%	99%
318	TRUE	FALSE	proteasome subunit alpha type, putative [Pediculus humanus corp	gALL v3 c34298 6	33 kDa		unknown	1,7806	0,88083	100%	99%
319	TRUE	FALSE	pyruvate dehydrogenase [Aedes aegypti]	gALL v3 c6687 4	39 kDa		unknown	1.7806	0.69313	100%	99%
320	TRUE	FALSE	thioredoxin-like protein [Maconellicoccus hirsutus]	gALL v3 rep c96389 2	56 kDa		unknown	1,944	5,6553	99%	100%
321	TRUE	FALSE	PREDICTED: similar to apolipophorin-III isoform 2 [Tribolium casta	gALL v3 rep c96310 2	30 kDa		unknown	4.8161	1.0261	100%	99%
322	TRUE	FALSE	hypothetical protein TcasGA2 TC015096 [Tribolium castaneum]	gALL v3 c16754 4	23 kDa		unknown	4,1129	0	100%	98%
323	TRUE	FALSE	PREDICTED: similar to proteasome subunit beta type 5.8 [Tribolium	gALL v3 rep c98891 4	16 kDa	TRUE	unknown	3.2108	1.0261	100%	98%
324	TRUE	FALSE	Antichymotrypsin-2 [Lepeophtheirus salmonis]	gALL v3 rep c98017 5	21 kDa		unknown	2,3683	0	100%	0
325	TRUE	FALSE	Profilin A [Trichomonas vaginalis G3]	gALL v3 c42274 3	13 kDa		unknown	1.944	1.8851	99%	99%
326	TRUE	FALSE	glutathione S-transferase O1 [Nasonia vitripennis]	gALL v3 c2087 5	34 kDa		unknown	1,1388	0,88083	99%	99%
327	TRUE	FALSE	alpha-2-macroglobulin precursor, putative [Pediculus humanus cor	gALL v3 rep c97571 4	67 kDa		unknown	1.371	0.68834	99%	98%
328	TRUE	FALSE	PREDICTED: similar to pyruvate dehydrogenase [Tribolium castane	gALL v3 c7648 6	33 kDa		unknown	0	2,0794	96%	100%
329	TRUE	FALSE	hypothetical protein TcasGA2 TC014166 [Tribolium castaneum]	gALL v3 rep c97220 1	38 kDa		unknown	0.89028	0	99%	96%
330	TRUE	FALSE	2-oxoglutarate dehydrogenase, putative [Pediculus humanus corpo	gALL v3 c49738 6	19 kDa	TRUE	unknown	2,742	1,3767	93%	100%
331	TRUE	FALSE	putative alcohol dehydrogenase [Gryllotalpa orientalis]	gALL v3 c20187 5	27 kDa	TRUE	unknown	2.2777	4.4041	92%	100%
332	TRUE	FALSE	PREDICTED: similar to luciferin-regenerating enzyme [Tribolium ca	gALL v3 c11532 6	39 kDa	TRUE	unknown	1,1388	2,6425	92%	100%

333	TRUE	FALSE	PREDICTED: similar to proteasome beta-subunit [Nasonia vitripenı	gALL v3 rep c99104 4	22 kDa		unknown	4,5553	0,88083	100%	91%
334	TRUE	FALSE	BTB/POZ domain-containing protein kctd15, putative [Pediculus hu	gALL v3 c139 4	53 kDa		unknown	1.1388	0.88083	100%	91%
335	TRUE	FALSE	PREDICTED: similar to citrate synthase [Tribolium castaneum]	gALL v3 c8150 6	30 kDa		unknown	0,89028	2,0794	91%	100%
336	TRUE	FALSE	crystallin, zeta (quinone reductase) [Xenopus laevis]	gALL v3 c1078 6	61 kDa		unknown	0,89028	1.3863	91%	100%
337	TRUE	FALSE	hypothetical protein TcasGA2 TC009819 [Tribolium castaneum]	gALL v3 c28000 2 (+1)	15 kDa		unknown	0,99733	1,8827	91%	100%
338	TRUE	FALSE	PREDICTED: alcohol dehydrogenase-like, partial [Saccoglossus kov	gALL v3 c15676 5	22 kDa		unknown	1.7806	2.0794	91%	100%
339	TRUE	FALSE	actinin [Trichomonas vaginalis G3]	gALL v3 c1297 5	58 kDa		unknown	0,99733	0,62757	91%	100%
340	TRUE	FALSE	GF11944 [Drosophila ananassae]	gALL v3 c1869 6	63 kDa		unknown	3.9893	0.62757	100%	89%
341	TRUE	FALSE	PREDICTED: similar to N-acetyl neuraminate lyase [Tribolium casta	gALL v3 c16302 2	32 kDa		unknown	0	0,88083	0	99%
342	TRUE	FALSE	PREDICTED: similar to galactokinase 1 [Strongylocentrotus purpur	gALL v3 c9775 2	36 kDa	TRUE	unknown	0.89028	0.69313	99%	89%
343	TRUE	FALSE	bleomycin hydrolase [Culex quinquefasciatus]	gALL v3 c333 1	49 kDa		unknown	2,6708	0,69313	100%	86%
344	TRUE	FALSE	S3e ribosomal protein [Carabus granulatus]	gALL v3 rep c96317 4	38 kDa		unknown	2.2777	0	100%	73%
345	TRUE	FALSE	Chloride intracellular channel exl-1, putative [Pediculus humanus c	gALL v3 rep c97447 5	45 kDa		unknown	2,2777	0	99%	68%
346	TRUE	FALSE	kynurenine/oxoglutarate transaminase 1, putative [Pediculus huma	gALL v3 c731 6	57 kDa		unknown	0	2.0794	0	100%
347	TRUE	FALSE	clathrin heavy chain, putative [Pediculus humanus corporis]	gALL v3 c1737 6	99 kDa		unknown	0	2,065	0	100%
348	TRUE	FALSE	fucU homolog [Caligus rogercresseyi]	gALL v3 c1470 3	19 kDa		unknown	0	5.6553	0	100%
349	TRUE	FALSE	PREDICTED: similar to rCG33798 [Nasonia vitripennis]	gALL v3 c3173 5	101 kDa		unknown	1,371	2,342	93%	90%
350	TRUE	FALSE	Transposable element Tc3 transposase [Lepeophtheirus salmonis]	gALL v3 c102029 3	10 kDa		unknown	2.2613	0.68834	93%	90%
351	TRUE	FALSE	cxpwmw03 [Periplaneta americana]	gALL v3 c10229 6	37 kDa		unknown	1,1388	2,7659	92%	94%
352	TRUE	FALSE	PREDICTED: similar to tripeptidylpeptidase II [Nasonia vitripennis]	gALL v3 c23298 6	23 kDa		unknown	1,371	1,3815	88%	90%
353	TRUE	FALSE	PREDICTED: similar to BMKETTIN [Tribolium castaneum]	gALL v3 rep c96238 5	542 kDa		unknown	0	1,8851	0	94%
354	TRUE	FALSE	hypothetical protein TcasGA2 TC010171 [Tribolium castaneum]	gALL v3 c44055 6	16 kDa		unknown	0,99733	0,68834	91%	90%
355	TRUE	FALSE	PREDICTED: similar to CG33291-PA [Apis mellifera]	gALL v3 c29103 4	21 kDa		unknown	0	0,68834	78%	90%
356	TRUE	FALSE	hypothetical protein TcasGA2 TC001048 [Tribolium castaneum]	gALL v3 rep c99727 6	26 kDa	TRUE	unknown	3,5494	1,8851	94%	98%
357	TRUE	FALSE	bridging integrator [Culex quinquefasciatus]	gALL v3 c6213 5	47 kDa		unknown	1,7806	2,7659	100%	94%
358	TRUE	FALSE	PREDICTED: hypothetical protein [Rattus norvegicus]	gALL v3 rep c96376 1	32 kDa		unknown	5,2399	0	100%	0
359	TRUE	FALSE	Cu/Zn superoxide dismutase [Rachycentron canadum]	gALL v3 c6342 6	23 kDa		unknown	2,7442	0,88083	98%	91%
360	TRUE	FALSE	conserved hypothetical protein [Pediculus humanus corporis]	gALL v3 c11903 1	32 kDa		unknown	3,7415	0	99%	0
361	TRUE	FALSE	PREDICTED: similar to RNA-binding protein, putative [Nasonia vitr	gALL v3 c14781 1	34 kDa		unknown	2.0291	1.7617	92%	100%
362	TRUE	FALSE	unnamed protein product [Blastocystis hominis]	gALL v3 c4113 1	46 kDa		unknown	0	3,6262	0	100%
363	TRUE	FALSE	PREDICTED: similar to GA13362-PA [Acyrthosiphon pisum]	gALL v3 rep c96296 4	70 kDa		unknown	0.99733	2.0042	91%	100%
364	TRUE	FALSE	PREDICTED: similar to microtubule-associated protein RP/EB famil	gALL v3 c6208 4	51 kDa		unknown	3,0828	3,7702	94%	94%
365	TRUE	FALSE	conserved hypothetical protein [Pediculus humanus corporis]	gALL v3 rep c96182 3 (+2)	172 kDa		unknown	2.742	1.3159	100%	90%
366	TRUE	FALSE	protein TcasGA2 TC015700 [Tribolium castaneum]	gALL v3 rep c104195 4hypothet	?	TRUE	unknown	1,8876	0,62757	98%	89%
367	TRUE	FALSE	serpin peptidase inhibitor 3 [Tribolium castaneum]	gALL v3 rep c97853 4	21 kDa	TRUE	unknown	0.89028	0.69313	97%	89%
368	TRUE	FALSE	GM18289 [Drosophila sechellia]	gALL v3 c3761 4	28 kDa		unknown	1,1388	0,88083	92%	99%
369	TRUE	FALSE	PREDICTED: similar to AGAP008909-PA [Tribolium castaneum]	gALL v3 rep c96749 4	28 kDa		unknown	2.7442	0	100%	76%
370	TRUE	FALSE	PREDICTED: similar to GA13327-PA [Nasonia vitripennis]	gALL v3 c4696 5	40 kDa	TRUE	unknown	0	2,009	0	89%
371	TRUE	FALSE	PREDICTED: similar to 26S protease regulatory subunit 6a [Nason	gALL v3 c988 6	53 kDa	TRUE	unknown	0	0.62757	50%	89%
372	TRUE	FALSE	PREDICTED: similar to proteasome beta 3 subunit [Nasonia vitripe	gALL v3 c8633 4	18 kDa		unknown	1,1388	1,7617	92%	100%
373	TRUE	FALSE	Flagellin and related hook-associated proteins [Eubacterium siraeu	gALL v3 c26025 4	15 kDa		unknown	0.89028	0	87%	55%
374	TRUE	FALSE	hypothetical protein TcasGA2 TC001939 [Tribolium castaneum]	gALL v3 c52403 4	15 kDa		unknown	1,1388	0	86%	66%
375	TRUE	FALSE	putative actin [Histomonas meleagridis]	gALL v3 c1937 1	41 kDa	TRUE	unknown	3.5611	6.5427	89%	98%
376	TRUE	FALSE	GK21597 [Drosophila willistoni]	gALL v3 c81055 5	16 kDa		unknown	2,742	0,68834	93%	90%
377	TRUE	FALSE	triosephosphate isomerase [Blattella germanica]	gALL v3 c20492 3	12 kDa	TRUE	unknown	2.2777	2.0522	92%	92%
378	TRUE	FALSE	PREDICTED: similar to CG7899-PB [Nasonia vitripennis]	gALL v3 c4267 5	42 kDa		unknown	0	0,69313	74%	99%
379	TRUE	FALSE	GH22627 [Drosophila grimshawi]	gALL v3 c7521 6	61 kDa		unknown	1,371	0	92%	93%
380	TRUE	FALSE	aconitase, putative [Pediculus humanus corporis]	gALL v3 c2786 2	67 kDa		unknown	0	1,3159	49%	90%
381	TRUE	FALSE	PREDICTED: similar to Ribosomal protein L4 CG5502-PA isoform 1	gALL v3 rep c96391 6	51 kDa		unknown	1,9947	0,62757	100%	89%
382	TRUE	FALSE	PREDICTED: similar to aldo-keto reductase isoform 1 [Tribolium ca	gALL v3 rep c97134 3	73 kDa	TRUE	unknown	0	4,6576	0	98%
383	TRUE	FALSE	catalase [Stylochus sp. KJP-2004]	gALL v3 c48664 4 (+1)	13 kDa		unknown	1,1388	0	87%	37%
384	TRUE	FALSE	MPA3 allergen [Periplaneta americana]	gALL v3 rep c96611 6	30 kDa	TRUE	unknown	2,2777	0	100%	61%
385	TRUE	FALSE	PREDICTED: similar to metalloprotease [Tribolium castaneum]	gALL v3 c2519 5	74 kDa		unknown	2,742	1,3767	100%	100%
386	TRUE	FALSE	alpha-amylase [Blattella germanica]	gALL v3 c56260 1	62 kDa	TRUE	unknown	1,9947	1,2551	100%	100%
387	TRUE	FALSE	fatty acid synthase, putative [Pediculus humanus corporis]	gALL v3 rep c96294 3	81 kDa		unknown	5,4884	0	100%	0
388	TRUE	FALSE	PREDICTED: similar to Proteasome subunit alpha type 3 (Proteaso	gALL v3 c36302 1	15 kDa		unknown	2.2777	0.88083	100%	99%
389	TRUE	FALSE	AGAP011476-PA [Anopheles gambiae str. PEST]	gALL v3 rep c96461 5	130 kDa		unknown	0,99733	1,2551	99%	100%
390	TRUE	FALSE	PREDICTED: similar to cAMP-dependent protein kinase R1 CG3263	gALL v3 c775 5 (+1)	56 kDa		unknown	0.89028	1.3863	99%	100%
391	TRUE	FALSE	Heat shock 70 kDa protein cognate 3 precursor, putative [Pediculu	gALL v3 c2288 4	29 kDa	TRUE	unknown	3,9893	1,2551	100%	98%
392	TRUE	FALSE	putative alcohol dehydrogenase [Gryllotalpa orientalis]	gALL v3 c24039 5 (+1)	27 kDa		unknown	1.1388	0.88083	99%	99%
393	TRUE	FALSE	arp2/3 complex 20 kd subunit [Aedes aegypti]	gALL v3 c2610 6	51 kDa		unknown	3,888	1,0261	100%	98%
394	TRUE	FALSE	extracellular superoxide dismutase precursor [Pacifastacus leniuscı	gALL v3 c25166 5	13 kDa		unknown	1.6054	1.0261	98%	98%
395	TRUE	FALSE	PREDICTED: similar to glycyl-tRNA synthetase [Nasonia vitripennis	gALL v3 c1519 5	63 kDa		unknown	0,99733	0	99%	95%
396	TRUE	FALSE	PREDICTED: histone H4-like [Ailuropoda melanoleuca]	gALL v3 c54679 2	12 kDa		unknown	5.832	1.8851	100%	94%
397	TRUE	FALSE	hypothetical protein BRAFLDRAFT 287383 [Branchiostoma floridae	gALL v3 rep c98435 4 (+1)	32 kDa		unknown	1,944	5,6553	94%	100%
398	TRUE	FALSE	AT15141p [Drosophila melanogaster]	gALL v3 c61258 1	24 kDa	TRUE	unknown	7.776	3.7702	94%	100%
399	TRUE	FALSE	Profilin A [Trichomonas vaginalis G3]	gALL v3 c15994 5	14 kDa		unknown	5,832	1,8851	100%	94%
400	TRUE	FALSE	PREDICTED: similar to CG33519-PB isoform 1 [Apis mellifera]	gALL v3 rep c96575 6	274 kDa		unknown	1.371	1.3767	93%	100%
401	TRUE	FALSE	PREDICTED: similar to cytochrome c oxidase subunit Va [Tribolium	gALL v3 rep c96606 2	19 kDa		unknown	3,888	1,8851	99%	94%
402	TRUE	FALSE	Neuroglian precursor, putative [Pediculus humanus corporis]	gALL v3 c1239 3	65 kDa		unknown	1.371	1.3767	93%	100%
403	TRUE	FALSE	RecName: Full=40S ribosomal protein S4	gALL v3 rep c96277 5	28 kDa		unknown	0	2,6425	91%	100%
404	TRUE	FALSE	PREDICTED: similar to alcohol dehydrogenase [Nasonia vitripennis	gALL v3 rep c97598 5	24 kDa	TRUE	unknown	0	2.9602	0	100%
405	TRUE	FALSE	putative glycosyl hydrolase family5 [uncultured symbiotic protist o	gALL v3 c2080 6	29 kDa		unknown	2,2777	0,88083	100%	91%
406	TRUE	FALSE	hydroxyacyl dehydrogenase [Aedes aegypti]	gALL v3 rep c98264 6	16 kDa	TRUE	unknown	1,1388	0,88083	100%	91%
407	TRUE	FALSE	aspartate aminotransferase, cytoplasmic, putative [Pediculus huma	gALL v3 c12039 6	19 kDa		unknown	0,89028	2,0794	91%	100%
408	TRUE	FALSE	GJ15425 [Drosophila virilis]	gALL v3 c2764 1	37 kDa		unknown	0,89028	2,0794	91%	100%
409	TRUE	FALSE	PREDICTED: similar to X-prolyl aminopeptidase (aminopeptidase P	gALL v3 c2299 5	51 kDa		unknown	0,99733	1,2551	91%	100%
410	TRUE	FALSE	PREDICTED: similar to CG30104-PA, isoform A [Apis mellifera]	gALL v3 c2457 6	56 kDa		unknown	0,99733	1,2551	91%	100%
411	TRUE	FALSE	hypothetical protein TcasGA2 TC000807 [Tribolium castaneum]	gALL v3 c2155 4	50 kDa		unknown	0,99733	1,2551	91%	100%
412	TRUE	FALSE	juvenile hormone esterase-like protein Est1 [Reticulitermes flavipe	gALL v3 c20639 4	27 kDa		unknown	1,9947	1,2551	91%	100%
413	TRUE	FALSE	PREDICTED: similar to CAP, adenylate cyclase-associated protein 1	gALL v3 c33560 5	24 kDa	TRUE	unknown	0	2,7454	0	98%
414	TRUE	FALSE	PREDICTED: similar to proteosome subunit RPN2 isoform 1 [Acyrtl	gALL v3 c130 6	99 kDa		unknown	2,742	0,68834	100%	90%
415	TRUE	FALSE	PREDICTED: similar to pur-alpha [Nasonia vitripennis]	gALL v3 c1334 5	67 kDa		unknown	2.6708	0.69313	100%	89%
416	TRUE	FALSE	succinate dehydrogenase complex, subunit A, flavoprotein (Fp) [N	gALL v3 rep c98496 5	22 kDa		unknown	2,992	0,62757	100%	89%
417	TRUE	FALSE	moesin/ezrin/radixin [Aedes aegypti]	gALL v3 rep c96823 3	45 kDa	TRUE	unknown	3.9893	0.62757	100%	89%
418	TRUE	FALSE	serine protease inhibitor, putative [Pediculus humanus corporis]	gALL v3 rep c96414 5	44 kDa		unknown	0	2,5759	0	100%
419	TRUE	FALSE	PREDICTED: similar to AGAP006448-PB [Tribolium castaneum]	gALL v3 c2981 2	25 kDa		unknown	1.7806	0.69313	99%	89%
420	TRUE	FALSE	PREDICTED: similar to tau CG31057-PA, partial [Apis mellifera]	gALL v3 c379 1	169 kDa		unknown	3,2108	1,0261	100%	88%
421	TRUE	FALSE	PREDICTED: similar to T-complex protein 1 subunit gamma (TCP-1	gALL v3 c479 5	75 kDa		unknown	2.992	0.62757	100%	86%
422	TRUE	FALSE	MPA13 allergen [Periplaneta americana]	gALL v3 rep c98840 6 (+1)	23 kDa	TRUE	unknown	9,7201	7,5404	92%	94%
423	TRUE	FALSE	PREDICTED: similar to AGAP000767-PA [Tribolium castaneum]	gALL v3 c17044 2	17 kDa		unknown	0	3.0784	86%	100%
424	TRUE	FALSE	PREDICTED: similar to predicted protein [Tribolium castaneum]	gALL v3 rep c97128 6	21 kDa	TRUE	unknown	4,8161	0	100%	84%
425	TRUE	FALSE	phosphoenolpyruvate carboxykinase (GTP) [Treponema vincentii A	gALL v3 c27419 3	27 kDa		unknown	0	1.9483	0	100%
426	TRUE	FALSE	glucose dehydrogenase precursor, putative [Pediculus humanus co	gALL v3 rep c97150 5	63 kDa	TRUE	unknown	4,1308	0	92%	0
427	TRUE	FALSE	Spectrin alpha chain, putative [Pediculus humanus corporis]	gALL v3 c32472 4	33 kDa	TRUE	unknown	0	2.9458	0	100%
428	TRUE	FALSE	PREDICTED: hypothetical protein [Saccoglossus kowalevskii]	gALL v3 c86745 5	17 kDa		unknown	1,944	0	98%	83%
429	TRUE	FALSE	PREDICTED: similar to saposin isoform 1 [Tribolium castaneum]	gALL v3 rep c96602 4	103 kDa		unknown	0	2.0522	0	100%
430	TRUE	FALSE	PREDICTED: similar to putative Rho1 isoform 1 [Nasonia vitripenni	gALL v3 c5242 2	58 kDa		unknown	0	1,0261	81%	99%
431	TRUE	FALSE	4-hydroxyphenylpyruvate dioxygenase [Aedes aegypti]	gALL v3 c3583 6	80 kDa		unknown	2.6708	0	100%	78%
432	TRUE	FALSE	PREDICTED: similar to bifunctional purine biosynthesis protein [Na	gALL v3 c1703 4	36 kDa		unknown	0	1,8827	77%	100%
433	TRUE	FALSE	PREDICTED: similar to troponin C type IIa [Acyrthosiphon pisum]	gALL v3 rep c96201 4	29 kDa		unknown	0	2,0522	73%	100%
434	TRUE	FALSE	PREDICTED: similar to SRY interacting protein 1 CG10939-PA [Apis	gALL v3 c3561 2	57 kDa		unknown	0,89028	0	100%	0
435	TRUE	FALSE	PREDICTED: similar to ENSANGP00000015190 [Nasonia vitripennis	gALL v3 c849 5	40 kDa		unknown	0	1,2551	59%	100%
436	TRUE	FALSE	carbonic anhydrase [Aedes aegypti]	gALL v3 rep c97261 1	34 kDa	TRUE	unknown	0	1,7617	57%	100%
437	TRUE	FALSE	conserved hypothetical protein [Pediculus humanus corporis]	gALL v3 c3723 3	26 kDa		unknown	0	2,0522	49%	98%
438	TRUE	FALSE	PREDICTED: similar to putative zinc-containing alcohol dehydroger	gALL v3 c147 5	41 kDa		unknown	0	2,0794	38%	100%
439	TRUE	FALSE	takeout/JHBP-like protein [Diploptera punctata]	gALL v3 c12888 2	25 kDa		unknown	4,5553	0	100%	0
440	TRUE	FALSE	hypothetical protein TcasGA2 TC004721 [Tribolium castaneum]	gALL v3 rep c96383 5	453 kDa		unknown	0	2,065	0	100%
441	TRUE	FALSE	Spectrin alpha chain, putative [Pediculus humanus corporis]	gALL v3 c6480 5	31 kDa	TRUE	unknown	0	2,065	0	100%
442	TRUE	FALSE	chemosensory protein [Papilio xuthus]	gALL v3 rep c96263 6	30 kDa		unknown	0	5.6553	0	100%
443	TRUE	FALSE	Ves G 5 allergen precursor, putative [Pediculus humanus corporis]	gALL v3 rep c96316 6	34 kDa		unknown	0	4,1045	0	100%
444	TRUE	FALSE	hypothetical protein TcasGA2 TC001523 [Tribolium castaneum]	gALL v3 c10866 6	19 kDa	TRUE	unknown	0	1.7617	0	100%
445	TRUE	FALSE	mitochondrial F0 ATP synthase D chain, putative [Aedes aegypti]	gALL v3 rep c96640 5	24 kDa		unknown	4,8161	0	100%	0
446	TRUE	FALSE	Mn superoxide dismutase [Bombyx mori]	gALL v3 rep c97252 3	25 kDa		unknown	0	3.0784	0	100%
447	TRUE	FALSE	PREDICTED: similar to ribosomal protein S5 [Nasonia vitripennis]	gALL v3 c25150 1	19 kDa		unknown	2,7442	1,0261	93%	92%
448	TRUE	FALSE	PREDICTED: hypothetical protein [Nasonia vitripennis]	gALL v3 c3750 3	20 kDa		unknown	2.9413	1.8851	94%	89%
449	TRUE	FALSE	AGAP008487-PA [Anopheles gambiae str. PEST]	gALL v3 rep c97681 2 (+1)	46 kDa		unknown	2,9413	0	91%	88%
450	TRUE	FALSE	AGAP010588-PA [Anopheles gambiae str. PEST]	gALL v3 c10958 6	27 kDa	TRUE	unknown	0.99733	1.3207	91%	89%
451	TRUE	FALSE	cytoplasmic heat shock protein 70 [Trichomonas vaginalis G3]	gALL v3 c89195 4	15 kDa		unknown	0,99733	1,3207	91%	89%
452	TRUE	FALSE	actin 5C [Dolomedes minor]	gALL v3 c14594 6	24 kDa		unknown	0.89028	2.5782	86%	94%
453	TRUE	FALSE	DNA replication factor/protein phosphatase inhibitor SET/SPR-2, pı	gALL v3 rep c97242 6	19 kDa		unknown	0,89028	0,69313	91%	89%
454	TRUE	FALSE	conserved hypothetical protein [Pediculus humanus corporis]	gALL v3 c12458 3	27 kDa		unknown	0.89028	0	94%	73%
455	TRUE	FALSE	PREDICTED: similar to protease m1 zinc metalloprotease [Nasonia	gALL v3 c11520 1	41 kDa		unknown	0,99733	0,68834	91%	90%
456	TRUE	FALSE	PREDICTED: similar to CG2947-PA, isoform A isoform 1 [Apis melli	gALL v3 c964 5	52 kDa		unknown	0	2.7659	0	94%
457	TRUE	FALSE	PREDICTED: similar to Helicase at 25E CG7269-PA, isoform A [Api	gALL v3 rep c96988 6	49 kDa		unknown	0	1,3207	0	89%
458	TRUE	FALSE	cohesin subunit rad21, putative [Pediculus humanus corporis]	gALL v3 c7311 4	64 kDa		unknown	1.371	0.62757	86%	89%
459	TRUE	FALSE	2-oxoglutarate dehydrogenase, putative [Pediculus humanus corpc	gALL v3 c6074 6	31 kDa	TRUE	unknown	4,1129	1,3767	93%	90%
460	TRUE	FALSE	cytochrome P450 [Hodotermopsis sjoestedti]	gALL v3 rep c96479 3	104 kDa		unknown	0,99733	0,62757	91%	89%
461	TRUE	FALSE	PREDICTED: similar to GDP-4-keto-6-deoxy-D-mannose 3,5-epime	gALL v3 c23839 4 (+1)	19 kDa		unknown	0,89028	0	91%	78%
462	TRUE	FALSE	chitin binding peritrophin-A, putative [Pediculus humanus corporis	gALL v3 c9623 6	27 kDa		unknown	1,1388	0,69313	92%	89%
463	TRUE	FALSE	hypothetical protein [Plasmodium berghei strain ANKA]	gALL v3 rep c96340 2	36 kDa		unknown	0	2,5782	0	94%
464	TRUE	FALSE	protein-L-isoaspartate O-methyltransferase, putative [Pediculus hu	gALL v3 c14218 3	24 kDa		unknown	1,1388	0,88083	92%	91%
465	TRUE	FALSE	hypothetical protein TcasGA2 TC012685 [Tribolium castaneum]	gALL v3 c8515 5	33 kDa		unknown	2,0291	0	92%	44%
466	TRUE	FALSE	homologue of Sarcophaga 26,29kDa proteinase [Periplaneta ameri	gALL v3 c2152 5	46 kDa		unknown	1,6054	0	93%	83%
467	TRUE	FALSE	isocitrate dehydrogenase [Heliconius melpomene melpomene]	gALL v3 c13765 1	19 kDa	TRUE	unknown	0,89028	0,69313	91%	86%
468	TRUE	FALSE	TPA inf: troponin I isoform 6a2 [Apis mellifera]	gALL v3 rep c97379 6	29 kDa	TRUE	unknown	2,2777	3,5233	92%	91%
469	TRUE	FALSE	glutathione S-transferase O1 [Nasonia vitripennis]	gALL v3 c37119 2	28 kDa	TRUE	unknown	0	1.7617	53%	86%
470	TRUE	FALSE	MPA3 allergen [Periplaneta americana]	gALL v3 rep c96462 2	29 kDa		unknown	0	0,69313	71%	89%
471	TRUE	FALSE	PREDICTED: similar to CG3344-PA [Nasonia vitripennis]	gALL v3 rep c96839 4	51 kDa		unknown	0	1.8851	44%	94%
472	TRUE	FALSE	PREDICTED: similar to protein phosphatase 1, catalytic subunit, be	gALL v3 c5462 5	22 kDa		unknown	0	0,88083	60%	91%
473	TRUE	FALSE	PREDICTED: similar to ORF2-encoded protein, partial [Hydra magr	gALL v3 c7338 4	71 kDa		unknown	0.99733	0	86%	62%
474	TRUE	FALSE	PREDICTED: similar to Ubiquitin activating enzyme 1 CG1782-PA i:	gALL v3 c9060 3	20 kDa		unknown	0,99733	0	91%	60%
475	TRUE	FALSE	PREDICTED: similar to PDCD-5 CG13072-PA [Tribolium castaneum	gALL v3 c2159 5	35 kDa		unknown	3.888	1.8851	100%	94%
476	TRUE	FALSE	PREDICTED: similar to prefoldin subunit 1 [Apis mellifera]	gALL v3 c8110 6	21 kDa		unknown	3,888	1,8851	100%	94%
477	TRUE	FALSE	PREDICTED: similar to Fatty acid-binding protein, adipocyte (AFAB	gALL v3 c2623 6	79 kDa		unknown	1.944	3.7702	94%	100%
478	TRUE	FALSE	PREDICTED: similar to ribosomal protein P1 [Nasonia vitripennis]	gALL v3 rep c96742 5 (+2)	25 kDa		unknown	1,944	3,7702	94%	100%
479	TRUE	FALSE	transcription elongation factor B polypeptide, putative [Pediculus h	gALL v3 c37183 4	21 kDa		unknown	3.888	1.8851	100%	94%
480	TRUE	FALSE	PREDICTED: similar to Myosin-2 essential light chain (Myosin II es	gALL v3 rep c96735 2	22 kDa		unknown	1,944	3,7702	94%	99%
481	TRUE	FALSE	PREDICTED: similar to RNA-binding protein musashi/mRNA cleava	gALL v3 c2068 3	94 kDa		unknown	0	2.9112	0	99%
482	TRUE	FALSE	PREDICTED: similar to AGAP004396-PA [Tribolium castaneum]	gALL v3 c14387 1	24 kDa		unknown	0,89028	1,8851	99%	94%
483	TRUE	FALSE	beta-mannosidase precursor, putative [Pediculus humanus corporis	gALL v3 c1701 2	60 kDa		unknown	1.371	1.3767	93%	100%
484	TRUE	FALSE	PREDICTED: similar to AGAP007123-PA [Tribolium castaneum]	gALL v3 c2613 4	46 kDa		unknown	1,371	1,3767	93%	100%
485	TRUE	FALSE	hypothetical protein TcasGA2 TC005186 [Tribolium castaneum]	gALL v3 c629 3	62 kDa		unknown	1.371	1.3767	93%	100%
486	TRUE	FALSE	LP20836p [Drosophila melanogaster]	gALL v3 c2500 6	29 kDa		unknown	1,371	1,3767	93%	100%
487	TRUE	FALSE	PREDICTED: similar to tripeptidyl peptidase II [Apis mellifera]	gALL v3 c42629 2	13 kDa		unknown	1,371	0,68834	93%	100%
488	TRUE	FALSE	PREDICTED: similar to Gb1-cadherin [Acyrthosiphon pisum]	gALL v3 c16033 5	18 kDa		unknown	0	1,3767	93%	100%
489	TRUE	FALSE	PREDICTED: ras-related protein Rab-11B-like [Ailuropoda melanol	gALL v3 c11481 5 (+2)	38 kDa		unknown	1,6054	1,0261	93%	99%
490	TRUE	FALSE	PREDICTED: peroxiredoxin 6-like [Saccoglossus kowalevskii]	gALL v3 c3323 1	48 kDa		unknown	1,6054	1,0261	93%	99%
491	TRUE	FALSE	GI18446 [Drosophila mojavensis]	gALL v3 c39151 5 (+1)	22 kDa		unknown	3,888	1,0261	100%	92%
492	TRUE	FALSE	conserved hypothetical protein [Pediculus humanus corporis]	gALL v3 c8369 3	18 kDa		unknown	3,2108	1,0261	100%	92%
493	TRUE	FALSE	APAF1 interacting protein [Nasonia vitripennis]	gALL v3 c16700 6	33 kDa		unknown	1,1388	1,7617	92%	100%
494	TRUE	FALSE	PREDICTED: similar to 20-beta-hydroxysteroid dehydrogenase [Ac	gALL v3 c16878 5	34 kDa		unknown	1,1388	1,7617	92%	100%
495	TRUE	FALSE	putative glycosyl hydrolase family5 [uncultured symbiotic protist o	gALL v3 c25216 2	27 kDa		unknown	1,1388	1,7617	92%	100%
496	TRUE	FALSE	PREDICTED: similar to ribose 5-phosphate isomerase A (ribose 5-ı	gALL v3 c25770 5	36 kDa		unknown	1.1388	1.7617	92%	100%
497	TRUE	FALSE	serine protease [Aedes aegypti]	gALL v3 c4109 4	29 kDa		unknown	1,1388	1,7617	92%	100%
498	TRUE	FALSE	proteasome alpha 4 subunit [Plutella xylostella]	gALL v3 rep c98290 2	16 kDa	TRUE	unknown	1.1388	1.7617	92%	100%
499	TRUE	FALSE	60S ribosomal protein L17, putative [Pediculus humanus corporis]	gALL v3 rep c96474 1	20 kDa		unknown	3,2108	1,0261	100%	92%
500	TRUE	FALSE	PREDICTED: similar to CG32019-PA [Tribolium castaneum]	gALL v3 rep c96365 3	310 kDa		unknown	1.371	0.68834	93%	99%
501	TRUE	FALSE	PREDICTED: similar to Succinate dehydrogenase B CG3283-PA [Ap	gALL v3 c52844 4	19 kDa		unknown	1,1388	0,88083	92%	99%
502	TRUE	FALSE	PREDICTED: similar to fk506-binding protein [Nasonia vitripennis]	gALL v3 c16489 6	25 kDa		unknown	0	0.88083	92%	99%
503	TRUE	FALSE	retinoid-inducible serine carboxypeptidase (serine carboxypeptidas	gALL v3 c44053 6	29 kDa		unknown	0,89028	1,2551	91%	100%
504	TRUE	FALSE	PREDICTED: similar to glycerol-3-phosphate dehydrogenase [Tribo	gALL v3 c5743 6	42 kDa	TRUE	unknown	0.89028	2.0794	91%	100%
505	TRUE	FALSE	paramyosin, putative [Pediculus humanus corporis]	gALL v3 c6571 4	48 kDa		unknown	0,99733	1,2551	91%	100%
506	TRUE	FALSE	hypothetical protein BRAFLDRAFT 68589 [Branchiostoma floridae]	gALL v3 c8934 2	19 kDa		unknown	0.99733	1.2551	91%	100%
507	TRUE	FALSE	soldier-caste specific protein 1 [Hodotermopsis japonica]	gALL v3 rep c96505 4 (+1)	26 kDa		unknown	3,275	0	100%	0
508	TRUE	FALSE	PREDICTED: similar to 3-hydroxybutyrate dehydrogenase type 2 [	gALL v3 c3950 5	32 kDa		unknown	1.1388	0.88083	92%	99%
509	TRUE	FALSE	hypothetical protein AaeL AAEL008274 [Aedes aegypti]	gALL v3 rep c96878 5	27 kDa		unknown	0,89028	1,3863	91%	100%
510	TRUE	FALSE	GK19773 [Drosophila willistoni]	gALL v3 c12702 2	16 kDa		unknown	0	1.8851	60%	94%
511	TRUE	FALSE	Endoplasmic reticulum protein ERp29, putative [Pediculus humanu	gALL v3 c14365 3	38 kDa		unknown	1,1388	0,88083	99%	91%
512	TRUE	FALSE	AGAP005400-PA [Anopheles gambiae str. PEST]	gALL v3 rep c96259 3	132 kDa		unknown	0.89028	0.69313	91%	99%
513	TRUE	FALSE	PREDICTED: similar to lysosomal alpha-mannosidase (mannosidas	gALL v3 c4588 6 (+1)	55 kDa		unknown	0	2,0746	0	100%

514	TRUE	FALSE	villin, putative [Ixodes scapularis]	gALL v3 c5417 6	23 kDa	TRUE	unknown	2,742	0,68834	100%	90%
515	TRUE	FALSE	Epididymal secretory protein E1 precursor, putative [Pediculus hum	gALL v3 rep c96311 3	46 kDa		unknown	0	2.9331	0	100%
516	TRUE	FALSE	PREDICTED: similar to CG31692-PB [Nasonia vitripennis]	gALL v3 c21048 1	22 kDa		unknown	0,89028	0,69313	91%	98%
517	TRUE	FALSE	Hsp60 protein, putative [Pediculus humanus corporis]	gALL v3 c7674 1	19 kDa		unknown	0.99733	0.62757	91%	98%
518	TRUE	FALSE	[Culex quinquefasciatus]	gALL v3 rep c105407 1fixR	?	TRUE	unknown	6,55	0	98%	0
519	TRUE	FALSE	venom dipeptidylpeptidase IV [Apis mellifera]	gALL v3 c2091 1	52 kDa		unknown	1.7806	0.69313	100%	89%
520	TRUE	FALSE	guanine nucleotide-binding protein subunit beta-like [Microplitis m	gALL v3 rep c97013 2	75 kDa		unknown	1,7806	0,69313	100%	89%
521	TRUE	FALSE	protein TcasGA2 TC015554 [Tribolium castaneum]	gALL v3 rep c106355 1hypothet	?	TRUE	unknown	5.6941	11.451	98%	91%
522	TRUE	FALSE	AGAP004366-PA [Anopheles gambiae str. PEST]	gALL v3 c2508 4	30 kDa		unknown	1,9947	0,62757	100%	89%
523	TRUE	FALSE	asparagine synthetase, putative [Pediculus humanus corporis]	gALL v3 c32990 3	55 kDa		unknown	1.9947	0.62757	100%	89%
524	TRUE	FALSE	ATP-citrate lyase [Glossina morsitans morsitans]	gALL v3 c8944 4	54 kDa		unknown	0	1,3159	0	99%
525	TRUE	FALSE	4-hydroxybutyrate coenzyme A transferase, putative [Pediculus hu	gALL v3 rep c97637 3	22 kDa		unknown	1.7806	0.62757	100%	88%
526	TRUE	FALSE	PREDICTED: similar to cAMP-dependent protein kinase C1 [Acyrth	gALL v3 c280 4	59 kDa		unknown	0,89028	0,69313	99%	89%
527	TRUE	FALSE	PREDICTED: similar to rab gdp-dissociation inhibitor [Tribolium cas	gALL v3 c2078 2	23 kDa		unknown	0	0.69313	99%	89%
528	TRUE	FALSE	T-complex protein 1 subunit theta, putative [Pediculus humanus co	gALL v3 c6461 5	17 kDa	TRUE	unknown	0,99733	1,2551	98%	89%
529	TRUE	FALSE	aldehyde dehydrogenase [Aedes aegypti]	gALL v3 c621 3	19 kDa	TRUE	unknown	0.99733	1.2551	98%	89%
530	TRUE	FALSE	PREDICTED: similar to adenosylhomocysteinase [Tribolium castane	gALL v3 rep c96506 5	41 kDa		unknown	0	1,3863	86%	100%
531	TRUE	FALSE	elongation factor-1 alpha [Periplaneta americana]	gALL v3 rep c96717 1	47 kDa		unknown	1.944	2.0522	94%	92%
532	TRUE	FALSE	ribosomal protein L7e [Scarabaeus laticollis]	gALL v3 rep c96429 5	27 kDa		unknown	1,1388	0	100%	0
533	TRUE	FALSE	PREDICTED: similar to 14-3-3 CG17870-PA, isoform A isoform 2 [/	gALL v3 rep c97673 2	22 kDa	TRUE	unknown	5,6941	3,5233	92%	91%
534	TRUE	FALSE	PREDICTED: similar to Annexin IX CG5730-PC [Tribolium castaneu	gALL v3 c3644 4	32 kDa		unknown	1,1388	0,69313	98%	84%
535	TRUE	FALSE	PREDICTED: similar to calbindin-32 [Acyrthosiphon pisum]	gALL v3 c55176 3	11 kDa		unknown	1,1388	0	99%	83%
536	TRUE	FALSE	pro-phenol oxidase subunit 2 [Tribolium castaneum]	gALL v3 c20262 4	93 kDa	TRUE	unknown	1,9947	0	100%	82%
537	TRUE	FALSE	26S proteasome non-ATPase regulatory subunit, putative [Pediculu	gALL v3 c381 4	32 kDa		unknown	0	0,62757	83%	98%
538	TRUE	FALSE	PREDICTED: similar to AGAP010885-PA [Tribolium castaneum]	gALL v3 c841 5	40 kDa		unknown	0	0,69313	91%	89%
539	TRUE	FALSE	heat shock protein 60 [Polypedilum vanderplanki]	gALL v3 c13450 6	14 kDa	TRUE	unknown	2,992	1,2551	91%	89%
540	TRUE	FALSE	glucose-6-phosphate isomerase [Euphydryas aurinia]	gALL v3 c21817 1	26 kDa	TRUE	unknown	0,99733	1,2551	91%	89%
541	TRUE	FALSE	troponin I [Polyrhachis vicina]	gALL v3 rep c96367 5	43 kDa	TRUE	unknown	1,1388	1,0261	87%	92%
542	TRUE	FALSE	PREDICTED: similar to thioredoxin reductase isoform 2 [Tribolium	gALL v3 c5528 6	55 kDa		unknown	0	1.2551	0	100%
543	TRUE	FALSE	PREDICTED: similar to putative Rab7 isoform 1 [Nasonia vitripenni	gALL v3 c11038 3	27 kDa		unknown	3,2108	0	100%	77%
544	TRUE	FALSE	hypothetical protein AaeL AAEL012860 [Aedes aegypti]	gALL v3 c42206 2	21 kDa		unknown	0	1.7617	76%	100%
545	TRUE	FALSE	PREDICTED: similar to isocitrate dehydrogenase [Acyrthosiphon pi	gALL v3 rep c97131 6	23 kDa		unknown	0	0,69313	77%	99%
546	TRUE	FALSE	ATPase, class VI, type 11C, putative [Pediculus humanus corporis]	gALL v3 c6147 4	55 kDa		unknown	0	0.62757	0	100%
547	TRUE	FALSE	PREDICTED: similar to CG32626-PA, isoform A isoform 2 [Apis me	gALL v3 c5248 6	51 kDa		unknown	0	1,3767	0	100%
548	TRUE	FALSE	lambda-crystallin [Culex quinquefasciatus]	gALL v3 c4221 2	26 kDa		unknown	0.89028	0	98%	75%
549	TRUE	FALSE	hypothetical protein TcasGA2 TC014157 [Tribolium castaneum]	gALL v3 c8548 4	35 kDa	TRUE	unknown	0	1,3767	66%	100%
550	TRUE	FALSE	PREDICTED: similar to hydroxyacyl-coenzyme A dehydrogenase [T	gALL v3 c5602 5	81 kDa		unknown	0	0.62757	66%	98%
551	TRUE	FALSE	14-3-3 protein [Trichomonas vaginalis G3]	gALL v3 c3390 5	26 kDa	TRUE	unknown	0	0,88083	0	98%
552	TRUE	FALSE	PREDICTED: similar to CG17331-PA isoform 1 [Apis mellifera]	gALL v3 c4610 2	30 kDa		unknown	2.2777	0	99%	61%
553	TRUE	FALSE	AGAP004742-PA [Anopheles gambiae str. PEST]	gALL v3 c2313 6	33 kDa		unknown	0	0,68834	62%	98%
554	TRUE	FALSE	RecName: Full=Juvenile hormone epoxide hydrolase 1; AltName: I	gALL v3 rep c96438 2	66 kDa		unknown	0	1.3863	57%	100%
555	TRUE	FALSE	40S ribosomal protein S6, putative [Pediculus humanus corporis]	gALL v3 rep c96800 5	28 kDa		unknown	0	1,3863	75%	81%
556	TRUE	FALSE	PREDICTED: similar to S14e ribosomal protein [Tribolium castaneu	gALL v3 rep c96523 2	20 kDa		unknown	0	3.7702	53%	100%
557	TRUE	FALSE	protein C6orf168, putative [Pediculus humanus corporis]	gALL v3 rep c97370 6	42 kDa		unknown	0	1,0261	53%	99%
558	TRUE	FALSE	PREDICTED: similar to SH3 domain-binding glutamic acid-rich prot	gALL v3 c8342 6	63 kDa		unknown	1.1388	0	91%	58%
559	TRUE	FALSE	GM18672 [Drosophila sechellia]	gALL v3 c25128 4	30 kDa		unknown	2,2777	0	100%	48%
560	TRUE	FALSE	juvenile hormone esterase-like protein Est1 [Reticulitermes flavipe	gALL v3 c350 2	55 kDa		unknown	0	1,2551	48%	100%
561	TRUE	FALSE	PREDICTED: hypothetical protein [Nasonia vitripennis]	gALL v3 c5578 1	31 kDa		unknown	0	1,3767	0	100%
562	TRUE	FALSE	Ves G 5 allergen precursor, putative [Pediculus humanus corporis]	gALL v3 c907 5	45 kDa		unknown	1,6054	0	98%	46%
563	TRUE	FALSE	glucose-6-phosphate isomerase [Nasonia vitripennis]	gALL v3 c2443 5	33 kDa		unknown	1,9947	0	100%	43%
564	TRUE	FALSE	PREDICTED: similar to Annexin IX CG5730-PC [Tribolium castaneu	gALL v3 c493 4	39 kDa		unknown	0	0,69313	43%	99%
565	TRUE	FALSE	troponin C type IIb [Apis mellifera]	gALL v3 c4063 6	18 kDa		unknown	3,888	0	100%	41%
566	TRUE	FALSE	E1b-55kD-associated protein, putative [Pediculus humanus corpori	gALL v3 rep c98140 5	25 kDa		unknown	2,742	0	99%	37%
567	TRUE	FALSE	PREDICTED: similar to Fimbrin CG8649-PA, isoform A [Apis mellife	gALL v3 c6599 5	37 kDa		unknown	0,99733	0	98%	35%
568	TRUE	FALSE	adenlylate kinase [Trichomonas vaginalis G3]	gALL v3 c24145 4	21 kDa		unknown	0	2,6425	0	100%
569	TRUE	FALSE	Aspartate aminotransferase, cytoplasmic [Caligus clemensi]	gALL v3 c5742 2	68 kDa		unknown	0	2.0794	0	100%
570	TRUE	FALSE	PREDICTED: similar to CRALBP [Tribolium castaneum]	gALL v3 c8020 3	42 kDa		unknown	0	2,0794	0	100%
571	TRUE	FALSE	PREDICTED: similar to CG17374 CG17374-PA [Acyrthosiphon pisu	gALL v3 c9200 1	57 kDa		unknown	0	2.065	0	100%
572	TRUE	FALSE	GTP-binding protein SAR1B, putative [Pediculus humanus corporis	gALL v3 rep c96667 2	43 kDa		unknown	0	3,0784	0	100%
573	TRUE	FALSE	PREDICTED: similar to hu li tai shao CG9325-PB, isoform B [Apis n	gALL v3 rep c96908 5	79 kDa		unknown	0	1.8827	0	100%
574	TRUE	FALSE	PREDICTED: similar to fatty acid synthase [Nasonia vitripennis]	gALL v3 c6288 5	41 kDa		unknown	0	1,3767	0	100%
575	TRUE	FALSE	PREDICTED: similar to RE11240p [Nasonia vitripennis]	gALL v3 c14523 6	40 kDa		unknown	0	1.2551	0	100%
576	TRUE	FALSE	PREDICTED: similar to putative alcohol dehydrogenase [Tribolium	gALL v3 c2221 3	37 kDa		unknown	0	1,7617	0	100%
577	TRUE	FALSE	hypothetical protein Phum PHUM538590 [Pediculus humanus corp	gALL v3 rep c96519 1	59 kDa		unknown	0	3.7702	0	100%
578	TRUE	FALSE	N(4)-(Beta-N-acetylglucosaminyl)-L-asparaginase [Culex quinquefa	gALL v3 c24279 6	27 kDa		unknown	3,888	0	100%	0
579	TRUE	FALSE	putative cytosolic malate dehydrogenase 2 [Histomonas meleagrid	gALL v3 c4617 5	23 kDa	TRUE	unknown	0	1.3863	0	100%
580	TRUE	FALSE	PREDICTED: similar to Hsc70/Hsp90-organizing protein HOP [Nasc	gALL v3 c8019 2	26 kDa		unknown	1,9947	0	100%	0
581	TRUE	FALSE	PREDICTED: similar to ENSANGP00000012893 [Nasonia vitripennis	gALL v3 c46968 4	39 kDa	TRUE	unknown	1.9947	0	100%	0
582	TRUE	FALSE	PREDICTED: similar to ENSANGP00000012364 [Nasonia vitripennis	gALL v3 c36399 6	20 kDa		unknown	2,742	0	100%	0
583	TRUE	FALSE	chemosensory protein [Bemisia tabaci]	gALL v3 rep c96673 1 (+1)	26 kDa		unknown	0	3.7702	0	100%
584	TRUE	FALSE	PREDICTED: similar to fatty acid synthase [Tribolium castaneum]	gALL v3 c9467 5	42 kDa		unknown	0	0,68834	0	100%
585	TRUE	FALSE	PREDICTED: similar to Ras-related protein Rac1 isoform 1 [Apis m	gALL v3 c49293 6	21 kDa		unknown	0	1.0261	0	100%
586	TRUE	FALSE	PREDICTED: similar to predicted protein [Tribolium castaneum]	gALL v3 c21809 5	19 kDa	TRUE	unknown	4,8161	0	100%	0
587	TRUE	FALSE	PREDICTED: similar to Spectrin beta chain [Apis mellifera]	gALL v3 c5488 6	26 kDa		unknown	0	0,68834	0	100%
588	TRUE	FALSE	PREDICTED: similar to inosine-uridine preferring nucleoside hydrol	gALL v3 c13759 6	43 kDa		unknown	0	0,69313	0	100%
589	TRUE	FALSE	fatty acid synthase, putative [Pediculus humanus corporis]	gALL v3 c6035 1	36 kDa		unknown	0	0,68834	0	100%
590	TRUE	FALSE	PREDICTED: similar to GA19489-PA [Nasonia vitripennis]	gALL v3 rep c96553 6	50 kDa		unknown	0	0,62757	0	100%
591	TRUE	FALSE	PREDICTED: similar to AGAP004744-PA [Tribolium castaneum]	gALL v3 c2547 4	73 kDa		unknown	0,89028	0	99%	0
592	TRUE	FALSE	Chain A, Crystal Structure Of Beta-Glucosidase From Termite Neot	gALL v3 c406 4	46 kDa		unknown	0,99733	0	99%	0
593	TRUE	FALSE	PREDICTED: similar to 3-oxoacyl-[acyl-carrier-protein] reductase [	gALL v3 c20762 1	18 kDa		unknown	0	1,7617	0	91%
594	TRUE	FALSE	zinc finger protein [Bombyx mori]	gALL v3 rep c96409 1	30 kDa		unknown	1,7806	0	91%	0
595	TRUE	FALSE	hypothetical protein BRAFLDRAFT 119204 [Branchiostoma floridae	gALL v3 c23093 2	27 kDa		unknown	1,944	1,8851	94%	94%
596	TRUE	FALSE	putative salivary secreted protein [Triatoma infestans]	gALL v3 c54087 1 (+1)	15 kDa		unknown	1.944	1.8851	94%	94%
597	TRUE	FALSE	cuticular protein [Tribolium castaneum]	gALL v3 c8529 4 (+1)	35 kDa		unknown	1,944	1,8851	94%	94%
598	TRUE	FALSE	hypothetical protein TcasGA2 TC001048 [Tribolium castaneum]	gALL v3 rep c96207 5 (+1)	26 kDa	TRUE	unknown	3.888	3.7702	94%	94%
599	TRUE	FALSE	sterol carrier protein-2, putative [Aedes aegypti]	gALL v3 rep c96825 5	48 kDa		unknown	1,944	1,8851	94%	94%
600	TRUE	FALSE	PREDICTED: similar to vesicle-associated membrane protein 2 [Na	gALL v3 c11264 2 (+4)	15 kDa		unknown	1.6054	1.8851	93%	94%
601	TRUE	FALSE	PREDICTED: similar to ubiquitin conjugating enzyme E2 [Tribolium	gALL v3 c9751 4	28 kDa		unknown	3,5494	0	94%	0
602	TRUE	FALSE	pacifastin-related serine protease inhibitor precursor [Locusta mig	gALL v3 rep c96264 3	145 kDa		unknown	0	2.9112	0	94%
603	TRUE	FALSE	PREDICTED: similar to ATP synthase delta chain, mitochondrial [Tr	gALL v3 rep c97331 1 (+1)	25 kDa		unknown	1,944	1,0261	94%	92%
604	TRUE	FALSE	uv excision repair protein rad23, putative [Pediculus humanus corp	gALL v3 c2422 2	68 kDa		unknown	1.1388	1.8851	92%	94%
605	TRUE	FALSE	PREDICTED: similar to Si:dkey-189p24.5 protein [Tribolium castan	gALL v3 c15945 5	19 kDa		unknown	1,6054	1,0261	93%	92%
606	TRUE	FALSE	PREDICTED: similar to Chd64 CG14996-PB [Apis mellifera]	gALL v3 c52432 1	21 kDa	TRUE	unknown	3.2108	2.0522	93%	92%
607	TRUE	FALSE	50 kDa midgut protein [Phlebotomus papatasi]	gALL v3 rep c96212 3	55 kDa		unknown	1,6054	1,0261	93%	92%
608	TRUE	FALSE	hypothetical protein TcasGA2 TC014014 [Tribolium castaneum]	gALL v3 rep c97044 6	29 kDa		unknown	1.6054	1.0261	93%	92%
609	TRUE	FALSE	salivary protein MYS2 precursor [Triatoma brasiliensis]	gALL v3 c10314 1 (+1)	18 kDa		unknown	1,944	1,8851	92%	94%
610	TRUE	FALSE	PREDICTED: similar to acyl-coa dehydrogenase [Acyrthosiphon pis	gALL v3 c8671 4 (+1)	45 kDa		unknown	0.99733	1.8851	91%	94%
611	TRUE	FALSE	PREDICTED: similar to squid CG16901-PC, isoform C [Apis mellifer	gALL v3 c1574 4	63 kDa		unknown	1,1388	1,0261	92%	92%
612	TRUE	FALSE	PREDICTED: similar to cathepsin F like protease [Nasonia vitripenn	gALL v3 rep c96610 3	131 kDa		unknown	1.1388	1.0261	92%	92%
613	TRUE	FALSE	fibrinolytic enzyme [Eupolyphaga sinensis]	gALL v3 rep c96793 3	25 kDa		unknown	1,1388	1,0261	92%	92%
614	TRUE	FALSE	Ankyrin [Sulfolobus islandicus Y.N.15.51]	gALL v3 c1335 6 (+1)	23 kDa		unknown	0	1,9069	0	92%
615	TRUE	FALSE	Parcxpwnx01 [Periplaneta americana]	gALL v3 rep c96406 5	39 kDa	TRUE	unknown	0	4,5494	0	92%
616	TRUE	FALSE	CG6696 [Drosophila melanogaster]	gALL v3 rep c96550 6	25 kDa		unknown	0	1,9069	0	92%
617	TRUE	FALSE	PREDICTED: similar to TatD DNase domain containing 1 [Apis mell	gALL v3 c11418 2 (+1)	23 kDa		unknown	1,1388	0,88083	92%	91%
618	TRUE	FALSE	PREDICTED: similar to Elongation factor 1 beta CG6341-PA [Triboli	gALL v3 c16250 5	20 kDa	TRUE	unknown	1,1388	0,88083	92%	91%
619	TRUE	FALSE	conserved hypothetical protein [Pediculus humanus corporis]	gALL v3 c4642 3	48 kDa		unknown	1,1388	0,88083	92%	91%
620	TRUE	FALSE	hydroxyacyl dehydrogenase [Aedes aegypti]	gALL v3 c7102 3	24 kDa	TRUE	unknown	2,2777	0,88083	92%	91%
621	TRUE	FALSE	PREDICTED: similar to RH74717p [Nasonia vitripennis]	gALL v3 c72939 6	14 kDa		unknown	1,1388	0,88083	92%	91%
622	TRUE	FALSE	GA18442 [Drosophila pseudoobscura pseudoobscura]	gALL v3 c87575 3	17 kDa		unknown	1,1388	0,88083	92%	91%
623	TRUE	FALSE	FK506-binding protein [Bombyx mori]	gALL v3 c9919 2	16 kDa		unknown	1.1388	0.88083	92%	91%
624	TRUE	FALSE	PREDICTED: similar to CG3937-PD [Nasonia vitripennis]	gALL v3 c103357 4	19 kDa		unknown	1,371	0,68834	93%	90%
625	TRUE	FALSE	hypothetical protein TcasGA2 TC012309 [Tribolium castaneum]	gALL v3 c15754 4	28 kDa		unknown	1.371	0.68834	93%	90%
626	TRUE	FALSE	heat shock factor binding protein, putative [Aedes aegypti]	gALL v3 c21781 5	18 kDa		unknown	1,371	0,68834	93%	90%
627	TRUE	FALSE	GJ20980 [Drosophila virilis]	gALL v3 c3593 3	35 kDa		unknown	1.371	0.68834	93%	90%
628	TRUE	FALSE	ATP-citrate synthase [Aedes aegypti]	gALL v3 c66756 4	20 kDa		unknown	1,371	0,68834	93%	90%
629	TRUE	FALSE	PREDICTED: similar to 5-oxoprolinase (ATP-hydrolysing) [Apis mel	gALL v3 c89388 4	16 kDa		unknown	1.371	0.68834	93%	90%
630	TRUE	FALSE	PREDICTED: dihydropyrimidine dehydrogenase [NADP+] [Xenopus	gALL v3 rep c97627 6	22 kDa		unknown	1,371	0,68834	93%	90%
631	TRUE	FALSE	glyceraldehyde-3-phosphate dehydrogenase [Pseudotrichonympha	gALL v3 c4812 6	30 kDa		unknown	0	2.5782	0	94%
632	TRUE	FALSE	PREDICTED: similar to AGAP002000-PA [Tribolium castaneum]	gALL v3 c74796 2	14 kDa		unknown	0,99733	1,8851	89%	94%
633	TRUE	FALSE	hypothetical protein TcasGA2 TC004723 [Tribolium castaneum]	gALL v3 c7030 1 (+1)	19 kDa		unknown	0	2.5127	0	94%
634	TRUE	FALSE	GL22014 [Drosophila persimilis]	gALL v3 c6811 3	55 kDa		unknown	1,6054	1,0261	90%	92%
635	TRUE	FALSE	PREDICTED: similar to ENSANGP00000027991 [Nasonia vitripennis	gALL v3 c5312 1	28 kDa		unknown	0	1.7145	0	92%
636	TRUE	FALSE	conserved hypothetical protein [Pediculus humanus corporis]	gALL v3 c22880 4	62 kDa		unknown	1,371	0,68834	92%	90%
637	TRUE	FALSE	PREDICTED: similar to luciferin-regenerating enzyme [Tribolium ca	gALL v3 c11532 3	40 kDa		unknown	0	0.68834	92%	90%
638	TRUE	FALSE	putative vacuolar ATP synthase subunit D [Homalodisca vitripennis	gALL v3 c4168 2	32 kDa		unknown	1,1388	0,69313	92%	89%
639	TRUE	FALSE	beta tubulin [Holomastigotoides mirabile]	gALL v3 rep c96534 2 (+2)	38 kDa	TRUE	unknown	1.1388	1.3863	92%	89%
640	TRUE	FALSE	conserved hypothetical protein [Pediculus humanus corporis]	gALL v3 rep c96702 1	98 kDa		unknown	2,7442	0	92%	0
641	TRUE	FALSE	b-glucosidase, glycoside hydrolase family 3 protein [Pedobacter sp	gALL v3 c13414 4	29 kDa		unknown	0,99733	0,68834	91%	90%
642	TRUE	FALSE	PREDICTED: similar to juvenile hormone esterase [Acyrthosiphon	gALL v3 c14436 2	18 kDa	FALSE	unknown	0,99733	0,68834	91%	90%
643	TRUE	FALSE	translocase [Ixodes scapularis]	gALL v3 rep c102559 6ADP/ATP	?		unknown	1,1388	0	92%	89%
644	TRUE	FALSE	hypothetical protein Phum PHUM516380 [Pediculus humanus corp	gALL v3 c5164 6	34 kDa		unknown	3,5494	0	93%	0
645	TRUE	FALSE	PREDICTED: similar to conserved hypothetical protein [Tribolium c	gALL v3 c1135 5	68 kDa		unknown	0	1,8851	87%	93%
646	TRUE	FALSE	hippocalcin [Aedes aegypti]	gALL v3 c2498 4	132 kDa		unknown	1,944	1,0261	94%	87%
647	TRUE	FALSE	serpin peptidase inhibitor 30 [Tribolium castaneum]	gALL v3 c15967 5	26 kDa		unknown	0,89028	0,69313	91%	89%
648	TRUE	FALSE	PREDICTED: CG10098-like [Saccoglossus kowalevskii]	gALL v3 c20884 1	31 kDa		unknown	0,89028	0,69313	91%	89%
649	TRUE	FALSE	PREDICTED: similar to AGAP006099-PA isoform 2 [Tribolium casta	gALL v3 rep c97962 5	15 kDa		unknown	0,89028	0,69313	91%	89%
650	TRUE	FALSE	aldose reductase, putative [Pediculus humanus corporis]	gALL v3 rep c98481 5	28 kDa	TRUE	unknown	0.89028	2.0794	91%	89%
651	TRUE	FALSE	d-lactate dehydrogenase 2, putative [Aedes aegypti]	gALL v3 c10141 4	36 kDa		unknown	0	0,62757	91%	89%
652	TRUE	FALSE	Chain A, Ketose Reductase (Sorbitol Dehydrogenase) From Silverle	gALL v3 rep c97550 5	21 kDa		unknown	0.89028	0.69313	91%	89%
653	TRUE	FALSE	PREDICTED: similar to CG14516-PA, isoform A isoform 1 [Apis me	gALL v3 rep c96909 2	52 kDa		unknown	1,371	0,68834	93%	87%
654	TRUE	FALSE	PREDICTED: similar to AGAP003869-PA [Tribolium castaneum]	gALL v3 c10778 1	35 kDa		unknown	0.99733	0.62757	91%	89%
655	TRUE	FALSE	Trehalase precursor, putative [Pediculus humanus corporis]	gALL v3 c11076 6	21 kDa		unknown	0,99733	0,62757	91%	89%
656	TRUE	FALSE	alkaline phosphatase [Heliothis virescens]	gALL v3 c11396 6	25 kDa		unknown	0.99733	0.62757	91%	89%
657	TRUE	FALSE	NADP-dependent malic enzyme, putative [Pediculus humanus corp	gALL v3 c17029 2	22 kDa	TRUE	unknown	0,99733	0,62757	91%	89%
658	TRUE	FALSE	conserved hypothetical protein [Pediculus humanus corporis]	gALL v3 c1795 2	20 kDa		unknown	0.99733	0.62757	91%	89%
659	TRUE	FALSE	AGAP005860-PA [Anopheles gambiae str. PEST]	gALL v3 c20323 1	17 kDa		unknown	0,99733	0,62757	91%	89%
660	TRUE	FALSE	luciferase homologue [Agrypnus binodulus binodulus]	gALL v3 c20735 5	24 kDa		unknown	0.99733	0.62757	91%	89%
661	TRUE	FALSE	PREDICTED: similar to putative esterase [Tribolium castaneum]	gALL v3 c21767 2	22 kDa		unknown	0,99733	0,62757	91%	89%
662	TRUE	FALSE	PREDICTED: similar to Glutamate dehydrogenase CG5320-PF, isof	gALL v3 c4351 6	25 kDa		unknown	0.99733	0.62757	91%	89%
663	TRUE	FALSE	cytosol aminopeptidase [Danio rerio]	gALL v3 c4675 4	22 kDa		unknown	0,99733	0,62757	91%	89%
664	TRUE	FALSE	PREDICTED: similar to glucose dehydrogenase [Acyrthosiphon pisu	gALL v3 rep c99896 6 (+1)	19 kDa	TRUE	unknown	0.99733	0.62757	91%	89%
665	TRUE	FALSE	aliphatic nitrilase [Culex quinquefasciatus]	gALL v3 c11311 1	25 kDa		unknown	0	0,69313	0	90%
666	TRUE	FALSE	ribosomal protein L11 [Triatoma infestans]	gALL v3 c17302 1	21 kDa	TRUE	unknown	1.6054	1.0261	87%	92%
667	TRUE	FALSE	transport protein Sec61 alpha subunit [Bombyx mori]	gALL v3 c697 2	19 kDa		unknown	0,89028	0,69313	90%	89%
668	TRUE	FALSE	PREDICTED: similar to acetyl-coa carboxylase [Tribolium castaneu	gALL v3 c5988 5	62 kDa		unknown	1,371	0,68834	89%	90%
669	TRUE	FALSE	PREDICTED: similar to small GTP binding protein RAB5 [Tribolium	gALL v3 c4358 4	38 kDa		unknown	1,1388	1,0261	87%	92%
670	TRUE	FALSE	PREDICTED: similar to Inter-alpha-trypsin inhibitor heavy chain H4	gALL v3 rep c96455 2	89 kDa	TRUE	unknown	0	1,3207	0	89%
671	TRUE	FALSE	beta-glucosidase [Odontotermes formosanus]	gALL v3 c8970 4	71 kDa		unknown	1,371	0,62757	89%	89%
672	TRUE	FALSE	PREDICTED: similar to RE11240p [Nasonia vitripennis]	gALL v3 c861 2	58 kDa		unknown	0,99733	0,62757	88%	89%
673	TRUE	FALSE	suppressor of variegation 3-9 isoform 2 [Apis mellifera]	gALL v3 c956 5	26 kDa		unknown	0	1,0261	84%	92%
674	TRUE	FALSE	troponin I transcript variant C [Bombyx mandarina]	gALL v3 rep c98516 5	29 kDa	TRUE	unknown	0	3,6686	0	91%
675	TRUE	FALSE	PREDICTED: similar to endopeptidase inhibitor-like [Nasonia vitrip	gALL v3 c30394 1	34 kDa		unknown	1,944	0	94%	82%
676	TRUE	FALSE	mitochondrial processing peptidase beta subunit [Glossina morsita	gALL v3 c39627 6	19 kDa		unknown	0	1,574	0	89%
677	TRUE	FALSE	hypothetical protein TcasGA2 TC004676 [Tribolium castaneum]	gALL v3 c8835 5 (+1)	18 kDa		unknown	0	1.0261	84%	91%
678	TRUE	FALSE	1,2-dihydroxy-3-keto-5-methylthiopentene dioxygenase, putative	gALL v3 c6401 3	25 kDa		unknown	1,6054	0	93%	81%
679	TRUE	FALSE	GL22014 [Drosophila persimilis]	gALL v3 c6811 2	52 kDa		unknown	1.6054	0	92%	82%
680	TRUE	FALSE	heterogeneous nuclear ribonucleoprotein k, putative [Pediculus hu	gALL v3 rep c97157 5	57 kDa		unknown	1,6054	0	93%	80%
681	TRUE	FALSE	PREDICTED: similar to apolipophorin-III isoform 2 [Tribolium casta	gALL v3 rep c96133 3	32 kDa		unknown	0	1.3159	0	90%
682	TRUE	FALSE	hypothetical protein BRAFLDRAFT 129259 [Branchiostoma floridae	gALL v3 c59636 6	16 kDa		unknown	1,371	0	93%	0
683	TRUE	FALSE	14-3-3 protein [Trichomonas vaginalis G3]	gALL v3 c16482 6	24 kDa	TRUE	unknown	0	0.88083	0	91%
684	TRUE	FALSE	hypothetical protein BRAFLDRAFT 113552 [Branchiostoma floridae	gALL v3 c4666 6	24 kDa		unknown	1,1388	0	92%	78%
685	TRUE	FALSE	PREDICTED: similar to Proteasome 26S subunit subunit 4 ATPase (	gALL v3 c4627 3	44 kDa		unknown	0.99733	0	91%	79%
686	TRUE	FALSE	ubiquitin-conjugating enzyme E2L [Bombyx mori]	gALL v3 c17072 6	53 kDa		unknown	1,944	0	94%	76%
687	TRUE	FALSE	krueppel c2h2-type zinc finger protein, putative [Pediculus human	gALL v3 c27820 5 (+1)	25 kDa		unknown	1.944	0	87%	82%
688	TRUE	FALSE	lysosomal-associated transmembrane protein, putative [Pediculus	gALL v3 rep c96759 5 (+1)	30 kDa		unknown	1,1388	0	92%	0
689	TRUE	FALSE	hypothetical protein Phum PHUM335460 [Pediculus humanus corp	gALL v3 c8549 4 (+2)	42 kDa		unknown	0.89028	0.69313	91%	79%
690	TRUE	FALSE	GA20497 [Drosophila pseudoobscura pseudoobscura]	gALL v3 c58781 5	14 kDa		unknown	0,99733	0	91%	78%
691	TRUE	FALSE	GL10236 [Drosophila persimilis]	gALL v3 c3560 4	59 kDa	TRUE	unknown	0	0.69313	80%	89%
692	TRUE	FALSE	PREDICTED: similar to Ribosomal protein L23 CG3661-PA [Apis me	gALL v3 c8273 5	20 kDa		unknown	0	1,8851	74%	94%
693	TRUE	FALSE	PREDICTED: similar to Ribosomal protein S10b CG14206-PC, isofo	gALL v3 rep c96336 4	22 kDa		unknown	1.944	0	92%	76%
694	TRUE	FALSE	PREDICTED: similar to tripeptidylpeptidase II [Nasonia vitripennis]	gALL v3 c23298 4	22 kDa		unknown	0	0,68834	77%	90%

695	TRUE	FALSE	PREDICTED: similar to Short-chain specific acyl-CoA dehydrogenas	gALL v3 c975 3	47 kDa		unknown	0	1,574	0	91%
696	TRUE	FALSE	venom carbohydrate-rich protein precursor [Apis mellifera]	gALL v3 rep c96218 3	84 kDa		unknown	0.89028	0	91%	0
697	TRUE	FALSE	phosphoserine aminotransferase, putative [Pediculus humanus cor	gALL v3 c13666 1	21 kDa		unknown	0	1,7192	0	92%
698	TRUE	FALSE	PREDICTED: similar to restin (Reed-Steinberg cell-expressed interi	gALL v3 c45306 3	22 kDa		unknown	1.371	0	93%	73%
699	TRUE	FALSE	PREDICTED: similar to glycyl-tRNA synthetase [Nasonia vitripennis	gALL v3 c1519 4	65 kDa		unknown	0,99733	0	91%	74%
700	TRUE	FALSE	dehydrogenase [Trichomonas vaginalis G3]	gALL v3 rep c105517 6malate	?	TRUE	unknown	1.1388	0	92%	0
701	TRUE	FALSE	PREDICTED: similar to proteasome subunit alpha type [Nasonia vit	gALL v3 c12694 1	20 kDa		unknown	0	0,88083	81%	83%
702	TRUE	FALSE	conserved hypothetical protein [Pediculus humanus corporis]	gALL v3 c71001 3	16 kDa		unknown	0	1.0261	0	89%
703	TRUE	FALSE	PREDICTED: similar to Vanin-like protein 1 precursor, putative [Na	gALL v3 rep c96518 4	103 kDa		unknown	0	0,69313	74%	89%
704	TRUE	FALSE	serine/threonine-protein kinase MRCK beta, putative [Pediculus hu	gALL v3 c2503 3	146 kDa		unknown	1.6054	0	93%	70%
705	TRUE	FALSE	PREDICTED: similar to cathepsin F like protease [Nasonia vitripenr	gALL v3 rep c96890 2	44 kDa		unknown	0	1,0261	0	91%
706	TRUE	FALSE	Charged multivesicular body protein 4C, putative [Pediculus humai	gALL v3 c7755 5	24 kDa		unknown	0	0.69313	74%	89%
707	TRUE	FALSE	PREDICTED: similar to AGAP005894-PA [Tribolium castaneum]	gALL v3 c1559 6	16 kDa		unknown	0	0,69313	80%	83%
708	TRUE	FALSE	PREDICTED: similar to Ubiquitin activating enzyme 1 CG1782-PA is	gALL v3 c880 6	27 kDa		unknown	1.6054	0	93%	69%
709	TRUE	FALSE	PREDICTED: similar to aldehyde dehydrogenase 7 family, member	gALL v3 c15741 6	18 kDa		unknown	0	0,62757	73%	89%
710	TRUE	FALSE	[Culex quinquefasciatus]	gALL v3 rep c105816 6fixR	?	TRUE	unknown	4.5553	11.451	92%	70%
711	TRUE	FALSE	Ankyrin repeat domain protein [Wolbachia endosymbiont of Culex	gALL v3 c20115 2	18 kDa		unknown	0	1,0261	79%	82%
712	TRUE	FALSE	PREDICTED: zgc:112427 [Danio rerio]	gALL v3 c2878 2 (+1)	32 kDa		unknown	1.1388	0	92%	69%
713	TRUE	FALSE	PREDICTED: similar to Protein phosphatase 2B at 14D CG9842-PA	gALL v3 rep c97104 2	38 kDa		unknown	1,6054	0	93%	0
714	TRUE	FALSE	choline/ethanolamine kinase [Aedes aegypti]	gALL v3 c2014 6	41 kDa		unknown	0	1,8851	72%	89%
715	TRUE	FALSE	PREDICTED: similar to prophenoloxidase activating factor [Triboliu	gALL v3 rep c97352 4	36 kDa		unknown	0,99733	0	91%	0
716	TRUE	FALSE	hypothetical protein TcasGA2 TC004702 [Tribolium castaneum]	gALL v3 c20550 1	35 kDa		unknown	0	0,68834	67%	90%
717	TRUE	FALSE	PREDICTED: similar to conserved hypothetical protein [Nasonia vit	gALL v3 c23391 5	42 kDa		unknown	0	1,0261	65%	92%
718	TRUE	FALSE	hypothetical protein TcasGA2 TC010362 [Tribolium castaneum]	gALL v3 rep c96638 4	30 kDa		unknown	0	1,0261	0	92%
719	TRUE	FALSE	PREDICTED: similar to chaperonin subunit 6a zeta [Tribolium casta	gALL v3 c3604 4	16 kDa		unknown	0,99733	0	91%	65%
720	TRUE	FALSE	peroxiredoxin-like protein [Phlebotomus papatasi]	gALL v3 rep c99088 1	17 kDa	TRUE	unknown	1,944	0	94%	0
721	TRUE	FALSE	PREDICTED: similar to ENSANGP00000012858 [Nasonia vitripennis	gALL v3 c42215 1	24 kDa		unknown	1,6054	0	92%	0
722	TRUE	FALSE	PREDICTED: similar to CG9117 CG9117-PA [Acyrthosiphon pisum]	gALL v3 c12178 1 (+1)	34 kDa		unknown	0	0,88083	0	91%
723	TRUE	FALSE	syntaxin [Periplaneta americana]	gALL v3 c29845 4	19 kDa		unknown	1.371	0	91%	64%
724	TRUE	FALSE	PREDICTED: similar to AGAP007393-PB [Tribolium castaneum]	gALL v3 rep c96896 3	31 kDa		unknown	0	0,62757	64%	89%
725	TRUE	FALSE	PREDICTED: similar to PNUTS CG33526-PD, isoform D [Apis mellif	gALL v3 c4246 1	57 kDa		unknown	1.1388	0	92%	0
726	TRUE	FALSE	glucose-6-phosphate isomerase [Spodoptera exigua]	gALL v3 rep c98580 6 (+2)	16 kDa		unknown	0	0,62757	62%	89%
727	TRUE	FALSE	PREDICTED: similar to BMKETTIN [Tribolium castaneum]	gALL v3 rep c96338 4	198 kDa		unknown	0	0.68834	61%	90%
728	TRUE	FALSE	PREDICTED: similar to AGAP010130-PA [Acyrthosiphon pisum]	gALL v3 c69987 4	33 kDa		unknown	0,89028	0	91%	59%
729	TRUE	FALSE	PREDICTED: similar to CG9674 CG9674-PA [Acyrthosiphon pisum]	gALL v3 c7596 4	73 kDa		unknown	0	0.68834	60%	90%
730	TRUE	FALSE	PREDICTED: similar to amyloid protein precursor-like CG7727-PA i	gALL v3 rep c96497 5 (+2)	163 kDa		unknown	0	0,68834	60%	90%
731	TRUE	FALSE	transcriptase [Bombyx mori]	gALL v3 rep c102694 2endonuc	?		unknown	1.371	0	93%	56%
732	TRUE	FALSE	PREDICTED: similar to dihydropyrimidine dehydrogenase isoform 1	gALL v3 c7019 2	43 kDa		unknown	0	0,68834	59%	90%
733	TRUE	FALSE	PREDICTED: similar to DNA-damage inducible protein [Tribolium ca	gALL v3 c14426 1	44 kDa		unknown	0.99733	0	91%	0
734	TRUE	FALSE	putative CG5976 [Heliconius melpomene]	gALL v3 c7425 3	29 kDa		unknown	0	0,69313	60%	89%
735	TRUE	FALSE	PREDICTED: similar to conserved hypothetical protein [Nasonia vit	gALL v3 c13294 2	27 kDa		unknown	0.89028	0	91%	58%
736	TRUE	FALSE	GK15132 [Drosophila willistoni]	gALL v3 c13831 6	31 kDa	TRUE	unknown	1,371	1,3767	58%	90%
737	TRUE	FALSE	cytosolic heat shock protein 70 [Trichomonas vaginalis G3]	gALL v3 c429 3	30 kDa		unknown	0	0.62757	59%	89%
738	TRUE	FALSE	PREDICTED: similar to CG1458-PA [Apis mellifera]	gALL v3 c22712 2	13 kDa		unknown	1,944	0	86%	60%
739	TRUE	FALSE	GH24027 [Drosophila grimshawi]	gALL v3 rep c96626 4	60 kDa		unknown	0	0.69313	55%	89%
740	TRUE	FALSE	aldehyde dehydrogenase [Aedes aegypti]	gALL v3 c13940 2	32 kDa	TRUE	unknown	0	3,1379	55%	89%
741	TRUE	FALSE	cathepsin B [Sus scrofa]	gALL v3 rep c96885 6	43 kDa		unknown	0	0,88083	52%	91%
742	TRUE	FALSE	hypothetical protein TcasGA2 TC004723 [Tribolium castaneum]	gALL v3 c5716 1	66 kDa		unknown	0,89028	0	91%	0
743	TRUE	FALSE	hypothetical protein TcasGA2 TC006964 [Tribolium castaneum]	gALL v3 c76644 6	33 kDa		unknown	1,1388	0	92%	51%
744	TRUE	FALSE	cytosolic heat shock protein 70 [Trichomonas vaginalis G3]	gALL v3 c58057 2	14 kDa		unknown	0,99733	0	91%	0
745	TRUE	FALSE	hypothetical protein TcasGA2 TC005989 [Tribolium castaneum]	gALL v3 c29708 2	19 kDa		unknown	1,371	0	86%	57%
746	TRUE	FALSE	PREDICTED: hypothetical protein LOC100439748 [Pongo abelii]	gALL v3 c24273 5 (+3)	15 kDa		unknown	0	1,8851	52%	89%
747	TRUE	FALSE	hypothetical protein BACOVA 00321 [Bacteroides ovatus ATCC 848	gALL v3 rep c96418 6	84 kDa		unknown	0	0,69313	59%	82%
748	TRUE	FALSE	Chain A, Crystal Structure Of A Designed Selected Ankyrin Repeat	gALL v3 c3597 4	29 kDa		unknown	0	0,62757	0	83%
749	TRUE	FALSE	alkaline phosphatase [Culex quinquefasciatus]	gALL v3 c3203 1	24 kDa		unknown	0	0,62757	51%	89%
750	TRUE	FALSE	transposase [Portunus pelagicus]	gALL v3 c18973 1 (+1)	37 kDa		unknown	0	1.0261	50%	90%
751	TRUE	FALSE	PREDICTED: similar to AGAP007642-PA [Acyrthosiphon pisum]	gALL v3 c44107 5	21 kDa		unknown	0	0,62757	0	89%
752	TRUE	FALSE	ribosomal protein L7A [Chrysomela tremulae]	gALL v3 rep c96634 1 (+1)	32 kDa		unknown	1.1388	0	92%	47%
753	TRUE	FALSE	PREDICTED: similar to AGAP001049-PA, partial [Hydra magnipapil	gALL v3 c32273 3	20 kDa		unknown	1,1388	0	92%	0
754	TRUE	FALSE	PREDICTED: similar to conserved hypothetical protein [Nasonia vit	gALL v3 c40690 2	16 kDa		unknown	0	0.88083	48%	91%
755	TRUE	FALSE	PREDICTED: similar to NADH-ubiquinone oxidoreductase 75 kDa su	gALL v3 c201 5	62 kDa		unknown	0	0,88083	47%	91%
756	TRUE	FALSE	predicted protein [Pediculus humanus corporis]	gALL v3 c38228 3	14 kDa		unknown	0	0.69313	0	89%
757	TRUE	FALSE	protein COLAER 01671 [Collinsella aerofaciens ATCC 25986]	gALL v3 rep c101055 5Hypothet	?		unknown	0	0,88083	55%	82%
758	TRUE	FALSE	hypothetical protein TcasGA2 TC001128 [Tribolium castaneum]	gALL v3 c4784 4	25 kDa		unknown	0	0.68834	55%	82%
759	TRUE	FALSE	heat shock protein [Trichomonas vaginalis G3]	gALL v3 c15866 6	20 kDa		unknown	0	1,8851	0	94%
760	TRUE	FALSE	hypothetical protein IscW ISCW011622 [Ixodes scapularis]	gALL v3 c65444 3	14 kDa		unknown	0	0.88083	0	91%
761	TRUE	FALSE	RecName: Full=Endocuticle structural glycoprotein ABD-5	gALL v3 rep c96578 1 (+1)	20 kDa		unknown	1,944	0	90%	44%
762	TRUE	FALSE	PREDICTED: similar to heat shock protein 70 B2 [Tribolium castan	gALL v3 c22184 2	28 kDa		unknown	0.99733	0	91%	43%
763	TRUE	FALSE	PREDICTED: similar to ADP ribosylation factor 79F CG8385-PF [Tri	gALL v3 c9788 4	14 kDa	TRUE	unknown	3,888	1,0261	94%	39%
764	TRUE	FALSE	PREDICTED: similar to hydroxypyruvate isomerase [Nasonia vitripe	gALL v3 c38426 5	17 kDa		unknown	1.1388	0	92%	38%
765	TRUE	FALSE	PREDICTED: similar to Choroideremia [Acyrthosiphon pisum]	gALL v3 c6368 6	77 kDa		unknown	0	0,62757	40%	89%
766	TRUE	FALSE	PREDICTED: similar to ENSANGP00000022464 [Nasonia vitripennis	gALL v3 c1039 2	30 kDa		unknown	0	0.88083	37%	91%
767	TRUE	FALSE	NADPH--cytochrome P450, putative [Pediculus humanus corporis]	gALL v3 c17236 6	36 kDa		unknown	0	0,62757	39%	89%
768	TRUE	FALSE	hypothetical protein TcasGA2 TC008620 [Tribolium castaneum]	gALL v3 rep c96405 1	219 kDa		unknown	1,371	0	83%	0
769	TRUE	FALSE	putative glycosyl hydrolase family7 [uncultured symbiotic protist o	gALL v3 c12313 5 (+1)	31 kDa		unknown	0,89028	0	85%	42%
770	TRUE	FALSE	caspase-1-like protein [Heliconius melpomene]	gALL v3 c8371 6	44 kDa		unknown	1,944	0	87%	39%
771	TRUE	FALSE	serine/threonine-protein phosphatase PP-V, putative [Pediculus hu	gALL v3 c6610 4	52 kDa		unknown	0	0,69313	36%	89%
772	TRUE	FALSE	conserved hypothetical protein [Culex quinquefasciatus]	gALL v3 c43827 2	22 kDa		unknown	0	0,69313	35%	89%
773	TRUE	FALSE	PREDICTED: similar to aldo-keto reductase isoform 1 [Tribolium ca	gALL v3 c15066 3	30 kDa	TRUE	unknown	0	2,0794	0	100%
774	TRUE	FALSE	fatty acid synthase, putative [Pediculus humanus corporis]	gALL v3 c15968 5	19 kDa		unknown	0	1,3767	0	100%
775	TRUE	FALSE	coronin [Trichomonas vaginalis G3]	gALL v3 c1624 5	42 kDa		unknown	0	1,2551	0	100%
776	TRUE	FALSE	PREDICTED: similar to isocitrate dehydrogenase [Tribolium castan	gALL v3 c1652 4	17 kDa		unknown	0	1,3863	0	100%
777	TRUE	FALSE	PREDICTED: similar to aldehyde dehydrogenase 7 family, member	gALL v3 c1715 3	46 kDa		unknown	0	1.2551	0	100%
778	TRUE	FALSE	lysosomal acid lipase, putative [Aedes aegypti]	gALL v3 c205 3	58 kDa		unknown	0	1,3863	0	100%
779	TRUE	FALSE	RecName: Full=Annulin; AltName: Full=Protein-glutamine gamma	gALL v3 c21861 5 (+1)	18 kDa		unknown	0	0.68834	0	100%
780	TRUE	FALSE	PREDICTED: similar to ENSANGP00000014908 [Nasonia vitripennis	gALL v3 c21941 1	24 kDa		unknown	0	1,2551	0	100%
781	TRUE	FALSE	beta-ureidopropionase, putative [Pediculus humanus corporis]	gALL v3 c23559 2	20 kDa	TRUE	unknown	0	3.4656	0	100%
782	TRUE	FALSE	ribosomal protein rps2 [Lineus viridis]	gALL v3 c2578 1	15 kDa		unknown	2,2777	0	100%	0
783	TRUE	FALSE	AGAP007975-PA [Anopheles gambiae str. PEST]	gALL v3 c2582 4	30 kDa		unknown	0	1.7617	0	100%
784	TRUE	FALSE	GH12513 [Drosophila grimshawi]	gALL v3 c2629 4	51 kDa		unknown	0	1,3863	0	100%
785	TRUE	FALSE	glutaryl-CoA dehydrogenase [Xenopus laevis]	gALL v3 c2707 3	37 kDa		unknown	0	1.3863	0	100%
786	TRUE	FALSE	PREDICTED: similar to annulin [Tribolium castaneum]	gALL v3 c3181 5	61 kDa		unknown	0	1,3767	0	100%
787	TRUE	FALSE	hypothetical protein TcasGA2 TC010876 [Tribolium castaneum]	gALL v3 c3283 5	33 kDa		unknown	1.9947	0	100%	0
788	TRUE	FALSE	EF hand family protein [Trichomonas vaginalis G3]	gALL v3 c33550 6	28 kDa		unknown	0	2,0522	0	100%
789	TRUE	FALSE	signal recognition particle [Triatoma infestans]	gALL v3 c3603 5	17 kDa		unknown	3.888	0	100%	0
790	TRUE	FALSE	PREDICTED: similar to acetoacetyl-CoA thiolase [Nasonia vitripenn	gALL v3 c3788 5	39 kDa		unknown	0	1,3863	0	100%
791	TRUE	FALSE	phosphoenolpyruvate carboxykinase [Calosoma scrutator]	gALL v3 c3888 4	26 kDa		unknown	0	1.2551	0	100%
792	TRUE	FALSE	PREDICTED: similar to ubiquitin specific peptidase 14 [Tribolium ca	gALL v3 c3908 4	36 kDa		unknown	1,9947	0	100%	0
793	TRUE	FALSE	GF11978 [Drosophila ananassae]	gALL v3 c3925 1	32 kDa		unknown	0	1.3863	0	100%
794	TRUE	FALSE	conserved hypothetical protein [Pediculus humanus corporis]	gALL v3 c607 3	164 kDa		unknown	0	1,3863	0	100%
795	TRUE	FALSE	PREDICTED: similar to Neuroglian CG1634-PA, isoform A [Apis me	gALL v3 c7281 4	62 kDa		unknown	2,742	0	100%	0
796	TRUE	FALSE	hypothetical protein TcasGA2 TC008405 [Tribolium castaneum]	gALL v3 c80443 1 (+1)	14 kDa		unknown	0	1,2551	0	100%
797	TRUE	FALSE	calpain M [Nephrops norvegicus]	gALL v3 c8594 2	25 kDa		unknown	0	1,3863	0	100%
798	TRUE	FALSE	PREDICTED: similar to juvenile hormone esterase [Nasonia vitriper	gALL v3 c9362 2	41 kDa		unknown	0	1,2551	0	100%
799	TRUE	FALSE	PREDICTED: similar to Cuticular protein 57A CG18066-PA [Triboliu	gALL v3 c9681 3	41 kDa		unknown	3,888	0	100%	0
800	TRUE	FALSE	PREDICTED: similar to S9e ribosomal protein isoform 1 [Tribolium	gALL v3 rep c96298 6	28 kDa		unknown	3,2108	0	100%	0
801	TRUE	FALSE	Parcxpwnx01 [Periplaneta americana]	gALL v3 rep c96347 6	36 kDa	TRUE	unknown	0	2,6425	0	100%
802	TRUE	FALSE	troponin I transcript variant A [Bombyx mandarina]	gALL v3 rep c96582 2	42 kDa	TRUE	unknown	0	3,5233	0	100%
803	TRUE	FALSE	Parcxpwnx03 [Periplaneta americana]	gALL v3 rep c96805 5	18 kDa		unknown	3,888	0	100%	0
804	TRUE	FALSE	PREDICTED: acetyl-Coenzyme A acyltransferase 2-like [Saccogloss	gALL v3 rep c97972 6	19 kDa	TRUE	unknown	1.7806	0	100%	0
805	TRUE	FALSE	EF hand family protein [Trichomonas vaginalis G3]	gALL v3 c15336 4	21 kDa		unknown	0	2.0522	0	100%
806	TRUE	FALSE	PREDICTED: similar to vacuolar ATP synthase subunit h isoform 1	gALL v3 c11328 2	24 kDa		unknown	0.99733	0	100%	0
807	TRUE	FALSE	PREDICTED: similar to F-actin capping protein subunit alpha [Apis	gALL v3 c13687 1	22 kDa		unknown	0	1,7617	0	100%
808	TRUE	FALSE	rab6 [Aedes aegypti]	gALL v3 c3098 1	27 kDa		unknown	4.8161	0	100%	0
809	TRUE	FALSE	GK17957 [Drosophila willistoni]	gALL v3 c75585 3	14 kDa		unknown	0	1,2551	0	100%
810	TRUE	FALSE	proteasome beta2 subunit, putative [Ixodes scapularis]	gALL v3 c34825 1 (+2)	14 kDa		unknown	3.2108	0	100%	0
811	TRUE	FALSE	serine carboxypeptidase SCP-1 [Triatoma brasiliensis]	gALL v3 c3968 1	48 kDa		unknown	0	1,2551	0	100%
812	TRUE	FALSE	cytoplasmic dynein light chain [Aedes aegypti]	gALL v3 c7482 3	32 kDa		unknown	0	1.8851	0	100%
813	TRUE	FALSE	PREDICTED: similar to reticulocalbin [Tribolium castaneum]	gALL v3 c7725 5 (+1)	18 kDa		unknown	0,89028	0	100%	0
814	TRUE	FALSE	Spectrin alpha chain, putative [Pediculus humanus corporis]	gALL v3 c7380 5	33 kDa	TRUE	unknown	0	1.3767	0	100%
815	TRUE	FALSE	AGAP003652-PA [Anopheles gambiae str. PEST]	gALL v3 c3518 6	63 kDa		unknown	0	0,62757	0	100%
816	TRUE	FALSE	PREDICTED: similar to pyruvate dehydrogenase [Tribolium castane	gALL v3 c14417 5	41 kDa		unknown	1.7806	0	100%	0
817	TRUE	FALSE	cytochrome P450 [Hodotermopsis sjoestedti]	gALL v3 c46657 4 (+3)	59 kDa		unknown	0	0,62757	0	100%
818	TRUE	FALSE	AGAP004890-PB [Anopheles gambiae str. PEST]	gALL v3 c1451 6	39 kDa		unknown	0	0.69313	0	99%
819	TRUE	FALSE	hypothetical protein TcasGA2 TC012309 [Tribolium castaneum]	gALL v3 c3653 2	52 kDa		unknown	0	0,68834	0	99%
820	TRUE	FALSE	Obg-like ATPase 1 [Acyrthosiphon pisum]	gALL v3 c2860 5	68 kDa		unknown	0	0.69313	0	99%
821	TRUE	FALSE	AGAP006670-PA [Anopheles gambiae str. PEST]	gALL v3 c5046 3	37 kDa		unknown	0,89028	0	99%	0
822	TRUE	FALSE	GA10495 [Drosophila pseudoobscura pseudoobscura]	gALL v3 c3327 5	72 kDa		unknown	0,99733	0	99%	0
823	TRUE	FALSE	PREDICTED: similar to glycinamide ribonucleotide synthetase-amir	gALL v3 c1214 2	95 kDa		unknown	0	0,68834	0	99%
824	TRUE	FALSE	glyceraldehyde-3-phosphate dehydrogenase [Pseudotrichonympha	gALL v3 rep c98248 5	17 kDa		unknown	0	1,0261	0	99%
825	TRUE	FALSE	Peritrophin-1 precursor, putative [Pediculus humanus corporis]	gALL v3 rep c98303 6	21 kDa		unknown	1,1388	0	99%	0
826	TRUE	FALSE	PREDICTED: similar to short-chain dehydrogenase [Nasonia vitripe	gALL v3 c13917 3	29 kDa		unknown	0	0,69313	0	99%
827	TRUE	FALSE	PREDICTED: similar to lysosomal alpha-mannosidase (mannosidas	gALL v3 c698 6	66 kDa		unknown	0	0,62757	0	99%
828	TRUE	FALSE	Phosphoglycerate kinase [Trichomonas vaginalis G3]	gALL v3 c6557 2	39 kDa		unknown	0	0,69313	0	99%
829	TRUE	FALSE	PREDICTED: similar to thioredoxin-like protein [Tribolium castaneu	gALL v3 c2384 6	46 kDa		unknown	1,1388	0	99%	0
830	TRUE	FALSE	p270 [Bombyx mori]	gALL v3 c17129 4	28 kDa		unknown	1,371	0	99%	0
831	TRUE	FALSE	hypothetical protein TcasGA2 TC014041 [Tribolium castaneum]	gALL v3 rep c96446 5 (+1)	44 kDa		unknown	1.944	0	99%	0
832	TRUE	FALSE	arylalkylamine N-acetyltransferase [Periplaneta americana]	gALL v3 c8365 6	25 kDa		unknown	0	0,88083	0	99%
833	TRUE	FALSE	enolase [Pseudotrichonympha grassii]	gALL v3 c12819 4	30 kDa		unknown	0	1.2551	0	99%
834	TRUE	FALSE	PREDICTED: similar to CG3731-PB, isoform B [Apis mellifera]	gALL v3 c20252 4 (+2)	24 kDa		unknown	0,89028	0	99%	0
835	TRUE	FALSE	PREDICTED: similar to AGAP005175-PA [Acyrthosiphon pisum]	gALL v3 c60316 3	17 kDa		unknown	0	0.68834	0	99%
836	TRUE	FALSE	PREDICTED: similar to aldehyde oxidase [Nasonia vitripennis]	gALL v3 c2305 4	44 kDa	TRUE	unknown	2,742	0	99%	0
837	TRUE	FALSE	PREDICTED: similar to CG6525-PA [Apis mellifera]	gALL v3 c63612 4	26 kDa		unknown	0	1.0261	0	99%
838	TRUE	FALSE	GG23881 [Drosophila erecta]	gALL v3 c30948 4	19 kDa	TRUE	unknown	0	0,88083	0	98%
839	TRUE	FALSE	PREDICTED: similar to 24-dehydrocholesterol reductase, partial [S	gALL v3 rep c96473 3 (+1)	35 kDa		unknown	0	0.62757	0	98%
840	TRUE	FALSE	putative glycosyl hydrolase family45 [uncultured symbiotic protist	gALL v3 c17434 3	20 kDa		unknown	1,6054	0	98%	0
841	TRUE	FALSE	PREDICTED: similar to ENSANGP00000031876 [Nasonia vitripennis	gALL v3 rep c96560 4 (+1)	26 kDa		unknown	0	1.8851	0	98%
842	TRUE	FALSE	PREDICTED: similar to fumarylacetoacetate hydrolase domain cont	gALL v3 c26728 2	19 kDa		unknown	0	0,88083	0	98%
843	TRUE	FALSE	L-allo-threonine aldolase, putative [Pediculus humanus corporis]	gALL v3 c15633 6	37 kDa		unknown	0	0.69313	0	98%
844	TRUE	FALSE	PREDICTED: similar to Phosphogluconate mutase CG5165-PA [Apis	gALL v3 c1965 1	82 kDa		unknown	0,99733	0	98%	0
845	TRUE	FALSE	hypothetical protein BRAFLDRAFT 287019 [Branchiostoma floridae	gALL v3 rep c97672 1	26 kDa		unknown	0	0.69313	0	98%
846	TRUE	FALSE	hypothetical protein TcasGA2 TC005186 [Tribolium castaneum]	gALL v3 c2918 5	44 kDa		unknown	0	0,68834	0	98%
847	TRUE	FALSE	PREDICTED: similar to Putative actin-interacting protein 1 (AIP1) [	gALL v3 c5727 5	17 kDa		unknown	0.99733	0	98%	0
848	TRUE	FALSE	PREDICTED: similar to Ribosomal protein L3 CG4863-PA, isoform A	gALL v3 rep c96219 2	47 kDa		unknown	0,89028	0	98%	0
849	TRUE	FALSE	RecName: Full=40S ribosomal protein S3a	gALL v3 c46488 1	18 kDa		unknown	1,1388	0	98%	0
850	TRUE	FALSE	membrane alanyl aminopeptidase [Tenebrio molitor]	gALL v3 rep c96879 6	40 kDa		unknown	0	0,68834	0	98%
851	TRUE	FALSE	putative accessory gland protein [Gryllus pennsylvanicus]	gALL v3 c12215 5	50 kDa		unknown	0	0,62757	0	98%
852	TRUE	FALSE	PREDICTED: similar to heat shock protein 70 B2 [Tribolium castan	gALL v3 c22184 1	29 kDa		unknown	0	0,62757	0	98%
853	TRUE	FALSE	Thioredoxin domain-containing protein 17 [Salmo salar]	gALL v3 c31534 2	28 kDa		unknown	1,944	0	98%	0
854	TRUE	FALSE	PREDICTED: similar to AGAP001449-PA [Tribolium castaneum]	gALL v3 c35813 4	23 kDa		unknown	1,1388	0	98%	0
855	TRUE	FALSE	mRNA cap-binding protein eIF4E [Spodoptera frugiperda]	gALL v3 c19504 4	45 kDa		unknown	1,6054	0	93%	0
856	TRUE	FALSE	PREDICTED: similar to cellular repressor of E1A-stimulated genes	gALL v3 c30884 1	19 kDa	TRUE	unknown	3,2108	0	93%	0
857	TRUE	FALSE	ribosomal protein L11 [Triatoma infestans]	gALL v3 c49122 4	19 kDa	TRUE	unknown	3,2108	0	93%	0
858	TRUE	FALSE	thaumatin-like protein 2 [Schistocerca gregaria]	gALL v3 rep c96258 5	35 kDa		unknown	1.6054	0	93%	0
859	TRUE	FALSE	GD14343 [Drosophila simulans]	gALL v3 c28442 3 (+1)	16 kDa		unknown	2,742	0	93%	0
860	TRUE	FALSE	conserved hypothetical protein [Culex quinquefasciatus]	gALL v3 rep c97424 5	38 kDa		unknown	0	1.0261	0	92%
861	TRUE	FALSE	hypothetical protein TcasGA2 TC000722 [Tribolium castaneum]	gALL v3 c47422 5	23 kDa		unknown	1,1388	0	92%	0
862	TRUE	FALSE	PREDICTED: similar to Dihydropteridine reductase CG4665-PA, iso	gALL v3 c13027 2	18 kDa	TRUE	unknown	0	2.6425	0	91%
863	TRUE	FALSE	PREDICTED: similar to apolipophorin [Nasonia vitripennis]	gALL v3 rep c97643 6	37 kDa	FALSE	unknown	0	26,157	0	90%
864	TRUE	FALSE	protein transport protein sec13, putative [Pediculus humanus corp	gALL v3 c12262 6	37 kDa		unknown	0	1.3863	0	89%
865	TRUE	FALSE	PREDICTED: similar to leukotriene a-4 hydrolase [Tribolium castan	gALL v3 c1206 5	38 kDa		unknown	0	1,2551	0	89%
866	TRUE	FALSE	disulfide-isomerase tigA [Culex quinquefasciatus]	gALL v3 c46013 6	18 kDa		unknown	0	0.69313	0	88%
867	TRUE	FALSE	PREDICTED: similar to ENSANGP00000011337 [Nasonia vitripennis	gALL v3 c7405 2	46 kDa		unknown	0,89028	0	87%	0
868	TRUE	FALSE	PREDICTED: similar to fucosidase, alpha-L- 2, plasma [Apis mellife	gALL v3 c472 5	50 kDa		unknown	0.89028	0	86%	0
869	TRUE	FALSE	PREDICTED: similar to RH03087p [Nasonia vitripennis]	gALL v3 c1090 2	17 kDa		unknown	0	1,8851	0	94%
870	TRUE	FALSE	PREDICTED: similar to 6-pyruvoyltetrahydropterin synthase, putat	gALL v3 c15291 5	43 kDa		unknown	0	1,8851	0	94%
871	TRUE	FALSE	PREDICTED: similar to conserved hypothetical protein [Tribolium c	gALL v3 c16066 5	25 kDa		unknown	0	1,8851	0	94%
872	TRUE	FALSE	mitochondrial import inner membrane translocase subunit TIM13,	gALL v3 c1721 1	47 kDa		unknown	0	1,8851	0	94%
873	TRUE	FALSE	PREDICTED: similar to sorting nexin isoform 1 [Tribolium castaneu	gALL v3 c1940 3	52 kDa		unknown	0	1,8851	0	94%
874	TRUE	FALSE	PREDICTED: similar to GA19800-PA [Tribolium castaneum]	gALL v3 c199 1 (+1)	80 kDa		unknown	0	1.8851	0	94%
875	TRUE	FALSE	hypothetical protein AaeL AAEL009032 [Aedes aegypti]	gALL v3 c20157 4	23 kDa		unknown	0	1,8851	0	94%

876	TRUE	FALSE	N(4)-(Beta-N-acetylglucosaminyl)-L-asparaginase [Culex quinquef	gALL v3 c24279 5	28 kDa		unknown	0	1,8851	0	94%
877	TRUE	FALSE	PREDICTED: similar to Cytochrome c oxidase copper chaperone [T	gALL v3 c25962 2	14 kDa		unknown	0	1,8851	0	94%
878	TRUE	FALSE	Cofilin/tropomyosin-type actin-binding protein [Trichomonas vagin	gALL v3 c32504 6	14 kDa		unknown	0	1,8851	0	94%
879	TRUE	FALSE	roadblock [Nasonia vitripennis]	gALL v3 c3673 4	30 kDa		unknown	0	1,8851	0	94%
880	TRUE	FALSE	peroxiredoxin-like protein [Phlebotomus papatasi]	gALL v3 c51880 2	17 kDa	TRUE	unknown	0	1,8851	0	94%
881	TRUE	FALSE	ATPase coupling factor 6 [Simulium nigrimanum]	gALL v3 c54633 4	17 kDa		unknown	0	1,8851	0	94%
882	TRUE	FALSE	profilin [Trichomonas vaginalis G3]	gALL v3 c56857 4	12 kDa		unknown	0	1,8851	0	94%
883	TRUE	FALSE	PREDICTED: similar to snare protein sec22 [Tribolium castaneum]	gALL v3 c6106 6	24 kDa		unknown	0	1,8851	0	94%
884	TRUE	FALSE	similar to piggyBac transposase Uribo1 [Hydra magnipapillata]	gALL v3 rep c105327 4PREDICT	?		unknown	0	1,8851	0	94%
885	TRUE	FALSE	PREDICTED: similar to AGAP001353-PA [Tribolium castaneum]	gALL v3 rep c96189 3	38 kDa	TRUE	unknown	0	1,8851	0	94%
886	TRUE	FALSE	antioxidant enzyme [Culex quinquefasciatus]	gALL v3 rep c96493 1 (+1)	62 kDa		unknown	0	1,8851	0	94%
887	TRUE	FALSE	PREDICTED: similar to karst CG12008-PA, isoform A [Apis mellifer	gALL v3 rep c97110 6	29 kDa		unknown	0	1,8851	0	94%
888	TRUE	FALSE	kinesin-like protein KIF21A isoform 1 [Mus musculus]	gALL v3 c10372 6	46 kDa		unknown	1,944	0	94%	0
889	TRUE	FALSE	PREDICTED: similar to cuticular protein [Tribolium castaneum]	gALL v3 c10506 6 (+3)	20 kDa		unknown	1,944	0	94%	0
890	TRUE	FALSE	PREDICTED: similar to prefoldin subunit 5 [Tribolium castaneum]	gALL v3 c1224 3	41 kDa		unknown	1,944	0	94%	0
891	TRUE	FALSE	hypothetical protein TcasGA2 TC002488 [Tribolium castaneum]	gALL v3 c12268 4 (+1)	22 kDa		unknown	1,944	0	94%	0
892	TRUE	FALSE	GF10252 [Drosophila ananassae]	gALL v3 c13398 1	35 kDa		unknown	1,944	0	94%	0
893	TRUE	FALSE	PREDICTED: similar to CG3777-PB, isoform B [Apis mellifera]	gALL v3 c16357 6	27 kDa		unknown	1,944	0	94%	0
894	TRUE	FALSE	heat shock factor binding protein, putative [Aedes aegypti]	gALL v3 c21781 4	18 kDa		unknown	1,944	0	94%	0
895	TRUE	FALSE	ribosomal protein L36e [Blattella germanica]	gALL v3 c3198 4 (+1)	65 kDa		unknown	1,944	0	94%	0
896	TRUE	FALSE	hypothetical protein BRAFLDRAFT 127172 [Branchiostoma floridae	gALL v3 c32864 5	10 kDa		unknown	1,944	0	94%	0
897	TRUE	FALSE	conserved hypothetical protein [Ixodes scapularis]	gALL v3 c36918 2 (+1)	27 kDa		unknown	1,944	0	94%	0
898	TRUE	FALSE	allergen Bla g 6.0301 [Blattella germanica]	gALL v3 c43181 2 (+2)	16 kDa	TRUE	unknown	1,944	0	94%	0
899	TRUE	FALSE	Profilin A [Trichomonas vaginalis G3]	gALL v3 c87182 4	14 kDa		unknown	1,944	0	94%	0
900	TRUE	FALSE	protein BRAFLDRAFT 287383 [Branchiostoma floridae]	gALL v3 rep c104529 3hypothet	?		unknown	1,944	0	94%	0
901	TRUE	FALSE	PREDICTED: similar to fau CG6544-PC, isoform C isoform 2 [Apis r	gALL v3 rep c96200 5	77 kDa		unknown	1,944	0	94%	0
902	TRUE	FALSE	allergen Bla g 6.0101 [Blattella germanica]	gALL v3 rep c96301 4	29 kDa	TRUE	unknown	1,944	0	94%	0
903	TRUE	FALSE	putative ribosomal protein S23e [Graphocephala atropunctata]	gALL v3 rep c96430 4	22 kDa		unknown	1,944	0	94%	0
904	TRUE	FALSE	PREDICTED: similar to cytochrome B5 [Tribolium castaneum]	gALL v3 rep c96543 5 (+1)	41 kDa		unknown	1,944	0	94%	0
905	TRUE	FALSE	PREDICTED: similar to CG33229-PA [Apis mellifera]	gALL v3 rep c96708 5	54 kDa		unknown	1,944	0	94%	0
906	TRUE	FALSE	predicted protein [Populus trichocarpa]	gALL v3 rep c96845 4 (+1)	70 kDa		unknown	1,944	0	94%	0
907	TRUE	FALSE	PREDICTED: similar to ras [Nasonia vitripennis]	gALL v3 c13611 6	16 kDa		unknown	1,6054	0	93%	0
908	TRUE	FALSE	PREDICTED: similar to CG18815-PA [Nasonia vitripennis]	gALL v3 c21716 5	63 kDa		unknown	1,6054	0	93%	0
909	TRUE	FALSE	PREDICTED: similar to Nck-associated protein 5 (NAP-5) (Peripher	gALL v3 c22285 6	17 kDa		unknown	1,6054	0	93%	0
910	TRUE	FALSE	PREDICTED: FGFR1 oncogene partner 2-like [Saccoglossus kowale	gALL v3 c27623 5	21 kDa		unknown	1,6054	0	93%	0
911	TRUE	FALSE	PREDICTED: similar to conserved hypothetical protein [Nasonia vit	gALL v3 c27944 5	34 kDa		unknown	1,6054	0	93%	0
912	TRUE	FALSE	ribosomal protein L28 [Chrysomela tremulae]	gALL v3 c297 5	20 kDa		unknown	1,6054	0	93%	0
913	TRUE	FALSE	RNA-binding protein 8A, putative [Pediculus humanus corporis]	gALL v3 c32315 4 (+1)	18 kDa		unknown	1,6054	0	93%	0
914	TRUE	FALSE	PREDICTED: similar to proteasome subunit beta type 5,8 [Triboliur	gALL v3 c32542 5	23 kDa	TRUE	unknown	3,2108	0	93%	0
915	TRUE	FALSE	PREDICTED: similar to J domain containing protein [Tribolium cast	gALL v3 c32785 2 (+1)	30 kDa		unknown	1,6054	0	93%	0
916	TRUE	FALSE	PREDICTED: similar to proteasome subunit beta 7 [Nasonia vitripe	gALL v3 c49671 2	18 kDa		unknown	1,6054	0	93%	0
917	TRUE	FALSE	mariner transposase [Chrysoperla plorabunda]	gALL v3 c5313 5	40 kDa		unknown	1,6054	0	93%	0
918	TRUE	FALSE	superoxide dismutase 1 [Tritrichomonas foetus]	gALL v3 c59228 1	17 kDa		unknown	1,6054	0	93%	0
919	TRUE	FALSE	PREDICTED: similar to calcyphosine-like [Apis mellifera]	gALL v3 c59585 5	17 kDa		unknown	1,6054	0	93%	0
920	TRUE	FALSE	GG15121 [Drosophila erecta]	gALL v3 c67527 6	21 kDa		unknown	1,6054	0	93%	0
921	TRUE	FALSE	PREDICTED: similar to Farsla protein [Strongylocentrotus purpurat	gALL v3 c87050 3	6 kDa		unknown	1,6054	0	93%	0
922	TRUE	FALSE	AGAP001354-PA [Anopheles gambiae str. PEST]	gALL v3 rep c96240 1 (+1)	32 kDa		unknown	1,6054	0	93%	0
923	TRUE	FALSE	PREDICTED: similar to Muscle-specific protein 300 CG33715-PD [T	gALL v3 rep c96371 5 (+1)	635 kDa		unknown	1,6054	0	93%	0
924	TRUE	FALSE	PREDICTED: similar to Rtnl1 CG33113-PB, isoform B [Apis mellifer	gALL v3 rep c96520 1	79 kDa		unknown	1,6054	0	93%	0
925	TRUE	FALSE	lectin-related protein [Periplaneta americana]	gALL v3 rep c97015 3	26 kDa		unknown	1,6054	0	93%	0
926	TRUE	FALSE	thaumatin-like protein 2 [Schistocerca gregaria]	gALL v3 rep c97957 5 (+1)	24 kDa		unknown	1,6054	0	93%	0
927	TRUE	FALSE	PREDICTED: similar to chromaffin granule amine transporter [Acyr	gALL v3 c9064 2	31 kDa		unknown	1,944	0	93%	0
928	TRUE	FALSE	protein BRAFLDRAFT 232052 [Branchiostoma floridae]	gALL v3 rep c104072 4hypothet	?		unknown	0	1,8851	0	93%
929	TRUE	FALSE	Thyroid hormone receptor-associated protein, putative [Pediculus	gALL v3 c18420 4 (+1)	43 kDa		unknown	1,6054	0	93%	0
930	TRUE	FALSE	beta-glucosidase [Zunongwangia profunda SM-A87]	gALL v3 c14740 6	25 kDa		unknown	1,371	0	93%	0
931	TRUE	FALSE	PREDICTED: similar to Tetraspanin 29Fb CG9496-PB, isoform B [A	gALL v3 c15358 4	25 kDa		unknown	1,371	0	93%	0
932	TRUE	FALSE	PREDICTED: similar to CG9674 CG9674-PA [Acyrthosiphon pisum]	gALL v3 c15615 6 (+1)	31 kDa		unknown	1,371	0	93%	0
933	TRUE	FALSE	sarcosine dehydrogenase [Aedes aegypti]	gALL v3 c17125 3 (+2)	19 kDa		unknown	1,371	0	93%	0
934	TRUE	FALSE	PREDICTED: hypothetical protein [Nasonia vitripennis]	gALL v3 c21286 6	43 kDa		unknown	1,371	0	93%	0
935	TRUE	FALSE	AGAP007653-PA [Anopheles gambiae str. PEST]	gALL v3 c24958 5	23 kDa		unknown	1,371	0	93%	0
936	TRUE	FALSE	leucine rich protein, putative [Aedes aegypti]	gALL v3 c25355 6	27 kDa		unknown	1,371	0	93%	0
937	TRUE	FALSE	Hsp90 protein, putative [Pediculus humanus corporis]	gALL v3 c2688 4	70 kDa		unknown	1,371	0	93%	0
938	TRUE	FALSE	Cadherin-23 precursor, putative [Pediculus humanus corporis]	gALL v3 c27663 1	22 kDa		unknown	1,371	0	93%	0
939	TRUE	FALSE	PREDICTED: similar to ENSANGP00000020237 [Nasonia vitripennis	gALL v3 c27974 1	26 kDa		unknown	1,371	0	93%	0
940	TRUE	FALSE	conserved hypothetical protein [Pediculus humanus corporis]	gALL v3 c30807 2	23 kDa		unknown	1,371	0	93%	0
941	TRUE	FALSE	neurexin-4 [Culex quinquefasciatus]	gALL v3 c3294 5	93 kDa		unknown	1,371	0	93%	0
942	TRUE	FALSE	PREDICTED: similar to fatty acid synthase [Tribolium castaneum]	gALL v3 c33021 5 (+1)	42 kDa		unknown	1,371	0	93%	0
943	TRUE	FALSE	PREDICTED: similar to Hsc70Cb CG6603-PA, isoform A isoform 1 [	gALL v3 c35087 3	25 kDa		unknown	1,371	0	93%	0
944	TRUE	FALSE	PREDICTED: similar to ebna2 binding protein P100 [Tribolium cast	gALL v3 c408 5	127 kDa		unknown	1,371	0	93%	0
945	TRUE	FALSE	PREDICTED: similar to ebna2 binding protein P100 [Tribolium cast	gALL v3 c408 6	120 kDa		unknown	1,371	0	93%	0
946	TRUE	FALSE	PREDICTED: similar to zinc finger protein 143 (clone pHZ-1) [Apis	gALL v3 c41591 5	17 kDa		unknown	1,371	0	93%	0
947	TRUE	FALSE	PREDICTED: similar to conserved hypothetical protein [Nasonia vit	gALL v3 c46589 3	9 kDa		unknown	1,371	0	93%	0
948	TRUE	FALSE	PREDICTED: similar to ENSANGP00000023647 [Nasonia vitripennis	gALL v3 c50878 4 (+1)	11 kDa		unknown	1,371	0	93%	0
949	TRUE	FALSE	heat shock protein 90 [Gryllus firmus]	gALL v3 c51823 5 (+1)	17 kDa		unknown	1,371	0	93%	0
950	TRUE	FALSE	PREDICTED: similar to Thiolester containing protein II CG7052-PB,	gALL v3 c6197 2	47 kDa		unknown	1,371	0	93%	0
951	TRUE	FALSE	protein purity of essence, putative [Pediculus humanus corporis]	gALL v3 c65106 5	10 kDa		unknown	1,371	0	93%	0
952	TRUE	FALSE	ATP-citrate synthase [Aedes aegypti]	gALL v3 c66756 6	19 kDa		unknown	1,371	0	93%	0
953	TRUE	FALSE	SARDH protein [Homo sapiens]	gALL v3 c77399 3	13 kDa		unknown	1,371	0	93%	0
954	TRUE	FALSE	PREDICTED: similar to CG9674 CG9674-PA [Acyrthosiphon pisum]	gALL v3 c8941 2	27 kDa		unknown	1,371	0	93%	0
955	TRUE	FALSE	Ves G 5 allergen precursor, putative [Pediculus humanus corporis]	gALL v3 c907 6	47 kDa		unknown	1,371	0	93%	0
956	TRUE	FALSE	ferredoxin-dependent glutamate synthase 2, putative [Pediculus h	gALL v3 c9375 6	42 kDa		unknown	1,371	0	93%	0
957	TRUE	FALSE	c oxidase subunit I [Eupolyphaga sinensis]	gALL v3 rep c101304 4cytochro	?		unknown	1,371	0	93%	0
958	TRUE	FALSE	[Teleogryllus emma]	gALL v3 rep c105815 6chymotry	?		unknown	1,371	0	93%	0
959	TRUE	FALSE	Plasma membrane calcium-transporting ATPase, putative [Pediculu	gALL v3 rep c97169 6	33 kDa		unknown	1,371	0	93%	0
960	TRUE	FALSE	hypothetical protein LOC100168726 [Acyrthosiphon pisum]	gALL v3 rep c97386 2	36 kDa		unknown	1,371	0	93%	0
961	TRUE	FALSE	Spectrin alpha chain, putative [Pediculus humanus corporis]	gALL v3 rep c97481 4	49 kDa	TRUE	unknown	1,371	0	93%	0
962	TRUE	FALSE	PREDICTED: similar to KIAA1513 protein [Monodelphis domestica]	gALL v3 c2478 4	78 kDa		unknown	1,6054	0	93%	0
963	TRUE	FALSE	coiled-coil domain-containing protein 47 precursor, putative [Pedic	gALL v3 c3505 4	48 kDa		unknown	1,371	0	92%	0
964	TRUE	FALSE	methylglutaconyl-CoA hydratase, putative [Pediculus humanus cor	gALL v3 c3600 5	22 kDa		unknown	0	1,8851	0	92%
965	TRUE	FALSE	hypothetical protein TcasGA2 TC011986 [Tribolium castaneum]	gALL v3 c8298 2	31 kDa		unknown	1,6054	0	92%	0
966	TRUE	FALSE	LOC496630 protein [Xenopus (Silurana) tropicalis]	gALL v3 c10059 3	119 kDa		unknown	0	1,0261	0	92%
967	TRUE	FALSE	RAS-like protein [Bombus ignitus]	gALL v3 c1006 4	63 kDa		unknown	0	1,0261	0	92%
968	TRUE	FALSE	ribosomal subunit S24e [Lygus lineolaris]	gALL v3 c11596 5 (+1)	30 kDa		unknown	0	1,0261	0	92%
969	TRUE	FALSE	PREDICTED: similar to RAB18, member RAS oncogene family [Trib	gALL v3 c12076 6	17 kDa		unknown	0	1,0261	0	92%
970	TRUE	FALSE	PREDICTED: similar to putative prefoldin [Nasonia vitripennis]	gALL v3 c16075 3	19 kDa		unknown	0	1,0261	0	92%
971	TRUE	FALSE	ACYPI009739 [Acyrthosiphon pisum]	gALL v3 c17065 2	46 kDa		unknown	0	1,0261	0	92%
972	TRUE	FALSE	glyceraldehyde-3-phosphate dehydrogenase [Pseudotrichonympha	gALL v3 c18872 3	19 kDa		unknown	0	1,0261	0	92%
973	TRUE	FALSE	PREDICTED: similar to LD32520p [Nasonia vitripennis]	gALL v3 c20967 6 (+3)	13 kDa		unknown	0	1,0261	0	92%
974	TRUE	FALSE	predicted xylanase/chitin deacetylase [Reticulitermes speratus]	gALL v3 c26010 1	14 kDa		unknown	0	1,0261	0	92%
975	TRUE	FALSE	14-3-3 protein [Trichomonas vaginalis G3]	gALL v3 c3454 6	20 kDa		unknown	0	1,0261	0	92%
976	TRUE	FALSE	PREDICTED: similar to UDP-glucose pyrophosphatase [Tribolium ca	gALL v3 c4040 1	55 kDa		unknown	0	1,0261	0	92%
977	TRUE	FALSE	Rab-protein 14 [Tribolium castaneum]	gALL v3 c4567 1	39 kDa	TRUE	unknown	0	2,0522	0	92%
978	TRUE	FALSE	translation machinery-associated protein, putative [Pediculus hum	gALL v3 c47044 5	24 kDa		unknown	0	1,0261	0	92%
979	TRUE	FALSE	mKIAA1601 protein [Mus musculus]	gALL v3 c5436 5	79 kDa		unknown	0	1,0261	0	92%
980	TRUE	FALSE	cathepsin C [Fenneropenaeus chinensis]	gALL v3 c5485 2	20 kDa		unknown	0	1,0261	0	92%
981	TRUE	FALSE	hypothetical protein BRAFLDRAFT 277165 [Branchiostoma floridae	gALL v3 c56415 6 (+1)	15 kDa		unknown	0	1,0261	0	92%
982	TRUE	FALSE	fibrinolytic enzyme [Eupolyphaga sinensis]	gALL v3 c5714 3	16 kDa		unknown	0	1,0261	0	92%
983	TRUE	FALSE	IP18656p [Drosophila melanogaster]	gALL v3 c57491 4	9 kDa		unknown	0	1,0261	0	92%
984	TRUE	FALSE	Chain A, Crystal Structure Of Beta-Glucosidase From Termite Neot	gALL v3 c5824 4	39 kDa		unknown	0	1,0261	0	92%
985	TRUE	FALSE	14-3-3 protein [Trichomonas vaginalis G3]	gALL v3 c58393 3	11 kDa	TRUE	unknown	0	1,0261	0	92%
986	TRUE	FALSE	conserved hypothetical protein [Pediculus humanus corporis]	gALL v3 c6976 1	40 kDa		unknown	0	1,0261	0	92%
987	TRUE	FALSE	PREDICTED: similar to CG1443 CG1443-PA [Tribolium castaneum]	gALL v3 c8683 4	39 kDa		unknown	0	1,0261	0	92%
988	TRUE	FALSE	light chain [Gryllotalpa orientalis]	gALL v3 rep c100231 2myosin	?	TRUE	unknown	0	3,0784	0	92%
989	TRUE	FALSE	PREDICTED: similar to eukaryotic initiation factor 4A [Tribolium ca	gALL v3 rep c96292 4 (+1)	80 kDa		unknown	0	1,0261	0	92%
990	TRUE	FALSE	Bla g 1.02 variant allergen [Blattella germanica]	gALL v3 rep c96321 4	133 kDa		unknown	0	1,0261	0	92%
991	TRUE	FALSE	thaumatin-like protein 1 [Schistocerca gregaria]	gALL v3 rep c96330 6 (+1)	34 kDa		unknown	0	1,0261	0	92%
992	TRUE	FALSE	alpha tubulin [Pseudotrichonympha grassii]	gALL v3 rep c96410 6	55 kDa	TRUE	unknown	0	2,0522	0	92%
993	TRUE	FALSE	conserved hypothetical protein [Pediculus humanus corporis]	gALL v3 rep c96702 3 (+3)	95 kDa		unknown	0	1,0261	0	92%
994	TRUE	FALSE	peptidoglycan-recognition protein-SC2 precursor-like protein [Brac	gALL v3 rep c96770 3	39 kDa		unknown	0	1,0261	0	92%
995	TRUE	FALSE	Mechanosensory protein, putative [Pediculus humanus corporis]	gALL v3 c1159 6	103 kDa		unknown	1,1388	0	92%	0
996	TRUE	FALSE	PREDICTED: similar to Rho GDP-dissociation inhibitor [Nasonia vit	gALL v3 c13085 3	24 kDa	TRUE	unknown	3,4165	0	92%	0
997	TRUE	FALSE	hypothetical protein TcasGA2 TC004440 [Tribolium castaneum]	gALL v3 c16540 5	18 kDa		unknown	1,1388	0	92%	0
998	TRUE	FALSE	PREDICTED: similar to calbindin-32 [Acyrthosiphon pisum]	gALL v3 c17983 5	21 kDa		unknown	1,1388	0	92%	0
999	TRUE	FALSE	class B secretin-like G-protein coupled receptor GPRcir1, putative	gALL v3 c1863 1	83 kDa		unknown	1,1388	0	92%	0
1000	TRUE	FALSE	PREDICTED: similar to AGAP004270-PA [Acyrthosiphon pisum]	gALL v3 c18839 2	28 kDa		unknown	1,1388	0	92%	0
1001	TRUE	FALSE	takeout/JHBP-like protein [Diploptera punctata]	gALL v3 c20700 1 (+1)	13 kDa		unknown	1,1388	0	92%	0
1002	TRUE	FALSE	female neotenic-specific protein 8 [Cryptotermes secundus]	gALL v3 c23911 5 (+3)	28 kDa		unknown	1,1388	0	92%	0
1003	TRUE	FALSE	endonuclease-reverse transcriptase [Bombyx mori]	gALL v3 c24563 2	21 kDa		unknown	1,1388	0	92%	0
1004	TRUE	FALSE	PREDICTED: similar to Sepiapterin reductase CG12117-PA [Apis m	gALL v3 c29681 3	26 kDa		unknown	1,1388	0	92%	0
1005	TRUE	FALSE	PREDICTED: similar to heat shock protein 20.6 isoform 1 [Nasonia	gALL v3 c30645 6	23 kDa		unknown	1,1388	0	92%	0
1006	TRUE	FALSE	syntaxin [Scolopendra polymorpha]	gALL v3 c30665 2 (+1)	14 kDa		unknown	1,1388	0	92%	0
1007	TRUE	FALSE	proliferating cell nuclear antigen [Marsupenaeus japonicus]	gALL v3 c3889 2	27 kDa		unknown	1,1388	0	92%	0
1008	TRUE	FALSE	PREDICTED: similar to T16G1.9 [Apis mellifera]	gALL v3 c41506 6	16 kDa		unknown	1,1388	0	92%	0
1009	TRUE	FALSE	PREDICTED: similar to 40S ribosomal protein S6 [Apis mellifera]	gALL v3 c42616 2	16 kDa		unknown	1,1388	0	92%	0
1010	TRUE	FALSE	PREDICTED: similar to CG11009-PA [Apis mellifera]	gALL v3 c4683 4	51 kDa		unknown	1,1388	0	92%	0
1011	TRUE	FALSE	PREDICTED: similar to CRK protein [Nasonia vitripennis]	gALL v3 c4903 5	46 kDa		unknown	1,1388	0	92%	0
1012	TRUE	FALSE	putative ribosomal protein L19e [Graphocephala atropunctata]	gALL v3 c63502 1 (+1)	29 kDa		unknown	1,1388	0	92%	0
1013	TRUE	FALSE	hypothetical protein [Entamoeba dispar SAW760]	gALL v3 c65309 2	8 kDa		unknown	1,1388	0	92%	0
1014	TRUE	FALSE	endonuclease-reverse transcriptase [Bombyx mori]	gALL v3 c70334 2	12 kDa		unknown	1,1388	0	92%	0
1015	TRUE	FALSE	PREDICTED: similar to soldier-specific protein-1 [Acyrthosiphon pi	gALL v3 c70722 3	22 kDa		unknown	1,1388	0	92%	0
1016	TRUE	FALSE	PREDICTED: similar to AGAP009953-PA [Tribolium castaneum]	gALL v3 c7084 2	18 kDa		unknown	1,1388	0	92%	0
1017	TRUE	FALSE	mitochondrial ribosomal protein S31 [Tribolium castaneum]	gALL v3 c7291 2	48 kDa		unknown	1,1388	0	92%	0
1018	TRUE	FALSE	hypothetical protein BRAFLDRAFT 126580 [Branchiostoma floridae	gALL v3 c75249 2	17 kDa		unknown	1,1388	0	92%	0
1019	TRUE	FALSE	cytosolic juvenile hormone binding protein 36 kDa subunit [Bomby	gALL v3 c8737 5	38 kDa		unknown	1,1388	0	92%	0
1020	TRUE	FALSE	truncated xanthine dehydrogenase [Bombyx mori]	gALL v3 c9410 4	23 kDa		unknown	1,1388	0	92%	0
1021	TRUE	FALSE	[Pyrrhocoris apterus]	gALL v3 rep c100889 5actin	?	TRUE	unknown	4,5553	0	92%	0
1022	TRUE	FALSE	dehydrogenase [Holomastigotoides mirabile]	gALL v3 rep c107398 4glycerald	?	TRUE	unknown	1,1388	0	92%	0
1023	TRUE	FALSE	PREDICTED: similar to F-actin capping protein subunit alpha [Apis	gALL v3 rep c96707 5	53 kDa		unknown	1,1388	0	92%	0
1024	TRUE	FALSE	mitochondrial cytochrome c1 [Tribolium castaneum]	gALL v3 rep c96852 2	32 kDa		unknown	1,1388	0	92%	0
1025	TRUE	FALSE	AGAP001053-PB [Anopheles gambiae str. PEST]	gALL v3 rep c97114 3	27 kDa	TRUE	unknown	2,2777	0	92%	0
1026	TRUE	FALSE	hypothetical protein [Acyrthosiphon pisum]	gALL v3 rep c98454 6	24 kDa	TRUE	unknown	1,1388	0	92%	0
1027	TRUE	FALSE	ACYPI002894 [Acyrthosiphon pisum]	gALL v3 rep c99115 2 (+1)	35 kDa		unknown	1,1388	0	92%	0
1028	TRUE	FALSE	PREDICTED: similar to mitochondrial single-stranded DNA-binding	gALL v3 c13367 5	15 kDa		unknown	1,944	0	92%	0
1029	TRUE	FALSE	archease [Acyrthosiphon pisum]	gALL v3 c28431 5	10 kDa		unknown	1,6054	0	92%	0
1030	TRUE	FALSE	PREDICTED: similar to Thioredoxin-like 2 protein (PKC-interacting	gALL v3 c6507 2	45 kDa		unknown	0	1,0261	0	92%
1031	TRUE	FALSE	60S ribosomal protein L30, putative [Pediculus humanus corporis]	gALL v3 c7835 6	19 kDa		unknown	1,944	0	92%	0
1032	TRUE	FALSE	PREDICTED: similar to beta chain spectrin [Acyrthosiphon pisum]	gALL v3 rep c96605 1 (+1)	86 kDa		unknown	0	1,8851	0	91%
1033	TRUE	FALSE	PREDICTED: similar to CG1319-PA [Apis mellifera]	gALL v3 c959 2	48 kDa		unknown	0	1,8851	0	91%
1034	TRUE	FALSE	PREDICTED: similar to CG2862-PA, isoform A [Apis mellifera]	gALL v3 c8050 1	23 kDa		unknown	0	1,8851	0	91%
1035	TRUE	FALSE	PREDICTED: similar to NADH:ubiquinone dehydrogenase, putative	gALL v3 c8805 1	20 kDa		unknown	1,6054	0	91%	0
1036	TRUE	FALSE	glyceraldehyde-3-phosphate dehydrogenase [Holomastigotoides m	gALL v3 c10967 2 (+3)	29 kDa		unknown	0	1,0261	0	91%
1037	TRUE	FALSE	hypothetical protein TcasGA2 TC009918 [Tribolium castaneum]	gALL v3 c62462 2	11 kDa		unknown	0	1,8851	0	91%
1038	TRUE	FALSE	PREDICTED: similar to fat2 CG7749-PA, isoform A [Apis mellifera]	gALL v3 c30352 4	21 kDa		unknown	1,944	0	91%	0
1039	TRUE	FALSE	serpin peptidase inhibitor 3 [Tribolium castaneum]	gALL v3 c12155 6	23 kDa	TRUE	unknown	0	0,88083	0	91%
1040	TRUE	FALSE	hypothetical protein TcasGA2 TC001523 [Tribolium castaneum]	gALL v3 c14511 4	31 kDa	TRUE	unknown	0	1,7617	0	91%
1041	TRUE	FALSE	14-3-3 protein [Trichomonas vaginalis G3]	gALL v3 c16482 5	25 kDa	TRUE	unknown	0	0,88083	0	91%
1042	TRUE	FALSE	PREDICTED: similar to conserved hypothetical protein [Tribolium c	gALL v3 c1887 4 (+3)	146 kDa		unknown	0	0,88083	0	91%
1043	TRUE	FALSE	PREDICTED: similar to AGAP008717-PA [Tribolium castaneum]	gALL v3 c21066 5	33 kDa		unknown	0	0,88083	0	91%
1044	TRUE	FALSE	PREDICTED: similar to Neu3 CG7649-PB, isoform B [Apis mellifera	gALL v3 c21760 6	35 kDa		unknown	0	0,88083	0	91%
1045	TRUE	FALSE	PREDICTED: similar to 3-hydroxyisobutyrate dehydrogenase [Nasc	gALL v3 c2382 4	66 kDa		unknown	0	0,88083	0	91%
1046	TRUE	FALSE	isopentenyl-diphosphate delta isomerase [Aedes aegypti]	gALL v3 c2415 3	58 kDa		unknown	0	0,88083	0	91%
1047	TRUE	FALSE	dynamin [Aedes aegypti]	gALL v3 c24739 6	42 kDa		unknown	0	0,88083	0	91%
1048	TRUE	FALSE	PREDICTED: similar to nad dependent epimerase/dehydratase [Tril	gALL v3 c29506 6 (+1)	19 kDa		unknown	0	0,88083	0	91%
1049	TRUE	FALSE	PREDICTED: similar to Sepiapterin reductase CG12117-PA [Apis m	gALL v3 c29681 1	25 kDa		unknown	0	0,88083	0	91%
1050	TRUE	FALSE	PREDICTED: similar to Putative phospholipase B-like 2 precursor (l	gALL v3 c30974 1	18 kDa		unknown	0	0,88083	0	91%
1051	TRUE	FALSE	ribosomal protein L13 [Chrysomela tremulae]	gALL v3 c31361 5	14 kDa		unknown	0	0,88083	0	91%
1052	TRUE	FALSE	14-3-3 protein [Trichomonas vaginalis G3]	gALL v3 c32946 5	20 kDa		unknown	0	0,88083	0	91%
1053	TRUE	FALSE	PREDICTED: similar to proteasome alpha 4 subunit [Nasonia vitrip	gALL v3 c36524 4	20 kDa	TRUE	unknown	0	1,7617	0	91%
1054	TRUE	FALSE	Phytanoyl-CoA dioxygenase, peroxisomal precursor, putative [Pedi	gALL v3 c4703 6	33 kDa		unknown	0	0,88083	0	91%
1055	TRUE	FALSE	proteasome inhibitor, putative [Pediculus humanus corporis]	gALL v3 c5008 4	60 kDa		unknown	0	0,88083	0	91%
1056	TRUE	FALSE	conserved hypothetical protein [Pediculus humanus corporis]	gALL v3 c525 6	48 kDa		unknown	0	0,88083	0	91%

1057	TRUE	FALSE	PREDICTED: similar to ENSANGP00000011437 [Nasonia vitripenni	gALL v3 c5260 5	42 kDa			unknown	0	0,88083	0	91%
1058	TRUE	FALSE	AGAP007475-PA [Anopheles gambiae str. PEST]	gALL v3 c5333 1 (+2)	33 kDa			unknown	0	0.88083	0	91%
1059	TRUE	FALSE	PREDICTED: similar to AGAP003514-PA [Tribolium castaneum]	gALL v3 c5533 4 (+1)	37 kDa			unknown	0	0,88083	0	91%
1060	TRUE	FALSE	putative alcohol dehydrogenase [Gryllotalpa orientalis]	gALL v3 c59393 1	13 kDa		TRUE	unknown	0	1.7617	0	91%
1061	TRUE	FALSE	14-3-3 protein [Trichomonas vaginalis G3]	gALL v3 c7534 2	24 kDa		TRUE	unknown	0	0,88083	0	91%
1062	TRUE	FALSE	PREDICTED: similar to beaten path IIa [Nasonia vitripennis]	gALL v3 c7947 2	88 kDa			unknown	0	0.88083	0	91%
1063	TRUE	FALSE	putative alcohol dehydrogenase [Gryllotalpa orientalis]	gALL v3 c80395 5	10 kDa			unknown	0	0,88083	0	91%
1064	TRUE	FALSE	elongation factor 1 delta [Bombyx mori]	gALL v3 c81451 3	15 kDa			unknown	0	0.88083	0	91%
1065	TRUE	FALSE	PREDICTED: similar to 6-phosphogluconolactonase [Nasonia vitrip	gALL v3 c8239 6	20 kDa			unknown	0	0,88083	0	91%
1066	TRUE	FALSE	putative alcohol dehydrogenase [Gryllotalpa orientalis]	gALL v3 c8260 2	27 kDa		TRUE	unknown	0	1.7617	0	91%
1067	TRUE	FALSE	PREDICTED: similar to ENSANGP00000014264 [Nasonia vitripenni	gALL v3 c8470 6 (+1)	24 kDa			unknown	0	0,88083	0	91%
1068	TRUE	FALSE	hypothetical protein TcasGA2 TC002587 [Tribolium castaneum]	gALL v3 c84858 6	12 kDa			unknown	0	0.88083	0	91%
1069	TRUE	FALSE	PREDICTED: similar to CG10399 CG10399-PA isoform 1 [Acyrthosi	gALL v3 c85932 6	16 kDa			unknown	0	0,88083	0	91%
1070	TRUE	FALSE	fibrinolytic enzyme [Eupolyphaga sinensis]	gALL v3 rep c96413 1	30 kDa			unknown	0	0.88083	0	91%
1071	TRUE	FALSE	conserved hypothetical protein [Pediculus humanus corporis]	gALL v3 rep c96721 2	43 kDa			unknown	0	0,88083	0	91%
1072	TRUE	FALSE	PREDICTED: similar to fatty acid synthase [Tribolium castaneum]	gALL v3 rep c96961 4	45 kDa			unknown	0	0.88083	0	91%
1073	TRUE	FALSE	GI21243 [Drosophila mojavensis]	gALL v3 rep c99725 4	15 kDa		TRUE	unknown	0	0,88083	0	91%
1074	TRUE	FALSE	PREDICTED: similar to retinol dehydrogenase 11 [Apis mellifera]	gALL v3 c873 5	95 kDa			unknown	1.944	0	91%	0
1075	TRUE	FALSE	PREDICTED: similar to GA18075-PA [Nasonia vitripennis]	gALL v3 c92779 1	15 kDa			unknown	0	1,0261	0	91%
1076	TRUE	FALSE	PREDICTED: similar to CG17374 CG17374-PA [Acyrthosiphon pisu	gALL v3 c41733 1 (+2)	22 kDa			unknown	1,371	0	91%	0
1077	TRUE	FALSE	PREDICTED: similar to ENSANGP00000028478 [Nasonia vitripenni	gALL v3 c1303 1	69 kDa			unknown	1,6054	0	91%	0
1078	TRUE	FALSE	serpin peptidase inhibitor 3 [Tribolium castaneum]	gALL v3 c11693 2	19 kDa		TRUE	unknown	1,7806	0	91%	0
1079	TRUE	FALSE	cuticular protein [Tribolium castaneum]	gALL v3 c12574 2 (+1)	16 kDa			unknown	0,89028	0	91%	0
1080	TRUE	FALSE	PREDICTED: similar to citrate synthase [Tribolium castaneum]	gALL v3 c12788 2	18 kDa			unknown	0,89028	0	91%	0
1081	TRUE	FALSE	PREDICTED: similar to homogentisate 1,2-dioxygenase [Nasonia v	gALL v3 c15471 2	31 kDa			unknown	0,89028	0	91%	0
1082	TRUE	FALSE	transaldolase [Aedes aegypti]	gALL v3 c16453 3	13 kDa		TRUE	unknown	1,7806	0	91%	0
1083	TRUE	FALSE	PREDICTED: similar to CG2247-PA [Apis mellifera]	gALL v3 c18104 4	29 kDa			unknown	0,89028	0	91%	0
1084	TRUE	FALSE	membrane alanyl aminopeptidase [Tenebrio molitor]	gALL v3 c2029 3 (+1)	54 kDa			unknown	0,89028	0	91%	0
1085	TRUE	FALSE	PREDICTED: similar to Rpt1 CG1341-PA [Apis mellifera]	gALL v3 c2524 1	49 kDa			unknown	0.89028	0	91%	0
1086	TRUE	FALSE	hypothetical protein TcasGA2 TC001493 [Tribolium castaneum]	gALL v3 c26131 3	22 kDa			unknown	0,89028	0	91%	0
1087	TRUE	FALSE	PREDICTED: similar to CG3609-PA isoform 2 [Apis mellifera]	gALL v3 c2618 4 (+1)	83 kDa			unknown	0.89028	0	91%	0
1088	TRUE	FALSE	hypothetical protein BRAFLDRAFT 211236 [Branchiostoma floridae	gALL v3 c26392 6	29 kDa		TRUE	unknown	1,7806	0	91%	0
1089	TRUE	FALSE	RecName: Full=Guanine nucleotide-binding protein G(o) subunit a	gALL v3 c2731 4 (+3)	26 kDa			unknown	0.89028	0	91%	0
1090	TRUE	FALSE	PREDICTED: similar to Zinc finger protein 271 (Zinc finger protein	gALL v3 c30820 1	24 kDa			unknown	0,89028	0	91%	0
1091	TRUE	FALSE	PREDICTED: piggyBac transposable element-derived protein 4-like	gALL v3 c33598 2	27 kDa			unknown	0,89028	0	91%	0
1092	TRUE	FALSE	PREDICTED: LOW QUALITY PROTEIN: midasin-like [Pongo abelii]	gALL v3 c39069 1	45 kDa			unknown	0,89028	0	91%	0
1093	TRUE	FALSE	PREDICTED: similar to GH13495p [Nasonia vitripennis]	gALL v3 c45626 4	21 kDa			unknown	0.89028	0	91%	0
1094	TRUE	FALSE	PREDICTED: similar to alcohol dehydrogenase [Nasonia vitripennis	gALL v3 c4940 5	18 kDa		TRUE	unknown	0,89028	0	91%	0
1095	TRUE	FALSE	PREDICTED: similar to Maternal protein exuperantia [Apis mellifera	gALL v3 c5581 3	54 kDa			unknown	0.89028	0	91%	0
1096	TRUE	FALSE	PREDICTED: similar to CG3609-PA [Apis mellifera]	gALL v3 c6686 1	41 kDa			unknown	0.89028	0	91%	0
1097	TRUE	FALSE	PREDICTED: similar to CG10576-PA, isoform A [Apis mellifera]	gALL v3 c7065 2	26 kDa			unknown	0,89028	0	91%	0
1098	TRUE	FALSE	PREDICTED: similar to histone H1 [Nasonia vitripennis]	gALL v3 c74434 1	16 kDa			unknown	0,89028	0	91%	0
1099	TRUE	FALSE	PREDICTED: similar to uricase [Nasonia vitripennis]	gALL v3 c7633 2	45 kDa			unknown	0,89028	0	91%	0
1100	TRUE	FALSE	PREDICTED: similar to microtubule-associated protein [Nasonia vit	gALL v3 c825 1	101 kDa			unknown	0,89028	0	91%	0
1101	TRUE	FALSE	dipeptidylpeptidase IV preproprotein [Vespula vulgaris]	gALL v3 c939 5	20 kDa			unknown	0.89028	0	91%	0
1102	TRUE	FALSE	[Drosophila willistoni]	gALL v3 rep c100417 1GK1536	?			unknown	0,89028	0	91%	0
1103	TRUE	FALSE	protein TcasGA2 TC015939 [Tribolium castaneum]	gALL v3 rep c104229 6hypothet	?			unknown	0,89028	0	91%	0
1104	TRUE	FALSE	venom dipeptidylpeptidase IV [Apis mellifera]	gALL v3 rep c97360 1	26 kDa			unknown	0,89028	0	91%	0
1105	TRUE	FALSE	60S acidic ribosomal protein P0 [Blaptica dubia]	gALL v3 rep c97615 5	29 kDa			unknown	0,89028	0	91%	0
1106	TRUE	FALSE	galactokinase [Danio rerio]	gALL v3 rep c98772 3	16 kDa		TRUE	unknown	0,89028	0	91%	0
1107	TRUE	FALSE	PREDICTED: similar to Ribosomal protein L38 [Nasonia vitripennis	gALL v3 c8622 5 (+5)	25 kDa			unknown	0	1,0261	0	91%
1108	TRUE	FALSE	U2 small nuclear ribonucleoprotein A, putative [Pediculus humanus	gALL v3 c25853 4	35 kDa			unknown	0	0,88083	0	91%
1109	TRUE	FALSE	PREDICTED: similar to glucose dehydrogenase [Tribolium castaneu	gALL v3 c10164 5 (+1)	79 kDa			unknown	0,99733	0	91%	0
1110	TRUE	FALSE	putative cytosolic malate dehydrogenase [Monocercomonas ATCC5	gALL v3 c10780 6	15 kDa			unknown	0,99733	0	91%	0
1111	TRUE	FALSE	PREDICTED: similar to juvenile hormone esterase [Acyrthosiphon	gALL v3 c13039 2	42 kDa			unknown	0,99733	0	91%	0
1112	TRUE	FALSE	PREDICTED: similar to AGAP003785-PA [Tribolium castaneum]	gALL v3 c1316 2	90 kDa		TRUE	unknown	0,99733	0	91%	0
1113	TRUE	FALSE	PREDICTED: similar to GA17312-PA [Tribolium castaneum]	gALL v3 c13265 1	24 kDa			unknown	0,99733	0	91%	0
1114	TRUE	FALSE	bifunctional purine biosynthesis protein [Aedes aegypti]	gALL v3 c14525 6	55 kDa			unknown	0,99733	0	91%	0
1115	TRUE	FALSE	PREDICTED: similar to transketolase-like 2 [Acyrthosiphon pisum]	gALL v3 c15298 1	16 kDa		TRUE	unknown	0,99733	0	91%	0
1116	TRUE	FALSE	PREDICTED: similar to acyl-CoA oxidase [Nasonia vitripennis]	gALL v3 c1745 2	80 kDa			unknown	0,99733	0	91%	0
1117	TRUE	FALSE	PREDICTED: similar to mannosidase, beta A, lysosomal [Acyrthosi	gALL v3 c19245 5	18 kDa			unknown	0,99733	0	91%	0
1118	TRUE	FALSE	ribosomal protein [Trichomonas vaginalis G3]	gALL v3 c20680 5	14 kDa			unknown	0,99733	0	91%	0
1119	TRUE	FALSE	PREDICTED: similar to 26S proteasome regulatory subunit rpn1 [N	gALL v3 c2604 5	58 kDa			unknown	0,99733	0	91%	0
1120	TRUE	FALSE	PREDICTED: similar to 26S proteasome regulatory subunit rpn1 [N	gALL v3 c2604 6	56 kDa			unknown	0.99733	0	91%	0
1121	TRUE	FALSE	GF18347 [Drosophila ananassae]	gALL v3 c27123 6 (+2)	14 kDa			unknown	0,99733	0	91%	0
1122	TRUE	FALSE	PREDICTED: similar to CG6904-PA, isoform A [Apis mellifera]	gALL v3 c292 1	33 kDa			unknown	0.99733	0	91%	0
1123	TRUE	FALSE	PREDICTED: similar to AGAP007642-PA [Acyrthosiphon pisum]	gALL v3 c29479 5 (+1)	39 kDa			unknown	0,99733	0	91%	0
1124	TRUE	FALSE	serpin peptidase inhibitor 5 [Tribolium castaneum]	gALL v3 c30744 5 (+1)	32 kDa			unknown	0.99733	0	91%	0
1125	TRUE	FALSE	PREDICTED: similar to pyruvate kinase [Nasonia vitripennis]	gALL v3 c31243 4	22 kDa		TRUE	unknown	3,9893	0	91%	0
1126	TRUE	FALSE	PREDICTED: similar to pyrroline-5-carboxylate dehydrogenase [Tri	gALL v3 c32823 1	15 kDa			unknown	0.99733	0	91%	0
1127	TRUE	FALSE	PREDICTED: similar to AGAP005630-PA [Acyrthosiphon pisum]	gALL v3 c35975 4	24 kDa		TRUE	unknown	0,99733	0	91%	0
1128	TRUE	FALSE	alanine aminotransferase [Aedes aegypti]	gALL v3 c3774 5	66 kDa			unknown	0.99733	0	91%	0
1129	TRUE	FALSE	RecName: Full=Hydroxymethylglutaryl-CoA synthase 1; Short=HM	gALL v3 c3817 4	48 kDa			unknown	0,99733	0	91%	0
1130	TRUE	FALSE	octopamine receptor [Periplaneta americana]	gALL v3 c44458 1	26 kDa			unknown	0,99733	0	91%	0
1131	TRUE	FALSE	PREDICTED: DDX19-like protein-like [Saccoglossus kowalevskii]	gALL v3 c47080 5	17 kDa			unknown	0,99733	0	91%	0
1132	TRUE	FALSE	GK18351 [Drosophila willistoni]	gALL v3 c5595 3	17 kDa			unknown	0,99733	0	91%	0
1133	TRUE	FALSE	PREDICTED: similar to acylpeptide hydrolase [Acyrthosiphon pisun	gALL v3 c59411 3	10 kDa			unknown	0,99733	0	91%	0
1134	TRUE	FALSE	PREDICTED: similar to endonuclease/reverse transcriptase [Stronc	gALL v3 c6916 1	28 kDa			unknown	0,99733	0	91%	0
1135	TRUE	FALSE	PREDICTED: similar to Prolyl endopeptidase [Nasonia vitripennis]	gALL v3 c6935 1	83 kDa			unknown	0,99733	0	91%	0
1136	TRUE	FALSE	PREDICTED: similar to Sb:cb283 protein [Tribolium castaneum]	gALL v3 c8237 4	39 kDa			unknown	0,99733	0	91%	0
1137	TRUE	FALSE	PREDICTED: similar to CG13397-PA [Apis mellifera]	gALL v3 c82461 1	8 kDa			unknown	0,99733	0	91%	0
1138	TRUE	FALSE	PREDICTED: similar to phosphoglucomutase 2 [Nasonia vitripennis	gALL v3 c8870 6	33 kDa			unknown	0,99733	0	91%	0
1139	TRUE	FALSE	hypothetical protein TcasGA2 TC007953 [Tribolium castaneum]	gALL v3 c94234 1	16 kDa			unknown	0.99733	0	91%	0
1140	TRUE	FALSE	PREDICTED: similar to malic enzyme [Tribolium castaneum]	gALL v3 c9427 2	51 kDa			unknown	0,99733	0	91%	0
1141	TRUE	FALSE	[Pyrrhocoris apterus]	gALL v3 rep c100281 6actin	?			unknown	0.99733	0	91%	0
1142	TRUE	FALSE	similar to serpin 6 [Nasonia vitripennis]	gALL v3 rep c100593 1PREDICT	?			unknown	0,99733	0	91%	0
1143	TRUE	FALSE	AGAP005124-PC [Anopheles gambiae str. PEST]	gALL v3 rep c96860 1 (+1)	111 kDa			unknown	0.99733	0	91%	0
1144	TRUE	FALSE	predicted protein [Nematostella vectensis]	gALL v3 s105961 3	11 kDa			unknown	0,99733	0	91%	0
1145	TRUE	FALSE	predicted protein [Naegleria gruberi]	gALL v3 c16375 2 (+1)	25 kDa			unknown	0	0.88083	0	91%
1146	TRUE	FALSE	GJ18493 [Drosophila virilis]	gALL v3 c577 6	65 kDa			unknown	1,1388	0	91%	0
1147	TRUE	FALSE	hypothetical protein TcasGA2 TC007347 [Tribolium castaneum]	gALL v3 c44537 2	13 kDa			unknown	1.371	0	91%	0
1148	TRUE	FALSE	similar to CG17374 CG17374-PA [Acyrthosiphon pisum]	gALL v3 rep c104571 5PREDICT	?			unknown	0,89028	0	91%	0
1149	TRUE	FALSE	hypothetical protein GFP L7 0730 [Glyptapanteles flavicoxis]	gALL v3 c37081 5	23 kDa			unknown	0.99733	0	91%	0
1150	TRUE	FALSE	PREDICTED: similar to conserved hypothetical protein [Tribolium c	gALL v3 c57694 4	15 kDa			unknown	1,371	0	91%	0
1151	TRUE	FALSE	polyprotein of viral origin, putative [Ixodes scapularis]	gALL v3 c50131 1	16 kDa			unknown	0.99733	0	90%	0
1152	TRUE	FALSE	hypothetical protein TcasGA2 TC030701 [Tribolium castaneum]	gALL v3 rep c96265 6	302 kDa			unknown	1,371	0	90%	0
1153	TRUE	FALSE	PREDICTED: similar to RNA binding protein Vera [Tribolium castan	gALL v3 c56975 1	15 kDa			unknown	0	1.8851	0	90%
1154	TRUE	FALSE	GK16633 [Drosophila willistoni]	gALL v3 c9605 3	32 kDa			unknown	0,99733	0	90%	0
1155	TRUE	FALSE	conserved hypothetical protein [Pediculus humanus corporis]	gALL v3 c63140 3	11 kDa			unknown	1.371	0	90%	0
1156	TRUE	FALSE	vacuolar protein sorting, putative [Pediculus humanus corporis]	gALL v3 c13586 4	26 kDa			unknown	0,89028	0	90%	0
1157	TRUE	FALSE	PREDICTED: similar to Insulin receptor precursor (IR) [Apis mellife	gALL v3 c102392 6	21 kDa			unknown	1,6054	0	90%	0
1158	TRUE	FALSE	PREDICTED: similar to karst CG12008-PA, isoform A [Apis mellifer	gALL v3 c10893 4 (+2)	30 kDa			unknown	0	0,68834	0	90%
1159	TRUE	FALSE	PREDICTED: similar to CG31132-PA [Apis mellifera]	gALL v3 c11962 5	65 kDa			unknown	0	0,68834	0	90%
1160	TRUE	FALSE	PREDICTED: similar to metalloprotease [Tribolium castaneum]	gALL v3 c1196 6 (+1)	36 kDa			unknown	0	0,68834	0	90%
1161	TRUE	FALSE	PREDICTED: similar to ENSANGP00000022750 [Nasonia vitripenni	gALL v3 c1199 5 (+1)	96 kDa			unknown	0	0,68834	0	90%
1162	TRUE	FALSE	hypothetical protein TcasGA2 TC005186 [Tribolium castaneum]	gALL v3 c12059 4	19 kDa			unknown	0	0,68834	0	90%
1163	TRUE	FALSE	Neuroglian precursor, putative [Pediculus humanus corporis]	gALL v3 c1239 1 (+1)	66 kDa			unknown	0	0,68834	0	90%
1164	TRUE	FALSE	PREDICTED: similar to CG9485-PA, isoform A [Apis mellifera]	gALL v3 c12572 5	19 kDa			unknown	0	0,68834	0	90%
1165	TRUE	FALSE	PREDICTED: similar to CG14516-PA, isoform A isoform 1 [Apis me	gALL v3 c1302 3	100 kDa			unknown	0	0,68834	0	90%
1166	TRUE	FALSE	PREDICTED: similar to proteosome subunit RPN2 isoform 1 [Acyrt	gALL v3 c130 5	104 kDa			unknown	0	0.68834	0	90%
1167	TRUE	FALSE	hypothetical protein TcasGA2 TC015465 [Tribolium castaneum]	gALL v3 c14198 3	16 kDa		TRUE	unknown	0	1,3767	0	90%
1168	TRUE	FALSE	PREDICTED: similar to alpha-2-macroglobulin-like 1 [Apis mellifera	gALL v3 c14587 1	62 kDa			unknown	0	0.68834	0	90%
1169	TRUE	FALSE	ferredoxin-dependent glutamate synthase 2, putative [Pediculus h	gALL v3 c17118 4	34 kDa			unknown	0	0,68834	0	90%
1170	TRUE	FALSE	aminopeptidase 1 [Lucilia cuprina]	gALL v3 c1729 6	41 kDa			unknown	0	0,68834	0	90%
1171	TRUE	FALSE	PREDICTED: similar to ENSANGP00000011247 [Nasonia vitripenni	gALL v3 c1807 5	56 kDa			unknown	0	0,68834	0	90%
1172	TRUE	FALSE	PREDICTED: similar to importin (ran-binding protein) [Nasonia vitr	gALL v3 c18951 4	16 kDa			unknown	0	0,68834	0	90%
1173	TRUE	FALSE	hypothetical protein TcasGA2 TC011050 [Tribolium castaneum]	gALL v3 c2006 5	57 kDa			unknown	0	0,68834	0	90%
1174	TRUE	FALSE	hypothetical protein TcasGA2 TC007173 [Tribolium castaneum]	gALL v3 c20387 3	26 kDa		TRUE	unknown	0	2.065	0	90%
1175	TRUE	FALSE	hypothetical protein TcasGA2 TC004702 [Tribolium castaneum]	gALL v3 c20550 3	33 kDa			unknown	0	0,68834	0	90%
1176	TRUE	FALSE	PREDICTED: similar to alpha-2-macroglobulin precursor splice vari	gALL v3 c211 2	89 kDa			unknown	0	0,68834	0	90%
1177	TRUE	FALSE	ala-tRNA synthetase [Periplaneta americana]	gALL v3 c2170 5	24 kDa			unknown	0	0,68834	0	90%
1178	TRUE	FALSE	PREDICTED: similar to CG14476-PB, isoform B isoform 2 [Apis me	gALL v3 c2190 5	74 kDa			unknown	0	0,68834	0	90%
1179	TRUE	FALSE	GF21016 [Drosophila ananassae]	gALL v3 c221 4	81 kDa			unknown	0	0,68834	0	90%
1180	TRUE	FALSE	Talin-2, putative [Pediculus humanus corporis]	gALL v3 c2409 3 (+1)	50 kDa			unknown	0	0,68834	0	90%
1181	TRUE	FALSE	PREDICTED: similar to acetyl-coa carboxylase [Tribolium castaneu	gALL v3 c24551 5	30 kDa			unknown	0	0,68834	0	90%
1182	TRUE	FALSE	PREDICTED: similar to Probable ubiquitin carboxyl-terminal hydrol	gALL v3 c2756 5	43 kDa			unknown	0	0.68834	0	90%
1183	TRUE	FALSE	Spectrin alpha chain, putative [Pediculus humanus corporis]	gALL v3 c30507 4	17 kDa		TRUE	unknown	0	1,3767	0	90%
1184	TRUE	FALSE	neurexin-4 [Culex quinquefasciatus]	gALL v3 c3294 4	92 kDa			unknown	0	0,68834	0	90%
1185	TRUE	FALSE	PREDICTED: similar to fatty acid synthase [Nasonia vitripennis]	gALL v3 c3309 6	34 kDa			unknown	0	0,68834	0	90%
1186	TRUE	FALSE	hypothetical protein TcasGA2 TC012309 [Tribolium castaneum]	gALL v3 c3653 3 (+1)	53 kDa			unknown	0	0,68834	0	90%
1187	TRUE	FALSE	Aminopeptidase N precursor, putative [Pediculus humanus corporis	gALL v3 c38336 5	16 kDa			unknown	0	0,68834	0	90%
1188	TRUE	FALSE	acetyl-CoA carboxylase, putative [Pediculus humanus corporis]	gALL v3 c38441 5	12 kDa			unknown	0	0,68834	0	90%
1189	TRUE	FALSE	Aminopeptidase N precursor, putative [Pediculus humanus corporis	gALL v3 c41950 5	21 kDa			unknown	0	0,68834	0	90%
1190	TRUE	FALSE	hypothetical protein TcasGA2 TC030696 [Tribolium castaneum]	gALL v3 c43322 2	15 kDa			unknown	0	0,68834	0	90%
1191	TRUE	FALSE	PREDICTED: similar to alanyl-tRNA synthetase [Tribolium castaneu	gALL v3 c47254 4	30 kDa			unknown	0	0,68834	0	90%
1192	TRUE	FALSE	Microsomal triglyceride transfer protein large subunit precursor, pu	gALL v3 c48050 1	17 kDa			unknown	0	0,68834	0	90%
1193	TRUE	FALSE	sodium/nucleoside cotransporter [Aedes aegypti]	gALL v3 c58288 2	12 kDa			unknown	0	0.68834	0	90%
1194	TRUE	FALSE	PREDICTED: similar to acetyl-coa carboxylase [Tribolium castaneu	gALL v3 c5988 4	65 kDa			unknown	0	0,68834	0	90%
1195	TRUE	FALSE	villin, putative [Ixodes scapularis]	gALL v3 c60955 1 (+1)	17 kDa		TRUE	unknown	0	1.3767	0	90%
1196	TRUE	FALSE	PREDICTED: hypothetical protein [Nasonia vitripennis]	gALL v3 c6524 1	77 kDa			unknown	0	0,68834	0	90%
1197	TRUE	FALSE	PREDICTED: similar to xanthine dehydrogenase [Tribolium castane	gALL v3 c7244 6	45 kDa			unknown	0	0.68834	0	90%
1198	TRUE	FALSE	xanthine dehydrogenase, putative [Pediculus humanus corporis]	gALL v3 c73037 5	16 kDa			unknown	0	0,68834	0	90%
1199	TRUE	FALSE	PREDICTED: similar to CG1381-PA [Apis mellifera]	gALL v3 c75483 2	16 kDa			unknown	0	0,68834	0	90%
1200	TRUE	FALSE	Coatomer subunit beta, putative [Pediculus humanus corporis]	gALL v3 c8601 2	61 kDa			unknown	0	0,68834	0	90%
1201	TRUE	FALSE	PREDICTED: similar to CG9674 CG9674-PA [Acyrthosiphon pisum]	gALL v3 c9288 5 (+1)	49 kDa			unknown	0	0,68834	0	90%
1202	TRUE	FALSE	lysosomal alpha-mannosidase [Culex quinquefasciatus]	gALL v3 c95732 3	20 kDa			unknown	0	0,68834	0	90%
1203	TRUE	FALSE	similar to apolipophorin [Nasonia vitripennis]	gALL v3 rep c104587 3PREDICT	?		FALSE	unknown	0	18.585	0	90%
1204	TRUE	FALSE	PREDICTED: similar to Spectrin alpha chain [Apis mellifera]	gALL v3 rep c96933 2	22 kDa		TRUE	unknown	0	1,3767	0	90%
1205	TRUE	FALSE	putative trehalose-6-phosphate synthase 1 [Maconellicoccus hirsut	gALL v3 rep c97866 6 (+1)	18 kDa			unknown	0	0.68834	0	90%
1206	TRUE	FALSE	lipophorin precursor [Nilaparvata lugens]	gALL v3 rep c97902 5	26 kDa		TRUE	unknown	0	9,6367	0	90%
1207	TRUE	FALSE	similar to ORF2-encoded protein, partial [Hydra magnipapillata]	gALL v3 rep c103221 4PREDICT	?			unknown	0.99733	0	90%	0
1208	TRUE	FALSE	conserved hypothetical protein [Pediculus humanus corporis]	gALL v3 c12928 1	22 kDa			unknown	1,1388	0	90%	0
1209	TRUE	FALSE	ETHE1 protein, putative [Pediculus humanus corporis]	gALL v3 c6499 4	38 kDa			unknown	1.1388	0	90%	0
1210	TRUE	FALSE	conserved hypothetical protein [Pediculus humanus corporis]	gALL v3 c9117 2	35 kDa			unknown	0	0,88083	0	90%
1211	TRUE	FALSE	putative regulator of ribosome biosynthesis [Heliconius melpomen	gALL v3 c1677 5	46 kDa			unknown	0	1,8851	0	90%
1212	TRUE	FALSE	PREDICTED: similar to Rs1 CG2173-PA [Apis mellifera]	gALL v3 c26175 3	16 kDa			unknown	0	0,88083	0	89%
1213	TRUE	FALSE	reverse transcriptase-like [Bos taurus]	gALL v3 c6723 1	61 kDa			unknown	1,371	0	89%	0
1214	TRUE	FALSE	[Drosophila willistoni]	gALL v3 rep c100489 2GK2181	?			unknown	0	0,88083	0	89%
1215	TRUE	FALSE	endonuclease-reverse transcriptase [Bombyx mori]	gALL v3 c5362 1	59 kDa			unknown	0	0,88083	0	89%
1216	TRUE	FALSE	hypothetical protein CLOLEP 01448 [Clostridium leptum DSM 753	gALL v3 c19177 1	25 kDa			unknown	0	0,88083	0	89%
1217	TRUE	FALSE	conserved hypothetical protein [Pediculus humanus corporis]	gALL v3 c11011 5 (+1)	37 kDa			unknown	0	0,69313	0	89%
1218	TRUE	FALSE	PREDICTED: similar to Mannose-6-phosphate isomerase (Phospho	gALL v3 c12439 3	49 kDa			unknown	0	0,69313	0	89%
1219	TRUE	FALSE	PREDICTED: similar to sulfotransferase (sult) [Tribolium castaneun	gALL v3 c13185 4 (+2)	17 kDa			unknown	0	0,69313	0	89%
1220	TRUE	FALSE	PREDICTED: similar to vacuolar ATPase subunit C [Acyrthosiphon	gALL v3 c1343 4	65 kDa			unknown	0	0.69313	0	89%
1221	TRUE	FALSE	PREDICTED: similar to Rpt4 CG3455-PA [Apis mellifera]	gALL v3 c1385 1	45 kDa			unknown	0	0,69313	0	89%
1222	TRUE	FALSE	hypothetical protein BACCELL 02261 [Bacteroides cellulosilyticus	gALL v3 c14652 4	44 kDa			unknown	0	0.69313	0	89%
1223	TRUE	FALSE	conserved hypothetical protein [Pediculus humanus corporis]	gALL v3 c17084 6	45 kDa			unknown	0	0,69313	0	89%
1224	TRUE	FALSE	hypothetical protein TcasGA2 TC030576 [Tribolium castaneum]	gALL v3 c18339 4	23 kDa			unknown	0	0,69313	0	89%
1225	TRUE	FALSE	GK22419 [Drosophila willistoni]	gALL v3 c18620 4	16 kDa			unknown	0	0,69313	0	89%
1226	TRUE	FALSE	PREDICTED: similar to HSP90 cochaperone CDC37 homologue [Na	gALL v3 c20935 6	16 kDa			unknown	0	0.69313	0	89%
1227	TRUE	FALSE	PREDICTED: similar to argininosuccinate synthetase [Tribolium cas	gALL v3 c21939 1	50 kDa			unknown	0	0,69313	0	89%
1228	TRUE	FALSE	PREDICTED: similar to Peroxin-5 [Nasonia vitripennis]	gALL v3 c2417 4	24 kDa			unknown	0	0,69313	0	89%
1229	TRUE	FALSE	abhydrolase domain-containing protein 7 [Culex quinquefasciatus]	gALL v3 c244 3 (+2)	53 kDa			unknown	0	0,69313	0	89%
1230	TRUE	FALSE	PREDICTED: similar to jerky [Ornithorhynchus anatinus]	gALL v3 c28095 1	24 kDa			unknown	0	0.69313	0	89%
1231	TRUE	FALSE	PREDICTED: similar to aspartate aminotransferase [Nasonia vitripe	gALL v3 c28780 1	23 kDa		TRUE	unknown	0	1,3863	0	89%
1232	TRUE	FALSE	lysosomal thioesterase PPT2 [Culex quinquefasciatus]	gALL v3 c29484 5	40 kDa			unknown	0	0,69313	0	89%
1233	TRUE	FALSE	ornithine aminotransferase precursor [Drosophila ananassae]	gALL v3 c31269 1 (+1)	13 kDa			unknown	0	0,69313	0	89%
1234	TRUE	FALSE	PREDICTED: similar to Ubiquitin carboxyl-terminal hydrolase isozy	gALL v3 c34913 5	15 kDa			unknown	0	0,69313	0	89%
1235	TRUE	FALSE	AGAP006576-PC [Anopheles gambiae str. PEST]	gALL v3 c38774 6	20 kDa			unknown	0	0,69313	0	89%
1236	TRUE	FALSE	conserved hypothetical protein [Pediculus humanus corporis]	gALL v3 c4065 3	64 kDa			unknown	0	0,69313	0	89%
1237	TRUE	FALSE	ribosomal protein [Trichomonas vaginalis G3]	gALL v3 c43278 6	19 kDa			unknown	0	0,69313	0	89%

1238	TRUE	FALSE	GDP-mannose pyrophosphorylase [Glossina morsitans morsitans]	gALL v3 c4596 4 (+1)	57 kDa		unknown	0	0,69313	0	89%
1239	TRUE	FALSE	Arc42 [Drosophila willistoni]	gALL v3 c49549 3	27 kDa		unknown	0	0.69313	0	89%
1240	TRUE	FALSE	PREDICTED: similar to serpin 3a [Nasonia vitripennis]	gALL v3 c50553 2 (+1)	17 kDa		unknown	0	0,69313	0	89%
1241	TRUE	FALSE	PREDICTED: similar to Eukaryotic translation initiation factor 2 sub	gALL v3 c5092 4	39 kDa		unknown	0	0.69313	0	89%
1242	TRUE	FALSE	predicted protein [Nematostella vectensis]	gALL v3 c53272 2 (+1)	14 kDa		unknown	0	0,69313	0	89%
1243	TRUE	FALSE	PREDICTED: similar to glycinamide ribonucleotide synthetase-amir	gALL v3 c5473 4	36 kDa		unknown	0	0.69313	0	89%
1244	TRUE	FALSE	Calumenin precursor, putative [Pediculus humanus corporis]	gALL v3 c5525 6	49 kDa		unknown	0	0,69313	0	89%
1245	TRUE	FALSE	PREDICTED: similar to AGAP004654-PA [Tribolium castaneum]	gALL v3 c55450 3	17 kDa		unknown	0	0.69313	0	89%
1246	TRUE	FALSE	PREDICTED: similar to Lipid storage droplet-1 CG10374-PC isoforn	gALL v3 c57334 5 (+2)	26 kDa		unknown	0	0,69313	0	89%
1247	TRUE	FALSE	Parcxpwnx02 [Periplaneta americana]	gALL v3 c63825 6 (+1)	20 kDa		unknown	0	0.69313	0	89%
1248	TRUE	FALSE	PREDICTED: similar to CG5946 CG5946-PB [Tribolium castaneum]	gALL v3 c8998 3	18 kDa		unknown	0	0,69313	0	89%
1249	TRUE	FALSE	fructose-1,6-bisphosphate aldolase [Trichomonas vaginalis G3]	gALL v3 c9215 6	22 kDa		unknown	0	0.69313	0	89%
1250	TRUE	FALSE	GK23907 [Drosophila willistoni]	gALL v3 c9673 2 (+2)	47 kDa		unknown	0	0,69313	0	89%
1251	TRUE	FALSE	dehydrogenase [Branchiostoma floridae]	gALL v3 rep c101497 63-hydrox	?		unknown	0	0.69313	0	89%
1252	TRUE	FALSE	isoform 3 [Bombyx mori]	gALL v3 rep c107637 1tropomy	?	TRUE	unknown	0	1,3863	0	89%
1253	TRUE	FALSE	gram negative bacteria-binding protein 2 [Reticulitermes flavipes]	gALL v3 rep c96609 5	40 kDa		unknown	0	0.69313	0	89%
1254	TRUE	FALSE	zinc finger protein RNA binding protein, putative [Pediculus human	gALL v3 rep c96718 5	92 kDa		unknown	0	0,69313	0	89%
1255	TRUE	FALSE	hypothetical protein BRAFLDRAFT 262219 [Branchiostoma floridae	gALL v3 rep c97949 2	27 kDa		unknown	0	0.69313	0	89%
1256	TRUE	FALSE	RhoGEF domain containing protein [Trichomonas vaginalis G3]	gALL v3 c82211 5	18 kDa		unknown	0	0,68834	0	89%
1257	TRUE	FALSE	Thymidine phosphorylase [Salmo salar]	gALL v3 c10109 3	23 kDa	TRUE	unknown	0	0,62757	0	89%
1258	TRUE	FALSE	enolase [Pseudotrichonympha grassii]	gALL v3 c10380 1	11 kDa		unknown	0	0,62757	0	89%
1259	TRUE	FALSE	PREDICTED: similar to GH02263p [Tribolium castaneum]	gALL v3 c10603 2	47 kDa		unknown	0	0,62757	0	89%
1260	TRUE	FALSE	yellow-1 [Tribolium castaneum]	gALL v3 c11182 2	33 kDa		unknown	0	0,62757	0	89%
1261	TRUE	FALSE	retiin, putative [Pediculus humanus corporis]	gALL v3 c11313 4 (+2)	47 kDa		unknown	0	0,62757	0	89%
1262	TRUE	FALSE	unzipped [Apis mellifera]	gALL v3 c1176 4	72 kDa		unknown	0	0,62757	0	89%
1263	TRUE	FALSE	cytochrome P450 [Hodotermopsis sjoestedti]	gALL v3 c11806 5 (+3)	32 kDa		unknown	0	0,62757	0	89%
1264	TRUE	FALSE	PREDICTED: similar to multiple inositol polyphosphate phosphatas	gALL v3 c1220 3	62 kDa		unknown	0	0,62757	0	89%
1265	TRUE	FALSE	fimbrin [Trichomonas vaginalis G3]	gALL v3 c14718 4	30 kDa		unknown	0	0.62757	0	89%
1266	TRUE	FALSE	thioredoxin reductase 1 [Apis cerana cerana]	gALL v3 c15863 5	34 kDa		unknown	0	0,62757	0	89%
1267	TRUE	FALSE	PREDICTED: similar to prolyl endopeptidase isoform 1 [Apis mellife	gALL v3 c1600 1	42 kDa		unknown	0	0.62757	0	89%
1268	TRUE	FALSE	GF21727 [Drosophila ananassae]	gALL v3 c16714 3	34 kDa		unknown	0	0,62757	0	89%
1269	TRUE	FALSE	PREDICTED: similar to acyl-CoA oxidase [Nasonia vitripennis]	gALL v3 c1745 1	81 kDa		unknown	0	0.62757	0	89%
1270	TRUE	FALSE	PREDICTED: similar to acyl-CoA oxidase [Nasonia vitripennis]	gALL v3 c1745 3	81 kDa		unknown	0	0.62757	0	89%
1271	TRUE	FALSE	GK17957 [Drosophila willistoni]	gALL v3 c17477 4	17 kDa		unknown	0	0.62757	0	89%
1272	TRUE	FALSE	PREDICTED: similar to leucine aminopeptidase [Tribolium castaneu	gALL v3 c19142 4	19 kDa		unknown	0	0,62757	0	89%
1273	TRUE	FALSE	REGA-1 protein [Schistocerca americana]	gALL v3 c2123 5	53 kDa		unknown	0	0,62757	0	89%
1274	TRUE	FALSE	PREDICTED: similar to phosphatidylinositol glycan, class A [Apis m	gALL v3 c2355 4	37 kDa		unknown	0	0,62757	0	89%
1275	TRUE	FALSE	hypothetical protein TcasGA2 TC006281 [Tribolium castaneum]	gALL v3 c23872 4 (+2)	14 kDa		unknown	0	0,62757	0	89%
1276	TRUE	FALSE	fructose-1,6-bisphosphate aldolase [Trichomonas vaginalis G3]	gALL v3 c25110 6	17 kDa		unknown	0	0.62757	0	89%
1277	TRUE	FALSE	enolase [Spirotrichonympha leidyi]	gALL v3 c26190 3	30 kDa		unknown	0	0,62757	0	89%
1278	TRUE	FALSE	PREDICTED: similar to wd-repeat protein [Nasonia vitripennis]	gALL v3 c26476 6	14 kDa		unknown	0	0.62757	0	89%
1279	TRUE	FALSE	PREDICTED: similar to phosphatase [Nasonia vitripennis]	gALL v3 c2675 3	47 kDa		unknown	0	0,62757	0	89%
1280	TRUE	FALSE	Cytosolic nonspecific dipeptidase, putative [Pediculus humanus cor	gALL v3 c29037 4	19 kDa	TRUE	unknown	0	1.8827	0	89%
1281	TRUE	FALSE	phosphogluconate dehydrogenase [Periplaneta americana]	gALL v3 c30915 5	33 kDa		unknown	0	0,62757	0	89%
1282	TRUE	FALSE	PREDICTED: similar to protein transport protein sec23 [Tribolium c	gALL v3 c30928 1	33 kDa		unknown	0	0.62757	0	89%
1283	TRUE	FALSE	PREDICTED: similar to pyrroline-5-carboxylate dehydrogenase [Tri	gALL v3 c32823 2	15 kDa		unknown	0	0,62757	0	89%
1284	TRUE	FALSE	hemocyanin subunit F, putative [Pediculus humanus corporis]	gALL v3 c33030 6	19 kDa	TRUE	unknown	0	1,2551	0	89%
1285	TRUE	FALSE	RecName: Full=Fasciclin-1; AltName: Full=Fasciclin I; Short=FAS I	gALL v3 c3412 5 (+1)	60 kDa		unknown	0	0,62757	0	89%
1286	TRUE	FALSE	GDP dissociation inhibitor family protein [Trichomonas vaginalis G3	gALL v3 c34357 5	18 kDa		unknown	0	0,62757	0	89%
1287	TRUE	FALSE	PREDICTED: similar to succinic semialdehyde dehydrogenase isofo	gALL v3 c3672 1	83 kDa		unknown	0	0,62757	0	89%
1288	TRUE	FALSE	Mannan endo-1,4-beta-mannosidase [Fibrobacter succinogenes su	gALL v3 c37810 4	14 kDa		unknown	0	0,62757	0	89%
1289	TRUE	FALSE	AGAP006096-PA [Anopheles gambiae str. PEST]	gALL v3 c38037 1	13 kDa		unknown	0	0,62757	0	89%
1290	TRUE	FALSE	PREDICTED: similar to gmp synthase [Tribolium castaneum]	gALL v3 c4262 2	86 kDa		unknown	0	0,62757	0	89%
1291	TRUE	FALSE	PREDICTED: similar to putative zinc-dependent alcohol dehydroge	gALL v3 c4643 4	61 kDa		unknown	0	0,62757	0	89%
1292	TRUE	FALSE	hypothetical protein Phum PHUM580380 [Pediculus humanus corp	gALL v3 c47499 1	34 kDa		unknown	0	0,62757	0	89%
1293	TRUE	FALSE	PREDICTED: similar to glutamate carboxypeptidase [Nasonia vitrip	gALL v3 c4860 1	18 kDa	TRUE	unknown	0	1.2551	0	89%
1294	TRUE	FALSE	PREDICTED: similar to protein phosphatase-5 [Tribolium castaneu	gALL v3 c4996 3	46 kDa		unknown	0	0,62757	0	89%
1295	TRUE	FALSE	conserved hypothetical protein [Pediculus humanus corporis]	gALL v3 c5265 3	78 kDa		unknown	0	0.62757	0	89%
1296	TRUE	FALSE	PREDICTED: similar to juvenile hormone esterase [Tribolium casta	gALL v3 c5472 5	36 kDa		unknown	0	0,62757	0	89%
1297	TRUE	FALSE	PREDICTED: similar to CG12512 CG12512-PA [Tribolium castaneu	gALL v3 c5932 2	70 kDa		unknown	0	0.62757	0	89%
1298	TRUE	FALSE	transmembrane protein, putative [Pediculus humanus corporis]	gALL v3 c6253 3	53 kDa		unknown	0	0,62757	0	89%
1299	TRUE	FALSE	PREDICTED: similar to AGAP010429-PA [Tribolium castaneum]	gALL v3 c63304 5 (+1)	16 kDa		unknown	0	0.62757	0	89%
1300	TRUE	FALSE	GK15680 [Drosophila willistoni]	gALL v3 c6450 4	37 kDa		unknown	0	0,62757	0	89%
1301	TRUE	FALSE	AMP dependent coa ligase [Aedes aegypti]	gALL v3 c6478 4	69 kDa		unknown	0	0.62757	0	89%
1302	TRUE	FALSE	PREDICTED: similar to Fimbrin CG8649-PA, isoform A [Apis mellife	gALL v3 c6599 4	37 kDa		unknown	0	0,62757	0	89%
1303	TRUE	FALSE	noggin [Trachemys scripta]	gALL v3 c66966 3	27 kDa		unknown	0	0.62757	0	89%
1304	TRUE	FALSE	PREDICTED: similar to oxidoreductase [Nasonia vitripennis]	gALL v3 c68784 1	22 kDa		unknown	0	0,62757	0	89%
1305	TRUE	FALSE	PREDICTED: similar to lysosomal alpha-mannosidase (mannosidas	gALL v3 c698 4	68 kDa		unknown	0	0.62757	0	89%
1306	TRUE	FALSE	AGAP001319-PA [Anopheles gambiae str. PEST]	gALL v3 c7177 2	33 kDa		unknown	0	0,62757	0	89%
1307	TRUE	FALSE	PREDICTED: similar to exosome complex exonuclease RRP44 [Trib	gALL v3 c7434 4	33 kDa		unknown	0	0.62757	0	89%
1308	TRUE	FALSE	yellow-x1c [Nasonia vitripennis]	gALL v3 c8603 5	25 kDa		unknown	0	0,62757	0	89%
1309	TRUE	FALSE	p450 [Reticulitermes flavipes]	gALL v3 rep c104501 6cytochro	?		unknown	0	0.62757	0	89%
1310	TRUE	FALSE	similar to BEL12 AG transposon polyprotein [Acyrthosiphon pisum	gALL v3 rep c105065 5PREDICT	?		unknown	0	0,62757	0	89%
1311	TRUE	FALSE	hexamerin II [Reticulitermes flavipes]	gALL v3 rep c96146 2	60 kDa	FALSE	unknown	0	11,924	0	89%
1312	TRUE	FALSE	paramyosin, long form, putative [Pediculus humanus corporis]	gALL v3 rep c96150 5	139 kDa		unknown	0	0,62757	0	89%
1313	TRUE	FALSE	alpha-amylase [Blattella germanica]	gALL v3 rep c96185 6 (+1)	61 kDa	TRUE	unknown	0	1,2551	0	89%
1314	TRUE	FALSE	cytochrome p450 [Reticulitermes flavipes]	gALL v3 rep c96280 1 (+3)	111 kDa		unknown	0	0,62757	0	89%
1315	TRUE	FALSE	PREDICTED: similar to CG14762-PA [Apis mellifera]	gALL v3 rep c96661 5	143 kDa		unknown	0	0,62757	0	89%
1316	TRUE	FALSE	tubulin beta-2 chain, putative [Pediculus humanus corporis]	gALL v3 rep c96802 4	32 kDa	TRUE	unknown	0	1,2551	0	89%
1317	TRUE	FALSE	angiotensin-converting enzyme 9 (AGAP004563-PA) [Anopheles ga	gALL v3 rep c97339 6	56 kDa	TRUE	unknown	0	0,62757	0	89%
1318	TRUE	FALSE	PREDICTED: similar to CG10375-PA isoform 1 [Apis mellifera]	gALL v3 c51200 2	13 kDa		unknown	0,89028	0	89%	0
1319	TRUE	FALSE	PREDICTED: similar to Nascent polypeptide associated complex pr	gALL v3 c12152 5 (+1)	26 kDa		unknown	0,89028	0	89%	0
1320	TRUE	FALSE	PREDICTED: similar to CG6084 CG6084-PA [Tribolium castaneum]	gALL v3 c25710 1	27 kDa		unknown	1.371	0	89%	0
1321	TRUE	FALSE	PREDICTED: hypothetical protein [Nasonia vitripennis]	gALL v3 c3300 5	25 kDa		unknown	0	1,0261	0	89%
1322	TRUE	FALSE	PREDICTED: similar to GTP-binding protein [Nasonia vitripennis]	gALL v3 c82486 2	26 kDa		unknown	0.89028	0	89%	0
1323	TRUE	FALSE	AGAP005400-PA [Anopheles gambiae str. PEST]	gALL v3 rep c96259 2 (+1)	131 kDa		unknown	0,89028	0	89%	0
1324	TRUE	FALSE	RecName: Full=Putative tyramine receptor 2; AltName: Full=Tyr-L	gALL v3 c17883 5	21 kDa		unknown	0	1.0261	0	89%
1325	TRUE	FALSE	PREDICTED: similar to AGAP012407-PA [Tribolium castaneum]	gALL v3 rep c96565 5	79 kDa		unknown	0	1,0261	0	89%
1326	TRUE	FALSE	PREDICTED: similar to Contactin CG1084-PA [Apis mellifera]	gALL v3 rep c98977 5	12 kDa		unknown	1.6054	0	88%	0
1327	TRUE	FALSE	Annexin-B10, putative [Pediculus humanus corporis]	gALL v3 c25156 5	27 kDa		unknown	1,1388	0	88%	0
1328	TRUE	FALSE	kurtz [Tribolium castaneum]	gALL v3 c3578 6	44 kDa		unknown	0.99733	0	88%	0
1329	TRUE	FALSE	PREDICTED: similar to CG31100-PA [Apis mellifera]	gALL v3 c16144 2	29 kDa		unknown	0	0,88083	0	88%
1330	TRUE	FALSE	PREDICTED: synaptotagmin IX-like, partial [Saccoglossus kowalev	gALL v3 c11786 5 (+2)	32 kDa		unknown	1.944	0	88%	0
1331	TRUE	FALSE	PREDICTED: similar to ENSANGP00000020237 [Nasonia vitripenni	gALL v3 c66710 1	16 kDa		unknown	1,371	0	88%	0
1332	TRUE	FALSE	PREDICTED: similar to RNA binding motif protein 4 [Apis mellifera]	gALL v3 c54233 2	40 kDa		unknown	0	1.8851	0	88%
1333	TRUE	FALSE	PREDICTED: similar to syndecan binding protein [Tribolium castan	gALL v3 c4915 3	83 kDa		unknown	0	0,68834	0	88%
1334	TRUE	FALSE	unnamed protein product [Homo sapiens]	gALL v3 c16259 1	23 kDa		unknown	0	0.68834	0	88%
1335	TRUE	FALSE	PREDICTED: similar to dehydrogenase [Nasonia vitripennis]	gALL v3 c4274 4	73 kDa		unknown	0	0,69313	0	88%
1336	TRUE	FALSE	conserved hypothetical protein [Pediculus humanus corporis]	gALL v3 c2092 6	47 kDa		unknown	1.371	0	88%	0
1337	TRUE	FALSE	hypothetical protein AaeL AAEL011344 [Aedes aegypti]	gALL v3 c27244 6	29 kDa		unknown	0,99733	0	88%	0
1338	TRUE	FALSE	PREDICTED: similar to Ribosomal protein L6 CG11522-PB, isoform	gALL v3 c18738 4 (+1)	16 kDa		unknown	0,89028	0	88%	0
1339	TRUE	FALSE	hypothetical protein BRAFLDRAFT 128102 [Branchiostoma floridae	gALL v3 c8528 3	36 kDa		unknown	1,371	0	88%	0
1340	TRUE	FALSE	hypothetical protein HM1 3149 [Heliobacterium modesticaldum Ic	gALL v3 c53291 5	17 kDa		unknown	1,6054	0	88%	0
1341	TRUE	FALSE	PREDICTED: hypothetical protein LOC100496354 [Xenopus (Silura	gALL v3 c43937 6	16 kDa		unknown	1,944	0	88%	0
1342	TRUE	FALSE	PREDICTED: similar to glycinamide ribonucleotide synthetase-amir	gALL v3 c1214 3	99 kDa		unknown	0	0,68834	0	87%
1343	TRUE	FALSE	protein TcasGA2 TC001390 [Tribolium castaneum]	gALL v3 rep c105075 2hypothet	?		unknown	0,99733	0	87%	0
1344	TRUE	FALSE	enzymatic polyprotein; Endonuclease; Reverse transcriptase, putat	gALL v3 c5091 1 (+1)	88 kDa		unknown	0	1,8851	0	87%
1345	TRUE	FALSE	Ankyrin repeat domain protein [Wolbachia endosymbiont of Culex	gALL v3 c45126 4	14 kDa		unknown	0,89028	0	87%	0
1346	TRUE	FALSE	ribosomal protein [Trichomonas vaginalis G3]	gALL v3 c20680 4	17 kDa		unknown	0	0,69313	0	87%
1347	TRUE	FALSE	endonuclease-reverse transcriptase [Bombyx mori]	gALL v3 c45296 6	15 kDa		unknown	1.1388	0	87%	0
1348	TRUE	FALSE	PREDICTED: similar to protein phosphatase 2A, regulatory subunit	gALL v3 c15219 4	24 kDa		unknown	0,89028	0	87%	0
1349	TRUE	FALSE	hypothetical protein TcasGA2 TC014157 [Tribolium castaneum]	gALL v3 rep c96813 5	43 kDa		unknown	0	0.68834	0	87%
1350	TRUE	FALSE	PREDICTED: similar to GA17312-PA [Tribolium castaneum]	gALL v3 c13265 3	25 kDa		unknown	0	0,62757	0	87%
1351	TRUE	FALSE	PREDICTED: similar to short-chain dehydrogenase [Nasonia vitripe	gALL v3 c8655 5	22 kDa		unknown	0	0.68834	0	87%
1352	TRUE	FALSE	PREDICTED: multiple PDZ domain protein-like [Saccoglossus kowa	gALL v3 c53610 4	16 kDa		unknown	1,944	0	87%	0
1353	TRUE	FALSE	[Thermofilum pendens Hrk 5]	gALL v3 rep c102156 4ankyrin	?		unknown	1.6054	0	87%	0
1354	TRUE	FALSE	PREDICTED: hypothetical protein [Saccoglossus kowalevskii]	gALL v3 c15898 3	22 kDa		unknown	0	1,0261	0	87%
1355	TRUE	FALSE	hypothetical protein TcasGA2 TC006945 [Tribolium castaneum]	gALL v3 c21523 4	19 kDa		unknown	0.99733	0	87%	0
1356	TRUE	FALSE	ubiquitin ligase sia-1, putative [Pediculus humanus corporis]	gALL v3 c60871 3	13 kDa		unknown	0	1,0261	0	87%
1357	TRUE	FALSE	regulator of G-protein signaling 19 [Danio rerio]	gALL v3 c72181 2	16 kDa		unknown	0	1.8851	0	87%
1358	TRUE	FALSE	beta-glucosidase [Coptotermes formosanus]	gALL v3 c53472 2	18 kDa		unknown	1,944	0	87%	0
1359	TRUE	FALSE	PREDICTED: similar to CG12325-PA [Apis mellifera]	gALL v3 c4441 3	72 kDa		unknown	0	0.69313	0	87%
1360	TRUE	FALSE	GTP-binding protein hflx [Aedes aegypti]	gALL v3 c41596 5	13 kDa		unknown	0	0,88083	0	86%
1361	TRUE	FALSE	PREDICTED: hypothetical protein [Nasonia vitripennis]	gALL v3 c819 6 (+1)	61 kDa		unknown	0	0.69313	0	86%
1362	TRUE	FALSE	hypothetical protein TcasGA2 TC000811 [Tribolium castaneum]	gALL v3 c8913 4	27 kDa		unknown	1,1388	0	86%	0
1363	TRUE	FALSE	PREDICTED: similar to CG32549-PD, isoform D isoform 1 [Apis me	gALL v3 c981 3	100 kDa		unknown	1.944	0	86%	0
1364	TRUE	FALSE	PREDICTED: similar to MGC52894 protein [Nasonia vitripennis]	gALL v3 c3017 3	37 kDa		unknown	1,371	0	86%	0
1365	TRUE	FALSE	PREDICTED: similar to myosin vii [Tribolium castaneum]	gALL v3 rep c98091 6	168 kDa		unknown	0,89028	0	86%	0
1366	TRUE	FALSE	AGAP006096-PA [Anopheles gambiae str. PEST]	gALL v3 c51805 3	14 kDa		unknown	0,99733	0	86%	0
1367	TRUE	FALSE	phospholipid-translocating P-type ATPase, flippase family protein [	gALL v3 c89359 3	21 kDa		unknown	1,944	0	86%	0
1368	TRUE	FALSE	PREDICTED: similar to F57G4.9 [Acyrthosiphon pisum]	gALL v3 c8162 6	29 kDa		unknown	1,6054	0	86%	0
1369	TRUE	FALSE	PREDICTED: similar to SIFamide receptor [Tribolium castaneum]	gALL v3 c103532 4	27 kDa		unknown	0	0,62757	0	86%
1370	TRUE	FALSE	PREDICTED: similar to seizure CG3182-PA, isoform A [Apis mellife	gALL v3 c12591 3	57 kDa		unknown	0,89028	0	86%	0
1371	TRUE	FALSE	PREDICTED: similar to AGAP003869-PA [Tribolium castaneum]	gALL v3 c10778 3	34 kDa		unknown	0	0,62757	0	86%
1372	TRUE	FALSE	CG13618 [Drosophila melanogaster]	gALL v3 c121 5	72 kDa		unknown	1,1388	0	86%	0
1373	TRUE	FALSE	conserved hypothetical protein [Pediculus humanus corporis]	gALL v3 c94679 6	19 kDa		unknown	0,89028	0	86%	0
1374	TRUE	FALSE	hypothetical protein [Nylanderia nr. pubens LZ-2010]	gALL v3 c31115 3	14 kDa		unknown	0.89028	0	85%	0
1375	TRUE	FALSE	PREDICTED: similar to l(2)01289 long form [Nasonia vitripennis]	gALL v3 c40438 1 (+1)	30 kDa		unknown	1,371	0	85%	0
1376	TRUE	FALSE	PREDICTED: similar to F57G4.9, partial [Acyrthosiphon pisum]	gALL v3 c64571 6	16 kDa		unknown	1.944	0	85%	0
1377	TRUE	FALSE	PREDICTED: similar to Myosin-2 essential light chain (Myosin II es	gALL v3 rep c96735 6	21 kDa		unknown	1,944	0	85%	0
1378	TRUE	FALSE	PREDICTED: similar to coatomer delta subunit [Tribolium castaneu	gALL v3 c945 2	40 kDa		unknown	0	1.8851	0	85%
1379	TRUE	FALSE	SEC14, putative [Aedes aegypti]	gALL v3 c2704 5	26 kDa		unknown	0	0,88083	0	85%
1380	TRUE	FALSE	hypothetical protein [Trichomonas vaginalis G3]	gALL v3 c43767 6	14 kDa		unknown	0	1.0261	0	85%
1381	TRUE	FALSE	hypothetical protein TcasGA2 TC005876 [Tribolium castaneum]	gALL v3 c9714 5	19 kDa		unknown	1,371	0	85%	0
1382	TRUE	FALSE	PREDICTED: similar to dihydropyrimidine dehydrogenase isoform 1	gALL v3 c7499 5	38 kDa		unknown	0	0.62757	0	85%
1383	TRUE	FALSE	Cytidine deaminase, putative [Pediculus humanus corporis]	gALL v3 c4488 5	46 kDa		unknown	0	1,8851	0	85%
1384	TRUE	FALSE	Abhydrolase domain-containing protein 14B [Lepeophtheirus salm	gALL v3 c110 4	28 kDa		unknown	0	0.88083	0	85%
1385	TRUE	FALSE	PREDICTED: similar to Angiopoietin-1 receptor precursor (Tyrosine	gALL v3 c22509 6 (+1)	29 kDa		unknown	0	0,68834	0	85%
1386	TRUE	FALSE	PREDICTED: similar to karst CG12008-PA, isoform A [Apis mellifer	gALL v3 c1982 1 (+2)	66 kDa		unknown	0	0.62757	0	85%
1387	TRUE	FALSE	PREDICTED: similar to 1-phosphatidylinositol-4-phosphate 5-kinas	gALL v3 c63009 2	14 kDa		unknown	1,944	0	85%	0
1388	TRUE	FALSE	PREDICTED: similar to p38b CG7393-PA [Apis mellifera]	gALL v3 c2777 1	48 kDa		unknown	0	0.69313	0	85%
1389	TRUE	FALSE	hypothetical protein TcasGA2 TC005054 [Tribolium castaneum]	gALL v3 c2435 5 (+3)	35 kDa		unknown	0	0,88083	0	85%
1390	TRUE	FALSE	PREDICTED: similar to protein disulfide isomerase [Tribolium casta	gALL v3 c11601 4	39 kDa		unknown	0	0.88083	0	84%
1391	TRUE	FALSE	PREDICTED: similar to zipper CG15792-PD, isoform D [Apis mellife	gALL v3 c12235 6	32 kDa		unknown	0	0,68834	0	84%
1392	TRUE	FALSE	PREDICTED: similar to AGAP005729-PA [Acyrthosiphon pisum]	gALL v3 c72096 4	20 kDa		unknown	1,371	0	84%	0
1393	TRUE	FALSE	conserved hypothetical protein [Pediculus humanus corporis]	gALL v3 c72990 5	12 kDa		unknown	0	0,69313	0	84%
1394	TRUE	FALSE	AGAP011352-PA [Anopheles gambiae str. PEST]	gALL v3 c22770 4 (+1)	16 kDa		unknown	0	0,69313	0	84%
1395	TRUE	FALSE	PREDICTED: similar to conserved hypothetical protein [Nasonia vit	gALL v3 c7115 4	29 kDa		unknown	1,1388	0	84%	0
1396	TRUE	FALSE	PREDICTED: similar to sulfotransferase (sult) [Nasonia vitripennis]	gALL v3 c40521 1 (+1)	17 kDa		unknown	0,89028	0	84%	0
1397	TRUE	FALSE	traf2 and nck interacting kinase, tnik, putative [Pediculus humanus	gALL v3 c50363 6	17 kDa		unknown	0	0,62757	0	84%
1398	TRUE	FALSE	neuroendocrine convertase, putative [Ixodes scapularis]	gALL v3 c6181 3	73 kDa		unknown	1,944	0	84%	0
1399	TRUE	FALSE	ORF MSV061 putative LINE reverse transcriptase, similar to Caeno	gALL v3 c52989 4 (+1)	12 kDa		unknown	0	0,68834	0	84%
1400	TRUE	FALSE	PREDICTED: similar to AGAP009560-PA, partial [Tribolium castane	gALL v3 c11757 1	46 kDa		unknown	1,371	0	84%	0
1401	TRUE	FALSE	PREDICTED: similar to CG15831-PA.3 [Apis mellifera]	gALL v3 rep c96796 3	396 kDa		unknown	1.1388	0	84%	0
1402	TRUE	FALSE	PREDICTED: similar to GA15161-PA [Nasonia vitripennis]	gALL v3 c3444 2	44 kDa		unknown	0,89028	0	84%	0
1403	TRUE	FALSE	AGAP007942-PA [Anopheles gambiae str. PEST]	gALL v3 c3766 6	41 kDa		unknown	0.99733	0	83%	0
1404	TRUE	FALSE	iron-responsive element-binding protein 1 [Culex quinquefasciatus	gALL v3 c3602 6	42 kDa		unknown	0	0,68834	0	83%
1405	TRUE	FALSE	protein SET, putative [Pediculus humanus corporis]	gALL v3 c7882 3	40 kDa		unknown	0.89028	0	83%	0
1406	TRUE	FALSE	hypothetical protein Phum PHUM387840 [Pediculus humanus corp	gALL v3 c1461 5	55 kDa		unknown	1,1388	0	83%	0
1407	TRUE	FALSE	PREDICTED: similar to cathepsin D isoform 1 [Tribolium castaneun	gALL v3 rep c96895 4 (+1)	80 kDa		unknown	0.89028	0	83%	0
1408	TRUE	FALSE	RecName: Full=Lachesin; Flags: Precursor	gALL v3 c17730 1	34 kDa		unknown	0	0,69313	0	83%
1409	TRUE	FALSE	class D atypical G-protein coupled receptor GPRstn1, putative [Pec	gALL v3 c29950 3	15 kDa		unknown	0	0.62757	0	82%
1410	TRUE	FALSE	PREDICTED: similar to Chromosome 3 open reading frame 31 [Orr	gALL v3 c71051 1	25 kDa		unknown	0,89028	0	82%	0
1411	TRUE	FALSE	PREDICTED: similar to dipeptidyl peptidase iii [Nasonia vitripennis	gALL v3 c8230 6	27 kDa		unknown	0.99733	0	82%	0
1412	TRUE	FALSE	aldose-1-epimerase [Aedes aegypti]	gALL v3 c27367 6	20 kDa		unknown	0	0,62757	0	82%
1413	TRUE	FALSE	p10 [Periplaneta americana]	gALL v3 rep c96327 3	42 kDa		unknown	0	0.62757	0	82%
1414	TRUE	FALSE	PREDICTED: similar to CG7888-PB, isoform B [Apis mellifera]	gALL v3 c34867 3	22 kDa		unknown	0	0,62757	0	82%
1415	TRUE	FALSE	malate dehydrogenase [Culex quinquefasciatus]	gALL v3 c8327 6 (+1)	20 kDa		unknown	0.89028	0	82%	0
1416	TRUE	FALSE	GH23483p [Drosophila melanogaster]	gALL v3 c25859 6	20 kDa		unknown	0,89028	0	82%	0
1417	TRUE	FALSE	PREDICTED: similar to CG30084-PC, isoform C [Apis mellifera]	gALL v3 rep c96284 6	179 kDa		unknown	0	1.0261	0	82%
1418	TRUE	FALSE	cytochrome P-450, putative [Pediculus humanus corporis]	gALL v3 c2488 6 (+3)	51 kDa		unknown	0	0,62757	0	81%

1419	TRUE	FALSE	juvenile hormone esterase-like protein Est1 [Reticulitermes flavipe	gALL v3 c2097 4	57 kDa	unknown	0	0.62757	0	81%
1420	TRUE	FALSE	PREDICTED: similar to Zinc finger protein 84 (Zinc finger protein H	gALL v3 c21505 1	27 kDa	unknown	0	0.62757	0	81%
