# Supplementary material for: Expanding the Knowledge on Lignocellulolytic and Redox Enzymes of Worker and Soldier Castes from the Lower Termite Coptotermes gestroi
Source: Front Microbiol. 2016 Oct 13;7:1518. doi: 10.3389/fmicb.2016.01518 (PMC5061848; doi:10.3389/fmicb.2016.01518)
Supplement: Table S1 — Total unigenes identified in worker and soldier castes of Coptotermes gestroi. [file Table1.PDF]

Contigs / Unigenes	Worker reads	Soldier reads	CDD ID	CDD description	NCBI/GI	NCBI description	Blast E-value
gALL_v3_c10004	1	10	-	-	ref|XP_003704575.1|	PREDICTED: histone deacetylase complex subunit SAP30 homolog [Megachile rotundata]	7,00E-39
gALL_v3_c1001	1	11	-	-	ref|XP_004932392.1|	PREDICTED: protein bunched, class 2/F/G isoform-like [Bombyx mori]	1,00E-48
gALL_v3_c10022	2	12	-	-	ref|XP_003727690.1|	PREDICTED: RNA-directed DNA polymerase from mobile element jockey-like [Strongylocentrotus purpuratus]	7,00E-23
gALL_v3_c1005	3	14	gnl|CDD|190521	pfam03096, Ndr, Ndr family. T	ref|XP_003708036.1|	PREDICTED: protein NDRG3-like [Megachile rotundata]	1,00E-164
gALL_v3_c10074	1	11	-	-	-	-	-
gALL_v3_c10115	1	9	-	-	-	-	-
gALL_v3_c1015	3	58	gnl|CDD|30099	cd01377, MYSc_type_II, Myos	ref|XP_001813596.1|	PREDICTED: similar to Myosin heavy chain CG17927-PF isoform 3 [Tribolium castaneum]	5,00E-17
gALL_v3_c10204	1	10	-	-	gb|EFN72974.1|	Protein ariadne-2 [Camponotus floridanus]	1,00E-142
gALL_v3_c10210	2	17	-	-	gb|EFA08290.1|	hypothetical protein TcasGA2_TC005924 [Tribolium castaneum]	1,00E-10
gALL_v3_c10276	4	16	gnl|CDD|30099	cd01377, MYSc_type_II, Myos	gb|AAM97639.1|	myosin heavy chain type II [Blatta sp. IRT-2002]	1,00E-32
gALL_v3_c10278	2	13	-	-	-	-	-
gALL_v3_c10287	2	14	gnl|CDD|189625	pfam00609, DAGK_acc, Diacyl	ref|XP_003425967.1|	PREDICTED: diacylglycerol kinase theta-like isoform 2 [Nasonia vitripennis]	1,00E-125
gALL_v3_c10317	3	24	-	-	-	-	-
gALL_v3_c10320	2	12	-	-	ref|XP_001951951.2|	PREDICTED: hypothetical protein LOC100164298 [Acyrthosiphon pisum]	1,00E-42
gALL_v3_c10363	3	18	-	-	ref|XP_006569104.1|	PREDICTED: ryanodine receptor 44F isoform X19 [Apis mellifera]	1,00E-133
gALL_v3_c10371	5	21	-	-	ref|XP_005754389.1|	PREDICTED: crossover junction endonuclease MUS81-like [Pundamilia nyererei]	1,00E-08
gALL_v3_c10417	1	14	-	-	-	-	-
gALL_v3_c10418	6	23	gnl|CDD|185473	PTZ00139, PTZ00139, Succinat	gb|AGN90964.1|	succinate dehydrogenase, partial [Diploptera punctata]	0
gALL_v3_c10438	4	15	-	-	-	-	-
gALL_v3_c10493	1	9	-	-	-	-	-
gALL_v3_c10503	1	9	-	-	gb|EGI70510.1|	Putative stoned B-like protein [Acromyrmex echinatior]	2,00E-12
gALL_v3_c10536	8	25	-	-	gb|EFN67634.1|	Putative uncharacterized transposon-derived protein F52C9.6 [Camponotus floridanus]	4,00E-22
gALL_v3_c10547	2	12	gnl|CDD|189741	pfam00850, Hist_deacetyl, His	ref|XP_970656.1|	PREDICTED: similar to Histone deacetylase 11 (HD11) [Tribolium castaneum] gb|EFA01868.1| hypothetical protein TcasGA2_TC007473 [Tribolium castaneum]	1,00E-111
gALL_v3_c10566	2	14	-	-	-	-	-
gALL_v3_c10574	10	32	-	-	-	-	-
gALL_v3_c10605	1	9	gnl|CDD|153255	cd07993, LPLAT_DHAPAT-like,	ref|XP_967684.1|	PREDICTED: similar to glycerol-3-phosphate acyltransferase [Tribolium castaneum] gb|EFA09975.1| hypothetical protein TcasGA2_TC012134 [Tribolium castaneum]	8,00E-64
gALL_v3_c10640	2	16	-	-	-	-	-
gALL_v3_c10711	2	13	-	-	-	-	-
gALL_v3_c10743	5	20	gnl|CDD|150921	pfam10325, 7TM_GPCR_Srz, S	-	-	-
gALL_v3_c10758	3	16	-	-	gb|EFN85870.1|	Xanthine dehydrogenase/oxidase [Harpegnathos saltator]	4,00E-10
gALL_v3_c1082	13	43	gnl|CDD|148205	pfam06459, RR_TM4-6, Ryanc	gb|EEZ99829.1|	hypothetical protein TcasGA2_TC002610 [Tribolium castaneum]	0
gALL_v3_c10872	4	15	gnl|CDD|34727	COG5126, FRQ1, Ca2+-binding	ref|XP_001604721.2|	PREDICTED: calcineurin subunit B type 2-like isoform 1 [Nasonia vitripennis]	6,00E-84
gALL_v3_c10884	1	10	-	-	-	-	-
gALL_v3_c10917	8	22	-	-	-	-	-
gALL_v3_c10949	3	13	-	-	-	-	-
gALL_v3_c11025	4	16	-	-	-	-	-
gALL_v3_c11026	1	9	-	-	-	-	-
gALL_v3_c11056	6	19	-	-	gb|ENN70519.1|	hypothetical protein YQE_12695, partial [Dendroctonus ponderosae]	3,00E-51
gALL_v3_c11073	3	14	-	-	-	-	-
gALL_v3_c11123	1	12	-	-	-	-	-
gALL_v3_c1113	6	20	-	-	gb|AGM32631.1|	EF-hand domain containing protein [Coptotermes formosanus]	1,00E-104
gALL_v3_c1114	2	12	gnl|CDD|128516	smart00220, S_TKc, Serine/Th	gb|ADX05541.1|	calcium/calmodulin-dependent protein kinase II isoform A [Periplaneta americana]	1,00E-134
gALL_v3_c11159	1	10	-	-	-	-	-
gALL_v3_c11174	1	10	-	-	-	-	-
gALL_v3_c11193	4	15	gnl|CDD|187698	cd09274, RNase_HI_RT_Ty3, T	ref|XP_001810599.1|	PREDICTED: similar to pol polyprotein [Tribolium castaneum]	4,00E-83
gALL_v3_c11206	4	41	gnl|CDD|191716	pfam07263, DMP1, Dentin ma	-	-	-
gALL_v3_c1123	14	43	gnl|CDD|189450	pfam00209, SNF, Sodium:neur	ref|XP_003703775.1|	PREDICTED: sodium- and chloride-dependent GABA transporter 1-like [Megachile rotundata]	0
gALL_v3_c11251	11	33	-	-	gb|AGM32403.1|	myosin light chain [Coptotermes formosanus]	1,00E-23
gALL_v3_c11313	5	19	gnl|CDD|189934	pfam01302, CAP_GLY, CAP-Gly	ref|XP_002427043.1|	retiin, putative [Pediculus humanus corporis] gb|EEB14305.1| retiin, putative [Pediculus humanus corporis]	7,00E-84
gALL_v3_c1135	9	41	-	-	ref|XP_973823.1|	PREDICTED: similar to conserved hypothetical protein [Tribolium castaneum] gb|EFA07415.1| brain peptide ITGQGNRIF-like protein [Tribolium castaneum]	8,00E-90
gALL_v3_c11432	2	11	gnl|CDD|128654	smart00360, RRM, RNA recogi	gb|ACZ52465.1|	Bruno-3 transcript variant 33 [Drosophila persimilis]	1,00E-58
gALL_v3_c1144	10	29	-	-	-	-	-
gALL_v3_c1149	8	26	gnl|CDD|30087	cd01365, KISc_KIF1A_KIF1B, K	ref|XP_006566337.1|	PREDICTED: kinesin 3B isoform X7 [Apis mellifera]	0
gALL_v3_c11499	2	11	-	-	-	-	-
gALL_v3_c1155	3	26	-	-	-	-	-
gALL_v3_c1157	1	9	-	-	-	-	-
gALL_v3_c11581	2	12	gnl|CDD|130706	TIGR01645, half-pint, poly-U b	ref|XP_006563577.1|	PREDICTED: poly(U)-binding-splicing factor half pint isoform X2 [Apis mellifera]	2,00E-46
gALL_v3_c11629	1	9	-	-	gb|EFN65619.1|	Putative uncharacterized transposon-derived protein F52C9.6 [Camponotus floridanus]	4,00E-06
gALL_v3_c11656	5	18	-	-	-	-	-
gALL_v3_c1168	8	37	gnl|CDD|191810	pfam07679, I-set, Immunoglol	ref|XP_003493149.1|	PREDICTED: hypothetical protein LOC100743398 [Bombus impatiens]	0
gALL_v3_c11684	3	13	gnl|CDD|193335	pfam12861, zf-Apc11, Anapha	ref|XP_003706224.1|	PREDICTED: anaphase-promoting complex subunit 11-like [Megachile rotundata]	4,00E-56
gALL_v3_c11716	5	19	gnl|CDD|147406	pfam05197, TRIC, TRIC channe	gb|EFZ13739.1|	hypothetical protein SINV_10016 [Solenopsis invicta]	7,00E-67
gALL_v3_c11737	7	24	-	-	ref|XP_003706954.1|	PREDICTED: uncharacterized protein LOC100880656 [Megachile rotundata]	0
gALL_v3_c11746	9	29	-	-	-	-	-
gALL_v3_c11817	1	9	gnl|CDD|30539	COG0190, FolD, 5,10-methyler	ref|XP_003700207.1|	PREDICTED: c-1-tetrahydrofolate synthase, cytoplasmic-like [Megachile rotundata]	8,00E-68
gALL_v3_c11824	10	28	-	-	gb|EFA13518.1|	hypothetical protein TcasGA2_TC001491 [Tribolium castaneum]	3,00E-27
gALL_v3_c11869	3	13	-	-	-	-	-
gALL_v3_c11882	1	12	-	-	-	-	-
gALL_v3_c11886	4	15	gnl|CDD|73154	cd01647, RT_LTR, RT_LTR: Rev	ref|XP_005172038.1|	PREDICTED: uncharacterized protein LOC101886116 isoform X1 [Danio rerio] ref|XP_005172039.1| PREDICTED: uncharacterized protein LOC101886116 isoform X2 [Danio	1,00E-35
gALL_v3_c11924	1	9	gnl|CDD|190537	pfam03137, OATP, Organic Ani	ref|XP_003695688.1|	PREDICTED: LOW QUALITY PROTEIN: solute carrier organic anion transporter family member 4A1-like [Apis florea]	1,00E-125
gALL_v3_c1194	5	17	gnl|CDD|189661	pfam00665, rve, Integrase cor	gb|ABD72267.1|	pol polyprotein [Drosophila buzzatii]	6,00E-41
gALL_v3_c11979	5	30	-	-	gb|EHJ71649.1|	hypothetical protein KGM_08503 [Danaus plexippus]	3,00E-24
gALL_v3_c1200	5	19	gnl|CDD|144648	pfam01130, CD36, CD36 famil	ref|XP_002432097.1|	scavenger receptor class B member, putative [Pediculus humanus corporis] gb|EEB19359.1| scavenger receptor class B member, putative [Pediculus humanus corporis]	1,00E-166
gALL_v3_c1201	5	22	-	-	-	-	-
gALL_v3_c1207	6	20	-	-	ref|XP_003426017.1|	PREDICTED: hypothetical protein LOC100678081 [Nasonia vitripennis]	3,00E-57
gALL_v3_c12165	2	13	-	-	dbj|BAN20631.1|	calsyntenin-1 precursor [Riptortus pedestris]	1,00E-136
gALL_v3_c12199	1	12	-	-	-	-	-
gALL_v3_c12205	2	12	-	-	gb|ADI61823.1|	endonuclease-reverse transcriptase [Bombyx mori]	6,00E-38
gALL_v3_c12216	1	9	-	-	-	-	-
gALL_v3_c12244	3	22	-	-	-	-	-
gALL_v3_c12261	3	16	gnl|CDD|189372	pfam00067, p450, Cytochrom	gb|AAC03111.2|	family 4 cytochrome P450 [Coptotermes acinaciformis]	1,00E-19
gALL_v3_c12276	2	12	-	-	-	-	-
gALL_v3_c12293	1	9	-	-	gb|ERL91443.1|	hypothetical protein D910_08773 [Dendroctonus ponderosae]	5,00E-56
gALL_v3_c12399	8	25	gnl|CDD|189008	cd09601, M1_APN_2, Peptida	ref|XP_001608209.2|	PREDICTED: aminopeptidase N-like [Nasonia vitripennis]	1,00E-150
gALL_v3_c1244	8	28	-	-	gb|EFA01045.1|	hypothetical protein TcasGA2_TC003961 [Tribolium castaneum]	1,00E-125
gALL_v3_c12444	1	9	-	-	-	-	-
gALL_v3_c12510	1	12	gnl|CDD|189378	pfam00079, Serpin, Serpin (se	ref|XP_002424080.1|	serine protease inhibitor, putative [Pediculus humanus corporis] gb|EEB11342.1| serine protease inhibitor, putative [Pediculus humanus corporis]	6,00E-43
gALL_v3_c1255	4	41	-	-	gb|EFN74640.1|	Myosin heavy chain, muscle [Camponotus floridanus]	2,00E-06
gALL_v3_c1265	5	21	-	-	-	-	-
gALL_v3_c12677	2	16	-	-	ref|XP_001809336.1|	PREDICTED: similar to Ank2 CG34416-PI [Tribolium castaneum]	7,00E-15
gALL_v3_c1270	1	33	gnl|CDD|188171	TIGR01833, HMG-CoA-S_euk,	dbj|BAM38487.2|	3-hydroxy-3-methylglutaryl coenzyme A synthase [Nasutitermes takasagoensis]	1,00E-47
gALL_v3_c12719	2	12	-	-	-	-	-
gALL_v3_c12721	1	12	gnl|CDD|189372	pfam00067, p450, Cytochrom	gb|ABB86762.2|	CYP4U3v1 [Reticulitermes flavipes]	1,00E-107
gALL_v3_c12733	2	14	-	-	-	-	-
gALL_v3_c12763	2	25	-	-	ref|NP_001037478.1|	p270 [Bombyx mori] gb|AAB53258.1| p270 [Bombyx mori]	1,00E-116
gALL_v3_c1279	7	27	-	-	gb|ERL95180.1|	hypothetical protein D910_12448, partial [Dendroctonus ponderosae]	1,00E-110
gALL_v3_c128	14	35	-	-	-	-	-
gALL_v3_c12832	2	11	gnl|CDD|190517	pfam03074, GCS, Glutamate-c	ref|XP_966349.1|	PREDICTED: similar to glutamate cysteine ligase isoform 1 [Tribolium castaneum] gb|EFA04528.1| hypothetical protein TcasGA2_TC016323 [Tribolium castaneum]	1,00E-138
gALL_v3_c12845	3	16	-	-	-	-	-
gALL_v3_c12847	1	12	-	-	ref|XP_312290.4|	AGAP002638-PA [Anopheles gambiae str. PEST] gb|EAA08206.4| AGAP002638-PA [Anopheles gambiae str. PEST]	7,00E-61
gALL_v3_c1287	9	35	gnl|CDD|188655	cd09823, peroxinectin_like, pe	gb|EFN68225.1|	Peroxidase [Camponotus floridanus]	1,00E-155
gALL_v3_c12878	1	10	-	-	ref|XP_003636096.1|	hypothetical protein MTR_027s0011 [Medicago truncatula] gb|AES83234.1| hypothetical protein MTR_027s0011 [Medicago truncatula]	6,00E-07
gALL_v3_c12888	3	16	gnl|CDD|148286	pfam06585, JHBP, Haemolymp	gb|AGM32527.1|	hemolymph juvenile hormone binding protein (JHBP) [Coptotermes formosanus]	1,00E-127
gALL_v3_c12909	1	9	-	-	-	-	-
gALL_v3_c12912	1	10	gnl|CDD|173789	cd04059, Peptidases_S8_Prot	ref|XP_972593.1|	PREDICTED: similar to AGAP002176-PA [Tribolium castaneum]	1,00E-127
gALL_v3_c13024	1	9	-	-	-	-	-
gALL_v3_c13094	1	12	-	-	-	-	-
gALL_v3_c1316	5	35	gnl|CDD|179368	PRK02106, PRK02106, choline	ref|XP_972225.1|	PREDICTED: similar to AGAP003785-PA [Tribolium castaneum] gb|EFA05527.1| hypothetical protein TcasGA2_TC015714 [Tribolium castaneum]	1,00E-153
gALL_v3_c13179	2	11	-	-	-	-	-
gALL_v3_c1319	1	9	-	-	-	-	-
gALL_v3_c13202	2	12	-	-	-	-	-
gALL_v3_c1323	7	22	gnl|CDD|143864	pfam00083, Sugar_tr, Sugar (a	gb|EGI58537.1|	Solute carrier family 2, facilitated glucose transporter member 1 [Acromyrmex echinatior]	1,00E-70
gALL_v3_c13270	7	30	-	-	-	-	-
gALL_v3_c13417	10	29	-	-	-	-	-
gALL_v3_c13437	4	15	-	-	ref|XP_974187.1|	PREDICTED: similar to vacuolar ATP synthase subunit S1 [Tribolium castaneum] gb|EFA11226.1| hypothetical protein TcasGA2_TC010758 [Tribolium castaneum]	2,00E-29
gALL_v3_c13445	3	16	-	-	-	-	-
gALL_v3_c13487	2	18	gnl|CDD|129058	smart00825, PKS_KS, Beta-ket	gb|EFZ16301.1|	hypothetical protein SINV_05241 [Solenopsis invicta]	1,00E-132
gALL_v3_c13603	2	12	-	-	gb|EFZ18046.1|	hypothetical protein SINV_01623 [Solenopsis invicta]	2,00E-18
gALL_v3_c13634	6	19	gnl|CDD|128358	smart00043, CY, Cystatin-like c	ref|XP_006638570.1|	PREDICTED: cystatin-like [Lepisosteus oculatus]	2,00E-34
gALL_v3_c13648	1	9	-	-	ref|XP_001812287.1|	PREDICTED: similar to AGAP004423-PA [Tribolium castaneum] gb|EFA09040.1| hypothetical protein TcasGA2_TC006753 [Tribolium castaneum]	1,00E-71
gALL_v3_c1375	9	26	gnl|CDD|128632	smart00338, BRLZ, basic regio	ref|XP_003397937.1|	PREDICTED: proto-oncogene c-Fos-like [Bombus terrestris] ref|XP_003484432.1| PREDICTED: proto-oncogene c-Fos-like [Bombus impatiens]	2,00E-44
gALL_v3_c1377	10	36	-	-	ref|XP_974195.1|	PREDICTED: similar to sterol regulatory element-binding protein 1 [Tribolium castaneum] gb|EFA01599.1| hypothetical protein TcasGA2_TC007163 [Tribolium castaneum]	1,00E-179
gALL_v3_c1381	9	25	gnl|CDD|191823	pfam07716, bZIP_2, Basic regi	gb|EFA06345.1|	hypothetical protein TcasGA2_TC009220 [Tribolium castaneum]	1,00E-67
gALL_v3_c1392	7	25	gnl|CDD|144648	pfam01130, CD36, CD36 famil	ref|XP_004521882.1|	PREDICTED: protein croquemort-like [Ceratitis capitata]	1,00E-136
gALL_v3_c13939	1	9	-	-	ref|XP_002422722.1|	type II transmembrane protein, putative [Pediculus humanus corporis] gb|EEB09984.1| type II transmembrane protein, putative [Pediculus humanus corporis]	1,00E-129
gALL_v3_c13948	1	12	-	-	-	-	-
gALL_v3_c13954	4	18	-	-	-	-	-
gALL_v3_c1399	12	31	gnl|CDD|148902	pfam07546, EMI, EMI domain.	ref|XP_003401527.1|	PREDICTED: multiple epidermal growth factor-like domains protein 10-like [Bombus terrestris]	0
gALL_v3_c13990	2	11	-	-	-	-	-
gALL_v3_c14013	1	10	gnl|CDD|128524	smart00228, PDZ, Domain pre	ref|XP_973222.1|	PREDICTED: similar to veli CG7662-PA [Tribolium castaneum] gb|EEZ99659.1| hypothetical protein TcasGA2_TC002416 [Tribolium castaneum]	1,00E-76
gALL_v3_c1406	14	45	gnl|CDD|165506	PHA03247, PHA03247, large te	ref|XP_002550650.1|	hypothetical protein CTRG_04948 [Candida tropicalis MYA-3404] gb|EER31218.1| hypothetical protein CTRG_04948 [Candida tropicalis MYA-3404]	1,00E-20
gALL_v3_c14064	1	14	gnl|CDD|109161	pfam00093, VWC, von Willebr	ref|XP_002429623.1|	cysteine-rich motor neuron, putative [Pediculus humanus corporis] gb|EEB16885.1| cysteine-rich motor neuron, putative [Pediculus humanus corporis]	5,00E-46
gALL_v3_c14067	6	20	gnl|CDD|128721	smart00444, GYF, Contains coi	ref|XP_003492916.1|	PREDICTED: hypothetical protein LOC100745066 [Bombus impatiens]	4,00E-75
gALL_v3_c14097	1	10	-	-	gb|ENN70339.1|	hypothetical protein YQE_12847, partial [Dendroctonus ponderosae]	4,00E-15
gALL_v3_c14155	2	14	gnl|CDD|31389	COG1196, Smc, Chromosome	gb|EFN85270.1|	hypothetical protein EAI_15707 [Harpegnathos saltator]	1,00E-26
gALL_v3_c14321	11	28	-	-	ref|XP_973767.2|	PREDICTED: similar to kakapo [Tribolium castaneum]	4,00E-65
gALL_v3_c14337	1	15	-	-	-	-	-
gALL_v3_c14368	3	15	gnl|CDD|190293	pfam02373, JmjC, JmjC domai	ref|XP_003693788.1|	PREDICTED: lysine-specific demethylase 6A-like [Apis florea]	0
gALL_v3_c14389	1	10	-	-	-	-	-
gALL_v3_c1440	2	29	-	-	ref|XP_004524756.1|	PREDICTED: myosin heavy chain, muscle-like isoform X18 [Ceratitis capitata]	8,00E-06
gALL_v3_c14463	1	18	-	-	-	-	-
gALL_v3_c1448	4	15	-	-	-	-	-
gALL_v3_c14484	12	49	gnl|CDD|188839	cd09455, LIM1_Enigma_like_1	ref|XP_006622622.1|	PREDICTED: PDZ and LIM domain protein Zasp-like isoform X4 [Apis dorsata]	1,00E-121

gALL_v3_c14517	1	9	-	-	-	-	-
gALL_v3_c14617	2	11	-	-	ref|XP_312290.4|	AGAP002638-PA [Anopheles gambiae str. PEST] gb|EAA08206.4| AGAP002638-PA [Anopheles gambiae str. PEST]	5,00E-46
gALL_v3_c1462	9	28	-	-	gb|EFA12332.1|	hypothetical protein TcasGA2_TC005160 [Tribolium castaneum]	5,00E-07
gALL_v3_c14774	4	15	-	-	gb|EFN62471.1|	Bullous pemphigoid antigen 1, isoforms 6/9/10 [Camponotus floridanus]	4,00E-65
gALL_v3_c14805	2	17	-	-	ref|XP_001947729.2|	PREDICTED: hypothetical protein LOC100167472 [Acyrthosiphon pisum]	5,00E-08
gALL_v3_c14812	3	13	-	-	-	-	-
gALL_v3_c1487	5	29	-	-	-	-	-
gALL_v3_c1489	12	49	gnl|CDD|189424	pfam00153, Mito_carr, Mitoch	ref|XP_001605622.2|	PREDICTED: calcium-binding mitochondrial carrier protein Aralar1-like isoform 1 [Nasonia vitripennis]	0
gALL_v3_c14909	2	14	-	-	-	-	-
gALL_v3_c14970	6	21	-	-	gb|EFN64027.1|	Myosin light chain kinase, smooth muscle [Camponotus floridanus]	1,00E-14
gALL_v3_c15003	3	13	-	-	-	-	-
gALL_v3_c15041	1	10	-	-	-	-	-
gALL_v3_c15042	2	12	-	-	-	-	-
gALL_v3_c1520	5	17	gnl|CDD|176732	cd08317, Death_ank, Death de	ref|XP_002432671.1|	ankyrin-1, putative [Pediculus humanus corporis] gb|EEB19933.1| ankyrin-1, putative [Pediculus humanus corporis]	0
gALL_v3_c1537	2	12	-	-	-	-	-
gALL_v3_c1545	9	26	-	-	ref|XP_967855.1|	PREDICTED: similar to CG34398 CG34398-PA [Tribolium castaneum]	6,00E-17
gALL_v3_c15489	2	12	-	-	gb|EFZ22930.1|	hypothetical protein SINV_01996 [Solenopsis invicta]	7,00E-29
gALL_v3_c155	7	24	-	-	gb|EGI65497.1|	Neurobeachin [Acromyrmex echinatior]	0
gALL_v3_c15520	2	13	gnl|CDD|173672	cd05581, STKc_PDK1, Catalytic	gb|EFN80387.1|	3-phosphoinositide-dependent protein kinase 1 [Harpegnathos saltator]	4,00E-92
gALL_v3_c15550	1	12	-	-	-	-	-
gALL_v3_c156	6	25	-	-	-	-	-
gALL_v3_c1572	3	15	-	-	-	-	-
gALL_v3_c15735	1	11	-	-	-	-	-
gALL_v3_c15790	2	16	-	-	-	-	-
gALL_v3_c1584	22	56	-	-	gb|EFX71524.1|	hypothetical protein DAPPUDRAFT_227924 [Daphnia pulex]	1,00E-16
gALL_v3_c15867	3	13	-	-	-	-	-
gALL_v3_c15872	1	12	-	-	-	-	-
gALL_v3_c15917	3	13	gnl|CDD|191320	pfam05622, HOOK, HOOK pro	ref|XP_006615442.1|	PREDICTED: protein Hook homolog 3-like [Apis dorsata]	3,00E-36
gALL_v3_c15937	1	9	-	-	-	-	-
gALL_v3_c16010	5	25	-	-	-	-	-
gALL_v3_c16034	2	12	-	-	-	-	-
gALL_v3_c1617	3	13	gnl|CDD|72993	cd03234, ABCG_White, The W	ref|XP_002432196.1|	ATP-binding cassette transporter, putative [Pediculus humanus corporis] gb|EEB19458.1| ATP-binding cassette transporter, putative [Pediculus humanus corporis]	4,00E-35
gALL_v3_c16177	1	9	-	-	-	-	-
gALL_v3_c16188	2	11	-	-	-	-	-
gALL_v3_c16192	6	27	-	-	-	-	-
gALL_v3_c162	9	39	-	-	-	-	-
gALL_v3_c1620	5	21	-	-	-	-	-
gALL_v3_c16202	2	20	gnl|CDD|148205	pfam06459, RR_TM4-6, Ryanc	emb|CAA79221.1|	ryanodine receptor [Drosophila melanogaster]	1,00E-147
gALL_v3_c1629	3	17	-	-	-	-	-
gALL_v3_c164	3	13	gnl|CDD|117853	pfam09311, Rab5-bind, Rab5 I	ref|XP_966804.2|	PREDICTED: similar to kinesin light chain 1 and [Tribolium castaneum]	0
gALL_v3_c1645	4	16	gnl|CDD|29257	cd00200, WD40, WD40 domai	gb|EEZ99274.1|	supernumerary limbs [Tribolium castaneum]	0
gALL_v3_c16510	2	11	-	-	-	-	-
gALL_v3_c16532	3	14	gnl|CDD|189527	pfam00387, PI-PLC-Y, Phospha	ref|NP_001167177.1|	1-phosphatidylinositol-4,5-bisphosphate phosphodiesterase delta-4 [Salmo salar] gb|ACN10997.1| 1-phosphatidylinositol-4,5-bisphosphate phosphodiesterase delta-4 [Sa	3,00E-78
gALL_v3_c16551	1	9	-	-	-	-	-
gALL_v3_c16591	1	13	gnl|CDD|189365	pfam00053, Laminin_EGF, Lam	gb|EFX77181.1|	hypothetical protein DAPPUDRAFT_321711 [Daphnia pulex]	3,00E-93
gALL_v3_c16640	2	15	-	-	-	-	-
gALL_v3_c16642	1	11	-	-	-	-	-
gALL_v3_c16705	2	16	gnl|CDD|73264	cd01385, MYSc_type_IX, Myos	ref|XP_003241548.1|	PREDICTED: myosin-IXb isoform 4 [Acyrthosiphon pisum]	4,00E-19
gALL_v3_c1671	3	13	gnl|CDD|29115	cd00170, SEC14, Sec14p-like li	ref|XP_002424139.1|	conserved hypothetical protein [Pediculus humanus corporis] gb|EEB11401.1| conserved hypothetical protein [Pediculus humanus corporis]	4,00E-84
gALL_v3_c16850	3	20	-	-	-	-	-
gALL_v3_c16934	7	21	-	-	-	-	-
gALL_v3_c17043	1	10	-	-	-	-	-
gALL_v3_c17069	2	11	-	-	-	-	-
gALL_v3_c17222	2	17	gnl|CDD|191810	pfam07679, I-set, Immunoglol	ref|XP_001848913.1|	titin [Culex quinquefasciatus] gb|EDS29256.1| titin [Culex quinquefasciatus]	7,00E-78
gALL_v3_c1727	12	39	gnl|CDD|30099	cd01377, MYSc_type_II, Myos	emb|CAA42753.1|	myosin heavy chain [Drosophila melanogaster]	7,00E-21
gALL_v3_c17468	1	9	gnl|CDD|28913	cd00031, CA, Cadherin repeat	gb|EGI59930.1|	Cadherin-87A [Acromyrmex echinatior]	1,00E-116
gALL_v3_c17542	1	21	-	-	ref|XP_624151.1|	PREDICTED: alpha-tocopherol transfer protein-like isoform X2 [Apis mellifera] ref|XP_006563328.1| PREDICTED: alpha-tocopherol transfer protein-like isoform X1 [Apis me	4,00E-21
gALL_v3_c1763	3	21	-	-	gb|EFA06228.1|	hypothetical protein TcasGA2_TC009077 [Tribolium castaneum]	2,00E-72
gALL_v3_c17633	1	9	-	-	gb|EFA01350.1|	Zn finger homeodomain 2 [Tribolium castaneum]	1,00E-140
gALL_v3_c1769	13	43	-	-	ref|XP_002432667.1|	Abnormal long morphology protein, putative [Pediculus humanus corporis] gb|EEB19929.1| Abnormal long morphology protein, putative [Pediculus humanus corporis]	6,00E-57
gALL_v3_c177	4	36	-	-	ref|XP_001946694.1|	PREDICTED: hypothetical protein LOC100167416 [Acyrthosiphon pisum]	3,00E-25
gALL_v3_c17713	1	9	gnl|CDD|190818	pfam03981, Ubiq_cyt_C_chap	ref|XP_308162.3|	AGAP007714-PA [Anopheles gambiae str. PEST] gb|EAA04372.3| AGAP007714-PA [Anopheles gambiae str. PEST]	4,00E-47
gALL_v3_c1774	5	19	-	-	-	-	-
gALL_v3_c1777	2	12	gnl|CDD|35161	COG5602, SIN3, Histone deace	gb|EFN67818.1|	Paired amphipathic helix protein Sin3a [Camponotus floridanus]	5,00E-75
gALL_v3_c1782	3	16	-	-	-	-	-
gALL_v3_c17851	1	9	-	-	-	-	-
gALL_v3_c17869	3	15	gnl|CDD|29151	cd00189, TPR, Tetratricopeptid	dbj|BAN20706.1|	conserved hypothetical protein [Riptortus pedestris]	1,00E-78
gALL_v3_c17874	2	12	gnl|CDD|179368	PRK02106, PRK02106, choline	ref|XP_972532.1|	PREDICTED: similar to glucose dehydrogenase [Tribolium castaneum] gb|EFA05532.1| hypothetical protein TcasGA2_TC015719 [Tribolium castaneum]	7,00E-79
gALL_v3_c1791	8	25	-	-	-	-	-
gALL_v3_c17955	5	18	-	-	-	-	-
gALL_v3_c17969	2	12	-	-	-	-	-
gALL_v3_c1798	5	19	-	-	-	-	-
gALL_v3_c17980	2	11	-	-	-	-	-
gALL_v3_c18039	1	10	-	-	-	-	-
gALL_v3_c1805	1	10	gnl|CDD|178285	PLN02681, PLN02681, proline	ref|XP_002427385.1|	proline oxidase, putative [Pediculus humanus corporis] gb|EEB14647.1| proline oxidase, putative [Pediculus humanus corporis]	1,00E-65
gALL_v3_c18141	2	12	gnl|CDD|132738	cd06940, NR_LBD_REV_ERB, T	emb|CAM97374.1|	ecdysone inducible protein 75 [Blattella germanica]	1,00E-113
gALL_v3_c18300	1	10	-	-	-	-	-
gALL_v3_c18406	13	37	-	-	ref|XP_003692820.1|	PREDICTED: PDZ and LIM domain protein 3-like [Apis florea]	8,00E-17
gALL_v3_c1844	5	19	-	-	gb|EFN67634.1|	Putative uncharacterized transposon-derived protein F52C9.6 [Camponotus floridanus]	8,00E-17
gALL_v3_c1855	3	17	-	-	-	-	-
gALL_v3_c1865	5	18	-	-	gb|EGI62644.1|	Ankyrin-2 [Acromyrmex echinatior]	6,00E-19
gALL_v3_c18693	1	9	-	-	gb|EFN80576.1|	Putative uncharacterized transposon-derived protein F52C9.6 [Harpegnathos saltator]	2,00E-45
gALL_v3_c1887	17	42	gnl|CDD|145040	pfam01682, DB, DB module. T	ref|XP_973440.2|	PREDICTED: similar to conserved hypothetical protein [Tribolium castaneum] gb|EFA08351.1| hypothetical protein TcasGA2_TC005994 [Tribolium castaneum]	0
gALL_v3_c18907	8	37	-	-	-	-	-
gALL_v3_c18909	1	10	-	-	-	-	-
gALL_v3_c18973	2	12	-	-	ref|XP_005104745.1|	PREDICTED: histone-lysine N-methyltransferase SETMAR-like [Aplysia californica]	3,00E-32
gALL_v3_c19076	9	25	-	-	-	-	-
gALL_v3_c19113	1	10	-	-	gb|ERL95905.1|	hypothetical protein D910_00580, partial [Dendroctonus ponderosae]	5,00E-10
gALL_v3_c1917	7	25	-	-	-	-	-
gALL_v3_c1922	3	17	gnl|CDD|99723	cd06402, PB1_p62, The PB1 d	ref|XP_001608187.1|	PREDICTED: sequestosome-1-like [Nasonia vitripennis]	1,00E-64
gALL_v3_c19285	2	11	-	-	gb|EFA01849.1|	hypothetical protein TcasGA2_TC007452 [Tribolium castaneum]	5,00E-39
gALL_v3_c19293	3	13	-	-	-	-	-
gALL_v3_c19338	1	10	gnl|CDD|188212	TIGR02349, DnaJ_bact, chaper	ref|XP_003407262.1|	PREDICTED: dnaJ homolog subfamily B member 9-like [Loxodonta africana]	1,00E-14
gALL_v3_c1938	3	14	-	-	-	-	-
gALL_v3_c19384	1	10	-	-	-	-	-
gALL_v3_c19419	1	12	gnl|CDD|191925	pfam08005, PHR, PHR domain	dbj|BAM18303.1|	lute protein [Papilio xuthus]	1,00E-73
gALL_v3_c1949	5	18	-	-	-	-	-
gALL_v3_c1950	1	9	-	-	-	-	-
gALL_v3_c1952	3	15	-	-	-	-	-
gALL_v3_c1957	9	32	-	-	ref|XP_002053211.1|	GJ23470 [Drosophila virilis] gb|EDW66731.1| GJ23470 [Drosophila virilis]	5,00E-12
gALL_v3_c1959	2	14	-	-	-	-	-
gALL_v3_c19665	1	15	-	-	dbj|BAM17741.1|	Z band alternatively spliced PDZ-motif protein 66 [Papilio xuthus]	6,00E-19
gALL_v3_c19680	2	12	gnl|CDD|187698	cd09274, RNase_HI_RT_Ty3, T	ref|XP_001810599.1|	PREDICTED: similar to pol polyprotein [Tribolium castaneum]	1,00E-09
gALL_v3_c19718	1	9	-	-	-	-	-
gALL_v3_c19863	5	19	-	-	-	-	-
gALL_v3_c1990	2	13	gnl|CDD|178101	PLN02483, PLN02483, serine p	ref|XP_002426204.1|	serine palmitoyltransferase, putative [Pediculus humanus corporis] gb|EEB13466.1| serine palmitoyltransferase, putative [Pediculus humanus corporis]	1,00E-78
gALL_v3_c1997	8	22	-	-	gb|EFN81187.1|	Iporin [Harpegnathos saltator]	2,00E-30
gALL_v3_c19998	3	20	-	-	-	-	-
gALL_v3_c20130	1	9	-	-	-	-	-
gALL_v3_c2015	13	34	-	-	-	-	-
gALL_v3_c20166	1	10	-	-	gb|ETN65914.1|	3-oxoacyl-[acyl-carrier-protein] reductase 1 [Anopheles darlingi]	2,00E-10
gALL_v3_c20187	2	11	gnl|CDD|187584	cd05323, ADH_SDR_c_like, ins	gb|AAR84629.1|	putative alcohol dehydrogenase [Gryllotalpa orientalis]	3,00E-60
gALL_v3_c20400	25	129	-	-	sp|Q9XZ71.1|TNNT_PEF	RecName: Full=Troponin T; Short=TnT gb|AAD33603.1|AF133520_1 troponin T [Periplaneta americana]	3,00E-48
gALL_v3_c20458	1	10	-	-	-	-	-
gALL_v3_c20559	1	9	-	-	-	-	-
gALL_v3_c2061	1	9	gnl|CDD|165506	PHA03247, PHA03247, large te	gb|EFX75492.1|	hypothetical protein DAPPUDRAFT_306823 [Daphnia pulex]	7,00E-33
gALL_v3_c2063	1	9	gnl|CDD|133006	cd02513, CMP-NeuAc_Synthas	ref|NP_001156112.1|	cmp-n-acetylneuraminic acid synthase-like [Acyrthosiphon pisum] dbj|BAH72225.1| ACYPI002677 [Acyrthosiphon pisum]	7,00E-25
gALL_v3_c20636	2	12	gnl|CDD|145733	pfam02738, Ald_Xan_dh_C2, I	gb|EGI61333.1|	Xanthine dehydrogenase [Acromyrmex echinatior]	4,00E-78
gALL_v3_c20720	8	26	-	-	-	-	-
gALL_v3_c20725	5	22	-	-	-	-	-
gALL_v3_c20765	1	9	-	-	-	-	-
gALL_v3_c2098	4	24	-	-	ref|XP_006620594.1|	PREDICTED: sorbin and SH3 domain-containing protein 2-like isoform X25 [Apis dorsata]	1,00E-134
gALL_v3_c2103	6	34	-	-	-	-	-
gALL_v3_c21036	1	10	gnl|CDD|133303	cd04103, Centaurin_gamma, (	ref|XP_969889.2|	PREDICTED: similar to AGAP009160-PA [Tribolium castaneum]	2,00E-67
gALL_v3_c21169	4	19	-	-	-	-	-
gALL_v3_c2142	1	11	gnl|CDD|128427	smart00119, HECTc, Domain H	emb|CAF88118.1|	unnamed protein product [Tetraodon nigroviridis]	5,00E-87
gALL_v3_c21460	1	9	-	-	ref|XP_005885005.1|	PREDICTED: piggyBac transposable element-derived protein 3-like [Myotis brandtii]	1,00E-18
gALL_v3_c2154	2	18	-	-	-	-	-
gALL_v3_c21590	1	10	-	-	ref|XP_971423.1|	PREDICTED: similar to cytochrome P450 monooxygenase [Tribolium castaneum] gb|EFA10757.1| cytochrome P450-like protein [Tribolium castaneum]	1,00E-09
gALL_v3_c2160	18	44	-	-	gb|EFA07696.1|	hypothetical protein TcasGA2_TC002172 [Tribolium castaneum]	2,00E-32
gALL_v3_c2165	2	13	-	-	dbj|GAA50551.1|	transposon Ty3-I gap-Pol polyprotein [Clonorchis sinensis]	8,00E-17
gALL_v3_c21811	1	9	-	-	gb|AGM32707.1|	hypothetical protein, partial [Coptotermes formosanus]	1,00E-11
gALL_v3_c21875	3	26	gnl|CDD|34627	COG5022, COG5022, Myosin h	gb|ACQ91270.1|	myosin heavy chain variant B [Bombyx mandarina]	9,00E-43
gALL_v3_c2196	9	30	-	-	ref|XP_006619088.1|	PREDICTED: titin-like [Apis dorsata]	2,00E-31
gALL_v3_c22002	10	44	gnl|CDD|34673	COG5069, SAC6, Ca2+-binding	ref|XP_004536724.1|	PREDICTED: alpha-actinin, sarcomeric-like isoform X3 [Ceratitis capitata]	1,00E-153
gALL_v3_c22162	10	51	-	-	-	-	-
gALL_v3_c22195	3	16	-	-	-	-	-
gALL_v3_c2228	3	13	-	-	-	-	-
gALL_v3_c2230	3	18	-	-	-	-	-
gALL_v3_c22341	5	22	gnl|CDD|30099	cd01377, MYSc_type_II, Myos	gb|AAA28706.1|	myosin heavy-chain, partial [Drosophila melanogaster]	5,00E-53
gALL_v3_c22351	3	24	-	cd01377, MYSc_type_II, Myos	-	-	-
gALL_v3_c2237	9	25	-	-	-	-	-
gALL_v3_c2257	9	24	-	-	-	-	-
gALL_v3_c22599	2	11	-	-	-	-	-

gALL_v3_c2266	4	15	-	-	gb|AGM32370.1|	troponin I [Coptotermes formosanus]	3,00E-11
gALL_v3_c2274	28	71	-	-	ref|XP_003095911.1|	CRE-FBN-1 protein [Caenorhabditis remanei] gb|EFO88234.1| CRE-FBN-1 protein [Caenorhabditis remanei]	3,00E-07
gALL_v3_c22834	6	21	gnl|CDD|29138	cd00176, SPEC, Spectrin repea	gb|EFZ23046.1|	hypothetical protein SINV_10403 [Solenopsis invicta]	7,00E-90
gALL_v3_c2294	2	18	gnl|CDD|189372	pfam00067, p450, Cytochrome	ref|XP_005180966.1|	PREDICTED: probable cytochrome P450 4p3-like [Musca domestica]	4,00E-27
gALL_v3_c23012	8	26	-	-	ref|XP_002427617.1|	conserved hypothetical protein [Pediculus humanus corporis] gb|EEB14879.1| conserved hypothetical protein [Pediculus humanus corporis]	1,00E-108
gALL_v3_c23042	4	19	gnl|CDD|153339	cd07655, F-BAR_PACSIN, The I	ref|XP_006610694.1|	PREDICTED: protein kinase C and casein kinase substrate in neurons protein 2-like isoform X3 [Apis dorsata]	1,00E-136
gALL_v3_c2305	4	28	gnl|CDD|178494	PLN02906, PLN02906, xanthin	gb|EFN85870.1|	Xanthine dehydrogenase/oxidase [Harpegnathos saltator]	1,00E-136
gALL_v3_c2329	6	20	-	-	-	-	-
gALL_v3_c23474	1	10	-	-	-	-	-
gALL_v3_c237	4	19	gnl|CDD|187601	cd05343, Mgc4172-like_SDR_c	ref|XP_970128.2|	PREDICTED: similar to serine 3-dehydrogenase [Tribolium castaneum]	8,00E-84
gALL_v3_c2387	9	28	-	-	-	-	-
gALL_v3_c2388	1	9	-	-	-	-	-
gALL_v3_c24051	1	11	-	-	-	-	-
gALL_v3_c24307	3	15	gnl|CDD|188900	cd09501, SAM_SARM1-like_re	ref|XP_001601657.2|	PREDICTED: sterile alpha and TIR motif-containing protein 1-like [Nasonia vitripennis]	1,00E-151
gALL_v3_c2431	6	26	-	-	-	-	-
gALL_v3_c24314	36	88	gnl|CDD|144972	pfam01576, Myosin_tail_1, M	ref|XP_002432355.1|	paramyosin, long form, putative [Pediculus humanus corporis] gb|EEB19617.1| paramyosin, long form, putative [Pediculus humanus corporis]	1,00E-60
gALL_v3_c24365	1	11	-	-	gb|AAB65093.1|	Lian-Aa1 retrotransposon protein [Aedes aegypti]	3,00E-46
gALL_v3_c2448	3	31	gnl|CDD|189527	pfam00387, PI-PLC-Y, Phospha	gb|EEZ97838.1|	hypothetical protein TcasGA2_TC000207 [Tribolium castaneum]	0
gALL_v3_c2451	3	14	gnl|CDD|30099	cd01377, MYSc_type_II, Myos	gb|EFZ13136.1|	hypothetical protein SINV_00390 [Solenopsis invicta]	2,00E-27
gALL_v3_c247	26	63	gnl|CDD|173623	cd00180, PKc, Catalytic domai	ref|XP_002429190.1|	conserved hypothetical protein [Pediculus humanus corporis] gb|EEB16452.1| conserved hypothetical protein [Pediculus humanus corporis]	0
gALL_v3_c2472	3	19	gnl|CDD|178083	PLN02464, PLN02464, glycerol	ref|XP_975782.1|	PREDICTED: similar to GA20935-PA isoform 2 [Tribolium castaneum]	1,00E-175
gALL_v3_c2475	4	48	-	-	gb|EFZ13136.1|	hypothetical protein SINV_00390 [Solenopsis invicta]	5,00E-10
gALL_v3_c248	25	62	gnl|CDD|34842	COG5245, DYN1, Dynein, heav	ref|XP_003492058.1|	PREDICTED: dynein heavy chain, cytoplasmic-like [Bombus impatiens]	0
gALL_v3_c24823	17	93	gnl|CDD|144972	pfam01576, Myosin_tail_1, M	gb|ACO90372.1|	myosin heavy chain variant [Bombyx mori]	1,00E-25
gALL_v3_c2494	1	9	-	-	ref|XP_002604867.1|	hypothetical protein BRAFLDRAFT_58504 [Branchiostoma floridae] sp|C3YFB4.1|DRE2_BRAFL RecName: Full=Anamorsin homolog; AltName: Full=Fe-S cluster assembly pr	2,00E-35
gALL_v3_c2497	17	40	gnl|CDD|129058	smart00825, PKS_KS, Beta-ket	ref|XP_001605685.1|	PREDICTED: fatty acid synthase-like [Nasonia vitripennis]	0
gALL_v3_c250	14	39	-	-	-	-	-
gALL_v3_c25033	1	9	-	-	-	-	-
gALL_v3_c2535	8	41	-	-	ref|XP_003702279.1|	PREDICTED: uncharacterized protein LOC100880486 [Megachile rotundata]	1,00E-25
gALL_v3_c2558	4	17	-	-	-	-	-
gALL_v3_c25608	1	9	-	-	ref|XP_003702583.1|	PREDICTED: histone-lysine N-methyltransferase SETMAR-like [Megachile rotundata]	3,00E-37
gALL_v3_c2589	4	16	-	-	gb|EFZ17295.1|	hypothetical protein SINV_14571 [Solenopsis invicta]	2,00E-06
gALL_v3_c260	17	40	-	-	-	-	-
gALL_v3_c26024	17	50	gnl|CDD|188805	cd09421, LIM3_LIMPETin, The	ref|XP_001945130.2|	PREDICTED: prickle-like protein 3-like [Acyrthosiphon pisum]	1,00E-124
gALL_v3_c262	1	10	-	-	-	-	-
gALL_v3_c26299	2	11	gnl|CDD|189956	pfam01365, RYDR_ITPR, RIH d	gb|EFN78897.1|	Ryanodine receptor 44F [Harpegnathos saltator]	5,00E-95
gALL_v3_c26392	4	15	gnl|CDD|180261	PRK05790, PRK05790, putative	gb|ELU00361.1|	hypothetical protein CAPTEDRAFT_145800 [Capitella teleta]	1,00E-78
gALL_v3_c264	13	32	-	-	-	-	-
gALL_v3_c26504	2	11	-	-	-	-	-
gALL_v3_c2668	2	11	-	-	gb|AGK40893.1|	toll-13 [Nilaparvata lugens]	3,00E-71
gALL_v3_c2685	3	16	gnl|CDD|128621	smart00326, SH3, Src homolog	ref|XP_001606578.2|	PREDICTED: SH3 domain-containing RING finger protein 3-like [Nasonia vitripennis]	7,00E-30
gALL_v3_c26870	2	33	-	-	ref|XP_001602237.2|	PREDICTED: hypothetical protein LOC100118209 [Nasonia vitripennis]	1,00E-156
gALL_v3_c270	9	27	gnl|CDD|189452	pfam00211, Guanylate_cyc, Ad	gb|EGI66142.1|	Adenylate cyclase type 8 [Acromyrmex echinatior]	1,00E-163
gALL_v3_c27271	1	9	-	-	ref|XP_006824323.1|	PREDICTED: E3 ubiquitin-protein ligase RNF170-like isoform X2 [Saccoglossus kowalevskii]	8,00E-27
gALL_v3_c2734	9	27	-	-	gb|EFN62471.1|	Bullous pemphigoid antigen 1, isoforms 6/9/10 [Camponotus floridanus]	1,00E-106
gALL_v3_c2742	8	31	gnl|CDD|176050	cd08405, C2B_Synaptotagmin	gb|EGI63407.1|	Synaptotagmin-7 [Acromyrmex echinatior]	0
gALL_v3_c2748	6	21	-	-	-	-	-
gALL_v3_c2753	1	10	-	-	-	-	-
gALL_v3_c27565	2	11	gnl|CDD|128944	smart00704, ZnF_CDGSH, CDG	gb|AGM32455.1|	CDGSH iron-sulfur domain-containing protein [Coptotermes formosanus]	5,00E-28
gALL_v3_c27989	12	45	-	-	dbj|BAG30740.1|	muscle myosin heavy chain [Papilio xuthus]	1,00E-26
gALL_v3_c2829	1	19	gnl|CDD|180261	PRK05790, PRK05790, putative	ref|XP_003384087.1|	PREDICTED: acetyl-CoA acetyltransferase, cytosolic-like [Amphimedon queenslandica]	2,00E-36
gALL_v3_c2831	7	22	gnl|CDD|189979	pfam01417, ENTH, ENTH dom	ref|XP_001603654.2|	PREDICTED: clathrin interactor 1-like, partial [Nasonia vitripennis]	4,00E-77
gALL_v3_c28426	1	14	gnl|CDD|182198	PRK10019, PRK10019, nickel/c	-	-	-
gALL_v3_c2853	1	12	-	-	gb|ENN82626.1|	hypothetical protein YQE_001000, partial [Dendroctonus ponderosae]	3,00E-26
gALL_v3_c2856	8	22	-	-	-	-	-
gALL_v3_c28681	5	17	-	-	-	-	-
gALL_v3_c2875	2	13	-	-	-	-	-
gALL_v3_c2889	13	35	-	-	-	-	-
gALL_v3_c2893	2	13	gnl|CDD|189934	pfam01302, CAP_GLY, CAP-Gly	ref|XP_006572769.1|	PREDICTED: restin homolog isoform X4 [Apis mellifera]	8,00E-46
gALL_v3_c28944	20	79	gnl|CDD|30099	cd01377, MYSc_type_II, Myos	gb|ACQ91272.1|	myosin heavy chain variant A [Bombyx mori]	5,00E-77
gALL_v3_c290	1	10	-	-	ref|XP_003243437.1|	PREDICTED: hypothetical protein LOC100572891 [Acyrthosiphon pisum]	4,00E-18
gALL_v3_c2911	5	17	-	-	-	-	-
gALL_v3_c2917	12	32	gnl|CDD|162106	TIGR00917, 2A060601, Niema	ref|XP_006621500.1|	PREDICTED: niemann-Pick C1 protein-like isoform X7 [Apis dorsata]	0
gALL_v3_c29295	1	9	-	-	-	-	-
gALL_v3_c2950	1	17	gnl|CDD|128829	smart00557, IG_FLMN, Filamin	ref|XP_002423659.1|	conserved hypothetical protein [Pediculus humanus corporis] gb|EEB10921.1| conserved hypothetical protein [Pediculus humanus corporis]	1,00E-165
gALL_v3_c2953	2	12	-	-	-	-	-
gALL_v3_c29604	1	9	-	-	-	-	-
gALL_v3_c2981	2	11	-	-	gb|ADP94161.1|	cAMP-dependent protein kinase regulatory subunit 1, partial [Schistocerca gregaria]	2,00E-66
gALL_v3_c2988	15	39	gnl|CDD|173625	cd05032, PTKc_InsR_like, Cata	ref|XP_004079577.1|	PREDICTED: insulin receptor-like [Oryzias latipes]	1,00E-159
gALL_v3_c2991	1	10	-	-	ref|XP_002425369.1|	conserved hypothetical protein [Pediculus humanus corporis] gb|EEB12631.1| conserved hypothetical protein [Pediculus humanus corporis]	9,00E-48
gALL_v3_c29926	1	9	-	-	-	-	-
gALL_v3_c29974	3	17	gnl|CDD|144098	pfam00379, Chitin_bind_4, Ins	ref|XP_001869012.1|	pupal cuticle protein [Culex quinquefasciatus] gb|EDS28238.1| pupal cuticle protein [Culex quinquefasciatus]	3,00E-30
gALL_v3_c3014	2	12	-	-	-	-	-
gALL_v3_c3018	5	21	-	-	-	-	-
gALL_v3_c3019	2	13	-	-	-	-	-
gALL_v3_c3040	10	33	-	-	-	-	-
gALL_v3_c30446	4	19	-	-	gb|EGI67485.1|	Nesprin-1 [Acromyrmex echinatior]	7,00E-54
gALL_v3_c3049	10	26	gnl|CDD|176349	cd01273, CED-6, CED-6 Phospl	ref|XP_003400463.1|	PREDICTED: PTB domain-containing adapter protein ced-6-like [Bombus terrestris]	1,00E-103
gALL_v3_c3060	3	22	-	-	ref|XP_002426065.1|	synaptonemal complex protein ZIP1, putative [Pediculus humanus corporis] gb|EEB13327.1| synaptonemal complex protein ZIP1, putative [Pediculus humanus corporis]	3,00E-39
gALL_v3_c30789	4	15	-	-	-	-	-
gALL_v3_c30906	1	12	gnl|CDD|187698	cd09274, RNase_HI_RT_Ty3, T	ref|XP_001810599.1|	PREDICTED: similar to pol polyprotein [Tribolium castaneum]	1,00E-103
gALL_v3_c31614	5	23	gnl|CDD|128336	smart00020, Tryp_SPc, Trypsin	gb|AEI58566.1|	serine protease [Eupolyphaga sinensis]	2,00E-65
gALL_v3_c3164	3	14	gnl|CDD|29029	cd00127, DSPc, Dual specificity	ref|XP_003424742.1|	PREDICTED: dual specificity protein phosphatase 3-like isoform 2 [Nasonia vitripennis]	1,00E-66
gALL_v3_c3166	7	30	-	-	-	-	-
gALL_v3_c3182	1	10	gnl|CDD|132940	cd06609, STKc_MST3_like, Cat	ref|XP_001948516.2|	PREDICTED: serine/threonine-protein kinase 24-like [Acyrthosiphon pisum]	5,00E-26
gALL_v3_c31860	2	12	-	-	-	-	-
gALL_v3_c31865	2	12	-	-	gb|EHJ64328.1|	hypothetical protein KGM_19125 [Danaus plexippus]	1,00E-82
gALL_v3_c31966	8	26	gnl|CDD|129062	smart00829, PKS_ER, Enoylred	ref|XP_003707545.1|	PREDICTED: fatty acid synthase-like [Megachile rotundata]	1,00E-142
gALL_v3_c3208	5	32	gnl|CDD|177628	PHA03392, egt, ecdysteroid UI	gb|AGC84403.1|	glucosyl glucuronosyl transferases [Locusta migratoria]	1,00E-127
gALL_v3_c32346	3	23	-	-	ref|XP_972324.1|	PREDICTED: similar to alpha actinin CG4376-PB [Tribolium castaneum]	1,00E-41
gALL_v3_c3241	10	28	gnl|CDD|185453	PTZ00110, PTZ00110, helicase	gb|EFN64092.1|	Probable ATP-dependent RNA helicase DDX17 [Camponotus floridanus]	5,00E-50
gALL_v3_c32440	1	12	-	-	-	-	-
gALL_v3_c3246	4	22	-	-	gb|ENN74680.1|	hypothetical protein YQE_08797, partial [Dendroctonus ponderosae]	1,00E-76
gALL_v3_c32579	7	23	-	-	gb|AGM32609.1|	hypothetical protein [Coptotermes formosanus]	5,00E-47
gALL_v3_c326	10	27	gnl|CDD|191810	pfam07679, I-set, Immunoglol	gb|EFA00141.1|	hypothetical protein TcasGA2_TC002962 [Tribolium castaneum]	3,00E-94
gALL_v3_c32639	3	14	gnl|CDD|189954	pfam01359, Transposase_1, Tr	ref|XP_003702583.1|	PREDICTED: histone-lysine N-methyltransferase SETMAR-like [Megachile rotundata]	2,00E-80
gALL_v3_c3274	7	21	-	-	ref|XP_006608156.1|	PREDICTED: synaptic vesicular amine transporter-like isoform X4 [Apis dorsata]	4,00E-49
gALL_v3_c3299	4	15	-	-	ref|XP_003488170.1|	PREDICTED: LOW QUALITY PROTEIN: E3 ubiquitin-protein ligase HUWE1-like [Bombus impatiens]	2,00E-86
gALL_v3_c33021	10	31	gnl|CDD|187657	cd08954, KR_1_FAS_SDR_x, be	ref|XP_970417.2|	PREDICTED: similar to fatty acid synthase [Tribolium castaneum]	1,00E-100
gALL_v3_c3304	5	22	gnl|CDD|144522	pfam00955, HCO3_cotransp, H	ref|XP_003707884.1|	PREDICTED: sodium-driven chloride bicarbonate exchanger [Megachile rotundata]	0
gALL_v3_c3306	4	19	-	-	ref|XP_968442.1|	PREDICTED: similar to conserved hypothetical protein [Tribolium castaneum]	3,00E-34
gALL_v3_c33092	1	9	-	-	-	-	-
gALL_v3_c331	6	31	-	-	ref|XP_002431333.1|	conserved hypothetical protein [Pediculus humanus corporis] gb|EEB18595.1| conserved hypothetical protein [Pediculus humanus corporis]	7,00E-41
gALL_v3_c3318	3	15	gnl|CDD|178101	PLN02483, PLN02483, serine p	ref|XP_002426204.1|	serine palmitoyltransferase, putative [Pediculus humanus corporis] gb|EEB13466.1| serine palmitoyltransferase, putative [Pediculus humanus corporis]	1,00E-166
gALL_v3_c3319	2	11	-	-	-	-	-
gALL_v3_c3320	6	23	-	-	ref|XP_969770.1|	PREDICTED: similar to jing CG9397-PG [Tribolium castaneum] gb|EFA02114.1| hypothetical protein TcasGA2_TC007760 [Tribolium castaneum]	3,00E-10
gALL_v3_c33591	1	9	-	-	ref|XP_004531538.1|	PREDICTED: uncharacterized protein LOC101452609 [Ceratitis capitata]	4,00E-40
gALL_v3_c3364	3	14	-	-	-	-	-
gALL_v3_c3397	3	23	-	-	-	-	-
gALL_v3_c3412	4	22	gnl|CDD|190318	pfam02469, Fasciclin, Fasciclin	sp|P10675.1|FAS1_SCHA	RecName: Full=Fasciclin-1; AltName: Full=Fasciclin I; Short=FAS I; Short=FCN; Flags: Precursor gb|AAA29809.1| FCN I precursor [Schistocerca americana]	1,00E-160
gALL_v3_c3414	1	14	gnl|CDD|128725	smart00449, SPRY, Domain in S	ref|XP_002430723.1|	SPRY domain-containing SOCS box protein, putative [Pediculus humanus corporis] gb|EEB17985.1| SPRY domain-containing SOCS box protein, putative [Pediculus humanu	1,00E-101
gALL_v3_c3430	1	11	gnl|CDD|189341	pfam00010, HLH, Helix-loop-h	ref|XP_001608182.2|	PREDICTED: sterol regulatory element-binding protein 1-like [Nasonia vitripennis]	1,00E-39
gALL_v3_c3431	4	19	-	-	ref|XP_002423425.1|	conserved hypothetical protein [Pediculus humanus corporis] gb|EEB10687.1| conserved hypothetical protein [Pediculus humanus corporis]	2,00E-21
gALL_v3_c3472	1	15	-	-	-	-	-
gALL_v3_c35106	32	86	gnl|CDD|144972	pfam01576, Myosin_tail_1, M	gb|EHJ79033.1|	paramyosin [Danaus plexippus]	1,00E-119
gALL_v3_c3517	8	31	-	-	gb|EFA11406.1|	hypothetical protein TcasGA2_TC013580 [Tribolium castaneum]	2,00E-45
gALL_v3_c3524	2	11	gnl|CDD|143985	pfam00232, Glyco_hydro_1, G	gb|ADK12988.1|	beta-glucosidase [Reticulitermes flavipes]	1,00E-116
gALL_v3_c3537	3	15	-	-	ref|XP_003707216.1|	PREDICTED: hemocyte protein-glutamine gamma-glutamyltransferase-like [Megachile rotundata]	1,00E-124
gALL_v3_c35585	3	13	-	-	-	-	-
gALL_v3_c356	11	35	-	-	gb|AAF87093.1|AF1675	secreted antigen SagBb [Enterococcus hirae]	2,00E-12
gALL_v3_c3572	3	13	-	-	-	-	-
gALL_v3_c358	11	30	-	-	gb|ADI61823.1|	endonuclease-reverse transcriptase [Bombyx mori]	2,00E-24
gALL_v3_c35809	3	16	gnl|CDD|129058	smart00825, PKS_KS, Beta-ket	gb|EFZ16301.1|	hypothetical protein SINV_05241 [Solenopsis invicta]	0
gALL_v3_c3601	18	54	gnl|CDD|29257	cd00200, WD40, WD40 domai	ref|XP_001601614.2|	PREDICTED: WD repeat-containing protein 47-like, partial [Nasonia vitripennis]	1,00E-105
gALL_v3_c36036	8	42	gnl|CDD|162209	TIGR01116, ATPase-IIA1_Ca, sa	gb|EFN64964.1|	Calcium-transporting ATPase sarcoplasmic/endoplasmic reticulum type [Camponotus floridanus]	2,00E-49
gALL_v3_c36114	2	15	-	-	-	-	-
gALL_v3_c36222	8	35	gnl|CDD|187657	cd08954, KR_1_FAS_SDR_x, be	ref|XP_001949072.2|	PREDICTED: fatty acid synthase-like [Acyrthosiphon pisum]	1,00E-153
gALL_v3_c3624	3	13	-	-	ref|XP_002429543.1|	eukaryotic translation initiation factor 3 subunit, putative [Pediculus humanus corporis] gb|EEB16805.1| eukaryotic translation initiation factor 3 subunit, putative [Pedicul	1,00E-162
gALL_v3_c3629	8	22	-	-	-	-	-
gALL_v3_c3630	6	22	-	-	-	-	-
gALL_v3_c36394	5	17	gnl|CDD|29138	cd00176, SPEC, Spectrin repea	ref|XP_003700798.1|	PREDICTED: LOW QUALITY PROTEIN: microtubule-actin cross-linking factor 1-like [Megachile rotundata]	3,00E-73
gALL_v3_c36574	2	45	gnl|CDD|162209	TIGR01116, ATPase-IIA1_Ca, sa	gb|EFN82575.1|	Calcium-transporting ATPase sarcoplasmic/endoplasmic reticulum type [Harpegnathos saltator]	3,00E-39
gALL_v3_c3685	1	16	-	-	ref|XP_003241855.1|	PREDICTED: hypothetical protein LOC100571222 [Acyrthosiphon pisum]	8,00E-19
gALL_v3_c3739	7	24	-	-	-	-	-
gALL_v3_c3741	2	41	gnl|CDD|173833	cd00685, Trans_IPPS_HT, Trans	gb|AGW99945.1|	geranylgeranyl diphosphate synthase [Choristoneura fumiferana]	1,00E-100
gALL_v3_c3767	3	13	-	-	gb|EFN85931.1|	Protein disulfide-isomerase A5 [Harpegnathos saltator]	1,00E-132
gALL_v3_c3796	9	32	gnl|CDD|29153	cd00191, TY, Thyroglobulin typ	ref|XP_966964.1|	PREDICTED: similar to GA12555-PA [Tribolium castaneum]	6,00E-22
gALL_v3_c3799	8	31	-	-	-	-	-
gALL_v3_c38053	2	20	-	-	-	-	-
gALL_v3_c3812	5	26	-	-	-	-	-
gALL_v3_c3817	1	33	gnl|CDD|188171	TIGR01833, HMG-CoA-S_euk,	dbj|BAM38487.2|	3-hydroxy-3-methylglutaryl coenzyme A synthase [Nasutitermes takasagoensis]	0
gALL_v3_c3827	3	18	gnl|CDD|128459	smart00154, ZnF_AN1, AN1-lik	ref|XP_976031.1|	PREDICTED: similar to CG33936 CG33936-PB isoform 2 [Tribolium castaneum] gb|EEZ97181.1| hypothetical protein TcasGA2_TC004368 [Tribolium castaneum]	1,00E-57
gALL_v3_c3833	1	9	gnl|CDD|48211	cd03399, Band_7_flotillin, Bar	gb|EFN84153.1|	Flotillin-2 [Harpegnathos saltator]	2,00E-90
gALL_v3_c3835	9	29	gnl|CDD|191023	pfam04547, Anoctamin, Calciu	ref|XP_001607899.1|	PREDICTED: anoctamin-5-like [Nasonia vitripennis]	0

gALL_v3_c3838	14	44	-	-	-	-	-
gALL_v3_c3857	6	22	-	-	gb|EHJ71649.1|	hypothetical protein KGM_08503 [Danaus plexippus]	1,00E-30
gALL_v3_c3864	7	21	-	-	-	-	-
gALL_v3_c3884	4	21	-	-	-	-	-
gALL_v3_c38921	1	10	-	-	-	-	-
gALL_v3_c3900	4	18	-	-	gb|EFN84917.1|	ABC transporter G family member 20 [Harpegnathos saltator]	9,00E-16
gALL_v3_c3901	13	46	gnl|CDD|193043	pfam12473, DUF3694, Kinesin	ref|XP_003692269.1|	PREDICTED: LOW QUALITY PROTEIN: kinesin-like protein unc-104-like [Apis florea]	0
gALL_v3_c3935	2	11	gnl|CDD|128524	smart00228, PDZ, Domain pre	ref|XP_971725.1|	PREDICTED: similar to Tyrosine-protein phosphatase non-receptor type 13 (Protein-tyrosine phosphatase 1E) (PTP-E1) (hPTPE1) (PTP-BAS) (Protein-tyrosine phosphatase P	2,00E-85
gALL_v3_c3947	7	24	gnl|CDD|144280	pfam00625, Guanylate_kin, Gu	gb|EGI65226.1|	Disks large 1 tumor suppressor protein [Acromyrmex echinatior]	1,00E-131
gALL_v3_c39596	1	16	gnl|CDD|191810	pfam07679, I-set, Immunoglol	ref|XP_002431694.1|	conserved hypothetical protein [Pediculus humanus corporis] gb|EEB18956.1| conserved hypothetical protein [Pediculus humanus corporis]	3,00E-35
gALL_v3_c3961	15	49	-	-	-	-	-
gALL_v3_c40054	23	62	gnl|CDD|191810	pfam07679, I-set, Immunoglol	ref|XP_001602095.2|	PREDICTED: titin-like [Nasonia vitripennis]	1,00E-130
gALL_v3_c40100	6	19	-	-	-	-	-
gALL_v3_c4014	2	21	gnl|CDD|30768	COG0419, SbcC, ATPase involv	ref|XP_003420339.1|	PREDICTED: CAP-Gly domain-containing linker protein 1 isoform 1 [Loxodonta africana]	6,00E-26
gALL_v3_c4031	8	32	-	-	-	-	-
gALL_v3_c4054	5	28	gnl|CDD|189653	pfam00650, CRAL_TRIO, CRAL	ref|XP_003700322.1|	PREDICTED: alpha-tocopherol transfer protein-like [Megachile rotundata]	2,00E-26
gALL_v3_c4056	5	38	-	-	-	-	-
gALL_v3_c4064	1	14	-	-	-	-	-
gALL_v3_c4065	10	28	-	-	ref|XP_002424018.1|	conserved hypothetical protein [Pediculus humanus corporis] gb|EEB11280.1| conserved hypothetical protein [Pediculus humanus corporis]	2,00E-35
gALL_v3_c4074	4	17	-	-	-	-	-
gALL_v3_c4081	3	16	-	-	-	-	-
gALL_v3_c4100	1	23	-	-	-	-	-
gALL_v3_c4112	7	22	-	-	ref|XP_002427448.1|	conserved hypothetical protein [Pediculus humanus corporis] gb|EEB14710.1| conserved hypothetical protein [Pediculus humanus corporis]	1,00E-06
gALL_v3_c41441	10	32	-	-	ref|XP_001277988.1|	hypothetical protein [Trichomonas vaginalis G3] gb|EAX65058.1| hypothetical protein TVAG_569780 [Trichomonas vaginalis G3]	3,00E-14
gALL_v3_c4158	6	19	-	-	ref|XP_003485191.1|	PREDICTED: hypothetical protein LOC100746481 isoform 1 [Bombus impatiens]	1,00E-07
gALL_v3_c41893	11	42	gnl|CDD|188801	cd09417, LIM2_LIMPETin_like	dbj|BAN20835.1|	four and a half lim domains [Riptortus pedestris]	1,00E-48
gALL_v3_c42055	16	104	-	-	ref|XP_975727.1|	PREDICTED: similar to Zeelin1 CG6803-PD isoform 2 [Tribolium castaneum] gb|EFA01468.1| hypothetical protein TcasGA2_TC007015 [Tribolium castaneum]	3,00E-09
gALL_v3_c42219	2	25	gnl|CDD|191810	pfam07679, I-set, Immunoglol	ref|XP_006564376.1|	PREDICTED: titin isoform X14 [Apis mellifera]	8,00E-65
gALL_v3_c4223	6	19	gnl|CDD|110632	pfam01645, Glu_synthase, Co	ref|NP_001041678.1|	glutamate synthase [Bombyx mori] dbj|BAF30425.1| glutamate synthase [Bombyx mori]	1,00E-114
gALL_v3_c4228	3	14	-	-	-	-	-
gALL_v3_c42309	10	38	-	-	-	-	-
gALL_v3_c4235	1	10	-	-	gb|EFX79065.1|	hypothetical protein DAPPUDRAFT_245407 [Daphnia pulex]	9,00E-15
gALL_v3_c42514	6	44	gnl|CDD|189793	pfam00992, Troponin, Troponi	ref|XP_001688628.1|	AGAP001053-PC [Anopheles gambiae str. PEST] tpg|DAA05513.1| TPA_inf: troponin I isoform 6b1 [Anopheles gambiae str. PEST] gb|EDO63969.1| AGAP001053-PC [Anoph	3,00E-47
gALL_v3_c42631	103	292	gnl|CDD|31389	COG1196, Smc, Chromosome	gb|ACQ91270.1|	myosin heavy chain variant B [Bombyx mandarina]	1,00E-99
gALL_v3_c4275	4	16	gnl|CDD|162066	TIGR00845, caca, sodium/calci	ref|XP_312055.5|	AGAP002859-PA [Anopheles gambiae str. PEST] gb|EAA07757.5| AGAP002859-PA [Anopheles gambiae str. PEST]	0
gALL_v3_c4281	9	24	gnl|CDD|130170	TIGR01100, V_ATP_synt_C, va	gb|EGI63429.1|	V-type proton ATPase 21 kDa proteolipid subunit [Acromyrmex echinatior]	1,00E-100
gALL_v3_c42898	4	17	-	-	-	-	-
gALL_v3_c4291	5	22	gnl|CDD|152017	pfam11581, Argos, Antagonist	ref|XP_002429035.1|	conserved hypothetical protein [Pediculus humanus corporis] gb|EEB16297.1| conserved hypothetical protein [Pediculus humanus corporis]	1,00E-101
gALL_v3_c4300	3	17	-	-	-	-	-
gALL_v3_c43181	3	45	gnl|CDD|185504	PTZ00184, PTZ00184, calmodu	gb|AGM32406.1|	troponin C [Coptotermes formosanus]	6,00E-86
gALL_v3_c4326	1	13	-	-	-	-	-
gALL_v3_c43270	2	14	-	-	-	-	-
gALL_v3_c43301	10	42	-	-	ref|NP_001091843.1|	titin2 [Bombyx mori] dbj|BAC10620.1| Titin-like protein [Bombyx mori]	6,00E-08
gALL_v3_c43372	3	13	-	-	-	-	-
gALL_v3_c4365	1	9	gnl|CDD|28945	cd00063, FN3, Fibronectin typ	ref|XP_003699418.1|	PREDICTED: Down syndrome cell adhesion molecule-like protein Dscam2-like [Megachile rotundata]	1,00E-129
gALL_v3_c4378	5	18	-	-	ref|XP_001943921.2|	PREDICTED: testican-1-like [Acyrthosiphon pisum]	2,00E-70
gALL_v3_c440	11	27	gnl|CDD|73156	cd01650, RT_nLTR_like, RT_nL	gb|AAL40415.1|AF3698	endonuclease/reverse transcriptase [Branchiostoma floridae]	4,00E-30
gALL_v3_c4412	7	26	-	-	-	-	-
gALL_v3_c44226	4	25	-	-	gb|AGM32701.1|	hypothetical protein [Coptotermes formosanus]	7,00E-62
gALL_v3_c443	20	52	gnl|CDD|191810	pfam07679, I-set, Immunoglol	ref|XP_006619088.1|	PREDICTED: titin-like [Apis dorsata]	0
gALL_v3_c4440	3	13	-	-	ref|XP_005998403.1|	PREDICTED: uncharacterized protein LOC102358965 isoform X1 [Latimeria chalumnae] ref|XP_005998404.1| PREDICTED: uncharacterized protein LOC102358965 isoform	4,00E-21
gALL_v3_c4447	1	9	-	-	-	-	-
gALL_v3_c4464	4	16	gnl|CDD|162267	TIGR01243, CDC48, AAA famil	ref|XP_966692.1|	PREDICTED: similar to transitional endoplasmic reticulum ATPase TER94 isoform 1 [Tribolium castaneum] gb|EFA07465.1| transitional endoplasmic reticulum ATPase TER9	0
gALL_v3_c4465	3	14	-	-	-	-	-
gALL_v3_c4477	3	17	-	-	gb|EFA08507.1|	hypothetical protein TcasGA2_TC006159 [Tribolium castaneum]	1,00E-40
gALL_v3_c4517	5	17	-	-	-	-	-
gALL_v3_c4522	2	11	gnl|CDD|165506	PHA03247, PHA03247, large te	ref|XP_392097.4|	PREDICTED: collagen alpha-1(IV) chain-like isoform 1 [Apis mellifera]	4,00E-62
gALL_v3_c45368	1	10	-	-	-	-	-
gALL_v3_c4550	2	12	-	-	-	-	-
gALL_v3_c4555	1	12	-	-	ref|XP_006620562.1|	PREDICTED: uncharacterized protein LOC102671329 [Apis dorsata]	2,00E-31
gALL_v3_c456	5	22	gnl|CDD|190753	pfam03798, TRAM_LAG1_CLN	ref|XP_003488933.1|	PREDICTED: ceramide synthase 6-like [Bombus impatiens]	1,00E-138
gALL_v3_c45753	13	39	-	-	-	-	-
gALL_v3_c4590	8	24	-	-	-	-	-
gALL_v3_c4607	6	21	-	-	-	-	-
gALL_v3_c46198	6	45	gnl|CDD|191810	pfam07679, I-set, Immunoglol	gb|EFN83272.1|	Titin [Harpegnathos saltator]	7,00E-77
gALL_v3_c46333	5	20	-	-	-	-	-
gALL_v3_c4661	3	14	-	-	ref|XP_002428705.1|	conserved hypothetical protein [Pediculus humanus corporis] gb|EEB15967.1| conserved hypothetical protein [Pediculus humanus corporis]	2,00E-19
gALL_v3_c4662	12	31	-	-	-	-	-
gALL_v3_c46757	4	18	gnl|CDD|191810	pfam07679, I-set, Immunoglol	gb|ERL85177.1|	hypothetical protein D910_02599, partial [Dendroctonus ponderosae]	7,00E-59
gALL_v3_c4679	4	27	gnl|CDD|149993	pfam09141, Talin_middle, Tali	gb|EGI67012.1|	Talin-1 [Acromyrmex echinatior]	0
gALL_v3_c4686	1	9	gnl|CDD|30331	cd02846, PAZ_argonaute_like,	emb|CCV01213.1|	Argonaute-2, partial [Blattella germanica]	2,00E-46
gALL_v3_c46954	2	12	-	-	-	-	-
gALL_v3_c4708	1	11	-	-	-	-	-
gALL_v3_c4718	2	21	-	-	ref|XP_002429599.1|	predicted protein [Pediculus humanus corporis] gb|EEB16861.1| predicted protein [Pediculus humanus corporis]	1,00E-119
gALL_v3_c4744	1	10	-	-	-	-	-
gALL_v3_c4748	5	18	-	-	-	-	-
gALL_v3_c4808	8	24	-	-	-	-	-
gALL_v3_c4843	6	25	-	-	-	-	-
gALL_v3_c4855	3	13	-	-	-	-	-
gALL_v3_c4858	2	12	-	-	-	-	-
gALL_v3_c48727	2	13	-	-	gb|EEZ99437.1|	hypothetical protein TcasGA2_TC030635 [Tribolium castaneum]	3,00E-75
gALL_v3_c48836	1	24	-	-	-	-	-
gALL_v3_c4884	7	25	gnl|CDD|72849	cd04709, BAH_MTA, BAH, or E	ref|XP_975498.2|	PREDICTED: similar to MTA1-like CG2244-PB [Tribolium castaneum] gb|EFA06006.1| hypothetical protein TcasGA2_TC008832 [Tribolium castaneum]	0
gALL_v3_c4928	1	12	gnl|CDD|173353	PTZ00057, PTZ00057, glutathi	gb|EFA00065.1|	hypothetical protein TcasGA2_TC002878 [Tribolium castaneum]	1,00E-99
gALL_v3_c4951	3	14	-	-	-	-	-
gALL_v3_c4953	4	15	-	-	ref|XP_968552.2|	PREDICTED: similar to calmodulin-binding transcription activator [Tribolium castaneum]	4,00E-86
gALL_v3_c49787	14	45	-	-	-	-	-
gALL_v3_c49803	3	19	-	-	-	-	-
gALL_v3_c4982	7	27	-	-	-	-	-
gALL_v3_c50219	2	21	-	-	ref|XP_002431693.1|	novex-3, putative [Pediculus humanus corporis] gb|EEB18955.1| novex-3, putative [Pediculus humanus corporis]	1,00E-07
gALL_v3_c5023	2	15	gnl|CDD|128421	smart00111, C4, C-terminal ta	gb|EFZ16869.1|	hypothetical protein SINV_02661 [Solenopsis invicta]	2,00E-77
gALL_v3_c5035	5	18	-	-	gb|AAA16577.1|	kinesin light chain, partial [Doryteuthis pealeii]	3,00E-13
gALL_v3_c5043	1	12	-	-	-	-	-
gALL_v3_c5053	2	12	-	-	-	-	-
gALL_v3_c5065	5	17	gnl|CDD|190314	pfam02453, Reticulon, Reticul	ref|XP_002400175.1|	conserved hypothetical protein [Ixodes scapularis] gb|EEC11248.1| conserved hypothetical protein [Ixodes scapularis]	3,00E-34
gALL_v3_c50738	4	18	gnl|CDD|191810	pfam07679, I-set, Immunoglol	gb|EFN80687.1|	Titin [Harpegnathos saltator]	2,00E-62
gALL_v3_c50836	1	12	-	-	gb|EFN84661.1|	hypothetical protein EAI_03447 [Harpegnathos saltator]	4,00E-18
gALL_v3_c51025	8	28	-	-	-	-	-
gALL_v3_c5103	1	9	-	-	ref|XP_003489344.1|	PREDICTED: vacuolar protein sorting-associated protein 72 homolog [Bombus impatiens]	6,00E-27
gALL_v3_c513	5	17	-	-	-	-	-
gALL_v3_c5140	3	13	-	-	-	-	-
gALL_v3_c51552	2	11	gnl|CDD|189372	pfam00067, p450, Cytochrom	sp|P29981.1|CP4C1_BL	RecName: Full=Cytochrome P450 4C1; AltName: Full=CYPIVC1 gb|AAA27819.1| cytochrome P450 [Blaberus discoidalis]	3,00E-17
gALL_v3_c51601	10	48	gnl|CDD|173412	PTZ00121, PTZ00121, MAEBL;	-	-	-
gALL_v3_c5162	1	12	-	-	ref|XP_968604.2|	PREDICTED: similar to CG18304 CG18304-PA [Tribolium castaneum]	9,00E-66
gALL_v3_c51686	4	15	-	-	-	-	-
gALL_v3_c51711	3	23	gnl|CDD|191810	pfam07679, I-set, Immunoglol	ref|XP_004926282.1|	PREDICTED: muscle M-line assembly protein unc-89-like [Bombyx mori]	1,00E-48
gALL_v3_c5199	3	21	-	-	-	-	-
gALL_v3_c5201	1	9	-	-	-	-	-
gALL_v3_c52082	2	12	-	-	ref|XP_001962559.1|	GF14380 [Drosophila ananassae] gb|EDV31780.1| GF14380 [Drosophila ananassae]	2,00E-11
gALL_v3_c52305	4	23	-	-	-	-	-
gALL_v3_c52310	1	35	-	-	-	-	-
gALL_v3_c52323	1	9	-	-	ref|XP_001606812.2|	PREDICTED: nesprin-1-like [Nasonia vitripennis]	3,00E-17
gALL_v3_c5256	6	19	gnl|CDD|29257	cd00200, WD40, WD40 domai	ref|XP_001199794.2|	PREDICTED: apoptotic protease-activating factor 1-like [Strongylocentrotus purpuratus]	7,00E-22
gALL_v3_c5271	2	13	-	-	-	-	-
gALL_v3_c528	7	24	-	-	-	-	-
gALL_v3_c52949	65	273	-	-	gb|AGM32827.1|	hypothetical protein [Coptotermes formosanus]	2,00E-73
gALL_v3_c5298	1	14	gnl|CDD|109126	pfam00057, Ldl_recept_a, Low	dbj|BAE00010.1|	Lipophorin receptor [Rhyparobia maderae]	0
gALL_v3_c5327	1	13	-	-	ref|XP_003398118.1|	PREDICTED: LOW QUALITY PROTEIN: lysine-specific demethylase 6A-like [Bombus terrestris]	2,00E-26
gALL_v3_c534	11	31	-	-	ref|XP_002427204.1|	conserved hypothetical protein [Pediculus humanus corporis] gb|EEB14466.1| conserved hypothetical protein [Pediculus humanus corporis]	2,00E-44
gALL_v3_c53423	1	26	gnl|CDD|28933	cd00051, EFh, EF-hand, calciu	gb|AGM32406.1|	troponin C [Coptotermes formosanus]	2,00E-44
gALL_v3_c535	1	16	gnl|CDD|178915	PRK00174, PRK00174, acetyl-C	ref|XP_001601268.1|	PREDICTED: acyl-CoA synthetase short-chain family member 3, mitochondrial-like [Nasonia vitripennis]	4,00E-71
gALL_v3_c5371	2	11	-	-	-	-	-
gALL_v3_c5395	5	17	-	-	-	-	-
gALL_v3_c54216	2	21	-	-	-	-	-
gALL_v3_c54253	6	34	-	-	ref|XP_006619090.1|	PREDICTED: titin-like [Apis dorsata]	2,00E-14
gALL_v3_c54387	2	15	-	-	-	-	-
gALL_v3_c54589	5	17	-	-	-	-	-
gALL_v3_c5495	5	17	gnl|CDD|162108	TIGR00920, 2A060605, 3-hydr	dbj|BAM45097.1|	3-hydroxy-3-methylglutaryl coenzyme A reductase [Nasutitermes takasagoensis]	0
gALL_v3_c55006	8	29	-	-	-	-	-
gALL_v3_c5502	3	13	-	-	-	-	-
gALL_v3_c55022	14	49	gnl|CDD|185604	PTZ00416, PTZ00416, elongati	gb|AAR01291.1|	elongation factor-2 [Forficula auricularia]	2,00E-62
gALL_v3_c55132	1	12	-	-	ref|NP_001155060.1|	serine protease homolog 21 precursor [Nasonia vitripennis]	7,00E-45
gALL_v3_c5550	1	13	-	-	-	-	-
gALL_v3_c5573	6	19	-	-	gb|ACD38216.1|	FUTSCH precursor [Drosophila melanogaster]	7,00E-56
gALL_v3_c5596	2	18	-	-	-	-	-
gALL_v3_c56483	13	33	-	-	-	-	-
gALL_v3_c5649	5	17	-	-	-	-	-
gALL_v3_c5662	5	22	-	-	ref|XP_002431082.1|	JmjC domain-containing histone demethylation protein 2B, putative [Pediculus humanus corporis] gb|EEB18344.1| JmjC domain-containing histone demethylation protein	1,00E-131
gALL_v3_c5666	8	23	-	-	-	-	-
gALL_v3_c56748	2	11	-	-	ref|XP_003241548.1|	PREDICTED: myosin-IXb isoform 4 [Acyrthosiphon pisum]	2,00E-20
gALL_v3_c5675	4	37	gnl|CDD|189372	pfam00067, p450, Cytochrom	sp|P29981.1|CP4C1_BL	RecName: Full=Cytochrome P450 4C1; AltName: Full=CYPIVC1 gb|AAA27819.1| cytochrome P450 [Blaberus discoidalis]	1,00E-132
gALL_v3_c5677	3	15	-	-	gb|ELR55055.1|	Jerky protein, partial [Bos mutus]	1,00E-11
gALL_v3_c5684	1	13	-	-	-	-	-
gALL_v3_c5687	13	32	-	-	ref|XP_003242865.1|	PREDICTED: hypothetical protein LOC100572069 [Acyrthosiphon pisum]	8,00E-13

gALL_v3_c56923	8	30	-	-	-	-	-
gALL_v3_c56924	17	60	gnl|CDD|111612	pfam02736, Myosin_N, Myosi	gb|ACO90373.1|	myosin heavy chain variant [Bombyx mori]	1,00E-63
gALL_v3_c5693	2	11	-	-	gb|ADI61823.1|	endonuclease-reverse transcriptase [Bombyx mori]	4,00E-10
gALL_v3_c57161	1	9	-	-	-	-	-
gALL_v3_c5733	3	19	-	-	-	-	-
gALL_v3_c57467	5	21	gnl|CDD|179368	PRK02106, PRK02106, choline	ref|XP_972532.1|	PREDICTED: similar to glucose dehydrogenase [Tribolium castaneum] gb|EFA05532.1| hypothetical protein TcasGA2_TC015719 [Tribolium castaneum]	1,00E-155
gALL_v3_c5764	8	25	gnl|CDD|29138	cd00176, SPEC, Spectrin repea	ref|XP_002430009.1|	Spectrin alpha chain, putative [Pediculus humanus corporis] gb|EEB17271.1| Spectrin alpha chain, putative [Pediculus humanus corporis]	0
gALL_v3_c5766	11	46	-	-	ref|XP_001949072.2|	PREDICTED: fatty acid synthase-like [Acyrthosiphon pisum]	1,00E-172
gALL_v3_c5768	1	9	-	-	-	-	-
gALL_v3_c5785	1	9	-	-	-	-	-
gALL_v3_c580	9	27	-	-	-	-	-
gALL_v3_c58104	11	40	gnl|CDD|189482	pfam00274, Glycolytic, Fructo	gb|AEV89754.1|	fructose 1,6-bisphosphate aldolase [Schistocerca gregaria]	1,00E-118
gALL_v3_c5820	8	32	-	-	gb|ETN58261.1|	thyroid receptor interacting protein [Anopheles darlingi]	4,00E-52
gALL_v3_c58440	19	61	gnl|CDD|192309	pfam09507, CDC27, DNA polyı	ref|XP_006564375.1|	PREDICTED: titin isoform X13 [Apis mellifera]	3,00E-14
gALL_v3_c586	1	18	-	-	ref|XP_002424114.1|	Vascular cell adhesion protein 1 precursor, putative [Pediculus humanus corporis] gb|EEB11376.1| Vascular cell adhesion protein 1 precursor, putative [Pediculus humanus	3,00E-17
gALL_v3_c59	4	21	-	-	-	-	-
gALL_v3_c5912	3	20	-	-	-	-	-
gALL_v3_c59261	12	36	-	-	gb|EFN85926.1|	hypothetical protein EAI_07848 [Harpegnathos saltator]	5,00E-24
gALL_v3_c5944	6	20	-	-	-	-	-
gALL_v3_c5987	2	16	-	-	gb|AGM32376.1|	troponin I [Coptotermes formosanus]	5,00E-06
gALL_v3_c5995	6	37	-	-	ref|XP_006607444.1|	PREDICTED: cysteine and histidine-rich protein 1 homolog [Apis dorsata]	1,00E-109
gALL_v3_c60025	4	16	-	-	ref|XP_004571762.1|	PREDICTED: A disintegrin and metalloproteinase with thrombospondin motifs 2-like [Maylandia zebra]	7,00E-09
gALL_v3_c6006	1	10	-	-	-	-	-
gALL_v3_c6011	1	10	-	-	-	-	-
gALL_v3_c6035	5	24	gnl|CDD|129058	smart00825, PKS_KS, Beta-ket	ref|XP_002432036.1|	fatty acid synthase, putative [Pediculus humanus corporis] gb|EEB19298.1| fatty acid synthase, putative [Pediculus humanus corporis]	1,00E-118
gALL_v3_c6038	5	17	-	-	gb|EGI57861.1|	hypothetical protein G5I_14048 [Acromyrmex echinatior]	3,00E-14
gALL_v3_c6061	2	14	-	-	gb|ETN63584.1|	hypothetical protein AND_004704 [Anopheles darlingi]	7,00E-08
gALL_v3_c60956	20	63	gnl|CDD|185498	PTZ00173, PTZ00173, 60S ribo	gb|AGM32193.1|	60S ribosomal protein L10 [Coptotermes formosanus]	5,00E-86
gALL_v3_c6096	5	20	gnl|CDD|128796	smart00523, DWA, Domain A i	ref|XP_001947078.2|	PREDICTED: nuclear factor 1 X-type-like [Acyrthosiphon pisum]	1,00E-136
gALL_v3_c610	3	33	-	-	-	-	-
gALL_v3_c6116	1	9	-	-	-	-	-
gALL_v3_c612	2	14	-	-	-	-	-
gALL_v3_c61244	18	88	-	-	-	-	-
gALL_v3_c61349	20	67	gnl|CDD|30099	cd01377, MYSc_type_II, Myos	gb|EHJ75788.1|	hypothetical protein KGM_08593 [Danaus plexippus]	1,00E-84
gALL_v3_c6137	2	12	-	-	-	-	-
gALL_v3_c6151	8	22	-	-	-	-	-
gALL_v3_c6164	2	14	-	-	ref|XP_001602237.2|	PREDICTED: hypothetical protein LOC100118209 [Nasonia vitripennis]	7,00E-13
gALL_v3_c61731	2	13	-	-	gb|AGM32701.1|	hypothetical protein [Coptotermes formosanus]	2,00E-63
gALL_v3_c6194	3	13	gnl|CDD|34842	COG5245, DYN1, Dynein, heav	ref|XP_003492058.1|	PREDICTED: dynein heavy chain, cytoplasmic-like [Bombus impatiens]	0
gALL_v3_c6201	15	37	gnl|CDD|165382	PHA03100, PHA03100, ankyrir	gb|EFA01044.1|	hypothetical protein TcasGA2_TC003960 [Tribolium castaneum]	0
gALL_v3_c6227	2	29	gnl|CDD|189372	pfam00067, p450, Cytochrom	gb|ABB86762.2|	CYP4U3v1 [Reticulitermes flavipes]	1,00E-175
gALL_v3_c6228	1	13	gnl|CDD|191810	pfam07679, I-set, Immunoglol	ref|XP_002431700.1|	myosin light chain kinase, putative [Pediculus humanus corporis] gb|EEB18962.1| myosin light chain kinase, putative [Pediculus humanus corporis]	4,00E-67
gALL_v3_c6234	8	28	gnl|CDD|165380	PHA03098, PHA03098, kelch-l	ref|XP_001605813.1|	PREDICTED: influenza virus NS1A-binding protein-like isoform 1 [Nasonia vitripennis] ref|XP_003424732.1| PREDICTED: influenza virus NS1A-binding protein-like isoform 2	6,00E-94
gALL_v3_c6253	6	20	gnl|CDD|31041	COG0697, RhaT, Permeases of	ref|XP_002423991.1|	transmembrane protein, putative [Pediculus humanus corporis] gb|EEB11253.1| transmembrane protein, putative [Pediculus humanus corporis]	2,00E-83
gALL_v3_c62687	6	24	-	-	-	-	-
gALL_v3_c6279	3	14	-	-	-	-	-
gALL_v3_c6287	4	15	-	-	-	-	-
gALL_v3_c6288	7	26	gnl|CDD|129062	smart00829, PKS_ER, Enoylred	ref|XP_003242717.1|	PREDICTED: fatty acid synthase-like isoform 2 [Acyrthosiphon pisum]	1,00E-151
gALL_v3_c6297	6	21	-	-	-	-	-
gALL_v3_c6315	1	12	-	-	gb|EHJ73180.1|	hypothetical protein KGM_12309 [Danaus plexippus]	7,00E-80
gALL_v3_c63235	3	16	-	-	-	-	-
gALL_v3_c634	8	22	gnl|CDD|189773	pfam00928, Adap_comp_sub,	gb|EFN68198.1|	Protein stoned-B [Camponotus floridanus]	0
gALL_v3_c6354	10	26	-	-	-	-	-
gALL_v3_c63613	2	11	-	-	-	-	-
gALL_v3_c6389	12	40	gnl|CDD|73156	cd01650, RT_nLTR_like, RT_nL	gb|EFA11778.1|	hypothetical protein TcasGA2_TC005283 [Tribolium castaneum]	1,00E-29
gALL_v3_c6391	4	15	-	-	ref|XP_002422722.1|	type II transmembrane protein, putative [Pediculus humanus corporis] gb|EEB09984.1| type II transmembrane protein, putative [Pediculus humanus corporis]	4,00E-54
gALL_v3_c6415	6	24	-	-	-	-	-
gALL_v3_c6427	3	16	-	-	-	-	-
gALL_v3_c64444	3	39	-	-	-	-	-
gALL_v3_c64542	7	27	gnl|CDD|144972	pfam01576, Myosin_tail_1, M	gb|AAR09774.1|	similar to Drosophila melanogaster Mhc, partial [Drosophila yakuba]	4,00E-35
gALL_v3_c64661	7	38	gnl|CDD|162209	TIGR01116, ATPase-IIA1_Ca, sa	gb|EFN64964.1|	Calcium-transporting ATPase sarcoplasmic/endoplasmic reticulum type [Camponotus floridanus]	2,00E-62
gALL_v3_c6467	1	9	gnl|CDD|29138	cd00176, SPEC, Spectrin repea	dbj|BAN20944.1|	spectrin [Riptortus pedestris]	1,00E-108
gALL_v3_c6470	3	13	gnl|CDD|29138	cd00176, SPEC, Spectrin repea	gb|EFN84144.1|	Nesprin-1 [Harpegnathos saltator]	4,00E-98
gALL_v3_c6484	1	10	-	-	ref|XP_003491216.1|	PREDICTED: hypothetical protein LOC100749566 [Bombus impatiens]	1,00E-36
gALL_v3_c6486	3	15	-	-	-	-	-
gALL_v3_c6514	2	11	-	-	ref|XP_002430960.1|	conserved hypothetical protein [Pediculus humanus corporis] gb|EEB18222.1| conserved hypothetical protein [Pediculus humanus corporis]	3,00E-35
gALL_v3_c6520	4	17	-	-	-	-	-
gALL_v3_c653	8	31	-	-	ref|XP_972687.2|	PREDICTED: similar to AGAP000727-PA [Tribolium castaneum]	3,00E-60
gALL_v3_c65322	1	21	-	-	-	-	-
gALL_v3_c6536	2	11	gnl|CDD|190521	pfam03096, Ndr, Ndr family. T	ref|XP_004537042.1|	PREDICTED: uncharacterized protein ZK1073.1-like isoform X7 [Ceratitis capitata]	2,00E-62
gALL_v3_c65687	1	9	gnl|CDD|73264	cd01385, MYSc_type_IX, Myo:	gb|EHJ74495.1|	hypothetical protein KGM_18974 [Danaus plexippus]	1,00E-19
gALL_v3_c6572	18	50	-	-	-	-	-
gALL_v3_c6576	3	15	-	-	-	-	-
gALL_v3_c66110	15	42	gnl|CDD|130689	TIGR01628, PABP-1234, polyac	gb|AAH03928.1|	Pabpc1 protein [Mus musculus]	5,00E-38
gALL_v3_c6613	2	11	-	-	gb|AGM32376.1|	troponin I [Coptotermes formosanus]	4,00E-11
gALL_v3_c66161	1	10	gnl|CDD|128829	smart00557, IG_FLMN, Filamir	ref|XP_002005147.1|	GI19229 [Drosophila mojavensis] gb|EDW09082.1| GI19229 [Drosophila mojavensis]	2,00E-57
gALL_v3_c6628	10	26	-	-	-	-	-
gALL_v3_c6631	1	10	gnl|CDD|188096	TIGR00930, 2a30, K-Cl cotrans	ref|XP_002424915.1|	conserved hypothetical protein [Pediculus humanus corporis] gb|EEB12177.1| conserved hypothetical protein [Pediculus humanus corporis]	0
gALL_v3_c66360	2	14	-	-	gb|EHJ79205.1|	p270 [Danaus plexippus]	1,00E-18
gALL_v3_c6654	1	9	-	-	-	-	-
gALL_v3_c66824	4	20	-	-	ref|XP_971849.2|	PREDICTED: similar to BMKETTIN [Tribolium castaneum]	7,00E-67
gALL_v3_c6689	3	22	-	-	-	-	-
gALL_v3_c67027	1	9	-	-	-	-	-
gALL_v3_c67122	5	20	gnl|CDD|132767	cd07306, Porin3_VDAC, Voltag	gb|AGM32824.1|	voltage-dependent anion-selective channel-like protein, partial [Coptotermes formosanus]	6,00E-89
gALL_v3_c6715	4	18	-	-	-	-	-
gALL_v3_c67220	7	24	-	-	-	-	-
gALL_v3_c6732	1	9	gnl|CDD|100104	cd00590, RRM, RRM (RNA rec	ref|XP_003486959.1|	PREDICTED: LOW QUALITY PROTEIN: CUGBP Elav-like family member 4-like [Bombus impatiens]	1,00E-106
gALL_v3_c6750	4	34	gnl|CDD|30099	cd01377, MYSc_type_II, Myos	gb|ACQ91272.1|	myosin heavy chain variant A [Bombyx mori]	1,00E-29
gALL_v3_c6754	3	17	-	-	-	-	-
gALL_v3_c6763	5	21	-	-	-	-	-
gALL_v3_c67786	7	23	gnl|CDD|128516	smart00220, S_TKc, Serine/Th	ref|XP_006624792.1|	PREDICTED: serine/threonine-protein kinase Doa-like isoform X2 [Apis dorsata]	1,00E-136
gALL_v3_c6788	1	9	gnl|CDD|128654	smart00360, RRM, RNA recogi	gb|EFX71524.1|	hypothetical protein DAPPUDRAFT_227924 [Daphnia pulex]	2,00E-65
gALL_v3_c679	15	37	-	-	-	-	-
gALL_v3_c67998	2	11	-	-	gb|EFA01440.1|	hypothetical protein TcasGA2_TC030701 [Tribolium castaneum]	6,00E-12
gALL_v3_c68257	6	21	-	-	gb|EFN83272.1|	Titin [Harpegnathos saltator]	5,00E-66
gALL_v3_c68277	14	37	-	-	-	-	-
gALL_v3_c6865	2	14	-	-	-	-	-
gALL_v3_c6868	1	12	gnl|CDD|187547	cd05236, FAR-N_SDR_e, fatty	ref|XP_002412479.1|	male sterility domain-containing protein, putative [Ixodes scapularis] gb|EEC15481.1| male sterility domain-containing protein, putative [Ixodes scapularis]	5,00E-19
gALL_v3_c68903	5	32	gnl|CDD|188712	cd09326, LIM_CRP_like, The LI	gb|EFN83539.1|	Muscle LIM protein Mlp84B [Harpegnathos saltator]	2,00E-97
gALL_v3_c6903	5	21	-	-	-	-	-
gALL_v3_c691	2	15	gnl|CDD|129972	TIGR00894, 2A0114euk, Na(+)	ref|XP_002433052.1|	conserved hypothetical protein [Pediculus humanus corporis] gb|EEB20314.1| conserved hypothetical protein [Pediculus humanus corporis]	0
gALL_v3_c6963	11	28	gnl|CDD|162740	TIGR02169, SMC_prok_A, chrc	ref|XP_392107.3|	PREDICTED: intracellular protein transport protein USO1 isoform X3 [Apis mellifera]	3,00E-26
gALL_v3_c6972	2	15	-	-	gb|EHJ68108.1|	hypothetical protein KGM_01207 [Danaus plexippus]	1,00E-130
gALL_v3_c7012	2	13	gnl|CDD|128591	smart00295, B41, Band 4.1 ho	emb|CCI09964.1|	coracle, partial [Blattella germanica]	1,00E-67
gALL_v3_c702	13	33	-	-	-	-	-
gALL_v3_c7025	4	16	-	-	gb|EFX74186.1|	hypothetical protein DAPPUDRAFT_307430 [Daphnia pulex]	1,00E-109
gALL_v3_c7027	1	13	-	-	-	-	-
gALL_v3_c705	5	18	gnl|CDD|30099	cd01377, MYSc_type_II, Myos	gb|EAW76685.1|	hCG2042559 [Homo sapiens]	3,00E-29
gALL_v3_c70669	1	12	gnl|CDD|129058	smart00825, PKS_KS, Beta-ket	ref|XP_001949072.2|	PREDICTED: fatty acid synthase-like [Acyrthosiphon pisum]	6,00E-67
gALL_v3_c7084	1	9	-	-	ref|XP_319087.2|	AGAP009953-PA [Anopheles gambiae str. PEST] ref|XP_001657939.1| G-protein, gamma-subunit, putative [Aedes aegypti] ref|XP_001842064.1| G-protein, gamma-subun	1,00E-28
gALL_v3_c7104	4	15	-	-	-	-	-
gALL_v3_c7128	4	24	-	-	gb|EFA12566.1|	hypothetical protein TcasGA2_TC004195 [Tribolium castaneum]	8,00E-19
gALL_v3_c7136	1	10	-	-	-	-	-
gALL_v3_c7138	6	23	-	-	-	-	-
gALL_v3_c7142	1	10	-	-	-	-	-
gALL_v3_c71702	1	9	-	-	ref|XP_001849863.1|	fatty acid synthase S-acetyl transferase [Culex quinquefasciatus] gb|EDS30986.1| fatty acid synthase S-acetyl transferase [Culex quinquefasciatus]	3,00E-15
gALL_v3_c7189	2	11	-	-	-	-	-
gALL_v3_c72	4	20	gnl|CDD|29005	cd02395, SF1_like-KH, Splicing	ref|NP_001164152.1|	held out wings [Tribolium castaneum]	1,00E-126
gALL_v3_c7205	4	22	gnl|CDD|191810	pfam07679, I-set, Immunoglol	gb|EFN89117.1|	Muscle M-line assembly protein unc-89 [Harpegnathos saltator]	1,00E-119
gALL_v3_c7228	2	25	-	-	gb|EFA13284.1|	hypothetical protein TcasGA2_TC010304 [Tribolium castaneum]	2,00E-28
gALL_v3_c7238	1	12	-	-	-	-	-
gALL_v3_c7251	12	31	-	-	ref|XP_005989206.1|	PREDICTED: tigger transposable element-derived protein 4-like [Latimeria chalumnae]	3,00E-21
gALL_v3_c726	1	9	-	-	-	-	-
gALL_v3_c7263	9	31	-	-	-	-	-
gALL_v3_c727	2	11	gnl|CDD|29261	cd00204, ANK, ankyrin repeat:	ref|XP_001811696.1|	PREDICTED: similar to sex-determining protein fem-1 [Tribolium castaneum] gb|EEZ97928.1| hypothetical protein TcasGA2_TC000315 [Tribolium castaneum]	3,00E-72
gALL_v3_c7275	3	13	-	-	-	-	-
gALL_v3_c728	1	14	-	-	-	-	-
gALL_v3_c7281	6	26	gnl|CDD|143179	cd04978, Ig4_L1-NrCAM_like,	ref|XP_006613706.1|	PREDICTED: neuroglian-like isoform X3 [Apis dorsata]	0
gALL_v3_c7282	1	10	gnl|CDD|144913	pfam01496, V_ATPase_I, V-typ	ref|XP_003486240.1|	PREDICTED: V-type proton ATPase 116 kDa subunit a isoform 1-like isoform 1 [Bombus impatiens]	1,00E-139
gALL_v3_c7293	2	11	-	-	gb|ERL85939.1|	hypothetical protein D910_03354 [Dendroctonus ponderosae]	4,00E-27
gALL_v3_c7338	8	26	gnl|CDD|73156	cd01650, RT_nLTR_like, RT_nL	ref|XP_006822105.1|	PREDICTED: RNA-directed DNA polymerase from mobile element jockey-like, partial [Saccoglossus kowalevskii]	2,00E-44
gALL_v3_c7367	1	11	-	-	-	-	-
gALL_v3_c7369	7	21	-	-	-	-	-
gALL_v3_c73694	2	14	-	-	gb|EFN75248.1|	PiggyBac transposable element-derived protein 4 [Harpegnathos saltator]	1,00E-10
gALL_v3_c737	1	12	-	-	ref|XP_002431914.1|	adenylate cyclase type, putative [Pediculus humanus corporis] gb|EEB19176.1| adenylate cyclase type, putative [Pediculus humanus corporis]	9,00E-19
gALL_v3_c7373	1	9	-	-	-	-	-
gALL_v3_c7380	6	19	gnl|CDD|29138	cd00176, SPEC, Spectrin repea	ref|XP_002026190.1|	GL16209 [Drosophila persimilis] gb|EDW33113.1| GL16209 [Drosophila persimilis]	1,00E-159
gALL_v3_c7409	1	9	-	-	-	-	-
gALL_v3_c75116	3	13	gnl|CDD|29020	cd00265, MADS_MEF2_like, M	ref|XP_002428246.1|	myocyte-specific enhancer factor 2A, putative [Pediculus humanus corporis] gb|EEB15508.1| myocyte-specific enhancer factor 2A, putative [Pediculus humanus corporis]	3,00E-54
gALL_v3_c7563	10	37	gnl|CDD|173412	PTZ00121, PTZ00121, MAEBL;	-	-	-
gALL_v3_c7587	1	13	-	-	-	-	-
gALL_v3_c7596	12	29	gnl|CDD|183763	PRK12810, gltD, glutamate syr	ref|XP_001948786.2|	PREDICTED: putative glutamate synthase [NADPH]-like [Acyrthosiphon pisum]	0
gALL_v3_c76762	96	248	gnl|CDD|189736	pfam00838, TCTP, Translation	gb|ABD98748.1|	putative translationally controlled tumor protein [Graphocephala atropunctata]	2,00E-84
gALL_v3_c7682	2	16	-	-	-	-	-

gALL_v3_c76861	4	21	-	-	gb|AGM32609.1|	hypothetical protein [Coptotermes formosanus]	6,00E-51
gALL_v3_c7699	1	9	-	-	-	-	-
gALL_v3_c7707	5	18	-	-	ref|XP_002081342.1|	GD25761 [Drosophila simulans] gb|EDX06927.1| GD25761 [Drosophila simulans]	3,00E-07
gALL_v3_c7714	1	9	-	-	-	-	-
gALL_v3_c7787	1	11	-	-	-	-	-
gALL_v3_c77871	1	12	-	-	-	-	-
gALL_v3_c7814	2	12	-	-	gb|EGI58212.1|	E3 ubiquitin-protein ligase RNF19A [Acromyrmex echinatior]	5,00E-46
gALL_v3_c7838	2	11	-	-	-	-	-
gALL_v3_c784	3	28	-	-	-	-	-
gALL_v3_c7874	2	15	-	-	gb|EFA13684.1|	hypothetical protein TcasGA2_TC016048 [Tribolium castaneum]	7,00E-14
gALL_v3_c7887	1	11	-	-	-	-	-
gALL_v3_c790	2	13	-	-	-	-	-
gALL_v3_c791	21	80	gnl|CDD|190193	pfam02026, RyR, RyR domain.	gb|AGW82429.1|	ryanodine receptor [Nilaparvata lugens]	0
gALL_v3_c7991	1	10	-	-	ref|XP_002423716.1|	hemicentin, putative [Pediculus humanus corporis] gb|EEB10978.1| hemicentin, putative [Pediculus humanus corporis]	9,00E-86
gALL_v3_c80	10	28	gnl|CDD|189954	pfam01359, Transposase_1, Tr	ref|XP_005104745.1|	PREDICTED: histone-lysine N-methyltransferase SETMAR-like [Aplysia californica]	2,00E-27
gALL_v3_c8028	2	12	-	-	-	-	-
gALL_v3_c8048	5	22	-	-	ref|XP_003489865.1|	PREDICTED: rab3 GTPase-activating protein non-catalytic subunit-like [Bombus impatiens]	6,00E-06
gALL_v3_c8084	1	9	-	-	-	-	-
gALL_v3_c8102	1	10	-	-	ref|XP_001862208.1|	conserved hypothetical protein [Culex quinquefasciatus] gb|EDS36746.1| conserved hypothetical protein [Culex quinquefasciatus]	1,00E-79
gALL_v3_c81076	8	30	gnl|CDD|191810	pfam07679, I-set, Immunoglol	ref|XP_971849.2|	PREDICTED: similar to BMKETTIN [Tribolium castaneum]	2,00E-69
gALL_v3_c8135	7	28	gnl|CDD|99947	cd05516, Bromo_SNF2L2, Broı	ref|XP_002432172.1|	Homeotic gene regulator, putative [Pediculus humanus corporis] gb|EEB19434.1| Homeotic gene regulator, putative [Pediculus humanus corporis]	4,00E-33
gALL_v3_c81586	2	11	gnl|CDD|100015	cd02186, alpha_tubulin, The t	dbj|BAF48685.1|	alpha tubulin [Spirotrichonympha leidyi]	0
gALL_v3_c8170	4	27	-	-	ref|XP_001629096.1|	predicted protein [Nematostella vectensis] gb|EDO37033.1| predicted protein [Nematostella vectensis]	5,00E-07
gALL_v3_c8172	5	19	gnl|CDD|147058	pfam04707, PRELI, PRELI-like f	gb|ADV76534.1|	slowmo protein variant 2 [Ostrinia nubilalis]	8,00E-69
gALL_v3_c8189	10	32	-	-	-	-	-
gALL_v3_c8199	1	14	-	-	-	-	-
gALL_v3_c821	5	23	gnl|CDD|190189	pfam02008, zf-CXXC, CXXC zin	ref|XP_002427386.1|	JmjC domain-containing histone demethylation protein 1B, putative [Pediculus humanus corporis] gb|EEB14648.1| JmjC domain-containing histone demethylation protein	0
gALL_v3_c822	3	15	-	-	-	-	-
gALL_v3_c8233	3	25	-	-	ref|XP_006619090.1|	PREDICTED: titin-like [Apis dorsata]	2,00E-19
gALL_v3_c825	12	32	gnl|CDD|177154	MTH00091, ND2, NADH dehyc	gb|EFN64278.1|	Microtubule-associated protein futsch [Camponotus floridanus]	2,00E-48
gALL_v3_c8279	2	11	-	-	-	-	-
gALL_v3_c8280	1	9	gnl|CDD|189706	pfam00759, Glyco_hydro_9, G	gb|AFE61612.1|	endo-beta-1,4-glucanase, partial [Coptotermes curvignathus]	2,00E-30
gALL_v3_c8293	3	19	-	-	-	-	-
gALL_v3_c8312	1	9	-	-	ref|XP_006568354.1|	PREDICTED: protein couch potato-like isoform X6 [Apis mellifera] ref|XP_006611193.1| PREDICTED: U1 small nuclear ribonucleoprotein A-like isoform X8 [Apis dorsata]	9,00E-52
gALL_v3_c8325	4	15	-	-	-	-	-
gALL_v3_c8326	2	12	gnl|CDD|192207	pfam09090, MIF4G_like_2, MI	ref|XP_973056.2|	PREDICTED: similar to AGAP001195-PA [Tribolium castaneum]	1,00E-111
gALL_v3_c8328	2	13	gnl|CDD|31326	COG1131, CcmA, ABC-type mu	ref|XP_003426604.1|	PREDICTED: ABC transporter G family member 20-like [Nasonia vitripennis]	5,00E-59
gALL_v3_c8372	5	19	-	-	-	-	-
gALL_v3_c8374	1	9	-	-	-	-	-
gALL_v3_c8406	9	72	-	-	sp|Q9XZ71.1|TNNT_PEF	RecName: Full=Troponin T; Short=TnT gb|AAD33603.1|AF133520_1 troponin T [Periplaneta americana]	2,00E-55
gALL_v3_c8440	1	9	-	-	ref|XP_005181068.1|	PREDICTED: tropomyosin-2-like isoform X1 [Musca domestica]	2,00E-06
gALL_v3_c846	2	13	-	-	gb|ENN72340.1|	hypothetical protein YQE_10975, partial [Dendroctonus ponderosae]	2,00E-26
gALL_v3_c8494	5	17	-	-	ref|XP_972333.1|	PREDICTED: similar to tetratricopeptide repeat protein [Tribolium castaneum] gb|EFA04363.1| hypothetical protein TcasGA2_TC014659 [Tribolium castaneum]	3,00E-32
gALL_v3_c8501	2	11	-	-	-	-	-
gALL_v3_c8506	3	14	-	-	-	-	-
gALL_v3_c8525	6	25	-	-	-	-	-
gALL_v3_c853	7	43	-	-	-	-	-
gALL_v3_c8549	3	14	-	-	ref|XP_003426184.1|	PREDICTED: uncharacterized protein KIAA1109-like [Nasonia vitripennis]	0
gALL_v3_c8555	1	10	-	-	-	-	-
gALL_v3_c8598	2	13	-	-	ref|XP_003245446.1|	PREDICTED: hypothetical protein LOC100571907 [Acyrthosiphon pisum]	5,00E-11
gALL_v3_c8631	2	11	-	-	-	-	-
gALL_v3_c8657	11	39	-	-	-	-	-
gALL_v3_c867	4	15	-	-	gb|EFA13593.1|	hypothetical protein TcasGA2_TC001480 [Tribolium castaneum]	2,00E-62
gALL_v3_c8691	6	24	-	-	ref|XP_971793.1|	PREDICTED: similar to zormin CG33484-PD [Tribolium castaneum]	6,00E-98
gALL_v3_c8714	4	30	-	-	ref|XP_557579.3|	AGAP004942-PA [Anopheles gambiae str. PEST] gb|EAL40201.3| AGAP004942-PA [Anopheles gambiae str. PEST]	8,00E-14
gALL_v3_c87562	1	10	-	-	-	-	-
gALL_v3_c876	10	32	-	-	-	-	-
gALL_v3_c8777	5	17	-	-	-	-	-
gALL_v3_c8796	18	43	-	-	ref|XP_006568907.1|	PREDICTED: microtubule-associated protein futsch-like isoform X1 [Apis mellifera]	1,00E-120
gALL_v3_c8815	5	18	-	-	ref|XP_006624130.1|	PREDICTED: uncharacterized protein LOC102673535 [Apis dorsata]	0
gALL_v3_c8817	3	15	-	-	-	-	-
gALL_v3_c884	15	42	gnl|CDD|189635	pfam00621, RhoGEF, RhoGEF (	gb|EFN89117.1|	Muscle M-line assembly protein unc-89 [Harpegnathos saltator]	1,00E-137
gALL_v3_c8875	1	9	-	-	-	-	-
gALL_v3_c88831	1	14	-	-	-	-	-
gALL_v3_c8915	2	11	-	-	-	-	-
gALL_v3_c894	5	17	-	-	-	-	-
gALL_v3_c90016	17	45	-	-	-	-	-
gALL_v3_c9018	1	9	-	-	ref|XP_001605921.2|	PREDICTED: neurotrimin-like [Nasonia vitripennis]	6,00E-27
gALL_v3_c9020	1	11	-	-	-	-	-
gALL_v3_c9027	1	11	-	-	-	-	-
gALL_v3_c9126	1	10	gnl|CDD|188822	cd09438, LIM3_Ajuba_like, Th	ref|XP_003703239.1|	PREDICTED: uncharacterized protein LOC100875117 [Megachile rotundata]	2,00E-26
gALL_v3_c913	4	15	gnl|CDD|28904	cd00020, ARM, Armadillo/beta	gb|AAL49501.1|AF4084	beta-catenin [Schistocerca americana]	0
gALL_v3_c9164	9	28	gnl|CDD|162209	TIGR01116, ATPase-IIA1_Ca, sa	gb|EFN64964.1|	Calcium-transporting ATPase sarcoplasmic/endoplasmic reticulum type [Camponotus floridanus]	1,00E-29
gALL_v3_c9182	17	72	gnl|CDD|191810	pfam07679, I-set, Immunoglol	ref|XP_971849.2|	PREDICTED: similar to BMKETTIN [Tribolium castaneum]	4,00E-55
gALL_v3_c9184	2	13	-	-	-	-	-
gALL_v3_c9200	13	33	gnl|CDD|129062	smart00829, PKS_ER, Enoylrec	ref|XP_001949072.2|	PREDICTED: fatty acid synthase-like [Acyrthosiphon pisum]	0
gALL_v3_c9218	1	11	-	-	-	-	-
gALL_v3_c930	4	16	-	-	-	-	-
gALL_v3_c931	5	26	gnl|CDD|176060	cd08678, C2_C21orf25-like, C2	ref|XP_970174.1|	PREDICTED: similar to GA10531-PA [Tribolium castaneum]	0
gALL_v3_c9402	7	24	-	-	-	-	-
gALL_v3_c9406	1	11	-	-	-	-	-
gALL_v3_c9423	1	10	-	-	ref|XP_002425303.1|	conserved hypothetical protein [Pediculus humanus corporis] gb|EEB12565.1| conserved hypothetical protein [Pediculus humanus corporis]	2,00E-44
gALL_v3_c946	7	24	-	-	-	-	-
gALL_v3_c9467	7	29	-	-	gb|EFA11365.1|	hypothetical protein TcasGA2_TC011522 [Tribolium castaneum]	1,00E-144
gALL_v3_c94959	11	34	gnl|CDD|128517	smart00221, STYKc, Protein kii	ref|XP_006618297.1|	PREDICTED: LOW QUALITY PROTEIN: twitchin-like [Apis dorsata]	1,00E-137
gALL_v3_c9508	1	10	-	-	-	-	-
gALL_v3_c9534	1	15	-	-	-	-	-
gALL_v3_c9606	4	17	-	-	-	-	-
gALL_v3_c9613	6	25	-	-	-	-	-
gALL_v3_c9669	5	27	-	-	-	-	-
gALL_v3_c9696	4	16	-	-	-	-	-
gALL_v3_c9713	3	14	gnl|CDD|177786	PLN00192, PLN00192, aldehyc	gb|EFZ19648.1|	hypothetical protein SINV_07033 [Solenopsis invicta]	1,00E-100
gALL_v3_c9715	2	12	gnl|CDD|31326	COG1131, CcmA, ABC-type mu	ref|XP_004933046.1|	PREDICTED: ABC transporter G family member 20-like [Bombyx mori]	8,00E-86
gALL_v3_c9720	2	19	-	-	ref|XP_004587838.1|	PREDICTED: fap1 adhesin-like [Ochotona princeps]	3,00E-10
gALL_v3_c9735	1	14	-	-	-	-	-
gALL_v3_c9818	3	14	-	-	ref|XP_003242415.1|	PREDICTED: hypothetical protein LOC100572493 [Acyrthosiphon pisum]	3,00E-09
gALL_v3_c9827	2	11	-	-	ref|XP_001599458.2|	PREDICTED: hypothetical protein LOC100114433 isoform 1 [Nasonia vitripennis]	4,00E-56
gALL_v3_c9857	4	19	-	-	-	-	-
gALL_v3_c9870	11	31	-	-	ref|XP_005714435.1|	unnamed protein product [Chondrus crispus] emb|CDF34616.1| unnamed protein product [Chondrus crispus]	2,00E-09
gALL_v3_c9884	8	27	gnl|CDD|34673	COG5069, SAC6, Ca2+-binding	ref|XP_003489920.1|	PREDICTED: nesprin-1-like [Bombus impatiens]	1,00E-134
gALL_v3_c9894	5	41	-	-	-	-	-
gALL_v3_c9918	3	13	-	-	-	-	-
gALL_v3_c9920	1	9	gnl|CDD|190162	pfam01925, TauE, Sulfite expo	gb|AGM32300.1|	sulfite exporter TauE/safE family protein, partial [Coptotermes formosanus]	1,00E-63
gALL_v3_c993	12	40	-	-	ref|XP_974751.2|	PREDICTED: similar to CG11321 CG11321-PA [Tribolium castaneum]	1,00E-73
gALL_v3_c9947	1	9	-	-	-	-	-
gALL_v3_c9961	2	12	-	-	-	-	-
gALL_v3_c9965	3	17	-	-	-	-	-
gALL_v3_c997	2	15	-	-	-	-	-
gALL_v3_c9977	7	21	gnl|CDD|187547	cd05236, FAR-N_SDR_e, fatty a	ref|XP_967757.1|	PREDICTED: similar to conserved hypothetical protein [Tribolium castaneum] gb|EFA08449.1| hypothetical protein TcasGA2_TC006096 [Tribolium castaneum]	1,00E-122
gALL_v3_rep_c101480	6	19	-	-	-	-	-
gALL_v3_rep_c103930	10	27	-	-	-	-	-
gALL_v3_rep_c104019	10	35	-	-	ref|XP_973700.2|	PREDICTED: similar to myosin vii [Tribolium castaneum]	9,00E-13
gALL_v3_rep_c104034	6	20	-	-	gb|ADI61823.1|	endonuclease-reverse transcriptase [Bombyx mori]	9,00E-62
gALL_v3_rep_c104038	1	10	gnl|CDD|180084	PRK05442, PRK05442, malate	gb|AGM32158.1|	malate dehydrogenase [Coptotermes formosanus]	7,00E-81
gALL_v3_rep_c104138	14	45	-	-	ref|XP_002609525.1|	hypothetical protein BRAFLDRAFT_232052 [Branchiostoma floridae] gb|EEN65535.1| hypothetical protein BRAFLDRAFT_232052 [Branchiostoma floridae]	2,00E-08
gALL_v3_rep_c104246	1	12	-	-	-	-	-
gALL_v3_rep_c104330	14	35	-	-	gb|EFZ17998.1|	hypothetical protein SINV_16519 [Solenopsis invicta]	1,00E-13
gALL_v3_rep_c104426	1	11	-	-	gb|AGM32465.1|	ejaculatory bulb-specific protein III [Coptotermes formosanus]	2,00E-12
gALL_v3_rep_c104456	3	19	gnl|CDD|100015	cd02186, alpha_tubulin, The t	dbj|BAF48684.1|	alpha tubulin [Holomastigotoides mirabile]	0
gALL_v3_rep_c104476	2	13	-	-	ref|XP_002609525.1|	hypothetical protein BRAFLDRAFT_232052 [Branchiostoma floridae] gb|EEN65535.1| hypothetical protein BRAFLDRAFT_232052 [Branchiostoma floridae]	1,00E-10
gALL_v3_rep_c104498	2	11	gnl|CDD|189372	pfam00067, p450, Cytochrome	ref|XP_002407241.1|	cytochrome P450, putative [Ixodes scapularis] gb|EEC03710.1| cytochrome P450, putative [Ixodes scapularis]	9,00E-39
gALL_v3_rep_c104555	3	17	-	-	ref|XP_002609525.1|	hypothetical protein BRAFLDRAFT_232052 [Branchiostoma floridae] gb|EEN65535.1| hypothetical protein BRAFLDRAFT_232052 [Branchiostoma floridae]	1,00E-09
gALL_v3_rep_c104637	3	17	-	-	-	-	-
gALL_v3_rep_c104787	1	9	-	-	-	-	-
gALL_v3_rep_c105196	3	14	-	-	-	-	-
gALL_v3_rep_c105423	2	11	-	-	-	-	-
gALL_v3_rep_c105708	3	18	-	-	-	-	-
gALL_v3_rep_c96114	828	2482	gnl|CDD|144972	pfam01576, Myosin_tail_1, M	ref|XP_003706244.1|	PREDICTED: myosin heavy chain, muscle-like isoform 5 [Megachile rotundata]	0
gALL_v3_rep_c96115	136	359	gnl|CDD|34798	COG5199, SCP1, Calponin [Cyt	gb|AGM32561.1|	calponin-likey domain containing protein [Coptotermes formosanus]	1,00E-100
gALL_v3_rep_c96150	400	1052	gnl|CDD|144972	pfam01576, Myosin_tail_1, M	ref|XP_002432355.1|	paramyosin, long form, putative [Pediculus humanus corporis] gb|EEB19617.1| paramyosin, long form, putative [Pediculus humanus corporis]	0
gALL_v3_rep_c96168	296	1343	gnl|CDD|162209	TIGR01116, ATPase-IIA1_Ca, sa	ref|XP_003707160.1|	PREDICTED: calcium-transporting ATPase sarcoplasmic/endoplasmic reticulum type-like [Megachile rotundata]	0
gALL_v3_rep_c96170	16	398	-	-	-	-	-
gALL_v3_rep_c96174	71	228	gnl|CDD|185474	PTZ00141, PTZ00141, elongati	gb|AGO46411.1|	elongation factor 1-alpha [Reticulitermes flavipes]	0
gALL_v3_rep_c96182	146	557	gnl|CDD|173412	PTZ00121, PTZ00121, MAEBL;	dbj|BAM19002.1|	fau protein [Papilio polytes]	6,00E-22
gALL_v3_rep_c96200	47	222	-	-	dbj|BAM17963.1|	fau protein [Papilio xuthus]	1,00E-10
gALL_v3_rep_c96207	49	136	gnl|CDD|34727	COG5126, FRQ1, Ca2+-binding	gb|AGM32403.1|	myosin light chain [Coptotermes formosanus]	3,00E-71
gALL_v3_rep_c96218	88	213	gnl|CDD|177221	MTH00166, ND6, NADH dehyc	ref|XP_003690729.1|	PREDICTED: icarapin-like [Apis florea]	5,00E-08
gALL_v3_rep_c96223	154	455	gnl|CDD|173412	PTZ00121, PTZ00121, MAEBL;	dbj|BAB64297.1|	I-connectin [Procambarus clarkii]	3,00E-36
gALL_v3_rep_c96235	77	269	gnl|CDD|191810	pfam07679, I-set, Immunoglol	gb|EGI57423.1|	Titin [Acromyrmex echinatior]	1,00E-113
gALL_v3_rep_c96238	530	1691	gnl|CDD|191810	pfam07679, I-set, Immunoglol	ref|XP_006564376.1|	PREDICTED: titin isoform X14 [Apis mellifera]	0
gALL_v3_rep_c96242	245	772	gnl|CDD|191810	pfam07679, I-set, Immunoglol	ref|XP_006564365.1|	PREDICTED: titin isoform X3 [Apis mellifera]	0
gALL_v3_rep_c96251	157	447	gnl|CDD|34673	COG5069, SAC6, Ca2+-binding	ref|XP_972324.1|	PREDICTED: similar to alpha actinin CG4376-PB [Tribolium castaneum]	0
gALL_v3_rep_c96254	157	523	gnl|CDD|191810	pfam07679, I-set, Immunoglol	ref|XP_006564376.1|	PREDICTED: titin isoform X14 [Apis mellifera]	0
gALL_v3_rep_c96261	26	84	gnl|CDD|162209	TIGR01116, ATPase-IIA1_Ca, sa	ref|NP_001157948.1|	sarco/endoplasmic reticulum calcium ATPase [Bombyx mori] gb|ACU25861.1| sarco/endoplasmic reticulum calcium ATPase [Bombyx mori]	1,00E-138

gALL_v3_rep_c96262	40	155	-	-	-	-	-
gALL_v3_rep_c96265	305	920	gnl|CDD|191810	pfam07679, I-set, Immunoglol	ref|XP_006564376.1|	PREDICTED: titin isoform X14 [Apis mellifera]	0
gALL_v3_rep_c96272	35	160	-	-	ref|XP_001604500.2|	PREDICTED: hypothetical protein LOC100120906 [Nasonia vitripennis]	1,00E-56
gALL_v3_rep_c96273	6	158	-	-	-	-	-
gALL_v3_rep_c96275	51	120	gnl|CDD|189793	pfam00992, Troponin, Troponi	gb|AGM32376.1|	troponin I [Coptotermes formosanus]	9,00E-63
gALL_v3_rep_c96294	77	209	-	-	ref|XP_002429689.1|	fatty acid synthase, putative [Pediculus humanus corporis] gb|EEB16951.1| fatty acid synthase, putative [Pediculus humanus corporis]	1,00E-32
gALL_v3_rep_c96297	32	92	-	-	ref|XP_002431799.1|	conserved hypothetical protein [Pediculus humanus corporis] gb|EEB19061.1| conserved hypothetical protein [Pediculus humanus corporis]	6,00E-43
gALL_v3_rep_c96301	3	109	gnl|CDD|185504	PTZ00184, PTZ00184, calmodu	gb|ABB89296.1|	allergen Bla g 6.0101 [Blattella germanica]	5,00E-74
gALL_v3_rep_c96305	19	97	gnl|CDD|189793	pfam00992, Troponin, Troponi	ref|XP_001688628.1|	AGAP001053-PC [Anopheles gambiae str. PEST] tpg|DAA05513.1| TPA_inf: troponin I isoform 6b1 [Anopheles gambiae str. PEST] gb|EDO63969.1| AGAP001053-PC [Anoph	7,00E-54
gALL_v3_rep_c96307	45	191	-	-	ref|XP_001951692.2|	PREDICTED: hypothetical protein LOC100160882 [Acyrthosiphon pisum]	4,00E-97
gALL_v3_rep_c96320	19	98	-	-	gb|AGM32105.1|	hypothetical protein [Coptotermes formosanus]	2,00E-88
gALL_v3_rep_c96325	18	62	-	-	emb|CAI46902.1|	flightin [Lethocerus indicus] emb|CAH17702.1| flightin [Lethocerus indicus]	3,00E-10
gALL_v3_rep_c96335	45	157	gnl|CDD|133138	cd05471, pepsin_like, Pepsin-l	gb|ADR82198.1|	Per a 2 allergen [Periplaneta americana]	1,00E-100
gALL_v3_rep_c96338	188	533	-	-	ref|XP_003693102.1|	PREDICTED: LOW QUALITY PROTEIN: titin-like [Apis florea]	1,00E-169
gALL_v3_rep_c96345	32	110	gnl|CDD|177154	MTH00091, ND2, NADH dehyc	ref|XP_003693102.1|	PREDICTED: LOW QUALITY PROTEIN: titin-like [Apis florea]	3,00E-29
gALL_v3_rep_c96349	27	114	-	-	-	-	-
gALL_v3_rep_c96359	20	58	gnl|CDD|162209	TIGR01116, ATPase-IIA1_Ca, sa	gb|EFA10430.1|	hypothetical protein TcasGA2_TC012671 [Tribolium castaneum]	4,00E-93
gALL_v3_rep_c96362	21	63	gnl|CDD|173412	PTZ00121, PTZ00121, MAEBL;	sp|Q9XZ71|TNNT_PERA	RecName: Full=Troponin T; Short=TnT gb|AAD33603.1|AF133520_1 troponin T [Periplaneta americana]	1,00E-134
gALL_v3_rep_c96365	208	593	gnl|CDD|173412	PTZ00121, PTZ00121, MAEBL;	ref|XP_971502.2|	PREDICTED: similar to CG32019-PA [Tribolium castaneum]	0
gALL_v3_rep_c96367	33	99	gnl|CDD|189793	pfam00992, Troponin, Troponi	gb|AGM32370.1|	troponin I [Coptotermes formosanus]	2,00E-58
gALL_v3_rep_c96370	32	146	gnl|CDD|173412	PTZ00121, PTZ00121, MAEBL;	ref|XP_006564376.1|	PREDICTED: titin isoform X14 [Apis mellifera]	1,00E-21
gALL_v3_rep_c96371	499	1187	gnl|CDD|29138	cd00176, SPEC, Spectrin repea	ref|XP_001809722.1|	PREDICTED: similar to Muscle-specific protein 300 CG33715-PD [Tribolium castaneum]	0
gALL_v3_rep_c96381	74	178	gnl|CDD|128539	smart00243, GAS2, Growth-Ar	gb|EFN78610.1|	Bullous pemphigoid antigen 1, isoforms 6/9/10 [Harpegnathos saltator]	0
gALL_v3_rep_c96383	265	1013	gnl|CDD|191810	pfam07679, I-set, Immunoglol	ref|XP_003701618.1|	PREDICTED: twitchin-like [Megachile rotundata]	0
gALL_v3_rep_c96400	69	197	gnl|CDD|173412	PTZ00121, PTZ00121, MAEBL;	gb|ADY75709.1|	projectin long variant [Libellula pulchella]	0
gALL_v3_rep_c96404	85	207	gnl|CDD|30768	COG0419, SbcC, ATPase involv	ref|XP_003393148.1|	PREDICTED: hypothetical protein LOC100648310 [Bombus terrestris]	0
gALL_v3_rep_c96411	4	65	gnl|CDD|185504	PTZ00184, PTZ00184, calmodu	gb|AGM32406.1|	troponin C [Coptotermes formosanus]	3,00E-78
gALL_v3_rep_c96419	19	56	gnl|CDD|31389	COG1196, Smc, Chromosome	ref|XP_972114.1|	PREDICTED: similar to Ofd1 protein, putative [Tribolium castaneum] gb|EEZ99674.1| hypothetical protein TcasGA2_TC002431 [Tribolium castaneum]	1,00E-21
gALL_v3_rep_c96428	47	114	gnl|CDD|191810	pfam07679, I-set, Immunoglol	ref|XP_971793.1|	PREDICTED: similar to zormin CG33484-PD [Tribolium castaneum]	1,00E-120
gALL_v3_rep_c96432	38	90	-	-	-	-	-
gALL_v3_rep_c96434	4	51	gnl|CDD|187601	cd05343, Mgc4172-like_SDR_c	gb|AGM32440.1|	enkurin [Coptotermes formosanus]	2,00E-56
gALL_v3_rep_c96444	23	79	gnl|CDD|31726	COG1537, PelA, Predicted RN/	ref|XP_002429525.1|	protein pelota, putative [Pediculus humanus corporis] gb|EEB16787.1| protein pelota, putative [Pediculus humanus corporis]	1,00E-174
gALL_v3_rep_c96450	27	64	-	-	-	-	-
gALL_v3_rep_c96452	8	55	gnl|CDD|191810	pfam07679, I-set, Immunoglol	ref|XP_006564376.1|	PREDICTED: titin isoform X14 [Apis mellifera]	1,00E-114
gALL_v3_rep_c96457	9	29	-	-	-	-	-
gALL_v3_rep_c96460	26	65	-	-	-	-	-
gALL_v3_rep_c96471	45	247	gnl|CDD|128829	smart00557, IG_FLMN, Filamin	gb|EFN86968.1|	Filamin-C [Harpegnathos saltator]	0
gALL_v3_rep_c96482	13	38	gnl|CDD|173456	PTZ00169, PTZ00169, ADP/ATI	gb|AGM32826.1|	ADP/ATP carrier protein 2, partial [Coptotermes formosanus]	2,00E-61
gALL_v3_rep_c96485	19	81	-	-	ref|XP_003692820.1|	PREDICTED: PDZ and LIM domain protein 3-like [Apis florea]	4,00E-64
gALL_v3_rep_c96494	30	85	gnl|CDD|185395	PTZ00009, PTZ00009, heat shc	gb|AFK49798.1|	heat shock protein 70 [Cryptocercus punctulatus]	0
gALL_v3_rep_c96500	13	42	-	-	ref|XP_002047225.1|	GJ13322 [Drosophila virilis] gb|EDW69567.1| GJ13322 [Drosophila virilis]	1,00E-12
gALL_v3_rep_c96505	9	32	-	-	gb|AGM32427.1|	soldier-caste specific protein [Coptotermes formosanus]	3,00E-69
gALL_v3_rep_c96507	65	188	gnl|CDD|31389	COG1196, Smc, Chromosome	gb|EFA06228.1|	hypothetical protein TcasGA2_TC009077 [Tribolium castaneum]	0
gALL_v3_rep_c96510	12	38	gnl|CDD|149702	pfam08726, efhand_Ca_insen	ref|XP_002427163.1|	alpha-actinin-4, putative [Pediculus humanus corporis] gb|EEB14425.1| alpha-actinin-4, putative [Pediculus humanus corporis]	1,00E-160
gALL_v3_rep_c96516	24	59	gnl|CDD|117221	pfam08648, DUF1777, Protein	gb|AGM32299.1|	serine-arginine protein 55-like protein, partial [Coptotermes formosanus]	8,00E-74
gALL_v3_rep_c96521	22	96	gnl|CDD|189476	pfam00261, Tropomyosin, Tro	gb|AGM32280.1|	putative tropomyosin, partial [Coptotermes formosanus]	1,00E-114
gALL_v3_rep_c96525	3	43	gnl|CDD|185757	cd09218, TLP-PA, allergenic/ar	gb|AFZ78845.1|	thaumatin-like protein 3 [Coptotermes formosanus]	3,00E-96
gALL_v3_rep_c96526	33	137	-	-	-	-	-
gALL_v3_rep_c96532	64	208	-	-	gb|EFN81734.1|	hypothetical protein EAI_13477 [Harpegnathos saltator]	5,00E-45
gALL_v3_rep_c96536	14	54	-	-	ref|XP_975727.1|	PREDICTED: similar to Zeelin1 CG6803-PD isoform 2 [Tribolium castaneum] gb|EFA01468.1| hypothetical protein TcasGA2_TC007015 [Tribolium castaneum]	9,00E-35
gALL_v3_rep_c96541	49	137	-	-	ref|XP_555219.3|	AGAP008244-PA [Anopheles gambiae str. PEST] gb|EAL39610.3| AGAP008244-PA [Anopheles gambiae str. PEST]	3,00E-37
gALL_v3_rep_c96544	12	36	-	-	ref|XP_003218078.1|	PREDICTED: hypothetical protein LOC100562870 [Anolis carolinensis]	4,00E-07
gALL_v3_rep_c96554	7	44	-	-	-	-	-
gALL_v3_rep_c96563	13	38	gnl|CDD|191685	pfam07084, Spot_14, Thyroid	ref|XP_971831.1|	PREDICTED: similar to CG2765 CG2765-PA [Tribolium castaneum] gb|EFA10830.1| hypothetical protein TcasGA2_TC001606 [Tribolium castaneum]	1,00E-44
gALL_v3_rep_c96575	111	304	gnl|CDD|128516	smart00220, S_TKc, Serine/Th	gb|EFN89117.1|	Muscle M-line assembly protein unc-89 [Harpegnathos saltator]	0
gALL_v3_rep_c96582	14	59	gnl|CDD|189793	pfam00992, Troponin, Troponi	ref|XP_003437191.1|	AGAP001053-PD [Anopheles gambiae str. PEST] ref|XP_003437192.1| AGAP001053-PG [Anopheles gambiae str. PEST] gb|EGK97268.1| AGAP001053-PD [Anopheles gamb	5,00E-61
gALL_v3_rep_c96585	21	55	gnl|CDD|182751	PRK10811, rne, ribonuclease E	-	-	-
gALL_v3_rep_c96591	10	59	gnl|CDD|191810	pfam07679, I-set, Immunoglol	ref|XP_002431694.1|	conserved hypothetical protein [Pediculus humanus corporis] gb|EEB18956.1| conserved hypothetical protein [Pediculus humanus corporis]	0
gALL_v3_rep_c96592	105	324	gnl|CDD|147872	pfam05955, Herpes_gp2, Equi	ref|YP_252241.1|	hypothetical protein SH0326 [Staphylococcus haemolyticus JCSC1435] ref|WP_011274654.1| adhesin [Staphylococcus haemolyticus] sp|Q4L9P0|SRAP_STAHJ RecName: F	4,00E-27
gALL_v3_rep_c96593	10	26	gnl|CDD|28945	cd00063, FN3, Fibronectin typ	gb|EFN80687.1|	Titin [Harpegnathos saltator]	7,00E-95
gALL_v3_rep_c96599	11	36	gnl|CDD|162209	TIGR01116, ATPase-IIA1_Ca, sa	gb|EGI60476.1|	Calcium-transporting ATPase sarcoplasmic/endoplasmic reticulum type [Acromyrmex echinatior]	1,00E-140
gALL_v3_rep_c96611	13	39	gnl|CDD|128336	smart00020, Tryp_SPc, Trypsin	gb|AEI58563.1|	serine protease [Eupolyphaga sinensis]	6,00E-49
gALL_v3_rep_c96623	24	75	-	-	gb|EFA07696.1|	hypothetical protein TcasGA2_TC002172 [Tribolium castaneum]	1,00E-32
gALL_v3_rep_c96625	52	131	-	-	-	-	-
gALL_v3_rep_c96636	23	59	-	-	ref|XP_002431692.1|	Titin, putative [Pediculus humanus corporis] gb|EEB18954.1| Titin, putative [Pediculus humanus corporis]	5,00E-20
gALL_v3_rep_c96637	33	98	-	-	-	-	-
gALL_v3_rep_c96655	5	23	gnl|CDD|185487	PTZ00158, PTZ00158, 40S ribo	gb|EFZ15546.1|	hypothetical protein SINV_12532 [Solenopsis invicta]	5,00E-68
gALL_v3_rep_c96656	11	28	-	-	gb|AGM32609.1|	hypothetical protein [Coptotermes formosanus]	3,00E-99
gALL_v3_rep_c96662	22	79	gnl|CDD|190521	pfam03096, Ndr, Ndr family. T	ref|XP_001946246.1|	PREDICTED: uncharacterized protein ZK1073.1-like [Acyrthosiphon pisum]	1,00E-120
gALL_v3_rep_c96665	6	38	gnl|CDD|143225	cd05748, Ig_Titin_like, Immun	ref|XP_002431692.1|	Titin, putative [Pediculus humanus corporis] gb|EEB18954.1| Titin, putative [Pediculus humanus corporis]	2,00E-63
gALL_v3_rep_c96679	19	58	gnl|CDD|29138	cd00176, SPEC, Spectrin repea	ref|XP_003245226.1|	PREDICTED: microtubule-actin cross-linking factor 1, isoforms 1/2/3/5-like isoform 6 [Acyrthosiphon pisum]	0
gALL_v3_rep_c96683	19	57	gnl|CDD|162209	TIGR01116, ATPase-IIA1_Ca, sa	ref|XP_002005872.1|	GI20714 [Drosophila mojavensis] gb|EDW09807.1| GI20714 [Drosophila mojavensis]	2,00E-59
gALL_v3_rep_c96684	5	30	-	-	ref|XP_006544049.1|	PREDICTED: NADH-ubiquinone oxidoreductase chain 2-like, partial [Mus musculus]	8,00E-08
gALL_v3_rep_c96687	15	60	-	-	-	-	-
gALL_v3_rep_c96700	13	52	-	-	ref|XP_003700798.1|	PREDICTED: LOW QUALITY PROTEIN: microtubule-actin cross-linking factor 1-like [Megachile rotundata]	1,00E-125
gALL_v3_rep_c96702	42	117	gnl|CDD|189496	pfam00307, CH, Calponin hom	ref|XP_004923712.1|	PREDICTED: microtubule-associated protein futsch-like [Bombyx mori]	1,00E-71
gALL_v3_rep_c96703	7	43	gnl|CDD|163023	TIGR02794, tolA_full, TolA pro	ref|XP_006567791.1|	PREDICTED: neurofilament heavy polypeptide-like isoform X4 [Apis mellifera]	2,00E-23
gALL_v3_rep_c96708	21	51	-	-	ref|XP_006570271.1|	PREDICTED: uncharacterized protein LOC102655090 [Apis mellifera]	1,00E-13
gALL_v3_rep_c96710	5	32	-	-	-	-	-
gALL_v3_rep_c96715	14	88	gnl|CDD|189372	pfam00067, p450, Cytochrom	sp|P29981|CP4C1_BLAD	RecName: Full=Cytochrome P450 4C1; AltName: Full=CYPIVC1 gb|AAA27819.1| cytochrome P450 [Blaberus discoidalis]	7,00E-66
gALL_v3_rep_c96737	50	125	gnl|CDD|189008	cd09601, M1_APN_2, Peptida	gb|EEZ99170.1|	aminopeptidase-like protein [Tribolium castaneum]	0
gALL_v3_rep_c96744	9	26	gnl|CDD|191810	pfam07679, I-set, Immunoglol	ref|XP_003693102.1|	PREDICTED: LOW QUALITY PROTEIN: titin-like [Apis florea]	8,00E-50
gALL_v3_rep_c96748	44	104	gnl|CDD|73264	cd01385, MYSc_type_IX, Myo	ref|XP_006608881.1|	PREDICTED: unconventional myosin-IXa-like isoform X1 [Apis dorsata]	1,00E-31
gALL_v3_rep_c96768	17	47	gnl|CDD|185524	PTZ00242, PTZ00242, protein	ref|XP_001603053.2|	PREDICTED: protein tyrosine phosphatase type IVA 1-like [Nasonia vitripennis]	1,00E-80
gALL_v3_rep_c96777	14	33	gnl|CDD|173623	cd00180, PKc, Catalytic domai	gb|EFA13259.1|	hypothetical protein TcasGA2_TC001529 [Tribolium castaneum]	3,00E-82
gALL_v3_rep_c96787	22	53	gnl|CDD|128358	smart00043, CY, Cystatin-like c	dbj|BAF94154.1|	cathepsin F-like cysteine protease [Plautia stali]	2,00E-35
gALL_v3_rep_c96790	6	20	-	-	-	-	-
gALL_v3_rep_c96801	10	30	-	-	-	-	-
gALL_v3_rep_c96820	24	59	-	-	ref|XP_006568918.1|	PREDICTED: microtubule-associated protein futsch-like isoform X12 [Apis mellifera]	1,00E-111
gALL_v3_rep_c96829	6	40	-	-	-	-	-
gALL_v3_rep_c96854	9	29	gnl|CDD|34798	COG5199, SCP1, Calponin [Cyt	ref|XP_001607280.1|	PREDICTED: myophilin-like [Nasonia vitripennis]	1,00E-81
gALL_v3_rep_c96857	7	30	gnl|CDD|166027	PLN02386, PLN02386, supero	ref|XP_775754.1|	hypothetical protein CNBD4830 [Cryptococcus neoformans var. neoformans B-3501A] gb|EAL21107.1| hypothetical protein CNBD4830 [Cryptococcus neoformans var. neo	2,00E-17
gALL_v3_rep_c96863	7	35	gnl|CDD|162739	TIGR02168, SMC_prok_B, chrc	ref|XP_004613536.1|	PREDICTED: golgin subfamily A member 2 [Sorex araneus]	8,00E-06
gALL_v3_rep_c96867	10	57	-	-	-	-	-
gALL_v3_rep_c96868	16	48	-	-	gb|EFN62471.1|	Bullous pemphigoid antigen 1, isoforms 6/9/10 [Camponotus floridanus]	1,00E-82
gALL_v3_rep_c96870	8	31	gnl|CDD|178494	PLN02906, PLN02906, xanthin	ref|XP_003705117.1|	PREDICTED: probable aldehyde oxidase 2-like [Megachile rotundata]	1,00E-101
gALL_v3_rep_c96874	6	19	-	-	-	-	-
gALL_v3_rep_c96888	7	36	-	-	ref|XP_001604500.2|	PREDICTED: hypothetical protein LOC100120906 [Nasonia vitripennis]	3,00E-50
gALL_v3_rep_c96897	14	34	-	-	ref|XP_006568911.1|	PREDICTED: microtubule-associated protein futsch-like isoform X5 [Apis mellifera]	2,33E-156
gALL_v3_rep_c96906	15	36	-	-	-	-	-
gALL_v3_rep_c96921	5	41	-	-	-	-	-
gALL_v3_rep_c96922	8	22	gnl|CDD|189372	pfam00067, p450, Cytochrom	gb|AAO20251.1|	cytochrome P450 monooxygenase CYP4G19 [Blattella germanica]	3,00E-70
gALL_v3_rep_c96933	12	29	gnl|CDD|29138	cd00176, SPEC, Spectrin repea	dbj|BAN20944.1|	spectrin [Riptortus pedestris]	4,00E-98
gALL_v3_rep_c96938	24	58	gnl|CDD|29020	cd00265, MADS_MEF2_like, N	ref|XP_971771.1|	PREDICTED: similar to myocyte-specific enhancer factor 2d [Tribolium castaneum]	1,00E-138
gALL_v3_rep_c96942	10	41	gnl|CDD|191810	pfam07679, I-set, Immunoglol	ref|XP_971849.2|	PREDICTED: similar to BMKETTIN [Tribolium castaneum]	1,00E-112
gALL_v3_rep_c96948	14	36	-	-	-	-	-
gALL_v3_rep_c96950	10	32	-	-	-	-	-
gALL_v3_rep_c96952	5	30	gnl|CDD|191810	pfam07679, I-set, Immunoglol	gb|EFA01440.1|	hypothetical protein TcasGA2_TC030701 [Tribolium castaneum]	1,00E-117
gALL_v3_rep_c96955	29	134	gnl|CDD|189372	pfam00067, p450, Cytochrom	gb|AAO20251.1|	cytochrome P450 monooxygenase CYP4G19 [Blattella germanica]	1,00E-148
gALL_v3_rep_c96960	18	45	gnl|CDD|189653	pfam00650, CRAL_TRIO, CRAL	ref|XP_968820.1|	PREDICTED: similar to alpha-tocopherol transfer protein [Tribolium castaneum] gb|EFA03949.1| hypothetical protein TcasGA2_TC014093 [Tribolium castaneum]	1,00E-94
gALL_v3_rep_c96982	4	15	-	-	gb|AGM32716.1|	uncharacterized protein [Coptotermes formosanus]	3,00E-51
gALL_v3_rep_c97004	6	19	-	-	-	-	-
gALL_v3_rep_c97025	8	26	gnl|CDD|29138	cd00176, SPEC, Spectrin repea	gb|EFN84144.1|	Nesprin-1 [Harpegnathos saltator]	2,00E-77
gALL_v3_rep_c97049	17	53	gnl|CDD|147872	pfam05955, Herpes_gp2, Equi	gb|EFA07696.1|	hypothetical protein TcasGA2_TC002172 [Tribolium castaneum]	4,00E-34
gALL_v3_rep_c97054	8	24	-	-	-	-	-
gALL_v3_rep_c97060	4	19	gnl|CDD|128829	smart00557, IG_FLMN, Filamin	ref|XP_002423659.1|	conserved hypothetical protein [Pediculus humanus corporis] gb|EEB10921.1| conserved hypothetical protein [Pediculus humanus corporis]	5,00E-95
gALL_v3_rep_c97063	5	19	gnl|CDD|147058	pfam04707, PRELI, PRELI-like f	gb|ADV76534.1|	slowmo protein variant 2 [Ostrinia nubilalis]	8,00E-87
gALL_v3_rep_c97065	20	49	-	-	ref|XP_005169830.1|	PREDICTED: mucin-2-like [Danio rerio]	4,00E-21
gALL_v3_rep_c97069	9	41	-	-	-	-	-
gALL_v3_rep_c97071	5	19	gnl|CDD|128524	smart00228, PDZ, Domain pre	ref|XP_003706903.1|	PREDICTED: uncharacterized protein LOC100874905 [Megachile rotundata]	3,00E-73
gALL_v3_rep_c97079	2	31	-	-	gb|EFN65619.1|	Putative uncharacterized transposon-derived protein F52C9.6 [Camponotus floridanus]	4,00E-16
gALL_v3_rep_c97082	5	93	gnl|CDD|32818	COG3000, ERG3, Sterol desatu	gb|EGI67593.1|	Uncharacterized protein C5orf4-like protein [Acromyrmex echinatior]	1,00E-147
gALL_v3_rep_c97109	11	61	-	-	ref|XP_006613849.1|	PREDICTED: sarcalumenin-like isoform X4 [Apis dorsata]	0
gALL_v3_rep_c97114	3	22	gnl|CDD|189793	pfam00992, Troponin, Troponi	ref|XP_003437194.1|	AGAP001053-PF [Anopheles gambiae str. PEST] gb|EGK97270.1| AGAP001053-PF [Anopheles gambiae str. PEST]	3,00E-51
gALL_v3_rep_c97120	6	22	gnl|CDD|191810	pfam07679, I-set, Immunoglol	ref|XP_001959462.1|	GF12043 [Drosophila ananassae] gb|EDV36284.1| GF12043 [Drosophila ananassae]	1,00E-69
gALL_v3_rep_c97123	11	29	gnl|CDD|149257	pfam08075, NOPS, NOPS (NUC	ref|XP_003706377.1|	PREDICTED: hrp65 protein-like [Megachile rotundata]	1,00E-115
gALL_v3_rep_c97129	8	26	gnl|CDD|144648	pfam01130, CD36, CD36 famil	ref|XP_002432097.1|	scavenger receptor class B member, putative [Pediculus humanus corporis] gb|EEB19359.1| scavenger receptor class B member, putative [Pediculus humanus corporis]	3,00E-52
gALL_v3_rep_c97132	4	18	gnl|CDD|162097	TIGR00898, 2A0119, cation tra	ref|XP_001659801.1|	organic cation transporter [Aedes aegypti] gb|EAT38951.1| AAEL009206-PA [Aedes aegypti]	2,00E-52
gALL_v3_rep_c97141	21	68	gnl|CDD|189372	pfam00067, p450, Cytochrom	sp|P29981|CP4C1_BLAD	RecName: Full=Cytochrome P450 4C1; AltName: Full=CYPIVC1 gb|AAA27819.1| cytochrome P450 [Blaberus discoidalis]	1,00E-71
gALL_v3_rep_c97149	6	19	gnl|CDD|191810	pfam07679, I-set, Immunoglol	gb|EFA01440.1|	hypothetical protein TcasGA2_TC030701 [Tribolium castaneum]	1,00E-91
gALL_v3_rep_c97150	9	26	gnl|CDD|179368	PRK02106, PRK02106, choline	ref|XP_002429699.1|	glucose dehydrogenase precursor, putative [Pediculus humanus corporis] gb|EEB16961.1| glucose dehydrogenase precursor, putative [Pediculus humanus corporis]	1,00E-132
gALL_v3_rep_c97159	1	32	gnl|CDD|162209	TIGR01116, ATPase-IIA1_Ca, sa	ref|XP_966783.1|	PREDICTED: similar to calcium-transporting atpase sarcoplasmic/endoplasmic reticulum type (calcium pump) isoform 1 [Tribolium castaneum]	3,00E-83
gALL_v3_rep_c97169	8	23	gnl|CDD|188151	TIGR01517, ATPase-IIB_Ca, pla	ref|XP_003705081.1|	PREDICTED: plasma membrane calcium-transporting ATPase 3 isoform 5 [Megachile rotundata]	1,00E-160
gALL_v3_rep_c97178	8	23	gnl|CDD|161744	TIGR00175, mito_nad_idh, iso	gb|EEZ99082.1|	hypothetical protein TcasGA2_TC004962 [Tribolium castaneum]	1,00E-125
gALL_v3_rep_c97191	5	17	-	-	-	-	-
gALL_v3_rep_c97195	6	19	-	-	-	-	-
gALL_v3_rep_c97201	16	39	-	-	-	-	-
gALL_v3_rep_c97209	14	54	gnl|CDD|187657	cd08954, KR_1_FAS_SDR_x, be	ref|NP_001037478.1|	p270 [Bombyx mori] gb|AAB53258.1| p270 [Bombyx mori]	1,00E-161
gALL_v3_rep_c97229	6	29	-	-	ref|XP_003489920.1|	PREDICTED: nesprin-1-like [Bombus impatiens]	5,00E-63
gALL_v3_rep_c97233	5	22	gnl|CDD|30099	cd01377, MYSc_type_II, Myos	ref|XP_001814139.1|	PREDICTED: similar to Myosin heavy chain CG17927-PF isoform 7 [Tribolium castaneum]	1,00E-133
gALL_v3_rep_c97235	3	13	-	-	sp|P82118|CUO5_BLAC	RecName: Full=Cuticle protein 5; AltName: Full=BcNCP14.6	2,00E-13
gALL_v3_rep_c97249	6	30	gnl|CDD|191810	pfam07679, I-set, Immunoglol	ref|XP_006613976.1|	PREDICTED: muscle M-line assembly protein unc-89-like isoform X5 [Apis dorsata]	3,00E-89
gALL_v3_rep_c97256	4	15	gnl|CDD|58648	cd00922, Cyt_c_Oxidase_IV, C	gb|AGG22607.1|	mitochondrial cytochrome c oxidase subunit IV [Locusta migratoria] gb|AGG22608.1| mitochondrial cytochrome c oxidase subunit IV [Locusta migratoria]	8,00E-64
gALL_v3_rep_c97267	9	30	gnl|CDD|73264	cd01385, MYSc_type_IX, Myo	gb|EFZ19338.1|	hypothetical protein SINV_09798 [Solenopsis invicta]	2,00E-36

gALL_v3_rep_c97275	1	16	-	-	-	-	-	
gALL_v3_rep_c97279	13	31	gnl|CDD|191810	pfam07679, I-set, Immunoglol	gb|EFA01442.1|	hypothetical protein TcasGA2_TC030718 [Tribolium castaneum]		1,00E-153
gALL_v3_rep_c97283	11	40	gnl|CDD|144972	pfam01576, Myosin_tail_1, M	ref|XP_006614996.1|	PREDICTED: LOW QUALITY PROTEIN: myosin heavy chain, muscle-like [Apis dorsata]		1,00E-68
gALL_v3_rep_c97294	3	19	-	-	dbj|BAN20764.1|	conserved hypothetical protein [Riptortus pedestris]		2,00E-22
gALL_v3_rep_c97319	3	13	gnl|CDD|146538	pfam03957, Jun, Jun-like trans	gb|AGM32319.1|	transcription factor AP-1(jun-like transcription factor), partial [Coptotermes formosanus]		1,00E-105
gALL_v3_rep_c97325	3	14	-	-	gb|EEZ99979.1|	hypothetical protein TcasGA2_TC002778 [Tribolium castaneum]		1,00E-16
gALL_v3_rep_c97335	3	20	-	-	ref|XP_001277988.1|	hypothetical protein [Trichomonas vaginalis G3] gb|EAX65058.1| hypothetical protein TVAG_569780 [Trichomonas vaginalis G3]		8,00E-06
gALL_v3_rep_c97352	4	20	gnl|CDD|128336	smart00020, Tryp_SPc, Trypsir	ref|XP_968658.1|	PREDICTED: similar to prophenoloxidase activating factor [Tribolium castaneum] gb|EEZ99181.1| serine protease H2 [Tribolium castaneum]		1,00E-133
gALL_v3_rep_c97380	4	17	gnl|CDD|31184	COG0842, COG0842, ABC-type	ref|XP_312290.4|	AGAP002638-PA [Anopheles gambiae str. PEST] gb|EAA08206.4| AGAP002638-PA [Anopheles gambiae str. PEST]		7,00E-60
gALL_v3_rep_c97393	3	20	gnl|CDD|178337	PLN02736, PLN02736, long-ch	gb|EGI67017.1|	Long-chain-fatty-acid--CoA ligase 1 [Acromyrmex echinatior]		1,00E-124
gALL_v3_rep_c97400	1	18	gnl|CDD|31588	COG1398, OLE1, Fatty-acid de:	gb|EFZ14309.1|	hypothetical protein SINV_09878 [Solenopsis invicta]		2,00E-62
gALL_v3_rep_c97414	2	20	-	-	-	-	-	
gALL_v3_rep_c97415	3	14	gnl|CDD|191810	pfam07679, I-set, Immunoglol	gb|EFA11121.1|	hypothetical protein TcasGA2_TC004721 [Tribolium castaneum]		2,00E-89
gALL_v3_rep_c97433	9	29	-	-	-	-	-	
gALL_v3_rep_c97456	9	29	gnl|CDD|189372	pfam00067, p450, Cytochrom	gb|AAC03111.2|	family 4 cytochrome P450 [Coptotermes acinaciformis]		1,00E-28
gALL_v3_rep_c97477	1	13	-	-	-	-	-	
gALL_v3_rep_c97481	6	31	gnl|CDD|29138	cd00176, SPEC, Spectrin repea	ref|XP_002430009.1|	Spectrin alpha chain, putative [Pediculus humanus corporis] gb|EEB17271.1| Spectrin alpha chain, putative [Pediculus humanus corporis]		0
gALL_v3_rep_c97492	1	13	gnl|CDD|189372	pfam00067, p450, Cytochrom	gb|AFI45015.1|	cytochrome P450 CYP49a1 [Dendroctonus ponderosae]		3,00E-30
gALL_v3_rep_c97499	1	15	-	-	-	-	-	
gALL_v3_rep_c97506	4	18	gnl|CDD|132718	cd07067, HP_PGM_like, Histid	ref|XP_003700618.1|	PREDICTED: 6-phosphofructo-2-kinase/fructose-2,6-bisphosphatase 1-like [Megachile rotundata]		2,00E-52
gALL_v3_rep_c97508	10	36	-	-	gb|AGM32701.1|	hypothetical protein [Coptotermes formosanus]		1,00E-49
gALL_v3_rep_c97514	1	11	gnl|CDD|191810	pfam07679, I-set, Immunoglol	ref|XP_002431700.1|	myosin light chain kinase, putative [Pediculus humanus corporis] gb|EEB18962.1| myosin light chain kinase, putative [Pediculus humanus corporis]		3,00E-53
gALL_v3_rep_c97518	2	14	-	-	gb|EFA01440.1|	hypothetical protein TcasGA2_TC030701 [Tribolium castaneum]		8,00E-18
gALL_v3_rep_c97557	6	34	-	-	-	-	-	
gALL_v3_rep_c97575	1	14	gnl|CDD|191810	pfam07679, I-set, Immunoglol	gb|EFN83272.1|	Titin [Harpegnathos saltator]		1,00E-82
gALL_v3_rep_c97586	9	24	-	-	-	-	-	
gALL_v3_rep_c97616	3	17	-	-	-	-	-	
gALL_v3_rep_c97625	3	13	-	-	ref|XP_971793.1|	PREDICTED: similar to zormin CG33484-PD [Tribolium castaneum]		2,00E-58
gALL_v3_rep_c97645	2	14	-	-	ref|XP_002425922.1|	Nesprin-1, putative [Pediculus humanus corporis] gb|EEB13184.1| Nesprin-1, putative [Pediculus humanus corporis]		2,00E-63
gALL_v3_rep_c97671	2	12	-	-	-	-	-	
gALL_v3_rep_c97680	6	27	gnl|CDD|189653	pfam00650, CRAL_TRIO, CRAL	ref|XP_968820.1|	PREDICTED: similar to alpha-tocopherol transfer protein [Tribolium castaneum] gb|EFA03949.1| hypothetical protein TcasGA2_TC014093 [Tribolium castaneum]		3,00E-93
gALL_v3_rep_c97692	2	11	gnl|CDD|162108	TIGR00920, 2A060605, 3-hydr	dbj|BAM45097.1|	3-hydroxy-3-methylglutaryl coenzyme A reductase [Nasutitermes takasagoensis]		2,00E-79
gALL_v3_rep_c97739	1	9	-	-	gb|EFN83270.1|	Myosin light chain kinase, smooth muscle [Harpegnathos saltator]		5,00E-59
gALL_v3_rep_c97756	5	29	-	-	ref|XP_001949072.2|	PREDICTED: fatty acid synthase-like [Acyrthosiphon pisum]		5,00E-34
gALL_v3_rep_c97762	1	12	-	-	-	-	-	
gALL_v3_rep_c97772	4	20	gnl|CDD|189659	pfam00658, PABP, Poly-adenyl	ref|XP_975975.1|	PREDICTED: similar to poly A binding protein isoform 4 [Tribolium castaneum] gb|EFA11220.1| hypothetical protein TcasGA2_TC005185 [Tribolium castaneum]		5,00E-21
gALL_v3_rep_c97792	1	11	-	-	-	-	-	
gALL_v3_rep_c97805	14	33	-	-	-	-	-	
gALL_v3_rep_c97829	1	10	gnl|CDD|189793	pfam00992, Troponin, Troponi	gb|AGM32376.1|	troponin I [Coptotermes formosanus]		1,00E-54
gALL_v3_rep_c97833	1	9	gnl|CDD|190438	pfam02815, MIR, MIR domain	ref|XP_002424547.1|	Ryanodine receptor, putative [Pediculus humanus corporis] gb|EEB11809.1| Ryanodine receptor, putative [Pediculus humanus corporis]		3,00E-75
gALL_v3_rep_c97835	6	19	-	-	ref|XP_006807855.1|	PREDICTED: crossover junction endonuclease MUS81-like [Neolamprologus brichardi]		9,00E-06
gALL_v3_rep_c97845	2	12	-	-	-	-	-	
gALL_v3_rep_c97846	6	20	-	-	ref|XP_006621881.1|	PREDICTED: uncharacterized protein LOC102678313 [Apis dorsata]		2,00E-41
gALL_v3_rep_c97852	2	14	gnl|CDD|29138	cd00176, SPEC, Spectrin repea	gb|EFN65250.1|	Nesprin-1 [Camponotus floridanus]		4,00E-61
gALL_v3_rep_c97905	3	14	gnl|CDD|128796	smart00523, DWA, Domain A i	ref|XP_001947078.2|	PREDICTED: nuclear factor 1 X-type-like [Acyrthosiphon pisum]		1,00E-125
gALL_v3_rep_c97907	5	24	gnl|CDD|129058	smart00825, PKS_KS, Beta-ket	ref|XP_001605685.1|	PREDICTED: fatty acid synthase-like [Nasonia vitripennis]		1,00E-162
gALL_v3_rep_c97909	1	9	-	-	gb|EFN70690.1|	Histone-lysine N-methyltransferase SETMAR [Camponotus floridanus]		6,00E-13
gALL_v3_rep_c97914	1	10	-	-	-	-	-	
gALL_v3_rep_c97927	1	9	gnl|CDD|144327	pfam00685, Sulfotransfer_1, S	ref|XP_002432901.1|	conserved hypothetical protein [Pediculus humanus corporis] gb|EEB20163.1| conserved hypothetical protein [Pediculus humanus corporis]		2,00E-51
gALL_v3_rep_c97983	1	9	-	-	-	-	-	
gALL_v3_rep_c98023	1	10	-	-	-	-	-	
gALL_v3_rep_c98035	1	10	gnl|CDD|176349	cd01273, CED-6, CED-6 Phospl	gb|EGI58300.1|	PTB domain-containing adapter protein ced-6 [Acromyrmex echinatior]		3,00E-74
gALL_v3_rep_c98105	9	42	gnl|CDD|189372	pfam00067, p450, Cytochrom	gb|AAO20251.1|	cytochrome P450 monooxygenase CYP4G19 [Blattella germanica]		1,00E-169
gALL_v3_rep_c98146	1	10	-	-	-	-	-	
gALL_v3_rep_c98152	9	32	gnl|CDD|163023	TIGR02794, tolA_full, TolA pro	ref|XP_002431799.1|	conserved hypothetical protein [Pediculus humanus corporis] gb|EEB19061.1| conserved hypothetical protein [Pediculus humanus corporis]		4,00E-43
gALL_v3_rep_c98167	2	11	-	-	-	-	-	
gALL_v3_rep_c98175	2	20	-	-	ref|XP_001277988.1|	hypothetical protein [Trichomonas vaginalis G3] gb|EAX65058.1| hypothetical protein TVAG_569780 [Trichomonas vaginalis G3]		4,00E-06
gALL_v3_rep_c98270	5	23	-	-	ref|XP_002431867.1|	reverse transcriptase, putative [Pediculus humanus corporis] gb|EEB19129.1| reverse transcriptase, putative [Pediculus humanus corporis]		7,00E-22
gALL_v3_rep_c98355	1	9	-	-	-	-	-	
gALL_v3_rep_c98489	1	10	gnl|CDD|176732	cd08317, Death_ank, Death d	ref|XP_001606081.2|	PREDICTED: hypothetical protein LOC100122475 [Nasonia vitripennis]		3,00E-60
gALL_v3_rep_c98579	9	26	-	-	ref|XP_002609525.1|	hypothetical protein BRAFLDRAFT_232052 [Branchiostoma floridae] gb|EEN65535.1| hypothetical protein BRAFLDRAFT_232052 [Branchiostoma floridae]		2,00E-06
gALL_v3_rep_c98602	10	26	gnl|CDD|187665	cd09805, type2_17beta_HSD-	gb|AGM32818.1|	putative corticosteroid 11-beta-dehydrogenase, partial [Coptotermes formosanus]		5,00E-94
gALL_v3_rep_c98615	4	21	-	-	-	-	-	
gALL_v3_rep_c98893	1	12	-	-	-	-	-	
gALL_v3_rep_c99068	2	14	-	-	-	-	-	
gALL_v3_rep_c99192	1	13	gnl|CDD|162209	TIGR01116, ATPase-IIA1_Ca, s	gb|EGI60476.1|	Calcium-transporting ATPase sarcoplasmic/endoplasmic reticulum type [Acromyrmex echinatior]		1,00E-123
gALL_v3_rep_c99247	10	26	gnl|CDD|189372	pfam00067, p450, Cytochrom	gb|AAO20251.1|	cytochrome P450 monooxygenase CYP4G19 [Blattella germanica]		1,00E-113
gALL_v3_rep_c99317	18	50	-	-	-	-	-	
gALL_v3_rep_c99404	6	19	-	-	-	-	-	
gALL_v3_rep_c99479	1	9	-	-	-	-	-	
gALL_v3_rep_c99649	1	9	-	-	ref|XP_003737382.1|	PREDICTED: uncharacterized protein LOC100904063 [Metaseiulus occidentalis]		4,00E-52
gALL_v3_rep_c99728	9	24	-	-	ref|XP_003488805.1|	PREDICTED: myosin-X-like [Bombus impatiens]		8,00E-10
gALL_v3_rep_c99861	1	14	-	-	ref|XP_002429033.1|	reverse transcriptase, putative [Pediculus humanus corporis] gb|EEB16295.1| reverse transcriptase, putative [Pediculus humanus corporis]		2,00E-24
gALL_v3_c1	8	7	gnl|CDD|189970	pfam01395, PBP_GOBP, PBP/G	gb|AFM77983.1|	oderant binding protein 5 [Spodoptera exigua]		2,00E-19
gALL_v3_c10	10	14	-	-	ref|XP_002424807.1|	hect E3 ubiquitin ligase, putative [Pediculus humanus corporis] gb|EEB12069.1| hect E3 ubiquitin ligase, putative [Pediculus humanus corporis]		4,00E-76
gALL_v3_c100	12	14	-	-	ref|XP_002426192.1|	hypothetical protein Phum_PHUM240780 [Pediculus humanus corporis] gb|EEB13454.1| hypothetical protein Phum_PHUM240780 [Pediculus humanus corporis]		5,00E-70
gALL_v3_c1000	4	11	-	-	-	-	-	
gALL_v3_c10000	1	6	-	-	-	-	-	
gALL_v3_c10001	1	4	-	-	-	-	-	
gALL_v3_c10002	11	8	-	-	gb|EFN88085.1|	Zinc transporter 1 [Harpegnathos saltator]		1,00E-14
gALL_v3_c10003	1	5	gnl|CDD|30859	COG0513, SrmB, Superfamily I	ref|XP_004929426.1|	PREDICTED: probable ATP-dependent RNA helicase DDX28-like [Bombyx mori]		3,00E-73
gALL_v3_c10005	5	2	gnl|CDD|152009	pfam11573, Med23, Mediator	gb|EFN85808.1|	Mediator of RNA polymerase II transcription subunit 23 [Harpegnathos saltator]		1,00E-108
gALL_v3_c10006	5	2	-	-	ref|XP_002428038.1|	reverse transcriptase, putative [Pediculus humanus corporis] gb|EEB15300.1| reverse transcriptase, putative [Pediculus humanus corporis]		3,00E-32
gALL_v3_c10008	4	3	gnl|CDD|143992	pfam00241, Cofilin_ADF, Cofili	gb|ADA79536.1|	actin depolymerization factor [Pieris rapae]		5,00E-22
gALL_v3_c10009	1	6	-	-	ref|XP_003488187.1|	PREDICTED: LOW QUALITY PROTEIN: protein strawberry notch-like [Bombus impatiens]		1,00E-66
gALL_v3_c10010	8	5	-	-	ref|XP_003424573.1|	PREDICTED: hypothetical protein LOC100679916 [Nasonia vitripennis]		1,00E-68
gALL_v3_c10011	1	6	-	-	-	-	-	
gALL_v3_c10013	4	4	-	-	-	-	-	
gALL_v3_c10014	8	15	-	-	dbj|BAD84176.1|	cytochrome P450 [Hodotermopsis sjostedti]		2,00E-15
gALL_v3_c10017	5	3	gnl|CDD|129972	TIGR00894, 2A0114euk, Na(+)	ref|XP_001607837.2|	PREDICTED: putative inorganic phosphate cotransporter-like [Nasonia vitripennis]		9,00E-81
gALL_v3_c10018	5	5	-	-	-	-	-	
gALL_v3_c10019	9	11	-	-	ref|XP_002425320.1|	Brefeldin A-inhibited guanine nucleotide-exchange protein, putative [Pediculus humanus corporis] gb|EEB12582.1| Brefeldin A-inhibited guanine nucleotide-exchange prot		4,00E-27
gALL_v3_c1002	15	10	-	-	-	-	-	
gALL_v3_c10020	3	5	-	-	-	-	-	
gALL_v3_c10021	8	4	gnl|CDD|30526	COG0177, Nth, Predicted End	ref|XP_001949525.2|	PREDICTED: endonuclease III-like protein 1-like [Acyrthosiphon pisum]		6,00E-93
gALL_v3_c10023	5	16	-	-	-	-	-	
gALL_v3_c10025	5	6	-	-	-	-	-	
gALL_v3_c10026	4	6	gnl|CDD|177883	PLN02240, PLN02240, UDP-glu	ref|XP_968616.1|	PREDICTED: similar to UDP-glucose 4-epimerase [Tribolium castaneum] gb|EFA06670.1| hypothetical protein TcasGA2_TC009598 [Tribolium castaneum]		4,00E-99
gALL_v3_c10027	7	8	-	-	ref|XP_006614470.1|	PREDICTED: calpain-B-like isoform X9 [Apis dorsata] ref|XP_006614471.1| PREDICTED: calpain-B-like isoform X10 [Apis dorsata]		4,00E-36
gALL_v3_c10029	1	4	-	-	gb|EOB06275.1|	Ankyrin repeat domain-containing protein 49, partial [Anas platyrhynchos]		6,00E-15
gALL_v3_c1003	2	6	-	-	-	-	-	
gALL_v3_c10030	2	6	-	-	-	-	-	
gALL_v3_c10031	2	4	gnl|CDD|29802	cd00862, ProRS_anticodon_zi	ref|XP_005182957.1|	PREDICTED: bifunctional glutamate/proline--tRNA ligase-like isoform X1 [Musca domestica] ref|XP_005182958.1| PREDICTED: bifunctional glutamate/proline--tRNA ligase-		1,00E-61
gALL_v3_c10032	7	10	-	-	-	-	-	
gALL_v3_c10033	3	4	-	-	-	-	-	
gALL_v3_c10034	4	7	gnl|CDD|58167	cd00886, MogA_MoaB, MogA	pdb|1IHC|A	Chain A, X-Ray Structure Of Gephyrin N-Terminal Domain		2,00E-42
gALL_v3_c10035	6	8	-	-	-	-	-	
gALL_v3_c10036	4	6	-	-	-	-	-	
gALL_v3_c10038	2	4	-	-	-	-	-	
gALL_v3_c10039	4	3	-	-	-	-	-	
gALL_v3_c1004	10	10	gnl|CDD|190051	pfam01603, B56, Protein phos	ref|XP_002425888.1|	serine/threonine-protein phosphatase 2A 56 kDa regulatory subunit delta isoform, putative [Pediculus humanus corporis] gb|EEB13150.1| serine/threonine-protein phosp		3,00E-56
gALL_v3_c10041	4	2	-	-	gb|EFZ15151.1|	hypothetical protein SINV_06003 [Solenopsis invicta]		2,00E-41
gALL_v3_c10042	13	26	-	-	-	-	-	
gALL_v3_c10043	3	5	-	-	-	-	-	
gALL_v3_c10044	1	6	-	-	-	-	-	
gALL_v3_c10045	3	3	-	-	-	-	-	
gALL_v3_c10046	16	18	-	-	-	-	-	
gALL_v3_c10047	0	6	-	-	-	-	-	
gALL_v3_c10048	9	12	-	-	ref|XP_003706544.1|	PREDICTED: uncharacterized protein LOC100879796 [Megachile rotundata]		7,00E-62
gALL_v3_c10049	4	4	-	-	-	-	-	
gALL_v3_c10051	8	9	-	-	-	-	-	
gALL_v3_c10053	3	1	-	-	-	-	-	
gALL_v3_c10054	6	4	gnl|CDD|31425	COG1232, HemY, Protoporphy	ref|NP_651278.2|	protoporphyrinogen oxidase [Drosophila melanogaster] gb|AAF56323.1| protoporphyrinogen oxidase [Drosophila melanogaster] gb|AAL13924.1| LD41392p [Drosophila r		2,00E-69
gALL_v3_c10055	4	2	gnl|CDD|153274	cd07590, BAR_Bin3, The Bin/A	ref|XP_003400664.1|	PREDICTED: bridging integrator 3 homolog [Bombus terrestris]		5,00E-35
gALL_v3_c10056	11	8	-	-	-	-	-	
gALL_v3_c10057	5	5	-	-	-	-	-	
gALL_v3_c10058	8	9	-	-	gb|ADD24484.1|	Transposable element Tc3 transposase [Lepeophtheirus salmonis]		1,00E-39
gALL_v3_c10059	13	24	-	-	ref|XP_006596134.1|	PREDICTED: endonuclease V isoform X3 [Glycine max]		1,00E-13
gALL_v3_c1006	20	15	gnl|CDD|133336	cd04136, Rap_like, Rap-like su	ref|XP_396692.3|	PREDICTED: roughened isoform X3 [Apis mellifera] ref|XP_003397118.1| PREDICTED: ras-related protein Rap-1b-like [Bombus terrestris] ref|XP_003488045.1| PREDICTED		1,00E-106
gALL_v3_c10060	8	10	-	-	ref|XP_003706679.1|	PREDICTED: dystrophin, isoforms A/C/F/G/H-like [Megachile rotundata]		8,00E-06
gALL_v3_c10061	5	2	-	-	ref|XP_004930031.1|	PREDICTED: leucine-rich PPR motif-containing protein, mitochondrial-like [Bombyx mori]		1,00E-34
gALL_v3_c10062	8	8	-	-	-	-	-	
gALL_v3_c10064	4	5	gnl|CDD|150878	pfam10272, Tmpp129, Putativ	gb|EFN69753.1|	Transmembrane protein 129 [Camponotus floridanus]		1,00E-103
gALL_v3_c10065	1	7	-	-	-	-	-	
gALL_v3_c10066	0	6	-	-	-	-	-	
gALL_v3_c10067	10	18	-	-	gb|AHB17950.1|	early growth response 2 [Locusta migratoria]		4,00E-29
gALL_v3_c10068	8	5	gnl|CDD|150314	pfam09606, Med15, ARC105 c	gb|EFX72834.1|	hypothetical protein DAPPUDRAFT_215757 [Daphnia pulex]		7,00E-49
gALL_v3_c1007	24	16	gnl|CDD|58652	cd00928, Cyt_c_Oxidase_VIIa,	gb|AGM32612.1|	cytochrome c oxidase subunit VIIa [Coptotermes formosanus]		4,00E-75
gALL_v3_c10070	4	6	-	-	ref|XP_003427394.1|	PREDICTED: phosphatidylcholine:ceramide cholinephosphotransferase 1-like isoform 2 [Nasonia vitripennis]		1,00E-123
gALL_v3_c10071	9	11	gnl|CDD|88597	cd05797, Ribosomal_L10, Ribc	ref|XP_969776.1|	PREDICTED: similar to GA11028-PA [Tribolium castaneum] gb|EFA04041.1| hypothetical protein TcasGA2_TC014272 [Tribolium castaneum]		4,00E-49
gALL_v3_c10073	10	15	-	-	ref|XP_003485172.1|	PREDICTED: hypothetical protein LOC100744278 [Bombus impatiens]		5,00E-31
gALL_v3_c100731	1	1	gnl|CDD|173456	PTZ00169, PTZ00169, ADP/ATI	gb|AGM32994.1|	ADP/ATP translocase [Coptotermes formosanus]		3,00E-43
gALL_v3_c10075	3	3	-	-	gb|EGI62261.1|	Zinc finger protein ush [Acromyrmex echinatior]		1,00E-11
gALL_v3_c10076	4	4	-	-	dbj|BAE44464.1|	non-LTR retrotransposon CATS [Bombyx mori]		8,00E-30

gALL_v3_c10077	14	9	gnl|CDD|58171	cd03505, Delta9-FADS-like, Th	ref|NP_001274329.1|	acyl-CoA Delta(11) desaturase-like [Bombyx mori] gb|AGO45855.1| acyl-CoA desaturase BmorPDSN [Bombyx mori]	3,00E-30
gALL_v3_c10078	1	4	-	-	-	-	-
gALL_v3_c10079	3	8	gnl|CDD|177580	PHA03307, PHA03307, transcr	emb|CCD14593.1|	unnamed protein product, partial [Trypanosoma congolense IL3000]	7,00E-11
gALL_v3_c10080	5	2	gnl|CDD|29259	cd00202, ZnF_GATA, Zinc finge	ref|XP_001943307.2|	PREDICTED: hypothetical protein LOC100166174 isoform 1 [Acyrthosiphon pisum] ref|XP_003247419.1| PREDICTED: hypothetical protein LOC100166174 isoform 2 [Acyrth	3,00E-53
gALL_v3_c10082	4	3	-	-	-	-	-
gALL_v3_c10084	5	9	-	-	-	-	-
gALL_v3_c10085	3	6	gnl|CDD|192559	pfam10374, EST1, Telomerase	ref|XP_003708139.1|	PREDICTED: uncharacterized protein LOC100880698 [Megachile rotundata]	3,00E-77
gALL_v3_c10086	1	6	-	-	-	-	-
gALL_v3_c10087	16	18	-	-	-	-	-
gALL_v3_c10088	5	8	gnl|CDD|30957	COG0612, PqqL, Predicted Zn-	gb|AGM32915.1|	mitochondrial-processing peptidase subunit alpha [Coptotermes formosanus]	1,00E-113
gALL_v3_c10089	5	5	gnl|CDD|73154	cd01647, RT_LTR, RT_LTR: Rev	gb|ABE26654.1|	pol polyprotein [Nosema bombycis]	3,00E-19
gALL_v3_c1009	4	5	gnl|CDD|72972	cd03213, ABCG_EPDR, ABCG t	ref|XP_002429859.1|	ABC transporter, putative [Pediculus humanus corporis] gb|EEB17121.1| ABC transporter, putative [Pediculus humanus corporis]	4,00E-29
gALL_v3_c10090	3	7	-	-	-	-	-
gALL_v3_c10093	3	3	-	-	-	-	-
gALL_v3_c10094	2	5	-	-	gb|ADI61823.1|	endonuclease-reverse transcriptase [Bombyx mori]	4,00E-41
gALL_v3_c10095	14	14	gnl|CDD|128409	smart00098, alkPPc, Alkaline p	gb|EFN82413.1|	Alkaline phosphatase 4 [Harpegnathos saltator]	3,00E-99
gALL_v3_c10096	4	6	-	-	gb|EFN79325.1|	Probable phosphoenolpyruvate synthase [Harpegnathos saltator]	6,00E-25
gALL_v3_c10097	4	3	gnl|CDD|191200	pfam05132, RNA_pol_Rpc4, R	ref|XP_003704604.1|	PREDICTED: DNA-directed RNA polymerase III subunit RPC4-like [Megachile rotundata]	5,00E-17
gALL_v3_c10098	7	6	gnl|CDD|191987	pfam08285, DPM3, Dolichol-p	gb|ETN62497.1|	hypothetical protein AND_005819 [Anopheles darlingi]	1,00E-21
gALL_v3_c10099	3	2	-	-	-	-	-
gALL_v3_c101	2	9	-	-	-	-	-
gALL_v3_c1010	5	4	gnl|CDD|176897	cd08888, SRPBCC_PITPNA-B_l	ref|XP_004529292.1|	PREDICTED: phosphatidylinositol transfer protein alpha isoform-like isoform X2 [Ceratitis capitata]	4,00E-75
gALL_v3_c10100	2	6	-	-	-	-	-
gALL_v3_c10101	5	5	-	-	-	-	-
gALL_v3_c10102	4	4	-	-	-	-	-
gALL_v3_c10103	7	6	-	-	ref|XP_001601132.2|	PREDICTED: probable RISC-loading complex subunit BRAFLDRAFT_242885-like [Nasonia vitripennis]	6,00E-09
gALL_v3_c10104	3	9	-	-	ref|XP_003424341.1|	PREDICTED: hypothetical protein LOC100119619 isoform 2 [Nasonia vitripennis] ref|XP_001603357.2| PREDICTED: hypothetical protein LOC100119619 isoform 1 [Nasonia	4,00E-42
gALL_v3_c10105	5	4	-	-	-	-	-
gALL_v3_c10106	6	4	-	-	-	-	-
gALL_v3_c10107	2	6	-	-	-	-	-
gALL_v3_c10108	4	4	-	-	-	-	-
gALL_v3_c1011	9	18	-	-	ref|XP_967855.1|	PREDICTED: similar to CG34398 CG34398-PA [Tribolium castaneum]	1,00E-11
gALL_v3_c10110	3	4	-	-	-	-	-
gALL_v3_c10111	5	7	-	-	ref|XP_003689601.1|	PREDICTED: LOW QUALITY PROTEIN: extracellular sulfatase SULF-1 homolog [Apis florea]	5,00E-59
gALL_v3_c10112	5	2	-	-	-	-	-
gALL_v3_c10113	3	7	gnl|CDD|48450	cd03752, proteasome_alpha_	gb|EFZ21978.1|	hypothetical protein SINV_09740 [Solenopsis invicta]	4,00E-78
gALL_v3_c10114	0	5	-	-	-	-	-
gALL_v3_c10116	2	5	-	-	gb|ELU16455.1|	hypothetical protein CAPTEDRAFT_190440 [Capitella teleta]	5,00E-23
gALL_v3_c10117	2	5	gnl|CDD|28959	cd00078, HECTc, HECT domain	ref|XP_001846594.1|	ubiquitin-protein ligase [Culex quinquefasciatus] gb|EDS44085.1| ubiquitin-protein ligase [Culex quinquefasciatus]	8,00E-60
gALL_v3_c10118	6	4	-	-	gb|AAI59127.1|	Unknown (protein for MGC:172468) [Xenopus (Silurana) tropicalis]	3,00E-28
gALL_v3_c10119	4	4	-	-	-	-	-
gALL_v3_c1012	2	7	-	-	-	-	-
gALL_v3_c10120	7	15	-	-	ref|XP_003394571.1|	PREDICTED: hypothetical protein LOC100648816 [Bombus terrestris]	9,00E-15
gALL_v3_c10121	3	12	-	-	-	-	-
gALL_v3_c10122	4	2	-	-	-	-	-
gALL_v3_c10123	3	8	-	-	ref|XP_003699685.1|	PREDICTED: tudor domain-containing protein 1-like [Megachile rotundata]	2,00E-20
gALL_v3_c10124	4	2	-	-	-	-	-
gALL_v3_c10125	3	5	-	-	-	-	-
gALL_v3_c10126	4	3	-	-	gb|EEZ99468.1|	hypothetical protein TcasGA2_TC000039 [Tribolium castaneum]	5,00E-18
gALL_v3_c10128	2	5	-	-	-	-	-
gALL_v3_c1013	4	4	gnl|CDD|113282	pfam04506, Rft-1, Rft protein.	ref|XP_001944399.2|	PREDICTED: protein RFT1 homolog [Acyrthosiphon pisum]	2,00E-51
gALL_v3_c10130	2	8	-	-	ref|XP_003700382.1|	PREDICTED: beclin 1-associated autophagy-related key regulator-like [Megachile rotundata]	8,00E-56
gALL_v3_c10131	6	3	-	-	-	-	-
gALL_v3_c10132	1	3	gnl|CDD|162266	TIGR01241, FtsH_fam, ATP-de	gb|AGT62470.1|	paraplegin, partial [Bicyclus anynana]	2,00E-61
gALL_v3_c10134	4	8	-	-	ref|XP_971835.2|	PREDICTED: similar to Inositol 1,4,5,-tris-phosphate receptor CG1063-PA [Tribolium castaneum]	1,00E-127
gALL_v3_c10135	2	3	-	-	gb|EFA09853.1|	hypothetical protein TcasGA2_TC012001 [Tribolium castaneum]	3,00E-41
gALL_v3_c101353	1	1	-	-	-	-	-
gALL_v3_c10136	2	3	-	-	-	-	-
gALL_v3_c10137	3	4	-	-	-	-	-
gALL_v3_c10138	6	4	-	-	ref|XP_002431679.1|	conserved hypothetical protein [Pediculus humanus corporis] gb|EEB18941.1| conserved hypothetical protein [Pediculus humanus corporis]	6,00E-24
gALL_v3_c10139	8	9	-	-	-	-	-
gALL_v3_c1014	6	5	-	-	gb|EFN64790.1|	Lipoma-preferred partner-like protein [Camponotus floridanus]	3,00E-08
gALL_v3_c10140	2	9	-	-	-	-	-
gALL_v3_c10141	5	5	gnl|CDD|30625	COG0277, GlcD, FAD/FMN-cor	gb|ERL93202.1|	hypothetical protein D910_10499 [Dendroctonus ponderosae]	1,00E-118
gALL_v3_c10142	13	8	-	-	-	-	-
gALL_v3_c10143	8	5	-	-	-	-	-
gALL_v3_c10144	3	2	-	-	-	-	-
gALL_v3_c10145	5	5	-	-	-	-	-
gALL_v3_c10146	3	3	-	-	-	-	-
gALL_v3_c10147	7	9	-	-	-	-	-
gALL_v3_c10148	4	3	-	-	ref|XP_003703405.1|	PREDICTED: uncharacterized protein LOC100875004, partial [Megachile rotundata]	4,00E-69
gALL_v3_c10149	2	5	-	-	-	-	-
gALL_v3_c10150	3	2	-	-	ref|XP_001599507.2|	PREDICTED: hypothetical protein LOC100114520 [Nasonia vitripennis]	6,00E-08
gALL_v3_c10153	7	7	-	-	-	-	-
gALL_v3_c10154	2	4	-	-	-	-	-
gALL_v3_c10155	7	5	gnl|CDD|63849	cd04380, RhoGAP_OCRL1, Rh	gb|EFN73438.1|	Inositol polyphosphate 5-phosphatase OCRL-1 [Camponotus floridanus]	1,00E-108
gALL_v3_c101552	0	3	-	-	-	-	-
gALL_v3_c10156	4	2	-	-	-	-	-
gALL_v3_c10158	5	11	gnl|CDD|128452	smart00147, RasGEF, Guanine	gb|EEZ97837.1|	hypothetical protein TcasGA2_TC000206 [Tribolium castaneum]	1,00E-161
gALL_v3_c10159	4	3	-	-	-	-	-
gALL_v3_c1016	9	13	-	-	-	-	-
gALL_v3_c10160	3	4	-	-	-	-	-
gALL_v3_c10161	4	2	-	-	-	-	-
gALL_v3_c10162	4	5	-	-	ref|XP_002428973.1|	hypothetical protein Phum_PHUM412780 [Pediculus humanus corporis] gb|EEB16235.1| hypothetical protein Phum_PHUM412780 [Pediculus humanus corporis]	2,00E-14
gALL_v3_c10163	3	3	-	-	ref|XP_003494051.1|	PREDICTED: hypothetical protein LOC100742403 [Bombus impatiens]	5,00E-07
gALL_v3_c10166	4	12	-	-	ref|XP_003375245.1|	putative integrase core domain protein [Trichinella spiralis] gb|EFV55218.1| putative integrase core domain protein [Trichinella spiralis]	7,00E-20
gALL_v3_c10167	8	8	-	-	-	-	-
gALL_v3_c10168	4	2	-	-	ref|XP_006619843.1|	PREDICTED: exportin-5-like [Apis dorsata]	2,00E-57
gALL_v3_c10169	7	4	gnl|CDD|34857	COG5260, TRF4, DNA polymer	gb|EFN70754.1|	Poly(A) RNA polymerase protein cid1 [Camponotus floridanus]	4,00E-48
gALL_v3_c1017	52	38	-	-	-	-	-
gALL_v3_c10170	11	14	gnl|CDD|128466	smart00164, TBC, Domain in T	ref|XP_003698752.1|	PREDICTED: TBC1 domain family member 4-like [Apis florea]	0
gALL_v3_c10171	4	5	-	-	-	-	-
gALL_v3_c10172	4	9	-	-	-	-	-
gALL_v3_c101725	0	2	-	-	-	-	-
gALL_v3_c10174	4	4	gnl|CDD|187611	cd05353, hydroxyacyl-CoA-like	ref|XP_001947608.2|	PREDICTED: peroxisomal multifunctional enzyme type 2-like isoform 1 [Acyrthosiphon pisum]	4,00E-56
gALL_v3_c101746	0	3	-	-	-	-	-
gALL_v3_c101747	1	1	-	-	-	-	-
gALL_v3_c10175	5	3	-	-	-	-	-
gALL_v3_c10176	2	5	-	-	-	-	-
gALL_v3_c10177	5	8	gnl|CDD|99927	cd05495, Bromo_cbp_like, Brc	ref|XP_006568897.1|	PREDICTED: LOW QUALITY PROTEIN: histone acetyltransferase p300-like [Apis mellifera]	3,00E-60
gALL_v3_c10178	4	7	-	-	ref|XP_003707110.1|	PREDICTED: protein kibra-like isoform 2 [Megachile rotundata]	1,00E-108
gALL_v3_c10179	4	13	gnl|CDD|88561	cd05382, SCP_GAPR-1_like, SC	ref|XP_001946213.1|	PREDICTED: hypothetical protein LOC100165954 isoform 1 [Acyrthosiphon pisum]	1,00E-103
gALL_v3_c10180	4	4	gnl|CDD|128808	smart00535, RIBOc, Ribonucle	emb|CAX68236.1|	dicer-1 [Blattella germanica]	0
gALL_v3_c101801	0	3	-	-	-	-	-
gALL_v3_c10181	4	5	-	-	ref|XP_975280.1|	PREDICTED: similar to B-cell CLL/lymphoma 11A [Tribolium castaneum]	3,00E-47
gALL_v3_c10183	5	2	-	-	gb|EFN72288.1|	Probable phenylalanyl-tRNA synthetase beta chain [Camponotus floridanus]	8,00E-11
gALL_v3_c10184	5	8	-	-	gb|AGM32136.1|	hypothetical protein [Coptotermes formosanus]	9,00E-11
gALL_v3_c10185	4	2	-	-	-	-	-
gALL_v3_c10186	9	6	-	-	ref|XP_004927506.1|	PREDICTED: uncharacterized protein LOC101744756 [Bombyx mori]	8,00E-18
gALL_v3_c10187	2	9	gnl|CDD|29010	cd00110, LamG, Laminin G do	dbj|BAD91051.1|	Gb2-cadherin [Gryllus bimaculatus]	1,00E-136
gALL_v3_c101875	1	1	-	-	gb|AGM32769.1|	chitin binding peritrophin-A domain containing protein [Coptotermes formosanus]	4,00E-08
gALL_v3_c10188	3	10	-	-	-	-	-
gALL_v3_c101885	1	2	-	-	-	-	-
gALL_v3_c10189	7	11	-	-	-	-	-
gALL_v3_c10190	1	6	-	-	ref|XP_006029730.1|	PREDICTED: histone-lysine N-methyltransferase SETMAR-like [Alligator sinensis]	2,00E-17
gALL_v3_c10191	1	5	-	-	-	-	-
gALL_v3_c10192	4	6	-	-	ref|XP_002404270.1|	zinc finger, C2H2 type, putative [Ixodes scapularis] gb|EEC14865.1| zinc finger, C2H2 type, putative [Ixodes scapularis]	7,00E-14
gALL_v3_c10193	13	16	-	-	gb|EHJ70865.1|	endonuclease-reverse transcriptase [Danaus plexippus]	1,00E-36
gALL_v3_c101931	1	1	-	-	-	-	-
gALL_v3_c10194	6	3	-	-	-	-	-
gALL_v3_c10195	7	15	gnl|CDD|191080	pfam04758, Ribosomal_S30, R	gb|AGM32058.1|	ubiquitin/ribosomal protein S30e fusion protein [Coptotermes formosanus]	2,00E-41
gALL_v3_c10196	3	2	-	-	-	-	-
gALL_v3_c10197	12	24	-	-	ref|XP_003707987.1|	PREDICTED: uncharacterized protein LOC100878272 [Megachile rotundata]	8,00E-29
gALL_v3_c10198	2	4	-	-	-	-	-
gALL_v3_c102	17	11	-	-	-	-	-
gALL_v3_c1020	15	30	-	-	-	-	-
gALL_v3_c10200	9	9	-	-	gb|EFZ20515.1|	hypothetical protein SINV_11226 [Solenopsis invicta]	1,00E-103
gALL_v3_c102005	1	1	-	-	-	-	-
gALL_v3_c102006	0	2	gnl|CDD|30792	COG0443, DnaK, Molecular ch	ref|WP_021962657.1|	chaperone protein DnaK [Mycoplasma sp. CAG:877] emb|CCY79942.1| chaperone protein DnaK [Mycoplasma sp. CAG:877]	4,00E-21
gALL_v3_c10201	9	5	gnl|CDD|145284	pfam02019, WIF, WIF domain.	dbj|BAN21300.1|	conserved hypothetical protein [Riptortus pedestris]	1,00E-152
gALL_v3_c102013	1	1	gnl|CDD|187698	cd09274, RNase_HI_RT_Ty3, T	ref|XP_001810599.1|	PREDICTED: similar to pol polyprotein [Tribolium castaneum]	2,00E-20
gALL_v3_c10202	1	5	-	-	gb|AGM32891.1|	putative pallidin [Coptotermes formosanus]	1,00E-41
gALL_v3_c102020	1	1	-	-	-	-	-
gALL_v3_c102025	0	2	gnl|CDD|187698	cd09274, RNase_HI_RT_Ty3, T	ref|XP_002431501.1|	enzymatic polyprotein, putative [Pediculus humanus corporis] gb|EEB18763.1| enzymatic polyprotein, putative [Pediculus humanus corporis]	5,00E-35
gALL_v3_c102029	0	2	-	-	ref|XP_003742325.1|	PREDICTED: uncharacterized protein LOC100904897 [Metaseiulus occidentalis]	3,00E-23
gALL_v3_c10203	6	5	-	-	-	-	-
gALL_v3_c102045	2	1	-	-	-	-	-
gALL_v3_c10205	3	4	-	-	-	-	-
gALL_v3_c102056	1	1	-	-	-	-	-
gALL_v3_c102057	1	1	-	-	-	-	-
gALL_v3_c10206	2	6	gnl|CDD|162471	TIGR01659, sex-lethal, sex-leth	gb|EFN79874.1|	Protein sex-lethal [Harpegnathos saltator]	1,00E-113
gALL_v3_c102061	0	2	gnl|CDD|73156	cd01650, RT_nLTR_like, RT_nL	ref|XP_003243973.1|	PREDICTED: hypothetical protein LOC100570299 [Acyrthosiphon pisum]	1,00E-21
gALL_v3_c102065	0	2	-	-	-	-	-
gALL_v3_c10207	4	6	gnl|CDD|144872	pfam01434, Peptidase_M41, F	gb|EFX69559.1|	hypothetical protein DAPPUDRAFT_202764 [Daphnia pulex]	6,00E-65

<table>
<tr><td>gALL_v3_c10209</td><td>8</td><td>4</td><td>gnl|CDD|188047</td><td>TIGR00376, TIGR00376, DNA h</td><td>ref|XP_393330.2|</td><td>PREDICTED: regulator of nonsense transcripts 1 isoform X4 [Apis mellifera] ref|XP_003691864.1| PREDICTED: regulator of nonsense transcripts 1-like [Apis florea] ref|XP_(</td><td>0</td></tr>
<tr><td>gALL_v3_c102092</td><td>0</td><td>2</td><td>gnl|CDD|190555</td><td>pfam03184, DDE_1, DDE supe</td><td>ref|XP_002735421.1|</td><td>PREDICTED: tigger transposable element-derived protein 6-like [Saccoglossus kowalevskii]</td><td>4,00E-12</td></tr>
<tr><td>gALL_v3_c1021</td><td>6</td><td>10</td><td>gnl|CDD|128342</td><td>smart00027, EH, Eps15 homol</td><td>gb|EFA00615.1|</td><td>hypothetical protein TcasGA2_TC003490 [Tribolium castaneum]</td><td>9,00E-33</td></tr>
<tr><td>gALL_v3_c10211</td><td>4</td><td>8</td><td>-</td><td>-</td><td>-</td><td>-</td><td>-</td></tr>
<tr><td>gALL_v3_c102119</td><td>0</td><td>2</td><td>-</td><td>-</td><td>-</td><td>-</td><td>-</td></tr>
<tr><td>gALL_v3_c10213</td><td>0</td><td>5</td><td>-</td><td>-</td><td>-</td><td>-</td><td>-</td></tr>
<tr><td>gALL_v3_c10216</td><td>6</td><td>8</td><td>gnl|CDD|30768</td><td>COG0419, SbcC, ATPase involv</td><td>gb|EFZ14539.1|</td><td>hypothetical protein SINV_14642 [Solenopsis invicta]</td><td>3,00E-12</td></tr>
<tr><td>gALL_v3_c10217</td><td>2</td><td>6</td><td>-</td><td>-</td><td>-</td><td>-</td><td>-</td></tr>
<tr><td>gALL_v3_c10218</td><td>2</td><td>3</td><td>gnl|CDD|192518</td><td>pfam10291, muHD, Muniscin (</td><td>ref|XP_002426409.1|</td><td>proline-serine-threonine phosphatase interacting protein, putative [Pediculus humanus corporis] gb|EEB13671.1| proline-serine-threonine phosphatase interacting protei</td><td>3,00E-54</td></tr>
<tr><td>gALL_v3_c102182</td><td>1</td><td>1</td><td>-</td><td>-</td><td>-</td><td>-</td><td>-</td></tr>
<tr><td>gALL_v3_c10219</td><td>1</td><td>6</td><td>-</td><td>-</td><td>ref|XP_003493609.1|</td><td>PREDICTED: protein SZT2-like [Bombus impatiens]</td><td>8,00E-42</td></tr>
<tr><td>gALL_v3_c102191</td><td>1</td><td>1</td><td>-</td><td>-</td><td>-</td><td>-</td><td>-</td></tr>
<tr><td>gALL_v3_c102199</td><td>1</td><td>1</td><td>-</td><td>-</td><td>-</td><td>-</td><td>-</td></tr>
<tr><td>gALL_v3_c10220</td><td>4</td><td>13</td><td>-</td><td>-</td><td>-</td><td>-</td><td>-</td></tr>
<tr><td>gALL_v3_c102203</td><td>0</td><td>2</td><td>-</td><td>-</td><td>-</td><td>-</td><td>-</td></tr>
<tr><td>gALL_v3_c10221</td><td>5</td><td>9</td><td>-</td><td>-</td><td>-</td><td>-</td><td>-</td></tr>
<tr><td>gALL_v3_c10222</td><td>7</td><td>15</td><td>-</td><td>-</td><td>-</td><td>-</td><td>-</td></tr>
<tr><td>gALL_v3_c10223</td><td>9</td><td>14</td><td>gnl|CDD|143860</td><td>pfam00078, RVT_1, Reverse tr</td><td>ref|XP_003723613.1|</td><td>PREDICTED: RNA-directed DNA polymerase from mobile element jockey-like [Strongylocentrotus purpuratus]</td><td>7,00E-34</td></tr>
<tr><td>gALL_v3_c102235</td><td>1</td><td>1</td><td>-</td><td>-</td><td>-</td><td>-</td><td>-</td></tr>
<tr><td>gALL_v3_c102238</td><td>1</td><td>1</td><td>-</td><td>-</td><td>-</td><td>-</td><td>-</td></tr>
<tr><td>gALL_v3_c10224</td><td>3</td><td>5</td><td>-</td><td>-</td><td>-</td><td>-</td><td>-</td></tr>
<tr><td>gALL_v3_c102245</td><td>1</td><td>1</td><td>-</td><td>-</td><td>-</td><td>-</td><td>-</td></tr>
<tr><td>gALL_v3_c102249</td><td>1</td><td>1</td><td>gnl|CDD|173730</td><td>cd06624, STKc_ASK, Catalytic (</td><td>ref|XP_006617157.1|</td><td>PREDICTED: mitogen-activated protein kinase kinase kinase 15-like isoform X4 [Apis dorsata]</td><td>3,00E-18</td></tr>
<tr><td>gALL_v3_c10225</td><td>9</td><td>6</td><td>-</td><td>-</td><td>-</td><td>-</td><td>-</td></tr>
<tr><td>gALL_v3_c102253</td><td>0</td><td>2</td><td>-</td><td>-</td><td>-</td><td>-</td><td>-</td></tr>
<tr><td>gALL_v3_c102259</td><td>1</td><td>1</td><td>-</td><td>-</td><td>-</td><td>-</td><td>-</td></tr>
<tr><td>gALL_v3_c10226</td><td>7</td><td>6</td><td>gnl|CDD|190442</td><td>pfam02823, ATP-synt_DE_N, A</td><td>ref|XP_968285.1|</td><td>PREDICTED: similar to ATP synthase delta chain, mitochondrial [Tribolium castaneum] gb|EFA04639.1| hypothetical protein TcasGA2_TC013931 [Tribolium castaneum]</td><td>7,00E-37</td></tr>
<tr><td>gALL_v3_c102261</td><td>0</td><td>2</td><td>-</td><td>-</td><td>-</td><td>-</td><td>-</td></tr>
<tr><td>gALL_v3_c102271</td><td>1</td><td>1</td><td>-</td><td>-</td><td>-</td><td>-</td><td>-</td></tr>
<tr><td>gALL_v3_c102273</td><td>0</td><td>2</td><td>-</td><td>-</td><td>-</td><td>-</td><td>-</td></tr>
<tr><td>gALL_v3_c10228</td><td>2</td><td>4</td><td>-</td><td>-</td><td>ref|XP_004926835.1|</td><td>PREDICTED: uncharacterized protein LOC101745333 [Bombyx mori]</td><td>1,00E-10</td></tr>
<tr><td>gALL_v3_c102281</td><td>0</td><td>2</td><td>-</td><td>-</td><td>-</td><td>-</td><td>-</td></tr>
<tr><td>gALL_v3_c10229</td><td>13</td><td>8</td><td>gnl|CDD|177660</td><td>PLN00022, PLN00022, electroı</td><td>gb|ABC61672.1|</td><td>cxpwmw03 [Periplaneta americana]</td><td>1,00E-143</td></tr>
<tr><td>gALL_v3_c102297</td><td>0</td><td>2</td><td>-</td><td>-</td><td>-</td><td>-</td><td>-</td></tr>
<tr><td>gALL_v3_c1023</td><td>4</td><td>6</td><td>-</td><td>-</td><td>-</td><td>-</td><td>-</td></tr>
<tr><td>gALL_v3_c10230</td><td>9</td><td>5</td><td>-</td><td>-</td><td>-</td><td>-</td><td>-</td></tr>
<tr><td>gALL_v3_c102300</td><td>0</td><td>2</td><td>-</td><td>-</td><td>ref|XP_006822638.1|</td><td>PREDICTED: RNA-directed DNA polymerase from mobile element jockey-like, partial [Saccoglossus kowalevskii]</td><td>3,00E-12</td></tr>
<tr><td>gALL_v3_c10231</td><td>3</td><td>8</td><td>-</td><td>-</td><td>gb|EEZ98230.1|</td><td>hypothetical protein TcasGA2_TC000669 [Tribolium castaneum]</td><td>7,00E-31</td></tr>
<tr><td>gALL_v3_c102311</td><td>0</td><td>2</td><td>-</td><td>-</td><td>gb|EFN80576.1|</td><td>Putative uncharacterized transposon-derived protein F52C9.6 [Harpegnathos saltator]</td><td>2,00E-10</td></tr>
<tr><td>gALL_v3_c102312</td><td>1</td><td>1</td><td>-</td><td>-</td><td>-</td><td>-</td><td>-</td></tr>
<tr><td>gALL_v3_c102314</td><td>1</td><td>1</td><td>-</td><td>-</td><td>-</td><td>-</td><td>-</td></tr>
<tr><td>gALL_v3_c102324</td><td>0</td><td>3</td><td>-</td><td>-</td><td>-</td><td>-</td><td>-</td></tr>
<tr><td>gALL_v3_c10234</td><td>5</td><td>14</td><td>-</td><td>-</td><td>-</td><td>-</td><td>-</td></tr>
<tr><td>gALL_v3_c102343</td><td>0</td><td>2</td><td>-</td><td>-</td><td>-</td><td>-</td><td>-</td></tr>
<tr><td>gALL_v3_c102348</td><td>1</td><td>1</td><td>-</td><td>-</td><td>gb|ADZ95942.1|</td><td>reverse transcriptase, partial [Oeneis sculda] gb|ADZ95943.1| reverse transcriptase, partial [Oeneis sculda]</td><td>3,00E-12</td></tr>
<tr><td>gALL_v3_c10235</td><td>2</td><td>3</td><td>-</td><td>-</td><td>-</td><td>-</td><td>-</td></tr>
<tr><td>gALL_v3_c102350</td><td>0</td><td>2</td><td>-</td><td>-</td><td>-</td><td>-</td><td>-</td></tr>
<tr><td>gALL_v3_c10236</td><td>8</td><td>13</td><td>-</td><td>-</td><td>-</td><td>-</td><td>-</td></tr>
<tr><td>gALL_v3_c10237</td><td>0</td><td>4</td><td>-</td><td>-</td><td>-</td><td>-</td><td>-</td></tr>
<tr><td>gALL_v3_c102376</td><td>0</td><td>2</td><td>-</td><td>-</td><td>-</td><td>-</td><td>-</td></tr>
<tr><td>gALL_v3_c10238</td><td>5</td><td>6</td><td>gnl|CDD|190578</td><td>pfam03248, Rer1, Rer1 family.</td><td>ref|XP_006608501.1|</td><td>PREDICTED: protein RER1-like isoform X1 [Apis dorsata]</td><td>6,00E-46</td></tr>
<tr><td>gALL_v3_c102380</td><td>0</td><td>2</td><td>-</td><td>-</td><td>gb|EFN67634.1|</td><td>Putative uncharacterized transposon-derived protein F52C9.6 [Camponotus floridanus]</td><td>5,00E-06</td></tr>
<tr><td>gALL_v3_c10239</td><td>8</td><td>14</td><td>gnl|CDD|189974</td><td>pfam01403, Sema, Sema dom</td><td>sp|Q26473.1|SEM1A_S(</td><td>RecName: Full=Semaphorin-1A; AltName: Full=Fasciclin IV; AltName: Full=Fasciclin-4; AltName: Full=Semaphorin-I; Short=Sema I; Flags: Precursor gb|AAA29808.1| fascicli</td><td>0</td></tr>
<tr><td>gALL_v3_c102392</td><td>1</td><td>1</td><td>gnl|CDD|107361</td><td>cd06366, PBP1_GABAb_recep</td><td>ref|XP_006562813.1|</td><td>PREDICTED: uncharacterized protein LOC413596 isoform X1 [Apis mellifera]</td><td>9,00E-65</td></tr>
<tr><td>gALL_v3_c102396</td><td>0</td><td>2</td><td>-</td><td>-</td><td>-</td><td>-</td><td>-</td></tr>
<tr><td>gALL_v3_c1024</td><td>16</td><td>28</td><td>-</td><td>-</td><td>ref|XP_003149243.1|</td><td>hypothetical protein LOAG_13689 [Loa loa] gb|EFO14826.1| hypothetical protein LOAG_13689 [Loa loa]</td><td>1,00E-06</td></tr>
<tr><td>gALL_v3_c10240</td><td>5</td><td>10</td><td>-</td><td>-</td><td>-</td><td>-</td><td>-</td></tr>
<tr><td>gALL_v3_c102401</td><td>1</td><td>1</td><td>-</td><td>-</td><td>-</td><td>-</td><td>-</td></tr>
<tr><td>gALL_v3_c10241</td><td>2</td><td>5</td><td>-</td><td>-</td><td>ref|XP_003246807.1|</td><td>PREDICTED: rho GTPase-activating protein 10-like isoform 2 [Acyrthosiphon pisum]</td><td>1,00E-23</td></tr>
<tr><td>gALL_v3_c10242</td><td>7</td><td>9</td><td>-</td><td>-</td><td>-</td><td>-</td><td>-</td></tr>
<tr><td>gALL_v3_c102421</td><td>0</td><td>2</td><td>-</td><td>-</td><td>-</td><td>-</td><td>-</td></tr>
<tr><td>gALL_v3_c102423</td><td>1</td><td>1</td><td>-</td><td>-</td><td>-</td><td>-</td><td>-</td></tr>
<tr><td>gALL_v3_c102425</td><td>1</td><td>1</td><td>-</td><td>-</td><td>-</td><td>-</td><td>-</td></tr>
<tr><td>gALL_v3_c10243</td><td>6</td><td>7</td><td>-</td><td>-</td><td>gb|EFN65619.1|</td><td>Putative uncharacterized transposon-derived protein F52C9.6 [Camponotus floridanus]</td><td>9,00E-12</td></tr>
<tr><td>gALL_v3_c102433</td><td>0</td><td>2</td><td>-</td><td>-</td><td>-</td><td>-</td><td>-</td></tr>
<tr><td>gALL_v3_c10244</td><td>1</td><td>5</td><td>-</td><td>-</td><td>-</td><td>-</td><td>-</td></tr>
<tr><td>gALL_v3_c102449</td><td>1</td><td>1</td><td>-</td><td>-</td><td>-</td><td>-</td><td>-</td></tr>
<tr><td>gALL_v3_c10245</td><td>4</td><td>10</td><td>-</td><td>-</td><td>ref|XP_003487980.1|</td><td>PREDICTED: hypothetical protein LOC100749908 [Bombus impatiens]</td><td>7,00E-41</td></tr>
<tr><td>gALL_v3_c102456</td><td>0</td><td>2</td><td>-</td><td>-</td><td>-</td><td>-</td><td>-</td></tr>
<tr><td>gALL_v3_c10246</td><td>9</td><td>6</td><td>-</td><td>-</td><td>ref|XP_974922.1|</td><td>PREDICTED: similar to zinc finger protein nocA [Tribolium castaneum] gb|EEZ99225.1| no ocelli [Tribolium castaneum]</td><td>6,00E-09</td></tr>
<tr><td>gALL_v3_c102461</td><td>0</td><td>2</td><td>-</td><td>-</td><td>-</td><td>-</td><td>-</td></tr>
<tr><td>gALL_v3_c102468</td><td>1</td><td>1</td><td>-</td><td>-</td><td>ref|NP_001154177.1|</td><td>zinc finger protein LOC100301947 [Acyrthosiphon pisum]</td><td>7,00E-06</td></tr>
<tr><td>gALL_v3_c10247</td><td>4</td><td>3</td><td>gnl|CDD|133266</td><td>cd01863, Rab18, Rab18 subfar</td><td>ref|XP_005170812.1|</td><td>PREDICTED: ras-related protein Rab-18-B isoform X1 [Danio rerio]</td><td>3,00E-32</td></tr>
<tr><td>gALL_v3_c102472</td><td>0</td><td>2</td><td>-</td><td>-</td><td>-</td><td>-</td><td>-</td></tr>
<tr><td>gALL_v3_c102476</td><td>0</td><td>2</td><td>-</td><td>-</td><td>-</td><td>-</td><td>-</td></tr>
<tr><td>gALL_v3_c10248</td><td>6</td><td>4</td><td>-</td><td>-</td><td>-</td><td>-</td><td>-</td></tr>
<tr><td>gALL_v3_c102483</td><td>1</td><td>1</td><td>-</td><td>-</td><td>-</td><td>-</td><td>-</td></tr>
<tr><td>gALL_v3_c10249</td><td>4</td><td>4</td><td>gnl|CDD|190296</td><td>pfam02383, Syja_N, SacI hom(</td><td>gb|EKC33652.1|</td><td>Phosphatidylinositide phosphatase SAC1 [Crassostrea gigas]</td><td>2,00E-73</td></tr>
<tr><td>gALL_v3_c102492</td><td>0</td><td>2</td><td>-</td><td>-</td><td>-</td><td>-</td><td>-</td></tr>
<tr><td>gALL_v3_c102495</td><td>0</td><td>2</td><td>-</td><td>-</td><td>-</td><td>-</td><td>-</td></tr>
<tr><td>gALL_v3_c1025</td><td>16</td><td>29</td><td>-</td><td>-</td><td>-</td><td>-</td><td>-</td></tr>
<tr><td>gALL_v3_c10250</td><td>3</td><td>3</td><td>-</td><td>-</td><td>-</td><td>-</td><td>-</td></tr>
<tr><td>gALL_v3_c102501</td><td>1</td><td>1</td><td>-</td><td>-</td><td>-</td><td>-</td><td>-</td></tr>
<tr><td>gALL_v3_c102504</td><td>1</td><td>1</td><td>-</td><td>-</td><td>-</td><td>-</td><td>-</td></tr>
<tr><td>gALL_v3_c102507</td><td>1</td><td>1</td><td>-</td><td>-</td><td>-</td><td>-</td><td>-</td></tr>
<tr><td>gALL_v3_c102509</td><td>1</td><td>1</td><td>-</td><td>-</td><td>-</td><td>-</td><td>-</td></tr>
<tr><td>gALL_v3_c10251</td><td>0</td><td>6</td><td>-</td><td>-</td><td>gb|EGI57373.1|</td><td>Mariner Mos1 transposase [Acromyrmex echinatior]</td><td>5,00E-09</td></tr>
<tr><td>gALL_v3_c102517</td><td>1</td><td>1</td><td>-</td><td>-</td><td>-</td><td>-</td><td>-</td></tr>
<tr><td>gALL_v3_c10252</td><td>2</td><td>10</td><td>gnl|CDD|189704</td><td>pfam00757, Furin-like, Furin-li</td><td>ref|XP_001952079.2|</td><td>PREDICTED: insulin-like receptor-like [Acyrthosiphon pisum]</td><td>3,00E-45</td></tr>
<tr><td>gALL_v3_c102521</td><td>1</td><td>1</td><td>-</td><td>-</td><td>-</td><td>-</td><td>-</td></tr>
<tr><td>gALL_v3_c102526</td><td>1</td><td>1</td><td>-</td><td>-</td><td>-</td><td>-</td><td>-</td></tr>
<tr><td>gALL_v3_c102527</td><td>0</td><td>2</td><td>-</td><td>-</td><td>-</td><td>-</td><td>-</td></tr>
<tr><td>gALL_v3_c10253</td><td>3</td><td>7</td><td>-</td><td>-</td><td>-</td><td>-</td><td>-</td></tr>
<tr><td>gALL_v3_c10254</td><td>2</td><td>3</td><td>-</td><td>-</td><td>-</td><td>-</td><td>-</td></tr>
<tr><td>gALL_v3_c102540</td><td>1</td><td>1</td><td>-</td><td>-</td><td>-</td><td>-</td><td>-</td></tr>
<tr><td>gALL_v3_c102543</td><td>1</td><td>1</td><td>-</td><td>-</td><td>gb|ERL91213.1|</td><td>hypothetical protein D910_08551 [Dendroctonus ponderosae]</td><td>9,00E-20</td></tr>
<tr><td>gALL_v3_c102544</td><td>1</td><td>1</td><td>-</td><td>-</td><td>-</td><td>-</td><td>-</td></tr>
<tr><td>gALL_v3_c102545</td><td>1</td><td>1</td><td>-</td><td>-</td><td>-</td><td>-</td><td>-</td></tr>
<tr><td>gALL_v3_c102546</td><td>1</td><td>1</td><td>-</td><td>-</td><td>-</td><td>-</td><td>-</td></tr>
<tr><td>gALL_v3_c10255</td><td>3</td><td>3</td><td>-</td><td>-</td><td>-</td><td>-</td><td>-</td></tr>
<tr><td>gALL_v3_c102556</td><td>1</td><td>1</td><td>-</td><td>-</td><td>ref|XP_001652156.1|</td><td>hypothetical protein AaeL_AAEL006654 [Aedes aegypti] gb|EAT41745.1| AAEL006654-PA [Aedes aegypti]</td><td>3,00E-13</td></tr>
<tr><td>gALL_v3_c10256</td><td>4</td><td>11</td><td>-</td><td>-</td><td>ref|XP_002425076.1|</td><td>conserved hypothetical protein [Pediculus humanus corporis] gb|EEB12338.1| conserved hypothetical protein [Pediculus humanus corporis]</td><td>6,00E-30</td></tr>
<tr><td>gALL_v3_c102564</td><td>0</td><td>2</td><td>-</td><td>-</td><td>-</td><td>-</td><td>-</td></tr>
<tr><td>gALL_v3_c102565</td><td>0</td><td>2</td><td>-</td><td>-</td><td>-</td><td>-</td><td>-</td></tr>
<tr><td>gALL_v3_c10257</td><td>3</td><td>4</td><td>-</td><td>-</td><td>-</td><td>-</td><td>-</td></tr>
<tr><td>gALL_v3_c102575</td><td>1</td><td>1</td><td>-</td><td>-</td><td>-</td><td>-</td><td>-</td></tr>
<tr><td>gALL_v3_c10258</td><td>2</td><td>4</td><td>-</td><td>-</td><td>-</td><td>-</td><td>-</td></tr>
<tr><td>gALL_v3_c102582</td><td>1</td><td>1</td><td>-</td><td>-</td><td>-</td><td>-</td><td>-</td></tr>
<tr><td>gALL_v3_c102587</td><td>0</td><td>2</td><td>-</td><td>-</td><td>-</td><td>-</td><td>-</td></tr>
<tr><td>gALL_v3_c102589</td><td>1</td><td>1</td><td>-</td><td>-</td><td>-</td><td>-</td><td>-</td></tr>
<tr><td>gALL_v3_c102594</td><td>1</td><td>1</td><td>-</td><td>-</td><td>-</td><td>-</td><td>-</td></tr>
<tr><td>gALL_v3_c102595</td><td>0</td><td>2</td><td>-</td><td>-</td><td>-</td><td>-</td><td>-</td></tr>
<tr><td>gALL_v3_c102596</td><td>0</td><td>2</td><td>-</td><td>-</td><td>-</td><td>-</td><td>-</td></tr>
<tr><td>gALL_v3_c1026</td><td>9</td><td>17</td><td>-</td><td>-</td><td>-</td><td>-</td><td>-</td></tr>
<tr><td>gALL_v3_c10260</td><td>6</td><td>5</td><td>-</td><td>-</td><td>-</td><td>-</td><td>-</td></tr>
<tr><td>gALL_v3_c102601</td><td>0</td><td>2</td><td>-</td><td>-</td><td>-</td><td>-</td><td>-</td></tr>
<tr><td>gALL_v3_c102609</td><td>0</td><td>2</td><td>-</td><td>-</td><td>-</td><td>-</td><td>-</td></tr>
<tr><td>gALL_v3_c10261</td><td>4</td><td>12</td><td>-</td><td>-</td><td>-</td><td>-</td><td>-</td></tr>
<tr><td>gALL_v3_c102611</td><td>0</td><td>2</td><td>-</td><td>-</td><td>ref|XP_003492605.1|</td><td>PREDICTED: zinc finger protein 643-like [Bombus impatiens]</td><td>3,00E-46</td></tr>
<tr><td>gALL_v3_c102613</td><td>0</td><td>2</td><td>-</td><td>-</td><td>emb|CAX68236.1|</td><td>dicer-1 [Blattella germanica]</td><td>5,00E-06</td></tr>
<tr><td>gALL_v3_c102616</td><td>0</td><td>2</td><td>-</td><td>-</td><td>-</td><td>-</td><td>-</td></tr>
<tr><td>gALL_v3_c102617</td><td>0</td><td>2</td><td>gnl|CDD|180194</td><td>PRK05678, PRK05678, succiny</td><td>gb|EFZ13224.1|</td><td>hypothetical protein SINV_06825 [Solenopsis invicta]</td><td>3,00E-06</td></tr>
<tr><td>gALL_v3_c102618</td><td>0</td><td>2</td><td>-</td><td>-</td><td>-</td><td>-</td><td>-</td></tr>
<tr><td>gALL_v3_c10262</td><td>5</td><td>4</td><td>gnl|CDD|29151</td><td>cd00189, TPR, Tetratricopeptic</td><td>ref|XP_006558583.1|</td><td>PREDICTED: peroxisomal targeting signal 1 receptor-like isoform X1 [Apis mellifera]</td><td>6,00E-78</td></tr>
<tr><td>gALL_v3_c102624</td><td>1</td><td>1</td><td>-</td><td>-</td><td>-</td><td>-</td><td>-</td></tr>
<tr><td>gALL_v3_c102626</td><td>0</td><td>2</td><td>-</td><td>-</td><td>-</td><td>-</td><td>-</td></tr>
<tr><td>gALL_v3_c102627</td><td>1</td><td>1</td><td>-</td><td>-</td><td>-</td><td>-</td><td>-</td></tr>
<tr><td>gALL_v3_c102628</td><td>1</td><td>1</td><td>-</td><td>-</td><td>ref|XP_002431036.1|</td><td>serine/threonine-protein kinase MARK2, putative [Pediculus humanus corporis] gb|EEB18298.1| serine/threonine-protein kinase MARK2, putative [Pediculus humanus cor</td><td>5,00E-17</td></tr>
<tr><td>gALL_v3_c102629</td><td>0</td><td>2</td><td>-</td><td>-</td><td>-</td><td>-</td><td>-</td></tr>
<tr><td>gALL_v3_c10263</td><td>9</td><td>9</td><td>-</td><td>-</td><td>-</td><td>-</td><td>-</td></tr>
<tr><td>gALL_v3_c102631</td><td>0</td><td>2</td><td>-</td><td>-</td><td>-</td><td>-</td><td>-</td></tr>
<tr><td>gALL_v3_c102633</td><td>0</td><td>2</td><td>-</td><td>-</td><td>-</td><td>-</td><td>-</td></tr>
<tr><td>gALL_v3_c102635</td><td>1</td><td>1</td><td>-</td><td>-</td><td>-</td><td>-</td><td>-</td></tr>
<tr><td>gALL_v3_c102636</td><td>1</td><td>1</td><td>-</td><td>-</td><td>-</td><td>-</td><td>-</td></tr>
<tr><td>gALL_v3_c102639</td><td>1</td><td>1</td><td>-</td><td>-</td><td>-</td><td>-</td><td>-</td></tr>
<tr><td>gALL_v3_c10264</td><td>4</td><td>2</td><td>-</td><td>-</td><td>-</td><td>-</td><td>-</td></tr>
<tr><td>gALL_v3_c102640</td><td>0</td><td>2</td><td>-</td><td>-</td><td>-</td><td>-</td><td>-</td></tr>
<tr><td>gALL_v3_c102641</td><td>1</td><td>1</td><td>-</td><td>-</td><td>-</td><td>-</td><td>-</td></tr>
<tr><td>gALL_v3_c102643</td><td>0</td><td>2</td><td>-</td><td>-</td><td>-</td><td>-</td><td>-</td></tr>
<tr><td>gALL_v3_c102644</td><td>1</td><td>1</td><td>-</td><td>-</td><td>-</td><td>-</td><td>-</td></tr>
<tr><td>gALL_v3_c102645</td><td>1</td><td>1</td><td>-</td><td>-</td><td>-</td><td>-</td><td>-</td></tr>
<tr><td>gALL_v3_c10265</td><td>7</td><td>7</td><td>-</td><td>-</td><td>ref|XP_001948838.1|</td><td>PREDICTED: RNA-binding protein 40-like [Acyrthosiphon pisum]</td><td>5,00E-73</td></tr>
<tr><td>gALL_v3_c102650</td><td>0</td><td>2</td><td>-</td><td>-</td><td>ref|XP_006220985.1|</td><td>PREDICTED: RING finger protein 157-like isoform X1 [Rattus norvegicus]</td><td>6,00E-08</td></tr>
<tr><td>gALL_v3_c102651</td><td>1</td><td>1</td><td>-</td><td>-</td><td>-</td><td>-</td><td>-</td></tr>
</table>

gALL_v3_c102652	0	2	-	-	-	-	-
gALL_v3_c102653	0	2	-	-	-	-	-
gALL_v3_c102656	0	2	-	-	-	-	-
gALL_v3_c10266	2	5	-	-	-	-	-
gALL_v3_c102662	0	2	-	-	-	-	-
gALL_v3_c102664	1	1	-	-	-	-	-
gALL_v3_c102668	0	2	-	-	ref|XP_004530354.1|	PREDICTED: uncharacterized protein LOC101454058 [Ceratitis capitata]	5,00E-17
gALL_v3_c102669	1	1	-	-	-	-	-
gALL_v3_c10267	5	3	-	-	gb|EFB28716.1|	hypothetical protein PANDA_003316 [Ailuropoda melanoleuca]	1,00E-43
gALL_v3_c102673	0	2	-	-	-	-	-
gALL_v3_c102675	0	2	-	-	-	-	-
gALL_v3_c102677	0	2	-	-	-	-	-
gALL_v3_c102678	1	1	-	-	-	-	-
gALL_v3_c102679	0	2	-	-	-	-	-
gALL_v3_c10268	2	5	-	-	-	-	-
gALL_v3_c102683	0	2	gnl|CDD|73156	cd01650, RT_nLTR_like, RT_nL	ref|NP_048132.1|	ORF MSV061 putative LINE reverse transcriptase, similar to Caenorhabditis elegans GB:U00063 [Melanoplus sanguinipes entomopoxvirus] gb|AAC97621.1| ORF MSV061 p	1,00E-21
gALL_v3_c102686	1	1	-	-	-	-	-
gALL_v3_c102689	0	2	-	-	-	-	-
gALL_v3_c10269	3	2	gnl|CDD|109266	pfam00201, UDPGT, UDP-gluc	ref|XP_001945411.1|	PREDICTED: UDP-glucuronosyltransferase 2A1-like [Acyrthosiphon pisum]	2,00E-52
gALL_v3_c102691	1	1	-	-	-	-	-
gALL_v3_c102692	0	2	-	-	-	-	-
gALL_v3_c102695	1	1	-	-	-	-	-
gALL_v3_c102697	1	1	-	-	-	-	-
gALL_v3_c102699	0	2	-	-	-	-	-
gALL_v3_c1027	7	9	gnl|CDD|176344	cd01268, Numb, Numb Phosp	ref|XP_002428818.1|	conserved hypothetical protein [Pediculus humanus corporis] gb|EEB16080.1| conserved hypothetical protein [Pediculus humanus corporis]	1,00E-165
gALL_v3_c10270	8	4	-	-	-	-	-
gALL_v3_c102707	1	1	-	-	-	-	-
gALL_v3_c102710	1	1	-	-	-	-	-
gALL_v3_c102718	0	2	-	-	gb|ADU04477.1|	transposase [Aphis gossypii]	3,00E-17
gALL_v3_c102719	1	1	-	-	ref|XP_003695479.1|	PREDICTED: uncharacterized protein LOC100865708 [Apis florea]	3,00E-27
gALL_v3_c10272	1	5	-	-	-	-	-
gALL_v3_c102722	0	2	-	-	-	-	-
gALL_v3_c102723	0	2	-	-	-	-	-
gALL_v3_c102725	0	2	-	-	-	-	-
gALL_v3_c10273	3	12	-	-	ref|XP_002431695.1|	titin, putative [Pediculus humanus corporis] gb|EEB18957.1| titin, putative [Pediculus humanus corporis]	6,00E-12
gALL_v3_c102731	1	1	-	-	-	-	-
gALL_v3_c102732	1	1	-	-	-	-	-
gALL_v3_c10274	5	3	-	-	-	-	-
gALL_v3_c102740	1	1	-	-	-	-	-
gALL_v3_c102741	0	2	-	-	-	-	-
gALL_v3_c102743	1	1	-	-	-	-	-
gALL_v3_c102744	0	2	-	-	-	-	-
gALL_v3_c102746	0	2	-	-	-	-	-
gALL_v3_c102749	0	2	-	-	-	-	-
gALL_v3_c10275	6	9	-	-	-	-	-
gALL_v3_c102752	1	1	-	-	-	-	-
gALL_v3_c102755	0	2	-	-	-	-	-
gALL_v3_c102757	0	2	-	-	-	-	-
gALL_v3_c102758	0	2	-	-	-	-	-
gALL_v3_c102759	0	2	-	-	-	-	-
gALL_v3_c102761	0	2	-	-	-	-	-
gALL_v3_c102768	1	1	-	-	ref|XP_005184740.1|	PREDICTED: organic cation transporter-like protein-like [Musca domestica]	1,00E-15
gALL_v3_c10277	1	5	-	-	-	-	-
gALL_v3_c102771	1	1	gnl|CDD|162048	TIGR00804, nupC, nucleoside	gb|EFZ17322.1|	hypothetical protein SINV_03962 [Solenopsis invicta]	4,00E-16
gALL_v3_c102773	0	2	-	-	-	-	-
gALL_v3_c102776	0	2	-	-	-	-	-
gALL_v3_c102782	1	1	-	-	-	-	-
gALL_v3_c102783	1	1	-	-	-	-	-
gALL_v3_c102784	1	1	-	-	ref|XP_001330228.1|	TPR Domain containing protein [Trichomonas vaginalis G3] gb|EAY01380.1| TPR Domain containing protein [Trichomonas vaginalis G3]	1,00E-25
gALL_v3_c102787	0	2	-	-	-	-	-
gALL_v3_c10279	3	2	-	-	-	-	-
gALL_v3_c102790	1	1	gnl|CDD|189485	pfam00282, Pyridoxal_deC, Py	ref|XP_001950555.2|	PREDICTED: aromatic-L-amino-acid decarboxylase-like [Acyrthosiphon pisum]	2,00E-51
gALL_v3_c102792	0	2	-	-	-	-	-
gALL_v3_c102793	0	2	-	-	-	-	-
gALL_v3_c102796	0	2	-	-	-	-	-
gALL_v3_c10280	3	6	-	-	-	-	-
gALL_v3_c102802	0	2	-	-	gb|AAK01090.1|	putative BSC1 sodium channel protein [Blattella germanica]	2,00E-31
gALL_v3_c102806	0	2	-	-	-	-	-
gALL_v3_c10281	0	6	-	-	-	-	-
gALL_v3_c102810	0	2	-	-	-	-	-
gALL_v3_c102811	1	1	-	-	ref|XP_005998028.1|	PREDICTED: zinc finger protein 574-like [Latimeria chalumnae]	3,00E-07
gALL_v3_c102812	0	2	-	-	ref|XP_005097750.1|	PREDICTED: baculoviral IAP repeat-containing protein 6-like [Aplysia californica]	7,00E-18
gALL_v3_c102813	1	1	-	-	-	-	-
gALL_v3_c102815	0	2	-	-	-	-	-
gALL_v3_c102816	1	1	-	-	-	-	-
gALL_v3_c102819	0	2	gnl|CDD|73156	cd01650, RT_nLTR_like, RT_nL	ref|XP_006822638.1|	PREDICTED: RNA-directed DNA polymerase from mobile element jockey-like, partial [Saccoglossus kowalevskii]	2,00E-06
gALL_v3_c10282	4	5	-	-	-	-	-
gALL_v3_c102822	0	2	-	-	-	-	-
gALL_v3_c102824	0	2	-	-	ref|XP_002435860.1|	conserved hypothetical protein [Ixodes scapularis] gb|EEC08690.1| conserved hypothetical protein [Ixodes scapularis]	7,00E-23
gALL_v3_c10283	1	5	-	-	-	-	-
gALL_v3_c102839	1	1	-	-	-	-	-
gALL_v3_c102840	1	1	-	-	-	-	-
gALL_v3_c102842	0	2	-	-	-	-	-
gALL_v3_c102849	1	1	gnl|CDD|150639	pfam09989, DUF2229, CoA en	ref|YP_004364745.1|	CoA-substrate-specific enzyme activase [Treponema succinifaciens DSM 2489] ref|WP_013700755.1| 2-hydroxyglutaryl-CoA dehydratase [Treponema succinifaciens] gb|A	2,00E-59
gALL_v3_c10285	2	2	-	-	-	-	-
gALL_v3_c102850	1	1	-	-	-	-	-
gALL_v3_c102851	0	2	-	-	-	-	-
gALL_v3_c102852	0	2	-	-	ref|XP_001602710.2|	PREDICTED: hypothetical protein LOC100118826 [Nasonia vitripennis]	7,00E-07
gALL_v3_c102856	1	1	-	-	-	-	-
gALL_v3_c102857	1	1	-	-	-	-	-
gALL_v3_c102859	1	1	-	-	-	-	-
gALL_v3_c102861	1	1	gnl|CDD|48413	cd02685, MIT_C, MIT_C; doma	ref|XP_004615876.1|	PREDICTED: MIT domain-containing protein 1 [Sorex araneus]	1,00E-18
gALL_v3_c102862	0	2	-	-	-	-	-
gALL_v3_c102870	0	2	-	-	-	-	-
gALL_v3_c102871	1	1	-	-	gb|ERL88036.1|	hypothetical protein D910_05425 [Dendroctonus ponderosae]	5,00E-07
gALL_v3_c102872	1	1	-	-	-	-	-
gALL_v3_c102873	0	2	gnl|CDD|173663	cd05572, STKc_cGK_PKG, Cata	gb|ERL94983.1|	hypothetical protein D910_12255 [Dendroctonus ponderosae]	4,00E-13
gALL_v3_c102887	1	1	-	-	-	-	-
gALL_v3_c102888	1	1	-	-	-	-	-
gALL_v3_c102889	0	2	-	-	-	-	-
gALL_v3_c102890	1	1	-	-	-	-	-
gALL_v3_c102891	0	2	-	-	-	-	-
gALL_v3_c102892	0	2	-	-	-	-	-
gALL_v3_c102894	0	2	-	-	-	-	-
gALL_v3_c102895	1	1	-	-	-	-	-
gALL_v3_c102896	1	1	-	-	-	-	-
gALL_v3_c1029	28	43	-	-	-	-	-
gALL_v3_c10290	7	7	gnl|CDD|190857	pfam04080, Per1, Per1-like. P	ref|XP_974518.2|	PREDICTED: similar to CG3271 CG3271-PB [Tribolium castaneum]	5,00E-91
gALL_v3_c102907	1	1	-	-	-	-	-
gALL_v3_c102908	1	1	gnl|CDD|189434	pfam00176, SNF2_N, SNF2 far	ref|XP_006608416.1|	PREDICTED: SWI/SNF-related matrix-associated actin-dependent regulator of chromatin subfamily A-like protein 1-like [Apis dorsata]	2,00E-35
gALL_v3_c102909	0	2	-	-	-	-	-
gALL_v3_c102911	0	2	-	-	-	-	-
gALL_v3_c102912	1	1	-	-	-	-	-
gALL_v3_c102914	1	1	-	-	-	-	-
gALL_v3_c102915	1	1	-	-	-	-	-
gALL_v3_c102916	1	1	-	-	-	-	-
gALL_v3_c102918	1	1	-	-	-	-	-
gALL_v3_c102919	1	1	-	-	-	-	-
gALL_v3_c10292	1	5	-	-	-	-	-
gALL_v3_c102922	1	1	-	-	-	-	-
gALL_v3_c102924	1	1	-	-	-	-	-
gALL_v3_c102925	1	1	-	-	-	-	-
gALL_v3_c102927	1	1	-	-	-	-	-
gALL_v3_c10293	3	3	gnl|CDD|30087	cd01365, KISc_KIF1A_KIF1B, K	gb|EHJ68621.1|	hypothetical protein KGM_04794 [Danaus plexippus]	3,00E-60
gALL_v3_c102933	0	2	-	-	ref|XP_004530092.1|	PREDICTED: THO complex subunit 3-like [Ceratitis capitata]	1,00E-51
gALL_v3_c102935	0	2	-	-	-	-	-
gALL_v3_c102936	0	2	-	-	-	-	-
gALL_v3_c102938	1	1	-	-	-	-	-
gALL_v3_c102941	0	2	-	-	-	-	-
gALL_v3_c102942	0	2	-	-	-	-	-
gALL_v3_c102944	0	2	-	-	-	-	-
gALL_v3_c102946	1	1	-	-	-	-	-
gALL_v3_c102948	0	2	-	-	-	-	-
gALL_v3_c10295	4	2	-	-	ref|XP_002425925.1|	predicted protein [Pediculus humanus corporis] gb|EEB13187.1| predicted protein [Pediculus humanus corporis]	2,00E-09
gALL_v3_c102951	1	1	-	-	-	-	-
gALL_v3_c102952	0	2	-	-	-	-	-
gALL_v3_c102956	1	1	-	-	-	-	-
gALL_v3_c102957	0	2	-	-	-	-	-
gALL_v3_c102959	0	2	-	-	-	-	-
gALL_v3_c10296	4	6	-	-	-	-	-
gALL_v3_c102965	1	1	-	-	-	-	-
gALL_v3_c102966	1	2	-	-	-	-	-
gALL_v3_c102974	0	2	gnl|CDD|147317	pfam05071, NDUFA12, NADH	ref|XP_002132451.1|	GA25471 [Drosophila pseudoobscura pseudoobscura] gb|EDY69853.1| GA25471 [Drosophila pseudoobscura pseudoobscura]	8,00E-35
gALL_v3_c102978	1	1	-	-	-	-	-

gALL_v3_c10298	7	4	-	-	gb|EFX76820.1|	hypothetical protein DAPPUDRAFT_213835 [Daphnia pulex]	2,00E-09
gALL_v3_c102980	0	2	-	-	-	-	-
gALL_v3_c102981	1	1	-	-	-	-	-
gALL_v3_c102982	1	1	-	-	-	-	-
gALL_v3_c102984	1	1	gnl|CDD|180142	PRK05578, PRK05578, cytidine	gb|AEX32920.1|	cytidine deaminase [Hypophthalmichthys nobilis]	2,00E-12
gALL_v3_c102985	0	2	-	-	ref|XP_006614496.1|	PREDICTED: uncharacterized protein DDB_G0271670-like, partial [Apis dorsata]	5,00E-34
gALL_v3_c102986	0	2	-	-	-	-	-
gALL_v3_c102987	1	1	-	-	-	-	-
gALL_v3_c10299	3	4	gnl|CDD|162054	TIGR00815, sulP, high affinity s	gb|EFZ18919.1|	hypothetical protein SINV_15260 [Solenopsis invicta]	2,00E-55
gALL_v3_c102990	1	1	-	-	-	-	-
gALL_v3_c102991	1	1	-	-	-	-	-
gALL_v3_c102992	1	1	-	-	-	-	-
gALL_v3_c102993	0	2	-	-	ref|XP_003240965.1|	PREDICTED: sodium- and chloride-dependent glycine transporter 2-like isoform 3 [Acyrthosiphon pisum]	9,00E-15
gALL_v3_c103	4	8	-	-	-	-	-
gALL_v3_c1030	8	19	-	-	ref|XP_001814289.1|	PREDICTED: similar to conserved hypothetical protein [Tribolium castaneum] gb|EFA02376.1| hypothetical protein TcasGA2_TC008051 [Tribolium castaneum]	3,00E-45
gALL_v3_c103000	0	2	-	-	-	-	-
gALL_v3_c103001	0	2	-	-	-	-	-
gALL_v3_c103002	1	1	-	-	-	-	-
gALL_v3_c10301	3	5	-	-	-	-	-
gALL_v3_c103011	1	1	-	-	emb|CAZ68905.1|	fruitless [Blattella germanica]	2,00E-21
gALL_v3_c103012	0	2	-	-	-	-	-
gALL_v3_c103016	0	2	gnl|CDD|128489	smart00192, LDLa, Low-densit	dbj|BAE00010.1|	Lipophorin receptor [Rhyparobia maderae]	6,00E-34
gALL_v3_c10302	2	8	gnl|CDD|144435	pfam00839, Cys_rich_FGFR, C	ref|XP_006565308.1|	PREDICTED: Golgi apparatus protein 1-like isoform X2 [Apis mellifera]	1,00E-91
gALL_v3_c103023	0	2	-	-	-	-	-
gALL_v3_c103024	0	2	-	-	-	-	-
gALL_v3_c103026	1	1	-	-	-	-	-
gALL_v3_c103027	1	1	-	-	-	-	-
gALL_v3_c103028	1	1	-	-	-	-	-
gALL_v3_c10303	1	7	-	-	ref|XP_003247373.1|	PREDICTED: lysophospholipid acyltransferase 7-like isoform 2 [Acyrthosiphon pisum] ref|XP_001946232.2| PREDICTED: lysophospholipid acyltransferase 7-like isoform 1 [A	1,00E-53
gALL_v3_c103030	1	1	-	-	-	-	-
gALL_v3_c103034	1	1	-	-	ref|XP_397318.4|	PREDICTED: CDP-diacylglycerol--glycerol-3-phosphate 3-phosphatidyltransferase, mitochondrial-like isoform X2 [Apis mellifera]	2,00E-34
gALL_v3_c103035	1	1	gnl|CDD|145880	pfam02956, TT_ORF1, TT viral	-	-	-
gALL_v3_c103037	1	1	-	-	-	-	-
gALL_v3_c10304	12	7	-	-	-	-	-
gALL_v3_c103040	0	2	-	-	-	-	-
gALL_v3_c103041	0	2	-	-	gb|AAB65093.1|	Lian-Aa1 retrotransposon protein [Aedes aegypti]	1,00E-11
gALL_v3_c103043	0	2	-	-	-	-	-
gALL_v3_c103044	1	1	-	-	-	-	-
gALL_v3_c103048	1	1	-	-	ref|XP_006132930.1|	PREDICTED: jerky protein-like [Pelodiscus sinensis]	3,00E-12
gALL_v3_c103049	0	2	-	-	-	-	-
gALL_v3_c10305	4	4	-	-	-	-	-
gALL_v3_c103050	1	1	-	-	-	-	-
gALL_v3_c103051	1	1	-	-	-	-	-
gALL_v3_c103054	1	1	-	-	-	-	-
gALL_v3_c103055	1	1	-	-	-	-	-
gALL_v3_c103056	0	2	-	-	-	-	-
gALL_v3_c103058	1	1	-	-	gb|ENN81139.1|	hypothetical protein YQE_02506, partial [Dendroctonus ponderosae] gb|ERL84537.1| hypothetical protein D910_01967 [Dendroctonus ponderosae]	2,00E-06
gALL_v3_c103059	1	1	-	-	-	-	-
gALL_v3_c10306	5	3	-	-	-	-	-
gALL_v3_c10307	5	3	-	-	gb|AFV79627.1|	membrane-bound trehalase [Bemisia tabaci]	3,00E-07
gALL_v3_c103074	1	1	-	-	-	-	-
gALL_v3_c103075	1	1	-	-	-	-	-
gALL_v3_c10308	1	7	-	-	-	-	-
gALL_v3_c103080	0	2	-	-	-	-	-
gALL_v3_c103088	0	2	-	-	-	-	-
gALL_v3_c103090	0	2	-	-	-	-	-
gALL_v3_c103094	1	1	-	-	-	-	-
gALL_v3_c103095	1	1	-	-	-	-	-
gALL_v3_c103096	1	1	gnl|CDD|150832	pfam10220, DUF2146, Unchar	gb|EFZ22785.1|	hypothetical protein SINV_02437 [Solenopsis invicta]	6,00E-40
gALL_v3_c103097	0	2	-	-	-	-	-
gALL_v3_c103099	0	2	-	-	-	-	-
gALL_v3_c1031	20	14	-	-	-	-	-
gALL_v3_c10310	6	11	-	-	-	-	-
gALL_v3_c103101	0	2	-	-	-	-	-
gALL_v3_c103102	0	2	-	-	-	-	-
gALL_v3_c103106	1	1	-	-	-	-	-
gALL_v3_c103108	0	2	-	-	-	-	-
gALL_v3_c103109	0	2	-	-	-	-	-
gALL_v3_c10311	2	5	-	-	-	-	-
gALL_v3_c103111	1	1	gnl|CDD|72943	cd04658, Piwi_piwi-like_Euk, I	gb|EHJ75824.1|	aubergine protein [Danaus plexippus]	2,00E-40
gALL_v3_c103112	1	1	-	-	-	-	-
gALL_v3_c103114	1	1	-	-	-	-	-
gALL_v3_c103116	0	2	-	-	gb|EFN85686.1|	hypothetical protein EAI_09677 [Harpegnathos saltator]	5,00E-08
gALL_v3_c103117	1	1	-	-	-	-	-
gALL_v3_c10312	5	2	-	-	-	-	-
gALL_v3_c10313	6	3	gnl|CDD|187698	cd09274, RNase_HI_RT_Ty3, T	ref|XP_004928373.1|	PREDICTED: uncharacterized protein LOC101738890 [Bombyx mori]	2,00E-11
gALL_v3_c103132	0	2	-	-	-	-	-
gALL_v3_c103133	0	2	-	-	-	-	-
gALL_v3_c103134	0	2	-	-	-	-	-
gALL_v3_c103135	1	1	gnl|CDD|152866	pfam12432, DUF3677, Protein	gb|EFN60827.1|	Integrator complex subunit 1 [Camponotus floridanus]	1,00E-62
gALL_v3_c103137	1	1	gnl|CDD|143833	pfam00046, Homeobox, Home	ref|XP_005377390.1|	PREDICTED: homeobox protein MOX-2 [Chinchilla lanigera]	1,00E-27
gALL_v3_c10314	8	6	-	-	gb|AGM32636.1|	hypothetical protein [Coptotermes formosanus]	3,00E-66
gALL_v3_c103140	0	2	-	-	-	-	-
gALL_v3_c103141	0	2	-	-	gb|ERL83322.1|	hypothetical protein D910_00217 [Dendroctonus ponderosae] gb|ERL84374.1| hypothetical protein D910_01802 [Dendroctonus ponderosae]	2,00E-06
gALL_v3_c103143	0	2	-	-	-	-	-
gALL_v3_c103144	1	1	-	-	-	-	-
gALL_v3_c103149	0	2	-	-	-	-	-
gALL_v3_c10315	3	4	-	-	gb|ESN99240.1|	hypothetical protein HELRODRAFT_162756 [Helobdella robusta]	4,00E-35
gALL_v3_c103150	0	2	-	-	-	-	-
gALL_v3_c103152	0	2	-	-	-	-	-
gALL_v3_c103153	0	2	-	-	-	-	-
gALL_v3_c103154	1	1	-	-	-	-	-
gALL_v3_c103155	0	2	-	-	-	-	-
gALL_v3_c103157	0	2	-	-	-	-	-
gALL_v3_c103158	0	2	-	-	-	-	-
gALL_v3_c103160	1	1	-	-	-	-	-
gALL_v3_c103164	0	2	-	-	-	-	-
gALL_v3_c103166	1	1	-	-	-	-	-
gALL_v3_c103168	0	2	-	-	-	-	-
gALL_v3_c103171	0	2	-	-	-	-	-
gALL_v3_c103172	1	1	-	-	-	-	-
gALL_v3_c103173	1	1	-	-	-	-	-
gALL_v3_c103174	1	1	-	-	ref|XP_005104745.1|	PREDICTED: histone-lysine N-methyltransferase SETMAR-like [Aplysia californica]	2,00E-08
gALL_v3_c103178	0	2	gnl|CDD|107361	cd06366, PBP1_GABAb_recep	emb|CDK37792.1|	metabotropic GABA-B receptor subtype 2 [Periplaneta americana]	8,00E-69
gALL_v3_c103179	1	1	-	-	ref|XP_002601231.1|	hypothetical protein BRAFLDRAFT_267115 [Branchiostoma floridae] gb|EEN57243.1| hypothetical protein BRAFLDRAFT_267115 [Branchiostoma floridae]	4,00E-06
gALL_v3_c10318	2	4	gnl|CDD|189734	pfam00832, Ribosomal_L39, R	ref|XP_002400257.1|	ribosomal protein L39, putative [Ixodes scapularis] gb|EEC20154.1| ribosomal protein L39, putative [Ixodes scapularis]	2,00E-30
gALL_v3_c103180	1	1	-	-	-	-	-
gALL_v3_c103182	0	2	-	-	-	-	-
gALL_v3_c103184	1	1	-	-	-	-	-
gALL_v3_c103186	0	2	-	-	-	-	-
gALL_v3_c103187	1	1	-	-	-	-	-
gALL_v3_c103188	1	1	-	-	-	-	-
gALL_v3_c103189	1	1	-	-	-	-	-
gALL_v3_c10319	1	5	-	-	-	-	-
gALL_v3_c103197	0	2	-	-	-	-	-
gALL_v3_c103198	0	2	-	-	-	-	-
gALL_v3_c103199	0	2	-	-	-	-	-
gALL_v3_c1032	11	14	-	-	-	-	-
gALL_v3_c103202	0	2	-	-	-	-	-
gALL_v3_c103203	0	2	-	-	-	-	-
gALL_v3_c103209	0	2	-	-	-	-	-
gALL_v3_c10321	2	3	-	-	ref|XP_005174546.1|	PREDICTED: dnaJ homolog subfamily C member 13-like [Danio rerio]	1,00E-66
gALL_v3_c103210	0	2	-	-	-	-	-
gALL_v3_c103212	0	2	-	-	-	-	-
gALL_v3_c103213	1	1	-	-	ref|XP_002680547.1|	fascin domain-containing protein [Naegleria gruberi] gb|EFC47803.1| fascin domain-containing protein [Naegleria gruberi]	4,00E-13
gALL_v3_c103214	1	1	-	-	-	-	-
gALL_v3_c103215	1	1	gnl|CDD|193497	cd03875, M28_Fxna_like, M28	ref|XP_311708.5|	AGAP003423-PA [Anopheles gambiae str. PEST] gb|EAA07273.5| AGAP003423-PA [Anopheles gambiae str. PEST]	1,00E-05
gALL_v3_c103216	0	2	-	-	-	-	-
gALL_v3_c103217	1	1	-	-	-	-	-
gALL_v3_c103218	1	1	-	-	-	-	-
gALL_v3_c103219	1	1	-	-	-	-	-
gALL_v3_c10322	5	3	gnl|CDD|144626	pfam01103, Bac_surface_Ag, S	ref|XP_003515230.1|	PREDICTED: sorting and assembly machinery component 50 homolog [Cricetulus griseus]	6,00E-40
gALL_v3_c103224	1	1	-	-	-	-	-
gALL_v3_c103225	1	1	-	-	-	-	-
gALL_v3_c103227	0	2	-	-	-	-	-
gALL_v3_c103228	1	1	-	-	-	-	-
gALL_v3_c103229	1	1	-	-	-	-	-
gALL_v3_c103230	1	1	-	-	-	-	-
gALL_v3_c103233	1	1	-	-	-	-	-
gALL_v3_c103235	0	2	-	-	-	-	-
gALL_v3_c103238	0	2	-	-	-	-	-
gALL_v3_c103239	0	2	-	-	-	-	-
gALL_v3_c10324	3	4	-	-	-	-	-
gALL_v3_c103240	0	2	-	-	-	-	-

gALL_v3_c103241	1	1	gnl|CDD|178056	PLN02437, PLN02437, ribonuc	ref|XP_004925552.1|	PREDICTED: ribonucleoside-diphosphate reductase large subunit-like [Bombyx mori]	1,00E-55
gALL_v3_c103242	0	2	gnl|CDD|107357	cd06362, PBP1_mGluR, Liganc	gb|ERL91106.1|	hypothetical protein D910_08448, partial [Dendroctonus ponderosae]	3,00E-58
gALL_v3_c103243	0	2	-	-	-	-	-
gALL_v3_c103244	0	2	gnl|CDD|189417	pfam00144, Beta-lactamase, E	ref|XP_005347785.1|	PREDICTED: serine beta-lactamase-like protein LACTB, mitochondrial [Microtus ochrogaster]	7,00E-39
gALL_v3_c103245	1	1	gnl|CDD|73063	cd02657, Peptidase_C19A, A s	gb|EFN86194.1|	Ubiquitin carboxyl-terminal hydrolase 14 [Harpegnathos saltator]	5,00E-23
gALL_v3_c103246	0	2	-	-	-	-	-
gALL_v3_c103247	1	1	-	-	-	-	-
gALL_v3_c103249	1	1	-	-	-	-	-
gALL_v3_c10325	7	12	gnl|CDD|111117	pfam02187, GAS2, Growth-Ar	ref|XP_003703307.1|	PREDICTED: growth arrest-specific protein 2-like [Megachile rotundata]	1,00E-152
gALL_v3_c103250	0	2	-	-	ref|XP_005462813.1|	PREDICTED: general transcription factor II-I repeat domain-containing protein 2-like [Oreochromis niloticus]	6,00E-07
gALL_v3_c103251	0	2	-	-	-	-	-
gALL_v3_c103253	0	2	-	-	-	-	-
gALL_v3_c103254	0	2	-	-	-	-	-
gALL_v3_c103256	0	2	-	-	ref|XP_002735168.1|	PREDICTED: tigger transposable element-derived protein 6-like [Saccoglossus kowalevskii]	4,00E-07
gALL_v3_c103257	1	1	-	-	ref|XP_002402227.1|	protein PTHB1, putative [Ixodes scapularis] gb|EEC11465.1| protein PTHB1, putative [Ixodes scapularis]	2,00E-44
gALL_v3_c103258	0	2	-	-	-	-	-
gALL_v3_c103259	0	2	-	-	-	-	-
gALL_v3_c10326	2	4	-	-	-	-	-
gALL_v3_c103260	0	2	-	-	-	-	-
gALL_v3_c103263	1	1	-	-	gb|EFA01568.1|	hypothetical protein TcasGA2_TC007129 [Tribolium castaneum]	5,00E-07
gALL_v3_c103266	0	2	-	-	-	-	-
gALL_v3_c103267	0	2	-	-	ref|XP_002432897.1|	hypothetical protein Phum_PHUM604090 [Pediculus humanus corporis] gb|EEB20159.1| hypothetical protein Phum_PHUM604090 [Pediculus humanus corporis]	2,00E-33
gALL_v3_c103268	1	1	-	-	-	-	-
gALL_v3_c103269	0	2	-	-	-	-	-
gALL_v3_c10327	2	4	-	-	-	-	-
gALL_v3_c103270	1	1	-	-	-	-	-
gALL_v3_c103271	1	1	-	-	-	-	-
gALL_v3_c103272	1	1	-	-	-	-	-
gALL_v3_c103273	0	2	-	-	ref|XP_003910457.1|	PREDICTED: jerky protein-like [Papio anubis]	2,00E-08
gALL_v3_c103275	1	1	-	-	ref|XP_004210053.1|	PREDICTED: RNA-directed DNA polymerase from mobile element jockey-like, partial [Hydra vulgaris]	2,00E-14
gALL_v3_c10328	8	13	-	-	ref|XP_970412.1|	PREDICTED: similar to MGC89587 protein [Tribolium castaneum] gb|EEZ99147.1| hypothetical protein TcasGA2_TC012928 [Tribolium castaneum]	1,00E-80
gALL_v3_c103283	1	1	-	-	-	-	-
gALL_v3_c103285	0	2	-	-	-	-	-
gALL_v3_c103289	0	2	-	-	-	-	-
gALL_v3_c10329	3	3	-	-	-	-	-
gALL_v3_c103290	0	2	-	-	-	-	-
gALL_v3_c103294	0	2	-	-	-	-	-
gALL_v3_c103295	1	1	-	-	-	-	-
gALL_v3_c103296	1	1	-	-	-	-	-
gALL_v3_c1033	7	9	gnl|CDD|189965	pfam01388, ARID, ARID/BRIGH	ref|XP_002425904.1|	at-rich interactive domain-containing protein 5B, putative [Pediculus humanus corporis] gb|EEB13166.1| at-rich interactive domain-containing protein 5B, putative [Pedicu	1,00E-152
gALL_v3_c10330	8	5	-	-	-	-	-
gALL_v3_c103304	0	2	-	-	-	-	-
gALL_v3_c103309	1	1	-	-	-	-	-
gALL_v3_c10331	8	5	gnl|CDD|148794	pfam07382, HC2, Histone H1-l	-	-	-
gALL_v3_c103310	0	2	-	-	-	-	-
gALL_v3_c103311	1	1	-	-	-	-	-
gALL_v3_c103312	1	1	-	-	-	-	-
gALL_v3_c103313	1	1	-	-	-	-	-
gALL_v3_c103314	1	1	-	-	ref|XP_002430432.1|	conserved hypothetical protein [Pediculus humanus corporis] gb|EEB17694.1| conserved hypothetical protein [Pediculus humanus corporis]	2,00E-56
gALL_v3_c10332	1	5	-	-	gb|ERL94554.1|	hypothetical protein D910_11831 [Dendroctonus ponderosae]	8,00E-09
gALL_v3_c103323	1	1	-	-	-	-	-
gALL_v3_c103324	0	2	-	-	-	-	-
gALL_v3_c103325	0	2	-	-	-	-	-
gALL_v3_c103327	1	1	-	-	-	-	-
gALL_v3_c103328	1	1	-	-	-	-	-
gALL_v3_c103329	0	2	-	-	-	-	-
gALL_v3_c10333	2	3	-	-	gb|EFN72995.1|	E3 ubiquitin-protein ligase hyd [Camponotus floridanus]	9,00E-46
gALL_v3_c103331	0	2	-	-	-	-	-
gALL_v3_c103332	1	1	gnl|CDD|88600	cd04824, eu_ALAD_PBGS_cyst	ref|XP_001604460.1|	PREDICTED: delta-aminolevulinic acid dehydratase-like [Nasonia vitripennis]	3,00E-25
gALL_v3_c103334	0	2	-	-	-	-	-
gALL_v3_c103335	1	1	-	-	-	-	-
gALL_v3_c103336	0	2	-	-	ref|XP_002432009.1|	dedicator of cytokinesis protein, putative [Pediculus humanus corporis] gb|EEB19271.1| dedicator of cytokinesis protein, putative [Pediculus humanus corporis]	2,00E-15
gALL_v3_c103337	1	1	-	-	-	-	-
gALL_v3_c103338	0	2	-	-	-	-	-
gALL_v3_c103339	1	1	gnl|CDD|147462	pfam05276, SH3BP5, SH3 dom	gb|EFN82176.1|	SH3 domain-binding protein 5-like [Harpegnathos saltator]	3,00E-59
gALL_v3_c10334	8	6	-	-	-	-	-
gALL_v3_c103340	0	2	-	-	-	-	-
gALL_v3_c103341	1	1	-	-	-	-	-
gALL_v3_c103342	0	2	-	-	-	-	-
gALL_v3_c103343	1	1	-	-	ref|XP_005513220.1|	PREDICTED: proteasome activator complex subunit 4 [Columba livia]	2,00E-24
gALL_v3_c103344	1	1	-	-	-	-	-
gALL_v3_c103345	0	2	-	-	-	-	-
gALL_v3_c103346	1	1	-	-	-	-	-
gALL_v3_c103347	1	1	-	-	-	-	-
gALL_v3_c103349	0	2	-	-	-	-	-
gALL_v3_c10335	3	6	-	-	-	-	-
gALL_v3_c103351	0	2	-	-	-	-	-
gALL_v3_c103352	0	2	-	-	-	-	-
gALL_v3_c103353	1	1	gnl|CDD|191480	pfam06248, Zw10, Centromer	gb|EFA07500.1|	zeste-white 10 [Tribolium castaneum]	5,00E-22
gALL_v3_c103354	0	2	-	-	-	-	-
gALL_v3_c103355	1	1	-	-	-	-	-
gALL_v3_c103356	0	2	-	-	-	-	-
gALL_v3_c103357	1	1	gnl|CDD|189496	pfam00307, CH, Calponin hom	gb|EFN70346.1|	Filamin-B [Camponotus floridanus]	1,00E-57
gALL_v3_c103358	1	1	-	-	ref|XP_003701712.1|	PREDICTED: BUD13 homolog [Megachile rotundata]	1,00E-17
gALL_v3_c103359	1	1	-	-	-	-	-
gALL_v3_c10336	0	5	-	-	gb|ELU10961.1|	hypothetical protein CAPTEDRAFT_154354 [Capitella teleta]	7,00E-32
gALL_v3_c103360	0	2	-	-	-	-	-
gALL_v3_c103361	1	1	-	-	-	-	-
gALL_v3_c103362	1	1	-	-	-	-	-
gALL_v3_c103363	1	1	-	-	-	-	-
gALL_v3_c103364	1	1	-	-	-	-	-
gALL_v3_c103365	0	2	-	-	-	-	-
gALL_v3_c103366	1	1	-	-	ref|XP_001602732.1|	PREDICTED: PCI domain-containing protein 2-like [Nasonia vitripennis]	5,00E-08
gALL_v3_c103367	1	1	-	-	-	-	-
gALL_v3_c103368	1	1	-	-	-	-	-
gALL_v3_c103369	1	1	-	-	-	-	-
gALL_v3_c10337	7	11	gnl|CDD|191337	pfam05672, MAP7, MAP7 (E-M	ref|XP_002429796.1|	Scaffold attachment factor B2, putative [Pediculus humanus corporis] gb|EEB17058.1| Scaffold attachment factor B2, putative [Pediculus humanus corporis]	9,00E-10
gALL_v3_c103370	1	1	-	-	-	-	-
gALL_v3_c103372	1	1	-	-	-	-	-
gALL_v3_c103373	1	1	-	-	-	-	-
gALL_v3_c103374	0	2	-	-	-	-	-
gALL_v3_c103375	1	1	-	-	-	-	-
gALL_v3_c103376	1	1	-	-	-	-	-
gALL_v3_c103377	1	1	-	-	gb|AHB39936.1|	phenoloxidase 2, partial [Coptotermes formosanus]	2,00E-07
gALL_v3_c103378	1	1	-	-	-	-	-
gALL_v3_c103379	1	1	-	-	-	-	-
gALL_v3_c10338	7	7	gnl|CDD|192575	pfam10409, PTEN_C2, C2 dom	ref|XP_002424660.1|	tens, putative [Pediculus humanus corporis] gb|EEB11922.1| tens, putative [Pediculus humanus corporis]	2,00E-54
gALL_v3_c103380	1	1	-	-	-	-	-
gALL_v3_c103381	1	1	-	-	gb|EGI60430.1|	Lysophospholipid acyltransferase 5 [Acromyrmex echinatior]	4,00E-14
gALL_v3_c103382	1	1	-	-	-	-	-
gALL_v3_c10339	6	4	-	-	-	-	-
gALL_v3_c10340	7	5	-	-	gb|AGM32451.1|	nitric oxide synthase-interacting protein-like protein, partial [Coptotermes formosanus]	2,00E-95
gALL_v3_c103401	0	2	-	-	-	-	-
gALL_v3_c103402	1	1	-	-	-	-	-
gALL_v3_c103405	1	1	-	-	-	-	-
gALL_v3_c103408	0	2	-	-	-	-	-
gALL_v3_c103419	0	2	-	-	-	-	-
gALL_v3_c10342	7	5	-	-	-	-	-
gALL_v3_c103421	0	2	-	-	-	-	-
gALL_v3_c103422	0	3	-	-	-	-	-
gALL_v3_c103423	0	2	-	-	-	-	-
gALL_v3_c103425	0	2	-	-	-	-	-
gALL_v3_c103426	0	2	-	-	-	-	-
gALL_v3_c103427	0	2	-	-	-	-	-
gALL_v3_c103428	1	1	-	-	-	-	-
gALL_v3_c10343	4	3	gnl|CDD|178703	PLN03158, PLN03158, methio	ref|XP_006612011.1|	PREDICTED: methionine aminopeptidase 1-like [Apis dorsata]	3,00E-45
gALL_v3_c103430	1	1	-	-	-	-	-
gALL_v3_c103436	0	2	-	-	-	-	-
gALL_v3_c103437	1	1	-	-	-	-	-
gALL_v3_c103438	1	1	-	-	-	-	-
gALL_v3_c10344	5	9	-	-	gb|AAF73794.1|AF1540	surface protein PspC [Streptococcus pneumoniae]	2,00E-23
gALL_v3_c103440	0	2	-	-	-	-	-
gALL_v3_c103442	0	2	-	-	-	-	-
gALL_v3_c103443	0	2	-	-	gb|AAB65093.1|	Lian-Aa1 retrotransposon protein [Aedes aegypti]	2,00E-24
gALL_v3_c103444	0	2	-	-	-	-	-
gALL_v3_c103445	1	1	-	-	-	-	-
gALL_v3_c103446	0	2	-	-	-	-	-
gALL_v3_c103447	0	2	-	-	-	-	-
gALL_v3_c103448	1	1	-	-	-	-	-
gALL_v3_c103449	0	2	-	-	-	-	-
gALL_v3_c103450	0	2	-	-	-	-	-
gALL_v3_c103451	1	1	gnl|CDD|189706	pfam00759, Glyco_hydro_9, G	dbj|BAB40697.1|	endo-b-1,4-glucanase [Coptotermes formosanus] gb|ACI45756.1| endo-beta-1,4-glucanase [Coptotermes formosanus]	5,00E-45
gALL_v3_c103452	0	2	-	-	-	-	-
gALL_v3_c103453	0	2	-	-	-	-	-

gALL_v3_c103454	0	2	-	-	-	-	-
gALL_v3_c103455	1	1	-	-	gb|EFA10884.1|	hypothetical protein TcasGA2_TC001708 [Tribolium castaneum]	4,00E-15
gALL_v3_c103456	0	2	-	-	-	-	-
gALL_v3_c103457	1	1	gnl|CDD|48087	cd03382, PAP2_dolichyldiphos	ref|XP_002430135.1|	Dolichyldiphosphatase, putative [Pediculus humanus corporis] gb|EEB17397.1| Dolichyldiphosphatase, putative [Pediculus humanus corporis]	5,00E-58
gALL_v3_c103458	1	1	-	-	-	-	-
gALL_v3_c103459	1	1	-	-	-	-	-
gALL_v3_c10346	6	5	-	-	-	-	-
gALL_v3_c103461	1	1	-	-	-	-	-
gALL_v3_c103462	1	1	-	-	-	-	-
gALL_v3_c103464	1	1	-	-	-	-	-
gALL_v3_c103465	0	2	-	-	-	-	-
gALL_v3_c103466	0	2	gnl|CDD|30109	cd01387, MYSc_type_XV, Myo	ref|XP_001944282.2|	PREDICTED: myosin-XV isoform 1 [Acyrthosiphon pisum]	5,00E-35
gALL_v3_c103467	1	1	-	-	-	-	-
gALL_v3_c103468	1	1	-	-	-	-	-
gALL_v3_c103469	0	2	-	-	-	-	-
gALL_v3_c10347	4	9	-	-	ref|XP_001605486.2|	PREDICTED: hypothetical protein LOC100121879 [Nasonia vitripennis]	1,00E-45
gALL_v3_c103470	0	2	-	-	ref|XP_001599653.2|	PREDICTED: tectonin beta-propeller repeat-containing protein-like [Nasonia vitripennis]	1,00E-24
gALL_v3_c103471	1	1	-	-	-	-	-
gALL_v3_c103472	1	1	-	-	-	-	-
gALL_v3_c103473	1	1	-	-	-	-	-
gALL_v3_c103475	0	2	-	-	-	-	-
gALL_v3_c103476	0	2	-	-	-	-	-
gALL_v3_c103477	1	1	-	-	ref|XP_003395289.1|	PREDICTED: DDB1- and CUL4-associated factor-like 1-like isoform 1 [Bombus terrestris] ref|XP_003395290.1| PREDICTED: DDB1- and CUL4-associated factor-like 1-like isof	6,00E-21
gALL_v3_c103478	1	1	-	-	-	-	-
gALL_v3_c103479	0	2	-	-	-	-	-
gALL_v3_c10348	1	6	gnl|CDD|72857	cd04717, BAH_polybromo, BA	ref|XP_003395905.1|	PREDICTED: LOW QUALITY PROTEIN: protein polybromo-1-like [Bombus terrestris]	6,00E-45
gALL_v3_c103480	0	2	-	-	-	-	-
gALL_v3_c103483	0	2	-	-	-	-	-
gALL_v3_c103485	1	1	-	-	ref|XP_002425580.1|	kynurenine/oxoglutarate transaminase 1, putative [Pediculus humanus corporis] gb|EEB12842.1| kynurenine/oxoglutarate transaminase 1, putative [Pediculus humanus co	4,00E-06
gALL_v3_c103486	1	1	-	-	-	-	-
gALL_v3_c103487	1	1	-	-	-	-	-
gALL_v3_c103488	1	1	-	-	-	-	-
gALL_v3_c10349	5	7	-	-	-	-	-
gALL_v3_c103496	0	2	-	-	-	-	-
gALL_v3_c103498	1	1	-	-	-	-	-
gALL_v3_c103499	0	2	gnl|CDD|193253	pfam12777, MT, Microtubule-	ref|XP_967358.2|	PREDICTED: similar to 1-beta dynein [Tribolium castaneum]	1,00E-58
gALL_v3_c1035	13	19	-	-	ref|XP_973863.1|	PREDICTED: similar to AGAP002913-PA [Tribolium castaneum]	1,00E-59
gALL_v3_c10350	3	5	-	-	-	-	-
gALL_v3_c103505	0	2	-	-	-	-	-
gALL_v3_c103507	1	1	-	-	-	-	-
gALL_v3_c103508	1	1	-	-	-	-	-
gALL_v3_c103509	1	1	-	-	-	-	-
gALL_v3_c10351	3	1	-	-	-	-	-
gALL_v3_c103510	1	1	-	-	ref|XP_795536.2|	PREDICTED: uncharacterized protein LOC590856 [Strongylocentrotus purpuratus]	1,00E-18
gALL_v3_c103511	0	2	-	-	-	-	-
gALL_v3_c103512	1	1	-	-	-	-	-
gALL_v3_c103513	1	1	-	-	-	-	-
gALL_v3_c103514	1	1	-	-	-	-	-
gALL_v3_c103515	1	1	-	-	-	-	-
gALL_v3_c103516	1	1	-	-	-	-	-
gALL_v3_c103519	1	1	-	-	-	-	-
gALL_v3_c10352	4	4	-	-	ref|XP_006608388.1|	PREDICTED: E3 ubiquitin-protein ligase Bre1-like isoform X2 [Apis dorsata]	1,00E-78
gALL_v3_c103520	0	2	gnl|CDD|146154	pfam03367, zf-ZPR1, ZPR1 zinc	ref|XP_975557.1|	PREDICTED: similar to zinc finger protein [Tribolium castaneum] gb|EFA05991.1| hypothetical protein TcasGA2_TC008817 [Tribolium castaneum]	2,00E-23
gALL_v3_c103522	0	2	-	-	-	-	-
gALL_v3_c103527	1	1	-	-	-	-	-
gALL_v3_c103528	0	2	-	-	-	-	-
gALL_v3_c103529	1	1	-	-	-	-	-
gALL_v3_c103530	1	1	-	-	-	-	-
gALL_v3_c103531	0	2	-	-	-	-	-
gALL_v3_c103532	1	1	-	-	gb|EHJ67721.1|	putative SIFamide receptor [Danaus plexippus]	9,00E-32
gALL_v3_c103533	0	2	-	-	-	-	-
gALL_v3_c103534	1	1	-	-	ref|XP_666157.1|	DNA-directed RNA polymerase II largest chain [Cryptosporidium hominis TU502]	4,00E-07
gALL_v3_c103535	0	2	-	-	-	-	-
gALL_v3_c103536	0	2	-	-	-	-	-
gALL_v3_c103537	1	1	-	-	-	-	-
gALL_v3_c103538	0	2	-	-	-	-	-
gALL_v3_c103539	1	1	-	-	gb|EFN87635.1|	Rotatin [Harpegnathos saltator]	1,00E-08
gALL_v3_c10354	0	5	-	-	-	-	-
gALL_v3_c103540	1	1	-	-	-	-	-
gALL_v3_c103541	1	1	-	-	ref|XP_001862585.1|	microtubule binding protein [Culex quinquefasciatus] gb|EDS37223.1| microtubule binding protein [Culex quinquefasciatus]	2,00E-08
gALL_v3_c103542	1	1	-	-	-	-	-
gALL_v3_c103546	1	1	-	-	-	-	-
gALL_v3_c103547	0	2	-	-	-	-	-
gALL_v3_c103548	0	2	-	-	-	-	-
gALL_v3_c103549	0	2	-	-	-	-	-
gALL_v3_c10355	1	5	-	-	-	-	-
gALL_v3_c103550	0	2	-	-	-	-	-
gALL_v3_c103551	1	1	-	-	-	-	-
gALL_v3_c103552	0	2	-	-	-	-	-
gALL_v3_c103553	0	2	-	-	-	-	-
gALL_v3_c103554	0	2	-	-	-	-	-
gALL_v3_c103555	1	1	-	-	-	-	-
gALL_v3_c103556	0	2	-	-	ref|XP_006613427.1|	PREDICTED: protein Daple-like isoform X4 [Apis dorsata]	3,00E-08
gALL_v3_c103557	1	1	-	-	ref|XP_006617790.1|	PREDICTED: zinc finger and SCAN domain-containing protein 25-like [Apis dorsata]	1,00E-07
gALL_v3_c103558	1	1	-	-	-	-	-
gALL_v3_c103559	1	1	-	-	ref|XP_005471583.1|	PREDICTED: zinc finger protein 135-like [Oreochromis niloticus]	2,00E-31
gALL_v3_c10356	2	4	-	-	ref|XP_002429833.1|	rest corepressor corest, protein, putative [Pediculus humanus corporis] gb|EEB17095.1| rest corepressor corest, protein, putative [Pediculus humanus corporis]	5,00E-33
gALL_v3_c103560	0	2	-	-	-	-	-
gALL_v3_c103561	0	2	-	-	-	-	-
gALL_v3_c103562	1	1	-	-	-	-	-
gALL_v3_c103563	1	1	-	-	-	-	-
gALL_v3_c103564	1	1	-	-	-	-	-
gALL_v3_c103565	1	1	-	-	-	-	-
gALL_v3_c103568	0	2	-	-	gb|EFN64073.1|	Craniofacial development protein 2 [Camponotus floridanus]	3,00E-10
gALL_v3_c10357	6	5	gnl|CDD|30180	cd01993, Alpha_ANH_like_II,	ref|XP_001952568.2|	PREDICTED: hypothetical protein LOC100161816 [Acyrthosiphon pisum]	1,00E-100
gALL_v3_c103570	0	2	-	-	-	-	-
gALL_v3_c103571	0	2	-	-	ref|XP_001601597.1|	PREDICTED: aryl-hydrocarbon-interacting protein-like 1-like [Nasonia vitripennis]	2,00E-44
gALL_v3_c103572	1	1	-	-	-	-	-
gALL_v3_c103573	1	1	-	-	-	-	-
gALL_v3_c103574	1	1	-	-	-	-	-
gALL_v3_c103575	1	1	-	-	-	-	-
gALL_v3_c103576	1	1	-	-	-	-	-
gALL_v3_c103577	1	1	-	-	-	-	-
gALL_v3_c103578	1	1	-	-	-	-	-
gALL_v3_c10358	3	4	-	-	-	-	-
gALL_v3_c103580	1	1	-	-	-	-	-
gALL_v3_c103581	0	2	-	-	-	-	-
gALL_v3_c103582	1	1	-	-	-	-	-
gALL_v3_c103583	0	2	-	-	-	-	-
gALL_v3_c103584	0	2	-	-	ref|XP_003241752.1|	PREDICTED: hypothetical protein LOC100568495 [Acyrthosiphon pisum]	1,00E-10
gALL_v3_c103585	0	2	-	-	-	-	-
gALL_v3_c103586	1	1	-	-	-	-	-
gALL_v3_c103587	1	1	-	-	gb|AGM32464.1|	E3 ubiquitin-protein ligase siah-1 [Coptotermes formosanus]	7,00E-15
gALL_v3_c103589	0	2	-	-	-	-	-
gALL_v3_c10359	1	5	-	-	ref|XP_972409.1|	PREDICTED: similar to 18 wheeler [Tribolium castaneum] gb|EEZ99327.1| toll-7-like protein [Tribolium castaneum]	2,00E-57
gALL_v3_c103590	1	1	-	-	-	-	-
gALL_v3_c103591	1	1	gnl|CDD|173839	cd01150, AXO, Peroxisomal ac	ref|XP_971714.1|	PREDICTED: similar to AGAP008016-PA [Tribolium castaneum] gb|EFA09569.1| hypothetical protein TcasGA2_TC011683 [Tribolium castaneum]	4,00E-36
gALL_v3_c103592	1	1	-	-	-	-	-
gALL_v3_c1036	2	6	-	-	-	-	-
gALL_v3_c10360	5	4	-	-	-	-	-
gALL_v3_c103606	0	2	-	-	ref|XP_001849515.1|	conserved hypothetical protein [Culex quinquefasciatus] gb|EDS30424.1| conserved hypothetical protein [Culex quinquefasciatus]	2,00E-34
gALL_v3_c103609	1	1	-	-	-	-	-
gALL_v3_c10361	5	4	-	-	-	-	-
gALL_v3_c103610	1	1	-	-	-	-	-
gALL_v3_c103611	1	1	-	-	-	-	-
gALL_v3_c103612	1	1	-	-	-	-	-
gALL_v3_c103616	1	1	-	-	-	-	-
gALL_v3_c103617	0	2	-	-	-	-	-
gALL_v3_c103618	1	1	-	-	-	-	-
gALL_v3_c103619	1	1	-	-	-	-	-
gALL_v3_c10362	20	14	-	-	gb|EFX86875.1|	hypothetical protein DAPPUDRAFT_235609 [Daphnia pulex]	2,00E-14
gALL_v3_c103623	1	1	-	-	ref|XP_003791348.1|	PREDICTED: M-phase phosphoprotein 6 [Otolemur garnettii]	6,00E-06
gALL_v3_c103625	0	2	-	-	-	-	-
gALL_v3_c103627	0	2	-	-	-	-	-
gALL_v3_c103628	0	2	-	-	-	-	-
gALL_v3_c103629	0	2	gnl|CDD|128466	smart00164, TBC, Domain in T	ref|XP_002423678.1|	conserved hypothetical protein [Pediculus humanus corporis] gb|EEB10940.1| conserved hypothetical protein [Pediculus humanus corporis]	6,00E-86
gALL_v3_c103631	1	1	-	-	-	-	-
gALL_v3_c103632	1	1	-	-	-	-	-
gALL_v3_c103633	0	2	-	-	-	-	-
gALL_v3_c103634	1	1	gnl|CDD|129984	TIGR00906, 2A0303, cationic a	ref|XP_001845829.1|	cationic amino acid transporter 4 [Culex quinquefasciatus] gb|EDS41811.1| cationic amino acid transporter 4 [Culex quinquefasciatus]	9,00E-18
gALL_v3_c103636	1	1	-	-	-	-	-
gALL_v3_c103637	0	2	-	-	-	-	-
gALL_v3_c103639	0	2	-	-	-	-	-
gALL_v3_c10364	3	5	-	-	gb|EFN84483.1|	Tetratricopeptide repeat protein 7B [Harpegnathos saltator]	1,00E-60

gALL_v3_c103640	0	2	-	-	-	-	-
gALL_v3_c103641	0	2	gnl|CDD|192367	pfam09758, FPL, Uncharacteri	gb|EGI69119.1|	Protein CLEC16A [Acromyrmex echinatior]	3,00E-64
gALL_v3_c103644	1	1	-	-	-	-	-
gALL_v3_c103645	1	1	-	-	-	-	-
gALL_v3_c103647	0	2	-	-	-	-	-
gALL_v3_c103648	0	2	-	-	-	-	-
gALL_v3_c103649	1	1	-	-	-	-	-
gALL_v3_c10365	11	16	gnl|CDD|162547	TIGR01821, 5aminolev_synth,	ref|XP_003427112.1|	PREDICTED: 5-aminolevulinate synthase, erythroid-specific, mitochondrial-like isoform 2 [Nasonia vitripennis]	1,00E-130
gALL_v3_c103650	0	2	-	-	gb|ETN64001.1|	suppressor of T-cell receptor signaling 1 [Anopheles darlingi]	2,00E-27
gALL_v3_c103651	0	2	-	-	-	-	-
gALL_v3_c103652	1	1	-	-	-	-	-
gALL_v3_c103653	1	1	-	-	-	-	-
gALL_v3_c103655	0	2	-	-	-	-	-
gALL_v3_c103658	1	1	-	-	-	-	-
gALL_v3_c103659	1	1	-	-	-	-	-
gALL_v3_c103660	1	1	-	-	-	-	-
gALL_v3_c103661	1	1	-	-	-	-	-
gALL_v3_c103662	1	1	-	-	-	-	-
gALL_v3_c103663	1	1	-	-	-	-	-
gALL_v3_c103664	1	1	-	-	-	-	-
gALL_v3_c103676	1	1	-	-	-	-	-
gALL_v3_c103678	0	2	-	-	-	-	-
gALL_v3_c103679	0	2	-	-	-	-	-
gALL_v3_c103680	1	1	-	-	-	-	-
gALL_v3_c103681	0	2	-	-	-	-	-
gALL_v3_c103682	1	1	-	-	-	-	-
gALL_v3_c103683	1	1	-	-	-	-	-
gALL_v3_c103684	1	1	-	-	ref|XP_972273.1|	PREDICTED: hypothetical protein [Tribolium castaneum] gb|EFA04059.1| hypothetical protein TcasGA2_TC014292 [Tribolium castaneum]	1,00E-39
gALL_v3_c103685	1	1	-	-	-	-	-
gALL_v3_c103686	1	1	-	-	-	-	-
gALL_v3_c103687	0	2	-	-	-	-	-
gALL_v3_c103688	1	1	-	-	-	-	-
gALL_v3_c10369	2	5	-	-	-	-	-
gALL_v3_c10370	4	9	-	-	ref|XP_001648824.1|	hypothetical protein AaeL_AAEL014486 [Aedes aegypti] gb|EAT33254.1| AAEL014486-PA, partial [Aedes aegypti]	1,00E-119
gALL_v3_c103700	0	2	-	-	-	-	-
gALL_v3_c103701	1	1	-	-	-	-	-
gALL_v3_c103702	1	1	gnl|CDD|133357	cd04157, Arl6, Arl6 subfamily.	gb|EFA05944.1|	hypothetical protein TcasGA2_TC008762 [Tribolium castaneum]	7,00E-09
gALL_v3_c103704	0	2	-	-	-	-	-
gALL_v3_c103705	0	2	-	-	-	-	-
gALL_v3_c103706	1	1	-	-	ref|XP_001600580.2|	PREDICTED: hypothetical protein LOC100116013 [Nasonia vitripennis]	5,00E-13
gALL_v3_c103710	1	1	-	-	-	-	-
gALL_v3_c103712	1	1	-	-	-	-	-
gALL_v3_c103715	0	2	-	-	-	-	-
gALL_v3_c103716	0	2	gnl|CDD|175994	cd04028, C2B_RIM1alpha, C2	ref|XP_974459.2|	PREDICTED: similar to AGAP001690-PA [Tribolium castaneum]	5,00E-45
gALL_v3_c103717	0	2	-	-	-	-	-
gALL_v3_c103718	0	2	-	-	-	-	-
gALL_v3_c103720	1	1	-	-	gb|ELT99582.1|	hypothetical protein CAPTEDRAFT_224076 [Capitella teleta]	3,00E-24
gALL_v3_c103721	0	2	-	-	-	-	-
gALL_v3_c103722	1	1	-	-	gb|EFZ16092.1|	hypothetical protein SINV_09891 [Solenopsis invicta]	7,00E-10
gALL_v3_c103728	0	2	-	-	-	-	-
gALL_v3_c10373	0	5	-	-	ref|XP_975062.2|	PREDICTED: similar to nuclear fallout CG33991-PC [Tribolium castaneum]	7,00E-27
gALL_v3_c103735	1	1	-	-	gb|AGM32591.1|	ammonium transporter family protein, partial [Coptotermes formosanus]	8,00E-46
gALL_v3_c103736	1	1	-	-	-	-	-
gALL_v3_c103737	0	2	-	-	-	-	-
gALL_v3_c103738	1	1	-	-	-	-	-
gALL_v3_c103739	0	2	-	-	-	-	-
gALL_v3_c103740	0	2	-	-	-	-	-
gALL_v3_c103741	1	1	-	-	ref|XP_003394905.1|	PREDICTED: vacuolar protein sorting-associated protein 16 homolog [Bombus terrestris]	1,00E-21
gALL_v3_c103743	1	1	-	-	-	-	-
gALL_v3_c103744	1	1	-	-	-	-	-
gALL_v3_c103745	1	1	-	-	-	-	-
gALL_v3_c103746	0	2	-	-	-	-	-
gALL_v3_c103749	0	2	-	-	-	-	-
gALL_v3_c103750	1	1	-	-	-	-	-
gALL_v3_c103751	1	1	-	-	ref|XP_006619884.1|	PREDICTED: epsin-2-like isoform X4 [Apis dorsata]	3,00E-18
gALL_v3_c103752	0	2	-	-	-	-	-
gALL_v3_c103759	0	2	-	-	-	-	-
gALL_v3_c10376	4	2	-	-	-	-	-
gALL_v3_c103760	1	1	-	-	-	-	-
gALL_v3_c103761	0	2	-	-	-	-	-
gALL_v3_c103763	1	1	-	-	-	-	-
gALL_v3_c103765	0	2	-	-	-	-	-
gALL_v3_c103766	1	1	-	-	-	-	-
gALL_v3_c10377	15	13	-	-	ref|XP_006563894.1|	PREDICTED: phosphatidylinositol-binding clathrin assembly protein LAP isoform X14 [Apis mellifera]	4,00E-32
gALL_v3_c103770	0	2	-	-	-	-	-
gALL_v3_c103772	1	1	-	-	-	-	-
gALL_v3_c103773	0	2	-	-	-	-	-
gALL_v3_c103778	1	1	-	-	-	-	-
gALL_v3_c103779	0	2	-	-	-	-	-
gALL_v3_c10378	1	6	-	-	-	-	-
gALL_v3_c103783	0	2	-	-	-	-	-
gALL_v3_c103788	1	1	-	-	-	-	-
gALL_v3_c10379	6	7	gnl|CDD|150873	pfam10267, Tmemb_cc2, Prec	ref|XP_006616966.1|	PREDICTED: transmembrane and coiled-coil domains protein 2-like isoform X5 [Apis dorsata]	1,00E-136
gALL_v3_c103792	1	1	-	-	-	-	-
gALL_v3_c103793	0	2	-	-	-	-	-
gALL_v3_c103794	1	1	-	-	-	-	-
gALL_v3_c1038	11	7	-	-	-	-	-
gALL_v3_c10380	1	5	gnl|CDD|178845	PRK00077, eno, enolase; Provi	gb|AGM32398.1|	enolase [Coptotermes formosanus]	1,00E-52
gALL_v3_c103800	1	1	-	-	-	-	-
gALL_v3_c103801	0	2	-	-	-	-	-
gALL_v3_c103802	1	1	-	-	-	-	-
gALL_v3_c103806	0	2	-	-	-	-	-
gALL_v3_c103808	2	2	gnl|CDD|32458	COG2303, BetA, Choline dehy	gb|EFA05513.1|	hypothetical protein TcasGA2_TC015700 [Tribolium castaneum]	1,00E-44
gALL_v3_c10381	9	13	-	-	ref|XP_002426862.1|	conserved hypothetical protein [Pediculus humanus corporis] gb|EEB14124.1| conserved hypothetical protein [Pediculus humanus corporis]	4,00E-49
gALL_v3_c103812	0	2	-	-	-	-	-
gALL_v3_c103814	0	2	-	-	-	-	-
gALL_v3_c103819	0	2	-	-	-	-	-
gALL_v3_c10382	2	10	-	-	-	-	-
gALL_v3_c103820	1	1	-	-	-	-	-
gALL_v3_c103821	1	1	-	-	-	-	-
gALL_v3_c103823	0	2	-	-	ref|XP_971200.1|	PREDICTED: similar to CG2260 CG2260-PA [Tribolium castaneum]	7,00E-41
gALL_v3_c103824	0	2	-	-	-	-	-
gALL_v3_c103825	0	2	-	-	-	-	-
gALL_v3_c103827	0	2	-	-	-	-	-
gALL_v3_c103829	1	1	-	-	-	-	-
gALL_v3_c10383	3	5	-	-	-	-	-
gALL_v3_c103831	1	1	-	-	-	-	-
gALL_v3_c103834	1	1	-	-	-	-	-
gALL_v3_c103835	1	1	-	-	-	-	-
gALL_v3_c103836	1	1	-	-	-	-	-
gALL_v3_c10384	4	6	-	-	ref|XP_001811380.1|	PREDICTED: similar to AGAP005418-PA [Tribolium castaneum] gb|EFA09968.1| hypothetical protein TcasGA2_TC012126 [Tribolium castaneum]	2,00E-42
gALL_v3_c103840	1	1	-	-	-	-	-
gALL_v3_c103846	0	2	-	-	-	-	-
gALL_v3_c103847	1	1	-	-	-	-	-
gALL_v3_c103848	1	1	-	-	-	-	-
gALL_v3_c10385	3	2	-	-	-	-	-
gALL_v3_c103851	1	1	-	-	-	-	-
gALL_v3_c103852	0	2	-	-	-	-	-
gALL_v3_c103853	1	1	-	-	-	-	-
gALL_v3_c103854	1	1	-	-	-	-	-
gALL_v3_c103856	0	2	gnl|CDD|178415	PLN02820, PLN02820, 3-meth	gb|ELU14608.1|	hypothetical protein CAPTEDRAFT_180561 [Capitella teleta]	1,00E-09
gALL_v3_c10386	7	4	gnl|CDD|191844	pfam07767, Nop53, Nop53 (6	gb|EEZ98070.1|	hypothetical protein TcasGA2_TC000477 [Tribolium castaneum]	2,00E-27
gALL_v3_c103865	1	1	-	-	-	-	-
gALL_v3_c103866	0	2	-	-	ref|XP_001850636.1|	ca-activated cl channel protein [Culex quinquefasciatus] gb|EDS32392.1| ca-activated cl channel protein [Culex quinquefasciatus]	4,00E-43
gALL_v3_c103867	0	2	-	-	-	-	-
gALL_v3_c103868	0	2	-	-	-	-	-
gALL_v3_c10387	4	3	gnl|CDD|189372	pfam00067, p450, Cytochrom	gb|AGM32645.1|	cytochrome P450, partial [Coptotermes formosanus]	7,00E-49
gALL_v3_c103873	1	1	-	-	-	-	-
gALL_v3_c103877	0	2	gnl|CDD|133346	cd04146, RERG_RasL11_like, F	ref|XP_975474.1|	PREDICTED: similar to MRAS2, putative [Tribolium castaneum] gb|EEZ98208.1| hypothetical protein TcasGA2_TC000640 [Tribolium castaneum]	7,00E-27
gALL_v3_c10388	4	6	gnl|CDD|149345	pfam08231, SYF2, SYF2 splicin	ref|XP_003694771.1|	PREDICTED: pre-mRNA-splicing factor Syf2-like [Apis florea]	7,00E-83
gALL_v3_c103884	1	1	-	-	-	-	-
gALL_v3_c10389	8	9	-	-	ref|XP_002428005.1|	YY1-associated factor, putative [Pediculus humanus corporis] gb|EEB15267.1| YY1-associated factor, putative [Pediculus humanus corporis]	1,00E-33
gALL_v3_c103893	1	1	-	-	-	-	-
gALL_v3_c103895	1	1	-	-	-	-	-
gALL_v3_c103899	0	2	-	-	-	-	-
gALL_v3_c1039	7	7	gnl|CDD|48213	cd03401, Band_7_prohibitin, I	gb|EFN75536.1|	Protein l(2)37Cc [Harpegnathos saltator]	1,00E-126
gALL_v3_c10390	2	7	-	-	-	-	-
gALL_v3_c103903	0	2	-	-	gb|EHJ71971.1|	hypothetical protein KGM_20351 [Danaus plexippus]	7,00E-28
gALL_v3_c10391	1	5	-	-	-	-	-
gALL_v3_c103912	1	1	-	-	-	-	-
gALL_v3_c103914	1	1	-	-	-	-	-
gALL_v3_c10393	7	5	-	-	-	-	-
gALL_v3_c10394	5	13	gnl|CDD|189432	pfam00169, PH, PH domain. F	ref|XP_002423426.1|	grb2-associated binder, gab, putative [Pediculus humanus corporis] gb|EEB10688.1| grb2-associated binder, gab, putative [Pediculus humanus corporis]	3,00E-73
gALL_v3_c10395	2	5	-	-	-	-	-

gALL_v3_c10396	6	8	-	-	ref|XP_006621752.1|	PREDICTED: uncharacterized protein LOC102673391 [Apis dorsata]	9,00E-23
gALL_v3_c10398	3	6	gnl|CDD|34777	COG5178, PRP8, U5 snRNP spl	ref|XP_003703080.1|	PREDICTED: pre-mRNA-processing-splicing factor 8-like [Megachile rotundata]	1,00E-131
gALL_v3_c104	18	14	gnl|CDD|185767	cd09244, BRO1_Rhophilin, Pro	gb|EFA06476.1|	hypothetical protein TcasGA2_TC009368 [Tribolium castaneum]	0
gALL_v3_c1040	9	6	-	-	-	-	-
gALL_v3_c10400	5	6	-	-	-	-	-
gALL_v3_c10401	4	8	-	-	-	-	-
gALL_v3_c10402	3	2	gnl|CDD|58652	cd00928, Cyt_c_Oxidase_VIIa,	gb|AGM32612.1|	cytochrome c oxidase subunit VIIa [Coptotermes formosanus]	3,00E-81
gALL_v3_c10403	5	4	-	-	-	-	-
gALL_v3_c10404	2	9	gnl|CDD|111859	pfam03015, Sterile, Male steri	gb|EFN65009.1|	Fatty acyl-CoA reductase 1 [Camponotus floridanus]	6,00E-21
gALL_v3_c104040	1	1	-	-	ref|XP_003241752.1|	PREDICTED: hypothetical protein LOC100568495 [Acyrthosiphon pisum]	1,00E-09
gALL_v3_c10406	5	5	-	-	-	-	-
gALL_v3_c10407	1	8	-	-	-	-	-
gALL_v3_c10408	4	5	-	-	-	-	-
gALL_v3_c10409	3	3	-	-	-	-	-
gALL_v3_c1041	16	17	-	-	-	-	-
gALL_v3_c10410	3	5	-	-	-	-	-
gALL_v3_c104107	0	2	-	-	-	-	-
gALL_v3_c10413	1	8	gnl|CDD|34675	COG5071, RPN5, 26S proteaso	gb|AGM32272.1|	putative 26S proteasome non-ATpase regulatory subunit, partial [Coptotermes formosanus]	1,00E-119
gALL_v3_c10415	10	7	gnl|CDD|179984	PRK05269, PRK05269, transalo	gb|AGM32831.1|	transaldolase-like protein [Coptotermes formosanus]	9,00E-53
gALL_v3_c10416	2	5	-	-	-	-	-
gALL_v3_c1042	5	9	-	-	-	-	-
gALL_v3_c10420	7	7	-	-	-	-	-
gALL_v3_c10422	7	6	-	-	ref|XP_969774.1|	PREDICTED: similar to CG6151 CG6151-PB [Tribolium castaneum]	5,00E-06
gALL_v3_c10424	5	2	gnl|CDD|189955	pfam01363, FYVE, FYVE zinc fi	gb|EEZ97934.1|	hypothetical protein TcasGA2_TC000322 [Tribolium castaneum]	8,00E-65
gALL_v3_c10425	1	7	-	-	gb|EFX73334.1|	hypothetical protein DAPPUDRAFT_307947 [Daphnia pulex]	1,00E-35
gALL_v3_c10426	3	2	-	-	-	-	-
gALL_v3_c10428	4	4	-	-	-	-	-
gALL_v3_c10429	5	8	-	-	ref|XP_002430024.1|	Fumarylacetoacetate hydrolase domain-containing protein, putative [Pediculus humanus corporis] gb|EEB17286.1| Fumarylacetoacetate hydrolase domain-containing pro	3,00E-10
gALL_v3_c1043	3	6	-	-	-	-	-
gALL_v3_c10430	12	19	-	-	-	-	-
gALL_v3_c10431	3	2	-	-	-	-	-
gALL_v3_c10432	5	6	gnl|CDD|161950	TIGR00600, rad2, DNA excisior	ref|XP_001845908.1|	DNA-repair protein complementing XP-G cells [Culex quinquefasciatus] gb|EDS41982.1| DNA-repair protein complementing XP-G cells [Culex quinquefasciatus]	5,00E-55
gALL_v3_c10434	5	4	-	-	-	-	-
gALL_v3_c10435	3	5	-	-	-	-	-
gALL_v3_c10436	1	4	-	-	-	-	-
gALL_v3_c10437	3	5	-	-	-	-	-
gALL_v3_c104389	13	11	gnl|CDD|189372	pfam00067, p450, Cytochrom	gb|ABX64399.1|	cytochrome P450 v2 [Liposcelis bostrychophila]	1,00E-117
gALL_v3_c10439	3	2	-	-	-	-	-
gALL_v3_c1044	8	13	gnl|CDD|173412	PTZ00121, PTZ00121, MAEBL;	-	-	-
gALL_v3_c10441	4	3	-	-	-	-	-
gALL_v3_c104410	1	1	-	-	-	-	-
gALL_v3_c10442	6	5	-	-	-	-	-
gALL_v3_c10443	5	4	-	-	ref|XP_005526609.1|	PREDICTED: proteasome assembly chaperone 1 [Pseudopodoces humilis]	5,00E-25
gALL_v3_c10444	13	12	-	-	-	-	-
gALL_v3_c10445	10	10	-	-	-	-	-
gALL_v3_c10447	0	5	-	-	-	-	-
gALL_v3_c10449	2	3	-	-	ref|XP_001664271.1|	hypothetical protein AaeL_AAEL003882 [Aedes aegypti] gb|EAT44784.1| AAEL003882-PA [Aedes aegypti]	2,00E-42
gALL_v3_c1045	22	32	-	-	gb|EGI61482.1|	Constitutive coactivator of PPAR-gamma-like protein 1-like protein [Acromyrmex echinatior]	1,00E-123
gALL_v3_c10451	4	5	-	-	ref|XP_003247491.1|	PREDICTED: hypothetical protein LOC100572899 [Acyrthosiphon pisum]	2,00E-15
gALL_v3_c10452	1	4	-	-	-	-	-
gALL_v3_c10453	4	2	-	-	-	-	-
gALL_v3_c10454	4	5	-	-	gb|EFZ22070.1|	hypothetical protein SINV_03808 [Solenopsis invicta]	1,00E-51
gALL_v3_c10455	4	4	-	-	-	-	-
gALL_v3_c10456	5	5	-	-	-	-	-
gALL_v3_c10457	4	7	gnl|CDD|128725	smart00449, SPRY, Domain in	ref|XP_003702391.1|	PREDICTED: E3 ubiquitin-protein ligase RNF123 [Megachile rotundata]	2,00E-65
gALL_v3_c10459	7	8	gnl|CDD|191286	pfam05497, Destabilase, Desta	gb|AFZ78836.1|	I-type lysozyme [Coptotermes formosanus]	2,00E-99
gALL_v3_c1046	19	13	-	-	ref|XP_001605693.1|	PREDICTED: protein CDV3-like [Nasonia vitripennis]	4,00E-26
gALL_v3_c10460	9	11	-	-	gb|AGM32800.1|	eukaryotic translation initiation factor 4 gamma 3-like protein [Coptotermes formosanus]	6,00E-78
gALL_v3_c10463	3	4	-	-	-	-	-
gALL_v3_c10464	10	15	gnl|CDD|189008	cd09601, M1_APN_2, Peptida	ref|XP_001651187.1|	protease m1 zinc metalloprotease [Aedes aegypti] gb|EAT32597.1| AAEL015248-PA, partial [Aedes aegypti]	2,00E-80
gALL_v3_c10465	7	8	gnl|CDD|119432	cd05172, PIKKc_DNA-PK, DNA	dbj|BAC40685.1|	unnamed protein product [Mus musculus]	1,00E-106
gALL_v3_c10466	4	6	-	-	-	-	-
gALL_v3_c10467	1	5	-	-	-	-	-
gALL_v3_c10468	5	3	-	-	gb|EFA09709.1|	hypothetical protein TcasGA2_TC011842 [Tribolium castaneum]	7,00E-65
gALL_v3_c10469	4	2	-	-	-	-	-
gALL_v3_c1047	4	6	gnl|CDD|188225	TIGR02478, 6PF1K_euk, 6-pho	ref|XP_004930182.1|	PREDICTED: 6-phosphofructokinase-like isoform X2 [Bombyx mori]	1,00E-117
gALL_v3_c10470	9	11	-	-	ref|XP_002425890.1|	pre-mRNA-splicing factor clf-1, putative [Pediculus humanus corporis] gb|EEB13152.1| pre-mRNA-splicing factor clf-1, putative [Pediculus humanus corporis]	1,00E-100
gALL_v3_c10471	3	5	-	-	-	-	-
gALL_v3_c10472	3	4	-	-	-	-	-
gALL_v3_c10473	2	3	-	-	-	-	-
gALL_v3_c10474	9	5	-	-	ref|XP_002385618.1|	LINE-1 retrotransposon-like element, putative [Aspergillus flavus NRRL3357] gb|EED44706.1| LINE-1 retrotransposon-like element, putative [Aspergillus flavus NRRL3357]	1,00E-20
gALL_v3_c10475	2	10	gnl|CDD|162459	TIGR01642, U2AF_lg, U2 snRN	gb|EFZ16137.1|	hypothetical protein SINV_12499 [Solenopsis invicta]	2,00E-53
gALL_v3_c10476	2	2	gnl|CDD|177159	MTH00097, ND6, NADH dehyc	-	-	-
gALL_v3_c10477	3	2	-	-	ref|XP_002430244.1|	sodium/hydrogen exchanger, putative [Pediculus humanus corporis] gb|EEB17506.1| sodium/hydrogen exchanger, putative [Pediculus humanus corporis]	4,00E-13
gALL_v3_c10478	3	3	-	-	-	-	-
gALL_v3_c104781	0	2	-	-	gb|AGM32289.1|	putative annexin-B10, partial [Coptotermes formosanus]	4,00E-16
gALL_v3_c10479	6	6	-	-	ref|XP_003492334.1|	PREDICTED: sortilin-related receptor-like [Bombus impatiens]	9,00E-31
gALL_v3_c104798	0	2	-	-	-	-	-
gALL_v3_c1048	24	33	-	-	-	-	-
gALL_v3_c10480	7	7	-	-	-	-	-
gALL_v3_c10481	4	3	gnl|CDD|176022	cd08376, C2B_MCTP_PRT, C2	ref|XP_003703105.1|	PREDICTED: uncharacterized protein LOC100879182 [Megachile rotundata]	9,00E-37
gALL_v3_c10482	5	15	gnl|CDD|163659	cd07416, MPP_PP2B, PP2B, m	gb|EEZ99522.1|	hypothetical protein TcasGA2_TC000103 [Tribolium castaneum]	0
gALL_v3_c10483	5	11	-	-	-	-	-
gALL_v3_c10484	4	3	gnl|CDD|128524	smart00228, PDZ, Domain pre	ref|XP_001607422.1|	PREDICTED: Na(+)/H(+) exchange regulatory cofactor NHE-RF1-like [Nasonia vitripennis]	7,00E-42
gALL_v3_c10485	6	5	gnl|CDD|149104	pfam07850, Renin_r, Renin rec	ref|XP_973593.1|	PREDICTED: similar to conserved hypothetical protein [Tribolium castaneum] gb|EFA03659.1| hypothetical protein TcasGA2_TC013754 [Tribolium castaneum]	7,00E-51
gALL_v3_c10486	28	19	gnl|CDD|162463	TIGR01648, hnRNP-R-Q, heter	ref|XP_006569433.1|	PREDICTED: heterogeneous nuclear ribonucleoprotein R-like isoform X19 [Apis mellifera]	0
gALL_v3_c10487	7	5	-	-	-	-	-
gALL_v3_c10488	2	3	gnl|CDD|189334	pfam00001, 7tm_1, 7 transme	ref|XP_001943863.2|	PREDICTED: Phe13-bombesin receptor-like [Acyrthosiphon pisum]	2,00E-16
gALL_v3_c104883	2	1	-	-	-	-	-
gALL_v3_c10489	2	4	-	-	-	-	-
gALL_v3_c10490	5	10	-	-	-	-	-
gALL_v3_c10491	3	4	-	-	-	-	-
gALL_v3_c10494	7	7	-	-	-	-	-
gALL_v3_c10495	4	6	gnl|CDD|192529	pfam10309, DUF2414, Protein	ref|XP_003394196.1|	PREDICTED: hypothetical protein LOC100651119 [Bombus terrestris]	6,00E-49
gALL_v3_c10496	3	3	-	-	-	-	-
gALL_v3_c10497	2	4	gnl|CDD|189704	pfam00757, Furin-like, Furin-li	ref|XP_003490625.1|	PREDICTED: insulin-like receptor-like [Bombus impatiens]	5,00E-64
gALL_v3_c10498	5	4	-	-	-	-	-
gALL_v3_c10499	5	5	gnl|CDD|192729	pfam11261, IRF-2BP1_2, Inter	ref|XP_002432917.1|	conserved hypothetical protein [Pediculus humanus corporis] gb|EEB20179.1| conserved hypothetical protein [Pediculus humanus corporis]	2,00E-37
gALL_v3_c1050	12	16	-	-	-	-	-
gALL_v3_c10504	5	2	-	-	-	-	-
gALL_v3_c10506	5	2	-	-	ref|XP_966333.1|	PREDICTED: similar to cuticular protein [Tribolium castaneum] gb|EFA06924.1| hypothetical protein TcasGA2_TC009873 [Tribolium castaneum]	9,00E-17
gALL_v3_c10507	5	8	-	-	ref|XP_003246118.1|	PREDICTED: major centromere autoantigen B-like [Acyrthosiphon pisum]	3,00E-23
gALL_v3_c10508	6	9	gnl|CDD|176069	cd08687, C2_PKN-like, C2 dom	ref|XP_002431613.1|	conserved hypothetical protein [Pediculus humanus corporis] gb|EEB18875.1| conserved hypothetical protein [Pediculus humanus corporis]	1,00E-145
gALL_v3_c1051	2	5	-	-	ref|XP_006619676.1|	PREDICTED: ankyrin-3-like isoform X5 [Apis dorsata]	6,00E-33
gALL_v3_c10510	4	7	-	-	gb|AGM32818.1|	putative corticosteroid 11-beta-dehydrogenase, partial [Coptotermes formosanus]	3,00E-23
gALL_v3_c10512	4	4	-	-	-	-	-
gALL_v3_c10513	1	6	-	-	-	-	-
gALL_v3_c10514	4	5	-	-	-	-	-
gALL_v3_c10515	3	6	-	-	-	-	-
gALL_v3_c10516	3	2	-	-	gb|EGI63802.1|	Paxillin [Acromyrmex echinatior]	4,00E-35
gALL_v3_c10517	1	7	-	-	-	-	-
gALL_v3_c10518	0	5	-	-	ref|XP_557938.3|	AGAP008770-PA [Anopheles gambiae str. PEST] gb|EAL40302.3| AGAP008770-PA [Anopheles gambiae str. PEST]	2,00E-83
gALL_v3_c10519	4	5	-	-	-	-	-
gALL_v3_c105197	0	2	-	-	gb|EFN67634.1|	Putative uncharacterized transposon-derived protein F52C9.6 [Camponotus floridanus]	1,00E-13
gALL_v3_c1052	4	9	-	-	-	-	-
gALL_v3_c10520	2	3	-	-	-	-	-
gALL_v3_c10521	3	2	-	-	-	-	-
gALL_v3_c10523	2	5	-	-	gb|ADO33045.1|	ring finger protein [Biston betularia]	3,00E-47
gALL_v3_c10524	9	8	-	-	-	-	-
gALL_v3_c10525	3	3	-	-	ref|XP_004377475.1|	PREDICTED: isoprenoid synthase domain-containing protein isoform 2 [Trichechus manatus latirostris]	9,00E-18
gALL_v3_c10526	2	6	gnl|CDD|30804	COG0456, RimI, Acetyltransfer	ref|XP_002400312.1|	conserved hypothetical protein [Ixodes scapularis] gb|EEC15783.1| conserved hypothetical protein [Ixodes scapularis]	2,00E-98
gALL_v3_c10527	2	3	-	-	-	-	-
gALL_v3_c10529	1	5	-	-	-	-	-
gALL_v3_c1053	33	36	-	-	-	-	-
gALL_v3_c10530	4	3	gnl|CDD|73003	cd03244, ABCC_MRP_domain	gb|EFA03011.1|	hypothetical protein TcasGA2_TC010434 [Tribolium castaneum]	1,00E-74
gALL_v3_c105318	1	1	-	-	ref|XP_002432590.1|	glycerol kinase, putative [Pediculus humanus corporis] gb|EEB19852.1| glycerol kinase, putative [Pediculus humanus corporis]	1,00E-06
gALL_v3_c10532	2	4	gnl|CDD|185504	PTZ00184, PTZ00184, calmodu	ref|XP_006557869.1|	PREDICTED: myosin-2 essential light chain isoform X1 [Apis mellifera] ref|XP_006617380.1| PREDICTED: myosin-2 essential light chain-like isoform X1 [Apis dorsata]	2,00E-60
gALL_v3_c10533	7	4	-	-	-	-	-
gALL_v3_c105341	2	1	-	-	-	-	-
gALL_v3_c10535	7	4	gnl|CDD|189544	pfam00431, CUB, CUB domain	ref|XP_002432117.1|	conserved hypothetical protein [Pediculus humanus corporis] gb|EEB19379.1| conserved hypothetical protein [Pediculus humanus corporis]	0
gALL_v3_c10538	8	6	-	-	ref|XP_001983511.1|	GH15540 [Drosophila grimshawi] gb|EDV95859.1| GH15540 [Drosophila grimshawi]	7,00E-31
gALL_v3_c105387	1	2	-	-	-	-	-
gALL_v3_c10539	1	3	-	-	-	-	-
gALL_v3_c1054	9	9	gnl|CDD|48564	cd03015, PRX_Typ2cys, Peroxi	gb|EFX63892.1|	hypothetical protein DAPPUDRAFT_305931 [Daphnia pulex]	1,00E-110
gALL_v3_c10540	7	9	-	-	-	-	-
gALL_v3_c10541	1	5	-	-	ref|XP_002425005.1|	Heparan-sulfate 6-O-sulfotransferase, putative [Pediculus humanus corporis] gb|EEB12267.1| Heparan-sulfate 6-O-sulfotransferase, putative [Pediculus humanus corporis]	5,00E-53
gALL_v3_c10542	0	6	-	-	ref|XP_003486934.1|	PREDICTED: STE20-like serine/threonine-protein kinase-like isoform 1 [Bombus impatiens]	9,00E-10
gALL_v3_c10543	4	8	-	-	gb|EFN67634.1|	Putative uncharacterized transposon-derived protein F52C9.6 [Camponotus floridanus]	6,00E-13
gALL_v3_c10544	6	14	-	-	-	-	-
gALL_v3_c10545	5	5	-	-	-	-	-
gALL_v3_c10546	3	3	-	-	-	-	-
gALL_v3_c10548	4	2	-	-	-	-	-
gALL_v3_c10549	2	4	gnl|CDD|153332	cd07648, F-BAR_FCHO, The F-	dbj|BAN20956.1|	conserved hypothetical protein [Riptortus pedestris]	8,00E-74
gALL_v3_c1055	7	4	-	-	-	-	-

gALL_v3_c10551	3	2	-	-	-	-	-
gALL_v3_c10552	3	4	-	-	gb|EGI60398.1|	U6 snRNA-specific terminal uridylyltransferase 1 [Acromyrmex echinatior]	2,00E-07
gALL_v3_c10554	4	6	-	-	-	-	-
gALL_v3_c10555	6	10	-	-	-	-	-
gALL_v3_c10556	3	2	-	-	-	-	-
gALL_v3_c105568	1	3	-	-	-	-	-
gALL_v3_c10557	4	5	-	-	-	-	-
gALL_v3_c1056	3	9	gnl|CDD|189810	pfam01030, Recep_L_domain,	ref|XP_006568759.1|	PREDICTED: insulin-like receptor-like isoform X3 [Apis mellifera]	4,00E-53
gALL_v3_c10560	5	3	-	-	-	-	-
gALL_v3_c10561	4	7	gnl|CDD|33079	COG3268, COG3268, Uncharac	ref|XP_001845605.1|	saccharopine dehydrogenase domain-containing protein [Culex quinquefasciatus] gb|EDS40900.1| saccharopine dehydrogenase domain-containing protein [Culex quinque	2,00E-50
gALL_v3_c10562	1	4	-	-	-	-	-
gALL_v3_c10563	5	2	-	-	-	-	-
gALL_v3_c10564	5	8	-	-	-	-	-
gALL_v3_c10565	3	3	-	-	-	-	-
gALL_v3_c10567	6	4	-	-	-	-	-
gALL_v3_c10568	8	10	gnl|CDD|29397	cd01926, cyclophilin_ABH_like	gb|EFN85054.1|	40 kDa peptidyl-prolyl cis-trans isomerase [Harpegnathos saltator]	1,00E-101
gALL_v3_c10569	4	2	-	-	-	-	-
gALL_v3_c10570	3	9	-	-	emb|CCC55948.1|	kruppel homologue 1 [Blattella germanica]	1,00E-147
gALL_v3_c10572	7	13	-	-	ref|XP_003704273.1|	PREDICTED: LOW QUALITY PROTEIN: talin-1-like [Megachile rotundata]	1,00E-131
gALL_v3_c10573	1	6	-	-	-	-	-
gALL_v3_c10575	4	3	-	-	ref|XP_321174.5|	AGAP001888-PA [Anopheles gambiae str. PEST] gb|EAA01061.5| AGAP001888-PA [Anopheles gambiae str. PEST]	7,00E-93
gALL_v3_c10576	5	5	-	-	-	-	-
gALL_v3_c10577	3	8	-	-	-	-	-
gALL_v3_c10578	6	9	gnl|CDD|128342	smart00027, EH, Eps15 homol	ref|XP_967469.1|	PREDICTED: similar to GA14224-PA [Tribolium castaneum]	1,00E-115
gALL_v3_c1058	4	7	-	-	-	-	-
gALL_v3_c10580	1	6	-	-	-	-	-
gALL_v3_c10581	6	14	-	-	-	-	-
gALL_v3_c10583	7	8	gnl|CDD|28933	cd00051, EFh, EF-hand, calciu	gb|AGM32341.1|	troponin C-like protein [Coptotermes formosanus]	8,00E-28
gALL_v3_c10584	6	12	-	-	-	-	-
gALL_v3_c10585	2	8	-	-	-	-	-
gALL_v3_c10588	8	4	gnl|CDD|180322	PRK05950, sdhB, succinate de	gb|AGO46415.1|	succinate dehydrogenase complex subunit A, partial [Reticulitermes flavipes]	8,00E-77
gALL_v3_c105880	1	1	gnl|CDD|28939	cd00057, FA58C, Coagulation f	ref|XP_001687897.1|	AGAP007545-PB [Anopheles gambiae str. PEST] gb|EDO64546.1| AGAP007545-PB [Anopheles gambiae str. PEST]	4,00E-29
gALL_v3_c10589	3	4	gnl|CDD|190839	pfam04045, P34-Arc, Arp2/3 c	ref|XP_002431454.1|	arp2/3, putative [Pediculus humanus corporis] gb|EEB18716.1| arp2/3, putative [Pediculus humanus corporis]	2,00E-46
gALL_v3_c1059	4	11	gnl|CDD|128654	smart00360, RRM, RNA recogi	ref|XP_967690.1|	PREDICTED: similar to RNA binding region containing protein 1 [Tribolium castaneum] gb|EFA10896.1| hypothetical protein TcasGA2_TC001720 [Tribolium castaneum]	3,00E-80
gALL_v3_c10590	0	6	-	-	-	-	-
gALL_v3_c10591	4	4	gnl|CDD|29138	cd00176, SPEC, Spectrin repea	ref|XP_001602426.2|	PREDICTED: microtubule-actin cross-linking factor 1-like [Nasonia vitripennis]	3,00E-80
gALL_v3_c10593	6	15	-	-	-	-	-
gALL_v3_c10594	14	13	-	-	gb|EEZ98720.1|	hypothetical protein TcasGA2_TC001266 [Tribolium castaneum]	1,00E-18
gALL_v3_c10595	1	7	-	-	-	-	-
gALL_v3_c10596	1	6	gnl|CDD|128524	smart00228, PDZ, Domain pre	ref|XP_003707820.1|	PREDICTED: uncharacterized protein LOC100877819, partial [Megachile rotundata]	1,00E-33
gALL_v3_c10597	5	3	-	-	ref|XP_003486085.1|	PREDICTED: clavesin-1-like [Bombus impatiens]	2,00E-09
gALL_v3_c10599	3	6	gnl|CDD|189372	pfam00067, p450, Cytochrome	gb|AGI03850.1|	cytochrome P450 6BQ11 [Tribolium castaneum]	1,00E-45
gALL_v3_c106	7	5	-	-	-	-	-
gALL_v3_c10600	8	5	gnl|CDD|192101	pfam08623, TIP120, TATA-binc	ref|XP_002433195.1|	Cullin-associated NEDD8-dissociated protein, putative [Pediculus humanus corporis] gb|EEB20457.1| Cullin-associated NEDD8-dissociated protein, putative [Pediculus hun	1,00E-151
gALL_v3_c10601	7	4	gnl|CDD|143833	pfam00046, Homeobox, Home	gb|AAL09697.1|	cephalothorax [Tribolium castaneum]	3,00E-57
gALL_v3_c10602	3	11	-	-	-	-	-
gALL_v3_c106022	0	2	-	-	-	-	-
gALL_v3_c10604	2	5	gnl|CDD|185504	PTZ00184, PTZ00184, calmodu	gb|AGM32895.1|	calmodulin-like protein [Coptotermes formosanus]	7,00E-46
gALL_v3_c10606	2	5	-	-	-	-	-
gALL_v3_c10607	2	2	-	-	-	-	-
gALL_v3_c10608	4	3	-	-	ref|XP_003487883.1|	PREDICTED: hypothetical protein LOC100748451 [Bombus impatiens]	1,00E-58
gALL_v3_c10609	9	12	gnl|CDD|178298	PLN02695, PLN02695, GDP-D-	gb|AGM32354.1|	epimerase/dehydratase [Coptotermes formosanus]	0
gALL_v3_c10611	5	4	-	-	-	-	-
gALL_v3_c10612	1	6	gnl|CDD|29261	cd00204, ANK, ankyrin repeat:	dbj|BAL61212.1|	fem-1 homolog B-like protein [Locusta migratoria manilensis]	1,00E-105
gALL_v3_c106127	1	2	-	-	gb|EFN67634.1|	Putative uncharacterized transposon-derived protein F52C9.6 [Camponotus floridanus]	3,00E-22
gALL_v3_c10614	6	17	gnl|CDD|178012	PLN02387, PLN02387, long-ch	ref|XP_002431052.1|	Long-chain-fatty-acid--CoA ligase, putative [Pediculus humanus corporis] gb|EEB18314.1| Long-chain-fatty-acid--CoA ligase, putative [Pediculus humanus corporis]	1,00E-124
gALL_v3_c10615	5	11	-	-	-	-	-
gALL_v3_c10617	5	12	-	-	-	-	-
gALL_v3_c10618	7	15	gnl|CDD|191823	pfam07716, bZIP_2, Basic regi	ref|XP_003697838.1|	PREDICTED: hepatic leukemia factor-like [Apis florea] ref|XP_006560301.1| PREDICTED: hepatic leukemia factor isoform X6 [Apis mellifera] ref|XP_006616198.1| PREDICT	1,00E-115
gALL_v3_c10619	3	1	-	-	-	-	-
gALL_v3_c1062	2	7	-	-	ref|XP_001843910.1|	Brahma associated protein 170kD [Culex quinquefasciatus] gb|EDS35242.1| Brahma associated protein 170kD [Culex quinquefasciatus]	2,00E-49
gALL_v3_c10620	2	5	gnl|CDD|149404	pfam08332, CaMKII_AD, Calci	gb|AGI98593.1|	CaMKII isoform E [Periplaneta americana]	1,00E-110
gALL_v3_c10621	1	4	-	-	ref|XP_002425259.1|	protein N-terminal asparagine amidohydrolase, putative [Pediculus humanus corporis] gb|EEB12521.1| protein N-terminal asparagine amidohydrolase, putative [Pediculus	5,00E-41
gALL_v3_c10622	6	5	-	-	-	-	-
gALL_v3_c10623	1	8	-	-	-	-	-
gALL_v3_c10624	4	3	-	-	-	-	-
gALL_v3_c10625	4	3	-	-	ref|XP_003395881.1|	PREDICTED: hypothetical protein LOC100649411 [Bombus terrestris]	9,00E-20
gALL_v3_c10626	3	6	gnl|CDD|185562	PTZ00335, PTZ00335, tubulin	emb|CAD22112.1|	tubulin [Ostertagia ostertagi]	3,00E-15
gALL_v3_c10627	9	7	-	-	ref|WP_007302981.1|	Ankyrin repeat domain protein [Wolbachia endosymbiont of Culex quinquefasciatus] gb|EEB55313.1| Ankyrin repeat domain protein [Wolbachia endosymbiont of Culex q	6,00E-18
gALL_v3_c10628	2	3	-	-	-	-	-
gALL_v3_c1063	7	8	-	-	-	-	-
gALL_v3_c10630	3	8	-	-	-	-	-
gALL_v3_c10631	10	9	gnl|CDD|178523	PLN02935, PLN02935, Bifuncti	ref|XP_002424361.1|	NADH kinase, putative [Pediculus humanus corporis] gb|EEB11623.1| NADH kinase, putative [Pediculus humanus corporis]	1,00E-178
gALL_v3_c10632	5	5	-	-	-	-	-
gALL_v3_c106328	0	2	-	-	-	-	-
gALL_v3_c10633	6	14	-	-	ref|XP_006132930.1|	PREDICTED: jerky protein-like [Pelodiscus sinensis]	8,00E-17
gALL_v3_c10634	4	6	gnl|CDD|150167	pfam09404, DUF2003, Eukary	gb|EFN81023.1|	UPF0536 protein C12orf66-like protein [Harpegnathos saltator]	7,00E-70
gALL_v3_c10635	5	7	gnl|CDD|191053	pfam04652, DUF605, Vta1 like	ref|XP_002069166.1|	GK23648 [Drosophila willistoni] gb|EDW80152.1| GK23648 [Drosophila willistoni]	4,00E-40
gALL_v3_c10636	7	11	-	-	-	-	-
gALL_v3_c10637	1	4	gnl|CDD|34495	COG4886, COG4886, Leucine-r	gb|EFX83378.1|	hypothetical protein DAPPUDRAFT_315899 [Daphnia pulex]	1,00E-72
gALL_v3_c10639	8	10	-	-	-	-	-
gALL_v3_c1064	4	4	gnl|CDD|192040	pfam08441, Integrin_alpha2, I	ref|XP_002426509.1|	Integrin alpha-PS1 precursor, putative [Pediculus humanus corporis] gb|EEB13771.1| Integrin alpha-PS1 precursor, putative [Pediculus humanus corporis]	2,00E-49
gALL_v3_c10641	15	14	-	-	-	-	-
gALL_v3_c10643	5	8	-	-	-	-	-
gALL_v3_c10644	3	3	-	-	-	-	-
gALL_v3_c10645	2	10	-	-	-	-	-
gALL_v3_c10647	6	10	gnl|CDD|177894	PLN02252, PLN02252, nitrate	gb|AEE62055.1|	unknown [Dendroctonus ponderosae]	2,00E-88
gALL_v3_c10649	8	6	-	-	-	-	-
gALL_v3_c10650	3	2	-	-	-	-	-
gALL_v3_c10651	4	2	-	-	-	-	-
gALL_v3_c10653	1	3	gnl|CDD|48447	cd03749, proteasome_alpha_	ref|XP_001325659.1|	Family T1, proteasome alpha subunit, threonine peptidase [Trichomonas vaginalis G3] gb|EAY13436.1| Family T1, proteasome alpha subunit, threonine peptidase [Trichom	2,00E-58
gALL_v3_c10654	2	3	-	-	-	-	-
gALL_v3_c10655	1	8	gnl|CDD|176897	cd08888, SRPBCC_PITPNA-B_I	ref|XP_005178209.1|	PREDICTED: phosphatidylinositol transfer protein beta isoform-like isoform X2 [Musca domestica]	1,00E-148
gALL_v3_c10656	5	5	-	-	-	-	-
gALL_v3_c10657	0	4	-	-	-	-	-
gALL_v3_c10658	4	12	gnl|CDD|175974	cd00275, C2_PLC_like, C2 dom	ref|XP_003487323.1|	PREDICTED: 1-phosphatidylinositol-4,5-bisphosphate phosphodiesterase-like [Bombus impatiens]	5,00E-99
gALL_v3_c1066	6	10	-	-	gb|EFN64304.1|	Probable G-protein coupled receptor 158 [Camponotus floridanus]	0
gALL_v3_c10660	7	15	-	-	ref|XP_001949072.2|	PREDICTED: fatty acid synthase-like [Acyrthosiphon pisum]	1,00E-14
gALL_v3_c10661	1	5	-	-	-	-	-
gALL_v3_c10662	2	6	gnl|CDD|190975	pfam04410, Gar1, Gar1/Naf1 I	ref|XP_004923979.1|	PREDICTED: H/ACA ribonucleoprotein complex non-core subunit NAF1-like [Bombyx mori]	1,00E-37
gALL_v3_c10663	3	3	gnl|CDD|175980	cd04013, C2_SynGAP_like, C2	ref|XP_001945701.2|	PREDICTED: probable Ras GTPase-activating protein-like [Acyrthosiphon pisum]	1,00E-95
gALL_v3_c10664	3	4	-	-	-	-	-
gALL_v3_c10666	3	5	-	-	-	-	-
gALL_v3_c10667	3	4	-	-	-	-	-
gALL_v3_c10668	2	5	-	-	-	-	-
gALL_v3_c10669	2	4	-	-	-	-	-
gALL_v3_c1067	10	11	-	-	ref|XP_004923856.1|	PREDICTED: piggyBac transposable element-derived protein 4-like isoform X1 [Bombyx mori]	1,00E-09
gALL_v3_c10670	2	7	-	-	-	-	-
gALL_v3_c10671	0	4	-	-	-	-	-
gALL_v3_c10672	4	3	-	-	-	-	-
gALL_v3_c10673	2	4	-	-	-	-	-
gALL_v3_c10674	7	8	-	-	-	-	-
gALL_v3_c10675	0	6	gnl|CDD|192350	pfam09730, BicD, Microtubule	ref|XP_001650055.1|	hypothetical protein AaeL_AAEL004936 [Aedes aegypti] gb|EAT43661.1| AAEL004936-PA [Aedes aegypti]	2,00E-50
gALL_v3_c10676	11	13	gnl|CDD|29004	cd02394, vigilin_like_KH, K ho	gb|EFN61569.1|	Vigilin [Camponotus floridanus]	1,00E-120
gALL_v3_c10677	1	5	-	-	-	-	-
gALL_v3_c106770	3	1	-	-	-	-	-
gALL_v3_c10678	10	7	gnl|CDD|175990	cd04024, C2A_Synaptotagmin	ref|XP_002431395.1|	conserved hypothetical protein [Pediculus humanus corporis] gb|EEB18657.1| conserved hypothetical protein [Pediculus humanus corporis]	1,00E-129
gALL_v3_c10679	2	5	-	-	-	-	-
gALL_v3_c1068	19	20	gnl|CDD|178955	PRK00277, clpP, ATP-depender	ref|XP_002428409.1|	ATP-dependent Clp protease proteolytic subunit, putative [Pediculus humanus corporis] gb|EEB15671.1| ATP-dependent Clp protease proteolytic subunit, putative [Pedicu	3,00E-82
gALL_v3_c10680	3	2	-	-	-	-	-
gALL_v3_c10681	4	3	-	-	-	-	-
gALL_v3_c10682	2	6	-	-	-	-	-
gALL_v3_c10683	2	5	-	-	-	-	-
gALL_v3_c10684	4	9	-	-	-	-	-
gALL_v3_c10685	3	3	-	-	-	-	-
gALL_v3_c10686	5	7	-	-	-	-	-
gALL_v3_c10687	14	10	-	-	-	-	-
gALL_v3_c10689	2	5	-	-	-	-	-
gALL_v3_c1069	17	10	gnl|CDD|148211	pfam06468, Spond_N, Spondi	ref|XP_975464.2|	PREDICTED: similar to f-spondin [Tribolium castaneum] gb|EFA01753.1| hypothetical protein TcasGA2_TC007351 [Tribolium castaneum]	0
gALL_v3_c10691	6	5	-	-	-	-	-
gALL_v3_c10692	6	13	gnl|CDD|145880	pfam02956, TT_ORF1, TT viral	dbj|BAF82021.1|	piggyBac transposase Uribo1 [Xenopus borealis]	9,00E-29
gALL_v3_c10694	3	2	-	-	-	-	-
gALL_v3_c10695	16	16	-	-	-	-	-
gALL_v3_c10697	3	4	-	-	-	-	-
gALL_v3_c10699	10	11	gnl|CDD|140280	PTZ00253, PTZ00253, tryparec	ref|XP_003699647.1|	PREDICTED: peroxiredoxin 1-like [Megachile rotundata]	2,00E-19
gALL_v3_c1070	5	3	-	-	-	-	-
gALL_v3_c10700	4	2	gnl|CDD|187665	cd09805, type2_17beta_HSD-	ref|XP_973118.1|	PREDICTED: similar to GA11371-PA [Tribolium castaneum]	7,00E-41
gALL_v3_c10701	2	4	-	-	-	-	-
gALL_v3_c10702	6	4	-	-	-	-	-
gALL_v3_c10703	5	7	gnl|CDD|188911	cd09512, SAM_Neurabin-like,	ref|XP_003398192.1|	PREDICTED: hypothetical protein LOC100646066 [Bombus terrestris]	6,00E-36
gALL_v3_c10706	5	7	-	-	-	-	-
gALL_v3_c10707	4	10	-	-	-	-	-
gALL_v3_c10708	3	5	-	-	-	-	-
gALL_v3_c10709	3	4	-	-	-	-	-

gALL_v3_c1071	3	7	-	-	-	-	-
gALL_v3_c10710	5	8	-	-	-	-	-
gALL_v3_c10712	4	12	-	-	-	-	-
gALL_v3_c10713	4	5	-	-	-	-	-
gALL_v3_c107134	1	1	-	-	-	-	-
gALL_v3_c10714	3	5	-	-	ref|XP_006616793.1|	PREDICTED: GPI ethanolamine phosphate transferase 2-like [Apis dorsata]	3,00E-25
gALL_v3_c10715	7	4	-	-	-	-	-
gALL_v3_c107157	0	2	-	-	-	-	-
gALL_v3_c10716	6	4	-	-	-	-	-
gALL_v3_c10718	3	9	-	-	-	-	-
gALL_v3_c10719	0	4	-	-	-	-	-
gALL_v3_c1072	8	4	-	-	-	-	-
gALL_v3_c10720	3	7	-	-	-	-	-
gALL_v3_c10721	11	18	-	-	-	-	-
gALL_v3_c10722	3	4	gnl|CDD|190978	pfam04418, DUF543, Domain	ref|XP_001808987.1|	PREDICTED: similar to chromosome 1 open reading frame 151 protein [Tribolium castaneum]	2,00E-18
gALL_v3_c10723	2	3	-	-	-	-	-
gALL_v3_c10724	3	4	-	-	-	-	-
gALL_v3_c10725	4	4	-	-	gb|EFN66873.1|	DmX-like protein 1 [Camponotus floridanus]	1,00E-119
gALL_v3_c10726	9	9	-	-	-	-	-
gALL_v3_c10727	4	8	gnl|CDD|190193	pfam02026, RyR, RyR domain.	ref|XP_006569095.1|	PREDICTED: ryanodine receptor 44F isoform X10 [Apis mellifera]	1,00E-144
gALL_v3_c10728	6	8	-	-	-	-	-
gALL_v3_c10729	7	12	-	-	ref|XP_003696144.1|	PREDICTED: homeodomain-only protein-like isoform 2 [Apis florea]	4,00E-24
gALL_v3_c1073	7	9	-	-	gb|EFA01218.1|	hypothetical protein TcasGA2_TC010545 [Tribolium castaneum]	3,00E-66
gALL_v3_c10730	10	9	-	-	-	-	-
gALL_v3_c10731	3	3	-	-	-	-	-
gALL_v3_c10732	3	5	-	-	-	-	-
gALL_v3_c10733	16	20	-	-	ref|XP_003708540.1|	PREDICTED: piggyBac transposable element-derived protein 4-like [Megachile rotundata]	4,00E-07
gALL_v3_c10734	2	5	gnl|CDD|28913	cd00031, CA, Cadherin repeat	ref|XP_001996747.1|	GH25257 [Drosophila grimshawi] gb|EDV90833.1| GH25257 [Drosophila grimshawi]	1,00E-118
gALL_v3_c10735	5	3	-	-	-	-	-
gALL_v3_c10736	20	14	-	-	-	-	-
gALL_v3_c10737	5	10	-	-	gb|EFA10673.1|	muscle-specific protein 300 [Tribolium castaneum]	3,00E-78
gALL_v3_c10738	3	2	-	-	-	-	-
gALL_v3_c1074	4	4	gnl|CDD|148752	pfam07324, DGCR6, DiGeorge	ref|XP_004931958.1|	PREDICTED: gonadal protein gdl-like [Bombyx mori]	2,00E-84
gALL_v3_c10740	4	9	-	-	-	-	-
gALL_v3_c10741	3	4	-	-	-	-	-
gALL_v3_c10742	2	4	-	-	-	-	-
gALL_v3_c10744	3	5	-	-	-	-	-
gALL_v3_c10745	4	4	-	-	-	-	-
gALL_v3_c10746	0	5	-	-	ref|XP_003246752.1|	PREDICTED: hypothetical protein LOC100161093 isoform 2 [Acyrthosiphon pisum]	2,00E-14
gALL_v3_c10747	6	7	-	-	-	-	-
gALL_v3_c107474	1	1	-	-	-	-	-
gALL_v3_c10748	8	10	-	-	-	-	-
gALL_v3_c10749	1	5	-	-	-	-	-
gALL_v3_c1075	7	20	-	-	gb|EGI60079.1|	Zinc finger protein on ecdysone puffs [Acromyrmex echinatior]	2,00E-54
gALL_v3_c10751	8	6	-	-	-	-	-
gALL_v3_c10752	3	5	-	-	-	-	-
gALL_v3_c10754	6	3	-	-	-	-	-
gALL_v3_c10755	3	4	gnl|CDD|153327	cd07643, I-BAR_IMD_MIM, In	gb|EFN79620.1|	Metastasis suppressor protein 1 [Harpegnathos saltator]	1,00E-63
gALL_v3_c10756	2	4	-	-	-	-	-
gALL_v3_c10757	3	6	-	-	-	-	-
gALL_v3_c10759	33	35	-	-	-	-	-
gALL_v3_c1076	9	14	gnl|CDD|173412	PTZ00121, PTZ00121, MAEBL;	gb|ABK41431.1|	glutamic acid reigh protein-like protein [Trichinella spiralis]	2,00E-12
gALL_v3_c107601	1	1	-	-	-	-	-
gALL_v3_c10761	10	24	-	-	-	-	-
gALL_v3_c10762	0	6	-	-	-	-	-
gALL_v3_c107628	1	1	-	-	-	-	-
gALL_v3_c10763	4	3	-	-	-	-	-
gALL_v3_c10764	12	8	-	-	-	-	-
gALL_v3_c10765	4	5	-	-	-	-	-
gALL_v3_c10766	5	3	gnl|CDD|133004	cd02510, pp-GalNAc-T, pp-Gal	ref|XP_003705600.1|	PREDICTED: N-acetylgalactosaminyltransferase 7-like [Megachile rotundata]	6,00E-92
gALL_v3_c10768	0	4	-	-	-	-	-
gALL_v3_c1077	6	17	-	-	-	-	-
gALL_v3_c10771	16	14	gnl|CDD|162739	TIGR02168, SMC_prok_B, chrc	ref|XP_002425085.1|	conserved hypothetical protein [Pediculus humanus corporis] gb|EEB12347.1| conserved hypothetical protein [Pediculus humanus corporis]	3,00E-39
gALL_v3_c10772	2	6	-	-	-	-	-
gALL_v3_c10773	1	4	-	-	-	-	-
gALL_v3_c10776	2	4	-	-	-	-	-
gALL_v3_c10777	2	3	-	-	-	-	-
gALL_v3_c107778	1	1	-	-	-	-	-
gALL_v3_c107786	1	1	-	-	-	-	-
gALL_v3_c10779	4	5	-	-	-	-	-
gALL_v3_c107792	1	1	-	-	-	-	-
gALL_v3_c1078	14	14	gnl|CDD|176215	cd08253, zeta_crystallin, Zeta-	ref|XP_003208977.1|	PREDICTED: quinone oxidoreductase-like [Meleagris gallopavo]	1,00E-103
gALL_v3_c107814	1	2	-	-	gb|AAO20251.1|	cytochrome P450 monooxygenase CYP4G19 [Blattella germanica]	1,00E-11
gALL_v3_c10782	10	7	gnl|CDD|192378	pfam09787, Golgin_A5, Golgir	gb|EGI70171.1|	Golgin subfamily A member 5 [Acromyrmex echinatior]	7,00E-54
gALL_v3_c10783	3	6	-	-	-	-	-
gALL_v3_c10784	2	8	-	-	-	-	-
gALL_v3_c10785	1	5	-	-	-	-	-
gALL_v3_c10786	4	6	gnl|CDD|193286	pfam12810, Gly_rich, Glycine	-	-	-
gALL_v3_c10787	2	4	-	-	-	-	-
gALL_v3_c107880	2	1	-	-	gb|AGM32816.1|	hypothetical protein [Coptotermes formosanus]	1,00E-07
gALL_v3_c10789	2	4	-	-	ref|XP_974863.1|	PREDICTED: similar to CG9448 CG9448-PA [Tribolium castaneum] gb|EFA09636.1| hypothetical protein TcasGA2_TC011759 [Tribolium castaneum]	6,00E-89
gALL_v3_c1079	7	18	-	-	-	-	-
gALL_v3_c10790	3	2	-	-	-	-	-
gALL_v3_c10791	4	3	-	-	-	-	-
gALL_v3_c10793	7	10	-	-	-	-	-
gALL_v3_c10794	4	3	-	-	-	-	-
gALL_v3_c10795	9	17	-	-	gb|EFA04463.1|	hypothetical protein TcasGA2_TC014767 [Tribolium castaneum]	1,00E-31
gALL_v3_c10796	2	4	-	-	-	-	-
gALL_v3_c107960	1	1	-	-	gb|AGM32080.1|	hemolymph juvenile hormone binding protein [Coptotermes formosanus]	2,00E-12
gALL_v3_c10797	8	9	-	-	-	-	-
gALL_v3_c107975	0	2	-	-	-	-	-
gALL_v3_c10798	2	3	gnl|CDD|176924	cd09071, FAR_C, C-terminal d	ref|XP_001602857.1|	PREDICTED: putative fatty acyl-CoA reductase CG5065-like [Nasonia vitripennis]	5,00E-23
gALL_v3_c107999	0	2	-	-	-	-	-
gALL_v3_c108	15	19	-	-	ref|XP_003491007.1|	PREDICTED: hypothetical protein LOC100743631 [Bombus impatiens]	2,00E-20
gALL_v3_c1080	3	12	-	-	ref|XP_002424562.1|	tartan protein, putative [Pediculus humanus corporis] gb|EEB11824.1| tartan protein, putative [Pediculus humanus corporis]	1,00E-37
gALL_v3_c10800	1	5	-	-	-	-	-
gALL_v3_c10802	5	12	-	-	-	-	-
gALL_v3_c10803	20	15	-	-	gb|AFA41503.1|	stau [Laodelphax striatella]	1,00E-33
gALL_v3_c10804	6	13	gnl|CDD|189452	pfam00211, Guanylate_cyc, Ad	ref|XP_002431914.1|	adenylate cyclase type, putative [Pediculus humanus corporis] gb|EEB19176.1| adenylate cyclase type, putative [Pediculus humanus corporis]	0
gALL_v3_c10805	4	3	-	-	-	-	-
gALL_v3_c10806	20	15	-	-	-	-	-
gALL_v3_c108062	1	1	-	-	-	-	-
gALL_v3_c10808	1	4	-	-	ref|XP_309555.4|	AGAP011095-PA [Anopheles gambiae str. PEST] gb|EAA05200.4| AGAP011095-PA [Anopheles gambiae str. PEST]	1,00E-18
gALL_v3_c10809	4	3	-	-	-	-	-
gALL_v3_c1081	8	17	-	-	ref|XP_002432670.1|	conserved hypothetical protein [Pediculus humanus corporis] gb|EEB19932.1| conserved hypothetical protein [Pediculus humanus corporis]	2,00E-07
gALL_v3_c10810	3	6	-	-	-	-	-
gALL_v3_c10811	4	8	-	-	gb|AGM32926.1|	hypothetical protein [Coptotermes formosanus]	8,00E-06
gALL_v3_c10812	3	5	-	-	-	-	-
gALL_v3_c10813	2	3	-	-	-	-	-
gALL_v3_c108133	1	1	-	-	-	-	-
gALL_v3_c10814	3	6	-	-	-	-	-
gALL_v3_c10815	4	11	gnl|CDD|143937	pfam00170, bZIP_1, bZIP trans	gb|AHB17949.1|	early growth response 1 [Locusta migratoria]	2,00E-52
gALL_v3_c10816	2	4	gnl|CDD|149560	pfam08547, CIA30, Complex I	ref|XP_975544.1|	PREDICTED: similar to CG7598 CG7598-PA [Tribolium castaneum]	1,00E-105
gALL_v3_c10817	2	5	-	-	-	-	-
gALL_v3_c10818	5	4	-	-	-	-	-
gALL_v3_c10819	6	5	gnl|CDD|29261	cd00204, ANK, ankyrin repeat:	gb|EGI65225.1|	BCL-6 corepressor [Acromyrmex echinatior]	1,00E-104
gALL_v3_c10821	4	6	-	-	-	-	-
gALL_v3_c108212	1	1	-	-	gb|EFN65619.1|	Putative uncharacterized transposon-derived protein F52C9.6 [Camponotus floridanus]	6,00E-12
gALL_v3_c10822	0	6	-	-	-	-	-
gALL_v3_c10823	5	4	-	-	gb|EFA00913.1|	hypothetical protein TcasGA2_TC003819 [Tribolium castaneum]	9,00E-18
gALL_v3_c10824	5	13	-	-	-	-	-
gALL_v3_c10825	13	16	-	-	-	-	-
gALL_v3_c10826	5	3	-	-	-	-	-
gALL_v3_c10827	4	7	-	-	-	-	-
gALL_v3_c10828	2	6	-	-	-	-	-
gALL_v3_c108286	1	1	-	-	-	-	-
gALL_v3_c10829	1	4	-	-	-	-	-
gALL_v3_c1083	9	18	-	-	-	-	-
gALL_v3_c10830	3	2	gnl|CDD|177889	PLN02246, PLN02246, 4-coum	ref|XP_001604694.1|	PREDICTED: probable 4-coumarate--CoA ligase 3-like [Nasonia vitripennis]	5,00E-49
gALL_v3_c10831	4	4	-	-	-	-	-
gALL_v3_c10832	5	9	-	-	-	-	-
gALL_v3_c10833	2	4	-	-	-	-	-
gALL_v3_c10834	4	4	gnl|CDD|162129	TIGR00954, 3a01203, Peroxys	ref|XP_005198344.1|	PREDICTED: ATP-binding cassette sub-family D member 2-like isoform X1 [Bos taurus]	4,00E-38
gALL_v3_c10835	1	4	-	-	-	-	-
gALL_v3_c10836	1	4	gnl|CDD|29261	cd00204, ANK, ankyrin repeat:	pdb|3Q9U|C	Chain C, In Silico And In Vitro Co-Evolution Of A High Affinity Complementary Protein-Protein Interface pdb|3Q9U|D Chain D, In Silico And In Vitro Co-Evolution Of A High A	4,00E-10
gALL_v3_c108367	2	2	-	-	gb|EFN65619.1|	Putative uncharacterized transposon-derived protein F52C9.6 [Camponotus floridanus]	2,00E-30
gALL_v3_c10837	4	2	-	-	gb|EFN66701.1|	Golgi apparatus protein 1 [Camponotus floridanus]	2,00E-81
gALL_v3_c10838	7	7	-	-	-	-	-
gALL_v3_c10839	3	8	-	-	-	-	-
gALL_v3_c1084	13	21	-	-	-	-	-
gALL_v3_c10840	5	4	gnl|CDD|180384	PRK06074, PRK06074, NADH c	ref|XP_001352740.1|	GA11380 [Drosophila pseudoobscura pseudoobscura] gb|EAL30240.1| GA11380 [Drosophila pseudoobscura pseudoobscura]	1,00E-84
gALL_v3_c10842	3	3	-	-	-	-	-
gALL_v3_c10843	2	6	-	-	ref|XP_003488017.1|	PREDICTED: patched domain-containing protein 3-like [Bombus impatiens]	1,00E-09
gALL_v3_c10844	4	3	gnl|CDD|128654	smart00360, RRM, RNA recogr	ref|XP_002431775.1|	RNA-binding protein, putative [Pediculus humanus corporis] gb|EEB19037.1| RNA-binding protein, putative [Pediculus humanus corporis]	4,00E-28

gALL_v3_c10845	2	7	-	-	ref|XP_001599701.2|	PREDICTED: hypothetical protein LOC100114793 [Nasonia vitripennis]	3,00E-14
gALL_v3_c10846	3	2	-	-	gb|EEZ97431.1|	hypothetical protein TcasGA2_TC011261 [Tribolium castaneum]	2,00E-13
gALL_v3_c10848	6	6	-	-	-	-	-
gALL_v3_c10849	5	7	-	-	-	-	-
gALL_v3_c1085	9	22	-	-	ref|XP_003698758.1|	PREDICTED: LOW QUALITY PROTEIN: sterile alpha and TIR motif-containing protein 1-like [Apis florea]	2,00E-73
gALL_v3_c10850	6	9	-	-	-	-	-
gALL_v3_c10851	7	5	-	-	-	-	-
gALL_v3_c10852	3	10	-	-	-	-	-
gALL_v3_c10853	3	4	-	-	-	-	-
gALL_v3_c108534	1	1	-	-	-	-	-
gALL_v3_c10855	4	8	-	-	ref|XP_974328.1|	PREDICTED: similar to CG6621 CG6621-PA [Tribolium castaneum] gb|EFA03920.1| hypothetical protein TcasGA2_TC014056 [Tribolium castaneum]	1,00E-08
gALL_v3_c10856	4	7	gnl|CDD|162469	TIGR01657, P-ATPase-V, P-type	gb|EKC41107.1|	Putative cation-transporting ATPase 13A3, partial [Crassostrea gigas]	1,00E-42
gALL_v3_c10857	4	2	gnl|CDD|189687	pfam00723, Glyco_hydro_15,	ref|XP_003426334.1|	PREDICTED: probable phosphorylase b kinase regulatory subunit alpha-like isoform 2 [Nasonia vitripennis]	4,00E-64
gALL_v3_c10858	4	3	-	-	ref|XP_003699011.1|	PREDICTED: uncharacterized protein LOC100868231 [Apis florea]	6,00E-07
gALL_v3_c1086	12	11	-	-	-	-	-
gALL_v3_c10860	3	3	-	-	ref|XP_002430696.1|	hypothetical protein Phum_PHUM495260 [Pediculus humanus corporis] gb|EEB17958.1| hypothetical protein Phum_PHUM495260 [Pediculus humanus corporis]	6,00E-13
gALL_v3_c10861	4	7	gnl|CDD|189646	pfam00637, Clathrin, Region i	gb|AHC70342.1|	clathrin, partial [Locusta migratoria]	1,00E-126
gALL_v3_c10864	5	3	-	-	-	-	-
gALL_v3_c108640	2	1	-	-	-	-	-
gALL_v3_c10865	5	2	gnl|CDD|147282	pfam05020, zf-NPL4, NPL4 fan	ref|XP_003706253.1|	PREDICTED: nuclear protein localization protein 4 homolog [Megachile rotundata]	1,00E-104
gALL_v3_c10866	5	3	gnl|CDD|180422	PRK06142, PRK06142, enoyl-C	dbj|BAH72249.1|	ACYPI005128 [Acyrthosiphon pisum]	3,00E-41
gALL_v3_c10867	5	2	gnl|CDD|48455	cd03757, proteasome_beta_ty	gb|EFZ19173.1|	hypothetical protein SINV_12036 [Solenopsis invicta]	2,00E-24
gALL_v3_c10868	59	97	gnl|CDD|100016	cd02187, beta_tubulin, The tu	gb|ABN11939.1|	putative beta-1 tubulin [Maconellicoccus hirsutus]	0
gALL_v3_c10869	4	5	-	-	-	-	-
gALL_v3_c108696	1	1	-	-	-	-	-
gALL_v3_c1087	16	13	-	-	gb|ENN73329.1|	hypothetical protein YQE_10091, partial [Dendroctonus ponderosae]	0
gALL_v3_c10870	2	8	gnl|CDD|31397	COG1204, COG1204, Superfan	ref|XP_002429968.1|	U520, putative [Pediculus humanus corporis] gb|EEB17230.1| U520, putative [Pediculus humanus corporis]	1,00E-152
gALL_v3_c10871	3	10	gnl|CDD|144648	pfam01130, CD36, CD36 famil	ref|XP_001948133.1|	PREDICTED: scavenger receptor class B member 1-like [Acyrthosiphon pisum]	4,00E-22
gALL_v3_c10873	3	7	-	-	-	-	-
gALL_v3_c10874	8	5	gnl|CDD|185453	PTZ00110, PTZ00110, helicase	ref|XP_003701916.1|	PREDICTED: probable ATP-dependent RNA helicase DDX5-like [Megachile rotundata]	1,00E-104
gALL_v3_c10875	6	4	gnl|CDD|192870	pfam11904, DUF3424, Protein	ref|XP_003706068.1|	PREDICTED: ankyrin repeat domain-containing protein 13B-like [Megachile rotundata]	2,00E-90
gALL_v3_c10876	5	5	gnl|CDD|146640	pfam04109, APG9, Autophagy	ref|XP_006607285.1|	PREDICTED: autophagy-related protein 9A-like isoform X1 [Apis dorsata] ref|XP_006607286.1| PREDICTED: autophagy-related protein 9A-like isoform X2 [Apis dorsata] ref	1,00E-91
gALL_v3_c10878	4	3	-	-	-	-	-
gALL_v3_c10879	6	5	-	-	gb|AGM32821.1|	hypothetical protein [Coptotermes formosanus]	8,00E-24
gALL_v3_c1088	5	15	-	-	-	-	-
gALL_v3_c10880	4	6	-	-	-	-	-
gALL_v3_c10881	5	2	gnl|CDD|192904	pfam11979, DUF3480, Domain	gb|EFN80026.1|	Zinc finger FYVE domain-containing protein 9 [Harpegnathos saltator]	6,00E-40
gALL_v3_c10882	9	8	gnl|CDD|190617	pfam03372, Exo_endo_phos, I	gb|EFA11632.1|	hypothetical protein TcasGA2_TC000010 [Tribolium castaneum]	2,00E-20
gALL_v3_c10883	10	12	-	-	-	-	-
gALL_v3_c10885	5	5	-	-	-	-	-
gALL_v3_c10886	4	2	-	-	-	-	-
gALL_v3_c10887	4	2	-	-	-	-	-
gALL_v3_c10888	4	5	-	-	-	-	-
gALL_v3_c1089	7	6	gnl|CDD|189810	pfam01030, Recep_L_domain,	ref|XP_006568757.1|	PREDICTED: insulin-like receptor-like isoform X1 [Apis mellifera] ref|XP_006568758.1| PREDICTED: insulin-like receptor-like isoform X2 [Apis mellifera]	3,00E-61
gALL_v3_c10890	3	5	-	-	-	-	-
gALL_v3_c10891	3	3	-	-	-	-	-
gALL_v3_c10892	5	3	-	-	-	-	-
gALL_v3_c10894	4	2	-	-	ref|XP_001844049.1|	major facilitator superfamily domain-containing protein 1 [Culex quinquefasciatus] gb|EDS35718.1| major facilitator superfamily domain-containing protein 1 [Culex quinq	1,00E-41
gALL_v3_c10896	4	4	-	-	-	-	-
gALL_v3_c10897	5	7	gnl|CDD|34495	COG4886, COG4886, Leucine-	gb|EFA11987.1|	hypothetical protein TcasGA2_TC014824 [Tribolium castaneum]	2,00E-69
gALL_v3_c10898	4	3	-	-	-	-	-
gALL_v3_c109	10	9	gnl|CDD|149105	pfam07851, TMPIT, TMPIT-like	ref|XP_001607631.1|	PREDICTED: transmembrane protein 120 homolog [Nasonia vitripennis]	1,00E-131
gALL_v3_c1090	5	3	gnl|CDD|188046	TIGR00365, TIGR00365, mono	ref|XP_001605234.1|	PREDICTED: glutaredoxin-related protein 5, mitochondrial-like [Nasonia vitripennis]	2,00E-62
gALL_v3_c10900	6	3	-	-	-	-	-
gALL_v3_c10901	1	4	-	-	ref|XP_002068884.1|	GK17789 [Drosophila willistoni] gb|EDW79870.1| GK17789 [Drosophila willistoni]	3,00E-08
gALL_v3_c10902	4	2	-	-	-	-	-
gALL_v3_c10904	3	5	-	-	-	-	-
gALL_v3_c10905	15	15	-	-	gb|ESO88633.1|	hypothetical protein LOTGIDRAFT_125742, partial [Lottia gigantea]	6,00E-07
gALL_v3_c10906	4	14	gnl|CDD|177721	PLN00104, PLN00104, MYST -I	ref|XP_001601199.2|	PREDICTED: hypothetical protein LOC100116786 [Nasonia vitripennis]	4,00E-92
gALL_v3_c10907	3	3	gnl|CDD|149648	pfam08662, eIF2A, Eukaryotic	ref|XP_003707307.1|	PREDICTED: eukaryotic translation initiation factor 2A-like [Megachile rotundata]	6,00E-90
gALL_v3_c10908	6	5	-	-	-	-	-
gALL_v3_c10909	6	11	-	-	emb|CAM36311.1|	hypothetical protein [Thermobia domestica]	2,00E-09
gALL_v3_c1091	10	10	-	-	-	-	-
gALL_v3_c10911	2	4	-	-	-	-	-
gALL_v3_c10913	4	14	-	-	-	-	-
gALL_v3_c10914	5	6	gnl|CDD|163615	cd00839, MPP_PAPs, purple a	gb|ETN59787.1|	purple acid phosphatase [Anopheles darlingi]	9,00E-99
gALL_v3_c10915	3	3	-	-	-	-	-
gALL_v3_c10916	10	9	-	-	-	-	-
gALL_v3_c10918	1	6	gnl|CDD|189654	pfam00651, BTB, BTB/POZ dor	ref|XP_001812940.1|	PREDICTED: similar to bric-a-brac [Tribolium castaneum]	1,00E-59
gALL_v3_c10919	7	6	-	-	-	-	-
gALL_v3_c1092	8	7	-	-	gb|EFZ20505.1|	hypothetical protein SINV_04265 [Solenopsis invicta]	1,00E-25
gALL_v3_c10920	4	9	-	-	-	-	-
gALL_v3_c10921	7	12	-	-	-	-	-
gALL_v3_c10922	3	4	gnl|CDD|189635	pfam00621, RhoGEF, RhoGEF (	ref|XP_006622045.1|	PREDICTED: protein ECT2-like isoform X8 [Apis dorsata]	8,00E-72
gALL_v3_c10923	2	5	-	-	-	-	-
gALL_v3_c10924	7	6	-	-	ref|XP_003699521.1|	PREDICTED: uncharacterized protein LOC100876226 [Megachile rotundata]	3,00E-22
gALL_v3_c10925	7	10	gnl|CDD|73156	cd01650, RT_nLTR_like, RT_nL	gb|ADI61823.1|	endonuclease-reverse transcriptase [Bombyx mori]	5,00E-63
gALL_v3_c10928	8	5	gnl|CDD|162459	TIGR01642, U2AF_lg, U2 snRN	gb|EHJ63283.1|	hypothetical protein KGM_19550 [Danaus plexippus]	4,00E-12
gALL_v3_c10929	5	7	gnl|CDD|190441	pfam02820, MBT, mbt repeat.	ref|XP_002056344.1|	GJ10281 [Drosophila virilis] gb|EDW59456.1| GJ10281 [Drosophila virilis]	3,00E-91
gALL_v3_c1093	26	32	-	-	-	-	-
gALL_v3_c10930	12	8	-	-	-	-	-
gALL_v3_c10931	1	4	-	-	-	-	-
gALL_v3_c10932	11	6	-	-	-	-	-
gALL_v3_c10933	2	6	-	-	-	-	-
gALL_v3_c10934	4	7	-	-	-	-	-
gALL_v3_c10936	6	8	-	-	gb|EGI69446.1|	Pecanex-like protein 1 [Acromyrmex echinatior]	5,00E-42
gALL_v3_c10937	5	2	-	-	-	-	-
gALL_v3_c10938	2	7	-	-	-	-	-
gALL_v3_c10939	4	3	-	-	-	-	-
gALL_v3_c1094	23	24	-	-	-	-	-
gALL_v3_c10940	8	7	-	-	ref|XP_001662669.1|	Juvenile hormone-inducible protein, putative [Aedes aegypti] gb|EAT45857.1| AAEL002904-PA [Aedes aegypti]	6,00E-07
gALL_v3_c10941	5	3	-	-	-	-	-
gALL_v3_c10942	3	4	-	-	-	-	-
gALL_v3_c10943	2	3	-	-	-	-	-
gALL_v3_c10944	4	2	-	-	-	-	-
gALL_v3_c10945	3	5	gnl|CDD|29015	cd00116, LRR_RI, Leucine-rich	ref|XP_001950955.1|	PREDICTED: platelet glycoprotein V-like [Acyrthosiphon pisum]	1,00E-81
gALL_v3_c10946	2	8	gnl|CDD|189715	pfam00780, CNH, CNH domair	gb|EEZ97268.1|	hypothetical protein TcasGA2_TC011069 [Tribolium castaneum]	2,00E-80
gALL_v3_c10947	5	5	gnl|CDD|133249	cd00066, G-alpha, G protein a	gb|EHJ68893.1|	guanine nucleotide-binding protein G(o) subunit alpha 1 [Danaus plexippus]	1,00E-114
gALL_v3_c1095	32	47	-	-	gb|AGM32333.1|	antioxidant enzyme (heavy metal associated) [Coptotermes formosanus]	1,00E-26
gALL_v3_c10950	5	2	-	-	-	-	-
gALL_v3_c10951	4	5	-	-	-	-	-
gALL_v3_c10953	3	3	-	-	-	-	-
gALL_v3_c10954	6	4	-	-	-	-	-
gALL_v3_c10955	2	3	-	-	-	-	-
gALL_v3_c10956	6	4	-	-	gb|AGM32283.1|	sterol desaturase, partial [Coptotermes formosanus]	1,00E-56
gALL_v3_c10958	5	4	gnl|CDD|162812	TIGR02346, chap_CCT_theta,	gb|AHB33466.1|	T-complex protein subunit theta [Locusta migratoria]	1,00E-108
gALL_v3_c10959	6	6	-	-	-	-	-
gALL_v3_c1096	8	10	-	-	gb|ADI61823.1|	endonuclease-reverse transcriptase [Bombyx mori]	6,00E-12
gALL_v3_c10961	3	8	gnl|CDD|143864	pfam00083, Sugar_tr, Sugar (a	ref|XP_002405458.1|	sugar transporter, putative [Ixodes scapularis] gb|EEC06442.1| sugar transporter, putative [Ixodes scapularis]	7,00E-72
gALL_v3_c10963	3	5	-	-	-	-	-
gALL_v3_c10964	5	8	-	-	gb|EGI63180.1|	Mariner Mos1 transposase [Acromyrmex echinatior]	1,00E-11
gALL_v3_c10965	6	4	-	-	-	-	-
gALL_v3_c10966	2	10	-	-	-	-	-
gALL_v3_c10968	3	3	-	-	-	-	-
gALL_v3_c10969	10	18	gnl|CDD|150853	pfam10243, MIP-T3, Microtub	-	-	-
gALL_v3_c1097	8	10	-	-	ref|XP_003424900.1|	PREDICTED: hypothetical protein LOC100115939 [Nasonia vitripennis]	1,00E-101
gALL_v3_c10970	9	11	-	-	ref|XP_005827594.1|	hypothetical protein GUITHDRAFT_113400 [Guillardia theta CCMP2712] gb|EKX40614.1| hypothetical protein GUITHDRAFT_113400 [Guillardia theta CCMP2712]	6,00E-06
gALL_v3_c10971	4	5	-	-	-	-	-
gALL_v3_c10972	8	11	-	-	-	-	-
gALL_v3_c10973	6	6	-	-	-	-	-
gALL_v3_c10978	8	7	-	-	-	-	-
gALL_v3_c1098	12	12	-	-	ref|XP_003702344.1|	PREDICTED: 97 kDa heat shock protein-like [Megachile rotundata]	7,00E-50
gALL_v3_c10980	5	2	-	-	gb|ADY39570.1|	hypothetical protein [Hottentotta judaicus]	3,00E-16
gALL_v3_c10981	6	9	-	-	gb|EOB02706.1|	Protein CREG1, partial [Anas platyrhynchos]	1,00E-08
gALL_v3_c10982	5	8	-	-	-	-	-
gALL_v3_c10983	3	2	-	-	ref|XP_002429764.1|	conserved hypothetical protein [Pediculus humanus corporis] gb|EEB17026.1| conserved hypothetical protein [Pediculus humanus corporis]	1,00E-22
gALL_v3_c10984	14	11	-	-	-	-	-
gALL_v3_c10985	1	6	-	-	ref|XP_001663271.1|	checkpoint kinase [Aedes aegypti] gb|EAT34725.1| AAEL013066-PA, partial [Aedes aegypti]	6,00E-26
gALL_v3_c10987	10	13	gnl|CDD|147567	pfam05454, DAG1, Dystroglyc	gb|EFN86632.1|	Dystroglycan [Harpegnathos saltator]	1,00E-147
gALL_v3_c10988	2	6	-	-	-	-	-
gALL_v3_c10989	2	4	gnl|CDD|189956	pfam01365, RYDR_ITPR, RIH d	ref|XP_003485955.1|	PREDICTED: inositol 1,4,5-trisphosphate receptor-like isoform 2 [Bombus impatiens]	1,00E-101
gALL_v3_c1099	3	10	-	-	-	-	-
gALL_v3_c10990	1	6	gnl|CDD|188829	cd09445, LIM_Mical_like_2, Tl	dbj|BAN20866.1|	conserved hypothetical protein [Riptortus pedestris]	3,00E-75
gALL_v3_c10991	29	49	gnl|CDD|185508	PTZ00194, PTZ00194, 60S ribo	ref|XP_972048.1|	PREDICTED: similar to ribosomal protein L26e [Tribolium castaneum] gb|EFA12204.1| hypothetical protein TcasGA2_TC005120 [Tribolium castaneum]	6,00E-59
gALL_v3_c10992	3	3	-	-	-	-	-
gALL_v3_c10993	2	6	-	-	-	-	-
gALL_v3_c10995	10	9	-	-	-	-	-
gALL_v3_c10996	5	5	-	-	gb|EGI62092.1|	Histone-lysine N-methyltransferase SETMAR [Acromyrmex echinatior]	3,00E-17
gALL_v3_c10997	11	9	-	-	-	-	-
gALL_v3_c10998	4	4	gnl|CDD|191638	pfam06911, Senescence, Sene	ref|XP_001656744.1|	hypothetical protein AaeL_AAEL003375 [Aedes aegypti] gb|EAT45337.1| AAEL003375-PA [Aedes aegypti]	8,00E-37
gALL_v3_c10999	7	7	gnl|CDD|73156	cd01650, RT_nLTR_like, RT_nL	gb|EFA10298.1|	hypothetical protein TcasGA2_TC012513 [Tribolium castaneum]	4,00E-35
gALL_v3_c11	2	9	-	-	ref|XP_002431247.1|	Pheromone receptor transcription factor, putative [Pediculus humanus corporis] gb|EEB18509.1| Pheromone receptor transcription factor, putative [Pediculus humanus co	6,00E-33
gALL_v3_c110	6	5	-	-	gb|ACO12154.1|	Abhydrolase domain-containing protein 14B [Lepeophtheirus salmonis]	8,00E-31
gALL_v3_c1100	32	24	-	-	ref|XP_972102.1|	PREDICTED: similar to GA12046-PA [Tribolium castaneum] gb|EFA11216.1| hypothetical protein TcasGA2_TC005180 [Tribolium castaneum]	5,00E-43

gALL_v3_c11001	2	7	-	-	-	-	-
gALL_v3_c11002	8	11	-	-	ref|WP_010755128.1|	LPXTG-domain-containing protein cell wall anchor domain [Enterococcus pallens] gb|EOH97395.1| LPXTG-domain-containing protein cell wall anchor domain [Enterococcu	9,00E-07
gALL_v3_c11003	4	5	-	-	ref|XP_006607876.1|	PREDICTED: reticulon-4-like isoform X4 [Apis dorsata]	9,00E-14
gALL_v3_c11004	6	3	-	-	ref|XP_001849829.1|	conserved hypothetical protein [Culex quinquefasciatus] gb|EDS30944.1| conserved hypothetical protein [Culex quinquefasciatus]	7,00E-09
gALL_v3_c11005	3	2	-	-	-	-	-
gALL_v3_c11006	9	7	-	-	gb|AGM32110.1|	hypothetical protein [Coptotermes formosanus]	1,00E-75
gALL_v3_c11007	4	8	-	-	ref|XP_001654724.1|	hypothetical protein AaeL_AAEL002122 [Aedes aegypti] gb|EAT46735.1| AAEL002122-PA [Aedes aegypti]	9,00E-67
gALL_v3_c11008	7	5	-	-	-	-	-
gALL_v3_c11009	1	7	-	-	-	-	-
gALL_v3_c1101	12	14	gnl|CDD|190744	pfam03770, IPK, Inositol polyp	ref|XP_001951720.2|	PREDICTED: inositol-trisphosphate 3-kinase B-like isoform 1 [Acyrthosiphon pisum]	1,00E-168
gALL_v3_c11010	1	4	-	-	-	-	-
gALL_v3_c11011	9	12	gnl|CDD|150835	pfam10223, DUF2181, Unchar	ref|XP_002427892.1|	conserved hypothetical protein [Pediculus humanus corporis] gb|EEB15154.1| conserved hypothetical protein [Pediculus humanus corporis]	1,00E-58
gALL_v3_c11013	1	7	-	-	-	-	-
gALL_v3_c11015	3	6	-	-	-	-	-
gALL_v3_c11016	3	6	gnl|CDD|32956	COG3142, CutC, Uncharacteriz	ref|XP_001121740.1|	PREDICTED: copper homeostasis protein cutC homolog [Apis mellifera]	2,00E-59
gALL_v3_c11017	4	2	gnl|CDD|48044	cd03449, R_hydratase, (R)-hyc	gb|EFN86387.1|	hypothetical protein EAI_02902 [Harpegnathos saltator]	3,00E-27
gALL_v3_c11018	5	3	-	-	ref|XP_004924617.1|	PREDICTED: uncharacterized protein LOC101738297 [Bombyx mori]	6,00E-67
gALL_v3_c11019	2	7	-	-	-	-	-
gALL_v3_c1102	11	19	gnl|CDD|29257	cd00200, WD40, WD40 domai	gb|EFA01972.1|	hypothetical protein TcasGA2_TC007593 [Tribolium castaneum]	1,00E-177
gALL_v3_c11020	7	9	gnl|CDD|161947	TIGR00596, rad1, DNA repair p	ref|XP_003705895.1|	PREDICTED: DNA repair endonuclease XPF-like [Megachile rotundata]	1,00E-136
gALL_v3_c11021	1	6	-	-	-	-	-
gALL_v3_c11022	3	3	-	-	-	-	-
gALL_v3_c11023	4	3	-	-	-	-	-
gALL_v3_c11028	6	13	gnl|CDD|128524	smart00228, PDZ, Domain pre	ref|XP_006571263.1|	PREDICTED: membrane-associated guanylate kinase, WW and PDZ domain-containing protein 2 isoform X1 [Apis mellifera] ref|XP_006571264.1| PREDICTED: membrane-a	1,00E-165
gALL_v3_c11029	2	3	gnl|CDD|34791	COG5192, BMS1, GTP-binding	ref|XP_003493481.1|	PREDICTED: hypothetical protein LOC100743997 [Bombus impatiens]	1,00E-104
gALL_v3_c11030	2	6	gnl|CDD|147983	pfam06113, BRE, Brain and re	sp|B6NXD5.1|BRE_BRAI	RecName: Full=BRCA1-A complex subunit BRE; AltName: Full=BRCA1/BRCA2-containing complex subunit 45; AltName: Full=Brain and reproductive organ-expressed protei	1,00E-31
gALL_v3_c11031	2	4	-	-	ref|XP_003485717.1|	PREDICTED: zinc finger MYND domain-containing protein 11-like [Bombus impatiens]	1,00E-28
gALL_v3_c11032	2	4	-	-	ref|XP_002430985.1|	conserved hypothetical protein [Pediculus humanus corporis] gb|EEB18247.1| conserved hypothetical protein [Pediculus humanus corporis]	7,00E-61
gALL_v3_c11033	1	4	-	-	-	-	-
gALL_v3_c11034	1	8	-	-	-	-	-
gALL_v3_c11035	4	3	-	-	-	-	-
gALL_v3_c11036	2	9	gnl|CDD|178915	PRK00174, PRK00174, acetyl-C	ref|XP_969296.1|	PREDICTED: similar to AGAP001473-PA [Tribolium castaneum] gb|EFA12019.1| hypothetical protein TcasGA2_TC001434 [Tribolium castaneum]	4,00E-33
gALL_v3_c11037	4	4	-	-	ref|XP_001947462.1|	PREDICTED: orcokinin peptides type A-like [Acyrthosiphon pisum]	9,00E-35
gALL_v3_c11038	5	6	gnl|CDD|133265	cd01862, Rab7, Rab7 subfamil	ref|XP_001607917.1|	PREDICTED: ras-related protein Rab-7a-like isoform 1 [Nasonia vitripennis] ref|XP_001607924.1| PREDICTED: ras-related protein Rab-7a-like isoform 2 [Nasonia vitripennis	1,00E-117
gALL_v3_c11039	5	3	-	-	-	-	-
gALL_v3_c1104	8	5	-	-	ref|XP_002431628.1|	protein twisted gastrulation precursor, putative [Pediculus humanus corporis] gb|EEB18890.1| protein twisted gastrulation precursor, putative [Pediculus humanus corpori	7,00E-58
gALL_v3_c11040	3	6	-	-	gb|ERL83664.1|	hypothetical protein D910_00834 [Dendroctonus ponderosae] gb|ERL83721.1| hypothetical protein D910_00934 [Dendroctonus ponderosae]	2,00E-31
gALL_v3_c11041	8	10	gnl|CDD|31444	COG1252, Ndh, NADH dehydro	ref|XP_002093671.1|	GE21427 [Drosophila yakuba] gb|EDW93383.1| GE21427 [Drosophila yakuba]	5,00E-75
gALL_v3_c11042	3	3	-	-	-	-	-
gALL_v3_c11043	5	10	-	-	ref|XP_003490137.1|	PREDICTED: hypothetical protein LOC100749055 [Bombus impatiens]	2,00E-79
gALL_v3_c11044	1	7	-	-	-	-	-
gALL_v3_c11046	8	6	-	-	gb|EGI61108.1|	Peptidyl-prolyl cis-trans isomerase, mitochondrial [Acromyrmex echinatior]	1,00E-121
gALL_v3_c11047	4	2	-	-	-	-	-
gALL_v3_c11048	3	7	-	-	-	-	-
gALL_v3_c11049	2	7	-	-	-	-	-
gALL_v3_c1105	7	12	-	-	-	-	-
gALL_v3_c11051	5	12	gnl|CDD|30447	COG0098, RpsE, Ribosomal pr	ref|XP_973859.2|	PREDICTED: similar to 28S ribosomal protein S5 [Tribolium castaneum] gb|EFA06900.1| hypothetical protein TcasGA2_TC009847 [Tribolium castaneum]	2,00E-87
gALL_v3_c11052	3	6	-	-	-	-	-
gALL_v3_c11053	3	6	-	-	-	-	-
gALL_v3_c11054	5	5	-	-	ref|XP_001656408.1|	hypothetical protein AaeL_AAEL000459 [Aedes aegypti] gb|EAT48510.1| AAEL000459-PA [Aedes aegypti]	1,00E-110
gALL_v3_c11055	2	4	-	-	-	-	-
gALL_v3_c11057	1	4	-	-	-	-	-
gALL_v3_c11058	5	5	-	-	ref|XP_973785.2|	PREDICTED: similar to CG34351 CG34351-PC [Tribolium castaneum]	1,00E-106
gALL_v3_c11059	3	7	-	-	ref|XP_001360661.2|	GA13229 [Drosophila pseudoobscura pseudoobscura] gb|EAL25236.2| GA13229 [Drosophila pseudoobscura pseudoobscura]	2,00E-37
gALL_v3_c1106	5	6	gnl|CDD|192236	pfam09258, Glyco_transf_64,	ref|XP_001651176.1|	exostosin-2 [Aedes aegypti] gb|EAT42871.1| AAEL005626-PA [Aedes aegypti]	1,00E-147
gALL_v3_c11060	17	22	-	-	-	-	-
gALL_v3_c11061	11	8	gnl|CDD|178526	PLN02938, PLN02938, phosph	ref|XP_003704936.1|	PREDICTED: phosphatidylserine decarboxylase proenzyme-like [Megachile rotundata]	9,00E-84
gALL_v3_c11062	5	10	gnl|CDD|128538	smart00242, MYSc, Myosin. La	ref|XP_002425047.1|	myosin IA, putative [Pediculus humanus corporis] gb|EEB12309.1| myosin IA, putative [Pediculus humanus corporis]	0
gALL_v3_c11063	17	13	gnl|CDD|162472	TIGR01661, ELAV_HUD_SF, ELA	ref|XP_003706065.1|	PREDICTED: protein alan shepard-like [Megachile rotundata]	1,00E-160
gALL_v3_c11065	2	10	gnl|CDD|189810	pfam01030, Recep_L_domain,	gb|EGI67492.1|	Insulin-like receptor [Acromyrmex echinatior]	1,00E-120
gALL_v3_c11066	4	4	gnl|CDD|128336	smart00020, Tryp_SPc, Trypsir	gb|EFN75261.1|	Trypsin-1 [Harpegnathos saltator]	3,00E-15
gALL_v3_c11067	9	9	gnl|CDD|152439	pfam12004, DUF3498, Domair	ref|XP_006612095.1|	PREDICTED: probable Ras GTPase-activating protein-like [Apis dorsata]	4,00E-26
gALL_v3_c11068	4	2	-	-	-	-	-
gALL_v3_c1107	7	8	-	-	gb|ENN77961.1|	hypothetical protein YQE_05638, partial [Dendroctonus ponderosae]	1,00E-34
gALL_v3_c11070	4	10	-	-	-	-	-
gALL_v3_c11071	1	8	-	-	-	-	-
gALL_v3_c11072	5	2	-	-	-	-	-
gALL_v3_c11074	4	6	gnl|CDD|190219	pfam02135, zf-TAZ, TAZ zinc fi	ref|XP_001606830.2|	PREDICTED: hypothetical protein LOC100123220 [Nasonia vitripennis]	3,00E-50
gALL_v3_c11075	2	10	-	-	ref|XP_002425038.1|	protein crooked neck, putative [Pediculus humanus corporis] gb|EEB12300.1| protein crooked neck, putative [Pediculus humanus corporis]	1,00E-115
gALL_v3_c11076	4	9	gnl|CDD|144704	pfam01204, Trehalase, Trehala	ref|XP_003696950.1|	PREDICTED: trehalase-like [Apis florea]	2,00E-62
gALL_v3_c11077	4	6	-	-	-	-	-
gALL_v3_c11078	3	8	gnl|CDD|162740	TIGR02169, SMC_prok_A, chro	gb|EEZ98889.1|	hypothetical protein TcasGA2_TC004504 [Tribolium castaneum]	4,00E-14
gALL_v3_c1108	6	5	-	-	gb|EGI68592.1|	Putative E3 ubiquitin-protein ligase HERC4 [Acromyrmex echinatior]	6,00E-67
gALL_v3_c11080	2	3	-	-	gb|EJW77603.1|	hypothetical protein WUBG_11491 [Wuchereria bancrofti]	4,00E-11
gALL_v3_c11081	9	14	-	-	gb|ADI61823.1|	endonuclease-reverse transcriptase [Bombyx mori]	2,00E-68
gALL_v3_c11082	1	1	-	-	-	-	-
gALL_v3_c11083	3	5	-	-	-	-	-
gALL_v3_c11084	5	2	-	-	-	-	-
gALL_v3_c11085	0	5	-	-	-	-	-
gALL_v3_c11087	3	2	gnl|CDD|31829	COG1643, HrpA, HrpA-like hel	gb|AEB61403.1|	putative ATP-dependent RNA helicase DHX36-like protein, partial [Equus caballus]	3,00E-76
gALL_v3_c11088	3	8	-	-	-	-	-
gALL_v3_c11089	5	11	gnl|CDD|162465	TIGR01652, ATPase-Plipid, pho	ref|XP_003485770.1|	PREDICTED: probable phospholipid-transporting ATPase IF-like [Bombus impatiens]	5,00E-48
gALL_v3_c1109	10	21	-	-	-	-	-
gALL_v3_c11090	5	4	-	-	-	-	-
gALL_v3_c11091	4	2	-	-	-	-	-
gALL_v3_c11093	10	6	-	-	-	-	-
gALL_v3_c11094	5	3	-	-	-	-	-
gALL_v3_c11097	5	6	gnl|CDD|145325	pfam02109, DAD, DAD family.	ref|XP_006624759.1|	PREDICTED: dolichyl-diphosphooligosaccharide--protein glycosyltransferase subunit DAD1-like [Apis dorsata]	2,00E-66
gALL_v3_c11098	10	8	-	-	-	-	-
gALL_v3_c111	8	11	-	-	-	-	-
gALL_v3_c1110	9	8	-	-	gb|EFA00556.1|	hypothetical protein TcasGA2_TC003423 [Tribolium castaneum]	2,00E-06
gALL_v3_c11100	2	4	-	-	-	-	-
gALL_v3_c11101	6	7	-	-	gb|EFN85210.1|	Double-strand-break repair protein rad21-like protein [Harpegnathos saltator]	1,00E-80
gALL_v3_c11102	7	9	gnl|CDD|189372	pfam00067, p450, Cytochrom	gb|AAR97606.1|	cytochrome P450 9E1 [Diploptera punctata]	7,00E-88
gALL_v3_c11103	6	3	-	-	ref|XP_001945461.1|	PREDICTED: hypothetical protein LOC100161307 [Acyrthosiphon pisum]	3,00E-10
gALL_v3_c11104	5	2	gnl|CDD|148254	pfam06535, RGM_N, Repulsiv	ref|XP_002428231.1|	conserved hypothetical protein [Pediculus humanus corporis] gb|EEB15493.1| conserved hypothetical protein [Pediculus humanus corporis]	3,00E-46
gALL_v3_c11106	5	7	-	-	-	-	-
gALL_v3_c11107	4	6	-	-	-	-	-
gALL_v3_c11109	5	6	gnl|CDD|109126	pfam00057, Ldl_recept_a, Low	ref|XP_005110646.1|	PREDICTED: basement membrane-specific heparan sulfate proteoglycan core protein-like [Aplysia californica]	5,00E-76
gALL_v3_c1111	17	16	gnl|CDD|31281	COG1084, COG1084, Predicte	gb|EFN64128.1|	Probable nucleolar GTP-binding protein 1 [Camponotus floridanus]	0
gALL_v3_c11110	2	5	-	-	ref|XP_002430824.1|	Ral guanine nucleotide exchange factor, putative [Pediculus humanus corporis] gb|EEB18086.1| Ral guanine nucleotide exchange factor, putative [Pediculus humanus corp	5,00E-07
gALL_v3_c11112	4	2	-	-	ref|XP_002430547.1|	conserved hypothetical protein [Pediculus humanus corporis] gb|EEB17809.1| conserved hypothetical protein [Pediculus humanus corporis]	5,00E-37
gALL_v3_c11113	5	3	gnl|CDD|191023	pfam04547, Anoctamin, Calciu	ref|XP_969407.1|	PREDICTED: similar to Abnormal X segregation, putative [Tribolium castaneum]	9,00E-80
gALL_v3_c11114	3	5	-	-	-	-	-
gALL_v3_c11115	3	3	-	-	-	-	-
gALL_v3_c11116	1	6	-	-	-	-	-
gALL_v3_c11117	6	7	gnl|CDD|129972	TIGR00894, 2A0114euk, Na(+)	gb|EFN74774.1|	Putative inorganic phosphate cotransporter [Camponotus floridanus]	1,00E-131
gALL_v3_c11118	0	6	-	-	ref|XP_001845387.1|	cell cycle checkpoint protein rad17 [Culex quinquefasciatus] gb|EDS40228.1| cell cycle checkpoint protein rad17 [Culex quinquefasciatus]	4,00E-10
gALL_v3_c11119	2	5	-	-	-	-	-
gALL_v3_c1112	7	11	-	-	-	-	-
gALL_v3_c11120	0	5	-	-	-	-	-
gALL_v3_c11121	3	6	gnl|CDD|152459	pfam12024, DUF3512, Domair	ref|XP_002427310.1|	Bromodomain-containing protein, putative [Pediculus humanus corporis] gb|EEB14572.1| Bromodomain-containing protein, putative [Pediculus humanus corporis]	8,00E-35
gALL_v3_c11122	11	6	-	-	-	-	-
gALL_v3_c11125	8	6	-	-	-	-	-
gALL_v3_c11126	5	5	-	-	-	-	-
gALL_v3_c11127	3	2	-	-	gb|EFZ13189.1|	hypothetical protein SINV_02571 [Solenopsis invicta]	9,00E-08
gALL_v3_c11128	7	14	-	-	ref|XP_006138987.1|	PREDICTED: tigger transposable element-derived protein 1-like [Pelodiscus sinensis]	2,00E-10
gALL_v3_c11129	6	4	-	-	-	-	-
gALL_v3_c11130	1	6	-	-	-	-	-
gALL_v3_c11131	7	12	-	-	-	-	-
gALL_v3_c11132	6	9	-	-	-	-	-
gALL_v3_c11133	0	6	-	-	-	-	-
gALL_v3_c11134	3	1	-	-	-	-	-
gALL_v3_c11135	9	14	-	-	-	-	-
gALL_v3_c11136	3	9	gnl|CDD|189452	pfam00211, Guanylate_cyc, A	gb|EFA10712.1|	rutabaga [Tribolium castaneum]	8,00E-98
gALL_v3_c11138	6	3	-	-	-	-	-
gALL_v3_c11139	1	6	-	-	-	-	-
gALL_v3_c11140	2	4	-	-	-	-	-
gALL_v3_c11141	6	3	-	-	gb|EFN68890.1|	E3 ubiquitin-protein ligase MARCH5 [Camponotus floridanus]	1,00E-32
gALL_v3_c11142	3	3	-	-	-	-	-
gALL_v3_c11143	7	15	-	-	-	-	-
gALL_v3_c11144	4	4	-	-	ref|XP_006561397.1|	PREDICTED: dentin sialophosphoprotein-like isoform X6 [Apis mellifera]	6,00E-31
gALL_v3_c11145	5	8	gnl|CDD|190690	pfam03619, DUF300, Domain	gb|EFN77390.1|	Transmembrane protein 184C [Harpegnathos saltator]	1,00E-113
gALL_v3_c11146	4	5	-	-	ref|XP_975086.2|	PREDICTED: similar to rab3-GEF CG5627-PB [Tribolium castaneum]	2,00E-35
gALL_v3_c11147	2	8	-	-	-	-	-
gALL_v3_c1115	21	15	-	-	-	-	-
gALL_v3_c11150	1	5	-	-	-	-	-
gALL_v3_c11151	7	6	gnl|CDD|190293	pfam02373, JmjC, JmjC domai	ref|XP_002431082.1|	JmjC domain-containing histone demethylation protein 2B, putative [Pediculus humanus corporis] gb|EEB18344.1| JmjC domain-containing histone demethylation protein	1,00E-152
gALL_v3_c11152	3	6	-	-	-	-	-
gALL_v3_c11153	19	30	-	-	dbj|BAN20872.1|	unkown protein [Riptortus pedestris]	1,00E-28
gALL_v3_c11154	4	2	gnl|CDD|192374	pfam09770, PAT1, Topoisomer	-	-	-
gALL_v3_c11155	4	8	-	-	-	-	-
gALL_v3_c11156	2	3	gnl|CDD|34791	COG5192, BMS1, GTP-binding	ref|XP_003398141.1|	PREDICTED: hypothetical protein LOC100649776 [Bombus terrestris]	1,00E-87
gALL_v3_c11157	11	6	-	-	-	-	-

gALL_v3_c11158	16	29	-	-	gb|EFN74736.1|	PiggyBac transposable element-derived protein 4 [Camponotus floridanus]	3,00E-51
gALL_v3_c1116	25	28	-	-	ref|XP_001861867.1|	tetratricopeptide repeat domain 21B [Culex quinquefasciatus] gb|EDS36206.1| tetratricopeptide repeat domain 21B [Culex quinquefasciatus]	0
gALL_v3_c11160	2	4	-	-	-	-	-
gALL_v3_c11161	2	1	-	-	-	-	-
gALL_v3_c11162	2	3	gnl|CDD|188981	cd09582, SAM_Scm-like-3MBT	ref|XP_003487907.1|	PREDICTED: lethal(3)malignant brain tumor-like protein 3-like [Bombus impatiens]	1,00E-28
gALL_v3_c11163	9	6	-	-	gb|EFN80576.1|	Putative uncharacterized transposon-derived protein F52C9.6 [Harpegnathos saltator]	1,00E-17
gALL_v3_c11164	9	6	-	-	-	-	-
gALL_v3_c11165	3	4	-	-	-	-	-
gALL_v3_c11166	3	6	-	-	gb|EGI58322.1|	Enhancer of polycomb-like protein 1 [Acromyrmex echinatior]	2,00E-11
gALL_v3_c11167	5	4	-	-	-	-	-
gALL_v3_c11168	5	5	-	-	-	-	-
gALL_v3_c11169	12	23	gnl|CDD|189008	cd09601, M1_APN_2, Peptidas	gb|EFZ09701.1|	hypothetical protein SINV_80061 [Solenopsis invicta]	1,00E-140
gALL_v3_c1117	8	6	-	-	gb|AAZ03545.1|	chitin synthase A [Spodoptera exigua]	8,00E-78
gALL_v3_c11170	8	10	gnl|CDD|128538	smart00242, MYSc, Myosin. La	gb|ETN65622.1|	myosin-IB [Anopheles darlingi]	1,00E-117
gALL_v3_c11171	4	3	-	-	ref|XP_002425759.1|	zinc finger protein CCCH domain-containing protein, putative [Pediculus humanus corporis] gb|EEB13021.1| zinc finger protein CCCH domain-containing protein, putative [	1,00E-30
gALL_v3_c11173	11	9	gnl|CDD|144462	pfam00876, Innexin, Innexin.	gb|ACN41955.1|	innexin 3 [Schistocerca gregaria]	1,00E-65
gALL_v3_c11175	2	4	-	-	-	-	-
gALL_v3_c11176	5	3	-	-	gb|EFX85681.1|	hypothetical protein DAPPUDRAFT_313764 [Daphnia pulex]	3,00E-07
gALL_v3_c11177	0	6	-	-	-	-	-
gALL_v3_c11178	2	8	-	-	-	-	-
gALL_v3_c11179	8	4	gnl|CDD|189054	cd09884, PIN_Smg5, PIN doma	ref|XP_002426268.1|	protein SMG5, putative [Pediculus humanus corporis] gb|EEB13530.1| protein SMG5, putative [Pediculus humanus corporis]	3,00E-64
gALL_v3_c1118	17	22	gnl|CDD|34781	COG5182, CUS1, Splicing facto	ref|XP_002426426.1|	Splicing factor 3B subunit, putative [Pediculus humanus corporis] gb|EEB13688.1| Splicing factor 3B subunit, putative [Pediculus humanus corporis]	1,00E-180
gALL_v3_c11181	5	4	-	-	gb|EHJ73785.1|	putative helicase MOV-10 [Danaus plexippus]	3,00E-07
gALL_v3_c11182	8	9	gnl|CDD|111866	pfam03022, MRJP, Major roya	gb|ACY71064.1|	yellow-1 [Tribolium castaneum]	6,00E-18
gALL_v3_c11183	9	18	-	-	ref|XP_003243437.1|	PREDICTED: hypothetical protein LOC100572891 [Acyrthosiphon pisum]	1,00E-09
gALL_v3_c11184	4	3	-	-	-	-	-
gALL_v3_c11185	5	7	gnl|CDD|147754	pfam05773, RWD, RWD doma	ref|XP_003705016.1|	PREDICTED: RWD domain-containing protein 4-like [Megachile rotundata]	4,00E-54
gALL_v3_c11186	2	6	-	-	-	-	-
gALL_v3_c11187	6	6	-	-	ref|XP_003758574.1|	PREDICTED: tigger transposable element-derived protein 1-like [Sarcophilus harrisii]	1,00E-06
gALL_v3_c11188	7	10	gnl|CDD|29232	cd01459, vWA_copine_like, V\	ref|XP_001942793.1|	PREDICTED: copine-5-like isoform 1 [Acyrthosiphon pisum]	1,00E-175
gALL_v3_c11189	2	3	-	-	gb|EFA13462.1|	hypothetical protein TcasGA2_TC005062 [Tribolium castaneum]	3,00E-21
gALL_v3_c1119	24	19	gnl|CDD|180529	PRK06327, PRK06327, dihydro	gb|AFP20531.1|	dihydrolipoamide dehydrogenase E3 subunit [Tribolium castaneum] gb|AGX84981.1| dihydrolipoamide dehydrogenase [Tribolium castaneum]	0
gALL_v3_c11190	5	4	-	-	emb|CAR94547.1|	troponin I [Blattella germanica]	7,00E-16
gALL_v3_c11191	3	2	-	-	ref|XP_001605368.1|	PREDICTED: metallophosphoesterase 1-like [Nasonia vitripennis]	2,00E-20
gALL_v3_c11192	3	8	-	-	-	-	-
gALL_v3_c11196	2	5	-	-	-	-	-
gALL_v3_c11197	4	4	-	-	-	-	-
gALL_v3_c11198	2	6	-	-	-	-	-
gALL_v3_c11199	4	3	-	-	-	-	-
gALL_v3_c1120	8	17	gnl|CDD|149160	pfam07926, TPR_MLP1_2, TPF	ref|XP_001664271.1|	hypothetical protein AaeL_AAEL003882 [Aedes aegypti] gb|EAT44784.1| AAEL003882-PA [Aedes aegypti]	5,00E-83
gALL_v3_c11200	10	13	-	-	-	-	-
gALL_v3_c11201	5	11	-	-	ref|XP_002432628.1|	Fasciclin-3 precursor, putative [Pediculus humanus corporis] gb|EEB19890.1| Fasciclin-3 precursor, putative [Pediculus humanus corporis]	2,00E-65
gALL_v3_c11202	4	6	-	-	-	-	-
gALL_v3_c11203	3	3	-	-	-	-	-
gALL_v3_c11204	4	3	gnl|CDD|147675	pfam05644, DUF800, Protein c	ref|XP_003398399.1|	PREDICTED: transport and Golgi organization protein 11-like isoform 2 [Bombus terrestris]	5,00E-64
gALL_v3_c11205	4	11	-	-	-	-	-
gALL_v3_c11207	10	12	-	-	-	-	-
gALL_v3_c11208	3	3	-	-	gb|ELT94599.1|	hypothetical protein CAPTEDRAFT_178563 [Capitella teleta]	3,00E-27
gALL_v3_c11209	3	3	-	-	-	-	-
gALL_v3_c1121	18	18	-	-	ref|XP_005096131.1|	PREDICTED: piggyBac transposable element-derived protein 3-like [Aplysia californica]	3,00E-63
gALL_v3_c11210	0	5	-	-	gb|EFA04021.1|	hypothetical protein TcasGA2_TC014249 [Tribolium castaneum]	7,00E-14
gALL_v3_c11211	5	4	-	-	-	-	-
gALL_v3_c11213	5	2	-	-	ref|XP_003100572.1|	hypothetical protein CRE_19767 [Caenorhabditis remanei] gb|EFP08781.1| hypothetical protein CRE_19767 [Caenorhabditis remanei]	1,00E-07
gALL_v3_c11215	4	6	gnl|CDD|188463	TIGR03948, butyr_acet_CoA, b	gb|AEE63401.1|	unknown [Dendroctonus ponderosae] gb|ENN70306.1| hypothetical protein YQE_12817, partial [Dendroctonus ponderosae] gb|ERL92766.1| hypothetical protein D910_1	4,00E-51
gALL_v3_c11216	3	1	-	-	gb|EFN72844.1|	Transcriptional repressor protein YY1 [Camponotus floridanus]	6,00E-94
gALL_v3_c11217	1	7	-	-	-	-	-
gALL_v3_c11218	3	7	-	-	-	-	-
gALL_v3_c11219	18	16	-	-	ref|XP_004206743.1|	PREDICTED: uncharacterized protein LOC101240849 [Hydra vulgaris]	9,00E-24
gALL_v3_c1122	3	9	-	-	-	-	-
gALL_v3_c11221	4	3	-	-	-	-	-
gALL_v3_c11222	7	17	-	-	gb|EON66515.1|	hypothetical protein W97_05760 [Coniosporium apollinis CBS 100218]	1,00E-09
gALL_v3_c11223	4	9	-	-	-	-	-
gALL_v3_c11224	3	2	-	-	ref|XP_003241232.1|	PREDICTED: multidrug resistance protein 3-like isoform 2 [Acyrthosiphon pisum]	8,00E-26
gALL_v3_c11225	3	2	gnl|CDD|29257	cd00200, WD40, WD40 domai	ref|XP_003487682.1|	PREDICTED: coatomer subunit alpha-like, partial [Bombus impatiens]	1,00E-123
gALL_v3_c11226	4	6	-	-	ref|XP_003486503.1|	PREDICTED: dual oxidase-like [Bombus impatiens]	3,00E-14
gALL_v3_c11227	1	4	gnl|CDD|191810	pfam07679, I-set, Immunoglol	gb|EFA00482.1|	hypothetical protein TcasGA2_TC003342 [Tribolium castaneum]	3,00E-29
gALL_v3_c11228	1	5	gnl|CDD|116392	pfam07778, Mis6, Mis6. Mis6	ref|XP_003485688.1|	PREDICTED: centromere protein I-like [Bombus impatiens]	3,00E-23
gALL_v3_c11230	8	9	-	-	-	-	-
gALL_v3_c11231	3	3	gnl|CDD|110072	pfam01044, Vinculin, Vinculin	gb|EFN68888.1|	Vinculin [Camponotus floridanus]	1,00E-80
gALL_v3_c11232	8	6	-	-	-	-	-
gALL_v3_c11233	1	4	-	-	-	-	-
gALL_v3_c11234	3	2	-	-	-	-	-
gALL_v3_c11235	3	6	-	-	-	-	-
gALL_v3_c11236	1	5	gnl|CDD|144804	pfam01342, SAND, SAND dom	ref|XP_003702992.1|	PREDICTED: deformed epidermal autoregulatory factor 1-like [Megachile rotundata]	1,00E-63
gALL_v3_c11237	2	6	-	-	ref|XP_002426350.1|	conserved hypothetical protein [Pediculus humanus corporis] gb|EEB13612.1| conserved hypothetical protein [Pediculus humanus corporis]	1,00E-48
gALL_v3_c11239	0	5	-	-	-	-	-
gALL_v3_c11240	4	8	-	-	ref|XP_001947845.2|	PREDICTED: hypothetical protein LOC100161677 [Acyrthosiphon pisum]	3,00E-66
gALL_v3_c11242	13	18	-	-	-	-	-
gALL_v3_c11243	3	5	-	-	-	-	-
gALL_v3_c11244	4	11	gnl|CDD|190535	pfam03134, TB2_DP1_HVA22,	ref|XP_006618357.1|	PREDICTED: receptor expression-enhancing protein 4-like isoform X5 [Apis dorsata]	8,00E-93
gALL_v3_c11245	3	9	-	-	-	-	-
gALL_v3_c11246	4	8	-	-	-	-	-
gALL_v3_c11247	4	5	-	-	-	-	-
gALL_v3_c11248	4	5	gnl|CDD|190266	pfam02270, TFIIF_beta, Transc	ref|XP_006620653.1|	PREDICTED: general transcription factor IIF subunit 2-like isoform X1 [Apis dorsata] ref|XP_006620654.1| PREDICTED: general transcription factor IIF subunit 2-like isoform	1,00E-97
gALL_v3_c11249	4	4	-	-	ref|XP_003705894.1|	PREDICTED: coiled-coil domain-containing protein 43-like [Megachile rotundata]	2,00E-27
gALL_v3_c1125	10	12	gnl|CDD|33700	COG3914, Spy, Predicted O-lin	ref|XP_006623355.1|	PREDICTED: UDP-N-acetylglucosamine--peptide N-acetylglucosaminyltransferase 110 kDa subunit-like isoform X2 [Apis dorsata]	0
gALL_v3_c11250	3	6	gnl|CDD|152056	pfam11620, GABP-alpha, GA-b	ref|XP_001599483.2|	PREDICTED: DNA-binding protein Ets97D-like [Nasonia vitripennis]	2,00E-20
gALL_v3_c11252	6	11	-	-	gb|EFA04804.1|	hypothetical protein TcasGA2_TC014853 [Tribolium castaneum]	7,00E-10
gALL_v3_c11253	3	2	-	-	-	-	-
gALL_v3_c11256	5	5	gnl|CDD|180029	PRK05342, clpX, ATP-depende	gb|EEZ98822.1|	hypothetical protein TcasGA2_TC004428 [Tribolium castaneum]	1,00E-130
gALL_v3_c11257	4	6	-	-	ref|XP_001869176.1|	conserved hypothetical protein [Culex quinquefasciatus] gb|EDS28561.1| conserved hypothetical protein [Culex quinquefasciatus]	2,00E-43
gALL_v3_c11258	4	9	gnl|CDD|128440	smart00135, LY, Low-density li	ref|XP_004529416.1|	PREDICTED: low-density lipoprotein receptor-related protein 2-like isoform X1 [Ceratitis capitata] ref|XP_004529417.1| PREDICTED: low-density lipoprotein receptor-relate	0
gALL_v3_c11259	0	4	-	-	-	-	-
gALL_v3_c1126	8	5	-	-	-	-	-
gALL_v3_c11260	5	3	-	-	-	-	-
gALL_v3_c11261	1	4	-	-	-	-	-
gALL_v3_c11262	5	4	gnl|CDD|33700	COG3914, Spy, Predicted O-lin	dbj|BAM18380.1|	super sex combs [Papilio xuthus]	1,00E-110
gALL_v3_c11264	6	3	gnl|CDD|189784	pfam00957, Synaptobrevin, Sy	gb|EFX67723.1|	hypothetical protein DAPPUDRAFT_218600 [Daphnia pulex]	5,00E-43
gALL_v3_c11265	9	8	-	-	gb|EFN73552.1|	Dynein intermediate chain, cytosolic [Camponotus floridanus]	6,00E-46
gALL_v3_c11266	4	2	gnl|CDD|149022	pfam07731, Cu-oxidase_2, Mu	gb|EFN64969.1|	Laccase-3 [Camponotus floridanus]	1,00E-56
gALL_v3_c11267	2	4	-	-	-	-	-
gALL_v3_c11268	2	4	-	-	-	-	-
gALL_v3_c11269	3	8	-	-	-	-	-
gALL_v3_c1127	7	6	gnl|CDD|178088	PLN02469, PLN02469, hydroxy	gb|ETE66616.1|	Hydroxyacylglutathione hydrolase, mitochondrial, partial [Ophiophagus hannah]	2,00E-60
gALL_v3_c11270	4	8	gnl|CDD|188096	TIGR00930, 2a30, K-Cl cotrans	ref|XP_003494405.1|	PREDICTED: bumetanide-sensitive sodium-(potassium)-chloride cotransporter-like [Bombus impatiens]	1,00E-156
gALL_v3_c11271	2	8	-	-	-	-	-
gALL_v3_c11272	7	5	-	-	ref|XP_003706913.1|	PREDICTED: rho GTPase-activating protein 28-like [Megachile rotundata]	1,00E-33
gALL_v3_c11273	1	6	-	-	-	-	-
gALL_v3_c11274	10	15	-	-	-	-	-
gALL_v3_c11276	2	3	-	-	-	-	-
gALL_v3_c11278	2	5	-	-	gb|EFN74962.1|	Zinc finger protein 252 [Camponotus floridanus]	2,00E-35
gALL_v3_c11279	4	7	-	-	-	-	-
gALL_v3_c11280	7	7	gnl|CDD|34495	COG4886, COG4886, Leucine-r	ref|XP_001816009.1|	PREDICTED: similar to AGAP002006-PA [Tribolium castaneum] gb|EFA02447.1| hypothetical protein TcasGA2_TC008134 [Tribolium castaneum]	3,00E-84
gALL_v3_c11281	3	6	-	-	gb|AAA92147.1|	reverse transcriptase [Bombyx mori]	2,00E-06
gALL_v3_c11282	2	8	-	-	-	-	-
gALL_v3_c11283	4	3	-	-	ref|XP_001814901.1|	PREDICTED: similar to Toutatis [Tribolium castaneum]	4,00E-33
gALL_v3_c11285	3	5	-	-	-	-	-
gALL_v3_c11286	4	6	gnl|CDD|192075	pfam08574, DUF1762, Protein	ref|XP_003707729.1|	PREDICTED: probable RNA polymerase II nuclear localization protein SLC7A6OS-like [Megachile rotundata]	8,00E-14
gALL_v3_c11288	3	6	-	-	ref|XP_002429721.1|	low-density lipoprotein receptor, putative [Pediculus humanus corporis] gb|EEB16983.1| low-density lipoprotein receptor, putative [Pediculus humanus corporis]	1,00E-100
gALL_v3_c11289	3	2	-	-	ref|XP_002431022.1|	conserved hypothetical protein [Pediculus humanus corporis] gb|EEB18284.1| conserved hypothetical protein [Pediculus humanus corporis]	2,00E-23
gALL_v3_c1129	11	21	gnl|CDD|31389	COG1196, Smc, Chromosome	ref|XP_006609710.1|	PREDICTED: protein outspread-like [Apis dorsata]	1,00E-116
gALL_v3_c11290	1	6	-	-	ref|XP_006560792.1|	PREDICTED: microtubule-associated protein futsch isoform X3 [Apis mellifera]	9,00E-30
gALL_v3_c11291	1	4	-	-	ref|XP_003427952.1|	PREDICTED: hypothetical protein LOC100677910 [Nasonia vitripennis]	3,00E-09
gALL_v3_c11292	1	6	-	-	-	-	-
gALL_v3_c11293	4	7	gnl|CDD|128508	smart00212, UBCc, Ubiquitin-c	ref|XP_393411.1|	PREDICTED: ubiquitin-conjugating enzyme E2 variant 2-like isoform 1 [Apis mellifera] ref|XP_003398336.1| PREDICTED: ubiquitin-conjugating enzyme E2 variant 2-like [Bo	1,00E-83
gALL_v3_c11294	5	5	-	-	-	-	-
gALL_v3_c11295	3	9	-	-	dbj|BAN21307.1|	putative inhibitor of apoptosis 1 [Riptortus pedestris]	1,00E-46
gALL_v3_c11297	3	3	gnl|CDD|178687	PLN03142, PLN03142, Probabl	ref|XP_002587792.1|	hypothetical protein BRAFLDRAFT_115396 [Branchiostoma floridae] gb|EEN43803.1| hypothetical protein BRAFLDRAFT_115396 [Branchiostoma floridae]	3,00E-65
gALL_v3_c11298	5	9	gnl|CDD|28922	cd00041, CUB, CUB domain; e	gb|EFX67485.1|	hypothetical protein DAPPUDRAFT_331029 [Daphnia pulex]	2,00E-46
gALL_v3_c11299	2	5	-	-	-	-	-
gALL_v3_c113	7	8	gnl|CDD|112027	pfam03194, LUC7, LUC7 N_ter	gb|EGI58200.1|	Cisplatin resistance-associated overexpressed protein [Acromyrmex echinatior]	1,00E-108
gALL_v3_c1130	9	15	-	-	-	-	-
gALL_v3_c11300	5	6	gnl|CDD|116147	pfam07528, DZF, DZF domain.	gb|ADF87944.1|	interleukin enhancer binding factor [Eriocheir sinensis]	0
gALL_v3_c11301	4	7	-	-	-	-	-
gALL_v3_c11302	7	6	-	-	-	-	-
gALL_v3_c11303	3	4	gnl|CDD|128725	smart00449, SPRY, Domain in S	ref|XP_001606114.1|	PREDICTED: ATP-dependent RNA helicase Ddx1-like [Nasonia vitripennis]	4,00E-99
gALL_v3_c11305	5	7	-	-	-	-	-
gALL_v3_c11306	8	6	gnl|CDD|193086	pfam12580, TPPII, Tripeptidyl	gb|EFN81782.1|	Tripeptidyl-peptidase 2 [Harpegnathos saltator]	1,00E-131
gALL_v3_c11307	2	3	-	-	-	-	-
gALL_v3_c11309	5	8	gnl|CDD|191886	pfam07896, DUF1674, Protein	ref|XP_001846499.1|	conserved hypothetical protein [Culex quinquefasciatus] gb|EDS43791.1| conserved hypothetical protein [Culex quinquefasciatus]	1,00E-33
gALL_v3_c1131	15	24	-	-	-	-	-
gALL_v3_c11310	3	4	-	-	ref|XP_006612176.1|	PREDICTED: peptidyl-prolyl cis-trans isomerase G-like isoform X4 [Apis dorsata] ref|XP_006612177.1| PREDICTED: peptidyl-prolyl cis-trans isomerase G-like isoform X5 [Api	1,00E-28
gALL_v3_c11314	4	4	gnl|CDD|72943	cd04658, Piwi_piwi-like_Euk, F	gb|AFW04603.1|	giwi protein [Gampsocleis gratiosa]	1,00E-121
gALL_v3_c11315	3	6	-	-	-	-	-

<table>
<tr><td>gALL_v3_c11316</td><td>4</td><td>4</td><td>-</td><td>-</td><td>ref|XP_003698587.1|</td><td>PREDICTED: uncharacterized protein LOC100869607 [Apis florea]</td><td>5,00E-54</td></tr>
<tr><td>gALL_v3_c11317</td><td>1</td><td>3</td><td>-</td><td>-</td><td>-</td><td>-</td><td>-</td></tr>
<tr><td>gALL_v3_c11319</td><td>7</td><td>5</td><td>-</td><td>-</td><td>-</td><td>-</td><td>-</td></tr>
<tr><td>gALL_v3_c1132</td><td>6</td><td>5</td><td>-</td><td>-</td><td>gb|AGM32905.1|</td><td>hypothetical protein [Coptotermes formosanus]</td><td>3,00E-06</td></tr>
<tr><td>gALL_v3_c11320</td><td>6</td><td>6</td><td>-</td><td>-</td><td>ref|XP_003426544.1|</td><td>PREDICTED: hypothetical protein LOC100679885 [Nasonia vitripennis]</td><td>8,00E-15</td></tr>
<tr><td>gALL_v3_c11321</td><td>3</td><td>3</td><td>gnl|CDD|193250</td><td>pfam12774, AAA_6, Hydrolytic</td><td>ref|XP_002427535.1|</td><td>dynein beta chain, ciliary, putative [Pediculus humanus corporis] gb|EEB14797.1| dynein beta chain, ciliary, putative [Pediculus humanus corporis]</td><td>1,00E-82</td></tr>
<tr><td>gALL_v3_c11322</td><td>3</td><td>4</td><td>gnl|CDD|173805</td><td>cd07479, Peptidases_S8_SKI-1</td><td>ref|XP_002428413.1|</td><td>membrane-bound transcription factor site 1 protease precursor, putative [Pediculus humanus corporis] gb|EEB15675.1| membrane-bound transcription factor site 1 prote</td><td>1,00E-126</td></tr>
<tr><td>gALL_v3_c11323</td><td>3</td><td>2</td><td>-</td><td>-</td><td>-</td><td>-</td><td>-</td></tr>
<tr><td>gALL_v3_c11324</td><td>2</td><td>3</td><td>-</td><td>-</td><td>-</td><td>-</td><td>-</td></tr>
<tr><td>gALL_v3_c11325</td><td>1</td><td>6</td><td>-</td><td>-</td><td>ref|XP_006812407.1|</td><td>PREDICTED: NFX1-type zinc finger-containing protein 1-like [Saccoglossus kowalevskii]</td><td>2,00E-38</td></tr>
<tr><td>gALL_v3_c11326</td><td>4</td><td>8</td><td>-</td><td>-</td><td>ref|XP_002425455.1|</td><td>hypothetical protein Phum_PHUM202100 [Pediculus humanus corporis] gb|EEB12717.1| hypothetical protein Phum_PHUM202100 [Pediculus humanus corporis]</td><td>1,00E-48</td></tr>
<tr><td>gALL_v3_c11327</td><td>3</td><td>2</td><td>-</td><td>-</td><td>-</td><td>-</td><td>-</td></tr>
<tr><td>gALL_v3_c11328</td><td>5</td><td>7</td><td>gnl|CDD|29331</td><td>cd00256, VATPase_H, VATPase</td><td>ref|XP_966693.2|</td><td>PREDICTED: similar to vacuolar ATP synthase subunit h isoform 1 [Tribolium castaneum]</td><td>1,00E-109</td></tr>
<tr><td>gALL_v3_c11329</td><td>2</td><td>5</td><td>-</td><td>-</td><td>-</td><td>-</td><td>-</td></tr>
<tr><td>gALL_v3_c1133</td><td>6</td><td>8</td><td>-</td><td>-</td><td>-</td><td>-</td><td>-</td></tr>
<tr><td>gALL_v3_c11331</td><td>2</td><td>9</td><td>gnl|CDD|152835</td><td>pfam12400, DUF3661, Vaculol</td><td>ref|XP_975528.1|</td><td>PREDICTED: similar to transmembrane protein 110 [Tribolium castaneum] gb|EFA05999.1| hypothetical protein TcasGA2_TC008825 [Tribolium castaneum]</td><td>9,00E-92</td></tr>
<tr><td>gALL_v3_c11332</td><td>5</td><td>2</td><td>gnl|CDD|110156</td><td>pfam01133, ER, Enhancer of ru</td><td>gb|ADD24363.1|</td><td>Enhancer of rudimentary homolog [Lepeophtheirus salmonis]</td><td>6,00E-48</td></tr>
<tr><td>gALL_v3_c11333</td><td>4</td><td>6</td><td>-</td><td>-</td><td>-</td><td>-</td><td>-</td></tr>
<tr><td>gALL_v3_c11334</td><td>0</td><td>6</td><td>-</td><td>-</td><td>-</td><td>-</td><td>-</td></tr>
<tr><td>gALL_v3_c11335</td><td>7</td><td>10</td><td>-</td><td>-</td><td>-</td><td>-</td><td>-</td></tr>
<tr><td>gALL_v3_c11336</td><td>16</td><td>13</td><td>-</td><td>-</td><td>-</td><td>-</td><td>-</td></tr>
<tr><td>gALL_v3_c11338</td><td>3</td><td>10</td><td>-</td><td>-</td><td>-</td><td>-</td><td>-</td></tr>
<tr><td>gALL_v3_c11339</td><td>3</td><td>7</td><td>gnl|CDD|73065</td><td>cd02659, peptidase_C19C, A s</td><td>ref|XP_002607840.1|</td><td>hypothetical protein BRAFLDRAFT_199699 [Branchiostoma floridae] gb|EEN63850.1| hypothetical protein BRAFLDRAFT_199699 [Branchiostoma floridae]</td><td>6,00E-35</td></tr>
<tr><td>gALL_v3_c1134</td><td>17</td><td>29</td><td>-</td><td>-</td><td>gb|AHH85823.1|</td><td>short neuropeptide F precursor [Schistocerca gregaria]</td><td>1,00E-18</td></tr>
<tr><td>gALL_v3_c11340</td><td>3</td><td>4</td><td>-</td><td>-</td><td>gb|EFX65940.1|</td><td>hypothetical protein DAPPUDRAFT_332702 [Daphnia pulex]</td><td>2,00E-14</td></tr>
<tr><td>gALL_v3_c11341</td><td>6</td><td>7</td><td>gnl|CDD|150314</td><td>pfam09606, Med15, ARC105 c</td><td>-</td><td>-</td><td>-</td></tr>
<tr><td>gALL_v3_c11342</td><td>3</td><td>2</td><td>-</td><td>-</td><td>-</td><td>-</td><td>-</td></tr>
<tr><td>gALL_v3_c11343</td><td>1</td><td>6</td><td>-</td><td>-</td><td>ref|XP_001607967.2|</td><td>PREDICTED: muskelin-like [Nasonia vitripennis]</td><td>1,00E-155</td></tr>
<tr><td>gALL_v3_c11344</td><td>4</td><td>7</td><td>gnl|CDD|173730</td><td>cd06624, STKc_ASK, Catalytic</td><td>gb|EGI67863.1|</td><td>Mitogen-activated protein kinase kinase kinase 15 [Acromyrmex echinatior]</td><td>1,00E-155</td></tr>
<tr><td>gALL_v3_c11345</td><td>3</td><td>2</td><td>-</td><td>-</td><td>ref|XP_002612677.1|</td><td>hypothetical protein BRAFLDRAFT_249711 [Branchiostoma floridae] gb|EEN68686.1| hypothetical protein BRAFLDRAFT_249711 [Branchiostoma floridae]</td><td>2,00E-29</td></tr>
<tr><td>gALL_v3_c11346</td><td>4</td><td>3</td><td>-</td><td>-</td><td>-</td><td>-</td><td>-</td></tr>
<tr><td>gALL_v3_c11347</td><td>5</td><td>5</td><td>gnl|CDD|188098</td><td>TIGR00957, MRP_assoc_pro, r</td><td>ref|XP_002424437.1|</td><td>multidrug resistance-associated protein, putative [Pediculus humanus corporis] gb|EEB11699.1| multidrug resistance-associated protein, putative [Pediculus humanus cor</td><td>1,00E-50</td></tr>
<tr><td>gALL_v3_c11348</td><td>12</td><td>7</td><td>-</td><td>-</td><td>-</td><td>-</td><td>-</td></tr>
<tr><td>gALL_v3_c11349</td><td>2</td><td>10</td><td>gnl|CDD|189693</td><td>pfam00735, Septin, Septin. M</td><td>ref|XP_003704985.1|</td><td>PREDICTED: septin-1-like [Megachile rotundata]</td><td>0</td></tr>
<tr><td>gALL_v3_c11350</td><td>2</td><td>5</td><td>-</td><td>-</td><td>-</td><td>-</td><td>-</td></tr>
<tr><td>gALL_v3_c11351</td><td>6</td><td>3</td><td>-</td><td>-</td><td>-</td><td>-</td><td>-</td></tr>
<tr><td>gALL_v3_c11352</td><td>4</td><td>3</td><td>-</td><td>-</td><td>-</td><td>-</td><td>-</td></tr>
<tr><td>gALL_v3_c11355</td><td>4</td><td>7</td><td>-</td><td>-</td><td>ref|XP_002429960.1|</td><td>conserved hypothetical protein [Pediculus humanus corporis] gb|EEB17222.1| conserved hypothetical protein [Pediculus humanus corporis]</td><td>3,00E-86</td></tr>
<tr><td>gALL_v3_c11356</td><td>1</td><td>7</td><td>-</td><td>-</td><td>-</td><td>-</td><td>-</td></tr>
<tr><td>gALL_v3_c11357</td><td>5</td><td>8</td><td>-</td><td>-</td><td>-</td><td>-</td><td>-</td></tr>
<tr><td>gALL_v3_c11359</td><td>3</td><td>5</td><td>gnl|CDD|128788</td><td>smart00512, Skp1, Found in Sl</td><td>gb|ENN72054.1|</td><td>hypothetical protein YQE_11342, partial [Dendroctonus ponderosae] gb|ERL91381.1| hypothetical protein D910_08713 [Dendroctonus ponderosae]</td><td>2,00E-72</td></tr>
<tr><td>gALL_v3_c1136</td><td>10</td><td>12</td><td>-</td><td>-</td><td>-</td><td>-</td><td>-</td></tr>
<tr><td>gALL_v3_c11360</td><td>3</td><td>8</td><td>-</td><td>-</td><td>-</td><td>-</td><td>-</td></tr>
<tr><td>gALL_v3_c11362</td><td>3</td><td>4</td><td>-</td><td>-</td><td>ref|XP_975305.1|</td><td>PREDICTED: similar to Kielin [Tribolium castaneum]</td><td>4,00E-07</td></tr>
<tr><td>gALL_v3_c11363</td><td>4</td><td>9</td><td>gnl|CDD|161744</td><td>TIGR00175, mito_nad_idh, iso</td><td>gb|AGM32732.1|</td><td>isocitrate dehydrogenase, subunit beta, partial [Coptotermes formosanus]</td><td>1,00E-116</td></tr>
<tr><td>gALL_v3_c11364</td><td>3</td><td>3</td><td>-</td><td>-</td><td>ref|XP_002431002.1|</td><td>low-density lipoprotein receptor, putative [Pediculus humanus corporis] gb|EEB18264.1| low-density lipoprotein receptor, putative [Pediculus humanus corporis]</td><td>3,00E-99</td></tr>
<tr><td>gALL_v3_c11365</td><td>3</td><td>3</td><td>-</td><td>-</td><td>-</td><td>-</td><td>-</td></tr>
<tr><td>gALL_v3_c11366</td><td>2</td><td>4</td><td>-</td><td>-</td><td>-</td><td>-</td><td>-</td></tr>
<tr><td>gALL_v3_c11368</td><td>4</td><td>2</td><td>-</td><td>-</td><td>ref|XP_005189294.1|</td><td>PREDICTED: gypsy retrotransposon integrase-like protein 1-like [Musca domestica]</td><td>8,00E-17</td></tr>
<tr><td>gALL_v3_c11369</td><td>5</td><td>6</td><td>-</td><td>-</td><td>-</td><td>-</td><td>-</td></tr>
<tr><td>gALL_v3_c1137</td><td>6</td><td>9</td><td>-</td><td>-</td><td>-</td><td>-</td><td>-</td></tr>
<tr><td>gALL_v3_c11370</td><td>10</td><td>12</td><td>-</td><td>-</td><td>-</td><td>-</td><td>-</td></tr>
<tr><td>gALL_v3_c11371</td><td>2</td><td>5</td><td>-</td><td>-</td><td>-</td><td>-</td><td>-</td></tr>
<tr><td>gALL_v3_c11372</td><td>2</td><td>5</td><td>gnl|CDD|192054</td><td>pfam08519, RFC1, Replication</td><td>gb|EFX80623.1|</td><td>hypothetical protein DAPPUDRAFT_318220 [Daphnia pulex]</td><td>4,00E-94</td></tr>
<tr><td>gALL_v3_c11373</td><td>4</td><td>11</td><td>-</td><td>-</td><td>-</td><td>-</td><td>-</td></tr>
<tr><td>gALL_v3_c11374</td><td>14</td><td>9</td><td>gnl|CDD|189605</td><td>pfam00566, TBC, TBC domain.</td><td>ref|XP_001601621.1|</td><td>PREDICTED: TBC1 domain family member 10B-like [Nasonia vitripennis]</td><td>3,00E-98</td></tr>
<tr><td>gALL_v3_c11375</td><td>4</td><td>8</td><td>-</td><td>-</td><td>gb|EFN74771.1|</td><td>hypothetical protein EAG_10179 [Camponotus floridanus]</td><td>1,00E-17</td></tr>
<tr><td>gALL_v3_c11376</td><td>4</td><td>3</td><td>gnl|CDD|176278</td><td>cd01202, FRS2, Fibroblast grov</td><td>ref|XP_003705314.1|</td><td>PREDICTED: uncharacterized protein LOC100877025 [Megachile rotundata]</td><td>6,00E-54</td></tr>
<tr><td>gALL_v3_c11377</td><td>6</td><td>5</td><td>gnl|CDD|189417</td><td>pfam00144, Beta-lactamase, E</td><td>ref|XP_002590141.1|</td><td>hypothetical protein BRAFLDRAFT_126051 [Branchiostoma floridae] gb|EEN46152.1| hypothetical protein BRAFLDRAFT_126051 [Branchiostoma floridae]</td><td>3,00E-61</td></tr>
<tr><td>gALL_v3_c11378</td><td>4</td><td>6</td><td>-</td><td>-</td><td>-</td><td>-</td><td>-</td></tr>
<tr><td>gALL_v3_c11379</td><td>2</td><td>5</td><td>gnl|CDD|128374</td><td>smart00060, FN3, Fibronectin</td><td>ref|XP_002423320.1|</td><td>conserved hypothetical protein [Pediculus humanus corporis] gb|EEB10582.1| conserved hypothetical protein [Pediculus humanus corporis]</td><td>7,00E-58</td></tr>
<tr><td>gALL_v3_c1138</td><td>14</td><td>13</td><td>-</td><td>-</td><td>ref|NP_001164113.1|</td><td>cap-n-collar [Tribolium castaneum]</td><td>3,00E-97</td></tr>
<tr><td>gALL_v3_c11380</td><td>7</td><td>9</td><td>gnl|CDD|173846</td><td>cd01157, MCAD, Medium chai</td><td>dbj|BAH72322.1|</td><td>ACYPI001113 [Acyrthosiphon pisum]</td><td>2,00E-07</td></tr>
<tr><td>gALL_v3_c11381</td><td>3</td><td>5</td><td>gnl|CDD|177804</td><td>PLN00410, PLN00410, U5 snRI</td><td>gb|AFZ78683.1|</td><td>thioredoxin-like protein [Coptotermes formosanus]</td><td>1,00E-39</td></tr>
<tr><td>gALL_v3_c11382</td><td>7</td><td>13</td><td>-</td><td>-</td><td>-</td><td>-</td><td>-</td></tr>
<tr><td>gALL_v3_c11383</td><td>2</td><td>3</td><td>-</td><td>-</td><td>ref|XP_002422908.1|</td><td>conserved hypothetical protein [Pediculus humanus corporis] gb|EEB10170.1| conserved hypothetical protein [Pediculus humanus corporis]</td><td>2,00E-07</td></tr>
<tr><td>gALL_v3_c11384</td><td>5</td><td>6</td><td>-</td><td>-</td><td>-</td><td>-</td><td>-</td></tr>
<tr><td>gALL_v3_c11385</td><td>3</td><td>7</td><td>gnl|CDD|147706</td><td>pfam05699, Dimer_Tnp_hAT, I</td><td>ref|XP_003241729.1|</td><td>PREDICTED: zinc finger BED domain-containing protein 1-like [Acyrthosiphon pisum]</td><td>5,00E-30</td></tr>
<tr><td>gALL_v3_c11386</td><td>1</td><td>5</td><td>-</td><td>-</td><td>-</td><td>-</td><td>-</td></tr>
<tr><td>gALL_v3_c11387</td><td>2</td><td>6</td><td>-</td><td>-</td><td>-</td><td>-</td><td>-</td></tr>
<tr><td>gALL_v3_c11389</td><td>2</td><td>9</td><td>-</td><td>-</td><td>-</td><td>-</td><td>-</td></tr>
<tr><td>gALL_v3_c1139</td><td>26</td><td>28</td><td>gnl|CDD|34777</td><td>COG5178, PRP8, U5 snRNP spl</td><td>ref|XP_003703080.1|</td><td>PREDICTED: pre-mRNA-processing-splicing factor 8-like [Megachile rotundata]</td><td>0</td></tr>
<tr><td>gALL_v3_c11390</td><td>4</td><td>5</td><td>-</td><td>-</td><td>-</td><td>-</td><td>-</td></tr>
<tr><td>gALL_v3_c11391</td><td>8</td><td>6</td><td>gnl|CDD|29805</td><td>cd00707, Pancreat_lipase_like</td><td>ref|XP_003244050.1|</td><td>PREDICTED: pancreatic lipase-related protein 1-like isoform 2 [Acyrthosiphon pisum]</td><td>4,00E-39</td></tr>
<tr><td>gALL_v3_c11392</td><td>2</td><td>10</td><td>-</td><td>-</td><td>ref|XP_001355095.1|</td><td>GA17538 [Drosophila pseudoobscura pseudoobscura] gb|EAL32151.1| GA17538 [Drosophila pseudoobscura pseudoobscura]</td><td>2,00E-74</td></tr>
<tr><td>gALL_v3_c11393</td><td>4</td><td>4</td><td>-</td><td>-</td><td>-</td><td>-</td><td>-</td></tr>
<tr><td>gALL_v3_c11395</td><td>4</td><td>2</td><td>-</td><td>-</td><td>-</td><td>-</td><td>-</td></tr>
<tr><td>gALL_v3_c11397</td><td>1</td><td>4</td><td>-</td><td>-</td><td>gb|EFA09485.1|</td><td>hypothetical protein TcasGA2_TC010979 [Tribolium castaneum]</td><td>8,00E-20</td></tr>
<tr><td>gALL_v3_c11398</td><td>7</td><td>18</td><td>-</td><td>-</td><td>gb|EFN88774.1|</td><td>Protein disabled [Harpegnathos saltator]</td><td>2,00E-13</td></tr>
<tr><td>gALL_v3_c11399</td><td>2</td><td>4</td><td>gnl|CDD|128500</td><td>smart00204, TGFB, Transformi</td><td>ref|XP_973577.1|</td><td>PREDICTED: similar to Bone morphogenetic protein 10 precursor (BMP-10) [Tribolium castaneum] gb|EFA09252.1| bone morphogenetic protein 10 [Tribolium castaneum]</td><td>3,00E-34</td></tr>
<tr><td>gALL_v3_c114</td><td>6</td><td>14</td><td>gnl|CDD|133311</td><td>cd04111, Rab39, Rab39 subfar</td><td>gb|EFZ12055.1|</td><td>hypothetical protein SINV_03176 [Solenopsis invicta] gb|EGI59580.1| Ras-related protein Rab-39B [Acromyrmex echinatior]</td><td>1,00E-141</td></tr>
<tr><td>gALL_v3_c1140</td><td>14</td><td>17</td><td>gnl|CDD|192801</td><td>pfam11708, Slu7, Pre-mRNA s</td><td>ref|XP_002424468.1|</td><td>pre-mRNA-splicing factor slu7, putative [Pediculus humanus corporis] gb|EEB11730.1| pre-mRNA-splicing factor slu7, putative [Pediculus humanus corporis]</td><td>0</td></tr>
<tr><td>gALL_v3_c11400</td><td>3</td><td>2</td><td>-</td><td>-</td><td>ref|XP_001947222.2|</td><td>PREDICTED: tubulin-specific chaperone cofactor E-like protein-like isoform 1 [Acyrthosiphon pisum] ref|XP_003243104.1| PREDICTED: tubulin-specific chaperone cofactor</td><td>6,00E-18</td></tr>
<tr><td>gALL_v3_c11402</td><td>5</td><td>7</td><td>gnl|CDD|144080</td><td>pfam00350, Dynamin_N, Dyna</td><td>ref|XP_002432275.1|</td><td>transmembrane GTPase Marf, putative [Pediculus humanus corporis] gb|EEB19537.1| transmembrane GTPase Marf, putative [Pediculus humanus corporis]</td><td>1,00E-123</td></tr>
<tr><td>gALL_v3_c11403</td><td>1</td><td>5</td><td>-</td><td>-</td><td>-</td><td>-</td><td>-</td></tr>
<tr><td>gALL_v3_c11404</td><td>3</td><td>6</td><td>-</td><td>-</td><td>-</td><td>-</td><td>-</td></tr>
<tr><td>gALL_v3_c11405</td><td>3</td><td>4</td><td>-</td><td>-</td><td>-</td><td>-</td><td>-</td></tr>
<tr><td>gALL_v3_c11407</td><td>2</td><td>7</td><td>-</td><td>-</td><td>-</td><td>-</td><td>-</td></tr>
<tr><td>gALL_v3_c11408</td><td>11</td><td>16</td><td>-</td><td>-</td><td>gb|EFA09839.1|</td><td>hypothetical protein TcasGA2_TC011986 [Tribolium castaneum]</td><td>0</td></tr>
<tr><td>gALL_v3_c11409</td><td>6</td><td>9</td><td>-</td><td>-</td><td>-</td><td>-</td><td>-</td></tr>
<tr><td>gALL_v3_c1141</td><td>12</td><td>14</td><td>gnl|CDD|133004</td><td>cd02510, pp-GalNAc-T, pp-Gal</td><td>gb|ENN83105.1|</td><td>hypothetical protein YQE_00533, partial [Dendroctonus ponderosae] gb|ENN83514.1| hypothetical protein YQE_00128, partial [Dendroctonus ponderosae]</td><td>1,00E-129</td></tr>
<tr><td>gALL_v3_c11410</td><td>7</td><td>11</td><td>gnl|CDD|31429</td><td>COG1236, YSH1, Predicted exc</td><td>ref|XP_969343.1|</td><td>PREDICTED: similar to CG1972 CG1972-PA [Tribolium castaneum] gb|EFA06334.1| hypothetical protein TcasGA2_TC009205 [Tribolium castaneum]</td><td>0</td></tr>
<tr><td>gALL_v3_c11411</td><td>6</td><td>5</td><td>-</td><td>-</td><td>-</td><td>-</td><td>-</td></tr>
<tr><td>gALL_v3_c11413</td><td>3</td><td>4</td><td>gnl|CDD|130820</td><td>TIGR01759, MalateDH-SF1, ma</td><td>gb|AGM32509.1|</td><td>malate dehydrogenase, partial [Coptotermes formosanus]</td><td>1,00E-109</td></tr>
<tr><td>gALL_v3_c11414</td><td>4</td><td>3</td><td>-</td><td>-</td><td>ref|XP_966624.1|</td><td>PREDICTED: similar to U3 small nucleolar RNA-associated protein 18 homolog (WD repeat protein l(2)k07824) [Tribolium castaneum] gb|EEZ97885.1| hypothetical protein</td><td>4,00E-14</td></tr>
<tr><td>gALL_v3_c11416</td><td>4</td><td>6</td><td>gnl|CDD|147820</td><td>pfam05871, ESCRT-II, ESCRT-II</td><td>ref|XP_003700354.1|</td><td>PREDICTED: vacuolar protein-sorting-associated protein 25-like [Megachile rotundata]</td><td>1,00E-38</td></tr>
<tr><td>gALL_v3_c11419</td><td>3</td><td>2</td><td>-</td><td>-</td><td>-</td><td>-</td><td>-</td></tr>
<tr><td>gALL_v3_c1142</td><td>12</td><td>7</td><td>-</td><td>-</td><td>-</td><td>-</td><td>-</td></tr>
<tr><td>gALL_v3_c11420</td><td>9</td><td>5</td><td>-</td><td>-</td><td>-</td><td>-</td><td>-</td></tr>
<tr><td>gALL_v3_c11422</td><td>3</td><td>6</td><td>-</td><td>-</td><td>-</td><td>-</td><td>-</td></tr>
<tr><td>gALL_v3_c11423</td><td>5</td><td>3</td><td>-</td><td>-</td><td>gb|AGM32669.1|</td><td>hypothetical protein [Coptotermes formosanus]</td><td>2,00E-34</td></tr>
<tr><td>gALL_v3_c11425</td><td>2</td><td>6</td><td>-</td><td>-</td><td>gb|EGI61090.1|</td><td>Calcium-binding atopy-related autoantigen 1 [Acromyrmex echinatior]</td><td>6,00E-88</td></tr>
<tr><td>gALL_v3_c11426</td><td>2</td><td>3</td><td>-</td><td>-</td><td>-</td><td>-</td><td>-</td></tr>
<tr><td>gALL_v3_c11429</td><td>4</td><td>6</td><td>-</td><td>-</td><td>-</td><td>-</td><td>-</td></tr>
<tr><td>gALL_v3_c1143</td><td>6</td><td>4</td><td>-</td><td>-</td><td>-</td><td>-</td><td>-</td></tr>
<tr><td>gALL_v3_c11431</td><td>2</td><td>3</td><td>-</td><td>-</td><td>-</td><td>-</td><td>-</td></tr>
<tr><td>gALL_v3_c11433</td><td>6</td><td>6</td><td>gnl|CDD|143860</td><td>pfam00078, RVT_1, Reverse tr</td><td>ref|XP_004205527.1|</td><td>PREDICTED: RNA-directed DNA polymerase from mobile element jockey-like [Hydra vulgaris]</td><td>1,00E-22</td></tr>
<tr><td>gALL_v3_c11434</td><td>1</td><td>5</td><td>gnl|CDD|177973</td><td>PLN02339, PLN02339, NAD+ s</td><td>ref|XP_392994.4|</td><td>PREDICTED: probable glutamine-dependent NAD(+) synthetase-like isoformX1 [Apis mellifera] ref|XP_006559458.1| PREDICTED: probable glutamine-dependent NAD(+) sy</td><td>1,00E-100</td></tr>
<tr><td>gALL_v3_c11435</td><td>6</td><td>3</td><td>-</td><td>-</td><td>-</td><td>-</td><td>-</td></tr>
<tr><td>gALL_v3_c11436</td><td>5</td><td>4</td><td>gnl|CDD|178824</td><td>PRK00050, PRK00050, 16S rRN</td><td>ref|XP_971437.1|</td><td>PREDICTED: similar to s-adenosyl-methyl transferase mraw [Tribolium castaneum] gb|EFA07238.1| hypothetical protein TcasGA2_TC010595 [Tribolium castaneum]</td><td>7,00E-75</td></tr>
<tr><td>gALL_v3_c11437</td><td>3</td><td>2</td><td>-</td><td>-</td><td>-</td><td>-</td><td>-</td></tr>
<tr><td>gALL_v3_c11438</td><td>2</td><td>3</td><td>-</td><td>-</td><td>-</td><td>-</td><td>-</td></tr>
<tr><td>gALL_v3_c11439</td><td>10</td><td>11</td><td>-</td><td>-</td><td>-</td><td>-</td><td>-</td></tr>
<tr><td>gALL_v3_c11440</td><td>1</td><td>4</td><td>-</td><td>-</td><td>emb|CAX68236.1|</td><td>dicer-1 [Blattella germanica]</td><td>3,00E-72</td></tr>
<tr><td>gALL_v3_c11441</td><td>3</td><td>5</td><td>-</td><td>-</td><td>-</td><td>-</td><td>-</td></tr>
<tr><td>gALL_v3_c11442</td><td>2</td><td>4</td><td>-</td><td>-</td><td>-</td><td>-</td><td>-</td></tr>
<tr><td>gALL_v3_c11443</td><td>5</td><td>14</td><td>gnl|CDD|150812</td><td>pfam10190, Tmemb_170, Puta</td><td>gb|ELU14341.1|</td><td>hypothetical protein CAPTEDRAFT_106761 [Capitella teleta]</td><td>3,00E-39</td></tr>
<tr><td>gALL_v3_c11444</td><td>1</td><td>4</td><td>gnl|CDD|133338</td><td>cd04138, H_N_K_Ras_like, H-F</td><td>ref|XP_005182611.1|</td><td>PREDICTED: ras-like protein 1-like isoform X1 [Musca domestica] ref|XP_005182612.1| PREDICTED: ras-like protein 1-like isoform X2 [Musca domestica]</td><td>1,00E-105</td></tr>
<tr><td>gALL_v3_c11445</td><td>4</td><td>4</td><td>-</td><td>-</td><td>-</td><td>-</td><td>-</td></tr>
<tr><td>gALL_v3_c11446</td><td>1</td><td>6</td><td>-</td><td>-</td><td>-</td><td>-</td><td>-</td></tr>
<tr><td>gALL_v3_c11447</td><td>10</td><td>9</td><td>gnl|CDD|163240</td><td>TIGR03376, glycerol3P_DH, gly</td><td>gb|EFN89159.1|</td><td>Glycerol-3-phosphate dehydrogenase [NAD+], cytoplasmic [Harpegnathos saltator]</td><td>3,00E-66</td></tr>
<tr><td>gALL_v3_c11448</td><td>1</td><td>7</td><td>-</td><td>-</td><td>-</td><td>-</td><td>-</td></tr>
<tr><td>gALL_v3_c11449</td><td>2</td><td>8</td><td>-</td><td>-</td><td>ref|XP_002423984.1|</td><td>ubiquitin-protein ligase BRE1A, putative [Pediculus humanus corporis] gb|EEB11246.1| ubiquitin-protein ligase BRE1A, putative [Pediculus humanus corporis]</td><td>2,00E-50</td></tr>
<tr><td>gALL_v3_c1145</td><td>2</td><td>8</td><td>gnl|CDD|189509</td><td>pfam00338, Ribosomal_S10, R</td><td>ref|XP_005180767.1|</td><td>PREDICTED: 28S ribosomal protein S10, mitochondrial-like [Musca domestica]</td><td>3,00E-53</td></tr>
<tr><td>gALL_v3_c11450</td><td>4</td><td>5</td><td>-</td><td>-</td><td>gb|ENN81611.1|</td><td>hypothetical protein YQE_01975, partial [Dendroctonus ponderosae]</td><td>3,00E-77</td></tr>
<tr><td>gALL_v3_c11451</td><td>4</td><td>7</td><td>-</td><td>-</td><td>gb|AEM44817.1|</td><td>unknown [Pectinophora gossypiella]</td><td>3,00E-11</td></tr>
<tr><td>gALL_v3_c11452</td><td>5</td><td>8</td><td>-</td><td>-</td><td>ref|XP_001601379.1|</td><td>PREDICTED: NFX1-type zinc finger-containing protein 1-like isoform 1 [Nasonia vitripennis]</td><td>8,00E-11</td></tr>
<tr><td>gALL_v3_c11453</td><td>4</td><td>2</td><td>gnl|CDD|34668</td><td>COG5064, SRP1, Karyopherin (</td><td>ref|XP_003704950.1|</td><td>PREDICTED: importin subunit alpha-2-like [Megachile rotundata]</td><td>5,00E-98</td></tr>
<tr><td>gALL_v3_c11455</td><td>6</td><td>4</td><td>-</td><td>-</td><td>ref|XP_003244855.1|</td><td>PREDICTED: 52 kDa repressor of the inhibitor of the protein kinase-like [Acyrthosiphon pisum]</td><td>1,00E-27</td></tr>
<tr><td>gALL_v3_c11457</td><td>4</td><td>4</td><td>-</td><td>-</td><td>ref|XP_003700720.1|</td><td>PREDICTED: LOW QUALITY PROTEIN: uncharacterized protein KIAA1109-like [Megachile rotundata]</td><td>7,00E-56</td></tr>
<tr><td>gALL_v3_c11459</td><td>5</td><td>7</td><td>-</td><td>-</td><td>-</td><td>-</td><td>-</td></tr>
<tr><td>gALL_v3_c1146</td><td>5</td><td>10</td><td>-</td><td>-</td><td>-</td><td>-</td><td>-</td></tr>
<tr><td>gALL_v3_c11461</td><td>1</td><td>4</td><td>-</td><td>-</td><td>-</td><td>-</td><td>-</td></tr>
<tr><td>gALL_v3_c11462</td><td>6</td><td>8</td><td>-</td><td>-</td><td>-</td><td>-</td><td>-</td></tr>
<tr><td>gALL_v3_c11463</td><td>1</td><td>8</td><td>-</td><td>-</td><td>-</td><td>-</td><td>-</td></tr>
<tr><td>gALL_v3_c11464</td><td>24</td><td>29</td><td>gnl|CDD|176398</td><td>cd01803, Ubiquitin, Ubiquitin.</td><td>ref|XP_003770187.1|</td><td>PREDICTED: polyubiquitin-B [Sarcophilus harrisii]</td><td>1,00E-109</td></tr>
<tr><td>gALL_v3_c11465</td><td>1</td><td>5</td><td>gnl|CDD|144872</td><td>pfam01434, Peptidase_M41, F</td><td>ref|XP_001813433.1|</td><td>PREDICTED: similar to paraplegin [Tribolium castaneum] gb|EFA00737.1| hypothetical protein TcasGA2_TC003619 [Tribolium castaneum]</td><td>6,00E-60</td></tr>
<tr><td>gALL_v3_c11466</td><td>1</td><td>5</td><td>gnl|CDD|115148</td><td>pfam06472, ABC_membrane_</td><td>ref|XP_001605995.1|</td><td>PREDICTED: ATP-binding cassette sub-family D member 3-like [Nasonia vitripennis]</td><td>9,00E-77</td></tr>
<tr><td>gALL_v3_c11467</td><td>4</td><td>6</td><td>-</td><td>-</td><td>-</td><td>-</td><td>-</td></tr>
<tr><td>gALL_v3_c11468</td><td>6</td><td>9</td><td>gnl|CDD|147706</td><td>pfam05699, Dimer_Tnp_hAT, I</td><td>gb|EFN90112.1|</td><td>Putative AC transposase [Harpegnathos saltator]</td><td>1,00E-34</td></tr>
<tr><td>gALL_v3_c11469</td><td>3</td><td>3</td><td>gnl|CDD|119392</td><td>cd06174, MFS, The Major Faci</td><td>ref|XP_002427227.1|</td><td>conserved hypothetical protein [Pediculus humanus corporis] gb|EEB14489.1| conserved hypothetical protein [Pediculus humanus corporis]</td><td>4,00E-51</td></tr>
<tr><td>gALL_v3_c11470</td><td>6</td><td>3</td><td>-</td><td>-</td><td>-</td><td>-</td><td>-</td></tr>
<tr><td>gALL_v3_c11471</td><td>7</td><td>12</td><td>-</td><td>-</td><td>-</td><td>-</td><td>-</td></tr>
<tr><td>gALL_v3_c11472</td><td>3</td><td>2</td><td>-</td><td>-</td><td>-</td><td>-</td><td>-</td></tr>
</table>

<table>
<tr><td>gALL_v3_c11473</td><td>5</td><td>4</td><td>-</td><td>-</td><td>-</td><td>-</td><td>-</td></tr>
<tr><td>gALL_v3_c11475</td><td>1</td><td>3</td><td>-</td><td>-</td><td>-</td><td>-</td><td>-</td></tr>
<tr><td>gALL_v3_c11476</td><td>5</td><td>6</td><td>-</td><td>-</td><td>-</td><td>-</td><td>-</td></tr>
<tr><td>gALL_v3_c11477</td><td>3</td><td>2</td><td>-</td><td>-</td><td>-</td><td>-</td><td>-</td></tr>
<tr><td>gALL_v3_c11478</td><td>2</td><td>5</td><td>-</td><td>-</td><td>-</td><td>-</td><td>-</td></tr>
<tr><td>gALL_v3_c11479</td><td>4</td><td>4</td><td>-</td><td>-</td><td>-</td><td>-</td><td>-</td></tr>
<tr><td>gALL_v3_c1148</td><td>6</td><td>17</td><td>gnl|CDD|144027</td><td>pfam00287, Na_K-ATPase, Soc</td><td>ref|XP_001654266.1|</td><td>sodium/potassium-dependent atpase beta-2 subunit [Aedes aegypti] ref|XP_001654268.1| sodium/potassium-dependent atpase beta-2 subunit [Aedes aegypti] gb|EAT37</td><td>1,00E-108</td></tr>
<tr><td>gALL_v3_c11480</td><td>3</td><td>3</td><td>-</td><td>-</td><td>-</td><td>-</td><td>-</td></tr>
<tr><td>gALL_v3_c11481</td><td>3</td><td>9</td><td>gnl|CDD|133271</td><td>cd01868, Rab11_like, Rab11-li</td><td>ref|XP_004319903.1|</td><td>PREDICTED: ras-related protein Rab-11A [Tursiops truncatus]</td><td>1,00E-45</td></tr>
<tr><td>gALL_v3_c11482</td><td>0</td><td>5</td><td>-</td><td>-</td><td>-</td><td>-</td><td>-</td></tr>
<tr><td>gALL_v3_c11483</td><td>3</td><td>4</td><td>-</td><td>-</td><td>-</td><td>-</td><td>-</td></tr>
<tr><td>gALL_v3_c11484</td><td>6</td><td>8</td><td>-</td><td>-</td><td>dbj|BAN21006.1|</td><td>mitochondrial ribosomal protein L53 [Riptortus pedestris]</td><td>5,00E-32</td></tr>
<tr><td>gALL_v3_c11487</td><td>7</td><td>7</td><td>gnl|CDD|162097</td><td>TIGR00898, 2A0119, cation tra</td><td>gb|AGM32704.1|</td><td>solute carrier family 22 protein [Coptotermes formosanus]</td><td>1,00E-105</td></tr>
<tr><td>gALL_v3_c11489</td><td>81</td><td>89</td><td>-</td><td>-</td><td>-</td><td>-</td><td>-</td></tr>
<tr><td>gALL_v3_c11490</td><td>6</td><td>5</td><td>-</td><td>-</td><td>tpg|DAA64517.1|</td><td>TPA_inf: venus kinase receptor [Linepithema humile]</td><td>2,00E-62</td></tr>
<tr><td>gALL_v3_c11491</td><td>5</td><td>5</td><td>-</td><td>-</td><td>-</td><td>-</td><td>-</td></tr>
<tr><td>gALL_v3_c11492</td><td>4</td><td>2</td><td>-</td><td>-</td><td>ref|XP_002428100.1|</td><td>conserved hypothetical protein [Pediculus humanus corporis] gb|EEB15362.1| conserved hypothetical protein [Pediculus humanus corporis]</td><td>2,00E-22</td></tr>
<tr><td>gALL_v3_c11493</td><td>1</td><td>7</td><td>gnl|CDD|128491</td><td>smart00194, PTPc, Protein tyr</td><td>ref|XP_002405097.1|</td><td>phosphotyrosyl phosphatase, putative [Ixodes scapularis] gb|EEC11947.1| phosphotyrosyl phosphatase, putative [Ixodes scapularis]</td><td>9,00E-77</td></tr>
<tr><td>gALL_v3_c11494</td><td>4</td><td>2</td><td>-</td><td>-</td><td>-</td><td>-</td><td>-</td></tr>
<tr><td>gALL_v3_c11495</td><td>14</td><td>22</td><td>-</td><td>-</td><td>-</td><td>-</td><td>-</td></tr>
<tr><td>gALL_v3_c11496</td><td>2</td><td>7</td><td>gnl|CDD|128627</td><td>smart00332, PP2Cc, Serine/th</td><td>ref|XP_002424249.1|</td><td>protein phosphatase 2C, putative [Pediculus humanus corporis] gb|EEB11511.1| protein phosphatase 2C, putative [Pediculus humanus corporis]</td><td>1,00E-123</td></tr>
<tr><td>gALL_v3_c11497</td><td>2</td><td>4</td><td>gnl|CDD|185499</td><td>PTZ00174, PTZ00174, phosph</td><td>ref|XP_002617190.1|</td><td>phosphomannomutase [Clavispora lusitaniae ATCC 42720] gb|EEQ38508.1| phosphomannomutase [Clavispora lusitaniae ATCC 42720]</td><td>5,00E-39</td></tr>
<tr><td>gALL_v3_c11498</td><td>3</td><td>7</td><td>-</td><td>-</td><td>-</td><td>-</td><td>-</td></tr>
<tr><td>gALL_v3_c115</td><td>4</td><td>14</td><td>-</td><td>-</td><td>-</td><td>-</td><td>-</td></tr>
<tr><td>gALL_v3_c1150</td><td>6</td><td>7</td><td>-</td><td>-</td><td>-</td><td>-</td><td>-</td></tr>
<tr><td>gALL_v3_c11500</td><td>4</td><td>7</td><td>-</td><td>-</td><td>-</td><td>-</td><td>-</td></tr>
<tr><td>gALL_v3_c11501</td><td>3</td><td>3</td><td>-</td><td>-</td><td>-</td><td>-</td><td>-</td></tr>
<tr><td>gALL_v3_c11502</td><td>3</td><td>4</td><td>-</td><td>-</td><td>-</td><td>-</td><td>-</td></tr>
<tr><td>gALL_v3_c11503</td><td>6</td><td>3</td><td>-</td><td>-</td><td>ref|XP_967312.2|</td><td>PREDICTED: similar to exportin 7 [Tribolium castaneum]</td><td>1,00E-139</td></tr>
<tr><td>gALL_v3_c11504</td><td>5</td><td>8</td><td>-</td><td>-</td><td>-</td><td>-</td><td>-</td></tr>
<tr><td>gALL_v3_c11505</td><td>0</td><td>4</td><td>-</td><td>-</td><td>-</td><td>-</td><td>-</td></tr>
<tr><td>gALL_v3_c11508</td><td>2</td><td>3</td><td>-</td><td>-</td><td>gb|AGM32614.1|</td><td>putative mitochondrial dicarboxylate carrier, partial [Coptotermes formosanus]</td><td>1,00E-28</td></tr>
<tr><td>gALL_v3_c11509</td><td>5</td><td>3</td><td>-</td><td>-</td><td>ref|XP_002427125.1|</td><td>conserved hypothetical protein [Pediculus humanus corporis] gb|EEB14387.1| conserved hypothetical protein [Pediculus humanus corporis]</td><td>7,00E-39</td></tr>
<tr><td>gALL_v3_c1151</td><td>7</td><td>9</td><td>-</td><td>-</td><td>-</td><td>-</td><td>-</td></tr>
<tr><td>gALL_v3_c11510</td><td>7</td><td>5</td><td>-</td><td>-</td><td>-</td><td>-</td><td>-</td></tr>
<tr><td>gALL_v3_c11511</td><td>3</td><td>8</td><td>-</td><td>-</td><td>-</td><td>-</td><td>-</td></tr>
<tr><td>gALL_v3_c11512</td><td>6</td><td>4</td><td>gnl|CDD|31309</td><td>COG1112, COG1112, Superfan</td><td>ref|XP_002429122.1|</td><td>conserved hypothetical protein [Pediculus humanus corporis] gb|EEB16384.1| conserved hypothetical protein [Pediculus humanus corporis]</td><td>1,00E-168</td></tr>
<tr><td>gALL_v3_c11513</td><td>11</td><td>8</td><td>gnl|CDD|190170</td><td>pfam01956, DUF106, Integral</td><td>gb|AGM32745.1|</td><td>transmembrane and coiled-coil domains protein 1 [Coptotermes formosanus]</td><td>2,00E-66</td></tr>
<tr><td>gALL_v3_c11515</td><td>4</td><td>4</td><td>-</td><td>-</td><td>ref|XP_003708037.1|</td><td>PREDICTED: FH1/FH2 domain-containing protein 3-like [Megachile rotundata]</td><td>2,00E-72</td></tr>
<tr><td>gALL_v3_c11516</td><td>5</td><td>3</td><td>-</td><td>-</td><td>ref|XP_001663796.1|</td><td>farnesyl-pyrophosphate synthetase [Aedes aegypti] gb|EAT45207.1| AAEL003497-PA [Aedes aegypti]</td><td>4,00E-43</td></tr>
<tr><td>gALL_v3_c11517</td><td>1</td><td>4</td><td>-</td><td>-</td><td>-</td><td>-</td><td>-</td></tr>
<tr><td>gALL_v3_c11518</td><td>6</td><td>6</td><td>-</td><td>-</td><td>-</td><td>-</td><td>-</td></tr>
<tr><td>gALL_v3_c1152</td><td>3</td><td>6</td><td>-</td><td>-</td><td>-</td><td>-</td><td>-</td></tr>
<tr><td>gALL_v3_c11520</td><td>15</td><td>9</td><td>gnl|CDD|30656</td><td>COG0308, PepN, Aminopeptid</td><td>gb|EFN87052.1|</td><td>Aminopeptidase N [Harpegnathos saltator]</td><td>1,00E-94</td></tr>
<tr><td>gALL_v3_c11521</td><td>1</td><td>5</td><td>-</td><td>-</td><td>gb|EHJ74308.1|</td><td>hypothetical protein KGM_07162 [Danaus plexippus]</td><td>6,00E-15</td></tr>
<tr><td>gALL_v3_c11522</td><td>2</td><td>4</td><td>-</td><td>-</td><td>-</td><td>-</td><td>-</td></tr>
<tr><td>gALL_v3_c11524</td><td>6</td><td>15</td><td>gnl|CDD|162052</td><td>TIGR00813, sss, transporter, S</td><td>ref|XP_781922.3|</td><td>PREDICTED: sodium/myo-inositol cotransporter-like [Strongylocentrotus purpuratus]</td><td>1,00E-129</td></tr>
<tr><td>gALL_v3_c11525</td><td>12</td><td>8</td><td>-</td><td>-</td><td>-</td><td>-</td><td>-</td></tr>
<tr><td>gALL_v3_c11526</td><td>9</td><td>17</td><td>-</td><td>-</td><td>-</td><td>-</td><td>-</td></tr>
<tr><td>gALL_v3_c11527</td><td>9</td><td>15</td><td>-</td><td>-</td><td>-</td><td>-</td><td>-</td></tr>
<tr><td>gALL_v3_c11529</td><td>3</td><td>3</td><td>-</td><td>-</td><td>-</td><td>-</td><td>-</td></tr>
<tr><td>gALL_v3_c1153</td><td>8</td><td>17</td><td>-</td><td>-</td><td>-</td><td>-</td><td>-</td></tr>
<tr><td>gALL_v3_c11530</td><td>1</td><td>4</td><td>-</td><td>-</td><td>ref|NP_955875.1|</td><td>DNA polymerase alpha subunit B [Danio rerio] gb|AAH45928.1| Polymerase (DNA directed), alpha 2 [Danio rerio]</td><td>3,00E-24</td></tr>
<tr><td>gALL_v3_c11531</td><td>1</td><td>4</td><td>-</td><td>-</td><td>-</td><td>-</td><td>-</td></tr>
<tr><td>gALL_v3_c11532</td><td>8</td><td>4</td><td>gnl|CDD|192042</td><td>pfam08450, SGL, SMP-30/Gluc</td><td>gb|AFJ24877.1|</td><td>luciferin-regenerating enzyme, partial [Pachnoda marginata peregrina]</td><td>1,00E-78</td></tr>
<tr><td>gALL_v3_c11533</td><td>4</td><td>4</td><td>-</td><td>-</td><td>-</td><td>-</td><td>-</td></tr>
<tr><td>gALL_v3_c11534</td><td>6</td><td>7</td><td>-</td><td>-</td><td>ref|XP_003693005.1|</td><td>PREDICTED: uncharacterized protein LOC100865947 [Apis florea]</td><td>2,00E-99</td></tr>
<tr><td>gALL_v3_c11535</td><td>4</td><td>6</td><td>-</td><td>-</td><td>ref|XP_004212314.1|</td><td>PREDICTED: uncharacterized protein LOC101241506 [Hydra vulgaris]</td><td>2,00E-07</td></tr>
<tr><td>gALL_v3_c11536</td><td>7</td><td>5</td><td>-</td><td>-</td><td>gb|ERL83281.1|</td><td>hypothetical protein D910_00079 [Dendroctonus ponderosae]</td><td>1,00E-52</td></tr>
<tr><td>gALL_v3_c11537</td><td>4</td><td>11</td><td>-</td><td>-</td><td>-</td><td>-</td><td>-</td></tr>
<tr><td>gALL_v3_c11538</td><td>2</td><td>6</td><td>-</td><td>-</td><td>-</td><td>-</td><td>-</td></tr>
<tr><td>gALL_v3_c11539</td><td>5</td><td>3</td><td>-</td><td>-</td><td>ref|XP_006566911.1|</td><td>PREDICTED: calcium homeostasis endoplasmic reticulum protein-like isoform X1 [Apis mellifera] ref|XP_006610881.1| PREDICTED: calcium homeostasis endoplasmic reticu</td><td>5,00E-47</td></tr>
<tr><td>gALL_v3_c11540</td><td>1</td><td>7</td><td>-</td><td>-</td><td>-</td><td>-</td><td>-</td></tr>
<tr><td>gALL_v3_c11541</td><td>4</td><td>13</td><td>gnl|CDD|190091</td><td>pfam01753, zf-MYND, MYND f</td><td>ref|XP_973542.2|</td><td>PREDICTED: similar to CG8503 CG8503-PA [Tribolium castaneum]</td><td>1,00E-106</td></tr>
<tr><td>gALL_v3_c11542</td><td>7</td><td>9</td><td>-</td><td>-</td><td>ref|XP_003698489.1|</td><td>PREDICTED: protein daughterless-like, partial [Apis florea]</td><td>2,00E-35</td></tr>
<tr><td>gALL_v3_c11544</td><td>3</td><td>4</td><td>-</td><td>-</td><td>ref|XP_001603526.2|</td><td>PREDICTED: nuclear hormone receptor FTZ-F1 beta [Nasonia vitripennis]</td><td>4,00E-09</td></tr>
<tr><td>gALL_v3_c11545</td><td>2</td><td>6</td><td>-</td><td>-</td><td>-</td><td>-</td><td>-</td></tr>
<tr><td>gALL_v3_c11546</td><td>4</td><td>3</td><td>-</td><td>-</td><td>-</td><td>-</td><td>-</td></tr>
<tr><td>gALL_v3_c11547</td><td>8</td><td>16</td><td>-</td><td>-</td><td>-</td><td>-</td><td>-</td></tr>
<tr><td>gALL_v3_c11548</td><td>7</td><td>6</td><td>-</td><td>-</td><td>ref|WP_007659034.1|</td><td>hypothetical protein [Pontibacter sp. BAB1700] gb|EJF08786.1| hypothetical protein O71_18855 [Pontibacter sp. BAB1700]</td><td>3,00E-06</td></tr>
<tr><td>gALL_v3_c11549</td><td>3</td><td>4</td><td>gnl|CDD|162072</td><td>TIGR00860, LIC, Cation transp</td><td>ref|XP_004925785.1|</td><td>PREDICTED: glutamate-gated chloride channel-like isoform X2 [Bombyx mori]</td><td>3,00E-80</td></tr>
<tr><td>gALL_v3_c11550</td><td>3</td><td>8</td><td>gnl|CDD|145898</td><td>pfam02990, EMP70, Endomen</td><td>gb|EFN87604.1|</td><td>Transmembrane 9 superfamily member 3 [Harpegnathos saltator]</td><td>1,00E-107</td></tr>
<tr><td>gALL_v3_c11553</td><td>5</td><td>7</td><td>gnl|CDD|178637</td><td>PLN03088, PLN03088, SGT1, s</td><td>ref|XP_001599865.2|</td><td>PREDICTED: suppressor of G2 allele of SKP1 homolog [Nasonia vitripennis]</td><td>3,00E-62</td></tr>
<tr><td>gALL_v3_c11554</td><td>0</td><td>6</td><td>-</td><td>-</td><td>-</td><td>-</td><td>-</td></tr>
<tr><td>gALL_v3_c11555</td><td>3</td><td>11</td><td>-</td><td>-</td><td>ref|XP_973574.1|</td><td>PREDICTED: similar to CG11160 CG11160-PA [Tribolium castaneum]</td><td>6,00E-31</td></tr>
<tr><td>gALL_v3_c11556</td><td>4</td><td>2</td><td>-</td><td>-</td><td>gb|AAH91438.1|</td><td>Phf12 protein [Rattus norvegicus]</td><td>1,00E-10</td></tr>
<tr><td>gALL_v3_c11557</td><td>1</td><td>4</td><td>-</td><td>-</td><td>-</td><td>-</td><td>-</td></tr>
<tr><td>gALL_v3_c11558</td><td>1</td><td>6</td><td>-</td><td>-</td><td>-</td><td>-</td><td>-</td></tr>
<tr><td>gALL_v3_c11559</td><td>1</td><td>3</td><td>-</td><td>-</td><td>-</td><td>-</td><td>-</td></tr>
<tr><td>gALL_v3_c1156</td><td>7</td><td>11</td><td>-</td><td>-</td><td>-</td><td>-</td><td>-</td></tr>
<tr><td>gALL_v3_c11560</td><td>3</td><td>4</td><td>-</td><td>-</td><td>-</td><td>-</td><td>-</td></tr>
<tr><td>gALL_v3_c11561</td><td>3</td><td>2</td><td>-</td><td>-</td><td>gb|EFN80780.1|</td><td>Cell division protein kinase 5 [Harpegnathos saltator]</td><td>1,00E-09</td></tr>
<tr><td>gALL_v3_c11562</td><td>6</td><td>9</td><td>gnl|CDD|176333</td><td>cd01257, PH_IRS, Insulin recep</td><td>ref|XP_002429604.1|</td><td>insulin receptor substrate-1, putative [Pediculus humanus corporis] gb|EEB16866.1| insulin receptor substrate-1, putative [Pediculus humanus corporis]</td><td>1,00E-84</td></tr>
<tr><td>gALL_v3_c11563</td><td>5</td><td>10</td><td>-</td><td>-</td><td>-</td><td>-</td><td>-</td></tr>
<tr><td>gALL_v3_c11564</td><td>1</td><td>4</td><td>-</td><td>-</td><td>-</td><td>-</td><td>-</td></tr>
<tr><td>gALL_v3_c11565</td><td>1</td><td>7</td><td>-</td><td>-</td><td>-</td><td>-</td><td>-</td></tr>
<tr><td>gALL_v3_c11566</td><td>2</td><td>3</td><td>gnl|CDD|29012</td><td>cd00112, LDLa, Low Density Li</td><td>gb|EFA04774.1|</td><td>hypothetical protein TcasGA2_TC030735 [Tribolium castaneum]</td><td>1,00E-58</td></tr>
<tr><td>gALL_v3_c11567</td><td>6</td><td>4</td><td>gnl|CDD|73156</td><td>cd01650, RT_nLTR_like, RT_nL</td><td>gb|ADF18552.1|</td><td>endonuclease-reverse transcriptase [Bombyx mori]</td><td>1,00E-48</td></tr>
<tr><td>gALL_v3_c11568</td><td>5</td><td>5</td><td>gnl|CDD|132746</td><td>cd06948, NR_LBD_COUP-TF, Li</td><td>gb|AAA93013.1|</td><td>svp, partial [Schistocerca americana]</td><td>2,00E-67</td></tr>
<tr><td>gALL_v3_c11569</td><td>1</td><td>4</td><td>-</td><td>-</td><td>-</td><td>-</td><td>-</td></tr>
<tr><td>gALL_v3_c11570</td><td>4</td><td>4</td><td>-</td><td>-</td><td>-</td><td>-</td><td>-</td></tr>
<tr><td>gALL_v3_c11571</td><td>3</td><td>5</td><td>-</td><td>-</td><td>-</td><td>-</td><td>-</td></tr>
<tr><td>gALL_v3_c11572</td><td>3</td><td>8</td><td>-</td><td>-</td><td>-</td><td>-</td><td>-</td></tr>
<tr><td>gALL_v3_c11573</td><td>4</td><td>5</td><td>-</td><td>-</td><td>-</td><td>-</td><td>-</td></tr>
<tr><td>gALL_v3_c11574</td><td>3</td><td>2</td><td>gnl|CDD|191256</td><td>pfam05327, RRN3, RNA polym</td><td>ref|XP_972768.1|</td><td>PREDICTED: similar to transcription initiation factor RRN3 [Tribolium castaneum]</td><td>3,00E-34</td></tr>
<tr><td>gALL_v3_c11575</td><td>5</td><td>4</td><td>gnl|CDD|152752</td><td>pfam12317, IFT46_B_C, Intrafl</td><td>ref|XP_795443.3|</td><td>PREDICTED: intraflagellar transport protein 46 homolog [Strongylocentrotus purpuratus]</td><td>3,00E-86</td></tr>
<tr><td>gALL_v3_c11576</td><td>8</td><td>4</td><td>-</td><td>-</td><td>-</td><td>-</td><td>-</td></tr>
<tr><td>gALL_v3_c11577</td><td>1</td><td>5</td><td>gnl|CDD|128621</td><td>smart00326, SH3, Src homolog</td><td>ref|XP_006622991.1|</td><td>PREDICTED: intersectin-2-like isoform X7 [Apis dorsata]</td><td>8,00E-31</td></tr>
<tr><td>gALL_v3_c11578</td><td>4</td><td>3</td><td>-</td><td>-</td><td>-</td><td>-</td><td>-</td></tr>
<tr><td>gALL_v3_c1158</td><td>7</td><td>11</td><td>-</td><td>-</td><td>-</td><td>-</td><td>-</td></tr>
<tr><td>gALL_v3_c11580</td><td>2</td><td>3</td><td>-</td><td>-</td><td>gb|EFZ19201.1|</td><td>hypothetical protein SINV_00189 [Solenopsis invicta]</td><td>1,00E-95</td></tr>
<tr><td>gALL_v3_c11582</td><td>8</td><td>5</td><td>gnl|CDD|175974</td><td>cd00275, C2_PLC_like, C2 dom</td><td>gb|EFN78774.1|</td><td>1-phosphatidylinositol-4,5-bisphosphate phosphodiesterase gamma-1 [Harpegnathos saltator]</td><td>1,00E-136</td></tr>
<tr><td>gALL_v3_c11583</td><td>1</td><td>4</td><td>-</td><td>-</td><td>gb|EFN75713.1|</td><td>hypothetical protein EAI_09715 [Harpegnathos saltator]</td><td>4,00E-09</td></tr>
<tr><td>gALL_v3_c11584</td><td>2</td><td>4</td><td>-</td><td>-</td><td>-</td><td>-</td><td>-</td></tr>
<tr><td>gALL_v3_c11585</td><td>4</td><td>8</td><td>-</td><td>-</td><td>gb|EFN89317.1|</td><td>Histone-lysine N-methyltransferase SETMAR [Harpegnathos saltator]</td><td>3,00E-07</td></tr>
<tr><td>gALL_v3_c11586</td><td>8</td><td>7</td><td>-</td><td>-</td><td>-</td><td>-</td><td>-</td></tr>
<tr><td>gALL_v3_c11588</td><td>8</td><td>10</td><td>-</td><td>-</td><td>gb|EEZ98831.1|</td><td>hypothetical protein TcasGA2_TC004440 [Tribolium castaneum]</td><td>2,00E-68</td></tr>
<tr><td>gALL_v3_c11589</td><td>1</td><td>4</td><td>gnl|CDD|190702</td><td>pfam03647, Tmemb_14, Trans</td><td>ref|NP_001093286.1|</td><td>transmembrane protein 14C [Bombyx mori] gb|ABF51523.1| transmembrane protein 14C [Bombyx mori]</td><td>3,00E-22</td></tr>
<tr><td>gALL_v3_c1159</td><td>20</td><td>39</td><td>-</td><td>-</td><td>dbj|BAN21160.1|</td><td>prohibitin, putative [Riptortus pedestris]</td><td>2,00E-16</td></tr>
<tr><td>gALL_v3_c11590</td><td>1</td><td>4</td><td>gnl|CDD|111356</td><td>pfam02450, LACT, Lecithin:chc</td><td>ref|XP_966553.1|</td><td>PREDICTED: similar to phosphatidylcholine-sterol acyltransferase (lecithin-cholesterol acyltransferase) [Tribolium castaneum] gb|EFA04227.1| hypothetical protein TcasGA</td><td>2,00E-54</td></tr>
<tr><td>gALL_v3_c11591</td><td>5</td><td>4</td><td>-</td><td>-</td><td>-</td><td>-</td><td>-</td></tr>
<tr><td>gALL_v3_c11592</td><td>4</td><td>11</td><td>-</td><td>-</td><td>-</td><td>-</td><td>-</td></tr>
<tr><td>gALL_v3_c11593</td><td>4</td><td>4</td><td>-</td><td>-</td><td>-</td><td>-</td><td>-</td></tr>
<tr><td>gALL_v3_c11595</td><td>6</td><td>6</td><td>gnl|CDD|189621</td><td>pfam00595, PDZ, PDZ domain</td><td>ref|XP_001660486.1|</td><td>calcium/calmodulin-dependent serine protein kinase membrane-associated guanylate kinase (cask) [Aedes aegypti] gb|EAT38134.1| AAEL009937-PA [Aedes aegypti]</td><td>3,00E-44</td></tr>
<tr><td>gALL_v3_c11596</td><td>3</td><td>8</td><td>gnl|CDD|185426</td><td>PTZ00071, PTZ00071, 40S ribo</td><td>gb|EFZ09298.1|</td><td>hypothetical protein SINV_13300 [Solenopsis invicta]</td><td>9,00E-60</td></tr>
<tr><td>gALL_v3_c11597</td><td>4</td><td>4</td><td>-</td><td>-</td><td>-</td><td>-</td><td>-</td></tr>
<tr><td>gALL_v3_c11598</td><td>5</td><td>13</td><td>-</td><td>-</td><td>-</td><td>-</td><td>-</td></tr>
<tr><td>gALL_v3_c11599</td><td>5</td><td>3</td><td>-</td><td>-</td><td>-</td><td>-</td><td>-</td></tr>
<tr><td>gALL_v3_c116</td><td>14</td><td>12</td><td>-</td><td>-</td><td>gb|EFN89718.1|</td><td>N-acetyllactosaminide beta-1,3-N-acetylglucosaminyltransferase [Harpegnathos saltator]</td><td>1,00E-124</td></tr>
<tr><td>gALL_v3_c1160</td><td>7</td><td>9</td><td>-</td><td>-</td><td>-</td><td>-</td><td>-</td></tr>
<tr><td>gALL_v3_c11600</td><td>4</td><td>4</td><td>-</td><td>-</td><td>-</td><td>-</td><td>-</td></tr>
<tr><td>gALL_v3_c11601</td><td>12</td><td>7</td><td>gnl|CDD|48554</td><td>cd03005, PDI_a_ERp46, PDIa f</td><td>gb|EEZ99095.1|</td><td>hypothetical protein TcasGA2_TC004980 [Tribolium castaneum]</td><td>1,00E-49</td></tr>
<tr><td>gALL_v3_c11602</td><td>3</td><td>5</td><td>-</td><td>-</td><td>-</td><td>-</td><td>-</td></tr>
<tr><td>gALL_v3_c11603</td><td>8</td><td>4</td><td>gnl|CDD|29261</td><td>cd00204, ANK, ankyrin repeat:</td><td>ref|YP_001958455.1|</td><td>hypothetical protein Aasi_1435 [Candidatus Amoebophilus asiaticus 5a2] ref|WP_012473465.1| hypothetical protein [Candidatus Amoebophilus asiaticus] gb|ACE06726.1</td><td>8,00E-45</td></tr>
<tr><td>gALL_v3_c11604</td><td>5</td><td>2</td><td>-</td><td>-</td><td>-</td><td>-</td><td>-</td></tr>
<tr><td>gALL_v3_c11606</td><td>5</td><td>3</td><td>-</td><td>-</td><td>-</td><td>-</td><td>-</td></tr>
<tr><td>gALL_v3_c11607</td><td>3</td><td>1</td><td>-</td><td>-</td><td>-</td><td>-</td><td>-</td></tr>
<tr><td>gALL_v3_c11608</td><td>11</td><td>8</td><td>-</td><td>-</td><td>-</td><td>-</td><td>-</td></tr>
<tr><td>gALL_v3_c11609</td><td>4</td><td>9</td><td>-</td><td>-</td><td>-</td><td>-</td><td>-</td></tr>
<tr><td>gALL_v3_c1161</td><td>15</td><td>16</td><td>-</td><td>-</td><td>ref|XP_003706564.1|</td><td>PREDICTED: probable ATP-dependent RNA helicase DDX17-like [Megachile rotundata]</td><td>1,00E-10</td></tr>
<tr><td>gALL_v3_c11611</td><td>0</td><td>6</td><td>-</td><td>-</td><td>-</td><td>-</td><td>-</td></tr>
<tr><td>gALL_v3_c11612</td><td>5</td><td>7</td><td>-</td><td>-</td><td>gb|EFA13373.1|</td><td>hypothetical protein TcasGA2_TC001390 [Tribolium castaneum]</td><td>1,00E-38</td></tr>
<tr><td>gALL_v3_c11614</td><td>2</td><td>4</td><td>gnl|CDD|144234</td><td>pfam00567, TUDOR, Tudor do</td><td>gb|EKC28416.1|</td><td>Tudor domain-containing protein 1 [Crassostrea gigas]</td><td>3,00E-11</td></tr>
<tr><td>gALL_v3_c11615</td><td>0</td><td>4</td><td>-</td><td>-</td><td>-</td><td>-</td><td>-</td></tr>
<tr><td>gALL_v3_c11616</td><td>5</td><td>3</td><td>-</td><td>-</td><td>-</td><td>-</td><td>-</td></tr>
<tr><td>gALL_v3_c11617</td><td>1</td><td>6</td><td>-</td><td>-</td><td>-</td><td>-</td><td>-</td></tr>
<tr><td>gALL_v3_c11618</td><td>0</td><td>5</td><td>-</td><td>-</td><td>-</td><td>-</td><td>-</td></tr>
<tr><td>gALL_v3_c11619</td><td>3</td><td>7</td><td>gnl|CDD|191065</td><td>pfam04695, Pex14_N, Peroxis</td><td>ref|XP_001654504.1|</td><td>hypothetical protein AaeL_AAEL010368 [Aedes aegypti] gb|EAT37655.1| AAEL010368-PC [Aedes aegypti]</td><td>1,00E-14</td></tr>
<tr><td>gALL_v3_c1162</td><td>6</td><td>5</td><td>-</td><td>-</td><td>ref|XP_002900711.1|</td><td>helitron helicase-like protein [Phytophthora infestans T30-4] gb|EEY59518.1| helitron helicase-like protein [Phytophthora infestans T30-4]</td><td>5,00E-26</td></tr>
<tr><td>gALL_v3_c11620</td><td>5</td><td>3</td><td>-</td><td>-</td><td>-</td><td>-</td><td>-</td></tr>
<tr><td>gALL_v3_c11621</td><td>2</td><td>7</td><td>-</td><td>-</td><td>-</td><td>-</td><td>-</td></tr>
<tr><td>gALL_v3_c11622</td><td>3</td><td>5</td><td>gnl|CDD|48558</td><td>cd03009, TryX_like_TryX_NRX,</td><td>gb|EFN62987.1|</td><td>Nucleoredoxin [Camponotus floridanus]</td><td>1,00E-68</td></tr>
<tr><td>gALL_v3_c11623</td><td>21</td><td>21</td><td>gnl|CDD|29257</td><td>cd00200, WD40, WD40 domai</td><td>ref|XP_003244974.1|</td><td>PREDICTED: echinoderm microtubule-associated protein-like 1-like isoform 2 [Acyrthosiphon pisum]</td><td>0</td></tr>
<tr><td>gALL_v3_c11624</td><td>4</td><td>9</td><td>-</td><td>-</td><td>-</td><td>-</td><td>-</td></tr>
</table>

gALL_v3_c11625	2	5	-	-	-	-	-	
gALL_v3_c11626	3	11	gnl|CDD|189901	pfam01237, Oxysterol_BP, Oxy	ref|XP_002430881.1|	oxysterol-binding protein 1A, putative [Pediculus humanus corporis] gb|EEB18143.1| oxysterol-binding protein 1A, putative [Pediculus humanus corporis]		9,00E-14
gALL_v3_c11627	4	4	gnl|CDD|190534	pfam03131, bZIP_Maf, bZIP M	gb|AGM32753.1|	transcription factor mafG-like protein [Coptotermes formosanus]		3,00E-67
gALL_v3_c11628	1	6	gnl|CDD|128691	smart00413, ETS, erythroblast	gb|EGI66710.1|	ETS-like proteinous factor [Acromyrmex echinatior]		2,00E-22
gALL_v3_c1163	5	8	gnl|CDD|128654	smart00360, RRM, RNA recogi	ref|XP_004923229.1|	PREDICTED: RNA-binding protein Musashi homolog Rbp6-like [Bombyx mori]		1,00E-150
gALL_v3_c11630	4	5	-	-	-	-	-	
gALL_v3_c11631	5	3	-	-	-	-	-	
gALL_v3_c11632	3	11	-	-	-	-	-	
gALL_v3_c11633	5	10	-	-	gb|EGI59169.1|	Myosin-I heavy chain [Acromyrmex echinatior]		4,00E-15
gALL_v3_c11634	6	15	-	-	-	-	-	
gALL_v3_c11635	5	3	gnl|CDD|29257	cd00200, WD40, WD40 domai	ref|XP_003696542.1|	PREDICTED: target of rapamycin complex subunit lst8-like [Apis florea] ref|XP_006622143.1| PREDICTED: target of rapamycin complex subunit lst8-like [Apis dorsata]		0
gALL_v3_c11636	2	5	-	-	-	-	-	
gALL_v3_c11637	2	9	gnl|CDD|150217	pfam09469, Cobl, Cordon-bleu	ref|XP_001943347.2|	PREDICTED: hypothetical protein LOC100164801 isoform 1 [Acyrthosiphon pisum]		7,00E-57
gALL_v3_c11638	5	5	-	-	-	-	-	
gALL_v3_c11640	5	9	-	-	-	-	-	
gALL_v3_c11641	4	10	gnl|CDD|178337	PLN02736, PLN02736, long-ch	ref|XP_970461.1|	PREDICTED: similar to CG3961 CG3961-PA [Tribolium castaneum] gb|EFA08781.1| hypothetical protein TcasGA2_TC006471 [Tribolium castaneum]		1,00E-116
gALL_v3_c11643	9	7	-	-	gb|EHJ70865.1|	endonuclease-reverse transcriptase [Danaus plexippus]		2,00E-68
gALL_v3_c11644	20	19	gnl|CDD|177216	MTH00160, ND2, NADH dehyc	ref|YP_006503948.1|	NADH dehydrogenase subunit 2 (mitochondrion) [Coptotermes lacteus] gb|AFM92448.1| NADH dehydrogenase subunit 2 (mitochondrion) [Coptotermes lacteus]		2,00E-39
gALL_v3_c11645	4	4	-	-	-	-	-	
gALL_v3_c11647	3	3	gnl|CDD|128847	smart00577, CPDc, catalytic do	ref|XP_002428729.1|	import inner membrane translocase subunit TIM50-C, putative [Pediculus humanus corporis] gb|EEB15991.1| import inner membrane translocase subunit TIM50-C, putati		1,00E-116
gALL_v3_c11648	9	13	gnl|CDD|187547	cd05236, FAR-N_SDR_e, fatty	ref|XP_001602762.2|	PREDICTED: putative fatty acyl-CoA reductase CG5065-like [Nasonia vitripennis]		1,00E-44
gALL_v3_c11649	5	9	-	-	-	-	-	
gALL_v3_c1165	5	11	-	-	-	-	-	
gALL_v3_c11650	3	11	-	-	-	-	-	
gALL_v3_c11652	4	5	-	-	-	-	-	
gALL_v3_c11653	2	7	gnl|CDD|187669	cd09809, human_WWOX_like_	ref|XP_001652932.1|	short-chain dehydrogenase [Aedes aegypti] gb|ABF18524.1| conserved dehydrogenase [Aedes aegypti] gb|EAT40479.1| AAEL007811-PA [Aedes aegypti]		4,00E-50
gALL_v3_c11654	6	13	gnl|CDD|128551	smart00255, TIR, Toll - interleu	ref|XP_002431445.1|	protein toll precursor, putative [Pediculus humanus corporis] gb|EEB18707.1| protein toll precursor, putative [Pediculus humanus corporis]		1,00E-172
gALL_v3_c11655	3	4	-	-	-	-	-	
gALL_v3_c11657	4	4	-	-	ref|XP_004526738.1|	PREDICTED: probable 28S ribosomal protein S6, mitochondrial-like [Ceratitis capitata]		5,00E-54
gALL_v3_c11658	1	5	-	-	-	-	-	
gALL_v3_c11659	7	6	-	-	-	-	-	
gALL_v3_c11660	3	4	gnl|CDD|190443	pfam02826, 2-Hacid_dh_C, D-	ref|XP_006557702.1|	PREDICTED: C-terminal-binding protein isoform X7 [Apis mellifera]		5,00E-87
gALL_v3_c11661	2	8	gnl|CDD|191053	pfam04652, DUF605, Vta1 like	ref|XP_003693457.1|	PREDICTED: LOW QUALITY PROTEIN: vacuolar protein sorting-associated protein VTA1 homolog [Apis florea]		6,00E-76
gALL_v3_c11662	3	4	-	-	-	-	-	
gALL_v3_c11663	5	7	-	-	gb|AGM32955.1|	clip domain serine protease [Coptotermes formosanus]		2,00E-79
gALL_v3_c11664	4	3	-	-	-	-	-	
gALL_v3_c11665	2	7	-	-	-	-	-	
gALL_v3_c11666	5	8	-	-	-	-	-	
gALL_v3_c11668	2	4	-	-	-	-	-	
gALL_v3_c11669	4	3	-	-	-	-	-	
gALL_v3_c11670	10	11	-	-	-	-	-	
gALL_v3_c11671	7	15	-	-	gb|EFN67634.1|	Putative uncharacterized transposon-derived protein F52C9.6 [Camponotus floridanus]		6,00E-11
gALL_v3_c11672	4	3	-	-	-	-	-	
gALL_v3_c11673	7	6	-	-	-	-	-	
gALL_v3_c11675	2	3	-	-	-	-	-	
gALL_v3_c11677	3	3	-	-	-	-	-	
gALL_v3_c11678	3	6	gnl|CDD|189799	pfam01007, IRK, Inward rectifi	gb|EFN84911.1|	ATP-sensitive inward rectifier potassium channel 12 [Harpegnathos saltator]		2,00E-89
gALL_v3_c11679	5	6	-	-	-	-	-	
gALL_v3_c11680	4	3	-	-	-	-	-	
gALL_v3_c11681	2	6	-	-	-	-	-	
gALL_v3_c11682	3	6	-	-	-	-	-	
gALL_v3_c11683	3	3	-	-	-	-	-	
gALL_v3_c11685	5	3	-	-	gb|EFN68890.1|	E3 ubiquitin-protein ligase MARCH5 [Camponotus floridanus]		1,00E-13
gALL_v3_c11686	7	10	-	-	gb|EFN80384.1|	MKL/myocardin-like protein 1 [Harpegnathos saltator]		1,00E-103
gALL_v3_c11687	15	23	-	-	-	-	-	
gALL_v3_c11688	10	8	-	-	gb|EFN75816.1|	hypothetical protein EAI_06359 [Harpegnathos saltator]		1,00E-06
gALL_v3_c1169	20	20	gnl|CDD|191015	pfam04516, CP2, CP2 transcrip	gb|EFN87587.1|	Upstream-binding protein 1 [Harpegnathos saltator]		3,00E-55
gALL_v3_c11690	2	3	-	-	-	-	-	
gALL_v3_c11691	2	3	gnl|CDD|119351	cd02872, GH18_chitolectin_ch	ref|XP_002426510.1|	conserved hypothetical protein [Pediculus humanus corporis] gb|EEB13772.1| conserved hypothetical protein [Pediculus humanus corporis]		1,00E-18
gALL_v3_c11692	5	4	-	-	-	-	-	
gALL_v3_c11693	4	5	gnl|CDD|189378	pfam00079, Serpin, Serpin (se	gb|AHC06147.1|	serpin 3 [Penaeus monodon]		6,00E-41
gALL_v3_c11694	3	5	-	-	-	-	-	
gALL_v3_c11695	2	3	-	-	-	-	-	
gALL_v3_c11696	4	2	-	-	-	-	-	
gALL_v3_c11699	2	4	-	-	-	-	-	
gALL_v3_c117	6	7	-	-	-	-	-	
gALL_v3_c11700	1	5	-	-	ref|WP_010815785.1|	hypothetical protein [Enterococcus faecalis] gb|EOJ52342.1| hypothetical protein WM9_00844 [Enterococcus faecalis EnGen0345]		3,00E-07
gALL_v3_c11701	4	5	gnl|CDD|161744	TIGR00175, mito_nad_idh, iso	ref|XP_973953.2|	PREDICTED: similar to CG6439 CG6439-PA [Tribolium castaneum] gb|EFA10361.1| hypothetical protein TcasGA2_TC012587 [Tribolium castaneum]		3,00E-33
gALL_v3_c11702	5	2	gnl|CDD|191403	pfam05920, Homeobox_KN, H	ref|XP_002432088.1|	Homeobox protein PKNOX2, putative [Pediculus humanus corporis] gb|EEB19350.1| Homeobox protein PKNOX2, putative [Pediculus humanus corporis]		4,00E-69
gALL_v3_c11703	6	10	-	-	-	-	-	
gALL_v3_c11704	4	10	-	-	-	-	-	
gALL_v3_c11705	3	1	-	-	ref|XP_973878.2|	PREDICTED: similar to AGAP008379-PA [Tribolium castaneum] gb|EFA02387.1| hypothetical protein TcasGA2_TC008067 [Tribolium castaneum]		2,00E-26
gALL_v3_c11706	16	10	gnl|CDD|190008	pfam01490, Aa_trans, Transm	ref|XP_001843326.1|	conserved hypothetical protein [Culex quinquefasciatus] gb|EDS32189.1| conserved hypothetical protein [Culex quinquefasciatus]		6,00E-52
gALL_v3_c11707	13	13	-	-	-	-	-	
gALL_v3_c11709	0	6	-	-	-	-	-	
gALL_v3_c11710	2	4	-	-	-	-	-	
gALL_v3_c11712	6	15	-	-	ref|XP_001605792.1|	PREDICTED: hypothetical protein LOC100122190 [Nasonia vitripennis]		2,00E-49
gALL_v3_c11713	3	7	gnl|CDD|128654	smart00360, RRM, RNA recogi	gb|EGI57152.1|	RNA-binding protein squid [Acromyrmex echinatior]		1,00E-56
gALL_v3_c11714	17	12	-	-	ref|WP_002232540.1|	identified by MetaGeneAnnotator domain protein [Neisseria meningitidis] gb|EJU52572.1| identified by MetaGeneAnnotator domain protein [Neisseria meningitidis 9300		2,00E-10
gALL_v3_c11715	5	4	-	-	gb|ELU06794.1|	hypothetical protein CAPTEDRAFT_221758 [Capitella teleta]		3,00E-62
gALL_v3_c11717	3	3	-	-	-	-	-	
gALL_v3_c11718	3	8	-	-	-	-	-	
gALL_v3_c11719	2	1	-	-	ref|XP_002431753.1|	Dihydroxyacetone phosphate acyltransferase, putative [Pediculus humanus corporis] gb|EEB19015.1| Dihydroxyacetone phosphate acyltransferase, putative [Pediculus hu		1,00E-16
gALL_v3_c1172	3	7	gnl|CDD|147872	pfam05955, Herpes_gp2, Equi	ref|XP_002774115.1|	GRA11, putative [Perkinsus marinus ATCC 50983] gb|EER05931.1| GRA11, putative [Perkinsus marinus ATCC 50983]		1,00E-08
gALL_v3_c11720	4	6	gnl|CDD|189784	pfam00957, Synaptobrevin, Sy	ref|XP_975087.1|	PREDICTED: similar to snare protein sec22 [Tribolium castaneum] gb|EFA02290.1| hypothetical protein TcasGA2_TC007954 [Tribolium castaneum]		2,00E-50
gALL_v3_c11721	12	15	-	-	-	-	-	
gALL_v3_c11722	0	5	-	-	-	-	-	
gALL_v3_c11723	3	2	gnl|CDD|176898	cd08889, SRPBCC_PITPNM1-2	gb|EFN86826.1|	Protein retinal degeneration B [Harpegnathos saltator]		1,00E-58
gALL_v3_c11724	7	8	-	-	-	-	-	
gALL_v3_c11725	2	4	-	-	-	-	-	
gALL_v3_c11726	2	4	-	-	-	-	-	
gALL_v3_c11727	1	6	-	-	ref|XP_001604760.1|	PREDICTED: arylsulfatase B-like [Nasonia vitripennis]		1,00E-23
gALL_v3_c1173	23	19	gnl|CDD|192950	pfam12166, DUF3595, Protein	ref|XP_002430086.1|	conserved hypothetical protein [Pediculus humanus corporis] gb|EEB17348.1| conserved hypothetical protein [Pediculus humanus corporis]		3,00E-70
gALL_v3_c11730	3	2	-	-	-	-	-	
gALL_v3_c11731	4	13	-	-	-	-	-	
gALL_v3_c11732	3	3	-	-	gb|EGI69122.1|	Triple functional domain protein [Acromyrmex echinatior]		7,00E-07
gALL_v3_c11733	5	2	-	-	-	-	-	
gALL_v3_c11734	13	13	-	-	-	-	-	
gALL_v3_c11735	3	3	-	-	-	-	-	
gALL_v3_c11738	9	15	-	-	ref|XP_002589666.1|	hypothetical protein BRAFLDRAFT_64030 [Branchiostoma floridae] gb|EEN45677.1| hypothetical protein BRAFLDRAFT_64030 [Branchiostoma floridae]		3,00E-06
gALL_v3_c11739	4	7	-	-	-	-	-	
gALL_v3_c11740	3	9	-	-	ref|XP_002954633.1|	hypothetical protein VOLCADRAFT_33332 [Volvox carteri f. nagariensis] gb|EFJ44274.1| hypothetical protein VOLCADRAFT_33332 [Volvox carteri f. nagariensis]		5,00E-14
gALL_v3_c11742	2	5	-	-	-	-	-	
gALL_v3_c11743	5	3	-	-	-	-	-	
gALL_v3_c11744	4	3	-	-	-	-	-	
gALL_v3_c11745	3	3	-	-	ref|XP_001605548.2|	PREDICTED: teneurin-3-like [Nasonia vitripennis]		3,00E-55
gALL_v3_c11747	12	9	-	-	-	-	-	
gALL_v3_c11748	6	9	-	-	-	-	-	
gALL_v3_c11749	6	14	-	-	-	-	-	
gALL_v3_c1175	21	33	-	-	-	-	-	
gALL_v3_c11750	8	7	-	-	-	-	-	
gALL_v3_c11751	4	5	gnl|CDD|144327	pfam00685, Sulfotransfer_1, S	ref|XP_002432901.1|	conserved hypothetical protein [Pediculus humanus corporis] gb|EEB20163.1| conserved hypothetical protein [Pediculus humanus corporis]		3,00E-25
gALL_v3_c11752	0	6	-	-	sp|Q964R1.1|CP6J1_BL/	RecName: Full=Cytochrome P450 6j1; AltName: Full=CYPVIJ1 gb|AAK57913.1|AF281325_1 cytochrome P450 CYP6J1 [Blattella germanica]		2,00E-11
gALL_v3_c11755	3	1	gnl|CDD|192355	pfam09736, Bud13, Pre-mRNA	ref|XP_392248.4|	PREDICTED: BUD13 homolog [Apis mellifera]		1,00E-43
gALL_v3_c11756	2	7	gnl|CDD|162843	TIGR02403, trehalose_treC, al	ref|XP_006608788.1|	PREDICTED: neutral and basic amino acid transport protein rBAT-like isoform X2 [Apis dorsata]		3,00E-44
gALL_v3_c11757	3	7	gnl|CDD|128548	smart00252, SH2, Src homolog	ref|XP_967708.2|	PREDICTED: similar to AGAP009560-PA, partial [Tribolium castaneum]		1,00E-109
gALL_v3_c11759	3	6	-	-	ref|XP_001995356.1|	GH23115 [Drosophila grimshawi] gb|EDV98428.1| GH23115 [Drosophila grimshawi]		2,00E-18
gALL_v3_c1176	13	29	-	-	ref|XP_003704004.1|	PREDICTED: protein unzipped-like [Megachile rotundata]		7,00E-39
gALL_v3_c11760	1	6	-	-	-	-	-	
gALL_v3_c11761	2	6	-	-	-	-	-	
gALL_v3_c11763	3	2	gnl|CDD|151718	pfam11277, Med24_N, Media	ref|XP_003694379.1|	PREDICTED: mediator of RNA polymerase II transcription subunit 24-like [Apis florea]		7,00E-42
gALL_v3_c11764	3	3	gnl|CDD|133070	cd03858, M14_CP_N-E_like, C	gb|EFN82672.1|	Carboxypeptidase D [Harpegnathos saltator]		2,00E-39
gALL_v3_c11765	2	8	-	-	ref|XP_003737327.1|	PREDICTED: N-acetyllactosaminide beta-1,3-N-acetylglucosaminyltransferase-like [Metaseiulus occidentalis]		7,00E-16
gALL_v3_c11766	3	3	-	-	-	-	-	
gALL_v3_c11768	3	3	-	-	-	-	-	
gALL_v3_c11769	8	7	-	-	-	-	-	
gALL_v3_c1177	13	9	gnl|CDD|147085	pfam04750, Far-17a_AIG1, FAI	ref|XP_001657926.1|	hypothetical protein AaeL_AAEL006676 [Aedes aegypti] gb|EAT41703.1| AAEL006676-PA [Aedes aegypti]		2,00E-84
gALL_v3_c11770	4	4	-	-	gb|AGM32690.1|	fatty acyl-coA reductase like protein [Coptotermes formosanus]		5,00E-09
gALL_v3_c11771	7	10	gnl|CDD|128548	smart00252, SH2, Src homolog	gb|EFZ20138.1|	hypothetical protein SINV_01703 [Solenopsis invicta]		1,00E-133
gALL_v3_c11772	3	6	-	-	-	-	-	
gALL_v3_c11773	8	5	-	-	-	-	-	
gALL_v3_c11774	3	2	gnl|CDD|189905	pfam01245, Ribosomal_L19, R	ref|NP_001155559.1|	39S ribosomal protein L19, mitochondrial [Acyrthosiphon pisum] dbj|BAH71940.1| ACYPI004065 [Acyrthosiphon pisum]		1,00E-91
gALL_v3_c11777	17	13	-	-	-	-	-	
gALL_v3_c11778	1	6	-	-	-	-	-	
gALL_v3_c11779	17	27	-	-	-	-	-	
gALL_v3_c1178	13	13	-	-	-	-	-	
gALL_v3_c11780	5	6	-	-	-	-	-	
gALL_v3_c11781	2	5	-	-	ref|XP_006559674.1|	PREDICTED: sister chromatid cohesion protein PDS5 homolog B-B-like isoform X3 [Apis mellifera] ref|XP_006618559.1| PREDICTED: sister chromatid cohesion protein PDS!		5,00E-88
gALL_v3_c11783	6	11	-	-	-	-	-	
gALL_v3_c11784	2	6	gnl|CDD|187601	cd05343, Mgc4172-like_SDR_c	ref|XP_006609163.1|	PREDICTED: dehydrogenase/reductase SDR family member 11-like isoform X1 [Apis dorsata] ref|XP_006609164.1| PREDICTED: dehydrogenase/reductase SDR family mem		1,00E-33
gALL_v3_c11786	6	5	-	-	ref|XP_002733974.1|	PREDICTED: synaptotagmin-15-like, partial [Saccoglossus kowalevskii]		8,00E-07
gALL_v3_c11787	3	7	-	-	-	-	-	
gALL_v3_c11788	2	5	gnl|CDD|147776	pfam05804, KAP, Kinesin-asso	ref|XP_006812486.1|	PREDICTED: kinesin-associated protein 3-like [Saccoglossus kowalevskii]		5,00E-77
gALL_v3_c11789	4	3	-	-	ref|XP_002403306.1|	arginyl-tRNA synthetase, putative [Ixodes scapularis] gb|EEC03056.1| arginyl-tRNA synthetase, putative [Ixodes scapularis]		3,00E-14

gALL_v3_c11791	3	2	-	-	gb|EFZ22771.1|	hypothetical protein SINV_12497 [Solenopsis invicta]	8,00E-43
gALL_v3_c11792	0	6	-	-	ref|XP_003691440.1|	PREDICTED: uncharacterized protein LOC100865610 [Apis florea]	8,00E-46
gALL_v3_c11793	4	2	-	-	-	-	-
gALL_v3_c11794	4	5	-	-	ref|XP_002430186.1|	conserved hypothetical protein [Pediculus humanus corporis] gb|EEB17448.1| conserved hypothetical protein [Pediculus humanus corporis]	4,00E-09
gALL_v3_c11795	4	3	gnl|CDD|147538	pfam05403, Plasmodium_HRP	-	-	-
gALL_v3_c11796	4	5	-	-	-	-	-
gALL_v3_c11797	7	8	-	-	-	-	-
gALL_v3_c11798	4	3	-	-	ref|WP_018035801.1|	hypothetical protein [Archaea]	5,00E-08
gALL_v3_c11799	1	3	-	-	-	-	-
gALL_v3_c118	9	11	-	-	-	-	-
gALL_v3_c11800	2	4	gnl|CDD|48512	cd02963, TRX_DnaJ, TRX doma	ref|XP_006613302.1|	PREDICTED: dnaJ homolog subfamily C member 16-like [Apis dorsata]	5,00E-92
gALL_v3_c11802	5	4	-	-	-	-	-
gALL_v3_c11803	4	3	-	-	-	-	-
gALL_v3_c11804	3	3	-	-	ref|XP_003694396.1|	PREDICTED: LOW QUALITY PROTEIN: signal recognition particle 68 kDa protein-like [Apis florea]	1,00E-55
gALL_v3_c11805	4	5	-	-	ref|XP_004930010.1|	PREDICTED: uncharacterized protein LOC101737786 [Bombyx mori]	3,00E-15
gALL_v3_c11806	4	5	gnl|CDD|189372	pfam00067, p450, Cytochrom	dbj|BAD84176.1|	cytochrome P450 [Hodotermopsis sjostedti]	1,00E-59
gALL_v3_c11807	2	6	-	-	-	-	-
gALL_v3_c11808	2	3	-	-	-	-	-
gALL_v3_c11809	5	16	-	-	ref|XP_001949080.2|	PREDICTED: protein PIEZO2-like [Acyrthosiphon pisum]	1,00E-102
gALL_v3_c1181	16	29	gnl|CDD|147872	pfam05955, Herpes_gp2, Equi	ref|XP_002424351.1|	protein gar2, putative [Pediculus humanus corporis] gb|EEB11613.1| protein gar2, putative [Pediculus humanus corporis]	1,00E-10
gALL_v3_c11810	3	5	-	-	ref|XP_002428050.1|	reverse transcriptase, putative [Pediculus humanus corporis] gb|EEB15312.1| reverse transcriptase, putative [Pediculus humanus corporis]	2,00E-13
gALL_v3_c11811	3	2	-	-	-	-	-
gALL_v3_c11812	1	4	-	-	ref|XP_004525845.1|	PREDICTED: neurobeachin-like isoform X1 [Ceratitis capitata]	3,00E-55
gALL_v3_c11813	5	3	-	-	-	-	-
gALL_v3_c11815	5	2	-	-	-	-	-
gALL_v3_c11816	0	4	-	-	-	-	-
gALL_v3_c11818	1	7	-	-	-	-	-
gALL_v3_c11819	3	7	gnl|CDD|130585	TIGR01522, ATPase-IIA2_Ca, g	ref|XP_002429714.1|	calcium-transporting ATPase type 2C member, putative [Pediculus humanus corporis] gb|EEB16976.1| calcium-transporting ATPase type 2C member, putative [Pediculus hu	4,00E-98
gALL_v3_c1182	11	11	-	-	ref|XP_001851900.1|	conserved hypothetical protein [Culex quinquefasciatus] gb|EDS33799.1| conserved hypothetical protein [Culex quinquefasciatus]	5,00E-20
gALL_v3_c11821	7	6	-	-	-	-	-
gALL_v3_c11822	2	3	-	-	-	-	-
gALL_v3_c11823	5	3	-	-	-	-	-
gALL_v3_c11825	3	5	gnl|CDD|177628	PHA03392, egt, ecdysteroid U	gb|EFA09910.1|	hypothetical protein TcasGA2_TC012061 [Tribolium castaneum]	2,00E-33
gALL_v3_c11826	11	18	gnl|CDD|144648	pfam01130, CD36, CD36 famil	ref|XP_003691612.1|	PREDICTED: LOW QUALITY PROTEIN: sensory neuron membrane protein 1-like [Apis florea]	3,00E-81
gALL_v3_c11827	7	6	-	-	-	-	-
gALL_v3_c11831	1	6	-	-	-	-	-
gALL_v3_c11833	9	8	gnl|CDD|140328	PTZ00307, PTZ00307, ethanol	ref|XP_006609179.1|	PREDICTED: choline/ethanolaminephosphotransferase 1-like isoform X5 [Apis dorsata]	1,00E-124
gALL_v3_c11834	2	10	-	-	-	-	-
gALL_v3_c11836	1	5	-	-	-	-	-
gALL_v3_c11837	3	7	-	-	-	-	-
gALL_v3_c11838	2	3	-	-	-	-	-
gALL_v3_c1184	8	10	gnl|CDD|34727	COG5126, FRQ1, Ca2+-binding	ref|XP_975592.2|	PREDICTED: similar to programmed cell death protein [Tribolium castaneum]	1,00E-107
gALL_v3_c11840	7	4	gnl|CDD|178291	PLN02688, PLN02688, pyrrolir	ref|XP_974776.1|	PREDICTED: similar to GA19292-PA [Tribolium castaneum] gb|EEZ98115.1| hypothetical protein TcasGA2_TC000533 [Tribolium castaneum]	4,00E-70
gALL_v3_c11841	4	3	gnl|CDD|181764	PRK09293, PRK09293, fructos	gb|AGM32987.1|	fructose-1,6-bisphosphatase [Coptotermes formosanus]	7,00E-45
gALL_v3_c11842	3	10	-	-	-	-	-
gALL_v3_c11843	9	15	-	-	-	-	-
gALL_v3_c11844	2	2	gnl|CDD|34770	COG5170, CDC55, Serine/thre	gb|EFN87073.1|	Protein phosphatase PP2A 55 kDa regulatory subunit [Harpegnathos saltator]	8,00E-98
gALL_v3_c11845	3	3	-	-	-	-	-
gALL_v3_c11846	7	5	gnl|CDD|128336	smart00020, Tryp_SPc, Trypsir	gb|EFN72618.1|	Serine proteinase stubble [Camponotus floridanus]	2,00E-27
gALL_v3_c11847	8	7	-	-	gb|ESO12521.1|	hypothetical protein HELRODRAFT_159075 [Helobdella robusta]	3,00E-35
gALL_v3_c11848	3	4	-	-	-	-	-
gALL_v3_c11849	8	6	-	-	-	-	-
gALL_v3_c1185	35	30	-	-	-	-	-
gALL_v3_c11850	1	4	-	-	gb|EHJ63223.1|	hypothetical protein KGM_16452 [Danaus plexippus]	4,00E-67
gALL_v3_c11851	7	9	-	-	-	-	-
gALL_v3_c11852	1	5	-	-	-	-	-
gALL_v3_c11853	9	10	-	-	-	-	-
gALL_v3_c11854	7	4	-	-	-	-	-
gALL_v3_c11855	11	17	gnl|CDD|163657	cd07414, MPP_PP1_PPKL, PP1	gb|EFN86649.1|	Serine/threonine-protein phosphatase alpha-1 isoform [Harpegnathos saltator]	2,00E-77
gALL_v3_c11856	5	5	-	-	-	-	-
gALL_v3_c11857	4	3	gnl|CDD|153093	cd04704, PLA2_bee_venom_li	ref|NP_001139342.1|	phospholipase A2D precursor [Tribolium castaneum] gb|ACN42750.1| phospholipase A2D [Tribolium castaneum] gb|EFA03431.1| hypothetical protein TcasGA2_TC0134	6,00E-41
gALL_v3_c11858	3	4	-	-	-	-	-
gALL_v3_c11859	2	5	-	-	-	-	-
gALL_v3_c1186	6	5	-	-	-	-	-
gALL_v3_c11863	3	8	-	-	-	-	-
gALL_v3_c11865	5	6	-	-	gb|ENN75426.1|	hypothetical protein YQE_07977, partial [Dendroctonus ponderosae] gb|ERL94153.1| hypothetical protein D910_11435 [Dendroctonus ponderosae]	5,00E-40
gALL_v3_c11866	4	13	-	-	ref|XP_003707144.1|	PREDICTED: uncharacterized protein LOC100883413 [Megachile rotundata]	1,00E-35
gALL_v3_c11867	5	5	gnl|CDD|48564	cd03015, PRX_Typ2cys, Peroxi	gb|AFZ78681.1|	peroxiredoxin [Coptotermes formosanus]	1,00E-100
gALL_v3_c11868	11	17	-	-	-	-	-
gALL_v3_c1187	5	7	gnl|CDD|173736	cd07832, STKc_CCRK, Catalytic	gb|EGI57797.1|	Cell cycle-related kinase [Acromyrmex echinatior]	1,00E-106
gALL_v3_c11870	2	5	-	-	-	-	-
gALL_v3_c11871	4	10	-	-	gb|AAZ15235.1|	reverse transcriptase [Aedes aegypti]	1,00E-26
gALL_v3_c11872	5	6	-	-	ref|XP_003700737.1|	PREDICTED: uncharacterized protein LOC100883463 [Megachile rotundata]	1,00E-07
gALL_v3_c11873	5	8	-	-	gb|AGM32627.1|	RCC1 domain-containing protein 1-like protein, partial [Coptotermes formosanus]	1,00E-131
gALL_v3_c11874	5	3	-	-	-	-	-
gALL_v3_c11875	2	4	-	-	-	-	-
gALL_v3_c11876	2	3	-	-	gb|EFZ09636.1|	hypothetical protein SINV_01910 [Solenopsis invicta]	2,00E-06
gALL_v3_c11877	4	3	-	-	-	-	-
gALL_v3_c11878	9	7	-	-	-	-	-
gALL_v3_c11879	1	5	-	-	-	-	-
gALL_v3_c11883	5	10	-	-	-	-	-
gALL_v3_c11884	6	9	-	-	gb|ADI61823.1|	endonuclease-reverse transcriptase [Bombyx mori]	1,00E-26
gALL_v3_c11885	4	9	gnl|CDD|58649	cd00923, Cyt_c_Oxidase_Va, C	gb|AGM32434.1|	cytochrome c oxidase subunit Va [Coptotermes formosanus]	5,00E-55
gALL_v3_c11887	1	6	-	-	gb|EGI61260.1|	Condensin-2 complex subunit D3 [Acromyrmex echinatior]	8,00E-14
gALL_v3_c11888	2	5	-	-	-	-	-
gALL_v3_c11889	1	5	-	-	-	-	-
gALL_v3_c11890	2	5	-	-	gb|AFH78148.1|	cytochrome P450 [Coptotermes formosanus]	1,00E-40
gALL_v3_c11891	1	3	-	-	-	-	-
gALL_v3_c11892	4	7	-	-	-	-	-
gALL_v3_c11893	1	7	-	-	-	-	-
gALL_v3_c11894	1	4	-	-	-	-	-
gALL_v3_c11896	5	4	-	-	-	-	-
gALL_v3_c11897	3	1	-	-	gb|AGM32860.1|	hypothetical protein [Coptotermes formosanus]	2,00E-61
gALL_v3_c11898	6	7	-	-	-	-	-
gALL_v3_c11899	1	5	-	-	gb|EEZ98574.1|	hypothetical protein TcasGA2_TC001087 [Tribolium castaneum]	6,00E-87
gALL_v3_c119	13	8	-	-	-	-	-
gALL_v3_c1190	3	12	gnl|CDD|129101	smart00868, zf-AD, Zinc-finger	ref|XP_002429662.1|	conserved hypothetical protein [Pediculus humanus corporis] gb|EEB16924.1| conserved hypothetical protein [Pediculus humanus corporis]	1,00E-57
gALL_v3_c11900	3	5	-	-	-	-	-
gALL_v3_c11901	2	6	-	-	ref|XP_001654706.1|	hypothetical protein AaeL_AAEL002134 [Aedes aegypti] gb|EAT46717.1| AAEL002134-PA [Aedes aegypti]	5,00E-30
gALL_v3_c11902	5	4	-	-	-	-	-
gALL_v3_c11903	6	8	-	-	ref|XP_002427580.1|	conserved hypothetical protein [Pediculus humanus corporis] gb|EEB14842.1| conserved hypothetical protein [Pediculus humanus corporis]	6,00E-56
gALL_v3_c11905	9	13	-	-	-	-	-
gALL_v3_c11906	3	3	-	-	gb|EFN83982.1|	Vacuolar protein sorting-associated protein 13B [Harpegnathos saltator]	1,00E-42
gALL_v3_c11907	2	3	-	-	-	-	-
gALL_v3_c11908	0	6	-	-	-	-	-
gALL_v3_c11910	1	7	-	-	-	-	-
gALL_v3_c11911	1	5	-	-	-	-	-
gALL_v3_c11914	8	7	gnl|CDD|144416	pfam00810, ER_lumen_recept	gb|EHJ75451.1|	putative ER lumen protein retaining receptor [Danaus plexippus]	6,00E-59
gALL_v3_c11915	6	11	-	-	-	-	-
gALL_v3_c11916	4	2	-	-	ref|NP_001087613.1|	GDNF-inducible zinc finger protein 1 [Xenopus laevis] gb|AAH80998.1| MGC81119 protein [Xenopus laevis]	1,00E-08
gALL_v3_c11918	9	5	-	-	ref|XP_005158739.1|	PREDICTED: uncharacterized protein LOC101886321 isoform X1 [Danio rerio]	3,00E-63
gALL_v3_c11919	9	5	gnl|CDD|72942	cd04657, Piwi_ago-like, Piwi_a	gb|AGO85972.1|	argonaute 2, partial [Locusta migratoria]	7,00E-94
gALL_v3_c11920	1	3	-	-	gb|AGM32589.1|	hypothetical protein [Coptotermes formosanus]	2,00E-88
gALL_v3_c11921	2	3	-	-	-	-	-
gALL_v3_c11922	7	5	gnl|CDD|30966	COG0621, MiaB, 2-methylthio	gb|EKC36670.1|	hypothetical protein CGI_10015051, partial [Crassostrea gigas]	9,00E-73
gALL_v3_c11923	1	5	-	-	-	-	-
gALL_v3_c11925	6	7	gnl|CDD|128516	smart00220, S_TKc, Serine/Th	ref|XP_002428172.1|	serine/threonine-protein kinase NIM1, putative [Pediculus humanus corporis] gb|EEB15434.1| serine/threonine-protein kinase NIM1, putative [Pediculus humanus corpor	1,00E-102
gALL_v3_c11926	0	6	gnl|CDD|152580	pfam12145, Med12-LCEWAV,	ref|XP_003703055.1|	PREDICTED: mediator of RNA polymerase II transcription subunit 12-like protein [Megachile rotundata]	6,00E-74
gALL_v3_c11927	2	4	-	-	-	-	-
gALL_v3_c11928	10	6	-	-	-	-	-
gALL_v3_c1193	17	14	-	-	gb|AGM32762.1|	hypothetical protein [Coptotermes formosanus]	9,00E-45
gALL_v3_c11931	1	4	-	-	ref|XP_003701344.1|	PREDICTED: zinc finger HIT domain-containing protein 1-like [Megachile rotundata]	1,00E-83
gALL_v3_c11932	5	3	-	-	-	-	-
gALL_v3_c11933	4	6	-	-	-	-	-
gALL_v3_c11934	3	9	-	-	gb|AGM32394.1|	cyclin-dependent kinase 2-interacting protein-like protein [Coptotermes formosanus]	1,00E-107
gALL_v3_c11935	3	5	gnl|CDD|34653	COG5048, COG5048, FOG: Zn-	ref|NP_001071858.1|	zinc finger protein [Ciona intestinalis] dbj|BAE06772.1| zinc finger protein [Ciona intestinalis]	3,00E-72
gALL_v3_c11936	0	6	-	-	-	-	-
gALL_v3_c11938	1	5	gnl|CDD|153093	cd04704, PLA2_bee_venom_li	gb|EFX87802.1|	hypothetical protein DAPPUDRAFT_306373 [Daphnia pulex]	2,00E-10
gALL_v3_c11939	3	8	-	-	gb|EKC30267.1|	B9 domain-containing protein 2 [Crassostrea gigas]	4,00E-27
gALL_v3_c11941	4	7	gnl|CDD|48548	cd02999, PDI_a_ERp44_like, P	ref|XP_002424279.1|	conserved hypothetical protein [Pediculus humanus corporis] gb|EEB11541.1| conserved hypothetical protein [Pediculus humanus corporis]	4,00E-33
gALL_v3_c11942	6	6	-	-	-	-	-
gALL_v3_c11943	8	8	gnl|CDD|148655	pfam07174, FAP, Fibronectin-a	ref|XP_001623078.1|	predicted protein [Nematostella vectensis] gb|EDO30978.1| predicted protein [Nematostella vectensis]	1,00E-09
gALL_v3_c11944	1	5	-	-	-	-	-
gALL_v3_c11945	4	2	-	-	-	-	-
gALL_v3_c11946	4	3	-	-	gb|ADC35398.1|	beta-integrin [Fenneropenaeus chinensis]	6,00E-25
gALL_v3_c11947	4	3	-	-	ref|WP_005937134.1|	conserved domain protein, partial [Faecalibacterium prausnitzii] gb|EFQ08391.1| hypothetical protein HMPREF9436_00081, partial [Faecalibacterium cf. prausnitzii KLE12	6,00E-68
gALL_v3_c11950	4	2	-	-	gb|EGI70159.1|	Ectonucleoside triphosphate diphosphohydrolase 5 [Acromyrmex echinatior]	2,00E-37
gALL_v3_c11951	13	11	-	-	-	-	-
gALL_v3_c11952	6	6	-	-	ref|XP_002010270.1|	GI14784 [Drosophila mojavensis] ref|XP_002055368.1| GJ18826 [Drosophila virilis] gb|EDW07587.1| GI14784 [Drosophila mojavensis] gb|EDW65569.1| GJ18826 [Drosop	4,00E-10
gALL_v3_c11953	3	3	gnl|CDD|162277	TIGR01271, CFTR_protein, cys	ref|XP_003401062.1|	PREDICTED: multidrug resistance-associated protein 4-like [Bombus terrestris]	2,00E-57
gALL_v3_c11956	6	3	-	-	gb|EFA09328.1|	hypothetical protein TcasGA2_TC001654 [Tribolium castaneum]	1,00E-19
gALL_v3_c11957	3	2	-	-	-	-	-
gALL_v3_c11958	3	4	-	-	-	-	-
gALL_v3_c11959	1	6	gnl|CDD|190080	pfam01725, Ham1p_like, Ham	gb|EFN75538.1|	Inosine triphosphate pyrophosphatase [Harpegnathos saltator]	1,00E-99

gALL_v3_c1196	12	11	-	-	ref|XP_971897.1|	PREDICTED: similar to metalloprotease [Tribolium castaneum] gb|EEZ97922.1| hypothetical protein TcasGA2_TC000308 [Tribolium castaneum]	5,00E-31
gALL_v3_c11960	0	6	-	-	-	-	-
gALL_v3_c11961	5	4	gnl|CDD|148965	pfam07651, ANTH, ANTH dom	gb|EFN85496.1|	Huntingtin-interacting protein 1 [Harpegnathos saltator]	6,00E-92
gALL_v3_c11962	11	6	gnl|CDD|29257	cd00200, WD40, WD40 domai	gb|EFN89603.1|	Bromodomain and WD repeat-containing protein 3 [Harpegnathos saltator]	0
gALL_v3_c11963	7	4	-	-	gb|AAU20851.2|	hexamerin I [Reticulitermes flavipes]	5,00E-20
gALL_v3_c11965	6	9	gnl|CDD|189450	pfam00209, SNF, Sodium:neur	gb|EFN87954.1|	Sodium- and chloride-dependent GABA transporter 1 [Harpegnathos saltator]	1,00E-149
gALL_v3_c11966	3	7	-	-	-	-	-
gALL_v3_c11967	1	5	-	-	gb|EGI58901.1|	Brain tumor protein [Acromyrmex echinatior]	9,00E-34
gALL_v3_c11968	6	8	-	-	-	-	-
gALL_v3_c11969	5	2	-	-	-	-	-
gALL_v3_c1197	8	9	-	-	-	-	-
gALL_v3_c11970	4	3	-	-	-	-	-
gALL_v3_c11971	1	5	-	-	dbj|BAD04856.1|	reverse transcriptase [Takifugu rubripes]	2,00E-10
gALL_v3_c11972	2	3	-	-	-	-	-
gALL_v3_c11973	7	12	-	-	-	-	-
gALL_v3_c11974	2	4	-	-	-	-	-
gALL_v3_c11975	2	4	-	-	gb|AGM32371.1|	ankyrin repeat protein [Coptotermes formosanus]	2,00E-08
gALL_v3_c11976	1	4	-	-	gb|EFN60628.1|	hypothetical protein EAG_05356 [Camponotus floridanus]	5,00E-12
gALL_v3_c11977	5	4	gnl|CDD|149081	pfam07815, Abi_HHR, Abl-inte	ref|XP_001946649.1|	PREDICTED: abl interactor 2-like [Acyrthosiphon pisum]	1,00E-119
gALL_v3_c11978	2	2	-	-	-	-	-
gALL_v3_c1198	10	18	gnl|CDD|190503	pfam03016, Exostosin, Exostos	ref|XP_002430502.1|	Exostosin-2, putative [Pediculus humanus corporis] gb|EEB17764.1| Exostosin-2, putative [Pediculus humanus corporis]	1,00E-118
gALL_v3_c11980	1	5	-	-	-	-	-
gALL_v3_c11981	2	5	-	-	gb|EFN69738.1|	hypothetical protein EAG_10059 [Camponotus floridanus]	3,00E-18
gALL_v3_c11982	1	8	-	-	gb|AAQ24550.1|	Blo t Gal d 1 allergen [Blomia tropicalis]	2,00E-28
gALL_v3_c11983	8	10	gnl|CDD|178676	PLN03130, PLN03130, ABC tra	ref|XP_004084145.1|	PREDICTED: multidrug resistance-associated protein 5-like [Oryzias latipes]	1,00E-119
gALL_v3_c11984	3	2	-	-	-	-	-
gALL_v3_c11985	10	14	-	-	gb|AAL40415.1|AF3698	endonuclease/reverse transcriptase [Branchiostoma floridae]	1,00E-06
gALL_v3_c11986	9	5	gnl|CDD|109812	pfam00769, ERM, Ezrin/radixi	ref|XP_002430488.1|	Hrp65 protein, putative [Pediculus humanus corporis] gb|EEB17750.1| Hrp65 protein, putative [Pediculus humanus corporis]	4,00E-46
gALL_v3_c11988	3	1	-	-	-	-	-
gALL_v3_c11989	7	4	-	-	-	-	-
gALL_v3_c1199	17	34	gnl|CDD|31389	COG1196, Smc, Chromosome	gb|EFZ21057.1|	hypothetical protein SINV_14443 [Solenopsis invicta]	1,00E-142
gALL_v3_c11990	3	5	-	-	-	-	-
gALL_v3_c11992	2	6	-	-	-	-	-
gALL_v3_c11993	8	6	gnl|CDD|32269	COG2086, FixA, Electron trans	ref|XP_003699376.1|	PREDICTED: electron transfer flavoprotein subunit beta-like [Megachile rotundata]	1,00E-136
gALL_v3_c11994	2	4	-	-	-	-	-
gALL_v3_c11995	11	13	-	-	ref|YP_002726911.1|	hypothetical protein WRi_003020 [Wolbachia sp. wRi] ref|WP_012673125.1| hypothetical protein [Wolbachia sp. wRi] gb|ACN95120.1| hypothetical protein WRi_003020	1,00E-24
gALL_v3_c11997	3	3	-	-	-	-	-
gALL_v3_c11998	7	10	-	-	ref|XP_003396853.1|	PREDICTED: hypothetical protein LOC100649961 [Bombus terrestris]	4,00E-13
gALL_v3_c11999	4	2	-	-	-	-	-
gALL_v3_c12	19	30	gnl|CDD|185628	PTZ00449, PTZ00449, 104 kDa	ref|XP_001602898.2|	PREDICTED: hypothetical protein LOC100119051 [Nasonia vitripennis]	1,00E-16
gALL_v3_c120	14	16	-	-	-	-	-
gALL_v3_c12000	2	3	gnl|CDD|145092	pfam01756, ACOX, Acyl-CoA o	ref|XP_002433236.1|	Acyl-coenzyme A oxidase 1, peroxisomal, putative [Pediculus humanus corporis] gb|EEB20498.1| Acyl-coenzyme A oxidase 1, peroxisomal, putative [Pediculus humanus cc	1,00E-30
gALL_v3_c12001	0	5	-	-	-	-	-
gALL_v3_c12002	1	5	-	-	-	-	-
gALL_v3_c12003	2	6	-	-	-	-	-
gALL_v3_c12004	8	17	gnl|CDD|132947	cd06616, PKc_MKK4, Catalytic	ref|XP_001603456.1|	PREDICTED: dual specificity mitogen-activated protein kinase kinase 4-like [Nasonia vitripennis]	0
gALL_v3_c12005	2	3	-	-	-	-	-
gALL_v3_c12006	2	4	gnl|CDD|193592	cd09978, LOTUS_2_Limkain_b	ref|XP_003400868.1|	PREDICTED: limkain-b1-like [Bombus terrestris]	1,00E-77
gALL_v3_c12007	4	4	-	-	gb|EFZ13055.1|	hypothetical protein SINV_08207 [Solenopsis invicta]	1,00E-105
gALL_v3_c12008	7	6	-	-	-	-	-
gALL_v3_c12010	7	13	gnl|CDD|190051	pfam01603, B56, Protein phos	ref|XP_004924522.1|	PREDICTED: serine/threonine-protein phosphatase 2A 56 kDa regulatory subunit epsilon isoform-like isoform X1 [Bombyx mori] ref|XP_004924523.1| PREDICTED: serine/t	1,00E-179
gALL_v3_c12011	4	3	-	-	-	-	-
gALL_v3_c12012	4	10	-	-	-	-	-
gALL_v3_c12013	2	4	-	-	-	-	-
gALL_v3_c12014	5	12	-	-	ref|XP_001849898.1|	mitochondrial ribosomal protein, L33 [Culex quinquefasciatus] gb|EDS31021.1| mitochondrial ribosomal protein, L33 [Culex quinquefasciatus]	6,00E-23
gALL_v3_c12015	2	6	-	-	ref|XP_001607184.2|	PREDICTED: LOW QUALITY PROTEIN: zinc finger protein 616-like [Nasonia vitripennis]	5,00E-56
gALL_v3_c12016	6	8	gnl|CDD|176096	cd08544, Reeler, Reeler, the N	ref|XP_002423127.1|	conserved hypothetical protein [Pediculus humanus corporis] gb|EEB10389.1| conserved hypothetical protein [Pediculus humanus corporis]	3,00E-54
gALL_v3_c12017	5	12	gnl|CDD|152787	pfam12352, V-SNARE_C, Snare	gb|ESO83159.1|	hypothetical protein LOTGIDRAFT_222883 [Lottia gigantea]	2,00E-46
gALL_v3_c12018	8	4	-	-	-	-	-
gALL_v3_c12019	1	5	-	-	gb|EFN89736.1|	hypothetical protein EAI_01984 [Harpegnathos saltator]	2,00E-48
gALL_v3_c1202	6	16	-	-	gb|EGI65470.1|	Neuroguidin-A [Acromyrmex echinatior]	1,00E-32
gALL_v3_c12020	2	4	-	-	-	-	-
gALL_v3_c12021	5	6	-	-	-	-	-
gALL_v3_c12022	7	4	-	-	-	-	-
gALL_v3_c12023	5	4	-	-	-	-	-
gALL_v3_c12024	6	7	gnl|CDD|190317	pfam02466, Tim17, Tim17/Tin	ref|XP_001662950.1|	hypothetical protein AaeL_AAEL012820 [Aedes aegypti] gb|EAT34978.1| AAEL012820-PA [Aedes aegypti]	2,00E-48
gALL_v3_c12025	5	2	-	-	-	-	-
gALL_v3_c12026	0	4	-	-	-	-	-
gALL_v3_c12027	1	5	-	-	-	-	-
gALL_v3_c12028	4	5	-	-	-	-	-
gALL_v3_c12029	7	8	-	-	gb|EFN64777.1|	WD repeat domain phosphoinositide-interacting protein 2 [Camponotus floridanus]	3,00E-07
gALL_v3_c1203	6	18	-	-	-	-	-
gALL_v3_c12030	3	5	gnl|CDD|128621	smart00326, SH3, Src homolog	gb|EFZ16157.1|	hypothetical protein SINV_05596 [Solenopsis invicta]	3,00E-30
gALL_v3_c12031	3	3	-	-	-	-	-
gALL_v3_c12032	6	6	-	-	-	-	-
gALL_v3_c12034	4	4	-	-	pdb|4GO6|B	Chain B, Crystal Structure Of Hcf-1 Self-association Sequence 1 pdb|4GO6|D Chain D, Crystal Structure Of Hcf-1 Self-association Sequence 1	7,00E-76
gALL_v3_c12035	2	3	-	-	-	-	-
gALL_v3_c12036	4	4	-	-	-	-	-
gALL_v3_c12037	12	7	gnl|CDD|189372	pfam00067, p450, Cytochrom	sp|P29981.1|CP4C1_BL	RecName: Full=Cytochrome P450 4C1; AltName: Full=CYPIVC1 gb|AAA27819.1| cytochrome P450 [Blaberus discoidalis]	7,00E-71
gALL_v3_c12038	4	4	gnl|CDD|189341	pfam00010, HLH, Helix-loop-h	gb|EFX82019.1|	hypothetical protein DAPPUDRAFT_317068 [Daphnia pulex]	1,00E-43
gALL_v3_c12039	2	3	gnl|CDD|178019	PLN02397, PLN02397, asparta	ref|XP_003401530.1|	PREDICTED: LOW QUALITY PROTEIN: probable aspartate aminotransferase, cytoplasmic-like [Bombus terrestris]	2,00E-52
gALL_v3_c1204	4	4	-	-	gb|EFZ21660.1|	hypothetical protein SINV_13482 [Solenopsis invicta]	5,00E-36
gALL_v3_c12040	5	4	-	-	-	-	-
gALL_v3_c12041	5	2	-	-	-	-	-
gALL_v3_c12042	9	12	-	-	ref|XP_006818867.1|	PREDICTED: next to BRCA1 gene 1 protein-like isoform X2 [Saccoglossus kowalevskii]	3,00E-21
gALL_v3_c12043	6	3	-	-	-	-	-
gALL_v3_c12044	3	4	-	-	dbj|BAM15638.1|	dopamine/ecdysteroid receptor [Gryllus bimaculatus]	8,00E-55
gALL_v3_c12045	4	6	-	-	ref|XP_970374.1|	PREDICTED: similar to AGAP010054-PA [Tribolium castaneum] gb|EFA02902.1| brain tumor protein [Tribolium castaneum]	2,33E-156
gALL_v3_c12046	7	8	-	-	ref|XP_003403333.1|	PREDICTED: hypothetical protein LOC100651907 isoform 3 [Bombus terrestris]	2,00E-35
gALL_v3_c12047	3	2	-	-	emb|CAP20022.1|	transposase [Eriphia verrucosa]	2,00E-23
gALL_v3_c12048	4	2	gnl|CDD|188675	cd08720, RGS_SNX25, Regulat	ref|XP_003700301.1|	PREDICTED: sorting nexin-25-like [Megachile rotundata]	2,00E-65
gALL_v3_c12050	5	7	gnl|CDD|189934	pfam01302, CAP_GLY, CAP-Gly	gb|EFN67804.1|	hypothetical protein EAG_07302 [Camponotus floridanus]	7,00E-45
gALL_v3_c12051	9	13	-	-	-	-	-
gALL_v3_c12052	4	3	-	-	-	-	-
gALL_v3_c12053	1	6	gnl|CDD|190230	pfam02156, Glyco_hydro_26,	dbj|BAL68133.1|	beta-mannanase, partial [Symbiotic protist of Reticulitermes speratus]	2,00E-68
gALL_v3_c12054	3	6	-	-	-	-	-
gALL_v3_c12055	4	2	-	-	-	-	-
gALL_v3_c12056	7	4	gnl|CDD|148093	pfam06278, DUF1032, Protein	gb|EEZ98859.1|	hypothetical protein TcasGA2_TC004469 [Tribolium castaneum]	2,00E-11
gALL_v3_c12057	1	8	gnl|CDD|144972	pfam01576, Myosin_tail_1, M	gb|AAB34531.2|	myosin heavy chain [Drosophila melanogaster]	4,00E-24
gALL_v3_c12058	3	6	-	-	-	-	-
gALL_v3_c12059	2	4	gnl|CDD|128829	smart00557, IG_FLMN, Filami	gb|EFX86436.1|	hypothetical protein DAPPUDRAFT_208250 [Daphnia pulex]	5,00E-75
gALL_v3_c12060	5	5	-	-	-	-	-
gALL_v3_c12061	3	1	-	-	-	-	-
gALL_v3_c12062	0	5	-	-	-	-	-
gALL_v3_c12063	3	3	-	-	ref|XP_002424406.1|	hypothetical protein Phum_PHUM128610 [Pediculus humanus corporis] gb|EEB11668.1| hypothetical protein Phum_PHUM128610 [Pediculus humanus corporis]	1,00E-26
gALL_v3_c12065	1	3	-	-	-	-	-
gALL_v3_c12066	3	1	gnl|CDD|161952	TIGR00603, rad25, DNA repair	ref|XP_001618128.1|	hypothetical protein NEMVEDRAFT_v1g155676 [Nematostella vectensis] gb|EDO26028.1| predicted protein [Nematostella vectensis]	1,00E-24
gALL_v3_c12067	2	4	-	-	ref|XP_004928680.1|	PREDICTED: putative RNA polymerase II subunit B1 CTD phosphatase RPAP2-like [Bombyx mori]	1,00E-33
gALL_v3_c12068	2	7	-	-	-	-	-
gALL_v3_c12069	9	12	-	-	-	-	-
gALL_v3_c12070	3	1	-	-	-	-	-
gALL_v3_c12071	3	2	-	-	-	-	-
gALL_v3_c12072	7	12	-	-	gb|EFN76912.1|	DnaJ-like protein subfamily C member 13 [Harpegnathos saltator]	2,00E-97
gALL_v3_c12074	2	4	-	-	-	-	-
gALL_v3_c12076	2	2	gnl|CDD|133266	cd01863, Rab18, Rab18 subfar	ref|XP_970378.1|	PREDICTED: similar to RAB18, member RAS oncogene family [Tribolium castaneum] gb|EFA03965.1| hypothetical protein TcasGA2_TC014110 [Tribolium castaneum]	7,00E-73
gALL_v3_c12077	4	4	gnl|CDD|193344	pfam12871, PRP38_assoc, Pre	-	-	-
gALL_v3_c12078	5	5	-	-	gb|EFA04619.1|	u-shaped [Tribolium castaneum]	1,00E-17
gALL_v3_c12079	4	4	-	-	ref|XP_001859049.1|	importin beta-3 [Culex quinquefasciatus] gb|EDS35016.1| importin beta-3 [Culex quinquefasciatus]	6,00E-09
gALL_v3_c1208	11	15	-	-	-	-	-
gALL_v3_c12080	6	12	gnl|CDD|128577	smart00281, LamB, Laminin B	gb|EFZ16979.1|	hypothetical protein SINV_11205 [Solenopsis invicta]	0
gALL_v3_c12082	11	7	gnl|CDD|178675	PLN03129, PLN03129, NADP-c	gb|AGC84405.1|	NADP-dependent malic enzyme-like protein [Locusta migratoria]	0
gALL_v3_c12083	2	5	-	-	gb|ENN82980.1|	hypothetical protein YQE_00656, partial [Dendroctonus ponderosae]	3,00E-06
gALL_v3_c12084	3	5	-	-	-	-	-
gALL_v3_c12085	3	5	gnl|CDD|128368	smart00053, DYNc, Dynamin,	ref|XP_006622930.1|	PREDICTED: dynamin-1-like protein-like [Apis dorsata]	1,00E-103
gALL_v3_c12086	3	5	-	-	-	-	-
gALL_v3_c12087	9	16	-	-	-	-	-
gALL_v3_c1209	6	5	-	-	-	-	-
gALL_v3_c12090	5	5	-	-	-	-	-
gALL_v3_c12091	3	3	-	-	ref|XP_004933636.1|	PREDICTED: splicing factor 3B subunit 3-like isoform X2 [Bombyx mori]	1,00E-105
gALL_v3_c12092	1	4	-	-	ref|XP_001604665.2|	PREDICTED: HEAT repeat-containing protein 1-like [Nasonia vitripennis]	2,00E-16
gALL_v3_c12095	2	4	-	-	-	-	-
gALL_v3_c12096	5	2	-	-	-	-	-
gALL_v3_c12097	5	7	gnl|CDD|190607	pfam03345, DDOST_48kD, Oli	gb|EDL80873.1|	rCG30980, isoform CRA_d [Rattus norvegicus]	3,00E-60
gALL_v3_c12099	8	5	-	-	-	-	-
gALL_v3_c1210	5	4	-	-	-	-	-
gALL_v3_c12101	3	2	-	-	-	-	-
gALL_v3_c12102	5	3	-	-	gb|ENN72037.1|	hypothetical protein YQE_11325, partial [Dendroctonus ponderosae]	2,00E-07
gALL_v3_c12103	1	5	-	-	-	-	-
gALL_v3_c12104	5	8	-	-	ref|XP_003700270.1|	PREDICTED: uncharacterized protein LOC100877239 [Megachile rotundata]	3,00E-58
gALL_v3_c12105	1	6	gnl|CDD|176034	cd08388, C2A_Synaptotagmin	ref|XP_968583.1|	PREDICTED: similar to AGAP001800-PA [Tribolium castaneum] gb|EEZ97753.1| synaptotagmin IV [Tribolium castaneum]	1,00E-151
gALL_v3_c12106	5	4	gnl|CDD|176272	cd00835, RanBD, Ran-binding	ref|XP_003702330.1|	PREDICTED: nuclear pore complex protein Nup50-like [Megachile rotundata]	1,00E-34
gALL_v3_c12107	4	6	-	-	-	-	-
gALL_v3_c12108	1	7	-	-	-	-	-
gALL_v3_c12109	6	10	gnl|CDD|189583	pfam00520, Ion_trans, Ion tra	ref|XP_003706369.1|	PREDICTED: mucolipin-3-like [Megachile rotundata]	1,00E-141

gALL_v3_c1211	7	4	-	-	ref|XP_006565823.1|	PREDICTED: transcription factor 12 isoform X10 [Apis mellifera]	1,00E-50
gALL_v3_c12111	4	2	-	-	-	-	-
gALL_v3_c12112	5	4	-	-	-	-	-
gALL_v3_c12115	9	11	gnl|CDD|144080	pfam00350, Dynamin_N, Dyna	ref|XP_006623229.1|	PREDICTED: dynamin-like 120 kDa protein, mitochondrial-like isoform X1 [Apis dorsata]	0
gALL_v3_c12116	7	5	gnl|CDD|31438	COG1245, COG1245, Predicted	ref|XP_002424051.1|	ATP-binding cassette subfamily E, member 1 [Pediculus humanus corporis] gb|EEB11313.1| ATP-binding cassette subfamily E, member 1 [Pediculus humanus corporis]	1,00E-148
gALL_v3_c12117	5	3	-	-	-	-	-
gALL_v3_c12118	2	7	-	-	-	-	-
gALL_v3_c12119	0	5	-	-	-	-	-
gALL_v3_c1212	5	6	-	-	-	-	-
gALL_v3_c12120	5	6	-	-	-	-	-
gALL_v3_c12121	3	3	-	-	ref|XP_002423257.1|	conserved hypothetical protein [Pediculus humanus corporis] gb|EEB10519.1| conserved hypothetical protein [Pediculus humanus corporis]	8,00E-73
gALL_v3_c12122	5	2	-	-	-	-	-
gALL_v3_c12123	2	3	-	-	-	-	-
gALL_v3_c12124	2	6	gnl|CDD|193474	pfam13001, Ecm29, Proteasor	ref|XP_003425122.1|	PREDICTED: proteasome-associated protein ECM29 homolog [Nasonia vitripennis]	1,00E-36
gALL_v3_c12126	4	4	-	-	-	-	-
gALL_v3_c12127	2	5	-	-	gb|AAN76819.1|AF452	large conductance calcium activated potassium channel pSlo spliceform 1-5A [Periplaneta americana]	6,00E-54
gALL_v3_c12128	2	3	-	-	gb|ADI61823.1|	endonuclease-reverse transcriptase [Bombyx mori]	5,00E-20
gALL_v3_c12129	3	8	-	-	-	-	-
gALL_v3_c1213	14	14	-	-	-	-	-
gALL_v3_c12130	2	6	-	-	-	-	-
gALL_v3_c12131	4	3	-	-	-	-	-
gALL_v3_c12132	3	2	-	-	-	-	-
gALL_v3_c12133	6	4	-	-	-	-	-
gALL_v3_c12134	9	9	-	-	gb|ADF18552.1|	endonuclease-reverse transcriptase [Bombyx mori]	1,00E-07
gALL_v3_c12136	4	3	-	-	-	-	-
gALL_v3_c12137	1	6	-	-	-	-	-
gALL_v3_c12138	5	8	-	-	-	-	-
gALL_v3_c12139	8	4	gnl|CDD|128621	smart00326, SH3, Src homolog	pdb|3ULR|B	Chain B, Lysozyme Contamination Facilitates Crystallization Of A Hetero- Trimericcortactin:arg:lysozyme Complex	1,00E-20
gALL_v3_c1214	15	13	gnl|CDD|180049	PRK05385, PRK05385, phosph	ref|XP_002427052.1|	phosphoribosylamine-glycine ligase, putative [Pediculus humanus corporis] gb|EEB14314.1| phosphoribosylamine-glycine ligase, putative [Pediculus humanus corporis]	0
gALL_v3_c12140	5	2	-	-	-	-	-
gALL_v3_c12141	4	2	-	-	-	-	-
gALL_v3_c12142	7	4	-	-	-	-	-
gALL_v3_c12143	2	3	-	-	ref|XP_395559.4|	PREDICTED: serine/threonine-protein phosphatase 1 regulatory subunit 10 isoform X3 [Apis mellifera] ref|XP_006562867.1| PREDICTED: serine/threonine-protein phosph	7,00E-15
gALL_v3_c12144	4	2	-	-	-	-	-
gALL_v3_c12145	3	2	-	-	-	-	-
gALL_v3_c12146	4	2	-	-	-	-	-
gALL_v3_c12147	5	2	-	-	-	-	-
gALL_v3_c12148	6	5	-	-	-	-	-
gALL_v3_c12149	1	7	-	-	-	-	-
gALL_v3_c1215	5	7	-	-	-	-	-
gALL_v3_c12150	4	3	-	-	-	-	-
gALL_v3_c12151	2	4	-	-	ref|XP_006563571.1|	PREDICTED: splicing factor 3A subunit 3 isoform X1 [Apis mellifera] ref|XP_006608253.1| PREDICTED: splicing factor 3A subunit 3-like isoform X2 [Apis dorsata]	6,00E-78
gALL_v3_c12152	8	5	gnl|CDD|31499	COG1308, EGD2, Transcription	ref|XP_003705050.1|	PREDICTED: nascent polypeptide-associated complex subunit alpha-like [Megachile rotundata]	2,00E-50
gALL_v3_c12153	3	3	-	-	-	-	-
gALL_v3_c12154	5	8	-	-	ref|XP_318981.4|	AGAP009864-PA [Anopheles gambiae str. PEST] gb|EAA14418.5| AGAP009864-PA [Anopheles gambiae str. PEST]	5,00E-77
gALL_v3_c12155	4	6	gnl|CDD|189378	pfam00079, Serpin, Serpin (se	gb|AHC06147.1|	serpin 3 [Penaeus monodon]	7,00E-41
gALL_v3_c12156	2	5	-	-	-	-	-
gALL_v3_c12157	0	6	-	-	-	-	-
gALL_v3_c12158	5	2	-	-	-	-	-
gALL_v3_c12159	1	6	-	-	-	-	-
gALL_v3_c12160	7	11	gnl|CDD|162267	TIGR01243, CDC48, AAA famil	gb|AEE61431.1|	unknown [Dendroctonus ponderosae]	1,00E-113
gALL_v3_c12162	6	5	gnl|CDD|185766	cd09243, BRO1_Brox_like, Pro	gb|EFZ10054.1|	hypothetical protein SINV_07963 [Solenopsis invicta]	6,00E-56
gALL_v3_c12163	3	4	-	-	ref|XP_006608578.1|	PREDICTED: neurogenic locus Notch protein-like [Apis dorsata]	4,00E-25
gALL_v3_c12164	2	5	-	-	-	-	-
gALL_v3_c12168	3	3	-	-	-	-	-
gALL_v3_c12169	5	5	-	-	-	-	-
gALL_v3_c12170	1	4	-	-	-	-	-
gALL_v3_c12171	3	5	gnl|CDD|140307	PTZ00284, PTZ00284, protein	dbj|BAM20054.1|	srpk protein, partial [Papilio xuthus]	5,00E-85
gALL_v3_c12173	5	8	-	-	-	-	-
gALL_v3_c12174	3	3	-	-	-	-	-
gALL_v3_c12175	6	4	-	-	-	-	-
gALL_v3_c12177	3	8	-	-	-	-	-
gALL_v3_c12178	3	5	gnl|CDD|129082	smart00849, Lactamase_B, Me	gb|EHJ77997.1|	hypothetical protein KGM_17388 [Danaus plexippus]	7,00E-71
gALL_v3_c12179	2	4	gnl|CDD|189390	pfam00102, Y_phosphatase, P	ref|XP_003702902.1|	PREDICTED: tyrosine-protein phosphatase Lar-like [Megachile rotundata]	4,00E-78
gALL_v3_c1218	16	17	-	-	ref|XP_002428860.1|	hypothetical protein Phum_PHUM405430 [Pediculus humanus corporis] gb|EEB16122.1| hypothetical protein Phum_PHUM405430 [Pediculus humanus corporis]	4,00E-09
gALL_v3_c12180	2	3	-	-	-	-	-
gALL_v3_c12181	8	10	gnl|CDD|153339	cd07655, F-BAR_PACSIN, The I	ref|XP_003427062.1|	PREDICTED: protein kinase C and casein kinase substrate in neurons protein 2-like isoform 2 [Nasonia vitripennis]	1,00E-166
gALL_v3_c12182	4	6	-	-	ref|XP_003758196.1|	PREDICTED: protein transport protein Sec23B [Sarcophilus harrisii]	8,00E-07
gALL_v3_c12183	1	6	-	-	-	-	-
gALL_v3_c12184	10	11	-	-	-	-	-
gALL_v3_c12185	0	5	-	-	-	-	-
gALL_v3_c12186	1	6	-	-	gb|EGI60794.1|	RNA-binding protein pno1 [Acromyrmex echinatior]	1,00E-05
gALL_v3_c12188	2	8	gnl|CDD|128559	smart00263, LYZ1, Alpha-lacta	gb|AFI81522.1|	c-1 lysozyme [Periplaneta americana]	2,00E-54
gALL_v3_c12189	1	6	gnl|CDD|189365	pfam00053, Laminin_EGF, Lam	ref|XP_002430825.1|	laminin A chain, putative [Pediculus humanus corporis] gb|EEB18087.1| laminin A chain, putative [Pediculus humanus corporis]	1,00E-108
gALL_v3_c1219	6	8	-	-	-	-	-
gALL_v3_c12191	6	6	-	-	-	-	-
gALL_v3_c12192	3	4	gnl|CDD|162469	TIGR01657, P-ATPase-V, P-type	ref|XP_003694407.1|	PREDICTED: probable cation-transporting ATPase 13A3-like [Apis florea]	1,00E-84
gALL_v3_c12193	3	5	gnl|CDD|128466	smart00164, TBC, Domain in T	gb|EFN78563.1|	TBC1 domain family member 15 [Harpegnathos saltator]	1,00E-128
gALL_v3_c12194	5	8	gnl|CDD|31695	COG1506, DAP2, Dipeptidyl ar	ref|XP_971949.1|	PREDICTED: similar to AGAP003138-PA [Tribolium castaneum]	0
gALL_v3_c12195	8	9	-	-	gb|EFA13373.1|	hypothetical protein TcasGA2_TC001390 [Tribolium castaneum]	2,00E-31
gALL_v3_c12196	6	8	-	-	-	-	-
gALL_v3_c12197	4	5	-	-	-	-	-
gALL_v3_c12198	4	10	gnl|CDD|190132	pfam01841, Transglut_core, Tr	ref|XP_003455294.1|	PREDICTED: peptide-N(4)-(N-acetyl-beta-glucosaminyl)asparagine amidase-like [Oreochromis niloticus]	1,00E-155
gALL_v3_c122	19	27	gnl|CDD|189799	pfam01007, IRK, Inward rectifi	ref|XP_006611032.1|	PREDICTED: inward rectifier potassium channel 4-like isoform X11 [Apis dorsata]	0
gALL_v3_c12200	3	5	gnl|CDD|29332	cd00257, Fascin, Fascin-like dc	gb|AEW67354.1|	carbohydrate binding module, partial [Coptotermes formosanus]	5,00E-20
gALL_v3_c12201	8	4	-	-	ref|XP_004757623.1|	PREDICTED: protein FAM188A isoform X2 [Mustela putorius furo] ref|XP_004800292.1| PREDICTED: protein FAM188A isoform X2 [Mustela putorius furo]	4,00E-72
gALL_v3_c12202	0	6	gnl|CDD|191046	pfam04615, Utp14, Utp14 pro	ref|XP_002430325.1|	Caldesmon, putative [Pediculus humanus corporis] gb|EEB17587.1| Caldesmon, putative [Pediculus humanus corporis]	6,00E-38
gALL_v3_c12204	0	5	-	-	-	-	-
gALL_v3_c12206	1	4	-	-	-	-	-
gALL_v3_c12207	1	3	gnl|CDD|144648	pfam01130, CD36, CD36 famil	sp|Q9GPH7.1|SNMP1_I	RecName: Full=Sensory neuron membrane protein 1; Short=SNMP1-Msex gb|AAG49366.1|AF323589_1 sensory neuron membrane protein 1 [Manduca sexta]	6,00E-27
gALL_v3_c12208	4	12	-	-	-	-	-
gALL_v3_c12209	4	4	-	-	-	-	-
gALL_v3_c1221	7	4	-	-	-	-	-
gALL_v3_c12210	5	3	-	-	gb|EFN67982.1|	Protein strawberry notch [Camponotus floridanus]	1,00E-124
gALL_v3_c12211	5	2	-	-	ref|XP_006614519.1|	PREDICTED: uncharacterized protein LOC102679013 isoform X2 [Apis dorsata]	2,00E-08
gALL_v3_c12212	2	5	-	-	-	-	-
gALL_v3_c12213	7	13	gnl|CDD|145211	pfam01920, Prefoldin_2, Prefo	gb|AGM32804.1|	prefoldin subunit 4-like protein [Coptotermes formosanus]	9,00E-68
gALL_v3_c12214	3	3	-	-	dbj|BAB86899.1|	phospholipase A2 inhibitor [Elaphe quadrivirgata]	5,00E-08
gALL_v3_c12215	10	6	gnl|CDD|185514	PTZ00212, PTZ00212, T-compl	gb|ABG01855.1|	putative accessory gland protein [Gryllus pennsylvanicus]	0
gALL_v3_c12218	5	6	-	-	-	-	-
gALL_v3_c12219	2	10	-	-	-	-	-
gALL_v3_c1222	17	20	-	-	-	-	-
gALL_v3_c12220	5	6	-	-	-	-	-
gALL_v3_c12221	4	10	-	-	-	-	-
gALL_v3_c12223	8	8	gnl|CDD|178012	PLN02387, PLN02387, long-ch	gb|AAK93475.1|	LP07340p [Drosophila melanogaster]	3,00E-76
gALL_v3_c12224	4	6	-	-	-	-	-
gALL_v3_c12225	5	2	-	-	ref|XP_003707101.1|	PREDICTED: cell division cycle and apoptosis regulator protein 1-like [Megachile rotundata]	1,00E-12
gALL_v3_c12227	4	7	-	-	gb|AGM32707.1|	hypothetical protein, partial [Coptotermes formosanus]	2,00E-14
gALL_v3_c12228	11	9	gnl|CDD|146377	pfam03712, Cu2_monoox_C, C	ref|XP_308418.4|	AGAP007420-PA [Anopheles gambiae str. PEST] gb|EAA04612.4| AGAP007420-PA [Anopheles gambiae str. PEST]	1,00E-154
gALL_v3_c12229	1	6	-	-	-	-	-
gALL_v3_c1223	23	16	gnl|CDD|128336	smart00020, Tryp_SPc, Trypsin	ref|XP_002433014.1|	hypothetical protein Phum_PHUM609370 [Pediculus humanus corporis] gb|EEB20276.1| hypothetical protein Phum_PHUM609370 [Pediculus humanus corporis]	2,00E-65
gALL_v3_c12230	3	3	-	-	-	-	-
gALL_v3_c12231	0	4	gnl|CDD|193272	pfam12796, Ank_2, Ankyrin re	ref|XP_006613851.1|	PREDICTED: ankyrin repeat and sterile alpha motif domain-containing protein 1B-like isoform X1 [Apis dorsata] ref|XP_006613852.1| PREDICTED: ankyrin repeat and sterile	6,00E-64
gALL_v3_c12232	6	13	-	-	gb|EFN88555.1|	Protein extra bases [Harpegnathos saltator]	1,00E-100
gALL_v3_c12233	3	2	-	-	ref|XP_002426914.1|	conserved hypothetical protein [Pediculus humanus corporis] gb|EEB14176.1| conserved hypothetical protein [Pediculus humanus corporis]	4,00E-54
gALL_v3_c12237	3	3	-	-	-	-	-
gALL_v3_c12238	4	3	-	-	-	-	-
gALL_v3_c12239	4	3	-	-	gb|EFN84335.1|	Nucleosome-remodeling factor subunit NURF301 [Harpegnathos saltator]	4,00E-14
gALL_v3_c1224	10	10	gnl|CDD|29840	cd00584, Prefoldin_alpha, Pre	dbj|BAM18170.1|	prefoldin subunit [Papilio xuthus]	2,00E-24
gALL_v3_c12240	6	7	-	-	-	-	-
gALL_v3_c12241	2	9	-	-	-	-	-
gALL_v3_c12242	1	6	-	-	-	-	-
gALL_v3_c12243	1	4	-	-	-	-	-
gALL_v3_c12245	4	2	-	-	-	-	-
gALL_v3_c12246	6	5	-	-	gb|EFN67634.1|	Putative uncharacterized transposon-derived protein F52C9.6 [Camponotus floridanus]	4,00E-07
gALL_v3_c12247	2	5	-	-	gb|AEE61651.1|	unknown [Dendroctonus ponderosae] gb|ENN70944.1| hypothetical protein YQE_12346, partial [Dendroctonus ponderosae] gb|ERL88184.1| hypothetical protein D910_(	3,00E-50
gALL_v3_c12248	2	6	-	-	ref|XP_003741356.1|	PREDICTED: uncharacterized protein LOC100901629 [Metaseiulus occidentalis]	2,00E-08
gALL_v3_c12249	4	2	-	-	-	-	-
gALL_v3_c1225	8	10	-	-	-	-	-
gALL_v3_c12250	7	5	-	-	-	-	-
gALL_v3_c12251	3	1	-	-	gb|AGM32739.1|	Sec14p-like lipid-binding domain containing protein, partial [Coptotermes formosanus]	1,00E-88
gALL_v3_c12253	4	13	gnl|CDD|189605	pfam00566, TBC, TBC domain.	ref|XP_973356.1|	PREDICTED: similar to CG4552 CG4552-PA [Tribolium castaneum] gb|EFA02068.1| hypothetical protein TcasGA2_TC007702 [Tribolium castaneum]	2,00E-94
gALL_v3_c12254	5	3	-	-	-	-	-
gALL_v3_c12255	4	4	-	-	ref|XP_003488412.1|	PREDICTED: hypothetical protein LOC100740015 [Bombus impatiens]	1,00E-39
gALL_v3_c12256	2	4	-	-	-	-	-
gALL_v3_c12257	3	3	-	-	-	-	-
gALL_v3_c12258	4	3	-	-	-	-	-
gALL_v3_c12259	1	5	-	-	-	-	-
gALL_v3_c1226	7	5	gnl|CDD|190614	pfam03366, YEATS, YEATS fam	ref|XP_002427250.1|	Neurofilament triplet M protein, putative [Pediculus humanus corporis] gb|EEB14512.1| Neurofilament triplet M protein, putative [Pediculus humanus corporis]	2,00E-51
gALL_v3_c12260	4	2	-	-	-	-	-
gALL_v3_c12263	2	6	-	-	ref|XP_006610745.1|	PREDICTED: uncharacterized protein LOC102676766 [Apis dorsata]	3,00E-10
gALL_v3_c12264	9	19	-	-	ref|XP_003425471.1|	PREDICTED: hypothetical protein LOC100679661 [Nasonia vitripennis]	2,00E-79
gALL_v3_c12265	4	7	-	-	-	-	-
gALL_v3_c12266	5	8	gnl|CDD|179675	PRK03918, PRK03918, chromo	-	-	-

gALL_v3_c12267	4	7	gnl|CDD|34810	COG5213, FIP1, Polyadenylatic	gb|EFN87216.1|	Pre-mRNA 3'-end-processing factor FIP1 [Harpegnathos saltator]	5,00E-85
gALL_v3_c12269	20	19	-	-	-	-	-
gALL_v3_c1227	5	6	-	-	-	-	-
gALL_v3_c12270	12	8	-	-	ref|XP_004332100.1|	PREDICTED: craniofacial development protein 2-like, partial [Tursiops truncatus]	2,00E-12
gALL_v3_c12272	4	5	-	-	gb|EGI65165.1|	Protein purity of essence [Acromyrmex echinatior]	7,00E-17
gALL_v3_c12273	4	5	-	-	-	-	-
gALL_v3_c12274	5	2	-	-	-	-	-
gALL_v3_c12275	5	3	-	-	gb|EFN65619.1|	Putative uncharacterized transposon-derived protein F52C9.6 [Camponotus floridanus]	5,00E-14
gALL_v3_c12277	1	5	-	-	ref|XP_003702902.1|	PREDICTED: tyrosine-protein phosphatase Lar-like [Megachile rotundata]	1,00E-92
gALL_v3_c12278	3	8	-	-	-	-	-
gALL_v3_c12279	3	4	gnl|CDD|147767	pfam05793, TFIIF_alpha, Trans	-	-	-
gALL_v3_c12280	3	2	-	-	ref|XP_003395464.1|	PREDICTED: hypothetical protein LOC100649328 [Bombus terrestris]	4,00E-68
gALL_v3_c12281	3	4	gnl|CDD|111146	pfam02218, HS1_rep, Repeat i	gb|ENN75707.1|	hypothetical protein YQE_07668, partial [Dendroctonus ponderosae] gb|ERL87702.1| hypothetical protein D910_05092 [Dendroctonus ponderosae]	7,00E-72
gALL_v3_c12282	6	9	-	-	-	-	-
gALL_v3_c12283	4	3	gnl|CDD|192134	pfam08718, GLTP, Glycolipid tr	gb|ETN59424.1|	hypothetical protein AND_008979 [Anopheles darlingi]	6,00E-21
gALL_v3_c12284	6	6	-	-	-	-	-
gALL_v3_c12285	2	2	-	-	-	-	-
gALL_v3_c12286	3	7	gnl|CDD|173623	cd00180, PKc, Catalytic domai	gb|EFZ09799.1|	hypothetical protein SINV_11132 [Solenopsis invicta]	2,00E-91
gALL_v3_c12287	4	4	gnl|CDD|132942	cd06611, STKc_SLK_like, Catal	ref|XP_003706003.1|	PREDICTED: uncharacterized protein LOC100883185 isoform 2 [Megachile rotundata]	7,00E-66
gALL_v3_c12288	3	3	-	-	-	-	-
gALL_v3_c12289	5	6	-	-	-	-	-
gALL_v3_c1229	6	4	gnl|CDD|189855	pfam01139, UPF0027, Unchar	ref|XP_002431746.1|	conserved hypothetical protein [Pediculus humanus corporis] gb|EEB19008.1| conserved hypothetical protein [Pediculus humanus corporis]	0
gALL_v3_c12290	3	9	-	-	ref|XP_002426599.1|	hypothetical protein Phum_PHUM262060 [Pediculus humanus corporis] gb|EEB13861.1| hypothetical protein Phum_PHUM262060 [Pediculus humanus corporis]	2,00E-15
gALL_v3_c12291	1	4	-	-	-	-	-
gALL_v3_c12292	2	4	gnl|CDD|190008	pfam01490, Aa_trans, Transm	ref|XP_003427558.1|	PREDICTED: proton-coupled amino acid transporter 1-like [Nasonia vitripennis]	7,00E-92
gALL_v3_c12294	3	2	-	-	-	-	-
gALL_v3_c12295	10	8	gnl|CDD|148329	pfam06662, C5-epim_C, D-glu	ref|XP_974487.1|	PREDICTED: similar to CG3194 CG3194-PA [Tribolium castaneum] gb|EFA10381.1| hypothetical protein TcasGA2_TC012612 [Tribolium castaneum]	0
gALL_v3_c12296	1	7	-	-	ref|XP_002432772.1|	Soluble NSF attachment protein, putative [Pediculus humanus corporis] gb|EEB20034.1| Soluble NSF attachment protein, putative [Pediculus humanus corporis]	4,00E-79
gALL_v3_c12297	7	8	-	-	-	-	-
gALL_v3_c12298	2	3	-	-	ref|XP_006610134.1|	PREDICTED: protein FAM8A1-like isoform X2 [Apis dorsata]	3,00E-56
gALL_v3_c12299	3	10	-	-	-	-	-
gALL_v3_c123	8	7	-	-	-	-	-
gALL_v3_c1230	9	10	-	-	ref|XP_003699236.1|	PREDICTED: transcription elongation factor SPT6-like [Megachile rotundata]	1,00E-112
gALL_v3_c12300	11	10	gnl|CDD|48119	cd03192, GST_C_Sigma_like, (	gb|AAB72147.1|	allergen Bla g 5, partial [Blattella germanica]	1,00E-30
gALL_v3_c12301	4	7	-	-	ref|XP_001846854.1|	oxysterol binding protein [Culex quinquefasciatus] gb|EDS44823.1| oxysterol binding protein [Culex quinquefasciatus]	5,00E-26
gALL_v3_c12303	2	5	gnl|CDD|189779	pfam00940, RNA_pol, DNA-de	ref|XP_001946877.1|	PREDICTED: DNA-directed RNA polymerase, mitochondrial-like [Acyrthosiphon pisum]	1,00E-124
gALL_v3_c12304	2	3	-	-	-	-	-
gALL_v3_c12305	4	5	gnl|CDD|128829	smart00557, IG_FLMN, Filamii	ref|XP_003398538.1|	PREDICTED: filamin-C-like [Bombus terrestris]	1,00E-104
gALL_v3_c12306	1	3	-	-	-	-	-
gALL_v3_c12307	5	4	gnl|CDD|188087	TIGR00841, bass, bile acid trar	ref|XP_001662576.1|	sodium-bile acid cotransporter [Aedes aegypti] gb|EAT35385.1| AAEL012440-PA [Aedes aegypti]	2,00E-58
gALL_v3_c12308	5	8	gnl|CDD|34857	COG5260, TRF4, DNA polymer	ref|XP_006824330.1|	PREDICTED: terminal uridylyltransferase 4-like [Saccoglossus kowalevskii]	9,00E-62
gALL_v3_c12309	1	4	-	-	-	-	-
gALL_v3_c1231	7	6	gnl|CDD|178012	PLN02387, PLN02387, long-ch	ref|XP_006613057.1|	PREDICTED: long-chain-fatty-acid--CoA ligase 3-like isoform X4 [Apis dorsata] ref|XP_006613058.1| PREDICTED: long-chain-fatty-acid--CoA ligase 3-like isoform X5 [Apis dor	1,00E-104
gALL_v3_c12310	4	3	gnl|CDD|178272	PLN02667, PLN02667, inositol	ref|XP_003701339.1|	PREDICTED: inositol polyphosphate multikinase-like [Megachile rotundata]	2,00E-47
gALL_v3_c12312	5	2	-	-	-	-	-
gALL_v3_c12314	5	2	-	-	-	-	-
gALL_v3_c12315	4	5	-	-	-	-	-
gALL_v3_c12317	2	6	-	-	ref|XP_003708199.1|	PREDICTED: protein purity of essence-like [Megachile rotundata]	5,00E-39
gALL_v3_c12318	5	6	-	-	ref|XP_002718425.1|	PREDICTED: tigger transposable element derived 6-like [Oryctolagus cuniculus]	3,00E-11
gALL_v3_c12319	2	7	-	-	-	-	-
gALL_v3_c1232	11	10	gnl|CDD|133186	cd05055, PTKc_PDGFR, Catal	ref|XP_004521817.1|	PREDICTED: vascular endothelial growth factor receptor 1-like isoform X5 [Ceratitis capitata]	3,00E-89
gALL_v3_c12320	0	6	-	-	-	-	-
gALL_v3_c12321	3	6	gnl|CDD|29138	cd00176, SPEC, Spectrin repea	ref|XP_003243074.1|	PREDICTED: triple functional domain protein-like isoform 3 [Acyrthosiphon pisum]	1,00E-139
gALL_v3_c12322	3	2	-	-	gb|EKV16106.1|	Retrotransposon polyprotein, putative [Penicillium digitatum Pd1]	2,00E-06
gALL_v3_c12324	3	5	-	-	-	-	-
gALL_v3_c12325	1	5	gnl|CDD|143459	cd07141, ALDH_F1AB_F2_RAL	gb|ENN76528.1|	hypothetical protein YQE_06979, partial [Dendroctonus ponderosae]	1,00E-16
gALL_v3_c12326	1	6	-	-	-	-	-
gALL_v3_c12327	6	8	-	-	-	-	-
gALL_v3_c12328	6	4	-	-	ref|XP_001654178.1|	cell cycle progression [Aedes aegypti] gb|EAT38022.1| AAEL010051-PA [Aedes aegypti]	3,00E-54
gALL_v3_c12329	4	5	-	-	gb|EFN67634.1|	Putative uncharacterized transposon-derived protein F52C9.6 [Camponotus floridanus]	8,00E-08
gALL_v3_c1233	12	12	gnl|CDD|128578	smart00282, LamG, Laminin G	gb|EFX90086.1|	hypothetical protein DAPPUDRAFT_186952 [Daphnia pulex]	1,00E-113
gALL_v3_c12330	3	6	-	-	-	-	-
gALL_v3_c12331	4	8	-	-	gb|AFH78148.1|	cytochrome P450 [Coptotermes formosanus]	2,00E-44
gALL_v3_c12332	2	3	-	-	-	-	-
gALL_v3_c12334	10	11	-	-	gb|EFN75248.1|	PiggyBac transposable element-derived protein 4 [Harpegnathos saltator]	7,00E-09
gALL_v3_c12335	1	5	-	-	-	-	-
gALL_v3_c12336	1	5	gnl|CDD|110027	pfam00996, GDI, GDP dissocia	ref|XP_002433122.1|	Rab GDP dissociation inhibitor alpha, putative [Pediculus humanus corporis] gb|EEB20384.1| Rab GDP dissociation inhibitor alpha, putative [Pediculus humanus corporis]	1,00E-31
gALL_v3_c12337	5	3	-	-	-	-	-
gALL_v3_c12339	1	5	gnl|CDD|153252	cd07990, LPLAT_LCLAT1-like, L	ref|XP_002428133.1|	1-acylglycerol-3-phosphate acyltransferase, putative [Pediculus humanus corporis] gb|EEB15395.1| 1-acylglycerol-3-phosphate acyltransferase, putative [Pediculus human	5,00E-29
gALL_v3_c1234	5	13	-	-	-	-	-
gALL_v3_c12340	1	5	-	-	-	-	-
gALL_v3_c12341	3	5	gnl|CDD|29257	cd00200, WD40, WD40 domai	ref|XP_002425236.1|	serine-threonine kinase receptor-associated protein, putative [Pediculus humanus corporis] gb|EEB12498.1| serine-threonine kinase receptor-associated protein, putative	1,00E-42
gALL_v3_c12342	4	5	gnl|CDD|115363	pfam06701, MIB_HERC2, Mib_	ref|XP_002432470.1|	mind bomb, putative [Pediculus humanus corporis] gb|EEB19732.1| mind bomb, putative [Pediculus humanus corporis]	1,00E-174
gALL_v3_c12343	3	9	-	-	-	-	-
gALL_v3_c12344	2	4	-	-	gb|EFA12027.1|	hypothetical protein TcasGA2_TC001442 [Tribolium castaneum]	9,00E-31
gALL_v3_c12345	4	3	gnl|CDD|34849	COG5252, COG5252, Uncharac	ref|NP_001191866.1|	zinc finger CCCH domain-containing protein 15 [Acyrthosiphon pisum]	5,00E-40
gALL_v3_c12346	2	4	-	-	-	-	-
gALL_v3_c12347	2	5	-	-	ref|XP_001602270.2|	PREDICTED: sister chromatid cohesion protein PDS5 homolog B-A isoform 1 [Nasonia vitripennis]	2,00E-37
gALL_v3_c12348	3	4	-	-	gb|AGM32626.1|	glucosyl/glucuronosyl transferase, partial [Coptotermes formosanus]	1,00E-100
gALL_v3_c12349	4	2	-	-	-	-	-
gALL_v3_c12350	5	12	-	-	ref|XP_005111750.1|	PREDICTED: piggyBac transposable element-derived protein 4-like [Aplysia californica]	6,00E-36
gALL_v3_c12352	2	9	gnl|CDD|128505	smart00209, TSP1, Thrombosp	ref|XP_005653807.1|	PREDICTED: hemicentin-1-like [Sus scrofa]	7,00E-98
gALL_v3_c12353	5	6	gnl|CDD|192730	pfam11262, Tho2, Transcriptic	gb|EFN65937.1|	THO complex subunit 2 [Camponotus floridanus]	1,00E-113
gALL_v3_c12355	4	8	gnl|CDD|153093	cd04704, PLA2_bee_venom_li	gb|EFZ16394.1|	hypothetical protein SINV_12161 [Solenopsis invicta]	4,00E-60
gALL_v3_c12356	2	7	-	-	-	-	-
gALL_v3_c12357	1	5	-	-	ref|XP_002427070.1|	Rho-GTPase-activating protein, putative [Pediculus humanus corporis] gb|EEB14332.1| Rho-GTPase-activating protein, putative [Pediculus humanus corporis]	9,00E-61
gALL_v3_c12358	3	3	-	-	-	-	-
gALL_v3_c1236	5	5	-	-	-	-	-
gALL_v3_c12360	2	4	-	-	-	-	-
gALL_v3_c12361	3	4	-	-	-	-	-
gALL_v3_c12364	3	5	-	-	ref|XP_006608948.1|	PREDICTED: type II inositol 1,4,5-trisphosphate 5-phosphatase-like isoform X4 [Apis dorsata]	5,00E-39
gALL_v3_c12366	9	10	gnl|CDD|183675	PRK12678, PRK12678, transcri	-	-	-
gALL_v3_c12367	3	6	-	-	-	-	-
gALL_v3_c12368	7	4	gnl|CDD|190114	pfam01794, Ferric_reduct, Fer	ref|XP_971837.2|	PREDICTED: similar to Dual oxidase CG3131-PA [Tribolium castaneum]	1,00E-118
gALL_v3_c12369	4	5	-	-	-	-	-
gALL_v3_c1237	5	7	-	-	ref|XP_002427399.1|	FYVE-containing protein, putative [Pediculus humanus corporis] gb|EEB14661.1| FYVE-containing protein, putative [Pediculus humanus corporis]	2,00E-36
gALL_v3_c12370	4	3	-	-	gb|EFB22320.1|	hypothetical protein PANDA_013309 [Ailuropoda melanoleuca]	6,00E-08
gALL_v3_c12371	4	5	-	-	-	-	-
gALL_v3_c12372	3	9	gnl|CDD|193473	pfam13000, Acatn, Acetyl-coe	ref|XP_004932099.1|	PREDICTED: acetyl-coenzyme A transporter 1-like isoform X1 [Bombyx mori] ref|XP_004932100.1| PREDICTED: acetyl-coenzyme A transporter 1-like isoform X2 [Bombyx n	1,00E-34
gALL_v3_c12373	2	10	-	-	-	-	-
gALL_v3_c12374	6	5	-	-	-	-	-
gALL_v3_c12375	1	7	-	-	-	-	-
gALL_v3_c12376	4	4	-	-	-	-	-
gALL_v3_c12377	4	8	-	-	-	-	-
gALL_v3_c12378	2	3	-	-	-	-	-
gALL_v3_c12379	3	11	-	-	-	-	-
gALL_v3_c1238	10	7	gnl|CDD|185474	PTZ00141, PTZ00141, elongati	dbj|BAM14416.1|	elongation factor-1 alpha [Spirotrichonympha leidyi]	0
gALL_v3_c12380	2	4	-	-	ref|XP_005112861.1|	PREDICTED: uncharacterized protein LOC101855698 [Aplysia californica]	3,00E-17
gALL_v3_c12381	4	7	-	-	-	-	-
gALL_v3_c12382	4	2	-	-	-	-	-
gALL_v3_c12383	4	7	-	-	ref|XP_003700192.1|	PREDICTED: spermatogenesis-associated protein 13-like [Megachile rotundata]	2,00E-15
gALL_v3_c12384	8	5	-	-	-	-	-
gALL_v3_c12385	2	7	gnl|CDD|144972	pfam01576, Myosin_tail_1, M	ref|XP_001952092.2|	PREDICTED: myosin heavy chain, muscle isoform 1 [Acyrthosiphon pisum]	2,00E-13
gALL_v3_c12387	8	7	-	-	-	-	-
gALL_v3_c12388	3	1	-	-	ref|WP_006565509.1|	hypothetical protein, partial [Anaerostipes caccae] gb|EDR96157.1| hypothetical protein ANACAC_03315, partial [Anaerostipes caccae DSM 14662]	2,00E-44
gALL_v3_c12389	0	5	-	-	-	-	-
gALL_v3_c1239	9	23	gnl|CDD|128688	smart00410, IG_like, Immuno	ref|XP_002430407.1|	Neuroglian precursor, putative [Pediculus humanus corporis] gb|EEB17669.1| Neuroglian precursor, putative [Pediculus humanus corporis]	0
gALL_v3_c12390	1	4	-	-	-	-	-
gALL_v3_c12391	2	9	-	-	-	-	-
gALL_v3_c12392	6	11	gnl|CDD|129108	smart00875, BACK, BTB And C	ref|XP_003485593.1|	PREDICTED: BTB/POZ domain-containing protein 17-like [Bombus impatiens]	1,00E-110
gALL_v3_c12393	10	21	-	-	ref|XP_970833.2|	PREDICTED: similar to conserved hypothetical protein [Tribolium castaneum]	3,00E-84
gALL_v3_c12394	2	5	gnl|CDD|73156	cd01650, RT_nLTR_like, RT_nL	ref|XP_003727690.1|	PREDICTED: RNA-directed DNA polymerase from mobile element jockey-like [Strongylocentrotus purpuratus]	2,00E-19
gALL_v3_c12395	2	6	-	-	gb|EFN66625.1|	Baculoviral IAP repeat-containing protein 6 [Camponotus floridanus]	7,00E-50
gALL_v3_c12396	3	3	-	-	-	-	-
gALL_v3_c12398	5	5	-	-	-	-	-
gALL_v3_c1240	11	17	-	-	-	-	-
gALL_v3_c12400	4	5	gnl|CDD|99896	cd05835, Dnmt3b_related, Th	gb|EFZ09184.1|	hypothetical protein SINV_03643 [Solenopsis invicta]	2,00E-33
gALL_v3_c12401	4	5	gnl|CDD|188098	TIGR00957, MRP_assoc_pro, r	ref|XP_006608256.1|	PREDICTED: multidrug resistance-associated protein 4-like [Apis dorsata]	6,00E-52
gALL_v3_c12403	11	8	-	-	ref|XP_002403127.1|	sulfide:quinone oxidoreductase/flavo-binding protein, putative [Ixodes scapularis] gb|EEC14485.1| sulfide:quinone oxidoreductase/flavo-binding protein, putative [Ixodes	2,00E-79
gALL_v3_c12404	5	3	-	-	-	-	-
gALL_v3_c12405	1	4	-	-	-	-	-
gALL_v3_c12406	5	2	-	-	-	-	-
gALL_v3_c12407	3	5	-	-	-	-	-
gALL_v3_c12408	0	6	gnl|CDD|162066	TIGR00845, caca, sodium/calc	ref|XP_001847399.1|	sodium/calcium exchanger [Culex quinquefasciatus] gb|EDS26132.1| sodium/calcium exchanger [Culex quinquefasciatus]	1,00E-122
gALL_v3_c12409	4	5	-	-	-	-	-
gALL_v3_c1241	4	9	-	-	ref|XP_001810409.1|	PREDICTED: similar to exocyst complex 84-kDa subunit [Tribolium castaneum]	9,00E-30
gALL_v3_c12411	5	4	gnl|CDD|31389	COG1196, Smc, Chromosome	ref|XP_974572.2|	PREDICTED: similar to viral A-type inclusion protein, putative [Tribolium castaneum]	2,00E-44
gALL_v3_c12412	6	3	-	-	-	-	-
gALL_v3_c12413	17	13	-	-	ref|XP_002424329.1|	eukaryotic translation initiation factor 5A, putative [Pediculus humanus corporis] gb|EEB11591.1| eukaryotic translation initiation factor 5A, putative [Pediculus humanus (	2,00E-16
gALL_v3_c12414	3	4	-	-	-	-	-
gALL_v3_c12416	4	2	-	-	-	-	-
gALL_v3_c12417	5	7	gnl|CDD|146336	pfam03643, Vps26, Vacuolar p	gb|EFN69227.1|	Vacuolar protein sorting-associated protein 26 [Camponotus floridanus]	1,00E-125
gALL_v3_c12418	3	3	gnl|CDD|34870	COG5273, COG5273, Uncharac	ref|XP_006561438.1|	PREDICTED: palmitoyltransferase ZDHHC15 isoform X8 [Apis mellifera] ref|XP_006620547.1| PREDICTED: palmitoyltransferase ZDHHC15-like isoform X9 [Apis dorsata]	4,00E-95
gALL_v3_c12419	4	4	-	-	-	-	-

gALL_v3_c1242	12	10	-	-	gb|EFN75801.1|	Histone deacetylase 4 [Harpegnathos saltator]	1,00E-138
gALL_v3_c12420	4	3	-	-	-	-	-
gALL_v3_c12421	3	4	-	-	-	-	-
gALL_v3_c12422	3	2	-	-	-	-	-
gALL_v3_c12423	7	4	-	-	-	-	-
gALL_v3_c12424	12	14	gnl|CDD|180385	PRK06075, PRK06075, NADH c	gb|ETN63841.1|	NADH-ubiquinone oxidoreductase fe-s protein 2 (ndufs2) [Anopheles darlingi]	0
gALL_v3_c12425	8	5	-	-	ref|XP_003427127.1|	PREDICTED: hypothetical protein LOC100120957 isoform 2 [Nasonia vitripennis]	1,00E-135
gALL_v3_c12426	3	2	-	-	-	-	-
gALL_v3_c12427	2	3	-	-	-	-	-
gALL_v3_c12428	5	12	-	-	-	-	-
gALL_v3_c1243	5	8	-	-	ref|XP_969770.1|	PREDICTED: similar to jing CG9397-PG [Tribolium castaneum] gb|EFA02114.1| hypothetical protein TcasGA2_TC007760 [Tribolium castaneum]	7,00E-91
gALL_v3_c12430	6	7	gnl|CDD|175987	cd04020, C2B_SLP_1-2-3-4, C2	gb|EEZ98554.1|	hypothetical protein TcasGA2_TC001061 [Tribolium castaneum]	7,00E-12
gALL_v3_c12431	4	4	-	-	-	-	-
gALL_v3_c12432	5	6	gnl|CDD|133313	cd04113, Rab4, Rab4 subfamil	gb|ACH92482.1|	FI09101p [Drosophila melanogaster]	1,00E-123
gALL_v3_c12433	2	7	-	-	-	-	-
gALL_v3_c12434	3	5	-	-	-	-	-
gALL_v3_c12435	7	8	-	-	-	-	-
gALL_v3_c12436	5	9	-	-	ref|XP_002423795.1|	conserved hypothetical protein [Pediculus humanus corporis] gb|EEB11057.1| conserved hypothetical protein [Pediculus humanus corporis]	1,00E-53
gALL_v3_c12437	2	5	-	-	-	-	-
gALL_v3_c12438	6	6	-	-	ref|XP_004536372.1|	PREDICTED: 39S ribosomal protein L3, mitochondrial-like [Ceratitis capitata]	2,00E-62
gALL_v3_c12440	3	5	gnl|CDD|147578	pfam05470, eIF-3c_N, Eukaryo	ref|XP_003701177.1|	PREDICTED: eukaryotic translation initiation factor 3 subunit C-like [Megachile rotundata]	1,00E-31
gALL_v3_c12441	4	5	-	-	-	-	-
gALL_v3_c12442	4	3	-	-	-	-	-
gALL_v3_c12445	2	4	-	-	-	-	-
gALL_v3_c12446	5	8	gnl|CDD|58171	cd03505, Delta9-FADS-like, Th	gb|EFZ14309.1|	hypothetical protein SINV_09878 [Solenopsis invicta]	1,00E-83
gALL_v3_c12447	3	2	-	-	-	-	-
gALL_v3_c12448	3	1	-	-	-	-	-
gALL_v3_c12449	1	4	-	-	-	-	-
gALL_v3_c1245	5	7	-	-	-	-	-
gALL_v3_c12450	1	6	-	-	-	-	-
gALL_v3_c12452	2	3	-	-	-	-	-
gALL_v3_c12453	3	3	-	-	-	-	-
gALL_v3_c12455	6	4	-	-	-	-	-
gALL_v3_c12456	2	7	-	-	-	-	-
gALL_v3_c12457	0	5	-	-	gb|EFA11915.1|	hypothetical protein TcasGA2_TC004072 [Tribolium castaneum]	3,00E-06
gALL_v3_c12458	6	8	-	-	ref|XP_002423897.1|	conserved hypothetical protein [Pediculus humanus corporis] gb|EEB11159.1| conserved hypothetical protein [Pediculus humanus corporis]	1,00E-25
gALL_v3_c12459	2	5	-	-	ref|XP_001508506.2|	PREDICTED: jerky protein-like [Ornithorhynchus anatinus]	2,00E-12
gALL_v3_c1246	4	8	gnl|CDD|34862	COG5265, ATM1, ABC-type tra	ref|XP_003400902.1|	PREDICTED: ATP-binding cassette sub-family B member 6, mitochondrial-like [Bombus terrestris]	1,00E-137
gALL_v3_c12460	23	22	gnl|CDD|132717	cd07061, HP_HAP_like, Histidi	ref|XP_002429332.1|	multiple inositol polyphosphate phosphatase 1 precursor, putative [Pediculus humanus corporis] gb|EEB16594.1| multiple inositol polyphosphate phosphatase 1 precurso	6,00E-80
gALL_v3_c12461	3	4	-	-	gb|AAB92393.1|	reverse transcriptase [Drosophila subobscura]	2,00E-22
gALL_v3_c12463	4	3	gnl|CDD|150427	pfam09752, DUF2048, Unchar	ref|XP_001843583.1|	conserved hypothetical protein [Culex quinquefasciatus] gb|EDS33226.1| conserved hypothetical protein [Culex quinquefasciatus]	8,00E-42
gALL_v3_c12464	5	12	gnl|CDD|145874	pfam02949, 7tm_6, 7tm Odor	gb|EEZ99415.1|	odorant receptor 60 [Tribolium castaneum]	2,00E-14
gALL_v3_c12465	3	5	gnl|CDD|192013	pfam08356, EF_assoc_2, EF ha	ref|XP_001842184.1|	conserved hypothetical protein [Culex quinquefasciatus] gb|EDS39689.1| conserved hypothetical protein [Culex quinquefasciatus]	6,00E-73
gALL_v3_c12466	7	17	-	-	-	-	-
gALL_v3_c12468	1	5	-	-	-	-	-
gALL_v3_c12469	3	5	-	-	-	-	-
gALL_v3_c1247	22	39	-	-	gb|EGI59549.1|	Remodeling and spacing factor 1 [Acromyrmex echinatior]	6,00E-65
gALL_v3_c12470	4	3	-	-	-	-	-
gALL_v3_c12471	6	10	-	-	ref|XP_003393192.1|	PREDICTED: hypothetical protein LOC100643962 [Bombus terrestris]	4,00E-59
gALL_v3_c12472	2	3	-	-	-	-	-
gALL_v3_c12474	4	3	gnl|CDD|48213	cd03401, Band_7_prohibitin, I	ref|XP_001600011.2|	PREDICTED: prohibitin-2 [Nasonia vitripennis]	7,00E-59
gALL_v3_c12475	2	3	-	-	-	-	-
gALL_v3_c12476	6	7	gnl|CDD|191320	pfam05622, HOOK, HOOK pro	ref|XP_002427063.1|	conserved hypothetical protein [Pediculus humanus corporis] gb|EEB14325.1| conserved hypothetical protein [Pediculus humanus corporis]	1,00E-130
gALL_v3_c12477	3	6	-	-	-	-	-
gALL_v3_c12478	2	6	-	-	-	-	-
gALL_v3_c12479	1	5	-	-	-	-	-
gALL_v3_c1248	16	34	-	-	-	-	-
gALL_v3_c12480	6	9	-	-	-	-	-
gALL_v3_c12483	5	5	-	-	-	-	-
gALL_v3_c12484	2	5	-	-	gb|EGI66919.1|	MKL/myocardin-like protein 2 [Acromyrmex echinatior]	3,00E-16
gALL_v3_c12485	1	4	-	-	-	-	-
gALL_v3_c12486	5	4	-	-	-	-	-
gALL_v3_c12487	3	4	gnl|CDD|190596	pfam03311, Cornichon, Cornic	ref|XP_974854.1|	PREDICTED: similar to cornichon protein, putative [Tribolium castaneum]	7,00E-65
gALL_v3_c12488	1	7	-	-	-	-	-
gALL_v3_c12489	3	3	-	-	gb|ADI19371.1|	hypothetical protein [uncultured Spirochaetales bacterium HF0500_06B09]	2,00E-30
gALL_v3_c1249	30	20	gnl|CDD|128886	smart00638, LPD_N, Lipoprote	ref|XP_971721.2|	PREDICTED: similar to conserved hypothetical protein [Tribolium castaneum]	0
gALL_v3_c12490	2	10	gnl|CDD|34819	COG5222, COG5222, Uncharac	ref|XP_003487947.1|	PREDICTED: hypothetical protein LOC100746010 [Bombus impatiens]	1,00E-55
gALL_v3_c12491	2	6	-	-	ref|XP_003250433.1|	PREDICTED: probable G-protein coupled receptor Mth-like 1-like isoform X1 [Apis mellifera] ref|XP_006564276.1| PREDICTED: probable G-protein coupled receptor Mth-lil	6,00E-33
gALL_v3_c12492	3	7	-	-	-	-	-
gALL_v3_c12493	3	7	-	-	gb|EFA06189.1|	hypothetical protein TcasGA2_TC009038 [Tribolium castaneum]	2,00E-81
gALL_v3_c12494	6	6	-	-	-	-	-
gALL_v3_c12496	3	6	-	-	ref|XP_003394647.1|	PREDICTED: hypothetical protein LOC100648429 [Bombus terrestris]	2,00E-76
gALL_v3_c12497	1	4	gnl|CDD|73154	cd01647, RT_LTR, RT_LTR: Rev	ref|XP_001808069.1|	PREDICTED: similar to protease, reverse transcriptase and RNase H [Tribolium castaneum]	7,00E-41
gALL_v3_c12498	5	3	-	-	-	-	-
gALL_v3_c12499	2	6	-	-	-	-	-
gALL_v3_c125	10	23	-	-	-	-	-
gALL_v3_c1250	8	16	gnl|CDD|128654	smart00360, RRM, RNA recogr	gb|EFN80772.1|	Splicing factor, arginine/serine-rich 10 [Harpegnathos saltator]	2,00E-60
gALL_v3_c12500	2	6	-	-	ref|XP_003393231.1|	PREDICTED: niemann-Pick C1 protein-like [Bombus terrestris]	9,00E-46
gALL_v3_c12501	3	8	-	-	ref|XP_001606081.2|	PREDICTED: hypothetical protein LOC100122475 [Nasonia vitripennis]	1,00E-105
gALL_v3_c12502	5	3	gnl|CDD|29257	cd00200, WD40, WD40 domai	dbj|BAN21016.1|	striatin, putative [Riptortus pedestris]	1,00E-168
gALL_v3_c12503	5	4	-	-	-	-	-
gALL_v3_c12504	4	9	-	-	-	-	-
gALL_v3_c12505	4	6	-	-	ref|XP_003425515.1|	PREDICTED: LOW QUALITY PROTEIN: protocadherin-15-like [Nasonia vitripennis]	9,00E-35
gALL_v3_c12506	7	5	-	-	-	-	-
gALL_v3_c12507	3	7	-	-	-	-	-
gALL_v3_c12508	4	6	gnl|CDD|99924	cd05492, Bromo_ZMYND11, B	ref|XP_003706643.1|	PREDICTED: zinc finger MYND domain-containing protein 11-like isoform 2 [Megachile rotundata]	4,00E-69
gALL_v3_c12509	3	7	-	-	ref|XP_974350.1|	PREDICTED: similar to CG4806 CG4806-PA [Tribolium castaneum] gb|EFA01819.1| hypothetical protein TcasGA2_TC007421 [Tribolium castaneum]	6,00E-20
gALL_v3_c1251	8	15	-	-	-	-	-
gALL_v3_c12511	1	4	-	-	-	-	-
gALL_v3_c12512	2	4	-	-	-	-	-
gALL_v3_c12513	4	4	-	-	-	-	-
gALL_v3_c12516	5	5	-	-	-	-	-
gALL_v3_c12517	3	9	-	-	ref|XP_969331.2|	PREDICTED: similar to orb2 CG5735-PB [Tribolium castaneum]	7,00E-69
gALL_v3_c12518	5	4	-	-	-	-	-
gALL_v3_c1252	15	12	-	-	-	-	-
gALL_v3_c12521	3	3	-	-	-	-	-
gALL_v3_c12522	8	5	-	-	-	-	-
gALL_v3_c12523	2	3	-	-	gb|EGI68754.1|	Teneurin-3 [Acromyrmex echinatior]	1,00E-96
gALL_v3_c12524	0	6	gnl|CDD|30936	COG0591, PutP, Na+/proline s	ref|XP_312589.3|	AGAP002366-PA [Anopheles gambiae str. PEST] gb|EAA07459.3| AGAP002366-PA [Anopheles gambiae str. PEST]	1,00E-65
gALL_v3_c12525	2	6	-	-	-	-	-
gALL_v3_c12526	6	5	-	-	gb|AGM32295.1|	E3 ubiquitin-protein ligase siah-1, partial [Coptotermes formosanus]	3,00E-65
gALL_v3_c12527	2	4	-	-	-	-	-
gALL_v3_c12528	5	2	-	-	gb|EFA11632.1|	hypothetical protein TcasGA2_TC000010 [Tribolium castaneum]	5,00E-09
gALL_v3_c12529	4	4	-	-	-	-	-
gALL_v3_c1253	12	25	gnl|CDD|143864	pfam00083, Sugar_tr, Sugar (a	gb|ENN70293.1|	hypothetical protein YQE_12804, partial [Dendroctonus ponderosae]	6,00E-38
gALL_v3_c12531	5	6	-	-	ref|XP_001599465.2|	PREDICTED: protein RRP5 homolog [Nasonia vitripennis]	6,00E-19
gALL_v3_c12532	4	3	-	-	-	-	-
gALL_v3_c12533	0	5	-	-	-	-	-
gALL_v3_c12534	3	3	-	-	-	-	-
gALL_v3_c12535	4	5	-	-	ref|XP_001946731.2|	PREDICTED: tigger transposable element-derived protein 4-like [Acyrthosiphon pisum]	2,00E-06
gALL_v3_c12536	5	3	-	-	ref|XP_002432233.1|	conserved hypothetical protein [Pediculus humanus corporis] gb|EEB19495.1| conserved hypothetical protein [Pediculus humanus corporis]	4,00E-53
gALL_v3_c12538	4	2	-	-	-	-	-
gALL_v3_c12539	5	3	-	-	-	-	-
gALL_v3_c1254	8	10	-	-	-	-	-
gALL_v3_c12540	12	19	-	-	-	-	-
gALL_v3_c12541	0	6	-	-	-	-	-
gALL_v3_c12542	4	2	-	-	-	-	-
gALL_v3_c12543	1	5	gnl|CDD|189653	pfam00650, CRAL_TRIO, CRAL	gb|AEE62464.1|	unknown [Dendroctonus ponderosae] gb|ENN80365.1| hypothetical protein YQE_03224, partial [Dendroctonus ponderosae] gb|ENN83365.1| hypothetical protein YQE_0	7,00E-61
gALL_v3_c12544	2	4	gnl|CDD|128417	smart00107, BTK, Bruton's tyr	gb|EFN60533.1|	Ras GTPase-activating protein 3 [Camponotus floridanus]	7,00E-59
gALL_v3_c12545	3	3	-	-	-	-	-
gALL_v3_c12546	4	3	-	-	-	-	-
gALL_v3_c12547	6	12	gnl|CDD|189372	pfam00067, p450, Cytochrom	gb|ABB86762.2|	CYP4U3v1 [Reticulitermes flavipes]	3,00E-85
gALL_v3_c12548	2	4	-	-	-	-	-
gALL_v3_c12549	1	7	-	-	gb|ESO09138.1|	hypothetical protein HELRODRAFT_169080 [Helobdella robusta]	4,00E-28
gALL_v3_c12550	2	7	-	-	-	-	-
gALL_v3_c12551	3	10	gnl|CDD|189974	pfam01403, Sema, Sema dom	gb|EFZ15870.1|	hypothetical protein SINV_05024 [Solenopsis invicta]	0
gALL_v3_c12552	12	8	-	-	ref|XP_003703511.1|	PREDICTED: uncharacterized protein LOC100877436 isoform 2 [Megachile rotundata]	7,00E-28
gALL_v3_c12553	5	3	-	-	-	-	-
gALL_v3_c12554	1	5	-	-	ref|XP_002426346.1|	transformation/transcription domain-associated protein, putative [Pediculus humanus corporis] gb|EEB13608.1| transformation/transcription domain-associated protein,	4,00E-68
gALL_v3_c12555	7	6	-	-	-	-	-
gALL_v3_c12556	2	4	-	-	gb|EKC25460.1|	Zinc finger homeobox protein 3 [Crassostrea gigas]	2,00E-11
gALL_v3_c12557	3	2	-	-	-	-	-
gALL_v3_c12558	2	4	-	-	-	-	-
gALL_v3_c12559	5	4	-	-	-	-	-
gALL_v3_c1256	7	13	-	-	-	-	-
gALL_v3_c12560	4	7	-	-	-	-	-
gALL_v3_c12561	2	7	-	-	-	-	-
gALL_v3_c12563	2	6	gnl|CDD|115393	pfam06732, Pescadillo_N, Pes	gb|EGI68312.1|	Pescadillo-like protein [Acromyrmex echinatior]	4,00E-99
gALL_v3_c12564	5	3	-	-	-	-	-
gALL_v3_c12565	2	4	-	-	-	-	-
gALL_v3_c12567	2	3	-	-	gb|EHJ68213.1|	hypothetical protein KGM_12625 [Danaus plexippus]	8,00E-13
gALL_v3_c12568	6	9	-	-	-	-	-
gALL_v3_c1257	4	12	gnl|CDD|128489	smart00192, LDLa, Low-densit	gb|EFA06629.1|	hypothetical protein TcasGA2_TC009548 [Tribolium castaneum]	1,00E-113
gALL_v3_c12570	8	19	-	-	-	-	-

gALL_v3_c12571	2	3	-	-	-	-	-
gALL_v3_c12572	3	2	gnl|CDD|162403	TIGR01531, glyc_debranch, gly	gb|EFN82290.1|	Glycogen debranching enzyme [Harpegnathos saltator]	9,00E-66
gALL_v3_c12573	3	1	-	-	-	-	-
gALL_v3_c12575	6	8	gnl|CDD|187550	cd05239, GDP_FS_SDR_e, GDI	ref|XP_967089.1|	PREDICTED: similar to nad dependent epimerase/dehydratase [Tribolium castaneum] gb|EFA04892.1| hypothetical protein TcasGA2_TC014956 [Tribolium castaneum]	1,00E-134
gALL_v3_c12576	4	5	gnl|CDD|189693	pfam00735, Septin, Septin. M	gb|EGI59097.1|	Protein peanut [Acromyrmex echinatior]	1,00E-115
gALL_v3_c12577	2	4	-	-	ref|XP_006614742.1|	PREDICTED: tensin-3-like isoform X1 [Apis dorsata]	4,00E-43
gALL_v3_c12578	5	3	gnl|CDD|73156	cd01650, RT_nLTR_like, RT_nL	gb|ADZ95942.1|	reverse transcriptase, partial [Oeneis sculda] gb|ADZ95943.1| reverse transcriptase, partial [Oeneis sculda]	8,00E-15
gALL_v3_c12579	2	5	gnl|CDD|132938	cd06607, STKc_TAO, Catalytic	ref|XP_003692280.1|	PREDICTED: serine/threonine-protein kinase TAO1-like [Apis florea] ref|XP_006572321.1| PREDICTED: serine/threonine-protein kinase TAO1 isoform X1 [Apis mellifera] ref	2,00E-31
gALL_v3_c1258	4	7	gnl|CDD|99943	cd05511, Bromo_TFIID, Bromo	ref|XP_002431609.1|	transcription initiation factor TFIID subunit, putative [Pediculus humanus corporis] gb|EEB18871.1| transcription initiation factor TFIID subunit, putative [Pediculus human	1,00E-124
gALL_v3_c12580	1	8	gnl|CDD|128524	smart00228, PDZ, Domain pre	ref|XP_002432943.1|	afadin, putative [Pediculus humanus corporis] gb|EEB20205.1| afadin, putative [Pediculus humanus corporis]	2,00E-84
gALL_v3_c12581	4	2	-	-	ref|XP_003427862.1|	PREDICTED: hypothetical protein LOC100117193 [Nasonia vitripennis]	4,00E-11
gALL_v3_c12583	8	7	gnl|CDD|193607	cd09633, Deltex_C, Domain fo	gb|EFN78766.1|	Protein deltex [Harpegnathos saltator]	5,00E-74
gALL_v3_c12584	5	3	-	-	-	-	-
gALL_v3_c12585	1	5	-	-	-	-	-
gALL_v3_c12586	1	4	gnl|CDD|145641	pfam02602, HEM4, Uroporphy	ref|XP_317395.4|	AGAP008064-PA [Anopheles gambiae str. PEST] gb|EAA12237.4| AGAP008064-PA [Anopheles gambiae str. PEST]	5,00E-31
gALL_v3_c12587	7	5	-	-	ref|XP_003699013.1|	PREDICTED: uncharacterized protein LOC100868479 [Apis florea]	7,00E-25
gALL_v3_c12588	2	6	-	-	-	-	-
gALL_v3_c1259	7	12	gnl|CDD|178687	PLN03142, PLN03142, Probabl	ref|XP_967398.2|	PREDICTED: similar to brahma CG5942-PA, partial [Tribolium castaneum]	1,00E-171
gALL_v3_c12590	1	6	-	-	-	-	-
gALL_v3_c12591	3	12	gnl|CDD|178734	PLN03192, PLN03192, Voltage	gb|EFX90436.1|	hypothetical protein DAPPUDRAFT_39704 [Daphnia pulex]	0
gALL_v3_c12592	2	5	-	-	-	-	-
gALL_v3_c12594	4	2	gnl|CDD|189773	pfam00928, Adap_comp_sub,	gb|AGM32967.1|	AP-3 complex subunit Mu-1 [Coptotermes formosanus]	1,00E-101
gALL_v3_c12595	5	5	gnl|CDD|192374	pfam09770, PAT1, Topoisomer	gb|EFN79257.1|	Protein PAT1-like protein 1 [Harpegnathos saltator]	4,00E-28
gALL_v3_c12596	8	8	gnl|CDD|189424	pfam00153, Mito_carr, Mitoch	ref|XP_002423193.1|	Peroxisomal membrane protein PMP34, putative [Pediculus humanus corporis] gb|EEB10455.1| Peroxisomal membrane protein PMP34, putative [Pediculus humanus corp	1,00E-126
gALL_v3_c12598	3	9	-	-	ref|XP_003699263.1|	PREDICTED: prohormone-1-like [Megachile rotundata]	8,00E-33
gALL_v3_c12599	2	3	-	-	-	-	-
gALL_v3_c126	4	9	-	-	-	-	-
gALL_v3_c1260	7	6	gnl|CDD|48517	cd02968, SCO, SCO (an acrony	ref|XP_969355.1|	PREDICTED: similar to GA21389-PA [Tribolium castaneum]	6,00E-90
gALL_v3_c12600	5	5	-	-	-	-	-
gALL_v3_c12601	3	3	-	-	-	-	-
gALL_v3_c12602	2	6	-	-	ref|XP_002432992.1|	Phosphatidylinositol 4-kinase alpha, putative [Pediculus humanus corporis] gb|EEB20254.1| Phosphatidylinositol 4-kinase alpha, putative [Pediculus humanus corporis]	2,00E-73
gALL_v3_c12603	0	4	-	-	-	-	-
gALL_v3_c12604	3	4	-	-	-	-	-
gALL_v3_c12605	4	6	gnl|CDD|185524	PTZ00242, PTZ00242, protein	ref|XP_003490800.1|	PREDICTED: protein tyrosine phosphatase type IVA 1-like [Bombus impatiens]	5,00E-51
gALL_v3_c12607	4	7	gnl|CDD|28945	cd00063, FN3, Fibronectin typ	ref|XP_973615.1|	PREDICTED: similar to AGAP009522-PA [Tribolium castaneum]	1,00E-109
gALL_v3_c12608	2	10	-	-	-	-	-
gALL_v3_c12609	7	4	gnl|CDD|34794	COG5195, COG5195, Uncharac	gb|EFN79774.1|	Uncharacterized protein C18orf37-like protein [Harpegnathos saltator]	2,00E-25
gALL_v3_c1261	6	7	-	-	-	-	-
gALL_v3_c12610	1	3	-	-	-	-	-
gALL_v3_c12611	6	4	-	-	-	-	-
gALL_v3_c12613	5	4	-	-	ref|XP_003704583.1|	PREDICTED: cytoskeleton-associated protein 5 [Megachile rotundata]	5,00E-64
gALL_v3_c12614	3	3	gnl|CDD|115196	pfam06524, NOA36, NOA36 p	ref|XP_974713.1|	PREDICTED: similar to Noa36 CG10009-PA [Tribolium castaneum] gb|EFA01944.1| hypothetical protein TcasGA2_TC007558 [Tribolium castaneum]	1,00E-115
gALL_v3_c12615	0	4	-	-	-	-	-
gALL_v3_c12616	5	3	-	-	gb|EFA00604.1|	hypothetical protein TcasGA2_TC003476 [Tribolium castaneum]	3,00E-73
gALL_v3_c12617	1	6	-	-	-	-	-
gALL_v3_c12618	9	19	-	-	-	-	-
gALL_v3_c12619	2	4	-	-	ref|XP_970572.1|	PREDICTED: similar to corticotropin releasing hormone binding protein [Tribolium castaneum] gb|EEZ98485.1| hypothetical protein TcasGA2_TC000979 [Tribolium castane	5,00E-14
gALL_v3_c1262	23	21	-	-	ref|XP_002423941.1|	conserved hypothetical protein [Pediculus humanus corporis] gb|EEB11203.1| conserved hypothetical protein [Pediculus humanus corporis]	3,00E-11
gALL_v3_c12620	11	6	gnl|CDD|163699	cd08068, MPN_BRCC36, Mov3	gb|EKC34695.1|	Lys-63-specific deubiquitinase BRCC36 [Crassostrea gigas]	3,00E-88
gALL_v3_c12621	2	3	-	-	-	-	-
gALL_v3_c12622	4	2	-	-	-	-	-
gALL_v3_c12623	3	2	gnl|CDD|189660	pfam00664, ABC_membrane,	ref|XP_002432260.1|	multidrug resistance protein, putative [Pediculus humanus corporis] gb|EEB19522.1| multidrug resistance protein, putative [Pediculus humanus corporis]	2,00E-48
gALL_v3_c12624	1	5	-	-	-	-	-
gALL_v3_c12627	3	4	-	-	-	-	-
gALL_v3_c12628	2	7	-	-	-	-	-
gALL_v3_c12629	0	6	-	-	-	-	-
gALL_v3_c1263	13	15	gnl|CDD|192732	pfam11265, Med25_VWA, Me	gb|EFN70189.1|	Mediator of RNA polymerase II transcription subunit 25 [Camponotus floridanus]	1,00E-124
gALL_v3_c12630	3	6	-	-	-	-	-
gALL_v3_c12631	18	12	-	-	-	-	-
gALL_v3_c12632	3	8	-	-	ref|XP_006488328.1|	PREDICTED: CAX-interacting protein 4-like [Citrus sinensis]	4,00E-06
gALL_v3_c12633	4	4	gnl|CDD|187665	cd09805, type2_17beta_HSD-	ref|XP_005269412.1|	PREDICTED: D-beta-hydroxybutyrate dehydrogenase, mitochondrial isoform X4 [Homo sapiens]	1,00E-59
gALL_v3_c12636	4	4	-	-	-	-	-
gALL_v3_c12637	11	13	gnl|CDD|147559	pfam05434, Tmemb_9, TMEM	gb|AGM32592.1|	TMEM9 family protein [Coptotermes formosanus]	2,00E-36
gALL_v3_c12638	3	3	-	-	-	-	-
gALL_v3_c12639	5	2	-	-	-	-	-
gALL_v3_c1264	10	17	-	-	gb|EFN72641.1|	E3 ubiquitin-protein ligase UBR2 [Camponotus floridanus]	1,00E-76
gALL_v3_c12640	5	6	gnl|CDD|190863	pfam04091, Sec15, Exocyst co	ref|XP_003689606.1|	PREDICTED: exocyst complex component 6B-like isoform 1 [Apis florea]	1,00E-160
gALL_v3_c12641	6	12	-	-	ref|XP_392446.1|	PREDICTED: death-associated protein 1-like [Apis mellifera] ref|XP_006619717.1| PREDICTED: death-associated protein 1-like [Apis dorsata]	2,00E-41
gALL_v3_c12644	3	6	-	-	-	-	-
gALL_v3_c12647	1	4	gnl|CDD|144071	pfam00339, Arrestin_N, Arres	ref|XP_002070813.1|	GK10823 [Drosophila willistoni] gb|EDW81799.1| GK10823 [Drosophila willistoni]	2,00E-26
gALL_v3_c12648	4	2	-	-	-	-	-
gALL_v3_c12649	3	1	-	-	-	-	-
gALL_v3_c12650	11	12	-	-	ref|XP_004518408.1|	PREDICTED: homeotic protein female sterile-like isoform X1 [Ceratitis capitata] ref|XP_004518409.1| PREDICTED: homeotic protein female sterile-like isoform X2 [Ceratitis	3,00E-20
gALL_v3_c12651	7	4	-	-	-	-	-
gALL_v3_c12652	3	4	gnl|CDD|190411	pfam02759, RUN, RUN domai	ref|XP_003493578.1|	PREDICTED: hypothetical protein LOC100746159 [Bombus impatiens]	1,00E-31
gALL_v3_c12653	4	6	gnl|CDD|72873	cd04758, Commd10, COMM_	ref|XP_002594623.1|	hypothetical protein BRAFLDRAFT_121749 [Branchiostoma floridae] gb|EEN50634.1| hypothetical protein BRAFLDRAFT_121749 [Branchiostoma floridae]	3,00E-53
gALL_v3_c12654	2	5	-	-	-	-	-
gALL_v3_c12655	2	8	-	-	gb|EFZ14366.1|	hypothetical protein SINV_09026 [Solenopsis invicta]	1,00E-09
gALL_v3_c12656	4	8	-	-	-	-	-
gALL_v3_c12658	6	9	-	-	ref|XP_002426350.1|	conserved hypothetical protein [Pediculus humanus corporis] gb|EEB13612.1| conserved hypothetical protein [Pediculus humanus corporis]	2,00E-18
gALL_v3_c12659	8	21	-	-	-	-	-
gALL_v3_c12661	3	4	-	-	-	-	-
gALL_v3_c12662	3	4	gnl|CDD|30503	COG0154, GatA, Asp-tRNAAsn	ref|XP_003707713.1|	PREDICTED: fatty-acid amide hydrolase 2-like [Megachile rotundata]	1,00E-44
gALL_v3_c12663	7	10	gnl|CDD|162218	TIGR01134, purF, amidophosp	ref|XP_001605718.1|	PREDICTED: amidophosphoribosyltransferase [Nasonia vitripennis]	0
gALL_v3_c12664	2	4	-	-	-	-	-
gALL_v3_c12665	4	4	-	-	gb|EFA09839.1|	hypothetical protein TcasGA2_TC011986 [Tribolium castaneum]	1,00E-117
gALL_v3_c12666	6	6	-	-	gb|AGM32707.1|	hypothetical protein, partial [Coptotermes formosanus]	5,00E-06
gALL_v3_c12668	0	4	-	-	-	-	-
gALL_v3_c12669	3	5	-	-	-	-	-
gALL_v3_c1267	11	26	-	-	-	-	-
gALL_v3_c12670	5	12	-	-	-	-	-
gALL_v3_c12671	5	3	-	-	ref|XP_003699834.1|	PREDICTED: limkain-b1-like [Megachile rotundata]	3,00E-37
gALL_v3_c12672	0	6	-	-	-	-	-
gALL_v3_c12673	1	7	-	-	ref|XP_971827.1|	PREDICTED: similar to AGAP005192-PA [Tribolium castaneum]	2,00E-73
gALL_v3_c12674	2	8	-	-	-	-	-
gALL_v3_c12676	6	3	-	-	ref|XP_003490025.1|	PREDICTED: sphingomyelin synthase-related 1-like [Bombus impatiens]	3,00E-67
gALL_v3_c12679	3	8	-	-	-	-	-
gALL_v3_c1268	10	7	-	-	-	-	-
gALL_v3_c12680	2	2	-	-	ref|XP_975065.1|	PREDICTED: similar to RWD domain containing 2A [Tribolium castaneum] gb|EFA02629.1| hypothetical protein TcasGA2_TC008349 [Tribolium castaneum]	8,00E-11
gALL_v3_c12684	2	4	gnl|CDD|144153	pfam00450, Peptidase_S10, Se	gb|AGM32338.1|	serine carboxypeptidase [Coptotermes formosanus]	8,00E-61
gALL_v3_c12685	4	3	gnl|CDD|189758	pfam00887, ACBP, Acyl CoA bi	gb|AGM32178.1|	acyl-coA-binding domain-containing protein 6-like protein [Coptotermes formosanus]	5,00E-42
gALL_v3_c12686	6	3	-	-	-	-	-
gALL_v3_c12687	5	3	-	-	emb|CAF98882.1|	unnamed protein product [Tetraodon nigroviridis]	1,00E-32
gALL_v3_c12689	2	1	gnl|CDD|192885	pfam11931, DUF3449, Domai	ref|XP_002045519.1|	GM15455 [Drosophila sechellia] gb|EDW51097.1| GM15455 [Drosophila sechellia]	1,00E-69
gALL_v3_c1269	11	9	-	-	-	-	-
gALL_v3_c12690	7	14	gnl|CDD|73156	cd01650, RT_nLTR_like, RT_nL	ref|XP_003724311.1|	PREDICTED: RNA-directed DNA polymerase from mobile element jockey-like [Strongylocentrotus purpuratus]	4,00E-26
gALL_v3_c12691	3	9	-	-	ref|XP_001662029.1|	N-acetyllactosaminide beta-1,3-N-acetylglucosaminyltransferase, putative [Aedes aegypti] gb|EAT35985.1| AAEL011900-PA [Aedes aegypti]	4,00E-43
gALL_v3_c12692	1	6	gnl|CDD|192548	pfam10351, Apt1, Golgi-body	ref|XP_002425112.1|	conserved hypothetical protein [Pediculus humanus corporis] gb|EEB12374.1| conserved hypothetical protein [Pediculus humanus corporis]	9,00E-77
gALL_v3_c12693	3	7	gnl|CDD|176559	cd08622, PI-PLCXDc_CG14945	gb|EFN84999.1|	Phosphatidylinositol-specific phospholipase C X domain-containing protein 1 [Harpegnathos saltator]	6,00E-51
gALL_v3_c12695	2	3	-	-	ref|XP_396204.4|	PREDICTED: solute carrier family 35 member F5-like isoform X3 [Apis mellifera] ref|XP_006562240.1| PREDICTED: solute carrier family 35 member F5-like isoform X1 [Apis	3,00E-61
gALL_v3_c12696	4	3	-	-	-	-	-
gALL_v3_c12697	4	7	-	-	-	-	-
gALL_v3_c12698	1	5	gnl|CDD|182198	PRK10019, PRK10019, nickel/c	ref|XP_002072643.1|	GK13714 [Drosophila willistoni] gb|EDW83629.1| GK13714 [Drosophila willistoni]	1,00E-17
gALL_v3_c12699	0	5	-	-	-	-	-
gALL_v3_c127	4	5	-	-	-	-	-
gALL_v3_c12700	3	5	gnl|CDD|132940	cd06609, STKc_MST3_like, Cat	dbj|BAM19867.1|	germinal centre kinase III, partial [Papilio xuthus]	2,00E-95
gALL_v3_c12701	2	4	gnl|CDD|72865	cd04750, Commd2, COMM_D	gb|EFN77282.1|	COMM domain-containing protein 2 [Harpegnathos saltator]	3,00E-35
gALL_v3_c12702	6	3	-	-	ref|XP_004927506.1|	PREDICTED: uncharacterized protein LOC101744756 [Bombyx mori]	3,00E-18
gALL_v3_c12703	2	3	-	-	-	-	-
gALL_v3_c12704	2	4	gnl|CDD|128524	smart00228, PDZ, Domain pre	ref|XP_002407136.1|	glutamate receptor interacting protein, putative [Ixodes scapularis] gb|EEC03692.1| glutamate receptor interacting protein, putative [Ixodes scapularis]	3,00E-67
gALL_v3_c12705	5	4	gnl|CDD|112027	pfam03194, LUC7, LUC7 N_ter	ref|XP_317153.4|	AGAP008314-PA [Anopheles gambiae str. PEST] gb|EAA43857.4| AGAP008314-PA [Anopheles gambiae str. PEST]	3,00E-46
gALL_v3_c12706	5	2	-	-	gb|EFN61865.1|	Protein MON2-like protein [Camponotus floridanus]	1,00E-128
gALL_v3_c12707	0	6	gnl|CDD|128517	smart00221, STYKc, Protein ki	gb|EFN66408.1|	NUAK family SNF1-like kinase 1 [Camponotus floridanus]	1,00E-55
gALL_v3_c12708	2	5	-	-	-	-	-
gALL_v3_c12709	1	3	-	-	-	-	-
gALL_v3_c1271	9	8	-	-	-	-	-
gALL_v3_c12710	5	2	-	-	-	-	-
gALL_v3_c12711	4	4	-	-	-	-	-
gALL_v3_c12713	5	5	-	-	-	-	-
gALL_v3_c12714	3	4	-	-	-	-	-
gALL_v3_c12715	0	5	-	-	-	-	-
gALL_v3_c12716	6	4	gnl|CDD|143180	cd04979, Ig_Semaphorin_C, Ir	ref|XP_968340.1|	PREDICTED: similar to semaphorin 2a [Tribolium castaneum]	1,00E-168
gALL_v3_c12717	3	8	-	-	-	-	-
gALL_v3_c12718	6	5	gnl|CDD|191256	pfam05327, RRN3, RNA polym	ref|XP_972768.1|	PREDICTED: similar to transcription initiation factor RRN3 [Tribolium castaneum]	2,00E-47
gALL_v3_c1272	6	10	-	-	-	-	-
gALL_v3_c12722	3	5	-	-	-	-	-
gALL_v3_c12723	6	3	-	-	-	-	-
gALL_v3_c12724	5	4	gnl|CDD|148917	pfam07565, Band_3_cyto, Bar	ref|XP_006622694.1|	PREDICTED: sodium-driven chloride bicarbonate exchanger-like isoform X1 [Apis dorsata]	3,00E-84
gALL_v3_c12725	12	8	-	-	ref|XP_003219874.1|	PREDICTED: UPF0436 protein C9orf6-like [Anolis carolinensis]	2,00E-50
gALL_v3_c12727	5	7	-	-	-	-	-
gALL_v3_c12729	3	4	gnl|CDD|119332	cd06562, GH20_HexA_HexB-li	gb|AFZ76982.1|	beta-N-acetylglucosaminidase [Locusta migratoria]	2,00E-91
gALL_v3_c1273	5	7	-	-	-	-	-
gALL_v3_c12730	5	6	-	-	gb|AGG09855.1|	mitochondrial glutamate dehydrogenase 1, partial [Nilaparvata lugens]	1,00E-21
gALL_v3_c12731	3	7	gnl|CDD|30859	COG0513, SrmB, Superfamily I	ref|XP_003486759.1|	PREDICTED: ATP-dependent RNA helicase Ddx1-like [Bombus impatiens]	1,00E-151

gALL_v3_c12732	2	4	-	-	gb|EFN67634.1|	Putative uncharacterized transposon-derived protein F52C9.6 [Camponotus floridanus]	5,00E-18
gALL_v3_c12734	5	10	gnl|CDD|192374	pfam09770, PAT1, Topoisomer	-	-	-
gALL_v3_c12735	2	2	-	-	-	-	-
gALL_v3_c12736	3	5	-	-	-	-	-
gALL_v3_c12737	2	4	-	-	gb|ADI61823.1|	endonuclease-reverse transcriptase [Bombyx mori]	3,00E-10
gALL_v3_c12738	2	3	-	-	-	-	-
gALL_v3_c12739	5	4	-	-	-	-	-
gALL_v3_c12740	3	2	-	-	-	-	-
gALL_v3_c12741	1	6	-	-	-	-	-
gALL_v3_c12742	6	7	-	-	-	-	-
gALL_v3_c12743	6	6	gnl|CDD|190590	pfam03281, Mab-21, Mab-21	gb|ENN80074.1|	hypothetical protein YQE_03490, partial [Dendroctonus ponderosae] gb|ENN80076.1| hypothetical protein YQE_03492, partial [Dendroctonus ponderosae]	1,00E-150
gALL_v3_c12744	2	6	-	-	dbj|BAC82595.1|	reverse transcriptase [Anopheles gambiae]	2,00E-24
gALL_v3_c12745	3	5	-	-	ref|XP_002430480.1|	conserved hypothetical protein [Pediculus humanus corporis] gb|EEB17742.1| conserved hypothetical protein [Pediculus humanus corporis]	6,00E-99
gALL_v3_c12746	4	3	-	-	-	-	-
gALL_v3_c12747	0	4	-	-	-	-	-
gALL_v3_c12748	2	6	-	-	-	-	-
gALL_v3_c12749	1	6	-	-	ref|XP_002432851.1|	activator protein, putative [Pediculus humanus corporis] gb|EEB20113.1| activator protein, putative [Pediculus humanus corporis]	1,00E-32
gALL_v3_c12750	2	2	gnl|CDD|176084	cd08534, SAM_PNT-GABP-alpl	gb|EFZ21919.1|	hypothetical protein SINV_00365 [Solenopsis invicta]	1,00E-45
gALL_v3_c12751	1	5	gnl|CDD|149776	pfam08824, Serine_rich, Serin	ref|XP_001688659.2|	AGAP005411-PB [Anopheles gambiae str. PEST] gb|EDO63665.2| AGAP005411-PB [Anopheles gambiae str. PEST]	8,00E-70
gALL_v3_c12752	1	6	-	-	-	-	-
gALL_v3_c12753	4	5	gnl|CDD|193060	pfam12513, SUV3_C, Mitocho	ref|XP_001811917.1|	PREDICTED: similar to ATP-dependent RNA and DNA helicase [Tribolium castaneum] gb|EFA10159.1| hypothetical protein TcasGA2_TC012348 [Tribolium castaneum]	0
gALL_v3_c12754	4	2	gnl|CDD|130026	TIGR00951, 2A43, Lysosomal C	dbj|BAM18596.1|	similar to CG3792 [Papilio xuthus]	4,00E-60
gALL_v3_c12755	3	7	gnl|CDD|34654	COG5049, XRN1, 5'-3' exonucl	ref|XP_001601647.2|	PREDICTED: 5'-3' exoribonuclease 2 homolog [Nasonia vitripennis]	2,00E-51
gALL_v3_c12757	18	16	-	-	-	-	-
gALL_v3_c12760	7	4	-	-	-	-	-
gALL_v3_c12762	1	4	-	-	-	-	-
gALL_v3_c12764	4	6	-	-	-	-	-
gALL_v3_c12765	5	13	-	-	-	-	-
gALL_v3_c12766	2	5	gnl|CDD|188096	TIGR00930, 2a30, K-Cl cotrans	ref|XP_002428768.1|	sodium-potassium-chloride cotransporter, putative [Pediculus humanus corporis] gb|EEB16030.1| sodium-potassium-chloride cotransporter, putative [Pediculus humanus	4,00E-54
gALL_v3_c12768	2	4	-	-	gb|AGK40936.1|	toll-7 [Nilaparvata lugens]	1,00E-106
gALL_v3_c12769	5	8	gnl|CDD|189955	pfam01363, FYVE, FYVE zinc fi	ref|XP_002425878.1|	FYVE, RhoGEF and PH domain-containing protein, putative [Pediculus humanus corporis] gb|EEB13140.1| FYVE, RhoGEF and PH domain-containing protein, putative [Pedi	1,00E-129
gALL_v3_c1277	6	10	gnl|CDD|178488	PLN02900, PLN02900, alanyl-t	ref|XP_966465.1|	PREDICTED: similar to alanyl-tRNA synthetase [Tribolium castaneum] gb|EFA03148.1| hypothetical protein TcasGA2_TC013061 [Tribolium castaneum]	1,00E-114
gALL_v3_c12770	2	4	gnl|CDD|88620	cd05157, ETNK_euk, Ethanola	gb|AGM32565.1|	ethanolamine kinase 1-like protein [Coptotermes formosanus]	2,00E-47
gALL_v3_c12772	2	5	-	-	-	-	-
gALL_v3_c12773	2	4	gnl|CDD|114903	pfam06211, BAMBI, BMP and	gb|EFA10728.1|	BMP and activin membrane-bound inhibitor-like [Tribolium castaneum]	2,00E-19
gALL_v3_c12774	5	5	-	-	ref|XP_001600611.1|	PREDICTED: hypothetical protein LOC100116049 [Nasonia vitripennis]	1,00E-164
gALL_v3_c12775	4	3	-	-	-	-	-
gALL_v3_c12777	2	2	-	-	ref|XP_006572289.1|	PREDICTED: LOW QUALITY PROTEIN: voltage-gated potassium channel subunit beta-1 [Apis mellifera]	1,00E-14
gALL_v3_c12778	8	5	-	-	-	-	-
gALL_v3_c12779	3	1	-	-	-	-	-
gALL_v3_c1278	5	5	-	-	gb|EFZ15674.1|	hypothetical protein SINV_00956 [Solenopsis invicta]	1,00E-113
gALL_v3_c12780	2	3	-	-	-	-	-
gALL_v3_c12782	3	3	-	-	-	-	-
gALL_v3_c12784	2	4	gnl|CDD|147310	pfam05063, MT-A70, MT-A70	ref|XP_974982.1|	PREDICTED: similar to N6-adenosine-methyltransferase IME4 [Tribolium castaneum] gb|EFA09090.1| hypothetical protein TcasGA2_TC006811 [Tribolium castaneum]	2,00E-85
gALL_v3_c12785	6	9	gnl|CDD|144126	pfam00413, Peptidase_M10, M	gb|EFN76651.1|	Matrix metalloproteinase-14 [Harpegnathos saltator]	7,00E-89
gALL_v3_c12786	4	8	-	-	-	-	-
gALL_v3_c12787	5	5	-	-	-	-	-
gALL_v3_c12788	3	3	gnl|CDD|99858	cd06105, ScCit1-2_like, Saccha	ref|XP_970124.1|	PREDICTED: similar to citrate synthase [Tribolium castaneum] gb|EFA03279.1| hypothetical protein TcasGA2_TC013214 [Tribolium castaneum]	9,00E-79
gALL_v3_c12789	4	8	-	-	-	-	-
gALL_v3_c12790	8	13	gnl|CDD|150704	pfam10058, DUF2296, Predict	ref|XP_003705030.1|	PREDICTED: protein lunapark-B-like [Megachile rotundata]	3,00E-64
gALL_v3_c12791	1	5	-	-	gb|EFA08667.1|	hypothetical protein TcasGA2_TC006333 [Tribolium castaneum]	5,00E-12
gALL_v3_c12792	3	10	gnl|CDD|150785	pfam10152, DUF2360, Predict	gb|ESO89342.1|	hypothetical protein LOTGIDRAFT_183391 [Lottia gigantea]	1,00E-32
gALL_v3_c12793	3	3	-	-	gb|EHJ74645.1|	hypothetical protein KGM_11037 [Danaus plexippus]	6,00E-09
gALL_v3_c12794	6	4	-	-	-	-	-
gALL_v3_c12795	6	3	-	-	-	-	-
gALL_v3_c12796	1	5	gnl|CDD|188979	cd09580, SAM_Scm-like-4MBT	ref|XP_006610730.1|	PREDICTED: polycomb protein Sfmbt-like isoform X4 [Apis dorsata]	4,00E-16
gALL_v3_c12797	13	21	-	-	-	-	-
gALL_v3_c12798	6	3	-	-	-	-	-
gALL_v3_c12799	9	10	gnl|CDD|117221	pfam08648, DUF1777, Protein	-	-	-
gALL_v3_c1280	7	19	-	-	-	-	-
gALL_v3_c12800	3	5	-	-	-	-	-
gALL_v3_c12801	5	5	-	-	ref|XP_006611986.1|	PREDICTED: uncharacterized protein LOC102676589 isoform X3 [Apis dorsata]	5,00E-40
gALL_v3_c12803	4	3	-	-	-	-	-
gALL_v3_c12806	3	6	-	-	-	-	-
gALL_v3_c12807	3	3	-	-	-	-	-
gALL_v3_c12810	7	7	-	-	-	-	-
gALL_v3_c12812	5	2	-	-	ref|XP_003394257.1|	PREDICTED: hypothetical protein LOC100648591 [Bombus terrestris]	2,00E-12
gALL_v3_c12813	5	10	-	-	-	-	-
gALL_v3_c12814	1	5	-	-	-	-	-
gALL_v3_c12815	1	6	-	-	ref|XP_002422868.1|	conserved hypothetical protein [Pediculus humanus corporis] gb|EEB10130.1| conserved hypothetical protein [Pediculus humanus corporis]	2,00E-26
gALL_v3_c12816	4	3	gnl|CDD|162063	TIGR00840, b_cpa1, sodium/h	ref|XP_001808952.1|	PREDICTED: similar to sodium/hydrogen exchanger 7, 9 (nhe7, nhe9) [Tribolium castaneum]	1,00E-111
gALL_v3_c1282	7	6	-	-	ref|XP_002603167.1|	hypothetical protein BRAFLDRAFT_63199 [Branchiostoma floridae] gb|EEN59178.1| hypothetical protein BRAFLDRAFT_63199 [Branchiostoma floridae]	3,00E-06
gALL_v3_c12820	0	4	-	-	ref|XP_002735602.2|	PREDICTED: protein FAM177A1-like [Saccoglossus kowalevskii]	2,00E-34
gALL_v3_c12821	4	2	-	-	gb|EFN82732.1|	RRP12-like protein [Harpegnathos saltator]	1,00E-23
gALL_v3_c12822	5	6	-	-	-	-	-
gALL_v3_c12824	4	3	-	-	-	-	-
gALL_v3_c12825	1	4	-	-	ref|XP_003241518.1|	PREDICTED: hypothetical protein LOC100572758 [Acyrthosiphon pisum]	5,00E-07
gALL_v3_c12826	7	10	gnl|CDD|80348	cd02126, PA_EDEM3_like, PA_	ref|XP_002431612.1|	conserved hypothetical protein [Pediculus humanus corporis] gb|EEB18874.1| conserved hypothetical protein [Pediculus humanus corporis]	4,00E-28
gALL_v3_c12827	3	3	-	-	-	-	-
gALL_v3_c12828	1	3	-	-	gb|AGM32705.1|	hypothetical protein [Coptotermes formosanus]	6,00E-46
gALL_v3_c12829	3	2	gnl|CDD|29151	cd00189, TPR, Tetratricopeptic	ref|XP_006607971.1|	PREDICTED: dnaJ homolog subfamily C member 3-like [Apis dorsata]	3,00E-44
gALL_v3_c12830	2	6	gnl|CDD|162469	TIGR01657, P-ATPase-V, P-type	ref|XP_972127.1|	PREDICTED: similar to cation-transporting ATPase [Tribolium castaneum] gb|EFA04812.1| hypothetical protein TcasGA2_TC014862 [Tribolium castaneum]	1,00E-81
gALL_v3_c12831	2	3	-	-	-	-	-
gALL_v3_c12833	5	2	-	-	-	-	-
gALL_v3_c12835	0	6	-	-	-	-	-
gALL_v3_c12836	6	4	gnl|CDD|185505	PTZ00187, PTZ00187, succinyl	ref|XP_966490.1|	PREDICTED: similar to succinyl-CoA synthetase small subunit, putative isoform 1 [Tribolium castaneum] ref|XP_975865.1| PREDICTED: similar to succinyl-CoA synthetase sr	1,00E-102
gALL_v3_c12837	0	5	gnl|CDD|192927	pfam12054, DUF3535, Domair	ref|XP_966659.1|	PREDICTED: similar to TATA-binding protein-associated factor 172 [Tribolium castaneum] gb|EFA08205.1| hypothetical protein TcasGA2_TC005832 [Tribolium castaneum]	5,00E-34
gALL_v3_c12838	3	8	-	-	-	-	-
gALL_v3_c12839	3	5	-	-	-	-	-
gALL_v3_c12840	2	3	-	-	-	-	-
gALL_v3_c12841	3	3	-	-	-	-	-
gALL_v3_c12842	2	2	-	-	-	-	-
gALL_v3_c12844	7	8	-	-	-	-	-
gALL_v3_c12848	4	3	-	-	-	-	-
gALL_v3_c12849	2	5	-	-	-	-	-
gALL_v3_c1285	30	35	-	-	ref|XP_004931810.1|	PREDICTED: uncharacterized protein LOC101742852 [Bombyx mori]	1,00E-64
gALL_v3_c12850	1	6	-	-	ref|XP_003704615.1|	PREDICTED: kinesin-like protein KIF13B-like [Megachile rotundata]	3,00E-92
gALL_v3_c12852	3	3	-	-	-	-	-
gALL_v3_c12853	1	4	-	-	ref|XP_003394202.1|	PREDICTED: sterile alpha and TIR motif-containing protein 1-like [Bombus terrestris]	2,00E-06
gALL_v3_c12854	6	10	gnl|CDD|73223	cd00653, RNA_pol_B_RPB2, R	dbj|BAJ78712.1|	RNA polymerase II second largest subunit [Reticulitermes speratus]	0
gALL_v3_c12855	5	5	-	-	ref|XP_002423004.1|	Jumonji/ARID domain-containing protein 1D, putative [Pediculus humanus corporis] gb|EEB10266.1| Jumonji/ARID domain-containing protein 1D, putative [Pediculus hum	1,00E-14
gALL_v3_c12856	1	4	-	-	-	-	-
gALL_v3_c12857	4	2	-	-	-	-	-
gALL_v3_c12858	1	8	-	-	-	-	-
gALL_v3_c12859	5	4	-	-	-	-	-
gALL_v3_c1286	11	18	-	-	-	-	-
gALL_v3_c12860	9	7	gnl|CDD|58171	cd03505, Delta9-FADS-like, Th	gb|AAK25796.1|AF3384	delta-9 desaturase 1 [Acheta domesticus] gb|AAK25797.1|AF338466_1 delta-9 desaturase 3 [Acheta domesticus]	9,00E-52
gALL_v3_c12862	4	5	-	-	-	-	-
gALL_v3_c12863	6	11	-	-	ref|XP_006622570.1|	PREDICTED: protein Gawky-like [Apis dorsata]	1,00E-62
gALL_v3_c12865	2	8	-	-	-	-	-
gALL_v3_c12866	9	10	-	-	-	-	-
gALL_v3_c12868	4	3	-	-	-	-	-
gALL_v3_c12869	9	9	gnl|CDD|29261	cd00204, ANK, ankyrin repeat:	ref|XP_002431393.1|	ankyrin repeat-containing protein, putative [Pediculus humanus corporis] gb|EEB18655.1| ankyrin repeat-containing protein, putative [Pediculus humanus corporis]	1,00E-113
gALL_v3_c12870	3	8	-	-	ref|XP_006571257.1|	PREDICTED: patched domain-containing protein 3-like isoform X2 [Apis mellifera]	4,00E-37
gALL_v3_c12871	19	18	-	-	gb|ENN77179.1|	hypothetical protein YQE_06317, partial [Dendroctonus ponderosae]	3,00E-21
gALL_v3_c12872	3	4	-	-	gb|ESO12521.1|	hypothetical protein HELRODRAFT_159075 [Helobdella robusta]	5,00E-20
gALL_v3_c12873	2	4	-	-	gb|ETN67205.1|	syntaxin [Anopheles darlingi]	5,00E-26
gALL_v3_c12874	3	6	-	-	-	-	-
gALL_v3_c12875	3	4	-	-	ref|XP_002430718.1|	conserved hypothetical protein [Pediculus humanus corporis] gb|EEB17980.1| conserved hypothetical protein [Pediculus humanus corporis]	6,00E-70
gALL_v3_c12876	1	8	gnl|CDD|73156	cd01650, RT_nLTR_like, RT_nL	gb|EFA12829.1|	hypothetical protein TcasGA2_TC015881 [Tribolium castaneum]	2,00E-25
gALL_v3_c12877	2	5	-	-	-	-	-
gALL_v3_c1288	11	6	gnl|CDD|34912	COG5333, CCL1, Cdk activating	gb|AEE61842.1|	unknown [Dendroctonus ponderosae] gb|ENN79461.1| hypothetical protein YQE_04105, partial [Dendroctonus ponderosae] gb|ERL91750.1| hypothetical protein D910_(	1,00E-100
gALL_v3_c12880	5	3	-	-	-	-	-
gALL_v3_c12881	2	3	-	-	-	-	-
gALL_v3_c12882	1	4	-	-	-	-	-
gALL_v3_c12883	6	4	-	-	-	-	-
gALL_v3_c12885	4	6	-	-	ref|XP_003399024.1|	PREDICTED: lethal(3)malignant brain tumor-like protein 3-like [Bombus terrestris]	4,00E-19
gALL_v3_c12886	4	6	-	-	-	-	-
gALL_v3_c12887	3	1	-	-	-	-	-
gALL_v3_c1289	21	14	-	-	-	-	-
gALL_v3_c12890	8	4	-	-	-	-	-
gALL_v3_c12891	5	2	-	-	gb|EFN89283.1|	PiggyBac transposable element-derived protein 4 [Harpegnathos saltator]	3,00E-12
gALL_v3_c12892	2	6	-	-	-	-	-
gALL_v3_c12893	2	5	-	-	-	-	-
gALL_v3_c12894	11	20	-	-	-	-	-
gALL_v3_c12895	3	3	-	-	-	-	-
gALL_v3_c12896	4	2	-	-	-	-	-
gALL_v3_c12897	3	1	-	-	-	-	-
gALL_v3_c12899	4	4	-	-	-	-	-
gALL_v3_c129	16	17	-	-	-	-	-
gALL_v3_c1290	4	8	gnl|CDD|190537	pfam03137, OATP, Organic Ani	gb|ADB25332.1|	LP02178p [Drosophila melanogaster]	1,00E-103
gALL_v3_c12900	4	3	gnl|CDD|190008	pfam01490, Aa_trans, Transm	ref|XP_001845151.1|	proton-coupled amino acid transporter 1 [Culex quinquefasciatus] gb|EDS39315.1| proton-coupled amino acid transporter 1 [Culex quinquefasciatus]	1,00E-52
gALL_v3_c12901	3	6	-	-	-	-	-
gALL_v3_c12904	10	8	-	-	-	-	-

gALL_v3_c12905	1	3	-	-	-	-	-
gALL_v3_c12906	3	3	-	-	-	-	-
gALL_v3_c12907	8	6	-	-	-	-	-
gALL_v3_c12908	0	5	-	-	-	-	-
gALL_v3_c1291	5	6	-	-	-	-	-
gALL_v3_c12910	6	5	-	-	-	-	-
gALL_v3_c12911	1	7	-	-	-	-	-
gALL_v3_c12913	6	3	-	-	ref|XP_002428038.1|	reverse transcriptase, putative [Pediculus humanus corporis] gb|EEB15300.1| reverse transcriptase, putative [Pediculus humanus corporis]	6,00E-24
gALL_v3_c12914	4	5	gnl|CDD|133376	cd04176, Rap2, Rap2 subgrou	ref|XP_003428169.1|	PREDICTED: ras-related protein Rap-2c-like isoform 1 [Nasonia vitripennis] ref|XP_003428170.1| PREDICTED: ras-related protein Rap-2c-like isoform 2 [Nasonia vitripennis	1,00E-111
gALL_v3_c12915	8	8	-	-	-	-	-
gALL_v3_c12916	1	5	-	-	-	-	-
gALL_v3_c12917	5	3	-	-	-	-	-
gALL_v3_c12918	4	12	-	-	-	-	-
gALL_v3_c12919	5	2	gnl|CDD|190673	pfam03556, DUF298, Domain	ref|XP_974920.1|	PREDICTED: similar to defective in cullin neddylation protein [Tribolium castaneum]	3,00E-96
gALL_v3_c12920	3	3	-	-	-	-	-
gALL_v3_c12921	4	3	-	-	ref|XP_003700188.1|	PREDICTED: ATP-dependent RNA helicase DDX54-like [Megachile rotundata]	2,00E-57
gALL_v3_c12922	5	7	-	-	-	-	-
gALL_v3_c12923	6	3	-	-	gb|EFX80681.1|	hypothetical protein DAPPUDRAFT_318379 [Daphnia pulex]	8,00E-11
gALL_v3_c12924	2	3	-	-	-	-	-
gALL_v3_c12925	2	3	-	-	-	-	-
gALL_v3_c12926	6	8	-	-	ref|XP_006568826.1|	PREDICTED: uncharacterized protein LOC409034 [Apis mellifera]	2,00E-23
gALL_v3_c12928	2	4	-	-	ref|XP_002423002.1|	conserved hypothetical protein [Pediculus humanus corporis] gb|EEB10264.1| conserved hypothetical protein [Pediculus humanus corporis]	2,00E-14
gALL_v3_c12929	3	2	-	-	-	-	-
gALL_v3_c1293	11	8	gnl|CDD|189635	pfam00621, RhoGEF, RhoGEF (	ref|XP_974773.2|	PREDICTED: similar to guanine nucleotide exchange factor [Tribolium castaneum]	1,00E-110
gALL_v3_c12931	9	10	-	-	-	-	-
gALL_v3_c12932	3	8	-	-	-	-	-
gALL_v3_c12933	6	9	-	-	-	-	-
gALL_v3_c12934	3	2	-	-	-	-	-
gALL_v3_c12935	4	2	-	-	-	-	-
gALL_v3_c12936	4	3	-	-	-	-	-
gALL_v3_c12937	3	8	gnl|CDD|109257	pfam00191, Annexin, Annexin	ref|XP_006621281.1|	PREDICTED: annexin-B9-like [Apis dorsata]	6,00E-47
gALL_v3_c12938	2	5	-	-	-	-	-
gALL_v3_c12939	0	4	-	-	-	-	-
gALL_v3_c1294	7	5	gnl|CDD|73065	cd02659, peptidase_C19C, A s	gb|EFZ17462.1|	hypothetical protein SINV_02027 [Solenopsis invicta]	2,00E-90
gALL_v3_c12940	2	4	-	-	-	-	-
gALL_v3_c12941	2	8	-	-	-	-	-
gALL_v3_c12942	3	7	-	-	-	-	-
gALL_v3_c12943	0	4	-	-	-	-	-
gALL_v3_c12944	8	6	-	-	dbj|BAC82628.1|	pol-like protein [Anopheles gambiae]	2,00E-14
gALL_v3_c12946	2	4	-	-	-	-	-
gALL_v3_c12947	2	6	gnl|CDD|150785	pfam10152, DUF2360, Predict	ref|NP_956467.1|	WASH complex subunit CCDC53 [Danio rerio] gb|AAH45438.1| Coiled-coil domain containing 53 [Danio rerio]	3,00E-48
gALL_v3_c12948	2	3	-	-	-	-	-
gALL_v3_c12949	5	5	-	-	gb|EFZ18720.1|	hypothetical protein SINV_05077 [Solenopsis invicta]	1,00E-101
gALL_v3_c1295	2	9	-	-	ref|XP_003400345.1|	PREDICTED: bombesin receptor subtype-3-like [Bombus terrestris]	5,00E-11
gALL_v3_c12950	4	4	-	-	-	-	-
gALL_v3_c12951	4	4	-	-	ref|XP_003704064.1|	PREDICTED: zinc finger protein 346-like [Megachile rotundata]	7,00E-14
gALL_v3_c12953	14	31	-	-	-	-	-
gALL_v3_c12954	9	14	-	-	-	-	-
gALL_v3_c12955	0	5	-	-	-	-	-
gALL_v3_c12956	4	4	-	-	-	-	-
gALL_v3_c12957	4	2	-	-	-	-	-
gALL_v3_c12958	6	5	-	-	gb|EFN66655.1|	Putative uncharacterized protein FLJ37770 [Camponotus floridanus]	7,00E-25
gALL_v3_c12959	2	4	-	-	-	-	-
gALL_v3_c1296	7	11	gnl|CDD|111019	pfam02077, SURF4, SURF4 fan	ref|XP_002425633.1|	surfeit locus protein, putative [Pediculus humanus corporis] gb|EEB12895.1| surfeit locus protein, putative [Pediculus humanus corporis]	3,00E-56
gALL_v3_c12960	3	8	-	-	-	-	-
gALL_v3_c12961	3	4	-	-	ref|XP_969616.2|	PREDICTED: similar to pre-mRNA splicing factor ATP-dependent RNA helicase PRP16 [Tribolium castaneum] gb|EFA00983.1| hypothetical protein TcasGA2_TC003896 [Tribc	4,00E-59
gALL_v3_c12963	2	5	-	-	-	-	-
gALL_v3_c12964	3	3	gnl|CDD|73154	cd01647, RT_LTR, RT_LTR: Rev	ref|XP_003389166.1|	PREDICTED: hypothetical protein LOC100636756 [Amphimedon queenslandica]	3,00E-39
gALL_v3_c12965	2	1	-	-	-	-	-
gALL_v3_c12966	4	5	gnl|CDD|193497	cd03875, M28_Fxna_like, M2ξ	gb|EFA07296.1|	hypothetical protein TcasGA2_TC014536 [Tribolium castaneum]	6,00E-73
gALL_v3_c12967	4	6	-	-	-	-	-
gALL_v3_c12968	3	7	-	-	gb|EFN70365.1|	DnaJ-like protein subfamily C member 21 [Camponotus floridanus]	6,00E-97
gALL_v3_c12969	4	3	-	-	-	-	-
gALL_v3_c1297	10	11	gnl|CDD|34673	COG5069, SAC6, Ca2+-binding	gb|AGM32534.1|	alpha actinin, partial [Coptotermes formosanus]	1,00E-132
gALL_v3_c12970	4	11	gnl|CDD|190314	pfam02453, Reticulon, Reticul	gb|EFN73447.1|	Reticulon-1 [Camponotus floridanus]	2,00E-35
gALL_v3_c12971	0	4	-	-	-	-	-
gALL_v3_c12972	1	4	-	-	-	-	-
gALL_v3_c12973	5	2	-	-	-	-	-
gALL_v3_c12974	1	4	-	-	gb|EFA03728.1|	hypothetical protein TcasGA2_TC013837 [Tribolium castaneum]	6,00E-30
gALL_v3_c12976	3	2	-	-	-	-	-
gALL_v3_c12977	6	7	-	-	-	-	-
gALL_v3_c12978	6	13	-	-	-	-	-
gALL_v3_c12980	1	4	-	-	-	-	-
gALL_v3_c12982	4	4	-	-	-	-	-
gALL_v3_c12983	2	7	-	-	-	-	-
gALL_v3_c12984	5	3	-	-	-	-	-
gALL_v3_c12985	4	5	-	-	-	-	-
gALL_v3_c12986	5	3	-	-	-	-	-
gALL_v3_c12987	3	3	gnl|CDD|128734	smart00458, RICIN, Ricin-type	gb|EFN85329.1|	Polypeptide N-acetylgalactosaminyltransferase 1 [Harpegnathos saltator]	2,00E-10
gALL_v3_c12988	3	2	-	-	gb|EFN66655.1|	Putative uncharacterized protein FLJ37770 [Camponotus floridanus]	8,00E-21
gALL_v3_c1299	12	8	-	-	-	-	-
gALL_v3_c12991	7	4	-	-	-	-	-
gALL_v3_c12992	2	3	-	-	-	-	-
gALL_v3_c12994	3	2	-	-	-	-	-
gALL_v3_c12996	2	4	-	-	-	-	-
gALL_v3_c12998	3	7	gnl|CDD|162740	TIGR02169, SMC_prok_A, chrc	ref|XP_003397729.1|	PREDICTED: hypothetical protein LOC100648680 [Bombus terrestris]	9,00E-33
gALL_v3_c12999	4	5	-	-	-	-	-
gALL_v3_c13	3	7	-	-	-	-	-
gALL_v3_c130	26	34	gnl|CDD|34718	COG5116, RPN2, 26S proteasc	ref|XP_001949774.2|	PREDICTED: 26S proteasome non-ATPase regulatory subunit 1-like isoform 1 [Acyrthosiphon pisum]	0
gALL_v3_c1300	2	9	gnl|CDD|189522	pfam00375, SDF, Sodium:dicar	gb|EFN74756.1|	Excitatory amino acid transporter [Camponotus floridanus]	2,00E-47
gALL_v3_c13000	4	4	-	-	-	-	-
gALL_v3_c13001	2	2	-	-	-	-	-
gALL_v3_c13002	2	2	gnl|CDD|191009	pfam04502, DUF572, Family o	ref|XP_320235.4|	AGAP012306-PA [Anopheles gambiae str. PEST] gb|EAA00389.4| AGAP012306-PA [Anopheles gambiae str. PEST]	6,00E-13
gALL_v3_c13003	3	3	-	-	-	-	-
gALL_v3_c13004	6	4	-	-	-	-	-
gALL_v3_c13005	24	19	gnl|CDD|148161	pfam06388, DUF1075, Protein	gb|ENN81569.1|	hypothetical protein YQE_02098, partial [Dendroctonus ponderosae] gb|ERL91834.1| hypothetical protein D910_09159 [Dendroctonus ponderosae]	1,00E-16
gALL_v3_c13006	2	4	-	-	-	-	-
gALL_v3_c13007	7	5	-	-	-	-	-
gALL_v3_c13008	1	6	-	-	-	-	-
gALL_v3_c13009	5	9	gnl|CDD|149494	pfam08454, RIH_assoc, RyR ar	gb|EFN73871.1|	Inositol 1,4,5-trisphosphate receptor [Camponotus floridanus]	1,00E-138
gALL_v3_c1301	12	12	-	-	-	-	-
gALL_v3_c13010	4	5	gnl|CDD|79366	cd03684, ClC_3_like, ClC-3-like	ref|XP_002431982.1|	Chloride channel protein, putative [Pediculus humanus corporis] gb|EEB19244.1| Chloride channel protein, putative [Pediculus humanus corporis]	1,00E-115
gALL_v3_c13011	3	4	-	-	-	-	-
gALL_v3_c13013	2	4	gnl|CDD|192930	pfam12066, DUF3546, Domair	ref|XP_003699267.1|	PREDICTED: serrate RNA effector molecule homolog [Megachile rotundata]	4,00E-42
gALL_v3_c13014	1	4	-	-	-	-	-
gALL_v3_c13015	3	3	-	-	-	-	-
gALL_v3_c13016	3	2	-	-	ref|XP_006132930.1|	PREDICTED: jerky protein-like [Pelodiscus sinensis]	3,00E-14
gALL_v3_c13017	2	2	-	-	-	-	-
gALL_v3_c13018	7	5	gnl|CDD|189551	pfam00443, UCH, Ubiquitin ca	gb|EHJ72623.1|	hypothetical protein KGM_20167 [Danaus plexippus]	1,00E-119
gALL_v3_c1302	19	30	gnl|CDD|189008	cd09601, M1_APN_2, Peptida:	gb|EFN87052.1|	Aminopeptidase N [Harpegnathos saltator]	0
gALL_v3_c13021	5	4	-	-	gb|EFN84466.1|	F-box only protein 33 [Harpegnathos saltator]	6,00E-65
gALL_v3_c13022	4	7	gnl|CDD|178658	PLN03111, PLN03111, DNA-di	ref|XP_623541.1|	PREDICTED: DNA-directed RNA polymerases I, II, and III subunit RPABC1-like [Apis mellifera]	1,00E-88
gALL_v3_c13025	2	3	gnl|CDD|178687	PLN03142, PLN03142, Probabl	ref|XP_001508174.2|	PREDICTED: DNA excision repair protein ERCC-6 [Ornithorhynchus anatinus]	1,00E-100
gALL_v3_c13026	3	11	-	-	ref|XP_396872.3|	PREDICTED: uncharacterized protein LOC413428 [Apis mellifera]	4,00E-39
gALL_v3_c13028	6	3	-	-	ref|XP_006634714.1|	PREDICTED: hemicentin-1-like [Lepisosteus oculatus]	2,00E-28
gALL_v3_c13029	4	8	-	-	gb|AGI96987.1|	tropomodulin [Nilaparvata lugens]	3,00E-17
gALL_v3_c1303	15	18	gnl|CDD|176395	cd01800, SF3a120_C, Ubiquiti	ref|XP_001607229.1|	PREDICTED: splicing factor 3A subunit 1-like [Nasonia vitripennis]	1,00E-33
gALL_v3_c13030	4	4	-	-	-	-	-
gALL_v3_c13031	5	3	-	-	-	-	-
gALL_v3_c13032	5	5	-	-	ref|XP_003398262.1|	PREDICTED: LOW QUALITY PROTEIN: beta-catenin-like protein 1-like [Bombus terrestris] ref|XP_003486015.1| PREDICTED: beta-catenin-like protein 1-like [Bombus impatie	1,00E-151
gALL_v3_c13033	4	2	-	-	-	-	-
gALL_v3_c13034	6	4	-	-	-	-	-
gALL_v3_c13035	1	5	gnl|CDD|177212	MTH00155, COX3, cytochrome	ref|YP_006503953.1|	cytochrome c oxidase subunit III (mitochondrion) [Coptotermes lacteus] gb|AFM92453.1| cytochrome c oxidase subunit III (mitochondrion) [Coptotermes lacteus]	5,00E-24
gALL_v3_c13036	1	4	-	-	-	-	-
gALL_v3_c13037	4	7	-	-	-	-	-
gALL_v3_c13038	4	8	gnl|CDD|162054	TIGR00815, sulP, high affinity s	ref|XP_003708291.1|	PREDICTED: sodium-independent sulfate anion transporter-like [Megachile rotundata]	2,00E-93
gALL_v3_c13039	5	7	gnl|CDD|189411	pfam00135, COesterase, Carb	gb|AGT57960.1|	juvenile hormone esterase, partial [Bemisia tabaci]	5,00E-83
gALL_v3_c1304	3	11	-	-	-	-	-
gALL_v3_c13040	3	3	-	-	-	-	-
gALL_v3_c13041	3	6	-	-	gb|EGI71030.1|	Trimethylguanosine synthase [Acromyrmex echinatior]	4,00E-06
gALL_v3_c13042	3	5	gnl|CDD|189373	pfam00069, Pkinase, Protein k	gb|ADY16729.1|	doubletime [Schistocerca gregaria]	5,00E-75
gALL_v3_c13043	3	3	-	-	-	-	-
gALL_v3_c13044	4	5	-	-	-	-	-
gALL_v3_c13045	2	5	-	-	-	-	-
gALL_v3_c13046	3	4	-	-	-	-	-
gALL_v3_c13047	14	23	-	-	-	-	-
gALL_v3_c13048	3	1	-	-	-	-	-
gALL_v3_c13049	3	5	-	-	-	-	-
gALL_v3_c1305	5	4	gnl|CDD|176070	cd08688, C2_KIAA0528-like, C	ref|XP_006566827.1|	PREDICTED: C2 domain-containing protein 5-like [Apis mellifera]	1,00E-142
gALL_v3_c13051	4	3	-	-	-	-	-
gALL_v3_c13052	7	6	-	-	gb|EFN80576.1|	Putative uncharacterized transposon-derived protein F52C9.6 [Harpegnathos saltator]	2,00E-08
gALL_v3_c13054	3	2	gnl|CDD|162459	TIGR01642, U2AF_lg, U2 snRN	-	-	-
gALL_v3_c13055	2	4	gnl|CDD|173682	cd05591, STKc_nPKC_epsilon,	ref|XP_002431153.1|	protein kinase C, putative [Pediculus humanus corporis] gb|EEB18415.1| protein kinase C, putative [Pediculus humanus corporis]	1,00E-112
gALL_v3_c13056	4	3	-	-	-	-	-

gALL_v3_c13057	1	4	-	-	-	-	-
gALL_v3_c13058	7	4	-	-	-	-	-
gALL_v3_c1306	6	4	-	-	ref|XP_001948864.2|	PREDICTED: CD109 antigen-like [Acyrthosiphon pisum]	1,00E-105
gALL_v3_c13060	3	4	-	-	-	-	-
gALL_v3_c13061	3	5	-	-	-	-	-
gALL_v3_c13062	6	5	gnl|CDD|177210	MTH00153, COX1, cytochrome	gb|AEG80123.1|	cytochrome oxidase subunit I [Coptotermes formosanus]	1,00E-23
gALL_v3_c13063	7	16	-	-	-	-	-
gALL_v3_c13064	5	4	gnl|CDD|32119	COG1936, COG1936, Predicted	gb|AGM32925.1|	TATA box binding protein (TBP)-associated factor [Coptotermes formosanus]	1,00E-120
gALL_v3_c13065	3	3	-	-	ref|XP_395930.3|	PREDICTED: 39S ribosomal protein L28, mitochondrial [Apis mellifera]	2,00E-70
gALL_v3_c13066	1	6	-	-	-	-	-
gALL_v3_c13067	2	4	-	-	gb|ADI61823.1|	endonuclease-reverse transcriptase [Bombyx mori]	2,00E-09
gALL_v3_c13068	12	14	-	-	-	-	-
gALL_v3_c13069	4	7	-	-	-	-	-
gALL_v3_c1307	8	20	gnl|CDD|193286	pfam12810, Gly_rich, Glycine	-	-	-
gALL_v3_c13070	3	2	-	-	-	-	-
gALL_v3_c13071	1	4	gnl|CDD|191333	pfam05649, Peptidase_M13_N	gb|EFZ17786.1|	hypothetical protein SINV_13863 [Solenopsis invicta]	3,00E-32
gALL_v3_c13072	4	2	-	-	-	-	-
gALL_v3_c13073	2	5	-	-	-	-	-
gALL_v3_c13074	3	2	-	-	-	-	-
gALL_v3_c13076	2	5	-	-	-	-	-
gALL_v3_c13077	2	4	-	-	ref|XP_001602111.2|	PREDICTED: probable cytochrome P450 4aa1 [Nasonia vitripennis]	2,00E-07
gALL_v3_c13078	5	7	-	-	gb|EFN81456.1|	Serine/threonine-protein phosphatase 2A 65 kDa regulatory subunit A alpha isoform [Harpegnathos saltator]	1,00E-97
gALL_v3_c13080	3	2	gnl|CDD|34870	COG5273, COG5273, Uncharac	ref|XP_001651194.1|	hypothetical protein AaeL_AAEL015257 [Aedes aegypti] gb|EAT32590.1| AAEL015257-PA, partial [Aedes aegypti]	8,00E-53
gALL_v3_c13081	14	18	-	-	-	-	-
gALL_v3_c13082	3	5	-	-	-	-	-
gALL_v3_c13083	7	10	-	-	gb|ESN97862.1|	hypothetical protein HELRODRAFT_177498 [Helobdella robusta]	9,00E-16
gALL_v3_c13084	3	2	-	-	gb|EHJ73936.1|	hypothetical protein KGM_00497 [Danaus plexippus]	6,00E-27
gALL_v3_c13086	5	4	gnl|CDD|187667	cd09807, retinol-DH_like_SDR	gb|AGM32767.1|	retinol dehydrogenase 12-like protein, partial [Coptotermes formosanus]	1,00E-156
gALL_v3_c13087	6	10	-	-	ref|XP_006029730.1|	PREDICTED: histone-lysine N-methyltransferase SETMAR-like [Alligator sinensis]	7,00E-09
gALL_v3_c13089	10	12	gnl|CDD|34654	COG5049, XRN1, 5'-3' exonucl	ref|XP_002120271.2|	PREDICTED: 5'-3' exoribonuclease 1 [Ciona intestinalis]	0
gALL_v3_c1309	8	6	-	-	-	-	-
gALL_v3_c13090	13	17	gnl|CDD|176289	cd01213, tensin, Tensin Phosp	gb|EFN63652.1|	Tensin [Camponotus floridanus]	1,00E-123
gALL_v3_c13091	1	6	-	-	-	-	-
gALL_v3_c13092	5	3	-	-	ref|XP_001605441.1|	PREDICTED: protein sidekick-1-like [Nasonia vitripennis]	1,00E-43
gALL_v3_c13093	0	4	gnl|CDD|128627	smart00332, PP2Cc, Serine/th	ref|XP_967669.1|	PREDICTED: similar to protein phosphatase 2c [Tribolium castaneum] gb|EFA05077.1| hypothetical protein TcasGA2_TC015174 [Tribolium castaneum]	2,00E-50
gALL_v3_c13095	3	5	-	-	ref|XP_002723545.1|	PREDICTED: hypothetical protein [Oryctolagus cuniculus]	2,00E-17
gALL_v3_c13096	1	2	-	-	-	-	-
gALL_v3_c13097	9	8	-	-	ref|XP_002429399.1|	mahogunin, putative [Pediculus humanus corporis] gb|EEB16661.1| mahogunin, putative [Pediculus humanus corporis]	7,00E-20
gALL_v3_c13098	7	18	-	-	-	-	-
gALL_v3_c13099	2	3	-	-	-	-	-
gALL_v3_c13102	3	4	-	-	-	-	-
gALL_v3_c13103	4	2	-	-	-	-	-
gALL_v3_c13104	3	3	gnl|CDD|192640	pfam10602, RPN7, 26S protea	gb|EFN86625.1|	26S proteasome non-ATPase regulatory subunit 6 [Harpegnathos saltator]	3,00E-91
gALL_v3_c13106	7	6	-	-	gb|EFN74649.1|	Histone-lysine N-methyltransferase SETMAR [Camponotus floridanus]	7,00E-13
gALL_v3_c13107	1	5	-	-	-	-	-
gALL_v3_c13108	2	3	-	-	-	-	-
gALL_v3_c13109	2	4	-	-	-	-	-
gALL_v3_c1311	10	16	gnl|CDD|189428	pfam00160, Pro_isomerase, C	ref|XP_001606888.2|	PREDICTED: hypothetical protein LOC100119773 [Nasonia vitripennis]	2,00E-85
gALL_v3_c13110	3	2	-	-	-	-	-
gALL_v3_c13111	2	2	-	-	ref|XP_002428673.1|	hypothetical protein Phum_PHUM397020 [Pediculus humanus corporis] gb|EEB15935.1| hypothetical protein Phum_PHUM397020 [Pediculus humanus corporis]	1,00E-33
gALL_v3_c13112	4	9	-	-	-	-	-
gALL_v3_c13113	5	9	-	-	ref|XP_006816497.1|	PREDICTED: condensin-2 complex subunit D3-like [Saccoglossus kowalevskii]	1,00E-17
gALL_v3_c13114	4	3	-	-	gb|EFN71868.1|	Probable ubiquitin carboxyl-terminal hydrolase FAF-X [Camponotus floridanus]	1,00E-105
gALL_v3_c13115	12	13	gnl|CDD|144732	pfam01248, Ribosomal_L7Ae,	gb|AAM33784.1|	ribosomal protein S12 [Periplaneta americana]	1,00E-71
gALL_v3_c13116	12	11	gnl|CDD|29257	cd00200, WD40, WD40 domai	ref|XP_002428753.1|	rabconnectin, putative [Pediculus humanus corporis] gb|EEB16015.1| rabconnectin, putative [Pediculus humanus corporis]	0
gALL_v3_c13117	2	9	-	-	ref|XP_001951286.2|	PREDICTED: focal adhesion kinase 1-like [Acyrthosiphon pisum]	1,00E-88
gALL_v3_c13118	1	4	-	-	-	-	-
gALL_v3_c1312	7	4	-	-	ref|XP_003248149.1|	PREDICTED: hypothetical protein LOC100575747 [Acyrthosiphon pisum]	2,00E-10
gALL_v3_c13123	3	3	-	-	-	-	-
gALL_v3_c13124	2	5	-	-	-	-	-
gALL_v3_c13125	5	4	-	-	-	-	-
gALL_v3_c13126	3	3	-	-	-	-	-
gALL_v3_c13127	3	3	-	-	gb|EEZ99133.1|	hypothetical protein TcasGA2_TC012914 [Tribolium castaneum]	7,00E-09
gALL_v3_c13128	3	4	-	-	-	-	-
gALL_v3_c13129	1	4	-	-	-	-	-
gALL_v3_c1313	5	8	gnl|CDD|162093	TIGR00892, 2A0113, monocar	ref|XP_003396980.1|	PREDICTED: hypothetical protein LOC100645876 [Bombus terrestris]	1,00E-115
gALL_v3_c13130	3	5	gnl|CDD|189000	cd08662, M13, Peptidase fami	ref|XP_001654072.1|	neprilysin [Aedes aegypti] gb|EAT38188.1| AAEL009895-PA, partial [Aedes aegypti]	1,00E-105
gALL_v3_c13131	9	7	gnl|CDD|146145	pfam03357, Snf7, Snf7. This fa	gb|AGM32114.1|	chromatin modifying protein 1B-like protein [Coptotermes formosanus]	4,00E-97
gALL_v3_c13132	5	8	gnl|CDD|189372	pfam00067, p450, Cytochrome	gb|AGQ48062.1|	cytochrome P450 monooxygenase [Coptotermes formosanus]	1,00E-130
gALL_v3_c13133	2	9	-	-	gb|ENN72821.1|	hypothetical protein YQE_10624, partial [Dendroctonus ponderosae]	2,00E-12
gALL_v3_c13135	3	6	-	-	-	-	-
gALL_v3_c13136	5	8	-	-	ref|XP_003702676.1|	PREDICTED: autophagy-related protein 2 homolog A [Megachile rotundata]	4,00E-88
gALL_v3_c13137	3	4	-	-	ref|XP_001599623.2|	PREDICTED: hypothetical protein LOC100114689 [Nasonia vitripennis]	2,00E-10
gALL_v3_c13138	3	10	-	-	-	-	-
gALL_v3_c13139	3	3	gnl|CDD|178917	PRK00179, pgi, glucose-6-pho	gb|EFN82269.1|	Glucose-6-phosphate isomerase [Harpegnathos saltator]	1,00E-32
gALL_v3_c1314	13	9	-	-	-	-	-
gALL_v3_c13140	2	5	-	-	-	-	-
gALL_v3_c13141	3	3	gnl|CDD|149439	pfam08375, Rpn3_C, Proteaso	ref|XP_001605959.1|	PREDICTED: probable 26S proteasome non-ATPase regulatory subunit 3-like [Nasonia vitripennis]	6,00E-48
gALL_v3_c13142	17	12	gnl|CDD|189507	pfam00335, Tetraspannin, Tetr	gb|AGM32463.1|	tetraspanin protein [Coptotermes formosanus]	1,00E-35
gALL_v3_c13144	4	2	gnl|CDD|162130	TIGR00955, 3a01204, The Eye	ref|XP_973458.1|	PREDICTED: similar to ABC transporter expressed in trachea CG2969-PD [Tribolium castaneum] gb|EFA02575.1| hypothetical protein TcasGA2_TC008293 [Tribolium castan	2,00E-35
gALL_v3_c13146	6	4	-	-	-	-	-
gALL_v3_c13147	5	4	-	-	-	-	-
gALL_v3_c13148	3	2	gnl|CDD|29257	cd00200, WD40, WD40 domai	ref|XP_003700406.1|	PREDICTED: cleavage stimulation factor subunit 1-like [Megachile rotundata]	3,00E-75
gALL_v3_c13149	4	3	-	-	ref|XP_002429256.1|	28S ribosomal protein S35, putative [Pediculus humanus corporis] gb|EEB16518.1| 28S ribosomal protein S35, putative [Pediculus humanus corporis]	4,00E-65
gALL_v3_c1315	11	24	gnl|CDD|193379	pfam12906, RINGv, RING-varia	ref|XP_001602313.2|	PREDICTED: hypothetical protein LOC100118313 [Nasonia vitripennis]	0
gALL_v3_c13150	2	8	-	-	ref|XP_788643.3|	PREDICTED: exportin-4-like, partial [Strongylocentrotus purpuratus]	2,00E-11
gALL_v3_c13151	1	5	-	-	ref|XP_002429272.1|	G-protein signaling modulator, putative [Pediculus humanus corporis] gb|EEB16534.1| G-protein signaling modulator, putative [Pediculus humanus corporis]	1,00E-106
gALL_v3_c13152	10	6	gnl|CDD|148286	pfam06585, JHBP, Haemolymp	gb|AGM32526.1|	hemolymph juvenile hormone binding protein (JHBP) [Coptotermes formosanus]	1,00E-116
gALL_v3_c13153	3	2	gnl|CDD|144172	pfam00479, G6PD_N, Glucose	gb|ERL94809.1|	hypothetical protein D910_12083 [Dendroctonus ponderosae]	1,00E-31
gALL_v3_c13154	2	3	-	-	-	-	-
gALL_v3_c13155	6	5	-	-	gb|EFN70829.1|	E3 ubiquitin-protein ligase MARCH6 [Camponotus floridanus]	8,00E-77
gALL_v3_c13156	3	3	gnl|CDD|180194	PRK05678, PRK05678, succiny	dbj|BAN20393.1|	succinyl-CoA synthetase small subunit, putative [Riptortus pedestris]	3,00E-26
gALL_v3_c13157	0	5	-	-	-	-	-
gALL_v3_c13158	2	4	-	-	-	-	-
gALL_v3_c13159	11	9	-	-	-	-	-
gALL_v3_c13160	7	4	-	-	-	-	-
gALL_v3_c13161	3	2	-	-	gb|ADC53707.1|	unknown [Blattella germanica]	6,00E-15
gALL_v3_c13162	7	4	-	-	-	-	-
gALL_v3_c13163	10	9	-	-	-	-	-
gALL_v3_c13166	9	19	-	-	-	-	-
gALL_v3_c13167	2	3	-	-	-	-	-
gALL_v3_c13169	6	11	-	-	ref|XP_001602330.2|	PREDICTED: hypothetical protein LOC100118338 isoform 1 [Nasonia vitripennis]	1,00E-101
gALL_v3_c1317	6	6	gnl|CDD|129037	smart00801, dDENN, Domain	ref|XP_975086.2|	PREDICTED: similar to rab3-GEF CG5627-PB [Tribolium castaneum]	3,00E-91
gALL_v3_c13170	2	3	-	-	-	-	-
gALL_v3_c13171	10	8	gnl|CDD|28964	cd00083, HLH, Helix-loop-heli	ref|XP_975837.2|	PREDICTED: similar to Mitf CG17469-PB [Tribolium castaneum] gb|EFA11511.1| hypothetical protein TcasGA2_TC014225 [Tribolium castaneum]	1,00E-95
gALL_v3_c13172	3	10	gnl|CDD|152498	pfam12063, DUF3543, Domair	gb|EFN88339.1|	Serine/threonine-protein kinase ULK2 [Harpegnathos saltator]	1,00E-64
gALL_v3_c13173	3	10	gnl|CDD|119026	pfam10506, MCC-bdg_PDZ, PI	gb|EKC32433.1|	Colorectal mutant cancer protein [Crassostrea gigas]	1,00E-53
gALL_v3_c13174	4	6	-	-	-	-	-
gALL_v3_c13175	2	7	-	-	ref|XP_393422.3|	PREDICTED: protein FAM69C-like isoform X3 [Apis mellifera]	1,00E-119
gALL_v3_c13178	2	3	-	-	-	-	-
gALL_v3_c1318	3	8	-	-	-	-	-
gALL_v3_c13180	5	3	-	-	ref|XP_002428022.1|	tetracycline-efflux transporter, putative [Pediculus humanus corporis] gb|EEB15284.1| tetracycline-efflux transporter, putative [Pediculus humanus corporis]	1,00E-23
gALL_v3_c13181	6	6	-	-	-	-	-
gALL_v3_c13182	18	14	-	-	-	-	-
gALL_v3_c13183	3	2	gnl|CDD|190246	pfam02210, Laminin_G_2, Lan	ref|XP_002423159.1|	class D atypical G-protein coupled receptor GPRstn1, putative [Pediculus humanus corporis] gb|EEB10421.1| class D atypical G-protein coupled receptor GPRstn1, putative	1,00E-111
gALL_v3_c13184	4	2	-	-	-	-	-
gALL_v3_c13186	3	3	-	-	-	-	-
gALL_v3_c13187	2	5	gnl|CDD|100104	cd00590, RRM, RRM (RNA reco	ref|XP_003699870.1|	PREDICTED: RNA binding protein fox-1 homolog 2-like, partial [Megachile rotundata]	3,00E-67
gALL_v3_c13188	2	3	-	-	gb|AGM32368.1|	n-acetyltransferase-like protein [Coptotermes formosanus]	1,00E-40
gALL_v3_c13189	5	5	-	-	-	-	-
gALL_v3_c13190	3	5	-	-	-	-	-
gALL_v3_c13191	7	12	-	-	-	-	-
gALL_v3_c13192	4	4	-	-	-	-	-
gALL_v3_c13195	6	3	gnl|CDD|163638	cd07395, MPP_CSTP1, Homo s	ref|XP_002416197.1|	purple acid phosphatase, putative [Ixodes scapularis] gb|EEC19864.1| purple acid phosphatase, putative [Ixodes scapularis]	1,00E-12
gALL_v3_c13196	3	5	-	-	-	-	-
gALL_v3_c13197	10	7	-	-	-	-	-
gALL_v3_c13198	1	3	-	-	-	-	-
gALL_v3_c13199	4	2	gnl|CDD|190253	pfam02230, Abhydrolase_2, P	ref|XP_971145.1|	PREDICTED: similar to AGAP004271-PA [Tribolium castaneum] gb|EFA02699.1| hypothetical protein TcasGA2_TC008421 [Tribolium castaneum]	5,00E-54
gALL_v3_c132	9	9	gnl|CDD|143945	pfam00179, UQ_con, Ubiquiti	gb|ENN76847.1|	hypothetical protein YQE_06688, partial [Dendroctonus ponderosae]	3,00E-99
gALL_v3_c1320	18	18	-	-	-	-	-
gALL_v3_c13200	2	3	-	-	ref|XP_006623355.1|	PREDICTED: UDP-N-acetylglucosamine--peptide N-acetylglucosaminyltransferase 110 kDa subunit-like isoform X2 [Apis dorsata]	2,00E-40
gALL_v3_c13201	3	2	-	-	-	-	-
gALL_v3_c13203	3	5	-	-	-	-	-
gALL_v3_c13204	3	8	-	-	-	-	-
gALL_v3_c13205	6	4	-	-	-	-	-
gALL_v3_c13206	6	8	-	-	-	-	-
gALL_v3_c13208	6	11	-	-	-	-	-
gALL_v3_c1321	6	15	-	-	-	-	-
gALL_v3_c13210	4	2	gnl|CDD|189372	pfam00067, p450, Cytochrome	ref|XP_006563996.1|	PREDICTED: cytochrome P450 315a1, mitochondrial isoform X4 [Apis mellifera] ref|XP_006563997.1| PREDICTED: cytochrome P450 315a1, mitochondrial isoform X5 [Apis	3,00E-51
gALL_v3_c13211	3	9	-	-	-	-	-
gALL_v3_c13213	10	14	-	-	-	-	-
gALL_v3_c13214	5	4	-	-	-	-	-
gALL_v3_c13215	2	7	-	-	-	-	-
gALL_v3_c13216	1	7	-	-	-	-	-

<table>
<tr><td></td><td></td><td></td><td></td><td></td><td></td><td></td><td></td></tr>
<tr><td>gALL_v3_c13217</td><td>5</td><td>5</td><td>-</td><td>-</td><td>-</td><td>-</td><td>-</td></tr>
<tr><td>gALL_v3_c13218</td><td>4</td><td>2</td><td>-</td><td>-</td><td>gb|EFN62826.1|</td><td>hypothetical protein EAG_09822 [Camponotus floridanus]</td><td>4,00E-15</td></tr>
<tr><td>gALL_v3_c13219</td><td>3</td><td>3</td><td>gnl|CDD|115057</td><td>pfam06375, BLVR, Bovine leuk</td><td>ref|XP_395563.4|</td><td>PREDICTED: AP-3 complex subunit delta-1 [Apis mellifera]</td><td>1,00E-20</td></tr>
<tr><td>gALL_v3_c1322</td><td>10</td><td>6</td><td>-</td><td>-</td><td>ref|XP_974840.1|</td><td>PREDICTED: similar to harmonin [Tribolium castaneum] gb|EFA01585.1| hypothetical protein TcasGA2_TC007147 [Tribolium castaneum]</td><td>7,00E-13</td></tr>
<tr><td>gALL_v3_c13220</td><td>3</td><td>4</td><td>-</td><td>-</td><td>-</td><td>-</td><td>-</td></tr>
<tr><td>gALL_v3_c13221</td><td>2</td><td>3</td><td>-</td><td>-</td><td>-</td><td>-</td><td>-</td></tr>
<tr><td>gALL_v3_c13223</td><td>5</td><td>4</td><td>-</td><td>-</td><td>-</td><td>-</td><td>-</td></tr>
<tr><td>gALL_v3_c13224</td><td>3</td><td>3</td><td>-</td><td>-</td><td>-</td><td>-</td><td>-</td></tr>
<tr><td>gALL_v3_c13225</td><td>1</td><td>3</td><td>-</td><td>-</td><td>-</td><td>-</td><td>-</td></tr>
<tr><td>gALL_v3_c13226</td><td>5</td><td>7</td><td>gnl|CDD|185474</td><td>PTZ00141, PTZ00141, elongati</td><td>dbj|BAM14416.1|</td><td>elongation factor-1 alpha [Spirotrichonympha leidyi]</td><td>0</td></tr>
<tr><td>gALL_v3_c13227</td><td>2</td><td>2</td><td>gnl|CDD|128671</td><td>smart00389, HOX, Homeodom</td><td>ref|XP_005190591.1|</td><td>PREDICTED: homeobox protein extradenticle-like, partial [Musca domestica]</td><td>4,00E-73</td></tr>
<tr><td>gALL_v3_c13228</td><td>1</td><td>8</td><td>-</td><td>-</td><td>-</td><td>-</td><td>-</td></tr>
<tr><td>gALL_v3_c13229</td><td>8</td><td>8</td><td>-</td><td>-</td><td>gb|EFN83680.1|</td><td>Serine/threonine-protein kinase MARK2 [Harpegnathos saltator]</td><td>8,00E-64</td></tr>
<tr><td>gALL_v3_c13231</td><td>3</td><td>1</td><td>-</td><td>-</td><td>-</td><td>-</td><td>-</td></tr>
<tr><td>gALL_v3_c13232</td><td>2</td><td>3</td><td>-</td><td>-</td><td>-</td><td>-</td><td>-</td></tr>
<tr><td>gALL_v3_c13233</td><td>2</td><td>7</td><td>gnl|CDD|176855</td><td>cd07813, COQ10p_like, Coenz</td><td>gb|AGM32879.1|</td><td>coenzyme Q-binding protein COQ10 [Coptotermes formosanus]</td><td>9,00E-85</td></tr>
<tr><td>gALL_v3_c13234</td><td>5</td><td>10</td><td>gnl|CDD|132742</td><td>cd06944, NR_LBD_Ftz-F1_like,</td><td>gb|EFN79758.1|</td><td>Nuclear hormone receptor FTZ-F1 [Harpegnathos saltator]</td><td>1,00E-19</td></tr>
<tr><td>gALL_v3_c13235</td><td>1</td><td>6</td><td>-</td><td>-</td><td>ref|XP_003395347.1|</td><td>PREDICTED: papilin-like [Bombus terrestris]</td><td>5,00E-67</td></tr>
<tr><td>gALL_v3_c13236</td><td>5</td><td>3</td><td>-</td><td>-</td><td>ref|XP_004930295.1|</td><td>PREDICTED: uncharacterized protein LOC101737452 [Bombyx mori]</td><td>5,00E-13</td></tr>
<tr><td>gALL_v3_c13237</td><td>2</td><td>3</td><td>-</td><td>-</td><td>-</td><td>-</td><td>-</td></tr>
<tr><td>gALL_v3_c13238</td><td>8</td><td>11</td><td>-</td><td>-</td><td>-</td><td>-</td><td>-</td></tr>
<tr><td>gALL_v3_c13239</td><td>5</td><td>4</td><td>-</td><td>-</td><td>-</td><td>-</td><td>-</td></tr>
<tr><td>gALL_v3_c1324</td><td>5</td><td>13</td><td>-</td><td>-</td><td>ref|XP_006559241.1|</td><td>PREDICTED: uncharacterized protein LOC100577972 [Apis mellifera]</td><td>9,00E-39</td></tr>
<tr><td>gALL_v3_c13240</td><td>5</td><td>5</td><td>-</td><td>-</td><td>ref|XP_002422642.1|</td><td>conserved hypothetical protein [Pediculus humanus corporis] gb|EEB09904.1| conserved hypothetical protein [Pediculus humanus corporis]</td><td>1,00E-144</td></tr>
<tr><td>gALL_v3_c13241</td><td>6</td><td>8</td><td>gnl|CDD|162464</td><td>TIGR01649, hnRNP-L_PTB, hnF</td><td>ref|XP_001604099.2|</td><td>PREDICTED: heterogeneous nuclear ribonucleoprotein L-like [Nasonia vitripennis]</td><td>1,00E-86</td></tr>
<tr><td>gALL_v3_c13242</td><td>6</td><td>11</td><td>-</td><td>-</td><td>-</td><td>-</td><td>-</td></tr>
<tr><td>gALL_v3_c13243</td><td>2</td><td>7</td><td>gnl|CDD|100104</td><td>cd00590, RRM, RRM (RNA rec</td><td>dbj|BAN20840.1|</td><td>myelinprotein expression factor [Riptortus pedestris]</td><td>2,00E-59</td></tr>
<tr><td>gALL_v3_c13244</td><td>6</td><td>6</td><td>-</td><td>-</td><td>-</td><td>-</td><td>-</td></tr>
<tr><td>gALL_v3_c13245</td><td>5</td><td>3</td><td>-</td><td>-</td><td>-</td><td>-</td><td>-</td></tr>
<tr><td>gALL_v3_c13246</td><td>2</td><td>10</td><td>-</td><td>-</td><td>-</td><td>-</td><td>-</td></tr>
<tr><td>gALL_v3_c13247</td><td>5</td><td>3</td><td>-</td><td>-</td><td>gb|ADK20127.1|</td><td>eater [Drosophila melanogaster]</td><td>1,00E-22</td></tr>
<tr><td>gALL_v3_c13248</td><td>8</td><td>8</td><td>gnl|CDD|188148</td><td>TIGR01511, ATPase-IB1_Cu, cc</td><td>gb|EFN76571.1|</td><td>Copper-transporting ATPase 1 [Harpegnathos saltator]</td><td>0</td></tr>
<tr><td>gALL_v3_c13249</td><td>3</td><td>5</td><td>-</td><td>-</td><td>-</td><td>-</td><td>-</td></tr>
<tr><td>gALL_v3_c1325</td><td>12</td><td>13</td><td>-</td><td>-</td><td>ref|XP_973180.1|</td><td>PREDICTED: similar to cytochrome P450 [Tribolium castaneum] gb|EEZ99196.1| cytochrome P450 304E1 [Tribolium castaneum]</td><td>1,00E-07</td></tr>
<tr><td>gALL_v3_c13250</td><td>2</td><td>2</td><td>-</td><td>-</td><td>gb|AGM32293.1|</td><td>methionine aminopeptidase, partial [Coptotermes formosanus]</td><td>6,00E-08</td></tr>
<tr><td>gALL_v3_c13251</td><td>4</td><td>2</td><td>gnl|CDD|182655</td><td>PRK10696, PRK10696, tRNA 2-</td><td>gb|EFN62277.1|</td><td>tRNA 2-thiocytidine biosynthesis protein ttcA [Camponotus floridanus]</td><td>3,00E-32</td></tr>
<tr><td>gALL_v3_c13252</td><td>1</td><td>6</td><td>-</td><td>-</td><td>-</td><td>-</td><td>-</td></tr>
<tr><td>gALL_v3_c13253</td><td>2</td><td>4</td><td>-</td><td>-</td><td>-</td><td>-</td><td>-</td></tr>
<tr><td>gALL_v3_c13254</td><td>2</td><td>3</td><td>-</td><td>-</td><td>-</td><td>-</td><td>-</td></tr>
<tr><td>gALL_v3_c13255</td><td>2</td><td>4</td><td>-</td><td>-</td><td>-</td><td>-</td><td>-</td></tr>
<tr><td>gALL_v3_c13258</td><td>7</td><td>16</td><td>-</td><td>-</td><td>ref|XP_003708409.1|</td><td>PREDICTED: stress-induced-phosphoprotein 1-like [Megachile rotundata]</td><td>7,00E-07</td></tr>
<tr><td>gALL_v3_c13259</td><td>4</td><td>3</td><td>-</td><td>-</td><td>-</td><td>-</td><td>-</td></tr>
<tr><td>gALL_v3_c13260</td><td>8</td><td>9</td><td>-</td><td>-</td><td>-</td><td>-</td><td>-</td></tr>
<tr><td>gALL_v3_c13261</td><td>6</td><td>4</td><td>gnl|CDD|29261</td><td>cd00204, ANK, ankyrin repeat:</td><td>gb|EFX84229.1|</td><td>hypothetical protein DAPPUDRAFT_301376 [Daphnia pulex]</td><td>1,00E-14</td></tr>
<tr><td>gALL_v3_c13262</td><td>6</td><td>7</td><td>-</td><td>-</td><td>-</td><td>-</td><td>-</td></tr>
<tr><td>gALL_v3_c13263</td><td>8</td><td>5</td><td>-</td><td>-</td><td>ref|XP_312351.5|</td><td>AGAP002587-PA [Anopheles gambiae str. PEST] gb|EAA08073.5| AGAP002587-PA [Anopheles gambiae str. PEST]</td><td>1,00E-26</td></tr>
<tr><td>gALL_v3_c13264</td><td>4</td><td>3</td><td>gnl|CDD|118278</td><td>pfam09746, Membralin, Tumo</td><td>ref|XP_002428438.1|</td><td>membralin, putative [Pediculus humanus corporis] gb|EEB15700.1| membralin, putative [Pediculus humanus corporis]</td><td>1,00E-139</td></tr>
<tr><td>gALL_v3_c13265</td><td>3</td><td>2</td><td>gnl|CDD|178066</td><td>PLN02447, PLN02447, 1,4-alpl</td><td>ref|XP_968648.1|</td><td>PREDICTED: similar to GA17312-PA [Tribolium castaneum] gb|EFA11030.1| hypothetical protein TcasGA2_TC004619 [Tribolium castaneum]</td><td>2,00E-70</td></tr>
<tr><td>gALL_v3_c13266</td><td>2</td><td>5</td><td>-</td><td>-</td><td>-</td><td>-</td><td>-</td></tr>
<tr><td>gALL_v3_c13268</td><td>2</td><td>7</td><td>gnl|CDD|173667</td><td>cd05576, STKc_RPK118_like, C</td><td>emb|CAB92850.1|</td><td>hypothetical protein [Homo sapiens]</td><td>9,00E-53</td></tr>
<tr><td>gALL_v3_c13269</td><td>5</td><td>8</td><td>-</td><td>-</td><td>-</td><td>-</td><td>-</td></tr>
<tr><td>gALL_v3_c1327</td><td>8</td><td>6</td><td>gnl|CDD|128361</td><td>smart00046, DAGKc, Diacylgly</td><td>ref|XP_002425169.1|</td><td>Diacylglycerol kinase zeta, putative [Pediculus humanus corporis] gb|EEB12431.1| Diacylglycerol kinase zeta, putative [Pediculus humanus corporis]</td><td>1,00E-104</td></tr>
<tr><td>gALL_v3_c13272</td><td>2</td><td>5</td><td>-</td><td>-</td><td>-</td><td>-</td><td>-</td></tr>
<tr><td>gALL_v3_c13273</td><td>3</td><td>4</td><td>-</td><td>-</td><td>ref|XP_975582.2|</td><td>PREDICTED: similar to Pecanex-like protein 1 (Pecanex homolog) [Tribolium castaneum] gb|EFA01728.1| hypothetical protein TcasGA2_TC007308 [Tribolium castaneum]</td><td>5,00E-28</td></tr>
<tr><td>gALL_v3_c13274</td><td>6</td><td>5</td><td>gnl|CDD|119392</td><td>cd06174, MFS, The Major Faci</td><td>ref|XP_006570552.1|</td><td>PREDICTED: solute carrier family 2, facilitated glucose transporter member 10-like [Apis mellifera]</td><td>4,00E-25</td></tr>
<tr><td>gALL_v3_c13276</td><td>3</td><td>3</td><td>-</td><td>-</td><td>-</td><td>-</td><td>-</td></tr>
<tr><td>gALL_v3_c13277</td><td>1</td><td>7</td><td>gnl|CDD|128534</td><td>smart00238, BIR, Baculoviral i</td><td>gb|EFN83346.1|</td><td>Baculoviral IAP repeat-containing protein 6 [Harpegnathos saltator]</td><td>2,00E-81</td></tr>
<tr><td>gALL_v3_c13278</td><td>4</td><td>6</td><td>gnl|CDD|191257</td><td>pfam05328, CybS, CybS. This</td><td>ref|XP_005186967.1|</td><td>PREDICTED: putative succinate dehydrogenase [ubiquinone] cytochrome b small subunit, mitochondrial-like isoform X1 [Musca domestica]</td><td>8,00E-40</td></tr>
<tr><td>gALL_v3_c13279</td><td>3</td><td>3</td><td>-</td><td>-</td><td>-</td><td>-</td><td>-</td></tr>
<tr><td>gALL_v3_c1328</td><td>3</td><td>7</td><td>-</td><td>-</td><td>-</td><td>-</td><td>-</td></tr>
<tr><td>gALL_v3_c13280</td><td>3</td><td>2</td><td>gnl|CDD|190846</td><td>pfam04054, Not1, CCR4-Not c</td><td>ref|XP_003643969.2|</td><td>PREDICTED: CCR4-NOT transcription complex subunit 1-like [Gallus gallus]</td><td>4,00E-76</td></tr>
<tr><td>gALL_v3_c13281</td><td>4</td><td>2</td><td>gnl|CDD|73156</td><td>cd01650, RT_nLTR_like, RT_nL</td><td>ref|XP_002385618.1|</td><td>LINE-1 retrotransposon-like element, putative [Aspergillus flavus NRRL3357] gb|EED44706.1| LINE-1 retrotransposon-like element, putative [Aspergillus flavus NRRL3357]</td><td>8,00E-49</td></tr>
<tr><td>gALL_v3_c13282</td><td>1</td><td>5</td><td>-</td><td>-</td><td>-</td><td>-</td><td>-</td></tr>
<tr><td>gALL_v3_c13283</td><td>3</td><td>6</td><td>gnl|CDD|177210</td><td>MTH00153, COX1, cytochrome</td><td>gb|AAL02260.1|</td><td>cytochrome oxidase subunit I [Coptotermes formosanus]</td><td>1,00E-107</td></tr>
<tr><td>gALL_v3_c13284</td><td>1</td><td>4</td><td>gnl|CDD|189650</td><td>pfam00645, zf-PARP, Poly(ADP</td><td>ref|XP_002425250.1|</td><td>DNA ligase, putative [Pediculus humanus corporis] gb|EEB12512.1| DNA ligase, putative [Pediculus humanus corporis]</td><td>2,00E-50</td></tr>
<tr><td>gALL_v3_c13285</td><td>8</td><td>6</td><td>gnl|CDD|173456</td><td>PTZ00169, PTZ00169, ADP/ATI</td><td>ref|XP_001603181.2|</td><td>PREDICTED: calcium-binding mitochondrial carrier protein SCaMC-2-like isoform 1 [Nasonia vitripennis] ref|XP_003425019.1| PREDICTED: calcium-binding mitochondrial c</td><td>0</td></tr>
<tr><td>gALL_v3_c13287</td><td>1</td><td>6</td><td>-</td><td>-</td><td>-</td><td>-</td><td>-</td></tr>
<tr><td>gALL_v3_c13288</td><td>4</td><td>2</td><td>-</td><td>-</td><td>-</td><td>-</td><td>-</td></tr>
<tr><td>gALL_v3_c13289</td><td>0</td><td>6</td><td>-</td><td>-</td><td>-</td><td>-</td><td>-</td></tr>
<tr><td>gALL_v3_c13290</td><td>2</td><td>3</td><td>-</td><td>-</td><td>-</td><td>-</td><td>-</td></tr>
<tr><td>gALL_v3_c13291</td><td>4</td><td>6</td><td>gnl|CDD|189860</td><td>pfam01151, ELO, GNS1/SUR4</td><td>ref|XP_003700519.1|</td><td>PREDICTED: elongation of very long chain fatty acids protein 7-like [Megachile rotundata]</td><td>6,00E-68</td></tr>
<tr><td>gALL_v3_c13292</td><td>2</td><td>4</td><td>-</td><td>-</td><td>-</td><td>-</td><td>-</td></tr>
<tr><td>gALL_v3_c13293</td><td>2</td><td>4</td><td>-</td><td>-</td><td>-</td><td>-</td><td>-</td></tr>
<tr><td>gALL_v3_c13294</td><td>1</td><td>7</td><td>gnl|CDD|117144</td><td>pfam08569, Mo25, Mo25-like.</td><td>gb|AGM32755.1|</td><td>Mo25-like protein, partial [Coptotermes formosanus]</td><td>7,00E-99</td></tr>
<tr><td>gALL_v3_c13295</td><td>13</td><td>9</td><td>-</td><td>-</td><td>-</td><td>-</td><td>-</td></tr>
<tr><td>gALL_v3_c13296</td><td>2</td><td>4</td><td>-</td><td>-</td><td>-</td><td>-</td><td>-</td></tr>
<tr><td>gALL_v3_c13297</td><td>6</td><td>5</td><td>gnl|CDD|178658</td><td>PLN03111, PLN03111, DNA-di</td><td>ref|XP_002429002.1|</td><td>DNA-directed RNA polymerase II 23 kDa polypeptide, putative [Pediculus humanus corporis] gb|EEB16264.1| DNA-directed RNA polymerase II 23 kDa polypeptide, putativ</td><td>4,00E-85</td></tr>
<tr><td>gALL_v3_c13298</td><td>6</td><td>3</td><td>-</td><td>-</td><td>ref|XP_003488445.1|</td><td>PREDICTED: hypothetical protein LOC100743974 isoform 1 [Bombus impatiens] ref|XP_003488446.1| PREDICTED: hypothetical protein LOC100743974 isoform 2 [Bombus</td><td>9,00E-22</td></tr>
<tr><td>gALL_v3_c13299</td><td>3</td><td>1</td><td>-</td><td>-</td><td>-</td><td>-</td><td>-</td></tr>
<tr><td>gALL_v3_c133</td><td>8</td><td>18</td><td>-</td><td>-</td><td>-</td><td>-</td><td>-</td></tr>
<tr><td>gALL_v3_c1330</td><td>24</td><td>27</td><td>gnl|CDD|73156</td><td>cd01650, RT_nLTR_like, RT_nL</td><td>gb|ADI61823.1|</td><td>endonuclease-reverse transcriptase [Bombyx mori]</td><td>4,00E-11</td></tr>
<tr><td>gALL_v3_c13301</td><td>7</td><td>5</td><td>-</td><td>-</td><td>-</td><td>-</td><td>-</td></tr>
<tr><td>gALL_v3_c13302</td><td>3</td><td>1</td><td>-</td><td>-</td><td>-</td><td>-</td><td>-</td></tr>
<tr><td>gALL_v3_c13304</td><td>10</td><td>12</td><td>-</td><td>-</td><td>-</td><td>-</td><td>-</td></tr>
<tr><td>gALL_v3_c13305</td><td>4</td><td>2</td><td>-</td><td>-</td><td>ref|XP_002432269.1|</td><td>myospheroid protein, putative [Pediculus humanus corporis] gb|EEB19531.1| myospheroid protein, putative [Pediculus humanus corporis]</td><td>6,00E-18</td></tr>
<tr><td>gALL_v3_c13306</td><td>2</td><td>3</td><td>-</td><td>-</td><td>-</td><td>-</td><td>-</td></tr>
<tr><td>gALL_v3_c13307</td><td>3</td><td>7</td><td>-</td><td>-</td><td>-</td><td>-</td><td>-</td></tr>
<tr><td>gALL_v3_c13308</td><td>0</td><td>6</td><td>gnl|CDD|191286</td><td>pfam05497, Destabilase, Dest</td><td>gb|AFZ78836.1|</td><td>I-type lysozyme [Coptotermes formosanus]</td><td>2,00E-21</td></tr>
<tr><td>gALL_v3_c13309</td><td>4</td><td>7</td><td>gnl|CDD|29257</td><td>cd00200, WD40, WD40 domai</td><td>ref|XP_393446.1|</td><td>PREDICTED: WD40 repeat-containing protein SMU1-like isoform 1 [Apis mellifera] ref|XP_003696175.1| PREDICTED: WD40 repeat-containing protein SMU1-like [Apis flore</td><td>0</td></tr>
<tr><td>gALL_v3_c1331</td><td>4</td><td>6</td><td>-</td><td>-</td><td>-</td><td>-</td><td>-</td></tr>
<tr><td>gALL_v3_c13310</td><td>2</td><td>7</td><td>-</td><td>-</td><td>ref|XP_006029730.1|</td><td>PREDICTED: histone-lysine N-methyltransferase SETMAR-like [Alligator sinensis]</td><td>1,00E-14</td></tr>
<tr><td>gALL_v3_c13312</td><td>2</td><td>8</td><td>-</td><td>-</td><td>-</td><td>-</td><td>-</td></tr>
<tr><td>gALL_v3_c13313</td><td>10</td><td>9</td><td>-</td><td>-</td><td>-</td><td>-</td><td>-</td></tr>
<tr><td>gALL_v3_c13314</td><td>3</td><td>2</td><td>-</td><td>-</td><td>-</td><td>-</td><td>-</td></tr>
<tr><td>gALL_v3_c13315</td><td>1</td><td>5</td><td>gnl|CDD|112485</td><td>pfam03670, UPF0184, Unchar</td><td>ref|XP_005293487.1|</td><td>PREDICTED: UPF0184 protein C9orf16 homolog [Chrysemys picta bellii]</td><td>1,00E-15</td></tr>
<tr><td>gALL_v3_c13316</td><td>5</td><td>9</td><td>-</td><td>-</td><td>-</td><td>-</td><td>-</td></tr>
<tr><td>gALL_v3_c13317</td><td>1</td><td>7</td><td>-</td><td>-</td><td>-</td><td>-</td><td>-</td></tr>
<tr><td>gALL_v3_c13318</td><td>7</td><td>8</td><td>-</td><td>-</td><td>-</td><td>-</td><td>-</td></tr>
<tr><td>gALL_v3_c13319</td><td>7</td><td>7</td><td>-</td><td>-</td><td>-</td><td>-</td><td>-</td></tr>
<tr><td>gALL_v3_c1332</td><td>4</td><td>6</td><td>-</td><td>-</td><td>-</td><td>-</td><td>-</td></tr>
<tr><td>gALL_v3_c13320</td><td>1</td><td>5</td><td>-</td><td>-</td><td>-</td><td>-</td><td>-</td></tr>
<tr><td>gALL_v3_c13321</td><td>1</td><td>5</td><td>-</td><td>-</td><td>-</td><td>-</td><td>-</td></tr>
<tr><td>gALL_v3_c13322</td><td>1</td><td>4</td><td>-</td><td>-</td><td>ref|XP_003704701.1|</td><td>PREDICTED: CCR4-NOT transcription complex subunit 1 isoform 1 [Megachile rotundata]</td><td>5,00E-41</td></tr>
<tr><td>gALL_v3_c13323</td><td>3</td><td>3</td><td>gnl|CDD|100117</td><td>cd06071, Beach, BEACH (Beige</td><td>gb|EFA01972.1|</td><td>hypothetical protein TcasGA2_TC007593 [Tribolium castaneum]</td><td>1,00E-130</td></tr>
<tr><td>gALL_v3_c13324</td><td>3</td><td>2</td><td>-</td><td>-</td><td>-</td><td>-</td><td>-</td></tr>
<tr><td>gALL_v3_c13325</td><td>1</td><td>4</td><td>-</td><td>-</td><td>-</td><td>-</td><td>-</td></tr>
<tr><td>gALL_v3_c13326</td><td>3</td><td>9</td><td>-</td><td>-</td><td>-</td><td>-</td><td>-</td></tr>
<tr><td>gALL_v3_c13327</td><td>6</td><td>9</td><td>-</td><td>-</td><td>gb|ESO99640.1|</td><td>hypothetical protein LOTGIDRAFT_141789 [Lottia gigantea]</td><td>2,00E-06</td></tr>
<tr><td>gALL_v3_c13328</td><td>1</td><td>5</td><td>-</td><td>-</td><td>gb|EFN66847.1|</td><td>hypothetical protein EAG_08499 [Camponotus floridanus]</td><td>7,00E-08</td></tr>
<tr><td>gALL_v3_c13330</td><td>2</td><td>4</td><td>-</td><td>-</td><td>-</td><td>-</td><td>-</td></tr>
<tr><td>gALL_v3_c13331</td><td>6</td><td>7</td><td>-</td><td>-</td><td>-</td><td>-</td><td>-</td></tr>
<tr><td>gALL_v3_c13332</td><td>4</td><td>13</td><td>-</td><td>-</td><td>-</td><td>-</td><td>-</td></tr>
<tr><td>gALL_v3_c13334</td><td>6</td><td>9</td><td>gnl|CDD|176376</td><td>cd01781, AF6_RA_repeat2, Ub</td><td>ref|XP_002432943.1|</td><td>afadin, putative [Pediculus humanus corporis] gb|EEB20205.1| afadin, putative [Pediculus humanus corporis]</td><td>0</td></tr>
<tr><td>gALL_v3_c13335</td><td>3</td><td>2</td><td>-</td><td>-</td><td>-</td><td>-</td><td>-</td></tr>
<tr><td>gALL_v3_c13337</td><td>4</td><td>6</td><td>-</td><td>-</td><td>-</td><td>-</td><td>-</td></tr>
<tr><td>gALL_v3_c13339</td><td>5</td><td>9</td><td>-</td><td>-</td><td>-</td><td>-</td><td>-</td></tr>
<tr><td>gALL_v3_c1334</td><td>21</td><td>20</td><td>gnl|CDD|113611</td><td>pfam04845, PurA, PurA ssDNA</td><td>ref|XP_001601888.1|</td><td>PREDICTED: transcriptional activator protein Pur-alpha-like [Nasonia vitripennis]</td><td>2,33E-156</td></tr>
<tr><td>gALL_v3_c13340</td><td>3</td><td>10</td><td>-</td><td>-</td><td>-</td><td>-</td><td>-</td></tr>
<tr><td>gALL_v3_c13342</td><td>1</td><td>4</td><td>-</td><td>-</td><td>-</td><td>-</td><td>-</td></tr>
<tr><td>gALL_v3_c13343</td><td>5</td><td>2</td><td>-</td><td>-</td><td>-</td><td>-</td><td>-</td></tr>
<tr><td>gALL_v3_c13344</td><td>2</td><td>4</td><td>-</td><td>-</td><td>-</td><td>-</td><td>-</td></tr>
<tr><td>gALL_v3_c13345</td><td>2</td><td>3</td><td>-</td><td>-</td><td>-</td><td>-</td><td>-</td></tr>
<tr><td>gALL_v3_c13346</td><td>3</td><td>6</td><td>-</td><td>-</td><td>-</td><td>-</td><td>-</td></tr>
<tr><td>gALL_v3_c13347</td><td>2</td><td>3</td><td>-</td><td>-</td><td>-</td><td>-</td><td>-</td></tr>
<tr><td>gALL_v3_c13348</td><td>2</td><td>4</td><td>-</td><td>-</td><td>-</td><td>-</td><td>-</td></tr>
<tr><td>gALL_v3_c13349</td><td>4</td><td>7</td><td>-</td><td>-</td><td>gb|EGI63619.1|</td><td>F-box/LRR-repeat protein 12 [Acromyrmex echinatior]</td><td>1,00E-42</td></tr>
<tr><td>gALL_v3_c13350</td><td>4</td><td>3</td><td>-</td><td>-</td><td>-</td><td>-</td><td>-</td></tr>
<tr><td>gALL_v3_c13352</td><td>6</td><td>4</td><td>-</td><td>-</td><td>-</td><td>-</td><td>-</td></tr>
<tr><td>gALL_v3_c13353</td><td>7</td><td>4</td><td>-</td><td>-</td><td>gb|EFN86035.1|</td><td>ATP-binding cassette sub-family G member 4 [Harpegnathos saltator]</td><td>1,00E-14</td></tr>
<tr><td>gALL_v3_c13354</td><td>2</td><td>3</td><td>-</td><td>-</td><td>-</td><td>-</td><td>-</td></tr>
<tr><td>gALL_v3_c13355</td><td>4</td><td>10</td><td>-</td><td>-</td><td>ref|XP_001605155.2|</td><td>PREDICTED: eukaryotic translation initiation factor 3 subunit C-like [Nasonia vitripennis]</td><td>1,00E-65</td></tr>
<tr><td>gALL_v3_c13356</td><td>2</td><td>5</td><td>-</td><td>-</td><td>-</td><td>-</td><td>-</td></tr>
<tr><td>gALL_v3_c13357</td><td>3</td><td>7</td><td>gnl|CDD|173675</td><td>cd05584, STKc_p70S6K, Catal</td><td>ref|XP_002425139.1|</td><td>RAC protein kinase DRAC-PK85, putative [Pediculus humanus corporis] gb|EEB12401.1| RAC protein kinase DRAC-PK85, putative [Pediculus humanus corporis]</td><td>8,00E-99</td></tr>
<tr><td>gALL_v3_c13358</td><td>5</td><td>3</td><td>-</td><td>-</td><td>-</td><td>-</td><td>-</td></tr>
<tr><td>gALL_v3_c13359</td><td>4</td><td>6</td><td>gnl|CDD|88596</td><td>cd05796, Ribosomal_P0_like,</td><td>ref|XP_002005566.1|</td><td>GI18999 [Drosophila mojavensis] gb|EDW09501.1| GI18999 [Drosophila mojavensis]</td><td>4,00E-31</td></tr>
<tr><td>gALL_v3_c1336</td><td>20</td><td>17</td><td>-</td><td>-</td><td>-</td><td>-</td><td>-</td></tr>
<tr><td>gALL_v3_c13360</td><td>2</td><td>4</td><td>gnl|CDD|34727</td><td>COG5126, FRQ1, Ca2+-binding</td><td>ref|XP_003704470.1|</td><td>PREDICTED: neuronal calcium sensor 2-like [Megachile rotundata]</td><td>2,00E-80</td></tr>
<tr><td>gALL_v3_c13361</td><td>3</td><td>5</td><td>-</td><td>-</td><td>-</td><td>-</td><td>-</td></tr>
<tr><td>gALL_v3_c13362</td><td>1</td><td>5</td><td>-</td><td>-</td><td>-</td><td>-</td><td>-</td></tr>
<tr><td>gALL_v3_c13363</td><td>2</td><td>4</td><td>-</td><td>-</td><td>-</td><td>-</td><td>-</td></tr>
<tr><td>gALL_v3_c13367</td><td>4</td><td>5</td><td>gnl|CDD|189547</td><td>pfam00436, SSB, Single-strand</td><td>ref|XP_003493419.1|</td><td>PREDICTED: single-stranded DNA-binding protein, mitochondrial-like [Bombus impatiens]</td><td>6,00E-47</td></tr>
<tr><td>gALL_v3_c13368</td><td>6</td><td>7</td><td>gnl|CDD|30384</td><td>COG0035, Upp, Uracil phosph</td><td>dbj|BAN20865.1|</td><td>uracil phosphoribosyltransferase [Riptortus pedestris]</td><td>3,00E-99</td></tr>
<tr><td>gALL_v3_c13369</td><td>2</td><td>4</td><td>gnl|CDD|180426</td><td>PRK06148, PRK06148, hypoth</td><td>ref|XP_002426908.1|</td><td>alanine-glyoxylate aminotransferase, putative [Pediculus humanus corporis] gb|EEB14170.1| alanine-glyoxylate aminotransferase, putative [Pediculus humanus corporis]</td><td>1,00E-114</td></tr>
<tr><td>gALL_v3_c1337</td><td>5</td><td>16</td><td>-</td><td>-</td><td>-</td><td>-</td><td>-</td></tr>
</table>

<table>
<tr><td>gALL_v3_c13370</td><td>1</td><td>4</td><td>-</td><td>-</td><td>gb|ETN63484.1|</td><td>calcium-activated potassium channel alpha chain [Anopheles darlingi]</td><td>1,00E-129</td></tr>
<tr><td>gALL_v3_c13371</td><td>5</td><td>2</td><td>-</td><td>-</td><td>-</td><td>-</td><td>-</td></tr>
<tr><td>gALL_v3_c13372</td><td>1</td><td>6</td><td>-</td><td>-</td><td>-</td><td>-</td><td>-</td></tr>
<tr><td>gALL_v3_c13373</td><td>2</td><td>3</td><td>-</td><td>-</td><td>-</td><td>-</td><td>-</td></tr>
<tr><td>gALL_v3_c13375</td><td>7</td><td>6</td><td>-</td><td>-</td><td>gb|EFA11445.1|</td><td>hypothetical protein TcasGA2_TC013627 [Tribolium castaneum]</td><td>2,00E-15</td></tr>
<tr><td>gALL_v3_c13376</td><td>3</td><td>6</td><td>-</td><td>-</td><td>-</td><td>-</td><td>-</td></tr>
<tr><td>gALL_v3_c13377</td><td>3</td><td>3</td><td>-</td><td>-</td><td>-</td><td>-</td><td>-</td></tr>
<tr><td>gALL_v3_c13378</td><td>5</td><td>5</td><td>-</td><td>-</td><td>gb|EFZ20376.1|</td><td>hypothetical protein SINV_11079 [Solenopsis invicta]</td><td>1,00E-06</td></tr>
<tr><td>gALL_v3_c1338</td><td>6</td><td>16</td><td>-</td><td>-</td><td>-</td><td>-</td><td>-</td></tr>
<tr><td>gALL_v3_c13380</td><td>3</td><td>5</td><td>-</td><td>-</td><td>-</td><td>-</td><td>-</td></tr>
<tr><td>gALL_v3_c13381</td><td>2</td><td>4</td><td>-</td><td>-</td><td>-</td><td>-</td><td>-</td></tr>
<tr><td>gALL_v3_c13382</td><td>5</td><td>5</td><td>gnl|CDD|188879</td><td>cd08533, SAM_PNT-ETS-1,2, S</td><td>ref|XP_003486023.1|</td><td>PREDICTED: hypothetical protein LOC100745810 isoform 2 [Bombus impatiens]</td><td>3,00E-52</td></tr>
<tr><td>gALL_v3_c13384</td><td>6</td><td>6</td><td>-</td><td>-</td><td>gb|EFN79083.1|</td><td>Ubiquitin carboxyl-terminal hydrolase 34 [Harpegnathos saltator]</td><td>1,00E-134</td></tr>
<tr><td>gALL_v3_c13385</td><td>3</td><td>6</td><td>gnl|CDD|189811</td><td>pfam01031, Dynamin_M, Dyn</td><td>ref|XP_003492061.1|</td><td>PREDICTED: dynamin-1-like protein-like [Bombus impatiens]</td><td>1,00E-175</td></tr>
<tr><td>gALL_v3_c13386</td><td>5</td><td>4</td><td>gnl|CDD|113205</td><td>pfam04424, DUF544, Protein c</td><td>gb|EFX83129.1|</td><td>hypothetical protein DAPPUDRAFT_48508 [Daphnia pulex]</td><td>2,00E-67</td></tr>
<tr><td>gALL_v3_c13388</td><td>4</td><td>4</td><td>-</td><td>-</td><td>gb|EEZ97180.1|</td><td>hypothetical protein TcasGA2_TC004367 [Tribolium castaneum]</td><td>2,00E-26</td></tr>
<tr><td>gALL_v3_c13389</td><td>1</td><td>3</td><td>-</td><td>-</td><td>-</td><td>-</td><td>-</td></tr>
<tr><td>gALL_v3_c1339</td><td>14</td><td>13</td><td>-</td><td>-</td><td>-</td><td>-</td><td>-</td></tr>
<tr><td>gALL_v3_c13390</td><td>6</td><td>16</td><td>-</td><td>-</td><td>emb|CAX83710.1|</td><td>endonuclease-reverse transcriptase [Schistosoma japonicum]</td><td>2,00E-99</td></tr>
<tr><td>gALL_v3_c13391</td><td>2</td><td>10</td><td>-</td><td>-</td><td>-</td><td>-</td><td>-</td></tr>
<tr><td>gALL_v3_c13392</td><td>14</td><td>18</td><td>-</td><td>-</td><td>-</td><td>-</td><td>-</td></tr>
<tr><td>gALL_v3_c13393</td><td>2</td><td>4</td><td>-</td><td>-</td><td>-</td><td>-</td><td>-</td></tr>
<tr><td>gALL_v3_c13394</td><td>2</td><td>6</td><td>-</td><td>-</td><td>-</td><td>-</td><td>-</td></tr>
<tr><td>gALL_v3_c13395</td><td>3</td><td>3</td><td>-</td><td>-</td><td>gb|EFN84766.1|</td><td>Transient receptor potential cation channel subfamily V member 6 [Harpegnathos saltator]</td><td>6,00E-50</td></tr>
<tr><td>gALL_v3_c13396</td><td>1</td><td>4</td><td>-</td><td>-</td><td>-</td><td>-</td><td>-</td></tr>
<tr><td>gALL_v3_c13397</td><td>3</td><td>3</td><td>-</td><td>-</td><td>-</td><td>-</td><td>-</td></tr>
<tr><td>gALL_v3_c13398</td><td>5</td><td>14</td><td>gnl|CDD|185504</td><td>PTZ00184, PTZ00184, calmodu</td><td>ref|XP_001957095.1|</td><td>GF10252 [Drosophila ananassae] gb|EDV39901.1| GF10252 [Drosophila ananassae]</td><td>4,00E-69</td></tr>
<tr><td>gALL_v3_c13399</td><td>4</td><td>5</td><td>gnl|CDD|143944</td><td>pfam00178, Ets, Ets-domain.</td><td>gb|EFN61993.1|</td><td>Ecdysone-induced protein 74EF isoform B [Camponotus floridanus]</td><td>1,00E-38</td></tr>
<tr><td>gALL_v3_c1340</td><td>10</td><td>13</td><td>-</td><td>-</td><td>-</td><td>-</td><td>-</td></tr>
<tr><td>gALL_v3_c13400</td><td>1</td><td>6</td><td>gnl|CDD|31225</td><td>COG1022, FAA1, Long-chain ac</td><td>ref|XP_967873.1|</td><td>PREDICTED: similar to AGAP008596-PA [Tribolium castaneum] gb|EFA11034.1| hypothetical protein TcasGA2_TC004625 [Tribolium castaneum]</td><td>2,00E-24</td></tr>
<tr><td>gALL_v3_c13401</td><td>1</td><td>5</td><td>-</td><td>-</td><td>-</td><td>-</td><td>-</td></tr>
<tr><td>gALL_v3_c13402</td><td>13</td><td>8</td><td>gnl|CDD|190211</td><td>pfam02115, Rho_GDI, RHO pr</td><td>gb|EFN80048.1|</td><td>Rho GDP-dissociation inhibitor 1 [Harpegnathos saltator]</td><td>8,00E-37</td></tr>
<tr><td>gALL_v3_c13403</td><td>2</td><td>2</td><td>-</td><td>-</td><td>-</td><td>-</td><td>-</td></tr>
<tr><td>gALL_v3_c13404</td><td>2</td><td>6</td><td>-</td><td>-</td><td>-</td><td>-</td><td>-</td></tr>
<tr><td>gALL_v3_c13405</td><td>2</td><td>6</td><td>-</td><td>-</td><td>-</td><td>-</td><td>-</td></tr>
<tr><td>gALL_v3_c13406</td><td>3</td><td>7</td><td>-</td><td>-</td><td>-</td><td>-</td><td>-</td></tr>
<tr><td>gALL_v3_c13407</td><td>5</td><td>6</td><td>-</td><td>-</td><td>gb|EKC34457.1|</td><td>hypothetical protein CGI_10012737 [Crassostrea gigas]</td><td>8,00E-10</td></tr>
<tr><td>gALL_v3_c13408</td><td>4</td><td>9</td><td>gnl|CDD|29257</td><td>cd00200, WD40, WD40 domai</td><td>ref|XP_002422904.1|</td><td>Methylosome protein, putative [Pediculus humanus corporis] gb|EEB10166.1| Methylosome protein, putative [Pediculus humanus corporis]</td><td>4,00E-78</td></tr>
<tr><td>gALL_v3_c13409</td><td>4</td><td>5</td><td>-</td><td>-</td><td>-</td><td>-</td><td>-</td></tr>
<tr><td>gALL_v3_c1341</td><td>6</td><td>6</td><td>-</td><td>-</td><td>-</td><td>-</td><td>-</td></tr>
<tr><td>gALL_v3_c13410</td><td>6</td><td>3</td><td>-</td><td>-</td><td>-</td><td>-</td><td>-</td></tr>
<tr><td>gALL_v3_c13411</td><td>4</td><td>3</td><td>-</td><td>-</td><td>ref|XP_001815637.1|</td><td>PREDICTED: similar to Tmc7 protein [Tribolium castaneum]</td><td>8,00E-36</td></tr>
<tr><td>gALL_v3_c13412</td><td>4</td><td>3</td><td>gnl|CDD|191996</td><td>pfam08315, cwf18, cwf18 pre-</td><td>ref|XP_003704154.1|</td><td>PREDICTED: coiled-coil domain-containing protein 12-like [Megachile rotundata]</td><td>4,00E-59</td></tr>
<tr><td>gALL_v3_c13413</td><td>2</td><td>6</td><td>-</td><td>-</td><td>-</td><td>-</td><td>-</td></tr>
<tr><td>gALL_v3_c13414</td><td>4</td><td>4</td><td>gnl|CDD|185053</td><td>PRK15098, PRK15098, beta-D-</td><td>ref|WP_010527720.1|</td><td>glycoside hydrolase family 3 [Thermophagus xiamenensis]</td><td>3,00E-65</td></tr>
<tr><td>gALL_v3_c13415</td><td>1</td><td>5</td><td>-</td><td>-</td><td>-</td><td>-</td><td>-</td></tr>
<tr><td>gALL_v3_c13416</td><td>6</td><td>3</td><td>-</td><td>-</td><td>-</td><td>-</td><td>-</td></tr>
<tr><td>gALL_v3_c13419</td><td>4</td><td>4</td><td>-</td><td>-</td><td>-</td><td>-</td><td>-</td></tr>
<tr><td>gALL_v3_c1342</td><td>34</td><td>48</td><td>gnl|CDD|188968</td><td>cd09569, SAM_liprin-beta1,2_</td><td>gb|EFA02520.1|</td><td>hypothetical protein TcasGA2_TC008225 [Tribolium castaneum]</td><td>1,00E-172</td></tr>
<tr><td>gALL_v3_c13421</td><td>6</td><td>4</td><td>gnl|CDD|58167</td><td>cd00886, MogA_MoaB, MogA</td><td>gb|ELT95068.1|</td><td>hypothetical protein CAPTEDRAFT_226958 [Capitella teleta]</td><td>2,00E-45</td></tr>
<tr><td>gALL_v3_c13422</td><td>1</td><td>5</td><td>-</td><td>-</td><td>gb|EFN64151.1|</td><td>hypothetical protein EAG_08253 [Camponotus floridanus]</td><td>8,00E-20</td></tr>
<tr><td>gALL_v3_c13423</td><td>0</td><td>4</td><td>-</td><td>-</td><td>-</td><td>-</td><td>-</td></tr>
<tr><td>gALL_v3_c13424</td><td>8</td><td>5</td><td>gnl|CDD|128654</td><td>smart00360, RRM, RNA recog</td><td>gb|EFN71758.1|</td><td>Scaffold attachment factor B2 [Camponotus floridanus]</td><td>2,00E-44</td></tr>
<tr><td>gALL_v3_c13425</td><td>1</td><td>5</td><td>-</td><td>-</td><td>-</td><td>-</td><td>-</td></tr>
<tr><td>gALL_v3_c13426</td><td>3</td><td>4</td><td>-</td><td>-</td><td>-</td><td>-</td><td>-</td></tr>
<tr><td>gALL_v3_c13427</td><td>2</td><td>3</td><td>-</td><td>-</td><td>-</td><td>-</td><td>-</td></tr>
<tr><td>gALL_v3_c13428</td><td>3</td><td>3</td><td>-</td><td>-</td><td>-</td><td>-</td><td>-</td></tr>
<tr><td>gALL_v3_c1343</td><td>19</td><td>17</td><td>gnl|CDD|146048</td><td>pfam03223, V-ATPase_C, V-AT</td><td>gb|EFN81041.1|</td><td>Vacuolar proton pump subunit C [Harpegnathos saltator]</td><td>0</td></tr>
<tr><td>gALL_v3_c13430</td><td>2</td><td>5</td><td>-</td><td>-</td><td>ref|XP_004525073.1|</td><td>PREDICTED: ubiquitin-conjugating enzyme E2 Q2-like isoform X2 [Ceratitis capitata]</td><td>1,00E-52</td></tr>
<tr><td>gALL_v3_c13431</td><td>10</td><td>6</td><td>-</td><td>-</td><td>-</td><td>-</td><td>-</td></tr>
<tr><td>gALL_v3_c13432</td><td>5</td><td>5</td><td>-</td><td>-</td><td>-</td><td>-</td><td>-</td></tr>
<tr><td>gALL_v3_c13433</td><td>0</td><td>6</td><td>-</td><td>-</td><td>-</td><td>-</td><td>-</td></tr>
<tr><td>gALL_v3_c13434</td><td>4</td><td>10</td><td>gnl|CDD|191998</td><td>pfam08320, PIG-X, PIG-X / PBN</td><td>gb|AEE62526.1|</td><td>unknown [Dendroctonus ponderosae]</td><td>6,00E-32</td></tr>
<tr><td>gALL_v3_c13435</td><td>4</td><td>3</td><td>-</td><td>-</td><td>ref|XP_003489768.1|</td><td>PREDICTED: dihydropyrimidinase-like isoform 3 [Bombus impatiens]</td><td>5,00E-26</td></tr>
<tr><td>gALL_v3_c13436</td><td>5</td><td>7</td><td>-</td><td>-</td><td>-</td><td>-</td><td>-</td></tr>
<tr><td>gALL_v3_c1344</td><td>4</td><td>9</td><td>-</td><td>-</td><td>-</td><td>-</td><td>-</td></tr>
<tr><td>gALL_v3_c13440</td><td>2</td><td>8</td><td>gnl|CDD|149454</td><td>pfam08399, VWA_N, VWA N-t</td><td>ref|XP_969952.2|</td><td>PREDICTED: similar to voltage-gated calcium channel alpha2-delta subunit 1 [Tribolium castaneum]</td><td>9,00E-90</td></tr>
<tr><td>gALL_v3_c13441</td><td>4</td><td>2</td><td>-</td><td>-</td><td>-</td><td>-</td><td>-</td></tr>
<tr><td>gALL_v3_c13442</td><td>1</td><td>7</td><td>gnl|CDD|192041</td><td>pfam08449, UAA, UAA transpo</td><td>ref|XP_003395629.1|</td><td>PREDICTED: adenosine 3'-phospho 5'-phosphosulfate transporter 1-like [Bombus terrestris]</td><td>3,00E-72</td></tr>
<tr><td>gALL_v3_c13444</td><td>3</td><td>2</td><td>-</td><td>-</td><td>-</td><td>-</td><td>-</td></tr>
<tr><td>gALL_v3_c13446</td><td>2</td><td>6</td><td>-</td><td>-</td><td>ref|XP_001814386.1|</td><td>PREDICTED: hypothetical protein [Tribolium castaneum] gb|EFA11507.1| hypothetical protein TcasGA2_TC014221 [Tribolium castaneum]</td><td>4,00E-35</td></tr>
<tr><td>gALL_v3_c13448</td><td>3</td><td>8</td><td>-</td><td>-</td><td>-</td><td>-</td><td>-</td></tr>
<tr><td>gALL_v3_c13449</td><td>3</td><td>3</td><td>-</td><td>-</td><td>-</td><td>-</td><td>-</td></tr>
<tr><td>gALL_v3_c13450</td><td>0</td><td>5</td><td>gnl|CDD|48161</td><td>cd03344, GroEL, GroEL_like ty</td><td>gb|AHK10124.1|</td><td>heat shock protein 60, partial [Formica cinerea]</td><td>2,00E-73</td></tr>
<tr><td>gALL_v3_c13451</td><td>3</td><td>2</td><td>-</td><td>-</td><td>ref|XP_003426828.1|</td><td>PREDICTED: type I inositol-3,4-bisphosphate 4-phosphatase-like isoform 3 [Nasonia vitripennis]</td><td>4,00E-30</td></tr>
<tr><td>gALL_v3_c13452</td><td>4</td><td>3</td><td>-</td><td>-</td><td>-</td><td>-</td><td>-</td></tr>
<tr><td>gALL_v3_c13455</td><td>1</td><td>4</td><td>-</td><td>-</td><td>-</td><td>-</td><td>-</td></tr>
<tr><td>gALL_v3_c13457</td><td>1</td><td>4</td><td>-</td><td>-</td><td>gb|ENN78652.1|</td><td>hypothetical protein YQE_04825, partial [Dendroctonus ponderosae]</td><td>1,00E-102</td></tr>
<tr><td>gALL_v3_c13459</td><td>3</td><td>2</td><td>-</td><td>-</td><td>-</td><td>-</td><td>-</td></tr>
<tr><td>gALL_v3_c1346</td><td>6</td><td>7</td><td>gnl|CDD|144078</td><td>pfam00348, polyprenyl_synt, I</td><td>ref|XP_002428407.1|</td><td>farnesyl-pyrophosphate synthetase, putative [Pediculus humanus corporis] gb|EEB15669.1| farnesyl-pyrophosphate synthetase, putative [Pediculus humanus corporis]</td><td>1,00E-92</td></tr>
<tr><td>gALL_v3_c13460</td><td>6</td><td>10</td><td>-</td><td>-</td><td>gb|EGI69977.1|</td><td>Transcription factor kayak [Acromyrmex echinatior]</td><td>6,00E-19</td></tr>
<tr><td>gALL_v3_c13461</td><td>5</td><td>5</td><td>-</td><td>-</td><td>-</td><td>-</td><td>-</td></tr>
<tr><td>gALL_v3_c13462</td><td>3</td><td>2</td><td>gnl|CDD|145236</td><td>pfam01951, Archease, Archea</td><td>ref|XP_006561717.1|</td><td>PREDICTED: protein archease-like [Apis mellifera]</td><td>9,00E-37</td></tr>
<tr><td>gALL_v3_c13463</td><td>3</td><td>4</td><td>-</td><td>-</td><td>-</td><td>-</td><td>-</td></tr>
<tr><td>gALL_v3_c13465</td><td>0</td><td>6</td><td>gnl|CDD|152565</td><td>pfam12130, DUF3585, Protein</td><td>ref|XP_002424941.1|</td><td>protein MLP1, putative [Pediculus humanus corporis] gb|EEB12203.1| protein MLP1, putative [Pediculus humanus corporis]</td><td>1,00E-33</td></tr>
<tr><td>gALL_v3_c13466</td><td>1</td><td>4</td><td>-</td><td>-</td><td>-</td><td>-</td><td>-</td></tr>
<tr><td>gALL_v3_c13467</td><td>8</td><td>5</td><td>-</td><td>-</td><td>ref|XP_005197337.1|</td><td>PREDICTED: pre-mRNA cleavage complex 2 protein Pcf11, partial [Bos taurus]</td><td>2,00E-16</td></tr>
<tr><td>gALL_v3_c13468</td><td>8</td><td>17</td><td>-</td><td>-</td><td>-</td><td>-</td><td>-</td></tr>
<tr><td>gALL_v3_c13469</td><td>4</td><td>7</td><td>gnl|CDD|190129</td><td>pfam01835, A2M_N, MG2 dor</td><td>ref|XP_002427015.1|</td><td>conserved hypothetical protein [Pediculus humanus corporis] gb|EEB14277.1| conserved hypothetical protein [Pediculus humanus corporis]</td><td>1,00E-124</td></tr>
<tr><td>gALL_v3_c13470</td><td>8</td><td>7</td><td>gnl|CDD|128842</td><td>smart00572, DZF, domain in D</td><td>gb|ELT89094.1|</td><td>hypothetical protein CAPTEDRAFT_49339, partial [Capitella teleta]</td><td>3,00E-47</td></tr>
<tr><td>gALL_v3_c13472</td><td>2</td><td>5</td><td>-</td><td>-</td><td>-</td><td>-</td><td>-</td></tr>
<tr><td>gALL_v3_c13474</td><td>10</td><td>11</td><td>-</td><td>-</td><td>-</td><td>-</td><td>-</td></tr>
<tr><td>gALL_v3_c13475</td><td>3</td><td>6</td><td>-</td><td>-</td><td>-</td><td>-</td><td>-</td></tr>
<tr><td>gALL_v3_c13476</td><td>4</td><td>4</td><td>-</td><td>-</td><td>ref|WP_010422314.1|</td><td>hypothetical protein [Rickettsia helvetica]</td><td>8,00E-06</td></tr>
<tr><td>gALL_v3_c13477</td><td>3</td><td>11</td><td>-</td><td>-</td><td>-</td><td>-</td><td>-</td></tr>
<tr><td>gALL_v3_c13478</td><td>7</td><td>14</td><td>gnl|CDD|173661</td><td>cd05570, STKc_PKC, Catalytic c</td><td>gb|EFA07407.1|</td><td>protein C kinase 98E-like protein [Tribolium castaneum]</td><td>5,00E-77</td></tr>
<tr><td>gALL_v3_c13479</td><td>1</td><td>5</td><td>-</td><td>-</td><td>-</td><td>-</td><td>-</td></tr>
<tr><td>gALL_v3_c1348</td><td>9</td><td>15</td><td>gnl|CDD|189424</td><td>pfam00153, Mito_carr, Mitoch</td><td>ref|XP_972074.1|</td><td>PREDICTED: similar to tricarboxylate transport protein [Tribolium castaneum] gb|EFA04991.1| hypothetical protein TcasGA2_TC015070 [Tribolium castaneum]</td><td>1,00E-121</td></tr>
<tr><td>gALL_v3_c13481</td><td>3</td><td>3</td><td>-</td><td>-</td><td>-</td><td>-</td><td>-</td></tr>
<tr><td>gALL_v3_c13482</td><td>3</td><td>4</td><td>-</td><td>-</td><td>-</td><td>-</td><td>-</td></tr>
<tr><td>gALL_v3_c13483</td><td>6</td><td>3</td><td>-</td><td>-</td><td>-</td><td>-</td><td>-</td></tr>
<tr><td>gALL_v3_c13484</td><td>5</td><td>4</td><td>-</td><td>-</td><td>gb|EFN83995.1|</td><td>Probable ubiquitin carboxyl-terminal hydrolase FAF-X [Harpegnathos saltator]</td><td>8,00E-48</td></tr>
<tr><td>gALL_v3_c13485</td><td>2</td><td>5</td><td>gnl|CDD|128464</td><td>smart00160, RanBD, Ran-bind</td><td>ref|XP_002430421.1|</td><td>ran-binding protein, putative [Pediculus humanus corporis] gb|EEB17683.1| ran-binding protein, putative [Pediculus humanus corporis]</td><td>1,00E-22</td></tr>
<tr><td>gALL_v3_c13488</td><td>1</td><td>5</td><td>-</td><td>-</td><td>-</td><td>-</td><td>-</td></tr>
<tr><td>gALL_v3_c13489</td><td>4</td><td>2</td><td>-</td><td>-</td><td>-</td><td>-</td><td>-</td></tr>
<tr><td>gALL_v3_c13490</td><td>3</td><td>3</td><td>-</td><td>-</td><td>ref|XP_002426407.1|</td><td>dedicator of cytokinesis protein, putative [Pediculus humanus corporis] gb|EEB13669.1| dedicator of cytokinesis protein, putative [Pediculus humanus corporis]</td><td>2,00E-28</td></tr>
<tr><td>gALL_v3_c13491</td><td>3</td><td>1</td><td>-</td><td>-</td><td>gb|ESN97698.1|</td><td>hypothetical protein HELRODRAFT_177753 [Helobdella robusta]</td><td>5,00E-13</td></tr>
<tr><td>gALL_v3_c13493</td><td>2</td><td>4</td><td>gnl|CDD|30094</td><td>cd01372, KISc_KIF4, Kinesin m</td><td>ref|XP_005810383.1|</td><td>PREDICTED: kinesin-like protein KIF21A-like [Xiphophorus maculatus]</td><td>9,00E-29</td></tr>
<tr><td>gALL_v3_c13494</td><td>6</td><td>8</td><td>gnl|CDD|179170</td><td>PRK00927, PRK00927, tryptop</td><td>ref|XP_001600112.1|</td><td>PREDICTED: tryptophanyl-tRNA synthetase, mitochondrial-like [Nasonia vitripennis]</td><td>1,00E-111</td></tr>
<tr><td>gALL_v3_c13495</td><td>4</td><td>3</td><td>-</td><td>-</td><td>ref|XP_005102045.1|</td><td>PREDICTED: tigger transposable element-derived protein 4-like [Aplysia californica]</td><td>3,00E-13</td></tr>
<tr><td>gALL_v3_c13496</td><td>3</td><td>12</td><td>-</td><td>-</td><td>ref|XP_006571196.1|</td><td>PREDICTED: nesprin-1-like [Apis mellifera]</td><td>1,00E-39</td></tr>
<tr><td>gALL_v3_c13497</td><td>4</td><td>3</td><td>-</td><td>-</td><td>-</td><td>-</td><td>-</td></tr>
<tr><td>gALL_v3_c13498</td><td>6</td><td>4</td><td>-</td><td>-</td><td>-</td><td>-</td><td>-</td></tr>
<tr><td>gALL_v3_c13499</td><td>5</td><td>10</td><td>-</td><td>-</td><td>-</td><td>-</td><td>-</td></tr>
<tr><td>gALL_v3_c1350</td><td>13</td><td>19</td><td>gnl|CDD|162464</td><td>TIGR01649, hnRNP-L_PTB, hnF</td><td>gb|EFN77847.1|</td><td>Polypyrimidine tract-binding protein 2 [Harpegnathos saltator]</td><td>1,00E-83</td></tr>
<tr><td>gALL_v3_c13500</td><td>8</td><td>4</td><td>-</td><td>-</td><td>-</td><td>-</td><td>-</td></tr>
<tr><td>gALL_v3_c13501</td><td>1</td><td>4</td><td>gnl|CDD|144561</td><td>pfam01017, STAT_alpha, STAT</td><td>ref|XP_003401031.1|</td><td>PREDICTED: signal transducer and activator of transcription 5B-like [Bombus terrestris] ref|XP_003486926.1| PREDICTED: signal transducer and activator of transcription 5</td><td>4,00E-59</td></tr>
<tr><td>gALL_v3_c13502</td><td>6</td><td>5</td><td>gnl|CDD|30554</td><td>COG0205, PfkA, 6-phosphofru</td><td>ref|XP_001325139.1|</td><td>pyrophosphate-dependent fructose 6-phosphate 1-phosphotransferase [Trichomonas vaginalis G3] gb|AAD13344.1| pyrophosphate-dependent fructose 6-phosphate 1-ph</td><td>1,00E-139</td></tr>
<tr><td>gALL_v3_c13503</td><td>5</td><td>12</td><td>-</td><td>-</td><td>-</td><td>-</td><td>-</td></tr>
<tr><td>gALL_v3_c13504</td><td>3</td><td>2</td><td>-</td><td>-</td><td>-</td><td>-</td><td>-</td></tr>
<tr><td>gALL_v3_c13505</td><td>3</td><td>5</td><td>-</td><td>-</td><td>-</td><td>-</td><td>-</td></tr>
<tr><td>gALL_v3_c13506</td><td>4</td><td>7</td><td>gnl|CDD|144648</td><td>pfam01130, CD36, CD36 famil</td><td>ref|XP_968534.1|</td><td>PREDICTED: similar to scavenger receptor class B (AGAP002738-PA) [Tribolium castaneum] gb|EFA05668.1| hypothetical protein TcasGA2_TC014946 [Tribolium castaneum</td><td>1,00E-114</td></tr>
<tr><td>gALL_v3_c13507</td><td>4</td><td>4</td><td>-</td><td>-</td><td>-</td><td>-</td><td>-</td></tr>
<tr><td>gALL_v3_c13509</td><td>2</td><td>5</td><td>-</td><td>-</td><td>gb|EKC23172.1|</td><td>Thyroid adenoma-associated protein [Crassostrea gigas]</td><td>1,00E-11</td></tr>
<tr><td>gALL_v3_c13510</td><td>4</td><td>2</td><td>-</td><td>-</td><td>-</td><td>-</td><td>-</td></tr>
<tr><td>gALL_v3_c13511</td><td>2</td><td>3</td><td>-</td><td>-</td><td>-</td><td>-</td><td>-</td></tr>
<tr><td>gALL_v3_c13512</td><td>3</td><td>3</td><td>-</td><td>-</td><td>-</td><td>-</td><td>-</td></tr>
<tr><td>gALL_v3_c13513</td><td>4</td><td>2</td><td>-</td><td>-</td><td>-</td><td>-</td><td>-</td></tr>
<tr><td>gALL_v3_c13514</td><td>5</td><td>3</td><td>-</td><td>-</td><td>-</td><td>-</td><td>-</td></tr>
<tr><td>gALL_v3_c13515</td><td>5</td><td>5</td><td>-</td><td>-</td><td>ref|XP_001865394.1|</td><td>dumpy [Culex quinquefasciatus] gb|EDS41643.1| dumpy [Culex quinquefasciatus]</td><td>2,00E-93</td></tr>
<tr><td>gALL_v3_c13516</td><td>4</td><td>5</td><td>-</td><td>-</td><td>-</td><td>-</td><td>-</td></tr>
<tr><td>gALL_v3_c13517</td><td>2</td><td>3</td><td>-</td><td>-</td><td>-</td><td>-</td><td>-</td></tr>
<tr><td>gALL_v3_c13518</td><td>7</td><td>8</td><td>-</td><td>-</td><td>ref|XP_002424944.1|</td><td>conserved hypothetical protein [Pediculus humanus corporis] gb|EEB12206.1| conserved hypothetical protein [Pediculus humanus corporis]</td><td>1,00E-16</td></tr>
<tr><td>gALL_v3_c13519</td><td>3</td><td>3</td><td>gnl|CDD|29102</td><td>cd00162, RING, RING-finger (R</td><td>ref|XP_002423393.1|</td><td>hypothetical protein Phum_PHUM055710 [Pediculus humanus corporis] gb|EEB10655.1| hypothetical protein Phum_PHUM055710 [Pediculus humanus corporis]</td><td>6,00E-36</td></tr>
<tr><td>gALL_v3_c1352</td><td>13</td><td>13</td><td>-</td><td>-</td><td>-</td><td>-</td><td>-</td></tr>
<tr><td>gALL_v3_c13520</td><td>2</td><td>6</td><td>-</td><td>-</td><td>-</td><td>-</td><td>-</td></tr>
<tr><td>gALL_v3_c13521</td><td>4</td><td>7</td><td>-</td><td>-</td><td>-</td><td>-</td><td>-</td></tr>
<tr><td>gALL_v3_c13522</td><td>7</td><td>12</td><td>gnl|CDD|143840</td><td>pfam00055, Laminin_N, Lamir</td><td>gb|EFX84988.1|</td><td>hypothetical protein DAPPUDRAFT_300823 [Daphnia pulex]</td><td>0</td></tr>
<tr><td>gALL_v3_c13523</td><td>3</td><td>2</td><td>-</td><td>-</td><td>-</td><td>-</td><td>-</td></tr>
<tr><td>gALL_v3_c13525</td><td>2</td><td>6</td><td>-</td><td>-</td><td>-</td><td>-</td><td>-</td></tr>
<tr><td>gALL_v3_c13526</td><td>2</td><td>4</td><td>-</td><td>-</td><td>-</td><td>-</td><td>-</td></tr>
<tr><td>gALL_v3_c13528</td><td>1</td><td>6</td><td>-</td><td>-</td><td>-</td><td>-</td><td>-</td></tr>
<tr><td>gALL_v3_c13529</td><td>2</td><td>3</td><td>-</td><td>-</td><td>-</td><td>-</td><td>-</td></tr>
<tr><td>gALL_v3_c1353</td><td>9</td><td>9</td><td>-</td><td>-</td><td>-</td><td>-</td><td>-</td></tr>
</table>

gALL_v3_c13530	8	5	-	-	-	-	-
gALL_v3_c13531	4	2	-	-	-	-	-
gALL_v3_c13532	1	4	-	-	-	-	-
gALL_v3_c13534	6	5	-	-	-	-	-
gALL_v3_c13535	3	3	gnl|CDD|189372	pfam00067, p450, Cytochrom	sp|P29981.1|CP4C1_BL	RecName: Full=Cytochrome P450 4C1; AltName: Full=CYPIVC1 gb|AAA27819.1| cytochrome P450 [Blaberus discoidalis]	2,00E-51
gALL_v3_c13536	5	3	-	-	-	-	-
gALL_v3_c13537	1	4	-	-	-	-	-
gALL_v3_c13538	2	4	-	-	-	-	-
gALL_v3_c13539	3	2	-	-	-	-	-
gALL_v3_c13540	3	3	gnl|CDD|129062	smart00829, PKS_ER, Enoylred	ref|XP_003423914.1|	PREDICTED: fatty acid synthase-like isoform 2 [Nasonia vitripennis]	8,00E-53
gALL_v3_c13541	4	6	-	-	-	-	-
gALL_v3_c13542	16	18	-	-	-	-	-
gALL_v3_c13543	2	6	gnl|CDD|192548	pfam10351, Apt1, Golgi-body	ref|XP_001945624.2|	PREDICTED: UPF0378 protein KIAA0100-like [Acyrthosiphon pisum]	1,00E-112
gALL_v3_c13546	5	3	-	-	-	-	-
gALL_v3_c13547	1	5	gnl|CDD|34791	COG5192, BMS1, GTP-binding	ref|XP_006620479.1|	PREDICTED: ribosome biogenesis protein BMS1 homolog [Apis dorsata]	1,00E-72
gALL_v3_c13548	4	4	-	-	-	-	-
gALL_v3_c13549	1	5	-	-	gb|EFZ19026.1|	hypothetical protein SINV_06436 [Solenopsis invicta]	1,00E-48
gALL_v3_c1355	4	3	gnl|CDD|163663	cd07420, MPP_RdgC, Drosoph	ref|XP_317894.4|	AGAP011414-PA [Anopheles gambiae str. PEST] gb|EAA12933.4| AGAP011414-PA [Anopheles gambiae str. PEST]	1,00E-81
gALL_v3_c13550	2	3	-	-	-	-	-
gALL_v3_c13551	4	6	-	-	-	-	-
gALL_v3_c13552	5	9	-	-	gb|EFA04479.1|	hypothetical protein TcasGA2_TC014783 [Tribolium castaneum]	3,00E-45
gALL_v3_c13553	2	5	-	-	gb|ENN80834.1|	hypothetical protein YQE_02741, partial [Dendroctonus ponderosae]	6,00E-69
gALL_v3_c13554	5	7	-	-	-	-	-
gALL_v3_c13555	5	10	-	-	-	-	-
gALL_v3_c13556	1	4	gnl|CDD|189968	pfam01391, Collagen, Collager	ref|XP_002428942.1|	collagen alpha-2 precursor, putative [Pediculus humanus corporis] gb|EEB16204.1| collagen alpha-2 precursor, putative [Pediculus humanus corporis]	5,00E-24
gALL_v3_c13557	3	5	-	-	ref|XP_003221861.1|	PREDICTED: hypothetical protein LOC100567984 [Anolis carolinensis]	8,00E-28
gALL_v3_c13559	2	3	-	-	-	-	-
gALL_v3_c1356	18	19	-	-	-	-	-
gALL_v3_c13560	3	5	-	-	ref|XP_006619236.1|	PREDICTED: unconventional myosin-XV-like isoform X2 [Apis dorsata]	2,00E-30
gALL_v3_c13561	2	6	-	-	-	-	-
gALL_v3_c13562	2	3	-	-	ref|XP_004205475.1|	PREDICTED: RNA-directed DNA polymerase from mobile element jockey-like [Hydra vulgaris]	1,00E-06
gALL_v3_c13563	3	11	-	-	gb|EGI67485.1|	Nesprin-1 [Acromyrmex echinatior]	2,00E-50
gALL_v3_c13564	1	5	-	-	-	-	-
gALL_v3_c13565	6	7	-	-	-	-	-
gALL_v3_c13566	3	7	-	-	-	-	-
gALL_v3_c13567	3	2	-	-	-	-	-
gALL_v3_c13568	1	5	-	-	-	-	-
gALL_v3_c13569	6	6	gnl|CDD|48344	cd00433, Peptidase_M17, Cyt	gb|EPQ16757.1|	Putative aminopeptidase NPEPL1 [Myotis brandtii]	1,00E-104
gALL_v3_c1357	8	10	gnl|CDD|31001	COG0656, ARA1, Aldo/keto re	gb|AGM32585.1|	aldo-keto reductase (AKR), partial [Coptotermes formosanus]	8,00E-94
gALL_v3_c13571	3	5	-	-	gb|ESO93623.1|	hypothetical protein LOTGIDRAFT_189817 [Lottia gigantea]	1,00E-65
gALL_v3_c13572	4	3	-	-	-	-	-
gALL_v3_c13573	6	3	gnl|CDD|177211	MTH00154, COX2, cytochrome	gb|ACT10252.1|	cytochrome oxidase subunit II (mitochondrion) [Coptotermes formosanus] gb|ACT10253.1| cytochrome oxidase subunit II (mitochondrion) [Coptotermes formosanus] gb|	1,00E-94
gALL_v3_c13574	4	5	gnl|CDD|73009	cd03250, ABCC_MRP_domain	gb|ENN75125.1|	hypothetical protein YQE_08305, partial [Dendroctonus ponderosae]	3,00E-83
gALL_v3_c13575	2	5	-	-	-	-	-
gALL_v3_c13576	2	7	-	-	-	-	-
gALL_v3_c13577	4	4	gnl|CDD|30859	COG0513, SrmB, Superfamily I	ref|XP_969373.2|	PREDICTED: similar to CG9143 CG9143-PA [Tribolium castaneum]	1,00E-99
gALL_v3_c13578	7	8	gnl|CDD|190436	pfam02812, ELFV_dehydrog_N	gb|EFN77465.1|	Glutamate dehydrogenase, mitochondrial [Harpegnathos saltator]	1,00E-160
gALL_v3_c13579	4	6	-	-	gb|EKC23692.1|	Golgi-specific brefeldin A-resistance guanine nucleotide exchange factor 1 [Crassostrea gigas]	9,00E-46
gALL_v3_c1358	18	32	gnl|CDD|189860	pfam01151, ELO, GNS1/SUR4	ref|XP_003399522.1|	PREDICTED: elongation of very long chain fatty acids protein AAEL008004-like [Bombus terrestris]	1,00E-141
gALL_v3_c13580	7	12	-	-	-	-	-
gALL_v3_c13581	2	2	-	-	ref|XP_001599359.2|	PREDICTED: eukaryotic translation initiation factor 4H-like [Nasonia vitripennis]	1,00E-26
gALL_v3_c13582	4	4	-	-	ref|XP_003704622.1|	PREDICTED: uncharacterized protein LOC100882150 [Megachile rotundata]	2,00E-09
gALL_v3_c13584	2	5	gnl|CDD|73151	cd01644, RT_pepA17, RT_pep	ref|XP_003699175.1|	PREDICTED: uncharacterized protein LOC100874762 [Megachile rotundata]	1,00E-38
gALL_v3_c13585	2	8	-	-	-	-	-
gALL_v3_c13586	3	3	gnl|CDD|189795	pfam00995, Sec1, Sec1 family.	ref|XP_002426256.1|	vacuolar protein sorting, putative [Pediculus humanus corporis] gb|EEB13518.1| vacuolar protein sorting, putative [Pediculus humanus corporis]	2,00E-36
gALL_v3_c13587	3	3	-	-	gb|EFN80577.1|	Craniofacial development protein 2 [Harpegnathos saltator]	7,00E-35
gALL_v3_c13589	2	7	-	-	-	-	-
gALL_v3_c1359	11	15	gnl|CDD|128621	smart00326, SH3, Src homolog	gb|EFX87681.1|	hypothetical protein DAPPUDRAFT_43086 [Daphnia pulex]	2,00E-72
gALL_v3_c13590	1	3	-	-	gb|EFA11918.1|	hypothetical protein TcasGA2_TC004075 [Tribolium castaneum]	1,00E-13
gALL_v3_c13591	5	3	-	-	ref|XP_003488398.1|	PREDICTED: integrator complex subunit 9-like isoform 2 [Bombus impatiens]	1,00E-123
gALL_v3_c13592	18	39	gnl|CDD|73156	cd01650, RT_nLTR_like, RT_nL	ref|XP_003723613.1|	PREDICTED: RNA-directed DNA polymerase from mobile element jockey-like [Strongylocentrotus purpuratus]	5,00E-22
gALL_v3_c13593	3	5	-	-	-	-	-
gALL_v3_c13594	2	3	-	-	-	-	-
gALL_v3_c13595	2	4	gnl|CDD|178127	PLN02511, PLN02511, hydrola	gb|ETN57811.1|	alpha/beta hydrolase 1 [Anopheles darlingi]	4,00E-57
gALL_v3_c13596	2	3	-	-	-	-	-
gALL_v3_c13597	5	4	-	-	gb|EFN87262.1|	hypothetical protein EAI_02703 [Harpegnathos saltator]	8,00E-06
gALL_v3_c13598	3	4	-	-	-	-	-
gALL_v3_c13599	7	4	gnl|CDD|190505	pfam03024, Folate_rec, Folate	ref|XP_002430155.1|	Folate receptor alpha precursor, putative [Pediculus humanus corporis] gb|EEB17417.1| Folate receptor alpha precursor, putative [Pediculus humanus corporis]	3,00E-64
gALL_v3_c136	15	18	gnl|CDD|100072	cd02643, R3H_NF-X1, R3H dor	gb|EFN77337.1|	Protein shuttle craft [Harpegnathos saltator]	0
gALL_v3_c1360	20	33	-	-	-	-	-
gALL_v3_c13600	6	5	-	-	-	-	-
gALL_v3_c13601	2	6	-	-	-	-	-
gALL_v3_c13602	6	4	-	-	-	-	-
gALL_v3_c13605	7	4	gnl|CDD|190195	pfam02036, SCP2, SCP-2 stero	gb|ADJ17343.1|	sterol carrier protein 2 [Manduca sexta]	4,00E-40
gALL_v3_c13606	3	7	gnl|CDD|34637	COG5032, TEL1, Phosphatidyli	gb|ACH47049.1|	target of rapamycin [Blattella germanica]	0
gALL_v3_c13607	3	2	-	-	-	-	-
gALL_v3_c13609	4	3	-	-	-	-	-
gALL_v3_c1361	5	9	-	-	-	-	-
gALL_v3_c13610	3	9	-	-	ref|NP_001164122.1|	star protein [Acyrthosiphon pisum] dbj|BAH72647.1| ACYPI002776 [Acyrthosiphon pisum]	2,00E-90
gALL_v3_c13613	4	9	-	-	ref|XP_002430404.1|	tetratricopeptide repeat protein, putative [Pediculus humanus corporis] gb|EEB17666.1| tetratricopeptide repeat protein, putative [Pediculus humanus corporis]	4,00E-66
gALL_v3_c13614	5	7	-	-	ref|XP_003494020.1|	PREDICTED: short spindle protein 4-like isoform 2 [Bombus impatiens]	1,00E-45
gALL_v3_c13615	4	3	-	-	gb|AFP49818.1|	cytochrome P450 [Bemisia tabaci]	4,00E-12
gALL_v3_c13616	2	4	gnl|CDD|185517	PTZ00217, PTZ00217, flap end	gb|EGI61676.1|	Flap endonuclease 1-B [Acromyrmex echinatior]	2,00E-48
gALL_v3_c13617	4	8	gnl|CDD|189641	pfam00628, PHD, PHD-finger.	ref|XP_006608523.1|	PREDICTED: nucleosome-remodeling factor subunit NURF301-like isoform X2 [Apis dorsata]	1,00E-135
gALL_v3_c13618	5	4	-	-	-	-	-
gALL_v3_c13619	7	5	-	-	ref|XP_002073687.1|	GK14239 [Drosophila willistoni] gb|EDW84673.1| GK14239 [Drosophila willistoni]	3,00E-12
gALL_v3_c1362	6	5	gnl|CDD|73154	cd01647, RT_LTR, RT_LTR: Rev	ref|XP_002431501.1|	enzymatic polyprotein, putative [Pediculus humanus corporis] gb|EEB18763.1| enzymatic polyprotein, putative [Pediculus humanus corporis]	2,00E-13
gALL_v3_c13621	0	4	-	-	-	-	-
gALL_v3_c13622	2	3	-	-	-	-	-
gALL_v3_c13624	0	4	-	-	-	-	-
gALL_v3_c13626	6	3	gnl|CDD|113782	pfam05024, Gpi1, N-acetylglu	ref|XP_003485120.1|	PREDICTED: phosphatidylinositol N-acetylglucosaminyltransferase subunit Q-like [Bombus impatiens]	3,00E-52
gALL_v3_c13627	7	10	-	-	-	-	-
gALL_v3_c13628	2	3	-	-	-	-	-
gALL_v3_c13629	0	5	-	-	-	-	-
gALL_v3_c13630	9	7	gnl|CDD|73009	cd03250, ABCC_MRP_domain	gb|EFZ19975.1|	hypothetical protein SINV_05651 [Solenopsis invicta]	5,00E-83
gALL_v3_c13636	3	6	-	-	-	-	-
gALL_v3_c13637	3	2	-	-	ref|XP_006610510.1|	PREDICTED: CCR4-NOT transcription complex subunit 3-like isoform X1 [Apis dorsata]	2,00E-09
gALL_v3_c13638	4	7	-	-	-	-	-
gALL_v3_c13639	2	4	-	-	-	-	-
gALL_v3_c1364	6	3	-	-	-	-	-
gALL_v3_c13640	7	9	-	-	-	-	-
gALL_v3_c13641	1	4	-	-	-	-	-
gALL_v3_c13642	4	2	-	-	-	-	-
gALL_v3_c13643	5	3	-	-	-	-	-
gALL_v3_c13644	2	1	-	-	gb|AGM32388.1|	ubiquitin-40S ribosomal protein [Coptotermes formosanus]	9,00E-08
gALL_v3_c13645	3	3	-	-	-	-	-
gALL_v3_c13647	2	5	-	-	-	-	-
gALL_v3_c13649	0	5	-	-	-	-	-
gALL_v3_c1365	5	7	gnl|CDD|35216	COG5657, CSE1, CAS/CSE prot	ref|XP_003707327.1|	PREDICTED: transportin-1 [Megachile rotundata]	0
gALL_v3_c13650	4	2	-	-	-	-	-
gALL_v3_c13651	4	5	gnl|CDD|128577	smart00281, LamB, Laminin B	gb|EFN62243.1|	Basement membrane-specific heparan sulfate proteoglycan core protein [Camponotus floridanus]	1,00E-84
gALL_v3_c13653	2	2	-	-	ref|XP_001814720.1|	PREDICTED: similar to ATP synthase subunit s, mitochondrial [Tribolium castaneum] gb|EFA07591.1| ATP synthase, H+ transporting, mitochondrial F0 complex, subunit s (f	4,00E-29
gALL_v3_c13654	3	2	-	-	-	-	-
gALL_v3_c13655	8	11	-	-	-	-	-
gALL_v3_c13656	2	3	-	-	-	-	-
gALL_v3_c13657	5	4	-	-	ref|XP_002430601.1|	conserved hypothetical protein [Pediculus humanus corporis] gb|EEB17863.1| conserved hypothetical protein [Pediculus humanus corporis]	1,00E-101
gALL_v3_c13659	3	1	-	-	-	-	-
gALL_v3_c1366	7	6	gnl|CDD|112467	pfam03650, UPF0041, Unchar	ref|NP_001040310.1|	light-induced protein-like brain protein 44 [Bombyx mori] gb|ABD36190.1| light-induced protein-like brain protein 44 [Bombyx mori]	7,00E-53
gALL_v3_c13661	2	9	-	-	gb|AAR84629.1|	putative alcohol dehydrogenase [Gryllotalpa orientalis]	3,00E-09
gALL_v3_c13662	3	4	-	-	-	-	-
gALL_v3_c13663	5	11	gnl|CDD|173839	cd01150, AXO, Peroxisomal ac	ref|XP_002427455.1|	Acyl-coenzyme A oxidase 3, peroxisomal, putative [Pediculus humanus corporis] gb|EEB14717.1| Acyl-coenzyme A oxidase 3, peroxisomal, putative [Pediculus humanus cc	1,00E-108
gALL_v3_c13664	1	3	gnl|CDD|72907	cd04671, Nudix_Hydrolase_13	ref|XP_001951360.1|	PREDICTED: nucleoside diphosphate-linked moiety X motif 18-like [Acyrthosiphon pisum]	1,00E-51
gALL_v3_c13665	3	4	gnl|CDD|73154	cd01647, RT_LTR, RT_LTR: Rev	ref|XP_001810599.1|	PREDICTED: similar to pol polyprotein [Tribolium castaneum]	2,00E-49
gALL_v3_c13667	9	5	-	-	gb|EFZ23225.1|	hypothetical protein SINV_14704 [Solenopsis invicta]	6,00E-72
gALL_v3_c13668	3	3	-	-	-	-	-
gALL_v3_c13669	5	9	-	-	-	-	-
gALL_v3_c13670	3	3	-	-	-	-	-
gALL_v3_c13671	3	3	gnl|CDD|144398	pfam00786, PBD, P21-Rho-bin	ref|XP_003704588.1|	PREDICTED: serine/threonine-protein kinase PAK 1 isoform 1 [Megachile rotundata]	1,00E-46
gALL_v3_c13672	4	6	-	-	-	-	-
gALL_v3_c13673	1	7	-	-	-	-	-
gALL_v3_c13674	3	3	gnl|CDD|190489	pfam02970, TBCA, Tubulin bin	gb|EFN88956.1|	Tubulin-specific chaperone A [Harpegnathos saltator]	5,00E-39
gALL_v3_c13675	4	6	gnl|CDD|118308	pfam09776, Mitoc_L55, Mitoc	ref|XP_001955813.1|	GF18943 [Drosophila ananassae] gb|EDV44374.1| GF18943 [Drosophila ananassae]	1,00E-23
gALL_v3_c13676	3	5	-	-	gb|AAL40415.1|AF3698	endonuclease/reverse transcriptase [Branchiostoma floridae]	2,00E-11
gALL_v3_c13677	0	6	-	-	-	-	-
gALL_v3_c13678	2	2	-	-	ref|XP_002427535.1|	dynein beta chain, ciliary, putative [Pediculus humanus corporis] gb|EEB14797.1| dynein beta chain, ciliary, putative [Pediculus humanus corporis]	3,00E-56
gALL_v3_c1368	17	24	-	-	-	-	-
gALL_v3_c13680	2	3	-	-	-	-	-
gALL_v3_c13681	3	3	-	-	-	-	-
gALL_v3_c13682	3	3	gnl|CDD|131393	TIGR02340, chap_CCT_alpha,	gb|AHB33468.1|	T-complex protein subunit alpha [Locusta migratoria]	2,00E-66
gALL_v3_c13683	8	7	-	-	-	-	-
gALL_v3_c13684	5	11	gnl|CDD|147590	pfam05493, ATP_synt_H, ATP	ref|XP_006608187.1|	PREDICTED: V-type proton ATPase subunit e 2-like [Apis dorsata]	1,00E-36
gALL_v3_c13686	2	4	-	-	-	-	-
gALL_v3_c13687	7	4	gnl|CDD|189917	pfam01267, F-actin_cap_A, F-	ref|XP_003696288.1|	PREDICTED: F-actin-capping protein subunit alpha-like [Apis florea] ref|XP_006562207.1| PREDICTED: F-actin-capping protein subunit alpha-like [Apis mellifera] ref|XP_00	3,00E-91
gALL_v3_c13688	3	3	-	-	ref|XP_002429422.1|	conserved hypothetical protein [Pediculus humanus corporis] gb|EEB16684.1| conserved hypothetical protein [Pediculus humanus corporis]	6,00E-92

gALL_v3_c1369	6	9	-	-	-	-	-
gALL_v3_c13690	8	5	-	-	-	-	-
gALL_v3_c13691	2	2	-	-	-	-	-
gALL_v3_c13693	1	6	-	-	-	-	-
gALL_v3_c13694	2	3	-	-	ref|XP_003706297.1|	PREDICTED: protein toll-like [Megachile rotundata]	5,00E-10
gALL_v3_c13695	6	6	-	-	-	-	-
gALL_v3_c13696	5	2	-	-	-	-	-
gALL_v3_c13698	2	7	-	-	ref|XP_001605028.2|	PREDICTED: hypothetical protein LOC100121415 [Nasonia vitripennis]	2,00E-52
gALL_v3_c13699	3	4	-	-	-	-	-
gALL_v3_c137	22	17	gnl|CDD|133276	cd01874, Cdc42, Cdc42 subfar	dbj|BAN20477.1|	cdc42 homolog precursor [Riptortus pedestris]	3,00E-35
gALL_v3_c1370	15	11	-	-	-	-	-
gALL_v3_c13700	5	5	-	-	-	-	-
gALL_v3_c13701	3	3	-	-	ref|XP_006564269.1|	PREDICTED: AT-rich interactive domain-containing protein 4B-like [Apis mellifera]	8,00E-75
gALL_v3_c13702	3	4	gnl|CDD|145408	pfam02233, PNTB, NAD(P) tra	gb|EFX83224.1|	hypothetical protein DAPPUDRAFT_315656 [Daphnia pulex]	2,00E-56
gALL_v3_c13703	4	3	-	-	-	-	-
gALL_v3_c13704	4	6	gnl|CDD|179368	PRK02106, PRK02106, choline	ref|XP_972225.1|	PREDICTED: similar to AGAP003785-PA [Tribolium castaneum] gb|EFA05527.1| hypothetical protein TcasGA2_TC015714 [Tribolium castaneum]	5,00E-40
gALL_v3_c13706	3	2	-	-	-	-	-
gALL_v3_c13707	1	7	-	-	ref|XP_002427925.1|	tubulointerstitial nephritis antigen, putative [Pediculus humanus corporis] gb|EEB15187.1| tubulointerstitial nephritis antigen, putative [Pediculus humanus corporis]	2,00E-70
gALL_v3_c13708	3	3	-	-	-	-	-
gALL_v3_c13709	4	6	gnl|CDD|190248	pfam02212, GED, Dynamin GT	gb|ABN11996.1|	putative dynamin 1-like protein [Maconellicoccus hirsutus]	7,00E-74
gALL_v3_c1371	16	35	-	-	-	-	-
gALL_v3_c13710	2	3	-	-	-	-	-
gALL_v3_c13711	3	2	-	-	-	-	-
gALL_v3_c13712	7	4	-	-	dbj|BAO04164.1|	transposase [Spodoptera frugiperda]	5,00E-08
gALL_v3_c13713	1	7	-	-	-	-	-
gALL_v3_c13714	0	5	-	-	gb|AEA51302.1|	calcitonin-like diuretic hormone prepropeptide variant A [Rhodnius prolixus]	2,00E-33
gALL_v3_c13715	1	4	-	-	-	-	-
gALL_v3_c13716	2	4	-	-	-	-	-
gALL_v3_c13717	3	3	-	-	gb|AGH30328.1|	argonaute-3 [Nilaparvata lugens]	1,00E-06
gALL_v3_c13718	2	3	-	-	ref|XP_002426515.1|	hypothetical protein Phum_PHUM257850 [Pediculus humanus corporis] gb|EEB13777.1| hypothetical protein Phum_PHUM257850 [Pediculus humanus corporis]	9,00E-22
gALL_v3_c13719	3	2	-	-	-	-	-
gALL_v3_c1372	7	15	gnl|CDD|188903	cd09504, SAM_STIM-1,2-like,	ref|XP_006570265.1|	PREDICTED: stromal interaction molecule homolog isoform X4 [Apis mellifera]	1,00E-156
gALL_v3_c13721	3	3	gnl|CDD|145882	pfam02958, DUF227, Domain	gb|EFA07343.1|	hypothetical protein TcasGA2_TC015939 [Tribolium castaneum]	4,00E-19
gALL_v3_c13722	2	9	gnl|CDD|128688	smart00410, IG_like, Immunog	gb|EFN82310.1|	Basement membrane-specific heparan sulfate proteoglycan core protein [Harpegnathos saltator]	4,00E-65
gALL_v3_c13724	3	3	gnl|CDD|192900	pfam11969, DcpS_C, Scavenge	ref|XP_002431317.1|	histidine triad nucleotide binding protein, putative [Pediculus humanus corporis] gb|EEB18579.1| histidine triad nucleotide binding protein, putative [Pediculus humanus c	6,00E-44
gALL_v3_c13725	3	1	-	-	-	-	-
gALL_v3_c13726	2	10	-	-	gb|EHJ79034.1|	miniparamyosin [Danaus plexippus]	1,00E-20
gALL_v3_c13727	2	8	-	-	gb|ADI61823.1|	endonuclease-reverse transcriptase [Bombyx mori]	1,00E-32
gALL_v3_c13729	2	2	-	-	-	-	-
gALL_v3_c13730	1	5	-	-	-	-	-
gALL_v3_c13731	6	10	-	-	-	-	-
gALL_v3_c13732	7	12	-	-	-	-	-
gALL_v3_c13733	13	8	-	-	-	-	-
gALL_v3_c13734	4	11	gnl|CDD|191813	pfam07690, MFS_1, Major Fac	ref|XP_006558726.1|	PREDICTED: vesicular glutamate transporter 2 isoform X1 [Apis mellifera] ref|XP_006558727.1| PREDICTED: vesicular glutamate transporter 2 isoform X2 [Apis mellifera] re	1,00E-124
gALL_v3_c13735	2	5	-	-	-	-	-
gALL_v3_c13738	4	3	gnl|CDD|72896	cd03676, Nudix_hydrolase_3,	ref|XP_973446.2|	PREDICTED: similar to thiamin pyrophosphokinase [Tribolium castaneum]	6,00E-92
gALL_v3_c13739	9	6	gnl|CDD|34383	COG4770, COG4770, Acetyl/p	gb|EFX88799.1|	hypothetical protein DAPPUDRAFT_187192 [Daphnia pulex]	1,00E-135
gALL_v3_c1374	26	33	gnl|CDD|144575	pfam01034, Syndecan, Syndec	ref|XP_002433150.1|	syndecan-2 precursor, putative [Pediculus humanus corporis] gb|EEB20412.1| syndecan-2 precursor, putative [Pediculus humanus corporis]	1,00E-17
gALL_v3_c13741	2	3	-	-	-	-	-
gALL_v3_c13743	6	18	-	-	emb|CDJ35613.1|	hypothetical protein, conserved [Eimeria mitis]	5,00E-16
gALL_v3_c13744	4	5	-	-	-	-	-
gALL_v3_c13746	3	2	-	-	-	-	-
gALL_v3_c13749	4	12	-	-	ref|XP_002425070.1|	conserved hypothetical protein [Pediculus humanus corporis] gb|EEB12332.1| conserved hypothetical protein [Pediculus humanus corporis]	2,00E-50
gALL_v3_c13750	1	6	gnl|CDD|191256	pfam05327, RRN3, RNA polym	ref|XP_972768.1|	PREDICTED: similar to transcription initiation factor RRN3 [Tribolium castaneum]	6,00E-37
gALL_v3_c13751	2	5	-	-	ref|XP_001661622.1|	hypothetical protein AaeL_AAEL011371 [Aedes aegypti] gb|EAT36549.1| AAEL011371-PA [Aedes aegypti]	2,00E-14
gALL_v3_c13752	4	3	-	-	-	-	-
gALL_v3_c13753	3	8	-	-	-	-	-
gALL_v3_c13754	5	4	gnl|CDD|152009	pfam11573, Med23, Mediator	ref|XP_002430359.1|	CRSP complex subunit, putative [Pediculus humanus corporis] gb|EEB17621.1| CRSP complex subunit, putative [Pediculus humanus corporis]	1,00E-163
gALL_v3_c13755	9	18	-	-	-	-	-
gALL_v3_c13756	1	3	-	-	-	-	-
gALL_v3_c13758	5	9	-	-	-	-	-
gALL_v3_c13759	2	6	gnl|CDD|29577	cd02649, nuc_hydro_CeIAG, n	gb|EFX65347.1|	hypothetical protein DAPPUDRAFT_333266 [Daphnia pulex]	1,00E-93
gALL_v3_c13760	4	2	-	-	-	-	-
gALL_v3_c13761	2	8	-	-	-	-	-
gALL_v3_c13762	3	5	-	-	-	-	-
gALL_v3_c13763	2	5	-	-	-	-	-
gALL_v3_c13764	10	9	gnl|CDD|189954	pfam01359, Transposase_1, Tr	ref|XP_003702583.1|	PREDICTED: histone-lysine N-methyltransferase SETMAR-like [Megachile rotundata]	4,00E-83
gALL_v3_c13765	4	6	gnl|CDD|185615	PTZ00435, PTZ00435, isocitrat	gb|ADI81395.1|	isocitrate dehydrogenase [Heliconius melpomene melpomene] gb|ADI81397.1| isocitrate dehydrogenase [Heliconius melpomene melpomene] gb|ADI81399.1| isocitrate	1,00E-100
gALL_v3_c13766	2	4	gnl|CDD|189795	pfam00995, Sec1, Sec1 family.	gb|EFN78885.1|	Sly1 protein-like protein [Harpegnathos saltator]	8,00E-42
gALL_v3_c13768	3	7	-	-	gb|EFZ12683.1|	hypothetical protein SINV_08641 [Solenopsis invicta]	2,00E-14
gALL_v3_c13769	2	3	-	-	gb|EFN88312.1|	hypothetical protein EAI_08807 [Harpegnathos saltator]	8,00E-55
gALL_v3_c13770	4	2	gnl|CDD|153310	cd07626, BAR_SNX9_like, The	ref|XP_004927774.1|	PREDICTED: sorting nexin-9-like [Bombyx mori]	2,00E-33
gALL_v3_c13771	5	2	-	-	-	-	-
gALL_v3_c13772	2	5	gnl|CDD|191463	pfam06159, DUF974, Protein o	gb|EFN79377.1|	UPF0533 protein [Harpegnathos saltator]	1,00E-89
gALL_v3_c13774	8	9	-	-	gb|EFZ09896.1|	hypothetical protein SINV_03206 [Solenopsis invicta]	2,00E-13
gALL_v3_c13775	1	6	-	-	-	-	-
gALL_v3_c13777	4	2	-	-	gb|EFN75852.1|	Craniofacial development protein 2 [Harpegnathos saltator]	4,00E-16
gALL_v3_c13778	1	4	-	-	-	-	-
gALL_v3_c13779	1	5	gnl|CDD|129062	smart00829, PKS_ER, Enoylred	gb|EFA05203.1|	hypothetical protein TcasGA2_TC015339 [Tribolium castaneum]	8,00E-18
gALL_v3_c1378	5	4	-	-	-	-	-
gALL_v3_c13780	7	7	-	-	-	-	-
gALL_v3_c13781	0	5	-	-	-	-	-
gALL_v3_c13783	5	2	-	-	-	-	-
gALL_v3_c13784	5	3	gnl|CDD|189372	pfam00067, p450, Cytochrom	gb|ACN93795.1|	cytochrome p450 [Reticulitermes flavipes]	8,00E-86
gALL_v3_c13785	4	4	-	-	-	-	-
gALL_v3_c13786	4	3	-	-	-	-	-
gALL_v3_c13787	1	5	-	-	-	-	-
gALL_v3_c13788	6	6	-	-	-	-	-
gALL_v3_c13789	1	3	-	-	-	-	-
gALL_v3_c1379	5	14	-	-	-	-	-
gALL_v3_c13790	6	5	-	-	-	-	-
gALL_v3_c13791	3	1	gnl|CDD|191879	pfam07885, Ion_trans_2, Ion c	ref|XP_001605130.2|	PREDICTED: hypothetical protein LOC100121518 [Nasonia vitripennis]	7,00E-50
gALL_v3_c13792	2	2	-	-	ref|WP_006588044.1|	permease [Lactobacillus jensenii] gb|EEQ24559.1| hypothetical protein LACJE0001_1464 [Lactobacillus jensenii 269-3]	9,00E-60
gALL_v3_c13793	2	4	-	-	-	-	-
gALL_v3_c13794	3	1	-	-	-	-	-
gALL_v3_c13795	3	3	-	-	dbj|BAN20361.1|	conserved hypothetical protein [Riptortus pedestris]	2,00E-36
gALL_v3_c13796	3	4	-	-	gb|EFA04696.1|	hypothetical protein TcasGA2_TC014708 [Tribolium castaneum]	1,00E-14
gALL_v3_c13797	5	7	-	-	-	-	-
gALL_v3_c13798	3	5	-	-	-	-	-
gALL_v3_c13799	1	3	-	-	ref|XP_001948912.1|	PREDICTED: feline leukemia virus subgroup C receptor-related protein 2-like [Acyrthosiphon pisum]	1,00E-25
gALL_v3_c138	12	22	-	-	ref|XP_004930669.1|	PREDICTED: uncharacterized protein LOC101745102, partial [Bombyx mori]	5,00E-20
gALL_v3_c1380	16	17	-	-	-	-	-
gALL_v3_c13800	7	8	gnl|CDD|147945	pfam06058, DCP1, Dcp1-like d	gb|EHJ72962.1|	hypothetical protein KGM_11333 [Danaus plexippus]	1,00E-67
gALL_v3_c13801	5	4	gnl|CDD|185479	PTZ00146, PTZ00146, fibrillari	ref|XP_002424938.1|	E1b-55kD-associated protein, putative [Pediculus humanus corporis] gb|EEB12200.1| E1b-55kD-associated protein, putative [Pediculus humanus corporis]	7,00E-18
gALL_v3_c13802	2	3	-	-	gb|EFX90073.1|	hypothetical protein DAPPUDRAFT_309859 [Daphnia pulex]	3,00E-06
gALL_v3_c13803	11	10	-	-	-	-	-
gALL_v3_c13804	0	4	-	-	-	-	-
gALL_v3_c13805	2	5	gnl|CDD|129112	smart00879, Brix, Brix domain	gb|EFN70879.1|	Probable ribosome production factor 1 [Camponotus floridanus]	4,00E-78
gALL_v3_c13806	1	4	-	-	-	-	-
gALL_v3_c13807	2	3	-	-	-	-	-
gALL_v3_c13808	3	1	-	-	-	-	-
gALL_v3_c13810	1	4	gnl|CDD|129984	TIGR00906, 2A0303, cationic a	ref|XP_004522528.1|	PREDICTED: LOW QUALITY PROTEIN: cationic amino acid transporter 4-like [Ceratitis capitata]	1,00E-80
gALL_v3_c13812	3	2	-	-	gb|AAO27481.1|	HERC2 [Homo sapiens]	2,00E-70
gALL_v3_c13813	7	4	-	-	ref|XP_001600307.1|	PREDICTED: hypothetical protein LOC100115640 isoform 1 [Nasonia vitripennis]	2,00E-25
gALL_v3_c13814	7	4	gnl|CDD|99765	cd01728, LSm1, The eukaryoti	ref|XP_003706040.1|	PREDICTED: U6 snRNA-associated Sm-like protein LSm1-like [Megachile rotundata]	3,00E-49
gALL_v3_c13815	3	5	-	-	-	-	-
gALL_v3_c13816	3	2	gnl|CDD|34495	COG4886, COG4886, Leucine-r	gb|EFA11128.1|	hypothetical protein TcasGA2_TC004729 [Tribolium castaneum]	2,00E-11
gALL_v3_c13818	4	4	gnl|CDD|183087	PRK11325, PRK11325, scaffold	ref|XP_005424159.1|	PREDICTED: iron-sulfur cluster assembly enzyme ISCU, mitochondrial [Geospiza fortis]	8,00E-37
gALL_v3_c13819	1	5	-	-	-	-	-
gALL_v3_c1382	21	22	-	-	ref|XP_006571926.1|	PREDICTED: protein PRRC2C-like isoform X4 [Apis mellifera]	1,00E-35
gALL_v3_c13820	14	9	-	-	-	-	-
gALL_v3_c13821	3	5	-	-	-	-	-
gALL_v3_c13822	5	2	-	-	gb|EFX71403.1|	hypothetical protein DAPPUDRAFT_255836 [Daphnia pulex]	2,00E-06
gALL_v3_c13824	3	2	-	-	-	-	-
gALL_v3_c13825	6	5	-	-	-	-	-
gALL_v3_c13826	8	4	-	-	-	-	-
gALL_v3_c13827	4	6	-	-	gb|AEE62975.1|	unknown [Dendroctonus ponderosae]	4,00E-06
gALL_v3_c13828	13	16	gnl|CDD|176329	cd01253, PH_beta_spectrin, B	ref|XP_001950095.2|	PREDICTED: spectrin beta chain-like isoform 1 [Acyrthosiphon pisum]	1,00E-47
gALL_v3_c13829	2	5	-	-	-	-	-
gALL_v3_c1383	4	6	gnl|CDD|118773	pfam10249, NDUFB10, NADH-	ref|XP_001846420.1|	NADH dehydrogenase [Culex quinquefasciatus] gb|EDS43557.1| NADH dehydrogenase [Culex quinquefasciatus]	1,00E-83
gALL_v3_c13830	4	9	-	-	-	-	-
gALL_v3_c13831	9	14	gnl|CDD|99996	cd04300, GT1_Glycogen_Phos	gb|AEV89749.1|	glycogen phosphorylase [Schistocerca gregaria]	2,00E-27
gALL_v3_c13832	3	2	gnl|CDD|100015	cd02186, alpha_tubulin, The t	ref|XP_006777768.1|	PREDICTED: tubulin alpha-3 chain-like, partial [Myotis davidii]	3,00E-52
gALL_v3_c13833	2	3	-	-	gb|EGI61662.1|	Mariner Mos1 transposase [Acromyrmex echinatior]	1,00E-11
gALL_v3_c13834	3	3	-	-	ref|XP_006615508.1|	PREDICTED: type I inositol 3,4-bisphosphate 4-phosphatase-like isoform X1 [Apis dorsata] ref|XP_006615509.1| PREDICTED: type I inositol 3,4-bisphosphate 4-phosphatas	1,00E-67
gALL_v3_c13835	0	5	-	-	-	-	-
gALL_v3_c13836	3	3	-	-	-	-	-
gALL_v3_c13838	5	3	gnl|CDD|146960	pfam04572, Gb3_synth, Alpha	ref|XP_006615584.1|	PREDICTED: lactosylceramide 4-alpha-galactosyltransferase-like isoform X2 [Apis dorsata]	8,00E-26
gALL_v3_c13839	2	3	-	-	gb|AGM32210.1|	meichroacidin [Coptotermes formosanus]	2,00E-33
gALL_v3_c1384	8	5	-	-	-	-	-
gALL_v3_c13840	3	2	gnl|CDD|130450	TIGR01383, not_thiJ, DJ-1 fam	ref|XP_002610855.1|	hypothetical protein BRAFLDRAFT_94906 [Branchiostoma floridae] gb|EEN66865.1| hypothetical protein BRAFLDRAFT_94906 [Branchiostoma floridae]	9,00E-46
gALL_v3_c13841	6	3	-	-	-	-	-
gALL_v3_c13842	3	2	-	-	-	-	-
gALL_v3_c13843	0	4	gnl|CDD|175992	cd04026, C2_PKC_alpha_gamı	gb|EFA00194.1|	hypothetical protein TcasGA2_TC003019 [Tribolium castaneum]	4,00E-12

gALL_v3_c13844	2	4	gnl|CDD|189335	pfam00002, 7tm_2, 7 transme	ref|XP_002427184.1|	class B secretin-like G-protein coupled receptor GPRmth5, putative [Pediculus humanus corporis] gb|EEB14446.1| class B secretin-like G-protein coupled receptor GPRmth	7,00E-29
gALL_v3_c13845	3	3	-	-	-	-	-
gALL_v3_c13847	3	4	-	-	-	-	-
gALL_v3_c13848	6	6	gnl|CDD|191759	pfam07500, TFIIS_M, Transcri	ref|XP_006611986.1|	PREDICTED: uncharacterized protein LOC102676589 isoform X3 [Apis dorsata]	9,00E-64
gALL_v3_c13849	4	3	-	-	-	-	-
gALL_v3_c1385	15	11	gnl|CDD|31415	COG1222, RPT1, ATP-depende	gb|AEE62048.1|	unknown [Dendroctonus ponderosae]	0
gALL_v3_c13850	1	4	-	-	-	-	-
gALL_v3_c13851	15	13	-	-	-	-	-
gALL_v3_c13852	3	2	-	-	gb|AGM32195.1|	ribosomal protein L30 [Coptotermes formosanus]	2,00E-89
gALL_v3_c13853	6	6	-	-	gb|EFZ12256.1|	hypothetical protein SINV_06031 [Solenopsis invicta]	5,00E-79
gALL_v3_c13854	2	8	gnl|CDD|34909	COG5329, COG5329, Phospho	ref|XP_002425951.1|	predicted protein [Pediculus humanus corporis] gb|EEB13213.1| predicted protein [Pediculus humanus corporis]	1,00E-143
gALL_v3_c13855	3	8	-	-	-	-	-
gALL_v3_c13856	1	6	-	-	-	-	-
gALL_v3_c13857	1	4	-	-	-	-	-
gALL_v3_c1386	11	6	-	-	-	-	-
gALL_v3_c13860	7	5	gnl|CDD|133323	cd04123, Rab21, Rab21 subfa	gb|EFN74693.1|	Ras-related protein Rab-21 [Camponotus floridanus]	8,00E-82
gALL_v3_c13861	12	9	gnl|CDD|30973	COG0628, PerM, Predicted pe	ref|XP_973506.1|	PREDICTED: similar to conserved hypothetical protein [Tribolium castaneum] gb|EFA06639.1| hypothetical protein TcasGA2_TC009562 [Tribolium castaneum]	1,00E-156
gALL_v3_c13862	5	4	-	-	-	-	-
gALL_v3_c13864	10	6	-	-	-	-	-
gALL_v3_c13865	3	6	-	-	-	-	-
gALL_v3_c13866	8	9	gnl|CDD|151058	pfam10498, IFT57, Intra-flagel	ref|XP_006813858.1|	PREDICTED: intraflagellar transport protein 57 homolog [Saccoglossus kowalevskii]	1,00E-144
gALL_v3_c13867	2	3	-	-	-	-	-
gALL_v3_c13868	0	4	-	-	ref|XP_002424916.1|	faint sausage, putative [Pediculus humanus corporis] gb|EEB12178.1| faint sausage, putative [Pediculus humanus corporis]	3,00E-37
gALL_v3_c1387	41	29	-	-	-	-	-
gALL_v3_c13871	0	5	-	-	-	-	-
gALL_v3_c13872	3	3	gnl|CDD|162101	TIGR00911, 2A0308, L-type an	gb|EHJ69446.1|	putative cationic amino acid transporter [Danaus plexippus]	3,00E-49
gALL_v3_c13875	1	4	-	-	-	-	-
gALL_v3_c13876	4	3	-	-	gb|EEZ98559.1|	hypothetical protein TcasGA2_TC001066 [Tribolium castaneum]	5,00E-39
gALL_v3_c13877	1	4	-	-	-	-	-
gALL_v3_c13878	0	5	-	-	-	-	-
gALL_v3_c13880	12	8	gnl|CDD|190885	pfam04147, Nop14, Nop14-lik	tpg|DAA34585.1|	TPA_exp: mapmodulin-like protein [Amblyomma variegatum]	1,00E-07
gALL_v3_c13881	1	3	-	-	-	-	-
gALL_v3_c13882	5	2	-	-	-	-	-
gALL_v3_c13883	1	3	-	-	-	-	-
gALL_v3_c13884	2	3	-	-	-	-	-
gALL_v3_c13885	4	2	-	-	-	-	-
gALL_v3_c13886	2	4	-	-	ref|XP_004921864.1|	PREDICTED: neurochondrin homolog [Bombyx mori]	5,00E-45
gALL_v3_c13887	9	6	-	-	-	-	-
gALL_v3_c13888	12	7	-	-	-	-	-
gALL_v3_c13889	4	7	-	-	gb|AGM32136.1|	hypothetical protein [Coptotermes formosanus]	2,00E-06
gALL_v3_c1389	7	17	-	-	-	-	-
gALL_v3_c13890	1	3	-	-	-	-	-
gALL_v3_c13891	4	2	-	-	dbj|BAO04164.1|	transposase [Spodoptera frugiperda]	9,00E-08
gALL_v3_c13892	3	2	-	-	-	-	-
gALL_v3_c13894	2	8	-	-	ref|XP_001862022.1|	conserved hypothetical protein [Culex quinquefasciatus] gb|EDS36361.1| conserved hypothetical protein [Culex quinquefasciatus]	4,00E-15
gALL_v3_c13895	3	4	-	-	-	-	-
gALL_v3_c13896	3	5	-	-	gb|EFN68426.1|	Zinc finger Ran-binding domain-containing protein 2 [Camponotus floridanus]	1,00E-69
gALL_v3_c13897	4	6	gnl|CDD|58571	cd04270, ZnMc_TACE_like, Zi	ref|XP_002426567.1|	adam, putative [Pediculus humanus corporis] gb|EEB13829.1| adam, putative [Pediculus humanus corporis]	5,00E-72
gALL_v3_c13898	2	6	-	-	-	-	-
gALL_v3_c13899	3	2	-	-	-	-	-
gALL_v3_c139	5	12	gnl|CDD|145398	pfam02214, K_tetra, K+ chann	ref|XP_002424062.1|	BTB/POZ domain-containing protein kctd15, putative [Pediculus humanus corporis] gb|EEB11324.1| BTB/POZ domain-containing protein kctd15, putative [Pediculus huma	1,00E-122
gALL_v3_c13900	1	5	-	-	-	-	-
gALL_v3_c13901	4	2	-	-	dbj|BAN09207.1|	expanded protein [Gryllus bimaculatus]	1,00E-40
gALL_v3_c13902	5	2	-	-	-	-	-
gALL_v3_c13903	52	66	-	-	ref|XP_975975.1|	PREDICTED: similar to poly A binding protein isoform 4 [Tribolium castaneum] gb|EFA11220.1| hypothetical protein TcasGA2_TC005185 [Tribolium castaneum]	4,00E-07
gALL_v3_c13905	5	2	gnl|CDD|178622	PLN03073, PLN03073, ABC tra	gb|EHJ67881.1|	putative ATP-dependent transporter [Danaus plexippus]	1,00E-91
gALL_v3_c13906	2	3	-	-	-	-	-
gALL_v3_c13907	3	4	gnl|CDD|188047	TIGR00376, TIGR00376, DNA h	ref|XP_001949997.2|	PREDICTED: NFX1-type zinc finger-containing protein 1-like [Acyrthosiphon pisum]	4,00E-40
gALL_v3_c13908	4	3	-	-	-	-	-
gALL_v3_c13909	3	3	gnl|CDD|162084	TIGR00879, SP, MFS transport	gb|ETN59145.1|	sugar transporter [Anopheles darlingi]	7,00E-42
gALL_v3_c1391	11	14	-	-	gb|EFA02573.1|	hypothetical protein TcasGA2_TC008291 [Tribolium castaneum]	4,00E-22
gALL_v3_c13910	3	7	gnl|CDD|189916	pfam01266, DAO, FAD depend	ref|XP_003708313.1|	PREDICTED: pyruvate dehydrogenase phosphatase regulatory subunit, mitochondrial-like [Megachile rotundata]	2,00E-58
gALL_v3_c13911	3	2	-	-	-	-	-
gALL_v3_c13912	3	4	-	-	-	-	-
gALL_v3_c13913	1	5	-	-	-	-	-
gALL_v3_c13914	4	5	-	-	-	-	-
gALL_v3_c13915	4	7	-	-	-	-	-
gALL_v3_c13916	9	8	gnl|CDD|189411	pfam00135, COesterase, Carb	gb|ACT53738.1|	feruloyl esterase-like protein Est3 [Reticulitermes flavipes]	1,00E-105
gALL_v3_c13917	12	8	gnl|CDD|187598	cd05339, 17beta-HSDXI-like_S	gb|EGI70513.1|	Short chain dehydrogenase/reductase family 16C member 6 [Acromyrmex echinatior]	1,00E-113
gALL_v3_c13918	1	5	-	-	-	-	-
gALL_v3_c13919	4	2	-	-	-	-	-
gALL_v3_c13920	1	8	-	-	ref|XP_003382107.1|	retrovirus-related Pol polyprotein from transposon TNT 1-94 [Trichinella spiralis] gb|EFV61839.1| retrovirus-related Pol polyprotein from transposon TNT 1-94 [Trichinella s	2,00E-13
gALL_v3_c13921	4	6	-	-	-	-	-
gALL_v3_c13922	2	3	-	-	ref|XP_002430515.1|	predicted protein [Pediculus humanus corporis] gb|EEB17777.1| predicted protein [Pediculus humanus corporis]	1,00E-07
gALL_v3_c13923	4	4	-	-	-	-	-
gALL_v3_c13924	2	3	-	-	-	-	-
gALL_v3_c13925	4	3	gnl|CDD|130689	TIGR01628, PABP-1234, polya	ref|XP_001302991.1|	Polyadenylate-binding protein [Trichomonas vaginalis G3] gb|EAX90061.1| Polyadenylate-binding protein, putative [Trichomonas vaginalis G3]	5,00E-78
gALL_v3_c13926	3	10	-	-	ref|XP_002429605.1|	hypothetical protein Phum_PHUM441300 [Pediculus humanus corporis] gb|EEB16867.1| hypothetical protein Phum_PHUM441300 [Pediculus humanus corporis]	1,00E-50
gALL_v3_c13927	5	14	-	-	-	-	-
gALL_v3_c13928	1	4	-	-	-	-	-
gALL_v3_c13929	4	7	-	-	ref|XP_005319950.1|	PREDICTED: pogo transposable element with KRAB domain [Ictidomys tridecemlineatus]	5,00E-40
gALL_v3_c1393	9	10	-	-	gb|EFN72100.1|	Transposable element Tc3 transposase [Camponotus floridanus]	1,00E-26
gALL_v3_c13931	1	4	-	-	-	-	-
gALL_v3_c13932	2	3	-	-	-	-	-
gALL_v3_c13933	2	6	-	-	-	-	-
gALL_v3_c13934	4	8	-	-	-	-	-
gALL_v3_c13935	2	3	-	-	gb|EFA06378.1|	hypothetical protein TcasGA2_TC009254 [Tribolium castaneum]	7,00E-12
gALL_v3_c13937	13	13	gnl|CDD|189507	pfam00335, Tetraspannin, Tet	gb|ERL83974.1|	hypothetical protein D910_01284 [Dendroctonus ponderosae] gb|ERL86589.1| hypothetical protein D910_03996 [Dendroctonus ponderosae] gb|ERL88647.1| hypothetic	1,00E-115
gALL_v3_c13938	1	8	-	-	-	-	-
gALL_v3_c1394	21	20	gnl|CDD|128779	smart00503, SynN, Syntaxin N	gb|EFN69501.1|	Syntaxin-12 [Camponotus floridanus]	2,00E-77
gALL_v3_c13940	8	6	gnl|CDD|143459	cd07141, ALDH_F1AB_F2_RAL	ref|XP_003402159.1|	PREDICTED: aldehyde dehydrogenase X, mitochondrial-like [Bombus terrestris]	1,00E-113
gALL_v3_c13941	1	5	-	-	-	-	-
gALL_v3_c13942	4	4	-	-	gb|AGM32493.1|	39S ribosomal protein L32-like protein [Coptotermes formosanus]	1,00E-116
gALL_v3_c13943	2	2	-	-	ref|XP_002425085.1|	conserved hypothetical protein [Pediculus humanus corporis] gb|EEB12347.1| conserved hypothetical protein [Pediculus humanus corporis]	3,00E-06
gALL_v3_c13944	6	6	gnl|CDD|32366	COG2183, Tex, Transcriptional	ref|XP_002424176.1|	transcription elongation factor SPT6, putative [Pediculus humanus corporis] gb|EEB11438.1| transcription elongation factor SPT6, putative [Pediculus humanus corporis]	1,00E-131
gALL_v3_c13945	4	2	-	-	-	-	-
gALL_v3_c13946	1	3	-	-	-	-	-
gALL_v3_c13947	9	8	-	-	-	-	-
gALL_v3_c13949	2	7	-	-	-	-	-
gALL_v3_c1395	8	8	-	-	-	-	-
gALL_v3_c13952	7	5	-	-	-	-	-
gALL_v3_c13953	2	3	-	-	-	-	-
gALL_v3_c13955	2	3	-	-	-	-	-
gALL_v3_c13956	1	3	gnl|CDD|189758	pfam00887, ACBP, Acyl CoA bi	gb|AGI15434.1|	acyl-CoA-binding protein 4 [Rhodnius prolixus]	3,00E-54
gALL_v3_c13957	2	3	-	-	-	-	-
gALL_v3_c13958	7	7	-	-	-	-	-
gALL_v3_c13959	3	2	-	-	gb|EFN77971.1|	2-hydroxyacyl-CoA lyase 1 [Harpegnathos saltator]	5,00E-08
gALL_v3_c1396	6	9	-	-	-	-	-
gALL_v3_c13960	4	7	-	-	-	-	-
gALL_v3_c13961	1	3	-	-	gb|EFA13284.1|	hypothetical protein TcasGA2_TC010304 [Tribolium castaneum]	2,00E-20
gALL_v3_c13962	2	2	-	-	-	-	-
gALL_v3_c13964	0	4	-	-	-	-	-
gALL_v3_c13965	1	4	-	-	-	-	-
gALL_v3_c13966	3	9	gnl|CDD|133284	cd01884, EF_Tu, EF-Tu subfam	ref|XP_003693252.1|	PREDICTED: elongation factor Tu, mitochondrial-like [Apis florea]	1,00E-160
gALL_v3_c13967	2	9	-	-	ref|XP_005105174.1|	PREDICTED: NADH-cytochrome b5 reductase-like [Aplysia californica]	1,00E-18
gALL_v3_c13968	1	6	-	-	-	-	-
gALL_v3_c13969	5	2	-	-	-	-	-
gALL_v3_c1397	4	6	-	-	ref|XP_004932245.1|	PREDICTED: mitochondrial import receptor subunit TOM22 homolog isoform X2 [Bombyx mori]	8,00E-30
gALL_v3_c13970	5	2	-	-	-	-	-
gALL_v3_c13971	3	4	-	-	-	-	-
gALL_v3_c13972	0	5	-	-	-	-	-
gALL_v3_c13973	4	2	-	-	-	-	-
gALL_v3_c13974	0	4	-	-	gb|AGM32707.1|	hypothetical protein, partial [Coptotermes formosanus]	2,00E-07
gALL_v3_c13976	2	4	gnl|CDD|178829	PRK00055, PRK00055, ribonuc	ref|XP_002598072.1|	hypothetical protein BRAFLDRAFT_124299 [Branchiostoma floridae] gb|EEN54084.1| hypothetical protein BRAFLDRAFT_124299 [Branchiostoma floridae]	4,00E-14
gALL_v3_c13977	1	4	-	-	-	-	-
gALL_v3_c13978	3	6	-	-	-	-	-
gALL_v3_c13979	3	2	-	-	-	-	-
gALL_v3_c1398	11	6	-	-	-	-	-
gALL_v3_c13980	3	4	-	-	-	-	-
gALL_v3_c13981	0	5	-	-	-	-	-
gALL_v3_c13983	3	2	gnl|CDD|191240	pfam05236, TAF4, Transcriptio	ref|XP_003702407.1|	PREDICTED: transcription initiation factor TFIID subunit 4-like [Megachile rotundata]	5,00E-29
gALL_v3_c13984	1	5	gnl|CDD|162863	TIGR02456, treS_nterm, treha	gb|AEP03183.1|	sucrase [Diuraphis noxia]	7,00E-53
gALL_v3_c13986	6	5	gnl|CDD|189654	pfam00651, BTB, BTB/POZ do	gb|EFA08790.1|	hypothetical protein TcasGA2_TC006481 [Tribolium castaneum]	5,00E-72
gALL_v3_c13987	2	4	-	-	-	-	-
gALL_v3_c13988	1	5	-	-	-	-	-
gALL_v3_c13989	4	5	-	-	ref|XP_006029730.1|	PREDICTED: histone-lysine N-methyltransferase SETMAR-like [Alligator sinensis]	1,00E-07
gALL_v3_c13991	3	3	gnl|CDD|192619	pfam10537, WAC_Acf1_DNA_	ref|XP_971434.2|	PREDICTED: similar to zinc finger protein [Tribolium castaneum]	1,00E-32
gALL_v3_c13993	4	8	-	-	-	-	-
gALL_v3_c13994	1	4	-	-	-	-	-
gALL_v3_c13995	2	4	-	-	-	-	-
gALL_v3_c13996	2	6	gnl|CDD|190777	pfam03868, Ribosomal_L6e_N	gb|AGM32491.1|	60S ribosomal protein L6 [Coptotermes formosanus]	2,00E-33
gALL_v3_c13997	7	20	-	-	ref|XP_970914.1|	PREDICTED: similar to CG13124 CG13124-PA [Tribolium castaneum] gb|EFA04338.1| hypothetical protein TcasGA2_TC014632 [Tribolium castaneum]	3,00E-11
gALL_v3_c13998	2	3	-	-	-	-	-
gALL_v3_c13999	9	13	-	-	-	-	-

<table>
<tr><td></td><td></td><td></td><td></td><td></td><td></td><td></td><td></td></tr>
<tr><td>gALL_v3_c14</td><td>9</td><td>7</td><td>-</td><td>-</td><td>-</td><td>-</td><td>-</td></tr>
<tr><td>gALL_v3_c140</td><td>7</td><td>4</td><td>gnl|CDD|128524</td><td>smart00228, PDZ, Domain pre</td><td>gb|EFZ12124.1|</td><td>hypothetical protein SINV_08215 [Solenopsis invicta]</td><td>7,00E-32</td></tr>
<tr><td>gALL_v3_c1400</td><td>6</td><td>4</td><td>gnl|CDD|178407</td><td>PLN02811, PLN02811, hydrola</td><td>ref|XP_004524721.1|</td><td>PREDICTED: probable pseudouridine-5'-monophosphatase-like isoform X1 [Ceratitis capitata]</td><td>1,00E-70</td></tr>
<tr><td>gALL_v3_c14000</td><td>3</td><td>2</td><td>-</td><td>-</td><td>-</td><td>-</td><td>-</td></tr>
<tr><td>gALL_v3_c14002</td><td>2</td><td>5</td><td>-</td><td>-</td><td>-</td><td>-</td><td>-</td></tr>
<tr><td>gALL_v3_c14003</td><td>1</td><td>7</td><td>gnl|CDD|173623</td><td>cd00180, PKc, Catalytic domai</td><td>gb|EGI63291.1|</td><td>Serine/threonine-protein kinase WNK1 [Acromyrmex echinatior]</td><td>7,00E-75</td></tr>
<tr><td>gALL_v3_c14004</td><td>0</td><td>6</td><td>-</td><td>-</td><td>-</td><td>-</td><td>-</td></tr>
<tr><td>gALL_v3_c14005</td><td>6</td><td>12</td><td>-</td><td>-</td><td>-</td><td>-</td><td>-</td></tr>
<tr><td>gALL_v3_c14006</td><td>3</td><td>1</td><td>-</td><td>-</td><td>gb|EGI62493.1|</td><td>Ankyrin repeat and sterile alpha motif domain-containing protein 1B [Acromyrmex echinatior]</td><td>7,00E-31</td></tr>
<tr><td>gALL_v3_c14007</td><td>2</td><td>3</td><td>-</td><td>-</td><td>ref|XP_001605325.2|</td><td>PREDICTED: hypothetical protein LOC100121715 [Nasonia vitripennis]</td><td>2,00E-06</td></tr>
<tr><td>gALL_v3_c14008</td><td>6</td><td>5</td><td>gnl|CDD|117221</td><td>pfam08648, DUF1777, Protein</td><td>-</td><td>-</td><td>-</td></tr>
<tr><td>gALL_v3_c14009</td><td>1</td><td>8</td><td>-</td><td>-</td><td>-</td><td>-</td><td>-</td></tr>
<tr><td>gALL_v3_c14010</td><td>5</td><td>3</td><td>gnl|CDD|128559</td><td>smart00263, LYZ1, Alpha-lacta</td><td>gb|ACH56920.1|</td><td>salivary lysozyme [Simulium vittatum]</td><td>3,00E-26</td></tr>
<tr><td>gALL_v3_c14012</td><td>3</td><td>3</td><td>-</td><td>-</td><td>ref|XP_003492108.1|</td><td>PREDICTED: RNA pseudouridylate synthase domain-containing protein 4-like [Bombus impatiens]</td><td>3,00E-21</td></tr>
<tr><td>gALL_v3_c14015</td><td>2</td><td>4</td><td>gnl|CDD|73156</td><td>cd01650, RT_nLTR_like, RT_nL</td><td>gb|ADI61823.1|</td><td>endonuclease-reverse transcriptase [Bombyx mori]</td><td>1,00E-43</td></tr>
<tr><td>gALL_v3_c14016</td><td>8</td><td>7</td><td>-</td><td>-</td><td>gb|ERL89933.1|</td><td>hypothetical protein D910_07292 [Dendroctonus ponderosae]</td><td>6,00E-59</td></tr>
<tr><td>gALL_v3_c14017</td><td>3</td><td>4</td><td>-</td><td>-</td><td>-</td><td>-</td><td>-</td></tr>
<tr><td>gALL_v3_c14018</td><td>3</td><td>10</td><td>-</td><td>-</td><td>-</td><td>-</td><td>-</td></tr>
<tr><td>gALL_v3_c1402</td><td>24</td><td>34</td><td>gnl|CDD|145291</td><td>pfam02029, Caldesmon, Calde</td><td>ref|XP_002431082.1|</td><td>JmjC domain-containing histone demethylation protein 2B, putative [Pediculus humanus corporis] gb|EEB18344.1| JmjC domain-containing histone demethylation protein</td><td>4,00E-14</td></tr>
<tr><td>gALL_v3_c14020</td><td>8</td><td>10</td><td>-</td><td>-</td><td>-</td><td>-</td><td>-</td></tr>
<tr><td>gALL_v3_c14021</td><td>4</td><td>7</td><td>gnl|CDD|28992</td><td>cd01177, IPT_NFkappaB, IPT d</td><td>gb|AAZ08475.1|</td><td>relish [Nasutitermes dixoni]</td><td>1,00E-132</td></tr>
<tr><td>gALL_v3_c14023</td><td>2</td><td>4</td><td>-</td><td>-</td><td>ref|XP_002422851.1|</td><td>hypothetical protein Phum_PHUM019010 [Pediculus humanus corporis] gb|EEB10113.1| hypothetical protein Phum_PHUM019010 [Pediculus humanus corporis]</td><td>1,00E-07</td></tr>
<tr><td>gALL_v3_c14025</td><td>1</td><td>3</td><td>-</td><td>-</td><td>-</td><td>-</td><td>-</td></tr>
<tr><td>gALL_v3_c14026</td><td>2</td><td>4</td><td>-</td><td>-</td><td>ref|XP_002430832.1|</td><td>conserved hypothetical protein [Pediculus humanus corporis] gb|EEB18094.1| conserved hypothetical protein [Pediculus humanus corporis]</td><td>1,00E-77</td></tr>
<tr><td>gALL_v3_c14027</td><td>1</td><td>4</td><td>gnl|CDD|191810</td><td>pfam07679, I-set, Immunoglol</td><td>ref|XP_002431788.1|</td><td>papilin, putative [Pediculus humanus corporis] gb|EEB19050.1| papilin, putative [Pediculus humanus corporis]</td><td>2,00E-35</td></tr>
<tr><td>gALL_v3_c14028</td><td>9</td><td>5</td><td>gnl|CDD|189654</td><td>pfam00651, BTB, BTB/POZ dor</td><td>ref|XP_002430150.1|</td><td>zinc finger protein and BTB domain-containing protein, putative [Pediculus humanus corporis] gb|EEB17412.1| zinc finger protein and BTB domain-containing protein, puta</td><td>2,00E-88</td></tr>
<tr><td>gALL_v3_c14029</td><td>1</td><td>3</td><td>-</td><td>-</td><td>-</td><td>-</td><td>-</td></tr>
<tr><td>gALL_v3_c1403</td><td>9</td><td>7</td><td>-</td><td>-</td><td>ref|NP_001232947.1|</td><td>uncharacterized protein LOC100168217 [Acyrthosiphon pisum] dbj|BAH70927.1| ACYPI008939 [Acyrthosiphon pisum]</td><td>3,00E-28</td></tr>
<tr><td>gALL_v3_c14030</td><td>0</td><td>6</td><td>-</td><td>-</td><td>-</td><td>-</td><td>-</td></tr>
<tr><td>gALL_v3_c14031</td><td>6</td><td>5</td><td>-</td><td>-</td><td>-</td><td>-</td><td>-</td></tr>
<tr><td>gALL_v3_c14032</td><td>1</td><td>3</td><td>-</td><td>-</td><td>-</td><td>-</td><td>-</td></tr>
<tr><td>gALL_v3_c14033</td><td>3</td><td>2</td><td>-</td><td>-</td><td>-</td><td>-</td><td>-</td></tr>
<tr><td>gALL_v3_c14034</td><td>3</td><td>6</td><td>-</td><td>-</td><td>-</td><td>-</td><td>-</td></tr>
<tr><td>gALL_v3_c14035</td><td>3</td><td>1</td><td>gnl|CDD|178175</td><td>PLN02561, PLN02561, triosepl</td><td>ref|XP_001320336.1|</td><td>triosephosphate isomerase [Trichomonas vaginalis G3] gb|EAY08113.1| triosephosphate isomerase, putative [Trichomonas vaginalis G3]</td><td>2,00E-69</td></tr>
<tr><td>gALL_v3_c14036</td><td>13</td><td>26</td><td>-</td><td>-</td><td>-</td><td>-</td><td>-</td></tr>
<tr><td>gALL_v3_c14037</td><td>9</td><td>9</td><td>-</td><td>-</td><td>gb|EFN82275.1|</td><td>Histone-lysine N-methyltransferase, H3 lysine-79 specific [Harpegnathos saltator]</td><td>2,00E-81</td></tr>
<tr><td>gALL_v3_c14038</td><td>10</td><td>12</td><td>gnl|CDD|165506</td><td>PHA03247, PHA03247, large te</td><td>ref|XP_007034397.1|</td><td>Uncharacterized protein TCM_046763 [Theobroma cacao] gb|EOY05323.1| Uncharacterized protein TCM_046763 [Theobroma cacao]</td><td>5,00E-06</td></tr>
<tr><td>gALL_v3_c1404</td><td>7</td><td>7</td><td>gnl|CDD|178204</td><td>PLN02594, PLN02594, phosph</td><td>gb|EGI57387.1|</td><td>Phosphatidate cytidylyltransferase, photoreceptor-specific [Acromyrmex echinatior]</td><td>1,00E-75</td></tr>
<tr><td>gALL_v3_c14040</td><td>5</td><td>3</td><td>gnl|CDD|112701</td><td>pfam03901, Glyco_transf_22,</td><td>ref|XP_971096.1|</td><td>PREDICTED: similar to CG11851 CG11851-PA [Tribolium castaneum] gb|EFA03085.1| hypothetical protein TcasGA2_TC012991 [Tribolium castaneum]</td><td>2,00E-49</td></tr>
<tr><td>gALL_v3_c14041</td><td>2</td><td>4</td><td>-</td><td>-</td><td>-</td><td>-</td><td>-</td></tr>
<tr><td>gALL_v3_c14042</td><td>3</td><td>5</td><td>-</td><td>-</td><td>-</td><td>-</td><td>-</td></tr>
<tr><td>gALL_v3_c14043</td><td>5</td><td>5</td><td>-</td><td>-</td><td>-</td><td>-</td><td>-</td></tr>
<tr><td>gALL_v3_c14046</td><td>4</td><td>2</td><td>gnl|CDD|109113</td><td>pfam00044, Gp_dh_N, Glycera</td><td>dbj|BAF48668.1|</td><td>glyceraldehyde-3-phosphate dehydrogenase [Pseudotrichonympha grassii]</td><td>1,00E-73</td></tr>
<tr><td>gALL_v3_c14047</td><td>1</td><td>7</td><td>-</td><td>-</td><td>-</td><td>-</td><td>-</td></tr>
<tr><td>gALL_v3_c14049</td><td>3</td><td>4</td><td>-</td><td>-</td><td>ref|XP_002427184.1|</td><td>class B secretin-like G-protein coupled receptor GPRmth5, putative [Pediculus humanus corporis] gb|EEB14446.1| class B secretin-like G-protein coupled receptor GPRmth</td><td>8,00E-06</td></tr>
<tr><td>gALL_v3_c1405</td><td>9</td><td>17</td><td>-</td><td>-</td><td>-</td><td>-</td><td>-</td></tr>
<tr><td>gALL_v3_c14050</td><td>3</td><td>3</td><td>-</td><td>-</td><td>-</td><td>-</td><td>-</td></tr>
<tr><td>gALL_v3_c14051</td><td>1</td><td>8</td><td>-</td><td>-</td><td>ref|XP_006622089.1|</td><td>PREDICTED: protein dopey-1 homolog isoform X5 [Apis dorsata]</td><td>8,00E-21</td></tr>
<tr><td>gALL_v3_c14052</td><td>5</td><td>2</td><td>-</td><td>-</td><td>gb|ADI61823.1|</td><td>endonuclease-reverse transcriptase [Bombyx mori]</td><td>2,00E-19</td></tr>
<tr><td>gALL_v3_c14053</td><td>3</td><td>6</td><td>gnl|CDD|107365</td><td>cd06370, PBP1_Speract_GC_li</td><td>ref|XP_002423970.1|</td><td>Guanylate cyclase 32E precursor, putative [Pediculus humanus corporis] gb|EEB11232.1| Guanylate cyclase 32E precursor, putative [Pediculus humanus corporis]</td><td>1,00E-115</td></tr>
<tr><td>gALL_v3_c14054</td><td>2</td><td>4</td><td>-</td><td>-</td><td>-</td><td>-</td><td>-</td></tr>
<tr><td>gALL_v3_c14055</td><td>2</td><td>6</td><td>-</td><td>-</td><td>-</td><td>-</td><td>-</td></tr>
<tr><td>gALL_v3_c14056</td><td>3</td><td>5</td><td>gnl|CDD|162740</td><td>TIGR02169, SMC_prok_A, chrc</td><td>ref|XP_975548.1|</td><td>PREDICTED: similar to AGAP010985-PA [Tribolium castaneum] gb|EFA02483.1| hypothetical protein TcasGA2_TC008178 [Tribolium castaneum]</td><td>2,00E-36</td></tr>
<tr><td>gALL_v3_c14057</td><td>5</td><td>3</td><td>-</td><td>-</td><td>-</td><td>-</td><td>-</td></tr>
<tr><td>gALL_v3_c14058</td><td>3</td><td>3</td><td>-</td><td>-</td><td>-</td><td>-</td><td>-</td></tr>
<tr><td>gALL_v3_c14059</td><td>2</td><td>6</td><td>-</td><td>-</td><td>-</td><td>-</td><td>-</td></tr>
<tr><td>gALL_v3_c14060</td><td>3</td><td>3</td><td>-</td><td>-</td><td>-</td><td>-</td><td>-</td></tr>
<tr><td>gALL_v3_c14062</td><td>2</td><td>6</td><td>gnl|CDD|153410</td><td>cd07956, Anticodon_Ia_Arg, A</td><td>ref|XP_966449.1|</td><td>PREDICTED: similar to arginyl-tRNA synthetase [Tribolium castaneum] gb|EFA00823.1| hypothetical protein TcasGA2_TC003711 [Tribolium castaneum]</td><td>9,00E-48</td></tr>
<tr><td>gALL_v3_c14063</td><td>3</td><td>11</td><td>-</td><td>-</td><td>ref|XP_006018972.1|</td><td>PREDICTED: protein CREG1, partial [Alligator sinensis]</td><td>3,00E-24</td></tr>
<tr><td>gALL_v3_c14065</td><td>2</td><td>6</td><td>-</td><td>-</td><td>ref|XP_003242319.1|</td><td>PREDICTED: histone-lysine N-methyltransferase SETMAR-like [Acyrthosiphon pisum]</td><td>3,00E-07</td></tr>
<tr><td>gALL_v3_c14066</td><td>2</td><td>3</td><td>-</td><td>-</td><td>-</td><td>-</td><td>-</td></tr>
<tr><td>gALL_v3_c14068</td><td>4</td><td>3</td><td>-</td><td>-</td><td>-</td><td>-</td><td>-</td></tr>
<tr><td>gALL_v3_c14069</td><td>7</td><td>7</td><td>-</td><td>-</td><td>-</td><td>-</td><td>-</td></tr>
<tr><td>gALL_v3_c14071</td><td>1</td><td>4</td><td>-</td><td>-</td><td>-</td><td>-</td><td>-</td></tr>
<tr><td>gALL_v3_c14072</td><td>4</td><td>8</td><td>-</td><td>-</td><td>-</td><td>-</td><td>-</td></tr>
<tr><td>gALL_v3_c14073</td><td>5</td><td>5</td><td>gnl|CDD|100104</td><td>cd00590, RRM, RRM (RNA reco</td><td>ref|XP_006570438.1|</td><td>PREDICTED: probable RNA-binding protein 19-like isoform X2 [Apis mellifera]</td><td>1,00E-144</td></tr>
<tr><td>gALL_v3_c14074</td><td>10</td><td>10</td><td>-</td><td>-</td><td>-</td><td>-</td><td>-</td></tr>
<tr><td>gALL_v3_c14075</td><td>3</td><td>5</td><td>gnl|CDD|189970</td><td>pfam01395, PBP_GOBP, PBP/G</td><td>gb|AEP27187.1|</td><td>pheromone-binding protein [Frankliniella occidentalis]</td><td>5,00E-29</td></tr>
<tr><td>gALL_v3_c14077</td><td>4</td><td>3</td><td>gnl|CDD|189956</td><td>pfam01365, RYDR_ITPR, RIH d</td><td>gb|EFN76414.1|</td><td>Inositol 1,4,5-trisphosphate receptor [Harpegnathos saltator]</td><td>1,00E-131</td></tr>
<tr><td>gALL_v3_c14079</td><td>2</td><td>6</td><td>-</td><td>-</td><td>-</td><td>-</td><td>-</td></tr>
<tr><td>gALL_v3_c1408</td><td>7</td><td>5</td><td>-</td><td>-</td><td>ref|XP_001812360.1|</td><td>PREDICTED: similar to RNA binding motif protein 34 [Tribolium castaneum] gb|EFA04264.1| hypothetical protein TcasGA2_TC014554 [Tribolium castaneum]</td><td>7,00E-23</td></tr>
<tr><td>gALL_v3_c14080</td><td>1</td><td>4</td><td>-</td><td>-</td><td>-</td><td>-</td><td>-</td></tr>
<tr><td>gALL_v3_c14081</td><td>3</td><td>1</td><td>-</td><td>-</td><td>gb|EHJ75861.1|</td><td>hypothetical protein KGM_17995 [Danaus plexippus]</td><td>3,00E-36</td></tr>
<tr><td>gALL_v3_c14082</td><td>2</td><td>2</td><td>-</td><td>-</td><td>-</td><td>-</td><td>-</td></tr>
<tr><td>gALL_v3_c14083</td><td>2</td><td>4</td><td>-</td><td>-</td><td>-</td><td>-</td><td>-</td></tr>
<tr><td>gALL_v3_c14084</td><td>3</td><td>4</td><td>-</td><td>-</td><td>dbj|BAC25351.1|</td><td>unnamed protein product [Mus musculus]</td><td>2,00E-07</td></tr>
<tr><td>gALL_v3_c14085</td><td>3</td><td>6</td><td>-</td><td>-</td><td>-</td><td>-</td><td>-</td></tr>
<tr><td>gALL_v3_c14086</td><td>4</td><td>4</td><td>-</td><td>-</td><td>-</td><td>-</td><td>-</td></tr>
<tr><td>gALL_v3_c14087</td><td>5</td><td>2</td><td>-</td><td>-</td><td>-</td><td>-</td><td>-</td></tr>
<tr><td>gALL_v3_c14088</td><td>5</td><td>5</td><td>-</td><td>-</td><td>-</td><td>-</td><td>-</td></tr>
<tr><td>gALL_v3_c14089</td><td>2</td><td>8</td><td>-</td><td>-</td><td>gb|ESO04366.1|</td><td>hypothetical protein HELRODRAFT_172731 [Helobdella robusta]</td><td>6,00E-08</td></tr>
<tr><td>gALL_v3_c1409</td><td>13</td><td>10</td><td>gnl|CDD|192296</td><td>pfam09440, eIF3_N, eIF3 subu</td><td>gb|ABM55547.1|</td><td>putative eukaryotic translation initiation factor 3 subunit 6 [Maconellicoccus hirsutus]</td><td>0</td></tr>
<tr><td>gALL_v3_c14092</td><td>4</td><td>6</td><td>-</td><td>-</td><td>-</td><td>-</td><td>-</td></tr>
<tr><td>gALL_v3_c14093</td><td>4</td><td>6</td><td>-</td><td>-</td><td>ref|XP_002428924.1|</td><td>conserved hypothetical protein [Pediculus humanus corporis] gb|EEB16186.1| conserved hypothetical protein [Pediculus humanus corporis]</td><td>1,00E-13</td></tr>
<tr><td>gALL_v3_c14094</td><td>1</td><td>5</td><td>-</td><td>-</td><td>-</td><td>-</td><td>-</td></tr>
<tr><td>gALL_v3_c14096</td><td>2</td><td>3</td><td>gnl|CDD|189977</td><td>pfam01412, ArfGap, Putative (</td><td>ref|XP_002426585.1|</td><td>ADP-ribosylation factor GTPase-activating protein, putative [Pediculus humanus corporis] gb|EEB13847.1| ADP-ribosylation factor GTPase-activating protein, putative [Ped</td><td>6,00E-76</td></tr>
<tr><td>gALL_v3_c14098</td><td>6</td><td>4</td><td>gnl|CDD|80321</td><td>cd04438, DEP_dishevelled, DE</td><td>ref|XP_006607146.1|</td><td>PREDICTED: segment polarity protein dishevelled homolog DVL-3-like isoform X2 [Apis dorsata]</td><td>8,00E-86</td></tr>
<tr><td>gALL_v3_c14099</td><td>3</td><td>2</td><td>-</td><td>-</td><td>-</td><td>-</td><td>-</td></tr>
<tr><td>gALL_v3_c1410</td><td>11</td><td>8</td><td>-</td><td>-</td><td>-</td><td>-</td><td>-</td></tr>
<tr><td>gALL_v3_c14100</td><td>4</td><td>2</td><td>-</td><td>-</td><td>-</td><td>-</td><td>-</td></tr>
<tr><td>gALL_v3_c14101</td><td>4</td><td>7</td><td>-</td><td>-</td><td>-</td><td>-</td><td>-</td></tr>
<tr><td>gALL_v3_c14102</td><td>2</td><td>3</td><td>gnl|CDD|192498</td><td>pfam10256, Erf4, Golgin subfa</td><td>ref|XP_002432167.1|</td><td>Cysteine-rich hydrophobic domain 2 protein, putative [Pediculus humanus corporis] gb|EEB19429.1| Cysteine-rich hydrophobic domain 2 protein, putative [Pediculus hum</td><td>3,00E-74</td></tr>
<tr><td>gALL_v3_c14104</td><td>2</td><td>2</td><td>-</td><td>-</td><td>-</td><td>-</td><td>-</td></tr>
<tr><td>gALL_v3_c14105</td><td>8</td><td>4</td><td>-</td><td>-</td><td>-</td><td>-</td><td>-</td></tr>
<tr><td>gALL_v3_c14106</td><td>1</td><td>6</td><td>-</td><td>-</td><td>gb|ETN67644.1|</td><td>hypothetical protein AND_000535 [Anopheles darlingi]</td><td>2,00E-08</td></tr>
<tr><td>gALL_v3_c14109</td><td>1</td><td>7</td><td>-</td><td>-</td><td>-</td><td>-</td><td>-</td></tr>
<tr><td>gALL_v3_c1411</td><td>7</td><td>7</td><td>-</td><td>-</td><td>-</td><td>-</td><td>-</td></tr>
<tr><td>gALL_v3_c14110</td><td>6</td><td>17</td><td>gnl|CDD|178204</td><td>PLN02594, PLN02594, phosph</td><td>gb|EHJ75514.1|</td><td>phosphatidate cytidylyltransferase [Danaus plexippus]</td><td>1,00E-150</td></tr>
<tr><td>gALL_v3_c14112</td><td>4</td><td>6</td><td>-</td><td>-</td><td>-</td><td>-</td><td>-</td></tr>
<tr><td>gALL_v3_c14114</td><td>4</td><td>4</td><td>-</td><td>-</td><td>-</td><td>-</td><td>-</td></tr>
<tr><td>gALL_v3_c14115</td><td>1</td><td>3</td><td>-</td><td>-</td><td>-</td><td>-</td><td>-</td></tr>
<tr><td>gALL_v3_c14116</td><td>3</td><td>6</td><td>-</td><td>-</td><td>gb|ACZ96370.1|</td><td>neuropeptide-like precursor 1 [Rhodnius prolixus]</td><td>4,00E-13</td></tr>
<tr><td>gALL_v3_c14117</td><td>4</td><td>3</td><td>gnl|CDD|189336</td><td>pfam00003, 7tm_3, 7 transme</td><td>emb|CDJ86727.1|</td><td>GPCR domain containing protein [Haemonchus contortus]</td><td>6,00E-08</td></tr>
<tr><td>gALL_v3_c14118</td><td>0</td><td>5</td><td>gnl|CDD|193174</td><td>pfam12698, ABC2_membrane</td><td>ref|XP_001947916.2|</td><td>PREDICTED: ATP-binding cassette sub-family A member 3-like [Acyrthosiphon pisum]</td><td>5,00E-26</td></tr>
<tr><td>gALL_v3_c14120</td><td>2</td><td>3</td><td>-</td><td>-</td><td>-</td><td>-</td><td>-</td></tr>
<tr><td>gALL_v3_c14121</td><td>1</td><td>5</td><td>-</td><td>-</td><td>-</td><td>-</td><td>-</td></tr>
<tr><td>gALL_v3_c14122</td><td>2</td><td>3</td><td>-</td><td>-</td><td>ref|XP_004518178.1|</td><td>PREDICTED: probable 39S ribosomal protein L45, mitochondrial-like [Ceratitis capitata]</td><td>3,00E-68</td></tr>
<tr><td>gALL_v3_c14123</td><td>1</td><td>5</td><td>-</td><td>-</td><td>-</td><td>-</td><td>-</td></tr>
<tr><td>gALL_v3_c14124</td><td>4</td><td>2</td><td>-</td><td>-</td><td>gb|EFN88245.1|</td><td>Vesicle transport through interaction with t-SNAREs-like protein 1B [Harpegnathos saltator]</td><td>4,00E-25</td></tr>
<tr><td>gALL_v3_c14125</td><td>6</td><td>4</td><td>-</td><td>-</td><td>gb|EFX77573.1|</td><td>hypothetical protein DAPPUDRAFT_54175 [Daphnia pulex]</td><td>5,00E-50</td></tr>
<tr><td>gALL_v3_c14126</td><td>2</td><td>3</td><td>-</td><td>-</td><td>-</td><td>-</td><td>-</td></tr>
<tr><td>gALL_v3_c14127</td><td>2</td><td>10</td><td>-</td><td>-</td><td>ref|XP_003706142.1|</td><td>PREDICTED: zinc finger CCHC domain-containing protein 8 homolog [Megachile rotundata]</td><td>3,00E-21</td></tr>
<tr><td>gALL_v3_c14128</td><td>4</td><td>2</td><td>-</td><td>-</td><td>-</td><td>-</td><td>-</td></tr>
<tr><td>gALL_v3_c14129</td><td>4</td><td>9</td><td>-</td><td>-</td><td>-</td><td>-</td><td>-</td></tr>
<tr><td>gALL_v3_c1413</td><td>10</td><td>9</td><td>gnl|CDD|128336</td><td>smart00020, Tryp_SPc, Trypsir</td><td>ref|XP_002431532.1|</td><td>low-density lipoprotein receptor, putative [Pediculus humanus corporis] gb|EEB18794.1| low-density lipoprotein receptor, putative [Pediculus humanus corporis]</td><td>1,00E-118</td></tr>
<tr><td>gALL_v3_c14130</td><td>4</td><td>6</td><td>-</td><td>-</td><td>-</td><td>-</td><td>-</td></tr>
<tr><td>gALL_v3_c14131</td><td>3</td><td>5</td><td>-</td><td>-</td><td>-</td><td>-</td><td>-</td></tr>
<tr><td>gALL_v3_c14133</td><td>4</td><td>2</td><td>-</td><td>-</td><td>-</td><td>-</td><td>-</td></tr>
<tr><td>gALL_v3_c14134</td><td>4</td><td>4</td><td>-</td><td>-</td><td>-</td><td>-</td><td>-</td></tr>
<tr><td>gALL_v3_c14135</td><td>2</td><td>9</td><td>-</td><td>-</td><td>-</td><td>-</td><td>-</td></tr>
<tr><td>gALL_v3_c14136</td><td>1</td><td>3</td><td>-</td><td>-</td><td>-</td><td>-</td><td>-</td></tr>
<tr><td>gALL_v3_c14137</td><td>2</td><td>4</td><td>gnl|CDD|128516</td><td>smart00220, S_TKc, Serine/Th</td><td>ref|XP_003435922.1|</td><td>AGAP001752-PB [Anopheles gambiae str. PEST] gb|EGK97491.1| AGAP001752-PB [Anopheles gambiae str. PEST]</td><td>6,00E-93</td></tr>
<tr><td>gALL_v3_c14139</td><td>0</td><td>4</td><td>-</td><td>-</td><td>-</td><td>-</td><td>-</td></tr>
<tr><td>gALL_v3_c1414</td><td>2</td><td>10</td><td>gnl|CDD|146346</td><td>pfam03661, UPF0121, Unchar</td><td>ref|XP_970657.1|</td><td>PREDICTED: similar to conserved hypothetical protein [Tribolium castaneum] gb|EFA01643.1| hypothetical protein TcasGA2_TC007213 [Tribolium castaneum]</td><td>2,00E-57</td></tr>
<tr><td>gALL_v3_c14140</td><td>0</td><td>4</td><td>-</td><td>-</td><td>-</td><td>-</td><td>-</td></tr>
<tr><td>gALL_v3_c14141</td><td>3</td><td>1</td><td>-</td><td>-</td><td>-</td><td>-</td><td>-</td></tr>
<tr><td>gALL_v3_c14142</td><td>4</td><td>3</td><td>gnl|CDD|161947</td><td>TIGR00596, rad1, DNA repair p</td><td>ref|XP_003705895.1|</td><td>PREDICTED: DNA repair endonuclease XPF-like [Megachile rotundata]</td><td>6,00E-15</td></tr>
<tr><td>gALL_v3_c14143</td><td>5</td><td>2</td><td>-</td><td>-</td><td>-</td><td>-</td><td>-</td></tr>
<tr><td>gALL_v3_c14144</td><td>7</td><td>4</td><td>gnl|CDD|162107</td><td>TIGR00918, 2A060602, The Eu</td><td>ref|XP_002428547.1|</td><td>patched 1, putative [Pediculus humanus corporis] gb|EEB15809.1| patched 1, putative [Pediculus humanus corporis]</td><td>1,00E-27</td></tr>
<tr><td>gALL_v3_c14145</td><td>2</td><td>3</td><td>-</td><td>-</td><td>-</td><td>-</td><td>-</td></tr>
<tr><td>gALL_v3_c14146</td><td>2</td><td>3</td><td>-</td><td>-</td><td>-</td><td>-</td><td>-</td></tr>
<tr><td>gALL_v3_c14147</td><td>5</td><td>4</td><td>gnl|CDD|48539</td><td>cd02990, UAS_FAF1, UAS fami</td><td>dbj|BAM63554.1|</td><td>FAS-associated factor 1 [Locusta migratoria manilensis]</td><td>6,00E-83</td></tr>
<tr><td>gALL_v3_c14148</td><td>3</td><td>4</td><td>gnl|CDD|152412</td><td>pfam11977, RNase_Zc3h12a, Z</td><td>gb|EGI63362.1|</td><td>Putative ribonuclease ZC3H12C [Acromyrmex echinatior]</td><td>2,00E-69</td></tr>
<tr><td>gALL_v3_c14149</td><td>8</td><td>15</td><td>-</td><td>-</td><td>-</td><td>-</td><td>-</td></tr>
<tr><td>gALL_v3_c1415</td><td>8</td><td>6</td><td>-</td><td>-</td><td>ref|XP_976409.1|</td><td>PREDICTED: similar to conserved hypothetical protein [Tribolium castaneum] gb|EFA10122.1| hypothetical protein TcasGA2_TC012302 [Tribolium castaneum]</td><td>1,00E-20</td></tr>
<tr><td>gALL_v3_c14150</td><td>0</td><td>5</td><td>-</td><td>-</td><td>-</td><td>-</td><td>-</td></tr>
<tr><td>gALL_v3_c14152</td><td>5</td><td>2</td><td>gnl|CDD|100101</td><td>cd00349, Ribosomal_L11, Ribc</td><td>gb|AGM32492.1|</td><td>39S ribosomal protein L11-like protein [Coptotermes formosanus]</td><td>1,00E-123</td></tr>
<tr><td>gALL_v3_c14153</td><td>3</td><td>2</td><td>-</td><td>-</td><td>-</td><td>-</td><td>-</td></tr>
<tr><td>gALL_v3_c14154</td><td>1</td><td>4</td><td>-</td><td>-</td><td>-</td><td>-</td><td>-</td></tr>
<tr><td>gALL_v3_c14156</td><td>4</td><td>2</td><td>-</td><td>-</td><td>ref|XP_001600116.2|</td><td>PREDICTED: hypothetical protein LOC100115379 [Nasonia vitripennis]</td><td>1,00E-32</td></tr>
<tr><td>gALL_v3_c14157</td><td>3</td><td>2</td><td>gnl|CDD|30292</td><td>cd02248, Peptidase_C1A, Pept</td><td>ref|XP_003701604.1|</td><td>PREDICTED: cathepsin O-like [Megachile rotundata]</td><td>9,00E-40</td></tr>
<tr><td>gALL_v3_c14158</td><td>4</td><td>4</td><td>-</td><td>-</td><td>-</td><td>-</td><td>-</td></tr>
<tr><td>gALL_v3_c14159</td><td>1</td><td>8</td><td>-</td><td>-</td><td>gb|EFZ10112.1|</td><td>hypothetical protein SINV_80283 [Solenopsis invicta]</td><td>2,00E-86</td></tr>
<tr><td>gALL_v3_c1416</td><td>4</td><td>8</td><td>-</td><td>-</td><td>-</td><td>-</td><td>-</td></tr>
</table>

gALL_v3_c14160	3	4	-	-	-	-	-
gALL_v3_c14162	5	5	-	-	-	-	-
gALL_v3_c14163	4	10	-	-	-	-	-
gALL_v3_c14164	2	3	-	-	ref|XP_003486046.1|	PREDICTED: 1-phosphatidylinositol-4,5-bisphosphate phosphodiesterase gamma-1-like [Bombus impatiens]	9,00E-64
gALL_v3_c14165	3	1	-	-	-	-	-
gALL_v3_c14166	4	4	-	-	-	-	-
gALL_v3_c14167	7	5	-	-	-	-	-
gALL_v3_c14168	3	2	-	-	-	-	-
gALL_v3_c14169	3	6	-	-	-	-	-
gALL_v3_c1417	4	13	-	-	-	-	-
gALL_v3_c14170	3	3	-	-	-	-	-
gALL_v3_c14171	3	6	-	-	-	-	-
gALL_v3_c14172	5	4	-	-	ref|XP_002428703.1|	Procollagen-lysine,2-oxoglutarate 5-dioxygenase 3 precursor, putative [Pediculus humanus corporis] gb|EEB15965.1| Procollagen-lysine,2-oxoglutarate 5-dioxygenase 3 pre	1,00E-137
gALL_v3_c14173	6	7	-	-	-	-	-
gALL_v3_c14174	3	2	-	-	-	-	-
gALL_v3_c14176	2	7	gnl|CDD|191059	pfam04678, DUF607, Protein c	ref|XP_004931989.1|	PREDICTED: calcium uniporter protein, mitochondrial-like [Bombyx mori]	2,00E-54
gALL_v3_c14177	5	6	gnl|CDD|117221	pfam08648, DUF1777, Protein	-	-	-
gALL_v3_c14178	3	4	-	-	ref|XP_002424222.1|	orthopedia homeobox protein, putative [Pediculus humanus corporis] gb|EEB11484.1| orthopedia homeobox protein, putative [Pediculus humanus corporis]	1,00E-26
gALL_v3_c14179	3	3	gnl|CDD|72875	cd04513, Glycosylasparaginase	ref|NP_001037686.1|	aspartylglucosaminidase [Bombyx mori] gb|ABG81362.1| aspartylglucosaminidase [Bombyx mori]	1,00E-13
gALL_v3_c1418	11	13	-	-	-	-	-
gALL_v3_c14180	3	5	-	-	-	-	-
gALL_v3_c14181	2	4	-	-	-	-	-
gALL_v3_c14182	4	2	-	-	-	-	-
gALL_v3_c14184	4	4	-	-	-	-	-
gALL_v3_c14185	2	4	-	-	-	-	-
gALL_v3_c14186	4	3	-	-	-	-	-
gALL_v3_c14187	2	2	-	-	ref|XP_972312.1|	PREDICTED: similar to toll [Tribolium castaneum] gb|EEZ99355.1| toll-7-like protein [Tribolium castaneum]	3,00E-24
gALL_v3_c14188	3	2	-	-	-	-	-
gALL_v3_c14189	3	6	-	-	-	-	-
gALL_v3_c1419	15	16	gnl|CDD|173310	PTZ00005, PTZ00005, phospho	gb|EFN66567.1|	Phosphoglycerate kinase [Camponotus floridanus]	1,00E-161
gALL_v3_c14190	1	5	-	-	-	-	-
gALL_v3_c14192	1	4	-	-	-	-	-
gALL_v3_c14193	3	11	-	-	ref|XP_004587838.1|	PREDICTED: fap1 adhesin-like [Ochotona princeps]	8,00E-09
gALL_v3_c14194	8	8	gnl|CDD|191088	pfam04777, Evr1_Alr, Erv1 / A	ref|XP_003398016.1|	PREDICTED: LOW QUALITY PROTEIN: FAD-linked sulfhydryl oxidase ALR-like [Bombus terrestris]	2,00E-15
gALL_v3_c14195	4	10	gnl|CDD|163657	cd07414, MPP_PP1_PPKL, PP1	ref|XP_002736947.1|	PREDICTED: serine/threonine-protein phosphatase PP1-like [Saccoglossus kowalevskii]	6,00E-39
gALL_v3_c14196	3	2	-	-	-	-	-
gALL_v3_c14197	3	3	-	-	-	-	-
gALL_v3_c14198	3	2	gnl|CDD|188999	cd06461, M2_ACE, Peptidase	gb|EFA05307.1|	hypothetical protein TcasGA2_TC015465 [Tribolium castaneum]	4,00E-15
gALL_v3_c14199	1	3	-	-	-	-	-
gALL_v3_c142	4	10	-	-	-	-	-
gALL_v3_c1420	10	14	-	-	-	-	-
gALL_v3_c14200	1	8	-	-	-	-	-
gALL_v3_c14201	3	2	-	-	-	-	-
gALL_v3_c14202	0	5	-	-	-	-	-
gALL_v3_c14203	2	3	-	-	-	-	-
gALL_v3_c14205	4	5	-	-	ref|XP_003493149.1|	PREDICTED: hypothetical protein LOC100743398 [Bombus impatiens]	2,00E-09
gALL_v3_c14206	2	3	-	-	ref|XP_002431828.1|	Thyroid hormone receptor-associated protein complex 95 kDa component, putative [Pediculus humanus corporis] gb|EEB19090.1| Thyroid hormone receptor-associated p	2,00E-74
gALL_v3_c14207	5	2	-	-	-	-	-
gALL_v3_c14208	2	3	-	-	-	-	-
gALL_v3_c14209	3	2	-	-	ref|XP_002424437.1|	multidrug resistance-associated protein, putative [Pediculus humanus corporis] gb|EEB11699.1| multidrug resistance-associated protein, putative [Pediculus humanus cor	9,00E-34
gALL_v3_c1421	13	8	-	-	-	-	-
gALL_v3_c14211	4	2	-	-	-	-	-
gALL_v3_c14212	1	8	-	-	-	-	-
gALL_v3_c14213	7	5	gnl|CDD|34495	COG4886, COG4886, Leucine-r	ref|XP_006622315.1|	PREDICTED: leucine-rich repeat and immunoglobulin-like domain-containing nogo receptor-interacting protein 2-like isoform X1 [Apis dorsata]	1,00E-56
gALL_v3_c14214	8	6	-	-	-	-	-
gALL_v3_c14215	6	11	-	-	-	-	-
gALL_v3_c14216	7	5	gnl|CDD|147160	pfam04858, TH1, TH1 protein.	ref|XP_002431657.1|	Negative elongation factor D, putative [Pediculus humanus corporis] gb|EEB18919.1| Negative elongation factor D, putative [Pediculus humanus corporis]	0
gALL_v3_c14217	4	5	-	-	-	-	-
gALL_v3_c14219	1	3	-	-	-	-	-
gALL_v3_c1422	10	6	gnl|CDD|173703	cd05612, STKc_PRKX_like, Cat	ref|XP_973065.1|	PREDICTED: similar to camp-dependent protein kinase catalytic subunit [Tribolium castaneum] gb|EFA03994.1| hypothetical protein TcasGA2_TC014143 [Tribolium castane	1,00E-168
gALL_v3_c14220	1	6	-	-	gb|ESO05799.1|	hypothetical protein HELRODRAFT_171472 [Helobdella robusta]	2,00E-07
gALL_v3_c14221	9	11	-	-	-	-	-
gALL_v3_c14222	2	4	-	-	-	-	-
gALL_v3_c14223	2	4	-	-	gb|ESO09414.1|	hypothetical protein HELRODRAFT_168397 [Helobdella robusta]	1,00E-31
gALL_v3_c14224	6	7	gnl|CDD|191079	pfam04757, Pex2_Pex12, Pex2	gb|EFZ18073.1|	hypothetical protein SINV_02368 [Solenopsis invicta]	2,00E-28
gALL_v3_c14225	2	4	-	-	-	-	-
gALL_v3_c14227	3	4	-	-	-	-	-
gALL_v3_c14228	2	5	gnl|CDD|163658	cd07415, MPP_PP2A_PP4_PP	ref|XP_006624066.1|	PREDICTED: serine/threonine-protein phosphatase 4 catalytic subunit-like [Apis dorsata]	0
gALL_v3_c14229	3	4	-	-	ref|XP_002430529.1|	conserved hypothetical protein [Pediculus humanus corporis] gb|EEB17791.1| conserved hypothetical protein [Pediculus humanus corporis]	4,00E-51
gALL_v3_c1423	7	17	-	-	-	-	-
gALL_v3_c14232	4	2	-	-	-	-	-
gALL_v3_c14233	2	5	-	-	ref|XP_968472.1|	PREDICTED: similar to GA16025-PA [Tribolium castaneum] gb|EFA09595.1| hypothetical protein TcasGA2_TC011713 [Tribolium castaneum]	2,00E-23
gALL_v3_c14234	2	3	-	-	-	-	-
gALL_v3_c14235	3	2	gnl|CDD|187547	cd05236, FAR-N_SDR_e, fatty	gb|EFN84876.1|	Fatty acyl-CoA reductase 1 [Harpegnathos saltator]	2,00E-50
gALL_v3_c14236	1	4	-	-	gb|EFN83486.1|	Midasin [Harpegnathos saltator]	6,00E-20
gALL_v3_c14237	3	1	-	-	-	-	-
gALL_v3_c14238	3	4	gnl|CDD|140307	PTZ00284, PTZ00284, protein	ref|XP_003695325.1|	PREDICTED: serine/threonine-protein kinase PRP4 homolog [Apis florea]	2,00E-75
gALL_v3_c14239	2	4	-	-	-	-	-
gALL_v3_c1424	5	6	gnl|CDD|128516	smart00220, S_TKc, Serine/Th	gb|EFZ13258.1|	hypothetical protein SINV_10963 [Solenopsis invicta]	1,00E-166
gALL_v3_c14240	4	4	-	-	-	-	-
gALL_v3_c14241	2	3	-	-	-	-	-
gALL_v3_c14242	3	1	-	-	-	-	-
gALL_v3_c14243	3	2	-	-	-	-	-
gALL_v3_c14244	6	13	-	-	-	-	-
gALL_v3_c14245	2	4	-	-	-	-	-
gALL_v3_c14246	2	4	-	-	-	-	-
gALL_v3_c14249	5	3	-	-	-	-	-
gALL_v3_c1425	4	6	-	-	ref|XP_001663043.1|	hypothetical protein AaeL_AAEL012905 [Aedes aegypti] gb|EAT34892.1| AAEL012905-PA [Aedes aegypti]	1,00E-113
gALL_v3_c14250	3	5	-	-	gb|EGI68289.1|	AP2-associated protein kinase 1 [Acromyrmex echinatior]	3,00E-48
gALL_v3_c14251	3	2	-	-	-	-	-
gALL_v3_c14252	4	2	-	-	-	-	-
gALL_v3_c14253	9	7	gnl|CDD|109126	pfam00057, Ldl_recept_a, Low	ref|XP_003493352.1|	PREDICTED: low-density lipoprotein receptor-related protein 6-like [Bombus impatiens]	1,00E-103
gALL_v3_c14254	4	5	-	-	ref|XP_001647844.1|	hypothetical protein AaeL_AAEL000070 [Aedes aegypti] gb|EAT48901.1| AAEL000070-PA [Aedes aegypti]	1,00E-107
gALL_v3_c14255	5	2	-	-	-	-	-
gALL_v3_c14256	3	6	gnl|CDD|29151	cd00189, TPR, Tetratricopeptic	ref|XP_396199.4|	PREDICTED: calcineurin-binding protein cabin-1-like isoform X2 [Apis mellifera] ref|XP_006564669.1| PREDICTED: calcineurin-binding protein cabin-1-like isoform X1 [Apis	4,00E-44
gALL_v3_c14257	3	3	gnl|CDD|188189	TIGR01983, UbiG, ubiquinone	ref|XP_395548.2|	PREDICTED: hexaprenyldihydroxybenzoate methyltransferase, mitochondrial-like [Apis mellifera]	1,00E-35
gALL_v3_c14258	3	4	-	-	-	-	-
gALL_v3_c1426	22	36	-	-	-	-	-
gALL_v3_c14260	5	4	gnl|CDD|112805	pfam04006, Mpp10, Mpp10 p	ref|XP_003703537.1|	PREDICTED: U3 small nucleolar ribonucleoprotein protein MPP10-like isoform 2 [Megachile rotundata]	1,00E-27
gALL_v3_c14261	4	2	-	-	-	-	-
gALL_v3_c14262	5	2	gnl|CDD|30093	cd01371, KISc_KIF3, Kinesin m	gb|AAI05210.1|	KIF3A protein [Bos taurus]	1,00E-106
gALL_v3_c14263	4	5	-	-	ref|XP_005465079.1|	PREDICTED: tRNA wybutosine-synthesizing protein 1 homolog isoform X2 [Oreochromis niloticus]	1,00E-25
gALL_v3_c14264	1	4	-	-	-	-	-
gALL_v3_c14265	4	6	gnl|CDD|189641	pfam00628, PHD, PHD-finger.	gb|EFN66778.1|	Supporter of activation of yellow protein [Camponotus floridanus]	8,00E-82
gALL_v3_c14266	2	6	-	-	-	-	-
gALL_v3_c14267	1	4	-	-	-	-	-
gALL_v3_c14268	4	11	-	-	-	-	-
gALL_v3_c1427	7	7	-	-	-	-	-
gALL_v3_c14271	2	7	-	-	-	-	-
gALL_v3_c14272	4	2	-	-	-	-	-
gALL_v3_c14273	4	5	-	-	-	-	-
gALL_v3_c14274	2	4	-	-	-	-	-
gALL_v3_c14275	3	6	-	-	ref|XP_006564181.1|	PREDICTED: uncharacterized threonine-rich GPI-anchored glycoprotein PJ4664.02-like isoform X3 [Apis mellifera]	9,00E-07
gALL_v3_c14276	2	4	-	-	-	-	-
gALL_v3_c14277	4	4	-	-	-	-	-
gALL_v3_c1428	18	27	-	-	gb|AGM32320.1|	serine protease, partial [Coptotermes formosanus]	4,00E-10
gALL_v3_c14280	1	5	-	-	-	-	-
gALL_v3_c14281	1	4	-	-	-	-	-
gALL_v3_c14283	3	5	-	-	-	-	-
gALL_v3_c14284	2	3	-	-	-	-	-
gALL_v3_c14285	6	4	gnl|CDD|193503	cd03882, M28_nicalin_like, M	ref|XP_971811.1|	PREDICTED: similar to MGC79091 protein [Tribolium castaneum] gb|EFA05879.1| hypothetical protein TcasGA2_TC008691 [Tribolium castaneum]	1,00E-155
gALL_v3_c14286	3	4	-	-	-	-	-
gALL_v3_c14287	2	4	gnl|CDD|100104	cd00590, RRM, RRM (RNA rec	gb|ETN63524.1|	hypothetical protein AND_004765 [Anopheles darlingi]	4,00E-38
gALL_v3_c14288	16	29	-	-	ref|XP_006570939.1|	PREDICTED: mediator of RNA polymerase II transcription subunit 12 [Apis mellifera]	0
gALL_v3_c14289	2	3	-	-	-	-	-
gALL_v3_c1429	3	5	-	-	-	-	-
gALL_v3_c14290	6	4	gnl|CDD|185558	PTZ00329, PTZ00329, eukaryo	gb|AGM32664.1|	eukaryotic translation initiation factor 1A [Coptotermes formosanus]	3,00E-73
gALL_v3_c14291	3	1	-	-	-	-	-
gALL_v3_c14292	1	4	-	-	ref|XP_966466.1|	PREDICTED: similar to CG9164 CG9164-PA [Tribolium castaneum]	1,00E-08
gALL_v3_c14295	2	3	-	-	-	-	-
gALL_v3_c14296	4	8	-	-	ref|XP_006029730.1|	PREDICTED: histone-lysine N-methyltransferase SETMAR-like [Alligator sinensis]	6,00E-33
gALL_v3_c14297	7	10	-	-	-	-	-
gALL_v3_c14298	4	9	-	-	-	-	-
gALL_v3_c14299	2	6	-	-	-	-	-
gALL_v3_c143	10	9	-	-	-	-	-
gALL_v3_c14300	2	4	-	-	-	-	-
gALL_v3_c14301	4	5	-	-	-	-	-
gALL_v3_c14302	2	5	-	-	-	-	-
gALL_v3_c14303	1	5	-	-	-	-	-
gALL_v3_c14305	2	3	-	-	-	-	-
gALL_v3_c14306	1	4	-	-	-	-	-
gALL_v3_c14307	6	5	-	-	-	-	-
gALL_v3_c14308	1	4	-	-	-	-	-
gALL_v3_c14310	5	3	gnl|CDD|29257	cd00200, WD40, WD40 domai	ref|XP_002427825.1|	Nuclear distribution protein nudF, putative [Pediculus humanus corporis] gb|EEB15087.1| Nuclear distribution protein nudF, putative [Pediculus humanus corporis]	1,00E-150

gALL_v3_c14311	1	5	-	-	ref|XP_003704904.1|	PREDICTED: uncharacterized protein LOC100876769 [Megachile rotundata]	1,00E-40
gALL_v3_c14312	0	4	-	-	-	-	-
gALL_v3_c14314	1	4	-	-	ref|XP_001651505.1|	hypothetical protein AaeL_AAEL005848 [Aedes aegypti] gb|EAT42622.1| AAEL005848-PA [Aedes aegypti]	4,00E-06
gALL_v3_c14316	4	4	-	-	-	-	-
gALL_v3_c14317	3	2	gnl|CDD|189538	pfam00415, RCC1, Regulator c	ref|XP_003486520.1|	PREDICTED: hypothetical protein LOC100745121 [Bombus impatiens]	2,00E-31
gALL_v3_c14318	0	5	-	-	-	-	-
gALL_v3_c14319	9	11	-	-	-	-	-
gALL_v3_c1432	15	24	-	-	gb|EGI70456.1|	Uncharacterized protein [Acromyrmex echinatior]	2,00E-79
gALL_v3_c14320	7	4	-	-	gb|EFA03894.1|	hypothetical protein TcasGA2_TC014020 [Tribolium castaneum]	2,00E-46
gALL_v3_c14322	0	5	-	-	gb|EEZ97294.1|	hypothetical protein TcasGA2_TC011099 [Tribolium castaneum]	1,00E-44
gALL_v3_c14323	3	2	-	-	-	-	-
gALL_v3_c14325	1	8	-	-	-	-	-
gALL_v3_c14326	4	8	-	-	ref|NP_001002427.1|	transcription and mRNA export factor ENY2 [Danio rerio] sp|Q6DH42.1|ENY2_DANRE RecName: Full=Transcription and mRNA export factor ENY2; AltName: Full=Enhancer	4,00E-09
gALL_v3_c14327	3	3	-	-	-	-	-
gALL_v3_c14328	8	4	-	-	-	-	-
gALL_v3_c14329	0	5	-	-	-	-	-
gALL_v3_c1433	5	3	-	-	-	-	-
gALL_v3_c14330	1	6	-	-	-	-	-
gALL_v3_c14331	4	9	-	-	-	-	-
gALL_v3_c14332	5	8	-	-	-	-	-
gALL_v3_c14333	9	8	-	-	ref|XP_006567452.1|	PREDICTED: sortilin-related receptor [Apis mellifera]	1,00E-53
gALL_v3_c14334	2	4	-	-	-	-	-
gALL_v3_c14335	3	4	-	-	-	-	-
gALL_v3_c14336	2	3	gnl|CDD|189527	pfam00387, PI-PLC-Y, Phospha	gb|AGZ15171.1|	phosphoesterase 16, partial [Laodelphax striatella]	7,00E-69
gALL_v3_c14338	2	6	gnl|CDD|176317	cd01241, PH_Akt, Akt pleckstr	ref|XP_001605990.1|	PREDICTED: RAC serine/threonine-protein kinase-like isoform 1 [Nasonia vitripennis] ref|XP_003423929.1| PREDICTED: RAC serine/threonine-protein kinase-like isoform 2	1,00E-35
gALL_v3_c14339	6	3	-	-	-	-	-
gALL_v3_c1434	4	7	gnl|CDD|180658	PRK06688, PRK06688, enoyl-C	gb|EFN80284.1|	Peroxisomal 3,2-trans-enoyl-CoA isomerase [Harpegnathos saltator]	2,00E-50
gALL_v3_c14340	3	6	-	-	-	-	-
gALL_v3_c14341	0	4	-	-	-	-	-
gALL_v3_c14342	2	6	-	-	-	-	-
gALL_v3_c14343	5	7	gnl|CDD|191574	pfam06650, DUF1162, Protein	ref|XP_002424403.1|	conserved hypothetical protein [Pediculus humanus corporis] gb|EEB11665.1| conserved hypothetical protein [Pediculus humanus corporis]	1,00E-169
gALL_v3_c14344	3	5	-	-	-	-	-
gALL_v3_c14345	1	4	-	-	-	-	-
gALL_v3_c14346	3	2	-	-	-	-	-
gALL_v3_c14347	6	7	-	-	gb|ADX60046.1|	R2 protein [Reticulitermes grassei]	7,00E-09
gALL_v3_c14348	3	3	-	-	-	-	-
gALL_v3_c14349	3	2	-	-	-	-	-
gALL_v3_c1435	4	7	-	-	-	-	-
gALL_v3_c14350	3	4	-	-	-	-	-
gALL_v3_c14352	9	20	-	-	-	-	-
gALL_v3_c14354	3	6	gnl|CDD|149948	pfam09072, TMA7, Translatior	ref|XP_004933296.1|	PREDICTED: translation machinery-associated protein 7 homolog [Bombyx mori]	5,00E-12
gALL_v3_c14355	4	3	-	-	-	-	-
gALL_v3_c14356	5	6	-	-	-	-	-
gALL_v3_c14358	0	4	-	-	-	-	-
gALL_v3_c14359	2	3	-	-	-	-	-
gALL_v3_c1436	35	35	-	-	-	-	-
gALL_v3_c14360	2	7	-	-	-	-	-
gALL_v3_c14361	6	13	-	-	ref|NP_001095946.1|	chitin deacetylase 1 precursor [Tribolium castaneum] gb|ABU25223.1| chitin deacetylase 1 [Tribolium castaneum] gb|EFA03956.1| hypothetical protein TcasGA2_TC0141C	1,00E-115
gALL_v3_c14362	2	7	-	-	ref|XP_003706159.1|	PREDICTED: TBC1 domain family member 15-like [Megachile rotundata]	8,00E-07
gALL_v3_c14363	4	6	-	-	gb|EFN64029.1|	Titin [Camponotus floridanus]	1,00E-21
gALL_v3_c14364	0	6	-	-	-	-	-
gALL_v3_c14366	5	2	-	-	ref|XP_002423748.1|	hypothetical protein Phum_PHUM083230 [Pediculus humanus corporis] gb|EEB11010.1| hypothetical protein Phum_PHUM083230 [Pediculus humanus corporis]	1,00E-14
gALL_v3_c14367	0	5	-	-	-	-	-
gALL_v3_c14369	4	2	-	-	-	-	-
gALL_v3_c14370	3	4	-	-	gb|EGI69566.1|	Protein FAM109A [Acromyrmex echinatior]	1,00E-16
gALL_v3_c14371	8	10	-	-	-	-	-
gALL_v3_c14372	6	16	-	-	ref|XP_003697132.1|	PREDICTED: uncharacterized protein LOC100866704 [Apis florea]	2,00E-72
gALL_v3_c14373	3	6	-	-	-	-	-
gALL_v3_c14374	0	6	-	-	-	-	-
gALL_v3_c14375	4	6	-	-	gb|EGI61188.1|	Transposable element Tc3 transposase [Acromyrmex echinatior]	9,00E-14
gALL_v3_c14376	2	4	-	-	-	-	-
gALL_v3_c14377	8	4	gnl|CDD|128336	smart00020, Tryp_SPc, Trypsir	gb|EFN82690.1|	Proclotting enzyme [Harpegnathos saltator]	4,00E-39
gALL_v3_c14378	1	5	-	-	-	-	-
gALL_v3_c14379	2	2	-	-	ref|XP_003396927.1|	PREDICTED: cleavage and polyadenylation specificity factor subunit 5-like isoform 1 [Bombus terrestris] ref|XP_003396928.1| PREDICTED: cleavage and polyadenylation sp	4,00E-69
gALL_v3_c1438	7	11	gnl|CDD|133007	cd02514, GT13_GLCNAC-TI, G'	gb|EFZ15620.1|	hypothetical protein SINV_06379 [Solenopsis invicta]	3,00E-95
gALL_v3_c14380	5	3	-	-	-	-	-
gALL_v3_c14381	2	3	-	-	-	-	-
gALL_v3_c14382	0	5	-	-	-	-	-
gALL_v3_c14383	9	7	-	-	-	-	-
gALL_v3_c14384	2	5	-	-	-	-	-
gALL_v3_c14386	5	4	-	-	gb|ADI61823.1|	endonuclease-reverse transcriptase [Bombyx mori]	2,00E-33
gALL_v3_c14387	11	14	gnl|CDD|30776	COG0427, ACH1, Acetyl-CoA h	ref|XP_969571.1|	PREDICTED: similar to AGAP004396-PA [Tribolium castaneum]	1,00E-81
gALL_v3_c14388	5	2	-	-	gb|EGI68912.1|	Mitogen-activated protein kinase-binding protein 1 [Acromyrmex echinatior]	1,00E-47
gALL_v3_c14391	2	4	gnl|CDD|190402	pfam02731, SKIP_SNW, SKIP/S	ref|XP_002424133.1|	Puff-specific protein Bx42, putative [Pediculus humanus corporis] gb|EEB11395.1| Puff-specific protein Bx42, putative [Pediculus humanus corporis]	1,00E-134
gALL_v3_c14392	3	1	-	-	-	-	-
gALL_v3_c14393	2	4	-	-	-	-	-
gALL_v3_c14394	3	4	-	-	gb|ACT53737.1|	feruloyl esterase-like protein Est2 [Reticulitermes flavipes]	5,00E-27
gALL_v3_c14395	2	3	-	-	-	-	-
gALL_v3_c14396	5	4	-	-	ref|XP_001842767.1|	ion channel nompc [Culex quinquefasciatus] gb|EDS28132.1| ion channel nompc [Culex quinquefasciatus]	3,00E-13
gALL_v3_c14397	1	4	-	-	gb|EFN76799.1|	Patched domain-containing protein 3 [Harpegnathos saltator]	2,00E-56
gALL_v3_c14398	4	10	-	-	-	-	-
gALL_v3_c14399	4	2	-	-	-	-	-
gALL_v3_c144	5	5	-	-	-	-	-
gALL_v3_c14400	8	8	-	-	-	-	-
gALL_v3_c14401	6	5	-	-	-	-	-
gALL_v3_c14402	0	5	-	-	-	-	-
gALL_v3_c14403	0	5	-	-	-	-	-
gALL_v3_c14404	0	5	-	-	-	-	-
gALL_v3_c14405	6	3	-	-	-	-	-
gALL_v3_c14406	4	10	-	-	-	-	-
gALL_v3_c14407	2	4	-	-	-	-	-
gALL_v3_c14408	5	5	-	-	ref|XP_002426847.1|	sphingosine kinase A, B, putative [Pediculus humanus corporis] gb|EEB14109.1| sphingosine kinase A, B, putative [Pediculus humanus corporis]	9,00E-42
gALL_v3_c14409	2	4	gnl|CDD|73151	cd01644, RT_pepA17, RT_pep	ref|XP_003241778.1|	PREDICTED: hypothetical protein LOC100573291 [Acyrthosiphon pisum]	5,00E-28
gALL_v3_c1441	6	6	-	-	-	-	-
gALL_v3_c14410	3	2	-	-	dbj|BAM20535.1|	conserved hypothetical protein, partial [Papilio polytes]	1,00E-94
gALL_v3_c14411	3	5	-	-	gb|EFA03113.1|	hypothetical protein TcasGA2_TC013023 [Tribolium castaneum]	7,00E-81
gALL_v3_c14412	3	6	-	-	-	-	-
gALL_v3_c14413	7	8	gnl|CDD|145278	pfam02010, REJ, REJ domain.	ref|XP_003485341.1|	PREDICTED: dyslexia-associated protein KIAA0319-like protein-like [Bombus impatiens]	0
gALL_v3_c14415	3	2	-	-	-	-	-
gALL_v3_c14416	3	2	-	-	-	-	-
gALL_v3_c14417	3	8	gnl|CDD|188297	TIGR03182, PDH_E1_alph_y, p	ref|XP_974523.1|	PREDICTED: similar to pyruvate dehydrogenase [Tribolium castaneum] gb|EFA00132.1| hypothetical protein TcasGA2_TC002948 [Tribolium castaneum]	1,00E-142
gALL_v3_c14418	2	6	gnl|CDD|153327	cd07643, I-BAR_IMD_MIM, In	gb|EFA06149.1|	hypothetical protein TcasGA2_TC008993 [Tribolium castaneum]	3,00E-43
gALL_v3_c14419	6	3	gnl|CDD|189631	pfam00617, RasGEF, RasGEF d	ref|XP_002432077.1|	conserved hypothetical protein [Pediculus humanus corporis] gb|EEB19339.1| conserved hypothetical protein [Pediculus humanus corporis]	0
gALL_v3_c1442	32	21	gnl|CDD|189943	pfam01326, PPDK_N, Pyruvate	gb|EFX82660.1|	hypothetical protein DAPPUDRAFT_316481 [Daphnia pulex]	1,00E-164
gALL_v3_c14420	4	6	-	-	-	-	-
gALL_v3_c14421	5	13	-	-	ref|XP_971793.1|	PREDICTED: similar to zormin CG33484-PD [Tribolium castaneum]	3,00E-39
gALL_v3_c14422	3	3	-	-	ref|XP_006614464.1|	PREDICTED: calpain-B-like isoform X3 [Apis dorsata]	1,00E-19
gALL_v3_c14423	3	4	-	-	ref|XP_003703972.1|	PREDICTED: gametogenetin-binding protein 2-like [Megachile rotundata]	1,00E-108
gALL_v3_c14424	5	4	gnl|CDD|177180	MTH00119, CYTB, cytochrome	gb|AFU51214.1|	cytochrome b, partial (mitochondrion) [Macrotermes subhyalinus] gb|AFU51216.1| cytochrome b, partial (mitochondrion) [Macrotermes subhyalinus] gb|AFU51217.1| cy	6,00E-06
gALL_v3_c14425	5	3	gnl|CDD|176329	cd01253, PH_beta_spectrin, B	gb|EGI61705.1|	Rho GTPase-activating protein 21 [Acromyrmex echinatior]	2,00E-41
gALL_v3_c14426	5	6	gnl|CDD|118200	pfam09668, Asp_protease, As	ref|XP_969775.2|	PREDICTED: similar to DNA-damage inducible protein [Tribolium castaneum]	1,00E-80
gALL_v3_c14427	3	2	-	-	-	-	-
gALL_v3_c14428	1	3	-	-	-	-	-
gALL_v3_c14429	6	3	gnl|CDD|165506	PHA03247, PHA03247, large t	-	-	-
gALL_v3_c1443	9	12	-	-	ref|XP_001603856.2|	PREDICTED: hypothetical protein LOC100120196 [Nasonia vitripennis]	3,00E-19
gALL_v3_c14430	0	2	-	-	ref|XP_004077729.1|	PREDICTED: inhibitor of Bruton tyrosine kinase-like [Oryzias latipes]	2,00E-14
gALL_v3_c14431	5	3	-	-	-	-	-
gALL_v3_c14432	0	4	-	-	-	-	-
gALL_v3_c14435	10	15	gnl|CDD|30091	cd01369, KISc_KHC_KIF5, Kine	pdb|2Y5W|A	Chain A, Crystal Structure Of Drosophila Melanogaster Kinesin-1 Motor Domain Dimer pdb|2Y5W|B Chain B, Crystal Structure Of Drosophila Melanogaster Kinesin-1 Moto	0
gALL_v3_c14436	5	4	gnl|CDD|189411	pfam00135, COesterase, Carb	gb|AGT57960.1|	juvenile hormone esterase, partial [Bemisia tabaci]	5,00E-62
gALL_v3_c14437	1	4	-	-	-	-	-
gALL_v3_c14439	3	3	-	-	ref|XP_003132829.1|	PREDICTED: receptor-interacting serine/threonine-protein kinase 4 [Sus scrofa]	9,00E-06
gALL_v3_c1444	28	46	-	-	-	-	-
gALL_v3_c14440	3	12	-	-	-	-	-
gALL_v3_c14442	3	3	gnl|CDD|191030	pfam04568, IATP, Mitochondri	-	-	-
gALL_v3_c14443	2	2	gnl|CDD|144003	pfam00254, FKBP_C, FKBP-typ	ref|XP_006621340.1|	PREDICTED: 12 kDa FK506-binding protein-like [Apis dorsata]	4,00E-69
gALL_v3_c14444	3	2	-	-	-	-	-
gALL_v3_c14445	5	3	-	-	-	-	-
gALL_v3_c14446	2	3	-	-	-	-	-
gALL_v3_c14447	2	2	-	-	ref|XP_006607167.1|	PREDICTED: diacylglycerol O-acyltransferase 1-like, partial [Apis dorsata]	4,00E-38
gALL_v3_c14448	2	4	-	-	-	-	-
gALL_v3_c14449	1	3	-	-	gb|EFN76067.1|	BCoR-like protein 1 [Harpegnathos saltator]	8,00E-22
gALL_v3_c1445	13	10	gnl|CDD|30104	cd01382, MYSc_type_VI, Myo	ref|XP_002429594.1|	myosin heavy chain 95F, putative [Pediculus humanus corporis] gb|EEB16856.1| myosin heavy chain 95F, putative [Pediculus humanus corporis]	0
gALL_v3_c14450	13	9	gnl|CDD|173464	PTZ00180, PTZ00180, 60S ribo	ref|XP_393671.2|	PREDICTED: 60S ribosomal protein L8 [Apis mellifera] ref|XP_003692736.1| PREDICTED: 60S ribosomal protein L8-like [Apis florea] ref|XP_006607973.1| PREDICTED: 60S r	2,00E-79
gALL_v3_c14451	5	3	gnl|CDD|133031	cd04188, DPG_synthase, DPG	dbj|BAN20766.1|	dolichyl-phosphate beta-D-mannosyltransferase, putative [Riptortus pedestris]	5,00E-45
gALL_v3_c14452	2	4	-	-	-	-	-
gALL_v3_c14453	6	3	-	-	ref|XP_002430620.1|	leucine-rich repeat-containing protein, putative [Pediculus humanus corporis] gb|EEB17882.1| leucine-rich repeat-containing protein, putative [Pediculus humanus corpor	2,00E-68
gALL_v3_c14455	1	4	-	-	-	-	-
gALL_v3_c14456	3	2	-	-	-	-	-
gALL_v3_c14457	3	3	-	-	-	-	-
gALL_v3_c14458	5	6	-	-	-	-	-
gALL_v3_c14459	3	4	-	-	-	-	-
gALL_v3_c14460	5	2	-	-	emb|CAJ90621.1|	HR3 isoform A [Blattella germanica]	1,00E-85
gALL_v3_c14461	6	5	-	-	-	-	-

gALL_v3_c14462	1	4	-	-	ref|XP_003486431.1|	PREDICTED: hypothetical protein LOC100744200 [Bombus impatiens]	3,00E-27
gALL_v3_c14465	3	2	gnl|CDD|180891	PRK07219, PRK07219, DNA to	gb|EHJ67631.1|	putative prokaryotic DNA topoisomerase [Danaus plexippus]	1,00E-115
gALL_v3_c14466	3	3	gnl|CDD|34856	COG5259, RSC8, RSC chromati	ref|XP_001812334.1|	PREDICTED: similar to moira CG18740-PA [Tribolium castaneum] gb|EFA10166.1| hypothetical protein TcasGA2_TC012356 [Tribolium castaneum]	3,00E-74
gALL_v3_c14467	7	9	-	-	-	-	-
gALL_v3_c14468	1	3	-	-	-	-	-
gALL_v3_c14469	2	3	-	-	-	-	-
gALL_v3_c1447	13	18	gnl|CDD|150416	pfam09738, DUF2051, Double	ref|XP_971050.2|	PREDICTED: similar to AGAP000207-PA [Tribolium castaneum]	1,00E-103
gALL_v3_c14470	1	4	-	-	ref|XP_003427162.1|	PREDICTED: hypothetical protein LOC100122846 [Nasonia vitripennis]	1,00E-13
gALL_v3_c14471	6	5	-	-	-	-	-
gALL_v3_c14472	2	4	gnl|CDD|179137	PRK00823, phhB, pterin-4-alph	ref|XP_970604.1|	PREDICTED: similar to pterin-4a-carbinolamine dehydratase CG1963-PA [Tribolium castaneum] gb|EFA07130.1| hypothetical protein TcasGA2_TC010121 [Tribolium castane	7,00E-51
gALL_v3_c14473	0	6	-	-	-	-	-
gALL_v3_c14474	2	6	-	-	-	-	-
gALL_v3_c14475	3	4	-	-	gb|AGM32821.1|	hypothetical protein [Coptotermes formosanus]	3,00E-08
gALL_v3_c14476	3	1	-	-	ref|XP_003708269.1|	PREDICTED: uncharacterized protein LOC100877935 [Megachile rotundata]	3,00E-08
gALL_v3_c14477	0	5	gnl|CDD|162028	TIGR00763, lon, ATP-depender	ref|XP_002606365.1|	hypothetical protein BRAFLDRAFT_67608 [Branchiostoma floridae] gb|EEN62375.1| hypothetical protein BRAFLDRAFT_67608 [Branchiostoma floridae]	3,00E-13
gALL_v3_c14478	3	4	gnl|CDD|150690	pfam10044, Ret_tiss, Retinal t	gb|EHJ73915.1|	Myb-MuvB complex subunit Lin-52 [Danaus plexippus]	2,00E-38
gALL_v3_c14479	1	4	gnl|CDD|191936	pfam08066, PMC2NT, PMC2NT	ref|XP_005101906.1|	PREDICTED: exosome component 10-like [Aplysia californica]	2,00E-39
gALL_v3_c14480	3	1	-	-	-	-	-
gALL_v3_c14481	3	9	-	-	-	-	-
gALL_v3_c14483	7	6	-	-	-	-	-
gALL_v3_c14485	3	2	-	-	-	-	-
gALL_v3_c14486	4	14	-	-	-	-	-
gALL_v3_c14487	2	3	-	-	-	-	-
gALL_v3_c14488	2	3	-	-	-	-	-
gALL_v3_c14489	0	5	-	-	-	-	-
gALL_v3_c1449	4	9	-	-	gb|EFX88574.1|	hypothetical protein DAPPUDRAFT_191054 [Daphnia pulex]	2,00E-75
gALL_v3_c14490	2	2	-	-	emb|CAB39733.1|	protease, reverse transcriptase, ribonuclease H, integrase [Drosophila buzzatii]	1,00E-06
gALL_v3_c14491	3	4	-	-	-	-	-
gALL_v3_c14492	2	6	-	-	ref|XP_001870370.1|	defective proboscis extension response [Culex quinquefasciatus] gb|EDS33375.1| defective proboscis extension response [Culex quinquefasciatus]	4,00E-80
gALL_v3_c14493	3	2	-	-	-	-	-
gALL_v3_c14494	11	15	-	-	-	-	-
gALL_v3_c14495	4	7	gnl|CDD|143860	pfam00078, RVT_1, Reverse tr	gb|ADI61823.1|	endonuclease-reverse transcriptase [Bombyx mori]	3,00E-24
gALL_v3_c14496	5	4	-	-	-	-	-
gALL_v3_c14497	3	4	-	-	-	-	-
gALL_v3_c14499	1	8	-	-	-	-	-
gALL_v3_c145	15	22	-	-	-	-	-
gALL_v3_c14500	5	3	-	-	-	-	-
gALL_v3_c14501	1	5	-	-	gb|AGM32707.1|	hypothetical protein, partial [Coptotermes formosanus]	1,00E-09
gALL_v3_c14502	2	6	-	-	-	-	-
gALL_v3_c14503	2	4	-	-	ref|XP_003424927.1|	PREDICTED: sterol regulatory element-binding protein cleavage-activating protein-like [Nasonia vitripennis]	2,00E-64
gALL_v3_c14504	3	6	-	-	-	-	-
gALL_v3_c14505	4	2	-	-	-	-	-
gALL_v3_c14506	4	2	-	-	-	-	-
gALL_v3_c14507	3	2	-	-	-	-	-
gALL_v3_c14508	3	2	gnl|CDD|173845	cd01156, IVD, Isovaleryl-CoA c	gb|AGM32357.1|	isovaleryl-coA dehydrogenase, mitochondrial-like protein [Coptotermes formosanus]	7,00E-71
gALL_v3_c14509	1	5	-	-	gb|EEZ99764.1|	hypothetical protein TcasGA2_TC002537 [Tribolium castaneum]	2,00E-51
gALL_v3_c14510	5	3	-	-	-	-	-
gALL_v3_c14511	2	8	gnl|CDD|180422	PRK06142, PRK06142, enoyl-C	gb|AGM32657.1|	delta 3,5-delta 2,4-dienoyl-coA isomerase, partial [Coptotermes formosanus]	9,00E-65
gALL_v3_c14512	4	5	-	-	-	-	-
gALL_v3_c14513	4	3	-	-	-	-	-
gALL_v3_c14515	1	4	-	-	ref|XP_006560235.1|	PREDICTED: glutamate-gated chloride channel isoform X5 [Apis mellifera] ref|XP_006616225.1| PREDICTED: glutamate-gated chloride channel-like isoform X8 [Apis dorsata	4,00E-19
gALL_v3_c14516	2	3	-	-	-	-	-
gALL_v3_c14518	4	5	-	-	gb|EFA03529.1|	hypothetical protein TcasGA2_TC013532 [Tribolium castaneum]	4,00E-28
gALL_v3_c14519	4	3	-	-	-	-	-
gALL_v3_c1452	7	5	-	-	-	-	-
gALL_v3_c14520	2	3	-	-	-	-	-
gALL_v3_c14521	2	3	-	-	-	-	-
gALL_v3_c14522	3	5	-	-	-	-	-
gALL_v3_c14524	9	8	-	-	-	-	-
gALL_v3_c14525	9	8	gnl|CDD|180841	PRK07106, PRK07106, 5-aminc	ref|XP_001662952.1|	bifunctional purine biosynthesis protein [Aedes aegypti] gb|EAT34980.1| AAEL012825-PA [Aedes aegypti]	3,00E-92
gALL_v3_c14526	3	2	-	-	-	-	-
gALL_v3_c14527	2	7	-	-	-	-	-
gALL_v3_c14528	3	4	-	-	gb|ERL93940.1|	hypothetical protein D910_11226 [Dendroctonus ponderosae]	8,00E-15
gALL_v3_c14530	4	6	-	-	-	-	-
gALL_v3_c14531	4	2	-	-	-	-	-
gALL_v3_c14532	5	5	-	-	ref|XP_001812033.1|	PREDICTED: similar to ring finger protein 216 [Tribolium castaneum]	1,00E-61
gALL_v3_c14533	9	6	-	-	ref|XP_974477.1|	PREDICTED: similar to selenoprotein T [Tribolium castaneum] gb|EFA03473.1| hypothetical protein TcasGA2_TC013470 [Tribolium castaneum]	1,00E-47
gALL_v3_c14534	1	4	-	-	-	-	-
gALL_v3_c14535	3	5	-	-	ref|XP_006622340.1|	PREDICTED: uncharacterized protein LOC102678817 isoform X4 [Apis dorsata]	3,00E-42
gALL_v3_c14536	2	2	gnl|CDD|151101	pfam10561, UPF0565, Unchar	ref|XP_001657628.1|	hypothetical protein AaeL_AAEL006290 [Aedes aegypti] gb|EAT42132.1| AAEL006290-PA [Aedes aegypti]	2,00E-27
gALL_v3_c14537	1	4	-	-	ref|XP_006615447.1|	PREDICTED: PH-interacting protein-like [Apis dorsata]	4,00E-31
gALL_v3_c14538	4	3	-	-	-	-	-
gALL_v3_c14539	4	4	-	-	-	-	-
gALL_v3_c1454	11	26	-	-	gb|EGI64642.1|	hypothetical protein G5I_06831 [Acromyrmex echinatior]	2,00E-12
gALL_v3_c14541	4	9	gnl|CDD|193050	pfam12490, BCAS3, Breast car	ref|XP_003707501.1|	PREDICTED: uncharacterized protein LOC100877372 [Megachile rotundata]	1,00E-72
gALL_v3_c14544	2	3	-	-	-	-	-
gALL_v3_c14547	5	3	-	-	-	-	-
gALL_v3_c14548	5	4	gnl|CDD|178494	PLN02906, PLN02906, xanthin	gb|EHJ74298.1|	xanthine dehydrogenase [Danaus plexippus]	1,00E-128
gALL_v3_c14549	1	6	gnl|CDD|29261	cd00204, ANK, ankyrin repeat:	ref|XP_003699218.1|	PREDICTED: ankyrin-2 [Megachile rotundata]	1,00E-100
gALL_v3_c1455	4	3	gnl|CDD|161970	TIGR00633, xth, exodeoxyribo	ref|XP_002422991.1|	Recombination repair protein, putative [Pediculus humanus corporis] gb|EEB10253.1| Recombination repair protein, putative [Pediculus humanus corporis]	9,00E-94
gALL_v3_c14551	3	5	gnl|CDD|191902	pfam07946, DUF1682, Protein	ref|XP_002424359.1|	coiled-coil domain-containing protein 47 precursor, putative [Pediculus humanus corporis] gb|EEB11621.1| coiled-coil domain-containing protein 47 precursor, putative [P	7,00E-32
gALL_v3_c14552	3	3	-	-	-	-	-
gALL_v3_c14553	2	3	-	-	-	-	-
gALL_v3_c14554	7	11	-	-	-	-	-
gALL_v3_c14556	5	5	-	-	ref|XP_003762084.1|	PREDICTED: insulin-like growth factor-binding protein complex acid labile subunit [Sarcophilus harrisii]	2,00E-29
gALL_v3_c14558	1	4	gnl|CDD|176290	cd01214, CG8312, CG8312 Ph	ref|XP_001948502.1|	PREDICTED: hypothetical protein LOC100164633 [Acyrthosiphon pisum]	2,00E-71
gALL_v3_c14559	2	6	-	-	-	-	-
gALL_v3_c1456	10	7	-	-	-	-	-
gALL_v3_c14560	3	6	-	-	-	-	-
gALL_v3_c14561	4	3	-	-	ref|XP_003704231.1|	PREDICTED: GATA zinc finger domain-containing protein 1-like [Megachile rotundata]	3,00E-62
gALL_v3_c14562	3	1	-	-	-	-	-
gALL_v3_c14563	4	5	-	-	-	-	-
gALL_v3_c14564	2	3	-	-	gb|AGM32231.1|	C10-like protein [Coptotermes formosanus]	2,00E-28
gALL_v3_c14565	2	2	-	-	-	-	-
gALL_v3_c14566	3	3	-	-	gb|EFN83346.1|	Baculoviral IAP repeat-containing protein 6 [Harpegnathos saltator]	2,00E-77
gALL_v3_c14567	4	2	-	-	-	-	-
gALL_v3_c14569	2	6	-	-	-	-	-
gALL_v3_c1457	7	13	-	-	ref|XP_002423560.1|	conserved hypothetical protein [Pediculus humanus corporis] gb|EEB10822.1| conserved hypothetical protein [Pediculus humanus corporis]	1,00E-120
gALL_v3_c14570	0	5	-	-	-	-	-
gALL_v3_c14571	13	11	gnl|CDD|73156	cd01650, RT_nLTR_like, RT_nL	gb|ADI61823.1|	endonuclease-reverse transcriptase [Bombyx mori]	2,00E-56
gALL_v3_c14572	2	5	-	-	ref|XP_003486430.1|	PREDICTED: uridine phosphorylase 1-like isoform 2 [Bombus impatiens]	6,00E-21
gALL_v3_c14573	8	4	-	-	-	-	-
gALL_v3_c14574	4	13	-	-	-	-	-
gALL_v3_c14575	1	5	-	-	-	-	-
gALL_v3_c14576	1	4	-	-	-	-	-
gALL_v3_c14577	8	12	-	-	gb|EFN83389.1|	Beta-1,4-mannosyltransferase egh [Harpegnathos saltator]	0
gALL_v3_c14578	1	5	-	-	-	-	-
gALL_v3_c14581	3	2	-	-	-	-	-
gALL_v3_c14582	5	6	-	-	gb|EFA01473.1|	hypothetical protein TcasGA2_TC007022 [Tribolium castaneum]	1,00E-09
gALL_v3_c14583	5	3	-	-	ref|XP_973573.2|	PREDICTED: similar to pasilla CG16765-PK [Tribolium castaneum]	5,00E-40
gALL_v3_c14584	4	4	-	-	ref|XP_003245025.1|	PREDICTED: hypothetical protein LOC100568766 [Acyrthosiphon pisum]	3,00E-10
gALL_v3_c14586	4	5	gnl|CDD|29151	cd00189, TPR, Tetratricopeptic	gb|ACY45102.1|	acetylglucosaminyl-transferase [Periplaneta americana]	1,00E-136
gALL_v3_c14587	6	7	gnl|CDD|29796	cd02897, A2M_2, Proteins sim	gb|EFZ22146.1|	hypothetical protein SINV_10929 [Solenopsis invicta]	1,00E-132
gALL_v3_c14588	3	3	-	-	-	-	-
gALL_v3_c14589	2	8	gnl|CDD|133179	cd05048, PTKc_Ror, Catalytic D	gb|EFN75674.1|	Tyrosine-protein kinase transmembrane receptor Ror [Harpegnathos saltator]	2,00E-31
gALL_v3_c1459	4	9	gnl|CDD|148263	pfam06553, BNIP3, BNIP3. Th	ref|XP_002427693.1|	BCL2/adenovirus E1B 19 kDa protein-interacting protein, putative [Pediculus humanus corporis] gb|EEB14955.1| BCL2/adenovirus E1B 19 kDa protein-interacting protein,	5,00E-83
gALL_v3_c14590	1	5	gnl|CDD|187700	cd09276, Rnase_HI_RT_non_L	-	-	-
gALL_v3_c14591	3	7	-	-	gb|AAZ15235.1|	reverse transcriptase [Aedes aegypti]	3,00E-19
gALL_v3_c14592	4	12	-	-	ref|XP_971157.1|	PREDICTED: similar to GA22042-PA [Tribolium castaneum] gb|EFA05000.1| hypothetical protein TcasGA2_TC015079 [Tribolium castaneum]	7,00E-08
gALL_v3_c14593	7	8	-	-	-	-	-
gALL_v3_c14594	3	3	-	-	gb|ADM21459.1|	beta-actin [Centroptilum triangulifer]	7,00E-09
gALL_v3_c14596	4	3	-	-	-	-	-
gALL_v3_c14597	3	4	-	-	-	-	-
gALL_v3_c14598	0	5	-	-	emb|CCO14466.1|	PREDICTED: similar to Zinc finger protein 271 (Zinc finger protein 7) (HZF7) (Zinc finger protein ZNFphex133) (Epstein-Barr virus-induced zinc finger protein) (ZNF-EB) (CT-ZF	3,00E-06
gALL_v3_c14599	2	6	-	-	-	-	-
gALL_v3_c146	28	17	gnl|CDD|150820	pfam10204, DuoxA, Dual oxid	ref|XP_971705.1|	PREDICTED: similar to moladietz CG4482-PD [Tribolium castaneum] gb|EFA08225.1| hypothetical protein TcasGA2_TC005852 [Tribolium castaneum]	1,00E-91
gALL_v3_c1460	4	7	-	-	-	-	-
gALL_v3_c14600	4	3	-	-	-	-	-
gALL_v3_c14601	2	3	-	-	gb|ACN86368.1|	troponin I transcript variant A [Bombyx mandarina]	5,00E-15
gALL_v3_c14602	3	4	gnl|CDD|147073	pfam04729, ASF1_hist_chap, /	ref|XP_002415044.1|	anti-silencing protein, putative [Ixodes scapularis] gb|EEC18709.1| anti-silencing protein, putative [Ixodes scapularis]	1,00E-94
gALL_v3_c14603	1	5	-	-	ref|XP_969792.2|	PREDICTED: similar to CG31772 CG31772-PA [Tribolium castaneum]	3,00E-43
gALL_v3_c14604	2	3	-	-	ref|XP_002423732.1|	conserved hypothetical protein [Pediculus humanus corporis] gb|EEB10994.1| conserved hypothetical protein [Pediculus humanus corporis]	9,00E-28
gALL_v3_c14605	3	6	gnl|CDD|29102	cd00162, RING, RING-finger (R	gb|EFN75399.1|	RING finger protein 181 [Harpegnathos saltator]	6,00E-51
gALL_v3_c14606	1	4	-	-	-	-	-
gALL_v3_c14607	3	5	-	-	-	-	-
gALL_v3_c14608	4	7	-	-	-	-	-
gALL_v3_c14609	4	2	gnl|CDD|173624	cd00192, PTKc, Catalytic doma	ref|XP_973940.1|	PREDICTED: similar to Wsck CG31127-PA [Tribolium castaneum] gb|EFA04011.1| hypothetical protein TcasGA2_TC014160 [Tribolium castaneum]	2,00E-40
gALL_v3_c1461	14	11	gnl|CDD|146000	pfam03154, Atrophin-1, Atrop	ref|XP_002428507.1|	hypothetical protein Phum_PHUM387840 [Pediculus humanus corporis] gb|EEB15769.1| hypothetical protein Phum_PHUM387840 [Pediculus humanus corporis]	1,00E-114
gALL_v3_c14610	1	4	-	-	-	-	-
gALL_v3_c14611	7	5	-	-	gb|AGM32990.1|	ferritin-like precursor [Coptotermes formosanus]	2,00E-22
gALL_v3_c14612	1	5	-	-	ref|XP_003702731.1|	PREDICTED: cAMP-specific 3',5'-cyclic phosphodiesterase-like [Megachile rotundata]	6,00E-41
gALL_v3_c14614	1	6	-	-	gb|EFN80294.1|	hypothetical protein EAI_10810 [Harpegnathos saltator]	2,00E-10
gALL_v3_c14615	3	1	-	-	-	-	-
gALL_v3_c14616	3	1	-	-	-	-	-

gALL_v3_c14618	11	8	-	-	-	-	-
gALL_v3_c14619	2	4	-	-	ref|XP_003725226.1|	PREDICTED: UPF0458 protein C7orf42-like isoform 1 [Strongylocentrotus purpuratus] ref|XP_003725227.1| PREDICTED: UPF0458 protein C7orf42-like isoform 2 [Strongylo	1,00E-05
gALL_v3_c14620	4	9	-	-	gb|AHB17950.1|	early growth response 2 [Locusta migratoria]	5,00E-48
gALL_v3_c14621	5	3	-	-	gb|EFN67034.1|	hypothetical protein EAG_04327 [Camponotus floridanus]	2,00E-06
gALL_v3_c14622	0	5	-	-	ref|XP_974477.1|	PREDICTED: similar to selenoprotein T [Tribolium castaneum] gb|EFA03473.1| hypothetical protein TcasGA2_TC013470 [Tribolium castaneum]	1,00E-32
gALL_v3_c14623	2	8	-	-	-	-	-
gALL_v3_c14624	0	6	gnl|CDD|30103	cd01381, MYSc_type_VII, Myc	ref|XP_003696352.1|	PREDICTED: LOW QUALITY PROTEIN: myosin-VIIa-like [Apis florea]	1,00E-157
gALL_v3_c14625	1	6	-	-	-	-	-
gALL_v3_c14627	3	4	-	-	-	-	-
gALL_v3_c1463	6	10	-	-	-	-	-
gALL_v3_c14630	9	8	-	-	-	-	-
gALL_v3_c14631	3	2	-	-	-	-	-
gALL_v3_c14632	5	11	-	-	-	-	-
gALL_v3_c14633	6	4	-	-	-	-	-
gALL_v3_c14634	2	4	gnl|CDD|190267	pfam02271, UCR_14kD, Ubiqu	ref|NP_001155372.1|	ubiquinol-cytochrome c reductase binding protein [Acyrthosiphon pisum] dbj|BAH72104.1| ACYPI000205 [Acyrthosiphon pisum]	3,00E-40
gALL_v3_c14635	3	9	-	-	-	-	-
gALL_v3_c14637	3	2	-	-	-	-	-
gALL_v3_c14638	3	4	-	-	-	-	-
gALL_v3_c14639	6	7	-	-	-	-	-
gALL_v3_c1464	11	14	gnl|CDD|144098	pfam00379, Chitin_bind_4, Ins	ref|XP_973729.1|	PREDICTED: similar to cuticular protein 78, RR-1 family (AGAP009876-PA) [Tribolium castaneum] gb|EFA03217.1| hypothetical protein TcasGA2_TC013137 [Tribolium casta	9,00E-26
gALL_v3_c14640	4	3	gnl|CDD|29257	cd00200, WD40, WD40 domai	ref|XP_004932210.1|	PREDICTED: notchless protein homolog 1-like isoform X2 [Bombyx mori]	1,00E-117
gALL_v3_c14641	0	4	-	-	-	-	-
gALL_v3_c14642	0	4	-	-	-	-	-
gALL_v3_c14643	3	7	-	-	ref|XP_001605197.2|	PREDICTED: dual specificity tyrosine-phosphorylation-regulated kinase 2-like isoform 1 [Nasonia vitripennis]	3,00E-08
gALL_v3_c14644	3	2	-	-	-	-	-
gALL_v3_c14645	2	8	gnl|CDD|177628	PHA03392, egt, ecdysteroid UI	ref|XP_001948117.1|	PREDICTED: UDP-glucuronosyltransferase 2B10-like [Acyrthosiphon pisum]	5,00E-82
gALL_v3_c14646	5	7	-	-	-	-	-
gALL_v3_c14647	4	5	-	-	ref|XP_007069719.1|	PREDICTED: zinc finger protein 271-like [Chelonia mydas]	7,00E-17
gALL_v3_c14648	3	7	gnl|CDD|73156	cd01650, RT_nLTR_like, RT_nL	gb|AAL40415.1|AF3698	endonuclease/reverse transcriptase [Branchiostoma floridae]	1,00E-22
gALL_v3_c14649	1	4	-	-	-	-	-
gALL_v3_c14650	3	3	-	-	-	-	-
gALL_v3_c14652	6	8	gnl|CDD|33212	COG3405, CelA, Endoglucanas	gb|AGM32797.1|	glycosyl hydrolases family 8, partial [Coptotermes formosanus]	1,00E-171
gALL_v3_c14653	1	6	-	-	ref|XP_976337.1|	PREDICTED: hypothetical protein [Tribolium castaneum] gb|EEZ98319.1| hypothetical protein TcasGA2_TC000773 [Tribolium castaneum]	8,00E-07
gALL_v3_c14654	1	5	-	-	-	-	-
gALL_v3_c14655	1	4	-	-	-	-	-
gALL_v3_c14657	2	5	-	-	gb|EGI60364.1|	Kinesin-like protein KIF13A [Acromyrmex echinatior]	1,00E-101
gALL_v3_c14658	2	2	-	-	-	-	-
gALL_v3_c14659	3	2	-	-	-	-	-
gALL_v3_c14660	2	3	-	-	-	-	-
gALL_v3_c14661	8	15	gnl|CDD|176398	cd01803, Ubiquitin, Ubiquitin.	prf||1212243B	ubiquitin S5	2,00E-42
gALL_v3_c14662	3	4	-	-	-	-	-
gALL_v3_c14663	3	2	-	-	-	-	-
gALL_v3_c14665	5	11	-	-	ref|XP_003699219.1|	PREDICTED: uncharacterized protein LOC100879726 [Megachile rotundata]	3,00E-92
gALL_v3_c14666	6	6	-	-	gb|ABN12031.1|	putative coatomer protein complex, subunit beta 2 [Maconellicoccus hirsutus]	1,00E-30
gALL_v3_c14668	4	6	gnl|CDD|152412	pfam11977, RNase_Zc3h12a, 2	gb|EHJ66150.1|	hypothetical protein KGM_22206 [Danaus plexippus]	3,00E-44
gALL_v3_c14669	11	15	gnl|CDD|73156	cd01650, RT_nLTR_like, RT_nL	gb|EFN64073.1|	Craniofacial development protein 2 [Camponotus floridanus]	7,00E-41
gALL_v3_c1467	11	19	-	-	ref|XP_002004988.1|	GI19305 [Drosophila mojavensis] gb|EDW08923.1| GI19305 [Drosophila mojavensis]	7,00E-18
gALL_v3_c14670	3	4	-	-	ref|XP_002401725.1|	hypothetical protein IscW_ISCW024171 [Ixodes scapularis] gb|EEC02849.1| hypothetical protein IscW_ISCW024171 [Ixodes scapularis]	2,00E-06
gALL_v3_c14671	1	4	-	-	-	-	-
gALL_v3_c14673	3	3	-	-	ref|XP_004922065.1|	PREDICTED: uncharacterized protein LOC101745645 [Bombyx mori]	7,00E-23
gALL_v3_c14674	3	3	-	-	-	-	-
gALL_v3_c14675	0	5	-	-	-	-	-
gALL_v3_c14676	2	6	-	-	ref|XP_003494656.1|	PREDICTED: hypothetical protein LOC100746638 [Bombus impatiens]	5,00E-10
gALL_v3_c14677	0	5	-	-	-	-	-
gALL_v3_c14678	0	5	-	-	-	-	-
gALL_v3_c14679	10	10	gnl|CDD|146069	pfam03250, Tropomodulin, Tro	ref|XP_003700640.1|	PREDICTED: tropomodulin-like [Megachile rotundata]	1,00E-150
gALL_v3_c1468	5	16	-	-	-	-	-
gALL_v3_c14680	3	5	-	-	-	-	-
gALL_v3_c14681	4	8	gnl|CDD|146538	pfam03957, Jun, Jun-like trans	gb|AGM32319.1|	transcription factor AP-1(jun-like transcription factor), partial [Coptotermes formosanus]	5,00E-74
gALL_v3_c14684	3	1	-	-	-	-	-
gALL_v3_c14685	3	1	-	-	-	-	-
gALL_v3_c14686	10	9	-	-	-	-	-
gALL_v3_c14687	3	4	-	-	-	-	-
gALL_v3_c14688	4	5	-	-	-	-	-
gALL_v3_c14689	4	9	gnl|CDD|143985	pfam00232, Glyco_hydro_1, G	gb|AEW67361.1|	beta-glucosidase [Coptotermes formosanus]	1,00E-142
gALL_v3_c1469	19	16	gnl|CDD|162106	TIGR00917, 2A060601, Niema	ref|XP_006621496.1|	PREDICTED: niemann-Pick C1 protein-like isoform X3 [Apis dorsata]	3,00E-51
gALL_v3_c14690	0	4	-	-	-	-	-
gALL_v3_c14691	2	2	-	-	-	-	-
gALL_v3_c14692	1	3	-	-	-	-	-
gALL_v3_c14693	1	4	-	-	ref|XP_001952847.1|	PREDICTED: hypothetical protein LOC100160253 [Acyrthosiphon pisum]	4,00E-30
gALL_v3_c14694	5	6	gnl|CDD|132999	cd00899, b4GalT, Beta-4-Galac	ref|XP_003486181.1|	PREDICTED: beta-1,4-N-acetylgalactosaminyltransferase bre-4-like isoform 1 [Bombus impatiens] ref|XP_003486182.1| PREDICTED: beta-1,4-N-acetylgalactosaminyltransfe	3,00E-35
gALL_v3_c14695	3	6	gnl|CDD|119392	cd06174, MFS, The Major Faci	ref|XP_002430729.1|	monocarboxylate transporter, putative [Pediculus humanus corporis] gb|EEB17991.1| monocarboxylate transporter, putative [Pediculus humanus corporis]	6,00E-92
gALL_v3_c14696	6	9	-	-	-	-	-
gALL_v3_c14697	8	10	-	-	-	-	-
gALL_v3_c14698	2	2	-	-	gb|ENN81519.1|	hypothetical protein YQE_02048, partial [Dendroctonus ponderosae]	4,00E-64
gALL_v3_c14699	4	3	-	-	-	-	-
gALL_v3_c1470	4	4	gnl|CDD|33904	COG4154, FucU, Fucose dissim	gb|ESO99013.1|	hypothetical protein LOTGIDRAFT_201494 [Lottia gigantea]	3,00E-52
gALL_v3_c14700	5	5	-	-	-	-	-
gALL_v3_c14701	3	4	-	-	-	-	-
gALL_v3_c14702	4	3	gnl|CDD|133283	cd01883, EF1_alpha, Eukaryot	ref|XP_002425558.1|	eukaryotic peptide chain release factor GTP-binding subunit, putative [Pediculus humanus corporis] gb|EEB12820.1| eukaryotic peptide chain release factor GTP-binding su	1,00E-115
gALL_v3_c14703	9	8	gnl|CDD|190555	pfam03184, DDE_1, DDE supe	ref|XP_006038273.1|	PREDICTED: jerky protein-like [Alligator sinensis]	2,00E-56
gALL_v3_c14704	2	3	gnl|CDD|128691	smart00413, ETS, erythroblast	dbj|BAM18250.1|	ETS transcription factor at 98B [Papilio xuthus]	4,00E-56
gALL_v3_c14706	5	4	-	-	-	-	-
gALL_v3_c14707	4	5	-	-	-	-	-
gALL_v3_c14708	1	3	-	-	gb|ETN66913.1|	guanylate cyclase [Anopheles darlingi]	3,00E-15
gALL_v3_c14709	3	6	-	-	-	-	-
gALL_v3_c1471	8	4	-	-	gb|EFN85387.1|	Protein dopey-1-like protein [Harpegnathos saltator]	1,00E-109
gALL_v3_c14711	4	4	-	-	-	-	-
gALL_v3_c14713	2	8	gnl|CDD|192052	pfam08510, PIG-P, PIG-P. PIG-	ref|NP_001155061.1|	phosphatidylinositol glycan anchor biosynthesis, class P [Nasonia vitripennis]	5,00E-33
gALL_v3_c14714	2	2	-	-	ref|XP_002430813.1|	hypothetical protein Phum_PHUM500510 [Pediculus humanus corporis] gb|EEB18075.1| hypothetical protein Phum_PHUM500510 [Pediculus humanus corporis]	1,00E-25
gALL_v3_c14715	4	2	gnl|CDD|148286	pfam06585, JHBP, Haemolymp	ref|XP_001947537.1|	PREDICTED: protein takeout-like [Acyrthosiphon pisum]	3,00E-53
gALL_v3_c14716	2	3	-	-	-	-	-
gALL_v3_c14717	2	4	-	-	-	-	-
gALL_v3_c14718	6	6	gnl|CDD|34673	COG5069, SAC6, Ca2+-binding	ref|XP_001326306.1|	fimbrin [Trichomonas vaginalis G3] gb|EAY14083.1| fimbrin, putative [Trichomonas vaginalis G3]	4,00E-95
gALL_v3_c14719	4	3	-	-	ref|XP_002425572.1|	lingerer, putative [Pediculus humanus corporis] gb|EEB12834.1| lingerer, putative [Pediculus humanus corporis]	2,00E-13
gALL_v3_c1472	5	12	gnl|CDD|143985	pfam00232, Glyco_hydro_1, G	gb|AGM32287.1|	beta-glucosidase, partial [Coptotermes formosanus]	6,00E-99
gALL_v3_c14720	4	8	-	-	ref|XP_967865.2|	PREDICTED: similar to pumilio [Tribolium castaneum]	4,00E-07
gALL_v3_c14721	1	5	-	-	-	-	-
gALL_v3_c14722	1	7	-	-	-	-	-
gALL_v3_c14723	8	4	gnl|CDD|32340	COG2157, RPL20A, Ribosomal	gb|ABD98777.1|	putative ribosomal protein L18A [Graphocephala atropunctata]	1,00E-112
gALL_v3_c14724	1	5	-	-	-	-	-
gALL_v3_c14725	4	4	-	-	-	-	-
gALL_v3_c14728	14	15	-	-	-	-	-
gALL_v3_c14729	6	4	-	-	gb|EFN68518.1|	Peritrophin-1 [Camponotus floridanus]	5,00E-18
gALL_v3_c1473	14	27	gnl|CDD|148425	pfam06809, NPDC1, Neural pr	ref|XP_003396489.1|	PREDICTED: hypothetical protein LOC100645490 isoform 1 [Bombus terrestris]	4,00E-41
gALL_v3_c14730	3	2	-	-	-	-	-
gALL_v3_c14731	5	3	gnl|CDD|32933	COG3119, AslA, Arylsulfatase /	gb|EFN61139.1|	Arylsulfatase B [Camponotus floridanus]	1,00E-153
gALL_v3_c14732	3	6	-	-	-	-	-
gALL_v3_c14734	3	6	gnl|CDD|179704	PRK04004, PRK04004, translat	gb|ETN57972.1|	translation initiation factor if-2, partial [Anopheles darlingi]	1,00E-44
gALL_v3_c14735	1	6	-	-	gb|ERL86997.1|	hypothetical protein D910_04400 [Dendroctonus ponderosae]	2,00E-12
gALL_v3_c14736	3	4	-	-	ref|XP_006251575.1|	PREDICTED: zinc finger protein 729-like [Rattus norvegicus]	1,00E-13
gALL_v3_c14738	0	4	-	-	-	-	-
gALL_v3_c14739	3	4	-	-	-	-	-
gALL_v3_c1474	9	5	-	-	-	-	-
gALL_v3_c14740	4	4	gnl|CDD|190157	pfam01915, Glyco_hydro_3_C	ref|WP_021935784.1|	uncharacterized protein [Bacteroides sp. CAG:189] emb|CCY52709.1| uncharacterized protein BN523_00927 [Bacteroides sp. CAG:189]	8,00E-75
gALL_v3_c14741	3	3	-	-	ref|XP_002430446.1|	leucine-rich repeat flightless-interacting protein, putative [Pediculus humanus corporis] gb|EEB17708.1| leucine-rich repeat flightless-interacting protein, putative [Pedicul	2,00E-17
gALL_v3_c14743	8	7	-	-	-	-	-
gALL_v3_c14744	2	8	gnl|CDD|110585	pfam01591, 6PF2K, 6-phosphc	ref|XP_001944498.1|	PREDICTED: 6-phosphofructo-2-kinase/fructose-2,6-biphosphatase-like [Acyrthosiphon pisum]	6,00E-60
gALL_v3_c14745	3	3	-	-	-	-	-
gALL_v3_c14746	2	5	-	-	ref|XP_002430295.1|	cAMP-dependent protein kinase catalytic subunit, putative [Pediculus humanus corporis] gb|EEB17557.1| cAMP-dependent protein kinase catalytic subunit, putative [Pedi	9,00E-75
gALL_v3_c14747	3	3	-	-	gb|EGI65030.1|	Sorting nexin-25 [Acromyrmex echinatior]	2,00E-25
gALL_v3_c14748	3	9	-	-	ref|XP_006572407.1|	PREDICTED: putative uncharacterized protein DDB_G0277255 isoform X1 [Apis mellifera] ref|XP_006572408.1| PREDICTED: putative uncharacterized protein DDB_G02772	3,00E-07
gALL_v3_c14749	2	4	gnl|CDD|162843	TIGR02403, trehalose_treC, al	ref|XP_006638704.1|	PREDICTED: neutral and basic amino acid transport protein rBAT-like [Lepisosteus oculatus]	1,00E-50
gALL_v3_c1475	8	10	gnl|CDD|189358	pfam00038, Filament, Interme	ref|XP_003424206.1|	PREDICTED: lamin Dm0-like isoform 2 [Nasonia vitripennis]	1,00E-118
gALL_v3_c14750	2	7	-	-	-	-	-
gALL_v3_c14751	3	7	-	-	ref|XP_002429338.1|	lis1-interacting protein nude, putative [Pediculus humanus corporis] gb|EEB16600.1| lis1-interacting protein nude, putative [Pediculus humanus corporis]	1,00E-35
gALL_v3_c14752	1	6	-	-	-	-	-
gALL_v3_c14753	1	4	-	-	-	-	-
gALL_v3_c14754	2	6	gnl|CDD|190403	pfam02732, ERCC4, ERCC4 doi	ref|XP_003699299.1|	PREDICTED: crossover junction endonuclease MUS81-like [Megachile rotundata]	5,00E-85
gALL_v3_c14757	3	6	gnl|CDD|189008	cd09601, M1_APN_2, Peptida:	ref|XP_396261.2|	PREDICTED: aminopeptidase N-like isoformX1 [Apis mellifera] ref|XP_006565538.1| PREDICTED: aminopeptidase N-like isoform X3 [Apis mellifera]	2,00E-38
gALL_v3_c14758	6	5	-	-	-	-	-
gALL_v3_c14759	5	14	-	-	-	-	-
gALL_v3_c1476	24	32	gnl|CDD|35161	COG5602, SIN3, Histone deace	ref|XP_006562809.1|	PREDICTED: paired amphipathic helix protein Sin3a isoform X3 [Apis mellifera] ref|XP_006607144.1| PREDICTED: paired amphipathic helix protein Sin3a-like isoform X4 [A	1,00E-162
gALL_v3_c14760	2	4	-	-	-	-	-
gALL_v3_c14761	2	3	-	-	-	-	-
gALL_v3_c14762	2	4	-	-	-	-	-
gALL_v3_c14763	5	2	gnl|CDD|114381	pfam05653, DUF803, Protein c	ref|XP_318951.3|	AGAP009838-PA [Anopheles gambiae str. PEST] gb|EAA14313.3| AGAP009838-PA [Anopheles gambiae str. PEST]	4,00E-64
gALL_v3_c14765	2	3	-	-	ref|XP_004923418.1|	PREDICTED: piggyBac transposable element-derived protein 4-like [Bombyx mori]	4,00E-16
gALL_v3_c14767	4	11	-	-	-	-	-
gALL_v3_c14768	5	6	-	-	ref|XP_973508.1|	PREDICTED: similar to d-alanyl-d-alanine carboxypeptidase [Tribolium castaneum] gb|EFA06807.1| hypothetical protein TcasGA2_TC009746 [Tribolium castaneum]	1,00E-15
gALL_v3_c14769	5	3	-	-	-	-	-
gALL_v3_c1477	8	9	-	-	-	-	-
gALL_v3_c14770	3	2	-	-	-	-	-
gALL_v3_c14771	3	3	-	-	-	-	-

gALL_v3_c14773	5	5	-	-	ref|XP_976386.1|	PREDICTED: hypothetical protein [Tribolium castaneum] gb|EFA00588.1| hypothetical protein TcasGA2_TC003458 [Tribolium castaneum]	4,00E-13
gALL_v3_c14775	5	5	-	-	-	-	-
gALL_v3_c14776	6	7	-	-	-	-	-
gALL_v3_c14777	1	7	-	-	-	-	-
gALL_v3_c14778	1	6	-	-	-	-	-
gALL_v3_c14779	1	5	-	-	-	-	-
gALL_v3_c14781	3	11	gnl|CDD|162472	TIGR01661, ELAV_HUD_SF, EL	gb|EGI69627.1|	ELAV-like protein 2 [Acromyrmex echinatior]	0
gALL_v3_c14782	1	5	-	-	-	-	-
gALL_v3_c14783	4	10	-	-	-	-	-
gALL_v3_c14784	2	6	-	-	-	-	-
gALL_v3_c14785	1	4	-	-	-	-	-
gALL_v3_c14787	3	1	-	-	-	-	-
gALL_v3_c14788	1	5	-	-	gb|EFA12330.1|	hypothetical protein TcasGA2_TC005158 [Tribolium castaneum]	4,00E-09
gALL_v3_c14789	1	4	-	-	-	-	-
gALL_v3_c1479	4	6	-	-	-	-	-
gALL_v3_c14790	1	6	-	-	-	-	-
gALL_v3_c14791	4	3	gnl|CDD|176401	cd01806, Nedd8, Nebb8-like	gb|AGM33003.1|	Nebb8-like ubiquitin protein [Coptotermes formosanus]	1,00E-25
gALL_v3_c14792	7	5	-	-	dbj|BAN20738.1|	sentrin/sumo-specific protease senp7 [Riptortus pedestris]	1,00E-22
gALL_v3_c14793	3	2	-	-	ref|XP_002433518.1|	conserved hypothetical protein [Ixodes scapularis] gb|EEC00582.1| conserved hypothetical protein [Ixodes scapularis]	6,00E-10
gALL_v3_c14794	5	5	gnl|CDD|165506	PHA03247, PHA03247, large te	ref|XP_001603511.2|	PREDICTED: LOW QUALITY PROTEIN: collagen alpha-1(IV) chain-like [Nasonia vitripennis]	9,00E-37
gALL_v3_c14795	4	7	-	-	gb|EFZ16343.1|	hypothetical protein SINV_08030 [Solenopsis invicta]	1,00E-124
gALL_v3_c14796	3	7	gnl|CDD|29257	cd00200, WD40, WD40 domai	ref|XP_003701876.1|	PREDICTED: nucleoporin seh1-A-like [Megachile rotundata]	1,00E-169
gALL_v3_c14797	8	7	gnl|CDD|143392	cd05402, NT_PAP_TUTase, Nu	ref|XP_006824330.1|	PREDICTED: terminal uridylyltransferase 4-like [Saccoglossus kowalevskii]	3,00E-89
gALL_v3_c14798	1	4	-	-	-	-	-
gALL_v3_c14799	3	7	-	-	-	-	-
gALL_v3_c148	11	25	-	-	-	-	-
gALL_v3_c1480	10	9	-	-	-	-	-
gALL_v3_c14800	2	2	-	-	-	-	-
gALL_v3_c14801	6	9	gnl|CDD|100104	cd00590, RRM, RRM (RNA rec	ref|XP_974350.1|	PREDICTED: similar to CG4806 CG4806-PA [Tribolium castaneum] gb|EFA01819.1| hypothetical protein TcasGA2_TC007421 [Tribolium castaneum]	2,00E-43
gALL_v3_c14803	4	3	-	-	ref|XP_004925238.1|	PREDICTED: multiple epidermal growth factor-like domains protein 11-like [Bombyx mori]	4,00E-21
gALL_v3_c14804	3	7	-	-	-	-	-
gALL_v3_c14806	5	2	-	-	-	-	-
gALL_v3_c14807	3	7	gnl|CDD|162099	TIGR00901, 2A0125, AmpG-re	ref|XP_001600710.1|	PREDICTED: acetyl-coenzyme A transporter 1-like [Nasonia vitripennis]	3,00E-84
gALL_v3_c14808	3	2	-	-	-	-	-
gALL_v3_c14809	0	4	-	-	-	-	-
gALL_v3_c1481	11	16	gnl|CDD|145919	pfam03028, Dynein_heavy, Dy	ref|XP_003707959.1|	PREDICTED: dynein heavy chain, cytoplasmic-like isoform 3 [Megachile rotundata]	3,00E-66
gALL_v3_c14810	3	7	-	-	-	-	-
gALL_v3_c14811	3	4	-	-	-	-	-
gALL_v3_c14813	0	6	-	-	-	-	-
gALL_v3_c14814	2	9	-	-	gb|EFZ09412.1|	hypothetical protein SINV_10688 [Solenopsis invicta]	1,00E-110
gALL_v3_c14815	0	6	-	-	-	-	-
gALL_v3_c14817	1	4	-	-	-	-	-
gALL_v3_c14818	1	3	-	-	-	-	-
gALL_v3_c14819	8	6	-	-	ref|XP_001950944.1|	PREDICTED: hypothetical protein LOC100163923 isoform 1 [Acyrthosiphon pisum] ref|XP_003245520.1| PREDICTED: hypothetical protein LOC100163923 isoform 2 [Acyrtl	2,00E-10
gALL_v3_c1482	24	25	gnl|CDD|150314	pfam09606, Med15, ARC105 c	ref|XP_002426459.1|	hypothetical protein Phum_PHUM254210 [Pediculus humanus corporis] gb|EEB13721.1| hypothetical protein Phum_PHUM254210 [Pediculus humanus corporis]	3,00E-12
gALL_v3_c14820	2	5	-	-	-	-	-
gALL_v3_c14821	0	4	-	-	-	-	-
gALL_v3_c14822	1	4	-	-	-	-	-
gALL_v3_c14823	3	5	-	-	-	-	-
gALL_v3_c14824	2	3	gnl|CDD|189373	pfam00069, Pkinase, Protein k	ref|XP_623397.2|	PREDICTED: casein kinase II subunit alpha isoformX2 [Apis mellifera] ref|XP_006570473.1| PREDICTED: casein kinase II subunit alpha isoform X3 [Apis mellifera]	6,00E-57
gALL_v3_c14826	5	5	-	-	-	-	-
gALL_v3_c14827	11	8	-	-	-	-	-
gALL_v3_c14828	2	3	-	-	-	-	-
gALL_v3_c14829	3	3	-	-	-	-	-
gALL_v3_c1483	4	4	-	-	ref|XP_002404568.1|	dual specificity protein phosphatase CDC-14 alpha, putative [Ixodes scapularis] gb|EEC01231.1| dual specificity protein phosphatase CDC-14 alpha, putative [Ixodes scapul	1,00E-20
gALL_v3_c14830	6	7	-	-	-	-	-
gALL_v3_c14831	2	4	gnl|CDD|29115	cd00170, SEC14, Sec14p-like li	gb|EFX90292.1|	hypothetical protein DAPPUDRAFT_309832 [Daphnia pulex]	5,00E-89
gALL_v3_c14832	2	4	-	-	-	-	-
gALL_v3_c14833	2	5	-	-	-	-	-
gALL_v3_c14835	7	6	-	-	ref|XP_003424199.1|	PREDICTED: tetratricopeptide repeat protein 17-like [Nasonia vitripennis]	5,00E-79
gALL_v3_c14837	6	4	-	-	ref|XP_003485429.1|	PREDICTED: transportin-1-like [Bombus impatiens]	1,00E-43
gALL_v3_c14838	1	5	-	-	-	-	-
gALL_v3_c14839	3	1	-	-	-	-	-
gALL_v3_c1484	6	7	-	-	-	-	-
gALL_v3_c14840	1	4	-	-	-	-	-
gALL_v3_c14841	2	3	-	-	-	-	-
gALL_v3_c14842	0	4	-	-	-	-	-
gALL_v3_c14843	1	4	-	-	-	-	-
gALL_v3_c14844	1	4	-	-	gb|EFN67750.1|	Transmembrane protein 131 [Camponotus floridanus]	1,00E-13
gALL_v3_c14845	1	5	-	-	-	-	-
gALL_v3_c14846	0	5	-	-	-	-	-
gALL_v3_c14847	1	5	gnl|CDD|192379	pfam09790, Hyccin, Hyccin. M	ref|XP_003491729.1|	PREDICTED: hyccin-like [Bombus impatiens]	3,00E-32
gALL_v3_c14848	2	3	-	-	gb|EFN89132.1|	PiggyBac transposable element-derived protein 4 [Harpegnathos saltator]	8,00E-32
gALL_v3_c14849	4	2	-	-	-	-	-
gALL_v3_c14850	3	4	-	-	ref|XP_003245706.1|	PREDICTED: hypothetical protein LOC100158733 isoform 2 [Acyrthosiphon pisum]	5,00E-32
gALL_v3_c14851	4	2	-	-	-	-	-
gALL_v3_c14852	3	8	gnl|CDD|109126	pfam00057, Ldl_recept_a, Low	gb|EFA12832.1|	hypothetical protein TcasGA2_TC004260 [Tribolium castaneum]	0
gALL_v3_c14853	5	2	-	-	-	-	-
gALL_v3_c14855	1	4	-	-	gb|EHJ71895.1|	hypothetical protein KGM_20654 [Danaus plexippus]	8,00E-07
gALL_v3_c14856	1	8	gnl|CDD|129055	smart00822, PKS_KR, This enz	ref|XP_002429689.1|	fatty acid synthase, putative [Pediculus humanus corporis] gb|EEB16951.1| fatty acid synthase, putative [Pediculus humanus corporis]	2,00E-39
gALL_v3_c14858	1	6	-	-	-	-	-
gALL_v3_c1486	9	14	-	-	-	-	-
gALL_v3_c14860	3	3	gnl|CDD|189432	pfam00169, PH, PH domain. F	gb|EFA03484.1|	hypothetical protein TcasGA2_TC013483 [Tribolium castaneum]	1,00E-120
gALL_v3_c14862	5	3	gnl|CDD|99772	cd01735, LSm12_N, LSm12 be	ref|XP_001607591.1|	PREDICTED: protein LSM12 homolog [Nasonia vitripennis]	1,00E-51
gALL_v3_c14864	3	4	-	-	-	-	-
gALL_v3_c14865	1	4	-	-	-	-	-
gALL_v3_c14867	5	3	-	-	-	-	-
gALL_v3_c14868	6	11	-	-	-	-	-
gALL_v3_c14869	4	3	gnl|CDD|129116	TIGR00004, TIGR00004, endor	ref|XP_006916719.1|	PREDICTED: ribonuclease UK114 [Pteropus alecto]	3,00E-37
gALL_v3_c14870	2	3	-	-	-	-	-
gALL_v3_c14871	2	3	gnl|CDD|129036	smart00800, uDENN, Domain	gb|EGI59736.1|	C-myc promoter-binding protein [Acromyrmex echinatior]	6,00E-62
gALL_v3_c14874	2	6	-	-	-	-	-
gALL_v3_c14875	5	6	-	-	-	-	-
gALL_v3_c14876	6	3	-	-	-	-	-
gALL_v3_c14877	5	3	gnl|CDD|188999	cd06461, M2_ACE, Peptidase	ref|XP_002431342.1|	Angiotensin-converting enzyme precursor, putative [Pediculus humanus corporis] gb|EEB18604.1| Angiotensin-converting enzyme precursor, putative [Pediculus humanus	1,00E-107
gALL_v3_c14878	0	5	-	-	-	-	-
gALL_v3_c14879	2	4	gnl|CDD|129024	smart00788, Adenylsucc_synt	gb|EFA07326.1|	hypothetical protein TcasGA2_TC015922 [Tribolium castaneum]	2,00E-13
gALL_v3_c1488	21	13	gnl|CDD|187737	cd09616, Peptidase_C12_UCH	gb|AGM32543.1|	cysteine peptidase C12 containing ubiquitin carboxy-terminal hydrolase [Coptotermes formosanus]	1,00E-104
gALL_v3_c14880	3	3	-	-	ref|XP_003425288.1|	PREDICTED: COP9 signalosome complex subunit 2-like isoform 2 [Nasonia vitripennis]	1,00E-94
gALL_v3_c14881	0	5	-	-	-	-	-
gALL_v3_c14882	2	3	-	-	ref|XP_006572074.1|	PREDICTED: FERM domain-containing protein 5-like isoform X3 [Apis mellifera]	6,00E-58
gALL_v3_c14883	5	3	-	-	-	-	-
gALL_v3_c14885	5	14	-	-	ref|XP_002406195.1|	centromere protein B, putative [Ixodes scapularis] gb|EEC10272.1| centromere protein B, putative [Ixodes scapularis]	7,00E-06
gALL_v3_c14886	3	3	-	-	ref|XP_006571612.1|	PREDICTED: bromodomain adjacent to zinc finger domain protein 2B isoform X8 [Apis mellifera]	6,00E-17
gALL_v3_c14887	3	2	gnl|CDD|146145	pfam03357, Snf7, Snf7. This fa	ref|XP_006623295.1|	PREDICTED: charged multivesicular body protein 2a-like isoform X1 [Apis dorsata] ref|XP_006623296.1| PREDICTED: charged multivesicular body protein 2a-like isoform X2	3,00E-50
gALL_v3_c14889	3	6	-	-	gb|EFZ09456.1|	hypothetical protein SINV_10618 [Solenopsis invicta]	3,00E-16
gALL_v3_c14890	1	3	-	-	-	-	-
gALL_v3_c14891	4	8	-	-	-	-	-
gALL_v3_c14892	5	4	gnl|CDD|30069	cd01438, tankyrase_like, Tank	ref|XP_967640.1|	PREDICTED: similar to tankyrase [Tribolium castaneum]	9,00E-22
gALL_v3_c14893	3	1	-	-	-	-	-
gALL_v3_c14894	2	5	gnl|CDD|58503	cd01826, acyloxyacyl_hydrola	ref|XP_969018.1|	PREDICTED: similar to acyloxyacyl hydrolase [Tribolium castaneum] gb|EEZ98824.1| hypothetical protein TcasGA2_TC004431 [Tribolium castaneum]	1,00E-37
gALL_v3_c14895	2	5	-	-	ref|XP_968141.2|	PREDICTED: similar to conserved hypothetical protein [Tribolium castaneum] gb|EFA05811.1| hypothetical protein TcasGA2_TC030766 [Tribolium castaneum]	8,00E-33
gALL_v3_c14897	4	14	-	-	-	-	-
gALL_v3_c14898	3	2	-	-	ref|XP_002032772.1|	GM20775 [Drosophila sechellia] gb|EDW46785.1| GM20775 [Drosophila sechellia]	8,00E-06
gALL_v3_c14899	8	10	-	-	ref|XP_001866982.1|	pre-mRNA-splicing factor prp1 [Culex quinquefasciatus] gb|EDS44272.1| pre-mRNA-splicing factor prp1 [Culex quinquefasciatus]	2,00E-70
gALL_v3_c1490	4	3	gnl|CDD|180295	PRK05862, PRK05862, enoyl-C	gb|AGM32524.1|	putative cyclohex-1-ene-1-carboxyl-coA hydratase, partial [Coptotermes formosanus]	6,00E-73
gALL_v3_c14900	0	4	-	-	-	-	-
gALL_v3_c14901	5	3	-	-	ref|XP_002425538.1|	conserved hypothetical protein [Pediculus humanus corporis] gb|EEB12800.1| conserved hypothetical protein [Pediculus humanus corporis]	1,00E-13
gALL_v3_c14902	3	4	-	-	-	-	-
gALL_v3_c14903	4	3	gnl|CDD|192035	pfam08430, Fork_head_N, For	ref|XP_006623811.1|	PREDICTED: zinc finger MIZ domain-containing protein 1-like [Apis dorsata]	4,00E-12
gALL_v3_c14904	7	7	gnl|CDD|181350	PRK08279, PRK08279, long-ch	ref|XP_971856.1|	PREDICTED: similar to AMP dependent ligase [Tribolium castaneum] gb|EFA03519.1| hypothetical protein TcasGA2_TC013521 [Tribolium castaneum]	1,00E-100
gALL_v3_c14905	0	5	-	-	-	-	-
gALL_v3_c14907	7	6	-	-	gb|EFZ10365.1|	hypothetical protein SINV_11683 [Solenopsis invicta]	3,00E-35
gALL_v3_c14908	2	3	-	-	-	-	-
gALL_v3_c1491	12	14	-	-	-	-	-
gALL_v3_c14910	2	3	-	-	-	-	-
gALL_v3_c14911	1	3	-	-	-	-	-
gALL_v3_c14912	4	10	-	-	-	-	-
gALL_v3_c14913	2	9	-	-	-	-	-
gALL_v3_c14915	2	6	-	-	-	-	-
gALL_v3_c14916	4	4	-	-	-	-	-
gALL_v3_c14917	6	3	-	-	-	-	-
gALL_v3_c14921	3	2	-	-	-	-	-
gALL_v3_c14922	2	3	-	-	-	-	-
gALL_v3_c14924	4	6	gnl|CDD|128591	smart00295, B41, Band 4.1 ho	ref|XP_003697160.1|	PREDICTED: FERM domain-containing protein 3-like [Apis florea]	2,33E-156
gALL_v3_c14925	2	3	-	-	-	-	-
gALL_v3_c14926	1	5	-	-	ref|XP_006638701.1|	PREDICTED: protein kinase C epsilon type-like [Lepisosteus oculatus]	9,00E-08
gALL_v3_c14927	0	4	-	-	-	-	-
gALL_v3_c14928	9	8	-	-	-	-	-
gALL_v3_c1493	13	16	-	-	-	-	-
gALL_v3_c14930	5	2	-	-	-	-	-
gALL_v3_c14931	2	3	-	-	-	-	-
gALL_v3_c14932	4	7	-	-	-	-	-
gALL_v3_c14933	5	3	gnl|CDD|193291	pfam12815, CTD, Spt5 C-termi	gb|EFZ17213.1|	hypothetical protein SINV_14267 [Solenopsis invicta]	3,00E-94

gALL_v3_c14934	6	9	gnl|CDD|191431	pfam06025, DUF913, Domain	gb|EFX86378.1|	hypothetical protein DAPPUDRAFT_313219 [Daphnia pulex]	0
gALL_v3_c14935	2	6	gnl|CDD|191288	pfam05499, DMAP1, DNA met	gb|AGM32673.1|	DNA methyltransferase 1-associated protein 1-like protein, partial [Coptotermes formosanus]	1,00E-98
gALL_v3_c14936	3	3	-	-	-	-	-
gALL_v3_c14937	2	9	-	-	-	-	-
gALL_v3_c14938	8	11	-	-	-	-	-
gALL_v3_c14939	2	3	-	-	-	-	-
gALL_v3_c1494	5	2	-	-	ref|XP_968052.1|	PREDICTED: similar to CG34013 CG34013-PA [Tribolium castaneum]	1,00E-44
gALL_v3_c14941	3	3	-	-	gb|EFA13594.1|	hypothetical protein TcasGA2_TC001481 [Tribolium castaneum]	2,00E-25
gALL_v3_c14942	3	4	-	-	-	-	-
gALL_v3_c14943	3	2	-	-	ref|XP_002426359.1|	conserved hypothetical protein [Pediculus humanus corporis] gb|EEB13621.1| conserved hypothetical protein [Pediculus humanus corporis]	1,00E-56
gALL_v3_c14945	10	12	-	-	-	-	-
gALL_v3_c14947	2	5	-	-	-	-	-
gALL_v3_c14948	8	6	-	-	-	-	-
gALL_v3_c14949	4	2	gnl|CDD|190271	pfam02296, Alpha_adaptin_C,	ref|XP_003491577.1|	PREDICTED: AP-2 complex subunit alpha-like [Bombus impatiens]	3,00E-97
gALL_v3_c1495	6	9	-	-	-	-	-
gALL_v3_c14950	1	5	-	-	ref|XP_003703474.1|	PREDICTED: Golgi SNAP receptor complex member 1-like [Megachile rotundata]	8,00E-27
gALL_v3_c14951	4	4	gnl|CDD|99929	cd05497, Bromo_Brdt_I_like, I	ref|XP_003399205.1|	PREDICTED: bromodomain-containing protein 2-like [Bombus terrestris]	3,00E-75
gALL_v3_c14952	3	3	-	-	-	-	-
gALL_v3_c14953	6	4	gnl|CDD|189373	pfam00069, Pkinase, Protein k	ref|XP_003703950.1|	PREDICTED: activin receptor type-2B-like [Megachile rotundata]	0
gALL_v3_c14954	3	3	gnl|CDD|58540	cd03470, Rieske_cytochrome_	gb|AAV41428.1|	Rieske Fe-S protein [Periplaneta americana]	2,00E-33
gALL_v3_c14955	7	4	-	-	ref|XP_002427724.1|	conserved hypothetical protein [Pediculus humanus corporis] gb|EEB14986.1| conserved hypothetical protein [Pediculus humanus corporis]	1,00E-125
gALL_v3_c14956	4	7	gnl|CDD|145817	pfam02864, STAT_bind, STAT p	ref|XP_003705899.1|	PREDICTED: signal transducer and activator of transcription 5B-like [Megachile rotundata]	1,00E-132
gALL_v3_c14957	8	4	gnl|CDD|192275	pfam09380, FERM_C, FERM C-	ref|XP_003246718.1|	PREDICTED: band 4.1-like protein 5-like [Acyrthosiphon pisum]	2,00E-76
gALL_v3_c14959	3	1	-	-	ref|XP_004207399.1|	PREDICTED: uncharacterized protein LOC101234583 [Hydra vulgaris]	5,00E-16
gALL_v3_c1496	8	10	-	-	-	-	-
gALL_v3_c14960	4	6	-	-	-	-	-
gALL_v3_c14962	2	4	gnl|CDD|132729	cd06931, NR_LBD_HNF4_like,	gb|EFA07427.1|	hepatocyte nuclear factor 4 [Tribolium castaneum]	8,00E-64
gALL_v3_c14963	7	12	gnl|CDD|189372	pfam00067, p450, Cytochrome	dbj|BAD84176.1|	cytochrome P450 [Hodotermopsis sjostedti]	7,00E-39
gALL_v3_c14964	1	3	-	-	-	-	-
gALL_v3_c14965	3	1	-	-	gb|AGM32679.1|	uncharacterized protein [Coptotermes formosanus]	2,00E-78
gALL_v3_c14966	3	3	-	-	ref|XP_006565355.1|	PREDICTED: teneurin-m isoform X1 [Apis mellifera]	1,00E-92
gALL_v3_c14967	3	3	-	-	ref|XP_003393830.1|	PREDICTED: solute carrier family 41 member 2-like [Bombus terrestris]	2,00E-66
gALL_v3_c14968	3	2	-	-	-	-	-
gALL_v3_c14969	2	7	-	-	-	-	-
gALL_v3_c14971	3	1	-	-	ref|XP_006823726.1|	PREDICTED: exportin-6-like [Saccoglossus kowalevskii]	8,00E-21
gALL_v3_c14972	2	3	-	-	ref|XP_002430318.1|	multiple ankyrin repeats single kh domain protein, putative [Pediculus humanus corporis] gb|EEB17580.1| multiple ankyrin repeats single kh domain protein, putative [Pec	9,00E-08
gALL_v3_c14973	2	3	-	-	-	-	-
gALL_v3_c14974	1	8	-	-	-	-	-
gALL_v3_c14975	8	5	gnl|CDD|144680	pfam01175, Urocanase, Uroca	ref|XP_005522310.1|	PREDICTED: urocanate hydratase [Pseudopodoces humilis]	1,00E-29
gALL_v3_c14976	5	5	-	-	gb|AGM32926.1|	hypothetical protein [Coptotermes formosanus]	2,00E-09
gALL_v3_c14977	1	4	-	-	-	-	-
gALL_v3_c14978	3	2	-	-	-	-	-
gALL_v3_c14979	0	4	-	-	-	-	-
gALL_v3_c1498	13	16	gnl|CDD|191348	pfam05705, DUF829, Eukaryot	gb|AGM32306.1|	eukaryotic protein of unknown function, partial [Coptotermes formosanus]	6,00E-88
gALL_v3_c14982	3	1	-	-	-	-	-
gALL_v3_c14984	6	13	-	-	-	-	-
gALL_v3_c14985	2	5	-	-	-	-	-
gALL_v3_c14986	5	3	-	-	ref|XP_002423636.1|	conserved hypothetical protein [Pediculus humanus corporis] gb|EEB10898.1| conserved hypothetical protein [Pediculus humanus corporis]	3,00E-66
gALL_v3_c14987	3	2	-	-	-	-	-
gALL_v3_c14988	3	1	-	-	-	-	-
gALL_v3_c14989	3	2	gnl|CDD|143429	cd07111, ALDH_F16, Aldehyde	ref|XP_797815.1|	PREDICTED: aldehyde dehydrogenase family 16 member A1-like [Strongylocentrotus purpuratus]	3,00E-22
gALL_v3_c1499	9	11	-	-	gb|EFZ14891.1|	hypothetical protein SINV_05158 [Solenopsis invicta]	2,00E-11
gALL_v3_c14990	2	3	-	-	-	-	-
gALL_v3_c14991	1	4	-	-	-	-	-
gALL_v3_c14992	2	4	-	-	-	-	-
gALL_v3_c14993	5	3	-	-	-	-	-
gALL_v3_c14994	1	5	-	-	-	-	-
gALL_v3_c14995	4	6	-	-	-	-	-
gALL_v3_c14997	3	1	-	-	-	-	-
gALL_v3_c14998	4	3	-	-	ref|XP_003731101.1|	PREDICTED: putative nuclease HARBI1-like [Strongylocentrotus purpuratus]	3,00E-44
gALL_v3_c14999	4	7	-	-	-	-	-
gALL_v3_c15	8	11	gnl|CDD|189372	pfam00067, p450, Cytochrome	dbj|BAD84176.1|	cytochrome P450 [Hodotermopsis sjostedti]	1,00E-153
gALL_v3_c1500	5	9	-	-	-	-	-
gALL_v3_c15000	2	7	-	-	-	-	-
gALL_v3_c15002	3	4	-	-	ref|XP_003243437.1|	PREDICTED: hypothetical protein LOC100572891 [Acyrthosiphon pisum]	7,00E-07
gALL_v3_c15004	8	7	-	-	-	-	-
gALL_v3_c15005	0	5	gnl|CDD|109297	pfam00233, PDEase_I, 3'5'-cyc	gb|EGI67624.1|	Dual 3',5'-cyclic-AMP and -GMP phosphodiesterase 11 [Acromyrmex echinatior]	6,00E-71
gALL_v3_c15006	7	7	gnl|CDD|115470	pfam06814, Lung_7-TM_R, Lu	ref|XP_003708062.1|	PREDICTED: protein GPR107-like [Megachile rotundata]	1,00E-164
gALL_v3_c15007	3	3	-	-	-	-	-
gALL_v3_c15008	4	2	-	-	-	-	-
gALL_v3_c15009	10	15	-	-	-	-	-
gALL_v3_c15010	2	4	-	-	-	-	-
gALL_v3_c15011	4	2	-	-	-	-	-
gALL_v3_c15012	1	6	-	-	-	-	-
gALL_v3_c15013	3	1	-	-	-	-	-
gALL_v3_c15014	4	6	gnl|CDD|152466	pfam12031, DUF3518, Domain	gb|EJY57697.1|	AAEL017280-PA [Aedes aegypti]	3,00E-68
gALL_v3_c15015	8	5	-	-	emb|CDH88817.1|	Ankyrin repeat domain protein [Wolbachia endosymbiont wPip_Mol of Culex molestus]	6,00E-13
gALL_v3_c15016	6	10	gnl|CDD|128983	smart00744, RINGv, The RING-	gb|EFN68890.1|	E3 ubiquitin-protein ligase MARCH5 [Camponotus floridanus]	1,00E-152
gALL_v3_c15017	3	5	-	-	-	-	-
gALL_v3_c15019	7	8	-	-	-	-	-
gALL_v3_c1502	4	11	-	-	-	-	-
gALL_v3_c15020	7	7	gnl|CDD|177889	PLN02246, PLN02246, 4-coum	ref|XP_003436222.1|	AGAP002718-PB [Anopheles gambiae str. PEST] gb|EGK96791.1| AGAP002718-PB [Anopheles gambiae str. PEST]	1,00E-113
gALL_v3_c15021	3	1	-	-	-	-	-
gALL_v3_c15022	3	5	-	-	-	-	-
gALL_v3_c15025	2	5	-	-	ref|XP_974036.1|	PREDICTED: similar to Tsc1 CG6147-PA [Tribolium castaneum] gb|EFA00138.1| hypothetical protein TcasGA2_TC002959 [Tribolium castaneum]	4,00E-09
gALL_v3_c15026	3	1	-	-	-	-	-
gALL_v3_c15028	2	3	-	-	-	-	-
gALL_v3_c15029	2	3	-	-	-	-	-
gALL_v3_c1503	5	7	-	-	-	-	-
gALL_v3_c15030	1	4	-	-	ref|XP_001605722.2|	PREDICTED: afadin-like [Nasonia vitripennis]	1,00E-28
gALL_v3_c15032	7	15	-	-	ref|XP_003706303.1|	PREDICTED: kinesin-like protein KIF3A-like [Megachile rotundata]	3,00E-56
gALL_v3_c15033	1	3	-	-	-	-	-
gALL_v3_c15034	4	7	gnl|CDD|29115	cd00170, SEC14, Sec14p-like li	ref|XP_001654466.1|	SEC14, putative [Aedes aegypti] gb|EAT37701.1| AAEL010344-PA [Aedes aegypti]	2,00E-44
gALL_v3_c15035	7	6	gnl|CDD|162313	TIGR01351, adk, adenylate kin	dbj|BAN20862.1|	adenylate kinase 3 [Riptortus pedestris]	1,00E-43
gALL_v3_c15036	6	6	-	-	-	-	-
gALL_v3_c15037	3	2	-	-	-	-	-
gALL_v3_c15039	2	3	gnl|CDD|192369	pfam09764, WDYHV, Uncharac	ref|XP_002423524.1|	conserved hypothetical protein [Pediculus humanus corporis] gb|EEB10786.1| conserved hypothetical protein [Pediculus humanus corporis]	3,00E-53
gALL_v3_c15040	2	4	-	-	-	-	-
gALL_v3_c15044	2	7	gnl|CDD|143945	pfam00179, UQ_con, Ubiquiti	gb|EHJ64632.1|	putative ubiquitin-conjugating enzyme E2 g [Danaus plexippus]	2,00E-61
gALL_v3_c15045	5	6	gnl|CDD|119422	cd00896, PI3Kc_III, Phosphoin	ref|XP_003703303.1|	PREDICTED: phosphatidylinositol 3-kinase catalytic subunit type 3-like isoform 2 [Megachile rotundata]	1,00E-96
gALL_v3_c15046	2	3	-	-	-	-	-
gALL_v3_c15049	5	7	gnl|CDD|189654	pfam00651, BTB, BTB/POZ dor	ref|XP_001812349.1|	PREDICTED: similar to AGAP006454-PA [Tribolium castaneum]	3,00E-63
gALL_v3_c1505	6	17	-	-	gb|EGI62320.1|	Protein furry-like protein-like protein [Acromyrmex echinatior]	1,00E-83
gALL_v3_c15050	3	2	-	-	-	-	-
gALL_v3_c15051	4	9	-	-	-	-	-
gALL_v3_c15052	2	4	-	-	-	-	-
gALL_v3_c15053	1	3	-	-	-	-	-
gALL_v3_c15054	3	1	-	-	-	-	-
gALL_v3_c15055	6	16	-	-	ref|XP_002427116.1|	hypothetical protein Phum_PHUM296360 [Pediculus humanus corporis] gb|EEB14378.1| hypothetical protein Phum_PHUM296360 [Pediculus humanus corporis]	6,00E-26
gALL_v3_c15056	2	3	-	-	-	-	-
gALL_v3_c15058	0	4	-	-	gb|EGI57505.1|	Putative ATP-dependent RNA helicase DHX35 [Acromyrmex echinatior]	6,00E-30
gALL_v3_c15059	0	5	-	-	-	-	-
gALL_v3_c15060	4	4	-	-	-	-	-
gALL_v3_c15062	4	6	-	-	ref|XP_003488381.1|	PREDICTED: hypothetical protein LOC100746491 [Bombus impatiens]	2,00E-66
gALL_v3_c15063	5	9	gnl|CDD|143200	cd05723, Ig4_Neogenin, Fourt	gb|EFX79705.1|	hypothetical protein DAPPUDRAFT_197333 [Daphnia pulex]	1,00E-150
gALL_v3_c15064	2	5	-	-	gb|EGI61189.1|	FLJ37770-like protein [Acromyrmex echinatior]	4,00E-06
gALL_v3_c15065	4	3	gnl|CDD|189372	pfam00067, p450, Cytochrome	ref|XP_002424254.1|	cytochrome P450, putative [Pediculus humanus corporis] gb|EEB11516.1| cytochrome P450, putative [Pediculus humanus corporis]	1,00E-34
gALL_v3_c15067	1	7	-	-	ref|XP_972752.1|	PREDICTED: similar to transglutaminase [Tribolium castaneum] gb|EFA03506.1| hypothetical protein TcasGA2_TC013507 [Tribolium castaneum]	4,00E-13
gALL_v3_c15068	4	3	gnl|CDD|188051	TIGR00446, nop2p, NOL1/NOP	ref|XP_974410.1|	PREDICTED: similar to CG8545 CG8545-PA [Tribolium castaneum] gb|EFA09757.1| hypothetical protein TcasGA2_TC011896 [Tribolium castaneum]	1,00E-149
gALL_v3_c15069	4	2	gnl|CDD|176484	cd08366, APC10, APC10 subur	gb|AGM32731.1|	APC10 subunit of the anaphase-promoting complex (APC) [Coptotermes formosanus]	1,00E-74
gALL_v3_c1507	5	8	-	-	-	-	-
gALL_v3_c15070	1	4	-	-	-	-	-
gALL_v3_c15072	3	2	-	-	-	-	-
gALL_v3_c15074	2	4	-	-	-	-	-
gALL_v3_c15075	3	1	-	-	gb|AAL78751.1|	translation elongation factor-1 gamma [Locusta migratoria]	8,00E-09
gALL_v3_c15076	5	2	gnl|CDD|31276	COG1078, COG1078, HD super	ref|XP_001950657.1|	PREDICTED: SAM domain and HD domain-containing protein 1-like [Acyrthosiphon pisum]	2,00E-72
gALL_v3_c15078	5	5	-	-	-	-	-
gALL_v3_c15079	0	6	-	-	-	-	-
gALL_v3_c1508	6	7	-	-	gb|EGI60155.1|	hypothetical protein G5I_11697 [Acromyrmex echinatior]	8,00E-22
gALL_v3_c15080	1	3	-	-	-	-	-
gALL_v3_c15082	12	15	gnl|CDD|173660	cd05123, STKc_AGC, Catalytic	ref|XP_004920206.1|	PREDICTED: protein kinase C theta type-like, partial [Xenopus (Silurana) tropicalis]	4,00E-10
gALL_v3_c15083	7	11	-	-	-	-	-
gALL_v3_c15084	9	5	gnl|CDD|119351	cd02872, GH18_chitolectin_ch	gb|AHB39935.1|	chitinase 2 [Coptotermes formosanus]	1,00E-111
gALL_v3_c15086	2	2	-	-	-	-	-
gALL_v3_c15087	2	2	-	-	-	-	-
gALL_v3_c15088	4	5	-	-	gb|EFA05094.1|	hypothetical protein TcasGA2_TC015192 [Tribolium castaneum]	1,00E-110
gALL_v3_c15089	3	11	gnl|CDD|63852	cd04383, RhoGAP_srGAP, Rho	ref|XP_002424891.1|	SLIT-ROBO Rho GTPase-activating protein, putative [Pediculus humanus corporis] gb|EEB12153.1| SLIT-ROBO Rho GTPase-activating protein, putative [Pediculus humanus	2,00E-89
gALL_v3_c1509	21	28	-	-	-	-	-
gALL_v3_c15090	0	5	-	-	-	-	-
gALL_v3_c15091	5	2	-	-	gb|EFN67634.1|	Putative uncharacterized transposon-derived protein F52C9.6 [Camponotus floridanus]	7,00E-07
gALL_v3_c15092	2	3	-	-	-	-	-
gALL_v3_c15093	3	2	-	-	-	-	-
gALL_v3_c15094	2	3	-	-	-	-	-
gALL_v3_c15095	4	7	-	-	ref|XP_002059628.1|	GJ14718 [Drosophila virilis] gb|EDW63050.1| GJ14718 [Drosophila virilis]	3,00E-18

gALL_v3_c15096	3	1	-	-	-	-	-
gALL_v3_c15097	2	4	-	-	-	-	-
gALL_v3_c15098	4	5	-	-	-	-	-
gALL_v3_c15099	1	5	-	-	-	-	-
gALL_v3_c151	7	20	-	-	-	-	-
gALL_v3_c1510	8	5	-	-	-	-	-
gALL_v3_c15100	3	7	gnl|CDD|162464	TIGR01649, hnRNP-L_PTB, hnF	gb|EFN89615.1|	Heterogeneous nuclear ribonucleoprotein L [Harpegnathos saltator]	1,00E-37
gALL_v3_c15101	3	2	gnl|CDD|162130	TIGR00955, 3a01204, The Eye	dbj|BAM20309.1|	white protein, partial [Papilio xuthus]	1,00E-83
gALL_v3_c15102	17	13	gnl|CDD|189464	pfam00235, Profilin, Profilin.	gb|AEE61626.1|	unknown [Dendroctonus ponderosae]	8,00E-82
gALL_v3_c15103	4	2	-	-	-	-	-
gALL_v3_c15104	4	4	-	-	-	-	-
gALL_v3_c15105	6	3	gnl|CDD|190050	pfam01602, Adaptin_N, Adapt	gb|ENN74993.1|	hypothetical protein YQE_08450, partial [Dendroctonus ponderosae]	1,00E-176
gALL_v3_c15106	7	8	gnl|CDD|73156	cd01650, RT_nLTR_like, RT_nL	gb|ADI61823.1|	endonuclease-reverse transcriptase [Bombyx mori]	7,00E-19
gALL_v3_c15107	1	4	gnl|CDD|190438	pfam02815, MIR, MIR domain	gb|EFX71511.1|	hypothetical protein DAPPUDRAFT_227934 [Daphnia pulex]	5,00E-93
gALL_v3_c15108	2	5	-	-	gb|EFN67634.1|	Putative uncharacterized transposon-derived protein F52C9.6 [Camponotus floridanus]	1,00E-07
gALL_v3_c15109	1	4	gnl|CDD|73080	cd02674, Peptidase_C19R, A s	ref|XP_002427486.1|	ubiquitin carboxyl-terminal hydrolase, putative [Pediculus humanus corporis] gb|EEB14748.1| ubiquitin carboxyl-terminal hydrolase, putative [Pediculus humanus corporis	6,00E-54
gALL_v3_c1511	11	15	gnl|CDD|162813	TIGR02347, chap_CCT_zeta, T-	gb|AHB33471.1|	T-complex protein subunit zeta [Locusta migratoria]	1,00E-173
gALL_v3_c15110	3	4	gnl|CDD|29257	cd00200, WD40, WD40 domai	gb|EFN67612.1|	WD repeat-containing protein 26 [Camponotus floridanus]	2,00E-99
gALL_v3_c15111	3	3	-	-	-	-	-
gALL_v3_c15112	5	3	-	-	-	-	-
gALL_v3_c15113	3	1	gnl|CDD|185447	PTZ00103, PTZ00103, 60S ribo	ref|XP_001584492.1|	hypothetical protein [Trichomonas vaginalis G3] gb|EAY23506.1| hypothetical protein TVAG_071700 [Trichomonas vaginalis G3]	2,00E-90
gALL_v3_c15114	3	5	-	-	ref|XP_393414.4|	PREDICTED: leishmanolysin-like peptidase [Apis mellifera]	2,00E-76
gALL_v3_c15115	3	3	-	-	-	-	-
gALL_v3_c15117	4	2	-	-	ref|XP_003398691.1|	PREDICTED: eukaryotic peptide chain release factor GTP-binding subunit ERF3B-like [Bombus terrestris]	5,00E-09
gALL_v3_c15118	7	8	-	-	-	-	-
gALL_v3_c15119	3	4	-	-	-	-	-
gALL_v3_c1512	19	21	gnl|CDD|153403	cd08034, LARP_1_2, La RNA-b	ref|XP_001605854.2|	PREDICTED: la-related protein 1-like [Nasonia vitripennis]	0
gALL_v3_c15120	8	7	-	-	ref|XP_003700818.1|	PREDICTED: sodium-dependent neutral amino acid transporter SLC6A17-like isoform 2 [Megachile rotundata]	9,00E-37
gALL_v3_c15121	3	2	-	-	-	-	-
gALL_v3_c15122	3	2	-	-	ref|XP_002427617.1|	conserved hypothetical protein [Pediculus humanus corporis] gb|EEB14879.1| conserved hypothetical protein [Pediculus humanus corporis]	4,00E-15
gALL_v3_c15123	4	7	-	-	-	-	-
gALL_v3_c15124	7	6	-	-	ref|XP_003417964.1|	PREDICTED: lisH domain-containing protein ARMC9-like [Loxodonta africana]	3,00E-09
gALL_v3_c15125	6	7	-	-	-	-	-
gALL_v3_c15126	3	5	-	-	ref|XP_001600680.2|	PREDICTED: prolyl 4-hydroxylase subunit alpha-2-like [Nasonia vitripennis]	2,00E-56
gALL_v3_c15127	1	5	-	-	-	-	-
gALL_v3_c15128	1	4	-	-	-	-	-
gALL_v3_c15129	3	2	-	-	-	-	-
gALL_v3_c1513	3	10	-	-	-	-	-
gALL_v3_c15130	3	2	-	-	-	-	-
gALL_v3_c15131	3	2	-	-	-	-	-
gALL_v3_c15132	1	5	gnl|CDD|187665	cd09805, type2_17beta_HSD-	ref|XP_002612557.1|	3-hydroxybutyrate dehydrogenase [Branchiostoma floridae] gb|EEN68566.1| 3-hydroxybutyrate dehydrogenase [Branchiostoma floridae]	1,00E-35
gALL_v3_c15133	5	2	-	-	-	-	-
gALL_v3_c15134	0	6	-	-	-	-	-
gALL_v3_c15135	2	3	-	-	ref|WP_009610708.1|	hypothetical protein [Caldanaerobacter subterraneus] gb|EEB75630.1| hypothetical protein CDSM653_949 [Carboxydibrachium pacificum DSM 12653]	2,00E-19
gALL_v3_c15136	2	2	-	-	-	-	-
gALL_v3_c15137	5	8	-	-	-	-	-
gALL_v3_c15138	2	5	-	-	-	-	-
gALL_v3_c15139	3	2	-	-	ref|XP_006564665.1|	PREDICTED: HMG box-containing protein 4-like isoform X2 [Apis mellifera]	8,00E-45
gALL_v3_c1514	6	11	-	-	gb|EFN67634.1|	Putative uncharacterized transposon-derived protein F52C9.6 [Camponotus floridanus]	8,00E-49
gALL_v3_c15141	3	5	-	-	-	-	-
gALL_v3_c15142	4	5	gnl|CDD|190188	pfam01997, Translin, Translin	ref|XP_003425818.1|	PREDICTED: translin-associated protein X-like [Nasonia vitripennis]	1,00E-57
gALL_v3_c15143	2	4	-	-	-	-	-
gALL_v3_c15144	2	4	gnl|CDD|178765	PLN03226, PLN03226, serine h	dbj|BAM17968.1|	glycine hydroxylmethyltransferase [Papilio xuthus]	2,00E-83
gALL_v3_c15145	4	3	-	-	-	-	-
gALL_v3_c15147	3	3	-	-	-	-	-
gALL_v3_c15148	2	9	gnl|CDD|162372	TIGR01458, HAD-SF-IIA-hyp3,	gb|EGI67079.1|	Haloacid dehalogenase-like hydrolase domain-containing protein 2 [Acromyrmex echinatior]	4,00E-69
gALL_v3_c15149	5	9	gnl|CDD|190293	pfam02373, JmjC, JmjC domai	ref|XP_003702264.1|	PREDICTED: lysine-specific demethylase lid-like isoform 2 [Megachile rotundata]	0
gALL_v3_c1515	18	25	-	-	-	-	-
gALL_v3_c15151	5	4	gnl|CDD|128564	smart00268, ACTIN, Actin. AC	ref|XP_002424614.1|	conserved hypothetical protein [Pediculus humanus corporis] gb|EEB11876.1| conserved hypothetical protein [Pediculus humanus corporis]	1,00E-173
gALL_v3_c15152	3	8	gnl|CDD|147872	pfam05955, Herpes_gp2, Equi	ref|XP_001751006.1|	hypothetical protein [Monosiga brevicollis MX1] gb|EDQ84176.1| predicted protein [Monosiga brevicollis MX1]	3,00E-12
gALL_v3_c15153	8	5	-	-	gb|EFN65619.1|	Putative uncharacterized transposon-derived protein F52C9.6 [Camponotus floridanus]	6,00E-11
gALL_v3_c15155	5	4	-	-	ref|XP_969441.1|	PREDICTED: similar to tpr repeat nuclear phosphoprotein [Tribolium castaneum] gb|EEZ99122.1| hypothetical protein TcasGA2_TC005228 [Tribolium castaneum]	1,00E-140
gALL_v3_c15156	3	3	-	-	ref|XP_001605902.2|	PREDICTED: GTPase-activating protein and VPS9 domain-containing protein 1-like [Nasonia vitripennis]	4,00E-53
gALL_v3_c15157	1	3	-	-	-	-	-
gALL_v3_c15158	0	3	-	-	-	-	-
gALL_v3_c15159	8	4	-	-	gb|EGI65557.1|	SET and MYND domain-containing protein 4 [Acromyrmex echinatior]	7,00E-43
gALL_v3_c1516	5	7	-	-	-	-	-
gALL_v3_c15160	5	3	-	-	-	-	-
gALL_v3_c15161	5	10	-	-	-	-	-
gALL_v3_c15162	3	3	-	-	-	-	-
gALL_v3_c15163	2	3	-	-	-	-	-
gALL_v3_c15164	3	3	gnl|CDD|30291	cd00585, Peptidase_C1B, Pept	emb|CAJ12161.1|	cg1440 protein [Drosophila simulans]	5,00E-82
gALL_v3_c15165	3	3	gnl|CDD|133358	cd04158, ARD1, ARD1 subfam	ref|XP_001952583.1|	PREDICTED: e3 ubiquitin-protein ligase TRIM23-like [Acyrthosiphon pisum]	1,00E-120
gALL_v3_c15166	5	2	-	-	-	-	-
gALL_v3_c15168	2	7	-	-	-	-	-
gALL_v3_c15170	3	4	-	-	-	-	-
gALL_v3_c15171	4	6	-	-	ref|XP_975583.1|	PREDICTED: similar to mitochondrial oxodicarboxylate carrier [Tribolium castaneum] gb|EFA02491.1| hypothetical protein TcasGA2_TC008186 [Tribolium castaneum]	5,00E-55
gALL_v3_c15172	2	3	-	-	-	-	-
gALL_v3_c15173	2	8	-	-	-	-	-
gALL_v3_c15174	6	4	-	-	ref|XP_003242319.1|	PREDICTED: histone-lysine N-methyltransferase SETMAR-like [Acyrthosiphon pisum]	1,00E-19
gALL_v3_c15175	3	8	-	-	-	-	-
gALL_v3_c15176	4	3	-	-	gb|EFZ14406.1|	hypothetical protein SINV_08300 [Solenopsis invicta]	2,00E-59
gALL_v3_c15177	4	3	gnl|CDD|28922	cd00041, CUB, CUB domain; e	ref|XP_003691728.1|	PREDICTED: LOW QUALITY PROTEIN: cubilin-like [Apis florea]	9,00E-28
gALL_v3_c15178	3	9	-	-	-	-	-
gALL_v3_c15179	1	5	-	-	ref|XP_003700110.1|	PREDICTED: LOW QUALITY PROTEIN: COP9 signalosome complex subunit 1-like [Megachile rotundata]	2,00E-39
gALL_v3_c1518	8	13	-	-	-	-	-
gALL_v3_c15180	0	5	-	-	gb|EFN77235.1|	Probable phospholipid-transporting ATPase ID [Harpegnathos saltator]	5,00E-21
gALL_v3_c15181	2	2	-	-	-	-	-
gALL_v3_c15182	4	7	-	-	-	-	-
gALL_v3_c15184	3	2	-	-	-	-	-
gALL_v3_c15185	3	1	-	-	-	-	-
gALL_v3_c15186	4	12	gnl|CDD|191810	pfam07679, I-set, Immunoglol	gb|ETN66453.1|	Unc-89 protein [Anopheles darlingi]	8,00E-38
gALL_v3_c15187	6	8	gnl|CDD|128593	smart00298, CHROMO, Chrom	ref|XP_003487128.1|	PREDICTED: hypothetical protein LOC100747718 [Bombus impatiens]	1,00E-28
gALL_v3_c15188	2	5	-	-	-	-	-
gALL_v3_c15189	3	1	-	-	-	-	-
gALL_v3_c15190	1	7	-	-	gb|ADA70416.1|	MIP15322p [Drosophila melanogaster]	3,00E-10
gALL_v3_c15191	3	4	-	-	-	-	-
gALL_v3_c15192	2	3	-	-	gb|AGM32926.1|	hypothetical protein [Coptotermes formosanus]	9,00E-09
gALL_v3_c15193	4	6	-	-	-	-	-
gALL_v3_c15194	4	13	-	-	-	-	-
gALL_v3_c15195	17	18	-	-	-	-	-
gALL_v3_c15197	2	3	-	-	-	-	-
gALL_v3_c15198	2	3	-	-	-	-	-
gALL_v3_c15199	6	3	-	-	-	-	-
gALL_v3_c15201	2	6	-	-	-	-	-
gALL_v3_c15202	1	6	-	-	-	-	-
gALL_v3_c15204	5	2	-	-	ref|XP_006825971.1|	PREDICTED: uncharacterized protein LOC100368918 [Saccoglossus kowalevskii]	1,00E-23
gALL_v3_c15205	2	2	-	-	ref|XP_004375662.1|	PREDICTED: DNA-dependent protein kinase catalytic subunit isoform 2 [Trichechus manatus latirostris]	4,00E-23
gALL_v3_c15206	1	4	-	-	-	-	-
gALL_v3_c15207	3	8	-	-	gb|EFN78871.1|	hypothetical protein EAI_02279 [Harpegnathos saltator]	2,00E-63
gALL_v3_c15208	3	11	-	-	-	-	-
gALL_v3_c15209	2	2	-	-	-	-	-
gALL_v3_c1521	17	16	-	-	-	-	-
gALL_v3_c15210	4	7	gnl|CDD|111117	pfam02187, GAS2, Growth-Ar	ref|XP_002425686.1|	hypothetical protein Phum_PHUM215460 [Pediculus humanus corporis] gb|EEB12948.1| hypothetical protein Phum_PHUM215460 [Pediculus humanus corporis]	3,00E-19
gALL_v3_c15211	4	2	-	-	-	-	-
gALL_v3_c15212	2	4	-	-	-	-	-
gALL_v3_c15213	4	4	gnl|CDD|192151	pfam08781, DP, Transcription	gb|EFZ09432.1|	hypothetical protein SINV_05415 [Solenopsis invicta]	1,00E-72
gALL_v3_c15214	6	3	-	-	-	-	-
gALL_v3_c15216	3	5	-	-	ref|XP_003491276.1|	PREDICTED: hypothetical protein LOC100746742 [Bombus impatiens]	4,00E-32
gALL_v3_c15217	6	8	-	-	-	-	-
gALL_v3_c15218	2	3	-	-	-	-	-
gALL_v3_c15219	1	5	gnl|CDD|190520	pfam03095, PTPA, Phosphotyr	ref|XP_003706092.1|	PREDICTED: serine/threonine-protein phosphatase 2A activator-like [Megachile rotundata]	2,00E-69
gALL_v3_c1522	17	20	-	-	-	-	-
gALL_v3_c15220	6	9	-	-	-	-	-
gALL_v3_c15221	5	6	-	-	ref|XP_003697045.1|	PREDICTED: uncharacterized protein LOC100865209 [Apis florea]	1,00E-117
gALL_v3_c15223	3	1	-	-	-	-	-
gALL_v3_c15224	3	3	-	-	gb|AGM32083.1|	hypothetical protein [Coptotermes formosanus]	7,00E-08
gALL_v3_c15225	5	3	-	-	ref|XP_002406504.1|	hypothetical protein IscW_ISCW003471 [Ixodes scapularis] gb|EEC06535.1| hypothetical protein IscW_ISCW003471 [Ixodes scapularis]	7,00E-15
gALL_v3_c15226	3	10	-	-	-	-	-
gALL_v3_c15227	5	6	-	-	-	-	-
gALL_v3_c15228	4	3	-	-	-	-	-
gALL_v3_c1523	9	20	gnl|CDD|189434	pfam00176, SNF2_N, SNF2 far	ref|XP_001948282.2|	PREDICTED: hypothetical protein LOC100164870 [Acyrthosiphon pisum]	0
gALL_v3_c15230	3	5	-	-	gb|EFN68378.1|	Calpain-A [Camponotus floridanus]	5,00E-56
gALL_v3_c15231	4	2	-	-	ref|XP_004927888.1|	PREDICTED: 97 kDa heat shock protein-like [Bombyx mori]	7,00E-21
gALL_v3_c15232	3	2	-	-	-	-	-
gALL_v3_c15234	2	5	gnl|CDD|177219	MTH00163, ND4, NADH dehyc	gb|ACU42721.1|	NADH dehydrogenase subunit 4 [Cryptocercus sp. BL120]	1,00E-11
gALL_v3_c15235	6	3	-	-	-	-	-
gALL_v3_c15236	1	5	-	-	-	-	-
gALL_v3_c15238	2	3	-	-	-	-	-
gALL_v3_c15239	2	4	-	-	gb|EFN65870.1|	E3 ubiquitin-protein ligase HUWE1 [Camponotus floridanus]	1,00E-13
gALL_v3_c1524	11	12	-	-	gb|ERL83407.1|	hypothetical protein D910_00366 [Dendroctonus ponderosae] gb|ERL83408.1| hypothetical protein D910_00367 [Dendroctonus ponderosae]	1,00E-178
gALL_v3_c15240	2	5	gnl|CDD|179265	PRK01259, PRK01259, ribose-p	gb|EHJ76166.1|	ribose-phosphate pyrophosphokinase 1,2 [Danaus plexippus]	5,00E-80
gALL_v3_c15241	2	2	-	-	gb|EFN64083.1|	Sorbin and SH3 domain-containing protein 1 [Camponotus floridanus]	4,00E-22
gALL_v3_c15242	5	4	-	-	ref|WP_004846157.1|	hypothetical protein [[Ruminococcus] torques] gb|EDK23019.1| hypothetical protein RUMTOR_02824 [Ruminococcus torques ATCC 27756]	4,00E-49

gALL_v3_c15243	11	11	gnl|CDD|177214	MTH00157, ATP6, ATP synthas	ref|YP_002261396.1|	cytochrome c oxidase subunit III [Troglophilus neglectus] gb|ACG59340.1| cytochrome c oxidase subunit III [Troglophilus neglectus]	3,00E-65
gALL_v3_c15244	0	5	-	-	-	-	-
gALL_v3_c15245	1	4	gnl|CDD|192221	pfam09174, Maf1, Maf1 regul	ref|XP_001600371.1|	PREDICTED: repressor of RNA polymerase III transcription MAF1 homolog [Nasonia vitripennis]	1,00E-84
gALL_v3_c15246	3	5	-	-	-	-	-
gALL_v3_c15247	3	5	-	-	-	-	-
gALL_v3_c15248	5	9	-	-	gb|ADI61823.1|	endonuclease-reverse transcriptase [Bombyx mori]	1,00E-06
gALL_v3_c15249	5	4	-	-	emb|CCA74892.1|	hypothetical protein PIIN_08862 [Piriformospora indica DSM 11827]	2,00E-97
gALL_v3_c1525	25	19	gnl|CDD|189569	pfam00487, FA_desaturase, Fa	ref|XP_975884.1|	PREDICTED: similar to CG17928 CG17928-PA isoform 2 [Tribolium castaneum] gb|EFA08577.1| hypothetical protein TcasGA2_TC006232 [Tribolium castaneum]	1,00E-96
gALL_v3_c15250	3	1	gnl|CDD|144493	pfam00916, Sulfate_transp, Su	ref|XP_971069.1|	PREDICTED: similar to sulfate transporter [Tribolium castaneum] gb|EEZ98816.1| hypothetical protein TcasGA2_TC004422 [Tribolium castaneum]	3,00E-51
gALL_v3_c15252	1	3	-	-	ref|XP_006623737.1|	PREDICTED: uncharacterized protein LOC102681750 [Apis dorsata]	9,00E-18
gALL_v3_c15253	9	6	-	-	gb|EEZ98815.1|	hypothetical protein TcasGA2_TC004421 [Tribolium castaneum]	5,00E-17
gALL_v3_c15254	4	5	-	-	-	-	-
gALL_v3_c15255	4	6	-	-	-	-	-
gALL_v3_c15256	6	4	-	-	-	-	-
gALL_v3_c15257	3	9	-	-	-	-	-
gALL_v3_c15258	2	3	-	-	-	-	-
gALL_v3_c15259	3	3	-	-	ref|XP_004583877.1|	PREDICTED: zinc finger protein 268-like [Ochotona princeps]	6,00E-24
gALL_v3_c1526	18	30	-	-	-	-	-
gALL_v3_c15260	2	5	-	-	-	-	-
gALL_v3_c15261	2	4	gnl|CDD|189821	pfam01055, Glyco_hydro_31,	ref|XP_002423306.1|	conserved hypothetical protein [Pediculus humanus corporis] gb|EEB10568.1| conserved hypothetical protein [Pediculus humanus corporis]	2,00E-54
gALL_v3_c15262	3	1	-	-	-	-	-
gALL_v3_c15263	3	7	-	-	-	-	-
gALL_v3_c15264	3	3	-	-	-	-	-
gALL_v3_c15265	1	4	gnl|CDD|162072	TIGR00860, LIC, Cation transp	gb|ACM50327.1|	GABA receptor Rdl subunit short transcript variant [Periplaneta americana]	1,00E-111
gALL_v3_c15266	2	3	-	-	-	-	-
gALL_v3_c15268	2	6	-	-	-	-	-
gALL_v3_c1527	10	11	-	-	-	-	-
gALL_v3_c15270	3	1	-	-	-	-	-
gALL_v3_c15271	6	6	gnl|CDD|189008	cd09601, M1_APN_2, Peptida	ref|XP_001600617.2|	PREDICTED: glutamyl aminopeptidase-like [Nasonia vitripennis]	1,00E-94
gALL_v3_c15272	2	8	-	-	-	-	-
gALL_v3_c15273	5	16	-	-	-	-	-
gALL_v3_c15274	4	6	-	-	-	-	-
gALL_v3_c15275	1	6	-	-	-	-	-
gALL_v3_c15276	5	4	-	-	dbj|BAC82595.1|	reverse transcriptase [Anopheles gambiae]	6,00E-40
gALL_v3_c15278	1	4	-	-	gb|EFN88817.1|	PiggyBac transposable element-derived protein 4 [Harpegnathos saltator]	1,00E-09
gALL_v3_c15279	1	4	-	-	-	-	-
gALL_v3_c1528	15	19	gnl|CDD|29138	cd00176, SPEC, Spectrin repea	ref|XP_003703682.1|	PREDICTED: spectrin beta chain, brain 4-like [Megachile rotundata]	0
gALL_v3_c15280	3	4	gnl|CDD|176056	cd08521, C2A_SLP, C2 domain	ref|XP_975183.1|	PREDICTED: similar to bitesize CG33555-PC [Tribolium castaneum]	2,00E-47
gALL_v3_c15283	2	3	-	-	-	-	-
gALL_v3_c15284	3	3	-	-	-	-	-
gALL_v3_c15285	2	2	-	-	-	-	-
gALL_v3_c15286	2	3	-	-	-	-	-
gALL_v3_c15287	3	3	-	-	-	-	-
gALL_v3_c15288	2	2	-	-	ref|XP_001623826.1|	predicted protein [Nematostella vectensis] gb|EDO31726.1| predicted protein [Nematostella vectensis]	2,00E-07
gALL_v3_c15289	3	2	-	-	-	-	-
gALL_v3_c1529	4	4	-	-	ref|XP_975329.1|	PREDICTED: similar to bicoid stability factor CG10302-PA [Tribolium castaneum] gb|EFA01919.1| hypothetical protein TcasGA2_TC007529 [Tribolium castaneum]	2,00E-79
gALL_v3_c15290	4	3	gnl|CDD|192138	pfam08736, FA, FERM adjacen	gb|EFN79629.1|	FERM, RhoGEF and pleckstrin domain-containing protein 2 [Harpegnathos saltator]	6,00E-50
gALL_v3_c15291	2	8	gnl|CDD|29761	cd00470, PTPS, 6-pyruvoyl tet	ref|XP_001606725.1|	PREDICTED: 6-pyruvoyl tetrahydrobiopterin synthase-like [Nasonia vitripennis]	1,00E-41
gALL_v3_c15292	1	4	-	-	-	-	-
gALL_v3_c15293	3	2	-	-	ref|XP_006624966.1|	PREDICTED: uncharacterized protein LOC102677138 [Apis dorsata]	2,00E-61
gALL_v3_c15295	5	6	-	-	-	-	-
gALL_v3_c15296	6	4	-	-	-	-	-
gALL_v3_c15297	1	4	-	-	-	-	-
gALL_v3_c15299	3	3	gnl|CDD|191630	pfam06881, Elongin_A, RNA p	ref|XP_003700656.1|	PREDICTED: transcription elongation factor B polypeptide 3-like isoform 2 [Megachile rotundata]	2,00E-70
gALL_v3_c153	8	16	-	-	-	-	-
gALL_v3_c1530	6	11	-	-	gb|EFA08533.1|	hypothetical protein TcasGA2_TC006187 [Tribolium castaneum]	6,00E-33
gALL_v3_c15300	4	4	-	-	-	-	-
gALL_v3_c15301	2	3	-	-	-	-	-
gALL_v3_c15303	1	5	-	-	-	-	-
gALL_v3_c15304	4	7	-	-	-	-	-
gALL_v3_c15305	4	2	-	-	-	-	-
gALL_v3_c15306	3	9	gnl|CDD|153307	cd07623, BAR_SNX1_2, The Bi	-	-	-
gALL_v3_c15307	7	5	-	-	-	-	-
gALL_v3_c15308	7	4	gnl|CDD|193272	pfam12796, Ank_2, Ankyrin re	gb|EHJ68880.1|	hypothetical protein KGM_01470 [Danaus plexippus]	2,00E-10
gALL_v3_c15310	7	12	-	-	-	-	-
gALL_v3_c15311	3	3	-	-	-	-	-
gALL_v3_c15313	7	7	-	-	-	-	-
gALL_v3_c15314	1	4	-	-	-	-	-
gALL_v3_c15315	2	3	-	-	-	-	-
gALL_v3_c15317	2	3	-	-	gb|EGI69284.1|	Huntingtin [Acromyrmex echinatior]	1,00E-42
gALL_v3_c15318	5	6	-	-	ref|XP_001946877.1|	PREDICTED: DNA-directed RNA polymerase, mitochondrial-like [Acyrthosiphon pisum]	1,00E-74
gALL_v3_c15319	0	5	-	-	-	-	-
gALL_v3_c1532	11	19	gnl|CDD|111745	pfam02891, zf-MIZ, MIZ/SP-RI	ref|XP_002424427.1|	restnoic acid-induced protein, putative [Pediculus humanus corporis] gb|EEB11689.1| restnoic acid-induced protein, putative [Pediculus humanus corporis]	0
gALL_v3_c15320	3	2	-	-	-	-	-
gALL_v3_c15321	4	2	-	-	-	-	-
gALL_v3_c15322	3	10	-	-	-	-	-
gALL_v3_c15323	0	4	-	-	-	-	-
gALL_v3_c15324	4	3	-	-	ref|XP_004527553.1|	PREDICTED: phosphatidylinositol phosphatase PTPRQ-like [Ceratitis capitata]	1,00E-08
gALL_v3_c15325	2	2	-	-	dbj|BAN09207.1|	expanded protein [Gryllus bimaculatus]	9,00E-20
gALL_v3_c15326	3	3	-	-	-	-	-
gALL_v3_c15327	4	6	gnl|CDD|128591	smart00295, B41, Band 4.1 ho	ref|XP_002423662.1|	FERM, RhoGEF and pleckstrin domain-containing protein, putative [Pediculus humanus corporis] gb|EEB10924.1| FERM, RhoGEF and pleckstrin domain-containing protein	1,00E-113
gALL_v3_c15328	0	5	-	-	-	-	-
gALL_v3_c15329	3	1	-	-	-	-	-
gALL_v3_c15330	6	9	-	-	gb|AGM32821.1|	hypothetical protein [Coptotermes formosanus]	2,00E-12
gALL_v3_c15331	2	6	gnl|CDD|118676	pfam10148, SCHIP-1, Schwanr	gb|EFA04405.1|	hypothetical protein TcasGA2_TC014704 [Tribolium castaneum]	1,00E-31
gALL_v3_c15333	4	2	-	-	-	-	-
gALL_v3_c15334	3	5	-	-	gb|EFA11771.1|	hypothetical protein TcasGA2_TC004098 [Tribolium castaneum]	3,00E-38
gALL_v3_c15335	3	1	-	-	-	-	-
gALL_v3_c15336	3	2	-	-	gb|AGM32364.1|	EF-hand family protein [Coptotermes formosanus]	2,00E-55
gALL_v3_c15337	4	6	-	-	-	-	-
gALL_v3_c15338	2	2	-	-	-	-	-
gALL_v3_c15339	0	4	-	-	-	-	-
gALL_v3_c1534	23	21	gnl|CDD|111993	pfam03157, Glutenin_hmw, H	gb|EGI63714.1|	Far upstream element-binding protein 1 [Acromyrmex echinatior]	2,00E-39
gALL_v3_c15340	5	3	-	-	-	-	-
gALL_v3_c15341	1	4	-	-	ref|XP_002423724.1|	conserved hypothetical protein [Pediculus humanus corporis] gb|EEB10986.1| conserved hypothetical protein [Pediculus humanus corporis]	1,00E-69
gALL_v3_c15342	4	7	-	-	-	-	-
gALL_v3_c15343	1	5	-	-	ref|WP_005642268.1|	glycosyl hydrolase family 3 [Parabacteroides merdae] gb|EKN17311.1| hypothetical protein HMPREF1060_00148 [Parabacteroides merdae CL03T12C32]	2,00E-36
gALL_v3_c15344	5	5	-	-	-	-	-
gALL_v3_c15345	5	4	-	-	-	-	-
gALL_v3_c15346	2	4	-	-	ref|XP_005519393.1|	PREDICTED: dual specificity tyrosine-phosphorylation-regulated kinase 2 [Pseudopodoces humilis]	1,00E-11
gALL_v3_c15347	2	3	-	-	-	-	-
gALL_v3_c15348	5	5	gnl|CDD|28966	cd01388, SOX-TCF_HMG-box,	ref|XP_002423919.1|	sox13, putative [Pediculus humanus corporis] gb|EEB11181.1| sox13, putative [Pediculus humanus corporis]	4,00E-98
gALL_v3_c15349	4	5	-	-	ref|XP_003708393.1|	PREDICTED: PR domain zinc finger protein 5-like isoform 2 [Megachile rotundata]	8,00E-96
gALL_v3_c1535	25	17	gnl|CDD|145580	pfam02516, STT3, Oligosaccha	ref|XP_002430030.1|	Dolichyl-diphosphooligosaccharide--protein glycosyltransferase subunit STT3B, putative [Pediculus humanus corporis] gb|EEB17292.1| Dolichyl-diphosphooligosaccharide-	0
gALL_v3_c15350	3	1	gnl|CDD|189843	pfam01105, EMP24_GP25L, er	ref|XP_001991639.1|	GH11950 [Drosophila grimshawi] gb|EDW00264.1| GH11950 [Drosophila grimshawi]	2,00E-37
gALL_v3_c15351	2	3	-	-	ref|XP_003690213.1|	PREDICTED: equilibrative nucleoside transporter 4-like [Apis florea]	1,00E-20
gALL_v3_c15352	0	5	gnl|CDD|29261	cd00204, ANK, ankyrin repeat:	pdb|4GPM|A	Chain A, Crystal Structure Of Engineered Protein. Northeast Structural Genomics Consortium Target Or264. pdb|4GPM|B Chain B, Crystal Structure Of Engineered Protein.	9,00E-14
gALL_v3_c15353	9	6	-	-	-	-	-
gALL_v3_c15354	1	6	-	-	-	-	-
gALL_v3_c15356	2	4	-	-	-	-	-
gALL_v3_c15357	5	3	gnl|CDD|173833	cd00685, Trans_IPPS_HT, Tran:	gb|ACT79809.3|	mitochondrial farnesyl diphosphate synthase [Aphis gossypii]	1,00E-75
gALL_v3_c15359	1	4	-	-	-	-	-
gALL_v3_c1536	10	20	-	-	gb|EFN78882.1|	hypothetical protein EAI_02290 [Harpegnathos saltator]	3,00E-12
gALL_v3_c15360	2	4	-	-	-	-	-
gALL_v3_c15361	3	4	-	-	-	-	-
gALL_v3_c15362	5	6	gnl|CDD|151191	pfam10695, Cw-hydrolase, Cel	ref|WP_002547602.1|	hypothetical protein [Propionibacterium acnes] gb|EFS80503.1| hypothetical protein HMPREF9597_00208 [Propionibacterium acnes HL005PA4]	5,00E-71
gALL_v3_c15363	11	11	-	-	-	-	-
gALL_v3_c15364	1	5	-	-	-	-	-
gALL_v3_c15365	2	7	gnl|CDD|189411	pfam00135, COesterase, Carb	gb|EFZ14712.1|	hypothetical protein SINV_09966 [Solenopsis invicta]	4,00E-33
gALL_v3_c15366	2	3	-	-	-	-	-
gALL_v3_c15367	4	2	-	-	-	-	-
gALL_v3_c15368	6	4	-	-	gb|AGM32707.1|	hypothetical protein, partial [Coptotermes formosanus]	2,00E-15
gALL_v3_c15369	4	4	-	-	-	-	-
gALL_v3_c15370	10	7	gnl|CDD|146903	pfam04495, GRASP55_65, GR/	ref|XP_001607136.1|	PREDICTED: Golgi reassembly-stacking protein 2-like [Nasonia vitripennis]	1,00E-120
gALL_v3_c15371	3	6	gnl|CDD|187657	cd08954, KR_1_FAS_SDR_x, be	ref|XP_002429689.1|	fatty acid synthase, putative [Pediculus humanus corporis] gb|EEB16951.1| fatty acid synthase, putative [Pediculus humanus corporis]	3,00E-70
gALL_v3_c15372	2	4	-	-	-	-	-
gALL_v3_c15373	3	4	-	-	-	-	-
gALL_v3_c15374	1	3	-	-	gb|AGM32707.1|	hypothetical protein, partial [Coptotermes formosanus]	1,00E-19
gALL_v3_c15375	1	7	-	-	-	-	-
gALL_v3_c15376	2	5	-	-	-	-	-
gALL_v3_c15377	3	6	-	-	gb|EGI70368.1|	Adenomatous polyposis coli protein [Acromyrmex echinatior]	5,00E-32
gALL_v3_c15378	3	6	-	-	-	-	-
gALL_v3_c15379	0	5	-	-	-	-	-
gALL_v3_c1538	6	5	-	-	-	-	-
gALL_v3_c15380	1	4	gnl|CDD|178625	PLN03076, PLN03076, ARF gu	ref|XP_002425320.1|	Brefeldin A-inhibited guanine nucleotide-exchange protein, putative [Pediculus humanus corporis] gb|EEB12582.1| Brefeldin A-inhibited guanine nucleotide-exchange pro	7,00E-92
gALL_v3_c15382	4	4	gnl|CDD|178253	PLN02647, PLN02647, acyl-Co	ref|XP_310406.7|	AGAP003848-PA [Anopheles gambiae str. PEST] gb|EAA05998.5| AGAP003848-PA [Anopheles gambiae str. PEST]	7,00E-89
gALL_v3_c15383	12	15	gnl|CDD|178174	PLN02560, PLN02560, enoyl-C	ref|XP_002423113.1|	synaptic glycoprotein SC2, putative [Pediculus humanus corporis] gb|EEB10375.1| synaptic glycoprotein SC2, putative [Pediculus humanus corporis]	1,00E-128
gALL_v3_c15387	3	6	-	-	-	-	-
gALL_v3_c15388	9	5	gnl|CDD|109297	pfam00233, PDEase_I, 3'5'-cyc	ref|XP_001943552.2|	PREDICTED: high affinity cAMP-specific and IBMX-insensitive 3',5'-cyclic phosphodiesterase 8A-like, partial [Acyrthosiphon pisum]	1,00E-152
gALL_v3_c15389	1	4	-	-	ref|XP_001809819.1|	PREDICTED: similar to forkhead box transcription factor subgroup N1 [Tribolium castaneum]	1,00E-08
gALL_v3_c1539	6	3	-	-	-	-	-
gALL_v3_c15390	2	3	-	-	-	-	-
gALL_v3_c15391	5	10	-	-	-	-	-
gALL_v3_c15392	7	7	-	-	-	-	-

gALL_v3_c15393	3	2	-	-	gb|EFN60413.1|	Ubiquitin carboxyl-terminal hydrolase 7 [Camponotus floridanus]	3,00E-63
gALL_v3_c15394	6	5	-	-	-	-	-
gALL_v3_c15397	4	2	-	-	-	-	-
gALL_v3_c15398	10	8	-	-	-	-	-
gALL_v3_c154	13	10	-	-	ref|XP_003485117.1|	PREDICTED: hypothetical protein LOC100747792 [Bombus impatiens]	1,00E-32
gALL_v3_c1540	8	11	-	-	-	-	-
gALL_v3_c15400	3	3	-	-	-	-	-
gALL_v3_c15402	4	9	-	-	-	-	-
gALL_v3_c15403	7	8	gnl|CDD|187667	cd09807, retinol-DH_like_SDR	gb|AGM32808.1|	retinol dehydrogenase 13-like protein [Coptotermes formosanus]	2,00E-72
gALL_v3_c15404	5	2	gnl|CDD|189507	pfam00335, Tetraspannin, Tetr	ref|XP_005798089.1|	PREDICTED: tetraspanin-7-like [Xiphophorus maculatus]	2,00E-09
gALL_v3_c15405	4	2	gnl|CDD|178625	PLN03076, PLN03076, ARF gua	ref|XP_004927408.1|	PREDICTED: brefeldin A-inhibited guanine nucleotide-exchange protein 1-like [Bombyx mori]	1,00E-111
gALL_v3_c15406	2	2	gnl|CDD|189507	pfam00335, Tetraspannin, Tetr	gb|AGM32395.1|	tetraspanin-18 [Coptotermes formosanus]	5,00E-87
gALL_v3_c15407	2	6	-	-	-	-	-
gALL_v3_c15408	2	2	-	-	-	-	-
gALL_v3_c15409	2	5	gnl|CDD|128433	smart00128, IPPc, Inositol poly	ref|XP_003492036.1|	PREDICTED: synaptojanin-1-like [Bombus impatiens]	1,00E-116
gALL_v3_c15410	3	6	gnl|CDD|192861	pfam11882, DUF3402, Domain	ref|XP_001603859.2|	PREDICTED: protein FAM40A-like [Nasonia vitripennis]	3,00E-72
gALL_v3_c15412	9	14	-	-	-	-	-
gALL_v3_c15413	6	5	-	-	-	-	-
gALL_v3_c15414	5	3	-	-	gb|AGM32283.1|	sterol desaturase, partial [Coptotermes formosanus]	2,00E-38
gALL_v3_c15415	4	4	-	-	-	-	-
gALL_v3_c15416	9	21	-	-	-	-	-
gALL_v3_c15417	5	4	-	-	-	-	-
gALL_v3_c15418	9	7	-	-	-	-	-
gALL_v3_c15419	4	5	-	-	ref|XP_003426754.1|	PREDICTED: hypothetical protein LOC100678498 [Nasonia vitripennis]	6,00E-16
gALL_v3_c1542	4	5	-	-	-	-	-
gALL_v3_c15420	6	3	gnl|CDD|189372	pfam00067, p450, Cytochrome	gb|EFA12633.1|	cytochrome P450 6BK5 [Tribolium castaneum]	5,00E-58
gALL_v3_c15421	6	5	-	-	-	-	-
gALL_v3_c15422	0	6	-	-	-	-	-
gALL_v3_c15423	3	7	-	-	ref|XP_006267629.1|	PREDICTED: TRMT1-like protein isoform X2 [Alligator mississippiensis]	6,00E-11
gALL_v3_c15426	4	9	-	-	-	-	-
gALL_v3_c15427	2	4	-	-	-	-	-
gALL_v3_c15428	2	4	-	-	-	-	-
gALL_v3_c15429	5	3	-	-	ref|XP_001650585.1|	hypothetical protein AaeL_AAEL005267 [Aedes aegypti] gb|EAT43267.1| AAEL005267-PA [Aedes aegypti]	2,00E-27
gALL_v3_c1543	19	28	-	-	-	-	-
gALL_v3_c15430	2	4	gnl|CDD|131304	TIGR02250, FCP1_euk, FCP1-li	ref|XP_002428378.1|	RNA polymerase II ctd phosphatase, putative [Pediculus humanus corporis] gb|EEB15640.1| RNA polymerase II ctd phosphatase, putative [Pediculus humanus corporis]	1,00E-81
gALL_v3_c15431	3	3	gnl|CDD|28928	cd00268, DEADc, DEAD-box he	ref|XP_001602245.2|	PREDICTED: probable ATP-dependent RNA helicase DDX27-like [Nasonia vitripennis]	2,00E-52
gALL_v3_c15432	2	3	-	-	gb|EFA02585.1|	hypothetical protein TcasGA2_TC008304 [Tribolium castaneum]	1,00E-21
gALL_v3_c15434	3	5	-	-	gb|EEZ98380.1|	hypothetical protein TcasGA2_TC000840 [Tribolium castaneum]	4,00E-24
gALL_v3_c15435	1	3	-	-	-	-	-
gALL_v3_c15436	0	4	-	-	-	-	-
gALL_v3_c15437	3	7	-	-	-	-	-
gALL_v3_c15438	1	4	-	-	-	-	-
gALL_v3_c1544	10	16	gnl|CDD|189915	pfam01265, Cyto_heme_lyase	gb|EFZ21242.1|	hypothetical protein SINV_01786 [Solenopsis invicta]	1,00E-105
gALL_v3_c15440	2	3	-	-	-	-	-
gALL_v3_c15441	6	7	gnl|CDD|128524	smart00228, PDZ, Domain pre	dbj|BAK23256.1|	discs large 1 [Gryllus bimaculatus]	1,00E-109
gALL_v3_c15444	5	6	gnl|CDD|128796	smart00523, DWA, Domain A i	ref|XP_002428963.1|	smad7, putative [Pediculus humanus corporis] gb|EEB16225.1| smad7, putative [Pediculus humanus corporis]	1,00E-47
gALL_v3_c15445	3	1	-	-	-	-	-
gALL_v3_c15446	2	4	-	-	-	-	-
gALL_v3_c15447	2	3	-	-	ref|XP_002432775.1|	Polyglutamine-binding protein, putative [Pediculus humanus corporis] gb|EEB20037.1| Polyglutamine-binding protein, putative [Pediculus humanus corporis]	1,00E-48
gALL_v3_c15448	4	3	-	-	-	-	-
gALL_v3_c15451	0	4	-	-	-	-	-
gALL_v3_c15452	0	6	gnl|CDD|189661	pfam00665, rve, Integrase cor	ref|XP_002012399.1|	GI11945 [Drosophila mojavensis] gb|EDW05382.1| GI11945 [Drosophila mojavensis]	2,00E-13
gALL_v3_c15453	9	16	gnl|CDD|162472	TIGR01661, ELAV_HUD_SF, EL	gb|EFN84815.1|	RNA-binding motif, single-stranded-interacting protein 1 [Harpegnathos saltator]	1,00E-80
gALL_v3_c15454	2	4	-	-	-	-	-
gALL_v3_c15455	2	2	gnl|CDD|128734	smart00458, RICIN, Ricin-type	ref|XP_002425051.1|	UDP-GalNAc:polypeptide N-acetylgalactosaminyltransferase, putative [Pediculus humanus corporis] gb|EEB12313.1| UDP-GalNAc:polypeptide N-acetylgalactosaminyltrans	4,00E-54
gALL_v3_c15456	4	2	-	-	-	-	-
gALL_v3_c15457	5	3	-	-	-	-	-
gALL_v3_c15458	3	5	-	-	gb|AAB65093.1|	Lian-Aa1 retrotransposon protein [Aedes aegypti]	3,00E-13
gALL_v3_c15459	2	6	-	-	-	-	-
gALL_v3_c1546	17	25	-	-	gb|EHJ70865.1|	endonuclease-reverse transcriptase [Danaus plexippus]	5,00E-70
gALL_v3_c15460	4	3	-	-	-	-	-
gALL_v3_c15461	4	4	-	-	gb|EFN83829.1|	WW domain-binding protein 11 [Harpegnathos saltator]	3,00E-19
gALL_v3_c15462	3	3	-	-	-	-	-
gALL_v3_c15463	4	3	gnl|CDD|146646	pfam04117, Mpv17_PMP22, M	ref|XP_001238256.1|	AGAP012415-PA [Anopheles gambiae str. PEST] gb|EAU75753.1| AGAP012415-PA [Anopheles gambiae str. PEST]	4,00E-15
gALL_v3_c15464	3	1	-	-	-	-	-
gALL_v3_c15465	2	3	gnl|CDD|188785	cd09401, LIM_TLP_like, The L	dbj|BAH36878.1|	Ras association family member [Gryllus bimaculatus]	3,00E-88
gALL_v3_c15466	1	3	-	-	-	-	-
gALL_v3_c15467	9	11	-	-	-	-	-
gALL_v3_c15468	3	7	-	-	-	-	-
gALL_v3_c15469	7	8	-	-	-	-	-
gALL_v3_c1547	2	7	-	-	-	-	-
gALL_v3_c15470	8	11	-	-	-	-	-
gALL_v3_c15471	5	5	gnl|CDD|162158	TIGR01015, hmgA, homogenti	gb|EHJ71950.1|	homogentisate 1,2-dioxygenase [Danaus plexippus]	1,00E-117
gALL_v3_c15472	5	2	-	-	-	-	-
gALL_v3_c15473	5	8	-	-	-	-	-
gALL_v3_c15475	5	4	-	-	-	-	-
gALL_v3_c15476	4	4	-	-	-	-	-
gALL_v3_c15477	0	4	-	-	-	-	-
gALL_v3_c15478	2	5	-	-	-	-	-
gALL_v3_c1548	6	7	gnl|CDD|29397	cd01926, cyclophilin_ABH_like	ref|XP_003700787.1|	PREDICTED: uncharacterized protein LOC100879476 [Megachile rotundata]	7,00E-78
gALL_v3_c15480	1	4	-	-	-	-	-
gALL_v3_c15481	4	4	-	-	gb|EGI68656.1|	hypothetical protein G5I_02660 [Acromyrmex echinatior]	7,00E-20
gALL_v3_c15482	3	3	-	-	ref|XP_003394068.1|	PREDICTED: transmembrane protein 8A-like [Bombus terrestris]	2,00E-26
gALL_v3_c15483	3	1	-	-	-	-	-
gALL_v3_c15485	2	4	-	-	-	-	-
gALL_v3_c15486	5	9	-	-	ref|XP_001602426.2|	PREDICTED: microtubule-actin cross-linking factor 1-like [Nasonia vitripennis]	3,00E-09
gALL_v3_c15487	3	3	-	-	gb|AGM32707.1|	hypothetical protein, partial [Coptotermes formosanus]	7,00E-11
gALL_v3_c15488	1	4	-	-	-	-	-
gALL_v3_c1549	6	3	-	-	-	-	-
gALL_v3_c15490	6	4	-	-	-	-	-
gALL_v3_c15491	2	2	-	-	ref|XP_001811347.1|	PREDICTED: similar to transcription initiation factor TFIID subunit 1 [Tribolium castaneum]	6,00E-33
gALL_v3_c15492	1	3	-	-	-	-	-
gALL_v3_c15493	1	4	-	-	ref|XP_003730851.1|	PREDICTED: uncharacterized protein LOC100891800 [Strongylocentrotus purpuratus]	3,00E-11
gALL_v3_c15494	0	4	gnl|CDD|190226	pfam02145, Rap_GAP, Rap/ran	ref|XP_004928319.1|	PREDICTED: uncharacterized protein LOC101743267 [Bombyx mori]	9,00E-97
gALL_v3_c15495	3	4	-	-	ref|XP_003694231.1|	PREDICTED: hemocytin-like [Apis florea]	2,00E-33
gALL_v3_c15496	1	5	-	-	-	-	-
gALL_v3_c15497	4	2	-	-	-	-	-
gALL_v3_c15498	2	3	-	-	-	-	-
gALL_v3_c15499	0	6	-	-	-	-	-
gALL_v3_c1550	5	3	-	-	-	-	-
gALL_v3_c15500	8	10	gnl|CDD|192960	pfam12230, PRP21_like_P, Pre	ref|XP_003395004.1|	PREDICTED: splicing factor 3A subunit 1-like isoform 2 [Bombus terrestris]	4,00E-86
gALL_v3_c15501	0	5	-	-	ref|XP_004932697.1|	PREDICTED: protein SON-like [Bombyx mori]	1,00E-14
gALL_v3_c15502	3	2	-	-	ref|XP_973485.1|	PREDICTED: similar to CG14591 CG14591-PA [Tribolium castaneum]	1,00E-50
gALL_v3_c15503	2	4	-	-	-	-	-
gALL_v3_c15505	5	3	-	-	-	-	-
gALL_v3_c15506	3	2	-	-	gb|AGM32283.1|	sterol desaturase, partial [Coptotermes formosanus]	4,00E-22
gALL_v3_c15507	4	4	-	-	-	-	-
gALL_v3_c15508	7	4	-	-	gb|EFX86814.1|	hypothetical protein DAPPUDRAFT_44590 [Daphnia pulex]	9,00E-31
gALL_v3_c15509	3	2	-	-	-	-	-
gALL_v3_c1551	6	10	-	-	ref|XP_003401736.1|	PREDICTED: hypothetical protein LOC100645102 [Bombus terrestris]	3,00E-16
gALL_v3_c15510	3	2	gnl|CDD|144379	pfam00755, Carn_acyltransf, C	gb|EFN78122.1|	Carnitine O-palmitoyltransferase I, liver isoform [Harpegnathos saltator]	8,00E-84
gALL_v3_c15512	2	2	-	-	-	-	-
gALL_v3_c15513	2	2	-	-	-	-	-
gALL_v3_c15515	1	5	-	-	-	-	-
gALL_v3_c15517	1	6	-	-	gb|EHJ78808.1|	hypothetical protein KGM_00330 [Danaus plexippus]	5,00E-11
gALL_v3_c15518	4	6	-	-	-	-	-
gALL_v3_c15519	2	3	-	-	ref|XP_002433230.1|	type II transmembrane protein, putative [Pediculus humanus corporis] gb|EEB20492.1| type II transmembrane protein, putative [Pediculus humanus corporis]	1,00E-80
gALL_v3_c1552	7	19	-	-	-	-	-
gALL_v3_c15521	3	6	-	-	-	-	-
gALL_v3_c15522	0	5	-	-	-	-	-
gALL_v3_c15523	4	5	-	-	ref|XP_002427184.1|	class B secretin-like G-protein coupled receptor GPRmth5, putative [Pediculus humanus corporis] gb|EEB14446.1| class B secretin-like G-protein coupled receptor GPRmth	4,00E-14
gALL_v3_c15525	1	7	-	-	-	-	-
gALL_v3_c15526	3	4	gnl|CDD|111999	pfam03164, Mon1, Trafficking	gb|EFZ20660.1|	hypothetical protein SINV_05878 [Solenopsis invicta]	5,00E-63
gALL_v3_c15527	5	3	gnl|CDD|189970	pfam01395, PBP_GOBP, PBP/G	-	-	-
gALL_v3_c15528	2	3	gnl|CDD|189660	pfam00664, ABC_membrane,	gb|EGI66812.1|	Sodium leak channel non-selective protein [Acromyrmex echinatior]	3,00E-63
gALL_v3_c15529	0	5	-	-	-	-	-
gALL_v3_c15532	9	6	-	-	-	-	-
gALL_v3_c15533	1	3	-	-	-	-	-
gALL_v3_c15534	3	1	-	-	-	-	-
gALL_v3_c15535	4	2	gnl|CDD|28913	cd00031, CA, Cadherin repeat	ref|XP_003693057.1|	PREDICTED: cadherin-87A-like [Apis florea]	1,00E-35
gALL_v3_c15536	3	12	-	-	-	-	-
gALL_v3_c15537	2	2	-	-	-	-	-
gALL_v3_c15538	3	1	-	-	-	-	-
gALL_v3_c15539	2	3	-	-	ref|XP_003488418.1|	PREDICTED: hypothetical protein LOC100740739 [Bombus impatiens]	1,00E-13
gALL_v3_c1554	13	18	gnl|CDD|190454	pfam02862, DDHD, DDHD don	ref|XP_003428106.1|	PREDICTED: hypothetical protein LOC100118865 isoform 2 [Nasonia vitripennis]	0
gALL_v3_c15542	4	4	gnl|CDD|31001	COG0656, ARA1, Aldo/keto re	gb|AGM32585.1|	aldo-keto reductase (AKR), partial [Coptotermes formosanus]	4,00E-57
gALL_v3_c15543	4	6	-	-	ref|XP_003493916.1|	PREDICTED: endoplasmic reticulum mannosyl-oligosaccharide 1,2-alpha-mannosidase-like [Bombus impatiens]	5,00E-07
gALL_v3_c15544	2	4	-	-	-	-	-
gALL_v3_c15545	5	2	-	-	-	-	-
gALL_v3_c15546	1	8	-	-	ref|XP_005192209.1|	PREDICTED: F-box/LRR-repeat protein 16-like, partial [Musca domestica]	9,00E-91
gALL_v3_c15547	3	2	-	-	-	-	-
gALL_v3_c15548	4	6	gnl|CDD|30753	COG0404, GcvT, Glycine cleava	ref|XP_002425644.1|	Sarcosine dehydrogenase, putative [Pediculus humanus corporis] gb|EEB12906.1| Sarcosine dehydrogenase, putative [Pediculus humanus corporis]	1,00E-142
gALL_v3_c15549	3	2	-	-	-	-	-
gALL_v3_c1555	3	12	-	-	-	-	-

<table>
<tr><th></th><th></th><th></th><th></th><th></th><th></th><th></th><th></th></tr>
<tr><td>gALL_v3_c15551</td><td>5</td><td>7</td><td>gnl|CDD|191211</td><td>pfam05154, TM2, TM2 domai</td><td>gb|EFZ18641.1|</td><td>hypothetical protein SINV_10993 [Solenopsis invicta]</td><td>3,00E-53</td></tr>
<tr><td>gALL_v3_c15553</td><td>6</td><td>10</td><td>-</td><td>-</td><td>-</td><td>-</td><td>-</td></tr>
<tr><td>gALL_v3_c15554</td><td>1</td><td>3</td><td>-</td><td>-</td><td>-</td><td>-</td><td>-</td></tr>
<tr><td>gALL_v3_c15555</td><td>6</td><td>3</td><td>-</td><td>-</td><td>ref|XP_006567052.1|</td><td>PREDICTED: LOW QUALITY PROTEIN: chromodomain-helicase-DNA-binding protein 7-like [Apis mellifera]</td><td>3,00E-13</td></tr>
<tr><td>gALL_v3_c15557</td><td>5</td><td>6</td><td>gnl|CDD|29549</td><td>cd01011, nicotinamidase, Nicc</td><td>ref|XP_973348.1|</td><td>PREDICTED: similar to pyrazinamidase/nicotinamidase [Tribolium castaneum]</td><td>0</td></tr>
<tr><td>gALL_v3_c15558</td><td>5</td><td>2</td><td>-</td><td>-</td><td>-</td><td>-</td><td>-</td></tr>
<tr><td>gALL_v3_c15559</td><td>5</td><td>3</td><td>-</td><td>-</td><td>-</td><td>-</td><td>-</td></tr>
<tr><td>gALL_v3_c1556</td><td>7</td><td>8</td><td>-</td><td>-</td><td>gb|EFN77525.1|</td><td>Inositol hexakisphosphate and diphosphoinositol-pentakisphosphate kinase [Harpegnathos saltator]</td><td>3,00E-27</td></tr>
<tr><td>gALL_v3_c15560</td><td>3</td><td>1</td><td>-</td><td>-</td><td>-</td><td>-</td><td>-</td></tr>
<tr><td>gALL_v3_c15561</td><td>2</td><td>4</td><td>-</td><td>-</td><td>-</td><td>-</td><td>-</td></tr>
<tr><td>gALL_v3_c15562</td><td>1</td><td>4</td><td>-</td><td>-</td><td>-</td><td>-</td><td>-</td></tr>
<tr><td>gALL_v3_c15563</td><td>0</td><td>3</td><td>-</td><td>-</td><td>gb|EFN89132.1|</td><td>PiggyBac transposable element-derived protein 4 [Harpegnathos saltator]</td><td>3,00E-17</td></tr>
<tr><td>gALL_v3_c15564</td><td>5</td><td>7</td><td>-</td><td>-</td><td>-</td><td>-</td><td>-</td></tr>
<tr><td>gALL_v3_c15565</td><td>4</td><td>7</td><td>-</td><td>-</td><td>ref|XP_003398278.1|</td><td>PREDICTED: mediator of RNA polymerase II transcription subunit 30-like [Bombus terrestris]</td><td>5,00E-09</td></tr>
<tr><td>gALL_v3_c15566</td><td>7</td><td>5</td><td>-</td><td>-</td><td>gb|AGM32864.1|</td><td>selenium-binding protein 1-like protein [Coptotermes formosanus]</td><td>5,00E-10</td></tr>
<tr><td>gALL_v3_c15567</td><td>2</td><td>5</td><td>-</td><td>-</td><td>-</td><td>-</td><td>-</td></tr>
<tr><td>gALL_v3_c15568</td><td>4</td><td>3</td><td>gnl|CDD|115470</td><td>pfam06814, Lung_7-TM_R, Lu</td><td>ref|XP_001662124.1|</td><td>hypothetical protein AaeL_AAEL002694 [Aedes aegypti]</td><td>2,00E-58</td></tr>
<tr><td>gALL_v3_c15571</td><td>3</td><td>3</td><td>-</td><td>-</td><td>ref|XP_002430182.1|</td><td>serine/threonine-protein kinase VRK1, putative [Pediculus humanus corporis] gb|EEB17444.1| serine/threonine-protein kinase VRK1, putative [Pediculus humanus corpori</td><td>3,00E-53</td></tr>
<tr><td>gALL_v3_c15572</td><td>2</td><td>4</td><td>-</td><td>-</td><td>-</td><td>-</td><td>-</td></tr>
<tr><td>gALL_v3_c15573</td><td>6</td><td>9</td><td>-</td><td>-</td><td>-</td><td>-</td><td>-</td></tr>
<tr><td>gALL_v3_c15574</td><td>1</td><td>4</td><td>-</td><td>-</td><td>-</td><td>-</td><td>-</td></tr>
<tr><td>gALL_v3_c15577</td><td>5</td><td>5</td><td>-</td><td>-</td><td>-</td><td>-</td><td>-</td></tr>
<tr><td>gALL_v3_c15578</td><td>9</td><td>5</td><td>gnl|CDD|177986</td><td>PLN02353, PLN02353, probabl</td><td>ref|XP_002061809.1|</td><td>GK17198 [Drosophila willistoni] gb|EDW72795.1| GK17198 [Drosophila willistoni]</td><td>0</td></tr>
<tr><td>gALL_v3_c15579</td><td>5</td><td>6</td><td>-</td><td>-</td><td>ref|XP_001950310.2|</td><td>PREDICTED: NFX1-type zinc finger-containing protein 1-like [Acyrthosiphon pisum]</td><td>4,00E-24</td></tr>
<tr><td>gALL_v3_c1558</td><td>4</td><td>13</td><td>-</td><td>-</td><td>-</td><td>-</td><td>-</td></tr>
<tr><td>gALL_v3_c15582</td><td>2</td><td>3</td><td>-</td><td>-</td><td>ref|XP_002425571.1|</td><td>conserved hypothetical protein [Pediculus humanus corporis] gb|EEB12833.1| conserved hypothetical protein [Pediculus humanus corporis]</td><td>3,00E-58</td></tr>
<tr><td>gALL_v3_c15583</td><td>2</td><td>6</td><td>gnl|CDD|162843</td><td>TIGR02403, trehalose_treC, al</td><td>ref|XP_001958835.1|</td><td>GF12364 [Drosophila ananassae] gb|EDV35657.1| GF12364 [Drosophila ananassae]</td><td>4,00E-48</td></tr>
<tr><td>gALL_v3_c15584</td><td>5</td><td>3</td><td>-</td><td>-</td><td>ref|XP_002431919.1|</td><td>Chordin precursor, putative [Pediculus humanus corporis] gb|EEB19181.1| Chordin precursor, putative [Pediculus humanus corporis]</td><td>1,00E-53</td></tr>
<tr><td>gALL_v3_c15585</td><td>1</td><td>5</td><td>-</td><td>-</td><td>gb|EFN67634.1|</td><td>Putative uncharacterized transposon-derived protein F52C9.6 [Camponotus floridanus]</td><td>2,00E-10</td></tr>
<tr><td>gALL_v3_c15586</td><td>4</td><td>4</td><td>gnl|CDD|189576</td><td>pfam00498, FHA, FHA domain</td><td>ref|XP_003770355.1|</td><td>PREDICTED: nibrin isoform 1 [Sarcophilus harrisii]</td><td>2,00E-52</td></tr>
<tr><td>gALL_v3_c15587</td><td>3</td><td>2</td><td>gnl|CDD|115363</td><td>pfam06701, MIB_HERC2, Mib_</td><td>ref|XP_002424807.1|</td><td>hect E3 ubiquitin ligase, putative [Pediculus humanus corporis] gb|EEB12069.1| hect E3 ubiquitin ligase, putative [Pediculus humanus corporis]</td><td>9,00E-70</td></tr>
<tr><td>gALL_v3_c15588</td><td>1</td><td>3</td><td>-</td><td>-</td><td>-</td><td>-</td><td>-</td></tr>
<tr><td>gALL_v3_c15589</td><td>2</td><td>6</td><td>-</td><td>-</td><td>ref|XP_006617143.1|</td><td>PREDICTED: glycerol-3-phosphate acyltransferase 1, mitochondrial-like isoform X3 [Apis dorsata]</td><td>8,00E-13</td></tr>
<tr><td>gALL_v3_c1559</td><td>2</td><td>5</td><td>gnl|CDD|30989</td><td>COG0644, FixC, Dehydrogenas</td><td>gb|ENN71830.1|</td><td>hypothetical protein YQE_11501, partial [Dendroctonus ponderosae]</td><td>5,00E-86</td></tr>
<tr><td>gALL_v3_c15591</td><td>5</td><td>2</td><td>-</td><td>-</td><td>ref|XP_006616124.1|</td><td>PREDICTED: uncharacterized protein LOC102673110 [Apis dorsata]</td><td>6,00E-19</td></tr>
<tr><td>gALL_v3_c15592</td><td>3</td><td>3</td><td>-</td><td>-</td><td>gb|EFN84697.1|</td><td>Mps one binder kinase activator-like 4 [Harpegnathos saltator]</td><td>4,00E-09</td></tr>
<tr><td>gALL_v3_c15593</td><td>3</td><td>2</td><td>-</td><td>-</td><td>ref|XP_970228.2|</td><td>PREDICTED: similar to formin 3 CG33556-PB [Tribolium castaneum] gb|EEZ97607.1| hypothetical protein TcasGA2_TC011477 [Tribolium castaneum]</td><td>9,00E-50</td></tr>
<tr><td>gALL_v3_c15594</td><td>3</td><td>6</td><td>-</td><td>-</td><td>gb|ERL92354.1|</td><td>hypothetical protein D910_09668 [Dendroctonus ponderosae]</td><td>1,00E-126</td></tr>
<tr><td>gALL_v3_c15595</td><td>3</td><td>4</td><td>gnl|CDD|31415</td><td>COG1222, RPT1, ATP-depende</td><td>gb|EFN60826.1|</td><td>Vesicle-fusing ATPase 1 [Camponotus floridanus]</td><td>6,00E-92</td></tr>
<tr><td>gALL_v3_c15596</td><td>2</td><td>6</td><td>-</td><td>-</td><td>ref|XP_003701766.1|</td><td>PREDICTED: uncharacterized protein LOC100878885 [Megachile rotundata]</td><td>8,00E-56</td></tr>
<tr><td>gALL_v3_c15597</td><td>4</td><td>5</td><td>-</td><td>-</td><td>-</td><td>-</td><td>-</td></tr>
<tr><td>gALL_v3_c15598</td><td>6</td><td>8</td><td>-</td><td>-</td><td>-</td><td>-</td><td>-</td></tr>
<tr><td>gALL_v3_c15599</td><td>8</td><td>10</td><td>-</td><td>-</td><td>-</td><td>-</td><td>-</td></tr>
<tr><td>gALL_v3_c1560</td><td>9</td><td>15</td><td>-</td><td>-</td><td>ref|XP_003693535.1|</td><td>PREDICTED: uncharacterized protein LOC100867689 [Apis florea]</td><td>4,00E-10</td></tr>
<tr><td>gALL_v3_c15600</td><td>5</td><td>5</td><td>-</td><td>-</td><td>-</td><td>-</td><td>-</td></tr>
<tr><td>gALL_v3_c15601</td><td>3</td><td>2</td><td>-</td><td>-</td><td>-</td><td>-</td><td>-</td></tr>
<tr><td>gALL_v3_c15602</td><td>10</td><td>19</td><td>-</td><td>-</td><td>gb|ENN73069.1|</td><td>hypothetical protein YQE_10307, partial [Dendroctonus ponderosae]</td><td>4,00E-16</td></tr>
<tr><td>gALL_v3_c15604</td><td>5</td><td>5</td><td>-</td><td>-</td><td>-</td><td>-</td><td>-</td></tr>
<tr><td>gALL_v3_c15605</td><td>2</td><td>3</td><td>-</td><td>-</td><td>gb|ETI29806.1|</td><td>hypothetical protein F443_23078 [Phytophthora parasitica P1569]</td><td>3,00E-15</td></tr>
<tr><td>gALL_v3_c15606</td><td>1</td><td>4</td><td>-</td><td>-</td><td>-</td><td>-</td><td>-</td></tr>
<tr><td>gALL_v3_c15607</td><td>3</td><td>3</td><td>-</td><td>-</td><td>-</td><td>-</td><td>-</td></tr>
<tr><td>gALL_v3_c15609</td><td>0</td><td>4</td><td>-</td><td>-</td><td>-</td><td>-</td><td>-</td></tr>
<tr><td>gALL_v3_c1561</td><td>7</td><td>8</td><td>-</td><td>-</td><td>ref|XP_003707950.1|</td><td>PREDICTED: uncharacterized protein LOC100883382 [Megachile rotundata]</td><td>1,00E-119</td></tr>
<tr><td>gALL_v3_c15610</td><td>4</td><td>5</td><td>gnl|CDD|145024</td><td>pfam01663, Phosphodiest, Typ</td><td>gb|ENN73120.1|</td><td>hypothetical protein YQE_10261, partial [Dendroctonus ponderosae]</td><td>8,00E-88</td></tr>
<tr><td>gALL_v3_c15611</td><td>3</td><td>1</td><td>-</td><td>-</td><td>ref|XP_970684.2|</td><td>PREDICTED: similar to conserved hypothetical protein [Tribolium castaneum]</td><td>4,00E-49</td></tr>
<tr><td>gALL_v3_c15612</td><td>2</td><td>3</td><td>-</td><td>-</td><td>gb|AGM32905.1|</td><td>hypothetical protein [Coptotermes formosanus]</td><td>2,00E-06</td></tr>
<tr><td>gALL_v3_c15613</td><td>6</td><td>7</td><td>gnl|CDD|128654</td><td>smart00360, RRM, RNA recogr</td><td>ref|XP_001866838.1|</td><td>conserved hypothetical protein [Culex quinquefasciatus] gb|EDS43986.1| conserved hypothetical protein [Culex quinquefasciatus]</td><td>1,00E-31</td></tr>
<tr><td>gALL_v3_c15614</td><td>5</td><td>5</td><td>-</td><td>-</td><td>ref|XP_003248558.1|</td><td>PREDICTED: KRAB-A domain-containing protein 2-like, partial [Acyrthosiphon pisum]</td><td>3,00E-06</td></tr>
<tr><td>gALL_v3_c15615</td><td>3</td><td>9</td><td>gnl|CDD|162300</td><td>TIGR01317, GOGAT_sm_gam,</td><td>gb|AGC92241.1|</td><td>putative glutamate synthase, partial [Laodelphax striatella]</td><td>1,00E-113</td></tr>
<tr><td>gALL_v3_c15616</td><td>1</td><td>4</td><td>-</td><td>-</td><td>-</td><td>-</td><td>-</td></tr>
<tr><td>gALL_v3_c15617</td><td>3</td><td>3</td><td>-</td><td>-</td><td>-</td><td>-</td><td>-</td></tr>
<tr><td>gALL_v3_c15618</td><td>5</td><td>2</td><td>-</td><td>-</td><td>-</td><td>-</td><td>-</td></tr>
<tr><td>gALL_v3_c15619</td><td>2</td><td>2</td><td>gnl|CDD|150870</td><td>pfam10264, Stork_head, Wing</td><td>ref|XP_003704142.1|</td><td>PREDICTED: uncharacterized protein LOC100883667 [Megachile rotundata]</td><td>2,00E-66</td></tr>
<tr><td>gALL_v3_c1562</td><td>12</td><td>9</td><td>gnl|CDD|191845</td><td>pfam07774, DUF1620, Protein</td><td>ref|XP_001604804.2|</td><td>PREDICTED: uncharacterized protein KIAA0090-like [Nasonia vitripennis]</td><td>1,00E-116</td></tr>
<tr><td>gALL_v3_c15620</td><td>1</td><td>3</td><td>gnl|CDD|143985</td><td>pfam00232, Glyco_hydro_1, G</td><td>pdb|3AHZ|A</td><td>Chain A, Crystal Structure Of Beta-Glucosidase From Termite Neotermes Koshunensis In Complex With Tris pdb|3VIF|A Chain A, Crystal Structure Of Beta-glucosidase From</td><td>1,00E-106</td></tr>
<tr><td>gALL_v3_c15622</td><td>3</td><td>1</td><td>-</td><td>-</td><td>ref|XP_003241752.1|</td><td>PREDICTED: hypothetical protein LOC100568495 [Acyrthosiphon pisum]</td><td>2,00E-07</td></tr>
<tr><td>gALL_v3_c15623</td><td>2</td><td>4</td><td>-</td><td>-</td><td>-</td><td>-</td><td>-</td></tr>
<tr><td>gALL_v3_c15625</td><td>3</td><td>3</td><td>-</td><td>-</td><td>gb|EKC19760.1|</td><td>Serine/threonine-protein kinase Pim-3 [Crassostrea gigas]</td><td>3,00E-06</td></tr>
<tr><td>gALL_v3_c15627</td><td>6</td><td>7</td><td>-</td><td>-</td><td>-</td><td>-</td><td>-</td></tr>
<tr><td>gALL_v3_c15628</td><td>1</td><td>6</td><td>-</td><td>-</td><td>-</td><td>-</td><td>-</td></tr>
<tr><td>gALL_v3_c15629</td><td>4</td><td>3</td><td>-</td><td>-</td><td>-</td><td>-</td><td>-</td></tr>
<tr><td>gALL_v3_c1563</td><td>18</td><td>12</td><td>-</td><td>-</td><td>-</td><td>-</td><td>-</td></tr>
<tr><td>gALL_v3_c15630</td><td>6</td><td>3</td><td>-</td><td>-</td><td>-</td><td>-</td><td>-</td></tr>
<tr><td>gALL_v3_c15631</td><td>3</td><td>6</td><td>gnl|CDD|153341</td><td>cd07657, F-BAR_Fes_Fer, The</td><td>ref|XP_971555.1|</td><td>PREDICTED: similar to GA21383-PA [Tribolium castaneum]</td><td>1,00E-128</td></tr>
<tr><td>gALL_v3_c15632</td><td>3</td><td>9</td><td>-</td><td>-</td><td>-</td><td>-</td><td>-</td></tr>
<tr><td>gALL_v3_c15633</td><td>5</td><td>3</td><td>gnl|CDD|178323</td><td>PLN02721, PLN02721, threoni</td><td>ref|XP_002429180.1|</td><td>L-allo-threonine aldolase, putative [Pediculus humanus corporis] gb|EEB16442.1| L-allo-threonine aldolase, putative [Pediculus humanus corporis]</td><td>1,00E-87</td></tr>
<tr><td>gALL_v3_c15634</td><td>2</td><td>3</td><td>-</td><td>-</td><td>-</td><td>-</td><td>-</td></tr>
<tr><td>gALL_v3_c15635</td><td>4</td><td>3</td><td>gnl|CDD|189434</td><td>pfam00176, SNF2_N, SNF2 far</td><td>ref|XP_001603100.2|</td><td>PREDICTED: transcriptional regulator ATRX homolog [Nasonia vitripennis]</td><td>1,00E-82</td></tr>
<tr><td>gALL_v3_c15636</td><td>1</td><td>3</td><td>-</td><td>-</td><td>-</td><td>-</td><td>-</td></tr>
<tr><td>gALL_v3_c15637</td><td>1</td><td>3</td><td>-</td><td>-</td><td>gb|ERL90571.1|</td><td>hypothetical protein D910_07918, partial [Dendroctonus ponderosae]</td><td>1,00E-09</td></tr>
<tr><td>gALL_v3_c15638</td><td>3</td><td>3</td><td>-</td><td>-</td><td>-</td><td>-</td><td>-</td></tr>
<tr><td>gALL_v3_c15639</td><td>3</td><td>2</td><td>-</td><td>-</td><td>-</td><td>-</td><td>-</td></tr>
<tr><td>gALL_v3_c15640</td><td>2</td><td>3</td><td>-</td><td>-</td><td>-</td><td>-</td><td>-</td></tr>
<tr><td>gALL_v3_c15641</td><td>3</td><td>9</td><td>-</td><td>-</td><td>-</td><td>-</td><td>-</td></tr>
<tr><td>gALL_v3_c15642</td><td>2</td><td>6</td><td>-</td><td>-</td><td>-</td><td>-</td><td>-</td></tr>
<tr><td>gALL_v3_c15645</td><td>0</td><td>4</td><td>-</td><td>-</td><td>-</td><td>-</td><td>-</td></tr>
<tr><td>gALL_v3_c15646</td><td>3</td><td>11</td><td>-</td><td>-</td><td>-</td><td>-</td><td>-</td></tr>
<tr><td>gALL_v3_c15647</td><td>4</td><td>7</td><td>-</td><td>-</td><td>ref|XP_006616365.1|</td><td>PREDICTED: uncharacterized protein LOC102671488 [Apis dorsata]</td><td>1,00E-125</td></tr>
<tr><td>gALL_v3_c15648</td><td>4</td><td>11</td><td>-</td><td>-</td><td>-</td><td>-</td><td>-</td></tr>
<tr><td>gALL_v3_c15649</td><td>2</td><td>10</td><td>-</td><td>-</td><td>-</td><td>-</td><td>-</td></tr>
<tr><td>gALL_v3_c1565</td><td>15</td><td>10</td><td>gnl|CDD|147569</td><td>pfam05456, eIF_4EBP, Eukaryo</td><td>ref|XP_001604271.1|</td><td>PREDICTED: eukaryotic translation initiation factor 4E-binding protein 1-like [Nasonia vitripennis]</td><td>5,00E-23</td></tr>
<tr><td>gALL_v3_c15650</td><td>4</td><td>4</td><td>-</td><td>-</td><td>-</td><td>-</td><td>-</td></tr>
<tr><td>gALL_v3_c15651</td><td>4</td><td>2</td><td>-</td><td>-</td><td>-</td><td>-</td><td>-</td></tr>
<tr><td>gALL_v3_c15652</td><td>4</td><td>4</td><td>-</td><td>-</td><td>-</td><td>-</td><td>-</td></tr>
<tr><td>gALL_v3_c15653</td><td>0</td><td>4</td><td>-</td><td>-</td><td>-</td><td>-</td><td>-</td></tr>
<tr><td>gALL_v3_c15654</td><td>3</td><td>3</td><td>-</td><td>-</td><td>ref|XP_003705053.1|</td><td>PREDICTED: diacylglycerol kinase 1-like [Megachile rotundata]</td><td>2,00E-67</td></tr>
<tr><td>gALL_v3_c15655</td><td>0</td><td>5</td><td>gnl|CDD|177159</td><td>MTH00097, ND6, NADH dehyc</td><td>-</td><td>-</td><td>-</td></tr>
<tr><td>gALL_v3_c15656</td><td>4</td><td>2</td><td>-</td><td>-</td><td>-</td><td>-</td><td>-</td></tr>
<tr><td>gALL_v3_c15657</td><td>3</td><td>4</td><td>-</td><td>-</td><td>ref|XP_003485679.1|</td><td>PREDICTED: hypothetical protein LOC100744877 [Bombus impatiens]</td><td>1,00E-27</td></tr>
<tr><td>gALL_v3_c15658</td><td>5</td><td>3</td><td>-</td><td>-</td><td>-</td><td>-</td><td>-</td></tr>
<tr><td>gALL_v3_c15659</td><td>2</td><td>4</td><td>-</td><td>-</td><td>-</td><td>-</td><td>-</td></tr>
<tr><td>gALL_v3_c1566</td><td>7</td><td>10</td><td>-</td><td>-</td><td>-</td><td>-</td><td>-</td></tr>
<tr><td>gALL_v3_c15660</td><td>6</td><td>14</td><td>-</td><td>-</td><td>-</td><td>-</td><td>-</td></tr>
<tr><td>gALL_v3_c15661</td><td>0</td><td>5</td><td>-</td><td>-</td><td>-</td><td>-</td><td>-</td></tr>
<tr><td>gALL_v3_c15662</td><td>3</td><td>8</td><td>-</td><td>-</td><td>-</td><td>-</td><td>-</td></tr>
<tr><td>gALL_v3_c15664</td><td>1</td><td>3</td><td>-</td><td>-</td><td>-</td><td>-</td><td>-</td></tr>
<tr><td>gALL_v3_c15665</td><td>1</td><td>6</td><td>-</td><td>-</td><td>-</td><td>-</td><td>-</td></tr>
<tr><td>gALL_v3_c15666</td><td>3</td><td>2</td><td>-</td><td>-</td><td>ref|XP_006617426.1|</td><td>PREDICTED: set1/Ash2 histone methyltransferase complex subunit ASH2-like isoform X5 [Apis dorsata]</td><td>1,00E-117</td></tr>
<tr><td>gALL_v3_c15667</td><td>3</td><td>1</td><td>-</td><td>-</td><td>ref|XP_002431217.1|</td><td>Signal recognition particle 72 kDa protein, putative [Pediculus humanus corporis] gb|EEB18479.1| Signal recognition particle 72 kDa protein, putative [Pediculus humanus</td><td>1,00E-55</td></tr>
<tr><td>gALL_v3_c15668</td><td>3</td><td>6</td><td>gnl|CDD|128992</td><td>smart00753, PAM, PCI/PINT as</td><td>gb|EGI59475.1|</td><td>COP9 signalosome complex subunit 3 [Acromyrmex echinatior]</td><td>1,00E-111</td></tr>
<tr><td>gALL_v3_c15669</td><td>3</td><td>2</td><td>-</td><td>-</td><td>-</td><td>-</td><td>-</td></tr>
<tr><td>gALL_v3_c1567</td><td>7</td><td>5</td><td>gnl|CDD|88440</td><td>cd05685, S1_Tex, S1_Tex: The</td><td>ref|XP_003698391.1|</td><td>PREDICTED: S1 RNA-binding domain-containing protein 1-like [Apis florea]</td><td>6,00E-14</td></tr>
<tr><td>gALL_v3_c15670</td><td>3</td><td>4</td><td>-</td><td>-</td><td>ref|XP_003491569.1|</td><td>PREDICTED: hypothetical protein LOC100742314 [Bombus impatiens]</td><td>5,00E-17</td></tr>
<tr><td>gALL_v3_c15671</td><td>9</td><td>12</td><td>gnl|CDD|189954</td><td>pfam01359, Transposase_1, Tr</td><td>ref|XP_006029730.1|</td><td>PREDICTED: histone-lysine N-methyltransferase SETMAR-like [Alligator sinensis]</td><td>1,00E-106</td></tr>
<tr><td>gALL_v3_c15672</td><td>4</td><td>7</td><td>-</td><td>-</td><td>-</td><td>-</td><td>-</td></tr>
<tr><td>gALL_v3_c15673</td><td>1</td><td>5</td><td>-</td><td>-</td><td>-</td><td>-</td><td>-</td></tr>
<tr><td>gALL_v3_c15675</td><td>0</td><td>4</td><td>-</td><td>-</td><td>-</td><td>-</td><td>-</td></tr>
<tr><td>gALL_v3_c15676</td><td>3</td><td>3</td><td>gnl|CDD|176260</td><td>cd08300, alcohol_DH_class_III</td><td>ref|XP_002738071.1|</td><td>PREDICTED: alcohol dehydrogenase class-3-like, partial [Saccoglossus kowalevskii]</td><td>4,00E-89</td></tr>
<tr><td>gALL_v3_c15677</td><td>2</td><td>3</td><td>-</td><td>-</td><td>-</td><td>-</td><td>-</td></tr>
<tr><td>gALL_v3_c15678</td><td>4</td><td>5</td><td>gnl|CDD|162130</td><td>TIGR00955, 3a01204, The Eye</td><td>ref|XP_001602320.1|</td><td>PREDICTED: ATP-binding cassette sub-family G member 4-like [Nasonia vitripennis]</td><td>2,00E-48</td></tr>
<tr><td>gALL_v3_c15679</td><td>4</td><td>5</td><td>-</td><td>-</td><td>-</td><td>-</td><td>-</td></tr>
<tr><td>gALL_v3_c1568</td><td>9</td><td>6</td><td>-</td><td>-</td><td>-</td><td>-</td><td>-</td></tr>
<tr><td>gALL_v3_c15680</td><td>16</td><td>16</td><td>-</td><td>-</td><td>-</td><td>-</td><td>-</td></tr>
<tr><td>gALL_v3_c15681</td><td>1</td><td>3</td><td>-</td><td>-</td><td>-</td><td>-</td><td>-</td></tr>
<tr><td>gALL_v3_c15682</td><td>0</td><td>4</td><td>-</td><td>-</td><td>-</td><td>-</td><td>-</td></tr>
<tr><td>gALL_v3_c15683</td><td>3</td><td>2</td><td>-</td><td>-</td><td>-</td><td>-</td><td>-</td></tr>
<tr><td>gALL_v3_c15684</td><td>3</td><td>6</td><td>-</td><td>-</td><td>dbj|BAA32795.1|</td><td>Rsp60 [Periplaneta americana]</td><td>5,00E-10</td></tr>
<tr><td>gALL_v3_c15686</td><td>3</td><td>3</td><td>-</td><td>-</td><td>-</td><td>-</td><td>-</td></tr>
<tr><td>gALL_v3_c15687</td><td>3</td><td>4</td><td>-</td><td>-</td><td>-</td><td>-</td><td>-</td></tr>
<tr><td>gALL_v3_c15688</td><td>5</td><td>12</td><td>-</td><td>-</td><td>-</td><td>-</td><td>-</td></tr>
<tr><td>gALL_v3_c1569</td><td>15</td><td>11</td><td>-</td><td>-</td><td>gb|EFN74196.1|</td><td>SEC14-like protein 2 [Camponotus floridanus]</td><td>4,00E-29</td></tr>
<tr><td>gALL_v3_c15690</td><td>0</td><td>5</td><td>-</td><td>-</td><td>-</td><td>-</td><td>-</td></tr>
<tr><td>gALL_v3_c15691</td><td>1</td><td>3</td><td>gnl|CDD|128491</td><td>smart00194, PTPc, Protein tyr</td><td>gb|EFA11051.1|</td><td>hypothetical protein TcasGA2_TC004645 [Tribolium castaneum]</td><td>9,00E-76</td></tr>
<tr><td>gALL_v3_c15692</td><td>2</td><td>2</td><td>gnl|CDD|109812</td><td>pfam00769, ERM, Ezrin/radixi</td><td>gb|EFZ14726.1|</td><td>hypothetical protein SINV_05434 [Solenopsis invicta]</td><td>1,00E-49</td></tr>
<tr><td>gALL_v3_c15693</td><td>5</td><td>2</td><td>-</td><td>-</td><td>-</td><td>-</td><td>-</td></tr>
<tr><td>gALL_v3_c15694</td><td>4</td><td>4</td><td>-</td><td>-</td><td>-</td><td>-</td><td>-</td></tr>
<tr><td>gALL_v3_c15695</td><td>27</td><td>20</td><td>gnl|CDD|29927</td><td>cd00916, Npc2_like, Niemann</td><td>ref|XP_001662215.1|</td><td>Niemann-Pick Type C-2, putative [Aedes aegypti] gb|EAT35799.1| AAEL012064-PA [Aedes aegypti]</td><td>2,00E-43</td></tr>
<tr><td>gALL_v3_c15696</td><td>4</td><td>6</td><td>-</td><td>-</td><td>-</td><td>-</td><td>-</td></tr>
<tr><td>gALL_v3_c15697</td><td>7</td><td>9</td><td>-</td><td>-</td><td>ref|XP_003425325.1|</td><td>PREDICTED: hypothetical protein LOC100679702 [Nasonia vitripennis]</td><td>5,00E-49</td></tr>
<tr><td>gALL_v3_c15698</td><td>4</td><td>5</td><td>-</td><td>-</td><td>-</td><td>-</td><td>-</td></tr>
<tr><td>gALL_v3_c157</td><td>16</td><td>30</td><td>-</td><td>-</td><td>-</td><td>-</td><td>-</td></tr>
<tr><td>gALL_v3_c1570</td><td>3</td><td>5</td><td>-</td><td>-</td><td>gb|EFN73941.1|</td><td>RING finger protein 31 [Camponotus floridanus]</td><td>5,00E-12</td></tr>
<tr><td>gALL_v3_c15700</td><td>1</td><td>5</td><td>-</td><td>-</td><td>gb|ESP02746.1|</td><td>hypothetical protein LOTGIDRAFT_100018, partial [Lottia gigantea]</td><td>8,00E-06</td></tr>
<tr><td>gALL_v3_c15701</td><td>2</td><td>6</td><td>-</td><td>-</td><td>-</td><td>-</td><td>-</td></tr>
</table>

gALL_v3_c15702	2	3	-	-	gb|EFN68567.1|	Potassium voltage-gated channel subfamily H member 7 [Camponotus floridanus]	8,00E-26
gALL_v3_c15703	5	7	-	-	ref|XP_002430255.1|	conserved hypothetical protein [Pediculus humanus corporis] gb|EEB17517.1| conserved hypothetical protein [Pediculus humanus corporis]	4,00E-54
gALL_v3_c15706	5	4	gnl|CDD|189372	pfam00067, p450, Cytochrom	gb|AAC03111.2|	family 4 cytochrome P450 [Coptotermes acinaciformis]	1,00E-150
gALL_v3_c15707	5	2	gnl|CDD|73156	cd01650, RT_nLTR_like, RT_nL	gb|ELU12805.1|	hypothetical protein CAPTEDRAFT_214513, partial [Capitella teleta]	2,00E-16
gALL_v3_c15709	4	4	-	-	-	-	-
gALL_v3_c1571	12	10	-	-	ref|XP_003399027.1|	PREDICTED: nuclear receptor-binding protein homolog [Bombus terrestris]	9,00E-56
gALL_v3_c15710	1	6	gnl|CDD|73080	cd02674, Peptidase_C19R, A s	ref|XP_002424409.1|	predicted protein [Pediculus humanus corporis] gb|EEB11671.1| predicted protein [Pediculus humanus corporis]	3,00E-19
gALL_v3_c15711	1	4	-	-	-	-	-
gALL_v3_c15712	2	4	-	-	gb|EFX89113.1|	hypothetical protein DAPPUDRAFT_304658 [Daphnia pulex]	1,00E-08
gALL_v3_c15714	4	2	-	-	-	-	-
gALL_v3_c15715	2	3	-	-	-	-	-
gALL_v3_c15716	7	5	-	-	-	-	-
gALL_v3_c15717	1	7	-	-	dbj|BAN21317.1|	hypothetical protein [Riptortus pedestris]	2,00E-38
gALL_v3_c15718	3	3	-	-	-	-	-
gALL_v3_c15719	0	4	-	-	-	-	-
gALL_v3_c15720	6	12	-	-	-	-	-
gALL_v3_c15721	5	4	-	-	-	-	-
gALL_v3_c15723	4	2	-	-	-	-	-
gALL_v3_c15724	3	5	-	-	-	-	-
gALL_v3_c15725	8	4	-	-	ref|XP_001601320.2|	PREDICTED: NFX1-type zinc finger-containing protein 1-like [Nasonia vitripennis]	1,00E-133
gALL_v3_c15726	2	3	gnl|CDD|144648	pfam01130, CD36, CD36 famil	ref|XP_001950032.1|	PREDICTED: scavenger receptor class B member 1-like [Acyrthosiphon pisum]	2,00E-28
gALL_v3_c15727	4	3	-	-	-	-	-
gALL_v3_c15728	1	5	-	-	-	-	-
gALL_v3_c15729	3	2	-	-	ref|XP_003425987.1|	PREDICTED: hypothetical protein LOC100678296 [Nasonia vitripennis]	3,00E-06
gALL_v3_c1573	11	12	gnl|CDD|192502	pfam10261, Scs3p, Inositol ph	ref|XP_004931497.1|	PREDICTED: FIT family protein CG10671-like [Bombyx mori]	2,00E-36
gALL_v3_c15730	2	4	gnl|CDD|144972	pfam01576, Myosin_tail_1, M	gb|EFN89532.1|	Myosin heavy chain, non-muscle [Harpegnathos saltator]	1,00E-45
gALL_v3_c15731	1	4	-	-	-	-	-
gALL_v3_c15732	2	4	-	-	-	-	-
gALL_v3_c15733	0	5	-	-	-	-	-
gALL_v3_c15734	3	1	-	-	-	-	-
gALL_v3_c15736	1	3	gnl|CDD|128621	smart00326, SH3, Src homolog	gb|EFN61288.1|	SH3 domain-containing RING finger protein 3 [Camponotus floridanus]	3,00E-30
gALL_v3_c15737	3	6	-	-	-	-	-
gALL_v3_c15738	2	3	-	-	-	-	-
gALL_v3_c15739	1	3	-	-	dbj|BAM35674.1|	putative reverse transcriptase [Marsupenaeus japonicus]	2,00E-19
gALL_v3_c1574	22	17	gnl|CDD|100104	cd00590, RRM, RRM (RNA rec	gb|EGI57152.1|	RNA-binding protein squid [Acromyrmex echinatior]	2,00E-76
gALL_v3_c15740	2	4	-	-	-	-	-
gALL_v3_c15742	3	3	-	-	-	-	-
gALL_v3_c15743	1	5	gnl|CDD|30641	COG0293, FtsJ, 23S rRNA meth	ref|XP_006615909.1|	PREDICTED: pre-rRNA processing protein FTSJ3-like isoform X1 [Apis dorsata]	1,00E-115
gALL_v3_c15744	6	3	-	-	-	-	-
gALL_v3_c15745	3	4	gnl|CDD|148211	pfam06468, Spond_N, Spondi	ref|XP_972867.1|	PREDICTED: similar to GA10108-PA [Tribolium castaneum]	1,00E-112
gALL_v3_c15746	3	5	-	-	-	-	-
gALL_v3_c15747	3	3	-	-	-	-	-
gALL_v3_c15749	4	5	gnl|CDD|191809	pfam07678, A2M_comp, A-ma	gb|EFN68790.1|	CD109 antigen [Camponotus floridanus]	2,00E-59
gALL_v3_c1575	7	6	gnl|CDD|177889	PLN02246, PLN02246, 4-coum	ref|XP_003243588.1|	PREDICTED: probable 4-coumarate--CoA ligase 3-like isoform 2 [Acyrthosiphon pisum] ref|XP_001948363.2| PREDICTED: probable 4-coumarate--CoA ligase 3-like isoform	5,00E-53
gALL_v3_c15750	2	2	-	-	-	-	-
gALL_v3_c15751	4	7	-	-	-	-	-
gALL_v3_c15752	2	3	-	-	-	-	-
gALL_v3_c15753	5	3	gnl|CDD|133321	cd04121, Rab40, Rab40 subfar	ref|XP_003698169.1|	PREDICTED: ras-related protein Rab-40C-like isoform 1 [Apis florea]	1,00E-102
gALL_v3_c15754	2	4	gnl|CDD|162403	TIGR01531, glyc_debranch, gl	ref|XP_973710.2|	PREDICTED: similar to glycogen debranching enzyme [Tribolium castaneum]	4,00E-57
gALL_v3_c15756	7	9	-	-	-	-	-
gALL_v3_c15757	1	4	-	-	-	-	-
gALL_v3_c15758	0	5	-	-	-	-	-
gALL_v3_c15759	2	3	-	-	-	-	-
gALL_v3_c1576	10	8	-	-	ref|XP_001650226.1|	hypothetical protein AaeL_AAEL005032 [Aedes aegypti] gb|EAT43557.1| AAEL005032-PA, partial [Aedes aegypti]	2,00E-21
gALL_v3_c15760	2	6	gnl|CDD|176389	cd01794, DC_UbP_C, dendritic	gb|EFN81688.1|	Ubiquitin domain-containing protein 2 [Harpegnathos saltator]	8,00E-77
gALL_v3_c15761	4	2	-	-	-	-	-
gALL_v3_c15762	3	4	-	-	-	-	-
gALL_v3_c15763	2	3	-	-	ref|XP_006610555.1|	PREDICTED: PAX-interacting protein 1-like isoform X3 [Apis dorsata]	1,00E-48
gALL_v3_c15765	4	6	-	-	-	-	-
gALL_v3_c15766	3	3	-	-	-	-	-
gALL_v3_c15768	3	1	-	-	-	-	-
gALL_v3_c15770	3	1	-	-	ref|XP_002170374.2|	PREDICTED: uncharacterized protein LOC100205880, partial [Hydra vulgaris]	3,00E-15
gALL_v3_c15771	5	2	gnl|CDD|147776	pfam05804, KAP, Kinesin-asso	ref|XP_001603246.2|	PREDICTED: kinesin-associated protein 3-like [Nasonia vitripennis]	5,00E-18
gALL_v3_c15772	5	5	-	-	-	-	-
gALL_v3_c15774	2	5	gnl|CDD|88591	cd05125, Mth938_2P1-like, M	gb|ETN62315.1|	hypothetical protein AND_005997 [Anopheles darlingi]	8,00E-49
gALL_v3_c15775	2	2	gnl|CDD|129101	smart00868, zf-AD, Zinc-finger	ref|XP_001359459.3|	GA10237 [Drosophila pseudoobscura pseudoobscura] gb|EAL28605.3| GA10237 [Drosophila pseudoobscura pseudoobscura]	8,00E-13
gALL_v3_c15776	4	2	gnl|CDD|188996	cd06457, M3A_MIP, Peptidase	gb|ETN61629.1|	mitochondrial intermediate peptidase [Anopheles darlingi]	5,00E-93
gALL_v3_c15777	3	4	-	-	-	-	-
gALL_v3_c15778	1	3	-	-	-	-	-
gALL_v3_c15779	2	5	gnl|CDD|153420	cd07920, Pumilio, Pumilio-fan	gb|AAO38522.1|	pumilio RBD [Schistocerca americana]	1,00E-102
gALL_v3_c15780	5	8	-	-	gb|ESN91435.1|	hypothetical protein HELRODRAFT_165469 [Helobdella robusta]	9,00E-15
gALL_v3_c15781	2	4	-	-	-	-	-
gALL_v3_c15783	1	8	-	-	-	-	-
gALL_v3_c15784	7	4	-	-	-	-	-
gALL_v3_c15785	2	7	-	-	-	-	-
gALL_v3_c15786	5	3	-	-	-	-	-
gALL_v3_c15789	1	3	-	-	-	-	-
gALL_v3_c1579	7	8	-	-	-	-	-
gALL_v3_c15791	1	3	-	-	-	-	-
gALL_v3_c15792	5	4	-	-	-	-	-
gALL_v3_c15793	1	5	-	-	-	-	-
gALL_v3_c15795	3	3	-	-	-	-	-
gALL_v3_c15796	6	4	gnl|CDD|128578	smart00282, LamG, Laminin G	gb|EFX60220.1|	hypothetical protein DAPPUDRAFT_344656 [Daphnia pulex]	9,00E-54
gALL_v3_c15797	2	5	-	-	-	-	-
gALL_v3_c15799	10	10	-	-	-	-	-
gALL_v3_c158	8	13	-	-	-	-	-
gALL_v3_c15800	1	4	-	-	-	-	-
gALL_v3_c15801	4	6	gnl|CDD|73156	cd01650, RT_nLTR_like, RT_nL	ref|XP_004205475.1|	PREDICTED: RNA-directed DNA polymerase from mobile element jockey-like [Hydra vulgaris]	1,00E-43
gALL_v3_c15802	5	3	-	-	-	-	-
gALL_v3_c15803	1	5	-	-	-	-	-
gALL_v3_c15804	5	2	gnl|CDD|128942	smart00702, P4Hc, Prolyl 4-hy	gb|AAM49928.1|	LD37702p [Drosophila melanogaster]	7,00E-68
gALL_v3_c15806	3	3	-	-	-	-	-
gALL_v3_c15807	4	6	-	-	gb|EGI65559.1|	hypothetical protein G5I_05949 [Acromyrmex echinatior]	4,00E-32
gALL_v3_c15808	2	2	-	-	-	-	-
gALL_v3_c15809	4	2	-	-	-	-	-
gALL_v3_c1581	11	13	-	-	ref|XP_004922428.1|	PREDICTED: inhibitor of Bruton tyrosine kinase-like [Bombyx mori]	7,00E-55
gALL_v3_c15810	0	6	-	-	-	-	-
gALL_v3_c15811	6	7	-	-	-	-	-
gALL_v3_c15812	2	4	-	-	-	-	-
gALL_v3_c15813	2	5	-	-	-	-	-
gALL_v3_c15814	2	3	gnl|CDD|189334	pfam00001, 7tm_1, 7 transme	gb|EFN86817.1|	Gustatory receptor trehalose 1 [Harpegnathos saltator]	1,00E-121
gALL_v3_c15815	3	3	gnl|CDD|29261	cd00204, ANK, ankyrin repeat:	gb|AFI09263.1|	LmrCD-specific DARPin, partial [synthetic construct]	2,00E-11
gALL_v3_c15817	4	5	-	-	-	-	-
gALL_v3_c15818	3	3	-	-	ref|XP_001868312.1|	N-acetyl lactosaminide beta-1,3-N-acetyl glucosaminyl transferase [Culex quinquefasciatus] gb|EDS26556.1| N-acetyl lactosaminide beta-1,3-N-acetyl glucosaminyl transfe	4,00E-40
gALL_v3_c15819	1	4	gnl|CDD|173673	cd05582, STKc_RSK_N, N-term	ref|XP_003246160.1|	PREDICTED: ribosomal protein S6 kinase alpha-3-like isoform 2 [Acyrthosiphon pisum]	1,00E-88
gALL_v3_c1582	8	12	gnl|CDD|133187	cd05056, PTKc_FAK, Catalytic	ref|XP_003708417.1|	PREDICTED: uncharacterized protein LOC100876637 [Megachile rotundata]	1,00E-124
gALL_v3_c15820	6	5	-	-	-	-	-
gALL_v3_c15821	4	2	gnl|CDD|192041	pfam08449, UAA, UAA transpo	gb|EGI64725.1|	Solute carrier family 35 member B1-like protein [Acromyrmex echinatior]	2,00E-65
gALL_v3_c15822	1	4	-	-	gb|AGM32136.1|	hypothetical protein [Coptotermes formosanus]	1,00E-06
gALL_v3_c15823	1	3	-	-	-	-	-
gALL_v3_c15824	3	2	-	-	-	-	-
gALL_v3_c15825	5	8	-	-	-	-	-
gALL_v3_c15826	3	3	-	-	-	-	-
gALL_v3_c15827	2	5	-	-	-	-	-
gALL_v3_c15828	6	4	-	-	-	-	-
gALL_v3_c15829	0	4	-	-	-	-	-
gALL_v3_c1583	15	10	gnl|CDD|185453	PTZ00110, PTZ00110, helicase	ref|XP_003704771.1|	PREDICTED: ATP-dependent RNA helicase abstrakt-like [Megachile rotundata]	0
gALL_v3_c15830	4	11	gnl|CDD|189954	pfam01359, Transposase_1, Tr	ref|XP_006029730.1|	PREDICTED: histone-lysine N-methyltransferase SETMAR-like [Alligator sinensis]	3,00E-68
gALL_v3_c15832	2	2	gnl|CDD|146069	pfam03250, Tropomodulin, Tr	ref|XP_003486246.1|	PREDICTED: tropomodulin-like [Bombus impatiens]	3,00E-30
gALL_v3_c15833	3	1	-	-	gb|EHJ70096.1|	hypothetical protein KGM_06661 [Danaus plexippus]	2,00E-11
gALL_v3_c15834	0	6	-	-	ref|XP_002426268.1|	protein SMG5, putative [Pediculus humanus corporis] gb|EEB13530.1| protein SMG5, putative [Pediculus humanus corporis]	1,00E-51
gALL_v3_c15835	0	5	-	-	-	-	-
gALL_v3_c15836	2	5	-	-	ref|XP_970807.1|	PREDICTED: similar to enoki mushroom CG11290-PA [Tribolium castaneum] gb|EFA08786.1| hypothetical protein TcasGA2_TC006477 [Tribolium castaneum]	5,00E-24
gALL_v3_c15837	1	6	-	-	-	-	-
gALL_v3_c15838	4	3	-	-	-	-	-
gALL_v3_c15839	3	2	-	-	-	-	-
gALL_v3_c15840	4	5	-	-	ref|XP_004932779.1|	PREDICTED: SET and MYND domain-containing protein 4-like [Bombyx mori]	2,00E-40
gALL_v3_c15841	7	5	-	-	-	-	-
gALL_v3_c15842	3	2	-	-	ref|XP_003692166.1|	PREDICTED: LOW QUALITY PROTEIN: wolframin-like [Apis florea]	5,00E-54
gALL_v3_c15843	4	2	-	-	-	-	-
gALL_v3_c15844	3	4	-	-	gb|AGM32707.1|	hypothetical protein, partial [Coptotermes formosanus]	4,00E-25
gALL_v3_c15845	2	2	-	-	-	-	-
gALL_v3_c15846	5	9	-	-	-	-	-
gALL_v3_c15847	1	3	-	-	-	-	-
gALL_v3_c15848	4	5	-	-	-	-	-
gALL_v3_c1585	17	11	gnl|CDD|188999	cd06461, M2_ACE, Peptidase	ref|XP_001659914.1|	angiotensin-converting enzyme (dipeptidyl carboxypeptidase [Aedes aegypti] gb|EAT38828.1| AAEL009316-PA, partial [Aedes aegypti]	1,00E-23
gALL_v3_c15850	4	3	-	-	gb|EHJ70532.1|	hypothetical protein KGM_09280 [Danaus plexippus]	4,00E-11
gALL_v3_c15851	3	4	-	-	-	-	-
gALL_v3_c15852	3	6	-	-	-	-	-
gALL_v3_c15854	6	5	gnl|CDD|146767	pfam04300, FBA, F-box associ	gb|EFX70375.1|	hypothetical protein DAPPUDRAFT_300481 [Daphnia pulex]	4,00E-45
gALL_v3_c15855	4	5	-	-	-	-	-
gALL_v3_c15856	2	5	-	-	-	-	-
gALL_v3_c15857	3	4	-	-	-	-	-
gALL_v3_c15858	5	4	gnl|CDD|57928	cd01857, HSR1_MMR1, HSR1/	gb|EFA00604.1|	hypothetical protein TcasGA2_TC003476 [Tribolium castaneum]	1,00E-101

gALL_v3_c1586	5	4	-	-	-	-	-
gALL_v3_c15860	0	4	-	-	-	-	-
gALL_v3_c15861	1	3	-	-	-	-	-
gALL_v3_c15862	5	9	-	-	dbj|BAN20903.1|	unkown protein [Riptortus pedestris]	4,00E-52
gALL_v3_c15863	2	5	gnl|CDD|162359	TIGR01438, TGR, thioredoxin a	ref|XP_006610499.1|	PREDICTED: thioredoxin reductase 1, mitochondrial-like isoform X3 [Apis dorsata]	7,00E-93
gALL_v3_c15864	3	1	-	-	-	-	-
gALL_v3_c15865	6	6	-	-	ref|XP_002415087.1|	muscle myosin heavy chain, putative [Ixodes scapularis] gb|EEC18752.1| muscle myosin heavy chain, putative [Ixodes scapularis]	7,00E-29
gALL_v3_c15868	3	4	-	-	ref|XP_001601133.1|	PREDICTED: ran-binding protein 9-like [Nasonia vitripennis]	2,00E-11
gALL_v3_c15869	2	5	-	-	-	-	-
gALL_v3_c1587	8	8	-	-	-	-	-
gALL_v3_c15870	2	7	-	-	ref|XP_002424868.1|	zinc finger protein, putative [Pediculus humanus corporis] gb|EEB12130.1| zinc finger protein, putative [Pediculus humanus corporis]	1,00E-84
gALL_v3_c15871	2	5	-	-	-	-	-
gALL_v3_c15874	0	4	-	-	-	-	-
gALL_v3_c15875	3	2	-	-	-	-	-
gALL_v3_c15876	2	5	-	-	-	-	-
gALL_v3_c15877	3	6	gnl|CDD|73156	cd01650, RT_nLTR_like, RT_nL	ref|XP_002385618.1|	LINE-1 retrotransposon-like element, putative [Aspergillus flavus NRRL3357] gb|EED44706.1| LINE-1 retrotransposon-like element, putative [Aspergillus flavus NRRL3357]	8,00E-58
gALL_v3_c15878	1	4	-	-	-	-	-
gALL_v3_c15879	2	4	-	-	-	-	-
gALL_v3_c1588	5	7	-	-	-	-	-
gALL_v3_c15880	4	3	gnl|CDD|189507	pfam00335, Tetraspannin, Tet	gb|AGM32481.1|	tetraspanin D107 [Coptotermes formosanus]	2,00E-45
gALL_v3_c15881	1	4	-	-	-	-	-
gALL_v3_c15882	3	2	gnl|CDD|133357	cd04157, Arl6, Arl6 subfamily.	ref|XP_975678.1|	PREDICTED: similar to ADP-ribosylation factor-like 6 (predicted) [Tribolium castaneum]	1,00E-57
gALL_v3_c15884	3	5	gnl|CDD|178676	PLN03130, PLN03130, ABC tra	ref|XP_969849.1|	PREDICTED: similar to ATP-binding cassette transporter [Tribolium castaneum]	3,00E-56
gALL_v3_c15885	6	3	-	-	ref|XP_001943228.2|	PREDICTED: thrombospondin type-1 domain-containing protein 7A [Acyrthosiphon pisum]	2,00E-71
gALL_v3_c1589	24	18	gnl|CDD|190542	pfam03145, Sina, Seven in abs	ref|XP_003385650.1|	PREDICTED: e3 ubiquitin-protein ligase Siah1-like, partial [Amphimedon queenslandica]	2,00E-27
gALL_v3_c15890	4	8	-	-	-	-	-
gALL_v3_c15891	2	6	-	-	-	-	-
gALL_v3_c15892	5	6	-	-	ref|XP_003707852.1|	PREDICTED: uncharacterized protein LOC100881694 [Megachile rotundata]	1,00E-140
gALL_v3_c15893	1	2	-	-	-	-	-
gALL_v3_c15894	2	3	gnl|CDD|112701	pfam03901, Glyco_transf_22,	ref|XP_003425903.1|	PREDICTED: alpha-1,2-mannosyltransferase ALG9-like isoform 2 [Nasonia vitripennis]	2,00E-53
gALL_v3_c15895	2	3	-	-	ref|XP_003484895.1|	PREDICTED: hypothetical protein LOC100741323 [Bombus impatiens]	3,00E-07
gALL_v3_c15896	3	5	-	-	-	-	-
gALL_v3_c15898	3	3	-	-	ref|XP_006825962.1|	PREDICTED: LOW QUALITY PROTEIN: ancient ubiquitous protein 1-like [Saccoglossus kowalevskii]	4,00E-21
gALL_v3_c15899	3	2	gnl|CDD|192384	pfam09801, SYS1, Integral me	ref|XP_002425486.1|	conserved hypothetical protein [Pediculus humanus corporis] gb|EEB12748.1| conserved hypothetical protein [Pediculus humanus corporis]	5,00E-21
gALL_v3_c159	12	11	-	-	-	-	-
gALL_v3_c1590	8	4	-	-	-	-	-
gALL_v3_c15900	2	6	-	-	-	-	-
gALL_v3_c15901	2	3	-	-	gb|EEZ99236.1|	hypothetical protein TcasGA2_TC000808 [Tribolium castaneum]	6,00E-51
gALL_v3_c15902	2	3	-	-	-	-	-
gALL_v3_c15903	6	3	-	-	-	-	-
gALL_v3_c15904	3	3	-	-	gb|EFN60509.1|	Protein FAM76A [Camponotus floridanus]	1,00E-119
gALL_v3_c15905	1	7	gnl|CDD|175999	cd04033, C2_NEDD4_NEDD4L	ref|XP_005183413.1|	PREDICTED: E3 ubiquitin-protein ligase Nedd-4-like isoform X1 [Musca domestica]	1,00E-47
gALL_v3_c15906	1	4	-	-	ref|XP_002431867.1|	reverse transcriptase, putative [Pediculus humanus corporis] gb|EEB19129.1| reverse transcriptase, putative [Pediculus humanus corporis]	7,00E-06
gALL_v3_c15907	5	9	-	-	-	-	-
gALL_v3_c15908	2	2	-	-	gb|EFA13373.1|	hypothetical protein TcasGA2_TC001390 [Tribolium castaneum]	5,00E-09
gALL_v3_c15909	4	3	-	-	gb|ENN73627.1|	hypothetical protein YQE_09874, partial [Dendroctonus ponderosae]	2,00E-08
gALL_v3_c1591	12	16	-	-	ref|XP_002430610.1|	conserved hypothetical protein [Pediculus humanus corporis] gb|EEB17872.1| conserved hypothetical protein [Pediculus humanus corporis]	2,00E-18
gALL_v3_c15910	4	2	-	-	gb|ENN82530.1|	hypothetical protein YQE_01098, partial [Dendroctonus ponderosae]	2,00E-23
gALL_v3_c15911	5	2	-	-	ref|XP_002423145.1|	U11/U12 snRNP 25 kDa protein, putative [Pediculus humanus corporis] gb|EEB10407.1| U11/U12 snRNP 25 kDa protein, putative [Pediculus humanus corporis]	1,00E-23
gALL_v3_c15912	2	2	-	-	-	-	-
gALL_v3_c15913	3	4	-	-	ref|XP_006618311.1|	PREDICTED: uncharacterized protein KIAA1109-like isoform X8 [Apis dorsata]	6,00E-48
gALL_v3_c15914	7	5	-	-	gb|EFZ14510.1|	hypothetical protein SINV_15432 [Solenopsis invicta]	3,00E-11
gALL_v3_c15915	7	6	gnl|CDD|178917	PRK00179, pgi, glucose-6-pho:	gb|ADA56787.1|	glucose-6-phosphate isomerase [Euphydryas aurinia]	2,00E-78
gALL_v3_c15916	3	3	-	-	ref|XP_003696500.1|	PREDICTED: papilin-like [Apis florea]	5,00E-99
gALL_v3_c15918	4	7	-	-	-	-	-
gALL_v3_c15919	3	5	gnl|CDD|30832	COG0484, DnaJ, DnaJ-class mc	ref|XP_003701855.1|	PREDICTED: protein tumorous imaginal discs, mitochondrial-like [Megachile rotundata]	4,00E-82
gALL_v3_c1592	11	12	-	-	-	-	-
gALL_v3_c15920	4	2	gnl|CDD|29233	cd01460, vWA_midasin, VWA_	ref|XP_003424179.1|	PREDICTED: midasin-like [Nasonia vitripennis]	7,00E-16
gALL_v3_c15921	2	2	-	-	-	-	-
gALL_v3_c15922	3	6	-	-	ref|XP_001658391.1|	hypothetical protein AaeL_AAEL007475 [Aedes aegypti] gb|EAT40842.1| AAEL007475-PA [Aedes aegypti]	5,00E-21
gALL_v3_c15923	8	14	gnl|CDD|58174	cd03508, Delta4-sphingolipid-	dbj|BAN20572.1|	sphingolipid delta 4 desaturase/c-4 hydroxylase protein des2 [Riptortus pedestris]	1,00E-102
gALL_v3_c15924	6	6	-	-	gb|EFA06217.1|	hypothetical protein TcasGA2_TC009066 [Tribolium castaneum]	2,00E-15
gALL_v3_c15925	1	3	-	-	-	-	-
gALL_v3_c15926	3	8	-	-	-	-	-
gALL_v3_c15927	1	5	-	-	-	-	-
gALL_v3_c15929	1	6	-	-	ref|XP_006624244.1|	PREDICTED: E3 ubiquitin-protein ligase HECTD1-like isoform X1 [Apis dorsata] ref|XP_006624245.1| PREDICTED: E3 ubiquitin-protein ligase HECTD1-like isoform X2 [Apis d	1,00E-125
gALL_v3_c1593	14	12	gnl|CDD|189508	pfam00337, Gal-bind_lectin, G	dbj|BAM20137.1|	unknown unsecreted protein [Papilio xuthus]	1,00E-48
gALL_v3_c15930	1	6	gnl|CDD|133318	cd04118, Rab24, Rab24 subfar	ref|XP_004284863.1|	PREDICTED: ras-related protein Rab-24 [Orcinus orca] ref|XP_004323236.1| PREDICTED: ras-related protein Rab-24 [Tursiops truncatus]	5,00E-42
gALL_v3_c15931	3	7	-	-	-	-	-
gALL_v3_c15932	0	5	-	-	-	-	-
gALL_v3_c15933	1	5	-	-	-	-	-
gALL_v3_c15934	8	4	-	-	-	-	-
gALL_v3_c15935	14	15	-	-	-	-	-
gALL_v3_c15936	3	1	gnl|CDD|183440	PRK12325, PRK12325, prolyl-t	gb|EEZ98764.1|	hypothetical protein TcasGA2_TC001328 [Tribolium castaneum]	1,00E-26
gALL_v3_c15938	0	4	-	-	ref|XP_001263507.1|	spindle-pole body protein (Pcp1), putative [Neosartorya fischeri NRRL 181] gb|EAW21610.1| spindle-pole body protein (Pcp1), putative [Neosartorya fischeri NRRL 181]	1,00E-06
gALL_v3_c15939	3	2	-	-	gb|EHJ76937.1|	putative tigger transposable element derived 6-like protein [Danaus plexippus]	2,00E-07
gALL_v3_c15940	4	3	gnl|CDD|191874	pfam07856, Orai-1, Mediator	ref|XP_006619804.1|	PREDICTED: calcium release-activated calcium channel protein 1-like [Apis dorsata]	3,00E-71
gALL_v3_c15941	5	5	-	-	gb|EFZ19369.1|	hypothetical protein SINV_02216 [Solenopsis invicta]	1,00E-95
gALL_v3_c15942	2	4	-	-	-	-	-
gALL_v3_c15943	3	6	-	-	-	-	-
gALL_v3_c15944	1	6	-	-	-	-	-
gALL_v3_c15945	1	4	gnl|CDD|190161	pfam01923, Cob_adeno_trans	gb|EGI65201.1|	Cob(I)yrinic acid a,c-diamide adenosyltransferase, mitochondrial [Acromyrmex echinatior]	2,00E-42
gALL_v3_c15946	3	3	gnl|CDD|176047	cd08402, C2B_Synaptotagmin	gb|AAK01129.1|AF3310	synaptotagmin [Manduca sexta]	7,00E-47
gALL_v3_c15947	1	3	-	-	-	-	-
gALL_v3_c15948	3	4	-	-	-	-	-
gALL_v3_c15949	9	19	gnl|CDD|73156	cd01650, RT_nLTR_like, RT_nL	dbj|BAE44464.1|	non-LTR retrotransposon CATS [Bombyx mori]	1,00E-116
gALL_v3_c1595	32	39	gnl|CDD|147618	pfam05536, Neurochondrin, N	ref|XP_004921864.1|	PREDICTED: neurochondrin homolog [Bombyx mori]	0
gALL_v3_c15950	7	6	gnl|CDD|29370	cd00288, Pyruvate_Kinase, Py	ref|XP_006566000.1|	PREDICTED: pyruvate kinase-like isoform X3 [Apis mellifera]	2,00E-93
gALL_v3_c15951	2	4	-	-	-	-	-
gALL_v3_c15952	4	2	-	-	-	-	-
gALL_v3_c15954	9	6	-	-	-	-	-
gALL_v3_c15955	1	4	gnl|CDD|149417	pfam08347, CTNNB1_binding,	ref|XP_006558800.1|	PREDICTED: protein pangolin, isoforms A/H/I/S-like isoform X3 [Apis mellifera]	1,00E-51
gALL_v3_c15956	2	4	-	-	-	-	-
gALL_v3_c15958	4	3	-	-	-	-	-
gALL_v3_c1596	1	8	gnl|CDD|116680	pfam08073, CHDNT, CHDNT (N	ref|XP_006564625.1|	PREDICTED: chromodomain-helicase-DNA-binding protein Mi-2 homolog isoform X1 [Apis mellifera]	8,00E-40
gALL_v3_c15960	3	4	-	-	-	-	-
gALL_v3_c15961	6	10	-	-	-	-	-
gALL_v3_c15962	3	1	-	-	-	-	-
gALL_v3_c15963	10	9	-	-	gb|EFN64049.1|	hypothetical protein EAG_08835 [Camponotus floridanus]	1,00E-10
gALL_v3_c15964	3	2	-	-	-	-	-
gALL_v3_c15965	2	4	-	-	-	-	-
gALL_v3_c15966	0	6	-	-	-	-	-
gALL_v3_c15967	7	7	gnl|CDD|189378	pfam00079, Serpin, Serpin (se	gb|ENN74325.1|	hypothetical protein YQE_09296, partial [Dendroctonus ponderosae] gb|ERL95537.1| hypothetical protein D910_12798 [Dendroctonus ponderosae]	2,00E-33
gALL_v3_c15968	4	3	gnl|CDD|129058	smart00825, PKS_KS, Beta-ket	gb|EFZ09689.1|	hypothetical protein SINV_13910 [Solenopsis invicta]	8,00E-89
gALL_v3_c15969	3	6	-	-	ref|XP_001600404.1|	PREDICTED: RCC1 and BTB domain-containing protein 1-like [Nasonia vitripennis]	2,00E-16
gALL_v3_c1597	18	18	gnl|CDD|178963	PRK00290, dnaK, molecular ch	ref|XP_002428084.1|	Heat shock 70 kDa protein cognate, putative [Pediculus humanus corporis] gb|EEB15346.1| Heat shock 70 kDa protein cognate, putative [Pediculus humanus corporis]	0
gALL_v3_c15970	3	1	-	-	ref|XP_001654993.1|	hypothetical protein AaeL_AAEL002179 [Aedes aegypti] gb|EAT46653.1| AAEL002179-PA [Aedes aegypti]	1,00E-16
gALL_v3_c15971	3	5	-	-	ref|XP_004211631.1|	PREDICTED: sialidase-like, partial [Hydra vulgaris]	7,00E-12
gALL_v3_c15972	5	3	-	-	-	-	-
gALL_v3_c15973	1	4	gnl|CDD|147872	pfam05955, Herpes_gp2, Equi	-	-	-
gALL_v3_c15974	2	3	-	-	-	-	-
gALL_v3_c15976	4	9	gnl|CDD|110585	pfam01591, 6PF2K, 6-phosphc	gb|EFA09842.1|	hypothetical protein TcasGA2_TC011990 [Tribolium castaneum]	9,00E-78
gALL_v3_c15978	9	5	gnl|CDD|153420	cd07920, Pumilio, Pumilio-far	gb|ERL92240.1|	hypothetical protein D910_09557 [Dendroctonus ponderosae]	2,00E-37
gALL_v3_c15979	1	3	-	-	-	-	-
gALL_v3_c15980	2	3	-	-	ref|XP_002423335.1|	conserved hypothetical protein [Pediculus humanus corporis] gb|EEB10597.1| conserved hypothetical protein [Pediculus humanus corporis]	9,00E-14
gALL_v3_c15981	2	3	gnl|CDD|173625	cd05032, PTKc_InsR_like, Cata	gb|EHJ65074.1|	insulin receptor [Danaus plexippus]	9,00E-16
gALL_v3_c15982	1	7	-	-	-	-	-
gALL_v3_c15983	3	2	-	-	dbj|BAM19230.1|	unknown unsecreted protein [Papilio polytes]	9,00E-12
gALL_v3_c15984	4	2	-	-	-	-	-
gALL_v3_c15985	1	5	-	-	ref|XP_003973856.1|	PREDICTED: protein SZT2-like [Takifugu rubripes]	4,00E-10
gALL_v3_c15986	5	4	-	-	ref|XP_002431865.1|	conserved hypothetical protein [Pediculus humanus corporis] gb|EEB19127.1| conserved hypothetical protein [Pediculus humanus corporis]	2,00E-11
gALL_v3_c15987	4	4	-	-	-	-	-
gALL_v3_c1599	13	15	-	-	-	-	-
gALL_v3_c15991	0	6	-	-	-	-	-
gALL_v3_c15992	4	3	-	-	ref|XP_001600072.2|	PREDICTED: RING finger and CHY zinc finger domain-containing protein 1-like [Nasonia vitripennis]	7,00E-23
gALL_v3_c15993	3	1	-	-	-	-	-
gALL_v3_c15995	3	4	gnl|CDD|189523	pfam00380, Ribosomal_S9, Ril	ref|XP_001870809.1|	28S ribosomal protein S9, mitochondrial [Culex quinquefasciatus] gb|EDS34191.1| 28S ribosomal protein S9, mitochondrial [Culex quinquefasciatus]	1,00E-114
gALL_v3_c15996	1	7	-	-	gb|EGI65748.1|	hypothetical protein G5I_05849 [Acromyrmex echinatior]	4,00E-09
gALL_v3_c15997	1	4	-	-	-	-	-
gALL_v3_c15998	5	9	-	-	-	-	-
gALL_v3_c15999	2	3	-	-	-	-	-
gALL_v3_c16	11	6	-	-	ref|XP_004932134.1|	PREDICTED: uncharacterized protein LOC101737699 [Bombyx mori]	2,00E-23
gALL_v3_c160	11	24	-	-	gb|EFA13258.1|	hypothetical protein TcasGA2_TC001528 [Tribolium castaneum]	2,00E-29
gALL_v3_c1600	6	9	gnl|CDD|189502	pfam00326, Peptidase_S9, Prc	gb|ETE68797.1|	Prolyl endopeptidase, partial [Ophiophagus hannah]	4,00E-39
gALL_v3_c16000	1	4	gnl|CDD|128725	smart00449, SPRY, Domain in :	gb|EFN81004.1|	RING finger and SPRY domain-containing protein 1 [Harpegnathos saltator]	1,00E-116
gALL_v3_c16002	4	4	-	-	-	-	-
gALL_v3_c16003	4	5	-	-	gb|EKC39344.1|	Transforming growth factor-beta receptor-associated protein 1 [Crassostrea gigas]	3,00E-70
gALL_v3_c16004	4	2	-	-	ref|XP_001651937.1|	epoxide hydrolase [Aedes aegypti] gb|EAT42097.1| AAEL006342-PA [Aedes aegypti]	4,00E-29
gALL_v3_c16006	3	3	-	-	-	-	-
gALL_v3_c16009	5	9	-	-	-	-	-
gALL_v3_c1601	5	14	-	-	-	-	-
gALL_v3_c16011	4	6	-	-	-	-	-
gALL_v3_c16012	2	7	gnl|CDD|48621	cd03072, PDI_b'_ERp44, PDIb'	ref|XP_971182.1|	PREDICTED: similar to CG9911 CG9911-PA [Tribolium castaneum] gb|EFA09317.1| hypothetical protein TcasGA2_TC001643 [Tribolium castaneum]	6,00E-69
gALL_v3_c16013	2	4	-	-	-	-	-

gALL_v3_c16014	3	2	-	-	-	-	-
gALL_v3_c16015	5	4	-	-	-	-	-
gALL_v3_c16016	2	6	-	-	-	-	-
gALL_v3_c16017	4	5	-	-	ref|XP_002429372.1|	myosin XV, putative [Pediculus humanus corporis] gb|EEB16634.1| myosin XV, putative [Pediculus humanus corporis]	3,00E-52
gALL_v3_c16018	3	4	-	-	-	-	-
gALL_v3_c16019	1	7	-	-	-	-	-
gALL_v3_c1602	4	5	gnl|CDD|190277	pfam02320, UCR_hinge, Ubiqu	ref|XP_969197.1|	PREDICTED: similar to AGAP002245-PA [Tribolium castaneum] gb|EFA05661.1| hypothetical protein TcasGA2_TC014895 [Tribolium castaneum]	5,00E-30
gALL_v3_c16020	5	3	gnl|CDD|31423	COG1230, CzcD, Co/Zn/Cd effl	gb|ELT96504.1|	hypothetical protein CAPTEDRAFT_20619 [Capitella teleta]	1,00E-38
gALL_v3_c16021	2	3	-	-	-	-	-
gALL_v3_c16022	2	5	-	-	ref|XP_974348.2|	PREDICTED: similar to GA13736-PA [Tribolium castaneum]	3,00E-52
gALL_v3_c16023	3	3	gnl|CDD|118676	pfam10148, SCHIP-1, Schwanr	ref|XP_006571595.1|	PREDICTED: ras guanine nucleotide exchange factor B-like isoform X3 [Apis mellifera]	1,00E-28
gALL_v3_c16024	3	4	-	-	-	-	-
gALL_v3_c16025	8	11	-	-	ref|XP_003699977.1|	PREDICTED: uncharacterized protein LOC100880926 [Megachile rotundata]	1,00E-71
gALL_v3_c16026	3	8	-	-	ref|XP_970727.1|	PREDICTED: similar to AGAP009860-PA [Tribolium castaneum]	3,00E-76
gALL_v3_c16027	6	13	-	-	-	-	-
gALL_v3_c16028	1	6	-	-	-	-	-
gALL_v3_c16029	3	4	-	-	-	-	-
gALL_v3_c1603	49	42	-	-	gb|EFN67634.1|	Putative uncharacterized transposon-derived protein F52C9.6 [Camponotus floridanus]	7,00E-28
gALL_v3_c16030	2	2	-	-	-	-	-
gALL_v3_c16031	4	12	gnl|CDD|48562	cd03013, PRX5_like, Peroxired	gb|AFZ78682.1|	peroxiredoxin-like protein [Coptotermes formosanus]	1,00E-115
gALL_v3_c16032	3	6	-	-	-	-	-
gALL_v3_c16033	3	1	gnl|CDD|28913	cd00031, CA, Cadherin repeat	ref|XP_003705963.1|	PREDICTED: DE-cadherin-like [Megachile rotundata]	4,00E-39
gALL_v3_c16035	1	3	-	-	-	-	-
gALL_v3_c16036	4	6	-	-	gb|EFN70549.1|	Proteasome assembly chaperone 2 [Camponotus floridanus]	3,00E-14
gALL_v3_c16037	0	6	-	-	-	-	-
gALL_v3_c16038	0	5	-	-	-	-	-
gALL_v3_c16039	7	5	-	-	-	-	-
gALL_v3_c1604	18	15	gnl|CDD|177889	PLN02246, PLN02246, 4-coum	ref|XP_001844398.1|	AMP dependent coa ligase [Culex quinquefasciatus] gb|EDS36895.1| AMP dependent coa ligase [Culex quinquefasciatus]	2,00E-90
gALL_v3_c16040	2	3	-	-	ref|XP_972290.1|	PREDICTED: similar to AGAP002331-PA [Tribolium castaneum] gb|EFA07819.1| hypothetical protein TcasGA2_TC005388 [Tribolium castaneum]	9,00E-62
gALL_v3_c16041	2	5	-	-	-	-	-
gALL_v3_c16043	2	3	-	-	-	-	-
gALL_v3_c16044	3	5	-	-	-	-	-
gALL_v3_c16045	3	1	-	-	ref|XP_006822699.1|	PREDICTED: LOW QUALITY PROTEIN: low-density lipoprotein receptor-related protein 6-like [Saccoglossus kowalevskii]	1,00E-39
gALL_v3_c16046	2	4	-	-	ref|XP_002426213.1|	O-linked N-acetylglucosamine transferase, ogt, putative [Pediculus humanus corporis] gb|EEB13475.1| O-linked N-acetylglucosamine transferase, ogt, putative [Pediculus h	2,00E-33
gALL_v3_c16047	1	7	gnl|CDD|190038	pfam01562, Pep_M12B_prope	ref|XP_003486941.1|	PREDICTED: disintegrin and metalloproteinase domain-containing protein 10-like [Bombus impatiens]	1,00E-108
gALL_v3_c16048	1	6	gnl|CDD|189373	pfam00069, Pkinase, Protein k	dbj|BAN20662.1|	TGF-beta receptor type-1 [Riptortus pedestris]	3,00E-94
gALL_v3_c16049	2	3	-	-	-	-	-
gALL_v3_c1605	7	13	-	-	ref|XP_002429741.1|	regulator of G protein signaling, putative [Pediculus humanus corporis] gb|EEB17003.1| regulator of G protein signaling, putative [Pediculus humanus corporis]	3,00E-95
gALL_v3_c16050	2	2	-	-	ref|XP_002432716.1|	Cyclin-G2, putative [Pediculus humanus corporis] gb|EEB19978.1| Cyclin-G2, putative [Pediculus humanus corporis]	2,00E-38
gALL_v3_c16051	3	3	-	-	ref|XP_002424722.1|	conserved hypothetical protein [Pediculus humanus corporis] gb|EEB11984.1| conserved hypothetical protein [Pediculus humanus corporis]	3,00E-28
gALL_v3_c16052	2	3	-	-	ref|XP_002429033.1|	reverse transcriptase, putative [Pediculus humanus corporis] gb|EEB16295.1| reverse transcriptase, putative [Pediculus humanus corporis]	5,00E-18
gALL_v3_c16053	3	2	gnl|CDD|128671	smart00389, HOX, Homeodom	ref|XP_002431193.1|	Homeobox protein SIX1, putative [Pediculus humanus corporis] gb|EEB18455.1| Homeobox protein SIX1, putative [Pediculus humanus corporis]	6,00E-65
gALL_v3_c16055	3	2	-	-	gb|EFN81295.1|	Protein CLEC16A [Harpegnathos saltator]	1,00E-38
gALL_v3_c16057	0	5	-	-	-	-	-
gALL_v3_c16058	2	4	-	-	-	-	-
gALL_v3_c16059	1	3	-	-	-	-	-
gALL_v3_c16060	7	5	-	-	-	-	-
gALL_v3_c16062	0	4	-	-	-	-	-
gALL_v3_c16063	0	3	-	-	ref|XP_001952799.2|	PREDICTED: rho GTPase-activating protein 20-like [Acyrthosiphon pisum]	8,00E-29
gALL_v3_c16064	5	2	-	-	-	-	-
gALL_v3_c16065	9	8	gnl|CDD|128777	smart00501, BRIGHT, BRIGHT,	gb|ETN59833.1|	hypothetical protein AND_008556 [Anopheles darlingi]	5,00E-73
gALL_v3_c16066	4	3	gnl|CDD|29630	cd00780, NTF2, Nuclear trans	ref|XP_392921.1|	PREDICTED: probable nuclear transport factor 2-like isoform 3 [Apis mellifera] ref|XP_003691053.1| PREDICTED: probable nuclear transport factor 2-like [Apis florea] ref|X	3,00E-27
gALL_v3_c16067	2	3	-	-	gb|AAB61714.1|	putative transposase [Homo sapiens]	3,00E-09
gALL_v3_c16068	3	3	-	-	-	-	-
gALL_v3_c16069	4	2	-	-	-	-	-
gALL_v3_c1607	7	10	-	-	ref|XP_002426193.1|	hypothetical protein Phum_PHUM240790 [Pediculus humanus corporis] gb|EEB13455.1| hypothetical protein Phum_PHUM240790 [Pediculus humanus corporis]	2,00E-24
gALL_v3_c16070	9	8	-	-	ref|XP_006612801.1|	PREDICTED: uncharacterized protein LOC102678925 [Apis dorsata]	3,00E-30
gALL_v3_c16071	2	2	-	-	-	-	-
gALL_v3_c16072	2	7	-	-	ref|XP_003489649.1|	PREDICTED: hemicentin-1-like isoform 2 [Bombus impatiens]	2,00E-70
gALL_v3_c16074	1	8	-	-	-	-	-
gALL_v3_c16075	8	5	gnl|CDD|145901	pfam02996, Prefoldin, Prefold	ref|XP_001599491.1|	PREDICTED: prefoldin subunit 3-like [Nasonia vitripennis]	4,00E-77
gALL_v3_c16076	2	3	-	-	ref|XP_973242.1|	PREDICTED: similar to CG3808 CG3808-PA [Tribolium castaneum] gb|EFA08275.1| hypothetical protein TcasGA2_TC005909 [Tribolium castaneum]	2,00E-42
gALL_v3_c16077	3	2	-	-	-	-	-
gALL_v3_c16078	0	4	-	-	-	-	-
gALL_v3_c16079	2	4	-	-	-	-	-
gALL_v3_c1608	16	13	-	-	-	-	-
gALL_v3_c16080	11	21	-	-	-	-	-
gALL_v3_c16081	2	4	gnl|CDD|29397	cd01926, cyclophilin_ABH_like	gb|AEV89763.1|	peptidyl-prolyl cis-trans isomerase, partial [Schistocerca gregaria]	4,00E-64
gALL_v3_c16082	2	3	-	-	-	-	-
gALL_v3_c16083	2	4	gnl|CDD|128349	smart00034, CLECT, C-type lec	ref|XP_001949468.2|	PREDICTED: hypothetical protein LOC100162025 [Acyrthosiphon pisum]	1,00E-124
gALL_v3_c16084	7	8	-	-	ref|XP_003393804.1|	PREDICTED: hypothetical protein LOC100642278 [Bombus terrestris]	1,00E-16
gALL_v3_c16085	0	6	-	-	-	-	-
gALL_v3_c16087	4	10	-	-	-	-	-
gALL_v3_c16088	2	4	-	-	-	-	-
gALL_v3_c16089	1	4	-	-	-	-	-
gALL_v3_c16091	4	4	gnl|CDD|191640	pfam06916, DUF1279, Protein	gb|ERL91488.1|	hypothetical protein D910_08818 [Dendroctonus ponderosae]	5,00E-24
gALL_v3_c16092	1	3	-	-	ref|XP_002423426.1|	grb2-associated binder, gab, putative [Pediculus humanus corporis] gb|EEB10688.1| grb2-associated binder, gab, putative [Pediculus humanus corporis]	9,00E-36
gALL_v3_c16093	4	4	gnl|CDD|128856	smart00588, NEUZ, domain in	ref|XP_001601781.2|	PREDICTED: neuralized-like protein 4-like [Nasonia vitripennis]	1,00E-115
gALL_v3_c16094	3	3	gnl|CDD|145051	pfam01697, DUF23, Domain o	ref|XP_968476.1|	PREDICTED: similar to CG9395 CG9395-PA [Tribolium castaneum] gb|EFA07701.1| hypothetical protein TcasGA2_TC002177 [Tribolium castaneum]	2,00E-89
gALL_v3_c16095	4	3	-	-	-	-	-
gALL_v3_c16097	3	5	-	-	-	-	-
gALL_v3_c16098	3	4	-	-	-	-	-
gALL_v3_c16099	4	2	-	-	ref|XP_006610962.1|	PREDICTED: LOW QUALITY PROTEIN: transformation/transcription domain-associated protein-like [Apis dorsata]	4,00E-90
gALL_v3_c1610	10	15	-	-	-	-	-
gALL_v3_c16102	5	7	-	-	-	-	-
gALL_v3_c16103	5	2	-	-	-	-	-
gALL_v3_c16104	3	3	-	-	-	-	-
gALL_v3_c16105	2	6	-	-	-	-	-
gALL_v3_c16106	3	4	-	-	ref|XP_003489740.1|	PREDICTED: LOW QUALITY PROTEIN: histone deacetylase 4-like [Bombus impatiens]	6,00E-06
gALL_v3_c16107	1	3	gnl|CDD|143833	pfam00046, Homeobox, Home	gb|EFX89655.1|	putative zinc finger homeobox protein 3 [Daphnia pulex]	3,00E-27
gALL_v3_c16108	2	3	-	-	-	-	-
gALL_v3_c16109	3	8	gnl|CDD|30108	cd01386, MYSc_type_XVIII, M	ref|XP_001604740.2|	PREDICTED: myosin-XVIIIa isoform 1 [Nasonia vitripennis]	1,00E-151
gALL_v3_c1611	3	8	-	-	ref|XP_001605486.2|	PREDICTED: hypothetical protein LOC100121879 [Nasonia vitripennis]	3,00E-20
gALL_v3_c16110	5	2	-	-	-	-	-
gALL_v3_c16111	3	2	-	-	-	-	-
gALL_v3_c16112	0	4	-	-	gb|EFN73722.1|	Putative uncharacterized protein FLJ37770 [Camponotus floridanus]	2,00E-08
gALL_v3_c16113	7	7	-	-	ref|XP_003242319.1|	PREDICTED: histone-lysine N-methyltransferase SETMAR-like [Acyrthosiphon pisum]	1,00E-59
gALL_v3_c16114	2	5	-	-	-	-	-
gALL_v3_c16115	2	4	gnl|CDD|30768	COG0419, SbcC, ATPase involv	ref|XP_001955240.1|	GF18661 [Drosophila ananassae] gb|EDV43801.1| GF18661 [Drosophila ananassae]	5,00E-09
gALL_v3_c16116	4	2	-	-	gb|AGM32626.1|	glucosyl/glucuronosyl transferase, partial [Coptotermes formosanus]	2,00E-07
gALL_v3_c16117	0	4	-	-	-	-	-
gALL_v3_c16118	6	5	gnl|CDD|191869	pfam07842, GCFC, GC-rich sec	ref|XP_003393588.1|	PREDICTED: GC-rich sequence DNA-binding factor 1-like [Bombus terrestris]	1,00E-140
gALL_v3_c1612	23	20	gnl|CDD|162787	TIGR02253, CTE7, HAD superfa	ref|XP_004921865.1|	PREDICTED: N-acylneuraminate-9-phosphatase-like [Bombyx mori]	6,00E-25
gALL_v3_c16120	10	6	gnl|CDD|189452	pfam00211, Guanylate_cyc, Ad	ref|XP_003702098.1|	PREDICTED: adenylate cyclase type 2-like [Megachile rotundata]	3,00E-72
gALL_v3_c16121	2	4	-	-	-	-	-
gALL_v3_c16122	4	12	gnl|CDD|29138	cd00176, SPEC, Spectrin repea	ref|XP_003700798.1|	PREDICTED: LOW QUALITY PROTEIN: microtubule-actin cross-linking factor 1-like [Megachile rotundata]	5,00E-88
gALL_v3_c16123	4	2	-	-	-	-	-
gALL_v3_c16124	3	5	-	-	ref|XP_562955.4|	AGAP003181-PA [Anopheles gambiae str. PEST] gb|EAL40726.4| AGAP003181-PA [Anopheles gambiae str. PEST]	3,00E-57
gALL_v3_c16125	2	3	gnl|CDD|129101	smart00868, zf-AD, Zinc-finger	ref|XP_003708269.1|	PREDICTED: uncharacterized protein LOC100877935 [Megachile rotundata]	4,00E-35
gALL_v3_c16126	8	10	gnl|CDD|173660	cd05123, STKc_AGC, Catalytic	ref|XP_002165000.1|	PREDICTED: protein kinase C-like 1B-like [Hydra vulgaris]	1,00E-24
gALL_v3_c16127	3	5	-	-	-	-	-
gALL_v3_c16128	5	2	-	-	-	-	-
gALL_v3_c16129	3	1	-	-	-	-	-
gALL_v3_c1613	15	16	gnl|CDD|181824	PRK09404, sucA, 2-oxoglutarat	ref|XP_006566132.1|	PREDICTED: 2-oxoglutarate dehydrogenase, mitochondrial-like isoform X9 [Apis mellifera]	0
gALL_v3_c16130	1	6	-	-	gb|ETN66164.1|	paraflagellar rod protein [Anopheles darlingi]	4,00E-11
gALL_v3_c16131	2	5	-	-	-	-	-
gALL_v3_c16132	3	4	gnl|CDD|88277	cd05147, RIO1_euk, RIO kinas	ref|XP_971515.1|	PREDICTED: similar to serine/threonine-protein kinase RIO1 [Tribolium castaneum] gb|EFA02123.1| hypothetical protein TcasGA2_TC007772 [Tribolium castaneum]	2,00E-93
gALL_v3_c16133	5	7	gnl|CDD|29257	cd00200, WD40, WD40 domai	gb|AEE63252.1|	unknown [Dendroctonus ponderosae]	1,00E-131
gALL_v3_c16134	4	4	-	-	gb|EFO25754.2|	multi drug resistance-associated protein [Loa loa]	9,00E-10
gALL_v3_c16135	1	3	-	-	gb|EEZ99765.1|	hypothetical protein TcasGA2_TC002538 [Tribolium castaneum]	3,00E-15
gALL_v3_c16136	3	5	-	-	-	-	-
gALL_v3_c16137	1	4	-	-	-	-	-
gALL_v3_c16138	2	3	-	-	ref|XP_002429833.1|	rest corepressor corest, protein, putative [Pediculus humanus corporis] gb|EEB17095.1| rest corepressor corest, protein, putative [Pediculus humanus corporis]	2,00E-22
gALL_v3_c1614	5	13	-	-	-	-	-
gALL_v3_c16140	5	16	gnl|CDD|162113	TIGR00927, 2A1904, K+-deper	ref|XP_001602467.2|	PREDICTED: hypothetical protein LOC100118520 [Nasonia vitripennis]	1,00E-10
gALL_v3_c16141	2	2	-	-	-	-	-
gALL_v3_c16142	4	2	-	-	-	-	-
gALL_v3_c16145	4	2	-	-	-	-	-
gALL_v3_c16146	2	2	-	-	-	-	-
gALL_v3_c16148	0	6	-	-	gb|EFA02187.1|	hypothetical protein TcasGA2_TC007843 [Tribolium castaneum]	4,00E-40
gALL_v3_c16149	5	2	-	-	-	-	-
gALL_v3_c1615	7	13	-	-	gb|EFN81372.1|	hypothetical protein EAI_15810 [Harpegnathos saltator]	7,00E-26
gALL_v3_c16151	3	2	-	-	ref|WP_006427240.1|	hypothetical protein [Dorea longicatena] gb|EDM61645.1| hypothetical protein DORLON_02997 [Dorea longicatena DSM 13814]	4,00E-11
gALL_v3_c16152	2	3	-	-	-	-	-
gALL_v3_c16153	7	4	-	-	-	-	-
gALL_v3_c16154	1	4	-	-	-	-	-
gALL_v3_c16155	3	2	-	-	-	-	-
gALL_v3_c16158	3	4	gnl|CDD|191427	pfam06012, DUF908, Domain	gb|EFN84895.1|	E3 ubiquitin-protein ligase HUWE1 [Harpegnathos saltator]	1,00E-126
gALL_v3_c1616	19	18	-	-	gb|AGM32830.1|	Rossmann-fold NAD(P)(+)-binding protein [Coptotermes formosanus]	1,00E-15
gALL_v3_c16160	2	4	gnl|CDD|28939	cd00057, FA58C, Coagulation f	ref|XP_003703718.1|	PREDICTED: discoidin domain-containing receptor 2-like [Megachile rotundata]	4,00E-96
gALL_v3_c16161	2	5	-	-	ref|XP_002431866.1|	transmembrane protein 16E, putative [Pediculus humanus corporis] gb|EEB19128.1| transmembrane protein 16E, putative [Pediculus humanus corporis]	3,00E-21
gALL_v3_c16162	1	5	gnl|CDD|144903	pfam01480, PWI, PWI domain	gb|EFX85677.1|	hypothetical protein DAPPUDRAFT_313769 [Daphnia pulex]	7,00E-36
gALL_v3_c16163	1	4	-	-	-	-	-
gALL_v3_c16164	2	7	-	-	gb|EGI65537.1|	cAMP-specific 3',5'-cyclic phosphodiesterase [Acromyrmex echinatior]	2,00E-25

gALL_v3_c16165	2	8	gnl|CDD|192642	pfam10607, RanBPM_CRA, Ra	gb|AGM32587.1|	CTLH/CRA C-terminal to lisH motif domain containing protein [Coptotermes formosanus]	1,00E-114
gALL_v3_c16166	2	2	-	-	-	-	-
gALL_v3_c16167	3	1	-	-	-	-	-
gALL_v3_c16168	1	8	-	-	ref|XP_002428797.1|	hypothetical protein Phum_PHUM401430 [Pediculus humanus corporis] gb|EEB16059.1| hypothetical protein Phum_PHUM401430 [Pediculus humanus corporis]	8,00E-06
gALL_v3_c16169	6	4	-	-	-	-	-
gALL_v3_c16170	8	7	gnl|CDD|145882	pfam02958, DUF227, Domain	ref|XP_003243915.1|	PREDICTED: hypothetical protein LOC100574123 [Acyrthosiphon pisum]	2,00E-26
gALL_v3_c16171	1	5	-	-	-	-	-
gALL_v3_c16172	2	3	gnl|CDD|189654	pfam00651, BTB, BTB/POZ dor	emb|CAZ68905.1|	fruitless [Blattella germanica]	1,00E-94
gALL_v3_c16173	0	5	-	-	-	-	-
gALL_v3_c16174	3	3	-	-	ref|XP_003393803.1|	PREDICTED: LOW QUALITY PROTEIN: WD repeat-containing protein CG11141-like [Bombus terrestris] ref|XP_003494709.1| PREDICTED: LOW QUALITY PROTEIN: WD repea	1,00E-16
gALL_v3_c16175	7	5	-	-	gb|EFN67721.1|	hypothetical protein EAG_07710 [Camponotus floridanus]	3,00E-15
gALL_v3_c16176	1	5	-	-	-	-	-
gALL_v3_c16178	2	4	gnl|CDD|191463	pfam06159, DUF974, Protein c	ref|XP_003485265.1|	PREDICTED: UPF0533 protein C5orf44 homolog [Bombus impatiens]	2,00E-39
gALL_v3_c16179	4	7	-	-	-	-	-
gALL_v3_c1618	3	7	gnl|CDD|112933	pfam04140, ICMT, Isoprenylcy	gb|EFN82311.1|	Protein-S-isoprenylcysteine O-methyltransferase [Harpegnathos saltator]	2,00E-58
gALL_v3_c16180	3	3	-	-	-	-	-
gALL_v3_c16181	4	7	-	-	-	-	-
gALL_v3_c16182	1	3	-	-	ref|XP_006618049.1|	PREDICTED: dentin sialophosphoprotein-like isoform X2 [Apis dorsata]	1,00E-63
gALL_v3_c16183	4	2	-	-	-	-	-
gALL_v3_c16184	5	4	-	-	-	-	-
gALL_v3_c16185	2	3	-	-	-	-	-
gALL_v3_c16186	2	3	-	-	-	-	-
gALL_v3_c16187	4	5	-	-	-	-	-
gALL_v3_c16189	2	3	gnl|CDD|150314	pfam09606, Med15, ARC105 c	emb|CDJ64246.1|	hypothetical protein ENH_00063870 [Eimeria necatrix]	3,00E-20
gALL_v3_c1619	8	17	-	-	-	-	-
gALL_v3_c16190	6	5	-	-	-	-	-
gALL_v3_c16191	1	7	-	-	-	-	-
gALL_v3_c16193	4	5	-	-	ref|XP_003689571.1|	PREDICTED: uncharacterized protein LOC100865216 [Apis florea]	4,00E-08
gALL_v3_c16194	3	2	-	-	-	-	-
gALL_v3_c16195	3	3	-	-	-	-	-
gALL_v3_c16196	2	4	-	-	ref|XP_972068.2|	PREDICTED: similar to AGAP003656-PA [Tribolium castaneum]	6,00E-25
gALL_v3_c16197	3	4	-	-	ref|XP_003696008.1|	PREDICTED: Hermansky-Pudlak syndrome 1 protein homolog [Apis florea]	1,00E-48
gALL_v3_c16198	1	4	-	-	-	-	-
gALL_v3_c16199	1	3	-	-	-	-	-
gALL_v3_c16200	3	4	-	-	-	-	-
gALL_v3_c16201	5	2	-	-	-	-	-
gALL_v3_c16203	3	2	gnl|CDD|147365	pfam05148, Methyltransf_8, H	ref|XP_003689750.1|	PREDICTED: ribosomal RNA-processing protein 8-like [Apis florea]	3,00E-53
gALL_v3_c16204	1	3	-	-	-	-	-
gALL_v3_c16205	2	4	-	-	-	-	-
gALL_v3_c16206	1	5	-	-	ref|XP_003248213.1|	PREDICTED: ubiquitin carboxyl-terminal hydrolase 32-like isoform 2 [Acyrthosiphon pisum]	1,00E-106
gALL_v3_c16207	2	5	-	-	-	-	-
gALL_v3_c16209	1	6	-	-	-	-	-
gALL_v3_c1621	4	6	-	-	-	-	-
gALL_v3_c16210	2	5	-	-	-	-	-
gALL_v3_c16211	3	3	-	-	-	-	-
gALL_v3_c16212	1	5	-	-	-	-	-
gALL_v3_c16213	2	3	-	-	-	-	-
gALL_v3_c16214	0	3	-	-	-	-	-
gALL_v3_c16215	4	4	-	-	-	-	-
gALL_v3_c16216	3	11	-	-	-	-	-
gALL_v3_c16217	3	3	gnl|CDD|31829	COG1643, HrpA, HrpA-like hel	ref|XP_002427077.1|	pre-mRNA-splicing factor ATP-dependent RNA helicase prp16, putative [Pediculus humanus corporis] gb|EEB14339.1| pre-mRNA-splicing factor ATP-dependent RNA helica	1,00E-78
gALL_v3_c16218	7	8	gnl|CDD|189372	pfam00067, p450, Cytochrome	sp|P29981.1|CP4C1_BL	RecName: Full=Cytochrome P450 4C1; AltName: Full=CYPIVC1 gb|AAA27819.1| cytochrome P450 [Blaberus discoidalis]	9,00E-46
gALL_v3_c16219	8	10	-	-	gb|EFZ20104.1|	hypothetical protein SINV_08494 [Solenopsis invicta]	1,00E-113
gALL_v3_c16220	5	3	-	-	-	-	-
gALL_v3_c16222	3	1	-	-	-	-	-
gALL_v3_c16223	4	7	-	-	-	-	-
gALL_v3_c16224	2	5	-	-	-	-	-
gALL_v3_c16225	3	6	gnl|CDD|192041	pfam08449, UAA, UAA transpo	ref|XP_001601459.1|	PREDICTED: adenosine 3'-phospho 5'-phosphosulfate transporter 2-like [Nasonia vitripennis]	1,00E-73
gALL_v3_c16226	3	2	-	-	-	-	-
gALL_v3_c16227	4	2	-	-	-	-	-
gALL_v3_c16228	1	4	-	-	-	-	-
gALL_v3_c16229	3	3	-	-	-	-	-
gALL_v3_c16230	3	2	-	-	-	-	-
gALL_v3_c16232	4	5	-	-	ref|XP_002429112.1|	conserved hypothetical protein [Pediculus humanus corporis] gb|EEB16374.1| conserved hypothetical protein [Pediculus humanus corporis]	3,00E-40
gALL_v3_c16233	5	6	-	-	-	-	-
gALL_v3_c16234	7	7	-	-	ref|XP_006616582.1|	PREDICTED: RNA-binding protein Nova-1-like isoform X2 [Apis dorsata]	5,00E-37
gALL_v3_c16235	2	2	-	-	-	-	-
gALL_v3_c16237	2	2	-	-	-	-	-
gALL_v3_c16238	0	5	-	-	-	-	-
gALL_v3_c16239	3	3	-	-	-	-	-
gALL_v3_c1624	11	6	gnl|CDD|173611	PTZ00421, PTZ00421, coronin;	ref|XP_001301263.1|	coronin [Trichomonas vaginalis G3] gb|EAX88333.1| coronin, putative [Trichomonas vaginalis G3]	1,00E-138
gALL_v3_c16240	3	2	gnl|CDD|178915	PRK00174, PRK00174, acetyl-C	ref|XP_001599652.1|	PREDICTED: acetyl-coenzyme A synthetase-like [Nasonia vitripennis]	2,00E-63
gALL_v3_c16241	5	2	-	-	-	-	-
gALL_v3_c16242	2	9	-	-	-	-	-
gALL_v3_c16243	4	9	-	-	gb|EFA10418.1|	hypothetical protein TcasGA2_TC012658 [Tribolium castaneum]	1,00E-11
gALL_v3_c16244	7	10	gnl|CDD|189955	pfam01363, FYVE, FYVE zinc fi	gb|EFN75234.1|	Zinc finger FYVE domain-containing protein 1 [Harpegnathos saltator]	2,00E-37
gALL_v3_c16245	3	2	-	-	-	-	-
gALL_v3_c16246	3	4	-	-	-	-	-
gALL_v3_c16247	2	2	-	-	-	-	-
gALL_v3_c16248	5	11	-	-	ref|XP_003488185.1|	PREDICTED: hypothetical protein LOC100743823 [Bombus impatiens]	3,00E-18
gALL_v3_c16249	6	5	-	-	-	-	-
gALL_v3_c1625	5	9	gnl|CDD|177854	PLN02204, PLN02204, diacylgl	gb|EFN84102.1|	Ceramide kinase [Harpegnathos saltator]	1,00E-140
gALL_v3_c16250	2	5	gnl|CDD|189694	pfam00736, EF1_GNE, EF-1 gu	gb|AGM32437.1|	elongation factor 1-beta-like isoform 1 [Coptotermes formosanus]	5,00E-68
gALL_v3_c16251	2	5	-	-	-	-	-
gALL_v3_c16252	2	8	-	-	-	-	-
gALL_v3_c16253	8	7	-	-	gb|EFA10298.1|	hypothetical protein TcasGA2_TC012513 [Tribolium castaneum]	6,00E-26
gALL_v3_c16255	6	5	gnl|CDD|146000	pfam03154, Atrophin-1, Atrop	emb|CCV01214.1|	Atrophin, partial [Blattella germanica]	6,00E-54
gALL_v3_c16256	6	7	-	-	-	-	-
gALL_v3_c16257	3	8	-	-	-	-	-
gALL_v3_c16259	3	3	-	-	gb|AFI81411.1|	major facilitator superfamily domain-containing protein 9-like protein [Phyllotreta striolata]	1,00E-30
gALL_v3_c1626	15	24	-	-	-	-	-
gALL_v3_c16260	1	7	gnl|CDD|162093	TIGR00892, 2A0113, monocar	ref|XP_003691132.1|	PREDICTED: monocarboxylate transporter 10-like [Apis florea]	1,00E-86
gALL_v3_c16261	2	3	-	-	-	-	-
gALL_v3_c16263	6	7	-	-	-	-	-
gALL_v3_c16264	0	5	gnl|CDD|191009	pfam04502, DUF572, Family o	ref|XP_002425336.1|	conserved hypothetical protein [Pediculus humanus corporis] gb|EEB12598.1| conserved hypothetical protein [Pediculus humanus corporis]	2,00E-92
gALL_v3_c16265	2	3	-	-	-	-	-
gALL_v3_c16266	4	2	-	-	-	-	-
gALL_v3_c16267	3	5	gnl|CDD|189450	pfam00209, SNF, Sodium:neur	ref|XP_309367.2|	AGAP011279-PA [Anopheles gambiae str. PEST] gb|EAA05172.3| AGAP011279-PA [Anopheles gambiae str. PEST]	1,00E-160
gALL_v3_c16269	2	3	-	-	-	-	-
gALL_v3_c1627	9	22	-	-	ref|XP_003490934.1|	PREDICTED: UPF0518 protein AGAP011705-like isoform 1 [Bombus impatiens] ref|XP_003490935.1| PREDICTED: UPF0518 protein AGAP011705-like isoform 2 [Bombus im	1,00E-55
gALL_v3_c16270	1	6	-	-	-	-	-
gALL_v3_c16271	4	10	-	-	gb|EFN60481.1|	hypothetical protein EAG_10563 [Camponotus floridanus]	7,00E-52
gALL_v3_c16273	4	4	-	-	-	-	-
gALL_v3_c16274	5	9	-	-	gb|EFN75932.1|	Zinc finger FYVE domain-containing protein 26 [Harpegnathos saltator]	6,00E-27
gALL_v3_c16275	2	3	-	-	-	-	-
gALL_v3_c16276	3	2	-	-	gb|EFA13462.1|	hypothetical protein TcasGA2_TC005062 [Tribolium castaneum]	1,00E-14
gALL_v3_c16277	3	3	-	-	gb|EFZ20256.1|	hypothetical protein SINV_12404 [Solenopsis invicta]	2,00E-36
gALL_v3_c16278	6	3	-	-	-	-	-
gALL_v3_c16279	7	5	-	-	-	-	-
gALL_v3_c1628	9	17	-	-	-	-	-
gALL_v3_c16280	1	4	gnl|CDD|173833	cd00685, Trans_IPPS_HT, Trans	gb|EHJ68647.1|	dimethylallyltransferase [Danaus plexippus]	3,00E-33
gALL_v3_c16281	1	3	-	-	-	-	-
gALL_v3_c16284	0	6	-	-	ref|XP_003703656.1|	PREDICTED: vacuolar protein sorting-associated protein 13C-like [Megachile rotundata]	9,00E-24
gALL_v3_c16285	2	3	-	-	-	-	-
gALL_v3_c16286	1	3	-	-	gb|EFA08886.1|	hypothetical protein TcasGA2_TC006587 [Tribolium castaneum]	2,00E-75
gALL_v3_c16287	3	3	gnl|CDD|110072	pfam01044, Vinculin, Vinculin	gb|EFZ16798.1|	hypothetical protein SINV_14069 [Solenopsis invicta]	1,00E-28
gALL_v3_c16288	4	2	-	-	-	-	-
gALL_v3_c16289	2	6	-	-	ref|XP_003228150.1|	PREDICTED: hypothetical protein LOC100560153 [Anolis carolinensis]	1,00E-24
gALL_v3_c16290	1	5	gnl|CDD|188225	TIGR02478, 6PF1K_euk, 6-pho	gb|AAR09748.1|	similar to Drosophila melanogaster Pfk, partial [Drosophila yakuba]	1,00E-26
gALL_v3_c16291	2	3	-	-	-	-	-
gALL_v3_c16292	1	3	-	-	-	-	-
gALL_v3_c16294	1	5	gnl|CDD|48616	cd03067, PDI_b_PDIR_N, PDIb	ref|XP_970942.1|	PREDICTED: similar to AGAP010217-PA [Tribolium castaneum] gb|EEZ97186.1| hypothetical protein TcasGA2_TC004373 [Tribolium castaneum]	1,00E-72
gALL_v3_c16295	3	4	gnl|CDD|128833	smart00561, MBT, Present in D	gb|EFA09361.1|	hypothetical protein TcasGA2_TC001922 [Tribolium castaneum]	1,00E-143
gALL_v3_c16296	3	4	gnl|CDD|189378	pfam00079, Serpin, Serpin (se	gb|ACR56865.1|	serine proteinase inhibitor-1B/C [Mamestra brassicae]	2,00E-12
gALL_v3_c16298	3	4	-	-	gb|EFN72391.1|	Transposable element Tc3 transposase [Camponotus floridanus]	7,00E-34
gALL_v3_c16299	1	4	-	-	-	-	-
gALL_v3_c163	13	11	-	-	-	-	-
gALL_v3_c1630	18	35	-	-	ref|XP_004206973.1|	PREDICTED: tigger transposable element-derived protein 1-like [Hydra vulgaris]	3,00E-10
gALL_v3_c16301	5	4	gnl|CDD|31603	COG1413, COG1413, FOG: HEA	ref|XP_001608071.1|	PREDICTED: deoxyhypusine hydroxylase-like [Nasonia vitripennis]	1,00E-137
gALL_v3_c16303	4	9	-	-	-	-	-
gALL_v3_c16304	2	4	-	-	-	-	-
gALL_v3_c16305	5	4	-	-	-	-	-
gALL_v3_c16306	2	3	-	-	ref|XP_003706662.1|	PREDICTED: uncharacterized protein LOC100883488 [Megachile rotundata]	1,00E-10
gALL_v3_c16307	3	1	-	-	ref|XP_005187819.1|	PREDICTED: protein NDUFAF4 homolog [Musca domestica]	8,00E-06
gALL_v3_c16308	3	4	-	-	-	-	-
gALL_v3_c16309	4	3	gnl|CDD|178257	PLN02651, PLN02651, cysteine	ref|XP_319845.3|	AGAP009094-PA [Anopheles gambiae str. PEST] gb|EAA14741.3| AGAP009094-PA [Anopheles gambiae str. PEST]	1,00E-115
gALL_v3_c1631	11	6	-	-	-	-	-
gALL_v3_c16310	0	5	-	-	-	-	-
gALL_v3_c16312	2	3	-	-	-	-	-
gALL_v3_c16313	12	28	-	-	-	-	-
gALL_v3_c16314	8	19	-	-	-	-	-
gALL_v3_c16315	0	4	-	-	-	-	-
gALL_v3_c16316	2	6	-	-	-	-	-
gALL_v3_c16317	13	12	gnl|CDD|189891	pfam01213, CAP_N, Adenylate	ref|XP_006612122.1|	PREDICTED: adenylyl cyclase-associated protein 1-like isoform X3 [Apis dorsata]	1,00E-141

gALL_v3_c16318	4	2	-	-	-	-	-
gALL_v3_c16319	0	4	-	-	gb|EHJ72827.1|	putative nudE nuclear distribution protein E [Danaus plexippus]	1,00E-08
gALL_v3_c16320	0	4	-	-	-	-	-
gALL_v3_c16321	4	4	gnl|CDD|144473	pfam00888, Cullin, Cullin fami	ref|XP_001606829.2|	PREDICTED: cullin-1 isoform 1 [Nasonia vitripennis] ref|XP_003424085.1| PREDICTED: cullin-1 isoform 2 [Nasonia vitripennis] ref|XP_003424086.1| PREDICTED: cullin-1 is	8,00E-90
gALL_v3_c16322	3	2	-	-	-	-	-
gALL_v3_c16324	4	2	-	-	-	-	-
gALL_v3_c16325	3	1	-	-	-	-	-
gALL_v3_c16326	2	2	-	-	-	-	-
gALL_v3_c16328	2	6	-	-	-	-	-
gALL_v3_c16329	2	2	-	-	-	-	-
gALL_v3_c1633	21	29	gnl|CDD|111185	pfam02263, GBP, Guanylate-bi	ref|XP_321004.2|	AGAP002047-PA [Anopheles gambiae str. PEST] gb|EAA01072.3| AGAP002047-PA [Anopheles gambiae str. PEST]	0
gALL_v3_c16330	3	1	-	-	-	-	-
gALL_v3_c16331	1	4	-	-	ref|XP_003707904.1|	PREDICTED: dual specificity protein phosphatase CDC14A-like [Megachile rotundata]	4,00E-45
gALL_v3_c16332	3	5	gnl|CDD|176491	cd08761, Cyt_b561_CYB561D2	ref|XP_798124.2|	PREDICTED: cytochrome b561 domain-containing protein 1-like [Strongylocentrotus purpuratus]	3,00E-28
gALL_v3_c16333	2	7	-	-	-	-	-
gALL_v3_c16335	1	5	-	-	gb|ENN73756.1|	hypothetical protein YQE_09652, partial [Dendroctonus ponderosae]	3,00E-13
gALL_v3_c16336	2	3	-	-	ref|XP_002423277.1|	Nucleolar GTP-binding protein, putative [Pediculus humanus corporis] gb|EEB10539.1| Nucleolar GTP-binding protein, putative [Pediculus humanus corporis]	8,00E-16
gALL_v3_c16337	5	9	-	-	ref|XP_002423115.1|	conserved hypothetical protein [Pediculus humanus corporis] gb|EEB10377.1| conserved hypothetical protein [Pediculus humanus corporis]	2,00E-73
gALL_v3_c16338	3	4	-	-	ref|XP_002026691.1|	GL11867 [Drosophila persimilis] gb|EDW33660.1| GL11867 [Drosophila persimilis]	2,00E-69
gALL_v3_c1634	4	9	-	-	gb|ACC62131.1|	muscle LIM protein-like protein isoform A variant 1 [Bombus hypocrita]	2,00E-09
gALL_v3_c16341	4	2	-	-	ref|XP_002425532.1|	bromodomain-containing protein, putative [Pediculus humanus corporis] gb|EEB12794.1| bromodomain-containing protein, putative [Pediculus humanus corporis]	3,00E-38
gALL_v3_c16342	3	2	-	-	-	-	-
gALL_v3_c16343	2	4	-	-	ref|XP_003692658.1|	PREDICTED: uncharacterized protein LOC100869297 [Apis florea]	2,00E-55
gALL_v3_c16345	40	49	gnl|CDD|177214	MTH00157, ATP6, ATP synthas	ref|YP_006503952.1|	ATP synthase F0 subunit 6 (mitochondrion) [Coptotermes lacteus] gb|AFM92452.1| ATP synthase F0 subunit 6 (mitochondrion) [Coptotermes lacteus]	1,00E-30
gALL_v3_c16346	1	5	-	-	-	-	-
gALL_v3_c16347	1	4	-	-	-	-	-
gALL_v3_c16348	1	4	-	-	-	-	-
gALL_v3_c16349	1	4	-	-	-	-	-
gALL_v3_c1635	7	18	-	-	-	-	-
gALL_v3_c16351	1	5	-	-	ref|XP_002427021.1|	protein purity of essence, putative [Pediculus humanus corporis] gb|EEB14283.1| protein purity of essence, putative [Pediculus humanus corporis]	1,00E-34
gALL_v3_c16352	3	3	gnl|CDD|73264	cd01385, MYSc_type_IX, Myos	ref|XP_002426282.1|	myosin IIIB, putative [Pediculus humanus corporis] gb|EEB13544.1| myosin IIIB, putative [Pediculus humanus corporis]	1,00E-13
gALL_v3_c16353	8	13	-	-	dbj|BAD00045.1|	armadillo protein [Gryllus bimaculatus]	1,00E-124
gALL_v3_c16354	3	6	-	-	gb|EFN64007.1|	hypothetical protein EAG_10542 [Camponotus floridanus]	5,00E-76
gALL_v3_c16355	3	4	-	-	-	-	-
gALL_v3_c16356	4	5	-	-	-	-	-
gALL_v3_c16357	3	3	-	-	gb|ENN75917.1|	hypothetical protein YQE_07558, partial [Dendroctonus ponderosae]	8,00E-08
gALL_v3_c16358	2	3	-	-	-	-	-
gALL_v3_c16359	2	2	-	-	gb|ENN83352.1|	hypothetical protein YQE_00289, partial [Dendroctonus ponderosae]	4,00E-35
gALL_v3_c1636	9	7	-	-	-	-	-
gALL_v3_c16360	2	6	gnl|CDD|191679	pfam07064, RIC1, RIC1. RIC1 I	ref|XP_002427318.1|	conserved hypothetical protein [Pediculus humanus corporis] gb|EEB14580.1| conserved hypothetical protein [Pediculus humanus corporis]	2,00E-88
gALL_v3_c16361	5	4	gnl|CDD|177210	MTH00153, COX1, cytochrome	dbj|BAH36928.1|	Warts kinase [Gryllus bimaculatus]	5,00E-39
gALL_v3_c16362	0	5	-	-	-	-	-
gALL_v3_c16363	4	3	-	-	-	-	-
gALL_v3_c16364	2	4	-	-	ref|XP_967104.1|	PREDICTED: similar to phd finger transcription factor [Tribolium castaneum] gb|EFA07714.1| hypothetical protein TcasGA2_TC002191 [Tribolium castaneum]	2,00E-11
gALL_v3_c16365	3	1	-	-	-	-	-
gALL_v3_c16367	1	3	gnl|CDD|188978	cd09579, SAM_Samd7,11, SAM	ref|XP_003704445.1|	PREDICTED: uncharacterized protein LOC100880494 [Megachile rotundata]	1,00E-27
gALL_v3_c16368	2	2	-	-	-	-	-
gALL_v3_c16369	3	2	-	-	ref|XP_004209444.1|	PREDICTED: RNA-directed DNA polymerase from mobile element jockey-like, partial [Hydra vulgaris]	3,00E-18
gALL_v3_c1637	8	8	-	-	-	-	-
gALL_v3_c16370	1	8	-	-	gb|EFN80369.1|	hypothetical protein EAI_14715 [Harpegnathos saltator]	4,00E-06
gALL_v3_c16371	4	8	-	-	-	-	-
gALL_v3_c16372	3	2	-	-	-	-	-
gALL_v3_c16373	2	3	-	-	-	-	-
gALL_v3_c16374	3	5	-	-	-	-	-
gALL_v3_c16376	3	2	-	-	-	-	-
gALL_v3_c16377	0	5	-	-	sp|P29981.1|CP4C1_BL	RecName: Full=Cytochrome P450 4C1; AltName: Full=CYPIVC1 gb|AAA27819.1| cytochrome P450 [Blaberus discoidalis]	2,00E-15
gALL_v3_c1638	5	5	-	-	ref|XP_003693621.1|	PREDICTED: uncharacterized protein LOC100869669 [Apis florea]	1,00E-27
gALL_v3_c16380	4	2	gnl|CDD|29261	cd00204, ANK, ankyrin repeat:	pdb|1N0R|A	Chain A, 4ank: A Designed Ankyrin Repeat Protein With Four Identical Consensus Repeats	4,00E-17
gALL_v3_c16381	2	2	-	-	-	-	-
gALL_v3_c16382	4	2	-	-	-	-	-
gALL_v3_c16383	2	9	-	-	ref|XP_001948735.1|	PREDICTED: hypothetical protein LOC100159761 isoform 1 [Acyrthosiphon pisum]	3,00E-09
gALL_v3_c16384	0	6	-	-	-	-	-
gALL_v3_c16385	3	5	-	-	-	-	-
gALL_v3_c16386	4	3	-	-	-	-	-
gALL_v3_c16388	4	2	-	-	-	-	-
gALL_v3_c16389	1	6	-	-	-	-	-
gALL_v3_c16390	0	5	-	-	-	-	-
gALL_v3_c16392	3	9	-	-	-	-	-
gALL_v3_c16393	1	4	-	-	-	-	-
gALL_v3_c16394	6	7	-	-	gb|EFA13284.1|	hypothetical protein TcasGA2_TC010304 [Tribolium castaneum]	5,00E-11
gALL_v3_c16395	3	8	-	-	-	-	-
gALL_v3_c16396	2	3	-	-	-	-	-
gALL_v3_c16397	2	3	-	-	-	-	-
gALL_v3_c16398	2	2	-	-	-	-	-
gALL_v3_c16399	1	2	gnl|CDD|133249	cd00066, G-alpha, G protein a	gb|AAW50314.1|	G protein alpha subunit AgGq3 [Anopheles gambiae]	5,00E-57
gALL_v3_c16400	3	8	-	-	-	-	-
gALL_v3_c16401	4	3	-	-	-	-	-
gALL_v3_c16402	9	6	-	-	-	-	-
gALL_v3_c16403	3	1	-	-	ref|XP_001599387.2|	PREDICTED: myosin-X [Nasonia vitripennis]	7,00E-12
gALL_v3_c16404	10	14	-	-	gb|ESN91435.1|	hypothetical protein HELRODRAFT_165469 [Helobdella robusta]	7,00E-14
gALL_v3_c16405	13	12	-	-	-	-	-
gALL_v3_c16406	4	3	-	-	gb|ELU07650.1|	hypothetical protein CAPTEDRAFT_227185 [Capitella teleta]	3,00E-20
gALL_v3_c16408	3	3	-	-	ref|XP_004537236.1|	PREDICTED: transmembrane protein 47-like isoform X6 [Ceratitis capitata]	5,00E-35
gALL_v3_c16409	3	2	-	-	gb|AGM32427.1|	soldier-caste specific protein [Coptotermes formosanus]	4,00E-55
gALL_v3_c1641	12	26	gnl|CDD|191361	pfam05739, SNARE, SNARE do	ref|XP_974721.1|	PREDICTED: similar to AGAP001394-PA [Tribolium castaneum]	3,00E-97
gALL_v3_c16410	5	6	gnl|CDD|190240	pfam02194, PXA, PXA domain.	ref|XP_002595724.1|	hypothetical protein BRAFLDRAFT_275391 [Branchiostoma floridae] gb|EEN51736.1| hypothetical protein BRAFLDRAFT_275391 [Branchiostoma floridae]	6,00E-41
gALL_v3_c16411	3	2	-	-	-	-	-
gALL_v3_c16412	2	3	-	-	-	-	-
gALL_v3_c16413	1	5	-	-	ref|XP_624798.3|	PREDICTED: intracellular protein transport protein USO1-like isoform X2 [Apis mellifera]	3,00E-24
gALL_v3_c16414	1	5	-	-	-	-	-
gALL_v3_c16415	10	19	-	-	-	-	-
gALL_v3_c16416	2	2	-	-	-	-	-
gALL_v3_c16417	3	2	-	-	-	-	-
gALL_v3_c16418	6	5	gnl|CDD|48478	cd00714, GFAT, Glutamine ami	dbj|BAN21605.1|	glucosamine-fructose-6-phosphate aminotransferase, partial [Riptortus pedestris]	3,00E-98
gALL_v3_c16419	2	9	-	-	-	-	-
gALL_v3_c16420	6	3	-	-	-	-	-
gALL_v3_c16421	6	9	-	-	-	-	-
gALL_v3_c16423	1	4	gnl|CDD|191787	pfam07569, Hira, TUP1-like en	gb|EGI67467.1|	Protein HIRA [Acromyrmex echinatior]	2,00E-26
gALL_v3_c16424	3	2	gnl|CDD|144978	pfam01585, G-patch, G-patch	ref|XP_001947898.2|	PREDICTED: SURP and G-patch domain-containing protein 1-like [Acyrthosiphon pisum]	1,00E-54
gALL_v3_c16425	3	5	-	-	-	-	-
gALL_v3_c16427	5	3	-	-	-	-	-
gALL_v3_c16428	3	1	-	-	-	-	-
gALL_v3_c16429	10	11	-	-	gb|EFA13284.1|	hypothetical protein TcasGA2_TC010304 [Tribolium castaneum]	2,00E-21
gALL_v3_c1643	2	6	-	-	-	-	-
gALL_v3_c16430	7	7	-	-	gb|EFN66434.1|	Zinc finger protein 583 [Camponotus floridanus]	1,00E-117
gALL_v3_c16431	4	8	-	-	-	-	-
gALL_v3_c16432	5	5	-	-	-	-	-
gALL_v3_c16433	2	4	-	-	-	-	-
gALL_v3_c16434	3	2	-	-	-	-	-
gALL_v3_c16436	4	6	-	-	-	-	-
gALL_v3_c16437	2	2	-	-	-	-	-
gALL_v3_c16438	3	4	-	-	-	-	-
gALL_v3_c16439	3	8	-	-	-	-	-
gALL_v3_c1644	19	35	gnl|CDD|190965	pfam04387, PTPLA, Protein ty	ref|XP_003701348.1|	PREDICTED: 3-hydroxyacyl-CoA dehydratase-like [Megachile rotundata]	1,00E-160
gALL_v3_c16440	4	4	gnl|CDD|29062	cd00143, PP2Cc, Serine/threo	gb|EFX74791.1|	hypothetical protein DAPPUDRAFT_323991 [Daphnia pulex]	1,00E-61
gALL_v3_c16442	3	5	gnl|CDD|110776	pfam01803, LIM_bind, LIM-do	gb|EEZ97869.1|	hypothetical protein TcasGA2_TC000244 [Tribolium castaneum]	1,00E-160
gALL_v3_c16443	3	2	-	-	ref|XP_006029730.1|	PREDICTED: histone-lysine N-methyltransferase SETMAR-like [Alligator sinensis]	1,00E-06
gALL_v3_c16444	2	3	-	-	-	-	-
gALL_v3_c16445	2	8	-	-	-	-	-
gALL_v3_c16446	5	3	-	-	-	-	-
gALL_v3_c16447	3	5	-	-	gb|ENN77069.1|	hypothetical protein YQE_06404, partial [Dendroctonus ponderosae]	3,00E-16
gALL_v3_c16448	0	4	-	-	-	-	-
gALL_v3_c16449	3	5	-	-	gb|ERL91348.1|	hypothetical protein D910_08680, partial [Dendroctonus ponderosae]	6,00E-22
gALL_v3_c16450	2	3	gnl|CDD|190411	pfam02759, RUN, RUN domai	gb|EFZ16359.1|	hypothetical protein SINV_09574 [Solenopsis invicta]	2,00E-45
gALL_v3_c16451	3	2	-	-	ref|XP_002422903.1|	U1 small nuclear ribonucleoprotein 70 kDa, putative [Pediculus humanus corporis] gb|EEB10165.1| U1 small nuclear ribonucleoprotein 70 kDa, putative [Pediculus human	3,00E-40
gALL_v3_c16452	1	5	-	-	ref|XP_002423816.1|	hypothetical protein Phum_PHUM087100 [Pediculus humanus corporis] gb|EEB11078.1| hypothetical protein Phum_PHUM087100 [Pediculus humanus corporis]	4,00E-17
gALL_v3_c16453	4	3	gnl|CDD|179984	PRK05269, PRK05269, transalc	ref|XP_001660027.1|	transaldolase [Aedes aegypti] gb|EAT38745.1| AAEL009389-PA [Aedes aegypti]	2,00E-31
gALL_v3_c16454	3	2	-	-	-	-	-
gALL_v3_c16455	5	13	-	-	-	-	-
gALL_v3_c16456	2	4	-	-	-	-	-
gALL_v3_c16457	1	3	-	-	-	-	-
gALL_v3_c16458	3	3	-	-	-	-	-
gALL_v3_c16459	4	4	-	-	-	-	-
gALL_v3_c1646	19	24	gnl|CDD|189507	pfam00335, Tetraspannin, Tet	gb|AGM32395.1|	tetraspanin-18 [Coptotermes formosanus]	5,00E-41
gALL_v3_c16460	5	2	-	-	-	-	-
gALL_v3_c16461	7	12	gnl|CDD|190457	pfam02872, 5_nucleotid_C, 5'	-	-	-
gALL_v3_c16462	4	2	-	-	-	-	-
gALL_v3_c16463	1	5	gnl|CDD|34668	COG5064, SRP1, Karyopherin (	ref|XP_001123270.2|	PREDICTED: importin subunit alpha-like [Apis mellifera]	2,00E-80
gALL_v3_c16465	4	2	-	-	-	-	-
gALL_v3_c16466	2	2	gnl|CDD|189397	pfam00112, Peptidase_C1, Pa	ref|XP_003492760.1|	PREDICTED: hypothetical protein LOC100745708 [Bombus impatiens]	1,00E-34
gALL_v3_c16467	1	6	-	-	-	-	-
gALL_v3_c1647	5	11	-	-	-	-	-
gALL_v3_c16470	6	5	-	-	-	-	-
gALL_v3_c16471	0	5	-	-	-	-	-

gALL_v3_c16473	3	4	-	-	-	-	-
gALL_v3_c16475	3	2	-	-	-	-	-
gALL_v3_c16476	4	5	-	-	-	-	-
gALL_v3_c16477	4	7	gnl|CDD|34786	COG5187, RPN7, 26S proteasc	ref|XP_003696943.1|	PREDICTED: 26S proteasome non-ATPase regulatory subunit 6-like [Apis florea] ref|XP_006568814.1| PREDICTED: 26S proteasome non-ATPase regulatory subunit 6-like [A	1,00E-104
gALL_v3_c16478	2	4	-	-	-	-	-
gALL_v3_c16479	3	1	-	-	-	-	-
gALL_v3_c16480	3	2	gnl|CDD|99970	cd03796, GT1_PIG-A_like, This	ref|XP_001950553.2|	PREDICTED: n-acetylglucosaminyl-phosphatidylinositol biosynthetic protein-like [Acyrthosiphon pisum]	2,00E-69
gALL_v3_c16481	4	3	-	-	ref|XP_002423451.1|	synembryn-A, putative [Pediculus humanus corporis] gb|EEB10713.1| synembryn-A, putative [Pediculus humanus corporis]	1,00E-07
gALL_v3_c16482	6	5	gnl|CDD|143994	pfam00244, 14-3-3, 14-3-3 prc	gb|AFZ78827.1|	14-3-3 protein [Coptotermes formosanus]	5,00E-98
gALL_v3_c16483	3	4	-	-	ref|XP_001688628.1|	AGAP001053-PC [Anopheles gambiae str. PEST] tpg|DAA05513.1| TPA_inf: troponin I isoform 6b1 [Anopheles gambiae str. PEST] gb|EDO63969.1| AGAP001053-PC [Anoph	4,00E-14
gALL_v3_c16484	2	5	gnl|CDD|177177	MTH00116, COX1, cytochrome	ref|YP_006503975.1|	cytochrome c oxidase subunit I (mitochondrion) [Heterotermes sp. SLC-2012] gb|AFM92475.1| cytochrome c oxidase subunit I (mitochondrion) [Heterotermes sp. SLC-201	2,00E-06
gALL_v3_c16485	7	7	-	-	-	-	-
gALL_v3_c16486	2	4	gnl|CDD|29257	cd00200, WD40, WD40 domai	ref|XP_970038.1|	PREDICTED: similar to CG8064 CG8064-PA [Tribolium castaneum] gb|EFA00178.1| hypothetical protein TcasGA2_TC003001 [Tribolium castaneum]	4,00E-60
gALL_v3_c16487	4	2	-	-	-	-	-
gALL_v3_c16488	1	3	-	-	ref|XP_001664301.1|	ancient conserved domain protein 2 (cyclin m2) [Aedes aegypti] gb|EAT33657.1| AAEL014059-PA [Aedes aegypti]	1,00E-18
gALL_v3_c1649	3	9	-	-	ref|XP_002412413.1|	cohesin subunit rad21, putative [Ixodes scapularis] gb|EEC15236.1| cohesin subunit rad21, putative [Ixodes scapularis]	2,00E-07
gALL_v3_c16490	4	4	gnl|CDD|129694	TIGR00606, rad50, rad50. All	ref|XP_004697045.1|	PREDICTED: DNA repair protein RAD50 [Echinops telfairi]	3,00E-56
gALL_v3_c16492	4	4	gnl|CDD|34783	COG5184, ATS1, Alpha-tubulin	ref|XP_001865743.1|	hect E3 ubiquitin ligase [Culex quinquefasciatus] gb|EDS42190.1| hect E3 ubiquitin ligase [Culex quinquefasciatus]	2,00E-65
gALL_v3_c16494	1	4	-	-	-	-	-
gALL_v3_c16496	9	9	-	-	-	-	-
gALL_v3_c16497	5	5	gnl|CDD|187593	cd05332, 11beta-HSD1_like_S	ref|XP_002430574.1|	alcohol dehydrogenase, putative [Pediculus humanus corporis] gb|EEB17836.1| alcohol dehydrogenase, putative [Pediculus humanus corporis]	4,00E-87
gALL_v3_c16498	8	4	gnl|CDD|188092	TIGR00861, MIP, MIP family ch	emb|CBY77924.1|	aquaporin [Blattella germanica]	7,00E-18
gALL_v3_c16499	1	3	-	-	gb|EHJ78550.1|	hypothetical protein KGM_10559 [Danaus plexippus]	1,00E-30
gALL_v3_c165	16	11	gnl|CDD|143885	pfam00108, Thiolase_N, Thiola	gb|EHJ76018.1|	fatty acid beta-oxidation complex subunit beta [Danaus plexippus]	1,00E-175
gALL_v3_c1650	10	14	-	-	-	-	-
gALL_v3_c16500	0	4	-	-	-	-	-
gALL_v3_c16501	2	3	gnl|CDD|192134	pfam08718, GLTP, Glycolipid tr	ref|XP_006617401.1|	PREDICTED: pleckstrin homology domain-containing family A member 8-like [Apis dorsata]	3,00E-32
gALL_v3_c16502	0	4	-	-	-	-	-
gALL_v3_c16503	0	6	-	-	-	-	-
gALL_v3_c16504	3	2	gnl|CDD|58651	cd00927, Cyt_c_Oxidase_VIc,	ref|XP_001945818.1|	PREDICTED: cytochrome c oxidase subunit 6C-like [Acyrthosiphon pisum]	1,00E-16
gALL_v3_c16505	4	8	gnl|CDD|144379	pfam00755, Carn_acyltransf, C	ref|XP_975499.1|	PREDICTED: similar to vesamicol binding protein, putative [Tribolium castaneum]	1,00E-113
gALL_v3_c16506	4	2	-	-	-	-	-
gALL_v3_c16507	6	5	-	-	ref|XP_001943520.2|	PREDICTED: hypothetical protein LOC100163960 [Acyrthosiphon pisum]	2,00E-17
gALL_v3_c16508	2	4	-	-	-	-	-
gALL_v3_c16509	4	3	-	-	ref|XP_003695523.1|	PREDICTED: mitochondrial sodium/hydrogen exchanger 9B2-like [Apis florea]	2,00E-67
gALL_v3_c1651	3	4	gnl|CDD|88576	cd05136, RasGAP_DAB2IP, The	ref|XP_002427120.1|	conserved hypothetical protein [Pediculus humanus corporis] gb|EEB14382.1| conserved hypothetical protein [Pediculus humanus corporis]	2,00E-46
gALL_v3_c16511	4	4	-	-	-	-	-
gALL_v3_c16513	1	6	-	-	-	-	-
gALL_v3_c16514	2	2	-	-	gb|EFN65650.1|	Uncharacterized protein KIAA1843 [Camponotus floridanus]	1,00E-08
gALL_v3_c16515	3	2	-	-	-	-	-
gALL_v3_c16516	2	3	-	-	-	-	-
gALL_v3_c16517	4	2	-	-	-	-	-
gALL_v3_c16518	3	2	-	-	ref|XP_003245382.1|	PREDICTED: hypothetical protein LOC100166039 [Acyrthosiphon pisum]	4,00E-51
gALL_v3_c16519	3	3	-	-	-	-	-
gALL_v3_c1652	5	3	gnl|CDD|181365	PRK08299, PRK08299, isocitra	gb|AGM32733.1|	isocitrate dehydrogenase-like protein, partial [Coptotermes formosanus]	3,00E-84
gALL_v3_c16521	3	2	gnl|CDD|178327	PLN02726, PLN02726, dolichyl	gb|AGM32186.1|	dolichyl-phosphate mannosyltransferase-like protein [Coptotermes formosanus]	2,00E-78
gALL_v3_c16522	2	3	-	-	-	-	-
gALL_v3_c16524	2	3	-	-	gb|EGI65659.1|	Histone deacetylase complex subunit SAP30-like protein [Acromyrmex echinatior]	7,00E-27
gALL_v3_c16525	4	4	-	-	-	-	-
gALL_v3_c16526	0	5	gnl|CDD|190471	pfam02906, Fe_hyd_lg_C, Iron	ref|XP_001808932.1|	PREDICTED: similar to nitrate, fromate, iron dehydrogenase [Tribolium castaneum] gb|EFA09440.1| hypothetical protein TcasGA2_TC010652 [Tribolium castaneum]	2,00E-61
gALL_v3_c16527	2	6	-	-	ref|XP_002424813.1|	tumor endothelial marker 7 precursor, putative [Pediculus humanus corporis] gb|EEB12075.1| tumor endothelial marker 7 precursor, putative [Pediculus humanus corpori	6,00E-71
gALL_v3_c16528	10	13	-	-	-	-	-
gALL_v3_c16529	3	8	-	-	-	-	-
gALL_v3_c1653	6	5	gnl|CDD|145898	pfam02990, EMP70, Endomen	gb|EJY58030.1|	AAEL017204-PA [Aedes aegypti]	1,00E-98
gALL_v3_c16530	5	2	-	-	ref|WP_007302981.1|	Ankyrin repeat domain protein [Wolbachia endosymbiont of Culex quinquefasciatus] gb|EEB55313.1| Ankyrin repeat domain protein [Wolbachia endosymbiont of Culex q	3,00E-21
gALL_v3_c16531	2	2	-	-	ref|XP_002424723.1|	conserved hypothetical protein [Pediculus humanus corporis] gb|EEB11985.1| conserved hypothetical protein [Pediculus humanus corporis]	7,00E-11
gALL_v3_c16533	3	2	-	-	-	-	-
gALL_v3_c16534	3	6	-	-	-	-	-
gALL_v3_c16535	3	9	gnl|CDD|128621	smart00326, SH3, Src homolog	gb|EFN66216.1|	Intersectin-1 [Camponotus floridanus]	1,00E-60
gALL_v3_c16537	2	4	gnl|CDD|29151	cd00189, TPR, Tetratricopeptic	ref|XP_003699533.1|	PREDICTED: serine/threonine-protein phosphatase 5-like [Megachile rotundata]	8,00E-70
gALL_v3_c16538	3	4	-	-	dbj|BAG65666.1|	epidermal growth factor receptor [Gryllus bimaculatus]	3,00E-77
gALL_v3_c1654	20	23	-	-	ref|XP_002430993.1|	immunodeficiency virus type I enhancer binding protein, putative [Pediculus humanus corporis] gb|EEB18255.1| immunodeficiency virus type I enhancer binding protein,	1,00E-52
gALL_v3_c16540	0	4	-	-	ref|XP_003397216.1|	PREDICTED: vacuolar protein sorting-associated protein 13C-like isoform 2 [Bombus terrestris]	1,00E-15
gALL_v3_c16541	2	7	gnl|CDD|192455	pfam10075, PCI_Csn8, COP9 s	gb|AGJ84698.1|	26S proteasome non-ATPase regulatory subunit 8 [Laodelphax striatella]	2,00E-62
gALL_v3_c16543	2	7	gnl|CDD|119335	cd06565, GH20_GcnA-like, Gly	ref|XP_968467.1|	PREDICTED: similar to conserved hypothetical protein [Tribolium castaneum] gb|EFA10074.1| hypothetical protein TcasGA2_TC012248 [Tribolium castaneum]	1,00E-108
gALL_v3_c16544	8	12	-	-	-	-	-
gALL_v3_c16545	2	7	-	-	-	-	-
gALL_v3_c16546	2	4	gnl|CDD|178935	PRK00222, PRK00222, methior	ref|XP_003704590.1|	PREDICTED: methionine-R-sulfoxide reductase B1-like isoform 1 [Megachile rotundata]	2,00E-56
gALL_v3_c16547	5	7	-	-	-	-	-
gALL_v3_c16548	3	1	gnl|CDD|178559	PLN02977, PLN02977, glutathi	ref|XP_002425312.1|	glutathione synthetase, putative [Pediculus humanus corporis] gb|EEB12574.1| glutathione synthetase, putative [Pediculus humanus corporis]	2,00E-48
gALL_v3_c16549	0	5	-	-	gb|AEM44817.1|	unknown [Pectinophora gossypiella]	7,00E-06
gALL_v3_c16550	5	6	-	-	-	-	-
gALL_v3_c16553	0	4	gnl|CDD|72943	cd04658, Piwi_piwi-like_Euk, F	gb|AGO85970.1|	PIWI 2, partial [Locusta migratoria]	3,00E-43
gALL_v3_c16554	2	3	-	-	-	-	-
gALL_v3_c16555	1	4	-	-	-	-	-
gALL_v3_c16556	0	5	-	-	ref|XP_003707985.1|	PREDICTED: leucine-rich repeat and calponin homology domain-containing protein 1-like [Megachile rotundata]	2,00E-16
gALL_v3_c16558	2	4	-	-	-	-	-
gALL_v3_c16559	2	4	-	-	-	-	-
gALL_v3_c1656	3	4	-	-	-	-	-
gALL_v3_c16560	3	2	-	-	ref|XP_003402654.1|	PREDICTED: hypothetical protein LOC100652098 [Bombus terrestris]	2,00E-23
gALL_v3_c16561	3	4	gnl|CDD|189810	pfam01030, Recep_L_domain,	gb|EFN75184.1|	Epidermal growth factor receptor [Harpegnathos saltator]	1,00E-114
gALL_v3_c16562	6	6	-	-	-	-	-
gALL_v3_c16563	0	3	-	-	-	-	-
gALL_v3_c16564	3	4	-	-	-	-	-
gALL_v3_c16565	4	4	-	-	-	-	-
gALL_v3_c16567	3	4	-	-	-	-	-
gALL_v3_c16568	2	3	-	-	-	-	-
gALL_v3_c16569	2	4	gnl|CDD|189721	pfam00791, ZU5, ZU5 domain	gb|ELK16870.1|	Tight junction protein ZO-1 [Pteropus alecto]	5,00E-12
gALL_v3_c1657	8	5	gnl|CDD|145177	pfam01873, eIF-5_eIF-2B, Don	dbj|BAH71330.1|	ACYPI004549 [Acyrthosiphon pisum]	1,00E-113
gALL_v3_c16570	2	2	-	-	-	-	-
gALL_v3_c16571	2	2	-	-	-	-	-
gALL_v3_c16572	2	2	gnl|CDD|189445	pfam00199, Catalase, Catalase	gb|AFV36369.1|	catalase [Reticulitermes flavipes]	5,00E-26
gALL_v3_c16574	8	7	gnl|CDD|189835	pfam01086, Clathrin_lg_ch, Cl	ref|XP_001657495.1|	clathrin light chain [Aedes aegypti] gb|EAT47993.1| AAEL000934-PA, partial [Aedes aegypti]	7,00E-52
gALL_v3_c16575	2	2	gnl|CDD|189970	pfam01395, PBP_GOBP, PBP/G	gb|AAM77027.1|	pheromone binding protein [Rhyparobia maderae]	2,00E-43
gALL_v3_c16576	1	2	-	-	-	-	-
gALL_v3_c16577	2	7	-	-	ref|XP_006825282.1|	PREDICTED: NAD-dependent protein deacetylase sirtuin-2-like [Saccoglossus kowalevskii]	1,00E-18
gALL_v3_c16579	6	3	-	-	-	-	-
gALL_v3_c1658	9	10	gnl|CDD|29321	cd00246, RabGEF, Nucleotide	dbj|BAN21248.1|	conserved hypothetical protein [Riptortus pedestris]	2,00E-34
gALL_v3_c16580	6	11	gnl|CDD|29257	cd00200, WD40, WD40 domai	ref|XP_006566682.1|	PREDICTED: transcription initiation factor TFIID subunit 5 isoform X1 [Apis mellifera]	0
gALL_v3_c16581	1	6	-	-	-	-	-
gALL_v3_c16582	1	3	-	-	-	-	-
gALL_v3_c16583	2	4	-	-	gb|EFN71797.1|	Tudor domain-containing protein 3 [Camponotus floridanus]	2,00E-26
gALL_v3_c16584	1	5	-	-	-	-	-
gALL_v3_c16585	4	4	-	-	gb|EFA13684.1|	hypothetical protein TcasGA2_TC016048 [Tribolium castaneum]	2,00E-07
gALL_v3_c16586	3	5	-	-	-	-	-
gALL_v3_c16588	3	3	-	-	-	-	-
gALL_v3_c16589	5	4	-	-	ref|XP_001946528.1|	PREDICTED: EF-hand calcium-binding domain-containing protein 4A-like [Acyrthosiphon pisum]	1,00E-09
gALL_v3_c1659	6	17	gnl|CDD|179368	PRK02106, PRK02106, choline	ref|XP_972532.1|	PREDICTED: similar to glucose dehydrogenase [Tribolium castaneum] gb|EFA05532.1| hypothetical protein TcasGA2_TC015719 [Tribolium castaneum]	1,00E-173
gALL_v3_c16592	3	4	-	-	-	-	-
gALL_v3_c16593	4	2	-	-	-	-	-
gALL_v3_c16594	3	1	gnl|CDD|58503	cd01826, acyloxyacyl_hydrola:	gb|EKC27176.1|	Acyloxyacyl hydrolase [Crassostrea gigas]	2,00E-23
gALL_v3_c16595	6	7	gnl|CDD|190869	pfam04112, Mak10, Mak10 su	ref|XP_002427016.1|	conserved hypothetical protein [Pediculus humanus corporis] gb|EEB14278.1| conserved hypothetical protein [Pediculus humanus corporis]	1,00E-164
gALL_v3_c16596	9	5	-	-	-	-	-
gALL_v3_c16597	3	6	-	-	-	-	-
gALL_v3_c16598	4	2	-	-	-	-	-
gALL_v3_c16599	2	3	-	-	-	-	-
gALL_v3_c166	9	10	-	-	ref|XP_003703647.1|	PREDICTED: uncharacterized protein LOC100883777, partial [Megachile rotundata]	1,00E-154
gALL_v3_c1660	11	11	-	-	-	-	-
gALL_v3_c16600	0	4	-	-	gb|EFA02455.1|	hypothetical protein TcasGA2_TC008143 [Tribolium castaneum]	4,00E-11
gALL_v3_c16601	2	3	-	-	-	-	-
gALL_v3_c16602	0	6	-	-	gb|EFZ17147.1|	hypothetical protein SINV_09383 [Solenopsis invicta]	2,00E-16
gALL_v3_c16603	0	5	-	-	-	-	-
gALL_v3_c16604	0	4	-	-	-	-	-
gALL_v3_c16605	4	2	-	-	-	-	-
gALL_v3_c16606	7	8	gnl|CDD|128538	smart00242, MYSc, Myosin. La	ref|XP_003703214.1|	PREDICTED: unconventional myosin-Va [Megachile rotundata]	1,00E-156
gALL_v3_c16607	1	3	-	-	-	-	-
gALL_v3_c16609	4	10	gnl|CDD|189411	pfam00135, COesterase, Carb	gb|ABB89946.1|	ace1 type acetylcholinesterase [Blattella germanica]	1,00E-131
gALL_v3_c1661	14	14	gnl|CDD|153420	cd07920, Pumilio, Pumilio-fam	ref|XP_003485990.1|	PREDICTED: pumilio homolog 1-like [Bombus impatiens]	1,00E-101
gALL_v3_c16610	0	3	gnl|CDD|147061	pfam04710, Pellino, Pellino. P	ref|XP_967308.1|	PREDICTED: similar to AGAP004232-PA isoform 1 [Tribolium castaneum]	3,00E-48
gALL_v3_c16611	1	3	-	-	-	-	-
gALL_v3_c16612	4	3	-	-	ref|XP_003248303.1|	PREDICTED: craniofacial development protein 2-like [Acyrthosiphon pisum]	5,00E-26
gALL_v3_c16613	2	3	-	-	-	-	-
gALL_v3_c16614	3	3	-	-	-	-	-
gALL_v3_c16615	2	2	-	-	-	-	-
gALL_v3_c16616	12	18	-	-	-	-	-
gALL_v3_c16617	2	2	-	-	-	-	-
gALL_v3_c16618	3	3	-	-	-	-	-
gALL_v3_c16619	2	2	gnl|CDD|144673	pfam01166, TSC22, TSC-22/dip	ref|NP_001162965.1|	bunched, isoform B [Drosophila melanogaster] sp|Q24522.2|BUN1_DROME RecName: Full=Protein bunched, class 1/class 3/D/E isoforms; AltName: Full=Protein shortsigh	2,00E-37
gALL_v3_c1662	8	8	-	-	-	-	-
gALL_v3_c16620	3	3	gnl|CDD|185575	PTZ00361, PTZ00361, 26 prote	gb|EHJ72875.1|	26S protease regulatory subunit [Danaus plexippus]	5,00E-76
gALL_v3_c16621	1	3	-	-	-	-	-
gALL_v3_c16622	3	4	-	-	-	-	-
gALL_v3_c16623	2	2	gnl|CDD|162296	TIGR01311, glycerol_kin, glyce	gb|AGM32757.1|	putative glycerol kinase 3-like protein, partial [Coptotermes formosanus]	5,00E-65
gALL_v3_c16624	0	4	-	-	-	-	-

gALL_v3_c16625	2	2	gnl|CDD|191234	pfam05222, AlaDh_PNT_N, Al	gb|EFX83224.1|	hypothetical protein DAPPUDRAFT_315656 [Daphnia pulex]	3,00E-25
gALL_v3_c16626	7	11	gnl|CDD|73008	cd03249, ABC_MTABC3_MDL1	ref|XP_966724.1|	PREDICTED: similar to AGAP002717-PA [Tribolium castaneum] gb|EFA02662.1| hypothetical protein TcasGA2_TC008383 [Tribolium castaneum]	1,00E-134
gALL_v3_c16628	0	5	-	-	-	-	-
gALL_v3_c16630	3	1	-	-	gb|ELT97999.1|	hypothetical protein CAPTEDRAFT_208751 [Capitella teleta]	1,00E-48
gALL_v3_c16631	2	2	-	-	gb|EFN88383.1|	Forkhead box protein P4 [Harpegnathos saltator]	4,00E-24
gALL_v3_c16632	2	2	-	-	-	-	-
gALL_v3_c16633	2	2	-	-	-	-	-
gALL_v3_c16634	3	6	gnl|CDD|30768	COG0419, SbcC, ATPase involv	ref|XP_003963100.1|	PREDICTED: GRIP1-associated protein 1-like [Takifugu rubripes]	8,00E-23
gALL_v3_c16635	2	4	-	-	ref|XP_005095693.1|	PREDICTED: putative uncharacterized protein FLJ37770-like [Aplysia californica]	2,00E-30
gALL_v3_c16636	3	4	-	-	-	-	-
gALL_v3_c16637	3	5	-	-	ref|XP_003402240.1|	PREDICTED: inositol-pentakisphosphate 2-kinase-like [Bombus terrestris]	5,00E-08
gALL_v3_c16638	4	5	-	-	ref|XP_002426327.1|	conserved hypothetical protein [Pediculus humanus corporis] gb|EEB13589.1| conserved hypothetical protein [Pediculus humanus corporis]	2,00E-74
gALL_v3_c16639	0	5	-	-	-	-	-
gALL_v3_c1664	7	9	gnl|CDD|28945	cd00063, FN3, Fibronectin typ	ref|NP_001260597.1|	Leukocyte-antigen-related-like, isoform I [Drosophila melanogaster] gb|AGB93132.1| Leukocyte-antigen-related-like, isoform I [Drosophila melanogaster]	1,00E-173
gALL_v3_c16643	2	6	gnl|CDD|133259	cd01850, CDC_Septin, CDC/Se	ref|XP_003693877.1|	PREDICTED: septin-2-like [Apis florea] ref|XP_006565397.1| PREDICTED: septin-2 isoform X3 [Apis mellifera] ref|XP_006613787.1| PREDICTED: septin-2-like isoform X3 [Ap	1,00E-151
gALL_v3_c16645	1	5	-	-	-	-	-
gALL_v3_c16646	2	5	-	-	-	-	-
gALL_v3_c16647	16	23	-	-	gb|EFN67906.1|	PiggyBac transposable element-derived protein 4 [Camponotus floridanus]	2,00E-26
gALL_v3_c16648	2	2	-	-	-	-	-
gALL_v3_c16649	0	4	-	-	-	-	-
gALL_v3_c1665	24	25	-	-	-	-	-
gALL_v3_c16650	4	4	-	-	-	-	-
gALL_v3_c16651	3	3	gnl|CDD|129661	TIGR00570, cdk7, CDK-activati	gb|EHJ70288.1|	hypothetical protein KGM_19668 [Danaus plexippus]	3,00E-91
gALL_v3_c16652	1	5	-	-	ref|XP_002426607.1|	conserved hypothetical protein [Pediculus humanus corporis] gb|EEB13869.1| conserved hypothetical protein [Pediculus humanus corporis]	3,00E-33
gALL_v3_c16653	1	5	-	-	gb|EFN63884.1|	hypothetical protein EAG_08086 [Camponotus floridanus]	6,00E-09
gALL_v3_c16654	1	6	-	-	-	-	-
gALL_v3_c16655	2	3	-	-	-	-	-
gALL_v3_c16656	2	3	-	-	ref|XP_002426048.1|	conserved hypothetical protein [Pediculus humanus corporis] gb|EEB13310.1| conserved hypothetical protein [Pediculus humanus corporis]	8,00E-06
gALL_v3_c16657	4	5	gnl|CDD|128336	smart00020, Tryp_SPc, Trypsir	gb|AGM32320.1|	serine protease, partial [Coptotermes formosanus]	1,00E-103
gALL_v3_c16658	5	6	-	-	-	-	-
gALL_v3_c16659	5	8	gnl|CDD|29261	cd00204, ANK, ankyrin repeat:	ref|XP_003424076.1|	PREDICTED: E3 ubiquitin-protein ligase mib1-like [Nasonia vitripennis]	0
gALL_v3_c1666	9	8	-	-	gb|AGM32323.1|	hypothetical protein, partial [Coptotermes formosanus]	1,00E-12
gALL_v3_c16660	1	4	-	-	-	-	-
gALL_v3_c16661	4	7	-	-	-	-	-
gALL_v3_c16662	5	2	-	-	-	-	-
gALL_v3_c16663	3	3	-	-	ref|XP_005107070.1|	PREDICTED: protein FAM91A1-like [Aplysia californica]	6,00E-25
gALL_v3_c16664	8	4	-	-	-	-	-
gALL_v3_c16665	6	3	-	-	-	-	-
gALL_v3_c16666	3	1	-	-	-	-	-
gALL_v3_c16667	3	1	-	-	-	-	-
gALL_v3_c16668	3	2	gnl|CDD|113501	pfam04733, Coatomer_E, Coa	ref|XP_003691875.1|	PREDICTED: LOW QUALITY PROTEIN: coatomer subunit epsilon-like [Apis florea]	2,00E-32
gALL_v3_c16669	8	8	gnl|CDD|128679	smart00398, HMG, high mobil	ref|XP_006824510.1|	PREDICTED: high mobility group protein 20A-like isoform X2 [Saccoglossus kowalevskii]	1,00E-102
gALL_v3_c1667	6	7	-	-	-	-	-
gALL_v3_c16670	3	2	-	-	-	-	-
gALL_v3_c16672	6	4	-	-	-	-	-
gALL_v3_c16673	2	5	-	-	ref|XP_975294.1|	PREDICTED: similar to nephrin [Tribolium castaneum] gb|EFA00099.1| hypothetical protein TcasGA2_TC002914 [Tribolium castaneum]	1,00E-66
gALL_v3_c16675	2	3	-	-	-	-	-
gALL_v3_c16676	3	9	-	-	-	-	-
gALL_v3_c16677	2	3	-	-	-	-	-
gALL_v3_c16678	0	4	-	-	-	-	-
gALL_v3_c16679	5	2	-	-	-	-	-
gALL_v3_c1668	3	5	-	-	ref|XP_003425499.1|	PREDICTED: hippocampus abundant transcript 1 protein-like isoform 3 [Nasonia vitripennis]	1,00E-10
gALL_v3_c16680	2	5	-	-	-	-	-
gALL_v3_c16681	3	6	gnl|CDD|191096	pfam04799, Fzo_mitofusin, fzo	ref|XP_001605562.2|	PREDICTED: transmembrane GTPase Marf-like isoform 1 [Nasonia vitripennis] ref|XP_003426351.1| PREDICTED: transmembrane GTPase Marf-like isoform 2 [Nasonia vitri	3,00E-46
gALL_v3_c16682	3	7	-	-	-	-	-
gALL_v3_c16683	2	3	-	-	-	-	-
gALL_v3_c16684	0	6	-	-	-	-	-
gALL_v3_c16685	2	10	gnl|CDD|173725	cd06608, STKc_myosinIII_like,	ref|XP_002024737.1|	GL22452 [Drosophila persimilis] gb|EDW30185.1| GL22452 [Drosophila persimilis]	2,00E-99
gALL_v3_c16686	1	4	gnl|CDD|162084	TIGR00879, SP, MFS transport	gb|ETN59145.1|	sugar transporter [Anopheles darlingi]	6,00E-41
gALL_v3_c16687	3	5	-	-	-	-	-
gALL_v3_c16688	1	4	-	-	ref|XP_001813433.1|	PREDICTED: similar to paraplegin [Tribolium castaneum] gb|EFA00737.1| hypothetical protein TcasGA2_TC003619 [Tribolium castaneum]	6,00E-57
gALL_v3_c16689	4	2	gnl|CDD|29332	cd00257, Fascin, Fascin-like dc	gb|ENN71250.1|	hypothetical protein YQE_12177, partial [Dendroctonus ponderosae] gb|ERL86715.1| hypothetical protein D910_04121 [Dendroctonus ponderosae]	3,00E-63
gALL_v3_c1669	8	11	-	-	-	-	-
gALL_v3_c16690	2	1	-	-	gb|AGM32136.1|	hypothetical protein [Coptotermes formosanus]	3,00E-10
gALL_v3_c16691	3	5	-	-	-	-	-
gALL_v3_c16692	4	5	-	-	-	-	-
gALL_v3_c16693	1	5	-	-	-	-	-
gALL_v3_c16694	4	2	-	-	-	-	-
gALL_v3_c16695	3	4	-	-	-	-	-
gALL_v3_c16696	3	3	-	-	-	-	-
gALL_v3_c16697	3	2	-	-	-	-	-
gALL_v3_c16698	5	6	gnl|CDD|190571	pfam03226, Yippee, Yippee pu	ref|XP_966830.2|	PREDICTED: similar to Protein yippee-like 1 [Tribolium castaneum]	7,00E-79
gALL_v3_c167	10	23	-	-	-	-	-
gALL_v3_c1670	9	10	gnl|CDD|190170	pfam01956, DUF106, Integral	ref|XP_002423708.1|	protein pob, putative [Pediculus humanus corporis] gb|EEB10970.1| protein pob, putative [Pediculus humanus corporis]	1,00E-136
gALL_v3_c16700	2	7	gnl|CDD|163216	TIGR03328, salvage_mtnB, me	ref|NP_001123086.2|	APAF1 interacting protein [Nasonia vitripennis]	9,00E-69
gALL_v3_c16701	0	6	-	-	ref|XP_002425602.1|	guanine-nucleotide exchange factor c3g, putative [Pediculus humanus corporis] gb|EEB12864.1| guanine-nucleotide exchange factor c3g, putative [Pediculus humanus cor	3,00E-26
gALL_v3_c16702	4	3	-	-	ref|XP_002432957.1|	B-cell lymphoma/leukemia, putative [Pediculus humanus corporis] gb|EEB20219.1| B-cell lymphoma/leukemia, putative [Pediculus humanus corporis]	5,00E-25
gALL_v3_c16704	1	3	gnl|CDD|173611	PTZ00421, PTZ00421, coronin;	ref|XP_002426462.1|	Coronin-6, putative [Pediculus humanus corporis] gb|EEB13724.1| Coronin-6, putative [Pediculus humanus corporis]	3,00E-95
gALL_v3_c16706	3	3	-	-	ref|XP_975582.2|	PREDICTED: similar to Pecanex-like protein 1 (Pecanex homolog) [Tribolium castaneum] gb|EFA01728.1| hypothetical protein TcasGA2_TC007308 [Tribolium castaneum]	1,00E-10
gALL_v3_c16707	2	7	-	-	-	-	-
gALL_v3_c16708	4	2	-	-	-	-	-
gALL_v3_c16711	3	2	gnl|CDD|128517	smart00221, STYKc, Protein ki	gb|EFN87411.1|	Serine/threonine-protein kinase tousled-like 2 [Harpegnathos saltator]	1,00E-120
gALL_v3_c16712	1	5	-	-	-	-	-
gALL_v3_c16714	2	7	gnl|CDD|63838	cd04517, TLF, TBP-like factors	gb|ENN76215.1|	hypothetical protein YQE_07182, partial [Dendroctonus ponderosae] gb|ERL85312.1| hypothetical protein D910_02732 [Dendroctonus ponderosae]	1,00E-69
gALL_v3_c16716	4	9	-	-	-	-	-
gALL_v3_c16717	3	5	-	-	-	-	-
gALL_v3_c16718	3	6	gnl|CDD|146145	pfam03357, Snf7, Snf7. This fa	gb|EGI63302.1|	Charged multivesicular body protein 3 [Acromyrmex echinatior]	5,00E-85
gALL_v3_c16719	3	6	-	-	-	-	-
gALL_v3_c1672	18	11	-	-	ref|XP_003424788.1|	PREDICTED: putative phosphoenolpyruvate synthase-like [Nasonia vitripennis]	4,00E-08
gALL_v3_c16720	1	4	-	-	-	-	-
gALL_v3_c16721	3	2	-	-	-	-	-
gALL_v3_c16722	2	5	-	-	-	-	-
gALL_v3_c16723	3	2	-	-	ref|XP_002424228.1|	predicted protein [Pediculus humanus corporis] gb|EEB11490.1| predicted protein [Pediculus humanus corporis]	6,00E-29
gALL_v3_c16724	7	4	-	-	-	-	-
gALL_v3_c16725	3	10	-	-	ref|XP_001943127.1|	PREDICTED: calsyntenin-1-like isoform 1 [Acyrthosiphon pisum] ref|XP_003245317.1| PREDICTED: calsyntenin-1-like isoform 2 [Acyrthosiphon pisum]	2,00E-35
gALL_v3_c16726	1	6	gnl|CDD|109266	pfam00201, UDPGT, UDP-gluc	gb|AGC84403.1|	glucosyl glucuronosyl transferases [Locusta migratoria]	2,00E-50
gALL_v3_c16727	7	5	-	-	-	-	-
gALL_v3_c16728	3	5	-	-	ref|XP_003400873.1|	PREDICTED: kinase D-interacting substrate of 220 kDa-like [Bombus terrestris]	1,00E-92
gALL_v3_c16729	3	8	-	-	-	-	-
gALL_v3_c1673	4	7	-	-	-	-	-
gALL_v3_c16730	0	6	-	-	-	-	-
gALL_v3_c16731	4	2	-	-	-	-	-
gALL_v3_c16732	2	2	-	-	gb|EFZ20916.1|	hypothetical protein SINV_80847 [Solenopsis invicta]	1,00E-42
gALL_v3_c16733	3	3	-	-	-	-	-
gALL_v3_c16735	3	2	gnl|CDD|178694	PLN03149, PLN03149, peptidy	gb|EFN86591.1|	Peptidyl-prolyl cis-trans isomerase H [Harpegnathos saltator]	5,00E-52
gALL_v3_c16736	7	9	gnl|CDD|128842	smart00572, DZF, domain in D	ref|XP_002428724.1|	zinc finger protein RNA binding protein, putative [Pediculus humanus corporis] gb|EEB15986.1| zinc finger protein RNA binding protein, putative [Pediculus humanus corp	1,00E-29
gALL_v3_c16737	4	12	-	-	gb|EFN83204.1|	Large proline-rich protein BAT2 [Harpegnathos saltator]	5,00E-10
gALL_v3_c16739	3	6	-	-	-	-	-
gALL_v3_c1674	9	17	-	-	-	-	-
gALL_v3_c16740	1	3	-	-	-	-	-
gALL_v3_c16741	1	7	-	-	-	-	-
gALL_v3_c16742	5	4	gnl|CDD|100083	cd06162, S2P-M50_PDZ_SREB	ref|XP_003393540.1|	PREDICTED: membrane-bound transcription factor site-2 protease-like [Bombus terrestris]	3,00E-68
gALL_v3_c16743	3	10	-	-	emb|CDJ26737.1|	cathepsin F-like cysteine peptidase protein [Tityus serrulatus]	1,00E-10
gALL_v3_c16744	3	2	-	-	-	-	-
gALL_v3_c16745	3	6	-	-	-	-	-
gALL_v3_c16746	1	5	-	-	-	-	-
gALL_v3_c16747	1	6	-	-	-	-	-
gALL_v3_c16748	3	2	-	-	ref|XP_002170374.2|	PREDICTED: uncharacterized protein LOC100205880, partial [Hydra vulgaris]	4,00E-20
gALL_v3_c16749	2	3	-	-	-	-	-
gALL_v3_c1675	3	7	-	-	-	-	-
gALL_v3_c16750	3	1	-	-	-	-	-
gALL_v3_c16751	2	8	-	-	ref|XP_002424204.1|	hypothetical protein Phum_PHUM118420 [Pediculus humanus corporis] gb|EEB11466.1| hypothetical protein Phum_PHUM118420 [Pediculus humanus corporis]	4,00E-08
gALL_v3_c16754	1	4	gnl|CDD|178137	PLN02522, PLN02522, ATP citr	gb|EFA05014.1|	hypothetical protein TcasGA2_TC015096 [Tribolium castaneum]	7,00E-81
gALL_v3_c16755	7	5	-	-	-	-	-
gALL_v3_c16756	3	5	gnl|CDD|48634	cd03419, GRX_GRXh_1_2_like	ref|XP_003699811.1|	PREDICTED: glutaredoxin-C4-like [Megachile rotundata]	9,00E-32
gALL_v3_c16757	2	2	-	-	-	-	-
gALL_v3_c16758	3	3	-	-	-	-	-
gALL_v3_c1676	18	25	-	-	-	-	-
gALL_v3_c16760	4	6	-	-	-	-	-
gALL_v3_c16761	5	4	-	-	-	-	-
gALL_v3_c16762	2	9	-	-	gb|EGI62819.1|	SET and MYND domain-containing protein 4 [Acromyrmex echinatior]	3,00E-10
gALL_v3_c16763	5	4	-	-	ref|XP_003150557.1|	hypothetical protein LOAG_15015 [Loa loa] gb|EFO13512.1| hypothetical protein LOAG_15015, partial [Loa loa]	4,00E-15
gALL_v3_c16764	3	4	-	-	-	-	-
gALL_v3_c16765	3	2	-	-	-	-	-
gALL_v3_c16767	4	3	-	-	-	-	-
gALL_v3_c16768	2	2	-	-	-	-	-
gALL_v3_c16769	3	1	gnl|CDD|152337	pfam11901, DUF3421, Protein	ref|NP_001233092.1|	uncharacterized protein LOC100164577 [Acyrthosiphon pisum] dbj|BAH71807.1| ACYPI005577 [Acyrthosiphon pisum]	5,00E-32
gALL_v3_c1677	10	10	gnl|CDD|191135	pfam04939, RRS1, Ribosome b	gb|AGC92697.1|	ribosome biogenesis regulatory protein-like protein [Heliconius erato]	1,00E-109
gALL_v3_c16770	3	2	-	-	-	-	-
gALL_v3_c16771	4	2	-	-	-	-	-
gALL_v3_c16772	5	10	gnl|CDD|34653	COG5048, COG5048, FOG: Zn-	gb|ENN76968.1|	hypothetical protein YQE_06536, partial [Dendroctonus ponderosae]	1,00E-128
gALL_v3_c16773	2	7	gnl|CDD|73154	cd01647, RT_LTR, RT_LTR: Rev	ref|XP_005157614.1|	PREDICTED: uncharacterized protein LOC101882541 [Danio rerio]	1,00E-42
gALL_v3_c16774	5	3	-	-	-	-	-
gALL_v3_c16775	6	13	gnl|CDD|30666	COG0318, CaiC, Acyl-CoA synt	ref|XP_002432341.1|	disco-interacting protein, putative [Pediculus humanus corporis] gb|EEB19603.1| disco-interacting protein, putative [Pediculus humanus corporis]	0

gALL_v3_c16776	2	3	-	-	-	-	-
gALL_v3_c16777	3	1	gnl|CDD|151076	pfam10524, NfI_DNAbd_pre-N	gb|EFX79415.1|	hypothetical protein DAPPUDRAFT_52430 [Daphnia pulex]	6,00E-64
gALL_v3_c16778	10	7	-	-	-	-	-
gALL_v3_c16779	3	2	-	-	-	-	-
gALL_v3_c1678	5	12	gnl|CDD|35197	COG5638, COG5638, Uncharac	ref|XP_003425125.1|	PREDICTED: ESF1 homolog isoform 1 [Nasonia vitripennis] ref|XP_003425126.1| PREDICTED: ESF1 homolog isoform 2 [Nasonia vitripennis]	2,00E-87
gALL_v3_c16780	3	1	-	-	-	-	-
gALL_v3_c16781	3	4	gnl|CDD|128621	smart00326, SH3, Src homolog	gb|EGI67260.1|	1-phosphatidylinositol-4,5-bisphosphate phosphodiesterase gamma-1 [Acromyrmex echinatior]	5,00E-65
gALL_v3_c16782	0	4	-	-	-	-	-
gALL_v3_c16784	2	5	-	-	-	-	-
gALL_v3_c16785	5	5	-	-	-	-	-
gALL_v3_c16786	0	6	-	-	gb|EFX90035.1|	hypothetical protein DAPPUDRAFT_300090 [Daphnia pulex]	5,00E-36
gALL_v3_c16787	2	5	-	-	-	-	-
gALL_v3_c16788	4	5	-	-	-	-	-
gALL_v3_c16789	2	2	gnl|CDD|191346	pfam05700, BCAS2, Breast car	ref|XP_003401130.1|	PREDICTED: pre-mRNA-splicing factor SPF27-like [Bombus terrestris] ref|XP_003490588.1| PREDICTED: pre-mRNA-splicing factor SPF27-like [Bombus impatiens]	3,00E-56
gALL_v3_c1679	15	28	-	-	gb|EFN80081.1|	Synaptogyrin-2 [Harpegnathos saltator]	1,00E-61
gALL_v3_c16790	6	3	-	-	-	-	-
gALL_v3_c16791	3	5	-	-	-	-	-
gALL_v3_c16792	3	4	-	-	-	-	-
gALL_v3_c16793	1	5	-	-	-	-	-
gALL_v3_c16794	0	4	-	-	-	-	-
gALL_v3_c16795	2	4	-	-	-	-	-
gALL_v3_c16796	3	3	-	-	ref|XP_006559086.1|	PREDICTED: serine/threonine-protein kinase N [Apis mellifera]	2,00E-27
gALL_v3_c16797	0	4	-	-	-	-	-
gALL_v3_c16799	2	3	-	-	-	-	-
gALL_v3_c1680	9	19	-	-	gb|EFA09839.1|	hypothetical protein TcasGA2_TC011986 [Tribolium castaneum]	0
gALL_v3_c16800	0	4	-	-	-	-	-
gALL_v3_c16802	0	3	gnl|CDD|182269	PRK10150, PRK10150, beta-D-	-	-	-
gALL_v3_c16803	6	10	-	-	-	-	-
gALL_v3_c16804	2	5	-	-	-	-	-
gALL_v3_c16805	1	4	gnl|CDD|129984	TIGR00906, 2A0303, cationic a	ref|XP_001810635.1|	PREDICTED: similar to AGAP001265-PA [Tribolium castaneum]	2,00E-22
gALL_v3_c16806	2	5	gnl|CDD|162101	TIGR00911, 2A0308, L-type an	ref|XP_003485650.1|	PREDICTED: large neutral amino acids transporter small subunit 2-like [Bombus impatiens]	5,00E-50
gALL_v3_c16807	3	3	-	-	-	-	-
gALL_v3_c16808	1	3	-	-	ref|XP_970065.2|	PREDICTED: similar to integral membrane pore glycoprotein gp210, putative [Tribolium castaneum]	2,00E-30
gALL_v3_c16809	2	5	-	-	-	-	-
gALL_v3_c1681	7	14	-	-	-	-	-
gALL_v3_c16810	5	8	-	-	-	-	-
gALL_v3_c16811	3	2	-	-	ref|XP_001847630.1|	helicase with zinc finger [Culex quinquefasciatus] gb|EDS26531.1| helicase with zinc finger [Culex quinquefasciatus]	1,00E-43
gALL_v3_c16812	2	3	-	-	-	-	-
gALL_v3_c16813	3	3	-	-	-	-	-
gALL_v3_c16814	1	3	-	-	-	-	-
gALL_v3_c16815	1	3	-	-	-	-	-
gALL_v3_c16816	0	6	-	-	-	-	-
gALL_v3_c16817	1	5	-	-	-	-	-
gALL_v3_c16818	2	3	-	-	-	-	-
gALL_v3_c16819	3	1	-	-	-	-	-
gALL_v3_c1682	6	15	-	-	ref|XP_003702583.1|	PREDICTED: histone-lysine N-methyltransferase SETMAR-like [Megachile rotundata]	5,00E-13
gALL_v3_c16820	1	4	-	-	-	-	-
gALL_v3_c16821	3	2	gnl|CDD|189851	pfam01131, Topoisom_bac, DI	ref|XP_002424239.1|	DNA topoisomerase 3-beta, putative [Pediculus humanus corporis] gb|EEB11501.1| DNA topoisomerase 3-beta, putative [Pediculus humanus corporis]	1,00E-66
gALL_v3_c16823	1	6	-	-	-	-	-
gALL_v3_c16824	1	6	-	-	-	-	-
gALL_v3_c16826	1	3	-	-	gb|AAH43477.1|	Tle4 protein, partial [Mus musculus]	1,00E-82
gALL_v3_c16827	2	2	-	-	gb|ERL92299.1|	hypothetical protein D910_09616 [Dendroctonus ponderosae]	2,00E-18
gALL_v3_c16828	2	2	-	-	-	-	-
gALL_v3_c16829	2	3	-	-	-	-	-
gALL_v3_c1683	3	6	-	-	-	-	-
gALL_v3_c16831	2	2	-	-	-	-	-
gALL_v3_c16832	0	3	gnl|CDD|173506	PTZ00281, PTZ00281, actin; Pr	gb|AAO40042.2|	actin [Festuca arundinacea]	4,00E-36
gALL_v3_c16833	0	5	-	-	-	-	-
gALL_v3_c16834	2	2	-	-	-	-	-
gALL_v3_c16835	6	6	gnl|CDD|128654	smart00360, RRM, RNA recogi	gb|AEE62081.1|	unknown [Dendroctonus ponderosae]	6,00E-49
gALL_v3_c16837	2	6	-	-	-	-	-
gALL_v3_c16838	2	3	-	-	-	-	-
gALL_v3_c1684	6	12	-	-	-	-	-
gALL_v3_c16841	1	4	-	-	-	-	-
gALL_v3_c16842	0	4	-	-	-	-	-
gALL_v3_c16844	1	4	-	-	-	-	-
gALL_v3_c16845	3	3	-	-	-	-	-
gALL_v3_c16846	3	5	-	-	-	-	-
gALL_v3_c16847	2	5	-	-	-	-	-
gALL_v3_c16848	1	3	-	-	-	-	-
gALL_v3_c16849	1	3	-	-	-	-	-
gALL_v3_c16851	4	3	-	-	-	-	-
gALL_v3_c16852	2	3	-	-	-	-	-
gALL_v3_c16853	1	3	-	-	-	-	-
gALL_v3_c16854	4	4	gnl|CDD|190024	pfam01532, Glyco_hydro_47,	ref|XP_002431612.1|	conserved hypothetical protein [Pediculus humanus corporis] gb|EEB18874.1| conserved hypothetical protein [Pediculus humanus corporis]	2,00E-69
gALL_v3_c16856	4	4	-	-	-	-	-
gALL_v3_c16857	2	3	-	-	ref|XP_004919079.1|	PREDICTED: uncharacterized protein LOC100487167 isoform X3 [Xenopus (Silurana) tropicalis] ref|XP_004919080.1| PREDICTED: uncharacterized protein LOC100487167 is	2,00E-07
gALL_v3_c16858	8	7	gnl|CDD|149256	pfam08074, CHDCT2, CHDCT2	gb|ENN70744.1|	hypothetical protein YQE_12533, partial [Dendroctonus ponderosae]	1,00E-148
gALL_v3_c16859	3	11	-	-	-	-	-
gALL_v3_c1686	10	11	-	-	dbj|BAM18988.1|	krotzkopf verkehrt [Papilio polytes]	5,00E-13
gALL_v3_c16861	2	7	-	-	ref|XP_004715875.1|	PREDICTED: zinc finger protein 570-like [Echinops telfairi]	2,00E-06
gALL_v3_c16862	2	2	-	-	-	-	-
gALL_v3_c16863	3	3	-	-	-	-	-
gALL_v3_c16864	0	4	-	-	-	-	-
gALL_v3_c16865	3	3	gnl|CDD|29799	cd00859, HisRS_anticodon, His	ref|XP_974715.2|	PREDICTED: similar to Histidyl-tRNA synthetase CG6335-PA [Tribolium castaneum]	3,00E-20
gALL_v3_c16867	3	3	-	-	-	-	-
gALL_v3_c16868	1	6	gnl|CDD|190050	pfam01602, Adaptin_N, Adapt	ref|XP_002423396.1|	Coatomer subunit beta, putative [Pediculus humanus corporis] gb|EEB10658.1| Coatomer subunit beta, putative [Pediculus humanus corporis]	1,00E-148
gALL_v3_c16869	3	4	-	-	-	-	-
gALL_v3_c1687	13	12	gnl|CDD|28848	cd02908, Macro_Appr_pase_l	dbj|BAN20995.1|	ganglioside induced differentiation associated protein [Riptortus pedestris]	8,00E-98
gALL_v3_c16870	2	3	-	-	-	-	-
gALL_v3_c16871	1	4	gnl|CDD|189034	cd09864, PIN_Fcf1, PIN domai	ref|XP_004926557.1|	PREDICTED: rRNA-processing protein FCF1 homolog [Bombyx mori]	6,00E-29
gALL_v3_c16872	1	7	gnl|CDD|144027	pfam00287, Na_K-ATPase, Soc	gb|ABV44715.1|	Na/K ATPase-like protein [Phlebotomus papatasi]	4,00E-76
gALL_v3_c16873	2	4	gnl|CDD|163618	cd00842, MPP_ASMase, acid s	ref|XP_003693356.1|	PREDICTED: LOW QUALITY PROTEIN: sphingomyelin phosphodiesterase-like [Apis florea]	8,00E-39
gALL_v3_c16874	2	3	-	-	-	-	-
gALL_v3_c16875	2	3	-	-	-	-	-
gALL_v3_c16876	5	2	gnl|CDD|31005	COG0661, AarF, Predicted unu	ref|XP_001605712.2|	PREDICTED: chaperone activity of bc1 complex-like, mitochondrial-like [Nasonia vitripennis]	1,00E-61
gALL_v3_c16877	3	3	-	-	ref|XP_006612928.1|	PREDICTED: zinc finger protein DPF3-like isoform X1 [Apis dorsata]	4,00E-06
gALL_v3_c16878	3	4	gnl|CDD|187585	cd05324, carb_red_PTCR-like_	ref|XP_003489050.1|	PREDICTED: carbonyl reductase [NADPH] 1-like [Bombus impatiens]	2,00E-42
gALL_v3_c16879	5	8	-	-	ref|XP_003486834.1|	PREDICTED: Golgi apparatus protein 1-like [Bombus impatiens]	5,00E-39
gALL_v3_c1688	8	5	gnl|CDD|128712	smart00435, TOPEUc, DNA Top	ref|XP_003697235.1|	PREDICTED: DNA topoisomerase 1-like [Apis florea]	1,00E-35
gALL_v3_c16880	8	5	-	-	-	-	-
gALL_v3_c16881	3	7	-	-	-	-	-
gALL_v3_c16882	2	3	-	-	-	-	-
gALL_v3_c16883	3	3	-	-	ref|XP_002430186.1|	conserved hypothetical protein [Pediculus humanus corporis] gb|EEB17448.1| conserved hypothetical protein [Pediculus humanus corporis]	2,00E-10
gALL_v3_c16884	3	1	-	-	-	-	-
gALL_v3_c16885	2	4	-	-	-	-	-
gALL_v3_c16886	3	3	-	-	-	-	-
gALL_v3_c16887	3	2	-	-	ref|XP_001647814.1|	sumo ligase [Aedes aegypti] gb|EAT32482.1| AAEL015099-PA [Aedes aegypti]	5,00E-27
gALL_v3_c16888	3	1	-	-	-	-	-
gALL_v3_c16889	4	3	gnl|CDD|29038	cd01093, CRIB_PAK_like, PAK (	ref|XP_002428585.1|	serine/threonine-protein kinase PAK mbt, putative [Pediculus humanus corporis] gb|EEB15847.1| serine/threonine-protein kinase PAK mbt, putative [Pediculus humanus c	2,00E-47
gALL_v3_c1689	5	4	gnl|CDD|148593	pfam07074, TRAP-gamma, Tra	ref|XP_003705748.1|	PREDICTED: translocon-associated protein subunit gamma-like [Megachile rotundata]	4,00E-21
gALL_v3_c16890	2	3	gnl|CDD|128452	smart00147, RasGEF, Guanine	gb|EFX85085.1|	hypothetical protein DAPPUDRAFT_30396 [Daphnia pulex]	2,00E-89
gALL_v3_c16891	6	4	-	-	-	-	-
gALL_v3_c16892	1	4	-	-	-	-	-
gALL_v3_c16893	2	4	-	-	-	-	-
gALL_v3_c16894	8	16	-	-	-	-	-
gALL_v3_c16897	2	2	-	-	-	-	-
gALL_v3_c16898	3	3	gnl|CDD|189715	pfam00780, CNH, CNH domair	gb|EFX65321.1|	hypothetical protein DAPPUDRAFT_219389 [Daphnia pulex]	3,00E-72
gALL_v3_c16899	0	6	-	-	-	-	-
gALL_v3_c169	5	9	-	-	-	-	-
gALL_v3_c1690	22	26	-	-	ref|XP_966893.1|	PREDICTED: similar to Transmembrane protein 98 (Protein TADA1) [Tribolium castaneum] gb|EFA03806.1| hypothetical protein TcasGA2_TC013919 [Tribolium castaneum]	5,00E-49
gALL_v3_c16901	2	4	-	-	ref|XP_793520.2|	PREDICTED: uncharacterized protein LOC588760 [Strongylocentrotus purpuratus]	3,00E-16
gALL_v3_c16902	1	3	-	-	-	-	-
gALL_v3_c16903	2	3	-	-	-	-	-
gALL_v3_c16904	4	4	-	-	ref|XP_001952536.2|	PREDICTED: ubiquitin carboxyl-terminal hydrolase 34-like [Acyrthosiphon pisum]	5,00E-25
gALL_v3_c16905	3	4	-	-	-	-	-
gALL_v3_c16906	4	2	-	-	-	-	-
gALL_v3_c16907	3	6	-	-	gb|AGM32477.1|	serine/arginine-rich splicing factor 7-like protein [Coptotermes formosanus]	3,00E-06
gALL_v3_c16908	1	4	-	-	-	-	-
gALL_v3_c16909	3	4	gnl|CDD|187698	cd09274, RNase_HI_RT_Ty3, T	ref|XP_001807263.1|	PREDICTED: similar to gag-pol polyprotein [Tribolium castaneum]	5,00E-44
gALL_v3_c1691	21	31	-	-	-	-	-
gALL_v3_c16910	4	2	-	-	-	-	-
gALL_v3_c16911	3	3	-	-	ref|XP_001607434.1|	PREDICTED: elongation factor Ts, mitochondrial-like [Nasonia vitripennis]	3,00E-33
gALL_v3_c16912	3	6	gnl|CDD|191417	pfam05986, ADAM_spacer1, A	gb|EFN71679.1|	Papilin [Camponotus floridanus]	1,00E-160
gALL_v3_c16913	2	3	gnl|CDD|73154	cd01647, RT_LTR, RT_LTR: Rev	ref|XP_001810599.1|	PREDICTED: similar to pol polyprotein [Tribolium castaneum]	3,00E-30
gALL_v3_c16914	2	3	-	-	-	-	-
gALL_v3_c16915	3	3	-	-	ref|XP_003694776.1|	PREDICTED: uncharacterized protein LOC100872339 [Apis florea]	1,00E-06
gALL_v3_c16917	0	4	-	-	-	-	-
gALL_v3_c16918	0	5	-	-	-	-	-
gALL_v3_c16919	4	2	-	-	-	-	-
gALL_v3_c1692	18	12	-	-	-	-	-
gALL_v3_c16920	2	3	-	-	-	-	-
gALL_v3_c16921	0	4	-	-	-	-	-
gALL_v3_c16922	1	8	-	-	-	-	-

gALL_v3_c16923	5	2	-	-	-	-	-
gALL_v3_c16924	3	2	-	-	-	-	-
gALL_v3_c16926	5	3	-	-	-	-	-
gALL_v3_c16927	2	2	-	-	-	-	-
gALL_v3_c16928	4	7	-	-	ref|XP_002432982.1|	Cold-inducible RNA-binding protein, putative [Pediculus humanus corporis] gb|EEB20244.1| Cold-inducible RNA-binding protein, putative [Pediculus humanus corporis]	2,00E-10
gALL_v3_c16929	6	6	gnl|CDD|163693	cd08062, MPN_RPN7_8, Mpr1	ref|XP_003494459.1|	PREDICTED: 26S proteasome non-ATPase regulatory subunit 7-like [Bombus impatiens]	1,00E-58
gALL_v3_c1693	12	15	-	-	-	-	-
gALL_v3_c16930	4	2	-	-	gb|EFA13373.1|	hypothetical protein TcasGA2_TC001390 [Tribolium castaneum]	1,00E-24
gALL_v3_c16931	6	5	gnl|CDD|29261	cd00204, ANK, ankyrin repeat	ref|XP_002424807.1|	hect E3 ubiquitin ligase, putative [Pediculus humanus corporis] gb|EEB12069.1| hect E3 ubiquitin ligase, putative [Pediculus humanus corporis]	1,00E-162
gALL_v3_c16932	5	2	-	-	ref|XP_003399356.1|	PREDICTED: WD repeat-containing protein 59-like isoform 2 [Bombus terrestris]	2,00E-57
gALL_v3_c16933	2	2	-	-	-	-	-
gALL_v3_c16935	4	5	-	-	-	-	-
gALL_v3_c16936	3	3	gnl|CDD|117091	pfam08514, STAG, STAG doma	ref|XP_966898.1|	PREDICTED: similar to stromal antigen [Tribolium castaneum]	7,00E-73
gALL_v3_c16937	3	2	-	-	-	-	-
gALL_v3_c16938	1	4	-	-	-	-	-
gALL_v3_c16939	1	6	gnl|CDD|193549	cd05674, M20_yscS, M20 Pep	gb|EKC41457.1|	Putative carboxypeptidase PM20D1 [Crassostrea gigas]	1,00E-17
gALL_v3_c1694	5	10	-	-	-	-	-
gALL_v3_c16941	2	3	-	-	-	-	-
gALL_v3_c16942	1	4	-	-	-	-	-
gALL_v3_c16943	2	2	gnl|CDD|133293	cd01893, Miro1, Miro1 subfan	gb|EFN76557.1|	Mitochondrial Rho GTPase [Harpegnathos saltator]	8,00E-42
gALL_v3_c16944	4	3	-	-	-	-	-
gALL_v3_c16945	1	3	-	-	-	-	-
gALL_v3_c16946	4	4	gnl|CDD|72996	cd03237, ABC_RNaseL_inhibit	ref|XP_003701716.1|	PREDICTED: ATP-binding cassette sub-family E member 1 [Megachile rotundata]	1,00E-149
gALL_v3_c16948	2	2	-	-	-	-	-
gALL_v3_c16949	5	2	-	-	gb|AGM32905.1|	hypothetical protein [Coptotermes formosanus]	3,00E-14
gALL_v3_c1695	11	16	gnl|CDD|34853	COG5256, TEF1, Translation el	ref|XP_968959.1|	PREDICTED: similar to eukaryotic peptide chain release factor GTP-binding subunit [Tribolium castaneum] gb|EFA00945.1| hypothetical protein TcasGA2_TC003854 [Triboli	0
gALL_v3_c16950	7	10	gnl|CDD|48156	cd03339, TCP1_epsilon, TCP-1	gb|AHB33467.1|	T-complex protein subunit epsilon [Locusta migratoria]	1,00E-123
gALL_v3_c16952	2	3	-	-	-	-	-
gALL_v3_c16953	4	2	-	-	-	-	-
gALL_v3_c16954	3	5	-	-	-	-	-
gALL_v3_c16956	2	4	-	-	-	-	-
gALL_v3_c16957	5	4	-	-	-	-	-
gALL_v3_c16958	4	4	-	-	-	-	-
gALL_v3_c16959	3	2	-	-	-	-	-
gALL_v3_c1696	5	10	-	-	-	-	-
gALL_v3_c16960	1	5	-	-	-	-	-
gALL_v3_c16961	4	5	-	-	-	-	-
gALL_v3_c16962	5	2	-	-	-	-	-
gALL_v3_c16964	1	5	-	-	-	-	-
gALL_v3_c16965	4	11	-	-	-	-	-
gALL_v3_c16966	3	3	-	-	-	-	-
gALL_v3_c16967	2	4	-	-	-	-	-
gALL_v3_c16968	3	2	-	-	-	-	-
gALL_v3_c16969	3	12	-	-	gb|EGI67485.1|	Nesprin-1 [Acromyrmex echinatior]	9,00E-67
gALL_v3_c1697	11	9	-	-	ref|XP_001946566.2|	PREDICTED: NFX1-type zinc finger-containing protein 1-like [Acyrthosiphon pisum]	4,00E-61
gALL_v3_c16970	1	4	-	-	-	-	-
gALL_v3_c16971	3	2	-	-	-	-	-
gALL_v3_c16972	1	5	-	-	-	-	-
gALL_v3_c16973	2	3	-	-	-	-	-
gALL_v3_c16974	3	5	-	-	-	-	-
gALL_v3_c16975	6	3	-	-	-	-	-
gALL_v3_c16976	0	3	-	-	-	-	-
gALL_v3_c16978	2	7	-	-	-	-	-
gALL_v3_c1698	17	18	gnl|CDD|112354	pfam03531, SSrecog, Structur	gb|EFN68140.1|	FACT complex subunit Ssrp1 [Camponotus floridanus]	1,00E-168
gALL_v3_c16980	2	4	-	-	ref|XP_969912.2|	PREDICTED: similar to GA22134-PA [Tribolium castaneum] gb|EFA04498.1| hypothetical protein TcasGA2_TC014806 [Tribolium castaneum]	6,00E-22
gALL_v3_c16981	2	4	-	-	-	-	-
gALL_v3_c16982	2	4	-	-	ref|XP_002433174.1|	Negative elongation factor E, putative [Pediculus humanus corporis] gb|EEB20436.1| Negative elongation factor E, putative [Pediculus humanus corporis]	1,00E-58
gALL_v3_c16983	2	6	-	-	-	-	-
gALL_v3_c16984	3	5	-	-	ref|XP_002432425.1|	hypothetical protein Phum_PHUM583410 [Pediculus humanus corporis] gb|EEB19687.1| hypothetical protein Phum_PHUM583410 [Pediculus humanus corporis]	2,00E-07
gALL_v3_c16985	2	6	-	-	-	-	-
gALL_v3_c16986	7	4	gnl|CDD|128564	smart00268, ACTIN, Actin. AC	gb|EFN62441.1|	Actin-related protein 6 [Camponotus floridanus]	1,00E-127
gALL_v3_c16987	9	6	gnl|CDD|176276	cd00934, PTB, Phosphotyrosin	ref|XP_004924999.1|	PREDICTED: low density lipoprotein receptor adapter protein 1-A-like isoform X1 [Bombyx mori] ref|XP_004925000.1| PREDICTED: low density lipoprotein receptor adapte	2,00E-62
gALL_v3_c16989	1	8	-	-	-	-	-
gALL_v3_c1699	3	8	-	-	-	-	-
gALL_v3_c16990	2	4	-	-	-	-	-
gALL_v3_c16991	4	3	-	-	-	-	-
gALL_v3_c16992	4	2	-	-	-	-	-
gALL_v3_c16993	4	3	-	-	ref|XP_006569381.1|	PREDICTED: focal adhesion kinase 1 isoform X2 [Apis mellifera] ref|XP_006569382.1| PREDICTED: focal adhesion kinase 1 isoform X3 [Apis mellifera] ref|XP_006569383.1|	1,00E-20
gALL_v3_c16994	2	4	gnl|CDD|176310	cd01234, PH_CADPS, CADPS (	gb|EFN66893.1|	Calcium-dependent secretion activator [Camponotus floridanus]	1,00E-105
gALL_v3_c16995	1	6	-	-	-	-	-
gALL_v3_c16997	2	5	-	-	-	-	-
gALL_v3_c16998	3	4	-	-	-	-	-
gALL_v3_c16999	3	4	-	-	-	-	-
gALL_v3_c17	18	23	gnl|CDD|176375	cd01780, PLC_epsilon_RA, Ub	gb|AGZ15174.1|	phosphoesterase 19, partial [Laodelphax striatella]	8,00E-83
gALL_v3_c170	4	6	gnl|CDD|28911	cd00029, C1, Protein kinase C	ref|XP_003694662.1|	PREDICTED: diacylglycerol kinase theta-like [Apis florea]	1,00E-107
gALL_v3_c1700	20	17	-	-	-	-	-
gALL_v3_c17000	2	2	-	-	-	-	-
gALL_v3_c17001	4	2	-	-	-	-	-
gALL_v3_c17002	2	3	-	-	-	-	-
gALL_v3_c17003	4	4	-	-	gb|EFN67807.1|	DEP domain-containing protein 5 [Camponotus floridanus]	1,00E-72
gALL_v3_c17004	8	10	-	-	-	-	-
gALL_v3_c17005	4	7	gnl|CDD|190555	pfam03184, DDE_1, DDE supe	ref|XP_002737102.1|	PREDICTED: tigger transposable element-derived protein 6-like [Saccoglossus kowalevskii]	3,00E-43
gALL_v3_c17006	4	4	gnl|CDD|146607	pfam04065, Not3, Not1 N-terr	ref|XP_003690656.1|	PREDICTED: uncharacterized protein LOC100868746 [Apis florea] ref|XP_006610511.1| PREDICTED: CCR4-NOT transcription complex subunit 3-like isoform X2 [Apis dorsat	1,00E-31
gALL_v3_c17007	3	2	gnl|CDD|189372	pfam00067, p450, Cytochrom	gb|EKC33403.1|	Putative cytochrome P450 CYP44 [Crassostrea gigas]	2,00E-71
gALL_v3_c17008	7	5	-	-	ref|XP_002432957.1|	B-cell lymphoma/leukemia, putative [Pediculus humanus corporis] gb|EEB20219.1| B-cell lymphoma/leukemia, putative [Pediculus humanus corporis]	1,00E-48
gALL_v3_c17009	4	9	gnl|CDD|128620	smart00325, RhoGEF, Guanine	ref|XP_006622045.1|	PREDICTED: protein ECT2-like isoform X8 [Apis dorsata]	1,00E-123
gALL_v3_c17010	3	3	-	-	-	-	-
gALL_v3_c17011	2	4	-	-	-	-	-
gALL_v3_c17012	2	3	-	-	-	-	-
gALL_v3_c17013	2	2	-	-	-	-	-
gALL_v3_c17014	3	2	-	-	-	-	-
gALL_v3_c17015	4	2	-	-	ref|XP_314341.1|	AGAP004850-PA [Anopheles gambiae str. PEST] gb|EAA09721.2| AGAP004850-PA [Anopheles gambiae str. PEST]	4,00E-85
gALL_v3_c17016	2	5	gnl|CDD|188710	cd08756, RGS_GEF_like, Regul	ref|XP_002424958.1|	conserved hypothetical protein [Pediculus humanus corporis] gb|EEB12220.1| conserved hypothetical protein [Pediculus humanus corporis]	4,00E-68
gALL_v3_c17018	1	3	-	-	-	-	-
gALL_v3_c17019	3	9	-	-	-	-	-
gALL_v3_c1702	27	42	-	-	-	-	-
gALL_v3_c17020	5	2	gnl|CDD|178174	PLN02560, PLN02560, enoyl-C	ref|XP_001601952.1|	PREDICTED: trans-2,3-enoyl-CoA reductase-like [Nasonia vitripennis]	7,00E-54
gALL_v3_c17021	2	2	-	-	ref|XP_002427221.1|	conserved hypothetical protein [Pediculus humanus corporis] gb|EEB14483.1| conserved hypothetical protein [Pediculus humanus corporis]	1,00E-38
gALL_v3_c17022	1	4	-	-	gb|AGM32924.1|	hypothetical protein [Coptotermes formosanus]	4,00E-17
gALL_v3_c17023	1	4	-	-	-	-	-
gALL_v3_c17024	4	2	-	-	-	-	-
gALL_v3_c17025	2	2	-	-	gb|ADI61823.1|	endonuclease-reverse transcriptase [Bombyx mori]	4,00E-25
gALL_v3_c17026	3	3	-	-	-	-	-
gALL_v3_c17027	3	3	-	-	-	-	-
gALL_v3_c17028	3	2	-	-	-	-	-
gALL_v3_c17029	2	4	gnl|CDD|178675	PLN03129, PLN03129, NADP-c	gb|AGC84405.1|	NADP-dependent malic enzyme-like protein [Locusta migratoria]	2,00E-79
gALL_v3_c1703	6	8	gnl|CDD|161835	TIGR00355, purH, phosphorib	ref|XP_967875.1|	PREDICTED: similar to 5-aminoimidazole-4-carboxamide ribonucleotide formyltransferase/IMP cyclohydrolase [Tribolium castaneum] gb|EEZ98830.1| hypothetical protein	1,00E-172
gALL_v3_c17030	3	8	-	-	ref|XP_003493222.1|	PREDICTED: protein bowel-like [Bombus impatiens]	1,00E-119
gALL_v3_c17031	3	2	-	-	ref|XP_002426346.1|	transformation/transcription domain-associated protein, putative [Pediculus humanus corporis] gb|EEB13608.1| transformation/transcription domain-associated protein,	9,00E-94
gALL_v3_c17032	4	4	-	-	ref|XP_002430696.1|	hypothetical protein Phum_PHUM495260 [Pediculus humanus corporis] gb|EEB17958.1| hypothetical protein Phum_PHUM495260 [Pediculus humanus corporis]	1,00E-31
gALL_v3_c17033	3	6	-	-	-	-	-
gALL_v3_c17034	4	3	-	-	-	-	-
gALL_v3_c17035	2	4	-	-	-	-	-
gALL_v3_c17036	5	4	gnl|CDD|30100	cd01378, MYSc_type_I, Myosi	ref|XP_001662030.1|	myosin i [Aedes aegypti] gb|EAT35966.1| AAEL011905-PA [Aedes aegypti]	3,00E-60
gALL_v3_c17037	2	3	gnl|CDD|33749	COG3968, COG3968, Uncharac	ref|YP_007373859.1|	glutamine synthetase GlnA [Clostridium stercorarium subsp. stercorarium DSM 8532] ref|YP_007680236.1| glutamine synthetase [Clostridium stercorarium subsp. stercora	1,00E-27
gALL_v3_c17038	3	4	-	-	-	-	-
gALL_v3_c17039	3	4	gnl|CDD|147338	pfam05110, AF-4, AF-4 proto-	gb|EHJ63440.1|	chromodomain-helicase-DNA-binding protein 1 [Danaus plexippus]	2,00E-08
gALL_v3_c17040	2	5	-	-	-	-	-
gALL_v3_c17042	7	10	-	-	-	-	-
gALL_v3_c17044	5	9	gnl|CDD|143939	pfam00173, Cyt-b5, Cytochror	gb|EFZ18849.1|	hypothetical protein SINV_80278 [Solenopsis invicta]	5,00E-64
gALL_v3_c17045	4	2	-	-	-	-	-
gALL_v3_c17046	1	6	-	-	gb|ADI61823.1|	endonuclease-reverse transcriptase [Bombyx mori]	8,00E-39
gALL_v3_c17047	2	2	-	-	-	-	-
gALL_v3_c17048	4	5	gnl|CDD|192033	pfam08427, DUF1741, Domair	ref|XP_002422642.1|	conserved hypothetical protein [Pediculus humanus corporis] gb|EEB09904.1| conserved hypothetical protein [Pediculus humanus corporis]	7,00E-65
gALL_v3_c17049	3	2	gnl|CDD|128524	smart00228, PDZ, Domain pre	ref|XP_005515853.1|	PREDICTED: protein lin-7 homolog C-like, partial [Columba livia]	9,00E-29
gALL_v3_c1705	18	20	-	-	-	-	-
gALL_v3_c17051	5	6	-	-	-	-	-
gALL_v3_c17052	2	5	gnl|CDD|30102	cd01380, MYSc_type_V, Myos	gb|ADM15669.1|	myosin Va [Eriocheir sinensis]	1,00E-87
gALL_v3_c17053	3	1	-	-	-	-	-
gALL_v3_c17054	4	3	-	-	-	-	-
gALL_v3_c17055	4	3	-	-	-	-	-
gALL_v3_c17056	4	7	-	-	-	-	-
gALL_v3_c17057	11	12	-	-	-	-	-
gALL_v3_c17058	7	6	-	-	-	-	-
gALL_v3_c17059	5	4	gnl|CDD|146069	pfam03250, Tropomodulin, Tr	ref|XP_006621207.1|	PREDICTED: tropomodulin-like isoform X9 [Apis dorsata] ref|XP_006621208.1| PREDICTED: tropomodulin-like isoform X10 [Apis dorsata]	3,00E-46
gALL_v3_c1706	2	8	gnl|CDD|128625	smart00330, PIPKc, Phosphati	ref|XP_002432206.1|	Phosphatidylinositol-4-phosphate 5-kinase type-2 alpha, putative [Pediculus humanus corporis] gb|EEB19468.1| Phosphatidylinositol-4-phosphate 5-kinase type-2 alpha,	1,00E-152
gALL_v3_c17060	0	4	-	-	-	-	-
gALL_v3_c17061	0	4	-	-	-	-	-
gALL_v3_c17062	3	2	-	-	ref|XP_004926763.1|	PREDICTED: F-box only protein 7-like [Bombyx mori]	3,00E-15
gALL_v3_c17063	1	3	-	-	-	-	-
gALL_v3_c17065	5	4	gnl|CDD|112796	pfam03997, VPS28, VPS28 pro	ref|NP_001233024.1|	uncharacterized protein LOC100169088 [Acyrthosiphon pisum] dbj|BAH72426.1| ACYPI009739 [Acyrthosiphon pisum]	1,00E-130
gALL_v3_c17066	0	5	-	-	ref|XP_312094.5|	AGAP002818-PA [Anopheles gambiae str. PEST] gb|EAA07853.5| AGAP002818-PA [Anopheles gambiae str. PEST]	1,00E-74
gALL_v3_c17067	2	1	gnl|CDD|185566	PTZ00340, PTZ00340, O-sialog	ref|XP_308804.2|	AGAP006952-PA [Anopheles gambiae str. PEST] gb|EAA04730.2| AGAP006952-PA [Anopheles gambiae str. PEST]	2,00E-47
gALL_v3_c17068	4	6	-	-	-	-	-

<table>
<tr><td>gALL_v3_c1707</td><td>24</td><td>39</td><td>-</td><td>-</td><td>ref|XP_001942791.2|</td><td>PREDICTED: hypothetical protein LOC100169373 [Acyrthosiphon pisum]</td><td>9,00E-30</td></tr>
<tr><td>gALL_v3_c17070</td><td>4</td><td>2</td><td>-</td><td>-</td><td>-</td><td>-</td><td>-</td></tr>
<tr><td>gALL_v3_c17072</td><td>11</td><td>7</td><td>gnl|CDD|128508</td><td>smart00212, UBCc, Ubiquitin-c</td><td>ref|NP_001037390.1|</td><td>ubiquitin-conjugating enzyme E2L [Bombyx mori] gb|ABB36655.1| ubiquitin-conjugating enzyme E2I [Bombyx mori] gb|ABF51360.1| ubiquitin-conjugating enzyme [Bomb</td><td>8,00E-94</td></tr>
<tr><td>gALL_v3_c17075</td><td>1</td><td>4</td><td>-</td><td>-</td><td>-</td><td>-</td><td>-</td></tr>
<tr><td>gALL_v3_c17076</td><td>4</td><td>4</td><td>gnl|CDD|128500</td><td>smart00204, TGFB, Transformi</td><td>emb|CCJ37791.1|</td><td>decapentaplegic, partial [Blattella germanica]</td><td>4,00E-17</td></tr>
<tr><td>gALL_v3_c17077</td><td>4</td><td>7</td><td>-</td><td>-</td><td>-</td><td>-</td><td>-</td></tr>
<tr><td>gALL_v3_c17078</td><td>6</td><td>16</td><td>-</td><td>-</td><td>-</td><td>-</td><td>-</td></tr>
<tr><td>gALL_v3_c17079</td><td>0</td><td>5</td><td>gnl|CDD|128625</td><td>smart00330, PIPKc, Phosphati</td><td>ref|XP_003393480.1|</td><td>PREDICTED: phosphatidylinositol-4-phosphate 5-kinase type-1 alpha-like isoform 2 [Bombus terrestris]</td><td>2,00E-38</td></tr>
<tr><td>gALL_v3_c17080</td><td>4</td><td>3</td><td>gnl|CDD|128498</td><td>smart00202, SR, Scavenger re</td><td>gb|EFN78073.1|</td><td>Lysyl oxidase-like protein 4 [Harpegnathos saltator]</td><td>7,00E-85</td></tr>
<tr><td>gALL_v3_c17081</td><td>3</td><td>3</td><td>-</td><td>-</td><td>ref|XP_002426224.1|</td><td>sugar transporter, putative [Pediculus humanus corporis] gb|EEB13486.1| sugar transporter, putative [Pediculus humanus corporis]</td><td>5,00E-31</td></tr>
<tr><td>gALL_v3_c17082</td><td>2</td><td>2</td><td>-</td><td>-</td><td>gb|ADD24484.1|</td><td>Transposable element Tc3 transposase [Lepeophtheirus salmonis]</td><td>9,00E-27</td></tr>
<tr><td>gALL_v3_c17083</td><td>4</td><td>4</td><td>-</td><td>-</td><td>-</td><td>-</td><td>-</td></tr>
<tr><td>gALL_v3_c17085</td><td>3</td><td>6</td><td>-</td><td>-</td><td>-</td><td>-</td><td>-</td></tr>
<tr><td>gALL_v3_c17086</td><td>3</td><td>7</td><td>-</td><td>-</td><td>-</td><td>-</td><td>-</td></tr>
<tr><td>gALL_v3_c17087</td><td>2</td><td>4</td><td>-</td><td>-</td><td>-</td><td>-</td><td>-</td></tr>
<tr><td>gALL_v3_c17088</td><td>1</td><td>5</td><td>-</td><td>-</td><td>-</td><td>-</td><td>-</td></tr>
<tr><td>gALL_v3_c17089</td><td>2</td><td>6</td><td>-</td><td>-</td><td>-</td><td>-</td><td>-</td></tr>
<tr><td>gALL_v3_c1709</td><td>5</td><td>7</td><td>-</td><td>-</td><td>-</td><td>-</td><td>-</td></tr>
<tr><td>gALL_v3_c17090</td><td>5</td><td>4</td><td>-</td><td>-</td><td>-</td><td>-</td><td>-</td></tr>
<tr><td>gALL_v3_c17091</td><td>0</td><td>4</td><td>gnl|CDD|31829</td><td>COG1643, HrpA, HrpA-like hel</td><td>ref|XP_001948871.2|</td><td>PREDICTED: ATP-dependent RNA helicase DHX8-like [Acyrthosiphon pisum]</td><td>3,00E-49</td></tr>
<tr><td>gALL_v3_c17093</td><td>3</td><td>7</td><td>gnl|CDD|29257</td><td>cd00200, WD40, WD40 domai</td><td>gb|ETN61780.1|</td><td>beach protein [Anopheles darlingi]</td><td>1,00E-111</td></tr>
<tr><td>gALL_v3_c17094</td><td>5</td><td>3</td><td>-</td><td>-</td><td>-</td><td>-</td><td>-</td></tr>
<tr><td>gALL_v3_c17095</td><td>2</td><td>1</td><td>-</td><td>-</td><td>-</td><td>-</td><td>-</td></tr>
<tr><td>gALL_v3_c17096</td><td>2</td><td>4</td><td>-</td><td>-</td><td>-</td><td>-</td><td>-</td></tr>
<tr><td>gALL_v3_c17097</td><td>3</td><td>1</td><td>-</td><td>-</td><td>ref|WP_007302981.1|</td><td>Ankyrin repeat domain protein [Wolbachia endosymbiont of Culex quinquefasciatus] gb|EEB55313.1| Ankyrin repeat domain protein [Wolbachia endosymbiont of Culex q</td><td>6,00E-12</td></tr>
<tr><td>gALL_v3_c17098</td><td>3</td><td>3</td><td>-</td><td>-</td><td>gb|EFA09971.1|</td><td>hypothetical protein TcasGA2_TC012129 [Tribolium castaneum]</td><td>5,00E-21</td></tr>
<tr><td>gALL_v3_c17099</td><td>3</td><td>8</td><td>-</td><td>-</td><td>ref|XP_001601754.2|</td><td>PREDICTED: LOW QUALITY PROTEIN: baculoviral IAP repeat-containing protein 6 [Nasonia vitripennis]</td><td>6,00E-70</td></tr>
<tr><td>gALL_v3_c171</td><td>6</td><td>11</td><td>-</td><td>-</td><td>gb|EFN75188.1|</td><td>Protein lava lamp [Harpegnathos saltator]</td><td>4,00E-23</td></tr>
<tr><td>gALL_v3_c1710</td><td>29</td><td>18</td><td>gnl|CDD|30367</td><td>COG0017, AsnS, Aspartyl/aspa</td><td>ref|XP_003979844.1|</td><td>PREDICTED: asparagine--tRNA ligase, cytoplasmic-like [Takifugu rubripes]</td><td>2,00E-19</td></tr>
<tr><td>gALL_v3_c17100</td><td>3</td><td>1</td><td>-</td><td>-</td><td>ref|XP_001810762.1|</td><td>PREDICTED: similar to CG33978 CG33978-PA [Tribolium castaneum]</td><td>5,00E-24</td></tr>
<tr><td>gALL_v3_c17102</td><td>2</td><td>3</td><td>gnl|CDD|162465</td><td>TIGR01652, ATPase-Plipid, phc</td><td>gb|EFN81070.1|</td><td>Probable phospholipid-transporting ATPase IIB [Harpegnathos saltator]</td><td>2,00E-64</td></tr>
<tr><td>gALL_v3_c17103</td><td>3</td><td>4</td><td>-</td><td>-</td><td>dbj|BAD84176.1|</td><td>cytochrome P450 [Hodotermopsis sjostedti]</td><td>3,00E-10</td></tr>
<tr><td>gALL_v3_c17105</td><td>1</td><td>4</td><td>-</td><td>-</td><td>-</td><td>-</td><td>-</td></tr>
<tr><td>gALL_v3_c17106</td><td>3</td><td>5</td><td>-</td><td>-</td><td>gb|AGM32427.1|</td><td>soldier-caste specific protein [Coptotermes formosanus]</td><td>2,00E-93</td></tr>
<tr><td>gALL_v3_c17107</td><td>2</td><td>5</td><td>-</td><td>-</td><td>ref|XP_002425918.1|</td><td>conserved hypothetical protein [Pediculus humanus corporis] gb|EEB13180.1| conserved hypothetical protein [Pediculus humanus corporis]</td><td>4,00E-95</td></tr>
<tr><td>gALL_v3_c17108</td><td>4</td><td>4</td><td>gnl|CDD|132943</td><td>cd06612, STKc_MST1_2, Catal</td><td>ref|XP_001604121.1|</td><td>PREDICTED: serine/threonine-protein kinase hippo-like isoform 1 [Nasonia vitripennis]</td><td>0</td></tr>
<tr><td>gALL_v3_c17109</td><td>7</td><td>5</td><td>-</td><td>-</td><td>-</td><td>-</td><td>-</td></tr>
<tr><td>gALL_v3_c1711</td><td>4</td><td>4</td><td>-</td><td>-</td><td>-</td><td>-</td><td>-</td></tr>
<tr><td>gALL_v3_c17110</td><td>1</td><td>3</td><td>-</td><td>-</td><td>-</td><td>-</td><td>-</td></tr>
<tr><td>gALL_v3_c17111</td><td>4</td><td>2</td><td>-</td><td>-</td><td>ref|XP_002432287.1|</td><td>conserved hypothetical protein [Pediculus humanus corporis] gb|EEB19549.1| conserved hypothetical protein [Pediculus humanus corporis]</td><td>3,00E-50</td></tr>
<tr><td>gALL_v3_c17112</td><td>0</td><td>5</td><td>-</td><td>-</td><td>-</td><td>-</td><td>-</td></tr>
<tr><td>gALL_v3_c17113</td><td>3</td><td>7</td><td>-</td><td>-</td><td>-</td><td>-</td><td>-</td></tr>
<tr><td>gALL_v3_c17115</td><td>11</td><td>11</td><td>gnl|CDD|177670</td><td>PLN00036, PLN00036, 40S ribo</td><td>ref|XP_002432788.1|</td><td>40S ribosomal protein S4, putative [Pediculus humanus corporis] gb|EEB20050.1| 40S ribosomal protein S4, putative [Pediculus humanus corporis]</td><td>5,00E-76</td></tr>
<tr><td>gALL_v3_c17116</td><td>1</td><td>4</td><td>-</td><td>-</td><td>gb|EFA05512.1|</td><td>hypothetical protein TcasGA2_TC015699 [Tribolium castaneum]</td><td>2,00E-73</td></tr>
<tr><td>gALL_v3_c17117</td><td>2</td><td>4</td><td>-</td><td>-</td><td>-</td><td>-</td><td>-</td></tr>
<tr><td>gALL_v3_c17118</td><td>7</td><td>6</td><td>gnl|CDD|48477</td><td>cd00713, GltS, Glutamine ami</td><td>ref|XP_002428593.1|</td><td>ferredoxin-dependent glutamate synthase 2, putative [Pediculus humanus corporis] gb|EEB15855.1| ferredoxin-dependent glutamate synthase 2, putative [Pediculus hum</td><td>1,00E-160</td></tr>
<tr><td>gALL_v3_c1712</td><td>17</td><td>21</td><td>-</td><td>-</td><td>-</td><td>-</td><td>-</td></tr>
<tr><td>gALL_v3_c17120</td><td>2</td><td>3</td><td>gnl|CDD|132723</td><td>cd02736, RNAP_III_Rpc1_C, La</td><td>ref|XP_003486538.1|</td><td>PREDICTED: DNA-directed RNA polymerase III subunit RPC1-like [Bombus impatiens]</td><td>6,00E-51</td></tr>
<tr><td>gALL_v3_c17122</td><td>3</td><td>2</td><td>-</td><td>-</td><td>gb|EFN82981.1|</td><td>F-box/WD repeat-containing protein 5 [Harpegnathos saltator]</td><td>1,00E-111</td></tr>
<tr><td>gALL_v3_c17123</td><td>3</td><td>6</td><td>-</td><td>-</td><td>ref|XP_002432406.1|</td><td>gamma-soluble NSF attachment protein, putative [Pediculus humanus corporis] gb|EEB19668.1| gamma-soluble NSF attachment protein, putative [Pediculus humanus cor</td><td>1,00E-107</td></tr>
<tr><td>gALL_v3_c17126</td><td>0</td><td>4</td><td>-</td><td>-</td><td>-</td><td>-</td><td>-</td></tr>
<tr><td>gALL_v3_c17127</td><td>1</td><td>4</td><td>-</td><td>-</td><td>-</td><td>-</td><td>-</td></tr>
<tr><td>gALL_v3_c17128</td><td>2</td><td>3</td><td>-</td><td>-</td><td>ref|XP_001814959.1|</td><td>PREDICTED: similar to AGAP000037-PA [Tribolium castaneum]</td><td>1,00E-16</td></tr>
<tr><td>gALL_v3_c17129</td><td>6</td><td>18</td><td>gnl|CDD|144533</td><td>pfam00975, Thioesterase, Thi</td><td>dbj|BAM19658.1|</td><td>fatty acid synthase [Papilio xuthus]</td><td>6,00E-58</td></tr>
<tr><td>gALL_v3_c1713</td><td>5</td><td>4</td><td>-</td><td>-</td><td>-</td><td>-</td><td>-</td></tr>
<tr><td>gALL_v3_c17130</td><td>6</td><td>12</td><td>-</td><td>-</td><td>gb|AGM32724.1|</td><td>cartilage oligomeric matrix protein (COMP), partial [Coptotermes formosanus]</td><td>9,00E-93</td></tr>
<tr><td>gALL_v3_c17131</td><td>9</td><td>18</td><td>-</td><td>-</td><td>-</td><td>-</td><td>-</td></tr>
<tr><td>gALL_v3_c17132</td><td>3</td><td>3</td><td>-</td><td>-</td><td>-</td><td>-</td><td>-</td></tr>
<tr><td>gALL_v3_c17133</td><td>1</td><td>4</td><td>-</td><td>-</td><td>-</td><td>-</td><td>-</td></tr>
<tr><td>gALL_v3_c17134</td><td>4</td><td>2</td><td>-</td><td>-</td><td>-</td><td>-</td><td>-</td></tr>
<tr><td>gALL_v3_c17135</td><td>6</td><td>4</td><td>-</td><td>-</td><td>-</td><td>-</td><td>-</td></tr>
<tr><td>gALL_v3_c17136</td><td>2</td><td>3</td><td>-</td><td>-</td><td>-</td><td>-</td><td>-</td></tr>
<tr><td>gALL_v3_c17137</td><td>1</td><td>5</td><td>-</td><td>-</td><td>-</td><td>-</td><td>-</td></tr>
<tr><td>gALL_v3_c17138</td><td>5</td><td>11</td><td>-</td><td>-</td><td>-</td><td>-</td><td>-</td></tr>
<tr><td>gALL_v3_c17139</td><td>3</td><td>2</td><td>-</td><td>-</td><td>gb|EFX77167.1|</td><td>hypothetical protein DAPPUDRAFT_225784 [Daphnia pulex]</td><td>3,00E-17</td></tr>
<tr><td>gALL_v3_c1714</td><td>6</td><td>8</td><td>-</td><td>-</td><td>-</td><td>-</td><td>-</td></tr>
<tr><td>gALL_v3_c17140</td><td>2</td><td>3</td><td>-</td><td>-</td><td>-</td><td>-</td><td>-</td></tr>
<tr><td>gALL_v3_c17141</td><td>2</td><td>10</td><td>-</td><td>-</td><td>-</td><td>-</td><td>-</td></tr>
<tr><td>gALL_v3_c17144</td><td>1</td><td>4</td><td>gnl|CDD|189334</td><td>pfam00001, 7tm_1, 7 transme</td><td>dbj|BAO01065.1|</td><td>neuropeptide GPCR A15 [Nilaparvata lugens]</td><td>1,00E-32</td></tr>
<tr><td>gALL_v3_c17145</td><td>9</td><td>7</td><td>-</td><td>-</td><td>gb|AGM32962.1|</td><td>hypothetical protein [Coptotermes formosanus]</td><td>2,00E-23</td></tr>
<tr><td>gALL_v3_c17146</td><td>3</td><td>7</td><td>-</td><td>-</td><td>-</td><td>-</td><td>-</td></tr>
<tr><td>gALL_v3_c17147</td><td>3</td><td>2</td><td>-</td><td>-</td><td>-</td><td>-</td><td>-</td></tr>
<tr><td>gALL_v3_c17150</td><td>3</td><td>2</td><td>-</td><td>-</td><td>-</td><td>-</td><td>-</td></tr>
<tr><td>gALL_v3_c17151</td><td>3</td><td>4</td><td>-</td><td>-</td><td>-</td><td>-</td><td>-</td></tr>
<tr><td>gALL_v3_c17152</td><td>1</td><td>6</td><td>-</td><td>-</td><td>-</td><td>-</td><td>-</td></tr>
<tr><td>gALL_v3_c17153</td><td>7</td><td>8</td><td>-</td><td>-</td><td>-</td><td>-</td><td>-</td></tr>
<tr><td>gALL_v3_c17154</td><td>3</td><td>2</td><td>-</td><td>-</td><td>ref|XP_001304167.1|</td><td>hypothetical protein [Trichomonas vaginalis G3] gb|EAX91237.1| hypothetical protein TVAG_210620 [Trichomonas vaginalis G3]</td><td>2,00E-32</td></tr>
<tr><td>gALL_v3_c17155</td><td>2</td><td>4</td><td>-</td><td>-</td><td>-</td><td>-</td><td>-</td></tr>
<tr><td>gALL_v3_c17156</td><td>2</td><td>1</td><td>gnl|CDD|187546</td><td>cd05235, SDR_e1, extended (e</td><td>ref|XP_002608488.1|</td><td>hypothetical protein BRAFLDRAFT_128033 [Branchiostoma floridae] gb|EEN64498.1| hypothetical protein BRAFLDRAFT_128033 [Branchiostoma floridae]</td><td>1,00E-30</td></tr>
<tr><td>gALL_v3_c17157</td><td>1</td><td>4</td><td>-</td><td>-</td><td>-</td><td>-</td><td>-</td></tr>
<tr><td>gALL_v3_c17158</td><td>0</td><td>3</td><td>gnl|CDD|176348</td><td>cd01272, FE65_N, Fe65 Phosp</td><td>ref|XP_001603939.2|</td><td>PREDICTED: amyloid beta A4 precursor protein-binding family B member 2-like [Nasonia vitripennis]</td><td>8,00E-55</td></tr>
<tr><td>gALL_v3_c17159</td><td>0</td><td>4</td><td>-</td><td>-</td><td>-</td><td>-</td><td>-</td></tr>
<tr><td>gALL_v3_c1716</td><td>9</td><td>7</td><td>-</td><td>-</td><td>gb|ENN79103.1|</td><td>hypothetical protein YQE_04441, partial [Dendroctonus ponderosae]</td><td>1,00E-50</td></tr>
<tr><td>gALL_v3_c17160</td><td>1</td><td>5</td><td>-</td><td>-</td><td>-</td><td>-</td><td>-</td></tr>
<tr><td>gALL_v3_c17161</td><td>1</td><td>6</td><td>-</td><td>-</td><td>-</td><td>-</td><td>-</td></tr>
<tr><td>gALL_v3_c17162</td><td>3</td><td>2</td><td>-</td><td>-</td><td>ref|XP_003490150.1|</td><td>PREDICTED: disks large homolog 5-like isoform 2 [Bombus impatiens]</td><td>8,00E-61</td></tr>
<tr><td>gALL_v3_c17163</td><td>2</td><td>3</td><td>-</td><td>-</td><td>-</td><td>-</td><td>-</td></tr>
<tr><td>gALL_v3_c17164</td><td>3</td><td>3</td><td>-</td><td>-</td><td>-</td><td>-</td><td>-</td></tr>
<tr><td>gALL_v3_c17165</td><td>0</td><td>4</td><td>-</td><td>-</td><td>-</td><td>-</td><td>-</td></tr>
<tr><td>gALL_v3_c17166</td><td>1</td><td>6</td><td>-</td><td>-</td><td>-</td><td>-</td><td>-</td></tr>
<tr><td>gALL_v3_c17168</td><td>2</td><td>2</td><td>-</td><td>-</td><td>-</td><td>-</td><td>-</td></tr>
<tr><td>gALL_v3_c17169</td><td>6</td><td>13</td><td>gnl|CDD|32449</td><td>COG2268, COG2268, Uncharac</td><td>ref|XP_002429709.1|</td><td>Flotillin-2, putative [Pediculus humanus corporis] gb|EEB16971.1| Flotillin-2, putative [Pediculus humanus corporis]</td><td>1,00E-89</td></tr>
<tr><td>gALL_v3_c17171</td><td>5</td><td>5</td><td>-</td><td>-</td><td>-</td><td>-</td><td>-</td></tr>
<tr><td>gALL_v3_c17173</td><td>4</td><td>8</td><td>-</td><td>-</td><td>-</td><td>-</td><td>-</td></tr>
<tr><td>gALL_v3_c17174</td><td>5</td><td>3</td><td>-</td><td>-</td><td>-</td><td>-</td><td>-</td></tr>
<tr><td>gALL_v3_c17175</td><td>1</td><td>5</td><td>-</td><td>-</td><td>ref|XP_002409900.1|</td><td>Grb2 associated binder (Gab), putative [Ixodes scapularis] gb|EEC10963.1| Grb2 associated binder (Gab), putative [Ixodes scapularis]</td><td>4,00E-12</td></tr>
<tr><td>gALL_v3_c17177</td><td>26</td><td>20</td><td>-</td><td>-</td><td>ref|WP_005679585.1|</td><td>hypothetical protein [Bacteroides caccae] gb|EDM19152.1| hypothetical protein BACCAC_03786 [Bacteroides caccae ATCC 43185]</td><td>4,00E-75</td></tr>
<tr><td>gALL_v3_c17178</td><td>3</td><td>1</td><td>-</td><td>-</td><td>-</td><td>-</td><td>-</td></tr>
<tr><td>gALL_v3_c17179</td><td>1</td><td>4</td><td>-</td><td>-</td><td>-</td><td>-</td><td>-</td></tr>
<tr><td>gALL_v3_c1718</td><td>17</td><td>12</td><td>-</td><td>-</td><td>gb|EGI70346.1|</td><td>UHRF1-binding protein 1-like protein [Acromyrmex echinatior]</td><td>0</td></tr>
<tr><td>gALL_v3_c17180</td><td>2</td><td>10</td><td>-</td><td>-</td><td>-</td><td>-</td><td>-</td></tr>
<tr><td>gALL_v3_c17182</td><td>2</td><td>3</td><td>-</td><td>-</td><td>-</td><td>-</td><td>-</td></tr>
<tr><td>gALL_v3_c17183</td><td>2</td><td>1</td><td>-</td><td>-</td><td>-</td><td>-</td><td>-</td></tr>
<tr><td>gALL_v3_c17184</td><td>2</td><td>2</td><td>-</td><td>-</td><td>ref|XP_006625601.1|</td><td>PREDICTED: nudC domain-containing protein 3-like [Lepisosteus oculatus]</td><td>6,00E-13</td></tr>
<tr><td>gALL_v3_c17185</td><td>2</td><td>9</td><td>-</td><td>-</td><td>-</td><td>-</td><td>-</td></tr>
<tr><td>gALL_v3_c17186</td><td>3</td><td>1</td><td>-</td><td>-</td><td>-</td><td>-</td><td>-</td></tr>
<tr><td>gALL_v3_c17187</td><td>9</td><td>10</td><td>-</td><td>-</td><td>dbj|BAF82020.1|</td><td>piggyBac transposase Uribo1 [Xenopus laevis]</td><td>2,00E-58</td></tr>
<tr><td>gALL_v3_c17188</td><td>3</td><td>3</td><td>-</td><td>-</td><td>ref|XP_002434663.1|</td><td>polybromo-1, putative [Ixodes scapularis] gb|EEC07487.1| polybromo-1, putative [Ixodes scapularis]</td><td>5,00E-10</td></tr>
<tr><td>gALL_v3_c17189</td><td>3</td><td>6</td><td>-</td><td>-</td><td>gb|EFN67634.1|</td><td>Putative uncharacterized transposon-derived protein F52C9.6 [Camponotus floridanus]</td><td>7,00E-26</td></tr>
<tr><td>gALL_v3_c1719</td><td>5</td><td>7</td><td>-</td><td>-</td><td>-</td><td>-</td><td>-</td></tr>
<tr><td>gALL_v3_c17191</td><td>2</td><td>1</td><td>-</td><td>-</td><td>-</td><td>-</td><td>-</td></tr>
<tr><td>gALL_v3_c17192</td><td>3</td><td>4</td><td>-</td><td>-</td><td>gb|AAB92389.1|</td><td>non-LTR retrotransposon reverse transcriptase [Drosophila subobscura]</td><td>9,00E-28</td></tr>
<tr><td>gALL_v3_c17193</td><td>3</td><td>3</td><td>-</td><td>-</td><td>-</td><td>-</td><td>-</td></tr>
<tr><td>gALL_v3_c17194</td><td>3</td><td>3</td><td>-</td><td>-</td><td>-</td><td>-</td><td>-</td></tr>
<tr><td>gALL_v3_c17195</td><td>1</td><td>4</td><td>-</td><td>-</td><td>-</td><td>-</td><td>-</td></tr>
<tr><td>gALL_v3_c17196</td><td>3</td><td>3</td><td>-</td><td>-</td><td>-</td><td>-</td><td>-</td></tr>
<tr><td>gALL_v3_c17197</td><td>3</td><td>8</td><td>gnl|CDD|31829</td><td>COG1643, HrpA, HrpA-like hel</td><td>ref|XP_006620317.1|</td><td>PREDICTED: putative pre-mRNA-splicing factor ATP-dependent RNA helicase DHX15-like [Apis dorsata]</td><td>7,00E-63</td></tr>
<tr><td>gALL_v3_c17198</td><td>1</td><td>6</td><td>-</td><td>-</td><td>gb|ELU16454.1|</td><td>hypothetical protein CAPTEDRAFT_60550, partial [Capitella teleta]</td><td>2,00E-06</td></tr>
<tr><td>gALL_v3_c172</td><td>7</td><td>19</td><td>-</td><td>-</td><td>gb|EFN67634.1|</td><td>Putative uncharacterized transposon-derived protein F52C9.6 [Camponotus floridanus]</td><td>2,00E-12</td></tr>
<tr><td>gALL_v3_c1720</td><td>2</td><td>7</td><td>-</td><td>-</td><td>-</td><td>-</td><td>-</td></tr>
<tr><td>gALL_v3_c17201</td><td>3</td><td>1</td><td>-</td><td>-</td><td>-</td><td>-</td><td>-</td></tr>
<tr><td>gALL_v3_c17202</td><td>2</td><td>6</td><td>-</td><td>-</td><td>-</td><td>-</td><td>-</td></tr>
<tr><td>gALL_v3_c17203</td><td>4</td><td>9</td><td>-</td><td>-</td><td>-</td><td>-</td><td>-</td></tr>
<tr><td>gALL_v3_c17204</td><td>2</td><td>7</td><td>-</td><td>-</td><td>-</td><td>-</td><td>-</td></tr>
<tr><td>gALL_v3_c17205</td><td>5</td><td>8</td><td>gnl|CDD|188726</td><td>cd09340, LIM1_Testin_like, Th</td><td>-</td><td>-</td><td>-</td></tr>
<tr><td>gALL_v3_c17206</td><td>2</td><td>10</td><td>gnl|CDD|34909</td><td>COG5329, COG5329, Phospho</td><td>gb|EGI70605.1|</td><td>Polyphosphoinositide phosphatase [Acromyrmex echinatior]</td><td>0</td></tr>
<tr><td>gALL_v3_c17207</td><td>0</td><td>4</td><td>gnl|CDD|181548</td><td>PRK08762, PRK08762, molybd</td><td>ref|XP_001649510.1|</td><td>molybdopterin biosynthesis moeb protein [Aedes aegypti] sp|Q17CA7.1|MOCS3_AEDAE RecName: Full=Adenylyltransferase and sulfurtransferase MOCS3; AltName: Full=</td><td>3,00E-33</td></tr>
<tr><td>gALL_v3_c17208</td><td>8</td><td>5</td><td>gnl|CDD|73156</td><td>cd01650, RT_nLTR_like, RT_nL</td><td>gb|AAB92394.1|</td><td>putative reverse transcriptase [Drosophila subobscura]</td><td>6,00E-58</td></tr>
<tr><td>gALL_v3_c17209</td><td>3</td><td>1</td><td>-</td><td>-</td><td>gb|EHJ76015.1|</td><td>hypothetical protein KGM_15708 [Danaus plexippus]</td><td>1,00E-09</td></tr>
<tr><td>gALL_v3_c1721</td><td>8</td><td>11</td><td>gnl|CDD|190487</td><td>pfam02953, zf-Tim10_DDP, Tir</td><td>gb|AGM32517.1|</td><td>Tim10/DDP family zinc finger protein [Coptotermes formosanus]</td><td>9,00E-54</td></tr>
<tr><td>gALL_v3_c17211</td><td>5</td><td>10</td><td>gnl|CDD|178304</td><td>PLN02701, PLN02701, alpha-n</td><td>ref|XP_002422964.1|</td><td>alpha-mannosidase, putative [Pediculus humanus corporis] gb|EEB10226.1| alpha-mannosidase, putative [Pediculus humanus corporis]</td><td>1,00E-135</td></tr>
<tr><td>gALL_v3_c17212</td><td>2</td><td>5</td><td>-</td><td>-</td><td>-</td><td>-</td><td>-</td></tr>
<tr><td>gALL_v3_c17213</td><td>4</td><td>4</td><td>-</td><td>-</td><td>-</td><td>-</td><td>-</td></tr>
<tr><td>gALL_v3_c17214</td><td>5</td><td>3</td><td>-</td><td>-</td><td>-</td><td>-</td><td>-</td></tr>
<tr><td>gALL_v3_c17215</td><td>1</td><td>3</td><td>-</td><td>-</td><td>-</td><td>-</td><td>-</td></tr>
<tr><td>gALL_v3_c17216</td><td>1</td><td>4</td><td>-</td><td>-</td><td>-</td><td>-</td><td>-</td></tr>
<tr><td>gALL_v3_c17217</td><td>1</td><td>7</td><td>-</td><td>-</td><td>-</td><td>-</td><td>-</td></tr>
<tr><td>gALL_v3_c17218</td><td>16</td><td>18</td><td>gnl|CDD|151474</td><td>pfam11027, DUF2615, Protein</td><td>gb|AGM32502.1|</td><td>hypothetical protein [Coptotermes formosanus]</td><td>3,00E-36</td></tr>
<tr><td>gALL_v3_c17219</td><td>2</td><td>2</td><td>-</td><td>-</td><td>ref|XP_002432334.1|</td><td>hypothetical protein Phum_PHUM580850 [Pediculus humanus corporis] gb|EEB19596.1| hypothetical protein Phum_PHUM580850 [Pediculus humanus corporis]</td><td>8,00E-40</td></tr>
<tr><td>gALL_v3_c17220</td><td>7</td><td>4</td><td>-</td><td>-</td><td>-</td><td>-</td><td>-</td></tr>
<tr><td>gALL_v3_c17221</td><td>0</td><td>4</td><td>-</td><td>-</td><td>-</td><td>-</td><td>-</td></tr>
<tr><td>gALL_v3_c17224</td><td>3</td><td>1</td><td>-</td><td>-</td><td>-</td><td>-</td><td>-</td></tr>
<tr><td>gALL_v3_c17225</td><td>7</td><td>10</td><td>-</td><td>-</td><td>-</td><td>-</td><td>-</td></tr>
<tr><td>gALL_v3_c17226</td><td>4</td><td>5</td><td>gnl|CDD|31361</td><td>COG1167, ARO8, Transcriptior</td><td>ref|XP_003708040.1|</td><td>PREDICTED: 2-aminoadipate transaminase-like [Megachile rotundata]</td><td>3,00E-70</td></tr>
</table>

<table>
<tr><td></td><td></td><td></td><td></td><td></td><td></td><td></td><td></td></tr>
<tr><td>gALL_v3_c17227</td><td>1</td><td>4</td><td>-</td><td>-</td><td>-</td><td>-</td><td>-</td></tr>
<tr><td>gALL_v3_c17228</td><td>1</td><td>5</td><td>-</td><td>-</td><td>ref|XP_975026.1|</td><td>PREDICTED: similar to vacuolar ATP synthase 21 kDa proteolipid subunit [Tribolium castaneum] gb|EEZ99208.1| VhaPPA1-1 [Tribolium castaneum]</td><td>2,00E-43</td></tr>
<tr><td>gALL_v3_c17230</td><td>2</td><td>2</td><td>gnl|CDD|31389</td><td>COG1196, Smc, Chromosome</td><td>ref|XP_001312904.1|</td><td>viral A-type inclusion protein [Trichomonas vaginalis G3] gb|EAX99974.1| viral A-type inclusion protein, putative [Trichomonas vaginalis G3]</td><td>2,00E-07</td></tr>
<tr><td>gALL_v3_c17231</td><td>3</td><td>2</td><td>-</td><td>-</td><td>-</td><td>-</td><td>-</td></tr>
<tr><td>gALL_v3_c17232</td><td>7</td><td>4</td><td>gnl|CDD|189372</td><td>pfam00067, p450, Cytochrom</td><td>gb|AGM32645.1|</td><td>cytochrome P450, partial [Coptotermes formosanus]</td><td>6,00E-96</td></tr>
<tr><td>gALL_v3_c17234</td><td>4</td><td>7</td><td>gnl|CDD|150780</td><td>pfam10146, zf-C4H2, Zinc fing</td><td>gb|EFN87270.1|</td><td>Hepatocellular carcinoma-associated antigen 127-like protein [Harpegnathos saltator]</td><td>2,00E-78</td></tr>
<tr><td>gALL_v3_c17235</td><td>5</td><td>4</td><td>gnl|CDD|190837</td><td>pfam04042, DNA_pol_E_B, DN</td><td>gb|EFN60471.1|</td><td>DNA polymerase subunit alpha B [Camponotus floridanus]</td><td>9,00E-63</td></tr>
<tr><td>gALL_v3_c17237</td><td>1</td><td>4</td><td>-</td><td>-</td><td>-</td><td>-</td><td>-</td></tr>
<tr><td>gALL_v3_c17238</td><td>2</td><td>4</td><td>-</td><td>-</td><td>-</td><td>-</td><td>-</td></tr>
<tr><td>gALL_v3_c17239</td><td>1</td><td>4</td><td>-</td><td>-</td><td>gb|EHJ72713.1|</td><td>putative Lysosomal trafficking regulator [Danaus plexippus]</td><td>2,00E-15</td></tr>
<tr><td>gALL_v3_c1724</td><td>5</td><td>3</td><td>-</td><td>-</td><td>-</td><td>-</td><td>-</td></tr>
<tr><td>gALL_v3_c17240</td><td>0</td><td>5</td><td>-</td><td>-</td><td>-</td><td>-</td><td>-</td></tr>
<tr><td>gALL_v3_c17241</td><td>4</td><td>3</td><td>-</td><td>-</td><td>-</td><td>-</td><td>-</td></tr>
<tr><td>gALL_v3_c17243</td><td>1</td><td>6</td><td>-</td><td>-</td><td>-</td><td>-</td><td>-</td></tr>
<tr><td>gALL_v3_c17244</td><td>3</td><td>4</td><td>-</td><td>-</td><td>-</td><td>-</td><td>-</td></tr>
<tr><td>gALL_v3_c17245</td><td>1</td><td>4</td><td>-</td><td>-</td><td>-</td><td>-</td><td>-</td></tr>
<tr><td>gALL_v3_c17246</td><td>2</td><td>10</td><td>-</td><td>-</td><td>gb|EFA11579.1|</td><td>brain peptide IDL-like protein [Tribolium castaneum]</td><td>1,00E-88</td></tr>
<tr><td>gALL_v3_c17247</td><td>1</td><td>6</td><td>gnl|CDD|189784</td><td>pfam00957, Synaptobrevin, Sy</td><td>gb|EFN80310.1|</td><td>Vesicle-associated membrane protein 7 [Harpegnathos saltator]</td><td>2,00E-11</td></tr>
<tr><td>gALL_v3_c17248</td><td>1</td><td>6</td><td>-</td><td>-</td><td>-</td><td>-</td><td>-</td></tr>
<tr><td>gALL_v3_c17249</td><td>1</td><td>4</td><td>-</td><td>-</td><td>-</td><td>-</td><td>-</td></tr>
<tr><td>gALL_v3_c1725</td><td>4</td><td>4</td><td>gnl|CDD|128401</td><td>smart00090, RIO, RIO-like kina</td><td>ref|XP_002408198.1|</td><td>serine/threonine protein kinase rio3, putative [Ixodes scapularis] gb|EEC02041.1| serine/threonine protein kinase rio3, putative [Ixodes scapularis]</td><td>9,00E-49</td></tr>
<tr><td>gALL_v3_c17250</td><td>7</td><td>10</td><td>gnl|CDD|147642</td><td>pfam05585, DUF1758, Putativ</td><td>ref|XP_002136118.1|</td><td>GA28115 [Drosophila pseudoobscura pseudoobscura] gb|EDY71085.1| GA28115 [Drosophila pseudoobscura pseudoobscura]</td><td>2,00E-38</td></tr>
<tr><td>gALL_v3_c17251</td><td>2</td><td>2</td><td>gnl|CDD|48157</td><td>cd03340, TCP1_eta, TCP-1 (CT</td><td>ref|XP_004518105.1|</td><td>PREDICTED: T-complex protein 1 subunit eta-like [Ceratitis capitata]</td><td>3,00E-22</td></tr>
<tr><td>gALL_v3_c17252</td><td>5</td><td>2</td><td>gnl|CDD|189390</td><td>pfam00102, Y_phosphatase, P</td><td>ref|XP_002432163.1|</td><td>protein tyrosine phosphatse n18, putative [Pediculus humanus corporis] gb|EEB19425.1| protein tyrosine phosphatse n18, putative [Pediculus humanus corporis]</td><td>1,00E-105</td></tr>
<tr><td>gALL_v3_c17254</td><td>1</td><td>4</td><td>-</td><td>-</td><td>-</td><td>-</td><td>-</td></tr>
<tr><td>gALL_v3_c17255</td><td>1</td><td>4</td><td>-</td><td>-</td><td>-</td><td>-</td><td>-</td></tr>
<tr><td>gALL_v3_c17256</td><td>2</td><td>3</td><td>-</td><td>-</td><td>-</td><td>-</td><td>-</td></tr>
<tr><td>gALL_v3_c17257</td><td>4</td><td>2</td><td>gnl|CDD|153273</td><td>cd07589, BAR_DNMBP, The Bi</td><td>gb|EGI61402.1|</td><td>Dynamin-binding protein [Acromyrmex echinatior]</td><td>5,00E-33</td></tr>
<tr><td>gALL_v3_c17258</td><td>5</td><td>4</td><td>gnl|CDD|176236</td><td>cd08275, MDR3, Medium chai</td><td>ref|XP_971755.1|</td><td>PREDICTED: similar to vesicle amine transport protein [Tribolium castaneum] gb|EFA06248.1| hypothetical protein TcasGA2_TC009106 [Tribolium castaneum]</td><td>3,00E-46</td></tr>
<tr><td>gALL_v3_c17259</td><td>5</td><td>6</td><td>-</td><td>-</td><td>gb|ESO12785.1|</td><td>hypothetical protein HELRODRAFT_159370 [Helobdella robusta]</td><td>6,00E-19</td></tr>
<tr><td>gALL_v3_c1726</td><td>3</td><td>6</td><td>-</td><td>-</td><td>-</td><td>-</td><td>-</td></tr>
<tr><td>gALL_v3_c17261</td><td>33</td><td>26</td><td>-</td><td>-</td><td>-</td><td>-</td><td>-</td></tr>
<tr><td>gALL_v3_c17263</td><td>4</td><td>2</td><td>-</td><td>-</td><td>gb|EPS41927.1|</td><td>hypothetical protein H072_4140 [Dactylellina haptotyla CBS 200.50]</td><td>3,00E-08</td></tr>
<tr><td>gALL_v3_c17265</td><td>5</td><td>2</td><td>-</td><td>-</td><td>-</td><td>-</td><td>-</td></tr>
<tr><td>gALL_v3_c17266</td><td>2</td><td>2</td><td>-</td><td>-</td><td>-</td><td>-</td><td>-</td></tr>
<tr><td>gALL_v3_c17267</td><td>5</td><td>2</td><td>-</td><td>-</td><td>-</td><td>-</td><td>-</td></tr>
<tr><td>gALL_v3_c17268</td><td>0</td><td>4</td><td>-</td><td>-</td><td>-</td><td>-</td><td>-</td></tr>
<tr><td>gALL_v3_c17269</td><td>1</td><td>7</td><td>-</td><td>-</td><td>-</td><td>-</td><td>-</td></tr>
<tr><td>gALL_v3_c17270</td><td>3</td><td>2</td><td>-</td><td>-</td><td>-</td><td>-</td><td>-</td></tr>
<tr><td>gALL_v3_c17271</td><td>3</td><td>7</td><td>-</td><td>-</td><td>gb|EFN74788.1|</td><td>Nuclear receptor coactivator 7 [Camponotus floridanus]</td><td>4,00E-31</td></tr>
<tr><td>gALL_v3_c17272</td><td>2</td><td>3</td><td>-</td><td>-</td><td>-</td><td>-</td><td>-</td></tr>
<tr><td>gALL_v3_c17273</td><td>2</td><td>3</td><td>-</td><td>-</td><td>-</td><td>-</td><td>-</td></tr>
<tr><td>gALL_v3_c17274</td><td>1</td><td>4</td><td>-</td><td>-</td><td>-</td><td>-</td><td>-</td></tr>
<tr><td>gALL_v3_c17276</td><td>3</td><td>2</td><td>-</td><td>-</td><td>-</td><td>-</td><td>-</td></tr>
<tr><td>gALL_v3_c17277</td><td>2</td><td>2</td><td>gnl|CDD|73156</td><td>cd01650, RT_nLTR_like, RT_nL</td><td>ref|XP_004213263.1|</td><td>PREDICTED: RNA-directed DNA polymerase from mobile element jockey-like, partial [Hydra vulgaris]</td><td>2,00E-30</td></tr>
<tr><td>gALL_v3_c17278</td><td>4</td><td>10</td><td>gnl|CDD|176924</td><td>cd09071, FAR_C, C-terminal d</td><td>gb|EHJ70556.1|</td><td>fatty-acyl CoA reductase 1 [Danaus plexippus]</td><td>2,00E-33</td></tr>
<tr><td>gALL_v3_c17279</td><td>1</td><td>4</td><td>-</td><td>-</td><td>-</td><td>-</td><td>-</td></tr>
<tr><td>gALL_v3_c1728</td><td>3</td><td>6</td><td>-</td><td>-</td><td>-</td><td>-</td><td>-</td></tr>
<tr><td>gALL_v3_c17280</td><td>2</td><td>3</td><td>-</td><td>-</td><td>-</td><td>-</td><td>-</td></tr>
<tr><td>gALL_v3_c17281</td><td>3</td><td>4</td><td>gnl|CDD|34673</td><td>COG5069, SAC6, Ca2+-binding</td><td>gb|EFN60848.1|</td><td>Plastin-3 [Camponotus floridanus]</td><td>8,00E-67</td></tr>
<tr><td>gALL_v3_c17282</td><td>0</td><td>4</td><td>-</td><td>-</td><td>-</td><td>-</td><td>-</td></tr>
<tr><td>gALL_v3_c17283</td><td>3</td><td>1</td><td>-</td><td>-</td><td>ref|XP_006564667.1|</td><td>PREDICTED: uncharacterized protein LOC413627 isoform X1 [Apis mellifera] ref|XP_006564668.1| PREDICTED: uncharacterized protein LOC413627 isoform X2 [Apis mellife</td><td>1,00E-14</td></tr>
<tr><td>gALL_v3_c17284</td><td>5</td><td>4</td><td>-</td><td>-</td><td>gb|AGM32821.1|</td><td>hypothetical protein [Coptotermes formosanus]</td><td>5,00E-62</td></tr>
<tr><td>gALL_v3_c17285</td><td>2</td><td>2</td><td>-</td><td>-</td><td>-</td><td>-</td><td>-</td></tr>
<tr><td>gALL_v3_c17286</td><td>4</td><td>3</td><td>-</td><td>-</td><td>-</td><td>-</td><td>-</td></tr>
<tr><td>gALL_v3_c17288</td><td>11</td><td>15</td><td>-</td><td>-</td><td>-</td><td>-</td><td>-</td></tr>
<tr><td>gALL_v3_c17289</td><td>4</td><td>4</td><td>-</td><td>-</td><td>gb|EHJ69710.1|</td><td>hypothetical protein KGM_19245 [Danaus plexippus]</td><td>4,00E-78</td></tr>
<tr><td>gALL_v3_c1729</td><td>7</td><td>8</td><td>gnl|CDD|189008</td><td>cd09601, M1_APN_2, Peptida</td><td>gb|EGI70839.1|</td><td>Aminopeptidase N [Acromyrmex echinatior]</td><td>5,00E-11</td></tr>
<tr><td>gALL_v3_c17290</td><td>2</td><td>5</td><td>-</td><td>-</td><td>-</td><td>-</td><td>-</td></tr>
<tr><td>gALL_v3_c17291</td><td>6</td><td>3</td><td>gnl|CDD|173673</td><td>cd05582, STKc_RSK_N, N-term</td><td>ref|XP_002432758.1|</td><td>Ribosomal protein S6 kinase alpha-2, putative [Pediculus humanus corporis] gb|EEB20020.1| Ribosomal protein S6 kinase alpha-2, putative [Pediculus humanus corporis]</td><td>1,00E-137</td></tr>
<tr><td>gALL_v3_c17292</td><td>3</td><td>1</td><td>-</td><td>-</td><td>-</td><td>-</td><td>-</td></tr>
<tr><td>gALL_v3_c17293</td><td>1</td><td>4</td><td>-</td><td>-</td><td>-</td><td>-</td><td>-</td></tr>
<tr><td>gALL_v3_c17294</td><td>3</td><td>2</td><td>-</td><td>-</td><td>-</td><td>-</td><td>-</td></tr>
<tr><td>gALL_v3_c17295</td><td>7</td><td>6</td><td>gnl|CDD|28913</td><td>cd00031, CA, Cadherin repeat</td><td>ref|XP_002432680.1|</td><td>conserved hypothetical protein [Pediculus humanus corporis] gb|EEB19942.1| conserved hypothetical protein [Pediculus humanus corporis]</td><td>1,00E-152</td></tr>
<tr><td>gALL_v3_c17296</td><td>1</td><td>5</td><td>gnl|CDD|79367</td><td>cd03685, ClC_6_like, ClC-6-like</td><td>gb|EFN81286.1|</td><td>Chloride channel protein 7 [Harpegnathos saltator]</td><td>2,00E-60</td></tr>
<tr><td>gALL_v3_c17297</td><td>6</td><td>5</td><td>gnl|CDD|173662</td><td>cd05571, STKc_PKB, Catalytic</td><td>ref|XP_002427161.1|</td><td>RAC protein kinase DRAC-PK85, putative [Pediculus humanus corporis] gb|EEB14423.1| RAC protein kinase DRAC-PK85, putative [Pediculus humanus corporis]</td><td>1,00E-179</td></tr>
<tr><td>gALL_v3_c17298</td><td>4</td><td>2</td><td>-</td><td>-</td><td>-</td><td>-</td><td>-</td></tr>
<tr><td>gALL_v3_c17299</td><td>2</td><td>3</td><td>gnl|CDD|190262</td><td>pfam02259, FAT, FAT domain.</td><td>gb|ACH47049.1|</td><td>target of rapamycin [Blattella germanica]</td><td>4,00E-71</td></tr>
<tr><td>gALL_v3_c173</td><td>3</td><td>12</td><td>-</td><td>-</td><td>-</td><td>-</td><td>-</td></tr>
<tr><td>gALL_v3_c1730</td><td>4</td><td>5</td><td>-</td><td>-</td><td>gb|ENN79495.1|</td><td>hypothetical protein YQE_04057, partial [Dendroctonus ponderosae] gb|ENN82658.1| hypothetical protein YQE_00971, partial [Dendroctonus ponderosae]</td><td>6,00E-38</td></tr>
<tr><td>gALL_v3_c17300</td><td>3</td><td>2</td><td>gnl|CDD|162812</td><td>TIGR02346, chap_CCT_theta,</td><td>ref|XP_003766078.1|</td><td>PREDICTED: T-complex protein 1 subunit theta-like [Sarcophilus harrisii]</td><td>2,00E-33</td></tr>
<tr><td>gALL_v3_c17301</td><td>6</td><td>3</td><td>-</td><td>-</td><td>-</td><td>-</td><td>-</td></tr>
<tr><td>gALL_v3_c17302</td><td>12</td><td>9</td><td>gnl|CDD|185485</td><td>PTZ00156, PTZ00156, 60S ribo</td><td>gb|AGM32143.1|</td><td>60S ribosomal protein L11 [Coptotermes formosanus]</td><td>1,00E-110</td></tr>
<tr><td>gALL_v3_c17303</td><td>8</td><td>15</td><td>-</td><td>-</td><td>ref|XP_004207304.1|</td><td>PREDICTED: uncharacterized protein LOC101237178 [Hydra vulgaris]</td><td>1,00E-08</td></tr>
<tr><td>gALL_v3_c17304</td><td>1</td><td>4</td><td>gnl|CDD|109126</td><td>pfam00057, Ldl_recept_a, Low</td><td>ref|XP_001814959.1|</td><td>PREDICTED: similar to AGAP000037-PA [Tribolium castaneum]</td><td>5,00E-40</td></tr>
<tr><td>gALL_v3_c17305</td><td>1</td><td>6</td><td>-</td><td>-</td><td>-</td><td>-</td><td>-</td></tr>
<tr><td>gALL_v3_c17306</td><td>1</td><td>5</td><td>-</td><td>-</td><td>-</td><td>-</td><td>-</td></tr>
<tr><td>gALL_v3_c17307</td><td>4</td><td>13</td><td>-</td><td>-</td><td>ref|XP_004930508.1|</td><td>PREDICTED: inositol hexakisphosphate and diphosphoinositol-pentakisphosphate kinase-like [Bombyx mori]</td><td>0</td></tr>
<tr><td>gALL_v3_c17308</td><td>1</td><td>5</td><td>-</td><td>-</td><td>-</td><td>-</td><td>-</td></tr>
<tr><td>gALL_v3_c17309</td><td>0</td><td>5</td><td>-</td><td>-</td><td>-</td><td>-</td><td>-</td></tr>
<tr><td>gALL_v3_c1731</td><td>10</td><td>20</td><td>-</td><td>-</td><td>-</td><td>-</td><td>-</td></tr>
<tr><td>gALL_v3_c17310</td><td>3</td><td>4</td><td>gnl|CDD|30046</td><td>cd01303, GDEase, Guanine de</td><td>gb|AGM32703.1|</td><td>guanine deaminase-like protein, partial [Coptotermes formosanus]</td><td>3,00E-36</td></tr>
<tr><td>gALL_v3_c17311</td><td>2</td><td>10</td><td>-</td><td>-</td><td>ref|XP_006617298.1|</td><td>PREDICTED: dnaJ homolog subfamily C member 13-like [Apis dorsata]</td><td>4,00E-91</td></tr>
<tr><td>gALL_v3_c17313</td><td>2</td><td>3</td><td>-</td><td>-</td><td>-</td><td>-</td><td>-</td></tr>
<tr><td>gALL_v3_c17314</td><td>1</td><td>5</td><td>-</td><td>-</td><td>-</td><td>-</td><td>-</td></tr>
<tr><td>gALL_v3_c17315</td><td>1</td><td>3</td><td>-</td><td>-</td><td>-</td><td>-</td><td>-</td></tr>
<tr><td>gALL_v3_c17316</td><td>1</td><td>3</td><td>-</td><td>-</td><td>-</td><td>-</td><td>-</td></tr>
<tr><td>gALL_v3_c17317</td><td>1</td><td>4</td><td>-</td><td>-</td><td>-</td><td>-</td><td>-</td></tr>
<tr><td>gALL_v3_c17318</td><td>4</td><td>2</td><td>gnl|CDD|178765</td><td>PLN03226, PLN03226, serine h</td><td>gb|AGC92253.1|</td><td>putative serine hydroxymethyltransferase, partial [Laodelphax striatella]</td><td>9,00E-87</td></tr>
<tr><td>gALL_v3_c17319</td><td>2</td><td>5</td><td>-</td><td>-</td><td>ref|XP_001602772.2|</td><td>PREDICTED: hypothetical protein LOC100118905 [Nasonia vitripennis]</td><td>4,00E-08</td></tr>
<tr><td>gALL_v3_c17320</td><td>1</td><td>3</td><td>-</td><td>-</td><td>-</td><td>-</td><td>-</td></tr>
<tr><td>gALL_v3_c17322</td><td>3</td><td>1</td><td>gnl|CDD|30777</td><td>COG0428, COG0428, Predicte</td><td>gb|EEZ99867.1|</td><td>hypothetical protein TcasGA2_TC002649 [Tribolium castaneum]</td><td>8,00E-31</td></tr>
<tr><td>gALL_v3_c17323</td><td>4</td><td>3</td><td>-</td><td>-</td><td>-</td><td>-</td><td>-</td></tr>
<tr><td>gALL_v3_c17324</td><td>3</td><td>1</td><td>-</td><td>-</td><td>-</td><td>-</td><td>-</td></tr>
<tr><td>gALL_v3_c17325</td><td>1</td><td>3</td><td>-</td><td>-</td><td>-</td><td>-</td><td>-</td></tr>
<tr><td>gALL_v3_c17326</td><td>3</td><td>2</td><td>-</td><td>-</td><td>-</td><td>-</td><td>-</td></tr>
<tr><td>gALL_v3_c17328</td><td>1</td><td>5</td><td>-</td><td>-</td><td>-</td><td>-</td><td>-</td></tr>
<tr><td>gALL_v3_c17329</td><td>1</td><td>3</td><td>-</td><td>-</td><td>-</td><td>-</td><td>-</td></tr>
<tr><td>gALL_v3_c1733</td><td>7</td><td>15</td><td>-</td><td>-</td><td>-</td><td>-</td><td>-</td></tr>
<tr><td>gALL_v3_c17330</td><td>5</td><td>2</td><td>-</td><td>-</td><td>gb|EKC20424.1|</td><td>Peptide-N(4)-(N-acetyl-beta-glucosaminyl)asparagine amidase [Crassostrea gigas]</td><td>2,00E-08</td></tr>
<tr><td>gALL_v3_c17332</td><td>0</td><td>4</td><td>-</td><td>-</td><td>gb|EFZ23153.1|</td><td>hypothetical protein SINV_03316 [Solenopsis invicta]</td><td>2,00E-54</td></tr>
<tr><td>gALL_v3_c17333</td><td>0</td><td>4</td><td>-</td><td>-</td><td>-</td><td>-</td><td>-</td></tr>
<tr><td>gALL_v3_c17334</td><td>5</td><td>2</td><td>-</td><td>-</td><td>ref|XP_003426403.1|</td><td>PREDICTED: lysosomal alpha-mannosidase-like isoform 2 [Nasonia vitripennis]</td><td>6,00E-54</td></tr>
<tr><td>gALL_v3_c17335</td><td>2</td><td>2</td><td>-</td><td>-</td><td>ref|XP_004207470.1|</td><td>PREDICTED: uncharacterized protein LOC100197081 [Hydra vulgaris]</td><td>5,00E-14</td></tr>
<tr><td>gALL_v3_c17337</td><td>1</td><td>3</td><td>-</td><td>-</td><td>-</td><td>-</td><td>-</td></tr>
<tr><td>gALL_v3_c17338</td><td>3</td><td>2</td><td>-</td><td>-</td><td>-</td><td>-</td><td>-</td></tr>
<tr><td>gALL_v3_c17339</td><td>3</td><td>2</td><td>-</td><td>-</td><td>-</td><td>-</td><td>-</td></tr>
<tr><td>gALL_v3_c1734</td><td>18</td><td>23</td><td>gnl|CDD|34760</td><td>COG5159, RPN6, 26S proteaso</td><td>gb|AGM32697.1|</td><td>PCI domain containing protein, partial [Coptotermes formosanus]</td><td>1,00E-178</td></tr>
<tr><td>gALL_v3_c17340</td><td>2</td><td>3</td><td>-</td><td>-</td><td>ref|XP_001604596.2|</td><td>PREDICTED: regulating synaptic membrane exocytosis protein 2-like [Nasonia vitripennis]</td><td>3,00E-22</td></tr>
<tr><td>gALL_v3_c17341</td><td>2</td><td>4</td><td>-</td><td>-</td><td>-</td><td>-</td><td>-</td></tr>
<tr><td>gALL_v3_c17342</td><td>4</td><td>2</td><td>-</td><td>-</td><td>-</td><td>-</td><td>-</td></tr>
<tr><td>gALL_v3_c17343</td><td>1</td><td>3</td><td>-</td><td>-</td><td>-</td><td>-</td><td>-</td></tr>
<tr><td>gALL_v3_c17344</td><td>2</td><td>2</td><td>-</td><td>-</td><td>-</td><td>-</td><td>-</td></tr>
<tr><td>gALL_v3_c17346</td><td>3</td><td>3</td><td>-</td><td>-</td><td>-</td><td>-</td><td>-</td></tr>
<tr><td>gALL_v3_c17347</td><td>4</td><td>6</td><td>-</td><td>-</td><td>-</td><td>-</td><td>-</td></tr>
<tr><td>gALL_v3_c17349</td><td>4</td><td>3</td><td>-</td><td>-</td><td>ref|XP_006029730.1|</td><td>PREDICTED: histone-lysine N-methyltransferase SETMAR-like [Alligator sinensis]</td><td>8,00E-14</td></tr>
<tr><td>gALL_v3_c1735</td><td>5</td><td>7</td><td>-</td><td>-</td><td>-</td><td>-</td><td>-</td></tr>
<tr><td>gALL_v3_c17350</td><td>4</td><td>2</td><td>-</td><td>-</td><td>-</td><td>-</td><td>-</td></tr>
<tr><td>gALL_v3_c17352</td><td>3</td><td>4</td><td>-</td><td>-</td><td>-</td><td>-</td><td>-</td></tr>
<tr><td>gALL_v3_c17353</td><td>0</td><td>4</td><td>-</td><td>-</td><td>-</td><td>-</td><td>-</td></tr>
<tr><td>gALL_v3_c17354</td><td>1</td><td>6</td><td>-</td><td>-</td><td>-</td><td>-</td><td>-</td></tr>
<tr><td>gALL_v3_c17355</td><td>0</td><td>4</td><td>-</td><td>-</td><td>-</td><td>-</td><td>-</td></tr>
<tr><td>gALL_v3_c17356</td><td>1</td><td>8</td><td>-</td><td>-</td><td>-</td><td>-</td><td>-</td></tr>
<tr><td>gALL_v3_c17357</td><td>4</td><td>2</td><td>-</td><td>-</td><td>gb|EEZ98560.1|</td><td>hypothetical protein TcasGA2_TC001067 [Tribolium castaneum]</td><td>2,00E-21</td></tr>
<tr><td>gALL_v3_c17359</td><td>3</td><td>2</td><td>-</td><td>-</td><td>-</td><td>-</td><td>-</td></tr>
<tr><td>gALL_v3_c1736</td><td>5</td><td>5</td><td>-</td><td>-</td><td>-</td><td>-</td><td>-</td></tr>
<tr><td>gALL_v3_c17360</td><td>2</td><td>3</td><td>-</td><td>-</td><td>gb|EFX80323.1|</td><td>hypothetical protein DAPPUDRAFT_304059 [Daphnia pulex]</td><td>1,00E-10</td></tr>
<tr><td>gALL_v3_c17361</td><td>8</td><td>5</td><td>-</td><td>-</td><td>-</td><td>-</td><td>-</td></tr>
<tr><td>gALL_v3_c17362</td><td>2</td><td>3</td><td>-</td><td>-</td><td>gb|EFN75239.1|</td><td>Nuclear pore complex protein Nup98-Nup96 [Harpegnathos saltator]</td><td>1,00E-42</td></tr>
<tr><td>gALL_v3_c17363</td><td>2</td><td>5</td><td>-</td><td>-</td><td>-</td><td>-</td><td>-</td></tr>
<tr><td>gALL_v3_c17365</td><td>7</td><td>17</td><td>-</td><td>-</td><td>-</td><td>-</td><td>-</td></tr>
<tr><td>gALL_v3_c17366</td><td>3</td><td>1</td><td>gnl|CDD|178252</td><td>PLN02646, PLN02646, argininc</td><td>ref|XP_005926381.1|</td><td>PREDICTED: argininosuccinate lyase-like [Haplochromis burtoni]</td><td>2,00E-38</td></tr>
<tr><td>gALL_v3_c17368</td><td>1</td><td>6</td><td>-</td><td>-</td><td>-</td><td>-</td><td>-</td></tr>
<tr><td>gALL_v3_c17369</td><td>4</td><td>4</td><td>-</td><td>-</td><td>-</td><td>-</td><td>-</td></tr>
<tr><td>gALL_v3_c1737</td><td>20</td><td>22</td><td>gnl|CDD|128594</td><td>smart00299, CLH, Clathrin hea</td><td>gb|EGI60613.1|</td><td>Clathrin heavy chain [Acromyrmex echinatior]</td><td>0</td></tr>
<tr><td>gALL_v3_c17370</td><td>5</td><td>8</td><td>gnl|CDD|187614</td><td>cd05356, 17beta-HSD1_like_S</td><td>ref|XP_971720.1|</td><td>PREDICTED: similar to steroid dehydrogenase [Tribolium castaneum] gb|EFA10853.1| hypothetical protein TcasGA2_TC001630 [Tribolium castaneum]</td><td>1,00E-133</td></tr>
<tr><td>gALL_v3_c17371</td><td>1</td><td>4</td><td>-</td><td>-</td><td>-</td><td>-</td><td>-</td></tr>
<tr><td>gALL_v3_c17372</td><td>2</td><td>4</td><td>-</td><td>-</td><td>-</td><td>-</td><td>-</td></tr>
<tr><td>gALL_v3_c17373</td><td>4</td><td>9</td><td>-</td><td>-</td><td>-</td><td>-</td><td>-</td></tr>
<tr><td>gALL_v3_c17374</td><td>11</td><td>24</td><td>gnl|CDD|178562</td><td>PLN02981, PLN02981, glucosa</td><td>ref|XP_003395056.1|</td><td>PREDICTED: glucosamine--fructose-6-phosphate aminotransferase [isomerizing] 2-like isoform 2 [Bombus terrestris]</td><td>1,00E-176</td></tr>
<tr><td>gALL_v3_c17375</td><td>3</td><td>5</td><td>gnl|CDD|188660</td><td>cd08705, RGS_R7-like, Regulat</td><td>ref|XP_967637.1|</td><td>PREDICTED: similar to regulator of g protein signaling [Tribolium castaneum] gb|EFA11094.1| hypothetical protein TcasGA2_TC004691 [Tribolium castaneum]</td><td>8,00E-57</td></tr>
<tr><td>gALL_v3_c17376</td><td>3</td><td>2</td><td>gnl|CDD|187564</td><td>cd05254, dTDP_HR_like_SDR_</td><td>gb|EFX90174.1|</td><td>hypothetical protein DAPPUDRAFT_299978 [Daphnia pulex]</td><td>6,00E-45</td></tr>
<tr><td>gALL_v3_c17377</td><td>9</td><td>5</td><td>-</td><td>-</td><td>gb|AAK61417.1|</td><td>transposase [Ceratitis rosa]</td><td>3,00E-28</td></tr>
<tr><td>gALL_v3_c17378</td><td>1</td><td>3</td><td>-</td><td>-</td><td>-</td><td>-</td><td>-</td></tr>
</table>

gALL_v3_c1738	8	12	-	-	gb|EGI59747.1|	N-acetyltransferase MAK3-like protein [Acromyrmex echinatior]	8,00E-68
gALL_v3_c17380	0	3	-	-	-	-	-
gALL_v3_c17381	1	3	-	-	-	-	-
gALL_v3_c17383	1	3	-	-	-	-	-
gALL_v3_c17384	2	4	-	-	-	-	-
gALL_v3_c17385	3	3	-	-	gb|ENN73544.1|	hypothetical protein YQE_09795, partial [Dendroctonus ponderosae]	1,00E-110
gALL_v3_c17386	6	13	-	-	ref|XP_002425828.1|	conserved hypothetical protein [Pediculus humanus corporis] gb|EEB13090.1| conserved hypothetical protein [Pediculus humanus corporis]	3,00E-23
gALL_v3_c17387	8	19	-	-	-	-	-
gALL_v3_c17389	2	3	-	-	-	-	-
gALL_v3_c1739	5	16	gnl|CDD|187700	cd09276, Rnase_HI_RT_non_L	dbj|BAE44464.1|	non-LTR retrotransposon CATS [Bombyx mori]	8,00E-58
gALL_v3_c17390	0	5	-	-	-	-	-
gALL_v3_c17391	2	4	-	-	ref|XP_004930958.1|	PREDICTED: uncharacterized protein LOC101737598 [Bombyx mori]	3,00E-31
gALL_v3_c17392	2	5	gnl|CDD|192780	pfam11594, Med28, Mediator	ref|XP_001815546.1|	PREDICTED: similar to mediator complex [Tribolium castaneum] gb|EEZ98645.1| hypothetical protein TcasGA2_TC001173 [Tribolium castaneum]	1,00E-55
gALL_v3_c17393	3	9	-	-	-	-	-
gALL_v3_c17394	2	3	-	-	gb|EFN69246.1|	Neurotrypsin [Camponotus floridanus]	6,00E-50
gALL_v3_c17395	1	5	-	-	-	-	-
gALL_v3_c17396	2	2	gnl|CDD|179074	PRK00591, prfA, peptide chair	gb|ETN62707.1|	peptide chain release factor 1 [Anopheles darlingi]	1,00E-54
gALL_v3_c17397	1	4	-	-	-	-	-
gALL_v3_c17398	3	1	-	-	-	-	-
gALL_v3_c174	10	9	-	-	ref|XP_002429507.1|	conserved hypothetical protein [Pediculus humanus corporis] gb|EEB16769.1| conserved hypothetical protein [Pediculus humanus corporis]	2,00E-17
gALL_v3_c17400	1	4	-	-	-	-	-
gALL_v3_c17401	3	5	-	-	-	-	-
gALL_v3_c17402	4	2	-	-	-	-	-
gALL_v3_c17403	2	4	gnl|CDD|117221	pfam08648, DUF1777, Protein	ref|WP_021510632.1|	copper resistance protein CopA, partial [Acinetobacter baumannii] gb|ERH70024.1| copper resistance protein CopA, partial [Acinetobacter baumannii EGD-HP18]	3,00E-12
gALL_v3_c17404	5	5	-	-	-	-	-
gALL_v3_c17405	3	3	gnl|CDD|128548	smart00252, SH2, Src homolog	gb|AGZ15161.1|	phosphoesterase 4, partial [Laodelphax striatella]	9,00E-57
gALL_v3_c17407	4	2	-	-	-	-	-
gALL_v3_c17408	3	1	-	-	-	-	-
gALL_v3_c17409	10	7	gnl|CDD|173949	cd08190, HOT, Hydroxyacid-ox	gb|EFZ17696.1|	hypothetical protein SINV_01501 [Solenopsis invicta]	2,33E-156
gALL_v3_c1741	9	5	gnl|CDD|145069	pfam01722, BolA, BolA-like pr	ref|XP_003700244.1|	PREDICTED: bolA-like protein 2-like [Megachile rotundata]	3,00E-30
gALL_v3_c17410	6	4	-	-	-	-	-
gALL_v3_c17411	2	2	-	-	-	-	-
gALL_v3_c17412	4	8	-	-	-	-	-
gALL_v3_c17413	0	4	-	-	ref|XP_969710.2|	PREDICTED: similar to CG4733 CG4733-PA [Tribolium castaneum] gb|EFA03124.1| hypothetical protein TcasGA2_TC013034 [Tribolium castaneum]	2,00E-46
gALL_v3_c17414	2	4	-	-	-	-	-
gALL_v3_c17417	0	4	-	-	-	-	-
gALL_v3_c17418	4	4	-	-	gb|EFN78676.1|	Histone-lysine N-methyltransferase SETMAR [Harpegnathos saltator]	5,00E-08
gALL_v3_c1742	11	17	-	-	-	-	-
gALL_v3_c17420	1	4	-	-	-	-	-
gALL_v3_c17421	3	4	-	-	gb|EFN83934.1|	Protein asteroid [Harpegnathos saltator]	4,00E-14
gALL_v3_c17422	2	5	-	-	-	-	-
gALL_v3_c17423	1	6	-	-	-	-	-
gALL_v3_c17424	1	4	-	-	-	-	-
gALL_v3_c17425	1	4	-	-	gb|EGI65398.1|	FLJ37770-like protein [Acromyrmex echinatior]	7,00E-14
gALL_v3_c17426	3	2	-	-	-	-	-
gALL_v3_c17427	4	3	-	-	-	-	-
gALL_v3_c17428	2	2	-	-	ref|XP_972609.1|	PREDICTED: similar to guanyl-nucleotide exchange factor [Tribolium castaneum] gb|EFA11203.1| hypothetical protein TcasGA2_TC005167 [Tribolium castaneum]	2,00E-51
gALL_v3_c17429	2	8	-	-	ref|XP_003702656.1|	PREDICTED: uncharacterized protein LOC100875153 [Megachile rotundata]	4,00E-10
gALL_v3_c1743	28	27	gnl|CDD|150314	pfam09606, Med15, ARC105 c	ref|XP_002425652.1|	hypothetical protein Phum_PHUM213210 [Pediculus humanus corporis] gb|EEB12914.1| hypothetical protein Phum_PHUM213210 [Pediculus humanus corporis]	1,00E-162
gALL_v3_c17430	3	5	-	-	ref|XP_001603678.2|	PREDICTED: stAR-related lipid transfer protein 7, mitochondrial-like [Nasonia vitripennis]	8,00E-25
gALL_v3_c17431	4	5	-	-	-	-	-
gALL_v3_c17433	1	4	gnl|CDD|144428	pfam00829, Ribosomal_L21p,	gb|EFZ15009.1|	hypothetical protein SINV_13821 [Solenopsis invicta]	3,00E-44
gALL_v3_c17434	4	4	gnl|CDD|145281	pfam02015, Glyco_hydro_45,	dbj|BAF57355.1|	putative glycosyl hydrolase family45 [uncultured symbiotic protist of Hodotermopsis sjoestedti]	1,00E-118
gALL_v3_c17435	10	10	-	-	-	-	-
gALL_v3_c17436	3	3	-	-	-	-	-
gALL_v3_c17437	2	5	-	-	-	-	-
gALL_v3_c17439	2	2	-	-	-	-	-
gALL_v3_c17440	1	4	-	-	ref|XP_006562745.1|	PREDICTED: uncharacterized protein LOC100578579 isoform X2 [Apis mellifera]	5,00E-22
gALL_v3_c17441	4	7	-	-	-	-	-
gALL_v3_c17442	0	5	-	-	-	-	-
gALL_v3_c17444	4	6	gnl|CDD|189661	pfam00665, rve, Integrase cor	gb|AAL90009.1|	AT07338p [Drosophila melanogaster]	1,00E-25
gALL_v3_c17445	3	8	-	-	-	-	-
gALL_v3_c17446	4	2	-	-	-	-	-
gALL_v3_c17447	1	3	-	-	ref|XP_005092232.1|	PREDICTED: E3 ubiquitin-protein ligase RNF34-like [Aplysia californica]	1,00E-06
gALL_v3_c17449	4	2	-	-	-	-	-
gALL_v3_c1745	12	17	gnl|CDD|173839	cd01150, AXO, Peroxisomal ac	ref|XP_001599966.2|	PREDICTED: probable peroxisomal acyl-coenzyme A oxidase 1-like [Nasonia vitripennis]	0
gALL_v3_c17450	3	2	-	-	-	-	-
gALL_v3_c17451	2	3	-	-	-	-	-
gALL_v3_c17452	6	6	-	-	-	-	-
gALL_v3_c17453	6	10	-	-	-	-	-
gALL_v3_c17454	2	2	-	-	-	-	-
gALL_v3_c17455	1	4	gnl|CDD|107357	cd06362, PBP1_mGluR, Liganc	ref|XP_001600546.2|	PREDICTED: metabotropic glutamate receptor-like [Nasonia vitripennis]	3,00E-67
gALL_v3_c17456	7	10	gnl|CDD|191262	pfam05362, Lon_C, Lon protea	ref|XP_006749843.1|	PREDICTED: lon protease homolog 2, peroxisomal-like, partial [Leptonychotes weddellii]	4,00E-36
gALL_v3_c17457	3	1	-	-	-	-	-
gALL_v3_c17458	0	5	-	-	-	-	-
gALL_v3_c17459	3	4	gnl|CDD|191687	pfam07093, SGT1, SGT1 prote	ref|XP_967648.1|	PREDICTED: similar to SGT1 protein homolog ecdysoneless [Tribolium castaneum] gb|EFA01447.1| hypothetical protein TcasGA2_TC030757 [Tribolium castaneum]	8,00E-63
gALL_v3_c1746	25	33	-	-	-	-	-
gALL_v3_c17460	0	4	-	-	-	-	-
gALL_v3_c17462	3	3	gnl|CDD|173623	cd00180, PKc, Catalytic domai	gb|EFN87865.1|	AP2-associated protein kinase 1 [Harpegnathos saltator]	4,00E-97
gALL_v3_c17463	2	2	-	-	gb|ENN73937.1|	hypothetical protein YQE_09439, partial [Dendroctonus ponderosae] gb|ERL94168.1| hypothetical protein D910_11450 [Dendroctonus ponderosae]	1,00E-24
gALL_v3_c17464	0	4	-	-	-	-	-
gALL_v3_c17465	2	4	gnl|CDD|162465	TIGR01652, ATPase-Plipid, phc	gb|EGI65273.1|	Putative phospholipid-transporting ATPase ID [Acromyrmex echinatior]	2,00E-74
gALL_v3_c17466	2	2	-	-	-	-	-
gALL_v3_c17467	5	10	gnl|CDD|29138	cd00176, SPEC, Spectrin repea	gb|EFA10673.1|	muscle-specific protein 300 [Tribolium castaneum]	1,00E-64
gALL_v3_c17469	0	3	-	-	-	-	-
gALL_v3_c1747	9	8	-	-	-	-	-
gALL_v3_c17470	3	4	gnl|CDD|189378	pfam00079, Serpin, Serpin (se	ref|XP_003402576.1|	PREDICTED: serine protease inhibitor 3/4-like, partial [Bombus terrestris]	2,00E-14
gALL_v3_c17471	3	5	-	-	-	-	-
gALL_v3_c17472	1	7	gnl|CDD|178650	PLN03103, PLN03103, GDP-L-g	ref|NP_001156693.1|	uncharacterized protein LOC100165161 [Acyrthosiphon pisum] dbj|BAH72188.1| ACYPI006123 [Acyrthosiphon pisum]	3,00E-86
gALL_v3_c17473	0	3	-	-	-	-	-
gALL_v3_c17474	4	10	gnl|CDD|58171	cd03505, Delta9-FADS-like, Th	gb|AAK25796.1|AF3384	delta-9 desaturase 1 [Acheta domesticus] gb|AAK25797.1|AF338466_1 delta-9 desaturase 3 [Acheta domesticus]	2,00E-49
gALL_v3_c17475	3	1	-	-	-	-	-
gALL_v3_c17477	3	2	gnl|CDD|144423	pfam00821, PEPCK, Phosphoe	emb|CDF66125.1|	phosphoenolpyruvate carboxykinase [Blattella germanica]	3,00E-62
gALL_v3_c17478	2	3	gnl|CDD|99764	cd01727, LSm8, The eukaryoti	ref|XP_624537.1|	PREDICTED: N-alpha-acetyltransferase 38, NatC auxiliary subunit-like [Apis mellifera] ref|XP_003401541.1| PREDICTED: n-alpha-acetyltransferase 38, NatC auxiliary subuni	2,00E-45
gALL_v3_c17479	3	2	-	-	ref|NP_001136324.1|	cytochrome b5-related-like [Nasonia vitripennis]	4,00E-27
gALL_v3_c1748	10	10	gnl|CDD|189970	pfam01395, PBP_GOBP, PBP/G	gb|AAQ20833.1|	odorant-binding protein precursor [Rhodnius prolixus]	2,00E-13
gALL_v3_c17480	3	3	-	-	ref|XP_001955264.1|	GF16322 [Drosophila ananassae] gb|EDV43825.1| GF16322 [Drosophila ananassae]	2,00E-20
gALL_v3_c17481	2	6	-	-	ref|XP_396649.4|	PREDICTED: fibroblast growth factor receptor homolog 1 isoform X2 [Apis mellifera]	9,00E-06
gALL_v3_c17482	3	2	-	-	-	-	-
gALL_v3_c17483	4	3	-	-	-	-	-
gALL_v3_c17484	3	1	-	-	-	-	-
gALL_v3_c17485	3	8	-	-	-	-	-
gALL_v3_c17486	2	6	-	-	-	-	-
gALL_v3_c17487	1	5	-	-	gb|EFN78473.1|	Zinc finger protein castor-like protein 1 [Harpegnathos saltator]	3,00E-99
gALL_v3_c17488	3	5	-	-	-	-	-
gALL_v3_c17489	2	3	-	-	-	-	-
gALL_v3_c1749	4	7	-	-	ref|XP_003396976.1|	PREDICTED: transcription initiation factor TFIID subunit 1-like isoform 1 [Bombus terrestris]	2,00E-77
gALL_v3_c17490	26	34	-	-	-	-	-
gALL_v3_c17491	0	5	-	-	-	-	-
gALL_v3_c17493	3	1	-	-	-	-	-
gALL_v3_c17494	2	3	-	-	-	-	-
gALL_v3_c17495	4	2	-	-	-	-	-
gALL_v3_c17496	5	16	-	-	gb|AAD16096.1|	reverse transcriptase [Nasonia vitripennis]	8,00E-21
gALL_v3_c17497	1	5	-	-	-	-	-
gALL_v3_c17498	2	10	-	-	-	-	-
gALL_v3_c17499	9	6	-	-	-	-	-
gALL_v3_c175	23	26	gnl|CDD|34710	COG5107, RNA14, Pre-mRNA 3	ref|XP_002425890.1|	pre-mRNA-splicing factor clf-1, putative [Pediculus humanus corporis] gb|EEB13152.1| pre-mRNA-splicing factor clf-1, putative [Pediculus humanus corporis]	0
gALL_v3_c1750	10	19	gnl|CDD|189758	pfam00887, ACBP, Acyl CoA bi	gb|EFN77524.1|	Acyl-CoA-binding domain-containing protein 4 [Harpegnathos saltator]	5,00E-78
gALL_v3_c17500	3	1	-	-	-	-	-
gALL_v3_c17501	2	5	-	-	gb|EFN60481.1|	hypothetical protein EAG_10563 [Camponotus floridanus]	3,00E-14
gALL_v3_c17503	2	2	-	-	-	-	-
gALL_v3_c17504	3	3	-	-	-	-	-
gALL_v3_c17505	0	5	-	-	-	-	-
gALL_v3_c17506	3	6	-	-	-	-	-
gALL_v3_c17507	1	4	-	-	gb|EFN74181.1|	Exportin-4 [Camponotus floridanus]	8,00E-51
gALL_v3_c1751	7	7	-	-	ref|XP_001952300.1|	PREDICTED: hypothetical protein LOC100168613 [Acyrthosiphon pisum]	9,00E-31
gALL_v3_c17510	2	3	gnl|CDD|162359	TIGR01438, TGR, thioredoxin a	gb|AHJ86276.1|	thioredoxin reductase, partial [Macrophthalmus japonicus]	6,00E-69
gALL_v3_c17511	5	3	-	-	ref|XP_001191267.2|	PREDICTED: protein cereblon-like [Strongylocentrotus purpuratus]	8,00E-61
gALL_v3_c17512	1	3	-	-	-	-	-
gALL_v3_c17513	0	4	-	-	-	-	-
gALL_v3_c17514	2	3	-	-	-	-	-
gALL_v3_c17515	4	4	-	-	-	-	-
gALL_v3_c17516	0	6	gnl|CDD|29006	cd02396, PCBP_like_KH, K hor	gb|EFX79671.1|	hypothetical protein DAPPUDRAFT_225024 [Daphnia pulex]	3,00E-58
gALL_v3_c17517	2	2	-	-	-	-	-
gALL_v3_c17519	1	6	gnl|CDD|188063	TIGR00558, pdxH, pyridoxami	gb|EFX70395.1|	hypothetical protein DAPPUDRAFT_129869 [Daphnia pulex]	3,00E-55
gALL_v3_c1752	8	7	-	-	gb|EGI67763.1|	Protein hu-li tai shao [Acromyrmex echinatior]	6,00E-48
gALL_v3_c17522	4	4	-	-	-	-	-
gALL_v3_c17523	1	4	-	-	-	-	-
gALL_v3_c17525	3	1	-	-	emb|CCJ37791.1|	decapentaplegic, partial [Blattella germanica]	1,00E-12
gALL_v3_c17526	4	3	gnl|CDD|191105	pfam04826, DUF634, Protein c	ref|XP_001947154.1|	PREDICTED: hypothetical protein LOC100165581 [Acyrthosiphon pisum]	2,00E-51
gALL_v3_c17527	7	13	-	-	-	-	-
gALL_v3_c17528	2	2	-	-	-	-	-
gALL_v3_c17529	7	4	gnl|CDD|152638	pfam12203, HDAC4_Gln, Gluta	gb|EGI63924.1|	Histone deacetylase 4 [Acromyrmex echinatior]	3,00E-25
gALL_v3_c1753	7	13	gnl|CDD|173737	cd07834, STKc_MAPK, Catalyti	gb|EFX89500.1|	hypothetical protein DAPPUDRAFT_40922 [Daphnia pulex]	5,00E-85

gALL_v3_c17530	1	5	gnl|CDD|189800	pfam01008, IF-2B, Initiation fa	ref|XP_001605978.2|	PREDICTED: translation initiation factor eIF-2B subunit delta-like isoform 1 [Nasonia vitripennis]	6,00E-74
gALL_v3_c17532	3	1	-	-	-	-	-
gALL_v3_c17534	6	3	-	-	-	-	-
gALL_v3_c17535	3	3	gnl|CDD|132961	cd06630, STKc_MEKK1, Cataly	ref|XP_005105346.1|	PREDICTED: mitogen-activated protein kinase kinase kinase 1-like isoform X3 [Aplysia californica]	1,00E-17
gALL_v3_c17536	7	4	-	-	ref|XP_006571274.1|	PREDICTED: rho GTPase-activating protein 190 isoform X3 [Apis mellifera]	1,00E-150
gALL_v3_c17537	2	3	gnl|CDD|128489	smart00192, LDLa, Low-densit	ref|XP_002429721.1|	low-density lipoprotein receptor, putative [Pediculus humanus corporis] gb|EEB16983.1| low-density lipoprotein receptor, putative [Pediculus humanus corporis]	2,00E-24
gALL_v3_c17538	2	4	-	-	-	-	-
gALL_v3_c17539	11	14	-	-	-	-	-
gALL_v3_c17540	3	6	gnl|CDD|29257	cd00200, WD40, WD40 domai	ref|XP_971473.2|	PREDICTED: similar to conserved hypothetical protein [Tribolium castaneum]	1,00E-140
gALL_v3_c17541	1	3	-	-	-	-	-
gALL_v3_c17543	3	2	-	-	-	-	-
gALL_v3_c17544	3	2	-	-	-	-	-
gALL_v3_c17545	1	3	-	-	-	-	-
gALL_v3_c17546	2	3	-	-	-	-	-
gALL_v3_c17547	4	3	-	-	-	-	-
gALL_v3_c17549	3	2	-	-	-	-	-
gALL_v3_c1755	8	17	gnl|CDD|79366	cd03684, ClC_3_like, ClC-3-like	gb|EFA00320.1|	hypothetical protein TcasGA2_TC003158 [Tribolium castaneum]	0
gALL_v3_c17550	0	3	-	-	-	-	-
gALL_v3_c17551	2	3	-	-	-	-	-
gALL_v3_c17552	3	1	-	-	-	-	-
gALL_v3_c17553	3	2	-	-	-	-	-
gALL_v3_c17554	8	4	-	-	ref|XP_006266998.1|	PREDICTED: zinc finger protein 135-like [Alligator mississippiensis]	1,00E-56
gALL_v3_c17555	9	13	gnl|CDD|185506	PTZ00189, PTZ00189, 60S ribo	ref|XP_001120360.1|	PREDICTED: 60S ribosomal protein L21 [Apis mellifera] ref|XP_003695518.1| PREDICTED: 60S ribosomal protein L21-like isoform 1 [Apis florea] ref|XP_003695519.1| PRED	2,00E-66
gALL_v3_c17556	2	1	-	-	-	-	-
gALL_v3_c17557	1	4	-	-	-	-	-
gALL_v3_c17559	0	4	-	-	ref|XP_005093017.1|	PREDICTED: uncharacterized protein LOC101850400 [Aplysia californica]	1,00E-11
gALL_v3_c1756	4	6	-	-	-	-	-
gALL_v3_c17560	6	4	-	-	gb|EGI69852.1|	Coiled-coil domain-containing protein 50 [Acromyrmex echinatior]	5,00E-19
gALL_v3_c17561	1	3	-	-	-	-	-
gALL_v3_c17562	0	5	-	-	-	-	-
gALL_v3_c17563	3	2	gnl|CDD|188096	TIGR00930, 2a30, K-Cl cotrans	ref|XP_001869952.1|	bumetanide-sensitive Na-K-Cl cotransport protein [Culex quinquefasciatus] gb|EDS30925.1| bumetanide-sensitive Na-K-Cl cotransport protein [Culex quinquefasciatus]	1,00E-54
gALL_v3_c17564	3	4	-	-	ref|XP_003699806.1|	PREDICTED: forkhead box protein K2-like [Megachile rotundata]	1,00E-94
gALL_v3_c17565	1	3	-	-	gb|EHJ75968.1|	hypothetical protein KGM_00415 [Danaus plexippus]	5,00E-20
gALL_v3_c17566	8	8	-	-	-	-	-
gALL_v3_c17567	2	4	-	-	-	-	-
gALL_v3_c17568	0	4	-	-	-	-	-
gALL_v3_c17569	2	6	-	-	-	-	-
gALL_v3_c1757	17	36	gnl|CDD|144972	pfam01576, Myosin_tail_1, M	ref|XP_002432985.1|	myosin-18A, putative [Pediculus humanus corporis] gb|EEB20247.1| myosin-18A, putative [Pediculus humanus corporis]	1,00E-150
gALL_v3_c17570	2	10	-	-	gb|EFN84153.1|	Flotillin-2 [Harpegnathos saltator]	4,00E-21
gALL_v3_c17572	4	4	gnl|CDD|143864	pfam00083, Sugar_tr, Sugar (a	ref|XP_315613.4|	AGAP005600-PA [Anopheles gambiae str. PEST] gb|EAA11457.4| AGAP005600-PA [Anopheles gambiae str. PEST]	1,00E-48
gALL_v3_c17573	3	1	-	-	-	-	-
gALL_v3_c17574	1	6	-	-	-	-	-
gALL_v3_c17575	0	4	-	-	-	-	-
gALL_v3_c17576	1	4	-	-	-	-	-
gALL_v3_c17577	3	2	-	-	gb|AGM32230.1|	hypothetical protein [Coptotermes formosanus]	6,00E-09
gALL_v3_c17578	4	6	-	-	gb|AGM32805.1|	endonuclease domain (L1-EN) containing protein [Coptotermes formosanus]	2,00E-11
gALL_v3_c17579	4	2	-	-	-	-	-
gALL_v3_c1758	5	4	gnl|CDD|144430	pfam00831, Ribosomal_L29, R	dbj|BAN20228.1|	ribosomal protein L35 [Riptortus pedestris]	4,00E-54
gALL_v3_c17580	3	6	-	-	-	-	-
gALL_v3_c17581	3	1	-	-	gb|EKC21711.1|	WD and tetratricopeptide repeats protein 1, partial [Crassostrea gigas]	6,00E-33
gALL_v3_c17582	3	3	-	-	-	-	-
gALL_v3_c17583	2	4	-	-	-	-	-
gALL_v3_c17584	3	4	-	-	-	-	-
gALL_v3_c17585	5	2	-	-	-	-	-
gALL_v3_c17586	10	21	-	-	ref|XP_006612814.1|	PREDICTED: serine/arginine repetitive matrix protein 2-like isoform X1 [Apis dorsata]	2,00E-20
gALL_v3_c17588	0	4	gnl|CDD|188096	TIGR00930, 2a30, K-Cl cotrans	gb|ABC84345.1|	cation-chloride cotransporter [Manduca sexta]	5,00E-43
gALL_v3_c1759	3	10	-	-	dbj|GAA55505.1|	hypothetical protein CLF_108196 [Clonorchis sinensis]	5,00E-13
gALL_v3_c17590	1	4	-	-	-	-	-
gALL_v3_c17591	2	5	gnl|CDD|148369	pfam06726, BC10, Bladder car	gb|EFN60530.1|	Bladder cancer-associated protein [Camponotus floridanus] gb|EFZ13113.1| hypothetical protein SINV_07762 [Solenopsis invicta] gb|EGI60369.1| Bladder cancer-associat	6,00E-51
gALL_v3_c17592	5	4	-	-	-	-	-
gALL_v3_c17593	0	4	gnl|CDD|29010	cd00110, LamG, Laminin G do	ref|XP_003400011.1|	PREDICTED: agrin-like [Bombus terrestris]	2,00E-40
gALL_v3_c17594	1	5	-	-	-	-	-
gALL_v3_c17595	2	3	-	-	ref|XP_002427998.1|	tight junction protein tama, putative [Pediculus humanus corporis] gb|EEB15260.1| tight junction protein tama, putative [Pediculus humanus corporis]	5,00E-51
gALL_v3_c17597	4	12	gnl|CDD|128336	smart00020, Tryp_SPc, Trypsir	ref|XP_003486297.1|	PREDICTED: ectopic P granules protein 5 homolog [Bombus impatiens]	2,00E-71
gALL_v3_c17598	0	4	-	-	gb|EHJ75938.1|	putative reverse transcriptase [Danaus plexippus]	7,00E-08
gALL_v3_c17599	2	3	-	-	ref|XP_004922853.1|	PREDICTED: torso-like protein-like [Bombyx mori]	3,00E-31
gALL_v3_c176	4	8	gnl|CDD|144027	pfam00287, Na_K-ATPase, Soc	emb|CCH80664.1|	Na/K-ATPase subunit beta 2 [Blattella germanica]	1,00E-58
gALL_v3_c1760	11	11	-	-	ref|XP_002426626.1|	hypothetical protein Phum_PHUM263920 [Pediculus humanus corporis] gb|EEB13888.1| hypothetical protein Phum_PHUM263920 [Pediculus humanus corporis]	2,00E-69
gALL_v3_c17600	2	3	-	-	-	-	-
gALL_v3_c17601	4	2	gnl|CDD|29257	cd00200, WD40, WD40 domai	ref|XP_969864.1|	PREDICTED: similar to AGAP006264-PA [Tribolium castaneum] gb|EFA09712.1| hypothetical protein TcasGA2_TC011845 [Tribolium castaneum]	2,00E-89
gALL_v3_c17603	1	4	-	-	ref|XP_001810838.1|	PREDICTED: similar to AGAP005037-PB [Tribolium castaneum]	6,00E-22
gALL_v3_c17604	1	3	-	-	-	-	-
gALL_v3_c17605	4	5	-	-	-	-	-
gALL_v3_c17606	2	2	-	-	-	-	-
gALL_v3_c17607	3	3	-	-	-	-	-
gALL_v3_c17608	2	2	-	-	-	-	-
gALL_v3_c17609	4	2	-	-	-	-	-
gALL_v3_c1761	7	6	-	-	-	-	-
gALL_v3_c17612	3	2	-	-	-	-	-
gALL_v3_c17613	3	2	-	-	-	-	-
gALL_v3_c17614	3	2	-	-	-	-	-
gALL_v3_c17615	0	4	-	-	-	-	-
gALL_v3_c17617	1	3	-	-	emb|CAA52847.1|	zinc finger protein 51 [Mus musculus]	1,00E-10
gALL_v3_c17618	2	2	-	-	ref|XP_006034374.1|	PREDICTED: tigger transposable element-derived protein 1-like [Alligator sinensis]	3,00E-19
gALL_v3_c17619	3	1	gnl|CDD|163652	cd07409, MPP_CD73_N, CD73	gb|EFN81716.1|	Apyrase [Harpegnathos saltator]	3,00E-54
gALL_v3_c1762	23	21	gnl|CDD|190744	pfam03770, IPK, Inositol polyp	ref|XP_003424457.1|	PREDICTED: hypothetical protein LOC100679103 [Nasonia vitripennis]	1,00E-131
gALL_v3_c17620	2	2	-	-	-	-	-
gALL_v3_c17621	1	3	-	-	-	-	-
gALL_v3_c17622	5	2	-	-	-	-	-
gALL_v3_c17623	2	8	-	-	-	-	-
gALL_v3_c17624	7	7	-	-	ref|XP_006616058.1|	PREDICTED: activating transcription factor of chaperone-like isoform X2 [Apis dorsata]	5,00E-08
gALL_v3_c17626	1	5	-	-	-	-	-
gALL_v3_c17627	2	3	-	-	-	-	-
gALL_v3_c17629	3	3	-	-	-	-	-
gALL_v3_c17630	0	5	-	-	-	-	-
gALL_v3_c17631	2	5	gnl|CDD|153252	cd07990, LPLAT_LCLAT1-like, L	ref|XP_002425983.1|	conserved hypothetical protein [Pediculus humanus corporis] gb|EEB13245.1| conserved hypothetical protein [Pediculus humanus corporis]	1,00E-110
gALL_v3_c17632	2	7	-	-	-	-	-
gALL_v3_c17634	4	3	-	-	-	-	-
gALL_v3_c17635	2	9	-	-	-	-	-
gALL_v3_c17637	4	6	-	-	-	-	-
gALL_v3_c17638	3	5	-	-	-	-	-
gALL_v3_c17639	1	4	-	-	-	-	-
gALL_v3_c1764	17	25	gnl|CDD|31389	COG1196, Smc, Chromosome	ref|XP_002424549.1|	conserved hypothetical protein [Pediculus humanus corporis] gb|EEB11811.1| conserved hypothetical protein [Pediculus humanus corporis]	4,00E-86
gALL_v3_c17641	3	3	-	-	-	-	-
gALL_v3_c17642	3	3	-	-	-	-	-
gALL_v3_c17643	3	2	-	-	-	-	-
gALL_v3_c17644	4	3	gnl|CDD|48355	cd00935, GlyRS_RNA, GlyRS_F	ref|XP_001606827.1|	PREDICTED: glycyl-tRNA synthetase-like [Nasonia vitripennis]	7,00E-64
gALL_v3_c17645	2	4	-	-	ref|XP_006562212.1|	PREDICTED: histone-lysine N-methyltransferase, H3 lysine-79 specific-like isoform X2 [Apis mellifera]	7,00E-06
gALL_v3_c17647	4	9	gnl|CDD|146221	pfam03465, eRF1_3, eRF1 dor	ref|XP_967126.1|	PREDICTED: similar to pelota [Tribolium castaneum] gb|EFA12200.1| pelota [Tribolium castaneum]	5,00E-38
gALL_v3_c17648	3	1	-	-	-	-	-
gALL_v3_c1765	10	8	-	-	-	-	-
gALL_v3_c17650	6	3	-	-	-	-	-
gALL_v3_c17651	2	4	gnl|CDD|177220	MTH00165, ND5, NADH dehyc	gb|AGM32507.1|	NADH dehydrogenase subunit 5, partial (mitochondrion) [Coptotermes formosanus]	2,00E-56
gALL_v3_c17652	5	4	-	-	gb|EFZ14976.1|	hypothetical protein SINV_01968 [Solenopsis invicta]	1,00E-08
gALL_v3_c17653	3	2	-	-	-	-	-
gALL_v3_c17654	2	10	-	-	-	-	-
gALL_v3_c17655	3	2	-	-	-	-	-
gALL_v3_c17656	2	2	-	-	-	-	-
gALL_v3_c17657	7	9	gnl|CDD|192301	pfam09468, RNase_H2-Ydr279	ref|XP_968060.1|	PREDICTED: similar to predicted protein [Tribolium castaneum] gb|EFA04271.1| hypothetical protein TcasGA2_TC014561 [Tribolium castaneum]	1,00E-106
gALL_v3_c17658	2	2	-	-	-	-	-
gALL_v3_c17659	5	4	gnl|CDD|128471	smart00174, RHO, Rho (Ras hc	ref|XP_004923971.1|	PREDICTED: rho-related GTP-binding protein RhoN-like [Bombyx mori]	1,00E-36
gALL_v3_c1766	4	11	gnl|CDD|29115	cd00170, SEC14, Sec14p-like li	ref|NP_001245486.1|	CG3191, isoform B [Drosophila melanogaster] gb|AFH07200.1| CG3191, isoform B [Drosophila melanogaster]	2,00E-13
gALL_v3_c17660	3	1	-	-	-	-	-
gALL_v3_c17661	4	3	gnl|CDD|191773	pfam07531, TAFH, NHR1 homo	ref|XP_006565564.1|	PREDICTED: LOW QUALITY PROTEIN: transcription initiation factor TFIID subunit 4-like [Apis mellifera]	9,00E-55
gALL_v3_c17662	0	4	-	-	ref|XP_007006356.1|	hypothetical protein TREMEDRAFT_33303, partial [Tremella mesenterica DSM 1558] ref|XP_007006383.1| hypothetical protein TREMEDRAFT_33474, partial [Tremella me	3,00E-12
gALL_v3_c17663	1	3	-	-	-	-	-
gALL_v3_c17664	2	3	-	-	-	-	-
gALL_v3_c17665	6	5	-	-	-	-	-
gALL_v3_c17667	4	4	-	-	-	-	-
gALL_v3_c17668	3	3	-	-	-	-	-
gALL_v3_c17669	0	4	gnl|CDD|176723	cd08307, Death_Pelle, Death (	ref|XP_003698234.1|	PREDICTED: LOW QUALITY PROTEIN: serine/threonine-protein kinase pelle-like [Apis florea]	1,00E-33
gALL_v3_c1767	7	5	-	-	-	-	-
gALL_v3_c17670	3	5	-	-	-	-	-
gALL_v3_c17672	1	3	-	-	-	-	-
gALL_v3_c17673	3	1	-	-	-	-	-
gALL_v3_c17674	1	4	-	-	gb|EGI66238.1|	Splicing factor 3B subunit 3 [Acromyrmex echinatior]	5,00E-73
gALL_v3_c17675	0	5	-	-	ref|XP_003201575.2|	PREDICTED: oocyte zinc finger protein XlCOF6-like [Danio rerio]	9,00E-34
gALL_v3_c17676	3	2	gnl|CDD|34495	COG4886, COG4886, Leucine-r	ref|XP_971675.1|	PREDICTED: similar to leucine rich repeat containing 59 [Tribolium castaneum] gb|EFA00165.1| hypothetical protein TcasGA2_TC002987 [Tribolium castaneum]	1,00E-44
gALL_v3_c17677	1	5	-	-	ref|XP_002427046.1|	predicted protein [Pediculus humanus corporis] gb|EEB14308.1| predicted protein [Pediculus humanus corporis]	2,00E-17
gALL_v3_c17678	4	4	-	-	gb|EFZ10687.1|	hypothetical protein SINV_12257 [Solenopsis invicta]	7,00E-45
gALL_v3_c17679	4	3	-	-	-	-	-
gALL_v3_c1768	5	6	-	-	-	-	-
gALL_v3_c17680	2	3	-	-	-	-	-
gALL_v3_c17681	2	3	-	-	-	-	-

gALL_v3_c17682	0	5	-	-	-	-	-
gALL_v3_c17683	2	2	gnl|CDD|189372	pfam00067, p450, Cytochrom	sp|P29981.1|CP4C1_BL	RecName: Full=Cytochrome P450 4C1; AltName: Full=CYPIVC1 gb|AAA27819.1| cytochrome P450 [Blaberus discoidalis]	1,00E-49
gALL_v3_c17685	3	4	-	-	-	-	-
gALL_v3_c17686	1	4	-	-	-	-	-
gALL_v3_c17687	4	8	gnl|CDD|133263	cd01860, Rab5_related, Rab5-	gb|ETN65323.1|	low-Mr GTP-binding protein Rab31 [Anopheles darlingi]	6,00E-09
gALL_v3_c17689	3	3	-	-	-	-	-
gALL_v3_c17690	5	2	-	-	ref|XP_001658941.1|	hypothetical protein AaeL_AAEL000160 [Aedes aegypti] gb|EAT48833.1| AAEL000160-PA [Aedes aegypti]	3,00E-06
gALL_v3_c17691	5	13	-	-	-	-	-
gALL_v3_c17692	1	4	-	-	-	-	-
gALL_v3_c17693	3	3	gnl|CDD|110172	pfam01150, GDA1_CD39, GDA	ref|XP_001948332.1|	PREDICTED: ectonucleoside triphosphate diphosphohydrolase 5-like isoform 1 [Acyrthosiphon pisum] ref|XP_003242584.1| PREDICTED: ectonucleoside triphosphate diph	1,00E-45
gALL_v3_c17694	5	2	-	-	-	-	-
gALL_v3_c17695	1	5	-	-	-	-	-
gALL_v3_c17697	4	3	-	-	gb|EGI62187.1|	Centrosomal protein of 164 kDa [Acromyrmex echinatior]	3,00E-06
gALL_v3_c17698	3	2	gnl|CDD|146527	pfam03942, DTW, DTW domai	ref|XP_004929624.1|	PREDICTED: DTW domain-containing protein 2-like [Bombyx mori]	3,00E-46
gALL_v3_c17699	4	8	-	-	-	-	-
gALL_v3_c17700	1	4	-	-	-	-	-
gALL_v3_c17701	2	3	-	-	-	-	-
gALL_v3_c17702	5	7	-	-	-	-	-
gALL_v3_c17703	3	2	-	-	ref|XP_003737428.1|	PREDICTED: tigger transposable element-derived protein 4-like [Metaseiulus occidentalis]	7,00E-08
gALL_v3_c17704	3	3	-	-	ref|XP_006617068.1|	PREDICTED: ATP-dependent RNA helicase abstrakt-like [Apis dorsata]	3,00E-66
gALL_v3_c17705	4	2	-	-	gb|EFN84335.1|	Nucleosome-remodeling factor subunit NURF301 [Harpegnathos saltator]	4,00E-14
gALL_v3_c17706	8	12	gnl|CDD|176293	cd01217, CG12581, CG12581	ref|XP_001946893.2|	PREDICTED: hypothetical protein LOC100164379 [Acyrthosiphon pisum]	1,00E-119
gALL_v3_c17707	2	2	gnl|CDD|143448	cd07130, ALDH_F7_AASADH,	gb|AGM32640.1|	aldehyde dehydrogenase 7 family, member A1-like protein, partial [Coptotermes formosanus]	3,00E-25
gALL_v3_c17708	1	3	-	-	-	-	-
gALL_v3_c17709	1	3	-	-	-	-	-
gALL_v3_c1771	5	7	-	-	-	-	-
gALL_v3_c17710	1	4	-	-	dbj|BAO01090.1|	neuropeptide GPCR A40 [Nilaparvata lugens]	2,00E-63
gALL_v3_c17711	3	2	gnl|CDD|191372	pfam05781, MRVI1, MRVI1 pr	gb|ELT90013.1|	hypothetical protein CAPTEDRAFT_225487 [Capitella teleta]	1,00E-20
gALL_v3_c17714	5	4	-	-	-	-	-
gALL_v3_c17715	5	4	gnl|CDD|29394	cd01923, cyclophilin_RING, cy	ref|XP_003707279.1|	PREDICTED: peptidyl-prolyl cis-trans isomerase-like 2-like [Megachile rotundata]	1,00E-144
gALL_v3_c17716	7	4	gnl|CDD|190487	pfam02953, zf-Tim10_DDP, Tir	gb|ENN74758.1|	hypothetical protein YQE_08666, partial [Dendroctonus ponderosae]	7,00E-28
gALL_v3_c17717	4	2	-	-	-	-	-
gALL_v3_c17718	1	3	-	-	-	-	-
gALL_v3_c1772	11	18	gnl|CDD|34673	COG5069, SAC6, Ca2+-binding	ref|XP_003397111.1|	PREDICTED: spectrin beta chain, brain 4-like isoform 2 [Bombus terrestris]	0
gALL_v3_c17721	2	7	-	-	-	-	-
gALL_v3_c17722	4	5	-	-	-	-	-
gALL_v3_c17723	2	2	-	-	-	-	-
gALL_v3_c17724	4	4	-	-	-	-	-
gALL_v3_c17726	3	4	-	-	-	-	-
gALL_v3_c17727	0	4	-	-	-	-	-
gALL_v3_c17728	1	4	-	-	-	-	-
gALL_v3_c17729	3	4	gnl|CDD|184178	PRK13607, PRK13607, proline	gb|AGM32934.1|	vacuolar protein sorting-associated 29-like protein [Coptotermes formosanus]	1,00E-65
gALL_v3_c1773	4	11	-	-	ref|XP_001604408.1|	PREDICTED: hypothetical protein LOC100120806 [Nasonia vitripennis]	3,00E-06
gALL_v3_c17730	6	9	gnl|CDD|143169	cd04968, Ig3_Contactin_like, T	sp|Q26474.1|LACH_SCH	RecName: Full=Lachesin; Flags: Precursor gb|AAC37185.1| lachesin [Schistocerca americana]	1,00E-161
gALL_v3_c17731	3	2	-	-	ref|XP_005466388.1|	PREDICTED: zinc finger protein 347-like, partial [Oreochromis niloticus]	5,00E-22
gALL_v3_c17732	3	1	-	-	-	-	-
gALL_v3_c17733	2	2	-	-	-	-	-
gALL_v3_c17734	0	5	-	-	gb|EFN89488.1|	Multiple inositol polyphosphate phosphatase 1 [Harpegnathos saltator]	7,00E-21
gALL_v3_c17735	0	4	-	-	-	-	-
gALL_v3_c17736	1	5	gnl|CDD|192495	pfam10250, O-FucT, GDP-fuco	ref|XP_002424451.1|	GDP-fucose protein O-fucosyltransferase 2 precursor, putative [Pediculus humanus corporis] gb|EEB11713.1| GDP-fucose protein O-fucosyltransferase 2 precursor, putative	9,00E-54
gALL_v3_c17738	4	4	-	-	-	-	-
gALL_v3_c17739	4	2	-	-	-	-	-
gALL_v3_c17740	2	2	gnl|CDD|110072	pfam01044, Vinculin, Vinculin	ref|XP_001944375.1|	PREDICTED: catenin alpha-like [Acyrthosiphon pisum]	7,00E-24
gALL_v3_c17743	2	2	-	-	ref|XP_004930364.1|	PREDICTED: uncharacterized protein LOC101741169 [Bombyx mori]	5,00E-43
gALL_v3_c17744	6	6	-	-	-	-	-
gALL_v3_c17745	1	6	gnl|CDD|191221	pfam05185, PRMT5, PRMT5 a	ref|XP_002427707.1|	protein arginine N-methyltransferase, putative [Pediculus humanus corporis] gb|EEB14969.1| protein arginine N-methyltransferase, putative [Pediculus humanus corporis]	8,00E-61
gALL_v3_c17746	2	3	-	-	gb|EHJ78197.1|	hypothetical protein KGM_18799 [Danaus plexippus]	4,00E-07
gALL_v3_c17747	3	3	-	-	ref|XP_001945130.2|	PREDICTED: prickle-like protein 3-like [Acyrthosiphon pisum]	7,00E-19
gALL_v3_c17748	3	2	-	-	-	-	-
gALL_v3_c17749	0	5	-	-	-	-	-
gALL_v3_c1775	6	13	gnl|CDD|173663	cd05572, STKc_cGK_PKG, Cata	gb|ERL94983.1|	hypothetical protein D910_12255 [Dendroctonus ponderosae]	1,00E-168
gALL_v3_c17750	4	3	gnl|CDD|29261	cd00204, ANK, ankyrin repeat	ref|XP_006560850.1|	PREDICTED: ankyrin repeat domain-containing protein 50 isoform X1 [Apis mellifera]	3,00E-64
gALL_v3_c17753	2	4	gnl|CDD|151756	pfam11315, Med30, Mediator	gb|EFN69141.1|	Mediator of RNA polymerase II transcription subunit 30 [Camponotus floridanus]	4,00E-29
gALL_v3_c17754	3	1	-	-	-	-	-
gALL_v3_c17755	1	3	-	-	-	-	-
gALL_v3_c17756	10	6	-	-	gb|EFN65619.1|	Putative uncharacterized transposon-derived protein F52C9.6 [Camponotus floridanus]	5,00E-08
gALL_v3_c17757	3	1	-	-	-	-	-
gALL_v3_c17758	6	16	-	-	-	-	-
gALL_v3_c17759	0	5	gnl|CDD|176553	cd08612, GDPD_GDE4, Glycer	ref|XP_003706147.1|	PREDICTED: glycerophosphodiester phosphodiesterase domain-containing protein 1-like [Megachile rotundata]	4,00E-66
gALL_v3_c1776	6	6	-	-	ref|WP_002412625.1|	hypothetical protein [Enterococcus faecalis] gb|EGG54264.1| hypothetical protein HMPREF9520_02283 [Enterococcus faecalis TX1467]	4,00E-50
gALL_v3_c17760	1	3	-	-	ref|XP_001662979.1|	hypothetical protein AaeL_AAEL003019 [Aedes aegypti] gb|EAT45709.1| AAEL003019-PA [Aedes aegypti]	8,00E-14
gALL_v3_c17761	1	5	-	-	ref|XP_001607590.1|	PREDICTED: importin-5-like [Nasonia vitripennis]	4,00E-78
gALL_v3_c17763	1	5	-	-	-	-	-
gALL_v3_c17764	2	3	-	-	-	-	-
gALL_v3_c17765	1	4	-	-	gb|EGI59011.1|	Coiled-coil domain-containing protein [Acromyrmex echinatior]	2,00E-44
gALL_v3_c17766	4	3	-	-	-	-	-
gALL_v3_c17768	3	2	-	-	-	-	-
gALL_v3_c17769	4	3	-	-	-	-	-
gALL_v3_c17770	1	4	-	-	-	-	-
gALL_v3_c17771	2	2	-	-	-	-	-
gALL_v3_c17772	4	7	-	-	ref|XP_004926839.1|	PREDICTED: globin, polymeric component P3-like [Bombyx mori]	1,00E-57
gALL_v3_c17773	0	5	-	-	-	-	-
gALL_v3_c17774	2	3	gnl|CDD|192354	pfam09735, Nckap1, Membra	ref|XP_006608131.1|	PREDICTED: membrane-associated protein Hem-like [Apis dorsata]	5,00E-91
gALL_v3_c17775	2	4	-	-	-	-	-
gALL_v3_c17776	2	4	-	-	-	-	-
gALL_v3_c17777	1	3	-	-	-	-	-
gALL_v3_c17778	0	4	-	-	-	-	-
gALL_v3_c1778	8	9	gnl|CDD|147578	pfam05470, eIF-3c_N, Eukaryo	ref|XP_003701177.1|	PREDICTED: eukaryotic translation initiation factor 3 subunit C-like [Megachile rotundata]	0
gALL_v3_c17781	5	2	-	-	-	-	-
gALL_v3_c17782	6	3	-	-	-	-	-
gALL_v3_c17783	0	4	-	-	-	-	-
gALL_v3_c17784	1	3	-	-	-	-	-
gALL_v3_c17785	4	5	gnl|CDD|128591	smart00295, B41, Band 4.1 ho	ref|XP_003701791.1|	PREDICTED: moesin/ezrin/radixin homolog 1-like isoform 1 [Megachile rotundata]	6,00E-69
gALL_v3_c17786	5	9	gnl|CDD|30768	COG0419, SbcC, ATPase involv	ref|XP_001459852.1|	hypothetical protein [Paramecium tetraurelia strain d4-2] emb|CAK92455.1| unnamed protein product [Paramecium tetraurelia]	1,00E-09
gALL_v3_c17787	4	3	-	-	-	-	-
gALL_v3_c17788	1	5	-	-	ref|XP_003698333.1|	PREDICTED: protein SZT2 [Apis florea]	3,00E-30
gALL_v3_c17789	3	9	gnl|CDD|147872	pfam05955, Herpes_gp2, Equi	-	-	-
gALL_v3_c1779	12	14	gnl|CDD|143864	pfam00083, Sugar_tr, Sugar (a	ref|XP_002427227.1|	conserved hypothetical protein [Pediculus humanus corporis] gb|EEB14489.1| conserved hypothetical protein [Pediculus humanus corporis]	5,00E-51
gALL_v3_c17790	4	5	gnl|CDD|128632	smart00338, BRLZ, basic regio	ref|XP_005177209.1|	PREDICTED: cyclic AMP response element-binding protein A-like [Musca domestica]	2,00E-11
gALL_v3_c17791	0	4	-	-	-	-	-
gALL_v3_c17792	2	4	-	-	-	-	-
gALL_v3_c17793	1	7	-	-	-	-	-
gALL_v3_c17794	6	4	-	-	-	-	-
gALL_v3_c17795	3	5	-	-	-	-	-
gALL_v3_c17796	0	5	gnl|CDD|185504	PTZ00184, PTZ00184, calmodu	ref|XP_006558598.1|	PREDICTED: calmodulin-like isoform X5 [Apis mellifera]	1,00E-134
gALL_v3_c17797	4	4	-	-	ref|XP_003704390.1|	PREDICTED: uncharacterized protein LOC100883782 [Megachile rotundata]	1,00E-57
gALL_v3_c17798	3	1	-	-	-	-	-
gALL_v3_c17799	3	6	-	-	-	-	-
gALL_v3_c178	5	11	-	-	-	-	-
gALL_v3_c1780	7	9	-	-	gb|AGM32456.1|	zinc metalloprotease-like protein [Coptotermes formosanus]	1,00E-23
gALL_v3_c17801	0	4	-	-	-	-	-
gALL_v3_c17803	0	5	-	-	-	-	-
gALL_v3_c17804	0	4	-	-	-	-	-
gALL_v3_c17805	3	3	-	-	-	-	-
gALL_v3_c17806	1	5	-	-	gb|EGI62409.1|	RILP-like protein-like protein [Acromyrmex echinatior] gb|EGI62411.1| RILP-like protein-like protein [Acromyrmex echinatior]	3,00E-60
gALL_v3_c17808	2	4	-	-	-	-	-
gALL_v3_c17809	3	3	-	-	-	-	-
gALL_v3_c1781	5	4	-	-	-	-	-
gALL_v3_c17810	8	4	-	-	ref|WP_006330213.1|	ribonuclease E [Gordonia rhizosphera] dbj|GAB88631.1| putative ribonuclease [Gordonia rhizosphera NBRC 16068]	7,00E-12
gALL_v3_c17813	4	5	-	-	-	-	-
gALL_v3_c17814	2	1	-	-	-	-	-
gALL_v3_c17815	7	4	-	-	ref|XP_003218078.1|	PREDICTED: hypothetical protein LOC100562870 [Anolis carolinensis]	8,00E-12
gALL_v3_c17816	10	11	-	-	dbj|BAJ11762.1|	HsjCib isoform4 [Hodotermopsis sjostedti]	4,00E-64
gALL_v3_c17817	4	8	gnl|CDD|48538	cd02989, Phd_like_TxnDC9, Pl	ref|XP_001649641.1|	viral IAP-associated factor, putative [Aedes aegypti] ref|XP_001660871.1| viral IAP-associated factor, putative [Aedes aegypti] gb|EAT32950.1| AAEL014813-PA [Aedes aeg	5,00E-57
gALL_v3_c17818	1	3	-	-	-	-	-
gALL_v3_c17819	1	8	-	-	-	-	-
gALL_v3_c17821	2	5	-	-	-	-	-
gALL_v3_c17823	3	1	-	-	-	-	-
gALL_v3_c17824	2	2	-	-	-	-	-
gALL_v3_c17825	2	4	-	-	-	-	-
gALL_v3_c17826	1	6	-	-	gb|EFA00151.1|	hypothetical protein TcasGA2_TC002972 [Tribolium castaneum]	1,00E-57
gALL_v3_c17827	1	4	-	-	-	-	-
gALL_v3_c17828	3	3	-	-	-	-	-
gALL_v3_c17829	8	8	-	-	-	-	-
gALL_v3_c1783	9	7	gnl|CDD|111665	pfam02792, Mago_nashi, Mag	ref|XP_001608006.1|	PREDICTED: protein mago nashi homolog 2-like [Nasonia vitripennis]	2,00E-94
gALL_v3_c17830	0	5	-	-	ref|XP_001657664.1|	hypothetical protein AaeL_AAEL000123 [Aedes aegypti] gb|EAT48855.1| AAEL000123-PA [Aedes aegypti]	1,00E-20
gALL_v3_c17831	1	4	-	-	-	-	-
gALL_v3_c17832	4	4	-	-	-	-	-
gALL_v3_c17833	1	4	gnl|CDD|29261	cd00204, ANK, ankyrin repeat	gb|AGM32371.1|	ankyrin repeat protein [Coptotermes formosanus]	3,00E-56
gALL_v3_c17835	7	4	-	-	gb|AGM32950.1|	prenylated Rab acceptor protein [Coptotermes formosanus]	9,00E-20
gALL_v3_c17836	5	3	-	-	ref|XP_003228154.1|	PREDICTED: jerky protein homolog-like [Anolis carolinensis]	3,00E-25
gALL_v3_c17837	5	3	-	-	-	-	-
gALL_v3_c17839	4	2	-	-	-	-	-
gALL_v3_c1784	22	23	-	-	ref|XP_001951939.2|	PREDICTED: hypothetical protein LOC100163229 [Acyrthosiphon pisum]	9,00E-10

gALL_v3_c17841	2	3	-	-	-	-	-
gALL_v3_c17842	1	5	-	-	-	-	-
gALL_v3_c17843	2	3	-	-	-	-	-
gALL_v3_c17844	3	3	-	-	-	-	-
gALL_v3_c17845	2	2	-	-	-	-	-
gALL_v3_c17846	3	8	-	-	-	-	-
gALL_v3_c17847	1	6	gnl|CDD|173674	cd05583, STKc_MSK_N, N-terr	ref|XP_006559725.1|	PREDICTED: ribosomal protein S6 kinase alpha-5 isoform X3 [Apis mellifera]	1,00E-63
gALL_v3_c17848	1	3	-	-	-	-	-
gALL_v3_c17849	2	5	-	-	-	-	-
gALL_v3_c1785	8	8	gnl|CDD|175988	cd04021, C2_E3_ubiquitin_lig	gb|EFN88071.1|	E3 ubiquitin-protein ligase suppressor of deltex [Harpegnathos saltator]	1,00E-68
gALL_v3_c17850	7	7	-	-	-	-	-
gALL_v3_c17852	0	3	-	-	-	-	-
gALL_v3_c17853	1	5	-	-	-	-	-
gALL_v3_c17854	1	3	-	-	-	-	-
gALL_v3_c17855	0	3	gnl|CDD|150479	pfam09815, XK-related, XK-rel	gb|EHJ65841.1|	hypothetical protein KGM_08531 [Danaus plexippus]	2,00E-19
gALL_v3_c17856	3	1	-	-	-	-	-
gALL_v3_c17857	1	3	-	-	ref|XP_002423712.1|	trithorax group protein osa, putative [Pediculus humanus corporis] gb|EEB10974.1| trithorax group protein osa, putative [Pediculus humanus corporis]	1,00E-12
gALL_v3_c17858	2	5	-	-	-	-	-
gALL_v3_c17859	0	6	-	-	-	-	-
gALL_v3_c1786	19	16	gnl|CDD|133421	cd01336, MDH_cytoplasmic_c	gb|AHB50500.1|	malate dehydrogenase 1 [Mayetiola destructor]	4,00E-06
gALL_v3_c17860	1	3	-	-	-	-	-
gALL_v3_c17861	2	5	-	-	-	-	-
gALL_v3_c17862	3	6	gnl|CDD|28959	cd00078, HECTc, HECT domain	ref|XP_002424807.1|	hect E3 ubiquitin ligase, putative [Pediculus humanus corporis] gb|EEB12069.1| hect E3 ubiquitin ligase, putative [Pediculus humanus corporis]	4,00E-86
gALL_v3_c17863	5	3	-	-	-	-	-
gALL_v3_c17864	3	6	-	-	-	-	-
gALL_v3_c17865	3	3	-	-	-	-	-
gALL_v3_c17866	2	3	-	-	gb|EFN87249.1|	Ankyrin repeat and fibronectin type-III domain-containing protein 1 [Harpegnathos saltator]	2,00E-25
gALL_v3_c17867	3	4	-	-	-	-	-
gALL_v3_c1787	7	5	gnl|CDD|189450	pfam00209, SNF, Sodium:neur	ref|XP_002427108.1|	sodium-dependent nutrient amino acid transporter, putative [Pediculus humanus corporis] gb|EEB14370.1| sodium-dependent nutrient amino acid transporter, putative [	1,00E-100
gALL_v3_c17872	3	1	gnl|CDD|185403	PTZ00027, PTZ00027, 60S ribo	ref|XP_001294322.1|	ribosomal protein L6 [Trichomonas vaginalis G3] gb|EAX81392.1| ribosomal protein L6, putative [Trichomonas vaginalis G3]	1,00E-54
gALL_v3_c17873	2	4	gnl|CDD|29257	cd00200, WD40, WD40 domai	gb|ETN59784.1|	wd-repeat protein [Anopheles darlingi]	1,00E-131
gALL_v3_c17875	3	10	gnl|CDD|189860	pfam01151, ELO, GNS1/SUR4	ref|XP_002428457.1|	elongation of very long chain fatty acids protein, putative [Pediculus humanus corporis] gb|EEB15719.1| elongation of very long chain fatty acids protein, putative [Pediculi	1,00E-26
gALL_v3_c17877	2	3	gnl|CDD|128440	smart00135, LY, Low-density li	emb|CAG10095.1|	unnamed protein product [Tetraodon nigroviridis]	5,00E-35
gALL_v3_c17878	7	12	gnl|CDD|189372	pfam00067, p450, Cytochrom	gb|EEZ97687.1|	hypothetical protein TcasGA2_TC016235 [Tribolium castaneum]	2,00E-56
gALL_v3_c17879	2	4	-	-	ref|XP_001601330.1|	PREDICTED: nicastrin-like [Nasonia vitripennis]	2,00E-44
gALL_v3_c1788	9	8	gnl|CDD|48092	cd03388, PAP2_SPPase1, PAP2	gb|EFA10076.1|	hypothetical protein TcasGA2_TC012252 [Tribolium castaneum]	1,00E-132
gALL_v3_c17882	0	6	-	-	-	-	-
gALL_v3_c17883	2	4	gnl|CDD|189334	pfam00001, 7tm_1, 7 transme	gb|AFA55167.1|	octopamine/tyramine receptor, partial [Trichoplusia ni]	3,00E-41
gALL_v3_c17884	1	5	-	-	-	-	-
gALL_v3_c17885	2	4	gnl|CDD|151718	pfam11277, Med24_N, Media	gb|EFN83518.1|	Mediator of RNA polymerase II transcription subunit 24 [Harpegnathos saltator]	1,00E-112
gALL_v3_c17886	5	2	-	-	-	-	-
gALL_v3_c17887	4	3	-	-	-	-	-
gALL_v3_c17888	7	7	-	-	-	-	-
gALL_v3_c17889	5	4	-	-	-	-	-
gALL_v3_c1789	13	17	gnl|CDD|29029	cd00127, DSPc, Dual specificity	gb|ADD38746.1|	Dual specificity protein phosphatase 3 [Lepeophtheirus salmonis]	7,00E-29
gALL_v3_c17890	3	2	-	-	-	-	-
gALL_v3_c17892	3	2	-	-	-	-	-
gALL_v3_c17893	1	3	gnl|CDD|73156	cd01650, RT_nLTR_like, RT_nL	dbj|BAC82617.1|	pol-like protein [Danio rerio]	6,00E-19
gALL_v3_c17895	2	3	-	-	-	-	-
gALL_v3_c17896	2	3	-	-	ref|XP_001601754.2|	PREDICTED: LOW QUALITY PROTEIN: baculoviral IAP repeat-containing protein 6 [Nasonia vitripennis]	3,00E-51
gALL_v3_c17897	2	2	-	-	-	-	-
gALL_v3_c17898	2	2	-	-	-	-	-
gALL_v3_c179	7	8	gnl|CDD|177880	PLN02236, PLN02236, choline	ref|XP_001603632.1|	PREDICTED: choline/ethanolamine kinase-like [Nasonia vitripennis]	1,00E-15
gALL_v3_c1790	12	16	gnl|CDD|48155	cd03338, TCP1_delta, TCP-1 (C	gb|AGM32355.1|	T-complex protein 1 subunit delta [Coptotermes formosanus]	0
gALL_v3_c17900	0	4	-	-	-	-	-
gALL_v3_c17901	3	3	-	-	-	-	-
gALL_v3_c17902	2	5	-	-	-	-	-
gALL_v3_c17903	1	3	-	-	-	-	-
gALL_v3_c17904	3	3	-	-	ref|XP_002413021.1|	endonuclease/reverse transcriptase, putative [Ixodes scapularis] gb|EEC16329.1| endonuclease/reverse transcriptase, putative [Ixodes scapularis]	4,00E-10
gALL_v3_c17905	2	4	gnl|CDD|162369	TIGR01452, PGP_euk, phosph	ref|XP_003396203.1|	PREDICTED: LOW QUALITY PROTEIN: phosphoglycolate phosphatase-like [Bombus terrestris]	3,00E-20
gALL_v3_c17906	2	2	-	-	-	-	-
gALL_v3_c17907	6	3	-	-	ref|YP_007885428.1|	Ankyrin repeat domain protein [Wolbachia endosymbiont of Drosophila simulans wNo] ref|WP_015587727.1| Ankyrin repeat domain protein [Wolbachia endosymbiont o	5,00E-06
gALL_v3_c17908	3	1	-	-	-	-	-
gALL_v3_c17909	3	2	-	-	ref|NP_001130003.1|	Fgfr1 oncogene partner [Nasonia vitripennis]	1,00E-23
gALL_v3_c17910	4	3	-	-	ref|XP_003693039.1|	PREDICTED: LOW QUALITY PROTEIN: small subunit processome component 20 homolog [Apis florea]	2,00E-13
gALL_v3_c17911	4	2	-	-	-	-	-
gALL_v3_c17912	8	10	gnl|CDD|189783	pfam00956, NAP, Nucleosome	ref|XP_003484877.1|	PREDICTED: nucleosome assembly protein 1-like 1-like [Bombus impatiens]	1,00E-102
gALL_v3_c17913	3	5	gnl|CDD|189390	pfam00102, Y_phosphatase, P	ref|XP_002424545.1|	conserved hypothetical protein [Pediculus humanus corporis] gb|EEB11807.1| conserved hypothetical protein [Pediculus humanus corporis]	1,00E-40
gALL_v3_c17914	3	4	-	-	ref|XP_002426824.1|	predicted protein [Pediculus humanus corporis] gb|EEB14086.1| predicted protein [Pediculus humanus corporis]	4,00E-86
gALL_v3_c17915	3	2	-	-	ref|XP_001600931.2|	PREDICTED: hypothetical protein LOC100116429 [Nasonia vitripennis]	5,00E-10
gALL_v3_c17916	2	3	gnl|CDD|133179	cd05048, PTKc_Ror, Catalytic D	ref|XP_002430799.1|	tyrosine-protein kinase transmembrane receptor ROR1 precursor, putative [Pediculus humanus corporis] gb|EEB18061.1| tyrosine-protein kinase transmembrane receptor	5,00E-87
gALL_v3_c17917	2	2	-	-	ref|XP_001599507.2|	PREDICTED: hypothetical protein LOC100114520 [Nasonia vitripennis]	2,00E-11
gALL_v3_c17918	2	2	-	-	-	-	-
gALL_v3_c17919	5	5	-	-	dbj|BAC11096.1|	unnamed protein product [Homo sapiens]	1,00E-41
gALL_v3_c1792	8	6	-	-	ref|XP_003492217.1|	PREDICTED: hypothetical protein LOC100740063 [Bombus impatiens]	9,00E-17
gALL_v3_c17920	5	3	gnl|CDD|31689	COG1500, COG1500, Predicted	gb|AGM32728.1|	Shwachman-Bodian-diamond syndrome (SBDS) C-terminal domain containing protein [Coptotermes formosanus]	1,00E-169
gALL_v3_c17921	2	4	gnl|CDD|30057	cd01314, D-HYD, D-hydantoina	gb|EKC20858.1|	Dihydropyrimidinase [Crassostrea gigas]	5,00E-70
gALL_v3_c17922	1	3	-	-	ref|XP_003690057.1|	PREDICTED: probable phospholipid-transporting ATPase IIB-like [Apis florea]	1,00E-17
gALL_v3_c17923	2	3	-	-	-	-	-
gALL_v3_c17924	26	27	gnl|CDD|147872	pfam05955, Herpes_gp2, Equi	-	-	-
gALL_v3_c17926	3	2	-	-	-	-	-
gALL_v3_c17927	2	4	-	-	gb|EFN67090.1|	CCR4-NOT transcription complex subunit 4 [Camponotus floridanus]	6,00E-06
gALL_v3_c17928	3	5	-	-	gb|AGM32905.1|	hypothetical protein [Coptotermes formosanus]	9,00E-09
gALL_v3_c17929	1	5	-	-	-	-	-
gALL_v3_c17930	4	3	gnl|CDD|176351	cd00153, RalGDS_RA, Ubiquiti	gb|EFA00851.1|	hypothetical protein TcasGA2_TC003754 [Tribolium castaneum]	1,00E-58
gALL_v3_c17932	2	2	-	-	-	-	-
gALL_v3_c17933	3	1	-	-	-	-	-
gALL_v3_c17935	2	3	-	-	-	-	-
gALL_v3_c17936	3	1	-	-	ref|XP_005104745.1|	PREDICTED: histone-lysine N-methyltransferase SETMAR-like [Aplysia californica]	4,00E-06
gALL_v3_c17937	1	4	-	-	-	-	-
gALL_v3_c17938	3	3	-	-	-	-	-
gALL_v3_c17939	4	5	gnl|CDD|190182	pfam01981, PTH2, Peptidyl-tR	ref|NP_001035503.1|	peptidyl-tRNA hydrolase 2, mitochondrial [Gallus gallus]	2,00E-49
gALL_v3_c1794	7	5	gnl|CDD|114686	pfam05978, UNC-93, Ion chan	gb|EFN70977.1|	UNC93-like protein [Camponotus floridanus]	1,00E-117
gALL_v3_c17940	2	7	gnl|CDD|162101	TIGR00911, 2A0308, L-type an	ref|XP_315672.4|	AGAP005653-PA [Anopheles gambiae str. PEST] gb|EAA11727.4| AGAP005653-PA [Anopheles gambiae str. PEST]	1,00E-108
gALL_v3_c17941	2	3	-	-	-	-	-
gALL_v3_c17942	9	5	-	-	-	-	-
gALL_v3_c17945	3	5	gnl|CDD|88274	cd05144, RIO2_C, RIO kinase f	ref|XP_003703345.1|	PREDICTED: serine/threonine-protein kinase RIO2-like [Megachile rotundata]	1,00E-95
gALL_v3_c17946	2	5	-	-	ref|XP_002422722.1|	type II transmembrane protein, putative [Pediculus humanus corporis] gb|EEB09984.1| type II transmembrane protein, putative [Pediculus humanus corporis]	1,00E-175
gALL_v3_c17947	0	6	-	-	-	-	-
gALL_v3_c17948	2	4	-	-	-	-	-
gALL_v3_c1795	4	7	gnl|CDD|144153	pfam00450, Peptidase_S10, Se	gb|ENN74996.1|	hypothetical protein YQE_08453, partial [Dendroctonus ponderosae]	4,00E-51
gALL_v3_c17950	0	3	-	-	-	-	-
gALL_v3_c17951	0	4	-	-	-	-	-
gALL_v3_c17952	2	1	gnl|CDD|128591	smart00295, B41, Band 4.1 ho	ref|XP_004931204.1|	PREDICTED: moesin/ezrin/radixin homolog 1-like isoform X1 [Bombyx mori]	7,00E-66
gALL_v3_c17953	3	1	-	-	-	-	-
gALL_v3_c17954	7	7	-	-	-	-	-
gALL_v3_c17956	2	3	-	-	-	-	-
gALL_v3_c17958	1	4	-	-	ref|XP_004930489.1|	PREDICTED: protein outspread-like [Bombyx mori]	4,00E-08
gALL_v3_c17959	2	5	-	-	-	-	-
gALL_v3_c1796	12	21	-	-	gb|ELU02007.1|	hypothetical protein CAPTEDRAFT_192879 [Capitella teleta]	1,00E-13
gALL_v3_c17960	8	4	gnl|CDD|150683	pfam10037, MRP-S27, Mitoch	ref|XP_001601605.2|	PREDICTED: hypothetical protein LOC100117334 [Nasonia vitripennis]	3,00E-51
gALL_v3_c17961	1	3	-	-	-	-	-
gALL_v3_c17964	3	6	-	-	-	-	-
gALL_v3_c17966	2	3	-	-	-	-	-
gALL_v3_c17967	2	2	-	-	ref|XP_002424546.1|	spire, putative [Pediculus humanus corporis] gb|EEB11808.1| spire, putative [Pediculus humanus corporis]	5,00E-21
gALL_v3_c17968	2	5	-	-	-	-	-
gALL_v3_c1797	16	34	gnl|CDD|153255	cd07993, LPLAT_DHAPAT-like,	ref|XP_003708610.1|	PREDICTED: glycerol-3-phosphate acyltransferase 1, mitochondrial-like [Megachile rotundata]	1,00E-162
gALL_v3_c17970	5	3	-	-	-	-	-
gALL_v3_c17971	0	4	-	-	-	-	-
gALL_v3_c17972	3	1	-	-	ref|XP_001582736.1|	intermediate dynein chain [Trichomonas vaginalis G3] gb|EAY21750.1| intermediate dynein chain, putative [Trichomonas vaginalis G3]	7,00E-58
gALL_v3_c17973	2	2	-	-	-	-	-
gALL_v3_c17974	5	6	-	-	-	-	-
gALL_v3_c17976	2	4	-	-	-	-	-
gALL_v3_c17977	4	3	-	-	-	-	-
gALL_v3_c17978	4	5	-	-	ref|XP_006617313.1|	PREDICTED: ribosome-binding protein 1-like isoform X7 [Apis dorsata]	9,00E-35
gALL_v3_c17979	2	3	gnl|CDD|191824	pfam07717, OB_NTP_bind, Ol	ref|XP_006637568.1|	PREDICTED: probable ATP-dependent RNA helicase DHX36-like [Lepisosteus oculatus]	2,00E-30
gALL_v3_c17981	4	4	-	-	-	-	-
gALL_v3_c17983	0	6	gnl|CDD|28933	cd00051, EFh, EF-hand, calciur	ref|XP_975683.1|	PREDICTED: similar to calbindin-32 [Tribolium castaneum] gb|EFA05940.1| hypothetical protein TcasGA2_TC008758 [Tribolium castaneum]	1,00E-121
gALL_v3_c17984	1	3	-	-	-	-	-
gALL_v3_c17986	4	2	gnl|CDD|190675	pfam03564, DUF1759, Protein	ref|XP_001948591.2|	PREDICTED: hypothetical protein LOC100167765 [Acyrthosiphon pisum]	6,00E-14
gALL_v3_c17987	4	4	-	-	-	-	-
gALL_v3_c17988	3	2	-	-	-	-	-
gALL_v3_c17989	2	3	-	-	-	-	-
gALL_v3_c1799	6	7	-	-	ref|XP_003424449.1|	PREDICTED: CLIP-associating protein 1-like isoform 2 [Nasonia vitripennis] ref|XP_003424452.1| PREDICTED: CLIP-associating protein 1-like isoform 5 [Nasonia vitripennis]	1,00E-57
gALL_v3_c17990	5	3	gnl|CDD|147446	pfam05251, UPF0197, Unchar	ref|XP_003708531.1|	PREDICTED: UPF0197 transmembrane protein C11orf10 homolog [Megachile rotundata]	6,00E-21
gALL_v3_c17991	1	4	-	-	-	-	-
gALL_v3_c17992	3	7	-	-	ref|XP_006559866.1|	PREDICTED: uncharacterized protein LOC725481 isoform X1 [Apis mellifera] ref|XP_006559867.1| PREDICTED: uncharacterized protein LOC725481 isoform X2 [Apis mellife	4,00E-68
gALL_v3_c17993	2	3	-	-	-	-	-
gALL_v3_c17994	0	4	-	-	-	-	-
gALL_v3_c17995	0	5	-	-	-	-	-
gALL_v3_c17996	3	3	-	-	gb|AAL40415.1|AF3698	endonuclease/reverse transcriptase [Branchiostoma floridae]	4,00E-25
gALL_v3_c17997	1	4	-	-	-	-	-
gALL_v3_c17999	3	7	gnl|CDD|129058	smart00825, PKS_KS, Beta-ket	ref|XP_002429689.1|	fatty acid synthase, putative [Pediculus humanus corporis] gb|EEB16951.1| fatty acid synthase, putative [Pediculus humanus corporis]	5,00E-77
gALL_v3_c18	6	8	gnl|CDD|189408	pfam00130, C1_1, Phorbol est	ref|XP_002424958.1|	conserved hypothetical protein [Pediculus humanus corporis] gb|EEB12220.1| conserved hypothetical protein [Pediculus humanus corporis]	7,00E-98

gALL_v3_c180	20	21	gnl|CDD|180305	PRK05888, PRK05888, NADH c	gb|ENN72467.1|	hypothetical protein YQE_10809, partial [Dendroctonus ponderosae]	1,00E-101
gALL_v3_c1800	3	9	-	-	-	-	-
gALL_v3_c18000	8	10	-	-	-	-	-
gALL_v3_c18001	1	3	-	-	ref|XP_006619301.1|	PREDICTED: 5'-AMP-activated protein kinase catalytic subunit alpha-2-like isoform X1 [Apis dorsata] ref|XP_006619302.1| PREDICTED: 5'-AMP-activated protein kinase cat	1,00E-06
gALL_v3_c18002	2	10	-	-	-	-	-
gALL_v3_c18004	2	2	-	-	-	-	-
gALL_v3_c18005	2	4	-	-	gb|ESO98647.1|	hypothetical protein LOTGIDRAFT_180523 [Lottia gigantea]	3,00E-22
gALL_v3_c18006	3	4	gnl|CDD|73264	cd01385, MYSc_type_IX, Myos	ref|XP_003241546.1|	PREDICTED: myosin-IXb isoform 2 [Acyrthosiphon pisum]	1,00E-138
gALL_v3_c18009	2	2	gnl|CDD|128591	smart00295, B41, Band 4.1 ho	gb|EFN79995.1|	Uncharacterized protein CG12467 [Harpegnathos saltator]	4,00E-89
gALL_v3_c1801	7	7	gnl|CDD|72942	cd04657, Piwi_ago-like, Piwi_a	gb|AGO85968.1|	argonaute 1, partial [Locusta migratoria]	1,00E-177
gALL_v3_c18010	4	2	-	-	-	-	-
gALL_v3_c18013	2	3	-	-	-	-	-
gALL_v3_c18016	1	2	-	-	ref|XP_002422781.1|	hypothetical protein Phum_PHUM012910 [Pediculus humanus corporis] gb|EEB10043.1| hypothetical protein Phum_PHUM012910 [Pediculus humanus corporis]	1,00E-20
gALL_v3_c18017	4	7	-	-	-	-	-
gALL_v3_c18018	3	1	-	-	-	-	-
gALL_v3_c18019	3	1	-	-	-	-	-
gALL_v3_c1802	15	23	gnl|CDD|188258	TIGR02917, PEP_TPR_lipo, put	ref|XP_002429488.1|	tpr repeat nuclear phosphoprotein, putative [Pediculus humanus corporis] gb|EEB16750.1| tpr repeat nuclear phosphoprotein, putative [Pediculus humanus corporis]	0
gALL_v3_c18020	2	2	-	-	-	-	-
gALL_v3_c18021	3	2	-	-	-	-	-
gALL_v3_c18022	2	4	gnl|CDD|34783	COG5184, ATS1, Alpha-tubulin	ref|XP_002407995.1|	conserved hypothetical protein [Ixodes scapularis] gb|EEC03915.1| conserved hypothetical protein [Ixodes scapularis]	6,00E-48
gALL_v3_c18023	1	8	-	-	-	-	-
gALL_v3_c18024	5	6	-	-	-	-	-
gALL_v3_c18025	0	5	-	-	-	-	-
gALL_v3_c18026	12	19	gnl|CDD|185453	PTZ00110, PTZ00110, helicase	ref|XP_006615333.1|	PREDICTED: probable ATP-dependent RNA helicase DDX17-like isoform X2 [Apis dorsata]	1,00E-138
gALL_v3_c18027	2	6	-	-	-	-	-
gALL_v3_c18028	3	3	-	-	-	-	-
gALL_v3_c18029	3	3	-	-	-	-	-
gALL_v3_c1803	13	22	-	-	ref|XP_001950452.1|	PREDICTED: hypothetical protein LOC100158766 [Acyrthosiphon pisum]	3,00E-34
gALL_v3_c18030	4	8	gnl|CDD|143944	pfam00178, Ets, Ets-domain.	gb|EFN87457.1|	DNA-binding protein Ets97D [Harpegnathos saltator]	4,00E-77
gALL_v3_c18031	2	3	-	-	-	-	-
gALL_v3_c18032	3	2	-	-	-	-	-
gALL_v3_c18033	3	5	-	-	-	-	-
gALL_v3_c18034	0	3	-	-	-	-	-
gALL_v3_c18037	7	4	-	-	gb|ETN58044.1|	hypothetical protein AND_010384 [Anopheles darlingi]	3,00E-12
gALL_v3_c1804	22	29	gnl|CDD|189635	pfam00621, RhoGEF, RhoGEF (	dbj|BAN20888.1|	hypothetical protein [Riptortus pedestris]	0
gALL_v3_c18040	0	5	-	-	-	-	-
gALL_v3_c18041	2	4	-	-	-	-	-
gALL_v3_c18042	1	8	gnl|CDD|150199	pfam09445, Methyltransf_15,	ref|XP_003706967.1|	PREDICTED: uncharacterized protein LOC100882090 [Megachile rotundata]	2,00E-72
gALL_v3_c18043	1	4	gnl|CDD|178622	PLN03073, PLN03073, ABC tra	ref|XP_002425562.1|	ATP-dependent transporter, putative [Pediculus humanus corporis] gb|EEB12824.1| ATP-dependent transporter, putative [Pediculus humanus corporis]	3,00E-69
gALL_v3_c18044	4	2	-	-	-	-	-
gALL_v3_c18045	2	3	-	-	-	-	-
gALL_v3_c18046	2	3	-	-	ref|XP_975329.1|	PREDICTED: similar to bicoid stability factor CG10302-PA [Tribolium castaneum] gb|EFA01919.1| hypothetical protein TcasGA2_TC007529 [Tribolium castaneum]	9,00E-41
gALL_v3_c18047	3	5	gnl|CDD|189954	pfam01359, Transposase_1, Tr	ref|XP_005104745.1|	PREDICTED: histone-lysine N-methyltransferase SETMAR-like [Aplysia californica]	1,00E-43
gALL_v3_c18049	2	6	-	-	-	-	-
gALL_v3_c18050	3	6	-	-	-	-	-
gALL_v3_c18051	2	4	-	-	-	-	-
gALL_v3_c18052	3	6	-	-	-	-	-
gALL_v3_c18053	0	4	-	-	-	-	-
gALL_v3_c18054	2	4	-	-	ref|XP_968383.1|	PREDICTED: similar to amidase [Tribolium castaneum]	9,00E-12
gALL_v3_c18056	3	5	gnl|CDD|190314	pfam02453, Reticulon, Reticul	gb|EFN73447.1|	Reticulon-1 [Camponotus floridanus]	2,00E-38
gALL_v3_c18057	1	4	-	-	-	-	-
gALL_v3_c18059	0	5	-	-	ref|XP_970001.2|	PREDICTED: similar to Egl-1 suppressor/DiO uptake defective/raf enhancer family member (eor-1) [Tribolium castaneum]	2,00E-70
gALL_v3_c1806	8	15	-	-	-	-	-
gALL_v3_c18060	3	5	gnl|CDD|173624	cd00192, PTKc, Catalytic doma	ref|XP_973940.1|	PREDICTED: similar to Wsck CG31127-PA [Tribolium castaneum] gb|EFA04011.1| hypothetical protein TcasGA2_TC014160 [Tribolium castaneum]	1,00E-36
gALL_v3_c18061	1	6	-	-	-	-	-
gALL_v3_c18062	1	5	gnl|CDD|187553	cd05242, SDR_a8, atypical (a)	ref|XP_002427474.1|	conserved hypothetical protein [Pediculus humanus corporis] gb|EEB14736.1| conserved hypothetical protein [Pediculus humanus corporis]	2,00E-68
gALL_v3_c18064	2	6	gnl|CDD|189654	pfam00651, BTB, BTB/POZ dor	gb|EFN82940.1|	Centrosome-associated zinc finger protein CP190 [Harpegnathos saltator]	9,00E-45
gALL_v3_c18065	6	4	-	-	gb|EFA11606.1|	hypothetical protein TcasGA2_TC014184 [Tribolium castaneum]	1,00E-55
gALL_v3_c18066	1	3	gnl|CDD|187700	cd09276, Rnase_HI_RT_non_L	pir||S37765	hypothetical protein - fruit fly (Drosophila miranda) transposon TRIM	2,00E-09
gALL_v3_c18067	4	4	-	-	-	-	-
gALL_v3_c18069	1	5	-	-	-	-	-
gALL_v3_c1807	10	7	gnl|CDD|178006	PLN02380, PLN02380, 1-acyl-s	ref|XP_001607215.1|	PREDICTED: 1-acyl-sn-glycerol-3-phosphate acyltransferase gamma-like [Nasonia vitripennis]	1,00E-144
gALL_v3_c18070	2	3	-	-	gb|AGM32707.1|	hypothetical protein, partial [Coptotermes formosanus]	8,00E-24
gALL_v3_c18072	3	5	-	-	-	-	-
gALL_v3_c18074	3	4	-	-	-	-	-
gALL_v3_c18077	4	3	-	-	-	-	-
gALL_v3_c18078	2	4	gnl|CDD|191049	pfam04627, ATP-synt_Eps, Mit	gb|AGG56526.1|	mitochondrial ATP synthase subunit epsilon [Antheraea yamamai]	4,00E-13
gALL_v3_c18079	4	4	-	-	-	-	-
gALL_v3_c18080	1	4	-	-	-	-	-
gALL_v3_c18081	2	1	-	-	-	-	-
gALL_v3_c18082	5	2	-	-	ref|XP_974871.1|	PREDICTED: similar to AGAP003131-PA [Tribolium castaneum]	5,00E-60
gALL_v3_c18083	0	4	gnl|CDD|118768	pfam10244, MRP-L51, Mitoch	ref|XP_001859028.1|	39S ribosomal protein L51, mitochondrial [Culex quinquefasciatus] gb|EDS35014.1| 39S ribosomal protein L51, mitochondrial [Culex quinquefasciatus]	3,00E-44
gALL_v3_c18084	1	5	-	-	-	-	-
gALL_v3_c18085	2	5	-	-	-	-	-
gALL_v3_c18086	2	4	gnl|CDD|191127	pfam04921, XAP5, XAP5 prote	ref|NP_651595.1|	CG12259 [Drosophila melanogaster] sp|Q9VAY7.1|FAM50_DROME RecName: Full=Protein FAM50 homolog gb|AAF56759.1| CG12259 [Drosophila melanogaster] gb|AAM	3,00E-61
gALL_v3_c18087	2	2	-	-	-	-	-
gALL_v3_c18088	3	1	-	-	-	-	-
gALL_v3_c18090	6	5	gnl|CDD|32366	COG2183, Tex, Transcriptional	ref|XP_002613450.1|	hypothetical protein BRAFLDRAFT_123927 [Branchiostoma floridae] gb|EEN69459.1| hypothetical protein BRAFLDRAFT_123927 [Branchiostoma floridae]	7,00E-71
gALL_v3_c18091	2	5	-	-	-	-	-
gALL_v3_c18092	0	4	-	-	-	-	-
gALL_v3_c18093	1	3	-	-	-	-	-
gALL_v3_c18094	3	1	-	-	ref|XP_002415128.1|	hypothetical protein IscW_ISCW024901 [Ixodes scapularis] gb|EEC18793.1| hypothetical protein IscW_ISCW024901 [Ixodes scapularis]	6,00E-20
gALL_v3_c18095	8	13	gnl|CDD|128538	smart00242, MYSc, Myosin. La	ref|XP_003396195.1|	PREDICTED: LOW QUALITY PROTEIN: myosin-IXa-like [Bombus terrestris]	1,00E-30
gALL_v3_c18096	4	2	gnl|CDD|162464	TIGR01649, hnRNP-L_PTB, hnF	ref|XP_003251730.2|	PREDICTED: heterogeneous nuclear ribonucleoprotein L isoform X1 [Apis mellifera]	2,00E-67
gALL_v3_c18097	2	3	-	-	-	-	-
gALL_v3_c18098	14	27	-	-	-	-	-
gALL_v3_c18099	1	3	-	-	-	-	-
gALL_v3_c181	1	7	-	-	ref|XP_002431466.1|	hypothetical protein Phum_PHUM530130 [Pediculus humanus corporis] gb|EEB18728.1| hypothetical protein Phum_PHUM530130 [Pediculus humanus corporis]	9,00E-33
gALL_v3_c1810	8	15	gnl|CDD|132720	cd02584, RNAP_II_Rpb1_C, La	dbj|BAJ78668.1|	RNA polymerase II largest subunit [Reticulitermes speratus]	0
gALL_v3_c18101	2	2	-	-	-	-	-
gALL_v3_c18102	1	4	-	-	ref|XP_003695453.1|	PREDICTED: THO complex subunit 5 homolog [Apis florea]	1,00E-06
gALL_v3_c18103	2	2	-	-	-	-	-
gALL_v3_c18104	3	4	-	-	ref|XP_003706873.1|	PREDICTED: uncharacterized protein LOC100881143 [Megachile rotundata]	4,00E-82
gALL_v3_c18105	2	3	-	-	-	-	-
gALL_v3_c18106	1	3	-	-	-	-	-
gALL_v3_c18107	1	8	-	-	-	-	-
gALL_v3_c18109	3	4	-	-	-	-	-
gALL_v3_c1811	12	10	-	-	gb|EFN64761.1|	Protein suppressor of sable [Camponotus floridanus]	5,00E-26
gALL_v3_c18110	4	7	gnl|CDD|99948	cd05517, Bromo_polybromo_	gb|EEZ98189.1|	hypothetical protein TcasGA2_TC000618 [Tribolium castaneum]	2,00E-86
gALL_v3_c18111	1	4	-	-	-	-	-
gALL_v3_c18112	1	3	-	-	ref|XP_005038429.1|	PREDICTED: c->U-editing enzyme APOBEC-1-like [Ficedula albicollis]	5,00E-06
gALL_v3_c18113	1	3	-	-	-	-	-
gALL_v3_c18115	2	3	-	-	-	-	-
gALL_v3_c18116	2	2	gnl|CDD|147414	pfam05206, TRM13, Methyltra	ref|XP_968859.1|	PREDICTED: similar to coiled-coil domain-containing protein 76 [Tribolium castaneum] gb|EFA11905.1| hypothetical protein TcasGA2_TC004062 [Tribolium castaneum]	2,00E-29
gALL_v3_c18117	2	4	-	-	-	-	-
gALL_v3_c18118	4	3	gnl|CDD|143945	pfam00179, UQ_con, Ubiquiti	ref|XP_004285436.1|	PREDICTED: ubiquitin-conjugating enzyme E2 T [Orcinus orca]	3,00E-51
gALL_v3_c18119	4	5	-	-	gb|EFN74007.1|	DnaJ-like protein subfamily C member 13 [Camponotus floridanus]	3,00E-73
gALL_v3_c1812	20	15	gnl|CDD|34633	COG5028, COG5028, Vesicle c	ref|XP_006561263.1|	PREDICTED: protein transport protein Sec24C-like [Apis mellifera]	1,00E-131
gALL_v3_c18120	3	4	-	-	-	-	-
gALL_v3_c18121	1	3	-	-	-	-	-
gALL_v3_c18122	2	5	-	-	-	-	-
gALL_v3_c18123	3	3	-	-	-	-	-
gALL_v3_c18124	6	7	gnl|CDD|146186	pfam03416, Peptidase_C54, P	gb|AGM32346.1|	cysteine protease ATG4A [Coptotermes formosanus]	0
gALL_v3_c18125	1	4	-	-	ref|XP_002431036.1|	serine/threonine-protein kinase MARK2, putative [Pediculus humanus corporis] gb|EEB18298.1| serine/threonine-protein kinase MARK2, putative [Pediculus humanus cor	3,00E-20
gALL_v3_c18126	4	3	-	-	ref|XP_394426.4|	PREDICTED: TBC1 domain family member 9 [Apis mellifera]	3,00E-70
gALL_v3_c18127	1	3	-	-	ref|XP_006613302.1|	PREDICTED: dnaJ homolog subfamily C member 16-like [Apis dorsata]	1,00E-33
gALL_v3_c18128	3	5	gnl|CDD|149850	pfam08913, VBS, Vinculin Binc	ref|XP_002432159.1|	Talin-2, putative [Pediculus humanus corporis] gb|EEB19421.1| Talin-2, putative [Pediculus humanus corporis]	9,00E-95
gALL_v3_c18129	2	2	-	-	-	-	-
gALL_v3_c1813	4	3	-	-	-	-	-
gALL_v3_c18130	3	8	-	-	-	-	-
gALL_v3_c18132	3	2	-	-	-	-	-
gALL_v3_c18133	2	5	-	-	-	-	-
gALL_v3_c18134	4	2	-	-	gb|AGM32302.1|	plastin-3-like protein, partial [Coptotermes formosanus]	9,00E-29
gALL_v3_c18135	0	5	-	-	ref|XP_003696276.1|	PREDICTED: uncharacterized protein LOC100872580 isoform 1 [Apis florea] ref|XP_003696277.1| PREDICTED: uncharacterized protein LOC100872580 isoform 2 [Apis flore	3,00E-09
gALL_v3_c18136	3	3	-	-	-	-	-
gALL_v3_c18137	2	2	-	-	ref|XP_004923347.1|	PREDICTED: lysM and putative peptidoglycan-binding domain-containing protein 2-like [Bombyx mori]	8,00E-25
gALL_v3_c18139	2	4	-	-	-	-	-
gALL_v3_c1814	16	20	-	-	-	-	-
gALL_v3_c18140	1	7	-	-	-	-	-
gALL_v3_c18143	5	3	gnl|CDD|189424	pfam00153, Mito_carr, Mitoch	ref|XP_002426070.1|	mitochondrial dicarboxylate carrier, putative [Pediculus humanus corporis] gb|EEB13332.1| mitochondrial dicarboxylate carrier, putative [Pediculus humanus corporis]	3,00E-97
gALL_v3_c18144	4	7	gnl|CDD|173696	cd05605, STKc_GRK4_like, Cat	gb|EFN66691.1|	G protein-coupled receptor kinase 2 [Camponotus floridanus]	1,00E-143
gALL_v3_c18145	3	1	gnl|CDD|191810	pfam07679, I-set, Immunoglol	gb|ABJ17059.1|	IP16011p [Drosophila melanogaster]	2,00E-46
gALL_v3_c18146	5	6	-	-	-	-	-
gALL_v3_c18148	2	4	-	-	gb|EFN65619.1|	Putative uncharacterized transposon-derived protein F52C9.6 [Camponotus floridanus]	9,00E-09
gALL_v3_c18149	5	5	-	-	-	-	-
gALL_v3_c18150	4	9	-	-	-	-	-
gALL_v3_c18151	2	2	-	-	-	-	-
gALL_v3_c18152	2	2	-	-	-	-	-
gALL_v3_c18153	2	4	-	-	-	-	-
gALL_v3_c18155	2	2	-	-	-	-	-
gALL_v3_c18156	1	3	-	-	-	-	-
gALL_v3_c18157	3	1	-	-	-	-	-
gALL_v3_c18159	2	3	-	-	-	-	-
gALL_v3_c1816	2	6	-	-	-	-	-

gALL_v3_c18160	3	1	-	-	-	-	-	
gALL_v3_c18161	2	3	gnl|CDD|128878	smart00630, Sema, semaphor	gb|ABK35089.2|	semaphorin 2a [Gryllus bimaculatus]		1,00E-129
gALL_v3_c18163	6	7	-	-	-	-	-	
gALL_v3_c18164	1	6	-	-	-	-	-	
gALL_v3_c18165	1	3	-	-	-	-	-	
gALL_v3_c18166	3	3	-	-	-	-	-	
gALL_v3_c18167	3	2	-	-	ref|XP_001653736.1|	fetal globin-inducing factor [Aedes aegypti] gb|EAT47207.1| AAEL001653-PA [Aedes aegypti]		9,00E-12
gALL_v3_c18168	0	4	-	-	-	-	-	
gALL_v3_c18169	4	4	-	-	-	-	-	
gALL_v3_c1817	8	6	-	-	dbj|BAN21337.1|	unkown protein [Riptortus pedestris]		2,00E-14
gALL_v3_c18170	2	4	-	-	ref|XP_003245329.1|	PREDICTED: hypothetical protein LOC100161833 isoform 2 [Acyrthosiphon pisum]		6,00E-12
gALL_v3_c18171	1	8	-	-	-	-	-	
gALL_v3_c18172	2	5	gnl|CDD|193604	cd09829, PET_testin, The PET	ref|XP_001949083.2|	PREDICTED: testin-like [Acyrthosiphon pisum]		2,00E-15
gALL_v3_c18173	1	4	-	-	-	-	-	
gALL_v3_c18175	3	2	-	-	gb|EFN81907.1|	Nucleolar pre-ribosomal-associated protein 1 [Harpegnathos saltator]		8,00E-27
gALL_v3_c18176	0	6	gnl|CDD|109266	pfam00201, UDPGT, UDP-gluc	ref|XP_001951105.2|	PREDICTED: ecdysteroid UDP-glucosyltransferase-like isoform 1 [Acyrthosiphon pisum]		1,00E-09
gALL_v3_c18177	2	2	gnl|CDD|133070	cd03858, M14_CP_N-E_like, C	gb|EFA09157.1|	carboxypeptidase A [Tribolium castaneum]		8,00E-83
gALL_v3_c18178	2	4	-	-	-	-	-	
gALL_v3_c18179	1	4	-	-	ref|XP_003401843.1|	PREDICTED: hypothetical protein LOC100649072 [Bombus terrestris]		6,00E-08
gALL_v3_c1818	5	3	gnl|CDD|147760	pfam05783, DLIC, Dynein light	gb|EGI65931.1|	Cytoplasmic dynein 1 light intermediate chain 2 [Acromyrmex echinatior]		1,00E-121
gALL_v3_c18180	0	5	-	-	-	-	-	
gALL_v3_c18181	4	4	gnl|CDD|192035	pfam08430, Fork_head_N, For	gb|EFA11073.1|	hypothetical protein TcasGA2_TC004669 [Tribolium castaneum]		2,00E-45
gALL_v3_c18182	2	1	-	-	ref|XP_006568605.1|	PREDICTED: cAMP-specific 3',5'-cyclic phosphodiesterase, isoforms N/G isoform X19 [Apis mellifera]		4,00E-53
gALL_v3_c18183	4	3	gnl|CDD|173623	cd00180, PKc, Catalytic domai	ref|NP_001155063.1|	eukaryotic translation initiation factor 2-alpha kinase 1 [Nasonia vitripennis]		3,00E-38
gALL_v3_c18184	0	4	gnl|CDD|30094	cd01372, KISc_KIF4, Kinesin m	ref|XP_002428395.1|	kif21, putative [Pediculus humanus corporis] gb|EEB15657.1| kif21, putative [Pediculus humanus corporis]		6,00E-19
gALL_v3_c18185	5	4	-	-	-	-	-	
gALL_v3_c18187	2	9	-	-	-	-	-	
gALL_v3_c18188	2	3	-	-	-	-	-	
gALL_v3_c18189	3	3	-	-	gb|EFA03892.1|	hypothetical protein TcasGA2_TC014016 [Tribolium castaneum]		2,00E-28
gALL_v3_c1819	3	5	-	-	-	-	-	
gALL_v3_c18190	2	3	gnl|CDD|189795	pfam00995, Sec1, Sec1 family.	gb|EGI63219.1|	Vacuolar protein sorting-associated protein 45 [Acromyrmex echinatior]		1,00E-96
gALL_v3_c18191	3	4	gnl|CDD|144191	pfam00503, G-alpha, G-protei	ref|XP_003702528.1|	PREDICTED: guanine nucleotide-binding protein G(q) subunit alpha-like isoform 5 [Megachile rotundata]		6,00E-95
gALL_v3_c18193	3	2	-	-	-	-	-	
gALL_v3_c18194	3	1	-	-	-	-	-	
gALL_v3_c18195	2	2	-	-	-	-	-	
gALL_v3_c18196	1	3	-	-	-	-	-	
gALL_v3_c18197	6	9	-	-	-	-	-	
gALL_v3_c18198	4	4	-	-	-	-	-	
gALL_v3_c18199	3	6	-	-	-	-	-	
gALL_v3_c182	17	13	-	-	ref|XP_003403120.1|	PREDICTED: hypothetical protein LOC100643472 [Bombus terrestris]		2,00E-11
gALL_v3_c1820	11	17	gnl|CDD|110745	pfam01769, MgtE, Divalent ca	gb|EFN69384.1|	Solute carrier family 41 member 1 [Camponotus floridanus]		1,00E-163
gALL_v3_c18200	2	5	-	-	-	-	-	
gALL_v3_c18201	3	2	gnl|CDD|33030	COG3217, COG3217, Uncharac	ref|XP_003488163.1|	PREDICTED: hypothetical protein LOC100741218 [Bombus impatiens]		7,00E-55
gALL_v3_c18202	5	4	-	-	ref|XP_006613087.1|	PREDICTED: lysosomal protein NCU-G1-A-like isoform X1 [Apis dorsata]		1,00E-67
gALL_v3_c18203	1	5	-	-	ref|XP_002423090.1|	conserved hypothetical protein [Pediculus humanus corporis] gb|EEB10352.1| conserved hypothetical protein [Pediculus humanus corporis]		7,00E-37
gALL_v3_c18204	0	6	-	-	-	-	-	
gALL_v3_c18205	2	3	-	-	-	-	-	
gALL_v3_c18206	2	6	-	-	-	-	-	
gALL_v3_c18207	2	3	-	-	-	-	-	
gALL_v3_c18208	3	2	gnl|CDD|177693	PLN00062, PLN00062, TATA-bo	ref|XP_003402092.1|	PREDICTED: TATA-box-binding protein-like [Bombus terrestris] ref|XP_003493334.1| PREDICTED: TATA-box-binding protein-like [Bombus impatiens]		2,00E-86
gALL_v3_c18209	1	2	-	-	-	-	-	
gALL_v3_c18210	1	3	-	-	-	-	-	
gALL_v3_c18212	0	4	-	-	-	-	-	
gALL_v3_c18213	1	3	-	-	-	-	-	
gALL_v3_c18214	3	2	-	-	gb|ESP00009.1|	hypothetical protein LOTGIDRAFT_113097 [Lottia gigantea]		2,00E-73
gALL_v3_c18215	1	3	-	-	-	-	-	
gALL_v3_c18216	4	6	-	-	gb|EFN65475.1|	Melanotransferrin [Camponotus floridanus]		2,00E-39
gALL_v3_c18217	2	6	-	-	-	-	-	
gALL_v3_c18218	0	5	gnl|CDD|189795	pfam00995, Sec1, Sec1 family.	ref|XP_001654152.1|	vesicle protein sorting-associated [Aedes aegypti] gb|EAT47006.1| AAEL001859-PA [Aedes aegypti]		1,00E-83
gALL_v3_c1822	6	4	-	-	-	-	-	
gALL_v3_c18220	2	3	-	-	-	-	-	
gALL_v3_c18221	2	4	-	-	ref|XP_002017502.1|	GL22333 [Drosophila persimilis] gb|EDW34602.1| GL22333 [Drosophila persimilis]		5,00E-48
gALL_v3_c18222	13	13	-	-	gb|EFA13462.1|	hypothetical protein TcasGA2_TC005062 [Tribolium castaneum]		4,00E-13
gALL_v3_c18224	5	2	-	-	-	-	-	
gALL_v3_c18225	1	5	-	-	-	-	-	
gALL_v3_c18226	3	2	-	-	-	-	-	
gALL_v3_c18227	2	4	-	-	-	-	-	
gALL_v3_c18229	5	5	-	-	-	-	-	
gALL_v3_c1823	17	10	-	-	-	-	-	
gALL_v3_c18230	4	4	-	-	-	-	-	
gALL_v3_c18231	3	2	-	-	-	-	-	
gALL_v3_c18232	2	2	-	-	-	-	-	
gALL_v3_c18233	3	2	-	-	-	-	-	
gALL_v3_c18234	3	2	-	-	-	-	-	
gALL_v3_c18235	2	4	-	-	-	-	-	
gALL_v3_c18236	3	1	gnl|CDD|146070	pfam03251, Tymo_45kd_70kd	ref|XP_001322449.1|	EF hand family protein [Trichomonas vaginalis G3] gb|EAY10226.1| EF hand family protein [Trichomonas vaginalis G3]		7,00E-26
gALL_v3_c18237	4	6	-	-	gb|EHJ71800.1|	putative integrator complex subunit 6 [Danaus plexippus]		7,00E-99
gALL_v3_c18238	1	4	gnl|CDD|190222	pfam02138, Beach, Beige/BEA	ref|XP_785921.3|	PREDICTED: neurobeachin-like protein 1-like [Strongylocentrotus purpuratus]		5,00E-78
gALL_v3_c18239	1	3	-	-	-	-	-	
gALL_v3_c18240	3	2	-	-	-	-	-	
gALL_v3_c18241	1	3	gnl|CDD|33380	COG3580, COG3580, Uncharac	ref|WP_002696157.1|	2-hydroxyglutaryl-CoA dehydratase [Treponema phagedenis] gb|EFW38908.1| putative CoA-substrate-specific enzyme activase [Treponema phagedenis F0421]		7,00E-39
gALL_v3_c18242	0	5	-	-	-	-	-	
gALL_v3_c18243	2	1	-	-	-	-	-	
gALL_v3_c18244	2	2	gnl|CDD|192372	pfam09768, Peptidase_M76, F	ref|XP_001846255.1|	conserved hypothetical protein [Culex quinquefasciatus] gb|EDS43081.1| conserved hypothetical protein [Culex quinquefasciatus]		2,00E-39
gALL_v3_c18245	6	3	gnl|CDD|73154	cd01647, RT_LTR, RT_LTR: Rev	gb|EFA13518.1|	hypothetical protein TcasGA2_TC001491 [Tribolium castaneum]		4,00E-89
gALL_v3_c18248	1	4	-	-	-	-	-	
gALL_v3_c18249	2	3	-	-	-	-	-	
gALL_v3_c1825	13	17	-	-	-	-	-	
gALL_v3_c18251	4	5	-	-	-	-	-	
gALL_v3_c18253	3	1	-	-	-	-	-	
gALL_v3_c18254	5	9	-	-	gb|EFN84895.1|	E3 ubiquitin-protein ligase HUWE1 [Harpegnathos saltator]		2,00E-44
gALL_v3_c18255	3	1	-	-	-	-	-	
gALL_v3_c18256	0	4	-	-	-	-	-	
gALL_v3_c18257	3	3	-	-	-	-	-	
gALL_v3_c18258	4	3	-	-	dbj|BAD84176.1|	cytochrome P450 [Hodotermopsis sjostedti]		4,00E-08
gALL_v3_c18259	1	3	-	-	gb|EFA10558.1|	hypothetical protein TcasGA2_TC012814 [Tribolium castaneum]		1,00E-34
gALL_v3_c1826	13	16	gnl|CDD|193173	pfam12697, Abhydrolase_6, A	ref|XP_001651936.1|	epoxide hydrolase [Aedes aegypti] gb|EAT42096.1| AAEL006342-PC [Aedes aegypti]		1,00E-65
gALL_v3_c18260	3	2	-	-	-	-	-	
gALL_v3_c18261	1	5	-	-	-	-	-	
gALL_v3_c18262	5	4	-	-	-	-	-	
gALL_v3_c18263	4	6	gnl|CDD|48664	cd03569, VHS_Hrs_Vps27p, VI	ref|XP_006610996.1|	PREDICTED: hepatocyte growth factor-regulated tyrosine kinase substrate-like isoform X3 [Apis dorsata]		1,00E-107
gALL_v3_c18264	2	4	-	-	-	-	-	
gALL_v3_c18265	2	4	-	-	-	-	-	
gALL_v3_c18266	2	4	-	-	-	-	-	
gALL_v3_c18267	1	4	-	-	-	-	-	
gALL_v3_c18268	2	6	gnl|CDD|193272	pfam12796, Ank_2, Ankyrin re	ref|XP_003698421.1|	PREDICTED: myotrophin-like isoform 2 [Apis florea]		3,00E-51
gALL_v3_c18269	5	5	-	-	ref|XP_003491245.1|	PREDICTED: protein lap4-like isoform 3 [Bombus impatiens]		1,00E-13
gALL_v3_c1827	4	7	-	-	-	-	-	
gALL_v3_c18270	1	3	-	-	gb|AGM48362.1|	transformer [Daphnia pulex]		2,00E-06
gALL_v3_c18271	1	8	-	-	ref|XP_003486324.1|	PREDICTED: SLIT-ROBO Rho GTPase-activating protein 1-like isoform 1 [Bombus impatiens] ref|XP_003486325.1| PREDICTED: SLIT-ROBO Rho GTPase-activating protein 1-li		2,00E-10
gALL_v3_c18272	5	3	-	-	-	-	-	
gALL_v3_c18273	4	5	-	-	gb|EFN89126.1|	Histone-lysine N-methyltransferase SETMAR [Harpegnathos saltator]		4,00E-06
gALL_v3_c18274	2	2	-	-	-	-	-	
gALL_v3_c18275	4	3	-	-	-	-	-	
gALL_v3_c18276	3	3	-	-	ref|XP_003691185.1|	PREDICTED: sodium-coupled monocarboxylate transporter 2-like [Apis florea]		1,00E-27
gALL_v3_c18277	1	4	-	-	-	-	-	
gALL_v3_c18278	6	3	gnl|CDD|192996	pfam12348, CLASP_N, CLASP I	gb|EFX67958.1|	hypothetical protein DAPPUDRAFT_330564 [Daphnia pulex]		1,00E-61
gALL_v3_c18279	3	3	-	-	ref|XP_003241548.1|	PREDICTED: myosin-IXb isoform 4 [Acyrthosiphon pisum]		8,00E-17
gALL_v3_c1828	10	9	-	-	ref|XP_002426585.1|	ADP-ribosylation factor GTPase-activating protein, putative [Pediculus humanus corporis] gb|EEB13847.1| ADP-ribosylation factor GTPase-activating protein, putative [Ped		5,00E-68
gALL_v3_c18280	2	4	-	-	-	-	-	
gALL_v3_c18281	8	6	gnl|CDD|145761	pfam02777, Sod_Fe_C, Iron/m	gb|AEM66982.1|	mitochondrial manganese superoxide dismutase [Tigriopus japonicus]		3,00E-48
gALL_v3_c18282	2	2	-	-	-	-	-	
gALL_v3_c18284	2	3	-	-	-	-	-	
gALL_v3_c18285	2	3	-	-	-	-	-	
gALL_v3_c18286	1	5	-	-	ref|XP_003707451.1|	PREDICTED: uncharacterized protein LOC100880882 [Megachile rotundata]		8,00E-11
gALL_v3_c18287	4	5	-	-	gb|EFN73551.1|	hypothetical protein EAG_11362 [Camponotus floridanus]		1,00E-135
gALL_v3_c18288	2	2	-	-	-	-	-	
gALL_v3_c18289	2	6	-	-	-	-	-	
gALL_v3_c1829	14	16	gnl|CDD|128595	smart00300, ChSh, Chromo Sh	ref|XP_002426543.1|	hypothetical protein Phum_PHUM259320 [Pediculus humanus corporis] gb|EEB13805.1| hypothetical protein Phum_PHUM259320 [Pediculus humanus corporis]		6,00E-71
gALL_v3_c18290	5	7	gnl|CDD|146000	pfam03154, Atrophin-1, Atrop	gb|EHJ65885.1|	hypothetical protein KGM_11067 [Danaus plexippus]		1,00E-09
gALL_v3_c18292	2	2	-	-	-	-	-	
gALL_v3_c18293	3	4	-	-	-	-	-	
gALL_v3_c18294	4	3	-	-	gb|EFX64671.1|	hypothetical protein DAPPUDRAFT_333961 [Daphnia pulex]		7,00E-32
gALL_v3_c18295	9	7	-	-	-	-	-	
gALL_v3_c18296	7	8	-	-	-	-	-	
gALL_v3_c18297	4	3	gnl|CDD|192145	pfam08755, YccV-like, Hemime	ref|XP_006813670.1|	PREDICTED: uncharacterized protein LOC102806273 [Saccoglossus kowalevskii]		1,00E-43
gALL_v3_c18298	2	1	gnl|CDD|31424	COG1231, COG1231, Monoam	ref|XP_003218985.1|	PREDICTED: amine oxidase [flavin-containing] A-like [Anolis carolinensis]		2,00E-22
gALL_v3_c18299	3	1	-	-	-	-	-	
gALL_v3_c183	12	9	gnl|CDD|30046	cd01303, GDEase, Guanine de	gb|EFN84526.1|	Guanine deaminase [Harpegnathos saltator]		1,00E-94
gALL_v3_c18301	1	4	-	-	ref|XP_006567858.1|	PREDICTED: transportin-1-like isoform X1 [Apis mellifera] ref|XP_006613719.1| PREDICTED: transportin-1-like isoform X2 [Apis dorsata]		3,00E-66
gALL_v3_c18302	1	4	-	-	-	-	-	
gALL_v3_c18303	3	1	gnl|CDD|166027	PLN02386, PLN02386, superox	gb|AEG79720.1|	Cu-Zn superoxide dismutase [Apostichopus japonicus]		1,00E-20
gALL_v3_c18304	2	2	-	-	-	-	-	
gALL_v3_c18306	2	7	-	-	-	-	-	

gALL_v3_c18307	2	3	-	-	-	-	-
gALL_v3_c18308	3	2	-	-	ref|XP_003708085.1|	PREDICTED: uncharacterized protein LOC100882642 [Megachile rotundata]	2,00E-74
gALL_v3_c1831	7	18	gnl|CDD|189372	pfam00067, p450, Cytochrom	sp|Q964R1.1|CP6J1_BL	RecName: Full=Cytochrome P450 6j1; AltName: Full=CYPVIJ1 gb|AAK57913.1|AF281325_1 cytochrome P450 CYP6J1 [Blattella germanica]	7,00E-93
gALL_v3_c18311	3	6	-	-	ref|WP_006235594.1|	hypothetical protein [Collinsella aerofaciens] gb|EBA38630.1| hypothetical protein COLAER_02284 [Collinsella aerofaciens ATCC 25986] gb|EBA38843.1| hypothetical prot	7,00E-57
gALL_v3_c18312	3	1	-	-	-	-	-
gALL_v3_c18313	3	2	gnl|CDD|190537	pfam03137, OATP, Organic Ani	gb|EFN80038.1|	Solute carrier organic anion transporter family member 4A1 [Harpegnathos saltator]	7,00E-67
gALL_v3_c18314	8	5	-	-	-	-	-
gALL_v3_c18315	3	1	-	-	-	-	-
gALL_v3_c18316	3	2	-	-	-	-	-
gALL_v3_c18317	3	2	-	-	-	-	-
gALL_v3_c18318	0	4	-	-	-	-	-
gALL_v3_c18319	14	13	gnl|CDD|110260	pfam01246, Ribosomal_L24e,	ref|XP_001601399.1|	PREDICTED: 60S ribosomal protein L24-like [Nasonia vitripennis]	4,00E-46
gALL_v3_c1832	7	9	gnl|CDD|117128	pfam08551, DUF1751, Eukary	ref|XP_003701309.1|	PREDICTED: transmembrane protein 115-like [Megachile rotundata]	0
gALL_v3_c18320	0	5	-	-	gb|ESO86520.1|	hypothetical protein LOTGIDRAFT_179365 [Lottia gigantea]	2,00E-10
gALL_v3_c18321	3	10	-	-	-	-	-
gALL_v3_c18322	1	7	gnl|CDD|143864	pfam00083, Sugar_tr, Sugar (a	ref|XP_002427227.1|	conserved hypothetical protein [Pediculus humanus corporis] gb|EEB14489.1| conserved hypothetical protein [Pediculus humanus corporis]	2,00E-16
gALL_v3_c18323	3	2	gnl|CDD|34774	COG5175, MOT2, Transcriptio	gb|EFN84292.1|	CCR4-NOT transcription complex subunit 4 [Harpegnathos saltator]	5,00E-94
gALL_v3_c18325	3	8	-	-	-	-	-
gALL_v3_c18326	1	4	-	-	ref|XP_001984026.1|	GH15237 [Drosophila grimshawi] gb|EDV96374.1| GH15237 [Drosophila grimshawi]	5,00E-17
gALL_v3_c18327	3	2	-	-	gb|EFN68437.1|	hypothetical protein EAG_10410 [Camponotus floridanus]	1,00E-13
gALL_v3_c18328	6	3	-	-	-	-	-
gALL_v3_c1833	8	13	gnl|CDD|165378	PHA03096, PHA03096, p28-lik	gb|EFN71788.1|	Makorin-1 [Camponotus floridanus]	1,00E-133
gALL_v3_c18330	1	4	-	-	-	-	-
gALL_v3_c18331	0	5	-	-	-	-	-
gALL_v3_c18332	1	4	-	-	-	-	-
gALL_v3_c18333	6	4	-	-	ref|XP_003242319.1|	PREDICTED: histone-lysine N-methyltransferase SETMAR-like [Acyrthosiphon pisum]	1,00E-12
gALL_v3_c18334	1	7	-	-	-	-	-
gALL_v3_c18335	1	5	-	-	-	-	-
gALL_v3_c18336	3	3	gnl|CDD|146646	pfam04117, Mpv17_PMP22, M	dbj|BAN20755.1|	pmp22 peroxisomal membrane protein, putative [Riptortus pedestris]	9,00E-76
gALL_v3_c18337	2	3	-	-	-	-	-
gALL_v3_c18338	2	3	-	-	-	-	-
gALL_v3_c1834	5	12	-	-	-	-	-
gALL_v3_c18340	6	8	-	-	-	-	-
gALL_v3_c18341	2	6	gnl|CDD|133269	cd01866, Rab2, Rab2 subfamil	ref|XP_002032634.1|	GM20894 [Drosophila sechellia] gb|EDW46647.1| GM20894 [Drosophila sechellia]	2,00E-78
gALL_v3_c18342	3	3	-	-	-	-	-
gALL_v3_c18343	1	3	-	-	-	-	-
gALL_v3_c18344	4	8	-	-	gb|EFN70536.1|	Supporter of activation of yellow protein [Camponotus floridanus]	3,00E-46
gALL_v3_c18345	4	6	-	-	gb|ADI61823.1|	endonuclease-reverse transcriptase [Bombyx mori]	1,00E-17
gALL_v3_c18346	3	7	gnl|CDD|152580	pfam12145, Med12-LCEWAV,	ref|XP_003703055.1|	PREDICTED: mediator of RNA polymerase II transcription subunit 12-like protein [Megachile rotundata]	1,00E-102
gALL_v3_c18347	2	2	-	-	-	-	-
gALL_v3_c18349	1	5	-	-	-	-	-
gALL_v3_c1835	6	4	-	-	-	-	-
gALL_v3_c18350	2	2	-	-	ref|XP_003248981.1|	PREDICTED: hypothetical protein LOC100570774, partial [Acyrthosiphon pisum]	2,00E-11
gALL_v3_c18351	2	8	-	-	-	-	-
gALL_v3_c18352	3	2	-	-	-	-	-
gALL_v3_c18353	3	1	-	-	-	-	-
gALL_v3_c18354	1	3	-	-	-	-	-
gALL_v3_c18355	1	3	-	-	ref|XP_003398416.1|	PREDICTED: putative ATP-dependent RNA helicase DHX57-like [Bombus terrestris]	8,00E-27
gALL_v3_c18356	6	6	-	-	ref|XP_002078332.1|	GD22598 [Drosophila simulans] gb|EDX03917.1| GD22598 [Drosophila simulans]	5,00E-45
gALL_v3_c18358	6	4	gnl|CDD|34495	COG4886, COG4886, Leucine-	ref|XP_003727956.1|	PREDICTED: leucine-rich repeat-containing protein 4-like [Strongylocentrotus purpuratus]	4,00E-35
gALL_v3_c18359	0	5	-	-	-	-	-
gALL_v3_c1836	20	37	gnl|CDD|176313	cd01237, Unc112, Unc-112 ple	ref|XP_392367.3|	PREDICTED: unc-112-related protein-like isoform 1 [Apis mellifera]	0
gALL_v3_c18361	2	5	-	-	gb|EKC35536.1|	hypothetical protein CGI_10020303 [Crassostrea gigas]	3,00E-06
gALL_v3_c18362	1	5	-	-	-	-	-
gALL_v3_c18363	1	4	-	-	-	-	-
gALL_v3_c18364	3	2	-	-	-	-	-
gALL_v3_c18366	2	3	-	-	-	-	-
gALL_v3_c18368	0	4	-	-	-	-	-
gALL_v3_c18369	3	4	gnl|CDD|144398	pfam00786, PBD, P21-Rho-bin	ref|XP_002041216.1|	GM15431 [Drosophila sechellia] ref|XP_002103181.1| GD20287 [Drosophila simulans] gb|EDW44864.1| GM15431 [Drosophila sechellia] gb|EDX12684.1| GD20287 [Dros	6,00E-29
gALL_v3_c18371	3	7	-	-	-	-	-
gALL_v3_c18372	3	1	-	-	gb|AGM32707.1|	hypothetical protein, partial [Coptotermes formosanus]	1,00E-09
gALL_v3_c18374	3	2	-	-	-	-	-
gALL_v3_c18375	6	4	-	-	-	-	-
gALL_v3_c18376	1	3	-	-	-	-	-
gALL_v3_c18377	1	4	-	-	emb|CCF23094.1|	Dicer 2 [Blattella germanica]	5,00E-39
gALL_v3_c18378	8	9	-	-	-	-	-
gALL_v3_c18379	1	4	-	-	-	-	-
gALL_v3_c1838	4	13	gnl|CDD|192002	pfam08326, ACC_central, Ace	ref|XP_002429216.1|	acetyl-CoA carboxylase, putative [Pediculus humanus corporis] gb|EEB16478.1| acetyl-CoA carboxylase, putative [Pediculus humanus corporis]	0
gALL_v3_c18380	1	6	-	-	gb|EFN74406.1|	hypothetical protein EAG_08095 [Camponotus floridanus]	5,00E-17
gALL_v3_c18381	2	2	-	-	-	-	-
gALL_v3_c18383	0	6	-	-	-	-	-
gALL_v3_c18385	1	3	-	-	-	-	-
gALL_v3_c18386	7	11	gnl|CDD|109812	pfam00769, ERM, Ezrin/radixi	dbj|BAN21261.1|	moesin/ezrin/radixin homolog 1 [Riptortus pedestris]	1,00E-104
gALL_v3_c18387	3	2	-	-	-	-	-
gALL_v3_c18388	3	2	-	-	-	-	-
gALL_v3_c18389	7	12	-	-	-	-	-
gALL_v3_c1839	21	18	-	-	-	-	-
gALL_v3_c18390	7	7	-	-	-	-	-
gALL_v3_c18391	2	4	-	-	ref|XP_970972.1|	PREDICTED: similar to arylsulfatase b [Tribolium castaneum] gb|EFA05056.1| hypothetical protein TcasGA2_TC015151 [Tribolium castaneum] gb|AGQ03798.1| arylsulfatas	5,00E-32
gALL_v3_c18392	1	4	-	-	-	-	-
gALL_v3_c18393	1	3	-	-	dbj|BAO00945.1|	CRF-related diuretic hormone 45 [Nilaparvata lugens]	4,00E-29
gALL_v3_c18394	2	3	-	-	gb|AAB65093.1|	Lian-Aa1 retrotransposon protein [Aedes aegypti]	2,00E-09
gALL_v3_c18395	18	20	gnl|CDD|73156	cd01650, RT_nLTR_like, RT_nL	ref|XP_003243437.1|	PREDICTED: hypothetical protein LOC100572891 [Acyrthosiphon pisum]	5,00E-68
gALL_v3_c18396	3	2	-	-	-	-	-
gALL_v3_c18397	15	16	-	-	ref|XP_003426275.1|	PREDICTED: hypothetical protein LOC100678253 [Nasonia vitripennis]	6,00E-11
gALL_v3_c18398	1	4	-	-	-	-	-
gALL_v3_c18399	2	5	-	-	-	-	-
gALL_v3_c1840	8	5	gnl|CDD|181380	PRK08315, PRK08315, AMP-bi	gb|ABE60814.1|	cxpwmw01 [Periplaneta americana]	9,00E-62
gALL_v3_c18400	0	5	-	-	-	-	-
gALL_v3_c18401	3	1	-	-	-	-	-
gALL_v3_c18402	2	2	-	-	ref|XP_005104758.1|	PREDICTED: collagen type IV alpha-3-binding protein-like isoform X2 [Aplysia californica]	4,00E-31
gALL_v3_c18403	2	3	-	-	-	-	-
gALL_v3_c18404	1	3	-	-	ref|XP_003427451.1|	PREDICTED: hypothetical protein LOC100678861 [Nasonia vitripennis]	4,00E-60
gALL_v3_c18405	2	2	-	-	-	-	-
gALL_v3_c18408	3	2	-	-	-	-	-
gALL_v3_c18409	17	23	-	-	-	-	-
gALL_v3_c1841	20	39	gnl|CDD|178337	PLN02736, PLN02736, long-ch	gb|ERL88489.1|	hypothetical protein D910_05875 [Dendroctonus ponderosae]	2,00E-18
gALL_v3_c18410	1	3	-	-	-	-	-
gALL_v3_c18412	0	6	-	-	-	-	-
gALL_v3_c18413	1	3	-	-	-	-	-
gALL_v3_c18414	4	4	-	-	-	-	-
gALL_v3_c18415	3	2	-	-	-	-	-
gALL_v3_c18418	2	2	gnl|CDD|185648	PTZ00469, PTZ00469, 60S ribo	ref|XP_001322192.1|	60S ribosomal protein L18 [Trichomonas vaginalis G3] gb|EAY09969.1| 60S ribosomal protein L18, putative [Trichomonas vaginalis G3]	1,00E-65
gALL_v3_c18419	1	4	-	-	-	-	-
gALL_v3_c1842	6	9	-	-	gb|AGM32943.1|	hypothetical protein [Coptotermes formosanus]	2,00E-06
gALL_v3_c18420	5	4	-	-	ref|XP_002425114.1|	Thyroid hormone receptor-associated protein, putative [Pediculus humanus corporis] gb|EEB12376.1| Thyroid hormone receptor-associated protein, putative [Pediculus h	1,00E-102
gALL_v3_c18421	2	3	-	-	-	-	-
gALL_v3_c18423	2	4	-	-	-	-	-
gALL_v3_c18424	0	6	-	-	ref|XP_005463598.1|	PREDICTED: probable E3 ubiquitin-protein ligase HERC1 isoform X1 [Oreochromis niloticus]	2,00E-29
gALL_v3_c18425	4	7	-	-	gb|EGI70769.1|	FLJ37770-like protein [Acromyrmex echinatior]	1,00E-12
gALL_v3_c18426	2	10	-	-	-	-	-
gALL_v3_c18427	0	4	-	-	ref|XP_002427318.1|	conserved hypothetical protein [Pediculus humanus corporis] gb|EEB14580.1| conserved hypothetical protein [Pediculus humanus corporis]	3,00E-13
gALL_v3_c18428	1	5	-	-	-	-	-
gALL_v3_c18429	2	4	-	-	-	-	-
gALL_v3_c1843	7	10	-	-	-	-	-
gALL_v3_c18431	0	6	-	-	gb|EHJ78479.1|	cytochrome P450 301B1 [Danaus plexippus]	1,00E-06
gALL_v3_c18432	2	3	-	-	-	-	-
gALL_v3_c18433	0	4	-	-	-	-	-
gALL_v3_c18434	1	4	-	-	-	-	-
gALL_v3_c18435	0	4	-	-	-	-	-
gALL_v3_c18437	3	6	-	-	-	-	-
gALL_v3_c18438	3	1	-	-	-	-	-
gALL_v3_c18439	4	4	-	-	-	-	-
gALL_v3_c18440	2	2	gnl|CDD|176013	cd04048, C2A_Copine, C2 dom	ref|XP_003703086.1|	PREDICTED: type I inositol-3,4-bisphosphate 4-phosphatase-like [Megachile rotundata]	4,00E-60
gALL_v3_c18442	2	2	-	-	-	-	-
gALL_v3_c18443	0	5	-	-	-	-	-
gALL_v3_c18445	6	10	-	-	-	-	-
gALL_v3_c18446	3	5	-	-	-	-	-
gALL_v3_c18447	6	4	gnl|CDD|48086	cd03381, PAP2_glucose_6_ph	ref|XP_004428277.1|	PREDICTED: glucose-6-phosphatase 2 isoform 1 [Ceratotherium simum simum]	9,00E-49
gALL_v3_c18448	3	6	-	-	-	-	-
gALL_v3_c18449	2	3	-	-	-	-	-
gALL_v3_c1845	10	12	gnl|CDD|189000	cd08662, M13, Peptidase fami	gb|AAN73018.1|	endothelin-converting enzyme [Locusta migratoria]	0
gALL_v3_c18450	2	3	gnl|CDD|132728	cd06930, NR_LBD_F2, Ligand-	gb|ADK46871.1|	nuclear hormone receptor FTZ-F1 beta, partial [Gryllus firmus]	2,00E-78
gALL_v3_c18451	2	2	-	-	-	-	-
gALL_v3_c18452	1	7	-	-	-	-	-
gALL_v3_c18453	8	4	-	-	-	-	-
gALL_v3_c18454	2	2	-	-	gb|ADI61823.1|	endonuclease-reverse transcriptase [Bombyx mori]	1,00E-09
gALL_v3_c18455	6	4	gnl|CDD|176072	cd08690, C2_Freud-1, C2 dom	gb|EHJ65026.1|	hypothetical protein KGM_09506 [Danaus plexippus]	9,00E-72
gALL_v3_c18457	4	2	-	-	ref|XP_006612957.1|	PREDICTED: adenomatous polyposis coli protein-like isoform X3 [Apis dorsata]	2,00E-37
gALL_v3_c18458	0	4	-	-	-	-	-
gALL_v3_c18460	0	4	-	-	-	-	-
gALL_v3_c18461	5	2	gnl|CDD|128941	smart00701, PGRP, Animal pe	ref|XP_004929966.1|	PREDICTED: peptidoglycan-recognition protein LE-like [Bombyx mori]	1,00E-13
gALL_v3_c18462	4	2	-	-	-	-	-
gALL_v3_c18463	0	6	-	-	-	-	-

gALL_v3_c18464	2	2	-	-	-	-	-
gALL_v3_c18465	1	4	-	-	-	-	-
gALL_v3_c18467	6	5	-	-	-	-	-
gALL_v3_c18469	4	2	-	-	-	-	-
gALL_v3_c1847	6	11	gnl|CDD|165506	PHA03247, PHA03247, large te	ref|XP_003633295.1|	PREDICTED: uncharacterized protein LOC100853570 [Vitis vinifera]	9,00E-07
gALL_v3_c18470	3	3	gnl|CDD|162296	TIGR01311, glycerol_kin, glyce	ref|XP_002430181.1|	glycerol kinase, putative [Pediculus humanus corporis] gb|EEB17443.1| glycerol kinase, putative [Pediculus humanus corporis]	3,00E-37
gALL_v3_c18471	4	3	-	-	ref|XP_003490942.1|	PREDICTED: hypothetical protein LOC100745580 [Bombus impatiens]	2,00E-06
gALL_v3_c18472	4	3	-	-	-	-	-
gALL_v3_c18473	2	8	gnl|CDD|29261	cd00204, ANK, ankyrin repeat	ref|XP_969896.2|	PREDICTED: similar to rolling pebbles [Tribolium castaneum]	4,00E-76
gALL_v3_c18474	5	3	-	-	-	-	-
gALL_v3_c18475	8	7	gnl|CDD|188153	TIGR01534, GAPDH-I, glycerald	dbj|BAF48668.1|	glyceraldehyde-3-phosphate dehydrogenase [Pseudotrichonympha grassii]	1,00E-128
gALL_v3_c18476	4	4	gnl|CDD|29261	cd00204, ANK, ankyrin repeat	ref|XP_002063038.1|	GK21595 [Drosophila willistoni] gb|EDW74024.1| GK21595 [Drosophila willistoni]	8,00E-87
gALL_v3_c18478	2	4	-	-	-	-	-
gALL_v3_c1848	7	17	gnl|CDD|99722	cd06401, PB1_TFG, The PB1 d	ref|XP_002423598.1|	protein TFG, putative [Pediculus humanus corporis] gb|EEB10860.1| protein TFG, putative [Pediculus humanus corporis]	4,00E-57
gALL_v3_c18480	2	4	-	-	-	-	-
gALL_v3_c18481	2	5	-	-	-	-	-
gALL_v3_c18483	3	5	gnl|CDD|192019	pfam08385, DHC_N1, Dynein	gb|EFN80965.1|	Dynein heavy chain, cytoplasmic [Harpegnathos saltator]	1,00E-107
gALL_v3_c18484	2	2	-	-	-	-	-
gALL_v3_c18485	3	3	-	-	ref|XP_003704317.1|	PREDICTED: protein ACN9 homolog, mitochondrial-like, partial [Megachile rotundata]	2,00E-21
gALL_v3_c18486	3	4	-	-	gb|EFX73121.1|	hypothetical protein DAPPUDRAFT_299659 [Daphnia pulex]	5,00E-07
gALL_v3_c18487	3	2	-	-	-	-	-
gALL_v3_c18488	2	2	-	-	-	-	-
gALL_v3_c18489	1	5	-	-	-	-	-
gALL_v3_c1849	11	9	-	-	ref|XP_002428892.1|	Hypoxia-inducible factor 1 alpha inhibitor, putative [Pediculus humanus corporis] gb|EEB16154.1| Hypoxia-inducible factor 1 alpha inhibitor, putative [Pediculus humanus	1,00E-131
gALL_v3_c18490	2	2	-	-	-	-	-
gALL_v3_c18492	3	4	-	-	ref|XP_006562874.1|	PREDICTED: transmembrane protein C9orf91 homolog isoform X4 [Apis mellifera]	1,00E-84
gALL_v3_c18493	3	1	-	-	-	-	-
gALL_v3_c18494	3	4	-	-	-	-	-
gALL_v3_c18495	3	4	-	-	ref|XP_001604905.2|	PREDICTED: WD repeat-containing protein 44-like [Nasonia vitripennis]	1,00E-23
gALL_v3_c18496	0	3	-	-	-	-	-
gALL_v3_c18497	3	2	-	-	-	-	-
gALL_v3_c18498	2	2	-	-	-	-	-
gALL_v3_c18499	2	4	-	-	-	-	-
gALL_v3_c185	9	7	-	-	-	-	-
gALL_v3_c1850	7	15	-	-	ref|XP_006823768.1|	PREDICTED: zinc finger RNA-binding protein-like [Saccoglossus kowalevskii]	7,00E-08
gALL_v3_c18500	3	1	-	-	-	-	-
gALL_v3_c18501	2	4	-	-	-	-	-
gALL_v3_c18502	4	5	-	-	-	-	-
gALL_v3_c18503	3	3	gnl|CDD|192489	pfam10235, Cript, Microtubul	ref|XP_003702361.1|	PREDICTED: cysteine-rich PDZ-binding protein-like [Megachile rotundata]	1,00E-59
gALL_v3_c18506	4	10	-	-	-	-	-
gALL_v3_c18507	3	3	-	-	-	-	-
gALL_v3_c18508	5	2	-	-	-	-	-
gALL_v3_c18509	10	15	-	-	-	-	-
gALL_v3_c18510	0	5	-	-	-	-	-
gALL_v3_c18511	1	4	-	-	-	-	-
gALL_v3_c18512	0	4	-	-	-	-	-
gALL_v3_c18513	4	5	-	-	-	-	-
gALL_v3_c18514	1	4	-	-	-	-	-
gALL_v3_c18516	3	2	-	-	ref|XP_973813.1|	PREDICTED: similar to AGAP005341-PA [Tribolium castaneum] gb|EFA02386.1| hypothetical protein TcasGA2_TC008066 [Tribolium castaneum]	1,00E-76
gALL_v3_c18517	2	1	-	-	-	-	-
gALL_v3_c18519	2	2	-	-	-	-	-
gALL_v3_c1852	9	5	gnl|CDD|128421	smart00111, C4, C-terminal ta	ref|XP_003247860.1|	PREDICTED: collagen alpha-1(IV) chain-like [Acyrthosiphon pisum]	9,00E-46
gALL_v3_c18520	2	2	-	-	-	-	-
gALL_v3_c18521	4	2	-	-	-	-	-
gALL_v3_c18522	1	3	-	-	-	-	-
gALL_v3_c18523	2	2	-	-	-	-	-
gALL_v3_c18524	3	2	gnl|CDD|189424	pfam00153, Mito_carr, Mitoch	gb|EFZ22763.1|	hypothetical protein SINV_07719 [Solenopsis invicta]	2,00E-57
gALL_v3_c18525	3	2	-	-	gb|AGM32311.1|	zinc finger-like protein, partial [Coptotermes formosanus]	2,00E-07
gALL_v3_c18526	3	6	-	-	-	-	-
gALL_v3_c18527	3	2	-	-	ref|NP_001233070.1|	uncharacterized protein LOC100162758 [Acyrthosiphon pisum] dbj|BAH71490.1| ACYPI003889 [Acyrthosiphon pisum]	3,00E-28
gALL_v3_c18528	3	4	-	-	-	-	-
gALL_v3_c18529	2	5	gnl|CDD|73156	cd01650, RT_nLTR_like, RT_nL	ref|XP_003243437.1|	PREDICTED: hypothetical protein LOC100572891 [Acyrthosiphon pisum]	8,00E-40
gALL_v3_c1853	9	13	-	-	-	-	-
gALL_v3_c18530	2	7	-	-	-	-	-
gALL_v3_c18531	3	1	gnl|CDD|150852	pfam10241, KxDL, Uncharacte	gb|AGM32611.1|	K X DL motif-containing protein [Coptotermes formosanus]	3,00E-62
gALL_v3_c18533	0	5	-	-	-	-	-
gALL_v3_c18534	1	3	-	-	-	-	-
gALL_v3_c18535	1	6	-	-	-	-	-
gALL_v3_c18537	2	3	-	-	-	-	-
gALL_v3_c18538	2	2	-	-	-	-	-
gALL_v3_c18539	1	4	gnl|CDD|29259	cd00202, ZnF_GATA, Zinc finge	gb|EFA04529.1|	serpent [Tribolium castaneum]	2,00E-42
gALL_v3_c1854	4	13	-	-	-	-	-
gALL_v3_c18540	1	5	-	-	gb|EFA07079.1|	hypothetical protein TcasGA2_TC010059 [Tribolium castaneum]	6,00E-32
gALL_v3_c18541	1	4	-	-	-	-	-
gALL_v3_c18543	2	4	-	-	-	-	-
gALL_v3_c18544	2	4	-	-	-	-	-
gALL_v3_c18545	3	1	-	-	-	-	-
gALL_v3_c18546	1	4	-	-	gb|ABB86762.2|	CYP4U3v1 [Reticulitermes flavipes]	1,00E-15
gALL_v3_c18547	2	3	-	-	-	-	-
gALL_v3_c18548	11	8	-	-	ref|YP_004198057.1|	hypothetical protein GM18_1313 [Geobacter sp. M18] ref|WP_015719640.1| hypothetical protein [Geobacter sp. M18] gb|ADW12781.1| hypothetical protein GM18_131	2,00E-09
gALL_v3_c18549	2	3	-	-	-	-	-
gALL_v3_c18550	1	4	-	-	-	-	-
gALL_v3_c18551	3	6	gnl|CDD|109294	pfam00230, MIP, Major intrins	dbj|BAG72255.1|	aquaporin [Coptotermes formosanus]	1,00E-68
gALL_v3_c18552	5	3	-	-	gb|ESN91588.1|	hypothetical protein HELRODRAFT_130122, partial [Helobdella robusta]	1,00E-11
gALL_v3_c18553	4	5	gnl|CDD|162469	TIGR01657, P-ATPase-V, P-type	gb|EGI65695.1|	Putative cation-transporting ATPase 13A1 [Acromyrmex echinatior]	1,00E-138
gALL_v3_c18554	4	3	gnl|CDD|29261	cd00204, ANK, ankyrin repeat	gb|AGM32371.1|	ankyrin repeat protein [Coptotermes formosanus]	3,00E-59
gALL_v3_c18555	2	4	-	-	-	-	-
gALL_v3_c18556	3	1	-	-	-	-	-
gALL_v3_c18557	0	5	-	-	-	-	-
gALL_v3_c18558	3	10	-	-	-	-	-
gALL_v3_c1856	22	32	-	-	-	-	-
gALL_v3_c18560	5	6	-	-	-	-	-
gALL_v3_c18561	2	3	-	-	-	-	-
gALL_v3_c18562	2	2	gnl|CDD|29151	cd00189, TPR, Tetratricopeptic	gb|ENN72543.1|	hypothetical protein YQE_10883, partial [Dendroctonus ponderosae]	1,00E-68
gALL_v3_c18563	2	2	-	-	-	-	-
gALL_v3_c18564	8	4	-	-	-	-	-
gALL_v3_c18565	1	4	-	-	gb|EHJ65748.1|	hypothetical protein KGM_03446 [Danaus plexippus]	4,00E-34
gALL_v3_c18566	1	3	-	-	-	-	-
gALL_v3_c18567	4	2	gnl|CDD|88611	cd04794, euk_LANCL, eukaryo	ref|XP_001807308.1|	PREDICTED: similar to LanC-like protein 2 (Testis-specific adriamycin sensitivity protein), partial [Tribolium castaneum]	5,00E-83
gALL_v3_c18568	1	4	-	-	-	-	-
gALL_v3_c18569	3	2	-	-	gb|AAQ19991.1|	intersectin 1 isoform 8 [Homo sapiens]	9,00E-11
gALL_v3_c1857	3	10	-	-	ref|XP_971386.1|	PREDICTED: similar to rCG37751 [Tribolium castaneum] gb|EEZ98164.1| hypothetical protein TcasGA2_TC000591 [Tribolium castaneum]	6,00E-19
gALL_v3_c18570	4	2	-	-	gb|EGI67863.1|	Mitogen-activated protein kinase kinase kinase 15 [Acromyrmex echinatior]	3,00E-56
gALL_v3_c18571	3	6	-	-	ref|XP_003697165.1|	PREDICTED: uncharacterized protein LOC100871315 [Apis florea]	6,00E-32
gALL_v3_c18573	3	4	-	-	-	-	-
gALL_v3_c18574	4	5	-	-	ref|XP_004212596.1|	PREDICTED: uncharacterized protein LOC101239283 [Hydra vulgaris]	2,00E-10
gALL_v3_c18575	4	4	-	-	gb|AGM32814.1|	G kinase-anchoring protein 1-like protein [Coptotermes formosanus]	1,00E-49
gALL_v3_c18576	0	6	-	-	-	-	-
gALL_v3_c18577	3	4	-	-	-	-	-
gALL_v3_c18578	0	5	-	-	-	-	-
gALL_v3_c18579	2	4	gnl|CDD|29261	cd00204, ANK, ankyrin repeat	gb|EHJ67371.1|	hypothetical protein KGM_19203 [Danaus plexippus]	4,00E-84
gALL_v3_c1858	4	3	-	-	-	-	-
gALL_v3_c18580	3	5	-	-	ref|XP_006571695.1|	PREDICTED: uncharacterized protein LOC413132 [Apis mellifera]	1,00E-12
gALL_v3_c18581	3	1	-	-	-	-	-
gALL_v3_c18582	4	3	-	-	ref|XP_973030.2|	PREDICTED: similar to CG8092 CG8092-PA [Tribolium castaneum] gb|EFA04153.1| hypothetical protein TcasGA2_TC014398 [Tribolium castaneum]	8,00E-06
gALL_v3_c18583	2	3	-	-	ref|XP_969282.1|	PREDICTED: similar to forkhead box transcription factor subgroup N2 [Tribolium castaneum] gb|EFA10316.1| hypothetical protein TcasGA2_TC012532 [Tribolium castaneu	6,00E-19
gALL_v3_c18584	3	6	-	-	-	-	-
gALL_v3_c18585	4	4	-	-	ref|XP_001605799.2|	PREDICTED: integrator complex subunit 6-like [Nasonia vitripennis]	1,00E-148
gALL_v3_c18587	2	7	-	-	-	-	-
gALL_v3_c18589	2	2	-	-	ref|XP_002432030.1|	hypothetical protein Phum_PHUM565670 [Pediculus humanus corporis] gb|EEB19292.1| hypothetical protein Phum_PHUM565670 [Pediculus humanus corporis]	4,00E-16
gALL_v3_c1859	32	75	-	-	gb|ABB00038.1|	reverse transcriptase family member [Glycine max]	7,00E-06
gALL_v3_c18590	0	4	-	-	gb|ENN71364.1|	hypothetical protein YQE_11979, partial [Dendroctonus ponderosae]	2,00E-26
gALL_v3_c18591	3	2	-	-	-	-	-
gALL_v3_c18592	0	6	-	-	-	-	-
gALL_v3_c18593	5	5	-	-	-	-	-
gALL_v3_c18594	3	4	-	-	-	-	-
gALL_v3_c18595	0	4	-	-	-	-	-
gALL_v3_c18596	2	3	gnl|CDD|189496	pfam00307, CH, Calponin hom	gb|EFN77269.1|	Protein vav [Harpegnathos saltator]	4,00E-54
gALL_v3_c18597	1	4	-	-	-	-	-
gALL_v3_c18598	1	4	-	-	-	-	-
gALL_v3_c18599	0	5	-	-	-	-	-
gALL_v3_c186	24	24	-	-	gb|AGM32947.1|	perilipin family protein [Coptotermes formosanus]	1,00E-54
gALL_v3_c1860	8	6	-	-	-	-	-
gALL_v3_c18600	3	1	-	-	-	-	-
gALL_v3_c18601	5	8	-	-	gb|EFA11632.1|	hypothetical protein TcasGA2_TC000010 [Tribolium castaneum]	6,00E-11
gALL_v3_c18602	1	4	-	-	-	-	-
gALL_v3_c18603	4	3	-	-	-	-	-
gALL_v3_c18604	4	8	-	-	gb|EFN79754.1|	Activating molecule in BECN1-regulated autophagy protein 1 [Harpegnathos saltator]	1,00E-16
gALL_v3_c18605	1	3	-	-	-	-	-
gALL_v3_c18606	1	3	-	-	gb|EFN71801.1|	Probable phospholipid-transporting ATPase IA [Camponotus floridanus]	3,00E-52
gALL_v3_c18607	2	3	-	-	ref|XP_006561479.1|	PREDICTED: uncharacterized protein LOC725662 [Apis mellifera]	3,00E-43
gALL_v3_c18608	3	1	-	-	-	-	-
gALL_v3_c1861	6	3	gnl|CDD|112934	pfam04142, Nuc_sug_transp,	gb|EFZ19409.1|	hypothetical protein SINV_16421 [Solenopsis invicta]	1,00E-61
gALL_v3_c18610	3	5	-	-	gb|ERL90160.1|	hypothetical protein D910_07514 [Dendroctonus ponderosae]	1,00E-16

gALL_v3_c18611	1	4	-	-	ref|XP_966570.1|	PREDICTED: similar to HEAT repeat containing 6 [Tribolium castaneum] gb|EFA08826.1| hypothetical protein TcasGA2_TC006523 [Tribolium castaneum]	2,00E-23
gALL_v3_c18612	0	4	-	-	-	-	-
gALL_v3_c18613	8	15	-	-	-	-	-
gALL_v3_c18615	4	6	-	-	gb|ERL93817.1|	hypothetical protein D910_11103 [Dendroctonus ponderosae]	5,00E-19
gALL_v3_c18616	3	6	-	-	-	-	-
gALL_v3_c18617	1	5	gnl|CDD|189424	pfam00153, Mito_carr, Mitoch	ref|XP_002426070.1|	mitochondrial dicarboxylate carrier, putative [Pediculus humanus corporis] gb|EEB13332.1| mitochondrial dicarboxylate carrier, putative [Pediculus humanus corporis]	1,00E-76
gALL_v3_c18618	5	7	-	-	-	-	-
gALL_v3_c18619	2	4	-	-	-	-	-
gALL_v3_c1862	7	7	-	-	ref|XP_002426079.1|	26S proteasome non-ATPase regulatory subunit, putative [Pediculus humanus corporis] gb|EEB13341.1| 26S proteasome non-ATPase regulatory subunit, putative [Pedicul	4,00E-62
gALL_v3_c18620	3	1	gnl|CDD|161908	TIGR00512, salvage_mtnA, S-r	ref|XP_002072272.1|	GK22419 [Drosophila willistoni] sp|B4NG41.1|MTNA_DROWI RecName: Full=Methylthioribose-1-phosphate isomerase; Short=M1Pi; Short=MTR-1-P isomerase; AltName:	3,00E-63
gALL_v3_c18621	6	15	-	-	emb|CAT00690.1|	putative hemomucin [Schistocerca gregaria]	1,00E-28
gALL_v3_c18622	2	3	-	-	-	-	-
gALL_v3_c18624	7	5	gnl|CDD|99738	cd00614, CGS_like, CGS_like: (	ref|XP_001648394.1|	cystathionine beta-lyase [Aedes aegypti] gb|EAT44603.1| AAEL004059-PB [Aedes aegypti]	1,00E-129
gALL_v3_c18625	5	2	-	-	-	-	-
gALL_v3_c18626	5	2	gnl|CDD|29261	cd00204, ANK, ankyrin repeat:	ref|XP_002426842.1|	protein phosphatase 1 regulatory subunit 16A, putative [Pediculus humanus corporis] gb|EEB14104.1| protein phosphatase 1 regulatory subunit 16A, putative [Pediculus I	1,00E-85
gALL_v3_c18627	2	2	-	-	-	-	-
gALL_v3_c18628	0	4	-	-	gb|EFA13082.1|	hypothetical protein TcasGA2_TC011582 [Tribolium castaneum]	1,00E-18
gALL_v3_c18629	2	3	-	-	-	-	-
gALL_v3_c1863	13	16	gnl|CDD|192912	pfam12003, DUF3497, Domair	ref|XP_003392997.1|	PREDICTED: latrophilin Cirl-like isoform 2 [Bombus terrestris]	0
gALL_v3_c18630	2	5	gnl|CDD|147310	pfam05063, MT-A70, MT-A70.	gb|EFZ20284.1|	hypothetical protein SINV_80518 [Solenopsis invicta]	1,00E-112
gALL_v3_c18631	6	4	gnl|CDD|145408	pfam02233, PNTB, NAD(P) trai	ref|XP_003213487.1|	PREDICTED: NAD(P) transhydrogenase, mitochondrial-like, partial [Meleagris gallopavo]	1,00E-76
gALL_v3_c18632	1	5	-	-	-	-	-
gALL_v3_c18633	0	5	-	-	-	-	-
gALL_v3_c18634	3	2	-	-	ref|XP_004211245.1|	PREDICTED: uncharacterized protein LOC101241739 [Hydra vulgaris]	5,00E-08
gALL_v3_c18635	2	3	gnl|CDD|31357	COG1163, DRG, Predicted GTP	gb|EHJ72042.1|	putative developmentally regulated GTP-binding protein 1 [Danaus plexippus]	9,00E-72
gALL_v3_c18636	1	6	-	-	-	-	-
gALL_v3_c18637	2	5	-	-	-	-	-
gALL_v3_c18638	3	1	gnl|CDD|128593	smart00298, CHROMO, Chron	ref|XP_002422576.1|	chromobox protein, putative [Pediculus humanus corporis] gb|EEB09838.1| chromobox protein, putative [Pediculus humanus corporis]	1,00E-34
gALL_v3_c18639	9	7	-	-	gb|EFA13284.1|	hypothetical protein TcasGA2_TC010304 [Tribolium castaneum]	5,00E-32
gALL_v3_c1864	10	10	gnl|CDD|179965	PRK05218, PRK05218, heat sh	ref|XP_004534550.1|	PREDICTED: heat shock protein 75 kDa, mitochondrial-like [Ceratitis capitata]	8,00E-80
gALL_v3_c18640	1	4	-	-	-	-	-
gALL_v3_c18641	6	3	-	-	-	-	-
gALL_v3_c18642	2	2	gnl|CDD|73168	cd04328, RNAP_I_Rpa43_N, R	ref|XP_003691072.1|	PREDICTED: DNA-directed RNA polymerase I subunit RPA43-like [Apis florea]	2,00E-39
gALL_v3_c18643	3	6	-	-	-	-	-
gALL_v3_c18644	5	2	-	-	ref|XP_315383.4|	AGAP005375-PA [Anopheles gambiae str. PEST] gb|EAA11292.5| AGAP005375-PA [Anopheles gambiae str. PEST]	3,00E-09
gALL_v3_c18647	1	2	-	-	-	-	-
gALL_v3_c18648	1	6	gnl|CDD|145494	pfam02376, CUT, CUT domain.	ref|XP_003706251.1|	PREDICTED: homeobox protein cut-like, partial [Megachile rotundata]	4,00E-61
gALL_v3_c18649	2	2	-	-	-	-	-
gALL_v3_c18650	0	4	-	-	gb|EFZ15092.1|	hypothetical protein SINV_12870 [Solenopsis invicta]	8,00E-30
gALL_v3_c18651	3	1	-	-	gb|EFN78775.1|	Histone-lysine N-methyltransferase SETMAR [Harpegnathos saltator]	2,00E-10
gALL_v3_c18652	3	1	-	-	-	-	-
gALL_v3_c18653	4	4	-	-	-	-	-
gALL_v3_c18655	2	3	gnl|CDD|192477	pfam10187, Nefa_Nip30_N, N	dbj|BAN21593.1|	conserved hypothetical protein, partial [Riptortus pedestris]	1,00E-35
gALL_v3_c18657	1	5	-	-	-	-	-
gALL_v3_c18658	3	1	-	-	-	-	-
gALL_v3_c18659	0	5	-	-	-	-	-
gALL_v3_c1866	6	3	-	-	ref|XP_006559241.1|	PREDICTED: uncharacterized protein LOC100577972 [Apis mellifera]	2,00E-13
gALL_v3_c18660	6	5	-	-	ref|XP_002416556.1|	cytochrome C oxidase, subunit VIb/COX12, putative [Ixodes scapularis] gb|EEC20473.1| cytochrome C oxidase, subunit VIb/COX12, putative [Ixodes scapularis]	2,00E-08
gALL_v3_c18661	6	3	gnl|CDD|165170	PHA02820, PHA02820, phospl	gb|EFN65992.1|	Phospholipase D3 [Camponotus floridanus]	7,00E-58
gALL_v3_c18662	0	6	-	-	-	-	-
gALL_v3_c18663	2	3	-	-	-	-	-
gALL_v3_c18664	3	5	-	-	-	-	-
gALL_v3_c18666	3	3	-	-	-	-	-
gALL_v3_c18668	1	3	-	-	-	-	-
gALL_v3_c18669	6	9	-	-	ref|XP_975314.1|	PREDICTED: similar to CG15449 CG15449-PA [Tribolium castaneum] gb|EFA08752.1| hypothetical protein TcasGA2_TC006429 [Tribolium castaneum]	2,00E-39
gALL_v3_c1867	8	15	-	-	-	-	-
gALL_v3_c18670	4	6	-	-	ref|XP_003699683.1|	PREDICTED: LOW QUALITY PROTEIN: MAP kinase-activating death domain protein-like [Megachile rotundata]	1,00E-163
gALL_v3_c18671	2	8	-	-	-	-	-
gALL_v3_c18672	3	9	-	-	-	-	-
gALL_v3_c18674	11	11	gnl|CDD|130026	TIGR00951, 2A43, Lysosomal (	ref|XP_003494130.1|	PREDICTED: cystinosin homolog isoform 1 [Bombus impatiens]	1,00E-133
gALL_v3_c18675	4	2	-	-	-	-	-
gALL_v3_c18677	2	2	-	-	-	-	-
gALL_v3_c18678	2	3	-	-	-	-	-
gALL_v3_c1868	10	14	-	-	-	-	-
gALL_v3_c18681	0	5	-	-	-	-	-
gALL_v3_c18682	9	9	-	-	-	-	-
gALL_v3_c18683	4	6	-	-	-	-	-
gALL_v3_c18684	3	4	gnl|CDD|73156	cd01650, RT_nLTR_like, RT_nL	gb|EFA13284.1|	hypothetical protein TcasGA2_TC010304 [Tribolium castaneum]	8,00E-45
gALL_v3_c18685	0	4	-	-	-	-	-
gALL_v3_c18686	5	5	-	-	-	-	-
gALL_v3_c18687	1	3	gnl|CDD|189706	pfam00759, Glyco_hydro_9, G	ref|XP_002426465.1|	Endoglucanase E-4 precursor, putative [Pediculus humanus corporis] gb|EEB13727.1| Endoglucanase E-4 precursor, putative [Pediculus humanus corporis]	8,00E-72
gALL_v3_c18688	2	6	-	-	-	-	-
gALL_v3_c18689	3	7	-	-	-	-	-
gALL_v3_c1869	17	13	gnl|CDD|178066	PLN02447, PLN02447, 1,4-alpl	ref|XP_002423057.1|	1,4-alpha-glucan branching enzyme, putative [Pediculus humanus corporis] gb|EEB10319.1| 1,4-alpha-glucan branching enzyme, putative [Pediculus humanus corporis]	0
gALL_v3_c18690	4	6	-	-	ref|XP_003698976.1|	PREDICTED: uncharacterized protein LOC100863626 [Apis florea]	3,00E-43
gALL_v3_c18691	2	3	-	-	ref|XP_003705348.1|	PREDICTED: WD repeat-containing protein 44-like [Megachile rotundata]	7,00E-66
gALL_v3_c18692	1	3	-	-	-	-	-
gALL_v3_c18694	1	3	-	-	-	-	-
gALL_v3_c18695	5	8	-	-	-	-	-
gALL_v3_c18696	0	4	-	-	-	-	-
gALL_v3_c18697	2	5	-	-	-	-	-
gALL_v3_c18698	3	4	gnl|CDD|113606	pfam04840, Vps16_C, Vps16, (	ref|XP_003488666.1|	PREDICTED: vacuolar protein sorting-associated protein 16 homolog [Bombus impatiens]	1,00E-79
gALL_v3_c18699	4	3	gnl|CDD|29232	cd01459, vWA_copine_like, V\	ref|XP_002425105.1|	Copine-8, putative [Pediculus humanus corporis] gb|EEB12367.1| Copine-8, putative [Pediculus humanus corporis]	7,00E-64
gALL_v3_c1870	27	52	-	-	-	-	-
gALL_v3_c18700	7	6	-	-	ref|XP_788643.3|	PREDICTED: exportin-4-like, partial [Strongylocentrotus purpuratus]	1,00E-08
gALL_v3_c18701	3	6	gnl|CDD|128524	smart00228, PDZ, Domain pre	ref|XP_002427998.1|	tight junction protein tama, putative [Pediculus humanus corporis] gb|EEB15260.1| tight junction protein tama, putative [Pediculus humanus corporis]	8,00E-78
gALL_v3_c18702	4	3	gnl|CDD|192867	pfam11894, DUF3414, Protein	ref|XP_001648879.1|	hypothetical protein AaeL_AAEL014503 [Aedes aegypti] gb|EAT33237.1| AAEL014503-PA [Aedes aegypti]	1,00E-46
gALL_v3_c18705	2	2	-	-	-	-	-
gALL_v3_c18706	1	3	-	-	-	-	-
gALL_v3_c18707	3	5	gnl|CDD|163657	cd07414, MPP_PP1_PPKL, PP1	gb|EFX67868.1|	hypothetical protein DAPPUDRAFT_231787 [Daphnia pulex]	1,00E-136
gALL_v3_c18709	2	4	-	-	gb|EFA01743.1|	hypothetical protein TcasGA2_TC007340 [Tribolium castaneum]	7,00E-16
gALL_v3_c1871	5	4	gnl|CDD|29257	cd00200, WD40, WD40 domai	gb|AGM32572.1|	WD40 domain containing protein, partial [Coptotermes formosanus]	1,00E-120
gALL_v3_c18710	2	1	-	-	-	-	-
gALL_v3_c18711	0	4	-	-	-	-	-
gALL_v3_c18713	2	3	-	-	gb|EFN74298.1|	Transcription elongation factor B polypeptide 3 [Camponotus floridanus]	6,00E-07
gALL_v3_c18715	2	3	gnl|CDD|190828	pfam04003, Utp12, Dip2/Utp1	gb|EFN68941.1|	WD repeat-containing protein 43 [Camponotus floridanus]	6,00E-37
gALL_v3_c18716	8	4	-	-	-	-	-
gALL_v3_c18717	1	3	gnl|CDD|29261	cd00204, ANK, ankyrin repeat:	ref|XP_004931592.1|	PREDICTED: ankyrin repeat domain-containing protein 54-like [Bombyx mori]	3,00E-29
gALL_v3_c18718	6	5	gnl|CDD|48458	cd03760, proteasome_beta_ty	ref|XP_001601321.1|	PREDICTED: proteasome subunit beta type-4-like [Nasonia vitripennis]	7,00E-76
gALL_v3_c18719	1	4	-	-	-	-	-
gALL_v3_c1872	5	8	gnl|CDD|185453	PTZ00110, PTZ00110, helicase	gb|EFZ16586.1|	hypothetical protein SINV_00105 [Solenopsis invicta]	2,00E-56
gALL_v3_c18720	1	2	-	-	-	-	-
gALL_v3_c18721	1	2	-	-	-	-	-
gALL_v3_c18722	1	3	-	-	ref|XP_002430793.1|	arrestin domain-containing protein, putative [Pediculus humanus corporis] gb|EEB18055.1| arrestin domain-containing protein, putative [Pediculus humanus corporis]	4,00E-35
gALL_v3_c18724	2	10	-	-	-	-	-
gALL_v3_c18725	3	2	-	-	-	-	-
gALL_v3_c18726	3	2	-	-	-	-	-
gALL_v3_c18727	2	2	-	-	-	-	-
gALL_v3_c18728	4	3	-	-	pdb|4CG5|C	Chain C, Cryo-em Of The Sec61-complex Bound To The 80s Ribosome Translating A Secretory Substrate pdb|4CG7|C Chain C, Cryo-em Of The Sec61-complex Bound To The	1,00E-11
gALL_v3_c18729	2	3	-	-	-	-	-
gALL_v3_c1873	8	16	-	-	gb|EFN80779.1|	T-cell immunomodulatory protein [Harpegnathos saltator]	1,00E-166
gALL_v3_c18731	3	3	-	-	-	-	-
gALL_v3_c18732	2	3	-	-	-	-	-
gALL_v3_c18733	5	3	gnl|CDD|178391	PLN02794, PLN02794, cardioli	ref|NP_001164128.1|	cardiolipin synthase 1 [Tribolium castaneum]	3,00E-73
gALL_v3_c18734	3	1	-	-	-	-	-
gALL_v3_c18735	3	6	-	-	gb|EEZ98539.1|	hypothetical protein TcasGA2_TC001043 [Tribolium castaneum]	3,00E-18
gALL_v3_c18737	5	2	-	-	ref|YP_003844767.1|	cell surface protein [Clostridium cellulovorans 743B] ref|WP_010073399.1| cell surface protein [Clostridium cellulovorans] gb|ADL53003.1| cell surface protein [Clostridiu	6,00E-14
gALL_v3_c18738	5	6	gnl|CDD|189864	pfam01159, Ribosomal_L6e, R	gb|AGM32491.1|	60S ribosomal protein L6 [Coptotermes formosanus]	3,00E-79
gALL_v3_c18739	1	5	-	-	-	-	-
gALL_v3_c1874	5	3	-	-	-	-	-
gALL_v3_c18740	0	4	-	-	-	-	-
gALL_v3_c18741	2	2	-	-	-	-	-
gALL_v3_c18742	0	6	-	-	ref|XP_975636.1|	PREDICTED: similar to hormone-sensitive lipase [Tribolium castaneum] gb|EFA02512.1| hypothetical protein TcasGA2_TC008216 [Tribolium castaneum]	1,00E-13
gALL_v3_c18743	2	2	-	-	-	-	-
gALL_v3_c18744	6	5	-	-	-	-	-
gALL_v3_c18745	0	6	-	-	ref|XP_003401426.1|	PREDICTED: hypothetical protein LOC100646364 [Bombus terrestris]	3,00E-24
gALL_v3_c18746	3	10	-	-	-	-	-
gALL_v3_c18748	4	4	-	-	ref|XP_003227027.1|	PREDICTED: zinc finger protein 91-like [Anolis carolinensis]	1,00E-55
gALL_v3_c18749	2	2	-	-	-	-	-
gALL_v3_c1875	13	9	gnl|CDD|29332	cd00257, Fascin, Fascin-like dc	gb|AFZ78853.1|	ricin-type beta-trefoil lectin domain-containing protein [Coptotermes formosanus]	8,00E-88
gALL_v3_c18750	2	3	-	-	-	-	-
gALL_v3_c18753	4	7	-	-	-	-	-
gALL_v3_c18754	2	4	-	-	-	-	-
gALL_v3_c18755	3	3	gnl|CDD|143812	pfam00022, Actin, Actin.	gb|EGI70016.1|	Actin-like protein 6A [Acromyrmex echinatior]	1,00E-94
gALL_v3_c18756	2	2	-	-	-	-	-
gALL_v3_c18757	4	6	-	-	ref|XP_308323.4|	AGAP007556-PA [Anopheles gambiae str. PEST] gb|EAA04475.5| AGAP007556-PA [Anopheles gambiae str. PEST]	1,00E-09
gALL_v3_c18758	4	4	-	-	-	-	-
gALL_v3_c18759	2	2	-	-	-	-	-
gALL_v3_c1876	9	21	gnl|CDD|191809	pfam07678, A2M_comp, A-ma	ref|XP_006621187.1|	PREDICTED: CD109 antigen-like isoform X1 [Apis dorsata] ref|XP_006621188.1| PREDICTED: CD109 antigen-like isoform X2 [Apis dorsata]	0
gALL_v3_c18760	3	2	-	-	ref|XP_004305112.1|	PREDICTED: uncharacterized protein LOC101308403 [Fragaria vesca subsp. vesca]	5,00E-31
gALL_v3_c18761	3	2	-	-	-	-	-
gALL_v3_c18762	2	2	-	-	-	-	-
gALL_v3_c18763	1	3	-	-	-	-	-
gALL_v3_c18765	1	5	-	-	-	-	-

gALL_v3_c18766	2	3	-	-	-	-	-
gALL_v3_c18767	3	2	-	-	ref|XP_001816265.1|	PREDICTED: similar to zinc finger protein 474 [Tribolium castaneum]	2,00E-42
gALL_v3_c18768	3	5	gnl|CDD|189973	pfam01399, PCI, PCI domain.	ref|XP_002432576.1|	COP9 signalosome complex subunit, putative [Pediculus humanus corporis] gb|EEB19838.1| COP9 signalosome complex subunit, putative [Pediculus humanus corporis]	1,00E-116
gALL_v3_c18769	1	3	-	-	-	-	-
gALL_v3_c1877	7	8	gnl|CDD|146328	pfam03630, Fumble, Fumble.	ref|XP_003425841.1|	PREDICTED: pantothenate kinase 2, mitochondrial-like isoform 2 [Nasonia vitripennis] ref|XP_001604541.2| PREDICTED: pantothenate kinase 2, mitochondrial-like isoform	2,00E-99
gALL_v3_c18771	4	2	-	-	ref|XP_001662533.1|	hypothetical protein AaeL_AAEL012389 [Aedes aegypti] gb|EAT35438.1| AAEL012389-PA [Aedes aegypti]	5,00E-47
gALL_v3_c18772	1	3	-	-	-	-	-
gALL_v3_c18773	3	7	-	-	-	-	-
gALL_v3_c18774	4	5	-	-	ref|XP_002997836.1|	helitron helicase-like protein [Phytophthora infestans T30-4] gb|EEY67811.1| helitron helicase-like protein [Phytophthora infestans T30-4]	3,00E-19
gALL_v3_c18777	2	2	gnl|CDD|189654	pfam00651, BTB, BTB/POZ dor	gb|EHJ66622.1|	hypothetical protein KGM_08757 [Danaus plexippus]	2,00E-47
gALL_v3_c1878	6	13	gnl|CDD|189954	pfam01359, Transposase_1, Tr	emb|CAP20051.1|	transposase [Portunus pelagicus]	4,00E-55
gALL_v3_c18780	1	5	-	-	-	-	-
gALL_v3_c18781	5	5	-	-	gb|ESN91472.1|	hypothetical protein HELRODRAFT_165510 [Helobdella robusta]	4,00E-14
gALL_v3_c18782	3	3	-	-	ref|XP_001641116.1|	predicted protein [Nematostella vectensis] gb|EDO49053.1| predicted protein [Nematostella vectensis]	4,00E-10
gALL_v3_c18783	4	3	-	-	-	-	-
gALL_v3_c18784	8	6	-	-	-	-	-
gALL_v3_c18786	3	6	-	-	-	-	-
gALL_v3_c18787	3	3	gnl|CDD|176068	cd08686, C2_ABR, C2 domain	ref|XP_002424349.1|	breakpoint cluster region protein, putative [Pediculus humanus corporis] gb|EEB11611.1| breakpoint cluster region protein, putative [Pediculus humanus corporis]	3,00E-73
gALL_v3_c18788	4	2	-	-	-	-	-
gALL_v3_c18789	3	1	-	-	-	-	-
gALL_v3_c1879	4	3	-	-	gb|EFX75731.1|	hypothetical protein DAPPUDRAFT_323098 [Daphnia pulex]	3,00E-07
gALL_v3_c18790	2	3	-	-	-	-	-
gALL_v3_c18791	3	12	-	-	ref|XP_003702583.1|	PREDICTED: histone-lysine N-methyltransferase SETMAR-like [Megachile rotundata]	2,00E-56
gALL_v3_c18792	4	4	gnl|CDD|30753	COG0404, GcvT, Glycine cleava	gb|EFX72833.1|	hypothetical protein DAPPUDRAFT_308047 [Daphnia pulex]	1,00E-55
gALL_v3_c18793	0	5	-	-	-	-	-
gALL_v3_c18794	2	3	-	-	dbj|BAC82628.1|	pol-like protein [Anopheles gambiae]	7,00E-16
gALL_v3_c18795	2	3	gnl|CDD|189583	pfam00520, Ion_trans, Ion tra	gb|ABE60888.1|	voltage-gated sodium channel alpha subunit [Helicoverpa armigera]	6,00E-96
gALL_v3_c18796	1	3	gnl|CDD|189334	pfam00001, 7tm_1, 7 transme	ref|XP_001651713.1|	beta adrenergic receptor [Aedes aegypti]	8,00E-55
gALL_v3_c18798	5	5	-	-	-	-	-
gALL_v3_c18799	1	3	-	-	-	-	-
gALL_v3_c188	3	6	-	-	-	-	-
gALL_v3_c1880	9	18	gnl|CDD|128827	smart00554, FAS1, Four repea	ref|XP_969478.1|	PREDICTED: similar to CG5758 CG5758-PA [Tribolium castaneum] gb|EFA04327.1| hypothetical protein TcasGA2_TC014621 [Tribolium castaneum]	1,00E-40
gALL_v3_c18800	2	9	-	-	ref|XP_003707987.1|	PREDICTED: uncharacterized protein LOC100878272 [Megachile rotundata]	5,00E-60
gALL_v3_c18801	5	2	gnl|CDD|189366	pfam00054, Laminin_G_1, Lar	ref|XP_002429083.1|	slit protein, putative [Pediculus humanus corporis] gb|EEB16345.1| slit protein, putative [Pediculus humanus corporis]	3,00E-96
gALL_v3_c18802	2	2	-	-	ref|XP_003408334.1|	PREDICTED: transmembrane protein 65-like [Loxodonta africana]	1,00E-07
gALL_v3_c18803	24	26	-	-	-	-	-
gALL_v3_c18804	2	7	-	-	ref|XP_001605401.2|	PREDICTED: metal transporter CNNM2-like [Nasonia vitripennis]	2,00E-38
gALL_v3_c18806	2	3	-	-	ref|XP_001942734.1|	PREDICTED: hypothetical protein LOC100160904 [Acyrthosiphon pisum]	3,00E-17
gALL_v3_c18807	2	3	-	-	-	-	-
gALL_v3_c18808	0	4	-	-	ref|XP_002434328.1|	ATP-dependent RNA helicase, putative [Ixodes scapularis] gb|EEC07152.1| ATP-dependent RNA helicase, putative [Ixodes scapularis]	8,00E-20
gALL_v3_c18809	1	3	-	-	-	-	-
gALL_v3_c1881	4	7	-	-	-	-	-
gALL_v3_c18810	2	4	-	-	-	-	-
gALL_v3_c18811	4	5	-	-	-	-	-
gALL_v3_c18812	2	1	-	-	ref|XP_004924396.1|	PREDICTED: uncharacterized protein LOC101737675 [Bombyx mori]	6,00E-08
gALL_v3_c18814	3	1	gnl|CDD|190408	pfam02752, Arrestin_C, Arrest	ref|XP_001998608.1|	GI23546 [Drosophila mojavensis] gb|EDW14069.1| GI23546 [Drosophila mojavensis]	4,00E-29
gALL_v3_c18815	1	6	-	-	-	-	-
gALL_v3_c18816	5	4	-	-	gb|EKM80493.1|	hypothetical protein AGABI1DRAFT_113660 [Agaricus bisporus var. burnettii JB137-S8]	3,00E-07
gALL_v3_c18817	5	3	-	-	-	-	-
gALL_v3_c18818	3	5	-	-	-	-	-
gALL_v3_c18819	0	5	-	-	-	-	-
gALL_v3_c1882	17	11	-	-	ref|XP_003485835.1|	PREDICTED: hypothetical protein LOC100743966 [Bombus impatiens]	1,00E-61
gALL_v3_c18820	0	4	-	-	-	-	-
gALL_v3_c18821	1	3	-	-	-	-	-
gALL_v3_c18822	4	2	gnl|CDD|151191	pfam10695, Cw-hydrolase, Cel	ref|WP_008866972.1|	hydrolase [Flavobacteria bacterium MS024-3C] gb|EEG43412.1| conserved hypothetical protein [Flavobacteria bacterium MS024-3C]	3,00E-63
gALL_v3_c18823	2	5	-	-	-	-	-
gALL_v3_c18824	1	4	-	-	ref|XP_003241651.1|	PREDICTED: hypothetical protein LOC100571806 [Acyrthosiphon pisum]	4,00E-14
gALL_v3_c18826	2	4	-	-	-	-	-
gALL_v3_c18828	0	5	-	-	gb|EFZ13178.1|	hypothetical protein SINV_00703 [Solenopsis invicta]	2,00E-30
gALL_v3_c1883	15	9	gnl|CDD|162215	TIGR01130, ER_PDI_fam, prot	gb|AEV89748.1|	protein disulfide-isomerase [Schistocerca gregaria]	3,00E-43
gALL_v3_c18830	2	2	-	-	-	-	-
gALL_v3_c18831	2	4	-	-	-	-	-
gALL_v3_c18832	4	5	-	-	-	-	-
gALL_v3_c18835	2	5	-	-	-	-	-
gALL_v3_c18836	6	6	-	-	-	-	-
gALL_v3_c18837	2	2	-	-	ref|XP_001846969.1|	conserved hypothetical protein [Culex quinquefasciatus] gb|EDS45211.1| conserved hypothetical protein [Culex quinquefasciatus]	5,00E-12
gALL_v3_c18838	3	4	-	-	-	-	-
gALL_v3_c18839	4	2	-	-	ref|XP_001950809.2|	PREDICTED: hexosaminidase D-like [Acyrthosiphon pisum]	8,00E-12
gALL_v3_c1884	12	9	-	-	-	-	-
gALL_v3_c18840	1	3	-	-	-	-	-
gALL_v3_c18841	3	2	-	-	-	-	-
gALL_v3_c18842	2	2	-	-	gb|AGO88040.1|	hypothetical protein [uncultured bacterium 122006-I05]	8,00E-36
gALL_v3_c18843	1	3	-	-	-	-	-
gALL_v3_c18844	3	2	-	-	-	-	-
gALL_v3_c18845	2	6	-	-	gb|AGM32821.1|	hypothetical protein [Coptotermes formosanus]	8,00E-14
gALL_v3_c18846	1	4	-	-	gb|EFA13284.1|	hypothetical protein TcasGA2_TC010304 [Tribolium castaneum]	3,00E-23
gALL_v3_c18847	1	5	-	-	-	-	-
gALL_v3_c18848	3	3	-	-	gb|EFN75044.1|	Uncharacterized protein KIAA1109 [Camponotus floridanus]	8,00E-85
gALL_v3_c18849	2	4	-	-	-	-	-
gALL_v3_c1885	7	4	gnl|CDD|181810	PRK09377, tsf, elongation fact	gb|ENN74820.1|	hypothetical protein YQE_08593, partial [Dendroctonus ponderosae] gb|ERL86096.1| hypothetical protein D910_03510 [Dendroctonus ponderosae]	1,00E-62
gALL_v3_c18850	1	5	-	-	-	-	-
gALL_v3_c18852	1	4	gnl|CDD|30100	cd01378, MYSc_type_I, Myosi	ref|XP_003395685.1|	PREDICTED: myosin-IA-like [Bombus terrestris]	1,00E-82
gALL_v3_c18853	2	2	-	-	-	-	-
gALL_v3_c18855	1	3	-	-	-	-	-
gALL_v3_c18856	0	3	-	-	-	-	-
gALL_v3_c18857	4	3	-	-	ref|XP_001607596.2|	PREDICTED: spectrin beta chain-like [Nasonia vitripennis]	7,00E-10
gALL_v3_c18858	1	5	-	-	-	-	-
gALL_v3_c18859	6	6	-	-	-	-	-
gALL_v3_c1886	15	13	-	-	-	-	-
gALL_v3_c18860	3	7	-	-	-	-	-
gALL_v3_c18861	2	8	-	-	-	-	-
gALL_v3_c18862	0	4	-	-	ref|XP_003701384.1|	PREDICTED: LOW QUALITY PROTEIN: protein unc-80 homolog [Megachile rotundata]	1,00E-100
gALL_v3_c18863	2	2	-	-	-	-	-
gALL_v3_c18864	3	1	-	-	-	-	-
gALL_v3_c18867	2	5	-	-	-	-	-
gALL_v3_c18868	3	4	-	-	-	-	-
gALL_v3_c18869	6	5	-	-	-	-	-
gALL_v3_c18870	5	11	gnl|CDD|191268	pfam05405, Mt_ATP-synt_B, M	ref|XP_001847481.1|	ATP synthase B chain, mitochondrial [Culex quinquefasciatus] gb|EDS26265.1| ATP synthase B chain, mitochondrial [Culex quinquefasciatus]	1,00E-50
gALL_v3_c18871	4	2	-	-	-	-	-
gALL_v3_c18872	5	6	gnl|CDD|188153	TIGR01534, GAPDH-I, glycerald	dbj|BAF48668.1|	glyceraldehyde-3-phosphate dehydrogenase [Pseudotrichonympha grassii]	1,00E-121
gALL_v3_c18873	5	7	-	-	ref|XP_003492249.1|	PREDICTED: zinc finger SWIM domain-containing protein KIAA0913-like [Bombus impatiens]	0
gALL_v3_c18874	2	5	gnl|CDD|34783	COG5184, ATS1, Alpha-tubulin	gb|AHJ25737.1|	HERC1, partial [Panthera tigris altaica]	2,00E-92
gALL_v3_c18875	3	3	-	-	-	-	-
gALL_v3_c18876	2	7	-	-	-	-	-
gALL_v3_c18877	1	5	-	-	-	-	-
gALL_v3_c18878	4	2	-	-	-	-	-
gALL_v3_c1888	11	10	-	-	ref|XP_002424403.1|	conserved hypothetical protein [Pediculus humanus corporis] gb|EEB11665.1| conserved hypothetical protein [Pediculus humanus corporis]	8,00E-68
gALL_v3_c18880	2	3	gnl|CDD|191069	pfam04718, ATP-synt_G, Mito	gb|AGM32841.1|	putative hydrogen-transporting ATP synthase, G-subunit [Coptotermes formosanus]	7,00E-65
gALL_v3_c18881	2	3	-	-	-	-	-
gALL_v3_c18882	3	2	gnl|CDD|191704	pfam07223, DUF1421, Protein	-	-	-
gALL_v3_c18883	2	3	-	-	-	-	-
gALL_v3_c18885	1	6	-	-	-	-	-
gALL_v3_c18886	3	1	gnl|CDD|189477	pfam00262, Calreticulin, Calre	ref|XP_001325490.1|	Calreticulin family protein [Trichomonas vaginalis G3] gb|EAY13267.1| Calreticulin family protein [Trichomonas vaginalis G3]	5,00E-70
gALL_v3_c18887	6	4	-	-	-	-	-
gALL_v3_c18888	5	12	gnl|CDD|191180	pfam05057, DUF676, Putative	gb|ABE01185.1|	IP15632p [Drosophila melanogaster]	1,00E-150
gALL_v3_c18889	4	6	-	-	gb|AEW67357.1|	trehalose-6-phosphate phosphatase, partial [Coptotermes formosanus]	1,00E-100
gALL_v3_c1889	6	3	-	-	-	-	-
gALL_v3_c18890	1	6	-	-	gb|EHJ73779.1|	putative ubiquitin specific protease 34 [Danaus plexippus]	2,00E-26
gALL_v3_c18891	2	5	-	-	gb|EFN83345.1|	X-linked retinitis pigmentosa GTPase regulator-like protein [Harpegnathos saltator]	5,00E-17
gALL_v3_c18893	1	6	-	-	ref|XP_006565537.1|	PREDICTED: aminopeptidase N-like isoform X2 [Apis mellifera]	2,00E-74
gALL_v3_c18894	1	4	-	-	-	-	-
gALL_v3_c18895	1	5	-	-	ref|XP_001845146.1|	chromodomain helicase DNA binding protein [Culex quinquefasciatus] gb|EDS39310.1| chromodomain helicase DNA binding protein [Culex quinquefasciatus]	5,00E-15
gALL_v3_c18896	2	3	-	-	-	-	-
gALL_v3_c18897	0	4	-	-	-	-	-
gALL_v3_c18898	2	3	-	-	ref|XP_966538.2|	PREDICTED: similar to lachesin, putative [Tribolium castaneum]	1,00E-68
gALL_v3_c18899	1	6	-	-	ref|XP_003426693.1|	PREDICTED: hypothetical protein LOC100678217 [Nasonia vitripennis]	2,00E-22
gALL_v3_c189	7	5	-	-	ref|XP_003243730.1|	PREDICTED: hypothetical protein LOC100573801 [Acyrthosiphon pisum]	1,00E-07
gALL_v3_c1890	5	7	gnl|CDD|34626	COG5021, HUL4, Ubiquitin-pro	dbj|BAM19251.1|	suppressor of deltex [Papilio polytes]	3,00E-36
gALL_v3_c18900	0	4	-	-	-	-	-
gALL_v3_c18901	0	4	-	-	-	-	-
gALL_v3_c18902	2	3	gnl|CDD|28914	cd00032, CASc, Caspase, inter	ref|XP_005174928.1|	PREDICTED: caspase-8-like [Musca domestica]	2,00E-09
gALL_v3_c18903	3	7	-	-	-	-	-
gALL_v3_c18905	0	5	-	-	-	-	-
gALL_v3_c18906	2	2	gnl|CDD|187553	cd05242, SDR_a8, atypical (a)	ref|XP_002427474.1|	conserved hypothetical protein [Pediculus humanus corporis] gb|EEB14736.1| conserved hypothetical protein [Pediculus humanus corporis]	1,00E-34
gALL_v3_c18908	4	5	-	-	-	-	-
gALL_v3_c1891	20	29	-	-	ref|XP_002424549.1|	conserved hypothetical protein [Pediculus humanus corporis] gb|EEB11811.1| conserved hypothetical protein [Pediculus humanus corporis]	1,00E-44
gALL_v3_c18910	4	6	-	-	-	-	-
gALL_v3_c18911	4	4	gnl|CDD|190411	pfam02759, RUN, RUN domain	ref|XP_003395792.1|	PREDICTED: hypothetical protein LOC100648360 [Bombus terrestris]	4,00E-31
gALL_v3_c18912	1	4	-	-	ref|XP_001845217.1|	150 kDa dynein-associated polypeptide [Culex quinquefasciatus] gb|EDS39730.1| 150 kDa dynein-associated polypeptide [Culex quinquefasciatus]	1,00E-15
gALL_v3_c18913	3	2	-	-	-	-	-
gALL_v3_c18914	1	4	-	-	-	-	-
gALL_v3_c18915	5	7	-	-	gb|EIW10742.1|	Bud27p [Saccharomyces cerevisiae CEN.PK113-7D]	1,00E-09
gALL_v3_c18916	5	3	-	-	gb|EFN64467.1|	Serine/threonine-protein kinase QSK [Camponotus floridanus]	2,00E-17
gALL_v3_c18918	1	5	-	-	-	-	-

gALL_v3_c18919	6	5	-	-	-	-	-
gALL_v3_c1892	6	9	gnl|CDD|191619	pfam06840, DUF1241, Protein	dbj|BAN21091.1|	conserved hypothetical protein [Riptortus pedestris]	1,00E-123
gALL_v3_c18920	0	5	gnl|CDD|144027	pfam00287, Na_K-ATPase, Soc	gb|AHH35012.1|	Na+/K+-ATPase beta1 [Locusta migratoria]	2,00E-21
gALL_v3_c18921	1	7	-	-	ref|XP_002427867.1|	rap GTPase-activating protein, putative [Pediculus humanus corporis] gb|EEB15129.1| rap GTPase-activating protein, putative [Pediculus humanus corporis]	2,00E-63
gALL_v3_c18922	2	5	-	-	-	-	-
gALL_v3_c18923	5	2	-	-	-	-	-
gALL_v3_c18924	3	2	-	-	-	-	-
gALL_v3_c18925	4	10	-	-	gb|AGM32905.1|	hypothetical protein [Coptotermes formosanus]	5,00E-08
gALL_v3_c18926	11	9	-	-	-	-	-
gALL_v3_c18927	2	4	-	-	-	-	-
gALL_v3_c18928	0	4	gnl|CDD|147673	pfam05640, NKAIN, Na,K-Atpa	ref|XP_001849987.1|	conserved hypothetical protein [Culex quinquefasciatus] gb|EDS31145.1| conserved hypothetical protein [Culex quinquefasciatus]	8,00E-21
gALL_v3_c18929	3	3	-	-	-	-	-
gALL_v3_c1893	7	6	gnl|CDD|100052	cd04646, LbH_Dynactin_6, Dy	gb|ELU06414.1|	hypothetical protein CAPTEDRAFT_173776 [Capitella teleta]	9,00E-55
gALL_v3_c18930	4	2	-	-	-	-	-
gALL_v3_c18931	3	1	-	-	ref|XP_006823070.1|	PREDICTED: TATA element modulatory factor-like [Saccoglossus kowalevskii]	8,00E-16
gALL_v3_c18932	3	2	-	-	ref|XP_003384033.1|	PREDICTED: hypothetical protein LOC100631389 [Amphimedon queenslandica]	4,00E-16
gALL_v3_c18933	4	8	-	-	-	-	-
gALL_v3_c18934	3	4	-	-	-	-	-
gALL_v3_c18935	2	2	-	-	-	-	-
gALL_v3_c18936	0	5	-	-	-	-	-
gALL_v3_c18937	3	1	gnl|CDD|189910	pfam01255, Prenyltransf, Puta	gb|EKC39111.1|	Dehydrodolichyl diphosphate synthase [Crassostrea gigas]	7,00E-34
gALL_v3_c18938	5	2	-	-	-	-	-
gALL_v3_c18939	5	4	-	-	-	-	-
gALL_v3_c1894	13	19	-	-	-	-	-
gALL_v3_c18940	1	4	-	-	-	-	-
gALL_v3_c18941	2	5	gnl|CDD|128489	smart00192, LDLa, Low-densit	ref|XP_001660469.1|	low-density lipoprotein receptor (ldl) [Aedes aegypti] gb|EAT38205.1| AAEL009869-PA [Aedes aegypti]	6,00E-65
gALL_v3_c18942	1	8	-	-	-	-	-
gALL_v3_c18943	4	6	-	-	ref|XP_002424692.1|	hypothetical protein Phum_PHUM146390 [Pediculus humanus corporis] gb|EEB11954.1| hypothetical protein Phum_PHUM146390 [Pediculus humanus corporis]	2,00E-10
gALL_v3_c18944	1	5	-	-	gb|EFN78328.1|	BTB/POZ domain-containing protein KCTD15 [Harpegnathos saltator]	7,00E-61
gALL_v3_c18945	1	3	-	-	-	-	-
gALL_v3_c18946	2	3	-	-	-	-	-
gALL_v3_c18949	2	1	-	-	-	-	-
gALL_v3_c1895	16	13	-	-	-	-	-
gALL_v3_c18950	1	3	-	-	-	-	-
gALL_v3_c18951	1	4	gnl|CDD|190619	pfam03378, CAS_CSE1, CAS/C	ref|XP_001606668.1|	PREDICTED: exportin-2-like [Nasonia vitripennis]	3,00E-59
gALL_v3_c18952	2	3	-	-	-	-	-
gALL_v3_c18954	0	5	-	-	-	-	-
gALL_v3_c18957	3	3	-	-	-	-	-
gALL_v3_c18959	6	4	-	-	-	-	-
gALL_v3_c1896	8	12	-	-	ref|XP_003700938.1|	PREDICTED: uncharacterized protein LOC100877871 [Megachile rotundata]	2,00E-18
gALL_v3_c18960	2	6	-	-	ref|XP_002431856.1|	zinc finger protein, putative [Pediculus humanus corporis] gb|EEB19118.1| zinc finger protein, putative [Pediculus humanus corporis]	5,00E-47
gALL_v3_c18961	10	10	-	-	-	-	-
gALL_v3_c18962	3	2	gnl|CDD|73012	cd03253, ABCC_ATM1_transp	gb|EFZ19311.1|	hypothetical protein SINV_13547 [Solenopsis invicta]	7,00E-47
gALL_v3_c18963	0	3	-	-	-	-	-
gALL_v3_c18965	2	2	-	-	-	-	-
gALL_v3_c18967	2	5	gnl|CDD|183327	PRK11824, PRK11824, polynuc	ref|XP_974155.1|	PREDICTED: similar to CG11337 CG11337-PA [Tribolium castaneum] gb|EFA06447.1| hypothetical protein TcasGA2_TC009329 [Tribolium castaneum]	2,00E-72
gALL_v3_c18969	5	11	-	-	-	-	-
gALL_v3_c1897	11	8	gnl|CDD|178780	PLN03242, PLN03242, diacylgl	gb|EKC38513.1|	Sterol O-acyltransferase 1 [Crassostrea gigas]	2,00E-62
gALL_v3_c18970	2	2	gnl|CDD|191125	pfam04910, DUF654, Protein (	gb|EFA01445.1|	hypothetical protein TcasGA2_TC030734 [Tribolium castaneum]	2,00E-61
gALL_v3_c18971	0	3	-	-	-	-	-
gALL_v3_c18972	1	3	-	-	-	-	-
gALL_v3_c18977	1	5	gnl|CDD|187704	cd09280, RNase_HI_eukaryote	ref|XP_003394155.1|	PREDICTED: ribonuclease H1-like isoform 1 [Bombus terrestris] ref|XP_003394156.1| PREDICTED: ribonuclease H1-like isoform 2 [Bombus terrestris]	4,00E-13
gALL_v3_c18978	3	3	-	-	-	-	-
gALL_v3_c18979	2	2	-	-	-	-	-
gALL_v3_c1898	9	19	gnl|CDD|187547	cd05236, FAR-N_SDR_e, fatty	ref|XP_004517505.1|	PREDICTED: putative fatty acyl-CoA reductase CG8306-like [Ceratitis capitata]	1,00E-103
gALL_v3_c18980	2	4	-	-	-	-	-
gALL_v3_c18981	3	4	-	-	-	-	-
gALL_v3_c18982	1	3	-	-	-	-	-
gALL_v3_c18983	2	3	-	-	-	-	-
gALL_v3_c18984	2	3	-	-	ref|XP_002427998.1|	tight junction protein tama, putative [Pediculus humanus corporis] gb|EEB15260.1| tight junction protein tama, putative [Pediculus humanus corporis]	2,00E-31
gALL_v3_c18986	1	4	-	-	-	-	-
gALL_v3_c18987	1	6	-	-	-	-	-
gALL_v3_c18988	2	2	-	-	ref|XP_006621613.1|	PREDICTED: cyclin-dependent kinase 11B-like [Apis dorsata]	6,00E-07
gALL_v3_c18989	10	10	-	-	gb|AGM32821.1|	hypothetical protein [Coptotermes formosanus]	2,00E-07
gALL_v3_c1899	7	13	gnl|CDD|176023	cd08377, C2C_MCTP_PRT, C2	ref|XP_003484702.1|	PREDICTED: multiple C2 and transmembrane domain-containing protein 1-like [Bombus impatiens]	0
gALL_v3_c18990	1	3	-	-	gb|EFZ16950.1|	hypothetical protein SINV_15000 [Solenopsis invicta]	3,00E-36
gALL_v3_c18991	7	4	gnl|CDD|34862	COG5265, ATM1, ABC-type tra	ref|XP_001813375.1|	PREDICTED: similar to ATP-binding cassette sub-family B member 7 [Tribolium castaneum] gb|EFA04501.1| hypothetical protein TcasGA2_TC014809 [Tribolium castaneum]	0
gALL_v3_c18992	2	7	-	-	-	-	-
gALL_v3_c18993	0	4	gnl|CDD|152675	pfam12240, Angiomotin_C, Ar	dbj|BAN09206.1|	angiomotin [Gryllus bimaculatus]	2,00E-42
gALL_v3_c18995	2	4	-	-	-	-	-
gALL_v3_c18996	3	4	-	-	-	-	-
gALL_v3_c18997	3	1	-	-	-	-	-
gALL_v3_c18999	3	5	-	-	-	-	-
gALL_v3_c190	4	5	gnl|CDD|188938	cd09539, SAM_TNK-like, SAM	ref|XP_006607313.1|	PREDICTED: tyrosine-protein kinase PR2-like [Apis dorsata]	1,00E-78
gALL_v3_c1900	8	7	gnl|CDD|193336	pfam12862, Apc5, Anaphase-p	ref|XP_967402.2|	PREDICTED: similar to anaphase-promoting complex, subunit-5, putative [Tribolium castaneum]	1,00E-81
gALL_v3_c19000	8	7	gnl|CDD|143979	pfam00224, PK, Pyruvate kina	gb|AEE62869.1|	unknown [Dendroctonus ponderosae] gb|ENN72684.1| hypothetical protein YQE_10780, partial [Dendroctonus ponderosae] gb|ERL85807.1| hypothetical protein D910_(	1,00E-152
gALL_v3_c19002	2	5	gnl|CDD|190542	pfam03145, Sina, Seven in abs	gb|AGM32764.1|	seven in absentia protein family [Coptotermes formosanus]	9,00E-63
gALL_v3_c19003	1	4	-	-	-	-	-
gALL_v3_c19005	0	3	-	-	-	-	-
gALL_v3_c19006	0	5	-	-	ref|XP_002429320.1|	NADH-Ubiquinone oxidoreductase AGGG subunit [Pediculus humanus corporis] gb|EEB16582.1| NADH-Ubiquinone oxidoreductase AGGG subunit [Pediculus humanus cor	6,00E-26
gALL_v3_c19007	2	2	gnl|CDD|179368	PRK02106, PRK02106, choline	ref|XP_002429699.1|	glucose dehydrogenase precursor, putative [Pediculus humanus corporis] gb|EEB16961.1| glucose dehydrogenase precursor, putative [Pediculus humanus corporis]	3,00E-61
gALL_v3_c19008	2	4	-	-	-	-	-
gALL_v3_c19009	3	2	-	-	-	-	-
gALL_v3_c1901	6	3	-	-	ref|XP_001606087.2|	PREDICTED: zinc finger and BTB domain-containing protein 17-like [Nasonia vitripennis]	4,00E-18
gALL_v3_c19010	3	1	-	-	-	-	-
gALL_v3_c19011	1	3	gnl|CDD|191908	pfam07970, COPIIcoated_ERV,	gb|ENN77656.1|	hypothetical protein YQE_05950, partial [Dendroctonus ponderosae]	2,00E-49
gALL_v3_c19013	19	17	-	-	gb|AGM32716.1|	uncharacterized protein [Coptotermes formosanus]	1,00E-105
gALL_v3_c19014	2	3	gnl|CDD|149077	pfam07810, TMC, TMC domai	ref|XP_320512.3|	AGAP012019-PA [Anopheles gambiae str. PEST] gb|AAP78792.1| TmcC-like protein [Anopheles gambiae] gb|EAA00457.3| AGAP012019-PA [Anopheles gambiae str. PEST]	8,00E-34
gALL_v3_c19015	0	3	-	-	-	-	-
gALL_v3_c19016	2	1	-	-	-	-	-
gALL_v3_c19017	2	2	-	-	gb|EFA03042.1|	hypothetical protein TcasGA2_TC010468 [Tribolium castaneum]	4,00E-22
gALL_v3_c19018	8	4	-	-	-	-	-
gALL_v3_c19019	3	1	-	-	gb|EWM29702.1|	activating signal cointegrator 1 complex subunit 3 [Nannochloropsis gaditana]	2,00E-09
gALL_v3_c1902	5	3	-	-	-	-	-
gALL_v3_c19020	0	5	-	-	-	-	-
gALL_v3_c19021	5	3	-	-	-	-	-
gALL_v3_c19022	4	10	-	-	-	-	-
gALL_v3_c19023	2	6	-	-	-	-	-
gALL_v3_c19024	0	4	-	-	-	-	-
gALL_v3_c19025	1	7	-	-	-	-	-
gALL_v3_c19026	2	2	-	-	-	-	-
gALL_v3_c19027	1	4	-	-	ref|XP_005184329.1|	PREDICTED: dnaJ homolog subfamily C member 21-like [Musca domestica]	2,00E-43
gALL_v3_c19028	3	1	-	-	-	-	-
gALL_v3_c19029	4	2	-	-	-	-	-
gALL_v3_c1903	10	13	-	-	ref|XP_002431484.1|	tyrosine-protein phosphatase 10D precursor, putative [Pediculus humanus corporis] gb|EEB18746.1| tyrosine-protein phosphatase 10D precursor, putative [Pediculus hum	0
gALL_v3_c19030	3	5	gnl|CDD|119398	cd06530, S26_SPase_I, The S2	ref|XP_001951649.2|	PREDICTED: integrin-alpha FG-GAP repeat-containing protein 2-like [Acyrthosiphon pisum]	5,00E-68
gALL_v3_c19031	0	4	-	-	-	-	-
gALL_v3_c19032	6	9	-	-	-	-	-
gALL_v3_c19033	4	6	-	-	-	-	-
gALL_v3_c19034	4	3	-	-	-	-	-
gALL_v3_c19035	1	3	-	-	-	-	-
gALL_v3_c19036	1	3	gnl|CDD|191846	pfam07776, zf-AD, Zinc-finger	ref|XP_003702930.1|	PREDICTED: uncharacterized protein LOC100878370 [Megachile rotundata]	1,00E-16
gALL_v3_c19037	3	2	-	-	-	-	-
gALL_v3_c1904	5	5	-	-	-	-	-
gALL_v3_c19040	2	4	-	-	-	-	-
gALL_v3_c19041	3	3	-	-	-	-	-
gALL_v3_c19042	0	3	-	-	-	-	-
gALL_v3_c19044	2	4	-	-	-	-	-
gALL_v3_c19045	2	6	-	-	-	-	-
gALL_v3_c19046	4	8	-	-	-	-	-
gALL_v3_c19047	1	3	gnl|CDD|178057	PLN02438, PLN02438, inositol	ref|XP_001330368.1|	hypothetical protein [Trichomonas vaginalis G3] gb|EAY01636.1| hypothetical protein TVAG_292690 [Trichomonas vaginalis G3]	1,00E-40
gALL_v3_c19049	2	4	-	-	-	-	-
gALL_v3_c1905	2	6	-	-	ref|XP_972532.1|	PREDICTED: similar to glucose dehydrogenase [Tribolium castaneum] gb|EFA05532.1| hypothetical protein TcasGA2_TC015719 [Tribolium castaneum]	9,00E-10
gALL_v3_c19051	2	4	-	-	-	-	-
gALL_v3_c19052	1	3	-	-	dbj|GAA51994.1|	leucine-rich repeat and WD repeat-containing protein 1 [Clonorchis sinensis]	4,00E-16
gALL_v3_c19053	6	10	-	-	ref|XP_002428797.1|	hypothetical protein Phum_PHUM401430 [Pediculus humanus corporis] gb|EEB16059.1| hypothetical protein Phum_PHUM401430 [Pediculus humanus corporis]	2,00E-06
gALL_v3_c19054	3	2	-	-	-	-	-
gALL_v3_c19055	4	8	gnl|CDD|115928	pfam07303, Occludin_ELL, Oc	ref|XP_967115.2|	PREDICTED: similar to AGAP004795-PA [Tribolium castaneum]	3,00E-41
gALL_v3_c19057	1	6	-	-	-	-	-
gALL_v3_c19058	1	4	-	-	-	-	-
gALL_v3_c19059	2	4	-	-	-	-	-
gALL_v3_c19060	2	3	-	-	-	-	-
gALL_v3_c19061	4	4	-	-	gb|EFZ15201.1|	hypothetical protein SINV_03318 [Solenopsis invicta]	5,00E-60
gALL_v3_c19062	3	9	gnl|CDD|34648	COG5043, MRS6, Vacuolar pro	gb|EEZ98831.1|	hypothetical protein TcasGA2_TC004440 [Tribolium castaneum]	5,00E-73
gALL_v3_c19064	3	2	-	-	-	-	-
gALL_v3_c19065	3	3	-	-	-	-	-
gALL_v3_c19066	1	3	-	-	-	-	-
gALL_v3_c19067	3	2	-	-	-	-	-
gALL_v3_c19068	2	4	-	-	gb|EFN74523.1|	Uncharacterized protein C14orf118-like protein [Camponotus floridanus]	2,00E-32
gALL_v3_c19069	2	1	-	-	-	-	-
gALL_v3_c19070	3	2	-	-	-	-	-
gALL_v3_c19071	2	1	-	-	-	-	-
gALL_v3_c19072	1	4	-	-	-	-	-
gALL_v3_c19073	3	12	-	-	gb|ADI61826.1|	endonuclease-reverse transcriptase [Bombyx mori]	5,00E-29

gALL_v3_c19077	5	3	-	-	-	-	-
gALL_v3_c19078	4	6	-	-	-	-	-
gALL_v3_c19079	6	3	-	-	-	-	-
gALL_v3_c1908	6	9	-	-	-	-	-
gALL_v3_c19080	3	1	-	-	-	-	-
gALL_v3_c19081	2	4	gnl|CDD|146633	pfam04100, Vps53_N, Vps53-l	ref|XP_002401876.1|	vacuolar protein-sorting protein, putative [Ixodes scapularis] gb|EEC11416.1| vacuolar protein-sorting protein, putative [Ixodes scapularis]	2,00E-62
gALL_v3_c19082	2	4	-	-	ref|XP_006613931.1|	PREDICTED: supervillin-like isoform X1 [Apis dorsata] ref|XP_006613932.1| PREDICTED: supervillin-like isoform X2 [Apis dorsata]	5,00E-65
gALL_v3_c19084	6	3	-	-	-	-	-
gALL_v3_c19085	1	5	-	-	-	-	-
gALL_v3_c19086	4	2	-	-	ref|XP_003741638.1|	PREDICTED: uncharacterized protein LOC100898406 [Metaseiulus occidentalis]	9,00E-24
gALL_v3_c19088	2	4	gnl|CDD|191897	pfam07933, DUF1681, Protein	gb|EFN69494.1|	NECAP-like protein CG9132 [Camponotus floridanus]	1,00E-73
gALL_v3_c19089	3	2	-	-	-	-	-
gALL_v3_c1909	8	8	gnl|CDD|133365	cd04165, GTPBP1_like, GTPBP	ref|XP_003395283.1|	PREDICTED: GTP-binding protein 1-like [Bombus terrestris]	0
gALL_v3_c19090	8	9	-	-	-	-	-
gALL_v3_c19091	1	3	gnl|CDD|192145	pfam08755, YccV-like, Hemime	ref|XP_002405977.1|	hypothetical protein IscW_ISCW019647 [Ixodes scapularis] gb|EEC10227.1| hypothetical protein IscW_ISCW019647 [Ixodes scapularis]	1,00E-22
gALL_v3_c19093	3	4	-	-	-	-	-
gALL_v3_c19094	1	5	-	-	-	-	-
gALL_v3_c19095	2	3	-	-	-	-	-
gALL_v3_c19096	6	4	-	-	-	-	-
gALL_v3_c19097	2	7	-	-	-	-	-
gALL_v3_c19098	3	1	gnl|CDD|128471	smart00174, RHO, Rho (Ras ho	ref|XP_001303727.1|	small GTP-binding protein [Trichomonas vaginalis G3] gb|EAX90797.1| small GTP-binding protein, putative [Trichomonas vaginalis G3]	8,00E-58
gALL_v3_c19099	19	13	gnl|CDD|112643	pfam03839, Sec62, Translocati	ref|XP_972897.1|	PREDICTED: similar to AGAP009788-PA [Tribolium castaneum] gb|EFA08689.1| hypothetical protein TcasGA2_TC006360 [Tribolium castaneum]	8,00E-55
gALL_v3_c191	13	17	-	-	-	-	-
gALL_v3_c19100	4	2	-	-	-	-	-
gALL_v3_c19101	4	7	-	-	ref|XP_006001723.1|	PREDICTED: histone-lysine N-methyltransferase 2C [Latimeria chalumnae]	4,00E-13
gALL_v3_c19102	3	6	-	-	-	-	-
gALL_v3_c19103	1	4	-	-	ref|XP_003701211.1|	PREDICTED: uncharacterized protein LOC100880297 [Megachile rotundata]	1,00E-53
gALL_v3_c19104	5	5	-	-	-	-	-
gALL_v3_c19106	2	2	-	-	-	-	-
gALL_v3_c19108	5	3	-	-	-	-	-
gALL_v3_c19109	5	9	-	-	-	-	-
gALL_v3_c19110	3	4	-	-	-	-	-
gALL_v3_c19111	2	3	-	-	-	-	-
gALL_v3_c19112	3	2	-	-	ref|XP_003241733.1|	PREDICTED: zinc finger MYM-type protein 1-like [Acyrthosiphon pisum]	1,00E-18
gALL_v3_c19114	3	4	-	-	-	-	-
gALL_v3_c19115	1	3	gnl|CDD|153272	cd07588, BAR_Amphiphysin, T	ref|XP_006615869.1|	PREDICTED: amphiphysin-like isoform X4 [Apis dorsata]	4,00E-77
gALL_v3_c19116	5	6	gnl|CDD|178285	PLN02681, PLN02681, proline	ref|XP_003426285.1|	PREDICTED: LOW QUALITY PROTEIN: proline dehydrogenase 1, mitochondrial-like [Nasonia vitripennis]	1,00E-47
gALL_v3_c19117	3	5	-	-	-	-	-
gALL_v3_c19118	2	2	gnl|CDD|191129	pfam04926, PAP_RNA-bind, Po	ref|XP_003706675.1|	PREDICTED: poly(A) polymerase gamma [Megachile rotundata]	3,00E-85
gALL_v3_c1912	0	6	-	-	-	-	-
gALL_v3_c19120	3	1	-	-	-	-	-
gALL_v3_c19121	3	5	gnl|CDD|189372	pfam00067, p450, Cytochrome	dbj|BAD84176.1|	cytochrome P450 [Hodotermopsis sjostedti]	4,00E-97
gALL_v3_c19122	2	5	gnl|CDD|191375	pfam05794, Tcp11, T-complex	ref|XP_003706462.1|	PREDICTED: T-complex protein 11-like protein 1-like isoform 1 [Megachile rotundata]	4,00E-27
gALL_v3_c19123	1	4	-	-	-	-	-
gALL_v3_c19124	1	5	-	-	ref|XP_003708232.1|	PREDICTED: probable serine hydrolase-like [Megachile rotundata]	6,00E-17
gALL_v3_c19125	1	4	-	-	-	-	-
gALL_v3_c19127	2	3	gnl|CDD|189858	pfam01147, Crust_neurohorm	ref|XP_002427293.1|	CHH XO-type, variant 4 precursor, putative [Pediculus humanus corporis] gb|EEB14555.1| CHH XO-type, variant 4 precursor, putative [Pediculus humanus corporis]	9,00E-46
gALL_v3_c19128	2	2	-	-	ref|XP_004609119.1|	PREDICTED: E3 ubiquitin-protein ligase SHPRH [Sorex araneus]	9,00E-45
gALL_v3_c19129	1	5	gnl|CDD|162084	TIGR00879, SP, MFS transporte	ref|XP_004530598.1|	PREDICTED: facilitated trehalose transporter Tret1-like isoform X2 [Ceratitis capitata]	3,00E-22
gALL_v3_c1913	13	28	-	-	-	-	-
gALL_v3_c19130	7	8	-	-	-	-	-
gALL_v3_c19131	2	2	-	-	-	-	-
gALL_v3_c19132	1	3	-	-	-	-	-
gALL_v3_c19133	1	4	-	-	-	-	-
gALL_v3_c19134	4	3	-	-	-	-	-
gALL_v3_c19135	4	6	-	-	gb|EFA11102.1|	hypothetical protein TcasGA2_TC004700 [Tribolium castaneum]	4,00E-67
gALL_v3_c19136	1	3	-	-	-	-	-
gALL_v3_c19137	2	2	gnl|CDD|191679	pfam07064, RIC1, RIC1. RIC1 I	gb|EFA06481.1|	hypothetical protein TcasGA2_TC009376 [Tribolium castaneum]	8,00E-56
gALL_v3_c19138	0	4	-	-	-	-	-
gALL_v3_c19139	2	2	-	-	-	-	-
gALL_v3_c1914	7	11	-	-	-	-	-
gALL_v3_c19140	2	2	-	-	-	-	-
gALL_v3_c19141	1	7	gnl|CDD|176047	cd08402, C2B_Synaptotagmin	gb|ENN73725.1|	hypothetical protein YQE_09675, partial [Dendroctonus ponderosae]	3,00E-43
gALL_v3_c19142	1	5	gnl|CDD|48344	cd00433, Peptidase_M17, Cyto	ref|XP_974656.1|	PREDICTED: similar to leucine aminopeptidase [Tribolium castaneum] gb|EEZ98647.1| hypothetical protein TcasGA2_TC001175 [Tribolium castaneum]	5,00E-36
gALL_v3_c19143	3	2	-	-	gb|AGM32754.1|	hypothetical protein, partial [Coptotermes formosanus]	1,00E-121
gALL_v3_c19144	3	3	-	-	-	-	-
gALL_v3_c19145	2	3	gnl|CDD|34710	COG5107, RNA14, Pre-mRNA 3	ref|XP_393870.2|	PREDICTED: protein suppressor of forked-like isoform X3 [Apis mellifera] ref|XP_003697399.1| PREDICTED: protein suppressor of forked-like [Apis florea]	2,00E-93
gALL_v3_c19146	1	2	-	-	-	-	-
gALL_v3_c19147	4	3	-	-	ref|XP_001948036.2|	PREDICTED: hypoxia-inducible factor 1-alpha inhibitor-like [Acyrthosiphon pisum]	3,00E-18
gALL_v3_c19149	10	16	gnl|CDD|189507	pfam00335, Tetraspannin, Tetr	gb|AGM32481.1|	tetraspanin D107 [Coptotermes formosanus]	6,00E-97
gALL_v3_c19150	0	5	-	-	-	-	-
gALL_v3_c19151	2	2	-	-	-	-	-
gALL_v3_c19152	3	2	-	-	-	-	-
gALL_v3_c19153	4	2	-	-	gb|ELU17166.1|	hypothetical protein CAPTEDRAFT_195632 [Capitella teleta]	3,00E-43
gALL_v3_c19154	1	3	-	-	-	-	-
gALL_v3_c19155	1	4	-	-	ref|XP_003707217.1|	PREDICTED: hemocyte protein-glutamine gamma-glutamyltransferase-like [Megachile rotundata]	3,00E-51
gALL_v3_c19156	0	5	-	-	-	-	-
gALL_v3_c19157	1	5	gnl|CDD|149669	pfam08687, ASD2, Apx/Shroo	ref|XP_001808038.1|	PREDICTED: similar to Protein Shroom [Tribolium castaneum]	1,00E-62
gALL_v3_c19158	3	2	-	-	-	-	-
gALL_v3_c19159	3	1	-	-	-	-	-
gALL_v3_c1916	8	11	gnl|CDD|190248	pfam02212, GED, Dynamin GT	ref|XP_003402356.1|	PREDICTED: dynamin-like [Bombus terrestris]	2,00E-85
gALL_v3_c19160	1	4	-	-	-	-	-
gALL_v3_c19162	1	4	-	-	-	-	-
gALL_v3_c19163	0	5	-	-	-	-	-
gALL_v3_c19164	2	9	gnl|CDD|190616	pfam03370, CBM_21, Putative	gb|EFA11655.1|	hypothetical protein TcasGA2_TC004080 [Tribolium castaneum]	3,00E-76
gALL_v3_c19165	4	2	-	-	-	-	-
gALL_v3_c19166	5	2	gnl|CDD|130057	TIGR00984, 3a0801s03tim44,	ref|XP_001599273.2|	PREDICTED: mitochondrial import inner membrane translocase subunit TIM44-like isoform 1 [Nasonia vitripennis]	1,00E-104
gALL_v3_c19167	5	3	-	-	gb|AAC37266.1|	blackjack [Schistocerca americana]	7,00E-17
gALL_v3_c19168	12	16	-	-	-	-	-
gALL_v3_c19170	2	5	gnl|CDD|144740	pfam01257, Complex1_24kDa	gb|AGM32334.1|	TRX-like [2Fe-2S] ferredoxin (Fd) family protein [Coptotermes formosanus]	2,00E-78
gALL_v3_c19171	4	2	-	-	-	-	-
gALL_v3_c19172	2	3	-	-	ref|XP_003248981.1|	PREDICTED: hypothetical protein LOC100570774, partial [Acyrthosiphon pisum]	2,00E-12
gALL_v3_c19174	1	3	-	-	-	-	-
gALL_v3_c19175	3	2	-	-	-	-	-
gALL_v3_c19176	3	4	-	-	-	-	-
gALL_v3_c19178	1	3	-	-	-	-	-
gALL_v3_c19179	1	4	-	-	-	-	-
gALL_v3_c1918	6	13	gnl|CDD|28913	cd00031, CA, Cadherin repeat	ref|XP_004931094.1|	PREDICTED: neural-cadherin-like [Bombyx mori]	0
gALL_v3_c19180	3	3	-	-	-	-	-
gALL_v3_c19182	3	4	-	-	-	-	-
gALL_v3_c19183	3	6	-	-	-	-	-
gALL_v3_c19184	3	7	-	-	-	-	-
gALL_v3_c19185	10	11	-	-	-	-	-
gALL_v3_c19186	7	7	gnl|CDD|189397	pfam00112, Peptidase_C1, Pap	dbj|BAA86911.1|	homologue of Sarcophaga 26,29kDa proteinase [Periplaneta americana]	1,00E-127
gALL_v3_c19187	3	2	-	-	-	-	-
gALL_v3_c19188	2	3	-	-	-	-	-
gALL_v3_c19189	5	3	-	-	gb|AGC92241.1|	putative glutamate synthase, partial [Laodelphax striatella]	1,00E-10
gALL_v3_c1919	17	15	gnl|CDD|191118	pfam04882, Peroxin-3, Peroxir	ref|XP_002427139.1|	Peroxisomal biogenesis factor, putative [Pediculus humanus corporis] gb|EEB14401.1| Peroxisomal biogenesis factor, putative [Pediculus humanus corporis]	3,00E-94
gALL_v3_c19191	0	3	-	-	-	-	-
gALL_v3_c19192	1	3	-	-	-	-	-
gALL_v3_c19194	1	4	-	-	ref|XP_971653.1|	PREDICTED: similar to U2 small nuclear ribonucleoprotein A [Tribolium castaneum] gb|EFA10336.1| hypothetical protein TcasGA2_TC012554 [Tribolium castaneum]	4,00E-47
gALL_v3_c19195	4	6	-	-	-	-	-
gALL_v3_c19196	1	3	-	-	-	-	-
gALL_v3_c19197	0	3	-	-	gb|EFN70893.1|	Myosin regulatory light chain 2 [Camponotus floridanus]	2,00E-17
gALL_v3_c19199	3	1	-	-	-	-	-
gALL_v3_c192	15	11	gnl|CDD|147425	pfam05221, AdoHcyase, S-ade	gb|EFA12023.1|	hypothetical protein TcasGA2_TC001438 [Tribolium castaneum]	0
gALL_v3_c1920	4	13	-	-	-	-	-
gALL_v3_c19200	0	4	-	-	ref|XP_006005955.1|	PREDICTED: constitutive coactivator of peroxisome proliferator-activated receptor gamma isoform X3 [Latimeria chalumnae]	4,00E-11
gALL_v3_c19201	1	2	gnl|CDD|111752	pfam02898, NO_synthase, Nit	dbj|BAH14964.1|	nitric oxide synthase [Gryllus bimaculatus]	5,00E-86
gALL_v3_c19202	18	13	gnl|CDD|132790	cd06880, PX_SNX22, The phos	gb|ESO91510.1|	hypothetical protein LOTGIDRAFT_228870 [Lottia gigantea]	2,00E-46
gALL_v3_c19203	4	2	-	-	-	-	-
gALL_v3_c19204	2	3	gnl|CDD|162093	TIGR00892, 2A0113, monocar	ref|XP_311255.5|	AGAP000717-PA [Anopheles gambiae str. PEST] gb|EAA06880.5| AGAP000717-PA [Anopheles gambiae str. PEST]	1,00E-35
gALL_v3_c19205	1	4	-	-	-	-	-
gALL_v3_c19207	3	7	gnl|CDD|147299	pfam05044, Prox1, Homeobox	ref|XP_001655942.1|	homeobox protein prospero/prox-1 [Aedes aegypti] gb|EAT46002.1| AAEL002769-PA, partial [Aedes aegypti]	2,00E-38
gALL_v3_c19208	0	5	-	-	-	-	-
gALL_v3_c19209	7	5	-	-	ref|XP_003707100.1|	PREDICTED: uncharacterized protein LOC100878688 [Megachile rotundata]	6,00E-45
gALL_v3_c1921	5	3	-	-	-	-	-
gALL_v3_c19210	2	5	-	-	gb|EFA02573.1|	hypothetical protein TcasGA2_TC008291 [Tribolium castaneum]	1,00E-55
gALL_v3_c19212	4	2	-	-	-	-	-
gALL_v3_c19214	3	3	-	-	-	-	-
gALL_v3_c19215	1	3	-	-	-	-	-
gALL_v3_c19216	2	2	-	-	-	-	-
gALL_v3_c19217	3	5	gnl|CDD|150799	pfam10174, Cast, RIM-binding	gb|EFA07673.1|	hypothetical protein TcasGA2_TC030684, partial [Tribolium castaneum]	2,00E-28
gALL_v3_c19218	2	3	gnl|CDD|132784	cd06874, PX_KIF16B_SNX23, T	gb|ETN65066.1|	kinesin heavy chain [Anopheles darlingi]	4,00E-21
gALL_v3_c19220	1	4	-	-	ref|XP_003706377.1|	PREDICTED: hrp65 protein-like [Megachile rotundata]	9,00E-32
gALL_v3_c19221	1	3	-	-	-	-	-
gALL_v3_c19222	1	5	-	-	-	-	-
gALL_v3_c19223	1	3	gnl|CDD|190846	pfam04054, Not1, CCR4-Not c	ref|XP_006618713.1|	PREDICTED: CCR4-NOT transcription complex subunit 1-like isoform X1 [Apis dorsata]	2,00E-55
gALL_v3_c19225	2	3	-	-	-	-	-
gALL_v3_c19226	1	4	gnl|CDD|173624	cd00192, PTKc, Catalytic doma	ref|XP_319333.4|	AGAP010162-PA [Anopheles gambiae str. PEST] gb|EAA13998.4| AGAP010162-PA [Anopheles gambiae str. PEST]	4,00E-46
gALL_v3_c19227	1	3	-	-	-	-	-
gALL_v3_c19228	1	4	-	-	ref|XP_001661282.1|	myosin light chain kinase [Aedes aegypti] gb|EAT36953.1| AAEL010997-PA [Aedes aegypti]	3,00E-16
gALL_v3_c1923	9	12	gnl|CDD|177158	MTH00095, ND5, NADH dehyc	-	-	-
gALL_v3_c19230	6	3	-	-	-	-	-

<table>
<tr><td>gALL_v3_c19232</td><td>3</td><td>4</td><td>-</td><td>-</td><td>-</td><td>-</td><td>-</td></tr>
<tr><td>gALL_v3_c19233</td><td>3</td><td>1</td><td>-</td><td>-</td><td>ref|XP_001862037.1|</td><td>conserved hypothetical protein [Culex quinquefasciatus] gb|EDS36376.1| conserved hypothetical protein [Culex quinquefasciatus]</td><td>4,00E-23</td></tr>
<tr><td>gALL_v3_c19234</td><td>2</td><td>3</td><td>-</td><td>-</td><td>-</td><td>-</td><td>-</td></tr>
<tr><td>gALL_v3_c19235</td><td>3</td><td>3</td><td>-</td><td>-</td><td>-</td><td>-</td><td>-</td></tr>
<tr><td>gALL_v3_c19236</td><td>0</td><td>6</td><td>-</td><td>-</td><td>gb|ENN78039.1|</td><td>hypothetical protein YQE_05476, partial [Dendroctonus ponderosae] gb|ERL87267.1| hypothetical protein D910_04663 [Dendroctonus ponderosae]</td><td>2,00E-14</td></tr>
<tr><td>gALL_v3_c19238</td><td>1</td><td>3</td><td>-</td><td>-</td><td>-</td><td>-</td><td>-</td></tr>
<tr><td>gALL_v3_c19239</td><td>0</td><td>5</td><td>-</td><td>-</td><td>-</td><td>-</td><td>-</td></tr>
<tr><td>gALL_v3_c1924</td><td>5</td><td>8</td><td>gnl|CDD|34909</td><td>COG5329, COG5329, Phospho</td><td>ref|XP_006560153.1|</td><td>PREDICTED: phosphatidylinositide phosphatase SAC2-like isoform X2 [Apis mellifera]</td><td>1,00E-176</td></tr>
<tr><td>gALL_v3_c19240</td><td>2</td><td>3</td><td>-</td><td>-</td><td>-</td><td>-</td><td>-</td></tr>
<tr><td>gALL_v3_c19241</td><td>3</td><td>1</td><td>gnl|CDD|178494</td><td>PLN02906, PLN02906, xanthin</td><td>ref|XP_003427572.1|</td><td>PREDICTED: LOW QUALITY PROTEIN: xanthine dehydrogenase-like [Nasonia vitripennis]</td><td>8,00E-44</td></tr>
<tr><td>gALL_v3_c19242</td><td>2</td><td>3</td><td>-</td><td>-</td><td>ref|XP_003703916.1|</td><td>PREDICTED: uncharacterized protein LOC100876764 [Megachile rotundata]</td><td>7,00E-46</td></tr>
<tr><td>gALL_v3_c19243</td><td>2</td><td>3</td><td>-</td><td>-</td><td>ref|XP_002424192.1|</td><td>Hsc70-interacting protein, putative [Pediculus humanus corporis] gb|EEB11454.1| Hsc70-interacting protein, putative [Pediculus humanus corporis]</td><td>3,00E-09</td></tr>
<tr><td>gALL_v3_c19244</td><td>2</td><td>4</td><td>-</td><td>-</td><td>-</td><td>-</td><td>-</td></tr>
<tr><td>gALL_v3_c19246</td><td>5</td><td>11</td><td>-</td><td>-</td><td>gb|EGI65398.1|</td><td>FLJ37770-like protein [Acromyrmex echinatior]</td><td>8,00E-37</td></tr>
<tr><td>gALL_v3_c19248</td><td>5</td><td>2</td><td>-</td><td>-</td><td>-</td><td>-</td><td>-</td></tr>
<tr><td>gALL_v3_c19249</td><td>4</td><td>2</td><td>-</td><td>-</td><td>-</td><td>-</td><td>-</td></tr>
<tr><td>gALL_v3_c1925</td><td>4</td><td>5</td><td>gnl|CDD|191317</td><td>pfam05615, THOC7, Tho comp</td><td>dbj|BAM20034.1|</td><td>THO complex subunit 7-like protein [Papilio xuthus]</td><td>8,00E-86</td></tr>
<tr><td>gALL_v3_c19250</td><td>2</td><td>3</td><td>-</td><td>-</td><td>-</td><td>-</td><td>-</td></tr>
<tr><td>gALL_v3_c19251</td><td>3</td><td>1</td><td>-</td><td>-</td><td>gb|EFX68681.1|</td><td>hypothetical protein DAPPUDRAFT_114312 [Daphnia pulex]</td><td>6,00E-23</td></tr>
<tr><td>gALL_v3_c19254</td><td>1</td><td>3</td><td>-</td><td>-</td><td>-</td><td>-</td><td>-</td></tr>
<tr><td>gALL_v3_c19255</td><td>4</td><td>4</td><td>-</td><td>-</td><td>ref|XP_002431022.1|</td><td>conserved hypothetical protein [Pediculus humanus corporis] gb|EEB18284.1| conserved hypothetical protein [Pediculus humanus corporis]</td><td>3,00E-40</td></tr>
<tr><td>gALL_v3_c19256</td><td>5</td><td>4</td><td>-</td><td>-</td><td>ref|XP_005305139.1|</td><td>PREDICTED: tigger transposable element-derived protein 1-like [Chrysemys picta bellii]</td><td>4,00E-29</td></tr>
<tr><td>gALL_v3_c19257</td><td>1</td><td>3</td><td>gnl|CDD|29257</td><td>cd00200, WD40, WD40 domai</td><td>gb|EFN60314.1|</td><td>WD repeat and FYVE domain-containing protein 3 [Camponotus floridanus]</td><td>9,00E-77</td></tr>
<tr><td>gALL_v3_c19259</td><td>2</td><td>4</td><td>-</td><td>-</td><td>-</td><td>-</td><td>-</td></tr>
<tr><td>gALL_v3_c19260</td><td>2</td><td>3</td><td>-</td><td>-</td><td>-</td><td>-</td><td>-</td></tr>
<tr><td>gALL_v3_c19261</td><td>2</td><td>3</td><td>-</td><td>-</td><td>-</td><td>-</td><td>-</td></tr>
<tr><td>gALL_v3_c19263</td><td>0</td><td>4</td><td>-</td><td>-</td><td>gb|ETN63691.1|</td><td>glycerol-3-phosphate acyltransferase [Anopheles darlingi]</td><td>5,00E-07</td></tr>
<tr><td>gALL_v3_c19264</td><td>7</td><td>9</td><td>gnl|CDD|29014</td><td>cd00115, LMWPc, Low molecu</td><td>dbj|BAM18286.1|</td><td>primo-1 [Papilio xuthus]</td><td>3,00E-60</td></tr>
<tr><td>gALL_v3_c19266</td><td>2</td><td>3</td><td>-</td><td>-</td><td>-</td><td>-</td><td>-</td></tr>
<tr><td>gALL_v3_c19267</td><td>3</td><td>1</td><td>gnl|CDD|29257</td><td>cd00200, WD40, WD40 domai</td><td>ref|XP_002429148.1|</td><td>conserved hypothetical protein [Pediculus humanus corporis] gb|EEB16410.1| conserved hypothetical protein [Pediculus humanus corporis]</td><td>2,00E-87</td></tr>
<tr><td>gALL_v3_c19269</td><td>2</td><td>2</td><td>-</td><td>-</td><td>-</td><td>-</td><td>-</td></tr>
<tr><td>gALL_v3_c1927</td><td>23</td><td>22</td><td>-</td><td>-</td><td>ref|WP_016250151.1|</td><td>hypothetical protein [Enterococcus villorum] gb|EOW78828.1| hypothetical protein I591_00371 [Enterococcus villorum ATCC 700913]</td><td>1,00E-06</td></tr>
<tr><td>gALL_v3_c19270</td><td>3</td><td>3</td><td>-</td><td>-</td><td>ref|XP_002740511.2|</td><td>PREDICTED: U3 small nucleolar RNA-associated protein 18 homolog [Saccoglossus kowalevskii]</td><td>2,00E-47</td></tr>
<tr><td>gALL_v3_c19272</td><td>6</td><td>9</td><td>gnl|CDD|147511</td><td>pfam05361, PP1_inhibitor, PK(</td><td>ref|XP_003401656.1|</td><td>PREDICTED: protein phosphatase 1 regulatory subunit 14B-like isoform 1 [Bombus terrestris] ref|XP_003401657.1| PREDICTED: protein phosphatase 1 regulatory subunit 1</td><td>7,00E-21</td></tr>
<tr><td>gALL_v3_c19273</td><td>2</td><td>4</td><td>-</td><td>-</td><td>gb|ETN60820.1|</td><td>hypothetical protein AND_007549 [Anopheles darlingi]</td><td>1,00E-31</td></tr>
<tr><td>gALL_v3_c19274</td><td>2</td><td>5</td><td>-</td><td>-</td><td>gb|AGM32828.1|</td><td>hypothetical protein [Coptotermes formosanus]</td><td>6,00E-11</td></tr>
<tr><td>gALL_v3_c19275</td><td>3</td><td>4</td><td>-</td><td>-</td><td>-</td><td>-</td><td>-</td></tr>
<tr><td>gALL_v3_c19276</td><td>1</td><td>5</td><td>-</td><td>-</td><td>-</td><td>-</td><td>-</td></tr>
<tr><td>gALL_v3_c19277</td><td>4</td><td>4</td><td>-</td><td>-</td><td>-</td><td>-</td><td>-</td></tr>
<tr><td>gALL_v3_c19279</td><td>4</td><td>4</td><td>gnl|CDD|152730</td><td>pfam12295, Symplekin_C, Syr</td><td>ref|XP_002428201.1|</td><td>Symplekin, putative [Pediculus humanus corporis] gb|EEB15463.1| Symplekin, putative [Pediculus humanus corporis]</td><td>2,00E-81</td></tr>
<tr><td>gALL_v3_c1928</td><td>9</td><td>17</td><td>-</td><td>-</td><td>gb|EFN87052.1|</td><td>Aminopeptidase N [Harpegnathos saltator]</td><td>1,00E-103</td></tr>
<tr><td>gALL_v3_c19280</td><td>2</td><td>3</td><td>-</td><td>-</td><td>-</td><td>-</td><td>-</td></tr>
<tr><td>gALL_v3_c19281</td><td>3</td><td>2</td><td>-</td><td>-</td><td>-</td><td>-</td><td>-</td></tr>
<tr><td>gALL_v3_c19282</td><td>3</td><td>3</td><td>-</td><td>-</td><td>ref|XP_003366722.1|</td><td>retrovirus-related Pol polyprotein from transposon TNT 1-94 [Trichinella spiralis] gb|EFV48956.1| retrovirus-related Pol polyprotein from transposon TNT 1-94 [Trichinella s</td><td>2,00E-39</td></tr>
<tr><td>gALL_v3_c19283</td><td>2</td><td>4</td><td>-</td><td>-</td><td>-</td><td>-</td><td>-</td></tr>
<tr><td>gALL_v3_c19284</td><td>7</td><td>13</td><td>gnl|CDD|189507</td><td>pfam00335, Tetraspannin, Tetr</td><td>ref|XP_002427607.1|</td><td>tetraspanin-18, putative [Pediculus humanus corporis] gb|EEB14869.1| tetraspanin-18, putative [Pediculus humanus corporis]</td><td>8,00E-34</td></tr>
<tr><td>gALL_v3_c19286</td><td>2</td><td>3</td><td>-</td><td>-</td><td>-</td><td>-</td><td>-</td></tr>
<tr><td>gALL_v3_c19288</td><td>1</td><td>4</td><td>-</td><td>-</td><td>-</td><td>-</td><td>-</td></tr>
<tr><td>gALL_v3_c19289</td><td>3</td><td>2</td><td>-</td><td>-</td><td>ref|XP_006622561.1|</td><td>PREDICTED: uncharacterized protein LOC102681917 [Apis dorsata]</td><td>2,00E-06</td></tr>
<tr><td>gALL_v3_c1929</td><td>42</td><td>64</td><td>-</td><td>-</td><td>gb|EFX63822.1|</td><td>hypothetical protein DAPPUDRAFT_118824 [Daphnia pulex]</td><td>1,00E-45</td></tr>
<tr><td>gALL_v3_c19290</td><td>5</td><td>6</td><td>gnl|CDD|149409</td><td>pfam08337, Plexin_cytopl, Ple</td><td>gb|EFN82927.1|</td><td>Plexin-B [Harpegnathos saltator]</td><td>1,00E-170</td></tr>
<tr><td>gALL_v3_c19291</td><td>2</td><td>3</td><td>-</td><td>-</td><td>ref|XP_003244015.1|</td><td>PREDICTED: hemicentin-1-like isoform 2 [Acyrthosiphon pisum]</td><td>1,00E-17</td></tr>
<tr><td>gALL_v3_c19292</td><td>2</td><td>3</td><td>-</td><td>-</td><td>ref|XP_005099571.1|</td><td>PREDICTED: protein YIPF1-like [Aplysia californica]</td><td>6,00E-36</td></tr>
<tr><td>gALL_v3_c19294</td><td>6</td><td>5</td><td>-</td><td>-</td><td>gb|ADY39587.1|</td><td>putative fusili isoform B [Hottentotta judaicus]</td><td>5,00E-32</td></tr>
<tr><td>gALL_v3_c19295</td><td>5</td><td>8</td><td>-</td><td>-</td><td>-</td><td>-</td><td>-</td></tr>
<tr><td>gALL_v3_c19296</td><td>6</td><td>4</td><td>-</td><td>-</td><td>-</td><td>-</td><td>-</td></tr>
<tr><td>gALL_v3_c19298</td><td>1</td><td>4</td><td>-</td><td>-</td><td>-</td><td>-</td><td>-</td></tr>
<tr><td>gALL_v3_c19299</td><td>0</td><td>3</td><td>-</td><td>-</td><td>-</td><td>-</td><td>-</td></tr>
<tr><td>gALL_v3_c193</td><td>9</td><td>6</td><td>gnl|CDD|144473</td><td>pfam00888, Cullin, Cullin fami</td><td>gb|EFZ19308.1|</td><td>hypothetical protein SINV_13032 [Solenopsis invicta]</td><td>0</td></tr>
<tr><td>gALL_v3_c1930</td><td>13</td><td>14</td><td>gnl|CDD|192999</td><td>pfam12353, eIF3g, Eukaryotic</td><td>gb|EFA10249.1|</td><td>hypothetical protein TcasGA2_TC012449 [Tribolium castaneum]</td><td>1,00E-140</td></tr>
<tr><td>gALL_v3_c19300</td><td>2</td><td>3</td><td>-</td><td>-</td><td>-</td><td>-</td><td>-</td></tr>
<tr><td>gALL_v3_c19302</td><td>2</td><td>5</td><td>-</td><td>-</td><td>-</td><td>-</td><td>-</td></tr>
<tr><td>gALL_v3_c19303</td><td>3</td><td>6</td><td>-</td><td>-</td><td>ref|XP_006029730.1|</td><td>PREDICTED: histone-lysine N-methyltransferase SETMAR-like [Alligator sinensis]</td><td>2,00E-09</td></tr>
<tr><td>gALL_v3_c19304</td><td>1</td><td>6</td><td>-</td><td>-</td><td>-</td><td>-</td><td>-</td></tr>
<tr><td>gALL_v3_c19305</td><td>3</td><td>5</td><td>-</td><td>-</td><td>-</td><td>-</td><td>-</td></tr>
<tr><td>gALL_v3_c19306</td><td>2</td><td>4</td><td>-</td><td>-</td><td>gb|EFZ21745.1|</td><td>hypothetical protein SINV_06928 [Solenopsis invicta]</td><td>1,00E-08</td></tr>
<tr><td>gALL_v3_c19307</td><td>0</td><td>5</td><td>-</td><td>-</td><td>ref|XP_002432667.1|</td><td>Abnormal long morphology protein, putative [Pediculus humanus corporis] gb|EEB19929.1| Abnormal long morphology protein, putative [Pediculus humanus corporis]</td><td>1,00E-13</td></tr>
<tr><td>gALL_v3_c19308</td><td>2</td><td>6</td><td>gnl|CDD|128627</td><td>smart00332, PP2Cc, Serine/th</td><td>ref|XP_967846.1|</td><td>PREDICTED: similar to protein phosphatase 2c [Tribolium castaneum]</td><td>1,00E-107</td></tr>
<tr><td>gALL_v3_c19309</td><td>0</td><td>3</td><td>-</td><td>-</td><td>-</td><td>-</td><td>-</td></tr>
<tr><td>gALL_v3_c1931</td><td>17</td><td>15</td><td>gnl|CDD|173412</td><td>PTZ00121, PTZ00121, MAEBL;</td><td>ref|XP_005046533.1|</td><td>PREDICTED: LOW QUALITY PROTEIN: selenoprotein H [Ficedula albicollis]</td><td>2,00E-19</td></tr>
<tr><td>gALL_v3_c19310</td><td>1</td><td>5</td><td>gnl|CDD|128516</td><td>smart00220, S_TKc, Serine/Th</td><td>ref|XP_002052243.1|</td><td>GJ22747 [Drosophila virilis] gb|EDW64398.1| GJ22747 [Drosophila virilis]</td><td>1,00E-87</td></tr>
<tr><td>gALL_v3_c19311</td><td>4</td><td>2</td><td>-</td><td>-</td><td>-</td><td>-</td><td>-</td></tr>
<tr><td>gALL_v3_c19312</td><td>4</td><td>5</td><td>gnl|CDD|191367</td><td>pfam05761, 5_nucleotid, 5' nu</td><td>gb|EFN77471.1|</td><td>Cytosolic purine 5'-nucleotidase [Harpegnathos saltator]</td><td>1,00E-136</td></tr>
<tr><td>gALL_v3_c19313</td><td>3</td><td>7</td><td>-</td><td>-</td><td>-</td><td>-</td><td>-</td></tr>
<tr><td>gALL_v3_c19314</td><td>1</td><td>4</td><td>-</td><td>-</td><td>-</td><td>-</td><td>-</td></tr>
<tr><td>gALL_v3_c19315</td><td>4</td><td>5</td><td>-</td><td>-</td><td>-</td><td>-</td><td>-</td></tr>
<tr><td>gALL_v3_c19316</td><td>3</td><td>2</td><td>-</td><td>-</td><td>-</td><td>-</td><td>-</td></tr>
<tr><td>gALL_v3_c19318</td><td>3</td><td>3</td><td>-</td><td>-</td><td>-</td><td>-</td><td>-</td></tr>
<tr><td>gALL_v3_c1932</td><td>2</td><td>5</td><td>-</td><td>-</td><td>gb|EFX72787.1|</td><td>hypothetical protein DAPPUDRAFT_308084 [Daphnia pulex]</td><td>1,00E-11</td></tr>
<tr><td>gALL_v3_c19320</td><td>0</td><td>4</td><td>-</td><td>-</td><td>-</td><td>-</td><td>-</td></tr>
<tr><td>gALL_v3_c19321</td><td>1</td><td>3</td><td>-</td><td>-</td><td>-</td><td>-</td><td>-</td></tr>
<tr><td>gALL_v3_c19322</td><td>4</td><td>2</td><td>-</td><td>-</td><td>dbj|BAB21515.1|</td><td>TRASDJ [Saturnia japonica]</td><td>2,00E-12</td></tr>
<tr><td>gALL_v3_c19324</td><td>2</td><td>4</td><td>-</td><td>-</td><td>-</td><td>-</td><td>-</td></tr>
<tr><td>gALL_v3_c19326</td><td>1</td><td>5</td><td>gnl|CDD|152473</td><td>pfam12038, DUF3524, Domair</td><td>ref|XP_002609710.1|</td><td>hypothetical protein BRAFLDRAFT_285910 [Branchiostoma floridae] gb|EEN65720.1| hypothetical protein BRAFLDRAFT_285910 [Branchiostoma floridae]</td><td>5,00E-29</td></tr>
<tr><td>gALL_v3_c19327</td><td>3</td><td>6</td><td>-</td><td>-</td><td>ref|XP_005660967.1|</td><td>PREDICTED: CUGBP, Elav-like family member 1 [Sus scrofa]</td><td>3,00E-36</td></tr>
<tr><td>gALL_v3_c19328</td><td>1</td><td>4</td><td>-</td><td>-</td><td>-</td><td>-</td><td>-</td></tr>
<tr><td>gALL_v3_c19329</td><td>2</td><td>2</td><td>-</td><td>-</td><td>-</td><td>-</td><td>-</td></tr>
<tr><td>gALL_v3_c1933</td><td>2</td><td>6</td><td>-</td><td>-</td><td>ref|XP_003489865.1|</td><td>PREDICTED: rab3 GTPase-activating protein non-catalytic subunit-like [Bombus impatiens]</td><td>1,00E-11</td></tr>
<tr><td>gALL_v3_c19330</td><td>4</td><td>7</td><td>gnl|CDD|143836</td><td>pfam00050, Kazal_1, Kazal-typ</td><td>gb|AFZ78843.1|</td><td>Kazal-type serine protease inhibitor-like protein [Coptotermes formosanus]</td><td>2,00E-33</td></tr>
<tr><td>gALL_v3_c19331</td><td>5</td><td>5</td><td>-</td><td>-</td><td>-</td><td>-</td><td>-</td></tr>
<tr><td>gALL_v3_c19332</td><td>1</td><td>4</td><td>-</td><td>-</td><td>-</td><td>-</td><td>-</td></tr>
<tr><td>gALL_v3_c19333</td><td>5</td><td>3</td><td>-</td><td>-</td><td>-</td><td>-</td><td>-</td></tr>
<tr><td>gALL_v3_c19334</td><td>1</td><td>5</td><td>-</td><td>-</td><td>ref|XP_002430267.1|</td><td>low-density lipoprotein receptor, putative [Pediculus humanus corporis] gb|EEB17529.1| low-density lipoprotein receptor, putative [Pediculus humanus corporis]</td><td>9,00E-91</td></tr>
<tr><td>gALL_v3_c19336</td><td>0</td><td>3</td><td>-</td><td>-</td><td>-</td><td>-</td><td>-</td></tr>
<tr><td>gALL_v3_c19337</td><td>4</td><td>2</td><td>-</td><td>-</td><td>-</td><td>-</td><td>-</td></tr>
<tr><td>gALL_v3_c19339</td><td>3</td><td>8</td><td>-</td><td>-</td><td>-</td><td>-</td><td>-</td></tr>
<tr><td>gALL_v3_c19340</td><td>26</td><td>34</td><td>gnl|CDD|29115</td><td>cd00170, SEC14, Sec14p-like li</td><td>ref|XP_003398725.1|</td><td>PREDICTED: clavesin-1-like [Bombus terrestris]</td><td>1,00E-148</td></tr>
<tr><td>gALL_v3_c19341</td><td>1</td><td>5</td><td>-</td><td>-</td><td>ref|XP_003247632.1|</td><td>PREDICTED: pyruvate dehydrogenase phosphatase regulatory subunit, mitochondrial-like isoform 1 [Acyrthosiphon pisum]</td><td>4,00E-96</td></tr>
<tr><td>gALL_v3_c19342</td><td>7</td><td>9</td><td>gnl|CDD|58168</td><td>cd00887, MoeA, MoeA family.</td><td>ref|XP_004908029.1|</td><td>PREDICTED: gephyrin, partial [Heterocephalus glaber]</td><td>8,00E-95</td></tr>
<tr><td>gALL_v3_c19343</td><td>4</td><td>7</td><td>gnl|CDD|144003</td><td>pfam00254, FKBP_C, FKBP-typ</td><td>ref|XP_002167164.1|</td><td>PREDICTED: peptidyl-prolyl cis-trans isomerase FKBP6-like [Hydra vulgaris]</td><td>5,00E-49</td></tr>
<tr><td>gALL_v3_c19344</td><td>6</td><td>13</td><td>-</td><td>-</td><td>ref|XP_002422769.1|</td><td>hypothetical protein Phum_PHUM011900 [Pediculus humanus corporis] gb|EEB10031.1| hypothetical protein Phum_PHUM011900 [Pediculus humanus corporis]</td><td>1,00E-07</td></tr>
<tr><td>gALL_v3_c19345</td><td>3</td><td>2</td><td>-</td><td>-</td><td>-</td><td>-</td><td>-</td></tr>
<tr><td>gALL_v3_c19347</td><td>4</td><td>4</td><td>-</td><td>-</td><td>-</td><td>-</td><td>-</td></tr>
<tr><td>gALL_v3_c19348</td><td>3</td><td>10</td><td>-</td><td>-</td><td>-</td><td>-</td><td>-</td></tr>
<tr><td>gALL_v3_c1935</td><td>6</td><td>7</td><td>-</td><td>-</td><td>-</td><td>-</td><td>-</td></tr>
<tr><td>gALL_v3_c19350</td><td>3</td><td>1</td><td>gnl|CDD|128427</td><td>smart00119, HECTc, Domain H</td><td>gb|EFZ13617.1|</td><td>hypothetical protein SINV_15550 [Solenopsis invicta]</td><td>1,00E-100</td></tr>
<tr><td>gALL_v3_c19351</td><td>9</td><td>7</td><td>-</td><td>-</td><td>-</td><td>-</td><td>-</td></tr>
<tr><td>gALL_v3_c19353</td><td>0</td><td>4</td><td>-</td><td>-</td><td>-</td><td>-</td><td>-</td></tr>
<tr><td>gALL_v3_c19354</td><td>4</td><td>5</td><td>-</td><td>-</td><td>-</td><td>-</td><td>-</td></tr>
<tr><td>gALL_v3_c19355</td><td>2</td><td>3</td><td>-</td><td>-</td><td>-</td><td>-</td><td>-</td></tr>
<tr><td>gALL_v3_c19356</td><td>3</td><td>1</td><td>-</td><td>-</td><td>-</td><td>-</td><td>-</td></tr>
<tr><td>gALL_v3_c19358</td><td>3</td><td>4</td><td>gnl|CDD|100104</td><td>cd00590, RRM, RRM (RNA rec</td><td>dbj|BAN21585.1|</td><td>gar2, putative, partial [Riptortus pedestris]</td><td>2,00E-69</td></tr>
<tr><td>gALL_v3_c19359</td><td>0</td><td>4</td><td>-</td><td>-</td><td>-</td><td>-</td><td>-</td></tr>
<tr><td>gALL_v3_c1936</td><td>13</td><td>14</td><td>-</td><td>-</td><td>-</td><td>-</td><td>-</td></tr>
<tr><td>gALL_v3_c19360</td><td>4</td><td>3</td><td>-</td><td>-</td><td>gb|EHJ77867.1|</td><td>Down syndrome cell adhesion molecule [Danaus plexippus]</td><td>2,00E-28</td></tr>
<tr><td>gALL_v3_c19362</td><td>3</td><td>2</td><td>-</td><td>-</td><td>-</td><td>-</td><td>-</td></tr>
<tr><td>gALL_v3_c19363</td><td>3</td><td>2</td><td>-</td><td>-</td><td>-</td><td>-</td><td>-</td></tr>
<tr><td>gALL_v3_c19364</td><td>3</td><td>4</td><td>-</td><td>-</td><td>-</td><td>-</td><td>-</td></tr>
<tr><td>gALL_v3_c19366</td><td>4</td><td>3</td><td>-</td><td>-</td><td>ref|XP_002428248.1|</td><td>p53 and DNA damage-regulated protein, putative [Pediculus humanus corporis] gb|EEB15510.1| p53 and DNA damage-regulated protein, putative [Pediculus humanus cor</td><td>1,00E-25</td></tr>
<tr><td>gALL_v3_c19367</td><td>2</td><td>6</td><td>-</td><td>-</td><td>-</td><td>-</td><td>-</td></tr>
<tr><td>gALL_v3_c19369</td><td>2</td><td>2</td><td>-</td><td>-</td><td>-</td><td>-</td><td>-</td></tr>
<tr><td>gALL_v3_c1937</td><td>11</td><td>7</td><td>gnl|CDD|185392</td><td>PTZ00004, PTZ00004, actin-2;</td><td>dbj|BAM14390.1|</td><td>actin [Spirotrichonympha leidyi]</td><td>0</td></tr>
<tr><td>gALL_v3_c19370</td><td>2</td><td>6</td><td>-</td><td>-</td><td>-</td><td>-</td><td>-</td></tr>
<tr><td>gALL_v3_c19372</td><td>5</td><td>3</td><td>gnl|CDD|192523</td><td>pfam10300, DUF3808, Protein</td><td>gb|EFN69425.1|</td><td>Tetratricopeptide repeat protein 39B [Camponotus floridanus]</td><td>3,00E-36</td></tr>
<tr><td>gALL_v3_c19373</td><td>1</td><td>2</td><td>-</td><td>-</td><td>-</td><td>-</td><td>-</td></tr>
<tr><td>gALL_v3_c19374</td><td>0</td><td>4</td><td>-</td><td>-</td><td>-</td><td>-</td><td>-</td></tr>
<tr><td>gALL_v3_c19375</td><td>0</td><td>3</td><td>-</td><td>-</td><td>-</td><td>-</td><td>-</td></tr>
<tr><td>gALL_v3_c19376</td><td>7</td><td>12</td><td>-</td><td>-</td><td>ref|XP_003248346.1|</td><td>PREDICTED: craniofacial development protein 2-like, partial [Acyrthosiphon pisum]</td><td>2,00E-52</td></tr>
<tr><td>gALL_v3_c19377</td><td>2</td><td>2</td><td>-</td><td>-</td><td>-</td><td>-</td><td>-</td></tr>
<tr><td>gALL_v3_c19378</td><td>0</td><td>3</td><td>-</td><td>-</td><td>-</td><td>-</td><td>-</td></tr>
<tr><td>gALL_v3_c19379</td><td>3</td><td>4</td><td>-</td><td>-</td><td>-</td><td>-</td><td>-</td></tr>
<tr><td>gALL_v3_c19380</td><td>2</td><td>3</td><td>gnl|CDD|191882</td><td>pfam07890, Rrp15p, Rrp15p.</td><td>gb|AGM32250.1|</td><td>RRP15-like protein [Coptotermes formosanus]</td><td>2,00E-25</td></tr>
<tr><td>gALL_v3_c19381</td><td>2</td><td>2</td><td>-</td><td>-</td><td>-</td><td>-</td><td>-</td></tr>
<tr><td>gALL_v3_c19383</td><td>2</td><td>2</td><td>-</td><td>-</td><td>gb|EFA13593.1|</td><td>hypothetical protein TcasGA2_TC001480 [Tribolium castaneum]</td><td>1,00E-34</td></tr>
<tr><td>gALL_v3_c19386</td><td>4</td><td>4</td><td>-</td><td>-</td><td>-</td><td>-</td><td>-</td></tr>
<tr><td>gALL_v3_c19387</td><td>2</td><td>1</td><td>-</td><td>-</td><td>ref|XP_001602535.2|</td><td>PREDICTED: hypothetical protein LOC100118603 [Nasonia vitripennis]</td><td>4,00E-16</td></tr>
<tr><td>gALL_v3_c19388</td><td>2</td><td>4</td><td>-</td><td>-</td><td>-</td><td>-</td><td>-</td></tr>
<tr><td>gALL_v3_c1939</td><td>8</td><td>8</td><td>-</td><td>-</td><td>-</td><td>-</td><td>-</td></tr>
<tr><td>gALL_v3_c19390</td><td>5</td><td>7</td><td>-</td><td>-</td><td>ref|XP_005191923.1|</td><td>PREDICTED: inositol 1,4,5-trisphosphate receptor-like, partial [Musca domestica]</td><td>6,00E-74</td></tr>
<tr><td>gALL_v3_c19391</td><td>1</td><td>3</td><td>-</td><td>-</td><td>-</td><td>-</td><td>-</td></tr>
<tr><td>gALL_v3_c19392</td><td>1</td><td>3</td><td>-</td><td>-</td><td>-</td><td>-</td><td>-</td></tr>
<tr><td>gALL_v3_c19393</td><td>1</td><td>4</td><td>-</td><td>-</td><td>gb|EFN72391.1|</td><td>Transposable element Tc3 transposase [Camponotus floridanus]</td><td>6,00E-08</td></tr>
<tr><td>gALL_v3_c19394</td><td>2</td><td>3</td><td>-</td><td>-</td><td>ref|XP_001847630.1|</td><td>helicase with zinc finger [Culex quinquefasciatus] gb|EDS26531.1| helicase with zinc finger [Culex quinquefasciatus]</td><td>9,00E-39</td></tr>
<tr><td>gALL_v3_c19395</td><td>2</td><td>2</td><td>-</td><td>-</td><td>-</td><td>-</td><td>-</td></tr>
</table>

gALL_v3_c19396	0	4	-	-	ref|XP_002423556.1|	hypothetical protein Phum_PHUM067010 [Pediculus humanus corporis] gb|EEB10818.1| hypothetical protein Phum_PHUM067010 [Pediculus humanus corporis]	4,00E-06
gALL_v3_c19397	2	1	-	-	-	-	-
gALL_v3_c19398	4	3	gnl|CDD|31389	COG1196, Smc, Chromosome	ref|XP_001581403.1|	viral A-type inclusion protein [Trichomonas vaginalis G3] gb|EAY20417.1| viral A-type inclusion protein, putative [Trichomonas vaginalis G3]	2,00E-17
gALL_v3_c19399	3	2	-	-	gb|EFZ16475.1|	hypothetical protein SINV_13267 [Solenopsis invicta]	3,00E-14
gALL_v3_c194	6	12	-	-	ref|XP_001605685.1|	PREDICTED: fatty acid synthase-like [Nasonia vitripennis]	1,00E-23
gALL_v3_c1940	12	20	gnl|CDD|153307	cd07623, BAR_SNX1_2, The Bi	ref|XP_966953.1|	PREDICTED: similar to sorting nexin isoform 1 [Tribolium castaneum] gb|EFA05966.1| hypothetical protein TcasGA2_TC008785 [Tribolium castaneum]	0
gALL_v3_c19400	1	3	-	-	-	-	-
gALL_v3_c19401	3	2	-	-	gb|EFN77311.1|	Alpha-tocopherol transfer protein-like [Harpegnathos saltator]	2,00E-26
gALL_v3_c19403	1	4	-	-	-	-	-
gALL_v3_c19404	1	2	-	-	gb|AEW67354.1|	carbohydrate binding module, partial [Coptotermes formosanus]	1,00E-35
gALL_v3_c19405	1	4	-	-	ref|XP_001947916.2|	PREDICTED: ATP-binding cassette sub-family A member 3-like [Acyrthosiphon pisum]	3,00E-12
gALL_v3_c19406	1	4	-	-	-	-	-
gALL_v3_c19407	3	2	gnl|CDD|31294	COG1097, RRP4, RNA-binding	ref|XP_002427432.1|	Exosome complex exonuclease RRP4, putative [Pediculus humanus corporis] gb|EEB14694.1| Exosome complex exonuclease RRP4, putative [Pediculus humanus corporis]	3,00E-33
gALL_v3_c19408	2	5	-	-	ref|XP_001944844.2|	PREDICTED: hypothetical protein LOC100168206 [Acyrthosiphon pisum]	2,00E-19
gALL_v3_c19409	2	3	-	-	gb|ELT97999.1|	hypothetical protein CAPTEDRAFT_208751 [Capitella teleta]	7,00E-40
gALL_v3_c1941	12	7	gnl|CDD|178765	PLN03226, PLN03226, serine h	gb|EFN60414.1|	Serine hydroxymethyltransferase [Camponotus floridanus]	1,00E-111
gALL_v3_c19410	11	15	-	-	ref|XP_006564251.1|	PREDICTED: myosin-I heavy chain isoform X2 [Apis mellifera]	9,00E-13
gALL_v3_c19411	0	3	-	-	-	-	-
gALL_v3_c19412	4	2	gnl|CDD|34734	COG5133, COG5133, Uncharac	gb|EFZ21581.1|	hypothetical protein SINV_01457 [Solenopsis invicta]	2,00E-54
gALL_v3_c19414	2	5	-	-	ref|XP_001811853.1|	PREDICTED: similar to CG5830 CG5830-PA [Tribolium castaneum] gb|EFA03107.1| hypothetical protein TcasGA2_TC013017 [Tribolium castaneum]	1,00E-19
gALL_v3_c19416	1	3	-	-	-	-	-
gALL_v3_c19417	1	4	-	-	ref|XP_002426255.1|	protein MLP1, putative [Pediculus humanus corporis] gb|EEB13517.1| protein MLP1, putative [Pediculus humanus corporis]	7,00E-28
gALL_v3_c19418	1	3	-	-	-	-	-
gALL_v3_c19420	3	4	-	-	-	-	-
gALL_v3_c19421	5	3	gnl|CDD|190517	pfam03074, GCS, Glutamate-c	ref|XP_003695679.1|	PREDICTED: glutamate--cysteine ligase catalytic subunit isoform 1 [Apis florea]	2,00E-33
gALL_v3_c19422	3	1	-	-	-	-	-
gALL_v3_c19424	3	4	gnl|CDD|192500	pfam10259, Rogdi_lz, Rogdi le	ref|XP_316684.3|	AGAP006653-PB [Anopheles gambiae str. PEST] gb|EAA10769.4| AGAP006653-PB [Anopheles gambiae str. PEST]	1,00E-69
gALL_v3_c19425	2	2	-	-	gb|EFN67634.1|	Putative uncharacterized transposon-derived protein F52C9.6 [Camponotus floridanus]	2,00E-11
gALL_v3_c19427	2	3	gnl|CDD|163230	TIGR03356, BGL, beta-galacto:	gb|AGM32287.1|	beta-glucosidase, partial [Coptotermes formosanus]	4,00E-69
gALL_v3_c19428	0	4	-	-	dbj|BAM17758.1|	elongation factor 1 gamma [Papilio xuthus]	5,00E-11
gALL_v3_c19429	3	2	-	-	-	-	-
gALL_v3_c19430	6	9	-	-	-	-	-
gALL_v3_c19431	2	4	-	-	-	-	-
gALL_v3_c19432	0	4	-	-	ref|XP_003705009.1|	PREDICTED: uncharacterized protein LOC100879930 [Megachile rotundata]	2,00E-13
gALL_v3_c19433	0	5	-	-	-	-	-
gALL_v3_c19434	4	2	-	-	gb|AFZ78842.1|	clan CD family C14 metacaspase-like cysteine peptidase [Coptotermes formosanus]	1,00E-113
gALL_v3_c19435	0	5	-	-	-	-	-
gALL_v3_c19436	4	2	-	-	-	-	-
gALL_v3_c19437	2	3	-	-	-	-	-
gALL_v3_c19438	3	2	-	-	-	-	-
gALL_v3_c19439	2	2	-	-	ref|XP_002427185.1|	hypothetical protein Phum_PHUM300620 [Pediculus humanus corporis] gb|EEB14447.1| hypothetical protein Phum_PHUM300620 [Pediculus humanus corporis]	2,00E-26
gALL_v3_c1944	2	8	gnl|CDD|162101	TIGR00911, 2A0308, L-type an	ref|XP_004928535.1|	PREDICTED: B(0,+)-type amino acid transporter 1-like [Bombyx mori]	5,00E-98
gALL_v3_c19440	3	3	-	-	-	-	-
gALL_v3_c19441	2	2	-	-	ref|XP_003745214.1|	PREDICTED: tigger transposable element-derived protein 6-like [Metaseiulus occidentalis]	2,00E-31
gALL_v3_c19442	3	6	-	-	-	-	-
gALL_v3_c19443	5	3	-	-	-	-	-
gALL_v3_c19444	2	3	-	-	ref|XP_003703290.1|	PREDICTED: ankyrin repeat and BTB/POZ domain-containing protein BTBD11-like [Megachile rotundata]	1,00E-28
gALL_v3_c19446	3	1	-	-	-	-	-
gALL_v3_c19448	0	5	-	-	-	-	-
gALL_v3_c19449	1	3	-	-	-	-	-
gALL_v3_c1945	11	12	-	-	-	-	-
gALL_v3_c19450	4	2	-	-	-	-	-
gALL_v3_c19451	0	5	-	-	-	-	-
gALL_v3_c19452	1	3	-	-	-	-	-
gALL_v3_c19453	3	1	-	-	-	-	-
gALL_v3_c19454	1	6	-	-	-	-	-
gALL_v3_c19456	3	2	-	-	-	-	-
gALL_v3_c19457	3	6	-	-	-	-	-
gALL_v3_c19458	1	2	-	-	-	-	-
gALL_v3_c19459	0	4	-	-	-	-	-
gALL_v3_c1946	5	6	-	-	-	-	-
gALL_v3_c19460	2	2	gnl|CDD|130176	TIGR01106, ATPase-IIC_X-K, so	gb|AFR60475.1|	sodium/potassium-transporting ATPase subunit alpha-1, partial [Bos indicus] gb|AFR60476.1| sodium/potassium-transporting ATPase subunit alpha-1, partial [Bos indicus]	1,00E-09
gALL_v3_c19461	2	3	-	-	pir||PC1123	hypothetical protein - bloodfluke planorb (fragment)	4,00E-08
gALL_v3_c19463	2	1	-	-	-	-	-
gALL_v3_c19464	3	2	-	-	ref|XP_970617.1|	PREDICTED: similar to UPF0171 protein CG8783 [Tribolium castaneum] gb|EFA09890.1| hypothetical protein TcasGA2_TC012039 [Tribolium castaneum]	8,00E-35
gALL_v3_c19465	3	5	-	-	-	-	-
gALL_v3_c19466	4	7	-	-	-	-	-
gALL_v3_c19467	5	4	-	-	-	-	-
gALL_v3_c19468	1	3	-	-	gb|ENN75852.1|	hypothetical protein YQE_07581, partial [Dendroctonus ponderosae]	2,00E-45
gALL_v3_c19469	1	4	-	-	-	-	-
gALL_v3_c1947	5	6	-	-	-	-	-
gALL_v3_c19470	3	2	-	-	ref|XP_970768.2|	PREDICTED: similar to CG10362 CG10362-PA [Tribolium castaneum]	3,00E-57
gALL_v3_c19472	3	7	-	-	ref|XP_001945953.2|	PREDICTED: hypothetical protein LOC100168852 [Acyrthosiphon pisum]	2,00E-08
gALL_v3_c19473	0	4	-	-	-	-	-
gALL_v3_c19474	2	3	gnl|CDD|29257	cd00200, WD40, WD40 domai	ref|XP_003251996.1|	PREDICTED: protein HIRA-like, partial [Apis mellifera]	1,00E-119
gALL_v3_c19475	3	3	-	-	dbj|BAJ79290.1|	geranylgeranyl diphosphate synthase [Reticulitermes speratus]	2,00E-08
gALL_v3_c19478	1	4	gnl|CDD|111841	pfam02995, DUF229, Protein c	ref|XP_003493439.1|	PREDICTED: hypothetical protein LOC100749022 [Bombus impatiens]	7,00E-17
gALL_v3_c19479	2	5	-	-	-	-	-
gALL_v3_c1948	2	6	gnl|CDD|143864	pfam00083, Sugar_tr, Sugar (a	ref|XP_002431804.1|	sugar transporter, putative [Pediculus humanus corporis] gb|EEB19066.1| sugar transporter, putative [Pediculus humanus corporis]	5,00E-69
gALL_v3_c19480	10	22	gnl|CDD|182198	PRK10019, PRK10019, nickel/c	gb|AGM32707.1|	hypothetical protein, partial [Coptotermes formosanus]	6,00E-13
gALL_v3_c19482	1	4	-	-	-	-	-
gALL_v3_c19484	6	11	-	-	ref|XP_003701981.1|	PREDICTED: uncharacterized protein LOC100875110 [Megachile rotundata]	1,00E-107
gALL_v3_c19485	2	2	-	-	-	-	-
gALL_v3_c19486	0	6	-	-	-	-	-
gALL_v3_c19487	3	3	-	-	-	-	-
gALL_v3_c19488	1	5	-	-	-	-	-
gALL_v3_c19489	0	4	-	-	ref|XP_006568352.1|	PREDICTED: protein couch potato-like isoform X4 [Apis mellifera] ref|XP_006611190.1| PREDICTED: U1 small nuclear ribonucleoprotein A-like isoform X5 [Apis dorsata]	3,00E-35
gALL_v3_c19490	2	3	-	-	-	-	-
gALL_v3_c19491	2	6	-	-	-	-	-
gALL_v3_c19492	3	2	-	-	-	-	-
gALL_v3_c19493	1	5	-	-	-	-	-
gALL_v3_c19494	4	4	gnl|CDD|111391	pfam02487, CLN3, CLN3 prote	gb|EFN82426.1|	Battenin [Harpegnathos saltator]	4,00E-36
gALL_v3_c19496	2	1	-	-	gb|EFZ16028.1|	hypothetical protein SINV_08146 [Solenopsis invicta]	1,00E-07
gALL_v3_c19497	2	4	-	-	gb|EFZ10687.1|	hypothetical protein SINV_12257 [Solenopsis invicta]	3,00E-10
gALL_v3_c19498	1	4	-	-	-	-	-
gALL_v3_c19499	1	3	-	-	-	-	-
gALL_v3_c195	19	13	-	-	-	-	-
gALL_v3_c19500	1	4	-	-	ref|XP_002024590.1|	GL22543 [Drosophila persimilis] gb|EDW30038.1| GL22543 [Drosophila persimilis]	8,00E-66
gALL_v3_c19501	14	26	-	-	gb|AGM32695.1|	hypothetical protein [Coptotermes formosanus]	4,00E-22
gALL_v3_c19502	1	6	gnl|CDD|111993	pfam03157, Glutenin_hmw, H	-	-	-
gALL_v3_c19503	2	2	-	-	-	-	-
gALL_v3_c19504	6	6	gnl|CDD|190062	pfam01652, IF4E, Eukaryotic ir	gb|EGI58627.1|	Eukaryotic translation initiation factor 4E [Acromyrmex echinatior]	1,00E-74
gALL_v3_c19505	3	2	-	-	ref|XP_006560315.1|	PREDICTED: SWI/SNF chromatin-remodeling complex subunit sol1 isoform X5 [Apis mellifera]	1,00E-54
gALL_v3_c19506	2	2	gnl|CDD|73154	cd01647, RT_LTR, RT_LTR: Rev	gb|EFA13518.1|	hypothetical protein TcasGA2_TC001491 [Tribolium castaneum]	6,00E-43
gALL_v3_c19507	1	3	-	-	-	-	-
gALL_v3_c19508	2	2	-	-	-	-	-
gALL_v3_c19509	3	3	-	-	-	-	-
gALL_v3_c1951	11	6	gnl|CDD|162378	TIGR01472, gmd, GDP-manno:	ref|XP_005179582.1|	PREDICTED: GDP-mannose 4,6 dehydratase-like [Musca domestica]	1,00E-159
gALL_v3_c19510	1	4	-	-	-	-	-
gALL_v3_c19511	1	5	-	-	-	-	-
gALL_v3_c19512	0	4	-	-	ref|XP_006618557.1|	PREDICTED: sister chromatid cohesion protein PDS5 homolog B-B-like isoform X2 [Apis dorsata]	1,00E-55
gALL_v3_c19513	2	5	-	-	-	-	-
gALL_v3_c19514	2	5	-	-	-	-	-
gALL_v3_c19515	3	2	-	-	-	-	-
gALL_v3_c19516	10	14	-	-	gb|AGM32088.1|	troponin T [Coptotermes formosanus]	4,00E-51
gALL_v3_c19517	2	4	-	-	-	-	-
gALL_v3_c19518	4	2	-	-	-	-	-
gALL_v3_c19519	2	4	-	-	-	-	-
gALL_v3_c19521	3	5	gnl|CDD|190508	pfam03036, Perilipin, Perilipin	ref|XP_005188653.1|	PREDICTED: lipid storage droplets surface-binding protein 2-like isoform X3 [Musca domestica]	3,00E-24
gALL_v3_c19522	3	3	-	-	ref|XP_002429833.1|	rest corepressor corest, protein, putative [Pediculus humanus corporis] gb|EEB17095.1| rest corepressor corest, protein, putative [Pediculus humanus corporis]	1,00E-14
gALL_v3_c19523	1	3	-	-	-	-	-
gALL_v3_c19524	2	2	-	-	-	-	-
gALL_v3_c19525	6	5	-	-	-	-	-
gALL_v3_c19526	2	4	-	-	-	-	-
gALL_v3_c19528	2	2	-	-	-	-	-
gALL_v3_c19529	3	2	gnl|CDD|109113	pfam00044, Gp_dh_N, Glycera	dbj|BAF48670.1|	glyceraldehyde-3-phosphate dehydrogenase [Spirotrichonympha leidyi]	5,00E-93
gALL_v3_c1953	9	20	-	-	-	-	-
gALL_v3_c19530	0	5	-	-	gb|EGI58885.1|	RING finger protein 145 [Acromyrmex echinatior]	7,00E-08
gALL_v3_c19531	16	13	-	-	gb|AGM32253.1|	hypothetical protein [Coptotermes formosanus]	1,00E-149
gALL_v3_c19533	2	4	-	-	-	-	-
gALL_v3_c19534	3	2	-	-	ref|XP_005095693.1|	PREDICTED: putative uncharacterized protein FLJ37770-like [Aplysia californica]	4,00E-10
gALL_v3_c19535	1	5	-	-	-	-	-
gALL_v3_c19536	5	3	-	-	-	-	-
gALL_v3_c19537	0	6	-	-	-	-	-
gALL_v3_c19538	3	1	-	-	-	-	-
gALL_v3_c19539	3	4	-	-	-	-	-
gALL_v3_c1954	15	18	gnl|CDD|133283	cd01883, EF1_alpha, Eukaryot	gb|AGM32606.1|	elongation factor-1 alpha (EF1-alpha) protein family, partial [Coptotermes formosanus]	2,00E-41
gALL_v3_c19540	3	2	-	-	-	-	-
gALL_v3_c19541	4	4	-	-	-	-	-
gALL_v3_c19544	5	4	-	-	-	-	-
gALL_v3_c19545	1	6	-	-	-	-	-
gALL_v3_c19546	3	4	-	-	gb|EFN68620.1|	TBC1 domain family member 8 [Camponotus floridanus]	8,00E-30
gALL_v3_c19547	2	2	-	-	-	-	-
gALL_v3_c19548	4	4	gnl|CDD|132998	cd00897, UGPase_euk, Eukary	ref|XP_005178508.1|	PREDICTED: UTP--glucose-1-phosphate uridylyltransferase-like isoform X2 [Musca domestica]	1,00E-74

gALL_v3_c19549	1	4	-	-	-	-	-
gALL_v3_c1955	28	27	gnl|CDD|178687	PLN03142, PLN03142, Probabl	ref|XP_002427447.1|	Helicase, putative [Pediculus humanus corporis] gb|EEB14709.1| Helicase, putative [Pediculus humanus corporis]	1,00E-121
gALL_v3_c19550	2	3	-	-	-	-	-
gALL_v3_c19551	1	7	-	-	gb|EFN80576.1|	Putative uncharacterized transposon-derived protein F52C9.6 [Harpegnathos saltator]	5,00E-08
gALL_v3_c19552	1	3	-	-	ref|XP_001812942.1|	PREDICTED: similar to ld14 CG12664-PB [Tribolium castaneum]	2,00E-75
gALL_v3_c19553	1	4	gnl|CDD|133346	cd04146, RERG_RasL11_like, F	ref|XP_002430837.1|	conserved hypothetical protein [Pediculus humanus corporis] gb|EEB18099.1| conserved hypothetical protein [Pediculus humanus corporis]	7,00E-51
gALL_v3_c19554	2	2	-	-	-	-	-
gALL_v3_c19555	5	4	-	-	-	-	-
gALL_v3_c19556	3	2	-	-	-	-	-
gALL_v3_c19557	1	5	gnl|CDD|152791	pfam12356, DUF3643, Protein	ref|XP_001844272.1|	survivin [Culex quinquefasciatus] gb|EDS36612.1| survivin [Culex quinquefasciatus]	4,00E-28
gALL_v3_c19558	1	5	-	-	gb|EGI68682.1|	hypothetical protein G5I_02618 [Acromyrmex echinatior]	6,00E-17
gALL_v3_c19560	2	1	-	-	-	-	-
gALL_v3_c19562	5	2	-	-	-	-	-
gALL_v3_c19563	5	4	-	-	-	-	-
gALL_v3_c19564	4	2	-	-	-	-	-
gALL_v3_c19565	2	4	-	-	-	-	-
gALL_v3_c19566	2	2	-	-	gb|ENN78969.1|	hypothetical protein YQE_04574, partial [Dendroctonus ponderosae]	5,00E-23
gALL_v3_c19567	1	4	-	-	gb|EGI59543.1|	hypothetical protein G5I_12326 [Acromyrmex echinatior]	9,00E-08
gALL_v3_c19569	1	4	-	-	-	-	-
gALL_v3_c19570	0	4	-	-	-	-	-
gALL_v3_c19571	2	1	gnl|CDD|30866	COG0520, CsdB, Selenocystein	ref|XP_001952568.2|	PREDICTED: hypothetical protein LOC100161816 [Acyrthosiphon pisum]	5,00E-53
gALL_v3_c19572	1	3	-	-	gb|EHJ77759.1|	DNA-directed DNA polymerase alpha 2 [Danaus plexippus]	1,00E-12
gALL_v3_c19573	2	4	-	-	-	-	-
gALL_v3_c19574	3	5	-	-	-	-	-
gALL_v3_c19575	0	3	-	-	-	-	-
gALL_v3_c19577	3	3	-	-	-	-	-
gALL_v3_c19579	1	2	-	-	ref|XP_002423179.1|	ubiquitin-protein ligase BRE1, putative [Pediculus humanus corporis] gb|EEB10441.1| ubiquitin-protein ligase BRE1, putative [Pediculus humanus corporis]	1,00E-10
gALL_v3_c1958	6	14	gnl|CDD|185547	PTZ00304, PTZ00304, NADH d	ref|XP_002430129.1|	NADH-ubiquinone oxidoreductase 51 kDa subunit, putative [Pediculus humanus corporis] gb|EEB17391.1| NADH-ubiquinone oxidoreductase 51 kDa subunit, putative [Pec	1,00E-127
gALL_v3_c19580	1	4	-	-	-	-	-
gALL_v3_c19581	0	5	-	-	-	-	-
gALL_v3_c19582	4	2	-	-	-	-	-
gALL_v3_c19584	2	3	-	-	-	-	-
gALL_v3_c19585	0	4	gnl|CDD|133343	cd04143, Rhes_like, Rhes_like	ref|XP_002430253.1|	dexras1, putative [Pediculus humanus corporis] gb|EEB17515.1| dexras1, putative [Pediculus humanus corporis]	3,00E-55
gALL_v3_c19586	4	4	-	-	-	-	-
gALL_v3_c19589	3	6	-	-	-	-	-
gALL_v3_c19590	5	9	-	-	ref|XP_003402783.1|	PREDICTED: hypothetical protein LOC100648614 [Bombus terrestris]	6,00E-12
gALL_v3_c19592	3	1	-	-	-	-	-
gALL_v3_c19593	3	2	gnl|CDD|113519	pfam04752, ChaC, ChaC-like p	gb|EFN73813.1|	Cation transport regulator-like protein 1 [Camponotus floridanus]	9,00E-70
gALL_v3_c19594	2	2	-	-	-	-	-
gALL_v3_c19595	3	3	-	-	-	-	-
gALL_v3_c19597	5	2	-	-	-	-	-
gALL_v3_c19598	4	4	gnl|CDD|178445	PLN02853, PLN02853, Probabl	gb|ACK57289.1|	CG2263-like protein, partial [Drosophila affinis]	2,00E-97
gALL_v3_c19599	2	7	-	-	-	-	-
gALL_v3_c196	14	14	-	-	-	-	-
gALL_v3_c1960	6	12	-	-	-	-	-
gALL_v3_c19600	4	5	-	-	-	-	-
gALL_v3_c19601	0	6	-	-	ref|XP_002427021.1|	protein purity of essence, putative [Pediculus humanus corporis] gb|EEB14283.1| protein purity of essence, putative [Pediculus humanus corporis]	2,00E-59
gALL_v3_c19602	5	2	-	-	-	-	-
gALL_v3_c19603	3	4	-	-	-	-	-
gALL_v3_c19604	2	2	-	-	-	-	-
gALL_v3_c19605	1	4	gnl|CDD|144234	pfam00567, TUDOR, Tudor do	gb|EFZ21178.1|	hypothetical protein SINV_07350 [Solenopsis invicta]	3,00E-23
gALL_v3_c19606	2	2	-	-	gb|EFA13373.1|	hypothetical protein TcasGA2_TC001390 [Tribolium castaneum]	3,00E-06
gALL_v3_c19607	3	6	-	-	-	-	-
gALL_v3_c19608	1	2	-	-	-	-	-
gALL_v3_c19609	1	3	-	-	-	-	-
gALL_v3_c1961	10	12	gnl|CDD|189910	pfam01255, Prenyltransf, Puta	gb|EFN89604.1|	Dehydrodolichyl diphosphate synthase [Harpegnathos saltator]	1,00E-75
gALL_v3_c19610	3	4	-	-	-	-	-
gALL_v3_c19612	3	1	-	-	-	-	-
gALL_v3_c19613	1	4	-	-	gb|EFN60957.1|	Cytohesin-1 [Camponotus floridanus]	2,00E-08
gALL_v3_c19614	1	6	-	-	-	-	-
gALL_v3_c19615	1	3	-	-	-	-	-
gALL_v3_c19616	3	2	-	-	-	-	-
gALL_v3_c19617	5	2	-	-	-	-	-
gALL_v3_c19618	1	5	-	-	ref|XP_002426229.1|	hypothetical protein Phum_PHUM243450 [Pediculus humanus corporis] gb|EEB13491.1| hypothetical protein Phum_PHUM243450 [Pediculus humanus corporis]	4,00E-12
gALL_v3_c19619	2	1	-	-	-	-	-
gALL_v3_c1962	14	23	gnl|CDD|128351	smart00036, CNH, Domain fou	ref|XP_001862121.1|	traf2 and nck interacting kinase, tnik [Culex quinquefasciatus] gb|EDS36529.1| traf2 and nck interacting kinase, tnik [Culex quinquefasciatus]	0
gALL_v3_c19620	3	3	-	-	-	-	-
gALL_v3_c19622	1	3	-	-	ref|XP_001606680.2|	PREDICTED: RRP12-like protein-like [Nasonia vitripennis]	2,00E-24
gALL_v3_c19625	1	5	-	-	-	-	-
gALL_v3_c19626	5	3	-	-	-	-	-
gALL_v3_c19627	2	3	-	-	-	-	-
gALL_v3_c19628	4	2	-	-	-	-	-
gALL_v3_c1963	4	3	gnl|CDD|150822	pfam10206, WRW, Mitochond	emb|CBH09270.1|	Similar to CG4692 [Heliconius melpomene]	1,00E-63
gALL_v3_c19630	3	2	-	-	-	-	-
gALL_v3_c19631	3	1	-	-	-	-	-
gALL_v3_c19632	1	4	-	-	-	-	-
gALL_v3_c19633	3	2	-	-	ref|XP_001603111.2|	PREDICTED: protein Red-like [Nasonia vitripennis]	4,00E-09
gALL_v3_c19634	2	3	-	-	ref|XP_001603914.1|	PREDICTED: dysbindin-like [Nasonia vitripennis]	1,00E-71
gALL_v3_c19635	2	5	gnl|CDD|153396	cd07323, LAM, LA motif RNA-I	ref|XP_002432679.1|	lupus la ribonucleoprotein, putative [Pediculus humanus corporis] gb|EEB19941.1| lupus la ribonucleoprotein, putative [Pediculus humanus corporis]	4,00E-67
gALL_v3_c19636	1	4	-	-	-	-	-
gALL_v3_c19637	5	3	gnl|CDD|178675	PLN03129, PLN03129, NADP-c	gb|ETN64604.1|	malic enzyme [Anopheles darlingi]	1,00E-155
gALL_v3_c19639	5	5	-	-	-	-	-
gALL_v3_c1964	5	2	-	-	-	-	-
gALL_v3_c19640	3	8	-	-	-	-	-
gALL_v3_c19642	1	4	-	-	-	-	-
gALL_v3_c19643	4	5	-	-	-	-	-
gALL_v3_c19644	4	3	-	-	-	-	-
gALL_v3_c19645	4	2	-	-	-	-	-
gALL_v3_c19646	1	5	gnl|CDD|176318	cd01242, PH_ROK, Rok (Rho- a	ref|XP_002431014.1|	Rho-associated protein kinase, putative [Pediculus humanus corporis] gb|EEB18276.1| Rho-associated protein kinase, putative [Pediculus humanus corporis]	4,00E-85
gALL_v3_c19647	0	4	-	-	-	-	-
gALL_v3_c19648	1	3	gnl|CDD|145907	pfam03006, HlyIII, Haemolysir	ref|XP_003493655.1|	PREDICTED: LOW QUALITY PROTEIN: ADIPOR-like receptor CG5315-like [Bombus impatiens]	1,00E-104
gALL_v3_c19649	2	2	-	-	-	-	-
gALL_v3_c1965	16	15	gnl|CDD|100087	cd03085, PGM1, Phosphogluc	ref|XP_003493647.1|	PREDICTED: phosphoglucomutase-like [Bombus impatiens]	1,00E-141
gALL_v3_c19650	4	5	gnl|CDD|189507	pfam00335, Tetraspannin, Teti	ref|XP_003695409.1|	PREDICTED: CD9 antigen-like [Apis florea]	5,00E-29
gALL_v3_c19652	1	6	gnl|CDD|73154	cd01647, RT_LTR, RT_LTR: Rev	ref|XP_005156176.1|	PREDICTED: uncharacterized protein LOC101885994 [Danio rerio]	3,00E-21
gALL_v3_c19653	0	5	-	-	ref|XP_001606662.2|	PREDICTED: vacuolar protein sorting-associated protein 13D-like [Nasonia vitripennis]	4,00E-22
gALL_v3_c19654	1	5	-	-	-	-	-
gALL_v3_c19655	1	3	-	-	ref|XP_002738079.1|	PREDICTED: cap-specific mRNA (nucleoside-2'-O-)-methyltransferase 1-like [Saccoglossus kowalevskii]	7,00E-20
gALL_v3_c19656	3	4	-	-	-	-	-
gALL_v3_c19657	3	1	-	-	-	-	-
gALL_v3_c19658	2	5	-	-	-	-	-
gALL_v3_c19659	1	4	-	-	-	-	-
gALL_v3_c1966	4	14	-	-	-	-	-
gALL_v3_c19661	2	3	-	-	-	-	-
gALL_v3_c19663	4	6	-	-	-	-	-
gALL_v3_c19664	3	1	-	-	-	-	-
gALL_v3_c19666	2	4	-	-	-	-	-
gALL_v3_c19667	0	3	-	-	-	-	-
gALL_v3_c19668	1	4	-	-	ref|XP_001945053.2|	PREDICTED: neurexin-4-like [Acyrthosiphon pisum]	9,00E-18
gALL_v3_c19669	3	7	-	-	-	-	-
gALL_v3_c1967	8	9	-	-	-	-	-
gALL_v3_c19670	2	2	-	-	-	-	-
gALL_v3_c19671	5	6	-	-	-	-	-
gALL_v3_c19672	2	4	-	-	-	-	-
gALL_v3_c19673	1	4	-	-	-	-	-
gALL_v3_c19674	1	4	-	-	-	-	-
gALL_v3_c19675	0	4	-	-	-	-	-
gALL_v3_c19676	2	3	-	-	gb|ENN81241.1|	hypothetical protein YQE_02335, partial [Dendroctonus ponderosae]	4,00E-27
gALL_v3_c19678	3	1	-	-	-	-	-
gALL_v3_c19679	5	11	-	-	-	-	-
gALL_v3_c1968	9	7	-	-	-	-	-
gALL_v3_c19681	2	4	gnl|CDD|189654	pfam00651, BTB, BTB/POZ dor	ref|XP_006617778.1|	PREDICTED: protein bric-a-brac 2-like isoform X5 [Apis dorsata]	1,00E-76
gALL_v3_c19682	2	2	-	-	-	-	-
gALL_v3_c19683	2	3	-	-	-	-	-
gALL_v3_c19684	0	5	-	-	-	-	-
gALL_v3_c19685	1	4	-	-	-	-	-
gALL_v3_c19686	1	4	-	-	-	-	-
gALL_v3_c19687	1	5	-	-	-	-	-
gALL_v3_c1969	3	10	-	-	-	-	-
gALL_v3_c19690	2	5	gnl|CDD|147141	pfam04831, Popeye, Popeye p	gb|EFN60407.1|	Blood vessel epicardial substance [Camponotus floridanus]	1,00E-105
gALL_v3_c19691	0	5	gnl|CDD|189583	pfam00520, Ion_trans, Ion trai	dbj|BAO52752.1|	sodium channel [Thrips palmi]	4,00E-89
gALL_v3_c19692	0	6	-	-	-	-	-
gALL_v3_c19693	1	3	-	-	-	-	-
gALL_v3_c19694	1	3	gnl|CDD|190222	pfam02138, Beach, Beige/BEA	ref|XP_006810621.1|	PREDICTED: neurobeachin-like protein 1-like, partial [Neolamprologus brichardi]	2,00E-77
gALL_v3_c19695	2	6	gnl|CDD|173725	cd06608, STKc_myosinIII_like,	ref|XP_003692767.1|	PREDICTED: serine/threonine-protein kinase mig-15-like [Apis florea]	2,00E-83
gALL_v3_c19696	1	2	gnl|CDD|146828	pfam04388, Hamartin, Hamar	gb|EGI62010.1|	Hamartin [Acromyrmex echinatior]	2,00E-26
gALL_v3_c19697	3	8	-	-	-	-	-
gALL_v3_c19698	2	5	-	-	-	-	-
gALL_v3_c19699	1	7	-	-	-	-	-
gALL_v3_c1970	5	5	-	-	gb|EAT40644.2|	AAEL007644-PA, partial [Aedes aegypti]	3,00E-41
gALL_v3_c19701	2	2	-	-	gb|AGM32296.1|	endoglucanase Z family protein, partial [Coptotermes formosanus]	3,00E-28
gALL_v3_c19702	2	6	-	-	gb|AGM32150.1|	ribosomal protein L10Ae [Coptotermes formosanus]	6,00E-10
gALL_v3_c19703	3	3	-	-	-	-	-
gALL_v3_c19704	3	2	-	-	-	-	-

gALL_v3_c19705	4	2	-	-	-	-	-
gALL_v3_c19706	1	3	-	-	-	-	-
gALL_v3_c19707	0	6	-	-	-	-	-
gALL_v3_c19708	2	2	-	-	-	-	-
gALL_v3_c19709	1	6	-	-	ref|XP_312589.3|	AGAP002366-PA [Anopheles gambiae str. PEST] gb|EAA07459.3| AGAP002366-PA [Anopheles gambiae str. PEST]	7,00E-80
gALL_v3_c1971	10	8	-	-	-	-	-
gALL_v3_c19710	0	6	-	-	-	-	-
gALL_v3_c19711	3	3	-	-	-	-	-
gALL_v3_c19712	1	4	-	-	-	-	-
gALL_v3_c19713	2	3	gnl|CDD|143994	pfam00244, 14-3-3, 14-3-3 pro	gb|AFZ78827.1|	14-3-3 protein [Coptotermes formosanus]	7,00E-66
gALL_v3_c19714	3	6	-	-	-	-	-
gALL_v3_c19715	3	8	-	-	-	-	-
gALL_v3_c19716	3	2	-	-	ref|XP_002600234.1|	hypothetical protein BRAFLDRAFT_66738 [Branchiostoma floridae] gb|EEN56246.1| hypothetical protein BRAFLDRAFT_66738 [Branchiostoma floridae]	6,00E-39
gALL_v3_c19717	7	12	gnl|CDD|147776	pfam05804, KAP, Kinesin-asso	ref|XP_002426380.1|	Kinesin-associated protein, putative [Pediculus humanus corporis] gb|EEB13642.1| Kinesin-associated protein, putative [Pediculus humanus corporis]	4,00E-26
gALL_v3_c19719	0	3	-	-	-	-	-
gALL_v3_c1972	2	8	-	-	-	-	-
gALL_v3_c19720	4	5	-	-	-	-	-
gALL_v3_c19721	2	2	-	-	-	-	-
gALL_v3_c19725	1	2	gnl|CDD|73156	cd01650, RT_nLTR_like, RT_nL	ref|XP_004208487.1|	PREDICTED: uncharacterized protein LOC101235837 [Hydra vulgaris]	1,00E-12
gALL_v3_c19726	2	4	-	-	-	-	-
gALL_v3_c19727	2	2	-	-	-	-	-
gALL_v3_c19728	2	2	-	-	-	-	-
gALL_v3_c19729	3	1	gnl|CDD|114396	pfam05670, DUF814, Domain	gb|EFZ09634.1|	hypothetical protein SINV_01682 [Solenopsis invicta]	2,00E-60
gALL_v3_c1973	4	10	gnl|CDD|189654	pfam00651, BTB, BTB/POZ dor	gb|EFA06364.1|	hypothetical protein TcasGA2_TC009240 [Tribolium castaneum]	1,00E-166
gALL_v3_c19730	2	2	-	-	-	-	-
gALL_v3_c19731	4	4	-	-	ref|NP_001127784.2|	tripartite motif-containing 2 protein-like [Nasonia vitripennis]	5,00E-90
gALL_v3_c19732	4	5	-	-	-	-	-
gALL_v3_c19733	5	6	-	-	gb|EHB15654.1|	Zinc finger protein 271 [Heterocephalus glaber]	7,00E-17
gALL_v3_c19735	5	4	-	-	gb|EFN68974.1|	Ubiquitin-conjugating enzyme E2-17 kDa [Camponotus floridanus]	8,00E-26
gALL_v3_c19736	1	5	-	-	-	-	-
gALL_v3_c19737	3	2	-	-	-	-	-
gALL_v3_c19739	2	5	-	-	ref|XP_004932495.1|	PREDICTED: uncharacterized protein LOC101740748 [Bombyx mori]	3,00E-19
gALL_v3_c19740	5	3	-	-	-	-	-
gALL_v3_c19741	1	4	gnl|CDD|128878	smart00630, Sema, semaphor	gb|EHJ75370.1|	hypothetical protein KGM_01509 [Danaus plexippus]	4,00E-64
gALL_v3_c19742	6	11	-	-	-	-	-
gALL_v3_c19743	3	1	-	-	-	-	-
gALL_v3_c19744	0	4	-	-	-	-	-
gALL_v3_c19745	4	2	-	-	-	-	-
gALL_v3_c19746	5	3	-	-	-	-	-
gALL_v3_c19747	0	4	-	-	ref|XP_001599309.2|	PREDICTED: hypothetical protein LOC100114206 [Nasonia vitripennis]	2,00E-06
gALL_v3_c19748	2	2	-	-	-	-	-
gALL_v3_c19749	3	3	-	-	gb|EFZ22389.1|	hypothetical protein SINV_11260 [Solenopsis invicta]	4,00E-35
gALL_v3_c1975	5	6	-	-	ref|XP_006620365.1|	PREDICTED: uncharacterized protein LOC102679753, partial [Apis dorsata]	7,00E-42
gALL_v3_c19750	1	4	-	-	-	-	-
gALL_v3_c19751	3	3	gnl|CDD|190050	pfam01602, Adaptin_N, Adapt	ref|XP_002106880.1|	GD15877 [Drosophila simulans] gb|EDX17849.1| GD15877 [Drosophila simulans]	1,00E-155
gALL_v3_c19752	2	2	-	-	-	-	-
gALL_v3_c19753	2	3	-	-	-	-	-
gALL_v3_c19754	2	4	-	-	-	-	-
gALL_v3_c19755	3	2	-	-	-	-	-
gALL_v3_c19756	3	1	-	-	ref|XP_004438436.1|	PREDICTED: NAC-alpha domain-containing protein 1 [Ceratotherium simum simum]	1,00E-07
gALL_v3_c19757	5	13	-	-	gb|AAX07258.1|	reverse transcriptase [Hadogenes bicolor]	3,00E-10
gALL_v3_c19758	2	4	-	-	-	-	-
gALL_v3_c19759	2	3	-	-	gb|ACC62131.1|	muscle LIM protein-like protein isoform A variant 1 [Bombus hypocrita]	2,00E-25
gALL_v3_c19760	2	4	-	-	-	-	-
gALL_v3_c19761	3	2	gnl|CDD|189372	pfam00067, p450, Cytochrome	ref|XP_001658815.1|	cytochrome P450 [Aedes aegypti] gb|EAT40232.1| AAEL008017-PA [Aedes aegypti]	2,00E-57
gALL_v3_c19762	0	4	gnl|CDD|163659	cd07416, MPP_PP2B, PP2B, m	ref|XP_002432635.1|	serine/threonine-protein phosphatase PP-V, putative [Pediculus humanus corporis] gb|EEB19897.1| serine/threonine-protein phosphatase PP-V, putative [Pediculus huma	2,00E-63
gALL_v3_c19763	2	2	-	-	ref|XP_006821570.1|	PREDICTED: leucine zipper transcription factor-like protein 1-like [Saccoglossus kowalevskii]	8,00E-43
gALL_v3_c19764	1	3	-	-	-	-	-
gALL_v3_c19765	5	3	gnl|CDD|113557	pfam04791, LMBR1, LMBR1-lil	ref|XP_003698409.1|	PREDICTED: LMBR1 domain-containing protein 2 homolog [Apis florea]	3,00E-96
gALL_v3_c19766	2	3	gnl|CDD|132949	cd06618, PKc_MKK7, Catalytic	gb|EPY73046.1|	dual specificity mitogen-activated protein kinase kinase 7 isoform 1, partial [Camelus ferus]	5,00E-82
gALL_v3_c19767	2	3	-	-	-	-	-
gALL_v3_c19768	3	4	-	-	-	-	-
gALL_v3_c19769	2	2	-	-	-	-	-
gALL_v3_c1977	6	9	-	-	-	-	-
gALL_v3_c19770	2	2	-	-	-	-	-
gALL_v3_c19771	5	3	-	-	-	-	-
gALL_v3_c19773	0	4	-	-	-	-	-
gALL_v3_c19774	2	4	-	-	-	-	-
gALL_v3_c19775	3	6	-	-	-	-	-
gALL_v3_c19777	8	8	-	-	-	-	-
gALL_v3_c19778	0	4	-	-	ref|XP_003708653.1|	PREDICTED: uncharacterized protein LOC100876451 [Megachile rotundata]	2,00E-34
gALL_v3_c19779	2	3	-	-	-	-	-
gALL_v3_c1978	9	10	gnl|CDD|192040	pfam08441, Integrin_alpha2, I	ref|XP_001947330.2|	PREDICTED: integrin alpha-PS1-like isoform 1 [Acyrthosiphon pisum]	1,00E-105
gALL_v3_c19780	3	7	gnl|CDD|153276	cd07592, BAR_Endophilin_A, '	ref|XP_006613393.1|	PREDICTED: endophilin-A-like isoform X13 [Apis dorsata]	1,00E-141
gALL_v3_c19781	1	3	-	-	-	-	-
gALL_v3_c19783	6	3	-	-	gb|EFX88494.1|	hypothetical protein DAPPUDRAFT_41548 [Daphnia pulex]	1,00E-06
gALL_v3_c19784	1	3	-	-	-	-	-
gALL_v3_c19785	2	1	-	-	gb|EHJ68999.1|	hypothetical protein KGM_17101 [Danaus plexippus]	2,00E-13
gALL_v3_c19786	3	4	-	-	-	-	-
gALL_v3_c19787	2	4	-	-	-	-	-
gALL_v3_c19788	3	2	-	-	-	-	-
gALL_v3_c19789	3	4	gnl|CDD|191077	pfam04749, PLAC8, PLAC8 fam	-	-	-
gALL_v3_c19790	1	5	-	-	-	-	-
gALL_v3_c19792	0	4	-	-	ref|XP_975384.1|	PREDICTED: similar to AGAP011535-PA [Tribolium castaneum] gb|EFA07009.1| hypothetical protein TcasGA2_TC009979 [Tribolium castaneum]	8,00E-23
gALL_v3_c19793	8	12	-	-	ref|XP_001619201.1|	hypothetical protein NEMVEDRAFT_v1g152081 [Nematostella vectensis] gb|EDO27101.1| predicted protein [Nematostella vectensis]	4,00E-07
gALL_v3_c19794	2	3	-	-	-	-	-
gALL_v3_c19797	2	8	gnl|CDD|189654	pfam00651, BTB, BTB/POZ dor	gb|EKC32091.1|	Ankyrin repeat and BTB/POZ domain-containing protein 1 [Crassostrea gigas]	2,00E-82
gALL_v3_c19798	4	5	-	-	-	-	-
gALL_v3_c19799	4	3	gnl|CDD|185540	PTZ00288, PTZ00288, glucokin	ref|XP_001327217.1|	hypothetical protein [Trichomonas vaginalis G3] gb|EAY14994.1| hypothetical protein TVAG_397250 [Trichomonas vaginalis G3]	2,00E-42
gALL_v3_c198	12	27	gnl|CDD|128516	smart00220, S_TKc, Serine/Th	ref|XP_002422947.1|	cAMP-dependent protein kinase catalytic subunit, putative [Pediculus humanus corporis] gb|EEB10209.1| cAMP-dependent protein kinase catalytic subunit, putative [Pedi	0
gALL_v3_c1980	20	13	gnl|CDD|100104	cd00590, RRM, RRM (RNA rec	ref|XP_003486336.1|	PREDICTED: myelin expression factor 2-like [Bombus impatiens]	5,00E-24
gALL_v3_c19800	1	4	-	-	-	-	-
gALL_v3_c19801	1	4	gnl|CDD|30300	cd02679, MIT_spastin, MIT: dc	ref|XP_006608089.1|	PREDICTED: spastin-like isoform X2 [Apis dorsata]	4,00E-52
gALL_v3_c19802	2	2	-	-	-	-	-
gALL_v3_c19803	5	2	-	-	-	-	-
gALL_v3_c19804	2	2	-	-	-	-	-
gALL_v3_c19805	4	3	-	-	-	-	-
gALL_v3_c19806	4	2	-	-	-	-	-
gALL_v3_c19807	1	4	-	-	-	-	-
gALL_v3_c1981	12	11	-	-	-	-	-
gALL_v3_c19810	3	3	-	-	-	-	-
gALL_v3_c19811	2	1	gnl|CDD|29261	cd00204, ANK, ankyrin repeats	ref|XP_002428411.1|	ankyrin repeat domain-containing protein, putative [Pediculus humanus corporis] gb|EEB15673.1| ankyrin repeat domain-containing protein, putative [Pediculus humanus	8,00E-36
gALL_v3_c19812	2	8	-	-	-	-	-
gALL_v3_c19813	3	2	-	-	gb|ABG82006.1|	putative elongation factor 1 beta' [Diaphorina citri]	2,00E-11
gALL_v3_c19814	0	5	-	-	-	-	-
gALL_v3_c19815	2	2	-	-	-	-	-
gALL_v3_c19816	0	6	-	-	-	-	-
gALL_v3_c19817	1	5	-	-	-	-	-
gALL_v3_c19819	1	4	-	-	-	-	-
gALL_v3_c1982	28	22	gnl|CDD|29138	cd00176, SPEC, Spectrin repea	ref|XP_006607773.1|	PREDICTED: spectrin beta chain, non-erythrocytic 5-like isoform X4 [Apis dorsata] ref|XP_006607774.1| PREDICTED: spectrin beta chain, non-erythrocytic 5-like isoform X5	0
gALL_v3_c19820	4	2	-	-	-	-	-
gALL_v3_c19821	2	2	-	-	-	-	-
gALL_v3_c19822	2	5	-	-	-	-	-
gALL_v3_c19824	3	2	-	-	-	-	-
gALL_v3_c19825	2	6	-	-	-	-	-
gALL_v3_c19826	0	3	-	-	-	-	-
gALL_v3_c19828	2	3	-	-	-	-	-
gALL_v3_c19829	3	4	-	-	-	-	-
gALL_v3_c1983	2	5	-	-	ref|XP_001599903.2|	PREDICTED: TBC1 domain family member 4-like isoform 1 [Nasonia vitripennis]	4,00E-08
gALL_v3_c19830	2	3	-	-	-	-	-
gALL_v3_c19831	0	4	-	-	-	-	-
gALL_v3_c19832	3	2	-	-	-	-	-
gALL_v3_c19834	3	1	-	-	gb|ERL85594.1|	hypothetical protein D910_03013 [Dendroctonus ponderosae]	5,00E-14
gALL_v3_c19835	2	2	-	-	-	-	-
gALL_v3_c19837	2	4	-	-	ref|XP_002423980.1|	NADPH--cytochrome P450, putative [Pediculus humanus corporis] gb|EEB11242.1| NADPH--cytochrome P450, putative [Pediculus humanus corporis]	1,00E-24
gALL_v3_c19838	2	2	-	-	-	-	-
gALL_v3_c19839	3	12	gnl|CDD|179088	PRK00696, sucC, succinyl-CoA	gb|AEE61406.1|	unknown [Dendroctonus ponderosae] gb|ERL83818.1| hypothetical protein D910_01070 [Dendroctonus ponderosae] gb|ERL93444.1| hypothetical protein D910_10736 [	1,00E-112
gALL_v3_c1984	7	11	-	-	ref|XP_006621456.1|	PREDICTED: transcription elongation factor SPT6-like isoform X2 [Apis dorsata]	2,00E-50
gALL_v3_c19840	2	3	-	-	-	-	-
gALL_v3_c19842	2	5	-	-	-	-	-
gALL_v3_c19843	2	5	-	-	-	-	-
gALL_v3_c19844	4	6	gnl|CDD|58587	cd04317, EcAspRS_like_N, EcA	ref|XP_005175374.1|	PREDICTED: aspartate--tRNA ligase, mitochondrial-like [Musca domestica]	1,00E-27
gALL_v3_c19845	0	4	-	-	ref|XP_392977.4|	PREDICTED: sodium-coupled monocarboxylate transporter 2-like isoformX1 [Apis mellifera]	3,00E-24
gALL_v3_c19846	1	2	-	-	-	-	-
gALL_v3_c19847	4	2	-	-	ref|XP_002426333.1|	DNA double-strand break repair Rad50 ATPase, putative [Pediculus humanus corporis] gb|EEB13595.1| DNA double-strand break repair Rad50 ATPase, putative [Pediculus	1,00E-23
gALL_v3_c19848	0	4	-	-	-	-	-
gALL_v3_c19849	0	6	-	-	-	-	-
gALL_v3_c19850	3	1	-	-	-	-	-
gALL_v3_c19851	1	6	-	-	ref|XP_003493381.1|	PREDICTED: alpha-(1,3)-fucosyltransferase 10-like [Bombus impatiens]	6,00E-10
gALL_v3_c19853	8	15	gnl|CDD|128621	smart00326, SH3, Src homolog	ref|XP_003701333.1|	PREDICTED: uncharacterized protein LOC100875671 [Megachile rotundata]	1,00E-115
gALL_v3_c19854	2	3	-	-	-	-	-
gALL_v3_c19855	2	4	-	-	-	-	-
gALL_v3_c19856	2	4	-	-	gb|EFA13593.1|	hypothetical protein TcasGA2_TC001480 [Tribolium castaneum]	1,00E-16
gALL_v3_c19857	4	2	-	-	-	-	-

gALL_v3_c19858	6	7	-	-	-	-	-
gALL_v3_c19859	3	9	-	-	-	-	-
gALL_v3_c1986	12	15	-	-	-	-	-
gALL_v3_c19860	3	5	-	-	-	-	-
gALL_v3_c19861	9	8	-	-	-	-	-
gALL_v3_c19862	3	1	-	-	-	-	-
gALL_v3_c19864	19	25	-	-	-	-	-
gALL_v3_c19865	3	3	-	-	-	-	-
gALL_v3_c19866	2	2	-	-	-	-	-
gALL_v3_c19867	2	3	gnl|CDD|29257	cd00200, WD40, WD40 domai	gb|EFA08346.1|	hypothetical protein TcasGA2_TC005989 [Tribolium castaneum]	1,00E-94
gALL_v3_c19868	2	2	-	-	ref|XP_002428357.1|	conserved hypothetical protein [Pediculus humanus corporis] gb|EEB15619.1| conserved hypothetical protein [Pediculus humanus corporis]	4,00E-55
gALL_v3_c1987	8	10	-	-	ref|XP_001370600.2|	PREDICTED: serine/threonine-protein phosphatase 4 regulatory subunit 1 [Monodelphis domestica]	2,00E-93
gALL_v3_c19870	14	17	-	-	-	-	-
gALL_v3_c19871	5	2	-	-	-	-	-
gALL_v3_c19872	3	1	-	-	ref|XP_002427883.1|	conserved hypothetical protein [Pediculus humanus corporis] gb|EEB15145.1| conserved hypothetical protein [Pediculus humanus corporis]	3,00E-99
gALL_v3_c19873	4	2	-	-	-	-	-
gALL_v3_c19874	5	2	-	-	-	-	-
gALL_v3_c19875	1	3	-	-	-	-	-
gALL_v3_c19876	3	2	-	-	-	-	-
gALL_v3_c19877	3	2	-	-	gb|EFA13594.1|	hypothetical protein TcasGA2_TC001481 [Tribolium castaneum]	1,00E-20
gALL_v3_c19879	4	10	gnl|CDD|153400	cd08031, LARP_4_5_like, La RI	ref|XP_002432403.1|	lupus la ribonucleoprotein, putative [Pediculus humanus corporis] gb|EEB19665.1| lupus la ribonucleoprotein, putative [Pediculus humanus corporis]	1,00E-122
gALL_v3_c1988	5	5	gnl|CDD|162185	TIGR01065, hlyIII, channel pro	ref|XP_003706341.1|	PREDICTED: monocyte to macrophage differentiation factor 2-like [Megachile rotundata]	2,00E-76
gALL_v3_c19880	2	3	-	-	-	-	-
gALL_v3_c19881	2	4	-	-	gb|EFN79589.1|	hypothetical protein EAI_10425 [Harpegnathos saltator]	8,00E-11
gALL_v3_c19882	2	3	-	-	-	-	-
gALL_v3_c19883	2	4	-	-	-	-	-
gALL_v3_c19884	4	2	-	-	-	-	-
gALL_v3_c19885	2	1	-	-	-	-	-
gALL_v3_c19886	8	5	gnl|CDD|185453	PTZ00110, PTZ00110, helicase	ref|XP_006615333.1|	PREDICTED: probable ATP-dependent RNA helicase DDX17-like isoform X2 [Apis dorsata]	3,00E-56
gALL_v3_c19888	3	3	-	-	-	-	-
gALL_v3_c19889	4	2	-	-	-	-	-
gALL_v3_c1989	17	18	gnl|CDD|190545	pfam03151, TPT, Triose-phosp	ref|XP_002431677.1|	conserved hypothetical protein [Pediculus humanus corporis] gb|EEB18939.1| conserved hypothetical protein [Pediculus humanus corporis]	4,00E-71
gALL_v3_c19890	0	4	-	-	-	-	-
gALL_v3_c19891	0	4	-	-	-	-	-
gALL_v3_c19892	1	3	-	-	-	-	-
gALL_v3_c19893	2	3	gnl|CDD|146288	pfam03571, Peptidase_M49, F	ref|XP_001601820.1|	PREDICTED: dipeptidyl peptidase 3-like [Nasonia vitripennis]	7,00E-68
gALL_v3_c19894	3	2	gnl|CDD|72942	cd04657, Piwi_ago-like, Piwi_a	emb|CCV01213.1|	Argonaute-2, partial [Blattella germanica]	3,00E-58
gALL_v3_c19897	1	3	-	-	-	-	-
gALL_v3_c19899	2	1	gnl|CDD|189424	pfam00153, Mito_carr, Mitoch	gb|EFZ21053.1|	hypothetical protein SINV_12577 [Solenopsis invicta]	2,00E-41
gALL_v3_c199	20	16	gnl|CDD|150455	pfam09788, Tmemb_55A, Trar	gb|EFN80590.1|	Transmembrane protein 55B [Harpegnathos saltator]	1,00E-116
gALL_v3_c19900	3	4	gnl|CDD|146001	pfam03159, XRN_N, XRN 5'-3'	gb|EFA06899.1|	hypothetical protein TcasGA2_TC009846 [Tribolium castaneum]	3,00E-71
gALL_v3_c19902	5	3	-	-	-	-	-
gALL_v3_c19905	3	4	-	-	ref|XP_002425307.1|	hypothetical protein Phum_PHUM191180 [Pediculus humanus corporis] gb|EEB12569.1| hypothetical protein Phum_PHUM191180 [Pediculus humanus corporis]	2,00E-19
gALL_v3_c19906	3	4	-	-	-	-	-
gALL_v3_c19907	2	2	-	-	-	-	-
gALL_v3_c19908	5	8	-	-	gb|EGI60277.1|	Lissencephaly-1-like protein [Acromyrmex echinatior]	5,00E-75
gALL_v3_c19909	1	2	-	-	-	-	-
gALL_v3_c1991	6	7	gnl|CDD|130689	TIGR01628, PABP-1234, polyac	gb|EFZ12586.1|	hypothetical protein SINV_05042 [Solenopsis invicta]	7,00E-80
gALL_v3_c19910	0	5	-	-	-	-	-
gALL_v3_c19912	2	3	-	-	-	-	-
gALL_v3_c19913	2	5	gnl|CDD|180111	PRK05477, gatB, aspartyl/gluta	ref|XP_001654104.1|	glutamyl-tRNA(Gln) amidotransferase, subunit-B, putative [Aedes aegypti] sp|Q16UG2.1|GATB_AEDAE RecName: Full=Glutamyl-tRNA(Gln) amidotransferase subunit B, mi	1,00E-74
gALL_v3_c19914	2	4	-	-	-	-	-
gALL_v3_c19915	2	7	-	-	-	-	-
gALL_v3_c19916	3	1	-	-	gb|ACX54561.1|	laccase 13 [Reticulitermes flavipes]	1,00E-33
gALL_v3_c19917	2	8	-	-	-	-	-
gALL_v3_c19918	4	4	-	-	-	-	-
gALL_v3_c19919	0	4	-	-	-	-	-
gALL_v3_c1992	5	7	-	-	-	-	-
gALL_v3_c19920	2	2	-	-	-	-	-
gALL_v3_c19921	0	4	-	-	-	-	-
gALL_v3_c19922	4	3	gnl|CDD|173637	cd05059, PTKc_Tec_like, Catal	ref|XP_006620520.1|	PREDICTED: tyrosine-protein kinase Btk29A-like isoform X7 [Apis dorsata] ref|XP_006620521.1| PREDICTED: tyrosine-protein kinase Btk29A-like isoform X8 [Apis dorsata]	1,00E-137
gALL_v3_c19923	2	3	gnl|CDD|189377	pfam00076, RRM_1, RNA reco	ref|XP_006566487.1|	PREDICTED: serine/arginine-rich splicing factor 4 isoform X3 [Apis mellifera] ref|XP_006614327.1| PREDICTED: serine/arginine-rich splicing factor 4-like isoform X3 [Apis do	1,00E-38
gALL_v3_c19924	3	5	gnl|CDD|29261	cd00204, ANK, ankyrin repeats	ref|XP_003708179.1|	PREDICTED: ankyrin repeat domain-containing protein 29-like [Megachile rotundata]	2,00E-86
gALL_v3_c19925	2	3	-	-	-	-	-
gALL_v3_c19926	1	4	-	-	-	-	-
gALL_v3_c19927	1	4	-	-	-	-	-
gALL_v3_c19928	2	3	-	-	gb|AAA28507.1|	binding protein (put.); putative, partial [Drosophila melanogaster]	3,00E-18
gALL_v3_c19929	0	5	-	-	-	-	-
gALL_v3_c19930	0	4	-	-	-	-	-
gALL_v3_c19931	1	4	gnl|CDD|132774	cd06864, PX_SNX4, The phosp	ref|XP_006624525.1|	PREDICTED: sorting nexin-4-like [Apis dorsata]	1,00E-107
gALL_v3_c19932	2	5	gnl|CDD|189373	pfam00069, Pkinase, Protein k	ref|XP_002431275.1|	conserved hypothetical protein [Pediculus humanus corporis] gb|EEB18537.1| conserved hypothetical protein [Pediculus humanus corporis]	2,00E-57
gALL_v3_c19933	3	2	-	-	-	-	-
gALL_v3_c19934	2	2	-	-	-	-	-
gALL_v3_c19935	1	4	-	-	-	-	-
gALL_v3_c19936	2	5	-	-	gb|AGM32470.1|	FK506-binding protein [Coptotermes formosanus]	8,00E-37
gALL_v3_c19937	0	5	gnl|CDD|193305	pfam12830, Nipped-B_C, Siste	ref|XP_002423090.1|	conserved hypothetical protein [Pediculus humanus corporis] gb|EEB10352.1| conserved hypothetical protein [Pediculus humanus corporis]	2,00E-64
gALL_v3_c19938	2	2	-	-	-	-	-
gALL_v3_c19939	2	5	gnl|CDD|190262	pfam02259, FAT, FAT domain.	gb|ACH47049.1|	target of rapamycin [Blattella germanica]	1,00E-103
gALL_v3_c19941	3	5	-	-	-	-	-
gALL_v3_c19942	2	1	-	-	-	-	-
gALL_v3_c19944	2	3	-	-	-	-	-
gALL_v3_c19945	1	3	gnl|CDD|34965	COG5406, COG5406, Nucleosc	gb|EFZ18248.1|	hypothetical protein SINV_12622 [Solenopsis invicta]	1,00E-63
gALL_v3_c19946	2	4	-	-	ref|XP_006819348.1|	PREDICTED: uncharacterized protein LOC100374480 [Saccoglossus kowalevskii]	7,00E-21
gALL_v3_c19947	9	7	-	-	-	-	-
gALL_v3_c19948	4	2	-	-	-	-	-
gALL_v3_c19949	1	4	-	-	-	-	-
gALL_v3_c19950	1	6	-	-	-	-	-
gALL_v3_c19952	1	3	-	-	-	-	-
gALL_v3_c19953	0	4	-	-	-	-	-
gALL_v3_c19954	3	4	-	-	-	-	-
gALL_v3_c19955	0	4	-	-	-	-	-
gALL_v3_c19956	6	4	gnl|CDD|189669	pfam00687, Ribosomal_L1, Ril	ref|XP_623930.2|	PREDICTED: ribosomal L1 domain-containing protein CG13096-like [Apis mellifera]	2,00E-39
gALL_v3_c19957	2	5	-	-	-	-	-
gALL_v3_c19958	1	4	gnl|CDD|189485	pfam00282, Pyridoxal_deC, Py	gb|ERL89786.1|	hypothetical protein D910_07147 [Dendroctonus ponderosae] gb|ERL95860.1| hypothetical protein D910_00475 [Dendroctonus ponderosae] gb|ERL95995.1| hypothetic	7,00E-74
gALL_v3_c19959	3	1	gnl|CDD|189424	pfam00153, Mito_carr, Mitoch	ref|XP_316164.4|	AGAP006104-PA [Anopheles gambiae str. PEST] gb|EAA11277.4| AGAP006104-PA [Anopheles gambiae str. PEST]	4,00E-85
gALL_v3_c19960	3	1	-	-	-	-	-
gALL_v3_c19961	1	3	-	-	-	-	-
gALL_v3_c19964	5	5	-	-	-	-	-
gALL_v3_c19965	0	4	gnl|CDD|190558	pfam03190, DUF255, Protein c	gb|EFX84155.1|	hypothetical protein DAPPUDRAFT_47524 [Daphnia pulex]	2,00E-76
gALL_v3_c19966	3	2	-	-	-	-	-
gALL_v3_c19967	6	12	gnl|CDD|73156	cd01650, RT_nLTR_like, RT_nL	gb|ADI61823.1|	endonuclease-reverse transcriptase [Bombyx mori]	2,00E-49
gALL_v3_c19969	2	4	-	-	-	-	-
gALL_v3_c19970	3	9	-	-	-	-	-
gALL_v3_c19971	4	5	-	-	ref|XP_003708062.1|	PREDICTED: protein GPR107-like [Megachile rotundata]	8,00E-43
gALL_v3_c19972	1	3	-	-	-	-	-
gALL_v3_c19973	3	6	-	-	gb|AGM32459.1|	pacifastin-related peptide precursor [Coptotermes formosanus]	1,00E-08
gALL_v3_c19974	2	3	-	-	-	-	-
gALL_v3_c19975	3	5	-	-	-	-	-
gALL_v3_c19976	3	3	-	-	ref|XP_001811693.1|	PREDICTED: similar to AGAP002517-PA [Tribolium castaneum] gb|EEZ98980.1| hypothetical protein TcasGA2_TC004841 [Tribolium castaneum]	2,00E-07
gALL_v3_c19977	1	6	-	-	-	-	-
gALL_v3_c19978	4	5	gnl|CDD|177910	PLN02269, PLN02269, Pyruvat	ref|XP_974523.1|	PREDICTED: similar to pyruvate dehydrogenase [Tribolium castaneum] gb|EFA00132.1| hypothetical protein TcasGA2_TC002948 [Tribolium castaneum]	4,00E-35
gALL_v3_c19979	3	9	-	-	-	-	-
gALL_v3_c1998	8	5	-	-	-	-	-
gALL_v3_c19980	4	3	-	-	ref|XP_001187910.1|	PREDICTED: craniofacial development protein 2-like [Strongylocentrotus purpuratus]	1,00E-36
gALL_v3_c19981	2	2	-	-	-	-	-
gALL_v3_c19982	4	2	-	-	gb|AGO14414.1|	hypothetical protein CsmBAC4b19.5 [Cotesia sesamiae]	8,00E-15
gALL_v3_c19983	2	5	-	-	ref|XP_006619089.1|	PREDICTED: uncharacterized protein LOC102675378 [Apis dorsata]	2,00E-52
gALL_v3_c19984	1	3	-	-	-	-	-
gALL_v3_c19985	6	8	-	-	-	-	-
gALL_v3_c19986	3	1	gnl|CDD|32933	COG3119, AslA, Arylsulfatase /	ref|XP_002432133.1|	arylsulfatase B precursor, putative [Pediculus humanus corporis] gb|EEB19395.1| arylsulfatase B precursor, putative [Pediculus humanus corporis]	1,00E-62
gALL_v3_c19987	2	4	-	-	gb|EFA12829.1|	hypothetical protein TcasGA2_TC015881 [Tribolium castaneum]	2,00E-22
gALL_v3_c19988	5	3	gnl|CDD|193568	cd08022, M28_PSMA_like, M2	gb|EFX83733.1|	hypothetical protein DAPPUDRAFT_315645 [Daphnia pulex]	2,00E-34
gALL_v3_c19989	3	3	gnl|CDD|128548	smart00252, SH2, Src homolog	ref|XP_002423307.1|	cAMP-dependent protein kinase catalytic subunit, putative [Pediculus humanus corporis] gb|EEB10569.1| cAMP-dependent protein kinase catalytic subunit, putative [Pedi	2,00E-71
gALL_v3_c1999	19	23	gnl|CDD|189635	pfam00621, RhoGEF, RhoGEF c	gb|EFN82420.1|	FYVE, RhoGEF and PH domain-containing protein 4 [Harpegnathos saltator]	0
gALL_v3_c19990	2	8	-	-	-	-	-
gALL_v3_c19991	1	3	-	-	-	-	-
gALL_v3_c19992	3	4	-	-	-	-	-
gALL_v3_c19994	2	2	-	-	-	-	-
gALL_v3_c19995	2	2	-	-	-	-	-
gALL_v3_c19996	3	2	-	-	ref|XP_001659879.1|	aaa atpase [Aedes aegypti] gb|EAT38889.1| AAEL009254-PA [Aedes aegypti]	8,00E-10
gALL_v3_c19997	3	1	-	-	-	-	-
gALL_v3_c19999	3	3	-	-	-	-	-
gALL_v3_c2	11	13	-	-	-	-	-
gALL_v3_c200	5	10	-	-	-	-	-
gALL_v3_c2000	8	4	gnl|CDD|191211	pfam05154, TM2, TM2 domai	gb|EFZ18641.1|	hypothetical protein SINV_10993 [Solenopsis invicta]	2,00E-70
gALL_v3_c20000	0	6	-	-	-	-	-
gALL_v3_c20001	4	2	-	-	gb|EFN74593.1|	Protein C1orf9-like protein [Camponotus floridanus]	5,00E-34
gALL_v3_c20002	1	5	-	-	-	-	-
gALL_v3_c20003	2	4	-	-	-	-	-
gALL_v3_c20004	1	5	gnl|CDD|177210	MTH00153, COX1, cytochrome	gb|AEF56583.1|	cytochrome oxidase subunit I [Coptotermes frenchi]	2,00E-32
gALL_v3_c20005	1	3	-	-	-	-	-
gALL_v3_c20007	2	4	-	-	-	-	-
gALL_v3_c20008	3	3	-	-	-	-	-
gALL_v3_c20009	1	8	gnl|CDD|166599	PLN02958, PLN02958, diacylgl	ref|XP_003708035.1|	PREDICTED: sphingosine kinase 1-like, partial [Megachile rotundata]	1,00E-100

gALL_v3_c2001	5	13	-	-	-	-	-	
gALL_v3_c20010	4	2	gnl|CDD|189621	pfam00595, PDZ, PDZ domain	gb|EGI62796.1|	Segment polarity protein dishevelled-like protein DVL-3 [Acromyrmex echinatior]		6,00E-72
gALL_v3_c20011	4	2	-	-	-	-	-	
gALL_v3_c20012	1	3	-	-	-	-	-	
gALL_v3_c20013	3	3	-	-	-	-	-	
gALL_v3_c20014	3	4	-	-	-	-	-	
gALL_v3_c20015	0	3	-	-	gb|EEZ97267.1|	hypothetical protein TcasGA2_TC011068 [Tribolium castaneum]		1,00E-39
gALL_v3_c20017	1	5	-	-	-	-	-	
gALL_v3_c20019	3	7	-	-	ref|XP_004928419.1|	PREDICTED: uncharacterized protein LOC101746992 [Bombyx mori]		2,00E-25
gALL_v3_c2002	5	8	gnl|CDD|189372	pfam00067, p450, Cytochrom	gb|ETN65670.1|	cytochrome P450 6a8 [Anopheles darlingi]		6,00E-68
gALL_v3_c20020	2	2	-	-	-	-	-	
gALL_v3_c20021	1	3	-	-	-	-	-	
gALL_v3_c20022	2	2	-	-	-	-	-	
gALL_v3_c20023	2	5	gnl|CDD|113931	pfam05179, CDC73, RNA pol II	ref|XP_006607558.1|	PREDICTED: parafibromin-like [Apis dorsata]		2,00E-33
gALL_v3_c20024	2	2	-	-	-	-	-	
gALL_v3_c20026	2	3	-	-	-	-	-	
gALL_v3_c20027	3	4	-	-	-	-	-	
gALL_v3_c20028	2	7	-	-	gb|EFN67634.1|	Putative uncharacterized transposon-derived protein F52C9.6 [Camponotus floridanus]		4,00E-23
gALL_v3_c20029	9	5	gnl|CDD|148139	pfam06346, Drf_FH1, Formin I	-	-	-	
gALL_v3_c20030	3	2	-	-	-	-	-	
gALL_v3_c20031	1	7	-	-	-	-	-	
gALL_v3_c20032	3	3	-	-	-	-	-	
gALL_v3_c20033	2	2	-	-	-	-	-	
gALL_v3_c20035	1	6	-	-	-	-	-	
gALL_v3_c20036	3	2	-	-	-	-	-	
gALL_v3_c20037	2	3	-	-	-	-	-	
gALL_v3_c20038	4	3	gnl|CDD|183363	PRK11892, PRK11892, pyruvat	ref|XP_001945646.2|	PREDICTED: dihydrolipoyllysine-residue acetyltransferase component 1 of pyruvate dehydrogenase complex, mitochondrial-like [Acyrthosiphon pisum]		9,00E-75
gALL_v3_c20039	4	2	-	-	gb|AGM32707.1|	hypothetical protein, partial [Coptotermes formosanus]		3,00E-12
gALL_v3_c2004	6	6	gnl|CDD|110234	pfam01217, Clat_adaptor_s, C	ref|XP_975476.1|	PREDICTED: similar to clathrin coat assembly protein ap17 [Tribolium castaneum] ref|XP_004925710.1| PREDICTED: AP-2 complex subunit sigma-like isoform X1 [Bombyx r		8,00E-89
gALL_v3_c20040	5	10	-	-	-	-	-	
gALL_v3_c20041	4	4	-	-	-	-	-	
gALL_v3_c20042	2	3	-	-	-	-	-	
gALL_v3_c20043	3	3	-	-	-	-	-	
gALL_v3_c20044	1	3	-	-	-	-	-	
gALL_v3_c20045	3	1	-	-	-	-	-	
gALL_v3_c20047	1	3	-	-	-	-	-	
gALL_v3_c20048	3	2	-	-	-	-	-	
gALL_v3_c20049	2	2	-	-	gb|EGI59805.1|	FLJ37770-like protein [Acromyrmex echinatior]		2,00E-10
gALL_v3_c2005	6	4	-	-	-	-	-	
gALL_v3_c20050	1	7	-	-	gb|EFN65619.1|	Putative uncharacterized transposon-derived protein F52C9.6 [Camponotus floridanus]		2,00E-10
gALL_v3_c20051	0	5	-	-	-	-	-	
gALL_v3_c20052	1	3	-	-	-	-	-	
gALL_v3_c20054	1	3	-	-	-	-	-	
gALL_v3_c20055	0	3	-	-	ref|XP_004931077.1|	PREDICTED: mitochondrial import inner membrane translocase subunit Tim21-like [Bombyx mori]		2,00E-17
gALL_v3_c20056	4	5	-	-	gb|EFN83807.1|	Probable phosphorylase b kinase regulatory subunit alpha [Harpegnathos saltator]		1,00E-92
gALL_v3_c20057	2	10	gnl|CDD|185453	PTZ00110, PTZ00110, helicase	gb|ADI82014.1|	dead box polypeptide 5 [Heliconius heurippa] gb|ADI82015.1| dead box polypeptide 5 [Heliconius heurippa] gb|ADI82016.1| dead box polypeptide 5 [Heliconius heurippa		9,00E-63
gALL_v3_c20058	0	5	-	-	-	-	-	
gALL_v3_c20059	2	2	-	-	-	-	-	
gALL_v3_c2006	9	16	gnl|CDD|188717	cd09331, LIM1_PINCH, The fir	gb|EFN69483.1|	LIM and senescent cell antigen-like-containing domain protein 2 [Camponotus floridanus]		0
gALL_v3_c20060	3	2	-	-	ref|XP_003490773.1|	PREDICTED: myotubularin-related protein 5-like isoform 1 [Bombus impatiens] ref|XP_003490774.1| PREDICTED: myotubularin-related protein 5-like isoform 2 [Bombus ir		1,00E-116
gALL_v3_c20061	3	1	-	-	ref|XP_971938.1|	PREDICTED: similar to CG8793 CG8793-PA [Tribolium castaneum] gb|EFA11313.1| hypothetical protein TcasGA2_TC010852 [Tribolium castaneum]		9,00E-07
gALL_v3_c20062	1	3	-	-	-	-	-	
gALL_v3_c20064	5	2	-	-	-	-	-	
gALL_v3_c20065	3	3	-	-	-	-	-	
gALL_v3_c20066	5	5	gnl|CDD|111366	pfam02460, Patched, Patched	ref|XP_006622680.1|	PREDICTED: patched domain-containing protein 3-like isoform X3 [Apis dorsata]		3,00E-69
gALL_v3_c20068	1	4	-	-	-	-	-	
gALL_v3_c20069	2	4	-	-	-	-	-	
gALL_v3_c2007	3	4	-	-	gb|EKC26122.1|	hypothetical protein CGI_10010255 [Crassostrea gigas]		5,00E-11
gALL_v3_c20070	0	3	-	-	-	-	-	
gALL_v3_c20071	0	3	-	-	-	-	-	
gALL_v3_c20072	6	8	-	-	-	-	-	
gALL_v3_c20073	1	3	-	-	-	-	-	
gALL_v3_c20074	2	1	-	-	-	-	-	
gALL_v3_c20075	1	5	-	-	ref|XP_003249427.1|	PREDICTED: uncharacterized protein LOC100576289 [Apis mellifera]		3,00E-20
gALL_v3_c20076	0	4	gnl|CDD|189372	pfam00067, p450, Cytochrom	sp|P29981.1|CP4C1_BL	RecName: Full=Cytochrome P450 4C1; AltName: Full=CYPIVC1 gb|AAA27819.1| cytochrome P450 [Blaberus discoidalis]		1,00E-37
gALL_v3_c20077	3	1	-	-	-	-	-	
gALL_v3_c20078	5	2	-	-	-	-	-	
gALL_v3_c20079	2	3	-	-	-	-	-	
gALL_v3_c2008	7	4	-	-	-	-	-	
gALL_v3_c20080	2	4	-	-	-	-	-	
gALL_v3_c20081	3	1	-	-	-	-	-	
gALL_v3_c20082	3	6	-	-	-	-	-	
gALL_v3_c20084	3	2	-	-	gb|EFX67109.1|	hypothetical protein DAPPUDRAFT_64161 [Daphnia pulex]		4,00E-49
gALL_v3_c20086	1	4	-	-	-	-	-	
gALL_v3_c20087	2	1	-	-	-	-	-	
gALL_v3_c20088	2	5	-	-	-	-	-	
gALL_v3_c20089	6	5	-	-	-	-	-	
gALL_v3_c2009	6	3	gnl|CDD|128405	smart00094, TR_FER, Transfer	gb|ENN73682.1|	hypothetical protein YQE_09712, partial [Dendroctonus ponderosae]		4,00E-65
gALL_v3_c20090	3	6	gnl|CDD|189372	pfam00067, p450, Cytochrom	gb|ABX64399.1|	cytochrome P450 v2 [Liposcelis bostrychophila]		4,00E-70
gALL_v3_c20091	2	4	-	-	-	-	-	
gALL_v3_c20092	0	4	-	-	-	-	-	
gALL_v3_c20093	3	1	-	-	-	-	-	
gALL_v3_c20094	3	6	-	-	ref|XP_001658316.1|	myosin vii [Aedes aegypti] sp|Q17LW0.1|MYO7A_AEDAE RecName: Full=Myosin-VIIa; AltName: Full=Protein crinkled gb|EAT47711.1| AAEL001220-PA [Aedes aegypti]		9,00E-52
gALL_v3_c20095	7	10	-	-	-	-	-	
gALL_v3_c20096	2	3	-	-	-	-	-	
gALL_v3_c20098	0	4	-	-	-	-	-	
gALL_v3_c20099	5	4	gnl|CDD|116500	pfam07888, CALCOCO1, Calciu	gb|ELU03518.1|	hypothetical protein CAPTEDRAFT_201007 [Capitella teleta]		5,00E-29
gALL_v3_c201	14	25	gnl|CDD|181665	PRK09130, PRK09130, NADH c	ref|XP_973797.1|	PREDICTED: similar to NADH-ubiquinone oxidoreductase 75 kDa subunit, mitochondrial precursor (Complex I-75kD) (CI-75kD) [Tribolium castaneum] gb|EFA09221.1| hypo		0
gALL_v3_c2010	13	17	-	-	ref|XP_001602535.2|	PREDICTED: hypothetical protein LOC100118603 [Nasonia vitripennis]		2,00E-68
gALL_v3_c20100	10	8	-	-	-	-	-	
gALL_v3_c20101	18	17	-	-	-	-	-	
gALL_v3_c20102	0	5	-	-	-	-	-	
gALL_v3_c20103	1	3	-	-	-	-	-	
gALL_v3_c20105	5	3	gnl|CDD|33749	COG3968, COG3968, Uncharac	ref|WP_022766385.1|	glutamine synthetase [Butyrivibrio sp. XPD2006]		1,00E-98
gALL_v3_c20107	2	4	-	-	-	-	-	
gALL_v3_c20108	2	5	gnl|CDD|100015	cd02186, alpha_tubulin, The t	gb|AAM44818.1|AF508	alpha-tubulin [Dreissena polymorpha]		1,00E-11
gALL_v3_c20109	3	4	-	-	-	-	-	
gALL_v3_c2011	13	14	gnl|CDD|145371	pfam02181, FH2, Formin Hom	ref|XP_001948092.2|	PREDICTED: hypothetical protein LOC100159987 [Acyrthosiphon pisum]		1,00E-140
gALL_v3_c20110	1	3	-	-	-	-	-	
gALL_v3_c20111	1	4	-	-	ref|XP_972197.1|	PREDICTED: similar to CG32479 CG32479-PA [Tribolium castaneum] gb|EFA01234.1| ubiquitin-specific protease [Tribolium castaneum]		4,00E-48
gALL_v3_c20112	2	6	-	-	-	-	-	
gALL_v3_c20113	2	2	-	-	ref|XP_003490644.1|	PREDICTED: SH2B adapter protein 1-like [Bombus impatiens]		6,00E-10
gALL_v3_c20114	2	2	-	-	-	-	-	
gALL_v3_c20116	2	2	-	-	-	-	-	
gALL_v3_c20117	4	7	gnl|CDD|143541	cd07167, NR_DBD_Lrh-1_like,	ref|XP_002427418.1|	Ecdysone receptor, putative [Pediculus humanus corporis] gb|EEB14680.1| Ecdysone receptor, putative [Pediculus humanus corporis]		6,00E-80
gALL_v3_c20118	0	4	-	-	-	-	-	
gALL_v3_c20119	0	6	-	-	ref|XP_003701933.1|	PREDICTED: zinc finger protein 676-like [Megachile rotundata]		2,00E-56
gALL_v3_c2012	22	23	gnl|CDD|187547	cd05236, FAR-N_SDR_e, fatty	gb|AGM32690.1|	fatty acyl-coA reductase like protein [Coptotermes formosanus]		1,00E-124
gALL_v3_c20120	3	2	-	-	ref|XP_002432905.1|	hypothetical protein Phum_PHUM604480 [Pediculus humanus corporis] gb|EEB20167.1| hypothetical protein Phum_PHUM604480 [Pediculus humanus corporis]		2,00E-35
gALL_v3_c20121	14	10	-	-	gb|AAP33157.1|	beta-NAC-like protein [Reticulitermes flavipes]		2,00E-75
gALL_v3_c20122	0	4	-	-	-	-	-	
gALL_v3_c20123	1	4	-	-	-	-	-	
gALL_v3_c20124	2	1	-	-	-	-	-	
gALL_v3_c20125	3	1	-	-	-	-	-	
gALL_v3_c20126	3	7	gnl|CDD|191079	pfam04757, Pex2_Pex12, Pex2	ref|XP_001601571.1|	PREDICTED: peroxisome assembly protein 12-like [Nasonia vitripennis]		1,00E-30
gALL_v3_c20127	6	3	-	-	-	-	-	
gALL_v3_c20128	0	6	-	-	-	-	-	
gALL_v3_c20129	1	4	-	-	-	-	-	
gALL_v3_c20131	1	3	-	-	-	-	-	
gALL_v3_c20132	4	4	-	-	ref|XP_003396035.1|	PREDICTED: hypothetical protein LOC100648747 [Bombus terrestris]		7,00E-14
gALL_v3_c20133	3	1	-	-	ref|XP_001664271.1|	hypothetical protein AaeL_AAEL003882 [Aedes aegypti] gb|EAT44784.1| AAEL003882-PA [Aedes aegypti]		2,00E-13
gALL_v3_c20134	1	7	-	-	-	-	-	
gALL_v3_c20135	3	2	-	-	-	-	-	
gALL_v3_c20137	2	2	-	-	-	-	-	
gALL_v3_c20138	9	7	-	-	gb|ACT53739.1|	feruloyl esterase-like protein Est4 [Reticulitermes flavipes]		1,00E-06
gALL_v3_c20139	4	3	-	-	-	-	-	
gALL_v3_c2014	7	9	gnl|CDD|88620	cd05157, ETNK_euk, Ethanola	ref|XP_001660334.1|	choline/ethanolamine kinase [Aedes aegypti] gb|EAT38330.1| AAEL009765-PA [Aedes aegypti]		1,00E-100
gALL_v3_c20140	3	6	gnl|CDD|147605	pfam05510, Sarcoglycan_2, Sa	ref|XP_002429920.1|	Epsilon-sarcoglycan precursor, putative [Pediculus humanus corporis] gb|EEB17182.1| Epsilon-sarcoglycan precursor, putative [Pediculus humanus corporis]		1,00E-61
gALL_v3_c20141	3	1	-	-	-	-	-	
gALL_v3_c20143	4	8	gnl|CDD|185609	PTZ00424, PTZ00424, helicase	gb|ETN64218.1|	DEAD box ATP-dependent RNA helicase [Anopheles darlingi]		0
gALL_v3_c20145	3	5	-	-	-	-	-	
gALL_v3_c20146	3	1	-	-	gb|ELU02007.1|	hypothetical protein CAPTEDRAFT_192879 [Capitella teleta]		2,00E-14
gALL_v3_c20147	8	9	gnl|CDD|128409	smart00098, alkPPc, Alkaline p	gb|ACI00855.1|	alkaline phosphatase [Pteromalus puparum]		2,00E-20
gALL_v3_c20149	2	3	-	-	-	-	-	
gALL_v3_c20150	0	4	-	-	-	-	-	
gALL_v3_c20151	3	7	-	-	-	-	-	
gALL_v3_c20152	4	5	-	-	-	-	-	
gALL_v3_c20153	2	3	-	-	-	-	-	
gALL_v3_c20154	4	3	-	-	ref|XP_003707685.1|	PREDICTED: ubiquitin carboxyl-terminal hydrolase calypso-like [Megachile rotundata]		9,00E-55
gALL_v3_c20155	4	5	-	-	-	-	-	
gALL_v3_c20156	4	3	gnl|CDD|133270	cd01867, Rab8_Rab10_Rab13	ref|XP_001654277.1|	ras-related protein Rab-8A, putative [Aedes aegypti] ref|XP_001654278.1| ras-related protein Rab-8A, putative [Aedes aegypti] ref|XP_001654279.1| ras-related protein R		8,00E-33
gALL_v3_c20157	2	3	gnl|CDD|189653	pfam00650, CRAL_TRIO, CRAL	ref|XP_001653647.1|	hypothetical protein AaeL_AAEL009032 [Aedes aegypti] gb|EAT39147.1| AAEL009032-PA [Aedes aegypti]		1,00E-23
gALL_v3_c20159	5	2	gnl|CDD|128425	smart00115, CASc, Caspase, ir	ref|XP_003243774.1|	PREDICTED: caspase-1-like isoform 2 [Acyrthosiphon pisum] ref|XP_001950891.2| PREDICTED: caspase-1-like isoform 1 [Acyrthosiphon pisum]		4,00E-53
gALL_v3_c2016	11	6	gnl|CDD|173742	cd07845, STKc_CDK10, Catalyt	ref|XP_392973.4|	PREDICTED: cyclin-dependent kinase 10 [Apis mellifera] ref|XP_006616152.1| PREDICTED: cyclin-dependent kinase 10-like [Apis dorsata]		1,00E-153

gALL_v3_c20160	3	1	-	-	-	-	-
gALL_v3_c20161	1	5	gnl|CDD|177966	PLN02332, PLN02332, membr	ref|XP_003424410.1|	PREDICTED: membrane-bound O-acyltransferase domain-containing protein 2-like [Nasonia vitripennis]	6,00E-63
gALL_v3_c20162	2	2	gnl|CDD|128452	smart00147, RasGEF, Guanine	gb|ENN75506.1|	hypothetical protein YQE_08055, partial [Dendroctonus ponderosae]	2,00E-83
gALL_v3_c20163	4	3	-	-	-	-	-
gALL_v3_c20164	0	6	-	-	-	-	-
gALL_v3_c20165	2	3	-	-	ref|XP_002425831.1|	hypothetical protein Phum_PHUM221870 [Pediculus humanus corporis] gb|EEB13093.1| hypothetical protein Phum_PHUM221870 [Pediculus humanus corporis]	2,00E-18
gALL_v3_c20167	3	3	-	-	-	-	-
gALL_v3_c20168	1	5	-	-	-	-	-
gALL_v3_c2017	7	15	-	-	-	-	-
gALL_v3_c20173	2	4	-	-	-	-	-
gALL_v3_c20174	1	3	-	-	-	-	-
gALL_v3_c20175	4	6	-	-	-	-	-
gALL_v3_c20176	1	7	gnl|CDD|176326	cd01250, PH_centaurin, Centa	ref|XP_001951726.2|	PREDICTED: centaurin-gamma-1A-like [Acyrthosiphon pisum]	7,00E-50
gALL_v3_c20177	0	6	gnl|CDD|29138	cd00176, SPEC, Spectrin repea	ref|XP_968509.2|	PREDICTED: similar to AGAP006107-PA [Tribolium castaneum]	7,00E-97
gALL_v3_c20178	1	3	-	-	-	-	-
gALL_v3_c2018	15	16	-	-	ref|XP_002424429.1|	conserved hypothetical protein [Pediculus humanus corporis] gb|EEB11691.1| conserved hypothetical protein [Pediculus humanus corporis]	1,00E-106
gALL_v3_c20180	2	6	-	-	-	-	-
gALL_v3_c20181	15	16	-	-	-	-	-
gALL_v3_c20182	2	1	-	-	-	-	-
gALL_v3_c20183	2	2	-	-	-	-	-
gALL_v3_c20184	4	3	-	-	-	-	-
gALL_v3_c20185	1	4	-	-	-	-	-
gALL_v3_c20186	5	2	-	-	-	-	-
gALL_v3_c20188	1	3	gnl|CDD|128944	smart00704, ZnF_CDGSH, CDC	gb|EFZ17376.1|	hypothetical protein SINV_15357 [Solenopsis invicta]	4,00E-54
gALL_v3_c20189	0	6	gnl|CDD|173456	PTZ00169, PTZ00169, ADP/ATI	gb|AGM32826.1|	ADP/ATP carrier protein 2, partial [Coptotermes formosanus]	1,00E-14
gALL_v3_c2019	34	38	-	-	-	-	-
gALL_v3_c20190	5	8	-	-	ref|NP_001191877.1|	uncharacterized protein LOC100163446 [Acyrthosiphon pisum]	1,00E-25
gALL_v3_c20191	3	6	-	-	-	-	-
gALL_v3_c20192	2	2	-	-	-	-	-
gALL_v3_c20193	3	1	gnl|CDD|130780	TIGR01719, euk_UDPppase, u	ref|XP_968417.2|	PREDICTED: similar to CG6330 CG6330-PB [Tribolium castaneum]	3,00E-52
gALL_v3_c20194	2	4	-	-	ref|XP_004205527.1|	PREDICTED: RNA-directed DNA polymerase from mobile element jockey-like [Hydra vulgaris]	1,00E-16
gALL_v3_c20195	3	1	-	-	-	-	-
gALL_v3_c20196	3	5	-	-	-	-	-
gALL_v3_c20198	3	1	gnl|CDD|118719	pfam10191, COG7, Golgi comp	ref|XP_003691700.1|	PREDICTED: conserved oligomeric Golgi complex subunit 7-like [Apis florea]	4,00E-37
gALL_v3_c20199	3	1	-	-	-	-	-
gALL_v3_c2020	20	28	gnl|CDD|133270	cd01867, Rab8_Rab10_Rab13	gb|EGI71055.1|	Ras-related protein Rab-10 [Acromyrmex echinatior]	1,00E-112
gALL_v3_c20200	1	7	gnl|CDD|192996	pfam12348, CLASP_N, CLASP I	gb|EFX67958.1|	hypothetical protein DAPPUDRAFT_330564 [Daphnia pulex]	6,00E-53
gALL_v3_c20201	2	3	-	-	-	-	-
gALL_v3_c20202	0	4	-	-	gb|ESO07079.1|	hypothetical protein HELRODRAFT_184661 [Helobdella robusta]	1,00E-15
gALL_v3_c20203	3	2	gnl|CDD|185697	cd09020, D-hex-6-P-epi_like, D	ref|XP_003251860.1|	PREDICTED: putative glucose-6-phosphate 1-epimerase-like [Apis mellifera]	1,00E-119
gALL_v3_c20204	2	3	-	-	-	-	-
gALL_v3_c20205	5	3	-	-	-	-	-
gALL_v3_c20207	2	3	gnl|CDD|119423	cd05163, TRRAP, TRansformat	ref|XP_004929365.1|	PREDICTED: transformation/transcription domain-associated protein-like [Bombyx mori]	4,00E-49
gALL_v3_c20208	4	6	-	-	-	-	-
gALL_v3_c20209	2	5	-	-	-	-	-
gALL_v3_c20210	0	5	-	-	ref|XP_002425013.1|	hypothetical protein Phum_PHUM168470 [Pediculus humanus corporis] gb|EEB12275.1| hypothetical protein Phum_PHUM168470 [Pediculus humanus corporis]	2,00E-23
gALL_v3_c20211	2	2	-	-	-	-	-
gALL_v3_c20212	4	8	-	-	-	-	-
gALL_v3_c20213	1	3	-	-	-	-	-
gALL_v3_c20214	4	5	-	-	ref|XP_004213114.1|	PREDICTED: RNA-directed DNA polymerase from mobile element jockey-like, partial [Hydra vulgaris]	8,00E-06
gALL_v3_c20215	3	1	gnl|CDD|99996	cd04300, GT1_Glycogen_Phos	gb|AAO86575.1|	glycogen phosphorylase [Tritrichomonas foetus]	3,00E-72
gALL_v3_c20216	1	5	-	-	-	-	-
gALL_v3_c20218	3	5	-	-	gb|EGI65165.1|	Protein purity of essence [Acromyrmex echinatior]	1,00E-104
gALL_v3_c20219	3	4	-	-	-	-	-
gALL_v3_c20221	5	2	-	-	ref|XP_005469027.1|	PREDICTED: uncharacterized protein LOC102076147 [Oreochromis niloticus]	2,00E-29
gALL_v3_c20222	2	2	gnl|CDD|29257	cd00200, WD40, WD40 domai	ref|XP_003749482.2|	PREDICTED: WD repeat-containing protein 5-like [Rattus norvegicus]	3,00E-50
gALL_v3_c20223	4	3	-	-	-	-	-
gALL_v3_c20224	3	5	-	-	-	-	-
gALL_v3_c20225	1	2	-	-	-	-	-
gALL_v3_c20226	2	8	-	-	-	-	-
gALL_v3_c2023	5	4	-	-	-	-	-
gALL_v3_c20230	4	3	gnl|CDD|189008	cd09601, M1_APN_2, Peptida	ref|XP_001658230.1|	glutamyl aminopeptidase [Aedes aegypti] gb|EAT41121.1| AAEL007201-PA, partial [Aedes aegypti]	9,00E-42
gALL_v3_c20231	1	2	gnl|CDD|191185	pfam05076, SUFU, Suppressor	gb|ERL85988.1|	hypothetical protein D910_03402 [Dendroctonus ponderosae]	2,00E-77
gALL_v3_c20232	0	5	-	-	-	-	-
gALL_v3_c20233	1	4	-	-	gb|AAM11327.1|	GH01626p [Drosophila melanogaster]	4,00E-22
gALL_v3_c20234	0	4	-	-	-	-	-
gALL_v3_c20235	3	7	-	-	-	-	-
gALL_v3_c20237	1	3	gnl|CDD|143441	cd07123, ALDH_F4-17_P5CDH	ref|XP_969408.1|	PREDICTED: similar to pyrroline-5-carboxylate dehydrogenase [Tribolium castaneum] gb|EFA05239.1| hypothetical protein TcasGA2_TC015385 [Tribolium castaneum]	8,00E-31
gALL_v3_c20238	1	5	-	-	-	-	-
gALL_v3_c20239	1	6	-	-	-	-	-
gALL_v3_c2024	18	18	gnl|CDD|114307	pfam05577, Peptidase_S28, Se	gb|EFZ12594.1|	hypothetical protein SINV_07473 [Solenopsis invicta]	0
gALL_v3_c20240	1	6	-	-	-	-	-
gALL_v3_c20241	2	3	-	-	-	-	-
gALL_v3_c20242	3	1	-	-	-	-	-
gALL_v3_c20243	1	8	-	-	gb|EFA08347.1|	hypothetical protein TcasGA2_TC005990 [Tribolium castaneum]	9,00E-55
gALL_v3_c20244	1	4	-	-	-	-	-
gALL_v3_c20246	1	4	-	-	-	-	-
gALL_v3_c20247	3	2	gnl|CDD|191576	pfam06658, DUF1168, Protein	gb|ERL91388.1|	hypothetical protein D910_08720 [Dendroctonus ponderosae]	1,00E-23
gALL_v3_c20248	0	5	-	-	-	-	-
gALL_v3_c20249	2	1	-	-	-	-	-
gALL_v3_c20250	2	4	-	-	-	-	-
gALL_v3_c20251	1	4	-	-	-	-	-
gALL_v3_c20252	6	6	gnl|CDD|189663	pfam00675, Peptidase_M16, I	gb|EFN84900.1|	Mitochondrial-processing peptidase subunit beta [Harpegnathos saltator]	1,00E-108
gALL_v3_c20253	0	6	-	-	ref|XP_003244198.1|	PREDICTED: hypothetical protein LOC100573332 [Acyrthosiphon pisum]	1,00E-88
gALL_v3_c20254	2	2	-	-	-	-	-
gALL_v3_c20255	0	3	-	-	-	-	-
gALL_v3_c20256	0	4	-	-	-	-	-
gALL_v3_c20257	0	4	-	-	-	-	-
gALL_v3_c20258	3	2	-	-	-	-	-
gALL_v3_c20259	3	1	gnl|CDD|191306	pfam05571, DUF766, Protein c	ref|XP_001601374.1|	PREDICTED: JNK1/MAPK8-associated membrane protein-like [Nasonia vitripennis]	1,00E-72
gALL_v3_c2026	2	8	-	-	-	-	-
gALL_v3_c20260	3	2	-	-	-	-	-
gALL_v3_c20261	0	3	-	-	-	-	-
gALL_v3_c20262	15	24	gnl|CDD|190726	pfam03723, Hemocyanin_C, H	gb|AHB39936.1|	phenoloxidase 2, partial [Coptotermes formosanus]	0
gALL_v3_c20263	2	1	-	-	ref|XP_973695.1|	PREDICTED: similar to conserved hypothetical protein [Tribolium castaneum] gb|EFA03493.1| hypothetical protein TcasGA2_TC013492 [Tribolium castaneum]	1,00E-10
gALL_v3_c20264	2	4	-	-	-	-	-
gALL_v3_c20265	3	3	-	-	-	-	-
gALL_v3_c20266	3	1	-	-	-	-	-
gALL_v3_c20267	1	5	-	-	-	-	-
gALL_v3_c20268	3	1	-	-	-	-	-
gALL_v3_c2027	9	5	-	-	-	-	-
gALL_v3_c20270	2	5	gnl|CDD|31763	COG1575, MenA, 1,4-dihydrox	dbj|BAN20770.1|	1,4-dihydroxy-2-naphthoate octaprenyltransferase [Riptortus pedestris]	1,00E-103
gALL_v3_c20271	4	5	gnl|CDD|29029	cd00127, DSPc, Dual specificity	ref|NP_651767.2|	CG15528 [Drosophila melanogaster] gb|ACD99532.1| IP21249p [Drosophila melanogaster] gb|AAF57003.2| CG15528 [Drosophila melanogaster]	2,00E-44
gALL_v3_c20272	2	4	-	-	-	-	-
gALL_v3_c20273	3	1	-	-	-	-	-
gALL_v3_c20274	2	7	-	-	-	-	-
gALL_v3_c20275	3	1	-	-	-	-	-
gALL_v3_c20276	2	4	-	-	-	-	-
gALL_v3_c20277	6	4	-	-	-	-	-
gALL_v3_c20279	2	3	gnl|CDD|31291	COG1094, COG1094, Predictec	ref|XP_003699413.1|	PREDICTED: KRR1 small subunit processome component homolog [Megachile rotundata]	1,00E-104
gALL_v3_c2028	13	21	gnl|CDD|163630	cd07387, MPP_PolD2_C, PolD:	ref|XP_001851385.1|	DNA polymerase delta small subunit [Culex quinquefasciatus] gb|EDS33455.1| DNA polymerase delta small subunit [Culex quinquefasciatus]	3,00E-94
gALL_v3_c20282	3	2	gnl|CDD|189712	pfam00773, RNB, RNB domain	gb|EFA08662.1|	hypothetical protein TcasGA2_TC006327 [Tribolium castaneum]	3,00E-57
gALL_v3_c20283	3	5	-	-	-	-	-
gALL_v3_c20284	6	9	-	-	ref|XP_003489881.1|	PREDICTED: dystrophin, isoforms A/C/F/G/H-like isoform 1 [Bombus impatiens]	9,00E-41
gALL_v3_c20285	3	3	-	-	ref|XP_004206212.1|	PREDICTED: RNA-directed DNA polymerase from mobile element jockey-like [Hydra vulgaris]	4,00E-17
gALL_v3_c20286	1	3	-	-	-	-	-
gALL_v3_c20287	1	4	-	-	-	-	-
gALL_v3_c2029	16	11	-	-	gb|AAP94045.1|	membrane alanyl aminopeptidase [Tenebrio molitor]	6,00E-17
gALL_v3_c20290	4	2	gnl|CDD|128516	smart00220, S_TKc, Serine/Th	ref|XP_002431071.1|	calcium/calmodulin-dependent protein kinase kinase, putative [Pediculus humanus corporis] gb|EEB18333.1| calcium/calmodulin-dependent protein kinase kinase, putati	7,00E-89
gALL_v3_c20291	2	8	-	-	ref|XP_001861320.1|	conserved hypothetical protein [Culex quinquefasciatus] gb|EDS35441.1| conserved hypothetical protein [Culex quinquefasciatus]	7,00E-38
gALL_v3_c20292	6	4	-	-	ref|XP_005062543.1|	PREDICTED: zinc finger protein 135-like [Ficedula albicollis]	2,00E-34
gALL_v3_c20293	1	3	gnl|CDD|128359	smart00044, CYCc, Adenylyl- /	gb|EFA04538.1|	hormone receptor 83 [Tribolium castaneum]	3,00E-59
gALL_v3_c20294	4	3	-	-	-	-	-
gALL_v3_c20295	2	2	-	-	-	-	-
gALL_v3_c20296	2	2	-	-	-	-	-
gALL_v3_c20297	0	3	-	-	ref|XP_002423360.1|	hypothetical protein Phum_PHUM052890 [Pediculus humanus corporis] gb|EEB10622.1| hypothetical protein Phum_PHUM052890 [Pediculus humanus corporis]	1,00E-48
gALL_v3_c20298	0	5	-	-	ref|XP_001952119.1|	PREDICTED: ATP-dependent RNA helicase abstrakt-like [Acyrthosiphon pisum]	5,00E-10
gALL_v3_c20299	1	2	gnl|CDD|176412	cd01817, RGS12_RBD, Ubiquit	ref|XP_002429741.1|	regulator of G protein signaling, putative [Pediculus humanus corporis] gb|EEB17003.1| regulator of G protein signaling, putative [Pediculus humanus corporis]	1,00E-37
gALL_v3_c203	19	23	-	-	-	-	-
gALL_v3_c20300	5	2	-	-	-	-	-
gALL_v3_c20301	2	3	gnl|CDD|112980	pfam04190, DUF410, Protein c	gb|EFN84411.1|	UPF0363 protein C7orf20 [Harpegnathos saltator]	1,00E-70
gALL_v3_c20302	2	3	-	-	-	-	-
gALL_v3_c20303	0	5	-	-	gb|EFN67634.1|	Putative uncharacterized transposon-derived protein F52C9.6 [Camponotus floridanus]	4,00E-06
gALL_v3_c20304	2	3	-	-	-	-	-
gALL_v3_c20305	3	5	-	-	gb|ERL90205.1|	hypothetical protein D910_07559 [Dendroctonus ponderosae]	3,00E-48
gALL_v3_c20306	1	4	gnl|CDD|35119	COG5560, UBP12, Ubiquitin C-	ref|XP_002427486.1|	ubiquitin carboxyl-terminal hydrolase, putative [Pediculus humanus corporis] gb|EEB14748.1| ubiquitin carboxyl-terminal hydrolase, putative [Pediculus humanus corporis	1,00E-62
gALL_v3_c20307	0	5	-	-	-	-	-
gALL_v3_c20308	1	4	gnl|CDD|29257	cd00200, WD40, WD40 domai	ref|XP_967396.1|	PREDICTED: similar to pre-mRNA-processing factor 17 [Tribolium castaneum] gb|EEZ97476.1| hypothetical protein TcasGA2_TC011310 [Tribolium castaneum]	2,00E-58
gALL_v3_c20309	0	5	-	-	-	-	-
gALL_v3_c2031	4	6	-	-	-	-	-
gALL_v3_c20313	5	3	gnl|CDD|129701	TIGR00614, recQ_fam, ATP-de	ref|XP_002431597.1|	ATP-dependent DNA helicase Q1, putative [Pediculus humanus corporis] gb|EEB18859.1| ATP-dependent DNA helicase Q1, putative [Pediculus humanus corporis]	2,00E-92
gALL_v3_c20315	1	4	gnl|CDD|28913	cd00031, CA, Cadherin repeat	ref|XP_001600457.2|	PREDICTED: fat-like cadherin-related tumor suppressor homolog [Nasonia vitripennis]	1,00E-105
gALL_v3_c20316	2	7	-	-	gb|EEZ99816.1|	hypothetical protein TcasGA2_TC002596 [Tribolium castaneum]	3,00E-12
gALL_v3_c20317	3	4	-	-	-	-	-

gALL_v3_c20319	1	2	gnl|CDD|30100	cd01378, MYSc_type_I, Myosi	ref|XP_005176766.1|	PREDICTED: myosin-IB-like isoform X1 [Musca domestica]	1,00E-28
gALL_v3_c2032	4	4	-	-	gb|EJY57408.1|	AAEL017571-PA [Aedes aegypti]	1,00E-07
gALL_v3_c20321	1	3	-	-	-	-	-
gALL_v3_c20322	2	3	gnl|CDD|29049	cd00992, PDZ_signaling, PDZ c	ref|XP_001657836.1|	par-6 gamma [Aedes aegypti] gb|EAT41948.1| AAEL006460-PA [Aedes aegypti]	1,00E-111
gALL_v3_c20323	0	4	gnl|CDD|100087	cd03085, PGM1, Phosphogluc	ref|XP_004525421.1|	PREDICTED: phosphoglucomutase-like [Ceratitis capitata]	1,00E-57
gALL_v3_c20324	10	11	-	-	-	-	-
gALL_v3_c20325	4	3	-	-	gb|ETN59824.1|	baculoviral IAP repeat-containing protein 6 (apollon) [Anopheles darlingi]	2,00E-42
gALL_v3_c20326	2	1	-	-	ref|XP_002424197.1|	NMDA receptor-regulated protein, putative [Pediculus humanus corporis] gb|EEB11459.1| NMDA receptor-regulated protein, putative [Pediculus humanus corporis]	6,00E-75
gALL_v3_c20327	3	1	gnl|CDD|147520	pfam05380, Peptidase_A17, P	ref|XP_004537675.1|	PREDICTED: uncharacterized protein LOC101463033, partial [Ceratitis capitata]	3,00E-22
gALL_v3_c20328	7	12	-	-	ref|XP_003727690.1|	PREDICTED: RNA-directed DNA polymerase from mobile element jockey-like [Strongylocentrotus purpuratus]	8,00E-06
gALL_v3_c20329	1	4	-	-	-	-	-
gALL_v3_c2033	5	5	gnl|CDD|29138	cd00176, SPEC, Spectrin repea	ref|XP_003703682.1|	PREDICTED: spectrin beta chain, brain 4-like [Megachile rotundata]	1,00E-111
gALL_v3_c20330	1	3	-	-	-	-	-
gALL_v3_c20331	0	6	-	-	-	-	-
gALL_v3_c20332	3	1	-	-	ref|WP_004976427.1|	hypothetical protein, partial [Acinetobacter towneri] gb|ENV71128.1| hypothetical protein F947_00014, partial [Acinetobacter towneri DSM 14962 = CIP 107472]	5,00E-88
gALL_v3_c20334	2	7	-	-	-	-	-
gALL_v3_c20335	0	6	-	-	-	-	-
gALL_v3_c20336	3	1	-	-	-	-	-
gALL_v3_c20337	2	2	-	-	-	-	-
gALL_v3_c20338	3	1	-	-	-	-	-
gALL_v3_c20339	1	4	-	-	-	-	-
gALL_v3_c2034	6	4	-	-	-	-	-
gALL_v3_c20340	2	4	-	-	ref|XP_003246767.1|	PREDICTED: integrin alpha-PS1-like isoform 3 [Acyrthosiphon pisum]	4,00E-36
gALL_v3_c20341	3	3	-	-	-	-	-
gALL_v3_c20342	39	28	-	-	-	-	-
gALL_v3_c20343	2	2	-	-	-	-	-
gALL_v3_c20344	1	4	-	-	ref|XP_003399273.1|	PREDICTED: hypothetical protein LOC100649779 [Bombus terrestris]	2,00E-18
gALL_v3_c20345	2	2	-	-	-	-	-
gALL_v3_c20346	4	7	-	-	-	-	-
gALL_v3_c20347	0	3	-	-	-	-	-
gALL_v3_c20348	2	2	-	-	-	-	-
gALL_v3_c2035	7	15	-	-	ref|XP_001944872.2|	PREDICTED: c-myc promoter-binding protein-like isoform 1 [Acyrthosiphon pisum]	4,00E-59
gALL_v3_c20350	2	3	-	-	-	-	-
gALL_v3_c20351	2	4	-	-	-	-	-
gALL_v3_c20352	1	3	-	-	-	-	-
gALL_v3_c20353	4	3	-	-	-	-	-
gALL_v3_c20354	1	5	gnl|CDD|178745	PLN03206, PLN03206, phosph	ref|XP_003704098.1|	PREDICTED: phosphoribosylformylglycinamidine synthase-like [Megachile rotundata]	1,00E-78
gALL_v3_c20355	2	7	gnl|CDD|128578	smart00282, LamG, Laminin G	ref|XP_002431590.1|	laminin A chain, putative [Pediculus humanus corporis] gb|EEB18852.1| laminin A chain, putative [Pediculus humanus corporis]	8,00E-30
gALL_v3_c20356	18	18	gnl|CDD|176359	cd01763, Sumo, Small ubiquiti	gb|AGM32544.1|	small ubiquitin-related modifier-like protein [Coptotermes formosanus]	3,00E-62
gALL_v3_c20357	3	4	-	-	-	-	-
gALL_v3_c20358	3	2	-	-	-	-	-
gALL_v3_c20359	4	4	-	-	-	-	-
gALL_v3_c2036	6	7	gnl|CDD|192091	pfam08610, Pex16, Peroxisom	ref|XP_970655.1|	PREDICTED: similar to peroxisomal membrane protein PEX16 [Tribolium castaneum] gb|EFA02007.1| hypothetical protein TcasGA2_TC007629 [Tribolium castaneum]	4,00E-45
gALL_v3_c20360	3	3	gnl|CDD|115145	pfam06469, DUF1088, Domain	ref|XP_006613157.1|	PREDICTED: neurobeachin-like [Apis dorsata]	2,00E-96
gALL_v3_c20361	4	5	-	-	-	-	-
gALL_v3_c20362	1	4	-	-	-	-	-
gALL_v3_c20363	2	4	-	-	ref|XP_003976713.1|	PREDICTED: E3 ubiquitin-protein ligase RNF170-like [Takifugu rubripes]	3,00E-20
gALL_v3_c20364	1	3	-	-	ref|XP_003701810.1|	PREDICTED: probable E3 ubiquitin-protein ligase HERC4-like [Megachile rotundata]	3,00E-12
gALL_v3_c20365	1	5	-	-	-	-	-
gALL_v3_c20366	4	2	-	-	gb|AGM32381.1|	C2 domain-containing protein [Coptotermes formosanus]	7,00E-74
gALL_v3_c20367	2	2	-	-	-	-	-
gALL_v3_c20368	3	5	-	-	gb|ELU02007.1|	hypothetical protein CAPTEDRAFT_192879 [Capitella teleta]	1,00E-11
gALL_v3_c20369	12	13	-	-	-	-	-
gALL_v3_c2037	8	18	-	-	ref|WP_005554450.1|	hypothetical protein [Natronococcus amylolyticus] gb|ELY59844.1| hypothetical protein C491_05731 [Natronococcus amylolyticus DSM 10524]	6,00E-09
gALL_v3_c20370	0	5	-	-	-	-	-
gALL_v3_c20371	1	3	-	-	-	-	-
gALL_v3_c20372	1	3	-	-	ref|XP_001186691.1|	PREDICTED: uncharacterized protein LOC754693 [Strongylocentrotus purpuratus]	5,00E-08
gALL_v3_c20374	1	3	-	-	ref|XP_002428142.1|	conserved hypothetical protein [Pediculus humanus corporis] gb|EEB15404.1| conserved hypothetical protein [Pediculus humanus corporis]	7,00E-12
gALL_v3_c20375	3	1	-	-	-	-	-
gALL_v3_c20376	2	2	-	-	-	-	-
gALL_v3_c20377	1	3	-	-	-	-	-
gALL_v3_c20379	0	4	-	-	-	-	-
gALL_v3_c2038	6	3	gnl|CDD|129972	TIGR00894, 2A0114euk, Na(+)	ref|XP_001607837.2|	PREDICTED: putative inorganic phosphate cotransporter-like [Nasonia vitripennis]	3,00E-97
gALL_v3_c20381	2	3	-	-	-	-	-
gALL_v3_c20382	3	3	-	-	ref|XP_003488516.1|	PREDICTED: hypothetical protein LOC100742095 [Bombus impatiens]	4,00E-27
gALL_v3_c20383	1	2	-	-	ref|XP_973818.1|	PREDICTED: similar to ubiquitin conjugating enzyme E2, J2 [Tribolium castaneum] gb|EFA03490.1| hypothetical protein TcasGA2_TC013489 [Tribolium castaneum]	2,00E-13
gALL_v3_c20384	3	4	-	-	-	-	-
gALL_v3_c20385	2	3	-	-	-	-	-
gALL_v3_c20386	0	3	-	-	-	-	-
gALL_v3_c20387	10	13	gnl|CDD|29138	cd00176, SPEC, Spectrin repea	gb|ENN82209.1|	hypothetical protein YQE_01415, partial [Dendroctonus ponderosae]	1,00E-105
gALL_v3_c20388	2	1	-	-	-	-	-
gALL_v3_c20389	4	3	-	-	-	-	-
gALL_v3_c2039	12	15	-	-	-	-	-
gALL_v3_c20391	2	3	-	-	gb|EFN88242.1|	hypothetical protein EAI_08768 [Harpegnathos saltator]	1,00E-106
gALL_v3_c20392	4	2	-	-	-	-	-
gALL_v3_c20394	3	4	-	-	-	-	-
gALL_v3_c20396	2	6	-	-	-	-	-
gALL_v3_c20397	1	3	-	-	-	-	-
gALL_v3_c20398	2	2	-	-	-	-	-
gALL_v3_c20399	0	6	-	-	-	-	-
gALL_v3_c204	21	28	-	-	ref|XP_006132930.1|	PREDICTED: jerky protein-like [Pelodiscus sinensis]	1,00E-13
gALL_v3_c2040	15	10	gnl|CDD|128853	smart00584, TLDc, domain in	gb|EFN74789.1|	Oxidation resistance protein 1 [Camponotus floridanus]	3,00E-22
gALL_v3_c20402	6	5	-	-	-	-	-
gALL_v3_c20403	1	3	-	-	-	-	-
gALL_v3_c20404	3	2	-	-	-	-	-
gALL_v3_c20406	4	2	-	-	-	-	-
gALL_v3_c20407	2	2	-	-	gb|EFN75852.1|	Craniofacial development protein 2 [Harpegnathos saltator]	3,00E-19
gALL_v3_c20408	4	7	-	-	gb|EFN89286.1|	PHD finger protein rhinoceros [Harpegnathos saltator]	6,00E-28
gALL_v3_c20409	4	8	-	-	-	-	-
gALL_v3_c2041	3	5	-	-	-	-	-
gALL_v3_c20410	5	2	-	-	ref|XP_005464674.1|	PREDICTED: RNA-directed DNA polymerase from mobile element jockey-like [Oreochromis niloticus]	2,00E-06
gALL_v3_c20411	3	3	-	-	gb|AGM32632.1|	EF-hand domain containing protein [Coptotermes formosanus]	7,00E-72
gALL_v3_c20412	2	2	-	-	ref|XP_006571883.1|	PREDICTED: GTPase-activating protein and VPS9 domain-containing protein 1-like isoform X2 [Apis mellifera]	2,00E-15
gALL_v3_c20413	3	5	gnl|CDD|148917	pfam07565, Band_3_cyto, Bar	ref|XP_002432408.1|	Anion exchange protein, putative [Pediculus humanus corporis] gb|EEB19670.1| Anion exchange protein, putative [Pediculus humanus corporis]	1,00E-172
gALL_v3_c20414	3	1	gnl|CDD|189795	pfam00995, Sec1, Sec1 family.	ref|XP_003484862.1|	PREDICTED: vacuolar protein sorting-associated protein 45-like [Bombus impatiens]	2,00E-92
gALL_v3_c20415	3	7	-	-	gb|EFA11544.1|	kurtz [Tribolium castaneum]	1,00E-100
gALL_v3_c20416	0	5	-	-	-	-	-
gALL_v3_c20417	2	1	-	-	-	-	-
gALL_v3_c20418	0	3	-	-	-	-	-
gALL_v3_c20419	2	3	-	-	-	-	-
gALL_v3_c2042	6	10	gnl|CDD|128591	smart00295, B41, Band 4.1 ho	ref|XP_006569638.1|	PREDICTED: tyrosine-protein phosphatase non-receptor type 4 isoform X2 [Apis mellifera]	0
gALL_v3_c20420	3	2	-	-	-	-	-
gALL_v3_c20421	1	3	-	-	-	-	-
gALL_v3_c20422	0	3	-	-	-	-	-
gALL_v3_c20423	4	4	gnl|CDD|144379	pfam00755, Carn_acyltransf, C	ref|XP_001658439.1|	carnitine o-acyltransferase [Aedes aegypti] gb|EAT40751.1| AAEL007536-PA [Aedes aegypti]	2,00E-96
gALL_v3_c20424	9	6	-	-	gb|ACO11046.1|	Adenosine deaminase [Caligus rogercresseyi]	4,00E-08
gALL_v3_c20425	1	4	-	-	-	-	-
gALL_v3_c20426	1	4	-	-	-	-	-
gALL_v3_c20428	0	4	-	-	-	-	-
gALL_v3_c20429	1	2	-	-	gb|EFN75852.1|	Craniofacial development protein 2 [Harpegnathos saltator]	4,00E-39
gALL_v3_c20430	3	4	-	-	-	-	-
gALL_v3_c20431	2	2	-	-	gb|EFZ22135.1|	hypothetical protein SINV_09804 [Solenopsis invicta]	3,00E-25
gALL_v3_c20433	3	2	-	-	-	-	-
gALL_v3_c20434	3	4	-	-	-	-	-
gALL_v3_c20435	2	4	-	-	-	-	-
gALL_v3_c20436	3	1	-	-	-	-	-
gALL_v3_c20437	3	2	-	-	-	-	-
gALL_v3_c20438	5	2	-	-	gb|EHJ63900.1|	RNA m5u methyltransferase [Danaus plexippus]	2,00E-11
gALL_v3_c20439	3	2	-	-	-	-	-
gALL_v3_c2044	41	56	gnl|CDD|29012	cd00112, LDLa, Low Density Li	ref|XP_004529416.1|	PREDICTED: low-density lipoprotein receptor-related protein 2-like isoform X1 [Ceratitis capitata] ref|XP_004529417.1| PREDICTED: low-density lipoprotein receptor-relate	0
gALL_v3_c20440	2	2	-	-	-	-	-
gALL_v3_c20441	1	7	-	-	-	-	-
gALL_v3_c20442	1	5	-	-	-	-	-
gALL_v3_c20443	4	3	-	-	-	-	-
gALL_v3_c20444	3	1	-	-	-	-	-
gALL_v3_c20446	2	6	-	-	emb|CCT61274.1|	ecdysone-induced protein 93, partial [Blattella germanica]	1,00E-15
gALL_v3_c20447	2	4	-	-	-	-	-
gALL_v3_c20448	2	4	-	-	-	-	-
gALL_v3_c20449	2	1	-	-	ref|WP_006873254.1|	hypothetical protein [Anaerotruncus colihominis] gb|EDS10617.1| hypothetical protein ANACOL_02699 [Anaerotruncus colihominis DSM 17241]	7,00E-59
gALL_v3_c2045	5	9	-	-	-	-	-
gALL_v3_c20450	5	2	-	-	emb|CAQ16898.1|	large HD antigen [Hepatitis delta virus dFr2239b]	1,00E-06
gALL_v3_c20451	2	5	-	-	-	-	-
gALL_v3_c20453	2	3	-	-	-	-	-
gALL_v3_c20454	3	2	-	-	-	-	-
gALL_v3_c20455	1	2	-	-	-	-	-
gALL_v3_c20456	2	3	-	-	gb|EFN65619.1|	Putative uncharacterized transposon-derived protein F52C9.6 [Camponotus floridanus]	1,00E-16
gALL_v3_c20457	2	3	-	-	-	-	-
gALL_v3_c20459	1	5	-	-	-	-	-
gALL_v3_c2046	26	18	-	-	-	-	-
gALL_v3_c20460	2	2	-	-	-	-	-
gALL_v3_c20462	2	2	gnl|CDD|129972	TIGR00894, 2A0114euk, Na(+)	ref|XP_002427133.1|	sialin, putative [Pediculus humanus corporis] gb|EEB14395.1| sialin, putative [Pediculus humanus corporis]	6,00E-19
gALL_v3_c20463	2	2	gnl|CDD|150878	pfam10272, Tmpp129, Putativ	gb|EFZ17913.1|	hypothetical protein SINV_02541 [Solenopsis invicta]	6,00E-23
gALL_v3_c20464	4	5	-	-	-	-	-
gALL_v3_c20466	0	5	-	-	-	-	-

gALL_v3_c20467	3	2	-	-	-	-	-
gALL_v3_c20468	0	3	-	-	ref|XP_003245995.1|	PREDICTED: hypothetical protein LOC100570266 [Acyrthosiphon pisum]	3,00E-32
gALL_v3_c2047	5	12	gnl|CDD|178220	PLN02610, PLN02610, probabl	gb|ERL92272.1|	hypothetical protein D910_09589 [Dendroctonus ponderosae]	1,00E-133
gALL_v3_c20470	2	6	-	-	-	-	-
gALL_v3_c20471	3	3	-	-	-	-	-
gALL_v3_c20472	0	4	-	-	-	-	-
gALL_v3_c20473	0	3	-	-	-	-	-
gALL_v3_c20474	3	2	gnl|CDD|173727	cd06613, STKc_MAP4K3_like,	gb|ENN80831.1|	hypothetical protein YQE_02738, partial [Dendroctonus ponderosae] gb|ERL90516.1| hypothetical protein D910_07864 [Dendroctonus ponderosae]	1,00E-117
gALL_v3_c20475	1	3	-	-	-	-	-
gALL_v3_c20476	2	2	-	-	-	-	-
gALL_v3_c20477	2	4	-	-	-	-	-
gALL_v3_c20478	4	4	gnl|CDD|34912	COG5333, CCL1, Cdk activating	ref|XP_968481.1|	PREDICTED: similar to g1/s-specific cyclin c [Tribolium castaneum] gb|EEZ99604.1| hypothetical protein TcasGA2_TC002120 [Tribolium castaneum]	1,00E-118
gALL_v3_c20479	3	1	gnl|CDD|189341	pfam00010, HLH, Helix-loop-h	ref|XP_003699529.1|	PREDICTED: neurogenin-3-like [Megachile rotundata]	9,00E-26
gALL_v3_c2048	7	7	-	-	-	-	-
gALL_v3_c20480	3	2	-	-	gb|AGM32719.1|	hypothetical protein, partial [Coptotermes formosanus]	2,00E-97
gALL_v3_c20481	4	3	-	-	-	-	-
gALL_v3_c20482	3	1	-	-	-	-	-
gALL_v3_c20483	4	2	-	-	-	-	-
gALL_v3_c20484	0	3	-	-	-	-	-
gALL_v3_c20485	3	2	-	-	ref|XP_003707770.1|	PREDICTED: neprilysin-1-like [Megachile rotundata]	4,00E-19
gALL_v3_c20487	1	3	gnl|CDD|190181	pfam01980, UPF0066, Unchar	ref|XP_003703070.1|	PREDICTED: uncharacterized protein LOC100875268 [Megachile rotundata]	1,00E-63
gALL_v3_c20489	0	4	-	-	ref|XP_001187910.1|	PREDICTED: craniofacial development protein 2-like [Strongylocentrotus purpuratus]	3,00E-15
gALL_v3_c20490	6	7	-	-	-	-	-
gALL_v3_c20491	2	5	gnl|CDD|191014	pfam04515, Choline_transpo,	ref|XP_004926444.1|	PREDICTED: CTL-like protein 1-like isoform X1 [Bombyx mori]	1,00E-77
gALL_v3_c20492	1	1	gnl|CDD|178816	PRK00042, tpiA, triosephosph	gb|AGM32485.1|	triosephosphate isomerase [Coptotermes formosanus]	1,00E-58
gALL_v3_c20493	3	7	-	-	ref|XP_001808400.1|	PREDICTED: similar to GA14032-PA [Tribolium castaneum] gb|EEZ99814.1| hypothetical protein TcasGA2_TC002594 [Tribolium castaneum]	5,00E-30
gALL_v3_c20494	2	8	-	-	-	-	-
gALL_v3_c20495	1	4	-	-	-	-	-
gALL_v3_c20496	7	10	-	-	-	-	-
gALL_v3_c20497	2	5	gnl|CDD|34495	COG4886, COG4886, Leucine-r	ref|XP_967720.1|	PREDICTED: similar to leucine-rich transmembrane protein [Tribolium castaneum]	1,00E-131
gALL_v3_c20498	2	1	gnl|CDD|185648	PTZ00469, PTZ00469, 60S ribo	gb|EFZ15690.1|	hypothetical protein SINV_04174 [Solenopsis invicta]	8,00E-16
gALL_v3_c20499	0	4	-	-	-	-	-
gALL_v3_c2050	6	14	-	-	-	-	-
gALL_v3_c20500	4	3	-	-	-	-	-
gALL_v3_c20501	0	4	-	-	-	-	-
gALL_v3_c20502	0	4	-	-	-	-	-
gALL_v3_c20503	3	1	-	-	gb|EFN68811.1|	Tetratricopeptide repeat protein 7B [Camponotus floridanus]	2,00E-19
gALL_v3_c20505	1	5	-	-	-	-	-
gALL_v3_c20506	11	9	-	-	ref|XP_003402058.1|	PREDICTED: hypothetical protein LOC100645895 [Bombus terrestris]	1,00E-21
gALL_v3_c20508	4	5	-	-	gb|EHJ75078.1|	putative apolipoprotein D [Danaus plexippus]	2,00E-48
gALL_v3_c20509	1	6	-	-	ref|XP_002425295.1|	discs large protein, putative [Pediculus humanus corporis] gb|EEB12557.1| discs large protein, putative [Pediculus humanus corporis]	1,00E-126
gALL_v3_c2051	17	14	-	-	-	-	-
gALL_v3_c20510	3	3	-	-	-	-	-
gALL_v3_c20511	2	3	-	-	-	-	-
gALL_v3_c20514	4	3	-	-	-	-	-
gALL_v3_c20516	19	17	-	-	-	-	-
gALL_v3_c20517	0	5	gnl|CDD|128928	smart00685, DM14, Repeats ir	ref|XP_001649840.1|	hypothetical protein AaeL_AAEL004802 [Aedes aegypti] gb|EAT43762.1| AAEL004802-PB [Aedes aegypti]	2,00E-47
gALL_v3_c20518	2	1	-	-	ref|XP_002425133.1|	slingshot dual specificity phosphatase, putative [Pediculus humanus corporis] gb|EEB12395.1| slingshot dual specificity phosphatase, putative [Pediculus humanus corpori	1,00E-28
gALL_v3_c20519	1	3	-	-	-	-	-
gALL_v3_c2052	16	13	-	-	-	-	-
gALL_v3_c20520	4	2	-	-	-	-	-
gALL_v3_c20521	3	2	-	-	-	-	-
gALL_v3_c20522	1	4	-	-	-	-	-
gALL_v3_c20525	6	5	-	-	-	-	-
gALL_v3_c20526	1	5	-	-	-	-	-
gALL_v3_c20527	1	3	gnl|CDD|128538	smart00242, MYSc, Myosin. La	ref|XP_001944282.2|	PREDICTED: myosin-XV isoform 1 [Acyrthosiphon pisum]	1,00E-100
gALL_v3_c20528	1	4	-	-	-	-	-
gALL_v3_c20529	1	3	-	-	ref|XP_002425428.1|	conserved hypothetical protein [Pediculus humanus corporis] gb|EEB12690.1| conserved hypothetical protein [Pediculus humanus corporis]	8,00E-35
gALL_v3_c2053	26	19	gnl|CDD|176924	cd09071, FAR_C, C-terminal do	ref|XP_001602857.1|	PREDICTED: putative fatty acyl-CoA reductase CG5065-like [Nasonia vitripennis]	7,00E-17
gALL_v3_c20530	1	5	-	-	-	-	-
gALL_v3_c20532	1	3	-	-	-	-	-
gALL_v3_c20533	2	4	-	-	-	-	-
gALL_v3_c20534	2	4	gnl|CDD|192348	pfam09725, Fra10Ac1, Folate-	gb|AGM32325.1|	protein FRA10AC1-like protein, partial [Coptotermes formosanus]	4,00E-32
gALL_v3_c20535	1	5	-	-	ref|XP_003702696.1|	PREDICTED: Down syndrome cell adhesion molecule-like protein Dscam2-like [Megachile rotundata]	9,00E-47
gALL_v3_c20536	1	7	-	-	-	-	-
gALL_v3_c20538	2	2	-	-	-	-	-
gALL_v3_c20539	2	3	-	-	-	-	-
gALL_v3_c2054	4	10	-	-	-	-	-
gALL_v3_c20540	1	5	-	-	-	-	-
gALL_v3_c20541	2	3	-	-	-	-	-
gALL_v3_c20542	0	4	-	-	-	-	-
gALL_v3_c20544	4	2	gnl|CDD|149010	pfam07713, DUF1604, Protein	ref|XP_001984307.1|	GH16377 [Drosophila grimshawi] gb|EDV96655.1| GH16377 [Drosophila grimshawi]	1,00E-57
gALL_v3_c20545	2	5	-	-	-	-	-
gALL_v3_c20547	2	4	gnl|CDD|189372	pfam00067, p450, Cytochrom	gb|AGQ48062.1|	cytochrome P450 monooxygenase [Coptotermes formosanus]	3,00E-97
gALL_v3_c20548	3	2	-	-	-	-	-
gALL_v3_c20549	3	6	-	-	gb|EFN66847.1|	hypothetical protein EAG_08499 [Camponotus floridanus]	3,00E-11
gALL_v3_c2055	9	11	-	-	-	-	-
gALL_v3_c20550	2	7	gnl|CDD|173796	cd04857, Peptidases_S8_Tripe	gb|EFA11104.1|	hypothetical protein TcasGA2_TC004702 [Tribolium castaneum]	3,00E-97
gALL_v3_c20551	3	3	gnl|CDD|189424	pfam00153, Mito_carr, Mitoch	ref|XP_972579.1|	PREDICTED: similar to mitochondrial carrier protein [Tribolium castaneum]	3,00E-95
gALL_v3_c20552	8	11	-	-	-	-	-
gALL_v3_c20553	3	4	-	-	-	-	-
gALL_v3_c20554	3	5	-	-	ref|XP_002431890.1|	Regulator of G-protein signaling, putative [Pediculus humanus corporis] gb|EEB19152.1| Regulator of G-protein signaling, putative [Pediculus humanus corporis]	3,00E-24
gALL_v3_c20555	1	5	gnl|CDD|189378	pfam00079, Serpin, Serpin (se	-	-	-
gALL_v3_c20556	1	4	-	-	-	-	-
gALL_v3_c20557	0	4	-	-	-	-	-
gALL_v3_c20558	0	3	-	-	-	-	-
gALL_v3_c2056	1	6	gnl|CDD|35182	COG5623, CLP1, Predicted GTI	gb|EFZ16539.1|	hypothetical protein SINV_07008 [Solenopsis invicta]	8,00E-85
gALL_v3_c20561	2	2	-	-	-	-	-
gALL_v3_c20562	1	8	-	-	gb|AGM32807.1|	hypothetical protein [Coptotermes formosanus]	2,00E-43
gALL_v3_c20563	1	5	-	-	-	-	-
gALL_v3_c20564	0	4	-	-	-	-	-
gALL_v3_c20565	2	1	-	-	gb|ESO99859.1|	hypothetical protein LOTGIDRAFT_141398 [Lottia gigantea]	3,00E-06
gALL_v3_c20566	2	2	-	-	-	-	-
gALL_v3_c20567	1	4	-	-	-	-	-
gALL_v3_c20569	1	3	-	-	-	-	-
gALL_v3_c2057	15	9	gnl|CDD|162451	TIGR01622, SF-CC1, splicing fa	gb|EFN77277.1|	RNA-binding protein 39 [Harpegnathos saltator]	3,00E-62
gALL_v3_c20570	1	4	gnl|CDD|189424	pfam00153, Mito_carr, Mitoch	gb|EGI68501.1|	Kidney mitochondrial carrier protein 1 [Acromyrmex echinatior]	2,00E-90
gALL_v3_c20571	0	4	-	-	ref|XP_002430691.1|	synaptotagmin-15, putative [Pediculus humanus corporis] gb|EEB17953.1| synaptotagmin-15, putative [Pediculus humanus corporis]	5,00E-19
gALL_v3_c20572	5	6	-	-	gb|EFN72971.1|	High affinity cAMP-specific 3',5'-cyclic phosphodiesterase 7A [Camponotus floridanus]	3,00E-15
gALL_v3_c20574	3	8	gnl|CDD|188655	cd09823, peroxinectin_like, pe	ref|XP_002426138.1|	conserved hypothetical protein [Pediculus humanus corporis] gb|EEB13400.1| conserved hypothetical protein [Pediculus humanus corporis]	1,00E-151
gALL_v3_c20576	5	2	-	-	-	-	-
gALL_v3_c20577	0	4	-	-	-	-	-
gALL_v3_c20578	0	4	-	-	ref|XP_001870624.1|	smile protein [Culex quinquefasciatus] gb|EDS35308.1| smile protein [Culex quinquefasciatus]	5,00E-18
gALL_v3_c20579	3	2	-	-	-	-	-
gALL_v3_c2058	2	5	-	-	-	-	-
gALL_v3_c20580	3	2	-	-	gb|EFX86430.1|	hypothetical protein DAPPUDRAFT_313131 [Daphnia pulex]	7,00E-12
gALL_v3_c20581	2	2	-	-	-	-	-
gALL_v3_c20582	3	1	gnl|CDD|29257	cd00200, WD40, WD40 domai	ref|XP_002399519.1|	dactylin, putative [Ixodes scapularis] gb|EEC11200.1| dactylin, putative [Ixodes scapularis]	3,00E-21
gALL_v3_c20583	2	1	-	-	-	-	-
gALL_v3_c20585	3	4	-	-	-	-	-
gALL_v3_c20586	0	5	-	-	-	-	-
gALL_v3_c20587	5	6	gnl|CDD|128477	smart00180, EGF_Lam, Lamini	ref|XP_968232.1|	PREDICTED: similar to cadherin [Tribolium castaneum]	0
gALL_v3_c20588	1	3	-	-	-	-	-
gALL_v3_c20589	1	4	-	-	-	-	-
gALL_v3_c2059	4	8	gnl|CDD|128379	smart00065, GAF, Domain pre	ref|XP_002430732.1|	dual 3',5'-cyclic-AMP and -GMP phosphodiesterase, putative [Pediculus humanus corporis] gb|EEB17994.1| dual 3',5'-cyclic-AMP and -GMP phosphodiesterase, putative [F	1,00E-139
gALL_v3_c20590	1	3	-	-	-	-	-
gALL_v3_c20592	2	3	-	-	ref|XP_004850358.1|	PREDICTED: uncharacterized protein LOC101721125 [Heterocephalus glaber]	7,00E-33
gALL_v3_c20593	2	3	-	-	-	-	-
gALL_v3_c20594	3	1	-	-	ref|XP_974331.2|	PREDICTED: similar to AGAP012144-PA [Tribolium castaneum]	1,00E-36
gALL_v3_c20595	1	4	gnl|CDD|99954	cd05524, Bromo_polybromo_	gb|ENN78044.1|	hypothetical protein YQE_05481, partial [Dendroctonus ponderosae]	2,00E-44
gALL_v3_c20596	5	2	-	-	-	-	-
gALL_v3_c20597	6	6	gnl|CDD|152565	pfam12130, DUF3585, Protein	ref|XP_006625006.1|	PREDICTED: protein-methionine sulfoxide oxidase MICAL3-like [Apis dorsata]	6,00E-27
gALL_v3_c20599	3	1	-	-	-	-	-
gALL_v3_c206	18	17	gnl|CDD|184712	PRK14501, PRK14501, putative	gb|AEW67358.1|	trehalose-6-phosphate synthase, partial [Coptotermes formosanus]	0
gALL_v3_c2060	5	8	-	-	gb|ERL91841.1|	hypothetical protein D910_09166, partial [Dendroctonus ponderosae]	9,00E-09
gALL_v3_c20600	2	2	-	-	-	-	-
gALL_v3_c20601	1	4	gnl|CDD|146653	pfam04124, Dor1, Dor1-like fa	ref|XP_003729424.1|	PREDICTED: conserved oligomeric Golgi complex subunit 8-like [Strongylocentrotus purpuratus]	1,00E-36
gALL_v3_c20602	15	17	gnl|CDD|73156	cd01650, RT_nLTR_like, RT_nL	gb|ADI61823.1|	endonuclease-reverse transcriptase [Bombyx mori]	1,00E-25
gALL_v3_c20603	4	3	-	-	gb|EFA08313.1|	hypothetical protein TcasGA2_TC005949 [Tribolium castaneum]	5,00E-78
gALL_v3_c20604	2	3	-	-	gb|EFN66655.1|	Putative uncharacterized protein FLJ37770 [Camponotus floridanus]	2,00E-44
gALL_v3_c20605	2	3	-	-	-	-	-
gALL_v3_c20606	3	2	gnl|CDD|189583	pfam00520, Ion_trans, Ion tra	gb|EFZ19826.1|	hypothetical protein SINV_00065 [Solenopsis invicta]	2,00E-89
gALL_v3_c20607	3	2	-	-	-	-	-
gALL_v3_c20609	0	6	-	-	-	-	-
gALL_v3_c20610	2	1	-	-	-	-	-
gALL_v3_c20611	0	4	-	-	gb|EFN88542.1|	Integrator complex subunit 5 [Harpegnathos saltator]	1,00E-10
gALL_v3_c20613	0	3	-	-	-	-	-
gALL_v3_c20614	3	1	gnl|CDD|145175	pfam01871, AMMECR1, AMM	ref|XP_006562347.1|	PREDICTED: uncharacterized protein LOC410361 [Apis mellifera]	5,00E-53
gALL_v3_c20616	2	8	gnl|CDD|117221	pfam08648, DUF1777, Protein	-	-	-
gALL_v3_c20617	3	3	-	-	-	-	-
gALL_v3_c20618	1	3	-	-	-	-	-
gALL_v3_c20620	0	4	-	-	-	-	-
gALL_v3_c20621	3	3	gnl|CDD|88601	cd00403, Ribosomal_L1, Ribos	ref|XP_005186410.1|	PREDICTED: 50S ribosomal protein L1, chloroplastic-like [Musca domestica]	1,00E-90
gALL_v3_c20623	2	3	-	-	-	-	-

gALL_v3_c20624	2	3	-	-	ref|XP_002429552.1|	zinc finger protein FYVE domain containing protein, putative [Pediculus humanus corporis] gb|EEB16814.1| zinc finger protein FYVE domain containing protein, putative [P	1,00E-47
gALL_v3_c20625	1	4	-	-	gb|EFZ21281.1|	hypothetical protein SINV_15254 [Solenopsis invicta]	3,00E-12
gALL_v3_c20626	4	2	-	-	ref|XP_395969.4|	PREDICTED: uncharacterized protein LOC412513 [Apis mellifera]	1,00E-103
gALL_v3_c20627	4	2	-	-	-	-	-
gALL_v3_c20628	1	3	-	-	-	-	-
gALL_v3_c20629	0	5	-	-	-	-	-
gALL_v3_c20630	2	4	gnl|CDD|34870	COG5273, COG5273, Uncharac	ref|XP_319681.2|	AGAP008928-PA [Anopheles gambiae str. PEST] gb|EAA14821.2| AGAP008928-PA [Anopheles gambiae str. PEST]	3,00E-74
gALL_v3_c20631	3	6	-	-	-	-	-
gALL_v3_c20632	3	1	-	-	-	-	-
gALL_v3_c20633	3	3	-	-	-	-	-
gALL_v3_c20634	3	1	-	-	-	-	-
gALL_v3_c20637	3	1	gnl|CDD|190875	pfam04130, Spc97_Spc98, Spc	gb|EFZ10339.1|	hypothetical protein SINV_04428 [Solenopsis invicta]	5,00E-43
gALL_v3_c20639	2	4	gnl|CDD|189411	pfam00135, COesterase, Carb	gb|ACT53736.1|	juvenile hormone esterase-like protein Est1 [Reticulitermes flavipes]	1,00E-109
gALL_v3_c2064	11	11	-	-	-	-	-
gALL_v3_c20640	1	3	-	-	-	-	-
gALL_v3_c20641	4	3	-	-	-	-	-
gALL_v3_c20642	0	4	-	-	-	-	-
gALL_v3_c20643	12	8	-	-	gb|EFN80576.1|	Putative uncharacterized transposon-derived protein F52C9.6 [Harpegnathos saltator]	9,00E-41
gALL_v3_c20644	2	6	-	-	-	-	-
gALL_v3_c20645	4	4	-	-	gb|EGI68315.1|	Neurogenic locus Notch protein [Acromyrmex echinatior]	3,00E-59
gALL_v3_c20646	2	4	-	-	-	-	-
gALL_v3_c20647	2	3	-	-	-	-	-
gALL_v3_c20648	1	3	gnl|CDD|162084	TIGR00879, SP, MFS transporte	gb|ESO89991.1|	hypothetical protein LOTGIDRAFT_124274, partial [Lottia gigantea]	9,00E-46
gALL_v3_c20649	2	4	-	-	-	-	-
gALL_v3_c2065	18	35	gnl|CDD|128829	smart00557, IG_FLMN, Filamin	ref|XP_002423659.1|	conserved hypothetical protein [Pediculus humanus corporis] gb|EEB10921.1| conserved hypothetical protein [Pediculus humanus corporis]	0
gALL_v3_c20650	1	4	-	-	-	-	-
gALL_v3_c20651	1	3	-	-	-	-	-
gALL_v3_c20652	0	4	-	-	-	-	-
gALL_v3_c20653	2	1	-	-	-	-	-
gALL_v3_c20655	3	1	-	-	gb|ESN93095.1|	hypothetical protein HELRODRAFT_165255 [Helobdella robusta]	2,00E-10
gALL_v3_c20656	5	3	-	-	-	-	-
gALL_v3_c20657	3	2	-	-	-	-	-
gALL_v3_c20658	3	1	-	-	gb|EFN86714.1|	hypothetical protein EAI_12270 [Harpegnathos saltator]	2,00E-10
gALL_v3_c20659	1	3	-	-	-	-	-
gALL_v3_c2066	8	4	-	-	-	-	-
gALL_v3_c20660	3	1	-	-	-	-	-
gALL_v3_c20661	3	6	-	-	-	-	-
gALL_v3_c20663	5	3	-	-	-	-	-
gALL_v3_c20664	0	3	-	-	-	-	-
gALL_v3_c20665	2	3	gnl|CDD|145898	pfam02990, EMP70, Endomem	ref|XP_006610901.1|	PREDICTED: transmembrane 9 superfamily member 3-like [Apis dorsata]	5,00E-61
gALL_v3_c20666	0	3	-	-	-	-	-
gALL_v3_c20667	3	3	-	-	-	-	-
gALL_v3_c20669	8	6	-	-	ref|XP_001627135.1|	predicted protein [Nematostella vectensis] gb|EDO35035.1| predicted protein [Nematostella vectensis]	2,00E-11
gALL_v3_c2067	22	14	-	-	-	-	-
gALL_v3_c20670	3	1	gnl|CDD|176076	cd08694, C2_Dock-A, C2 doma	ref|XP_003691692.1|	PREDICTED: dedicator of cytokinesis protein 1-like, partial [Apis florea]	3,00E-69
gALL_v3_c20671	1	3	-	-	-	-	-
gALL_v3_c20673	0	4	-	-	ref|XP_003700732.1|	PREDICTED: tyrosine-protein phosphatase 10D-like [Megachile rotundata]	2,00E-46
gALL_v3_c20674	3	3	-	-	-	-	-
gALL_v3_c20675	2	2	gnl|CDD|144462	pfam00876, Innexin, Innexin.	sp|Q9XYN0.1|INX1_SCH	RecName: Full=Innexin inx1; Short=Innexin-1; AltName: Full=G-Inx1 gb|AAD29305.1|AF115853_1 innexin-1 [Schistocerca americana]	3,00E-50
gALL_v3_c20676	3	1	gnl|CDD|177768	PLN00172, PLN00172, ubiquiti	gb|EFN71567.1|	Ubiquitin-conjugating enzyme E2-17 kDa [Camponotus floridanus]	5,00E-82
gALL_v3_c20677	2	2	-	-	-	-	-
gALL_v3_c20679	1	4	-	-	-	-	-
gALL_v3_c2068	25	27	gnl|CDD|130689	TIGR01628, PABP-1234, polyac	ref|XP_006609603.1|	PREDICTED: heterogeneous nuclear ribonucleoprotein 27C-like isoform X1 [Apis dorsata]	1,00E-109
gALL_v3_c20680	2	3	gnl|CDD|185611	PTZ00428, PTZ00428, 60S ribo	ref|XP_001325568.1|	ribosomal protein [Trichomonas vaginalis G3] gb|EAY13345.1| ribosomal protein, putative [Trichomonas vaginalis G3]	1,00E-18
gALL_v3_c20681	4	3	-	-	-	-	-
gALL_v3_c20682	0	4	-	-	-	-	-
gALL_v3_c20685	2	2	-	-	-	-	-
gALL_v3_c20686	0	5	-	-	-	-	-
gALL_v3_c20687	2	3	-	-	-	-	-
gALL_v3_c20688	5	6	-	-	-	-	-
gALL_v3_c20689	3	4	gnl|CDD|189544	pfam00431, CUB, CUB domain	gb|EFA01466.1|	hypothetical protein TcasGA2_TC007013 [Tribolium castaneum]	2,00E-63
gALL_v3_c20691	1	5	-	-	-	-	-
gALL_v3_c20692	1	4	-	-	-	-	-
gALL_v3_c20693	5	3	gnl|CDD|189636	pfam00622, SPRY, SPRY domai	ref|XP_002428402.1|	Ran-binding protein, putative [Pediculus humanus corporis] gb|EEB15664.1| Ran-binding protein, putative [Pediculus humanus corporis]	1,00E-152
gALL_v3_c20694	4	2	-	-	-	-	-
gALL_v3_c20695	4	3	-	-	-	-	-
gALL_v3_c20696	2	2	-	-	-	-	-
gALL_v3_c20697	3	4	-	-	-	-	-
gALL_v3_c20698	2	5	-	-	-	-	-
gALL_v3_c207	6	6	-	-	-	-	-
gALL_v3_c20701	2	7	gnl|CDD|146447	pfam03810, IBN_N, Importin-b	gb|EGI61947.1|	Importin-7 [Acromyrmex echinatior]	8,00E-96
gALL_v3_c20702	2	5	-	-	gb|EFN65619.1|	Putative uncharacterized transposon-derived protein F52C9.6 [Camponotus floridanus]	9,00E-12
gALL_v3_c20704	4	3	-	-	-	-	-
gALL_v3_c20705	2	2	-	-	-	-	-
gALL_v3_c20707	1	6	-	-	-	-	-
gALL_v3_c20708	4	8	-	-	gb|EHJ76937.1|	putative tigger transposable element derived 6-like protein [Danaus plexippus]	7,00E-15
gALL_v3_c20709	0	3	-	-	-	-	-
gALL_v3_c2071	4	7	-	-	-	-	-
gALL_v3_c20710	3	3	-	-	-	-	-
gALL_v3_c20711	1	3	-	-	-	-	-
gALL_v3_c20712	4	2	gnl|CDD|144473	pfam00888, Cullin, Cullin fami	gb|EFZ19308.1|	hypothetical protein SINV_13032 [Solenopsis invicta]	1,00E-121
gALL_v3_c20713	6	4	-	-	-	-	-
gALL_v3_c20714	3	3	gnl|CDD|73151	cd01644, RT_pepA17, RT_pep	ref|XP_003699527.1|	PREDICTED: uncharacterized protein LOC100876895 [Megachile rotundata]	2,00E-50
gALL_v3_c20715	6	5	-	-	-	-	-
gALL_v3_c20716	5	4	-	-	-	-	-
gALL_v3_c20717	3	1	-	-	-	-	-
gALL_v3_c20718	2	2	-	-	-	-	-
gALL_v3_c2072	7	9	-	-	ref|XP_006559674.1|	PREDICTED: sister chromatid cohesion protein PDS5 homolog B-B-like isoform X3 [Apis mellifera] ref|XP_006618559.1| PREDICTED: sister chromatid cohesion protein PDS!	3,00E-46
gALL_v3_c20721	2	3	-	-	-	-	-
gALL_v3_c20722	5	7	-	-	-	-	-
gALL_v3_c20723	1	2	-	-	-	-	-
gALL_v3_c20724	0	5	-	-	-	-	-
gALL_v3_c20726	1	3	-	-	-	-	-
gALL_v3_c20727	2	3	-	-	-	-	-
gALL_v3_c20729	1	5	-	-	ref|XP_001607590.1|	PREDICTED: importin-5-like [Nasonia vitripennis]	1,00E-93
gALL_v3_c20730	2	2	-	-	-	-	-
gALL_v3_c20731	4	3	-	-	-	-	-
gALL_v3_c20733	10	8	gnl|CDD|181327	PRK08256, PRK08256, lipid-tra	ref|XP_317563.2|	AGAP007921-PA [Anopheles gambiae str. PEST] gb|EAA12893.2| AGAP007921-PA [Anopheles gambiae str. PEST]	3,00E-56
gALL_v3_c20734	1	2	-	-	-	-	-
gALL_v3_c20735	2	3	gnl|CDD|177889	PLN02246, PLN02246, 4-coum	ref|XP_001604903.1|	PREDICTED: luciferin 4-monooxygenase-like [Nasonia vitripennis]	1,00E-16
gALL_v3_c20736	2	3	gnl|CDD|191810	pfam07679, I-set, Immunoglol	ref|XP_973424.1|	PREDICTED: similar to neuronal cell adhesion molecule [Tribolium castaneum]	1,00E-135
gALL_v3_c20737	3	4	-	-	-	-	-
gALL_v3_c20738	3	1	-	-	-	-	-
gALL_v3_c20739	0	4	-	-	-	-	-
gALL_v3_c2074	4	9	-	-	-	-	-
gALL_v3_c20740	1	3	-	-	-	-	-
gALL_v3_c20741	3	2	-	-	-	-	-
gALL_v3_c20742	3	2	-	-	-	-	-
gALL_v3_c20743	4	4	-	-	-	-	-
gALL_v3_c20744	3	1	gnl|CDD|192786	pfam11635, Med16, Mediator	ref|XP_003699798.1|	PREDICTED: mediator of RNA polymerase II transcription subunit 16-like isoform 1 [Megachile rotundata]	3,00E-56
gALL_v3_c20745	2	4	-	-	ref|XP_624877.1|	PREDICTED: ubiquitin domain-containing protein UBFD1-like [Apis mellifera] ref|XP_003690216.1| PREDICTED: ubiquitin domain-containing protein UBFD1-like [Apis florea	1,00E-126
gALL_v3_c20748	1	5	-	-	-	-	-
gALL_v3_c20749	4	6	-	-	-	-	-
gALL_v3_c2075	13	16	-	-	-	-	-
gALL_v3_c20750	1	6	gnl|CDD|100104	cd00590, RRM, RRM (RNA rec	gb|AGM32900.1|	putative RNA-binding protein [Coptotermes formosanus]	8,00E-74
gALL_v3_c20751	1	4	-	-	-	-	-
gALL_v3_c20752	5	8	-	-	ref|XP_003394202.1|	PREDICTED: sterile alpha and TIR motif-containing protein 1-like [Bombus terrestris]	2,00E-08
gALL_v3_c20753	3	1	-	-	ref|XP_003699105.1|	PREDICTED: lysosomal-trafficking regulator-like [Apis florea]	2,00E-25
gALL_v3_c20754	4	2	-	-	-	-	-
gALL_v3_c20756	2	4	-	-	-	-	-
gALL_v3_c20757	4	3	-	-	gb|EFN75852.1|	Craniofacial development protein 2 [Harpegnathos saltator]	9,00E-14
gALL_v3_c20758	0	4	-	-	-	-	-
gALL_v3_c20759	2	2	-	-	-	-	-
gALL_v3_c2076	5	6	-	-	ref|XP_003241980.1|	PREDICTED: neural-cadherin-like [Acyrthosiphon pisum]	1,00E-65
gALL_v3_c20760	2	2	-	-	-	-	-
gALL_v3_c20762	10	6	gnl|CDD|187601	cd05343, Mgc4172-like_SDR_	gb|AGM32440.1|	enkurin [Coptotermes formosanus]	1,00E-59
gALL_v3_c20763	3	1	-	-	-	-	-
gALL_v3_c20764	0	4	-	-	ref|XP_004933140.1|	PREDICTED: uncharacterized protein LOC101744245 [Bombyx mori]	5,00E-07
gALL_v3_c20766	1	3	-	-	-	-	-
gALL_v3_c20767	2	3	-	-	-	-	-
gALL_v3_c20768	2	1	-	-	ref|XP_970467.1|	PREDICTED: similar to GA12051-PA [Tribolium castaneum] gb|EFA10286.1| hypothetical protein TcasGA2_TC012490 [Tribolium castaneum]	7,00E-36
gALL_v3_c20769	1	4	gnl|CDD|177802	PLN00221, PLN00221, tubulin	ref|XP_005702138.1|	PREDICTED: tubulin alpha-1A chain-like, partial [Capra hircus]	6,00E-11
gALL_v3_c2077	11	10	gnl|CDD|112970	pfam04180, LTV, Low tempera	ref|XP_006611818.1|	PREDICTED: protein LTV1 homolog [Apis dorsata]	3,00E-92
gALL_v3_c20770	2	4	-	-	ref|XP_006144953.1|	PREDICTED: putative protein FAM200B isoform X1 [Tupaia chinensis] ref|XP_006144954.1| PREDICTED: putative protein FAM200B isoform X2 [Tupaia chinensis]	8,00E-15
gALL_v3_c20771	8	15	-	-	-	-	-
gALL_v3_c20772	4	10	-	-	ref|XP_798897.3|	PREDICTED: uncharacterized protein LOC594361, partial [Strongylocentrotus purpuratus]	4,00E-25
gALL_v3_c20773	1	3	-	-	-	-	-
gALL_v3_c20774	1	4	-	-	-	-	-
gALL_v3_c20775	1	5	-	-	-	-	-
gALL_v3_c20776	3	3	-	-	-	-	-
gALL_v3_c20777	3	8	gnl|CDD|133355	cd04155, Arl3, Arl3 subfamily.	ref|NP_001191878.1|	dead end [Acyrthosiphon pisum] dbj|BAH71869.1| ACYPI29249 [Acyrthosiphon pisum]	5,00E-57
gALL_v3_c20778	1	4	-	-	-	-	-
gALL_v3_c2078	3	5	gnl|CDD|110027	pfam00996, GDI, GDP dissocia	ref|XP_968281.1|	PREDICTED: similar to rab gdp-dissociation inhibitor [Tribolium castaneum] gb|EFA01887.1| hypothetical protein TcasGA2_TC007497 [Tribolium castaneum]	1,00E-92
gALL_v3_c20780	2	5	gnl|CDD|191925	pfam08005, PHR, PHR domain	ref|XP_003691571.1|	PREDICTED: E3 ubiquitin-protein ligase highwire-like, partial [Apis florea]	1,00E-102

gALL_v3_c20781	3	3	-	-	-	-	-
gALL_v3_c20782	2	2	-	-	ref|XP_005469027.1|	PREDICTED: uncharacterized protein LOC102076147 [Oreochromis niloticus]	6,00E-15
gALL_v3_c20784	2	4	-	-	-	-	-
gALL_v3_c20785	3	4	-	-	-	-	-
gALL_v3_c20786	2	2	-	-	-	-	-
gALL_v3_c20787	0	4	-	-	-	-	-
gALL_v3_c20789	4	5	-	-	-	-	-
gALL_v3_c20790	3	1	-	-	-	-	-
gALL_v3_c20792	1	5	-	-	-	-	-
gALL_v3_c20793	2	1	-	-	-	-	-
gALL_v3_c20794	6	6	gnl|CDD|162379	TIGR01473, cyoE_ctaB, protoh	ref|XP_003703119.1|	PREDICTED: protoheme IX farnesyltransferase, mitochondrial-like [Megachile rotundata]	5,00E-50
gALL_v3_c20795	2	4	-	-	-	-	-
gALL_v3_c20796	3	3	-	-	-	-	-
gALL_v3_c20797	2	2	-	-	-	-	-
gALL_v3_c20798	1	4	-	-	ref|XP_003703090.1|	PREDICTED: pyridoxal-dependent decarboxylase domain-containing protein 1-like [Megachile rotundata]	2,00E-37
gALL_v3_c20799	3	1	-	-	-	-	-
gALL_v3_c208	4	9	-	-	-	-	-
gALL_v3_c2080	4	8	gnl|CDD|189421	pfam00150, Cellulase, Cellulas	gb|AGM32066.1|	putative glycosyl hydrolase family 5 [Coptotermes formosanus]	0
gALL_v3_c20800	2	2	-	-	-	-	-
gALL_v3_c20802	1	3	-	-	-	-	-
gALL_v3_c20803	1	7	gnl|CDD|48125	cd03198, GST_C_CLIC, GST_C	gb|EFX88801.1|	hypothetical protein DAPPUDRAFT_191462 [Daphnia pulex]	4,00E-33
gALL_v3_c20804	2	5	-	-	ref|XP_002429833.1|	rest corepressor corest, protein, putative [Pediculus humanus corporis] gb|EEB17095.1| rest corepressor corest, protein, putative [Pediculus humanus corporis]	1,00E-102
gALL_v3_c20805	3	1	-	-	-	-	-
gALL_v3_c20806	4	5	-	-	ref|XP_003244602.1|	PREDICTED: hypothetical protein LOC100568563 [Acyrthosiphon pisum]	3,00E-27
gALL_v3_c20807	0	4	-	-	-	-	-
gALL_v3_c20808	3	1	-	-	ref|XP_001654057.1|	hypothetical protein AaeL_AAEL009862 [Aedes aegypti] gb|EAT38224.1| AAEL009862-PA [Aedes aegypti]	9,00E-23
gALL_v3_c20809	4	6	-	-	-	-	-
gALL_v3_c2081	6	9	-	-	-	-	-
gALL_v3_c20811	2	3	-	-	-	-	-
gALL_v3_c20812	4	3	-	-	-	-	-
gALL_v3_c20814	2	4	-	-	ref|XP_002401039.1|	OTU domain-containing protein, putative [Ixodes scapularis] gb|EEC17748.1| OTU domain-containing protein, putative [Ixodes scapularis]	1,00E-11
gALL_v3_c20815	3	1	-	-	-	-	-
gALL_v3_c20816	3	1	-	-	-	-	-
gALL_v3_c20817	3	3	-	-	-	-	-
gALL_v3_c20819	0	4	gnl|CDD|190189	pfam02008, zf-CXXC, CXXC zin	ref|XP_970863.2|	PREDICTED: similar to F-box and leucine-rich repeat protein 11 [Tribolium castaneum]	7,00E-18
gALL_v3_c2082	7	4	gnl|CDD|130056	TIGR00983, 3a0801s02tim23,	ref|XP_002426223.1|	mitochondrial import inner membrane translocase subunit Tim23, putative [Pediculus humanus corporis] gb|EEB13485.1| mitochondrial import inner membrane transloca	4,00E-74
gALL_v3_c20820	0	4	-	-	-	-	-
gALL_v3_c20821	3	1	gnl|CDD|30070	cd01439, TCCD_inducible_PAR	gb|EFA06117.1|	hypothetical protein TcasGA2_TC008960 [Tribolium castaneum]	5,00E-26
gALL_v3_c20822	2	2	-	-	-	-	-
gALL_v3_c20823	3	2	-	-	-	-	-
gALL_v3_c20824	4	3	-	-	-	-	-
gALL_v3_c20825	0	4	-	-	-	-	-
gALL_v3_c20826	1	4	-	-	-	-	-
gALL_v3_c20828	3	2	-	-	-	-	-
gALL_v3_c20829	1	4	-	-	-	-	-
gALL_v3_c20830	2	3	-	-	-	-	-
gALL_v3_c20831	3	4	gnl|CDD|30832	COG0484, DnaJ, DnaJ-class mo	ref|XP_002041584.1|	GM16745 [Drosophila sechellia] gb|EDW45400.1| GM16745 [Drosophila sechellia]	1,00E-105
gALL_v3_c20832	3	1	-	-	-	-	-
gALL_v3_c20833	6	5	-	-	gb|AGM32707.1|	hypothetical protein, partial [Coptotermes formosanus]	6,00E-16
gALL_v3_c20834	0	6	-	-	-	-	-
gALL_v3_c20835	4	3	-	-	-	-	-
gALL_v3_c20836	1	5	-	-	-	-	-
gALL_v3_c20837	2	2	-	-	-	-	-
gALL_v3_c20838	2	3	-	-	ref|XP_002423716.1|	hemicentin, putative [Pediculus humanus corporis] gb|EEB10978.1| hemicentin, putative [Pediculus humanus corporis]	7,00E-30
gALL_v3_c2084	4	11	gnl|CDD|113557	pfam04791, LMBR1, LMBR1-lil	ref|XP_003703518.1|	PREDICTED: protein LMBR1L-like [Megachile rotundata]	1,00E-139
gALL_v3_c20840	3	2	-	-	-	-	-
gALL_v3_c20841	2	3	-	-	-	-	-
gALL_v3_c20843	2	2	-	-	-	-	-
gALL_v3_c20844	4	2	-	-	gb|EFN67634.1|	Putative uncharacterized transposon-derived protein F52C9.6 [Camponotus floridanus]	2,00E-13
gALL_v3_c20845	5	3	-	-	gb|AGE61266.1|	hoar peptide [Chrysodeixis chalcites nucleopolyhedrovirus]	1,00E-06
gALL_v3_c20846	3	3	-	-	ref|XP_006612955.1|	PREDICTED: adenomatous polyposis coli protein-like isoform X1 [Apis dorsata]	1,00E-74
gALL_v3_c20847	2	1	-	-	-	-	-
gALL_v3_c20849	3	2	-	-	-	-	-
gALL_v3_c2085	3	4	-	-	gb|EFN62996.1|	Uncharacterized protein KIAA0090 [Camponotus floridanus]	1,00E-35
gALL_v3_c20851	1	4	-	-	ref|XP_003397386.1|	PREDICTED: WD repeat-containing protein 24-like [Bombus terrestris]	3,00E-84
gALL_v3_c20852	0	4	gnl|CDD|143596	cd07572, nit, Nit1, Nit 2, and r	ref|XP_002410848.1|	carbon-nitrogen hydrolase, putative [Ixodes scapularis] gb|EEC13068.1| carbon-nitrogen hydrolase, putative [Ixodes scapularis]	4,00E-52
gALL_v3_c20853	2	7	gnl|CDD|162129	TIGR00954, 3a01203, Peroxys	ref|XP_002427287.1|	peroxisomal membrane protein 70 abcd3, putative [Pediculus humanus corporis] gb|EEB14549.1| peroxisomal membrane protein 70 abcd3, putative [Pediculus humanus	1,00E-158
gALL_v3_c20854	1	3	-	-	-	-	-
gALL_v3_c20855	1	4	-	-	-	-	-
gALL_v3_c20857	1	3	-	-	-	-	-
gALL_v3_c20858	2	2	-	-	-	-	-
gALL_v3_c20859	3	3	gnl|CDD|150446	pfam09777, OSTMP1, Osteop	ref|XP_004932044.1|	PREDICTED: osteopetrosis-associated transmembrane protein 1-like, partial [Bombyx mori]	3,00E-42
gALL_v3_c2086	5	13	-	-	-	-	-
gALL_v3_c20860	1	3	-	-	-	-	-
gALL_v3_c20861	0	4	-	-	-	-	-
gALL_v3_c20862	3	2	gnl|CDD|143459	cd07141, ALDH_F1AB_F2_RAL	ref|XP_003695342.1|	PREDICTED: aldehyde dehydrogenase, mitochondrial-like [Apis florea]	2,00E-93
gALL_v3_c20863	1	4	-	-	ref|XP_002603692.1|	hypothetical protein BRAFLDRAFT_126897 [Branchiostoma floridae] gb|EEN59703.1| hypothetical protein BRAFLDRAFT_126897 [Branchiostoma floridae]	5,00E-07
gALL_v3_c20864	5	3	-	-	ref|XP_002404942.1|	hypothetical protein IscW_ISCW020410 [Ixodes scapularis] gb|EEC11899.1| hypothetical protein IscW_ISCW020410 [Ixodes scapularis]	3,00E-75
gALL_v3_c20866	0	3	-	-	-	-	-
gALL_v3_c20867	3	3	-	-	-	-	-
gALL_v3_c20868	3	5	-	-	-	-	-
gALL_v3_c20869	7	4	-	-	gb|EFN88817.1|	PiggyBac transposable element-derived protein 4 [Harpegnathos saltator]	1,00E-13
gALL_v3_c2087	7	5	gnl|CDD|48604	cd03055, GST_N_Omega, GST	gb|AFK10494.1|	GSTO1 [Locusta migratoria] gb|AHC08061.1| omega glutathione S-transferase [Locusta migratoria]	1,00E-111
gALL_v3_c20870	1	6	-	-	ref|XP_002166044.2|	PREDICTED: uncharacterized protein LOC100202447 [Hydra vulgaris]	5,00E-10
gALL_v3_c20871	2	3	-	-	-	-	-
gALL_v3_c20872	0	5	-	-	-	-	-
gALL_v3_c20875	3	2	-	-	-	-	-
gALL_v3_c20876	1	3	-	-	ref|XP_001603235.1|	PREDICTED: hypothetical protein LOC100119461 [Nasonia vitripennis]	1,00E-40
gALL_v3_c20877	3	2	-	-	ref|XP_003730218.1|	PREDICTED: uncharacterized protein LOC100889850 [Strongylocentrotus purpuratus]	3,00E-07
gALL_v3_c20878	1	4	-	-	-	-	-
gALL_v3_c20879	1	3	-	-	gb|EFA13462.1|	hypothetical protein TcasGA2_TC005062 [Tribolium castaneum]	2,00E-19
gALL_v3_c2088	20	20	-	-	-	-	-
gALL_v3_c20880	3	2	gnl|CDD|30058	cd01315, L-HYD_ALN, L-Hydar	ref|XP_001605232.2|	PREDICTED: allantoinase-like [Nasonia vitripennis]	1,00E-100
gALL_v3_c20881	6	6	gnl|CDD|191513	pfam06417, DUF1077, Protein	ref|XP_003697904.1|	PREDICTED: transmembrane protein 85-like [Apis florea] ref|XP_006620638.1| PREDICTED: ER membrane protein complex subunit 4-like [Apis dorsata]	1,00E-82
gALL_v3_c20882	1	3	-	-	-	-	-
gALL_v3_c20883	3	3	-	-	-	-	-
gALL_v3_c20884	3	4	gnl|CDD|34306	COG4690, PepD, Dipeptidase [	ref|XP_002740786.1|	PREDICTED: secernin-2-like [Saccoglossus kowalevskii]	7,00E-80
gALL_v3_c20885	2	1	-	-	-	-	-
gALL_v3_c20886	0	5	-	-	-	-	-
gALL_v3_c20887	4	6	-	-	-	-	-
gALL_v3_c20888	2	2	-	-	-	-	-
gALL_v3_c20889	3	1	-	-	-	-	-
gALL_v3_c2089	8	7	-	-	-	-	-
gALL_v3_c20890	1	4	-	-	-	-	-
gALL_v3_c20891	1	4	-	-	-	-	-
gALL_v3_c20892	1	3	-	-	-	-	-
gALL_v3_c20893	3	3	-	-	-	-	-
gALL_v3_c20894	3	2	-	-	ref|XP_002432677.1|	pdz domain containing guanine nucleotide exchange factor, pdz-gef, putative [Pediculus humanus corporis] gb|EEB19939.1| pdz domain containing guanine nucleotide exc	5,00E-60
gALL_v3_c20895	2	2	-	-	ref|XP_970571.2|	PREDICTED: similar to CG34401 CG34401-PA [Tribolium castaneum]	1,00E-58
gALL_v3_c20897	1	3	-	-	-	-	-
gALL_v3_c20898	2	2	-	-	-	-	-
gALL_v3_c20899	2	4	-	-	-	-	-
gALL_v3_c209	4	7	gnl|CDD|163693	cd08062, MPN_RPN7_8, Mpr1	ref|XP_003690861.1|	PREDICTED: LOW QUALITY PROTEIN: 26S proteasome non-ATPase regulatory subunit 7-like [Apis florea]	1,00E-111
gALL_v3_c2090	5	4	-	-	gb|EGI68365.1|	Pre-mRNA-splicing regulator female-lethal(2)D [Acromyrmex echinatior]	1,00E-07
gALL_v3_c20900	3	1	-	-	-	-	-
gALL_v3_c20901	1	3	-	-	-	-	-
gALL_v3_c20902	0	5	-	-	-	-	-
gALL_v3_c20903	2	6	-	-	gb|EGI62099.1|	hypothetical protein G5I_09593 [Acromyrmex echinatior]	5,00E-79
gALL_v3_c20904	1	3	-	-	-	-	-
gALL_v3_c20906	5	2	-	-	-	-	-
gALL_v3_c20907	5	4	-	-	-	-	-
gALL_v3_c20908	2	6	-	-	-	-	-
gALL_v3_c20909	4	4	gnl|CDD|177220	MTH00165, ND5, NADH dehyc	ref|YP_006503981.1|	NADH dehydrogenase subunit 5 (mitochondrion) [Heterotermes sp. SLC-2012] gb|AFM92481.1| NADH dehydrogenase subunit 5 (mitochondrion) [Heterotermes sp. SLC-2(	1,00E-68
gALL_v3_c20911	1	2	-	-	-	-	-
gALL_v3_c20913	2	6	gnl|CDD|128524	smart00228, PDZ, Domain pre	ref|XP_002407136.1|	glutamate receptor interacting protein, putative [Ixodes scapularis] gb|EEC03692.1| glutamate receptor interacting protein, putative [Ixodes scapularis]	5,00E-79
gALL_v3_c20915	1	4	-	-	ref|XP_003704944.1|	PREDICTED: myb-binding protein 1A-like [Megachile rotundata]	2,00E-23
gALL_v3_c20917	2	3	-	-	-	-	-
gALL_v3_c20918	3	4	-	-	gb|EFA02872.1|	hypothetical protein TcasGA2_TC007906 [Tribolium castaneum]	1,00E-33
gALL_v3_c20919	3	3	-	-	-	-	-
gALL_v3_c2092	11	13	-	-	ref|XP_002431225.1|	conserved hypothetical protein [Pediculus humanus corporis] gb|EEB18487.1| conserved hypothetical protein [Pediculus humanus corporis]	1,00E-08
gALL_v3_c20920	4	2	-	-	-	-	-
gALL_v3_c20921	1	3	-	-	-	-	-
gALL_v3_c20922	1	4	gnl|CDD|73075	cd02669, Peptidase_C19M, A	ref|XP_002028112.1|	GL21349 [Drosophila persimilis] gb|EDW37895.1| GL21349 [Drosophila persimilis]	2,00E-61
gALL_v3_c20923	4	12	-	-	ref|XP_003692692.1|	PREDICTED: tensin-3-like [Apis florea]	3,00E-09
gALL_v3_c20924	3	4	-	-	-	-	-
gALL_v3_c20925	0	5	-	-	gb|EFN82165.1|	hypothetical protein EAI_09564 [Harpegnathos saltator]	2,00E-08
gALL_v3_c20926	6	4	-	-	-	-	-
gALL_v3_c20927	1	6	-	-	-	-	-
gALL_v3_c20928	2	2	-	-	-	-	-
gALL_v3_c20929	0	3	-	-	-	-	-
gALL_v3_c2093	4	5	-	-	-	-	-
gALL_v3_c20931	2	1	-	-	-	-	-
gALL_v3_c20933	26	33	gnl|CDD|117221	pfam08648, DUF1777, Protein	-	-	-
gALL_v3_c20934	3	2	-	-	-	-	-

gALL_v3_c20935	2	3	gnl|CDD|149568	pfam08565, CDC37_M, Cdc37	gb|EFN69558.1|	Hsp90 co-chaperone Cdc37 [Camponotus floridanus]	3,00E-61
gALL_v3_c20937	3	1	-	-	-	-	-
gALL_v3_c20938	3	1	-	-	-	-	-
gALL_v3_c2094	7	10	-	-	-	-	-
gALL_v3_c20941	3	2	-	-	-	-	-
gALL_v3_c20942	4	2	-	-	-	-	-
gALL_v3_c20943	3	2	-	-	gb|AGM32368.1|	n-acetyltransferase-like protein [Coptotermes formosanus]	3,00E-80
gALL_v3_c20944	3	1	-	-	-	-	-
gALL_v3_c20945	2	4	-	-	-	-	-
gALL_v3_c20946	1	4	-	-	-	-	-
gALL_v3_c20948	3	2	-	-	-	-	-
gALL_v3_c20949	1	5	-	-	-	-	-
gALL_v3_c20950	3	4	-	-	-	-	-
gALL_v3_c20951	3	2	-	-	ref|XP_002423405.1|	conserved hypothetical protein [Pediculus humanus corporis] gb|EEB10667.1| conserved hypothetical protein [Pediculus humanus corporis]	5,00E-25
gALL_v3_c20953	2	3	-	-	-	-	-
gALL_v3_c20954	1	3	-	-	-	-	-
gALL_v3_c20955	3	4	-	-	-	-	-
gALL_v3_c20956	2	1	-	-	-	-	-
gALL_v3_c20957	2	2	-	-	dbj|BAB13738.1|	DD5 [Marsupenaeus japonicus]	3,00E-23
gALL_v3_c20958	1	2	-	-	gb|AGM32295.1|	E3 ubiquitin-protein ligase siah-1, partial [Coptotermes formosanus]	1,00E-23
gALL_v3_c20959	2	1	-	-	gb|EFA12076.1|	hypothetical protein TcasGA2_TC006883 [Tribolium castaneum]	2,00E-07
gALL_v3_c2096	7	10	-	-	-	-	-
gALL_v3_c20960	4	9	gnl|CDD|132979	cd06648, STKc_PAK_II, Catalyt	ref|XP_002428585.1|	serine/threonine-protein kinase PAK mbt, putative [Pediculus humanus corporis] gb|EEB15847.1| serine/threonine-protein kinase PAK mbt, putative [Pediculus humanus c	1,00E-116
gALL_v3_c20961	3	5	-	-	-	-	-
gALL_v3_c20962	3	2	-	-	-	-	-
gALL_v3_c20963	1	3	-	-	-	-	-
gALL_v3_c20964	3	3	-	-	-	-	-
gALL_v3_c20965	2	3	-	-	gb|ERL93495.1|	hypothetical protein D910_10784 [Dendroctonus ponderosae]	5,00E-53
gALL_v3_c20967	0	5	gnl|CDD|29006	cd02396, PCBP_like_KH, K hor	ref|XP_006618944.1|	PREDICTED: poly(rC)-binding protein 2-like isoform X6 [Apis dorsata]	9,00E-27
gALL_v3_c20968	1	5	-	-	-	-	-
gALL_v3_c20969	3	4	-	-	-	-	-
gALL_v3_c20970	1	2	-	-	-	-	-
gALL_v3_c20972	2	3	gnl|CDD|189036	cd09866, PIN_Fcf1-Utp23-H, P	gb|EFN61883.1|	rRNA-processing protein UTP23-like protein [Camponotus floridanus]	2,00E-47
gALL_v3_c20973	2	4	-	-	-	-	-
gALL_v3_c20974	1	3	-	-	-	-	-
gALL_v3_c20976	3	4	gnl|CDD|48199	cd03324, rTSbeta_L-fuconate_	gb|ERL90097.1|	hypothetical protein D910_07451 [Dendroctonus ponderosae]	3,00E-66
gALL_v3_c20977	2	3	-	-	-	-	-
gALL_v3_c20978	3	9	-	-	-	-	-
gALL_v3_c20979	2	3	-	-	ref|XP_972713.1|	PREDICTED: similar to suppressor of yeast mitotic catastrophe [Tribolium castaneum]	2,00E-62
gALL_v3_c20980	3	4	-	-	-	-	-
gALL_v3_c20981	3	3	-	-	-	-	-
gALL_v3_c20983	16	15	-	-	dbj|BAC82595.1|	reverse transcriptase [Anopheles gambiae]	7,00E-32
gALL_v3_c20984	3	1	-	-	-	-	-
gALL_v3_c20985	2	3	gnl|CDD|149420	pfam08351, DUF1726, Domair	gb|EGI63113.1|	N-acetyltransferase 10 [Acromyrmex echinatior]	1,00E-50
gALL_v3_c20986	3	2	-	-	-	-	-
gALL_v3_c20987	2	4	gnl|CDD|189635	pfam00621, RhoGEF, RhoGEF (	ref|XP_002430006.1|	protein vav, putative [Pediculus humanus corporis] gb|EEB17268.1| protein vav, putative [Pediculus humanus corporis]	1,00E-105
gALL_v3_c20988	1	2	-	-	-	-	-
gALL_v3_c2099	4	6	gnl|CDD|133171	cd05039, PTKc_Csk_like, Catal	ref|XP_002423307.1|	cAMP-dependent protein kinase catalytic subunit, putative [Pediculus humanus corporis] gb|EEB10569.1| cAMP-dependent protein kinase catalytic subunit, putative [Pedi	7,00E-88
gALL_v3_c20990	2	1	-	-	-	-	-
gALL_v3_c20991	2	1	-	-	-	-	-
gALL_v3_c20992	1	5	-	-	-	-	-
gALL_v3_c20993	0	4	-	-	-	-	-
gALL_v3_c20994	3	5	-	-	gb|EGI66182.1|	hypothetical protein G5I_05300 [Acromyrmex echinatior]	2,00E-35
gALL_v3_c20995	2	1	-	-	gb|EFA03799.1|	hypothetical protein TcasGA2_TC013912 [Tribolium castaneum]	2,00E-48
gALL_v3_c20996	3	3	-	-	ref|XP_003242319.1|	PREDICTED: histone-lysine N-methyltransferase SETMAR-like [Acyrthosiphon pisum]	3,00E-24
gALL_v3_c20997	2	5	-	-	ref|XP_002430619.1|	conserved hypothetical protein [Pediculus humanus corporis] gb|EEB17881.1| conserved hypothetical protein [Pediculus humanus corporis]	3,00E-22
gALL_v3_c20998	3	2	-	-	-	-	-
gALL_v3_c20999	1	3	-	-	-	-	-
gALL_v3_c21	11	10	-	-	ref|XP_002430466.1|	highwire, putative [Pediculus humanus corporis] gb|EEB17728.1| highwire, putative [Pediculus humanus corporis]	0
gALL_v3_c210	14	15	-	-	gb|ERL94129.1|	hypothetical protein D910_11411 [Dendroctonus ponderosae]	5,00E-37
gALL_v3_c2100	4	14	-	-	-	-	-
gALL_v3_c21000	16	24	-	-	dbj|BAN20719.1|	conserved hypothetical protein [Riptortus pedestris]	1,00E-48
gALL_v3_c21001	2	3	-	-	-	-	-
gALL_v3_c21002	1	5	-	-	gb|EFA08044.1|	hypothetical protein TcasGA2_TC005638 [Tribolium castaneum]	4,00E-26
gALL_v3_c21003	1	3	-	-	-	-	-
gALL_v3_c21004	5	3	-	-	ref|XP_003400360.1|	PREDICTED: LOW QUALITY PROTEIN: DEP domain-containing protein 5-like [Bombus terrestris]	3,00E-59
gALL_v3_c21005	2	5	-	-	-	-	-
gALL_v3_c21006	2	3	-	-	-	-	-
gALL_v3_c21007	4	7	-	-	-	-	-
gALL_v3_c21008	2	3	-	-	-	-	-
gALL_v3_c21009	2	4	-	-	ref|XP_001606946.2|	PREDICTED: zinc transporter 9-like [Nasonia vitripennis]	7,00E-72
gALL_v3_c2101	24	53	gnl|CDD|173412	PTZ00121, PTZ00121, MAEBL;	gb|EFZ13087.1|	hypothetical protein SINV_01289 [Solenopsis invicta]	2,00E-29
gALL_v3_c21010	1	5	-	-	gb|EFN66973.1|	Tyrosine-protein phosphatase 69D [Camponotus floridanus]	3,00E-60
gALL_v3_c21011	2	2	-	-	-	-	-
gALL_v3_c21012	2	2	-	-	-	-	-
gALL_v3_c21013	2	5	-	-	-	-	-
gALL_v3_c21015	4	2	gnl|CDD|176924	cd09071, FAR_C, C-terminal d	ref|XP_001602857.1|	PREDICTED: putative fatty acyl-CoA reductase CG5065-like [Nasonia vitripennis]	7,00E-23
gALL_v3_c21017	1	5	-	-	-	-	-
gALL_v3_c21018	3	1	-	-	-	-	-
gALL_v3_c21019	3	2	-	-	ref|XP_003493777.1|	PREDICTED: protein unc-45 homolog B-like [Bombus impatiens]	5,00E-46
gALL_v3_c2102	15	18	-	-	-	-	-
gALL_v3_c21020	3	1	-	-	ref|XP_006134159.1|	PREDICTED: histone-lysine N-methyltransferase SETMAR-like [Pelodiscus sinensis]	2,00E-15
gALL_v3_c21021	1	3	gnl|CDD|178842	PRK00074, guaA, GMP synthas	gb|AEV89778.1|	glutamine amidotransferase, partial [Schistocerca gregaria]	2,00E-58
gALL_v3_c21022	5	5	gnl|CDD|189824	pfam01061, ABC2_membrane	ref|XP_001607395.1|	PREDICTED: ATP-binding cassette sub-family G member 1-like isoform 1 [Nasonia vitripennis] ref|XP_003423973.1| PREDICTED: ATP-binding cassette sub-family G member	1,00E-73
gALL_v3_c21023	0	3	-	-	-	-	-
gALL_v3_c21024	3	1	-	-	-	-	-
gALL_v3_c21025	1	3	-	-	-	-	-
gALL_v3_c21026	3	4	-	-	-	-	-
gALL_v3_c21028	6	7	-	-	ref|XP_966849.2|	PREDICTED: similar to endonuclease and reverse transcriptase-like protein [Tribolium castaneum]	7,00E-37
gALL_v3_c21029	1	5	-	-	-	-	-
gALL_v3_c21030	1	4	gnl|CDD|175983	cd04016, C2_Tollip, C2 domair	ref|XP_002422717.1|	conserved hypothetical protein [Pediculus humanus corporis] gb|EEB09979.1| conserved hypothetical protein [Pediculus humanus corporis]	7,00E-64
gALL_v3_c21031	3	1	-	-	-	-	-
gALL_v3_c21032	1	3	-	-	-	-	-
gALL_v3_c21033	2	3	-	-	-	-	-
gALL_v3_c21034	5	13	-	-	ref|XP_001842971.1|	ryanodine receptor [Culex quinquefasciatus] gb|EDS29360.1| ryanodine receptor [Culex quinquefasciatus]	4,00E-26
gALL_v3_c21037	5	6	-	-	gb|ADI61823.1|	endonuclease-reverse transcriptase [Bombyx mori]	9,00E-12
gALL_v3_c21038	3	4	-	-	-	-	-
gALL_v3_c21039	2	3	-	-	-	-	-
gALL_v3_c2104	8	8	-	-	-	-	-
gALL_v3_c21040	1	4	-	-	-	-	-
gALL_v3_c21041	3	1	gnl|CDD|189654	pfam00651, BTB, BTB/POZ dor	gb|ENN74814.1|	hypothetical protein YQE_08587, partial [Dendroctonus ponderosae] gb|ERL86090.1| hypothetical protein D910_03504 [Dendroctonus ponderosae]	3,00E-24
gALL_v3_c21042	3	2	-	-	-	-	-
gALL_v3_c21043	0	5	-	-	ref|XP_003424526.1|	PREDICTED: vacuolar protein sorting-associated protein 41 homolog [Nasonia vitripennis]	6,00E-32
gALL_v3_c21044	1	1	-	-	-	-	-
gALL_v3_c21045	4	4	-	-	-	-	-
gALL_v3_c21046	2	2	-	-	gb|AGM32881.1|	hypothetical protein [Coptotermes formosanus]	2,00E-27
gALL_v3_c21047	1	3	-	-	-	-	-
gALL_v3_c21048	2	2	gnl|CDD|177903	PLN02262, PLN02262, fructos	ref|XP_001607969.1|	PREDICTED: fructose-1,6-bisphosphatase 1-like [Nasonia vitripennis]	9,00E-39
gALL_v3_c21049	1	3	-	-	ref|XP_003707100.1|	PREDICTED: uncharacterized protein LOC100878688 [Megachile rotundata]	7,00E-06
gALL_v3_c2105	6	10	-	-	-	-	-
gALL_v3_c21050	1	3	gnl|CDD|34753	COG5152, COG5152, Uncharac	ref|XP_002425037.1|	RING finger protein 113A, putative [Pediculus humanus corporis] gb|EEB12299.1| RING finger protein 113A, putative [Pediculus humanus corporis]	1,00E-72
gALL_v3_c21051	3	3	gnl|CDD|189424	pfam00153, Mito_carr, Mitoch	ref|XP_001122019.2|	PREDICTED: mitochondrial carrier homolog 2 isoform X1 [Apis mellifera] ref|XP_003693549.1| PREDICTED: mitochondrial carrier homolog 2-like [Apis florea] ref|XP_0066	6,00E-68
gALL_v3_c21052	2	5	-	-	-	-	-
gALL_v3_c21053	4	5	gnl|CDD|111114	pfam02184, HAT, HAT (Half-A-	ref|XP_006609978.1|	PREDICTED: protein crooked neck-like [Apis dorsata]	0
gALL_v3_c21054	2	2	-	-	-	-	-
gALL_v3_c21055	4	3	gnl|CDD|178745	PLN03206, PLN03206, phosph	ref|XP_002422737.1|	Phosphoribosylformylglycinamidine synthase, putative [Pediculus humanus corporis] gb|EEB09999.1| Phosphoribosylformylglycinamidine synthase, putative [Pediculus hu	2,00E-66
gALL_v3_c21056	2	2	-	-	-	-	-
gALL_v3_c21057	3	1	-	-	-	-	-
gALL_v3_c21058	4	2	-	-	-	-	-
gALL_v3_c21059	2	4	-	-	-	-	-
gALL_v3_c21060	6	18	gnl|CDD|48629	cd03080, GST_N_Metaxin_like	dbj|BAN20940.1|	failed axon connections protein, putative [Riptortus pedestris]	1,00E-123
gALL_v3_c21061	2	2	-	-	-	-	-
gALL_v3_c21063	3	6	-	-	-	-	-
gALL_v3_c21064	2	1	-	-	-	-	-
gALL_v3_c21066	3	3	gnl|CDD|163676	cd07938, DRE_TIM_HMGL, 3-l	gb|AGM32521.1|	putative hydroxymethylglutaryl-coA lyase, partial [Coptotermes formosanus]	2,00E-53
gALL_v3_c21067	0	5	-	-	ref|XP_001946070.2|	PREDICTED: hypothetical protein LOC100169018 [Acyrthosiphon pisum]	8,00E-46
gALL_v3_c21068	1	2	-	-	-	-	-
gALL_v3_c21069	7	11	gnl|CDD|113680	pfam04916, Phospholip_B, Ph	ref|XP_004932087.1|	PREDICTED: putative phospholipase B-like lamina ancestor-like isoform X1 [Bombyx mori] ref|XP_004932088.1| PREDICTED: putative phospholipase B-like lamina ancestor	1,00E-40
gALL_v3_c2107	17	20	gnl|CDD|30346	cd02859, AMPKbeta_GBD_like	gb|EFN83049.1|	5'-AMP-activated protein kinase subunit beta-2 [Harpegnathos saltator]	9,00E-95
gALL_v3_c21070	0	4	gnl|CDD|191920	pfam07992, Pyr_redox_2, Pyri	ref|XP_002431722.1|	programmed cell death protein 8, putative [Pediculus humanus corporis] gb|EEB18984.1| programmed cell death protein 8, putative [Pediculus humanus corporis]	5,00E-45
gALL_v3_c21071	4	2	gnl|CDD|133327	cd04127, Rab27A, Rab27a sub	ref|XP_967715.1|	PREDICTED: similar to Ras-related protein Rab-27A (Rab-27) (GTP-binding protein Ram) (Ram p25) [Tribolium castaneum] gb|EEZ98999.1| hypothetical protein TcasGA2_TC	3,00E-78
gALL_v3_c21073	1	2	-	-	ref|XP_002427184.1|	class B secretin-like G-protein coupled receptor GPRmth5, putative [Pediculus humanus corporis] gb|EEB14446.1| class B secretin-like G-protein coupled receptor GPRmth	3,00E-09
gALL_v3_c21075	2	3	-	-	-	-	-
gALL_v3_c21076	2	3	-	-	-	-	-
gALL_v3_c21077	3	2	-	-	-	-	-
gALL_v3_c21078	2	5	gnl|CDD|162469	TIGR01657, P-ATPase-V, P-type	ref|XP_002425353.1|	cation-transporting ATPase fly, putative [Pediculus humanus corporis] gb|EEB12615.1| cation-transporting ATPase fly, putative [Pediculus humanus corporis]	6,00E-66
gALL_v3_c21079	3	3	-	-	-	-	-
gALL_v3_c2108	5	3	gnl|CDD|180029	PRK05342, clpX, ATP-depende	ref|XP_002428757.1|	ATP-dependent Clp protease ATP-binding subunit Clpx, putative [Pediculus humanus corporis] gb|EEB16019.1| ATP-dependent Clp protease ATP-binding subunit Clpx, puta	3,00E-49
gALL_v3_c21080	3	3	gnl|CDD|151663	pfam11221, Med21, Subunit 2	gb|EHJ71174.1|	putative Srb7pRNA polymerase II holoenzyme component [Danaus plexippus]	3,00E-46
gALL_v3_c21081	1	3	gnl|CDD|28945	cd00063, FN3, Fibronectin typ	ref|XP_006624185.1|	PREDICTED: tyrosine-protein phosphatase Lar-like [Apis dorsata]	3,00E-48
gALL_v3_c21082	3	5	-	-	-	-	-
gALL_v3_c21083	3	2	gnl|CDD|147949	pfam06068, TIP49, TIP49 C-ter	ref|XP_004321297.1|	PREDICTED: ruvB-like 1-like [Tursiops truncatus]	2,00E-19
gALL_v3_c21084	2	3	gnl|CDD|80333	cd04450, DEP_RGS7-like, DEP	ref|XP_967637.1|	PREDICTED: similar to regulator of g protein signaling [Tribolium castaneum] gb|EFA11094.1| hypothetical protein TcasGA2_TC004691 [Tribolium castaneum]	8,00E-40
gALL_v3_c21085	3	4	-	-	-	-	-

<table>
<tr><td>gALL_v3_c21086</td><td>3</td><td>3</td><td>-</td><td>-</td><td>-</td><td>-</td><td>-</td></tr>
<tr><td>gALL_v3_c21087</td><td>1</td><td>4</td><td>-</td><td>-</td><td>-</td><td>-</td><td>-</td></tr>
<tr><td>gALL_v3_c21088</td><td>2</td><td>1</td><td>-</td><td>-</td><td>ref|XP_001353353.1|</td><td>GA22014 [Drosophila pseudoobscura pseudoobscura] ref|XP_002021094.1| GL25014 [Drosophila persimilis] gb|EAL30859.1| GA22014 [Drosophila pseudoobscura pseudo</td><td>3,00E-41</td></tr>
<tr><td>gALL_v3_c21089</td><td>1</td><td>3</td><td>-</td><td>-</td><td>-</td><td>-</td><td>-</td></tr>
<tr><td>gALL_v3_c2109</td><td>12</td><td>16</td><td>-</td><td>-</td><td>-</td><td>-</td><td>-</td></tr>
<tr><td>gALL_v3_c21090</td><td>1</td><td>7</td><td>-</td><td>-</td><td>-</td><td>-</td><td>-</td></tr>
<tr><td>gALL_v3_c21092</td><td>2</td><td>3</td><td>-</td><td>-</td><td>-</td><td>-</td><td>-</td></tr>
<tr><td>gALL_v3_c21093</td><td>0</td><td>5</td><td>-</td><td>-</td><td>ref|WP_021882513.1|</td><td>Putative uncharacterized protein [Paenibacillus sp. P22] emb|CDN41082.1| Putative uncharacterized protein [Paenibacillus sp. P22]</td><td>1,00E-15</td></tr>
<tr><td>gALL_v3_c21094</td><td>0</td><td>4</td><td>-</td><td>-</td><td>-</td><td>-</td><td>-</td></tr>
<tr><td>gALL_v3_c21096</td><td>3</td><td>5</td><td>gnl|CDD|31308</td><td>COG1111, MPH1, ERCC4-like h</td><td>gb|ESO87655.1|</td><td>hypothetical protein LOTGIDRAFT_127429 [Lottia gigantea]</td><td>1,00E-99</td></tr>
<tr><td>gALL_v3_c21097</td><td>2</td><td>2</td><td>-</td><td>-</td><td>-</td><td>-</td><td>-</td></tr>
<tr><td>gALL_v3_c21098</td><td>2</td><td>1</td><td>-</td><td>-</td><td>gb|AAG17116.1|AF1892</td><td>homogentisate 1,2-dioxygenase [Danio rerio]</td><td>6,00E-07</td></tr>
<tr><td>gALL_v3_c21099</td><td>3</td><td>1</td><td>gnl|CDD|29257</td><td>cd00200, WD40, WD40 domai</td><td>ref|XP_001604198.2|</td><td>PREDICTED: actin-interacting protein 1-like [Nasonia vitripennis]</td><td>9,00E-98</td></tr>
<tr><td>gALL_v3_c211</td><td>20</td><td>18</td><td>gnl|CDD|148981</td><td>pfam07677, A2M_recep, A-ma</td><td>gb|EFN69033.1|</td><td>Alpha-2-macroglobulin-like protein 1 [Camponotus floridanus]</td><td>1,00E-106</td></tr>
<tr><td>gALL_v3_c2110</td><td>10</td><td>8</td><td>gnl|CDD|147609</td><td>pfam05517, p25-alpha, p25-al</td><td>gb|ETN66092.1|</td><td>TPPP family protein [Anopheles darlingi]</td><td>3,00E-74</td></tr>
<tr><td>gALL_v3_c21100</td><td>3</td><td>2</td><td>-</td><td>-</td><td>-</td><td>-</td><td>-</td></tr>
<tr><td>gALL_v3_c21102</td><td>2</td><td>6</td><td>-</td><td>-</td><td>-</td><td>-</td><td>-</td></tr>
<tr><td>gALL_v3_c21104</td><td>2</td><td>2</td><td>-</td><td>-</td><td>-</td><td>-</td><td>-</td></tr>
<tr><td>gALL_v3_c21105</td><td>0</td><td>4</td><td>-</td><td>-</td><td>-</td><td>-</td><td>-</td></tr>
<tr><td>gALL_v3_c21106</td><td>1</td><td>5</td><td>-</td><td>-</td><td>-</td><td>-</td><td>-</td></tr>
<tr><td>gALL_v3_c21107</td><td>4</td><td>2</td><td>gnl|CDD|149463</td><td>pfam08409, DUF1736, Domair</td><td>ref|XP_002426792.1|</td><td>smile protein, putative [Pediculus humanus corporis] gb|EEB14054.1| smile protein, putative [Pediculus humanus corporis]</td><td>1,00E-120</td></tr>
<tr><td>gALL_v3_c21108</td><td>0</td><td>4</td><td>-</td><td>-</td><td>-</td><td>-</td><td>-</td></tr>
<tr><td>gALL_v3_c2111</td><td>7</td><td>14</td><td>gnl|CDD|147654</td><td>pfam05600, DUF773, Protein c</td><td>ref|XP_006612952.1|</td><td>PREDICTED: CDK5 regulatory subunit-associated protein 3-like [Apis dorsata]</td><td>1,00E-115</td></tr>
<tr><td>gALL_v3_c21110</td><td>2</td><td>4</td><td>-</td><td>-</td><td>-</td><td>-</td><td>-</td></tr>
<tr><td>gALL_v3_c21112</td><td>1</td><td>3</td><td>-</td><td>-</td><td>-</td><td>-</td><td>-</td></tr>
<tr><td>gALL_v3_c21113</td><td>2</td><td>6</td><td>-</td><td>-</td><td>-</td><td>-</td><td>-</td></tr>
<tr><td>gALL_v3_c21114</td><td>4</td><td>4</td><td>-</td><td>-</td><td>-</td><td>-</td><td>-</td></tr>
<tr><td>gALL_v3_c21115</td><td>1</td><td>5</td><td>-</td><td>-</td><td>-</td><td>-</td><td>-</td></tr>
<tr><td>gALL_v3_c21116</td><td>2</td><td>2</td><td>-</td><td>-</td><td>-</td><td>-</td><td>-</td></tr>
<tr><td>gALL_v3_c21118</td><td>2</td><td>8</td><td>gnl|CDD|191030</td><td>pfam04568, IATP, Mitochondri</td><td>-</td><td>-</td><td>-</td></tr>
<tr><td>gALL_v3_c21119</td><td>1</td><td>6</td><td>-</td><td>-</td><td>-</td><td>-</td><td>-</td></tr>
<tr><td>gALL_v3_c2112</td><td>5</td><td>10</td><td>-</td><td>-</td><td>-</td><td>-</td><td>-</td></tr>
<tr><td>gALL_v3_c21120</td><td>0</td><td>4</td><td>-</td><td>-</td><td>-</td><td>-</td><td>-</td></tr>
<tr><td>gALL_v3_c21121</td><td>2</td><td>7</td><td>-</td><td>-</td><td>gb|EFN70822.1|</td><td>Activating signal cointegrator 1 complex subunit 2 [Camponotus floridanus]</td><td>3,00E-09</td></tr>
<tr><td>gALL_v3_c21122</td><td>1</td><td>6</td><td>-</td><td>-</td><td>-</td><td>-</td><td>-</td></tr>
<tr><td>gALL_v3_c21123</td><td>3</td><td>4</td><td>-</td><td>-</td><td>ref|XP_001812120.1|</td><td>PREDICTED: similar to voltage-gated ion channel [Tribolium castaneum]</td><td>8,00E-26</td></tr>
<tr><td>gALL_v3_c21124</td><td>2</td><td>5</td><td>-</td><td>-</td><td>ref|XP_006570658.1|</td><td>PREDICTED: MAP/microtubule affinity-regulating kinase 3 isoform X12 [Apis mellifera] ref|XP_006570659.1| PREDICTED: MAP/microtubule affinity-regulating kinase 3 isofo</td><td>5,00E-27</td></tr>
<tr><td>gALL_v3_c21125</td><td>3</td><td>2</td><td>-</td><td>-</td><td>-</td><td>-</td><td>-</td></tr>
<tr><td>gALL_v3_c21126</td><td>1</td><td>4</td><td>-</td><td>-</td><td>-</td><td>-</td><td>-</td></tr>
<tr><td>gALL_v3_c21127</td><td>1</td><td>2</td><td>-</td><td>-</td><td>-</td><td>-</td><td>-</td></tr>
<tr><td>gALL_v3_c21128</td><td>3</td><td>4</td><td>-</td><td>-</td><td>-</td><td>-</td><td>-</td></tr>
<tr><td>gALL_v3_c21129</td><td>4</td><td>2</td><td>-</td><td>-</td><td>ref|XP_006622085.1|</td><td>PREDICTED: protein dopey-1 homolog isoform X1 [Apis dorsata]</td><td>2,00E-75</td></tr>
<tr><td>gALL_v3_c2113</td><td>6</td><td>11</td><td>-</td><td>-</td><td>-</td><td>-</td><td>-</td></tr>
<tr><td>gALL_v3_c21130</td><td>2</td><td>2</td><td>-</td><td>-</td><td>-</td><td>-</td><td>-</td></tr>
<tr><td>gALL_v3_c21131</td><td>3</td><td>9</td><td>-</td><td>-</td><td>-</td><td>-</td><td>-</td></tr>
<tr><td>gALL_v3_c21132</td><td>2</td><td>2</td><td>-</td><td>-</td><td>gb|ERL87055.1|</td><td>hypothetical protein D910_04456 [Dendroctonus ponderosae]</td><td>3,00E-22</td></tr>
<tr><td>gALL_v3_c21133</td><td>3</td><td>6</td><td>-</td><td>-</td><td>-</td><td>-</td><td>-</td></tr>
<tr><td>gALL_v3_c21134</td><td>3</td><td>2</td><td>gnl|CDD|176533</td><td>cd08591, PI-PLCc_beta, Cataly</td><td>ref|XP_002041565.1|</td><td>GM16735 [Drosophila sechellia] gb|EDW45381.1| GM16735 [Drosophila sechellia]</td><td>1,00E-113</td></tr>
<tr><td>gALL_v3_c21135</td><td>3</td><td>3</td><td>-</td><td>-</td><td>-</td><td>-</td><td>-</td></tr>
<tr><td>gALL_v3_c21136</td><td>3</td><td>1</td><td>-</td><td>-</td><td>-</td><td>-</td><td>-</td></tr>
<tr><td>gALL_v3_c21137</td><td>3</td><td>2</td><td>-</td><td>-</td><td>-</td><td>-</td><td>-</td></tr>
<tr><td>gALL_v3_c21138</td><td>1</td><td>2</td><td>-</td><td>-</td><td>-</td><td>-</td><td>-</td></tr>
<tr><td>gALL_v3_c21139</td><td>1</td><td>3</td><td>gnl|CDD|73009</td><td>cd03250, ABCC_MRP_domain</td><td>ref|XP_001370292.1|</td><td>PREDICTED: ATP-binding cassette sub-family C member 11 [Monodelphis domestica]</td><td>3,00E-35</td></tr>
<tr><td>gALL_v3_c2114</td><td>6</td><td>12</td><td>gnl|CDD|143833</td><td>pfam00046, Homeobox, Home</td><td>ref|XP_969252.1|</td><td>PREDICTED: similar to Zinc finger homeobox protein 3 (Zinc finger homeodomain protein 3) (ZFH-3) (Alpha-fetoprotein enhancer-binding protein) (AT motif-binding factor)</td><td>1,00E-151</td></tr>
<tr><td>gALL_v3_c21141</td><td>2</td><td>3</td><td>-</td><td>-</td><td>-</td><td>-</td><td>-</td></tr>
<tr><td>gALL_v3_c21142</td><td>0</td><td>4</td><td>-</td><td>-</td><td>-</td><td>-</td><td>-</td></tr>
<tr><td>gALL_v3_c21144</td><td>4</td><td>3</td><td>-</td><td>-</td><td>-</td><td>-</td><td>-</td></tr>
<tr><td>gALL_v3_c21145</td><td>0</td><td>4</td><td>-</td><td>-</td><td>ref|XP_003393236.1|</td><td>PREDICTED: laminin subunit alpha-1-like [Bombus terrestris]</td><td>3,00E-16</td></tr>
<tr><td>gALL_v3_c21148</td><td>4</td><td>4</td><td>-</td><td>-</td><td>-</td><td>-</td><td>-</td></tr>
<tr><td>gALL_v3_c21149</td><td>4</td><td>3</td><td>-</td><td>-</td><td>ref|XP_004524227.1|</td><td>PREDICTED: ceramide glucosyltransferase-B-like [Ceratitis capitata]</td><td>4,00E-53</td></tr>
<tr><td>gALL_v3_c2115</td><td>6</td><td>14</td><td>-</td><td>-</td><td>-</td><td>-</td><td>-</td></tr>
<tr><td>gALL_v3_c21150</td><td>5</td><td>2</td><td>-</td><td>-</td><td>-</td><td>-</td><td>-</td></tr>
<tr><td>gALL_v3_c21151</td><td>2</td><td>2</td><td>-</td><td>-</td><td>-</td><td>-</td><td>-</td></tr>
<tr><td>gALL_v3_c21152</td><td>2</td><td>2</td><td>-</td><td>-</td><td>-</td><td>-</td><td>-</td></tr>
<tr><td>gALL_v3_c21153</td><td>3</td><td>2</td><td>gnl|CDD|192368</td><td>pfam09763, Sec3, Exocyst con</td><td>gb|ETN67764.1|</td><td>exocyst complex component sec3 [Anopheles darlingi]</td><td>3,00E-36</td></tr>
<tr><td>gALL_v3_c21154</td><td>0</td><td>5</td><td>-</td><td>-</td><td>-</td><td>-</td><td>-</td></tr>
<tr><td>gALL_v3_c21155</td><td>2</td><td>3</td><td>gnl|CDD|191518</td><td>pfam06424, PRP1_N, PRP1 spl</td><td>ref|XP_002428334.1|</td><td>pre-mRNA splicing factor, putative [Pediculus humanus corporis] gb|EEB15596.1| pre-mRNA splicing factor, putative [Pediculus humanus corporis]</td><td>8,00E-66</td></tr>
<tr><td>gALL_v3_c21156</td><td>2</td><td>3</td><td>-</td><td>-</td><td>-</td><td>-</td><td>-</td></tr>
<tr><td>gALL_v3_c21157</td><td>2</td><td>2</td><td>-</td><td>-</td><td>-</td><td>-</td><td>-</td></tr>
<tr><td>gALL_v3_c21158</td><td>1</td><td>4</td><td>-</td><td>-</td><td>-</td><td>-</td><td>-</td></tr>
<tr><td>gALL_v3_c21159</td><td>22</td><td>42</td><td>-</td><td>-</td><td>gb|AGM32562.1|</td><td>hypothetical protein [Coptotermes formosanus]</td><td>6,00E-22</td></tr>
<tr><td>gALL_v3_c2116</td><td>20</td><td>18</td><td>-</td><td>-</td><td>-</td><td>-</td><td>-</td></tr>
<tr><td>gALL_v3_c21160</td><td>3</td><td>7</td><td>-</td><td>-</td><td>-</td><td>-</td><td>-</td></tr>
<tr><td>gALL_v3_c21161</td><td>10</td><td>9</td><td>-</td><td>-</td><td>-</td><td>-</td><td>-</td></tr>
<tr><td>gALL_v3_c21162</td><td>11</td><td>7</td><td>gnl|CDD|73156</td><td>cd01650, RT_nLTR_like, RT_nL</td><td>ref|XP_004928419.1|</td><td>PREDICTED: uncharacterized protein LOC101746992 [Bombyx mori]</td><td>7,00E-48</td></tr>
<tr><td>gALL_v3_c21163</td><td>1</td><td>3</td><td>-</td><td>-</td><td>gb|EFN72947.1|</td><td>Dystrophin [Camponotus floridanus]</td><td>2,00E-29</td></tr>
<tr><td>gALL_v3_c21164</td><td>3</td><td>2</td><td>-</td><td>-</td><td>-</td><td>-</td><td>-</td></tr>
<tr><td>gALL_v3_c21165</td><td>0</td><td>4</td><td>-</td><td>-</td><td>-</td><td>-</td><td>-</td></tr>
<tr><td>gALL_v3_c21166</td><td>2</td><td>3</td><td>gnl|CDD|128656</td><td>smart00362, RRM_2, RNA rec</td><td>gb|AGM32299.1|</td><td>serine-arginine protein 55-like protein, partial [Coptotermes formosanus]</td><td>6,00E-55</td></tr>
<tr><td>gALL_v3_c21167</td><td>1</td><td>5</td><td>-</td><td>-</td><td>-</td><td>-</td><td>-</td></tr>
<tr><td>gALL_v3_c21168</td><td>1</td><td>3</td><td>-</td><td>-</td><td>-</td><td>-</td><td>-</td></tr>
<tr><td>gALL_v3_c2117</td><td>8</td><td>19</td><td>gnl|CDD|130585</td><td>TIGR01522, ATPase-IIA2_Ca, g</td><td>ref|XP_003398033.1|</td><td>PREDICTED: calcium-transporting ATPase type 2C member 1-like [Bombus terrestris]</td><td>0</td></tr>
<tr><td>gALL_v3_c21170</td><td>3</td><td>3</td><td>-</td><td>-</td><td>-</td><td>-</td><td>-</td></tr>
<tr><td>gALL_v3_c21172</td><td>0</td><td>6</td><td>-</td><td>-</td><td>-</td><td>-</td><td>-</td></tr>
<tr><td>gALL_v3_c21173</td><td>6</td><td>3</td><td>-</td><td>-</td><td>gb|EFX89555.1|</td><td>hypothetical protein DAPPUDRAFT_310546 [Daphnia pulex]</td><td>2,00E-07</td></tr>
<tr><td>gALL_v3_c21174</td><td>1</td><td>4</td><td>-</td><td>-</td><td>-</td><td>-</td><td>-</td></tr>
<tr><td>gALL_v3_c21175</td><td>4</td><td>2</td><td>-</td><td>-</td><td>-</td><td>-</td><td>-</td></tr>
<tr><td>gALL_v3_c21176</td><td>2</td><td>1</td><td>-</td><td>-</td><td>ref|XP_001605633.1|</td><td>PREDICTED: small subunit processome component 20 homolog [Nasonia vitripennis]</td><td>3,00E-25</td></tr>
<tr><td>gALL_v3_c21177</td><td>2</td><td>2</td><td>-</td><td>-</td><td>-</td><td>-</td><td>-</td></tr>
<tr><td>gALL_v3_c21178</td><td>3</td><td>2</td><td>-</td><td>-</td><td>-</td><td>-</td><td>-</td></tr>
<tr><td>gALL_v3_c21179</td><td>7</td><td>6</td><td>-</td><td>-</td><td>-</td><td>-</td><td>-</td></tr>
<tr><td>gALL_v3_c21182</td><td>2</td><td>2</td><td>-</td><td>-</td><td>-</td><td>-</td><td>-</td></tr>
<tr><td>gALL_v3_c21183</td><td>3</td><td>3</td><td>-</td><td>-</td><td>-</td><td>-</td><td>-</td></tr>
<tr><td>gALL_v3_c21184</td><td>3</td><td>1</td><td>-</td><td>-</td><td>-</td><td>-</td><td>-</td></tr>
<tr><td>gALL_v3_c21185</td><td>3</td><td>9</td><td>-</td><td>-</td><td>-</td><td>-</td><td>-</td></tr>
<tr><td>gALL_v3_c21186</td><td>3</td><td>2</td><td>-</td><td>-</td><td>-</td><td>-</td><td>-</td></tr>
<tr><td>gALL_v3_c21187</td><td>2</td><td>2</td><td>-</td><td>-</td><td>-</td><td>-</td><td>-</td></tr>
<tr><td>gALL_v3_c21188</td><td>1</td><td>3</td><td>-</td><td>-</td><td>-</td><td>-</td><td>-</td></tr>
<tr><td>gALL_v3_c21189</td><td>2</td><td>4</td><td>-</td><td>-</td><td>-</td><td>-</td><td>-</td></tr>
<tr><td>gALL_v3_c2119</td><td>2</td><td>9</td><td>-</td><td>-</td><td>-</td><td>-</td><td>-</td></tr>
<tr><td>gALL_v3_c21190</td><td>2</td><td>1</td><td>-</td><td>-</td><td>-</td><td>-</td><td>-</td></tr>
<tr><td>gALL_v3_c21191</td><td>1</td><td>4</td><td>-</td><td>-</td><td>-</td><td>-</td><td>-</td></tr>
<tr><td>gALL_v3_c21193</td><td>4</td><td>4</td><td>-</td><td>-</td><td>-</td><td>-</td><td>-</td></tr>
<tr><td>gALL_v3_c21194</td><td>4</td><td>2</td><td>gnl|CDD|133274</td><td>cd01871, Rac1_like, Rac1-like</td><td>ref|XP_623951.1|</td><td>PREDICTED: ras-related protein Rac1 isoform 1 [Apis mellifera] ref|XP_003401931.1| PREDICTED: ras-related protein Rac1-like [Bombus terrestris] ref|XP_003493322.1| PI</td><td>7,00E-81</td></tr>
<tr><td>gALL_v3_c21195</td><td>2</td><td>5</td><td>-</td><td>-</td><td>-</td><td>-</td><td>-</td></tr>
<tr><td>gALL_v3_c21196</td><td>3</td><td>1</td><td>-</td><td>-</td><td>-</td><td>-</td><td>-</td></tr>
<tr><td>gALL_v3_c21197</td><td>5</td><td>4</td><td>-</td><td>-</td><td>-</td><td>-</td><td>-</td></tr>
<tr><td>gALL_v3_c21198</td><td>0</td><td>3</td><td>-</td><td>-</td><td>-</td><td>-</td><td>-</td></tr>
<tr><td>gALL_v3_c21199</td><td>2</td><td>2</td><td>gnl|CDD|130689</td><td>TIGR01628, PABP-1234, polya</td><td>gb|EGI70226.1|</td><td>tRNA selenocysteine 1-associated protein 1 [Acromyrmex echinatior]</td><td>8,00E-77</td></tr>
<tr><td>gALL_v3_c212</td><td>32</td><td>35</td><td>gnl|CDD|80345</td><td>cd02123, PA_C_RZF_like, PA_C</td><td>gb|EFZ10438.1|</td><td>hypothetical protein SINV_04750 [Solenopsis invicta]</td><td>1,00E-122</td></tr>
<tr><td>gALL_v3_c2120</td><td>11</td><td>14</td><td>-</td><td>-</td><td>-</td><td>-</td><td>-</td></tr>
<tr><td>gALL_v3_c21200</td><td>2</td><td>6</td><td>gnl|CDD|133365</td><td>cd04165, GTPBP1_like, GTPBP</td><td>ref|XP_002429073.1|</td><td>GTP-binding protein, putative [Pediculus humanus corporis] gb|EEB16335.1| GTP-binding protein, putative [Pediculus humanus corporis]</td><td>6,00E-76</td></tr>
<tr><td>gALL_v3_c21201</td><td>3</td><td>2</td><td>-</td><td>-</td><td>-</td><td>-</td><td>-</td></tr>
<tr><td>gALL_v3_c21202</td><td>2</td><td>6</td><td>gnl|CDD|30100</td><td>cd01378, MYSc_type_I, Myosi</td><td>ref|XP_002424017.1|</td><td>myosin Ib, putative [Pediculus humanus corporis] gb|EEB11279.1| myosin Ib, putative [Pediculus humanus corporis]</td><td>5,00E-64</td></tr>
<tr><td>gALL_v3_c21203</td><td>0</td><td>4</td><td>-</td><td>-</td><td>-</td><td>-</td><td>-</td></tr>
<tr><td>gALL_v3_c21205</td><td>2</td><td>2</td><td>-</td><td>-</td><td>-</td><td>-</td><td>-</td></tr>
<tr><td>gALL_v3_c21206</td><td>2</td><td>2</td><td>gnl|CDD|144098</td><td>pfam00379, Chitin_bind_4, Ins</td><td>ref|XP_974046.1|</td><td>PREDICTED: similar to cuticular protein 78, RR-1 family (AGAP009876-PA) [Tribolium castaneum] gb|EFA03208.1| hypothetical protein TcasGA2_TC013128 [Tribolium casta</td><td>2,00E-52</td></tr>
<tr><td>gALL_v3_c21207</td><td>2</td><td>8</td><td>-</td><td>-</td><td>-</td><td>-</td><td>-</td></tr>
<tr><td>gALL_v3_c21208</td><td>3</td><td>3</td><td>-</td><td>-</td><td>ref|XP_004206212.1|</td><td>PREDICTED: RNA-directed DNA polymerase from mobile element jockey-like [Hydra vulgaris]</td><td>5,00E-21</td></tr>
<tr><td>gALL_v3_c21209</td><td>0</td><td>4</td><td>-</td><td>-</td><td>-</td><td>-</td><td>-</td></tr>
<tr><td>gALL_v3_c2121</td><td>4</td><td>6</td><td>-</td><td>-</td><td>ref|XP_966441.1|</td><td>PREDICTED: similar to exocyst complex component 2 [Tribolium castaneum] gb|EFA11086.1| hypothetical protein TcasGA2_TC004683 [Tribolium castaneum]</td><td>3,00E-58</td></tr>
<tr><td>gALL_v3_c21210</td><td>2</td><td>7</td><td>-</td><td>-</td><td>-</td><td>-</td><td>-</td></tr>
<tr><td>gALL_v3_c21211</td><td>4</td><td>8</td><td>-</td><td>-</td><td>-</td><td>-</td><td>-</td></tr>
<tr><td>gALL_v3_c21212</td><td>2</td><td>2</td><td>-</td><td>-</td><td>ref|XP_001663659.1|</td><td>solute carrier family 35 member C2, putative [Aedes aegypti] gb|EAT34267.1| AAEL013468-PA [Aedes aegypti]</td><td>4,00E-37</td></tr>
<tr><td>gALL_v3_c21213</td><td>1</td><td>3</td><td>-</td><td>-</td><td>-</td><td>-</td><td>-</td></tr>
<tr><td>gALL_v3_c21214</td><td>0</td><td>6</td><td>gnl|CDD|133271</td><td>cd01868, Rab11_like, Rab11-li</td><td>ref|XP_004319903.1|</td><td>PREDICTED: ras-related protein Rab-11A [Tursiops truncatus]</td><td>1,00E-47</td></tr>
<tr><td>gALL_v3_c21215</td><td>3</td><td>6</td><td>-</td><td>-</td><td>-</td><td>-</td><td>-</td></tr>
<tr><td>gALL_v3_c21216</td><td>2</td><td>3</td><td>gnl|CDD|145580</td><td>pfam02516, STT3, Oligosaccha</td><td>gb|EFN72934.1|</td><td>Dolichyl-diphosphooligosaccharide--protein glycosyltransferase subunit STT3A [Camponotus floridanus]</td><td>3,00E-66</td></tr>
<tr><td>gALL_v3_c21217</td><td>2</td><td>4</td><td>-</td><td>-</td><td>-</td><td>-</td><td>-</td></tr>
<tr><td>gALL_v3_c21218</td><td>1</td><td>2</td><td>-</td><td>-</td><td>-</td><td>-</td><td>-</td></tr>
<tr><td>gALL_v3_c21219</td><td>4</td><td>4</td><td>-</td><td>-</td><td>-</td><td>-</td><td>-</td></tr>
<tr><td>gALL_v3_c21221</td><td>2</td><td>9</td><td>gnl|CDD|176345</td><td>cd01269, PLX, Pollux (PLX) Phc</td><td>ref|XP_003706138.1|</td><td>PREDICTED: TBC1 domain family member 4-like isoform 1 [Megachile rotundata]</td><td>1,00E-100</td></tr>
<tr><td>gALL_v3_c21222</td><td>1</td><td>4</td><td>-</td><td>-</td><td>-</td><td>-</td><td>-</td></tr>
<tr><td>gALL_v3_c21223</td><td>17</td><td>18</td><td>-</td><td>-</td><td>emb|CAJ38862.1|</td><td>cytochrome c oxidase 1, partial (mitochondrion) [Periplaneta americana]</td><td>7,00E-06</td></tr>
<tr><td>gALL_v3_c21224</td><td>4</td><td>3</td><td>-</td><td>-</td><td>ref|XP_314721.3|</td><td>AGAP008626-PA [Anopheles gambiae str. PEST] gb|EAA10218.3| AGAP008626-PA [Anopheles gambiae str. PEST]</td><td>1,00E-11</td></tr>
<tr><td>gALL_v3_c21225</td><td>4</td><td>4</td><td>gnl|CDD|190189</td><td>pfam02008, zf-CXXC, CXXC zin</td><td>ref|XP_002427386.1|</td><td>JmjC domain-containing histone demethylation protein 1B, putative [Pediculus humanus corporis] gb|EEB14648.1| JmjC domain-containing histone demethylation protein</td><td>6,00E-70</td></tr>
<tr><td>gALL_v3_c21226</td><td>2</td><td>1</td><td>-</td><td>-</td><td>-</td><td>-</td><td>-</td></tr>
<tr><td>gALL_v3_c21227</td><td>2</td><td>3</td><td>-</td><td>-</td><td>-</td><td>-</td><td>-</td></tr>
<tr><td>gALL_v3_c21228</td><td>1</td><td>4</td><td>-</td><td>-</td><td>ref|XP_002432163.1|</td><td>protein tyrosine phosphatse n18, putative [Pediculus humanus corporis] gb|EEB19425.1| protein tyrosine phosphatse n18, putative [Pediculus humanus corporis]</td><td>2,00E-14</td></tr>
<tr><td>gALL_v3_c21229</td><td>2</td><td>9</td><td>gnl|CDD|144338</td><td>pfam00698, Acyl_transf_1, Ac</td><td>ref|XP_003703606.1|</td><td>PREDICTED: fatty acid synthase-like [Megachile rotundata]</td><td>2,00E-98</td></tr>
<tr><td>gALL_v3_c2123</td><td>14</td><td>27</td><td>gnl|CDD|191810</td><td>pfam07679, I-set, Immunoglol</td><td>emb|CAA63800.1|</td><td>REGA-1 protein [Schistocerca americana]</td><td>8,00E-63</td></tr>
<tr><td>gALL_v3_c21230</td><td>3</td><td>1</td><td>-</td><td>-</td><td>-</td><td>-</td><td>-</td></tr>
<tr><td>gALL_v3_c21231</td><td>1</td><td>2</td><td>-</td><td>-</td><td>-</td><td>-</td><td>-</td></tr>
<tr><td>gALL_v3_c21232</td><td>1</td><td>2</td><td>-</td><td>-</td><td>-</td><td>-</td><td>-</td></tr>
</table>

gALL_v3_c21233	3	1	-	-	-	-	-
gALL_v3_c21234	3	7	gnl|CDD|178473	PLN02885, PLN02885, nicotina	ref|XP_002429884.1|	nicotinate phosphoribosyltransferase, putative [Pediculus humanus corporis] gb|EEB17146.1| nicotinate phosphoribosyltransferase, putative [Pediculus humanus corporis	1,00E-103
gALL_v3_c21235	4	3	-	-	-	-	-
gALL_v3_c21236	1	3	-	-	-	-	-
gALL_v3_c21237	1	7	gnl|CDD|190262	pfam02259, FAT, FAT domain.	ref|XP_003702958.1|	PREDICTED: transformation/transcription domain-associated protein isoform 1 [Megachile rotundata]	0
gALL_v3_c21238	0	5	-	-	-	-	-
gALL_v3_c2124	6	3	-	-	-	-	-
gALL_v3_c21240	2	2	-	-	-	-	-
gALL_v3_c21241	1	8	gnl|CDD|189507	pfam00335, Tetraspannin, Tet	-	-	-
gALL_v3_c21242	1	3	-	-	gb|EFN66021.1|	NSFL1 cofactor p47 [Camponotus floridanus]	3,00E-53
gALL_v3_c21243	6	6	-	-	ref|XP_006568818.1|	PREDICTED: LOW QUALITY PROTEIN: twitchin [Apis mellifera]	3,00E-27
gALL_v3_c21244	1	4	-	-	-	-	-
gALL_v3_c21245	3	2	gnl|CDD|193577	cd09888, NGN_Euk, Eukaryoti	ref|XP_003490220.1|	PREDICTED: transcription elongation factor SPT5-like [Bombus impatiens]	2,00E-67
gALL_v3_c21246	1	3	-	-	-	-	-
gALL_v3_c21247	1	4	-	-	-	-	-
gALL_v3_c21248	2	2	-	-	-	-	-
gALL_v3_c2125	10	12	-	-	-	-	-
gALL_v3_c21250	1	3	-	-	-	-	-
gALL_v3_c21251	3	2	-	-	gb|ACT79642.1|	transposase [Acropora millepora]	9,00E-11
gALL_v3_c21252	2	4	-	-	-	-	-
gALL_v3_c21253	2	5	gnl|CDD|189000	cd08662, M13, Peptidase fami	ref|XP_003486119.1|	PREDICTED: endothelin-converting enzyme 1-like [Bombus impatiens]	6,00E-37
gALL_v3_c21254	3	1	gnl|CDD|177210	MTH00153, COX1, cytochrome	ref|XP_002423262.1|	conserved hypothetical protein [Pediculus humanus corporis] gb|EEB10524.1| conserved hypothetical protein [Pediculus humanus corporis]	2,00E-48
gALL_v3_c21255	3	2	-	-	-	-	-
gALL_v3_c21256	2	3	-	-	-	-	-
gALL_v3_c21258	2	3	-	-	-	-	-
gALL_v3_c21259	1	4	-	-	-	-	-
gALL_v3_c2126	6	4	gnl|CDD|189843	pfam01105, EMP24_GP25L, e	gb|AGM32196.1|	transmembrane emp24 domain-containing protein 2 precursor [Coptotermes formosanus]	1,00E-104
gALL_v3_c21260	0	4	-	-	-	-	-
gALL_v3_c21261	3	3	-	-	-	-	-
gALL_v3_c21262	4	2	-	-	-	-	-
gALL_v3_c21263	2	5	gnl|CDD|189507	pfam00335, Tetraspannin, Tet	ref|XP_966752.1|	PREDICTED: similar to tetraspanin, putative [Tribolium castaneum] gb|EFA09920.1| hypothetical protein TcasGA2_TC012071 [Tribolium castaneum]	1,00E-149
gALL_v3_c21264	3	2	gnl|CDD|162782	TIGR02244, HAD-IG-Ncltidse, I	ref|XP_972204.1|	PREDICTED: similar to CG1814 CG1814-PA [Tribolium castaneum]	1,00E-79
gALL_v3_c21265	5	8	gnl|CDD|180219	PRK05716, PRK05716, methio	ref|XP_002430065.1|	methionine aminopeptidase 1B, putative [Pediculus humanus corporis] gb|EEB17327.1| methionine aminopeptidase 1B, putative [Pediculus humanus corporis]	1,00E-134
gALL_v3_c21266	0	3	-	-	-	-	-
gALL_v3_c21267	3	3	-	-	-	-	-
gALL_v3_c21269	0	5	-	-	-	-	-
gALL_v3_c2127	14	32	gnl|CDD|192358	pfam09742, Dymeclin, Dyggve	ref|XP_001602489.1|	PREDICTED: dymeclin-like [Nasonia vitripennis]	1,00E-161
gALL_v3_c21270	2	6	-	-	gb|ADI61826.1|	endonuclease-reverse transcriptase [Bombyx mori]	4,00E-63
gALL_v3_c21271	2	2	-	-	-	-	-
gALL_v3_c21272	4	3	-	-	-	-	-
gALL_v3_c21273	2	2	-	-	-	-	-
gALL_v3_c21274	2	1	-	-	-	-	-
gALL_v3_c21275	2	4	gnl|CDD|163657	cd07414, MPP_PP1_PPKL, PP1	tpg|DAA34129.1|	TPA_exp: serine/threonine specific protein phosphatase PP1 catalytic subunit [Amblyomma variegatum]	1,00E-109
gALL_v3_c21276	0	5	-	-	ref|XP_971830.1|	PREDICTED: similar to ecto-NOX disulfide-thiol exchanger 2 [Tribolium castaneum] gb|EEZ99476.1| hypothetical protein TcasGA2_TC000049 [Tribolium castaneum]	1,00E-10
gALL_v3_c21277	3	1	-	-	-	-	-
gALL_v3_c21279	3	1	-	-	-	-	-
gALL_v3_c2128	3	10	gnl|CDD|144473	pfam00888, Cullin, Cullin fami	gb|EGI59840.1|	Cullin-4B [Acromyrmex echinatior]	1,00E-139
gALL_v3_c21280	3	1	-	-	-	-	-
gALL_v3_c21281	4	7	-	-	gb|ERL92281.1|	hypothetical protein D910_09598 [Dendroctonus ponderosae]	3,00E-33
gALL_v3_c21282	0	4	-	-	-	-	-
gALL_v3_c21283	6	9	-	-	-	-	-
gALL_v3_c21285	1	3	gnl|CDD|153343	cd07659, BAR_PICK1, The Bin	ref|XP_002425202.1|	PRKCA-binding protein, putative [Pediculus humanus corporis] gb|EEB12464.1| PRKCA-binding protein, putative [Pediculus humanus corporis]	6,00E-85
gALL_v3_c21286	2	9	-	-	ref|XP_006610559.1|	PREDICTED: squamous cell carcinoma antigen recognized by T-cells 3-like [Apis dorsata]	1,00E-120
gALL_v3_c21287	3	3	-	-	gb|EGI59813.1|	E3 ubiquitin-protein ligase Bre1 [Acromyrmex echinatior]	2,00E-55
gALL_v3_c21288	7	5	-	-	-	-	-
gALL_v3_c21289	2	3	-	-	ref|XP_003241651.1|	PREDICTED: hypothetical protein LOC100571806 [Acyrthosiphon pisum]	1,00E-15
gALL_v3_c21293	2	3	-	-	-	-	-
gALL_v3_c21294	1	3	-	-	-	-	-
gALL_v3_c21295	2	4	-	-	-	-	-
gALL_v3_c21296	2	2	-	-	-	-	-
gALL_v3_c21297	1	5	-	-	ref|XP_002427168.1|	micronuclear linker histone polyprotein MIC LH, putative [Pediculus humanus corporis] gb|EEB14430.1| micronuclear linker histone polyprotein MIC LH, putative [Pediculu	1,00E-60
gALL_v3_c21298	3	4	-	-	-	-	-
gALL_v3_c21299	2	2	-	-	-	-	-
gALL_v3_c213	9	21	-	-	gb|EFN81304.1|	Ubiquitin carboxyl-terminal hydrolase 47 [Harpegnathos saltator]	1,00E-115
gALL_v3_c2130	11	6	-	-	ref|XP_003691675.1|	PREDICTED: ancient ubiquitous protein 1-like [Apis florea]	9,00E-21
gALL_v3_c21300	3	2	-	-	-	-	-
gALL_v3_c21302	3	5	-	-	gb|EGI69284.1|	Huntingtin [Acromyrmex echinatior]	6,00E-56
gALL_v3_c21303	1	3	-	-	-	-	-
gALL_v3_c21304	3	5	gnl|CDD|31829	COG1643, HrpA, HrpA-like hel	gb|EHJ69451.1|	putative ATP-dependent RNA helicase [Danaus plexippus]	1,00E-144
gALL_v3_c21305	3	4	-	-	-	-	-
gALL_v3_c21306	2	3	-	-	-	-	-
gALL_v3_c21307	1	3	-	-	-	-	-
gALL_v3_c21308	1	5	-	-	-	-	-
gALL_v3_c21309	4	2	-	-	-	-	-
gALL_v3_c2131	25	28	-	-	-	-	-
gALL_v3_c21310	5	4	-	-	-	-	-
gALL_v3_c21311	2	1	-	-	-	-	-
gALL_v3_c21312	2	5	-	-	-	-	-
gALL_v3_c21313	0	3	-	-	-	-	-
gALL_v3_c21314	1	4	-	-	-	-	-
gALL_v3_c21315	2	2	-	-	-	-	-
gALL_v3_c21316	1	3	gnl|CDD|133004	cd02510, pp-GalNAc-T, pp-Gal	ref|XP_001946977.2|	PREDICTED: polypeptide N-acetylgalactosaminyltransferase 3-like isoform 1 [Acyrthosiphon pisum] ref|XP_003247833.1| PREDICTED: polypeptide N-acetylgalactosaminylt	5,00E-63
gALL_v3_c21317	0	3	-	-	-	-	-
gALL_v3_c21318	1	3	-	-	-	-	-
gALL_v3_c21319	2	2	-	-	-	-	-
gALL_v3_c2132	6	10	-	-	-	-	-
gALL_v3_c21320	2	2	gnl|CDD|185505	PTZ00187, PTZ00187, succinyl	gb|AGM32445.1|	alpha-succinyl coenzyme A synthetase [Coptotermes formosanus]	8,00E-51
gALL_v3_c21321	3	2	-	-	-	-	-
gALL_v3_c21322	1	2	-	-	-	-	-
gALL_v3_c21323	3	1	-	-	-	-	-
gALL_v3_c21324	4	2	-	-	-	-	-
gALL_v3_c21325	2	3	-	-	-	-	-
gALL_v3_c21327	2	4	-	-	-	-	-
gALL_v3_c21328	3	1	-	-	ref|XP_796419.3|	PREDICTED: BRCA1-A complex subunit BRE-like [Strongylocentrotus purpuratus]	7,00E-22
gALL_v3_c21329	1	2	-	-	gb|EHJ73287.1|	hypothetical protein KGM_08079 [Danaus plexippus]	7,00E-08
gALL_v3_c21330	8	4	-	-	-	-	-
gALL_v3_c21331	1	3	-	-	-	-	-
gALL_v3_c21332	4	6	-	-	gb|EHJ75938.1|	putative reverse transcriptase [Danaus plexippus]	7,00E-22
gALL_v3_c21333	3	6	gnl|CDD|129108	smart00875, BACK, BTB And C	ref|XP_002115044.1|	hypothetical protein TRIADDRAFT_50655 [Trichoplax adhaerens] gb|EDV22500.1| hypothetical protein TRIADDRAFT_50655 [Trichoplax adhaerens]	4,00E-26
gALL_v3_c21334	3	4	-	-	-	-	-
gALL_v3_c21335	2	2	-	-	-	-	-
gALL_v3_c21336	1	3	-	-	-	-	-
gALL_v3_c21337	1	3	-	-	-	-	-
gALL_v3_c21338	2	6	-	-	ref|XP_002424278.1|	tkr, putative [Pediculus humanus corporis] gb|EEB11540.1| tkr, putative [Pediculus humanus corporis]	2,00E-59
gALL_v3_c21339	2	1	-	-	-	-	-
gALL_v3_c2134	8	6	-	-	gb|ENN79838.1|	hypothetical protein YQE_03660, partial [Dendroctonus ponderosae]	5,00E-68
gALL_v3_c21340	2	1	-	-	-	-	-
gALL_v3_c21341	2	5	-	-	emb|CAM97374.1|	ecdysone inducible protein 75 [Blattella germanica]	1,00E-44
gALL_v3_c21342	6	4	-	-	-	-	-
gALL_v3_c21344	0	4	-	-	-	-	-
gALL_v3_c21345	3	2	-	-	-	-	-
gALL_v3_c21346	3	1	-	-	-	-	-
gALL_v3_c21347	2	3	-	-	gb|EHJ71473.1|	putative phosphatidate phosphatase [Danaus plexippus]	4,00E-12
gALL_v3_c21348	5	2	-	-	-	-	-
gALL_v3_c21349	3	2	-	-	-	-	-
gALL_v3_c2135	11	9	-	-	ref|XP_002430436.1|	conserved hypothetical protein [Pediculus humanus corporis] gb|EEB17698.1| conserved hypothetical protein [Pediculus humanus corporis]	6,00E-23
gALL_v3_c21351	2	3	-	-	ref|XP_004932167.1|	PREDICTED: uncharacterized protein LOC101741832 isoform X1 [Bombyx mori] ref|XP_004932168.1| PREDICTED: uncharacterized protein LOC101741832 isoform X2 [Bom	1,00E-60
gALL_v3_c21352	3	1	-	-	-	-	-
gALL_v3_c21354	1	6	-	-	-	-	-
gALL_v3_c21356	0	4	-	-	-	-	-
gALL_v3_c21357	2	2	-	-	-	-	-
gALL_v3_c21358	1	8	-	-	ref|XP_002430354.1|	hemolectin, putative [Pediculus humanus corporis] gb|EEB17616.1| hemolectin, putative [Pediculus humanus corporis]	3,00E-70
gALL_v3_c21359	2	3	-	-	-	-	-
gALL_v3_c2136	9	6	gnl|CDD|192455	pfam10075, PCI_Csn8, COP9 s	gb|EGI69549.1|	Eukaryotic translation initiation factor 3 subunit K [Acromyrmex echinatior]	1,00E-108
gALL_v3_c21360	3	1	-	-	gb|ESO98647.1|	hypothetical protein LOTGIDRAFT_180523 [Lottia gigantea]	2,00E-49
gALL_v3_c21361	0	4	-	-	-	-	-
gALL_v3_c21362	2	3	-	-	-	-	-
gALL_v3_c21363	2	6	-	-	-	-	-
gALL_v3_c21364	1	4	-	-	-	-	-
gALL_v3_c21365	2	4	-	-	gb|ELU16798.1|	hypothetical protein CAPTEDRAFT_38823, partial [Capitella teleta]	2,00E-32
gALL_v3_c21366	3	1	-	-	-	-	-
gALL_v3_c21367	2	2	-	-	-	-	-
gALL_v3_c21369	4	4	gnl|CDD|179992	PRK05286, PRK05286, dihydro	ref|XP_002428276.1|	Dihydroorotate dehydrogenase, putative [Pediculus humanus corporis] gb|EEB15538.1| Dihydroorotate dehydrogenase, putative [Pediculus humanus corporis]	1,00E-136
gALL_v3_c2137	8	9	-	-	-	-	-
gALL_v3_c21370	1	4	-	-	-	-	-
gALL_v3_c21371	2	4	-	-	-	-	-
gALL_v3_c21375	2	2	-	-	ref|XP_005095784.1|	PREDICTED: TBC1 domain family member 2B-like [Aplysia californica]	2,00E-17
gALL_v3_c21376	4	8	-	-	gb|AEE63633.1|	unknown [Dendroctonus ponderosae] gb|ENN78092.1| hypothetical protein YQE_05246, partial [Dendroctonus ponderosae] gb|ERL87496.1| hypothetical protein D910_(	1,00E-26
gALL_v3_c21377	2	2	-	-	-	-	-
gALL_v3_c21378	0	3	-	-	-	-	-
gALL_v3_c21379	3	2	-	-	-	-	-
gALL_v3_c2138	11	23	-	-	-	-	-
gALL_v3_c21380	3	3	gnl|CDD|184497	PRK14086, dnaA, chromosom	-	-	-
gALL_v3_c21381	1	5	-	-	-	-	-

gALL_v3_c21382	2	3	-	-	ref|XP_001312061.1|	hypothetical protein [Trichomonas vaginalis G3] gb|EAX99131.1| hypothetical protein TVAG_115350 [Trichomonas vaginalis G3]	2,00E-10
gALL_v3_c21384	1	3	-	-	ref|XP_006569592.1|	PREDICTED: mitochondrial carrier homolog 2 isoform X3 [Apis mellifera] ref|XP_006624661.1| PREDICTED: mitochondrial carrier homolog 2-like isoform X2 [Apis dorsata]	4,00E-34
gALL_v3_c21385	3	2	-	-	-	-	-
gALL_v3_c21388	2	2	gnl|CDD|128922	smart00678, WWE, Domain in	ref|XP_003393322.1|	PREDICTED: e3 ubiquitin-protein ligase RNF146-like [Bombus terrestris]	3,00E-22
gALL_v3_c21389	3	1	-	-	-	-	-
gALL_v3_c21390	1	3	gnl|CDD|190555	pfam03184, DDE_1, DDE supe	ref|XP_003708049.1|	PREDICTED: tigger transposable element-derived protein 1-like [Megachile rotundata]	4,00E-22
gALL_v3_c21391	0	3	-	-	-	-	-
gALL_v3_c21392	1	2	-	-	-	-	-
gALL_v3_c21393	3	2	-	-	ref|XP_001999355.1|	GI23113 [Drosophila mojavensis] gb|EDW14816.1| GI23113 [Drosophila mojavensis]	4,00E-11
gALL_v3_c21394	3	1	-	-	-	-	-
gALL_v3_c21395	0	4	-	-	-	-	-
gALL_v3_c21397	8	4	-	-	-	-	-
gALL_v3_c21398	2	2	-	-	-	-	-
gALL_v3_c21399	2	2	-	-	-	-	-
gALL_v3_c214	6	4	-	-	-	-	-
gALL_v3_c2140	11	13	-	-	-	-	-
gALL_v3_c21400	9	7	-	-	dbj|BAD00045.1|	armadillo protein [Gryllus bimaculatus]	1,00E-13
gALL_v3_c21402	1	6	gnl|CDD|189707	pfam00762, Ferrochelatase, Fe	gb|EFN69335.1|	Ferrochelatase, mitochondrial [Camponotus floridanus]	7,00E-97
gALL_v3_c21404	2	2	-	-	-	-	-
gALL_v3_c21405	5	6	-	-	-	-	-
gALL_v3_c21406	3	4	-	-	ref|XP_006559090.1|	PREDICTED: anion exchange protein 2-like [Apis mellifera]	2,00E-13
gALL_v3_c21407	3	6	-	-	-	-	-
gALL_v3_c21408	2	5	gnl|CDD|29261	cd00204, ANK, ankyrin repeat:	ref|XP_003702970.1|	PREDICTED: kinase D-interacting substrate of 220 kDa-like [Megachile rotundata]	1,00E-103
gALL_v3_c21409	2	1	-	-	ref|XP_001652955.1|	f-spondin [Aedes aegypti] gb|EAT40381.1| AAEL007889-PA [Aedes aegypti]	1,00E-18
gALL_v3_c2141	20	15	gnl|CDD|150314	pfam09606, Med15, ARC105 c	ref|XP_002426584.1|	transcription factor, putative [Pediculus humanus corporis] gb|EEB13846.1| transcription factor, putative [Pediculus humanus corporis]	1,00E-07
gALL_v3_c21410	4	3	-	-	-	-	-
gALL_v3_c21413	0	4	-	-	-	-	-
gALL_v3_c21414	2	3	-	-	-	-	-
gALL_v3_c21416	0	4	-	-	-	-	-
gALL_v3_c21417	1	2	-	-	-	-	-
gALL_v3_c21419	1	6	-	-	-	-	-
gALL_v3_c21420	3	4	-	-	-	-	-
gALL_v3_c21421	1	4	-	-	-	-	-
gALL_v3_c21422	3	2	-	-	-	-	-
gALL_v3_c21423	1	3	-	-	gb|ENN83221.1|	hypothetical protein YQE_00420, partial [Dendroctonus ponderosae]	2,00E-23
gALL_v3_c21424	1	3	-	-	-	-	-
gALL_v3_c21425	1	5	-	-	-	-	-
gALL_v3_c21426	4	4	-	-	-	-	-
gALL_v3_c21427	1	4	-	-	gb|ENN72556.1|	hypothetical protein YQE_10896, partial [Dendroctonus ponderosae]	2,00E-16
gALL_v3_c21428	1	4	-	-	-	-	-
gALL_v3_c21429	6	4	-	-	-	-	-
gALL_v3_c21431	6	6	gnl|CDD|143860	pfam00078, RVT_1, Reverse tr	gb|AGM32707.1|	hypothetical protein, partial [Coptotermes formosanus]	9,00E-33
gALL_v3_c21432	3	1	-	-	-	-	-
gALL_v3_c21433	2	5	-	-	-	-	-
gALL_v3_c21434	2	2	-	-	gb|ELU16116.1|	hypothetical protein CAPTEDRAFT_202670, partial [Capitella teleta]	2,00E-22
gALL_v3_c21436	2	4	-	-	-	-	-
gALL_v3_c21437	2	5	-	-	-	-	-
gALL_v3_c21438	3	2	-	-	-	-	-
gALL_v3_c21439	0	4	-	-	-	-	-
gALL_v3_c2144	5	8	-	-	-	-	-
gALL_v3_c21440	1	3	-	-	ref|XP_006819348.1|	PREDICTED: uncharacterized protein LOC100374480 [Saccoglossus kowalevskii]	2,00E-07
gALL_v3_c21441	3	2	-	-	-	-	-
gALL_v3_c21442	0	3	-	-	-	-	-
gALL_v3_c21443	2	2	gnl|CDD|191786	pfam07562, NCD3G, Nine Cyst	ref|XP_001849843.1|	conserved hypothetical protein [Culex quinquefasciatus] gb|EDS30958.1| conserved hypothetical protein [Culex quinquefasciatus]	8,00E-50
gALL_v3_c21446	2	3	-	-	-	-	-
gALL_v3_c21447	2	3	-	-	-	-	-
gALL_v3_c21448	0	3	-	-	-	-	-
gALL_v3_c21449	1	3	-	-	-	-	-
gALL_v3_c21450	1	2	-	-	-	-	-
gALL_v3_c21452	1	3	-	-	-	-	-
gALL_v3_c21453	3	2	gnl|CDD|128524	smart00228, PDZ, Domain pre	ref|XP_967250.1|	PREDICTED: similar to CG6619 CG6619-PA [Tribolium castaneum] gb|EEZ99637.1| hypothetical protein TcasGA2_TC002393 [Tribolium castaneum]	5,00E-87
gALL_v3_c21455	0	5	-	-	-	-	-
gALL_v3_c21456	3	1	-	-	-	-	-
gALL_v3_c21457	3	4	-	-	ref|XP_002426139.1|	DNA repair/transcription protein met18/mms19, putative [Pediculus humanus corporis] gb|EEB13401.1| DNA repair/transcription protein met18/mms19, putative [Pedicu	2,00E-18
gALL_v3_c21459	1	4	-	-	-	-	-
gALL_v3_c2146	8	6	-	-	gb|ENN81532.1|	hypothetical protein YQE_02061, partial [Dendroctonus ponderosae] gb|ERL91798.1| hypothetical protein D910_09123 [Dendroctonus ponderosae]	8,00E-93
gALL_v3_c21461	18	22	-	-	-	-	-
gALL_v3_c21462	2	3	-	-	-	-	-
gALL_v3_c21463	2	1	-	-	gb|ESO01442.1|	hypothetical protein HELRODRAFT_82060, partial [Helobdella robusta]	6,00E-06
gALL_v3_c21465	2	2	-	-	-	-	-
gALL_v3_c21466	3	1	-	-	-	-	-
gALL_v3_c21467	3	1	-	-	-	-	-
gALL_v3_c21468	1	3	-	-	-	-	-
gALL_v3_c21469	3	2	-	-	-	-	-
gALL_v3_c2147	11	12	-	-	ref|XP_002424309.1|	conserved hypothetical protein [Pediculus humanus corporis] gb|EEB11571.1| conserved hypothetical protein [Pediculus humanus corporis]	1,00E-115
gALL_v3_c21470	1	3	-	-	-	-	-
gALL_v3_c21471	2	5	gnl|CDD|189670	pfam00688, TGFb_propeptide	ref|XP_003690068.1|	PREDICTED: uncharacterized protein LOC100871171 [Apis florea]	7,00E-58
gALL_v3_c21472	1	4	-	-	ref|XP_003394849.1|	PREDICTED: hypothetical protein LOC100643686 [Bombus terrestris]	1,00E-45
gALL_v3_c21473	3	2	gnl|CDD|133072	cd03860, M14_CP_A-B_like, T	ref|XP_973407.2|	PREDICTED: similar to CG3108 CG3108-PA [Tribolium castaneum]	6,00E-63
gALL_v3_c21474	3	4	-	-	-	-	-
gALL_v3_c21475	2	3	-	-	-	-	-
gALL_v3_c21476	3	7	-	-	ref|XP_002430407.1|	Neuroglian precursor, putative [Pediculus humanus corporis] gb|EEB17669.1| Neuroglian precursor, putative [Pediculus humanus corporis]	2,00E-41
gALL_v3_c21477	1	3	-	-	ref|XP_003706679.1|	PREDICTED: dystrophin, isoforms A/C/F/G/H-like [Megachile rotundata]	7,00E-19
gALL_v3_c21478	3	4	gnl|CDD|193173	pfam12697, Abhydrolase_6, A	ref|XP_003706775.1|	PREDICTED: epoxide hydrolase 4-like [Megachile rotundata]	1,00E-122
gALL_v3_c21479	3	3	-	-	-	-	-
gALL_v3_c2148	3	9	gnl|CDD|72867	cd04752, Commd4, COMM_D	ref|XP_004687639.1|	PREDICTED: COMM domain-containing protein 4 [Condylura cristata]	4,00E-30
gALL_v3_c21480	1	7	-	-	-	-	-
gALL_v3_c21481	2	2	-	-	-	-	-
gALL_v3_c21483	4	8	-	-	-	-	-
gALL_v3_c21484	5	5	-	-	-	-	-
gALL_v3_c21486	1	3	-	-	-	-	-
gALL_v3_c21487	2	4	-	-	-	-	-
gALL_v3_c21488	0	5	-	-	gb|ACR44248.1|	IP20291p [Drosophila melanogaster]	8,00E-19
gALL_v3_c21489	3	4	gnl|CDD|28953	cd00072, GYF, GYF domain: co	gb|EFZ10734.1|	hypothetical protein SINV_13108 [Solenopsis invicta]	1,00E-17
gALL_v3_c2149	6	13	gnl|CDD|133004	cd02510, pp-GalNAc-T, pp-Gal	ref|XP_973543.1|	PREDICTED: similar to polypeptide GalNAc transferase 5 CG31651-PA [Tribolium castaneum] gb|EFA08271.1| hypothetical protein TcasGA2_TC005902 [Tribolium castaneu	1,00E-135
gALL_v3_c21490	1	4	-	-	-	-	-
gALL_v3_c21491	3	7	-	-	-	-	-
gALL_v3_c21492	2	2	-	-	-	-	-
gALL_v3_c21493	4	2	-	-	-	-	-
gALL_v3_c21494	3	6	-	-	ref|XP_002425306.1|	zfh4, putative [Pediculus humanus corporis] gb|EEB12568.1| zfh4, putative [Pediculus humanus corporis]	1,00E-101
gALL_v3_c21495	1	4	gnl|CDD|190903	pfam04189, Gcd10p, Gcd10p f	ref|XP_002427966.1|	conserved hypothetical protein [Pediculus humanus corporis] gb|EEB15228.1| conserved hypothetical protein [Pediculus humanus corporis]	2,00E-29
gALL_v3_c21496	1	4	-	-	-	-	-
gALL_v3_c21497	0	6	-	-	-	-	-
gALL_v3_c21498	4	3	-	-	-	-	-
gALL_v3_c21499	1	3	-	-	-	-	-
gALL_v3_c215	21	37	gnl|CDD|147730	pfam05735, TSP_C, Thrombos	ref|XP_002428229.1|	Thrombospondin-3 precursor, putative [Pediculus humanus corporis] gb|EEB15491.1| Thrombospondin-3 precursor, putative [Pediculus humanus corporis]	0
gALL_v3_c21501	4	4	gnl|CDD|128886	smart00638, LPD_N, Lipoprote	ref|XP_001604024.1|	PREDICTED: apolipophorins [Nasonia vitripennis]	3,00E-34
gALL_v3_c21502	3	2	-	-	-	-	-
gALL_v3_c21503	2	4	-	-	-	-	-
gALL_v3_c21504	5	3	gnl|CDD|189413	pfam00137, ATP-synt_C, ATP s	ref|XP_392599.1|	PREDICTED: V-type proton ATPase 21 kDa proteolipid subunit-like [Apis mellifera] ref|XP_006622928.1| PREDICTED: V-type proton ATPase 21 kDa proteolipid subunit-like [/	2,00E-87
gALL_v3_c21505	2	3	gnl|CDD|129101	smart00868, zf-AD, Zinc-finger	gb|EFN67495.1|	Zinc finger protein 84 [Camponotus floridanus]	2,00E-08
gALL_v3_c21506	2	4	-	-	gb|EFN80631.1|	Multiple C2 and transmembrane domain-containing protein 1 [Harpegnathos saltator]	2,00E-58
gALL_v3_c21507	6	7	-	-	gb|EFN67804.1|	hypothetical protein EAG_07302 [Camponotus floridanus]	5,00E-60
gALL_v3_c21508	1	4	-	-	-	-	-
gALL_v3_c2151	17	34	gnl|CDD|189963	pfam01384, PHO4, Phosphate	ref|XP_005186869.1|	PREDICTED: sodium-dependent phosphate transporter 1-B-like isoform X2 [Musca domestica]	1,00E-119
gALL_v3_c21510	4	2	-	-	ref|XP_006618187.1|	PREDICTED: dnaJ homolog dnj-5-like [Apis dorsata]	3,00E-07
gALL_v3_c21511	0	4	-	-	-	-	-
gALL_v3_c21512	1	3	gnl|CDD|189390	pfam00102, Y_phosphatase, P	gb|EHJ75504.1|	hypothetical protein KGM_22425 [Danaus plexippus]	7,00E-46
gALL_v3_c21513	1	3	-	-	ref|XP_001604914.2|	PREDICTED: UPF0505 protein C16orf62 homolog [Nasonia vitripennis]	1,00E-12
gALL_v3_c21514	4	4	gnl|CDD|29261	cd00204, ANK, ankyrin repeat:	gb|EHJ73801.1|	putative ankyrin repeat domain protein 17 isoform a [Danaus plexippus]	1,00E-123
gALL_v3_c21516	2	2	gnl|CDD|173660	cd05123, STKc_AGC, Catalytic	gb|ABC48929.1|	protein kinase C1 [Eisenia fetida]	2,00E-24
gALL_v3_c21517	1	3	-	-	-	-	-
gALL_v3_c2152	12	12	gnl|CDD|140227	PTZ00200, PTZ00200, cysteine	dbj|BAA86911.1|	homologue of Sarcophaga 26,29kDa proteinase [Periplaneta americana]	0
gALL_v3_c21520	0	3	-	-	-	-	-
gALL_v3_c21521	3	3	-	-	-	-	-
gALL_v3_c21522	3	3	-	-	-	-	-
gALL_v3_c21523	2	2	-	-	gb|EFA12860.1|	hypothetical protein TcasGA2_TC006945 [Tribolium castaneum]	5,00E-10
gALL_v3_c21524	2	2	-	-	ref|XP_973551.1|	PREDICTED: similar to legless CG2041-PA [Tribolium castaneum] gb|EFA11241.1| hypothetical protein TcasGA2_TC010773 [Tribolium castaneum]	3,00E-29
gALL_v3_c21525	2	3	-	-	ref|XP_002429689.1|	fatty acid synthase, putative [Pediculus humanus corporis] gb|EEB16951.1| fatty acid synthase, putative [Pediculus humanus corporis]	7,00E-22
gALL_v3_c21526	4	2	-	-	-	-	-
gALL_v3_c21527	1	4	gnl|CDD|173729	cd06617, PKc_MKK3_6, Cataly	ref|XP_003704469.1|	PREDICTED: dual specificity mitogen-activated protein kinase kinase 6-like [Megachile rotundata]	4,00E-36
gALL_v3_c21528	10	15	-	-	-	-	-
gALL_v3_c21529	4	3	-	-	-	-	-
gALL_v3_c2153	6	9	-	-	-	-	-
gALL_v3_c21530	4	4	-	-	-	-	-
gALL_v3_c21531	1	4	-	-	-	-	-
gALL_v3_c21532	2	6	-	-	-	-	-
gALL_v3_c21534	3	1	gnl|CDD|188098	TIGR00957, MRP_assoc_pro, r	gb|EGI66812.1|	Sodium leak channel non-selective protein [Acromyrmex echinatior]	4,00E-31
gALL_v3_c21535	0	4	-	-	-	-	-
gALL_v3_c21536	4	3	gnl|CDD|73156	cd01650, RT_nLTR_like, RT_nL	ref|XP_003724311.1|	PREDICTED: RNA-directed DNA polymerase from mobile element jockey-like [Strongylocentrotus purpuratus]	5,00E-41
gALL_v3_c21537	2	2	-	-	-	-	-
gALL_v3_c21539	5	5	-	-	-	-	-
gALL_v3_c21540	2	2	-	-	-	-	-
gALL_v3_c21541	5	4	gnl|CDD|162369	TIGR01452, PGP_euk, phosph	gb|EFZ12766.1|	hypothetical protein SINV_01329 [Solenopsis invicta]	5,00E-70

gALL_v3_c21542	2	1	gnl|CDD|145862	pfam02931, Neur_chan_LBD,	gb|AHJ11222.1|	nicotinic acetylcholine receptor alpha9 [Locusta migratoria]	1,00E-97
gALL_v3_c21543	1	5	gnl|CDD|190027	pfam01545, Cation_efflux, Cat	ref|XP_975475.1|	PREDICTED: similar to CG8632 CG8632-PA [Tribolium castaneum] gb|EFA01750.1| hypothetical protein TcasGA2_TC007348 [Tribolium castaneum]	1,00E-121
gALL_v3_c21544	1	5	-	-	-	-	-
gALL_v3_c21545	2	3	-	-	-	-	-
gALL_v3_c21546	2	4	-	-	-	-	-
gALL_v3_c21547	1	5	-	-	gb|ADI61823.1|	endonuclease-reverse transcriptase [Bombyx mori]	3,00E-15
gALL_v3_c21548	0	4	-	-	-	-	-
gALL_v3_c2155	6	15	gnl|CDD|185748	cd09235, V_Alix, Middle V-dor	ref|XP_973055.2|	PREDICTED: similar to programmed cell death 6-interacting protein [Tribolium castaneum]	0
gALL_v3_c21550	1	6	-	-	-	-	-
gALL_v3_c21551	2	2	-	-	-	-	-
gALL_v3_c21553	4	5	-	-	gb|EFA12644.1|	hypothetical protein TcasGA2_TC004170 [Tribolium castaneum]	8,00E-09
gALL_v3_c21554	1	3	-	-	ref|XP_002431877.1|	conserved hypothetical protein [Pediculus humanus corporis] gb|EEB19139.1| conserved hypothetical protein [Pediculus humanus corporis]	1,00E-35
gALL_v3_c21555	5	2	-	-	ref|XP_006614074.1|	PREDICTED: muscleblind-like protein 1-like, partial [Apis dorsata]	3,00E-25
gALL_v3_c21557	1	3	-	-	-	-	-
gALL_v3_c21558	2	4	-	-	-	-	-
gALL_v3_c21559	2	2	-	-	-	-	-
gALL_v3_c2156	17	19	-	-	-	-	-
gALL_v3_c21560	1	3	-	-	-	-	-
gALL_v3_c21561	5	7	-	-	-	-	-
gALL_v3_c21562	5	4	-	-	-	-	-
gALL_v3_c21563	2	2	-	-	gb|EFN77478.1|	Neogenin [Harpegnathos saltator]	5,00E-15
gALL_v3_c21564	2	5	-	-	-	-	-
gALL_v3_c21566	1	3	-	-	-	-	-
gALL_v3_c21567	0	5	gnl|CDD|189446	pfam00200, Disintegrin, Disint	gb|EFZ21792.1|	hypothetical protein SINV_09244 [Solenopsis invicta]	1,00E-78
gALL_v3_c21568	3	4	-	-	-	-	-
gALL_v3_c21569	2	3	-	-	-	-	-
gALL_v3_c2157	6	5	-	-	-	-	-
gALL_v3_c21570	1	3	-	-	gb|ETN65127.1|	Protein zer-1 [Anopheles darlingi]	1,00E-41
gALL_v3_c21571	0	5	-	-	gb|EFX71180.1|	hypothetical protein DAPPUDRAFT_60396 [Daphnia pulex]	2,00E-70
gALL_v3_c21572	5	2	-	-	gb|EHJ70865.1|	endonuclease-reverse transcriptase [Danaus plexippus]	2,00E-36
gALL_v3_c21573	3	2	-	-	-	-	-
gALL_v3_c21574	2	3	-	-	-	-	-
gALL_v3_c21575	4	2	-	-	-	-	-
gALL_v3_c21576	0	3	-	-	-	-	-
gALL_v3_c21577	1	3	-	-	ref|XP_005814536.1|	PREDICTED: piggyBac transposable element-derived protein 3-like [Xiphophorus maculatus]	5,00E-06
gALL_v3_c21579	0	4	-	-	-	-	-
gALL_v3_c2158	11	10	-	-	gb|ERL94485.1|	hypothetical protein D910_11762 [Dendroctonus ponderosae]	9,00E-26
gALL_v3_c21580	3	2	-	-	-	-	-
gALL_v3_c21581	2	2	-	-	-	-	-
gALL_v3_c21582	2	3	-	-	ref|XP_003402845.1|	PREDICTED: multiple epidermal growth factor-like domains protein 8-like [Bombus terrestris]	2,00E-53
gALL_v3_c21583	3	1	gnl|CDD|190885	pfam04147, Nop14, Nop14-lik	ref|XP_002430143.1|	histone-lysine N-methyltransferase SUVR5, putative [Pediculus humanus corporis] gb|EEB17405.1| histone-lysine N-methyltransferase SUVR5, putative [Pediculus humanu	7,00E-14
gALL_v3_c21584	2	1	-	-	-	-	-
gALL_v3_c21585	3	1	-	-	-	-	-
gALL_v3_c21586	3	7	-	-	-	-	-
gALL_v3_c21587	2	4	-	-	-	-	-
gALL_v3_c21589	0	5	-	-	-	-	-
gALL_v3_c2159	5	8	gnl|CDD|190183	pfam01984, dsDNA_bind, Dou	gb|AGM32563.1|	programmed cell death protein 5-like protein [Coptotermes formosanus]	6,00E-79
gALL_v3_c21591	8	8	-	-	gb|AGM32427.1|	soldier-caste specific protein [Coptotermes formosanus]	1,00E-33
gALL_v3_c21592	1	7	-	-	ref|XP_003395729.1|	PREDICTED: voltage-dependent calcium channel subunit alpha-2/delta-3-like [Bombus terrestris]	6,00E-77
gALL_v3_c21593	3	3	-	-	gb|EKC29295.1|	Piwi-like protein 2 [Crassostrea gigas]	5,00E-25
gALL_v3_c21594	2	2	-	-	-	-	-
gALL_v3_c21595	4	5	-	-	-	-	-
gALL_v3_c21596	1	3	-	-	-	-	-
gALL_v3_c21597	5	9	gnl|CDD|147416	pfam05210, Sprouty, Sprouty	ref|XP_973145.2|	PREDICTED: similar to sprouty [Tribolium castaneum]	3,00E-56
gALL_v3_c21598	2	4	gnl|CDD|190865	pfam04096, Nucleoporin2, Nu	ref|XP_312609.5|	AGAP002351-PA [Anopheles gambiae str. PEST] gb|EAA08227.5| AGAP002351-PA [Anopheles gambiae str. PEST]	3,00E-58
gALL_v3_c21599	6	6	gnl|CDD|190856	pfam04078, Rcd1, Cell differer	ref|XP_002425706.1|	Cell differentiation protein rcd1, putative [Pediculus humanus corporis] gb|EEB12968.1| Cell differentiation protein rcd1, putative [Pediculus humanus corporis]	1,00E-144
gALL_v3_c216	17	25	gnl|CDD|143860	pfam00078, RVT_1, Reverse tr	gb|AGM32821.1|	hypothetical protein [Coptotermes formosanus]	2,00E-57
gALL_v3_c21600	2	2	-	-	-	-	-
gALL_v3_c21601	2	3	-	-	-	-	-
gALL_v3_c21602	2	2	-	-	-	-	-
gALL_v3_c21603	3	1	gnl|CDD|192037	pfam08433, KTI12, Chromatin	ref|XP_005995709.1|	PREDICTED: protein KTI12 homolog isoform X1 [Latimeria chalumnae]	1,00E-42
gALL_v3_c21606	2	1	-	-	ref|XP_003425532.1|	PREDICTED: neuronal membrane glycoprotein M6-b-like isoform 2 [Nasonia vitripennis]	1,00E-27
gALL_v3_c21607	1	2	-	-	-	-	-
gALL_v3_c21608	1	3	-	-	-	-	-
gALL_v3_c21609	2	4	-	-	gb|EFN61969.1|	PDZ domain-containing protein 6 [Camponotus floridanus]	1,00E-15
gALL_v3_c2161	8	7	gnl|CDD|146004	pfam03166, MH2, MH2 domai	gb|ENN78137.1|	hypothetical protein YQE_05291, partial [Dendroctonus ponderosae]	8,00E-84
gALL_v3_c21612	3	7	-	-	-	-	-
gALL_v3_c21613	3	3	-	-	ref|XP_001606853.2|	PREDICTED: nucleolar protein 14 homolog [Nasonia vitripennis]	4,00E-12
gALL_v3_c21614	0	3	-	-	-	-	-
gALL_v3_c21615	4	2	-	-	-	-	-
gALL_v3_c21616	2	4	-	-	-	-	-
gALL_v3_c21617	4	2	-	-	-	-	-
gALL_v3_c21618	4	3	gnl|CDD|191014	pfam04515, Choline_transpo,	ref|XP_003484500.1|	PREDICTED: CTL-like protein 1-like [Bombus impatiens]	1,00E-24
gALL_v3_c21619	1	5	-	-	-	-	-
gALL_v3_c21620	3	1	-	-	gb|EFN65619.1|	Putative uncharacterized transposon-derived protein F52C9.6 [Camponotus floridanus]	4,00E-12
gALL_v3_c21621	2	3	-	-	-	-	-
gALL_v3_c21622	3	1	gnl|CDD|30103	cd01381, MYSc_type_VII, Myc	ref|XP_002079511.1|	GD21973 [Drosophila simulans] gb|EDX05096.1| GD21973 [Drosophila simulans]	5,00E-75
gALL_v3_c21623	2	3	-	-	-	-	-
gALL_v3_c21624	2	3	-	-	-	-	-
gALL_v3_c21625	1	5	-	-	ref|XP_001984531.1|	GH14973 [Drosophila grimshawi] gb|EDV96879.1| GH14973 [Drosophila grimshawi]	3,00E-27
gALL_v3_c21626	2	4	-	-	-	-	-
gALL_v3_c21627	2	3	-	-	-	-	-
gALL_v3_c21628	5	3	-	-	gb|EST45669.1|	Rab1a [Spironucleus salmonicida]	3,00E-12
gALL_v3_c21629	12	10	gnl|CDD|173661	cd05570, STKc_PKC, Catalytic	ref|XP_002431153.1|	protein kinase C, putative [Pediculus humanus corporis] gb|EEB18415.1| protein kinase C, putative [Pediculus humanus corporis]	4,00E-33
gALL_v3_c2163	7	10	-	-	ref|XP_003396182.1|	PREDICTED: hypothetical protein LOC100647097 [Bombus terrestris]	5,00E-27
gALL_v3_c21630	2	1	-	-	-	-	-
gALL_v3_c21631	2	1	-	-	-	-	-
gALL_v3_c21632	1	2	-	-	-	-	-
gALL_v3_c21633	3	3	-	-	ref|XP_006617553.1|	PREDICTED: protein CNPPD1-like [Apis dorsata]	1,00E-80
gALL_v3_c21634	2	6	-	-	-	-	-
gALL_v3_c21635	1	4	-	-	-	-	-
gALL_v3_c21636	2	2	gnl|CDD|181989	PRK09612, rpl2p, 50S ribosom	gb|AGM32342.1|	ribosomal protein L8 [Coptotermes formosanus]	4,00E-61
gALL_v3_c21637	2	2	-	-	-	-	-
gALL_v3_c21638	1	4	-	-	ref|XP_002431312.1|	conserved hypothetical protein [Pediculus humanus corporis] gb|EEB18574.1| conserved hypothetical protein [Pediculus humanus corporis]	2,00E-21
gALL_v3_c21639	1	3	-	-	-	-	-
gALL_v3_c2164	8	7	gnl|CDD|162028	TIGR00763, lon, ATP-depende	ref|XP_002426258.1|	predicted protein [Pediculus humanus corporis] gb|EEB13520.1| predicted protein [Pediculus humanus corporis]	1,00E-160
gALL_v3_c21640	1	3	gnl|CDD|29257	cd00200, WD40, WD40 domai	gb|EFN79387.1|	WD repeat, SAM and U-box domain-containing protein 1 [Harpegnathos saltator]	4,00E-29
gALL_v3_c21641	2	5	gnl|CDD|73156	cd01650, RT_nLTR_like, RT_nL	ref|XP_002385618.1|	LINE-1 retrotransposon-like element, putative [Aspergillus flavus NRRL3357] gb|EED44706.1| LINE-1 retrotransposon-like element, putative [Aspergillus flavus NRRL3357]	9,00E-38
gALL_v3_c21642	2	2	-	-	-	-	-
gALL_v3_c21643	6	11	-	-	-	-	-
gALL_v3_c21644	3	2	-	-	-	-	-
gALL_v3_c21645	0	4	-	-	-	-	-
gALL_v3_c21646	3	1	-	-	-	-	-
gALL_v3_c21649	1	3	-	-	-	-	-
gALL_v3_c21650	2	1	-	-	-	-	-
gALL_v3_c21651	2	3	gnl|CDD|190226	pfam02145, Rap_GAP, Rap/rar	ref|XP_001946843.2|	PREDICTED: ral GTPase-activating protein subunit alpha-1-like isoform 1 [Acyrthosiphon pisum]	6,00E-19
gALL_v3_c21652	2	2	-	-	-	-	-
gALL_v3_c21654	3	2	-	-	-	-	-
gALL_v3_c21655	2	4	-	-	-	-	-
gALL_v3_c21656	3	2	-	-	gb|ERL95037.1|	hypothetical protein D910_12307 [Dendroctonus ponderosae]	1,00E-27
gALL_v3_c21657	3	2	gnl|CDD|144327	pfam00685, Sulfotransfer_1, S	ref|XP_004525917.1|	PREDICTED: heparan sulfate glucosamine 3-O-sulfotransferase 3B1-like [Ceratitis capitata]	5,00E-88
gALL_v3_c21658	2	2	-	-	-	-	-
gALL_v3_c21659	2	3	-	-	ref|XP_006643527.1|	PREDICTED: zinc finger protein 418-like, partial [Lepisosteus oculatus]	3,00E-35
gALL_v3_c2166	8	13	-	-	ref|XP_001606953.2|	PREDICTED: hypothetical protein LOC100123328 [Nasonia vitripennis]	2,00E-23
gALL_v3_c21661	8	11	-	-	gb|EFZ21067.1|	hypothetical protein SINV_02537 [Solenopsis invicta]	5,00E-42
gALL_v3_c21662	3	1	-	-	-	-	-
gALL_v3_c21663	9	10	-	-	-	-	-
gALL_v3_c21665	3	3	-	-	ref|XP_006620393.1|	PREDICTED: fat-like cadherin-related tumor suppressor homolog, partial [Apis dorsata]	7,00E-46
gALL_v3_c21666	3	3	-	-	-	-	-
gALL_v3_c21667	1	3	-	-	-	-	-
gALL_v3_c21668	2	3	-	-	-	-	-
gALL_v3_c2167	21	31	-	-	-	-	-
gALL_v3_c21670	1	4	-	-	-	-	-
gALL_v3_c21671	1	4	-	-	-	-	-
gALL_v3_c21672	0	3	-	-	gb|EMC90702.1|	Pogo transposable element with KRAB domain, partial [Columba livia]	1,00E-16
gALL_v3_c21673	1	2	-	-	-	-	-
gALL_v3_c21674	2	2	-	-	-	-	-
gALL_v3_c21675	2	2	-	-	-	-	-
gALL_v3_c21676	0	4	-	-	-	-	-
gALL_v3_c21677	1	4	-	-	ref|XP_005469027.1|	PREDICTED: uncharacterized protein LOC102076147 [Oreochromis niloticus]	1,00E-10
gALL_v3_c21678	3	3	-	-	-	-	-
gALL_v3_c21679	2	5	-	-	-	-	-
gALL_v3_c2168	7	8	-	-	-	-	-
gALL_v3_c21680	1	2	-	-	-	-	-
gALL_v3_c21681	3	3	-	-	-	-	-
gALL_v3_c21682	3	1	-	-	ref|XP_006811856.1|	PREDICTED: RNA-directed DNA polymerase from mobile element jockey-like [Saccoglossus kowalevskii]	7,00E-23
gALL_v3_c21684	0	4	-	-	-	-	-
gALL_v3_c21685	1	4	-	-	-	-	-
gALL_v3_c21686	1	3	-	-	-	-	-
gALL_v3_c21687	4	4	-	-	ref|XP_002422551.1|	translational activator GCN1, putative [Pediculus humanus corporis] gb|EEB09813.1| translational activator GCN1, putative [Pediculus humanus corporis]	8,00E-91
gALL_v3_c21688	2	2	-	-	ref|XP_001957250.1|	GF24148 [Drosophila ananassae] gb|EDV40056.1| GF24148 [Drosophila ananassae]	6,00E-06
gALL_v3_c21689	0	3	-	-	gb|EGI67459.1|	Eukaryotic translation elongation factor 1 epsilon-1 [Acromyrmex echinatior]	1,00E-33
gALL_v3_c2169	22	19	gnl|CDD|147795	pfam05835, Synaphin, Synaph	gb|EHJ64094.1|	complexin [Danaus plexippus]	5,00E-08
gALL_v3_c21690	2	5	-	-	-	-	-

gALL_v3_c21691	3	2	gnl|CDD|128386	smart00072, GuKc, Guanylate	gb|EGI66768.1|	MAGUK p55 subfamily member 7 [Acromyrmex echinatior]	1,00E-102
gALL_v3_c21692	1	5	-	-	gb|EEZ97322.1|	hypothetical protein TcasGA2_TC011133 [Tribolium castaneum]	4,00E-67
gALL_v3_c21693	3	2	-	-	-	-	-
gALL_v3_c21694	1	3	gnl|CDD|144562	pfam01019, G_glu_transpept,	gb|EFA08906.1|	hypothetical protein TcasGA2_TC006611 [Tribolium castaneum]	7,00E-23
gALL_v3_c21695	3	2	-	-	ref|XP_002426916.1|	conserved hypothetical protein [Pediculus humanus corporis] gb|EEB14178.1| conserved hypothetical protein [Pediculus humanus corporis]	9,00E-08
gALL_v3_c21696	3	3	gnl|CDD|176336	cd01260, PH_CNK, Connector	gb|EFZ18054.1|	hypothetical protein SINV_14611 [Solenopsis invicta]	2,00E-40
gALL_v3_c21697	2	2	-	-	gb|EFN87173.1|	Codanin-1 [Harpegnathos saltator]	2,00E-11
gALL_v3_c21698	0	5	-	-	-	-	-
gALL_v3_c21699	4	2	-	-	ref|XP_003700160.1|	PREDICTED: heterogeneous nuclear ribonucleoprotein K-like [Megachile rotundata]	8,00E-06
gALL_v3_c217	20	25	-	-	-	-	-
gALL_v3_c2170	4	6	gnl|CDD|178488	PLN02900, PLN02900, alanyl-t	gb|ACY43283.1|	ala-tRNA synthetase [Periplaneta americana]	1,00E-104
gALL_v3_c21700	3	1	gnl|CDD|189955	pfam01363, FYVE, FYVE zinc fi	ref|XP_002404404.1|	ankyrin repeat containing protein [Ixodes scapularis] gb|EEC09892.1| ankyrin repeat containing protein [Ixodes scapularis]	4,00E-74
gALL_v3_c21701	1	3	-	-	-	-	-
gALL_v3_c21702	3	1	-	-	-	-	-
gALL_v3_c21703	0	5	-	-	-	-	-
gALL_v3_c21704	2	4	-	-	gb|EEZ98056.1|	hypothetical protein TcasGA2_TC000461 [Tribolium castaneum]	3,00E-56
gALL_v3_c21705	3	5	-	-	-	-	-
gALL_v3_c21706	1	3	-	-	-	-	-
gALL_v3_c21707	6	6	gnl|CDD|34668	COG5064, SRP1, Karyopherin (	ref|XP_968505.1|	PREDICTED: similar to karyopherin alpha 6 [Tribolium castaneum] gb|EEZ98471.1| hypothetical protein TcasGA2_TC000963 [Tribolium castaneum]	0
gALL_v3_c21708	4	2	-	-	gb|ENN77019.1|	hypothetical protein YQE_06513, partial [Dendroctonus ponderosae]	5,00E-24
gALL_v3_c21709	2	2	-	-	-	-	-
gALL_v3_c2171	5	13	-	-	-	-	-
gALL_v3_c21710	1	3	-	-	-	-	-
gALL_v3_c21711	5	4	-	-	-	-	-
gALL_v3_c21713	3	4	-	-	ref|XP_972169.2|	PREDICTED: similar to Calcium activated protein for secretion CG33653-PB [Tribolium castaneum]	1,00E-151
gALL_v3_c21716	11	6	gnl|CDD|190253	pfam02230, Abhydrolase_2, P	ref|XP_001599074.1|	PREDICTED: acyl-protein thioesterase 2-like [Nasonia vitripennis]	1,00E-110
gALL_v3_c21717	1	4	-	-	gb|AGM32283.1|	sterol desaturase, partial [Coptotermes formosanus]	5,00E-88
gALL_v3_c21718	1	3	-	-	-	-	-
gALL_v3_c21719	2	4	-	-	-	-	-
gALL_v3_c2172	5	6	gnl|CDD|133181	cd05050, PTKc_Musk, Catalyti	ref|XP_001942959.2|	PREDICTED: tyrosine-protein kinase transmembrane receptor Ror2-like [Acyrthosiphon pisum]	2,00E-58
gALL_v3_c21720	3	1	gnl|CDD|149861	pfam08925, DUF1907, Domain	ref|XP_968861.1|	PREDICTED: similar to CG9119 CG9119-PA [Tribolium castaneum]	5,00E-42
gALL_v3_c21721	2	4	-	-	-	-	-
gALL_v3_c21722	1	3	-	-	-	-	-
gALL_v3_c21723	3	5	gnl|CDD|191332	pfam05648, PEX11, Peroxisom	ref|XP_003700982.1|	PREDICTED: peroxisomal membrane protein 11B-like [Megachile rotundata]	4,00E-60
gALL_v3_c21724	0	5	-	-	-	-	-
gALL_v3_c21727	0	3	gnl|CDD|128409	smart00098, alkPPc, Alkaline p	gb|EHJ73485.1|	alkaline phosphatase [Danaus plexippus]	4,00E-18
gALL_v3_c21728	0	3	-	-	-	-	-
gALL_v3_c21729	1	3	gnl|CDD|189555	pfam00457, Glyco_hydro_11,	dbj|BAH56524.1|	endo-beta-1,4-xylanase [Holomastigotoides mirabile]	6,00E-84
gALL_v3_c2173	4	5	-	-	gb|EFX86104.1|	hypothetical protein DAPPUDRAFT_230485 [Daphnia pulex]	4,00E-09
gALL_v3_c21730	4	2	-	-	-	-	-
gALL_v3_c21731	1	5	-	-	-	-	-
gALL_v3_c21732	1	6	-	-	-	-	-
gALL_v3_c21733	3	2	-	-	ref|XP_003248246.1|	PREDICTED: dedicator of cytokinesis protein 1-like isoform 3 [Acyrthosiphon pisum]	4,00E-89
gALL_v3_c21734	6	4	-	-	-	-	-
gALL_v3_c21735	1	3	-	-	-	-	-
gALL_v3_c21736	3	2	-	-	-	-	-
gALL_v3_c21737	2	5	-	-	-	-	-
gALL_v3_c21738	2	2	-	-	ref|XP_003707323.1|	PREDICTED: uncharacterized protein LOC100876258 [Megachile rotundata]	2,00E-59
gALL_v3_c2174	22	27	gnl|CDD|30099	cd01377, MYSc_type_II, Myos	ref|XP_003701010.1|	PREDICTED: myosin heavy chain, non-muscle-like [Megachile rotundata]	0
gALL_v3_c21741	2	2	gnl|CDD|28913	cd00031, CA, Cadherin repeat	gb|EFN87109.1|	Putative fat-like cadherin-related tumor suppressor-like protein [Harpegnathos saltator]	1,00E-81
gALL_v3_c21742	1	3	-	-	-	-	-
gALL_v3_c21743	3	1	-	-	ref|XP_006610246.1|	PREDICTED: protein kibra-like isoform X3 [Apis dorsata]	2,00E-50
gALL_v3_c21744	1	5	-	-	ref|XP_003705153.1|	PREDICTED: dnaJ homolog subfamily C member 13 [Megachile rotundata]	1,00E-47
gALL_v3_c21745	4	3	-	-	gb|ADI61823.1|	endonuclease-reverse transcriptase [Bombyx mori]	1,00E-30
gALL_v3_c21746	0	4	-	-	-	-	-
gALL_v3_c21747	2	4	-	-	-	-	-
gALL_v3_c21748	3	3	gnl|CDD|128797	smart00524, DWB, Domain B i	ref|XP_001944190.2|	PREDICTED: hypothetical protein LOC100165611 [Acyrthosiphon pisum]	2,00E-99
gALL_v3_c2175	14	13	gnl|CDD|181474	PRK08566, PRK08566, DNA-di	dbj|BAJ78666.1|	RNA polymerase II largest subunit [Tenodera aridifolia]	0
gALL_v3_c21750	3	1	-	-	ref|XP_004924423.1|	PREDICTED: SH2 domain-containing protein 4B-like [Bombyx mori]	3,00E-21
gALL_v3_c21752	0	4	gnl|CDD|189625	pfam00609, DAGK_acc, Diacyl	gb|EFN67260.1|	Diacylglycerol kinase epsilon [Camponotus floridanus]	4,00E-52
gALL_v3_c21753	0	4	-	-	-	-	-
gALL_v3_c21755	1	4	-	-	-	-	-
gALL_v3_c21756	0	4	-	-	gb|ERL96034.1|	hypothetical protein D910_00851 [Dendroctonus ponderosae]	2,00E-06
gALL_v3_c21757	0	4	-	-	-	-	-
gALL_v3_c21758	3	4	-	-	-	-	-
gALL_v3_c21759	2	3	-	-	ref|XP_001650151.1|	hypothetical protein AaeL_AAEL014972 [Aedes aegypti] gb|EAT32796.1| AAEL014972-PA [Aedes aegypti]	3,00E-23
gALL_v3_c2176	19	22	gnl|CDD|190537	pfam03137, OATP, Organic Ani	ref|XP_001606202.2|	PREDICTED: solute carrier organic anion transporter family member 5A1-like [Nasonia vitripennis]	0
gALL_v3_c21760	1	8	gnl|CDD|128871	smart00608, ACR, ADAM Cyste	gb|ENN78027.1|	hypothetical protein YQE_05512, partial [Dendroctonus ponderosae]	1,00E-106
gALL_v3_c21761	2	2	-	-	-	-	-
gALL_v3_c21764	4	2	-	-	-	-	-
gALL_v3_c21765	5	4	-	-	gb|EFA12422.1|	hypothetical protein TcasGA2_TC004414 [Tribolium castaneum]	0
gALL_v3_c21766	0	3	-	-	-	-	-
gALL_v3_c21767	9	7	gnl|CDD|189411	pfam00135, COesterase, Carbo	ref|XP_968215.1|	PREDICTED: similar to putative esterase [Tribolium castaneum] gb|EFA05148.1| hypothetical protein TcasGA2_TC015265 [Tribolium castaneum]	5,00E-44
gALL_v3_c21768	3	11	-	-	-	-	-
gALL_v3_c21769	0	3	-	-	-	-	-
gALL_v3_c21770	0	4	-	-	-	-	-
gALL_v3_c21771	4	7	gnl|CDD|189672	pfam00690, Cation_ATPase_N	gb|EFA10430.1|	hypothetical protein TcasGA2_TC012671 [Tribolium castaneum]	1,00E-29
gALL_v3_c21773	2	1	-	-	-	-	-
gALL_v3_c21774	3	4	-	-	ref|XP_003394686.1|	PREDICTED: lipoyltransferase 1, mitochondrial-like [Bombus terrestris]	4,00E-48
gALL_v3_c21775	1	2	-	-	-	-	-
gALL_v3_c21776	4	4	gnl|CDD|189450	pfam00209, SNF, Sodium:neur	ref|XP_001842965.1|	sodium- and chloride-dependent neurotransmitter transporter [Culex quinquefasciatus] gb|EDS29354.1| sodium- and chloride-dependent neurotransmitter transporter [C	5,00E-85
gALL_v3_c21777	2	6	-	-	-	-	-
gALL_v3_c21778	4	3	gnl|CDD|131299	TIGR02245, HAD_IIID1, HAD-s	gb|EKC25887.1|	Ubiquitin-like domain-containing CTD phosphatase 1 [Crassostrea gigas]	3,00E-39
gALL_v3_c21779	3	4	-	-	gb|EGI66077.1|	Putative nucleoporin Nup58 [Acromyrmex echinatior]	9,00E-81
gALL_v3_c21780	2	3	-	-	-	-	-
gALL_v3_c21781	3	1	gnl|CDD|148437	pfam06825, HSBP1, Heat shoc	ref|NP_001129658.1|	heat shock factor binding protein 1-like [Nasonia vitripennis]	2,00E-23
gALL_v3_c21782	2	2	-	-	emb|CAM36420.1|	hypothetical protein [Thermobia domestica]	1,00E-12
gALL_v3_c21783	3	1	-	-	-	-	-
gALL_v3_c21784	4	2	gnl|CDD|73368	cd02801, DUS_like_FMN, Dihy	ref|XP_002423579.1|	tRNA-dihydrouridine synthase, putative [Pediculus humanus corporis] gb|EEB10841.1| tRNA-dihydrouridine synthase, putative [Pediculus humanus corporis]	6,00E-80
gALL_v3_c21785	3	1	gnl|CDD|189372	pfam00067, p450, Cytochrom	gb|EFN78521.1|	Cytochrome P450 302a1, mitochondrial [Harpegnathos saltator]	3,00E-46
gALL_v3_c21786	3	5	-	-	-	-	-
gALL_v3_c21787	0	5	-	-	-	-	-
gALL_v3_c21788	7	4	-	-	-	-	-
gALL_v3_c21789	2	10	-	-	gb|EFA03940.1|	hypothetical protein TcasGA2_TC014081 [Tribolium castaneum]	1,00E-74
gALL_v3_c2179	14	27	gnl|CDD|189860	pfam01151, ELO, GNS1/SUR4	ref|XP_003401825.1|	PREDICTED: elongation of very long chain fatty acids protein 6-like [Bombus terrestris] ref|XP_003490277.1| PREDICTED: elongation of very long chain fatty acids protein 6	1,00E-136
gALL_v3_c21790	3	1	-	-	-	-	-
gALL_v3_c21791	1	3	-	-	-	-	-
gALL_v3_c21792	1	3	-	-	-	-	-
gALL_v3_c21793	3	2	-	-	-	-	-
gALL_v3_c21794	2	1	-	-	-	-	-
gALL_v3_c21795	1	4	-	-	-	-	-
gALL_v3_c21796	3	5	-	-	-	-	-
gALL_v3_c21797	1	5	gnl|CDD|152638	pfam12203, HDAC4_Gln, Gluta	-	-	-
gALL_v3_c21798	2	3	-	-	-	-	-
gALL_v3_c21799	1	3	-	-	-	-	-
gALL_v3_c218	8	9	gnl|CDD|150071	pfam09279, efhand_like, Phos	ref|XP_003742513.1|	PREDICTED: 1-phosphatidylinositol 4,5-bisphosphate phosphodiesterase delta-4-like [Metaseiulus occidentalis]	1,00E-69
gALL_v3_c2180	4	8	-	-	gb|EFA13301.1|	hypothetical protein TcasGA2_TC014831 [Tribolium castaneum]	4,00E-34
gALL_v3_c21802	1	5	gnl|CDD|183675	PRK12678, PRK12678, transcri	ref|XP_001600207.2|	PREDICTED: LOW QUALITY PROTEIN: eukaryotic translation initiation factor 4B [Nasonia vitripennis]	2,00E-07
gALL_v3_c21803	2	2	gnl|CDD|190023	pfam01529, zf-DHHC, DHHC zi	ref|XP_003490598.1|	PREDICTED: probable palmitoyltransferase ZDHHC16-like [Bombus impatiens]	1,00E-103
gALL_v3_c21805	3	7	-	-	-	-	-
gALL_v3_c21806	3	4	-	-	-	-	-
gALL_v3_c21807	3	1	-	-	-	-	-
gALL_v3_c21808	2	1	-	-	-	-	-
gALL_v3_c21809	5	12	gnl|CDD|48460	cd03762, proteasome_beta_ty	gb|AGM32385.1|	proteasome subunit beta type-6 [Coptotermes formosanus]	1,00E-100
gALL_v3_c2181	8	6	-	-	-	-	-
gALL_v3_c21810	4	6	-	-	-	-	-
gALL_v3_c21812	2	4	-	-	-	-	-
gALL_v3_c21813	0	3	-	-	-	-	-
gALL_v3_c21814	3	1	-	-	-	-	-
gALL_v3_c21815	1	5	-	-	-	-	-
gALL_v3_c21817	8	8	gnl|CDD|178917	PRK00179, pgi, glucose-6-phos	gb|ADB11228.1|	phosphoglucose isomerase [Melitaea phoebe]	1,00E-147
gALL_v3_c21818	2	2	-	-	-	-	-
gALL_v3_c21820	3	3	-	-	gb|EFA09839.1|	hypothetical protein TcasGA2_TC011986 [Tribolium castaneum]	1,00E-129
gALL_v3_c21821	2	2	-	-	-	-	-
gALL_v3_c21823	4	3	-	-	-	-	-
gALL_v3_c21824	0	4	-	-	-	-	-
gALL_v3_c21826	4	7	-	-	-	-	-
gALL_v3_c21828	10	25	-	-	-	-	-
gALL_v3_c21829	12	9	-	-	-	-	-
gALL_v3_c2183	7	4	-	-	gb|AAB39926.1|	leucomyosuppressin precursor [Diploptera punctata]	9,00E-38
gALL_v3_c21830	1	3	-	-	-	-	-
gALL_v3_c21833	0	4	-	-	-	-	-
gALL_v3_c21835	1	3	-	-	gb|EFZ19881.1|	hypothetical protein SINV_12618 [Solenopsis invicta]	1,00E-17
gALL_v3_c21836	3	1	-	-	gb|EFX84793.1|	hypothetical protein DAPPUDRAFT_314322 [Daphnia pulex]	7,00E-32
gALL_v3_c21837	1	4	-	-	-	-	-
gALL_v3_c21838	0	5	-	-	-	-	-
gALL_v3_c21840	4	3	gnl|CDD|178560	PLN02978, PLN02978, pyridox	ref|XP_972953.1|	PREDICTED: similar to pyridoxal kinase [Tribolium castaneum] gb|EFA00408.1| hypothetical protein TcasGA2_TC003259 [Tribolium castaneum]	1,00E-111
gALL_v3_c21841	2	2	-	-	ref|XP_968039.1|	PREDICTED: similar to CG14182 CG14182-PA [Tribolium castaneum] gb|EFA00462.1| hypothetical protein TcasGA2_TC003319 [Tribolium castaneum]	2,00E-50
gALL_v3_c21842	5	2	-	-	-	-	-
gALL_v3_c21843	1	3	-	-	-	-	-
gALL_v3_c21844	0	3	-	-	-	-	-
gALL_v3_c21845	1	3	-	-	-	-	-
gALL_v3_c21846	2	2	-	-	-	-	-
gALL_v3_c21848	4	9	gnl|CDD|189372	pfam00067, p450, Cytochrom	dbj|BAD84176.1|	cytochrome P450 [Hodotermopsis sjostedti]	1,00E-104

gALL_v3_c21849	8	5	-	-	-	-	-
gALL_v3_c2185	17	20	gnl|CDD|189008	cd09601, M1_APN_2, Peptida	ref|XP_006620201.1|	PREDICTED: puromycin-sensitive aminopeptidase-like isoform X3 [Apis dorsata]	0
gALL_v3_c21850	2	2	-	-	ref|XP_003493292.1|	PREDICTED: 28S ribosomal protein S35, mitochondrial-like [Bombus impatiens]	2,00E-12
gALL_v3_c21851	2	3	-	-	ref|XP_001602592.2|	PREDICTED: hypothetical protein LOC100118685 [Nasonia vitripennis]	1,00E-105
gALL_v3_c21852	3	2	-	-	-	-	-
gALL_v3_c21853	2	1	-	-	-	-	-
gALL_v3_c21854	2	3	gnl|CDD|191860	pfam07814, WAPL, Wings apa	ref|XP_974865.2|	PREDICTED: similar to AAEL002532-PA [Tribolium castaneum]	1,00E-113
gALL_v3_c21855	4	2	-	-	-	-	-
gALL_v3_c21856	5	10	-	-	-	-	-
gALL_v3_c21857	2	4	-	-	-	-	-
gALL_v3_c21858	1	4	-	-	-	-	-
gALL_v3_c2186	3	7	-	-	ref|XP_002431492.1|	reverse transcriptase, putative [Pediculus humanus corporis] gb|EEB18754.1| reverse transcriptase, putative [Pediculus humanus corporis]	6,00E-08
gALL_v3_c21860	4	2	-	-	ref|XP_002433230.1|	type II transmembrane protein, putative [Pediculus humanus corporis] gb|EEB20492.1| type II transmembrane protein, putative [Pediculus humanus corporis]	1,00E-107
gALL_v3_c21861	5	5	gnl|CDD|189772	pfam00927, Transglut_C, Trans	sp|P52183.1|ANNU_SCI	RecName: Full=Annulin; AltName: Full=Protein-glutamine gamma-glutamyltransferase; AltName: Full=Transglutaminase gb|AAA29806.1| annulin [Schistocerca americana]	5,00E-45
gALL_v3_c21862	2	3	-	-	gb|AEE61890.1|	unknown [Dendroctonus ponderosae]	5,00E-52
gALL_v3_c21863	1	4	-	-	-	-	-
gALL_v3_c21864	2	4	gnl|CDD|178854	PRK00089, era, GTPase Era; Re	gb|AGM32835.1|	putative GTP-binding protein Era [Coptotermes formosanus]	7,00E-53
gALL_v3_c21865	1	5	-	-	-	-	-
gALL_v3_c21866	2	4	-	-	-	-	-
gALL_v3_c21867	2	3	-	-	-	-	-
gALL_v3_c21868	3	4	-	-	ref|XP_005347796.1|	PREDICTED: probable E3 ubiquitin-protein ligase HERC1 isoform X1 [Microtus ochrogaster] ref|XP_005347797.1| PREDICTED: probable E3 ubiquitin-protein ligase HERC1 is	1,00E-20
gALL_v3_c21869	2	2	-	-	-	-	-
gALL_v3_c21870	2	2	-	-	-	-	-
gALL_v3_c21872	0	5	gnl|CDD|188713	cd09327, LIM1_abLIM, The fir	ref|XP_002429421.1|	ablim, putative [Pediculus humanus corporis] gb|EEB16683.1| ablim, putative [Pediculus humanus corporis]	7,00E-35
gALL_v3_c21874	1	3	gnl|CDD|147949	pfam06068, TIP49, TIP49 C-ter	ref|XP_006557811.1|	PREDICTED: RuvB-like 2 isoform X1 [Apis mellifera] ref|XP_006557812.1| PREDICTED: RuvB-like 2 isoform X2 [Apis mellifera] ref|XP_006557813.1| PREDICTED: RuvB-like 2	1,00E-93
gALL_v3_c21876	0	4	-	-	-	-	-
gALL_v3_c21877	1	3	-	-	ref|XP_001649449.1|	small calcium-binding mitochondrial carrier, putative [Aedes aegypti] gb|EAT44005.1| AAEL004589-PA [Aedes aegypti]	3,00E-17
gALL_v3_c21878	3	5	-	-	-	-	-
gALL_v3_c21879	3	7	-	-	gb|EFN62471.1|	Bullous pemphigoid antigen 1, isoforms 6/9/10 [Camponotus floridanus]	4,00E-14
gALL_v3_c2188	5	4	gnl|CDD|176085	cd08535, SAM_PNT-Tel_Yan, S	ref|XP_975017.2|	PREDICTED: similar to ets [Tribolium castaneum]	6,00E-52
gALL_v3_c21882	0	3	-	-	-	-	-
gALL_v3_c21884	0	3	-	-	gb|ELT96366.1|	hypothetical protein CAPTEDRAFT_217368, partial [Capitella teleta]	4,00E-10
gALL_v3_c21885	6	7	gnl|CDD|192240	pfam09273, Rubis-subs-bind, I	ref|XP_006609970.1|	PREDICTED: histone-lysine N-methyltransferase setd3-like isoform X1 [Apis dorsata]	1,00E-119
gALL_v3_c21886	0	5	-	-	-	-	-
gALL_v3_c21887	0	4	-	-	-	-	-
gALL_v3_c21888	1	5	-	-	ref|XP_002431700.1|	myosin light chain kinase, putative [Pediculus humanus corporis] gb|EEB18962.1| myosin light chain kinase, putative [Pediculus humanus corporis]	3,00E-22
gALL_v3_c21889	3	1	-	-	-	-	-
gALL_v3_c21890	2	4	gnl|CDD|143524	cd06966, NR_DBD_CAR, DNA-	gb|EFN64539.1|	Nuclear hormone receptor HR96 [Camponotus floridanus]	2,00E-45
gALL_v3_c21891	4	8	-	-	gb|EFA08977.1|	hypothetical protein TcasGA2_TC006684 [Tribolium castaneum]	8,00E-27
gALL_v3_c21893	2	4	-	-	-	-	-
gALL_v3_c21894	5	3	-	-	gb|ELU12162.1|	hypothetical protein CAPTEDRAFT_19526 [Capitella teleta]	3,00E-24
gALL_v3_c21895	9	18	-	-	gb|AGM32467.1|	pleiotrophin-like protein [Coptotermes formosanus]	1,00E-35
gALL_v3_c21896	0	6	-	-	-	-	-
gALL_v3_c21897	3	4	-	-	-	-	-
gALL_v3_c21898	0	6	-	-	-	-	-
gALL_v3_c21899	2	2	-	-	-	-	-
gALL_v3_c219	9	12	-	-	-	-	-
gALL_v3_c2190	13	15	gnl|CDD|133134	cd06603, GH31_GANC_GANAI	ref|XP_003492162.1|	PREDICTED: neutral alpha-glucosidase AB-like [Bombus impatiens]	0
gALL_v3_c21900	2	3	-	-	-	-	-
gALL_v3_c21902	2	6	-	-	gb|ESN93095.1|	hypothetical protein HELRODRAFT_165255 [Helobdella robusta]	3,00E-38
gALL_v3_c21903	3	1	-	-	-	-	-
gALL_v3_c21904	1	4	-	-	-	-	-
gALL_v3_c21905	1	5	-	-	ref|XP_003399245.1|	PREDICTED: LOW QUALITY PROTEIN: gem-associated protein 5-like [Bombus terrestris]	7,00E-49
gALL_v3_c21906	1	6	-	-	-	-	-
gALL_v3_c21907	1	5	-	-	-	-	-
gALL_v3_c21908	2	6	gnl|CDD|189368	pfam00060, Lig_chan, Ligand-|	ref|XP_002431269.1|	glutamate receptor, ionotropic kainate 2 precursor, putative [Pediculus humanus corporis] gb|EEB18531.1| glutamate receptor, ionotropic kainate 2 precursor, putative [Pe	1,00E-106
gALL_v3_c21909	2	1	-	-	-	-	-
gALL_v3_c21910	5	6	-	-	-	-	-
gALL_v3_c21911	3	2	-	-	-	-	-
gALL_v3_c21912	1	3	-	-	gb|EFN65619.1|	Putative uncharacterized transposon-derived protein F52C9.6 [Camponotus floridanus]	1,00E-10
gALL_v3_c21916	2	5	-	-	-	-	-
gALL_v3_c21917	4	5	-	-	-	-	-
gALL_v3_c21918	2	6	-	-	-	-	-
gALL_v3_c21919	1	5	-	-	-	-	-
gALL_v3_c2192	18	33	-	-	-	-	-
gALL_v3_c21920	3	5	-	-	-	-	-
gALL_v3_c21921	2	2	-	-	-	-	-
gALL_v3_c21923	4	2	-	-	-	-	-
gALL_v3_c21924	2	1	-	-	-	-	-
gALL_v3_c21925	2	2	-	-	-	-	-
gALL_v3_c21926	2	5	-	-	-	-	-
gALL_v3_c21927	2	3	-	-	-	-	-
gALL_v3_c21928	2	2	gnl|CDD|128886	smart00638, LPD_N, Lipoprote	gb|AHJ78589.1|	dLp/HDL-BGBP precursor [Pontastacus leptodactylus]	3,00E-19
gALL_v3_c21929	1	3	-	-	-	-	-
gALL_v3_c2193	16	27	-	-	-	-	-
gALL_v3_c21930	3	2	-	-	-	-	-
gALL_v3_c21931	2	5	gnl|CDD|28959	cd00078, HECTc, HECT domain	ref|XP_001813187.1|	PREDICTED: similar to hect E3 ubiquitin ligase [Tribolium castaneum]	2,00E-78
gALL_v3_c21932	2	6	gnl|CDD|128627	smart00332, PP2Cc, Serine/th	ref|XP_006613631.1|	PREDICTED: protein phosphatase 1B-like isoform X1 [Apis dorsata]	1,00E-130
gALL_v3_c21933	1	2	-	-	-	-	-
gALL_v3_c21934	4	3	-	-	-	-	-
gALL_v3_c21935	1	3	-	-	-	-	-
gALL_v3_c21936	3	1	gnl|CDD|173754	cd07865, STKc_CDK9, Catalyti	ref|XP_002430819.1|	mitogen-activated protein kinase ERK-A, putative [Pediculus humanus corporis] gb|EEB18081.1| mitogen-activated protein kinase ERK-A, putative [Pediculus humanus cor	1,00E-47
gALL_v3_c21937	2	6	-	-	gb|EHJ68065.1|	hypothetical protein KGM_01227 [Danaus plexippus]	2,00E-29
gALL_v3_c21938	3	3	-	-	-	-	-
gALL_v3_c2194	14	19	-	-	-	-	-
gALL_v3_c21940	3	2	gnl|CDD|30669	COG0321, LipB, Lipoate-protei	gb|EEZ98826.1|	hypothetical protein TcasGA2_TC004433 [Tribolium castaneum]	3,00E-40
gALL_v3_c21942	3	2	-	-	ref|XP_396152.4|	PREDICTED: sphingomyelin synthase-related 1 [Apis mellifera]	2,00E-29
gALL_v3_c21943	5	4	-	-	-	-	-
gALL_v3_c21944	0	3	-	-	-	-	-
gALL_v3_c21945	2	2	-	-	gb|EFA11911.1|	hypothetical protein TcasGA2_TC004068 [Tribolium castaneum]	2,00E-24
gALL_v3_c21946	1	2	-	-	-	-	-
gALL_v3_c21947	3	1	-	-	ref|XP_002410415.1|	conserved hypothetical protein [Ixodes scapularis] gb|EEC11125.1| conserved hypothetical protein [Ixodes scapularis]	2,00E-41
gALL_v3_c21948	2	2	-	-	-	-	-
gALL_v3_c21949	2	1	-	-	-	-	-
gALL_v3_c21951	6	4	-	-	-	-	-
gALL_v3_c21952	1	3	-	-	-	-	-
gALL_v3_c21953	4	2	-	-	-	-	-
gALL_v3_c21955	2	3	-	-	-	-	-
gALL_v3_c21956	3	6	gnl|CDD|176373	cd01778, RASSF1_RA, Ubiquiti	ref|XP_002404761.1|	rassf1, putative [Ixodes scapularis] gb|EEC09975.1| rassf1, putative [Ixodes scapularis]	2,00E-66
gALL_v3_c21958	0	4	-	-	gb|EKC39344.1|	Transforming growth factor-beta receptor-associated protein 1 [Crassostrea gigas]	4,00E-49
gALL_v3_c21959	3	2	-	-	-	-	-
gALL_v3_c21963	3	2	gnl|CDD|149272	pfam08122, NDUF_B12, NADH	gb|ENN77980.1|	hypothetical protein YQE_05656, partial [Dendroctonus ponderosae] gb|ERL85964.1| hypothetical protein D910_03379 [Dendroctonus ponderosae]	2,00E-31
gALL_v3_c21964	2	2	-	-	-	-	-
gALL_v3_c21965	3	2	-	-	-	-	-
gALL_v3_c21966	3	2	gnl|CDD|190676	pfam03567, Sulfotransfer_2, S	ref|XP_321686.5|	AGAP001444-PA [Anopheles gambiae str. PEST] gb|EAA01731.5| AGAP001444-PA [Anopheles gambiae str. PEST]	1,00E-98
gALL_v3_c21968	2	3	-	-	-	-	-
gALL_v3_c2197	4	9	-	-	-	-	-
gALL_v3_c21970	1	4	-	-	-	-	-
gALL_v3_c21971	5	6	-	-	-	-	-
gALL_v3_c21972	3	3	-	-	-	-	-
gALL_v3_c21973	4	3	-	-	-	-	-
gALL_v3_c21974	5	2	gnl|CDD|150447	pfam09778, Guanylate_cyc_2,	ref|XP_967590.1|	PREDICTED: similar to CG13760 CG13760-PB [Tribolium castaneum] gb|EFA05371.1| hypothetical protein TcasGA2_TC015537 [Tribolium castaneum]	9,00E-85
gALL_v3_c21975	3	8	-	-	dbj|BAM18338.1|	similar to CG7759 [Papilio xuthus]	1,00E-25
gALL_v3_c21976	0	4	-	-	ref|XP_002943925.2|	PREDICTED: pogo transposable element with KRAB domain-like [Xenopus (Silurana) tropicalis]	2,00E-14
gALL_v3_c21977	0	4	-	-	-	-	-
gALL_v3_c21979	1	5	-	-	-	-	-
gALL_v3_c2198	4	6	-	-	-	-	-
gALL_v3_c21980	4	9	-	-	-	-	-
gALL_v3_c21981	1	3	-	-	-	-	-
gALL_v3_c21982	3	4	gnl|CDD|189641	pfam00628, PHD, PHD-finger.	ref|XP_001121700.2|	PREDICTED: transcription initiation factor TFIID subunit 3-like [Apis mellifera]	3,00E-28
gALL_v3_c21985	3	2	-	-	-	-	-
gALL_v3_c21986	0	4	-	-	-	-	-
gALL_v3_c21987	2	2	-	-	-	-	-
gALL_v3_c21988	2	2	-	-	-	-	-
gALL_v3_c2199	5	15	-	-	-	-	-
gALL_v3_c21990	0	4	-	-	-	-	-
gALL_v3_c21991	3	1	-	-	ref|XP_003766809.1|	PREDICTED: tigger transposable element-derived protein 1-like [Sarcophilus harrisii]	2,00E-09
gALL_v3_c21992	6	3	-	-	ref|XP_003242853.1|	PREDICTED: zinc finger protein 62 homolog [Acyrthosiphon pisum]	2,00E-25
gALL_v3_c21993	0	3	-	-	-	-	-
gALL_v3_c21994	2	2	-	-	-	-	-
gALL_v3_c21995	2	2	-	-	-	-	-
gALL_v3_c21997	2	8	-	-	gb|EFZ19031.1|	hypothetical protein SINV_07059 [Solenopsis invicta]	5,00E-37
gALL_v3_c21998	3	1	-	-	-	-	-
gALL_v3_c21999	3	3	-	-	-	-	-
gALL_v3_c22	24	28	-	-	ref|XP_004212194.1|	PREDICTED: uncharacterized protein LOC101240998 [Hydra vulgaris]	1,00E-10
gALL_v3_c2200	2	10	-	-	dbj|BAH71856.1|	ACYPI004534 [Acyrthosiphon pisum]	1,00E-26
gALL_v3_c22000	2	3	-	-	gb|EEZ99246.1|	hypothetical protein TcasGA2_TC000871 [Tribolium castaneum]	1,00E-81
gALL_v3_c22001	0	4	-	-	-	-	-
gALL_v3_c22004	2	2	-	-	-	-	-
gALL_v3_c22005	0	6	-	-	-	-	-
gALL_v3_c22006	4	2	-	-	-	-	-
gALL_v3_c22007	5	2	gnl|CDD|147490	pfam05334, DUF719, Protein c	gb|EFZ16348.1|	hypothetical protein SINV_08769 [Solenopsis invicta]	5,00E-43
gALL_v3_c22008	1	4	-	-	-	-	-
gALL_v3_c22009	1	3	-	-	-	-	-

gALL_v3_c2201	14	19	gnl|CDD|173697	cd05606, STKc_beta_ARK, Cat	ref|XP_002422801.1|	cAMP-dependent protein kinase catalytic subunit, putative [Pediculus humanus corporis] gb|EEB10063.1| cAMP-dependent protein kinase catalytic subunit, putative [Pedi	0
gALL_v3_c22010	2	2	-	-	-	-	-
gALL_v3_c22011	1	4	gnl|CDD|145920	pfam03029, ATP_bind_1, Cons	ref|XP_003699390.1|	PREDICTED: GPN-loop GTPase 3-like [Megachile rotundata]	8,00E-78
gALL_v3_c22012	2	3	-	-	-	-	-
gALL_v3_c22013	1	3	gnl|CDD|187643	cd08939, KDSR-like_SDR_c, 3-	ref|XP_003703294.1|	PREDICTED: 3-ketodihydrosphingosine reductase-like [Megachile rotundata]	3,00E-39
gALL_v3_c22017	0	3	-	-	-	-	-
gALL_v3_c22018	2	7	gnl|CDD|100104	cd00590, RRM, RRM (RNA rec	gb|EFZ18035.1|	hypothetical protein SINV_11488 [Solenopsis invicta]	7,00E-87
gALL_v3_c22019	5	2	-	-	-	-	-
gALL_v3_c2202	1	8	gnl|CDD|190318	pfam02469, Fasciclin, Fasciclin	sp|P10675.1|FAS1_SCH	RecName: Full=Fasciclin-1; AltName: Full=Fasciclin I; Short=FAS I; Short=FCN; Flags: Precursor gb|AAA29809.1| FCN I precursor [Schistocerca americana]	6,00E-67
gALL_v3_c22020	2	3	-	-	gb|EFZ19928.1|	hypothetical protein SINV_08896 [Solenopsis invicta]	9,00E-11
gALL_v3_c22021	0	4	-	-	-	-	-
gALL_v3_c22022	4	5	-	-	-	-	-
gALL_v3_c22024	1	6	-	-	-	-	-
gALL_v3_c22025	2	2	-	-	ref|XP_005185081.1|	PREDICTED: leucine-rich PPR motif-containing protein, mitochondrial-like [Musca domestica]	3,00E-31
gALL_v3_c22026	2	2	-	-	-	-	-
gALL_v3_c22027	1	7	-	-	-	-	-
gALL_v3_c22028	0	3	-	-	-	-	-
gALL_v3_c22029	1	4	-	-	-	-	-
gALL_v3_c2203	8	9	gnl|CDD|173964	cd07981, TAF12, TATA Binding	ref|XP_001653211.1|	transcription initiation factor TFIID subunit 12 [Aedes aegypti] ref|XP_001653214.1| transcription initiation factor TFIID subunit 12 [Aedes aegypti] gb|EAT39861.1| AAEL0	1,00E-51
gALL_v3_c22031	2	2	-	-	-	-	-
gALL_v3_c22032	2	4	gnl|CDD|162296	TIGR01311, glycerol_kin, glyce	ref|XP_003708004.1|	PREDICTED: putative glycerol kinase 3-like [Megachile rotundata]	1,00E-53
gALL_v3_c22033	2	2	-	-	-	-	-
gALL_v3_c22034	5	5	-	-	-	-	-
gALL_v3_c22035	5	2	-	-	-	-	-
gALL_v3_c22036	4	7	-	-	-	-	-
gALL_v3_c22037	3	3	-	-	-	-	-
gALL_v3_c22038	1	4	-	-	-	-	-
gALL_v3_c22039	5	3	-	-	gb|AGM32311.1|	zinc finger-like protein, partial [Coptotermes formosanus]	1,00E-108
gALL_v3_c2204	5	9	-	-	-	-	-
gALL_v3_c22040	1	3	-	-	-	-	-
gALL_v3_c22041	2	4	-	-	ref|XP_001812041.1|	PREDICTED: similar to AGAP007807-PA [Tribolium castaneum]	1,00E-22
gALL_v3_c22042	2	4	-	-	-	-	-
gALL_v3_c22043	6	3	gnl|CDD|73156	cd01650, RT_nLTR_like, RT_nL	ref|XP_006811856.1|	PREDICTED: RNA-directed DNA polymerase from mobile element jockey-like [Saccoglossus kowalevskii]	2,00E-47
gALL_v3_c22044	1	4	-	-	-	-	-
gALL_v3_c22045	22	23	gnl|CDD|128405	smart00094, TR_FER, Transfer	gb|AAN03488.1|	transferrin [Mastotermes darwiniensis]	4,00E-83
gALL_v3_c22047	2	4	-	-	-	-	-
gALL_v3_c22048	3	1	-	-	-	-	-
gALL_v3_c22049	1	5	-	-	gb|EFZ14801.1|	hypothetical protein SINV_15055 [Solenopsis invicta]	1,00E-152
gALL_v3_c22050	2	3	gnl|CDD|31009	COG0665, DadA, Glycine/D-an	gb|AAC18056.1|	unknown protein [Anopheles gambiae]	7,00E-45
gALL_v3_c22052	1	3	-	-	-	-	-
gALL_v3_c22053	2	4	-	-	-	-	-
gALL_v3_c22054	1	3	gnl|CDD|191873	pfam07855, DUF1649, Protein	dbj|BAN20406.1|	autophagy-related protein [Riptortus pedestris]	1,00E-116
gALL_v3_c22055	3	1	-	-	-	-	-
gALL_v3_c22056	3	2	-	-	ref|XP_966817.2|	PREDICTED: similar to sorting and assembly machinery component 50 homolog [Tribolium castaneum]	6,00E-85
gALL_v3_c22057	1	4	gnl|CDD|144327	pfam00685, Sulfotransfer_1, S	ref|XP_002432901.1|	conserved hypothetical protein [Pediculus humanus corporis] gb|EEB20163.1| conserved hypothetical protein [Pediculus humanus corporis]	1,00E-40
gALL_v3_c22058	2	4	gnl|CDD|189981	pfam01425, Amidase, Amidas	ref|XP_968383.1|	PREDICTED: similar to amidase [Tribolium castaneum]	7,00E-56
gALL_v3_c2206	5	10	gnl|CDD|144445	pfam00852, Glyco_transf_10,	ref|XP_002430615.1|	glycoprotein 3-alpha-L-fucosyltransferase A, putative [Pediculus humanus corporis] gb|EEB17877.1| glycoprotein 3-alpha-L-fucosyltransferase A, putative [Pediculus huma	1,00E-124
gALL_v3_c22061	3	3	gnl|CDD|73003	cd03244, ABCC_MRP_domain	ref|XP_003698789.1|	PREDICTED: LOW QUALITY PROTEIN: ATP-binding cassette sub-family C member 9-like [Apis florea]	3,00E-86
gALL_v3_c22062	4	2	gnl|CDD|189849	pfam01125, G10, G10 protein.	ref|XP_003698345.1|	PREDICTED: protein BUD31 homolog [Apis florea] ref|XP_003699807.1| PREDICTED: protein BUD31 homolog [Megachile rotundata] ref|XP_006623755.1| PREDICTED: pro	9,00E-13
gALL_v3_c22063	2	2	-	-	-	-	-
gALL_v3_c22064	0	6	-	-	-	-	-
gALL_v3_c22065	3	1	-	-	-	-	-
gALL_v3_c22066	3	2	-	-	-	-	-
gALL_v3_c22068	5	4	-	-	-	-	-
gALL_v3_c22069	3	2	-	-	-	-	-
gALL_v3_c2207	11	11	gnl|CDD|188212	TIGR02349, DnaJ_bact, chape	ref|XP_006564601.1|	PREDICTED: dnaJ homolog subfamily B member 6-B-like isoform X4 [Apis mellifera] ref|XP_006564602.1| PREDICTED: dnaJ homolog subfamily B member 6-B-like isoform	3,00E-45
gALL_v3_c22070	1	3	-	-	gb|EFN82604.1|	Serine/threonine-protein kinase mig-15 [Harpegnathos saltator]	2,00E-19
gALL_v3_c22071	2	2	-	-	-	-	-
gALL_v3_c22072	2	1	-	-	-	-	-
gALL_v3_c22073	10	6	gnl|CDD|177801	PLN00220, PLN00220, tubulin	gb|AGM32401.1|	beta tubulin-3 [Coptotermes formosanus]	2,00E-73
gALL_v3_c22074	2	1	-	-	-	-	-
gALL_v3_c22075	0	6	-	-	-	-	-
gALL_v3_c22076	2	3	-	-	gb|EGI64696.1|	Uncharacterized protein [Acromyrmex echinatior]	3,00E-60
gALL_v3_c22077	3	1	gnl|CDD|190340	pfam02535, Zip, ZIP Zinc trans	ref|XP_004930976.1|	PREDICTED: zinc transporter ZIP10-like [Bombyx mori]	8,00E-45
gALL_v3_c22078	1	3	-	-	-	-	-
gALL_v3_c22079	1	3	-	-	-	-	-
gALL_v3_c2208	7	14	gnl|CDD|193044	pfam12474, PKK, Polo kinase k	gb|EGI62528.1|	STE20-like serine/threonine-protein kinase [Acromyrmex echinatior]	1,00E-143
gALL_v3_c22080	2	2	-	-	emb|CAR82249.1|	hypothetical protein [Cotesia congregata]	1,00E-12
gALL_v3_c22081	3	4	-	-	-	-	-
gALL_v3_c22082	4	3	gnl|CDD|193549	cd05674, M20_yscS, M20 Pep	ref|XP_005094168.1|	PREDICTED: probable carboxypeptidase PM20D1.2-like [Aplysia californica]	9,00E-86
gALL_v3_c22083	0	4	-	-	-	-	-
gALL_v3_c22084	2	5	gnl|CDD|178496	PLN02908, PLN02908, threony	ref|XP_001949601.1|	PREDICTED: 39S ribosomal protein L39, mitochondrial-like [Acyrthosiphon pisum]	1,00E-96
gALL_v3_c22087	2	2	-	-	-	-	-
gALL_v3_c22088	3	2	-	-	-	-	-
gALL_v3_c22089	1	4	-	-	-	-	-
gALL_v3_c22090	1	6	-	-	-	-	-
gALL_v3_c22092	3	1	-	-	-	-	-
gALL_v3_c22093	0	3	-	-	-	-	-
gALL_v3_c22095	1	6	-	-	-	-	-
gALL_v3_c22097	2	2	-	-	-	-	-
gALL_v3_c22098	4	2	-	-	-	-	-
gALL_v3_c22099	4	2	-	-	-	-	-
gALL_v3_c221	14	30	-	-	ref|XP_001963892.1|	GF21016 [Drosophila ananassae] gb|EDV34341.1| GF21016 [Drosophila ananassae]	2,00E-22
gALL_v3_c2210	19	28	gnl|CDD|178675	PLN03129, PLN03129, NADP-d	ref|XP_001319074.1|	adhesin AP65-1 precursor [Trichomonas vaginalis G3] gb|EAY06851.1| adhesin AP65-1 precursor [Trichomonas vaginalis G3]	0
gALL_v3_c22100	1	3	-	-	-	-	-
gALL_v3_c22101	0	4	-	-	ref|XP_002585751.1|	hypothetical protein BRAFLDRAFT_111201 [Branchiostoma floridae] gb|EEN41762.1| hypothetical protein BRAFLDRAFT_111201 [Branchiostoma floridae]	5,00E-43
gALL_v3_c22103	3	2	-	-	gb|ELU16116.1|	hypothetical protein CAPTEDRAFT_202670, partial [Capitella teleta]	6,00E-13
gALL_v3_c22105	3	1	-	-	-	-	-
gALL_v3_c22107	2	4	gnl|CDD|150784	pfam10151, DUF2359, Unchar	gb|AAH54691.1|	LOC402840 protein, partial [Danio rerio]	2,00E-39
gALL_v3_c22108	2	2	-	-	-	-	-
gALL_v3_c22109	2	2	-	-	-	-	-
gALL_v3_c2211	15	10	-	-	-	-	-
gALL_v3_c22111	5	6	-	-	ref|XP_555219.3|	AGAP008244-PA [Anopheles gambiae str. PEST] gb|EAL39610.3| AGAP008244-PA [Anopheles gambiae str. PEST]	3,00E-10
gALL_v3_c22113	0	4	-	-	-	-	-
gALL_v3_c22114	3	9	-	-	-	-	-
gALL_v3_c22115	0	5	-	-	-	-	-
gALL_v3_c22116	4	3	-	-	ref|XP_001600691.2|	PREDICTED: ribosomal protein S6 kinase delta-1-like [Nasonia vitripennis]	2,00E-06
gALL_v3_c22117	1	2	-	-	-	-	-
gALL_v3_c22119	3	1	gnl|CDD|162063	TIGR00840, b_cpa1, sodium/h	ref|XP_002430817.1|	sodium/hydrogen exchanger, putative [Pediculus humanus corporis] gb|EEB18079.1| sodium/hydrogen exchanger, putative [Pediculus humanus corporis]	5,00E-46
gALL_v3_c2212	5	6	-	-	ref|XP_002609095.1|	hypothetical protein BRAFLDRAFT_91068 [Branchiostoma floridae] gb|EEN65105.1| hypothetical protein BRAFLDRAFT_91068 [Branchiostoma floridae]	6,00E-14
gALL_v3_c22120	0	5	-	-	-	-	-
gALL_v3_c22121	2	2	-	-	-	-	-
gALL_v3_c22123	1	4	gnl|CDD|176050	cd08405, C2B_Synaptotagmin	ref|XP_004537379.1|	PREDICTED: synaptotagmin-7-like, partial [Ceratitis capitata]	8,00E-50
gALL_v3_c22124	2	2	-	-	-	-	-
gALL_v3_c22127	2	3	-	-	-	-	-
gALL_v3_c22128	2	5	-	-	ref|XP_002428910.1|	conserved hypothetical protein [Pediculus humanus corporis] gb|EEB16172.1| conserved hypothetical protein [Pediculus humanus corporis]	1,00E-66
gALL_v3_c22129	1	3	-	-	gb|AGM32832.1|	AP-3 complex subunit beta-2-like protein [Coptotermes formosanus]	4,00E-97
gALL_v3_c2213	4	7	-	-	gb|EMC80560.1|	hypothetical protein A306_11776, partial [Columba livia]	1,00E-07
gALL_v3_c22130	3	2	gnl|CDD|29257	cd00200, WD40, WD40 domai	gb|EFA10370.1|	hypothetical protein TcasGA2_TC012601 [Tribolium castaneum]	4,00E-62
gALL_v3_c22131	2	4	-	-	gb|EFN61784.1|	E3 ubiquitin-protein ligase RNF123 [Camponotus floridanus]	1,00E-115
gALL_v3_c22132	6	8	gnl|CDD|73156	cd01650, RT_nLTR_like, RT_nL	gb|EFA11785.1|	hypothetical protein TcasGA2_TC008564 [Tribolium castaneum]	7,00E-40
gALL_v3_c22134	2	2	-	-	-	-	-
gALL_v3_c22135	2	2	-	-	-	-	-
gALL_v3_c22136	5	7	-	-	-	-	-
gALL_v3_c22137	3	1	-	-	ref|XP_003427749.1|	PREDICTED: male-specific lethal 1 homolog isoform 2 [Nasonia vitripennis]	1,00E-13
gALL_v3_c22138	1	5	-	-	-	-	-
gALL_v3_c22139	2	3	gnl|CDD|143985	pfam00232, Glyco_hydro_1, G	gb|AEW67361.1|	beta-glucosidase [Coptotermes formosanus]	8,00E-82
gALL_v3_c22140	2	2	-	-	-	-	-
gALL_v3_c22141	3	1	-	-	ref|XP_006608987.1|	PREDICTED: pumilio domain-containing protein KIAA0020-like, partial [Apis dorsata]	4,00E-29
gALL_v3_c22142	5	10	-	-	-	-	-
gALL_v3_c22143	0	4	-	-	-	-	-
gALL_v3_c22144	2	3	-	-	-	-	-
gALL_v3_c22145	2	2	-	-	-	-	-
gALL_v3_c22146	1	3	-	-	-	-	-
gALL_v3_c22147	3	3	-	-	-	-	-
gALL_v3_c22148	4	3	-	-	-	-	-
gALL_v3_c22149	1	6	-	-	-	-	-
gALL_v3_c2215	10	17	gnl|CDD|128524	smart00228, PDZ, Domain pre	ref|XP_003491243.1|	PREDICTED: protein lap4-like isoform 1 [Bombus impatiens]	1,00E-129
gALL_v3_c22150	3	1	gnl|CDD|73063	cd02657, Peptidase_C19A, A s	ref|XP_003487136.1|	PREDICTED: ubiquitin carboxyl-terminal hydrolase 14-like isoform 2 [Bombus impatiens]	1,00E-41
gALL_v3_c22151	1	4	-	-	gb|ELU06943.1|	hypothetical protein CAPTEDRAFT_71830, partial [Capitella teleta]	1,00E-08
gALL_v3_c22152	0	3	-	-	-	-	-
gALL_v3_c22153	0	3	-	-	-	-	-
gALL_v3_c22154	0	3	-	-	-	-	-
gALL_v3_c22155	0	4	gnl|CDD|189968	pfam01391, Collagen, Collage	ref|XP_001603511.2|	PREDICTED: LOW QUALITY PROTEIN: collagen alpha-1(IV) chain-like [Nasonia vitripennis]	6,00E-31
gALL_v3_c22156	4	2	gnl|CDD|144473	pfam00888, Cullin, Cullin fami	ref|XP_002406452.1|	cullin, putative [Ixodes scapularis] gb|EEC15654.1| cullin, putative [Ixodes scapularis]	6,00E-67
gALL_v3_c22157	2	1	-	-	-	-	-
gALL_v3_c22158	0	4	-	-	-	-	-
gALL_v3_c22160	3	2	-	-	gb|EFN64073.1|	Craniofacial development protein 2 [Camponotus floridanus]	1,00E-29
gALL_v3_c22161	2	1	-	-	ref|XP_976040.1|	PREDICTED: similar to clathrin light chain isoform 2 [Tribolium castaneum]	8,00E-14
gALL_v3_c22163	1	4	gnl|CDD|147503	pfam05351, GMP_PDE_delta,	ref|XP_002590729.1|	hypothetical protein BRAFLDRAFT_89535 [Branchiostoma floridae] gb|EEN46740.1| hypothetical protein BRAFLDRAFT_89535 [Branchiostoma floridae]	4,00E-79
gALL_v3_c22164	2	3	-	-	-	-	-
gALL_v3_c22165	5	7	-	-	-	-	-
gALL_v3_c22166	2	7	-	-	-	-	-
gALL_v3_c22167	9	15	gnl|CDD|148286	pfam06585, JHBP, Haemolymp	gb|ABP51960.1|	takeout/JHBP-like protein [Diploptera punctata]	2,00E-45

gALL_v3_c22168	3	7	-	-	-	-	-
gALL_v3_c22169	5	5	-	-	-	-	-
gALL_v3_c2217	5	12	gnl|CDD|190641	pfam03456, uDENN, uDENN d	ref|XP_006609167.1|	PREDICTED: MAP kinase-activating death domain protein-like isoform X1 [Apis dorsata]	1,00E-109
gALL_v3_c22172	3	1	-	-	-	-	-
gALL_v3_c22173	1	3	-	-	-	-	-
gALL_v3_c22174	4	2	gnl|CDD|29018	cd00119, LYZ1, C-type lysozym	gb|AGA16574.1|	P-type lysozyme [Coptotermes formosanus]	1,00E-78
gALL_v3_c22175	3	7	-	-	-	-	-
gALL_v3_c22176	3	3	-	-	-	-	-
gALL_v3_c22177	3	2	-	-	-	-	-
gALL_v3_c22178	3	2	-	-	gb|AGM32926.1|	hypothetical protein [Coptotermes formosanus]	4,00E-10
gALL_v3_c22179	8	5	-	-	-	-	-
gALL_v3_c2218	7	9	-	-	-	-	-
gALL_v3_c22180	1	2	-	-	-	-	-
gALL_v3_c22181	2	2	-	-	gb|ETE58142.1|	Acetolactate synthase-like protein, partial [Ophiophagus hannah]	4,00E-12
gALL_v3_c22183	3	1	-	-	-	-	-
gALL_v3_c22184	1	4	gnl|CDD|185395	PTZ00009, PTZ00009, heat sho	ref|XP_973521.1|	PREDICTED: similar to heat shock protein 70 B2 [Tribolium castaneum] gb|EEZ97821.1| hypothetical protein TcasGA2_TC000188 [Tribolium castaneum]	1,00E-119
gALL_v3_c22185	3	2	-	-	ref|XP_002425660.1|	hypothetical protein Phum_PHUM213790 [Pediculus humanus corporis] gb|EEB12922.1| hypothetical protein Phum_PHUM213790 [Pediculus humanus corporis]	2,00E-36
gALL_v3_c22186	0	4	-	-	-	-	-
gALL_v3_c22187	2	4	-	-	-	-	-
gALL_v3_c22188	2	4	-	-	-	-	-
gALL_v3_c22189	1	5	-	-	-	-	-
gALL_v3_c2219	10	13	-	-	-	-	-
gALL_v3_c22190	1	3	-	-	ref|XP_002407891.1|	transmembrane protein 151B, putative [Ixodes scapularis] gb|EEC10569.1| transmembrane protein 151B, putative [Ixodes scapularis]	2,00E-53
gALL_v3_c22191	1	8	-	-	-	-	-
gALL_v3_c22192	0	3	-	-	ref|XP_002425731.1|	Homeobox protein orthopedia, putative [Pediculus humanus corporis] gb|EEB12993.1| Homeobox protein orthopedia, putative [Pediculus humanus corporis]	6,00E-33
gALL_v3_c22193	4	3	-	-	-	-	-
gALL_v3_c22194	6	3	-	-	gb|ELT92849.1|	hypothetical protein CAPTEDRAFT_224069 [Capitella teleta]	6,00E-09
gALL_v3_c22197	11	11	-	-	ref|XP_002027078.1|	GL18134 [Drosophila persimilis] gb|EDW34899.1| GL18134 [Drosophila persimilis]	4,00E-09
gALL_v3_c22198	2	2	-	-	-	-	-
gALL_v3_c22199	1	5	-	-	-	-	-
gALL_v3_c22200	3	6	gnl|CDD|149348	pfam08235, LNS2, LNS2 (Lipin,	ref|XP_003708109.1|	PREDICTED: protein retinal degeneration B-like [Megachile rotundata]	1,00E-144
gALL_v3_c22201	1	5	-	-	gb|EFN80151.1|	Putative uncharacterized protein FLJ37770 [Harpegnathos saltator]	5,00E-14
gALL_v3_c22202	2	2	-	-	-	-	-
gALL_v3_c22203	2	3	gnl|CDD|173629	cd05041, PTKc_Fes_like, Catal	ref|XP_005190905.1|	PREDICTED: tyrosine-protein kinase Fps85D-like [Musca domestica]	5,00E-83
gALL_v3_c22204	3	1	-	-	-	-	-
gALL_v3_c22205	2	2	-	-	-	-	-
gALL_v3_c22206	3	8	-	-	-	-	-
gALL_v3_c22207	2	2	-	-	-	-	-
gALL_v3_c22208	2	2	-	-	-	-	-
gALL_v3_c22209	3	3	gnl|CDD|178450	PLN02859, PLN02859, glutami	ref|XP_004928054.1|	PREDICTED: probable glutamine--tRNA ligase-like [Bombyx mori]	5,00E-91
gALL_v3_c22211	2	2	-	-	-	-	-
gALL_v3_c22212	1	3	gnl|CDD|144234	pfam00567, TUDOR, Tudor do	gb|EHJ72857.1|	putative ebna2 binding protein P100 [Danaus plexippus]	1,00E-24
gALL_v3_c22213	1	4	-	-	-	-	-
gALL_v3_c22214	1	3	-	-	-	-	-
gALL_v3_c22215	0	4	-	-	ref|XP_001814289.1|	PREDICTED: similar to conserved hypothetical protein [Tribolium castaneum] gb|EFA02376.1| hypothetical protein TcasGA2_TC008051 [Tribolium castaneum]	3,00E-12
gALL_v3_c22216	2	8	-	-	gb|EGI64696.1|	Uncharacterized protein [Acromyrmex echinatior]	3,00E-87
gALL_v3_c22217	1	3	-	-	ref|XP_003395947.1|	PREDICTED: hypothetical protein LOC100648204 [Bombus terrestris]	1,00E-09
gALL_v3_c22218	5	3	-	-	-	-	-
gALL_v3_c22219	0	4	-	-	-	-	-
gALL_v3_c22220	0	6	gnl|CDD|128433	smart00128, IPPc, Inositol poly	gb|EFA09776.1|	hypothetical protein TcasGA2_TC011918 [Tribolium castaneum]	1,00E-70
gALL_v3_c22221	0	5	-	-	-	-	-
gALL_v3_c22222	0	3	-	-	-	-	-
gALL_v3_c22223	1	3	gnl|CDD|128816	smart00543, MIF4G, Middle d	gb|EEZ98143.1|	hypothetical protein TcasGA2_TC000568 [Tribolium castaneum]	4,00E-70
gALL_v3_c22224	2	2	-	-	-	-	-
gALL_v3_c22226	2	2	-	-	-	-	-
gALL_v3_c22227	2	1	-	-	-	-	-
gALL_v3_c22228	1	3	-	-	-	-	-
gALL_v3_c2223	6	4	gnl|CDD|115083	pfam06403, Lamprin, Lamprin	-	-	-
gALL_v3_c22230	2	4	-	-	-	-	-
gALL_v3_c22231	0	4	-	-	-	-	-
gALL_v3_c22232	0	5	-	-	ref|XP_003699458.1|	PREDICTED: ral GTPase-activating protein subunit beta [Megachile rotundata]	9,00E-73
gALL_v3_c22233	2	1	-	-	gb|EFA09839.1|	hypothetical protein TcasGA2_TC011986 [Tribolium castaneum]	4,00E-13
gALL_v3_c22234	4	4	gnl|CDD|73154	cd01647, RT_LTR, RT_LTR: Rev	gb|ADV03170.1|	gag-pol polyprotein, partial [Leptopilina clavipes]	3,00E-57
gALL_v3_c22235	0	4	-	-	-	-	-
gALL_v3_c22236	3	1	-	-	-	-	-
gALL_v3_c22237	1	6	-	-	-	-	-
gALL_v3_c22238	2	7	-	-	gb|ENN82268.1|	hypothetical protein YQE_01357, partial [Dendroctonus ponderosae]	1,00E-136
gALL_v3_c22239	0	3	-	-	-	-	-
gALL_v3_c22240	1	3	-	-	-	-	-
gALL_v3_c22242	2	3	-	-	-	-	-
gALL_v3_c22243	3	2	-	-	-	-	-
gALL_v3_c22244	5	7	-	-	-	-	-
gALL_v3_c22246	2	4	-	-	-	-	-
gALL_v3_c22247	1	2	-	-	-	-	-
gALL_v3_c22248	5	7	-	-	-	-	-
gALL_v3_c22249	4	6	gnl|CDD|148286	pfam06585, JHBP, Haemolymp	ref|NP_001155387.1|	uncharacterized protein LOC100159064 precursor [Acyrthosiphon pisum] dbj|BAH71809.1| ACYPI000473 [Acyrthosiphon pisum]	7,00E-18
gALL_v3_c2225	22	19	gnl|CDD|144462	pfam00876, Innexin, Innexin.	sp|Q9XYN1.1|INX2_SCH	RecName: Full=Innexin inx2; Short=Innexin-2; AltName: Full=G-Inx2 gb|AAD29306.1|AF115854_1 innexin-2 [Schistocerca americana]	8,00E-56
gALL_v3_c22250	3	4	-	-	-	-	-
gALL_v3_c22251	3	5	-	-	-	-	-
gALL_v3_c22252	1	5	-	-	-	-	-
gALL_v3_c22253	0	3	-	-	-	-	-
gALL_v3_c22255	0	3	-	-	-	-	-
gALL_v3_c22256	2	2	-	-	-	-	-
gALL_v3_c22257	1	2	-	-	-	-	-
gALL_v3_c22258	3	1	-	-	-	-	-
gALL_v3_c22259	4	12	gnl|CDD|189482	pfam00274, Glycolytic, Fructo:	gb|ACH81781.1|	fructose 1,6-bisphosphate aldolase [Artemia franciscana]	7,00E-46
gALL_v3_c2226	5	12	-	-	-	-	-
gALL_v3_c22260	2	2	-	-	-	-	-
gALL_v3_c22262	2	2	-	-	ref|XP_002432724.1|	conserved hypothetical protein [Pediculus humanus corporis] gb|EEB19986.1| conserved hypothetical protein [Pediculus humanus corporis]	6,00E-40
gALL_v3_c22263	4	4	gnl|CDD|191443	pfam06079, Apyrase, Apyrase.	ref|XP_005098146.1|	PREDICTED: soluble calcium-activated nucleotidase 1-like isoform X1 [Aplysia californica] ref|XP_005098147.1| PREDICTED: soluble calcium-activated nucleotidase 1-like is	1,00E-103
gALL_v3_c22264	1	3	-	-	ref|XP_005464674.1|	PREDICTED: RNA-directed DNA polymerase from mobile element jockey-like [Oreochromis niloticus]	2,00E-20
gALL_v3_c22265	3	4	-	-	-	-	-
gALL_v3_c22268	6	7	-	-	-	-	-
gALL_v3_c22269	2	4	-	-	-	-	-
gALL_v3_c2227	9	15	-	-	-	-	-
gALL_v3_c22270	1	3	-	-	-	-	-
gALL_v3_c22272	1	3	-	-	-	-	-
gALL_v3_c22273	2	2	-	-	-	-	-
gALL_v3_c22274	0	4	-	-	-	-	-
gALL_v3_c22275	3	2	-	-	-	-	-
gALL_v3_c22276	3	5	gnl|CDD|144648	pfam01130, CD36, CD36 famil	ref|XP_396852.2|	PREDICTED: scavenger receptor class B member 1 [Apis mellifera]	5,00E-75
gALL_v3_c22277	6	15	-	-	-	-	-
gALL_v3_c22278	2	2	-	-	ref|XP_006809461.1|	PREDICTED: protein NLRC3-like [Neolamprologus brichardi]	6,00E-33
gALL_v3_c22279	3	5	-	-	-	-	-
gALL_v3_c22280	2	3	-	-	-	-	-
gALL_v3_c22281	1	3	-	-	-	-	-
gALL_v3_c22282	2	5	-	-	-	-	-
gALL_v3_c22283	1	3	-	-	-	-	-
gALL_v3_c22285	2	2	-	-	ref|XP_003424445.1|	PREDICTED: hypothetical protein LOC100678286 isoform 2 [Nasonia vitripennis]	5,00E-08
gALL_v3_c22286	2	3	-	-	-	-	-
gALL_v3_c22287	0	6	gnl|CDD|187657	cd08954, KR_1_FAS_SDR_x, be	gb|EFZ09751.1|	hypothetical protein SINV_06292 [Solenopsis invicta]	8,00E-39
gALL_v3_c22288	2	2	-	-	-	-	-
gALL_v3_c22289	4	3	-	-	-	-	-
gALL_v3_c2229	5	3	-	-	gb|AGM32490.1|	60S ribosomal protein L5-like protein, partial [Coptotermes formosanus]	9,00E-13
gALL_v3_c22290	2	3	-	-	gb|EFN65874.1|	RNA-binding protein 10 [Camponotus floridanus]	3,00E-54
gALL_v3_c22292	7	5	-	-	-	-	-
gALL_v3_c22294	3	4	-	-	ref|XP_003396718.1|	PREDICTED: THUMP domain-containing protein 1-like [Bombus terrestris]	2,00E-47
gALL_v3_c22295	3	6	-	-	gb|EFN68425.1|	Round spermatid basic protein 1-like protein [Camponotus floridanus]	1,00E-118
gALL_v3_c22296	0	4	-	-	-	-	-
gALL_v3_c22297	0	3	-	-	-	-	-
gALL_v3_c22298	3	2	-	-	ref|XP_002423105.1|	transcriptional repressor p66 beta, putative [Pediculus humanus corporis] gb|EEB10367.1| transcriptional repressor p66 beta, putative [Pediculus humanus corporis]	2,00E-22
gALL_v3_c22300	5	2	-	-	-	-	-
gALL_v3_c22301	7	16	gnl|CDD|189954	pfam01359, Transposase_1, Tr	ref|XP_003702583.1|	PREDICTED: histone-lysine N-methyltransferase SETMAR-like [Megachile rotundata]	2,00E-77
gALL_v3_c22303	2	8	-	-	-	-	-
gALL_v3_c22304	4	4	-	-	-	-	-
gALL_v3_c22305	5	5	-	-	-	-	-
gALL_v3_c22307	1	3	-	-	-	-	-
gALL_v3_c22309	6	7	-	-	-	-	-
gALL_v3_c2231	5	11	-	-	-	-	-
gALL_v3_c22310	3	1	-	-	-	-	-
gALL_v3_c22311	2	2	-	-	-	-	-
gALL_v3_c22312	0	4	-	-	-	-	-
gALL_v3_c22313	0	4	-	-	-	-	-
gALL_v3_c22314	2	3	-	-	-	-	-
gALL_v3_c22315	4	6	-	-	-	-	-
gALL_v3_c22316	2	1	-	-	ref|XP_970294.2|	PREDICTED: similar to echinus CG2904-PB [Tribolium castaneum]	2,00E-10
gALL_v3_c22317	3	1	-	-	-	-	-
gALL_v3_c22318	1	3	-	-	-	-	-
gALL_v3_c22319	1	4	-	-	-	-	-
gALL_v3_c22320	7	6	-	-	-	-	-
gALL_v3_c22321	2	5	-	-	-	-	-
gALL_v3_c22323	1	5	-	-	-	-	-
gALL_v3_c22324	1	3	-	-	-	-	-
gALL_v3_c22325	2	8	-	-	-	-	-

gALL_v3_c22326	2	2	gnl|CDD|144191	pfam00503, G-alpha, G-protei	gb|AEV76928.1|	guanine nucleotide binding protein [Homo sapiens]	2,00E-50
gALL_v3_c22327	3	3	-	-	-	-	-
gALL_v3_c22328	2	5	-	-	-	-	-
gALL_v3_c22329	2	3	gnl|CDD|191150	pfam04987, PigN, Phosphatidy	ref|XP_006816986.1|	PREDICTED: GPI ethanolamine phosphate transferase 1-like [Saccoglossus kowalevskii]	5,00E-12
gALL_v3_c2233	1	7	-	-	-	-	-
gALL_v3_c22330	3	2	-	-	-	-	-
gALL_v3_c22331	1	4	-	-	-	-	-
gALL_v3_c22332	7	6	-	-	-	-	-
gALL_v3_c22333	0	3	-	-	-	-	-
gALL_v3_c22334	1	4	-	-	-	-	-
gALL_v3_c22335	3	4	-	-	ref|XP_003251399.1|	PREDICTED: anoctamin-8-like isoformX1 [Apis mellifera]	3,00E-88
gALL_v3_c22336	2	4	-	-	-	-	-
gALL_v3_c22339	4	4	-	-	-	-	-
gALL_v3_c2234	13	8	-	-	-	-	-
gALL_v3_c22340	2	2	-	-	gb|EFN83010.1|	Protein LAP4 [Harpegnathos saltator]	2,00E-43
gALL_v3_c22342	2	3	-	-	-	-	-
gALL_v3_c22343	20	16	gnl|CDD|181753	PRK09281, PRK09281, F0F1 AT	gb|AGM32898.1|	putative ATP synthase subunit alpha [Coptotermes formosanus]	1,00E-59
gALL_v3_c22344	1	3	-	-	-	-	-
gALL_v3_c22346	4	4	-	-	-	-	-
gALL_v3_c22347	4	3	-	-	gb|EGI66083.1|	hypothetical protein G5I_05476 [Acromyrmex echinatior]	3,00E-11
gALL_v3_c22348	1	3	-	-	-	-	-
gALL_v3_c22349	1	2	-	-	gb|EFA03599.1|	hypothetical protein TcasGA2_TC013686 [Tribolium castaneum]	1,00E-27
gALL_v3_c2235	20	18	-	-	ref|XP_003699923.1|	PREDICTED: uncharacterized protein LOC100874765 [Megachile rotundata]	1,00E-156
gALL_v3_c22352	5	3	-	-	-	-	-
gALL_v3_c22353	2	1	-	-	-	-	-
gALL_v3_c22354	1	5	-	-	tpg|DAA34497.1|	TPA_inf: transposase Tn3 [Amblyomma variegatum]	9,00E-12
gALL_v3_c22355	1	4	gnl|CDD|193195	pfam12719, Cnd3, Nuclear cor	gb|EFN85401.1|	60S ribosomal protein L7 [Harpegnathos saltator]	4,00E-15
gALL_v3_c22356	2	4	gnl|CDD|192503	pfam10262, Rdx, Rdx family. T	ref|XP_001605689.2|	PREDICTED: hypothetical protein LOC100122087 [Nasonia vitripennis]	5,00E-27
gALL_v3_c22357	2	2	gnl|CDD|173748	cd07853, STKc_NLK, Catalytic	gb|EFN71528.1|	Serine/threonine kinase NLK [Camponotus floridanus]	3,00E-18
gALL_v3_c22358	4	3	-	-	-	-	-
gALL_v3_c22360	3	3	-	-	-	-	-
gALL_v3_c22361	2	1	-	-	-	-	-
gALL_v3_c22363	2	2	-	-	-	-	-
gALL_v3_c22365	3	1	-	-	ref|XP_974145.1|	PREDICTED: similar to dikar CG32393-PA [Tribolium castaneum]	3,00E-26
gALL_v3_c22366	3	3	-	-	gb|AAW57775.1|	Parcxpwnx05 [Periplaneta americana]	2,00E-28
gALL_v3_c22367	3	4	-	-	ref|XP_003424729.1|	PREDICTED: hypothetical protein LOC100120820 isoform 2 [Nasonia vitripennis] ref|XP_003424730.1| PREDICTED: hypothetical protein LOC100120820 isoform 3 [Nasonia	9,00E-12
gALL_v3_c22369	3	1	-	-	-	-	-
gALL_v3_c22370	2	2	-	-	-	-	-
gALL_v3_c22371	0	3	-	-	-	-	-
gALL_v3_c22372	7	7	-	-	gb|AGM32136.1|	hypothetical protein [Coptotermes formosanus]	8,00E-10
gALL_v3_c22373	0	4	-	-	-	-	-
gALL_v3_c22374	4	7	-	-	-	-	-
gALL_v3_c22375	3	2	gnl|CDD|128833	smart00561, MBT, Present in D	ref|XP_003487216.1|	PREDICTED: polycomb protein Sfmbt-like isoform 1 [Bombus impatiens]	2,00E-69
gALL_v3_c22376	2	5	-	-	-	-	-
gALL_v3_c22377	3	2	-	-	-	-	-
gALL_v3_c22378	1	5	-	-	-	-	-
gALL_v3_c22379	2	2	-	-	-	-	-
gALL_v3_c2238	5	6	gnl|CDD|192393	pfam09816, EAF, RNA polymer	ref|XP_006619547.1|	PREDICTED: ell-associated factor Eaf-like isoform X1 [Apis dorsata] ref|XP_006619548.1| PREDICTED: ell-associated factor Eaf-like isoform X2 [Apis dorsata]	6,00E-32
gALL_v3_c22380	3	1	-	-	-	-	-
gALL_v3_c22381	4	2	-	-	-	-	-
gALL_v3_c22382	2	2	-	-	-	-	-
gALL_v3_c22383	1	2	-	-	-	-	-
gALL_v3_c22384	0	4	-	-	-	-	-
gALL_v3_c22385	1	6	gnl|CDD|145919	pfam03028, Dynein_heavy, Dy	ref|XP_002428815.1|	dynein heavy chain, cytosolic, putative [Pediculus humanus corporis] gb|EEB16077.1| dynein heavy chain, cytosolic, putative [Pediculus humanus corporis]	1,00E-81
gALL_v3_c22386	2	2	-	-	-	-	-
gALL_v3_c22387	2	5	-	-	-	-	-
gALL_v3_c22388	1	5	-	-	-	-	-
gALL_v3_c22389	1	4	-	-	-	-	-
gALL_v3_c2239	4	2	-	-	-	-	-
gALL_v3_c22390	1	3	-	-	gb|EFA04696.1|	hypothetical protein TcasGA2_TC014708 [Tribolium castaneum]	2,00E-14
gALL_v3_c22392	5	3	gnl|CDD|162101	TIGR00911, 2A0308, L-type an	ref|XP_315672.4|	AGAP005653-PA [Anopheles gambiae str. PEST] gb|EAA11727.4| AGAP005653-PA [Anopheles gambiae str. PEST]	4,00E-63
gALL_v3_c22393	6	4	-	-	-	-	-
gALL_v3_c22394	3	1	-	-	-	-	-
gALL_v3_c22397	2	3	-	-	-	-	-
gALL_v3_c22398	4	7	-	-	ref|XP_968114.2|	PREDICTED: similar to AT-rich interactive domain-containing protein 5B (ARID domain-containing protein 5B) (Mrf1-like) (Modulator recognition factor 2) (MRF-2) [Triboliun	6,00E-11
gALL_v3_c22399	2	4	-	-	-	-	-
gALL_v3_c224	13	14	-	-	-	-	-
gALL_v3_c2240	6	11	-	-	-	-	-
gALL_v3_c22400	1	4	-	-	-	-	-
gALL_v3_c22402	3	8	-	-	-	-	-
gALL_v3_c22403	2	2	-	-	ref|XP_002431637.1|	heat containing protein, putative [Pediculus humanus corporis] gb|EEB18899.1| heat containing protein, putative [Pediculus humanus corporis]	7,00E-14
gALL_v3_c22404	2	8	-	-	ref|XP_006620648.1|	PREDICTED: general transcription factor 3C polypeptide 1-like [Apis dorsata]	4,00E-73
gALL_v3_c22405	2	2	-	-	-	-	-
gALL_v3_c22406	3	2	-	-	-	-	-
gALL_v3_c22407	6	7	gnl|CDD|130171	TIGR01101, V_ATP_synt_F, vac	ref|XP_002428572.1|	vacuolar ATP synthase subunit F, putative [Pediculus humanus corporis] gb|EEB15834.1| vacuolar ATP synthase subunit F, putative [Pediculus humanus corporis]	2,00E-36
gALL_v3_c22408	3	6	-	-	-	-	-
gALL_v3_c22409	3	1	gnl|CDD|189635	pfam00621, RhoGEF, RhoGEF	gb|EFN78743.1|	Pleckstrin-like proteiny domain-containing family G member 5 [Harpegnathos saltator]	3,00E-30
gALL_v3_c2241	12	9	gnl|CDD|187593	cd05332, 11beta-HSD1_like_S	ref|XP_001603507.1|	PREDICTED: dehydrogenase/reductase SDR family member 7-like [Nasonia vitripennis]	1,00E-111
gALL_v3_c22410	3	2	-	-	-	-	-
gALL_v3_c22411	1	4	-	-	-	-	-
gALL_v3_c22412	2	2	-	-	ref|XP_006611377.1|	PREDICTED: ral GTPase-activating protein subunit alpha-1-like isoform X1 [Apis dorsata]	3,00E-33
gALL_v3_c22413	2	2	-	-	-	-	-
gALL_v3_c22414	3	1	-	-	-	-	-
gALL_v3_c22415	0	4	gnl|CDD|30099	cd01377, MYSc_type_II, Myos	gb|ACQ91272.1|	myosin heavy chain variant A [Bombyx mori]	4,00E-10
gALL_v3_c22416	3	3	-	-	-	-	-
gALL_v3_c22418	3	5	-	-	-	-	-
gALL_v3_c22419	0	4	-	-	emb|CAG11395.1|	unnamed protein product [Tetraodon nigroviridis]	1,00E-17
gALL_v3_c2242	7	7	gnl|CDD|147089	pfam04756, OST3_OST6, OST3	ref|XP_971395.1|	PREDICTED: similar to CG7830 CG7830-PA [Tribolium castaneum] gb|EFA01636.1| hypothetical protein TcasGA2_TC007206 [Tribolium castaneum]	3,00E-93
gALL_v3_c22421	0	3	gnl|CDD|132978	cd06647, STKc_PAK_I, Catalyti	ref|XP_001599793.2|	PREDICTED: serine/threonine-protein kinase PAK 3-like [Nasonia vitripennis]	1,00E-70
gALL_v3_c22422	0	5	-	-	ref|XP_002432736.1|	membrane-bound transcription factor site 2 protease, putative [Pediculus humanus corporis] gb|EEB19998.1| membrane-bound transcription factor site 2 protease, putat	7,00E-15
gALL_v3_c22423	2	2	-	-	ref|XP_003490441.1|	PREDICTED: probable multidrug resistance-associated protein lethal(2)03659-like [Bombus impatiens]	5,00E-24
gALL_v3_c22425	4	5	gnl|CDD|178512	PLN02924, PLN02924, thymidy	ref|XP_789676.2|	PREDICTED: thymidylate kinase-like [Strongylocentrotus purpuratus] ref|XP_784160.2| PREDICTED: thymidylate kinase-like [Strongylocentrotus purpuratus]	1,00E-48
gALL_v3_c22426	2	3	-	-	-	-	-
gALL_v3_c22427	0	4	-	-	-	-	-
gALL_v3_c22428	4	2	gnl|CDD|73154	cd01647, RT_LTR, RT_LTR: Rev	gb|EFA12566.1|	hypothetical protein TcasGA2_TC004195 [Tribolium castaneum]	1,00E-26
gALL_v3_c22429	4	5	gnl|CDD|128524	smart00228, PDZ, Domain pre	ref|XP_002427780.1|	pdz domain protein arc, putative [Pediculus humanus corporis] gb|EEB15042.1| pdz domain protein arc, putative [Pediculus humanus corporis]	1,00E-51
gALL_v3_c2243	18	17	gnl|CDD|185453	PTZ00110, PTZ00110, helicase	ref|XP_002425300.1|	DEAD box ATP-dependent RNA helicase, putative [Pediculus humanus corporis] gb|EEB12562.1| DEAD box ATP-dependent RNA helicase, putative [Pediculus humanus corp	0
gALL_v3_c22430	1	3	-	-	ref|XP_004917635.1|	PREDICTED: putative nuclease HARBI1-like isoform X1 [Xenopus (Silurana) tropicalis] ref|XP_004917636.1| PREDICTED: putative nuclease HARBI1-like isoform X2 [Xenopus	1,00E-18
gALL_v3_c22431	3	6	-	-	-	-	-
gALL_v3_c22432	0	5	-	-	-	-	-
gALL_v3_c22433	8	12	-	-	-	-	-
gALL_v3_c22434	2	3	-	-	-	-	-
gALL_v3_c22435	2	3	gnl|CDD|189424	pfam00153, Mito_carr, Mitoch	ref|XP_002412568.1|	oxodicarboxylate carrier protein, putative [Ixodes scapularis] gb|EEC15864.1| oxodicarboxylate carrier protein, putative [Ixodes scapularis]	1,00E-58
gALL_v3_c22436	3	1	-	-	-	-	-
gALL_v3_c22438	1	2	gnl|CDD|113519	pfam04752, ChaC, ChaC-like p	ref|XP_002409327.1|	conserved hypothetical protein [Ixodes scapularis] gb|EEC04166.1| conserved hypothetical protein [Ixodes scapularis]	7,00E-23
gALL_v3_c22439	3	5	-	-	gb|EFN67804.1|	hypothetical protein EAG_07302 [Camponotus floridanus]	6,00E-34
gALL_v3_c2244	14	22	-	-	-	-	-
gALL_v3_c22440	1	3	-	-	-	-	-
gALL_v3_c22441	3	3	-	-	-	-	-
gALL_v3_c22442	2	4	-	-	-	-	-
gALL_v3_c22443	1	3	gnl|CDD|190008	pfam01490, Aa_trans, Transm	gb|EFN83214.1|	Proton-coupled amino acid transporter 1 [Harpegnathos saltator]	9,00E-57
gALL_v3_c22444	5	2	gnl|CDD|178742	PLN03201, PLN03201, RAB ger	ref|XP_002423260.1|	Geranylgeranyl transferase type-2 subunit beta, putative [Pediculus humanus corporis] gb|EEB10522.1| Geranylgeranyl transferase type-2 subunit beta, putative [Pediculu:	1,00E-115
gALL_v3_c22445	0	4	-	-	-	-	-
gALL_v3_c22446	2	3	-	-	-	-	-
gALL_v3_c22448	0	6	gnl|CDD|190530	pfam03124, EXS, EXS family. V	ref|XP_001811015.1|	PREDICTED: similar to xenotropic and polytropic murine leukemia virus receptor xpr1 [Tribolium castaneum] gb|EFA12730.1| hypothetical protein TcasGA2_TC002365 [Trib	1,00E-97
gALL_v3_c2245	9	13	-	-	gb|EFN74187.1|	Translational activator GCN1 [Camponotus floridanus]	0
gALL_v3_c22451	5	7	-	-	-	-	-
gALL_v3_c22452	4	2	-	-	-	-	-
gALL_v3_c22453	2	3	-	-	ref|XP_003240808.1|	PREDICTED: hypothetical protein LOC100569264 [Acyrthosiphon pisum]	1,00E-13
gALL_v3_c22455	3	1	-	-	-	-	-
gALL_v3_c22457	3	1	-	-	ref|XP_003486619.1|	PREDICTED: hypothetical protein LOC100746843 isoform 1 [Bombus impatiens]	5,00E-11
gALL_v3_c22458	0	5	-	-	-	-	-
gALL_v3_c2246	9	17	gnl|CDD|189954	pfam01359, Transposase_1, Tr	ref|XP_003242319.1|	PREDICTED: histone-lysine N-methyltransferase SETMAR-like [Acyrthosiphon pisum]	3,00E-37
gALL_v3_c22460	1	3	-	-	-	-	-
gALL_v3_c22461	1	6	gnl|CDD|128917	smart00672, CAP10, Putative l	gb|ERL92818.1|	hypothetical protein D910_10126 [Dendroctonus ponderosae]	2,00E-78
gALL_v3_c22462	3	3	-	-	ref|XP_001602467.2|	PREDICTED: hypothetical protein LOC100118520 [Nasonia vitripennis]	8,00E-08
gALL_v3_c22463	3	4	-	-	-	-	-
gALL_v3_c22464	2	5	-	-	-	-	-
gALL_v3_c22465	1	2	-	-	-	-	-
gALL_v3_c22466	2	5	-	-	-	-	-
gALL_v3_c22467	0	6	-	-	-	-	-
gALL_v3_c22468	0	4	-	-	-	-	-
gALL_v3_c22469	2	1	-	-	-	-	-
gALL_v3_c2247	21	21	gnl|CDD|191111	pfam04849, HAP1_N, HAP1 N-	ref|XP_006569841.1|	PREDICTED: trafficking kinesin-binding protein milt isoform X3 [Apis mellifera]	0
gALL_v3_c22470	2	1	-	-	-	-	-
gALL_v3_c22471	3	1	-	-	-	-	-
gALL_v3_c22473	2	2	-	-	-	-	-
gALL_v3_c22475	0	4	-	-	-	-	-
gALL_v3_c22476	2	6	-	-	gb|EGI65851.1|	Tubulin glycylase 3B [Acromyrmex echinatior]	1,00E-12
gALL_v3_c22477	3	3	-	-	gb|EFA13299.1|	hypothetical protein TcasGA2_TC014829 [Tribolium castaneum]	8,00E-23
gALL_v3_c22478	2	1	-	-	-	-	-
gALL_v3_c22479	4	2	-	-	-	-	-
gALL_v3_c2248	8	4	-	-	-	-	-
gALL_v3_c22480	2	4	-	-	-	-	-

gALL_v3_c22482	1	3	-	-	-	-	-
gALL_v3_c22483	1	6	gnl|CDD|146024	pfam03189, Otopetrin, Protei	gb|EFN73486.1|	Otopetrin-2 [Camponotus floridanus]	2,00E-79
gALL_v3_c22484	2	2	-	-	-	-	-
gALL_v3_c22485	1	2	-	-	-	-	-
gALL_v3_c22486	3	1	-	-	-	-	-
gALL_v3_c22487	2	2	gnl|CDD|162147	TIGR00990, 3a0801s09, mitoc	ref|XP_006623963.1|	PREDICTED: RNA polymerase II-associated protein 3-like, partial [Apis dorsata]	8,00E-46
gALL_v3_c22489	1	5	-	-	-	-	-
gALL_v3_c22491	2	3	-	-	-	-	-
gALL_v3_c22493	1	4	-	-	gb|EGI57713.1|	Golgi apparatus protein 1 [Acromyrmex echinatior]	3,00E-27
gALL_v3_c22494	3	1	-	-	-	-	-
gALL_v3_c22495	1	2	-	-	-	-	-
gALL_v3_c22496	6	5	-	-	-	-	-
gALL_v3_c22497	2	4	-	-	-	-	-
gALL_v3_c22499	3	1	-	-	-	-	-
gALL_v3_c225	4	10	-	-	-	-	-
gALL_v3_c2250	9	21	-	-	gb|EFN71845.1|	Selenide, water dikinase [Camponotus floridanus]	3,00E-49
gALL_v3_c22500	1	1	-	-	-	-	-
gALL_v3_c22501	3	1	-	-	gb|EFN83838.1|	hypothetical protein EAI_02652 [Harpegnathos saltator]	1,00E-06
gALL_v3_c22503	9	9	-	-	dbj|BAG75121.1|	lipophorin precursor [Nilaparvata lugens]	2,00E-13
gALL_v3_c22504	0	5	-	-	-	-	-
gALL_v3_c22506	0	4	-	-	-	-	-
gALL_v3_c22507	0	3	-	-	-	-	-
gALL_v3_c22508	2	1	-	-	-	-	-
gALL_v3_c22509	13	9	-	-	ref|XP_001943393.2|	PREDICTED: hypothetical protein LOC100166399 [Acyrthosiphon pisum]	8,00E-44
gALL_v3_c2251	10	16	-	-	ref|XP_002431737.1|	protein krueppel, putative [Pediculus humanus corporis] gb|EEB18999.1| protein krueppel, putative [Pediculus humanus corporis]	5,00E-69
gALL_v3_c22511	2	2	-	-	-	-	-
gALL_v3_c22512	2	2	-	-	ref|XP_003426334.1|	PREDICTED: probable phosphorylase b kinase regulatory subunit alpha-like isoform 2 [Nasonia vitripennis]	7,00E-89
gALL_v3_c22513	1	4	-	-	-	-	-
gALL_v3_c22514	4	2	-	-	-	-	-
gALL_v3_c22516	0	4	-	-	gb|EFX74631.1|	hypothetical protein DAPPUDRAFT_307206 [Daphnia pulex]	3,00E-47
gALL_v3_c22517	5	2	gnl|CDD|128578	smart00282, LamG, Laminin G	ref|XP_001950530.2|	PREDICTED: laminin subunit alpha-1-like [Acyrthosiphon pisum]	1,00E-83
gALL_v3_c22518	2	6	-	-	-	-	-
gALL_v3_c22519	2	5	gnl|CDD|32302	COG2119, COG2119, Predicte	ref|XP_001605345.2|	PREDICTED: transmembrane protein 165-like [Nasonia vitripennis]	7,00E-58
gALL_v3_c2252	10	18	gnl|CDD|189773	pfam00928, Adap_comp_sub,	ref|XP_002427003.1|	AP-2 complex subunit mu-1, putative [Pediculus humanus corporis] gb|EEB14265.1| AP-2 complex subunit mu-1, putative [Pediculus humanus corporis]	1,00E-156
gALL_v3_c22520	1	3	-	-	-	-	-
gALL_v3_c22522	5	4	gnl|CDD|48215	cd03403, Band_7_stomatin_li	ref|XP_973602.2|	PREDICTED: similar to AGAP009439-PA [Tribolium castaneum]	5,00E-93
gALL_v3_c22523	3	4	-	-	-	-	-
gALL_v3_c22524	2	7	gnl|CDD|128386	smart00072, GuKc, Guanylate	gb|EFZ10976.1|	hypothetical protein SINV_12559 [Solenopsis invicta]	1,00E-107
gALL_v3_c22525	3	2	-	-	-	-	-
gALL_v3_c22526	2	2	-	-	ref|XP_003399129.1|	PREDICTED: WD repeat-containing protein mio-B-like [Bombus terrestris]	2,00E-37
gALL_v3_c22527	3	4	-	-	gb|EFN87505.1|	Protein crumbs [Harpegnathos saltator]	1,00E-144
gALL_v3_c22528	2	3	-	-	-	-	-
gALL_v3_c2253	10	8	gnl|CDD|128963	smart00724, TLC, TRAM, LAG1	ref|XP_966650.1|	PREDICTED: similar to CG17841 CG17841-PA [Tribolium castaneum] gb|EFA05283.1| hypothetical protein TcasGA2_TC015440 [Tribolium castaneum]	3,00E-74
gALL_v3_c22531	1	2	-	-	-	-	-
gALL_v3_c22533	2	2	-	-	-	-	-
gALL_v3_c22535	2	5	-	-	-	-	-
gALL_v3_c22536	1	3	-	-	-	-	-
gALL_v3_c22537	2	2	gnl|CDD|131231	TIGR02176, pyruv_ox_red, pyr	gb|AAV74342.1|	pyruvate:ferredoxin oxidoreductase A-like protein [Trichomonas vaginalis]	2,00E-31
gALL_v3_c22538	2	4	-	-	-	-	-
gALL_v3_c22539	1	3	-	-	-	-	-
gALL_v3_c2254	7	7	-	-	gb|ADI61823.1|	endonuclease-reverse transcriptase [Bombyx mori]	2,00E-40
gALL_v3_c22540	3	3	-	-	-	-	-
gALL_v3_c22541	1	5	-	-	-	-	-
gALL_v3_c22543	0	4	-	-	gb|EFN65619.1|	Putative uncharacterized transposon-derived protein F52C9.6 [Camponotus floridanus]	4,00E-07
gALL_v3_c22544	0	3	-	-	-	-	-
gALL_v3_c22545	2	2	-	-	-	-	-
gALL_v3_c22548	2	3	-	-	-	-	-
gALL_v3_c22549	2	4	-	-	-	-	-
gALL_v3_c2255	8	12	-	-	dbj|BAB84572.1|	envelope glycoprotein precursor [Crimean-Congo hemorrhagic fever virus]	2,00E-08
gALL_v3_c22550	1	3	-	-	-	-	-
gALL_v3_c22551	0	3	-	-	gb|AAC03111.2|	family 4 cytochrome P450 [Coptotermes acinaciformis]	8,00E-32
gALL_v3_c22552	1	4	-	-	-	-	-
gALL_v3_c22553	1	2	-	-	-	-	-
gALL_v3_c22555	0	4	-	-	-	-	-
gALL_v3_c22556	5	4	gnl|CDD|29261	cd00204, ANK, ankyrin repeat	ref|XP_002427786.1|	NF-kappa-B inhibitor alpha, putative [Pediculus humanus corporis] gb|EEB15048.1| NF-kappa-B inhibitor alpha, putative [Pediculus humanus corporis]	8,00E-46
gALL_v3_c22557	2	2	-	-	-	-	-
gALL_v3_c22558	3	1	gnl|CDD|190188	pfam01997, Translin, Translin	gb|ENN77678.1|	hypothetical protein YQE_05829, partial [Dendroctonus ponderosae] gb|ERL88621.1| hypothetical protein D910_06006 [Dendroctonus ponderosae]	2,00E-34
gALL_v3_c22559	3	2	-	-	-	-	-
gALL_v3_c2256	3	8	-	-	gb|ABB89947.1|	ace2 type acetylcholinesterase [Blattella germanica]	1,00E-11
gALL_v3_c22560	0	5	-	-	-	-	-
gALL_v3_c22561	2	2	gnl|CDD|128524	smart00228, PDZ, Domain pre	ref|XP_001813427.1|	PREDICTED: similar to arc CG6741-PB [Tribolium castaneum] gb|EFA10855.1| hypothetical protein TcasGA2_TC001632 [Tribolium castaneum]	1,00E-59
gALL_v3_c22562	1	3	-	-	-	-	-
gALL_v3_c22564	5	4	-	-	ref|XP_002430884.1|	conserved hypothetical protein [Pediculus humanus corporis] gb|EEB18146.1| conserved hypothetical protein [Pediculus humanus corporis]	0
gALL_v3_c22565	2	2	gnl|CDD|31229	COG1026, COG1026, Predicte	gb|EFN77510.1|	Presequence protease, mitochondrial [Harpegnathos saltator]	2,00E-50
gALL_v3_c22566	5	2	-	-	-	-	-
gALL_v3_c22567	3	2	-	-	-	-	-
gALL_v3_c22568	0	5	-	-	-	-	-
gALL_v3_c22569	4	3	-	-	-	-	-
gALL_v3_c22570	3	1	-	-	-	-	-
gALL_v3_c22571	1	3	-	-	-	-	-
gALL_v3_c22572	1	3	-	-	-	-	-
gALL_v3_c22573	3	2	gnl|CDD|190530	pfam03124, EXS, EXS family.	ref|XP_969695.1|	PREDICTED: similar to xenotropic and polytropic murine leukemia virus receptor xpr1 [Tribolium castaneum] gb|EEZ99778.1| hypothetical protein TcasGA2_TC002557 [Trib	2,00E-88
gALL_v3_c22574	2	5	-	-	-	-	-
gALL_v3_c22576	3	1	gnl|CDD|133072	cd03860, M14_CP_A-B_like, T	ref|XP_003396553.1|	PREDICTED: zinc carboxypeptidase A 1-like [Bombus terrestris]	3,00E-69
gALL_v3_c22577	2	5	-	-	-	-	-
gALL_v3_c22578	3	2	-	-	ref|XP_003394602.1|	PREDICTED: disks large homolog 5-like isoform 1 [Bombus terrestris]	5,00E-29
gALL_v3_c22579	1	3	-	-	-	-	-
gALL_v3_c2258	11	15	-	-	-	-	-
gALL_v3_c22580	6	4	-	-	-	-	-
gALL_v3_c22581	0	3	-	-	-	-	-
gALL_v3_c22582	1	4	-	-	-	-	-
gALL_v3_c22583	1	2	-	-	-	-	-
gALL_v3_c22586	3	2	gnl|CDD|190820	pfam03987, Autophagy_act_C	ref|XP_002431060.1|	conserved hypothetical protein [Pediculus humanus corporis] gb|EEB18322.1| conserved hypothetical protein [Pediculus humanus corporis]	6,00E-63
gALL_v3_c22587	2	3	-	-	-	-	-
gALL_v3_c22588	5	2	gnl|CDD|189372	pfam00067, p450, Cytochrom	gb|EFA12633.1|	cytochrome P450 6BK5 [Tribolium castaneum]	3,00E-41
gALL_v3_c22589	1	4	gnl|CDD|149205	pfam07993, NAD_binding_4,	ref|XP_003394733.1|	PREDICTED: putative fatty acyl-CoA reductase CG5065-like [Bombus terrestris]	1,00E-09
gALL_v3_c22590	2	3	-	-	-	-	-
gALL_v3_c22591	1	4	-	-	-	-	-
gALL_v3_c22592	4	5	-	-	gb|EFA01095.1|	hypothetical protein TcasGA2_TC004018 [Tribolium castaneum]	1,00E-44
gALL_v3_c22593	2	1	-	-	-	-	-
gALL_v3_c22594	2	2	-	-	-	-	-
gALL_v3_c22595	5	5	-	-	-	-	-
gALL_v3_c22596	1	3	-	-	-	-	-
gALL_v3_c22597	0	3	-	-	-	-	-
gALL_v3_c22598	0	4	-	-	-	-	-
gALL_v3_c226	11	13	-	-	-	-	-
gALL_v3_c22602	0	4	-	-	-	-	-
gALL_v3_c22603	2	4	gnl|CDD|189954	pfam01359, Transposase_1, T	gb|AAC28159.1|	mariner transposase [Pycnoscelus surinamensis]	6,00E-59
gALL_v3_c22604	2	3	-	-	-	-	-
gALL_v3_c22605	2	1	-	-	-	-	-
gALL_v3_c22607	2	3	-	-	ref|XP_002432891.1|	conserved hypothetical protein [Pediculus humanus corporis] gb|EEB20153.1| conserved hypothetical protein [Pediculus humanus corporis]	3,00E-45
gALL_v3_c22608	3	2	-	-	ref|XP_002430605.1|	hypothetical protein Phum_PHUM491660 [Pediculus humanus corporis] gb|EEB17867.1| hypothetical protein Phum_PHUM491660 [Pediculus humanus corporis]	2,00E-22
gALL_v3_c22609	3	1	-	-	-	-	-
gALL_v3_c22611	3	4	-	-	-	-	-
gALL_v3_c22612	1	5	-	-	-	-	-
gALL_v3_c22613	1	4	-	-	-	-	-
gALL_v3_c22615	3	3	-	-	-	-	-
gALL_v3_c22616	3	2	-	-	gb|EFN87076.1|	hypothetical protein EAI_04977 [Harpegnathos saltator]	2,00E-37
gALL_v3_c22617	3	1	-	-	-	-	-
gALL_v3_c22618	1	5	-	-	-	-	-
gALL_v3_c22619	0	3	-	-	-	-	-
gALL_v3_c2262	5	4	-	-	gb|ERL85515.1|	hypothetical protein D910_02934 [Dendroctonus ponderosae]	5,00E-15
gALL_v3_c22620	2	1	-	-	-	-	-
gALL_v3_c22621	5	2	-	-	ref|XP_003704565.1|	PREDICTED: COMM domain-containing protein 3-like [Megachile rotundata]	8,00E-08
gALL_v3_c22622	1	2	-	-	-	-	-
gALL_v3_c22623	2	2	-	-	-	-	-
gALL_v3_c22624	1	4	-	-	-	-	-
gALL_v3_c22625	3	1	-	-	ref|XP_005998403.1|	PREDICTED: uncharacterized protein LOC102358965 isoform X1 [Latimeria chalumnae] ref|XP_005998404.1| PREDICTED: uncharacterized protein LOC102358965 isoform	7,00E-25
gALL_v3_c22627	3	3	-	-	gb|ACO12427.1|	Transmembrane protein C18orf45 [Lepeophtheirus salmonis]	3,00E-11
gALL_v3_c22628	3	1	-	-	-	-	-
gALL_v3_c22629	0	4	-	-	-	-	-
gALL_v3_c2263	17	16	gnl|CDD|128525	smart00229, RasGEFN, Guanir	ref|XP_003708241.1|	PREDICTED: protein son of sevenless-like [Megachile rotundata]	0
gALL_v3_c22631	3	9	gnl|CDD|34843	COG5246, PRP11, Splicing fact	gb|EFN78538.1|	Splicing factor 3A subunit 2 [Harpegnathos saltator]	5,00E-62
gALL_v3_c22632	1	3	-	-	-	-	-
gALL_v3_c22633	2	4	gnl|CDD|176302	cd01226, PH_exo84, Exocyst c	ref|XP_001810409.1|	PREDICTED: similar to exocyst complex 84-kDa subunit [Tribolium castaneum]	3,00E-32
gALL_v3_c22634	0	4	-	-	ref|XP_004923651.1|	PREDICTED: nitrogen permease regulator 2-like protein-like [Bombyx mori]	5,00E-10
gALL_v3_c22636	2	2	-	-	-	-	-
gALL_v3_c22637	2	2	-	-	gb|EHJ66555.1|	hypothetical protein KGM_20679 [Danaus plexippus]	4,00E-15
gALL_v3_c22638	6	3	-	-	-	-	-
gALL_v3_c22639	4	2	gnl|CDD|30130	cd00354, FBPase, Fructose-1,	gb|AGM32987.1|	fructose-1,6-bisphosphatase [Coptotermes formosanus]	1,00E-117
gALL_v3_c2264	12	15	-	-	gb|EFA11049.1|	hypothetical protein TcasGA2_TC004643 [Tribolium castaneum]	5,00E-96
gALL_v3_c22640	2	5	gnl|CDD|143833	pfam00046, Homeobox, Hom	emb|CBL87030.1|	ptx protein [Tribolium castaneum]	1,00E-20
gALL_v3_c22641	2	1	-	-	-	-	-
gALL_v3_c22642	0	3	-	-	-	-	-

gALL_v3_c22644	1	4	gnl|CDD|128505	smart00209, TSP1, Thrombosp	gb|EFX81744.1|	hypothetical protein DAPPUDRAFT_303360 [Daphnia pulex]	4,00E-96
gALL_v3_c22645	2	3	-	-	-	-	-
gALL_v3_c22646	4	2	-	-	gb|EFN76211.1|	Mediator of RNA polymerase II transcription subunit 12 [Harpegnathos saltator]	1,00E-29
gALL_v3_c22647	2	1	-	-	gb|ENN82460.1|	hypothetical protein YQE_01165, partial [Dendroctonus ponderosae]	3,00E-18
gALL_v3_c22648	2	2	-	-	-	-	-
gALL_v3_c2265	7	18	-	-	-	-	-
gALL_v3_c22652	3	4	-	-	-	-	-
gALL_v3_c22653	3	5	-	-	-	-	-
gALL_v3_c22654	3	3	-	-	-	-	-
gALL_v3_c22655	0	5	-	-	-	-	-
gALL_v3_c22656	1	3	-	-	-	-	-
gALL_v3_c22658	2	2	-	-	-	-	-
gALL_v3_c22659	2	2	-	-	-	-	-
gALL_v3_c22660	0	4	-	-	-	-	-
gALL_v3_c22661	0	6	-	-	ref|XP_006610918.1|	PREDICTED: heterogeneous nuclear ribonucleoprotein H2-like isoform X1 [Apis dorsata] ref|XP_006610919.1| PREDICTED: heterogeneous nuclear ribonucleoprotein H2-lik	2,00E-73
gALL_v3_c22662	0	4	-	-	-	-	-
gALL_v3_c22663	4	4	-	-	-	-	-
gALL_v3_c22664	3	1	-	-	-	-	-
gALL_v3_c22665	4	2	-	-	gb|ENN70165.1|	hypothetical protein YQE_00024, partial [Dendroctonus ponderosae]	5,00E-80
gALL_v3_c22666	3	1	-	-	-	-	-
gALL_v3_c22667	5	2	gnl|CDD|191197	pfam05127, DUF699, Putative	gb|EFA01833.1|	hypothetical protein TcasGA2_TC007435 [Tribolium castaneum]	3,00E-83
gALL_v3_c22668	0	3	-	-	-	-	-
gALL_v3_c22669	4	5	-	-	-	-	-
gALL_v3_c2267	15	15	-	-	ref|XP_003706754.1|	PREDICTED: piggyBac transposable element-derived protein 4-like [Megachile rotundata]	3,00E-09
gALL_v3_c22670	11	10	-	-	-	-	-
gALL_v3_c22671	1	4	gnl|CDD|29257	cd00200, WD40, WD40 domai	ref|XP_003700651.1|	PREDICTED: syntaxin-binding protein 5 isoform 1 [Megachile rotundata]	1,00E-148
gALL_v3_c22672	3	3	-	-	-	-	-
gALL_v3_c22673	5	5	gnl|CDD|29006	cd02396, PCBP_like_KH, K hor	ref|XP_006610129.1|	PREDICTED: heterogeneous nuclear ribonucleoprotein K-like isoform X5 [Apis dorsata]	2,00E-27
gALL_v3_c22674	1	4	-	-	-	-	-
gALL_v3_c22675	5	3	gnl|CDD|192512	pfam10280, Med11, Mediator	ref|XP_002424714.1|	conserved hypothetical protein [Pediculus humanus corporis] gb|EEB11976.1| conserved hypothetical protein [Pediculus humanus corporis]	4,00E-63
gALL_v3_c22676	3	1	-	-	ref|XP_003700739.1|	PREDICTED: monocarboxylate transporter 12-B-like [Megachile rotundata]	8,00E-16
gALL_v3_c22678	2	4	-	-	-	-	-
gALL_v3_c22679	2	1	-	-	-	-	-
gALL_v3_c22680	4	4	-	-	-	-	-
gALL_v3_c22681	3	2	-	-	-	-	-
gALL_v3_c22682	3	7	-	-	-	-	-
gALL_v3_c22683	3	1	-	-	gb|EFN83221.1|	Probable nucleoporin Nup54 [Harpegnathos saltator]	2,00E-26
gALL_v3_c22684	0	3	-	-	ref|XP_004209437.1|	PREDICTED: uncharacterized protein LOC101238983 [Hydra vulgaris]	3,00E-06
gALL_v3_c22685	0	4	-	-	-	-	-
gALL_v3_c22686	1	3	-	-	-	-	-
gALL_v3_c22687	1	3	-	-	-	-	-
gALL_v3_c22688	1	6	-	-	-	-	-
gALL_v3_c22689	2	3	-	-	-	-	-
gALL_v3_c2269	4	5	gnl|CDD|128349	smart00034, CLECT, C-type lec	ref|XP_003694618.1|	PREDICTED: contactin-like [Apis florea]	2,00E-81
gALL_v3_c22690	0	3	-	-	-	-	-
gALL_v3_c22692	4	5	-	-	-	-	-
gALL_v3_c22693	0	3	-	-	-	-	-
gALL_v3_c22695	3	3	-	-	-	-	-
gALL_v3_c22696	3	4	-	-	-	-	-
gALL_v3_c22697	0	4	-	-	-	-	-
gALL_v3_c22698	1	5	-	-	-	-	-
gALL_v3_c22699	1	4	gnl|CDD|190596	pfam03311, Cornichon, Cornic	ref|XP_001604514.1|	PREDICTED: protein cornichon homolog 4 [Nasonia vitripennis]	1,00E-14
gALL_v3_c227	13	9	gnl|CDD|73156	cd01650, RT_nLTR_like, RT_nL	gb|EFA12306.1|	hypothetical protein TcasGA2_TC016101 [Tribolium castaneum]	2,00E-40
gALL_v3_c22700	0	4	-	-	-	-	-
gALL_v3_c22701	1	4	-	-	gb|EFN86595.1|	Uncharacterized protein KIAA1109 [Harpegnathos saltator]	7,00E-33
gALL_v3_c22702	1	3	-	-	ref|XP_002430986.1|	zinc finger protein DHHC domain containing protein, putative [Pediculus humanus corporis] gb|EEB18248.1| zinc finger protein DHHC domain containing protein, putative	3,00E-33
gALL_v3_c22703	3	1	-	-	-	-	-
gALL_v3_c22704	6	9	-	-	-	-	-
gALL_v3_c22705	1	2	-	-	-	-	-
gALL_v3_c22706	0	4	-	-	-	-	-
gALL_v3_c22708	2	5	gnl|CDD|30053	cd01310, TatD_DNAse, TatD lil	ref|XP_975374.1|	PREDICTED: similar to AGAP002253-PA [Tribolium castaneum] gb|EFA07007.1| hypothetical protein TcasGA2_TC009977 [Tribolium castaneum]	1,00E-118
gALL_v3_c22709	3	1	-	-	-	-	-
gALL_v3_c2271	6	6	-	-	ref|XP_004929373.1|	PREDICTED: uncharacterized protein LOC101742112 [Bombyx mori]	3,00E-31
gALL_v3_c22711	1	5	gnl|CDD|179042	PRK00476, aspS, aspartyl-tRNA	ref|XP_966901.2|	PREDICTED: similar to aspartyl-tRNA synthetase [Tribolium castaneum]	4,00E-83
gALL_v3_c22712	3	5	gnl|CDD|151166	pfam10660, MitoNEET_N, Iron	gb|AGM32455.1|	CDGSH iron-sulfur domain-containing protein [Coptotermes formosanus]	7,00E-86
gALL_v3_c22713	0	3	-	-	-	-	-
gALL_v3_c22716	3	5	gnl|CDD|148646	pfam07159, DUF1394, Protein	ref|XP_002432052.1|	protein FAM49B, putative [Pediculus humanus corporis] gb|EEB19314.1| protein FAM49B, putative [Pediculus humanus corporis]	1,00E-124
gALL_v3_c22717	2	2	-	-	-	-	-
gALL_v3_c22718	6	12	-	-	gb|AGM32707.1|	hypothetical protein, partial [Coptotermes formosanus]	7,00E-09
gALL_v3_c2272	11	8	gnl|CDD|34495	COG4886, COG4886, Leucine-r	ref|XP_006571220.1|	PREDICTED: leucine-rich repeat and calponin homology domain-containing protein 1-like isoform X1 [Apis mellifera]	1,00E-130
gALL_v3_c22720	2	6	-	-	-	-	-
gALL_v3_c22721	3	4	-	-	-	-	-
gALL_v3_c22722	0	5	-	-	-	-	-
gALL_v3_c22723	2	3	-	-	-	-	-
gALL_v3_c22724	2	2	-	-	-	-	-
gALL_v3_c22725	1	7	-	-	-	-	-
gALL_v3_c22726	3	3	-	-	-	-	-
gALL_v3_c22727	2	2	-	-	-	-	-
gALL_v3_c22729	4	3	-	-	-	-	-
gALL_v3_c2273	8	9	-	-	ref|XP_006614921.1|	PREDICTED: ER membrane protein complex subunit 2-B-like isoform X1 [Apis dorsata] ref|XP_006614922.1| PREDICTED: ER membrane protein complex subunit 2-B-like iso	2,00E-96
gALL_v3_c22730	2	4	-	-	dbj|BAH72907.1|	ACYPI005967 [Acyrthosiphon pisum]	2,00E-15
gALL_v3_c22731	4	6	-	-	-	-	-
gALL_v3_c22732	0	5	-	-	-	-	-
gALL_v3_c22734	0	5	-	-	-	-	-
gALL_v3_c22735	0	4	-	-	-	-	-
gALL_v3_c22736	7	4	-	-	-	-	-
gALL_v3_c22737	1	5	-	-	-	-	-
gALL_v3_c22738	1	2	gnl|CDD|146692	pfam04184, ST7, ST7 protein.	ref|XP_003399913.1|	PREDICTED: protein ST7 homolog [Bombus terrestris]	2,00E-90
gALL_v3_c22739	7	16	-	-	-	-	-
gALL_v3_c22740	3	1	-	-	-	-	-
gALL_v3_c22741	4	3	-	-	-	-	-
gALL_v3_c22742	3	5	-	-	-	-	-
gALL_v3_c22743	1	3	-	-	gb|EFN80086.1|	Pecanex-like protein 1 [Harpegnathos saltator]	3,00E-32
gALL_v3_c22744	1	4	gnl|CDD|191857	pfam07808, RED_N, RED-like p	ref|XP_002426740.1|	axoneme-associated protein mst101, putative [Pediculus humanus corporis] gb|EEB14002.1| axoneme-associated protein mst101, putative [Pediculus humanus corporis]	1,00E-56
gALL_v3_c22745	0	4	-	-	-	-	-
gALL_v3_c22746	1	3	-	-	-	-	-
gALL_v3_c22747	2	4	-	-	-	-	-
gALL_v3_c22748	1	4	-	-	ref|XP_002597259.1|	hypothetical protein BRAFLDRAFT_203459 [Branchiostoma floridae] gb|EEN53271.1| hypothetical protein BRAFLDRAFT_203459 [Branchiostoma floridae]	1,00E-19
gALL_v3_c22749	1	3	-	-	gb|EFN76653.1|	Kinesin-like protein KIF13A [Harpegnathos saltator]	9,00E-72
gALL_v3_c2275	9	17	-	-	-	-	-
gALL_v3_c22750	3	1	-	-	-	-	-
gALL_v3_c22751	2	1	-	-	-	-	-
gALL_v3_c22752	0	6	gnl|CDD|73156	cd01650, RT_nLTR_like, RT_nL	gb|EEZ99812.1|	hypothetical protein TcasGA2_TC002592 [Tribolium castaneum]	8,00E-34
gALL_v3_c22755	3	1	-	-	-	-	-
gALL_v3_c22756	0	3	-	-	-	-	-
gALL_v3_c22757	4	2	-	-	-	-	-
gALL_v3_c22758	3	6	gnl|CDD|162101	TIGR00911, 2A0308, L-type an	gb|ETN58413.1|	cationic amino acid transporter [Anopheles darlingi]	1,00E-116
gALL_v3_c22759	1	5	-	-	-	-	-
gALL_v3_c2276	5	4	-	-	ref|XP_001608013.1|	PREDICTED: hypothetical protein LOC100123516 isoform 1 [Nasonia vitripennis]	4,00E-44
gALL_v3_c22760	1	3	-	-	ref|XP_006612702.1|	PREDICTED: von Willebrand factor A domain-containing protein 8-like [Apis dorsata]	6,00E-52
gALL_v3_c22761	3	1	-	-	-	-	-
gALL_v3_c22762	1	2	-	-	ref|XP_002607840.1|	hypothetical protein BRAFLDRAFT_199699 [Branchiostoma floridae] gb|EEN63850.1| hypothetical protein BRAFLDRAFT_199699 [Branchiostoma floridae]	2,00E-08
gALL_v3_c22763	1	3	-	-	gb|EFZ18518.1|	hypothetical protein SINV_14949 [Solenopsis invicta]	3,00E-32
gALL_v3_c22764	1	3	-	-	ref|XP_004472198.1|	PREDICTED: zinc finger protein 157 isoform 1 [Dasypus novemcinctus] ref|XP_004472199.1| PREDICTED: zinc finger protein 157 isoform 2 [Dasypus novemcinctus] ref|XP_	1,00E-24
gALL_v3_c22765	1	3	-	-	-	-	-
gALL_v3_c22768	2	3	-	-	-	-	-
gALL_v3_c22769	3	3	-	-	-	-	-
gALL_v3_c2277	31	28	-	-	-	-	-
gALL_v3_c22770	1	2	-	-	gb|ETN61425.1|	glucosamine-fructose-6-phosphate aminotransferase [Anopheles darlingi]	4,00E-17
gALL_v3_c22771	2	2	gnl|CDD|184309	PRK13762, PRK13762, tRNA-m	gb|AAS07520.1|	unknown [Homo sapiens]	5,00E-73
gALL_v3_c22772	2	3	-	-	gb|EFN75044.1|	Uncharacterized protein KIAA1109 [Camponotus floridanus]	4,00E-51
gALL_v3_c22773	2	2	-	-	-	-	-
gALL_v3_c22774	3	1	-	-	-	-	-
gALL_v3_c22777	6	12	gnl|CDD|178621	PLN03072, PLN03072, 60S ribo	gb|AAX62480.1|	ribosomal protein L12 [Lysiphlebus testaceipes] gb|AAX62481.1| ribosomal protein L12 variant 1 [Lysiphlebus testaceipes]	6,00E-95
gALL_v3_c22778	2	3	-	-	gb|EGI58975.1|	Baculoviral IAP repeat-containing protein 6 [Acromyrmex echinatior]	4,00E-41
gALL_v3_c22779	1	3	-	-	-	-	-
gALL_v3_c2278	4	5	-	-	-	-	-
gALL_v3_c22780	1	3	-	-	ref|XP_001606169.2|	PREDICTED: hypothetical protein LOC100122558 [Nasonia vitripennis]	1,00E-15
gALL_v3_c22781	1	3	-	-	-	-	-
gALL_v3_c22782	0	4	-	-	-	-	-
gALL_v3_c22783	1	2	-	-	-	-	-
gALL_v3_c22784	2	2	-	-	gb|EFN76355.1|	hypothetical protein EAI_02827 [Harpegnathos saltator]	1,00E-22
gALL_v3_c22785	3	1	-	-	-	-	-
gALL_v3_c22789	1	2	gnl|CDD|144338	pfam00698, Acyl_transf_1, Ac	gb|ENN73526.1|	hypothetical protein YQE_09777, partial [Dendroctonus ponderosae]	2,00E-29
gALL_v3_c2279	8	8	gnl|CDD|140328	PTZ00307, PTZ00307, ethanol	gb|EFN67042.1|	Ethanolaminephosphotransferase 1 [Camponotus floridanus]	1,00E-127
gALL_v3_c22790	3	1	-	-	-	-	-
gALL_v3_c22791	3	7	-	-	-	-	-
gALL_v3_c22792	0	4	-	-	-	-	-
gALL_v3_c22795	3	4	-	-	-	-	-
gALL_v3_c22796	0	4	-	-	-	-	-
gALL_v3_c22797	0	3	gnl|CDD|107368	cd06373, PBP1_NPR_like, Liga	ref|XP_003692135.1|	PREDICTED: atrial natriuretic peptide receptor 1-like [Apis florea]	7,00E-54
gALL_v3_c22798	2	6	gnl|CDD|73272	cd01493, APPBP1_RUB, Ubiqu	gb|EFZ22238.1|	hypothetical protein SINV_00190 [Solenopsis invicta]	1,00E-93
gALL_v3_c228	15	28	gnl|CDD|191140	pfam04960, Glutaminase, Glu	gb|EFN75035.1|	Glutaminase kidney isoform, mitochondrial [Camponotus floridanus]	0
gALL_v3_c2280	14	14	-	-	-	-	-

gALL_v3_c22800	1	4	-	-	-	-	-
gALL_v3_c22802	1	3	-	-	-	-	-
gALL_v3_c22803	2	2	gnl|CDD|133307	cd04107, Rab32_Rab38, Rab3	ref|XP_002423809.1|	GTP-binding nuclear protein RAN1, putative [Pediculus humanus corporis] gb|EEB11071.1| GTP-binding nuclear protein RAN1, putative [Pediculus humanus corporis]	4,00E-81
gALL_v3_c22804	1	3	-	-	-	-	-
gALL_v3_c22805	0	4	-	-	-	-	-
gALL_v3_c22807	1	2	-	-	-	-	-
gALL_v3_c22808	2	2	-	-	-	-	-
gALL_v3_c22809	0	4	-	-	-	-	-
gALL_v3_c2281	8	7	gnl|CDD|185622	PTZ00443, PTZ00443, Thiored	gb|AGM32999.1|	thioredoxin domain-containing protein, partial [Coptotermes formosanus]	0
gALL_v3_c22810	2	1	-	-	-	-	-
gALL_v3_c22811	1	3	-	-	-	-	-
gALL_v3_c22812	3	3	-	-	ref|XP_002010918.1|	GI21440 [Drosophila mojavensis] gb|EDW06573.1| GI21440 [Drosophila mojavensis]	2,00E-17
gALL_v3_c22813	3	2	-	-	-	-	-
gALL_v3_c22816	2	3	-	-	-	-	-
gALL_v3_c22817	5	9	gnl|CDD|29796	cd02897, A2M_2, Proteins sim	gb|EFN68790.1|	CD109 antigen [Camponotus floridanus]	1,00E-114
gALL_v3_c22818	3	2	-	-	-	-	-
gALL_v3_c22819	3	1	-	-	-	-	-
gALL_v3_c2282	5	5	-	-	-	-	-
gALL_v3_c22821	2	2	-	-	-	-	-
gALL_v3_c22823	4	8	-	-	-	-	-
gALL_v3_c22824	2	1	-	-	-	-	-
gALL_v3_c22826	6	3	-	-	-	-	-
gALL_v3_c22827	3	1	-	-	-	-	-
gALL_v3_c22829	0	4	-	-	-	-	-
gALL_v3_c2283	8	14	-	-	-	-	-
gALL_v3_c22830	3	3	gnl|CDD|119351	cd02872, GH18_chitolectin_ch	gb|ETN67862.1|	hypothetical protein AND_000310 [Anopheles darlingi]	1,00E-144
gALL_v3_c22832	6	8	gnl|CDD|178092	PLN02474, PLN02474, UTP--gl	ref|XP_004029366.1|	PREDICTED: UTP--glucose-1-phosphate uridylyltransferase-like [Gorilla gorilla gorilla]	4,00E-28
gALL_v3_c22833	2	2	-	-	-	-	-
gALL_v3_c22835	1	2	-	-	-	-	-
gALL_v3_c22836	2	7	-	-	ref|XP_005994949.1|	PREDICTED: UV-stimulated scaffold protein A [Latimeria chalumnae]	9,00E-46
gALL_v3_c22837	1	4	-	-	-	-	-
gALL_v3_c22839	3	3	-	-	-	-	-
gALL_v3_c2284	5	10	-	-	-	-	-
gALL_v3_c22840	2	1	-	-	-	-	-
gALL_v3_c22841	5	5	-	-	ref|XP_006617518.1|	PREDICTED: polycomb group protein Pc-like [Apis dorsata]	8,00E-46
gALL_v3_c22842	3	1	-	-	-	-	-
gALL_v3_c22843	4	2	-	-	-	-	-
gALL_v3_c22844	1	3	-	-	gb|ACN10408.1|	Ubiquitin carboxyl-terminal hydrolase 15 [Salmo salar] gb|ACN12773.1| Ubiquitin carboxyl-terminal hydrolase 15 [Salmo salar]	1,00E-47
gALL_v3_c22846	3	1	-	-	-	-	-
gALL_v3_c22847	3	11	-	-	gb|AGM32707.1|	hypothetical protein, partial [Coptotermes formosanus]	1,00E-74
gALL_v3_c22848	0	4	-	-	-	-	-
gALL_v3_c22849	6	5	gnl|CDD|192470	pfam10176, DUF2370, Protein	ref|XP_006622317.1|	PREDICTED: NEDD4 family-interacting protein 1-like [Apis dorsata]	7,00E-71
gALL_v3_c2285	5	8	-	-	-	-	-
gALL_v3_c22850	0	3	-	-	-	-	-
gALL_v3_c22851	0	4	-	-	-	-	-
gALL_v3_c22852	2	1	-	-	-	-	-
gALL_v3_c22854	3	3	-	-	-	-	-
gALL_v3_c22855	2	2	-	-	gb|AAC05332.1|	extra sex combs [Schistocerca americana]	2,00E-33
gALL_v3_c22856	2	2	-	-	-	-	-
gALL_v3_c22857	2	2	-	-	-	-	-
gALL_v3_c22858	0	4	-	-	-	-	-
gALL_v3_c22859	3	7	gnl|CDD|144027	pfam00287, Na_K-ATPase, Soc	gb|AHH35013.1|	Na+/K+-ATPase beta2 [Locusta migratoria]	5,00E-82
gALL_v3_c2286	15	22	gnl|CDD|191810	pfam07679, I-set, Immunoglol	sp|P22648.2|FAS2_SCHA	RecName: Full=Fasciclin-2; AltName: Full=Fasciclin II; Short=FAS II; Flags: Precursor	1,00E-178
gALL_v3_c22860	3	1	-	-	-	-	-
gALL_v3_c22861	0	4	-	-	-	-	-
gALL_v3_c22863	1	5	-	-	-	-	-
gALL_v3_c22864	0	3	-	-	-	-	-
gALL_v3_c22865	2	6	-	-	ref|XP_003706066.1|	PREDICTED: kinase suppressor of Ras 1-like [Megachile rotundata]	3,00E-73
gALL_v3_c22866	2	5	-	-	-	-	-
gALL_v3_c22867	1	7	gnl|CDD|190071	pfam01694, Rhomboid, Rhom	ref|XP_970266.1|	PREDICTED: similar to rhomboid [Tribolium castaneum]	3,00E-94
gALL_v3_c22868	2	3	-	-	ref|XP_003244974.1|	PREDICTED: echinoderm microtubule-associated protein-like 1-like isoform 2 [Acyrthosiphon pisum]	6,00E-56
gALL_v3_c22869	2	9	-	-	-	-	-
gALL_v3_c22870	4	5	-	-	gb|ENN81916.1|	hypothetical protein YQE_01628, partial [Dendroctonus ponderosae]	3,00E-83
gALL_v3_c22871	2	2	-	-	-	-	-
gALL_v3_c22872	0	3	-	-	-	-	-
gALL_v3_c22873	8	8	-	-	gb|ABC84492.1|	heat shock protein 20.5 [Locusta migratoria]	9,00E-25
gALL_v3_c22875	0	5	-	-	gb|EFA09519.1|	hypothetical protein TcasGA2_TC011621 [Tribolium castaneum]	2,00E-16
gALL_v3_c22876	0	3	-	-	-	-	-
gALL_v3_c22877	1	2	-	-	-	-	-
gALL_v3_c22878	2	4	-	-	-	-	-
gALL_v3_c22879	0	3	-	-	-	-	-
gALL_v3_c2288	6	7	gnl|CDD|143803	pfam00012, HSP70, Hsp70 prc	gb|ACS75353.1|	ER protein gp78 [Locusta migratoria]	1,00E-149
gALL_v3_c22880	4	6	-	-	ref|XP_002430351.1|	conserved hypothetical protein [Pediculus humanus corporis] gb|EEB17613.1| conserved hypothetical protein [Pediculus humanus corporis]	2,00E-34
gALL_v3_c22881	2	2	-	-	-	-	-
gALL_v3_c22883	3	1	-	-	-	-	-
gALL_v3_c22885	4	3	-	-	-	-	-
gALL_v3_c22886	2	3	gnl|CDD|73065	cd02659, peptidase_C19C, A s	gb|EGI68021.1|	Ubiquitin carboxyl-terminal hydrolase 34 [Acromyrmex echinatior]	2,00E-97
gALL_v3_c22887	1	4	-	-	-	-	-
gALL_v3_c22888	3	4	-	-	-	-	-
gALL_v3_c22889	5	8	-	-	ref|XP_972543.2|	PREDICTED: similar to split ends CG18497-PA [Tribolium castaneum]	1,00E-48
gALL_v3_c2289	12	11	-	-	-	-	-
gALL_v3_c22890	2	2	-	-	-	-	-
gALL_v3_c22892	2	1	gnl|CDD|189372	pfam00067, p450, Cytochrom	gb|ABB51130.2|	cytochrome p450 family 4 [Brontispa longissima]	1,00E-32
gALL_v3_c22894	2	6	-	-	gb|EFN82169.1|	hypothetical protein EAI_09568 [Harpegnathos saltator]	2,00E-13
gALL_v3_c22895	0	5	-	-	-	-	-
gALL_v3_c22896	3	3	-	-	gb|EFN75656.1|	hypothetical protein EAI_04958 [Harpegnathos saltator]	2,00E-06
gALL_v3_c22897	2	3	-	-	-	-	-
gALL_v3_c22898	3	3	-	-	ref|XP_002423198.1|	conserved hypothetical protein [Pediculus humanus corporis] gb|EEB10460.1| conserved hypothetical protein [Pediculus humanus corporis]	1,00E-27
gALL_v3_c22899	1	6	-	-	-	-	-
gALL_v3_c2290	16	17	gnl|CDD|173412	PTZ00121, PTZ00121, MAEBL;	gb|EFZ09216.1|	hypothetical protein SINV_06109 [Solenopsis invicta]	1,00E-09
gALL_v3_c22900	10	17	-	-	-	-	-
gALL_v3_c22901	3	1	-	-	-	-	-
gALL_v3_c22902	1	4	-	-	-	-	-
gALL_v3_c22903	8	10	-	-	ref|XP_003012611.1|	conserved hypothetical protein [Arthroderma benhamiae CBS 112371] gb|EFE31971.1| conserved hypothetical protein [Arthroderma benhamiae CBS 112371]	3,00E-10
gALL_v3_c22904	3	1	-	-	-	-	-
gALL_v3_c22905	4	2	gnl|CDD|162054	TIGR00815, sulP, high affinity s	ref|XP_002099330.1|	GE23427 [Drosophila yakuba] gb|EDW99042.1| GE23427 [Drosophila yakuba]	4,00E-29
gALL_v3_c22906	1	4	-	-	gb|EFN67634.1|	Putative uncharacterized transposon-derived protein F52C9.6 [Camponotus floridanus]	5,00E-15
gALL_v3_c22907	3	1	-	-	-	-	-
gALL_v3_c22909	3	1	-	-	-	-	-
gALL_v3_c2291	12	10	gnl|CDD|73156	cd01650, RT_nLTR_like, RT_nL	dbj|BAA07467.1|	ORF2 [Bombyx mori]	2,00E-64
gALL_v3_c22910	2	2	-	-	-	-	-
gALL_v3_c22911	0	5	gnl|CDD|187586	cd05325, carb_red_sniffer_like	ref|XP_003702515.1|	PREDICTED: C-factor-like [Megachile rotundata]	2,00E-20
gALL_v3_c22912	2	2	-	-	ref|XP_003698770.1|	PREDICTED: THO complex subunit 6 homolog [Apis florea]	6,00E-32
gALL_v3_c22913	1	3	gnl|CDD|29805	cd00707, Pancreat_lipase_like	ref|XP_004923072.1|	PREDICTED: pancreatic triacylglycerol lipase-like [Bombyx mori]	5,00E-37
gALL_v3_c22914	3	1	-	-	-	-	-
gALL_v3_c22915	0	4	-	-	-	-	-
gALL_v3_c22916	6	5	-	-	-	-	-
gALL_v3_c22917	3	1	-	-	-	-	-
gALL_v3_c22918	3	1	-	-	ref|XP_970721.2|	PREDICTED: similar to iPLA-2 [Tribolium castaneum] gb|EFA02006.1| hypothetical protein TcasGA2_TC007628 [Tribolium castaneum]	2,00E-22
gALL_v3_c22919	3	2	-	-	-	-	-
gALL_v3_c2292	4	10	-	-	-	-	-
gALL_v3_c22921	3	1	-	-	-	-	-
gALL_v3_c22922	0	3	-	-	-	-	-
gALL_v3_c22923	2	2	-	-	-	-	-
gALL_v3_c22925	6	4	-	-	-	-	-
gALL_v3_c22926	5	2	gnl|CDD|190906	pfam04194, PDCD2_C, Progra	gb|EEZ98118.1|	hypothetical protein TcasGA2_TC000536 [Tribolium castaneum]	9,00E-40
gALL_v3_c22927	3	4	-	-	-	-	-
gALL_v3_c22928	0	4	-	-	gb|EFN75185.1|	Uncharacterized aminotransferase SSO0104 [Harpegnathos saltator]	4,00E-15
gALL_v3_c22929	3	1	-	-	-	-	-
gALL_v3_c2293	11	7	-	-	gb|ELR46283.1|	hypothetical protein M91_01358, partial [Bos mutus]	6,00E-12
gALL_v3_c22930	5	7	-	-	-	-	-
gALL_v3_c22931	2	2	-	-	ref|XP_006623608.1|	PREDICTED: ectopic P granules protein 5 homolog isoform X1 [Apis dorsata]	8,00E-66
gALL_v3_c22932	1	2	-	-	-	-	-
gALL_v3_c22933	3	1	-	-	-	-	-
gALL_v3_c22934	1	2	-	-	-	-	-
gALL_v3_c22935	0	4	-	-	-	-	-
gALL_v3_c22936	3	3	-	-	-	-	-
gALL_v3_c22937	1	3	-	-	-	-	-
gALL_v3_c22938	3	1	gnl|CDD|58574	cd04273, ZnMc_ADAMTS_like	gb|EFA06592.1|	hypothetical protein TcasGA2_TC009506 [Tribolium castaneum]	5,00E-14
gALL_v3_c22939	2	1	-	-	-	-	-
gALL_v3_c22940	3	2	-	-	-	-	-
gALL_v3_c22941	4	2	-	-	-	-	-
gALL_v3_c22942	3	2	-	-	-	-	-
gALL_v3_c22943	1	3	-	-	-	-	-
gALL_v3_c22944	2	4	-	-	-	-	-
gALL_v3_c22945	6	6	-	-	ref|XP_006614061.1|	PREDICTED: uncharacterized protein LOC102674687 [Apis dorsata]	5,00E-59
gALL_v3_c22946	2	9	-	-	-	-	-
gALL_v3_c22947	2	2	-	-	-	-	-
gALL_v3_c22948	1	2	-	-	-	-	-
gALL_v3_c22949	2	4	-	-	-	-	-
gALL_v3_c2295	14	14	-	-	ref|XP_002427393.1|	vacuole membrane protein, putative [Pediculus humanus corporis] gb|EEB14655.1| vacuole membrane protein, putative [Pediculus humanus corporis]	1,00E-127
gALL_v3_c22950	1	4	-	-	-	-	-
gALL_v3_c22952	2	2	-	-	-	-	-
gALL_v3_c22953	1	3	-	-	-	-	-

gALL_v3_c22954	2	2	gnl|CDD|189544	pfam00431, CUB, CUB domain	ref|XP_001843996.1|	cubilin [Culex quinquefasciatus] gb|EDS35495.1| cubilin [Culex quinquefasciatus]	3,00E-33
gALL_v3_c22955	2	2	-	-	-	-	-
gALL_v3_c22956	3	1	gnl|CDD|189360	pfam00041, fn3, Fibronectin ty	ref|XP_001811323.1|	PREDICTED: similar to sdk-P1 [Tribolium castaneum]	3,00E-79
gALL_v3_c22957	1	3	-	-	gb|EFZ20099.1|	hypothetical protein SINV_06790 [Solenopsis invicta]	8,00E-42
gALL_v3_c22958	2	4	-	-	-	-	-
gALL_v3_c22959	1	6	-	-	-	-	-
gALL_v3_c2296	6	10	-	-	-	-	-
gALL_v3_c22960	0	4	-	-	gb|EFA11628.1|	hypothetical protein TcasGA2_TC000006 [Tribolium castaneum]	3,00E-08
gALL_v3_c22961	2	2	-	-	-	-	-
gALL_v3_c22962	3	2	-	-	-	-	-
gALL_v3_c22963	3	1	-	-	-	-	-
gALL_v3_c22964	3	4	-	-	-	-	-
gALL_v3_c22966	1	3	-	-	-	-	-
gALL_v3_c22967	1	3	-	-	gb|AAL25404.1|	LD21275p [Drosophila melanogaster]	1,00E-53
gALL_v3_c22968	2	2	-	-	-	-	-
gALL_v3_c22969	3	1	-	-	-	-	-
gALL_v3_c2297	5	7	-	-	gb|AGM32136.1|	hypothetical protein [Coptotermes formosanus]	2,00E-10
gALL_v3_c22970	3	3	-	-	-	-	-
gALL_v3_c22971	3	3	gnl|CDD|109126	pfam00057, Ldl_recept_a, Low	ref|XP_003486382.1|	PREDICTED: LOW QUALITY PROTEIN: basement membrane-specific heparan sulfate proteoglycan core protein-like [Bombus impatiens]	1,00E-108
gALL_v3_c22972	3	5	-	-	-	-	-
gALL_v3_c22973	1	4	-	-	-	-	-
gALL_v3_c22974	2	4	-	-	-	-	-
gALL_v3_c22975	2	3	-	-	-	-	-
gALL_v3_c22976	4	7	-	-	-	-	-
gALL_v3_c22977	3	3	gnl|CDD|185557	PTZ00327, PTZ00327, eukaryo	emb|CAX17290.1|	translation initiation factor 2, gamma subunit [Medauroidea extradentata]	1,00E-174
gALL_v3_c22978	1	5	-	-	gb|EFN69063.1|	Zinc finger protein 180 [Camponotus floridanus]	2,00E-56
gALL_v3_c22979	5	4	-	-	ref|XP_004082589.1|	PREDICTED: tigger transposable element-derived protein 1-like [Oryzias latipes]	4,00E-06
gALL_v3_c22980	6	5	-	-	gb|EFN67634.1|	Putative uncharacterized transposon-derived protein F52C9.6 [Camponotus floridanus]	1,00E-20
gALL_v3_c22981	2	2	-	-	-	-	-
gALL_v3_c22982	3	2	-	-	gb|EFZ20434.1|	hypothetical protein SINV_01500 [Solenopsis invicta]	1,00E-50
gALL_v3_c22983	3	2	-	-	-	-	-
gALL_v3_c22985	3	2	-	-	-	-	-
gALL_v3_c22986	3	3	-	-	ref|XP_002426659.1|	WD-repeat protein, putative [Pediculus humanus corporis] gb|EEB13921.1| WD-repeat protein, putative [Pediculus humanus corporis]	4,00E-35
gALL_v3_c22987	3	1	gnl|CDD|128336	smart00020, Tryp_SPc, Trypsin	ref|XP_003692297.1|	PREDICTED: LOW QUALITY PROTEIN: mite allergen Der p 3-like [Apis florea]	8,00E-22
gALL_v3_c22988	3	2	-	-	-	-	-
gALL_v3_c22989	2	5	-	-	-	-	-
gALL_v3_c2299	13	16	gnl|CDD|29970	cd01085, APP, X-Prolyl Aminop	ref|XP_974698.1|	PREDICTED: similar to X-prolyl aminopeptidase (aminopeptidase P) 1, soluble [Tribolium castaneum] gb|EFA04188.1| hypothetical protein TcasGA2_TC014437 [Tribolium c	1,00E-169
gALL_v3_c22991	3	7	-	-	-	-	-
gALL_v3_c22992	3	2	-	-	-	-	-
gALL_v3_c22993	4	2	-	-	-	-	-
gALL_v3_c22994	1	3	-	-	-	-	-
gALL_v3_c22996	3	1	-	-	-	-	-
gALL_v3_c22997	2	2	-	-	-	-	-
gALL_v3_c22998	4	3	-	-	ref|XP_004930525.1|	PREDICTED: uncharacterized protein LOC101740833 [Bombyx mori]	2,00E-27
gALL_v3_c230	4	6	-	-	ref|XP_314560.4|	AGAP010595-PA [Anopheles gambiae str. PEST] gb|EAA09894.4| AGAP010595-PA [Anopheles gambiae str. PEST]	4,00E-13
gALL_v3_c2300	16	21	gnl|CDD|34681	COG5077, COG5077, Ubiquitin	ref|XP_006624903.1|	PREDICTED: ubiquitin carboxyl-terminal hydrolase 7-like isoform X1 [Apis dorsata]	0
gALL_v3_c23000	1	4	-	-	-	-	-
gALL_v3_c23001	1	3	gnl|CDD|30665	COG0317, SpoT, Guanosine po	ref|XP_001662714.1|	hypothetical protein AaeL_AAEL012581 [Aedes aegypti] gb|EAT35244.1| AAEL012581-PA [Aedes aegypti]	9,00E-57
gALL_v3_c23002	3	2	-	-	-	-	-
gALL_v3_c23003	3	2	-	-	gb|EFA09839.1|	hypothetical protein TcasGA2_TC011986 [Tribolium castaneum]	2,00E-69
gALL_v3_c23004	1	4	-	-	ref|XP_006641317.1|	PREDICTED: Fanconi anemia-associated protein of 24 kDa-like [Lepisosteus oculatus]	2,00E-19
gALL_v3_c23005	4	5	-	-	-	-	-
gALL_v3_c23006	0	3	-	-	-	-	-
gALL_v3_c23007	2	2	-	-	sp|Q25637.2|PER_PERA	RecName: Full=Period circadian protein gb|AAA64677.2| period clock protein homolog [Periplaneta americana]	5,00E-37
gALL_v3_c23008	0	3	-	-	-	-	-
gALL_v3_c23009	1	3	-	-	-	-	-
gALL_v3_c2301	13	17	gnl|CDD|152354	pfam11919, DUF3437, Domair	gb|EFN87710.1|	Proteasome activator complex subunit 4 [Harpegnathos saltator]	2,00E-76
gALL_v3_c23010	1	3	-	-	-	-	-
gALL_v3_c23011	4	5	-	-	-	-	-
gALL_v3_c23013	5	5	-	-	gb|EFN72245.1|	hypothetical protein EAG_04135 [Camponotus floridanus]	3,00E-28
gALL_v3_c23014	3	2	-	-	-	-	-
gALL_v3_c23015	3	1	-	-	-	-	-
gALL_v3_c23016	2	8	gnl|CDD|191810	pfam07679, I-set, Immunoglol	ref|XP_003425477.1|	PREDICTED: twitchin-like [Nasonia vitripennis]	8,00E-49
gALL_v3_c23018	1	4	-	-	-	-	-
gALL_v3_c23019	3	5	-	-	-	-	-
gALL_v3_c2302	8	11	gnl|CDD|189522	pfam00375, SDF, Sodium:dicar	gb|AAF71701.1|AF2085	high-affinity Na+-dependent glutamate transporter [Diploptera punctata]	6,00E-90
gALL_v3_c23020	3	2	-	-	-	-	-
gALL_v3_c23021	2	7	-	-	-	-	-
gALL_v3_c23022	3	3	-	-	-	-	-
gALL_v3_c23023	1	5	-	-	-	-	-
gALL_v3_c23024	2	2	gnl|CDD|129709	TIGR00622, ssl1, transcription	gb|ENN70741.1|	hypothetical protein YQE_12531, partial [Dendroctonus ponderosae]	6,00E-48
gALL_v3_c23025	1	3	-	-	-	-	-
gALL_v3_c23026	2	1	-	-	-	-	-
gALL_v3_c23027	1	3	-	-	-	-	-
gALL_v3_c23029	0	4	-	-	-	-	-
gALL_v3_c2303	27	41	-	-	-	-	-
gALL_v3_c23030	3	1	gnl|CDD|146186	pfam03416, Peptidase_C54, Pe	ref|XP_971091.2|	PREDICTED: similar to conserved hypothetical protein [Tribolium castaneum] gb|EFA11495.1| hypothetical protein TcasGA2_TC014208 [Tribolium castaneum]	4,00E-52
gALL_v3_c23033	2	6	gnl|CDD|162085	TIGR00880, 2_A_01_02, Multi	ref|XP_970834.2|	PREDICTED: similar to AGAP004476-PA [Tribolium castaneum]	1,00E-114
gALL_v3_c23035	3	1	-	-	-	-	-
gALL_v3_c23036	2	2	-	-	ref|XP_003425190.1|	PREDICTED: SCF E3 ubiquitin ligase complex F-box protein grrA-like [Nasonia vitripennis]	2,00E-14
gALL_v3_c23037	6	3	-	-	-	-	-
gALL_v3_c23038	2	2	gnl|CDD|189925	pfam01284, MARVEL, Membra	gb|EFX88929.1|	hypothetical protein DAPPUDRAFT_230263 [Daphnia pulex]	3,00E-16
gALL_v3_c23039	2	3	-	-	-	-	-
gALL_v3_c2304	4	13	-	-	-	-	-
gALL_v3_c23040	3	1	gnl|CDD|129141	TIGR00030, S21p, ribosomal p	dbj|BAN20540.1|	mitochondrial ribosomal protein S21 [Riptortus pedestris]	1,00E-37
gALL_v3_c23041	2	2	-	-	ref|XP_002432088.1|	Homeobox protein PKNOX2, putative [Pediculus humanus corporis] gb|EEB19350.1| Homeobox protein PKNOX2, putative [Pediculus humanus corporis]	5,00E-72
gALL_v3_c23043	3	2	-	-	-	-	-
gALL_v3_c23045	4	4	-	-	ref|XP_006557487.1|	PREDICTED: three prime repair exonuclease 2-like [Apis mellifera]	5,00E-09
gALL_v3_c23046	1	3	gnl|CDD|132757	cd07072, NR_LBD_DHR38_like	ref|XP_002428935.1|	retinoid X receptor, putative [Pediculus humanus corporis] gb|EEB16197.1| retinoid X receptor, putative [Pediculus humanus corporis]	1,00E-12
gALL_v3_c23047	4	3	gnl|CDD|29376	cd01408, SIRT1, SIRT1: Eukary	ref|XP_969055.1|	PREDICTED: similar to NAD-dependent deacetylase sirtuin 2 homolog [Tribolium castaneum] gb|EFA06770.1| hypothetical protein TcasGA2_TC009705 [Tribolium castaneu	1,00E-122
gALL_v3_c23048	1	2	-	-	-	-	-
gALL_v3_c23049	2	2	-	-	-	-	-
gALL_v3_c23050	2	2	-	-	-	-	-
gALL_v3_c23051	4	11	gnl|CDD|189954	pfam01359, Transposase_1, Tr	emb|CAP20051.1|	transposase [Portunus pelagicus]	5,00E-41
gALL_v3_c23052	2	1	-	-	-	-	-
gALL_v3_c23053	0	3	-	-	-	-	-
gALL_v3_c23054	3	1	-	-	-	-	-
gALL_v3_c23055	2	2	-	-	-	-	-
gALL_v3_c23056	3	3	-	-	gb|ETN68062.1|	hypothetical protein AND_000102 [Anopheles darlingi]	2,00E-12
gALL_v3_c23057	6	10	-	-	-	-	-
gALL_v3_c23058	1	4	-	-	ref|XP_314788.4|	AGAP008689-PA [Anopheles gambiae str. PEST] gb|EAA10190.4| AGAP008689-PA [Anopheles gambiae str. PEST]	2,00E-16
gALL_v3_c23059	5	6	-	-	-	-	-
gALL_v3_c2306	6	7	gnl|CDD|189583	pfam00520, Ion_trans, Ion tra	gb|EFN84356.1|	Voltage-dependent calcium channel type D subunit alpha-1 [Harpegnathos saltator]	0
gALL_v3_c23061	3	1	-	-	-	-	-
gALL_v3_c23062	1	3	-	-	-	-	-
gALL_v3_c23064	2	1	-	-	-	-	-
gALL_v3_c23065	5	5	-	-	-	-	-
gALL_v3_c23066	2	2	gnl|CDD|143964	pfam00207, A2M, Alpha-2-ma	gb|EGI70799.1|	CD109 antigen [Acromyrmex echinatior]	2,00E-58
gALL_v3_c23067	1	4	-	-	-	-	-
gALL_v3_c23068	3	1	gnl|CDD|99848	cd06145, REX1_like, DEDDh 3'	ref|XP_003488252.1|	PREDICTED: hypothetical protein LOC100741380 [Bombus impatiens]	2,00E-70
gALL_v3_c23069	3	5	-	-	-	-	-
gALL_v3_c23070	3	1	-	-	-	-	-
gALL_v3_c23071	1	2	-	-	-	-	-
gALL_v3_c23073	0	3	-	-	-	-	-
gALL_v3_c23074	3	3	-	-	-	-	-
gALL_v3_c23076	1	3	gnl|CDD|190680	pfam03587, EMG1, EMG1/NEI	ref|XP_003705174.1|	PREDICTED: ribosomal RNA small subunit methyltransferase NEP1-like [Megachile rotundata]	2,00E-44
gALL_v3_c23077	3	1	-	-	-	-	-
gALL_v3_c23078	1	3	-	-	-	-	-
gALL_v3_c23079	2	2	-	-	ref|XP_003706081.1|	PREDICTED: protein lin-37 homolog [Megachile rotundata]	1,00E-15
gALL_v3_c2308	6	9	-	-	ref|XP_006639799.1|	PREDICTED: cyclin-D1-binding protein 1 homolog [Lepisosteus oculatus]	2,00E-49
gALL_v3_c23080	5	8	-	-	-	-	-
gALL_v3_c23081	1	3	-	-	-	-	-
gALL_v3_c23082	3	3	-	-	-	-	-
gALL_v3_c23083	2	3	-	-	-	-	-
gALL_v3_c23084	2	4	-	-	-	-	-
gALL_v3_c23085	4	4	gnl|CDD|128621	smart00326, SH3, Src homolog	gb|EFN64992.1|	Dynamin-binding protein [Camponotus floridanus]	5,00E-12
gALL_v3_c23087	2	2	-	-	-	-	-
gALL_v3_c23088	0	3	-	-	-	-	-
gALL_v3_c23089	0	4	-	-	-	-	-
gALL_v3_c2309	26	27	-	-	-	-	-
gALL_v3_c23091	0	5	-	-	-	-	-
gALL_v3_c23093	11	12	gnl|CDD|29101	cd00161, RICIN, Ricin-type bet	gb|AGM32244.1|	ricin-type beta-trefoil lectin domain-containing protein [Coptotermes formosanus]	2,00E-37
gALL_v3_c23097	0	4	-	-	-	-	-
gALL_v3_c23098	2	4	-	-	gb|EFN60669.1|	Tip elongation aberrant protein 1 [Camponotus floridanus]	1,00E-11
gALL_v3_c23099	7	8	-	-	-	-	-
gALL_v3_c231	6	9	gnl|CDD|179368	PRK02106, PRK02106, choline	ref|XP_002429701.1|	glucose dehydrogenase precursor, putative [Pediculus humanus corporis] gb|EEB16963.1| glucose dehydrogenase precursor, putative [Pediculus humanus corporis]	7,00E-29
gALL_v3_c23100	2	3	-	-	-	-	-
gALL_v3_c23101	0	3	-	-	-	-	-
gALL_v3_c23103	1	1	-	-	ref|XP_003706022.1|	PREDICTED: uncharacterized protein LOC100875873 [Megachile rotundata]	2,00E-27
gALL_v3_c23104	1	2	-	-	-	-	-
gALL_v3_c23105	3	6	gnl|CDD|190532	pfam03127, GAT, GAT domain.	ref|XP_003395795.1|	PREDICTED: ADP-ribosylation factor-binding protein GGA1-like [Bombus terrestris]	4,00E-62
gALL_v3_c23107	1	2	-	-	-	-	-
gALL_v3_c23108	2	7	-	-	gb|ENN75443.1|	hypothetical protein YQE_07994, partial [Dendroctonus ponderosae] gb|ERL86366.1| hypothetical protein D910_03774 [Dendroctonus ponderosae]	5,00E-11
gALL_v3_c23109	0	3	-	-	-	-	-

gALL_v3_c2311	20	13	-	-	ref|XP_005319950.1|	PREDICTED: pogo transposable element with KRAB domain [Ictidomys tridecemlineatus]	9,00E-09
gALL_v3_c23111	2	4	-	-	-	-	-
gALL_v3_c23112	2	1	-	-	-	-	-
gALL_v3_c23114	1	4	-	-	-	-	-
gALL_v3_c23115	3	1	-	-	-	-	-
gALL_v3_c23116	1	3	-	-	-	-	-
gALL_v3_c23117	2	4	-	-	-	-	-
gALL_v3_c23118	4	3	-	-	-	-	-
gALL_v3_c23119	1	3	-	-	ref|XP_002431318.1|	translation machinery-associated protein, putative [Pediculus humanus corporis] gb|EEB18580.1| translation machinery-associated protein, putative [Pediculus humanus c	1,00E-06
gALL_v3_c2312	2	10	gnl|CDD|178935	PRK00222, PRK00222, methio	ref|XP_002425000.1|	Methionine-R-sulfoxide reductase, putative [Pediculus humanus corporis] gb|EEB12262.1| Methionine-R-sulfoxide reductase, putative [Pediculus humanus corporis]	1,00E-67
gALL_v3_c23120	4	7	-	-	-	-	-
gALL_v3_c23121	1	2	-	-	-	-	-
gALL_v3_c23122	0	3	-	-	gb|AGM32905.1|	hypothetical protein [Coptotermes formosanus]	4,00E-07
gALL_v3_c23123	3	3	-	-	-	-	-
gALL_v3_c23124	2	2	-	-	-	-	-
gALL_v3_c23126	2	2	-	-	-	-	-
gALL_v3_c23127	1	6	-	-	-	-	-
gALL_v3_c23128	1	4	-	-	-	-	-
gALL_v3_c23129	3	2	-	-	-	-	-
gALL_v3_c2313	6	6	gnl|CDD|183835	PRK12999, PRK12999, pyruvat	ref|XP_002080823.1|	GD10044 [Drosophila simulans] gb|EDX06408.1| GD10044 [Drosophila simulans]	1,00E-172
gALL_v3_c23130	2	2	-	-	-	-	-
gALL_v3_c23131	0	5	-	-	-	-	-
gALL_v3_c23132	0	6	gnl|CDD|189507	pfam00335, Tetraspannin, Tetr	ref|XP_006615822.1|	PREDICTED: tetraspanin-31-A-like isoform X2 [Apis dorsata]	7,00E-38
gALL_v3_c23133	3	1	-	-	-	-	-
gALL_v3_c23135	4	7	-	-	gb|EFN82933.1|	hypothetical protein EAI_15889 [Harpegnathos saltator]	2,00E-08
gALL_v3_c23136	0	6	-	-	-	-	-
gALL_v3_c23137	0	4	-	-	-	-	-
gALL_v3_c23138	1	5	gnl|CDD|30899	COG0553, HepA, Superfamily I	gb|EFN84572.1|	TATA-binding protein-associated factor 172 [Harpegnathos saltator]	4,00E-71
gALL_v3_c23139	0	4	-	-	ref|XP_002431400.1|	conserved hypothetical protein [Pediculus humanus corporis] gb|EEB18662.1| conserved hypothetical protein [Pediculus humanus corporis]	7,00E-75
gALL_v3_c23140	1	7	-	-	ref|XP_001659801.1|	organic cation transporter [Aedes aegypti] gb|EAT38951.1| AAEL009206-PA [Aedes aegypti]	2,00E-18
gALL_v3_c23141	2	1	-	-	gb|ACB45439.1|	atypical olfactory receptor, partial [Philotrypesis pilosa] gb|ACB45441.1| putative chemosensory receptor 2, partial [Philotrypesis sp. NNW-2008]	4,00E-21
gALL_v3_c23142	4	4	-	-	gb|EFN65862.1|	Chromodomain Y-like protein 2 [Camponotus floridanus]	2,00E-18
gALL_v3_c23143	4	3	-	-	-	-	-
gALL_v3_c23144	0	4	-	-	-	-	-
gALL_v3_c23146	3	2	-	-	ref|XP_003242319.1|	PREDICTED: histone-lysine N-methyltransferase SETMAR-like [Acyrthosiphon pisum]	1,00E-07
gALL_v3_c23147	2	2	-	-	-	-	-
gALL_v3_c23148	4	8	-	-	ref|XP_003490254.1|	PREDICTED: rap1 GTPase-GDP dissociation stimulator 1-B-like [Bombus impatiens]	2,00E-77
gALL_v3_c23149	1	3	-	-	-	-	-
gALL_v3_c2315	20	23	-	-	-	-	-
gALL_v3_c23150	2	3	-	-	-	-	-
gALL_v3_c23151	3	2	-	-	-	-	-
gALL_v3_c23152	0	4	-	-	-	-	-
gALL_v3_c23153	1	2	-	-	-	-	-
gALL_v3_c23154	3	3	-	-	-	-	-
gALL_v3_c23155	0	3	-	-	-	-	-
gALL_v3_c23156	3	1	-	-	-	-	-
gALL_v3_c23158	1	2	-	-	-	-	-
gALL_v3_c2316	14	21	-	-	ref|XP_967855.1|	PREDICTED: similar to CG34398 CG34398-PA [Tribolium castaneum]	6,00E-30
gALL_v3_c23160	3	2	-	-	-	-	-
gALL_v3_c23161	1	2	-	-	-	-	-
gALL_v3_c23162	1	3	-	-	-	-	-
gALL_v3_c23163	2	2	gnl|CDD|143945	pfam00179, UQ_con, Ubiquiti	gb|AGM32545.1|	ubiquitin-conjugating enzyme E2, catalytic (UBCc) domain protein, partial [Coptotermes formosanus]	1,00E-107
gALL_v3_c23164	2	2	-	-	ref|XP_006115957.1|	PREDICTED: inhibitor of Bruton tyrosine kinase isoform X3 [Pelodiscus sinensis]	1,00E-08
gALL_v3_c23165	1	3	-	-	ref|XP_005157614.1|	PREDICTED: uncharacterized protein LOC101882541 [Danio rerio]	6,00E-21
gALL_v3_c23166	1	4	-	-	ref|XP_001950070.1|	PREDICTED: hypothetical protein LOC100161830 [Acyrthosiphon pisum]	6,00E-20
gALL_v3_c23167	1	5	-	-	-	-	-
gALL_v3_c23168	2	1	-	-	ref|NP_001161316.1|	cuticular protein precursor [Tribolium castaneum]	8,00E-54
gALL_v3_c23169	2	4	-	-	-	-	-
gALL_v3_c23170	1	3	-	-	gb|ESO95830.1|	hypothetical protein LOTGIDRAFT_214928 [Lottia gigantea]	4,00E-34
gALL_v3_c23171	1	3	-	-	-	-	-
gALL_v3_c23172	1	5	-	-	-	-	-
gALL_v3_c23173	3	6	-	-	gb|EFN72391.1|	Transposable element Tc3 transposase [Camponotus floridanus]	9,00E-19
gALL_v3_c23175	0	3	-	-	-	-	-
gALL_v3_c23176	5	3	gnl|CDD|149022	pfam07731, Cu-oxidase_2, Mu	ref|XP_002044786.1|	GM22047 [Drosophila sechellia] gb|EDW43634.1| GM22047 [Drosophila sechellia]	2,00E-29
gALL_v3_c23177	1	6	-	-	ref|XP_002431959.1|	Iroquois-class homeodomain protein IRX-1, putative [Pediculus humanus corporis] gb|EEB19221.1| Iroquois-class homeodomain protein IRX-1, putative [Pediculus human	3,00E-22
gALL_v3_c23178	2	2	-	-	-	-	-
gALL_v3_c23180	3	4	-	-	-	-	-
gALL_v3_c23181	2	7	-	-	-	-	-
gALL_v3_c23182	2	1	-	-	-	-	-
gALL_v3_c23183	2	3	-	-	-	-	-
gALL_v3_c23184	1	3	gnl|CDD|191229	pfam05199, GMC_oxred_C, GI	ref|XP_001942555.1|	PREDICTED: glucose dehydrogenase [acceptor]-like [Acyrthosiphon pisum]	4,00E-44
gALL_v3_c23185	2	3	-	-	-	-	-
gALL_v3_c23186	3	2	-	-	-	-	-
gALL_v3_c23187	2	4	-	-	-	-	-
gALL_v3_c23188	2	3	-	-	-	-	-
gALL_v3_c23189	3	2	-	-	ref|XP_001868197.1|	conserved hypothetical protein [Culex quinquefasciatus] gb|EDS26306.1| conserved hypothetical protein [Culex quinquefasciatus]	5,00E-36
gALL_v3_c2319	4	5	-	-	-	-	-
gALL_v3_c23190	1	3	-	-	-	-	-
gALL_v3_c23191	2	2	-	-	-	-	-
gALL_v3_c23193	0	4	-	-	ref|XP_003427444.1|	PREDICTED: H(+)/Cl(-) exchange transporter 3-like isoform 2 [Nasonia vitripennis]	1,00E-63
gALL_v3_c23194	3	2	-	-	-	-	-
gALL_v3_c23195	2	2	-	-	-	-	-
gALL_v3_c23196	3	1	-	-	-	-	-
gALL_v3_c23197	2	2	-	-	-	-	-
gALL_v3_c23198	3	6	gnl|CDD|100117	cd06071, Beach, BEACH (Beige	ref|XP_001602669.2|	PREDICTED: neurobeachin-like [Nasonia vitripennis]	2,00E-93
gALL_v3_c23199	0	3	-	-	-	-	-
gALL_v3_c232	3	8	gnl|CDD|99967	cd03793, GT1_Glycogen_synt	gb|EGI68911.1|	Putative glycogen [starch] synthase [Acromyrmex echinatior]	2,00E-99
gALL_v3_c2320	10	21	-	-	ref|XP_001604168.1|	PREDICTED: abnormal spindle-like microcephaly-associated protein homolog [Nasonia vitripennis]	2,00E-22
gALL_v3_c23200	6	7	-	-	-	-	-
gALL_v3_c23201	3	3	-	-	gb|ELT96652.1|	hypothetical protein CAPTEDRAFT_79103, partial [Capitella teleta]	4,00E-20
gALL_v3_c23202	1	5	-	-	-	-	-
gALL_v3_c23203	1	3	-	-	-	-	-
gALL_v3_c23204	2	3	-	-	-	-	-
gALL_v3_c23205	7	5	-	-	-	-	-
gALL_v3_c23206	5	3	gnl|CDD|180422	PRK06142, PRK06142, enoyl-C	gb|AGM32657.1|	delta 3,5-delta 2,4-dienoyl-coA isomerase, partial [Coptotermes formosanus]	1,00E-147
gALL_v3_c23207	2	3	-	-	ref|XP_001443168.1|	hypothetical protein [Paramecium tetraurelia strain d4-2] emb|CAK75771.1| unnamed protein product [Paramecium tetraurelia]	4,00E-06
gALL_v3_c23208	2	4	-	-	-	-	-
gALL_v3_c2321	1	6	-	-	-	-	-
gALL_v3_c23210	2	3	-	-	-	-	-
gALL_v3_c23211	0	3	-	-	ref|XP_006565045.1|	PREDICTED: glucose dehydrogenase [FAD, quinone] [Apis mellifera]	4,00E-11
gALL_v3_c23212	3	2	-	-	-	-	-
gALL_v3_c23214	3	3	-	-	-	-	-
gALL_v3_c23215	3	3	-	-	-	-	-
gALL_v3_c23216	1	4	-	-	gb|AGM32926.1|	hypothetical protein [Coptotermes formosanus]	9,00E-07
gALL_v3_c23217	2	2	gnl|CDD|29261	cd00204, ANK, ankyrin repeat:	ref|XP_001314624.1|	ankyrin repeat protein [Trichomonas vaginalis G3] gb|EAY02309.1| ankyrin repeat protein, putative [Trichomonas vaginalis G3]	3,00E-08
gALL_v3_c23219	1	2	gnl|CDD|190866	pfam04097, Nic96, Nup93/Nic	ref|XP_001633614.1|	predicted protein [Nematostella vectensis] gb|EDO41551.1| predicted protein [Nematostella vectensis]	1,00E-37
gALL_v3_c2322	8	18	-	-	-	-	-
gALL_v3_c23221	0	6	-	-	-	-	-
gALL_v3_c23222	17	25	-	-	-	-	-
gALL_v3_c23223	1	3	-	-	-	-	-
gALL_v3_c23225	3	1	gnl|CDD|190024	pfam01532, Glyco_hydro_47,	ref|XP_001848378.1|	EDEM [Culex quinquefasciatus] gb|EDS28207.1| EDEM [Culex quinquefasciatus]	4,00E-75
gALL_v3_c23226	2	4	-	-	gb|EFA11446.1|	hypothetical protein TcasGA2_TC013628 [Tribolium castaneum]	3,00E-08
gALL_v3_c23227	2	5	-	-	-	-	-
gALL_v3_c23228	2	2	-	-	-	-	-
gALL_v3_c23229	0	5	-	-	-	-	-
gALL_v3_c23230	6	3	-	-	-	-	-
gALL_v3_c23231	2	5	-	-	-	-	-
gALL_v3_c23232	2	4	-	-	-	-	-
gALL_v3_c23233	2	2	-	-	-	-	-
gALL_v3_c23234	1	2	-	-	gb|AAC34928.1|	putative chimeric R1/R2 retrotransposon [Nasonia vitripennis]	6,00E-09
gALL_v3_c23235	3	1	-	-	ref|XP_002422627.1|	zfpl1, putative [Pediculus humanus corporis] gb|EEB09889.1| zfpl1, putative [Pediculus humanus corporis]	1,00E-13
gALL_v3_c23236	3	2	-	-	-	-	-
gALL_v3_c23237	3	1	-	-	-	-	-
gALL_v3_c23239	4	2	-	-	-	-	-
gALL_v3_c23240	1	6	-	-	ref|NP_694834.1|	nonstructural protein [Bombyx mori densovirus 5] dbj|BAA96073.1| nonstructural protein [Bombyx mori densovirus 5]	6,00E-24
gALL_v3_c23242	1	3	-	-	-	-	-
gALL_v3_c23243	1	4	-	-	-	-	-
gALL_v3_c23244	3	2	gnl|CDD|28920	cd00038, CAP_ED, effector do	gb|ABN11942.1|	hypothetical protein [Maconellicoccus hirsutus]	3,00E-44
gALL_v3_c23245	14	9	-	-	-	-	-
gALL_v3_c23246	4	4	-	-	-	-	-
gALL_v3_c23247	2	4	-	-	-	-	-
gALL_v3_c23248	57	35	-	-	ref|WP_002430991.1|	conserved hypothetical protein [Escherichia sp. 3_2_53FAA] gb|EEH89633.1| hypothetical protein ESAG_07158 [Escherichia sp. 3_2_53FAA]	3,00E-20
gALL_v3_c23249	2	1	-	-	-	-	-
gALL_v3_c23250	2	2	-	-	-	-	-
gALL_v3_c23251	1	5	-	-	-	-	-
gALL_v3_c23252	2	2	gnl|CDD|189373	pfam00069, Pkinase, Protein k	ref|XP_002425367.1|	serine-threonine protein kinase, plant-type, putative [Pediculus humanus corporis] gb|EEB12629.1| serine-threonine protein kinase, plant-type, putative [Pediculus humar	2,00E-30
gALL_v3_c23253	1	4	-	-	-	-	-
gALL_v3_c23254	2	4	-	-	-	-	-
gALL_v3_c23255	1	3	-	-	-	-	-
gALL_v3_c23257	2	2	-	-	-	-	-
gALL_v3_c23258	1	5	-	-	-	-	-
gALL_v3_c23259	0	4	-	-	-	-	-
gALL_v3_c2326	4	12	gnl|CDD|173966	cd08047, TAF7, TATA Binding P	ref|XP_006609816.1|	PREDICTED: transcription initiation factor TFIID subunit 7-like [Apis dorsata]	2,00E-74
gALL_v3_c23260	2	6	-	-	ref|XP_004934044.1|	PREDICTED: probable elongation factor 1-delta-like, partial [Bombyx mori]	2,00E-32

gALL_v3_c23261	2	2	-	-	-	-	-
gALL_v3_c23262	3	2	-	-	gb|EFN88266.1|	Centaurin-delta-1 [Harpegnathos saltator]	2,00E-44
gALL_v3_c23263	2	3	-	-	-	-	-
gALL_v3_c23264	3	2	-	-	ref|XP_004934295.1|	PREDICTED: polyol transporter 5-like, partial [Bombyx mori]	2,00E-18
gALL_v3_c23266	1	4	-	-	-	-	-
gALL_v3_c23267	3	1	-	-	-	-	-
gALL_v3_c23268	1	2	-	-	-	-	-
gALL_v3_c2327	9	12	-	-	-	-	-
gALL_v3_c23270	0	6	-	-	gb|EGI63234.1|	RNA-binding protein 26 [Acromyrmex echinatior]	6,00E-30
gALL_v3_c23271	2	2	-	-	-	-	-
gALL_v3_c23272	1	3	-	-	-	-	-
gALL_v3_c23273	1	3	-	-	-	-	-
gALL_v3_c23274	2	4	-	-	-	-	-
gALL_v3_c23275	2	4	gnl|CDD|128548	smart00252, SH2, Src homolog	ref|XP_002424401.1|	sh2 domain containing protein, putative [Pediculus humanus corporis] gb|EEB11663.1| sh2 domain containing protein, putative [Pediculus humanus corporis]	2,00E-13
gALL_v3_c23276	2	1	-	-	-	-	-
gALL_v3_c23277	3	2	-	-	-	-	-
gALL_v3_c23278	1	3	-	-	-	-	-
gALL_v3_c23279	1	4	-	-	-	-	-
gALL_v3_c2328	13	13	gnl|CDD|114630	pfam05918, API5, Apoptosis ir	gb|EFN70040.1|	Apoptosis inhibitor 5 [Camponotus floridanus]	1,00E-151
gALL_v3_c23280	1	5	-	-	-	-	-
gALL_v3_c23281	0	4	gnl|CDD|162277	TIGR01271, CFTR_protein, cys	ref|XP_003241132.1|	PREDICTED: multidrug resistance-associated protein 4-like [Acyrthosiphon pisum]	6,00E-24
gALL_v3_c23282	1	4	-	-	-	-	-
gALL_v3_c23283	1	3	gnl|CDD|187698	cd09274, RNase_HI_RT_Ty3, T	ref|XP_003707187.1|	PREDICTED: histone-lysine N-methyltransferase SETMAR-like [Megachile rotundata]	3,00E-40
gALL_v3_c23284	1	3	-	-	-	-	-
gALL_v3_c23285	1	4	-	-	-	-	-
gALL_v3_c23286	1	2	-	-	-	-	-
gALL_v3_c23287	4	3	gnl|CDD|191074	pfam04734, Ceramidase_alk, I	ref|XP_005183819.1|	PREDICTED: neutral ceramidase-like isoform X1 [Musca domestica] ref|XP_005183820.1| PREDICTED: neutral ceramidase-like isoform X2 [Musca domestica] ref|XP_00518	4,00E-13
gALL_v3_c23288	1	2	gnl|CDD|190523	pfam03098, An_peroxidase, A	ref|XP_004932596.1|	PREDICTED: chorion peroxidase-like [Bombyx mori]	8,00E-39
gALL_v3_c23289	1	2	-	-	-	-	-
gALL_v3_c23290	3	1	-	-	-	-	-
gALL_v3_c23291	4	2	gnl|CDD|153340	cd07656, F-BAR_srGAP, The F-	ref|XP_002424891.1|	SLIT-ROBO Rho GTPase-activating protein, putative [Pediculus humanus corporis] gb|EEB12153.1| SLIT-ROBO Rho GTPase-activating protein, putative [Pediculus humanus	1,00E-95
gALL_v3_c23292	6	3	gnl|CDD|150314	pfam09606, Med15, ARC105 c	ref|XP_001605352.2|	PREDICTED: hypothetical protein LOC100121744 [Nasonia vitripennis]	6,00E-17
gALL_v3_c23293	2	1	gnl|CDD|189334	pfam00001, 7tm_1, 7 transme	ref|XP_310742.3|	AGAP000369-PA [Anopheles gambiae str. PEST] gb|EAA06377.4| AGAP000369-PA [Anopheles gambiae str. PEST]	2,00E-32
gALL_v3_c23294	0	3	-	-	-	-	-
gALL_v3_c23295	2	4	-	-	-	-	-
gALL_v3_c23296	1	4	-	-	-	-	-
gALL_v3_c23297	3	5	gnl|CDD|177214	MTH00157, ATP6, ATP synthas	ref|YP_006503978.1|	ATP synthase F0 subunit 6 (mitochondrion) [Heterotermes sp. SLC-2012] gb|AFM92478.1| ATP synthase F0 subunit 6 (mitochondrion) [Heterotermes sp. SLC-2012]	6,00E-14
gALL_v3_c23298	3	3	-	-	ref|XP_004923904.1|	PREDICTED: tripeptidyl-peptidase 2-like [Bombyx mori]	4,00E-20
gALL_v3_c2330	9	9	-	-	-	-	-
gALL_v3_c23300	2	2	-	-	ref|XP_002426669.1|	hypothetical protein Phum_PHUM267750 [Pediculus humanus corporis] gb|EEB13931.1| hypothetical protein Phum_PHUM267750 [Pediculus humanus corporis]	2,00E-49
gALL_v3_c23302	3	2	-	-	gb|EFA12688.1|	hypothetical protein TcasGA2_TC001995 [Tribolium castaneum]	4,00E-21
gALL_v3_c23303	3	2	-	-	gb|EFN72876.1|	hypothetical protein EAG_01351 [Camponotus floridanus]	5,00E-07
gALL_v3_c23304	1	2	-	-	-	-	-
gALL_v3_c23305	1	2	-	-	ref|XP_002429764.1|	conserved hypothetical protein [Pediculus humanus corporis] gb|EEB17026.1| conserved hypothetical protein [Pediculus humanus corporis]	1,00E-22
gALL_v3_c23306	1	2	-	-	-	-	-
gALL_v3_c23307	1	3	-	-	-	-	-
gALL_v3_c23308	0	3	-	-	-	-	-
gALL_v3_c23309	2	2	gnl|CDD|109161	pfam00093, VWC, von Willebr	ref|XP_003703692.1|	PREDICTED: protein kinase C-binding protein NELL1-like [Megachile rotundata]	2,00E-82
gALL_v3_c2331	8	16	gnl|CDD|128612	smart00317, SET, SET (Su(var):	ref|XP_001599536.2|	PREDICTED: hypothetical protein LOC100114564 [Nasonia vitripennis]	2,00E-85
gALL_v3_c23310	2	1	-	-	-	-	-
gALL_v3_c23311	2	2	-	-	gb|ADI61823.1|	endonuclease-reverse transcriptase [Bombyx mori]	4,00E-10
gALL_v3_c23312	0	4	-	-	-	-	-
gALL_v3_c23313	2	2	-	-	-	-	-
gALL_v3_c23315	3	4	-	-	-	-	-
gALL_v3_c23316	3	4	-	-	gb|EFZ19338.1|	hypothetical protein SINV_09798 [Solenopsis invicta]	4,00E-67
gALL_v3_c23317	2	2	-	-	-	-	-
gALL_v3_c23318	2	3	-	-	-	-	-
gALL_v3_c23319	1	3	-	-	-	-	-
gALL_v3_c23320	1	3	-	-	ref|XP_002430929.1|	hypothetical protein Phum_PHUM504170 [Pediculus humanus corporis] gb|EEB18191.1| hypothetical protein Phum_PHUM504170 [Pediculus humanus corporis]	6,00E-10
gALL_v3_c23321	3	1	-	-	gb|EFN61784.1|	E3 ubiquitin-protein ligase RNF123 [Camponotus floridanus]	6,00E-31
gALL_v3_c23322	1	3	gnl|CDD|188712	cd09326, LIM_CRP_like, The LI	ref|XP_002092765.1|	GE14374 [Drosophila yakuba] gb|EDW92477.1| GE14374 [Drosophila yakuba]	5,00E-12
gALL_v3_c23323	2	1	-	-	ref|YP_005855168.1|	hypothetical protein LC2W_0248 [Lactobacillus casei LC2W] ref|YP_005855828.1| hypothetical protein LC2W_0910 [Lactobacillus casei LC2W] ref|YP_005855844.1| hypot	3,00E-25
gALL_v3_c23324	0	3	-	-	-	-	-
gALL_v3_c23325	2	1	-	-	-	-	-
gALL_v3_c23326	2	3	gnl|CDD|177906	PLN02265, PLN02265, probabl	ref|XP_003402782.1|	PREDICTED: leucine-rich repeat-containing protein 47-like [Bombus terrestris]	1,00E-64
gALL_v3_c23327	1	3	-	-	-	-	-
gALL_v3_c23328	3	1	-	-	-	-	-
gALL_v3_c23329	1	5	gnl|CDD|191574	pfam06650, DUF1162, Protein	ref|XP_003703656.1|	PREDICTED: vacuolar protein sorting-associated protein 13C-like [Megachile rotundata]	3,00E-76
gALL_v3_c23330	9	14	-	-	-	-	-
gALL_v3_c23331	3	3	-	-	-	-	-
gALL_v3_c23332	0	4	gnl|CDD|119351	cd02872, GH18_chitolectin_ch	gb|EFA10488.1|	hypothetical protein TcasGA2_TC012734 [Tribolium castaneum]	1,00E-58
gALL_v3_c23333	2	3	-	-	-	-	-
gALL_v3_c23334	98	149	-	-	-	-	-
gALL_v3_c23335	1	2	-	-	-	-	-
gALL_v3_c23336	1	3	-	-	-	-	-
gALL_v3_c23337	3	1	-	-	-	-	-
gALL_v3_c23338	0	3	-	-	-	-	-
gALL_v3_c23339	5	7	-	-	-	-	-
gALL_v3_c2334	5	5	-	-	ref|XP_003738337.1|	PREDICTED: uncharacterized protein LOC100905802 [Metaseiulus occidentalis]	6,00E-14
gALL_v3_c23341	0	3	-	-	-	-	-
gALL_v3_c23342	5	4	-	-	gb|EFN84371.1|	hypothetical protein EAI_13551 [Harpegnathos saltator]	8,00E-08
gALL_v3_c23343	1	3	-	-	gb|EFN89642.1|	Vacuolar protein sorting-associated protein 11-like protein [Harpegnathos saltator]	8,00E-75
gALL_v3_c23344	3	2	-	-	-	-	-
gALL_v3_c23345	5	5	gnl|CDD|185696	cd09019, galactose_mutarotas	ref|XP_001958447.1|	GF10927 [Drosophila ananassae] gb|EDV41253.1| GF10927 [Drosophila ananassae]	4,00E-39
gALL_v3_c23346	1	2	-	-	-	-	-
gALL_v3_c23348	1	3	-	-	gb|ERL86912.1|	hypothetical protein D910_04315, partial [Dendroctonus ponderosae]	1,00E-91
gALL_v3_c23349	2	2	-	-	-	-	-
gALL_v3_c2335	9	5	-	-	-	-	-
gALL_v3_c23350	2	2	-	-	-	-	-
gALL_v3_c23352	2	4	-	-	-	-	-
gALL_v3_c23353	3	2	gnl|CDD|117221	pfam08648, DUF1777, Protein	-	-	-
gALL_v3_c23354	0	3	-	-	-	-	-
gALL_v3_c23355	3	3	-	-	-	-	-
gALL_v3_c23356	2	1	-	-	-	-	-
gALL_v3_c23357	2	2	-	-	gb|EFN84631.1|	Myosin-VIIa [Harpegnathos saltator]	1,00E-114
gALL_v3_c23358	3	2	gnl|CDD|130689	TIGR01628, PABP-1234, polya(	ref|XP_001303266.1|	Polyadenylate-binding protein [Trichomonas vaginalis G3] gb|EAX90336.1| Polyadenylate-binding protein, putative [Trichomonas vaginalis G3]	3,00E-64
gALL_v3_c23359	2	2	gnl|CDD|190885	pfam04147, Nop14, Nop14-lik	gb|EFN70441.1|	VPRBP-like protein [Camponotus floridanus]	1,00E-13
gALL_v3_c2336	6	3	-	-	-	-	-
gALL_v3_c23360	0	4	-	-	ref|XP_797146.2|	PREDICTED: leucine-rich repeat flightless-interacting protein 2-like [Strongylocentrotus purpuratus]	3,00E-06
gALL_v3_c23361	1	3	-	-	-	-	-
gALL_v3_c23362	2	1	-	-	-	-	-
gALL_v3_c23363	1	3	-	-	-	-	-
gALL_v3_c23364	2	4	-	-	-	-	-
gALL_v3_c23365	2	3	gnl|CDD|193474	pfam13001, Ecm29, Proteasor	ref|XP_003707848.1|	PREDICTED: proteasome-associated protein ECM29 homolog [Megachile rotundata]	1,00E-36
gALL_v3_c23366	3	1	-	-	-	-	-
gALL_v3_c23367	0	4	-	-	-	-	-
gALL_v3_c23368	3	5	-	-	gb|ESO05370.1|	hypothetical protein HELRODRAFT_171762 [Helobdella robusta]	7,00E-54
gALL_v3_c23369	0	4	-	-	-	-	-
gALL_v3_c2337	13	17	-	-	-	-	-
gALL_v3_c23370	2	1	-	-	-	-	-
gALL_v3_c23372	1	2	-	-	-	-	-
gALL_v3_c23373	1	2	-	-	-	-	-
gALL_v3_c23374	3	2	gnl|CDD|185535	PTZ00272, PTZ00272, heat sho	dbj|BAD83618.1|	cytosolic-type hsp90 [Trichomonas vaginalis]	1,00E-49
gALL_v3_c23375	4	3	gnl|CDD|146145	pfam03357, Snf7, Snf7. This fa	ref|XP_973478.2|	PREDICTED: similar to CG4618 CG4618-PA [Tribolium castaneum]	1,00E-48
gALL_v3_c23377	1	3	-	-	-	-	-
gALL_v3_c23378	2	2	-	-	-	-	-
gALL_v3_c23379	4	2	-	-	ref|XP_003240951.1|	PREDICTED: zinc finger protein 665-like [Acyrthosiphon pisum]	4,00E-48
gALL_v3_c2338	9	6	gnl|CDD|189378	pfam00079, Serpin, Serpin (se	gb|AGK40926.1|	serpin-1, partial [Nilaparvata lugens]	2,00E-90
gALL_v3_c23381	2	3	-	-	-	-	-
gALL_v3_c23382	2	2	-	-	-	-	-
gALL_v3_c23383	3	4	-	-	-	-	-
gALL_v3_c23385	2	1	-	-	-	-	-
gALL_v3_c23387	2	1	-	-	-	-	-
gALL_v3_c23388	1	3	-	-	-	-	-
gALL_v3_c23389	6	6	-	-	-	-	-
gALL_v3_c2339	8	6	-	-	-	-	-
gALL_v3_c23391	4	5	gnl|CDD|189807	pfam01025, GrpE, GrpE.	gb|AGM32937.1|	GrpE protein-like protein [Coptotermes formosanus]	4,00E-94
gALL_v3_c23392	2	6	-	-	-	-	-
gALL_v3_c23393	2	3	-	-	-	-	-
gALL_v3_c23394	0	3	-	-	-	-	-
gALL_v3_c23395	0	3	-	-	-	-	-
gALL_v3_c23396	2	2	-	-	ref|XP_003401851.1|	PREDICTED: hypothetical protein LOC100649948 [Bombus terrestris]	1,00E-31
gALL_v3_c23397	2	2	-	-	-	-	-
gALL_v3_c23398	2	1	-	-	gb|EHJ76831.1|	hypothetical protein KGM_17362 [Danaus plexippus]	4,00E-09
gALL_v3_c23399	3	2	gnl|CDD|190870	pfam04113, Gpi16, Gpi16 subu	gb|EFN64949.1|	GPI transamidase component PIG-T [Camponotus floridanus]	1,00E-79
gALL_v3_c2340	11	20	-	-	-	-	-
gALL_v3_c23400	4	5	-	-	ref|XP_006622691.1|	PREDICTED: bone morphogenetic protein receptor type-2-like isoform X1 [Apis dorsata] ref|XP_006622692.1| PREDICTED: bone morphogenetic protein receptor type-2-lik	4,00E-18
gALL_v3_c23401	1	3	-	-	ref|XP_002433006.1|	conserved hypothetical protein [Pediculus humanus corporis] gb|EEB20268.1| conserved hypothetical protein [Pediculus humanus corporis]	4,00E-22
gALL_v3_c23402	2	2	-	-	-	-	-
gALL_v3_c23403	1	3	-	-	-	-	-
gALL_v3_c23404	2	1	-	-	-	-	-
gALL_v3_c23405	3	1	gnl|CDD|116380	pfam07766, LETM1, LETM1-lik	ref|XP_002426162.1|	leucine zipper-EF-hand-containing transmembrane protein 1, putative [Pediculus humanus corporis] gb|EEB13424.1| leucine zipper-EF-hand-containing transmembrane pı	1,00E-29
gALL_v3_c23407	4	7	-	-	gb|ERG87344.1|	protein sidekick-1 [Ascaris suum]	4,00E-13
gALL_v3_c2341	10	13	-	-	ref|XP_004527145.1|	PREDICTED: uncharacterized family 31 glucosidase KIAA1161-like isoform X2 [Ceratitis capitata]	1,00E-18

gALL_v3_c23410	2	2	-	-	-	-	-
gALL_v3_c23411	2	4	-	-	-	-	-
gALL_v3_c23412	5	2	-	-	ref|XP_002426039.1|	DNA repair and recombination protein RAD26, putative [Pediculus humanus corporis] gb|EEB13301.1| DNA repair and recombination protein RAD26, putative [Pediculus h	1,00E-11
gALL_v3_c23413	5	8	-	-	ref|XP_002427523.1|	conserved hypothetical protein [Pediculus humanus corporis] gb|EEB14785.1| conserved hypothetical protein [Pediculus humanus corporis]	5,00E-09
gALL_v3_c23414	1	4	-	-	-	-	-
gALL_v3_c23415	3	2	-	-	gb|EFA06228.1|	hypothetical protein TcasGA2_TC009077 [Tribolium castaneum]	6,00E-11
gALL_v3_c23416	3	1	-	-	gb|EFZ16921.1|	hypothetical protein SINV_15983 [Solenopsis invicta]	5,00E-93
gALL_v3_c23417	2	1	-	-	-	-	-
gALL_v3_c23418	3	1	gnl|CDD|143833	pfam00046, Homeobox, Home	gb|AAF03888.1|AF1870	proboscipedia ortholog [Tribolium castaneum]	4,00E-62
gALL_v3_c23419	0	3	-	-	-	-	-
gALL_v3_c2342	6	12	-	-	-	-	-
gALL_v3_c23420	2	1	-	-	ref|XP_003492021.1|	PREDICTED: hypothetical protein LOC100747221 [Bombus impatiens]	1,00E-09
gALL_v3_c23421	4	3	-	-	ref|XP_001987539.1|	GH21975 [Drosophila grimshawi] gb|EDW02406.1| GH21975 [Drosophila grimshawi]	4,00E-10
gALL_v3_c23422	2	3	-	-	gb|ELT90309.1|	hypothetical protein CAPTEDRAFT_130437, partial [Capitella teleta]	7,00E-31
gALL_v3_c23423	2	1	-	-	-	-	-
gALL_v3_c23424	3	2	-	-	-	-	-
gALL_v3_c23425	2	2	-	-	-	-	-
gALL_v3_c23426	4	6	-	-	-	-	-
gALL_v3_c23427	3	2	-	-	-	-	-
gALL_v3_c23428	0	4	-	-	gb|EFA13656.1|	hypothetical protein TcasGA2_TC016129 [Tribolium castaneum]	1,00E-08
gALL_v3_c23429	3	1	-	-	-	-	-
gALL_v3_c2343	6	12	-	-	-	-	-
gALL_v3_c23430	1	3	-	-	-	-	-
gALL_v3_c23431	1	3	-	-	-	-	-
gALL_v3_c23432	2	2	gnl|CDD|176398	cd01803, Ubiquitin, Ubiquitin.	gb|AAT01911.1|	40S ribosomal protein S27A [Pseudopleuronectes americanus]	3,00E-26
gALL_v3_c23433	0	4	-	-	-	-	-
gALL_v3_c23434	0	5	-	-	-	-	-
gALL_v3_c23435	2	3	-	-	-	-	-
gALL_v3_c23436	3	3	-	-	-	-	-
gALL_v3_c23437	2	2	-	-	-	-	-
gALL_v3_c23438	3	1	-	-	-	-	-
gALL_v3_c2344	9	12	-	-	-	-	-
gALL_v3_c23440	2	1	-	-	-	-	-
gALL_v3_c23441	2	4	-	-	ref|NP_046813.1|	nonstructural protein NS1 [Diatraea saccharalis densovirus] sp|O71153.1|VNCS_DSDNV RecName: Full=Non-capsid protein NS-1; AltName: Full=NCVP1; AltName: Full=Nor	1,00E-18
gALL_v3_c23442	1	3	-	-	-	-	-
gALL_v3_c23443	0	4	-	-	gb|AGM32241.1|	hypothetical protein [Coptotermes formosanus]	1,00E-13
gALL_v3_c23445	0	3	-	-	-	-	-
gALL_v3_c23446	0	4	-	-	-	-	-
gALL_v3_c23447	1	6	-	-	ref|XP_001688893.1|	AGAP006529-PB [Anopheles gambiae str. PEST] ref|XP_003435735.1| AGAP013364-PA [Anopheles gambiae str. PEST] gb|EGK97287.1| AGAP013364-PA [Anopheles gambi	8,00E-10
gALL_v3_c23448	2	2	gnl|CDD|144473	pfam00888, Cullin, Cullin fami	gb|AEK05179.1|	putative DNA methyltransferase, partial [Schistocerca gregaria]	2,00E-82
gALL_v3_c23449	1	3	-	-	-	-	-
gALL_v3_c2345	18	21	-	-	gb|EGI61457.1|	Zinc finger CCCH domain-containing protein 10 [Acromyrmex echinatior]	3,00E-17
gALL_v3_c23451	3	2	-	-	-	-	-
gALL_v3_c23452	3	1	-	-	ref|XP_002430095.1|	chitin binding peritrophin-A, putative [Pediculus humanus corporis] gb|EEB17357.1| chitin binding peritrophin-A, putative [Pediculus humanus corporis]	8,00E-29
gALL_v3_c23453	0	3	-	-	gb|EFA13373.1|	hypothetical protein TcasGA2_TC001390 [Tribolium castaneum]	8,00E-15
gALL_v3_c23454	1	3	-	-	gb|AGM32328.1|	hypothetical protein, partial [Coptotermes formosanus]	1,00E-79
gALL_v3_c23456	2	3	-	-	-	-	-
gALL_v3_c23457	1	3	-	-	-	-	-
gALL_v3_c23458	5	3	-	-	ref|XP_003708553.1|	PREDICTED: uncharacterized protein LOC100884051 [Megachile rotundata]	4,00E-71
gALL_v3_c23459	0	6	-	-	-	-	-
gALL_v3_c2346	8	21	-	-	-	-	-
gALL_v3_c23460	3	1	-	-	-	-	-
gALL_v3_c23461	1	2	-	-	ref|XP_006562198.1|	PREDICTED: muscle M-line assembly protein unc-89 isoform X5 [Apis mellifera]	1,00E-22
gALL_v3_c23462	3	3	gnl|CDD|129984	TIGR00906, 2A0303, cationic a	gb|EFA09380.1|	hypothetical protein TcasGA2_TC001941 [Tribolium castaneum]	7,00E-12
gALL_v3_c23463	3	5	-	-	ref|XP_003705553.1|	PREDICTED: uncharacterized protein LOC100876550 [Megachile rotundata]	1,00E-11
gALL_v3_c23464	2	1	-	-	-	-	-
gALL_v3_c23465	0	4	-	-	-	-	-
gALL_v3_c23466	3	4	-	-	-	-	-
gALL_v3_c23467	4	3	-	-	ref|XP_970789.1|	PREDICTED: similar to Integrator complex subunit 4 (Int4) [Tribolium castaneum] gb|EFA03749.1| hypothetical protein TcasGA2_TC013858 [Tribolium castaneum]	2,00E-76
gALL_v3_c23468	2	5	gnl|CDD|191949	pfam08148, DSHCT, DSHCT (N	ref|XP_006623182.1|	PREDICTED: superkiller viralicidic activity 2-like 2-like isoform X2 [Apis dorsata]	8,00E-80
gALL_v3_c23469	5	3	gnl|CDD|191895	pfam07923, N1221, N1221-lik	gb|EFX82053.1|	hypothetical protein DAPPUDRAFT_302834 [Daphnia pulex]	4,00E-97
gALL_v3_c23470	1	6	-	-	ref|XP_002432456.1|	XPA-binding protein, putative [Pediculus humanus corporis] gb|EEB19718.1| XPA-binding protein, putative [Pediculus humanus corporis]	8,00E-93
gALL_v3_c23471	3	1	-	-	-	-	-
gALL_v3_c23472	2	3	-	-	ref|XP_006122740.1|	PREDICTED: tigger transposable element-derived protein 1-like [Pelodiscus sinensis]	2,00E-06
gALL_v3_c23473	1	3	-	-	ref|XP_003692964.1|	PREDICTED: mitogen-activated protein kinase kinase kinase 13-like [Apis florea]	2,00E-28
gALL_v3_c23475	1	4	-	-	-	-	-
gALL_v3_c23476	1	4	gnl|CDD|30093	cd01371, KISc_KIF3, Kinesin m	ref|XP_003490795.1|	PREDICTED: kinesin-like protein KIF3A-like [Bombus impatiens]	9,00E-36
gALL_v3_c23477	3	2	-	-	-	-	-
gALL_v3_c23479	1	2	-	-	-	-	-
gALL_v3_c2348	12	12	gnl|CDD|119429	cd05169, PIKKc_TOR, TOR (Tar	gb|ACH47049.1|	target of rapamycin [Blattella germanica]	0
gALL_v3_c23480	3	2	gnl|CDD|31753	COG1565, COG1565, Uncharac	ref|XP_003402630.1|	PREDICTED: protein midA homolog, mitochondrial-like [Bombus terrestris]	1,00E-64
gALL_v3_c23481	4	3	-	-	-	-	-
gALL_v3_c23482	2	3	-	-	ref|XP_006607404.1|	PREDICTED: twinfilin-like isoform X4 [Apis dorsata]	2,00E-06
gALL_v3_c23484	3	1	-	-	-	-	-
gALL_v3_c23485	3	2	gnl|CDD|31829	COG1643, HrpA, HrpA-like hel	gb|EFA05543.1|	hypothetical protein TcasGA2_TC015731 [Tribolium castaneum]	2,00E-82
gALL_v3_c23487	3	3	-	-	-	-	-
gALL_v3_c23488	2	2	-	-	-	-	-
gALL_v3_c23489	3	2	-	-	-	-	-
gALL_v3_c23490	2	2	gnl|CDD|130360	TIGR01293, Kv_beta, voltage-c	ref|XP_970125.2|	PREDICTED: similar to Hyperkinetic CG32688-PA [Tribolium castaneum]	1,00E-50
gALL_v3_c23491	1	4	gnl|CDD|190555	pfam03184, DDE_1, DDE supe	ref|XP_003737196.1|	PREDICTED: tigger transposable element-derived protein 6-like [Metaseiulus occidentalis]	2,00E-25
gALL_v3_c23492	2	3	-	-	-	-	-
gALL_v3_c23493	2	2	gnl|CDD|73073	cd02667, Peptidase_C19K, A s	gb|ENN73945.1|	hypothetical protein YQE_09447, partial [Dendroctonus ponderosae] gb|ERL94176.1| hypothetical protein D910_11458 [Dendroctonus ponderosae]	1,00E-31
gALL_v3_c23494	1	4	-	-	-	-	-
gALL_v3_c23495	2	2	-	-	-	-	-
gALL_v3_c23499	1	5	-	-	ref|XP_003700140.1|	PREDICTED: zinc finger protein 622-like [Megachile rotundata]	2,00E-40
gALL_v3_c2350	14	10	-	-	-	-	-
gALL_v3_c23502	3	2	-	-	-	-	-
gALL_v3_c23503	3	1	-	-	-	-	-
gALL_v3_c23504	1	6	gnl|CDD|132723	cd02736, RNAP_III_Rpc1_C, La	ref|XP_001602571.2|	PREDICTED: DNA-directed RNA polymerase III subunit RPC1-like [Nasonia vitripennis]	7,00E-66
gALL_v3_c23505	0	4	-	-	-	-	-
gALL_v3_c23506	0	5	-	-	-	-	-
gALL_v3_c23508	3	3	gnl|CDD|191810	pfam07679, I-set, Immunoglol	ref|XP_002423716.1|	hemicentin, putative [Pediculus humanus corporis] gb|EEB10978.1| hemicentin, putative [Pediculus humanus corporis]	2,00E-32
gALL_v3_c23509	3	1	-	-	-	-	-
gALL_v3_c23510	3	3	-	-	-	-	-
gALL_v3_c23511	1	2	-	-	-	-	-
gALL_v3_c23512	5	12	-	-	-	-	-
gALL_v3_c23513	1	4	-	-	ref|XP_798384.3|	PREDICTED: HEAT repeat-containing protein 7A [Strongylocentrotus purpuratus]	3,00E-19
gALL_v3_c23514	2	3	-	-	ref|XP_003394754.1|	PREDICTED: centromere/kinetochore protein zw10 homolog [Bombus terrestris]	8,00E-33
gALL_v3_c23515	2	2	-	-	ref|XP_002431640.1|	heat containing protein, putative [Pediculus humanus corporis] gb|EEB18902.1| heat containing protein, putative [Pediculus humanus corporis]	2,00E-58
gALL_v3_c23516	10	19	-	-	-	-	-
gALL_v3_c23517	3	3	-	-	gb|AGM32815.1|	protein of unknown function (DUF1113) [Coptotermes formosanus]	5,00E-89
gALL_v3_c23518	2	3	-	-	-	-	-
gALL_v3_c23519	1	3	-	-	-	-	-
gALL_v3_c2352	8	12	-	-	-	-	-
gALL_v3_c23520	4	6	gnl|CDD|178727	PLN03185, PLN03185, phosph	ref|XP_003397996.1|	PREDICTED: LOW QUALITY PROTEIN: alsin-like [Bombus terrestris]	1,00E-120
gALL_v3_c23522	2	2	-	-	-	-	-
gALL_v3_c23523	3	2	-	-	-	-	-
gALL_v3_c23526	2	6	gnl|CDD|190222	pfam02138, Beach, Beige/BEA	ref|XP_002427881.1|	lysosomal trafficking regulator, putative [Pediculus humanus corporis] gb|EEB15143.1| lysosomal trafficking regulator, putative [Pediculus humanus corporis]	1,00E-131
gALL_v3_c23527	0	4	-	-	-	-	-
gALL_v3_c23528	8	4	-	-	-	-	-
gALL_v3_c23529	2	3	-	-	-	-	-
gALL_v3_c2353	20	18	-	-	gb|ENN81243.1|	hypothetical protein YQE_02337, partial [Dendroctonus ponderosae] gb|ERL89568.1| hypothetical protein D910_06933 [Dendroctonus ponderosae]	2,00E-08
gALL_v3_c23530	1	3	-	-	-	-	-
gALL_v3_c23531	5	9	-	-	-	-	-
gALL_v3_c23532	0	6	-	-	-	-	-
gALL_v3_c23533	1	3	-	-	-	-	-
gALL_v3_c23536	2	4	-	-	-	-	-
gALL_v3_c23537	3	1	-	-	gb|AGM32135.1|	hypothetical protein [Coptotermes formosanus]	2,00E-13
gALL_v3_c23538	2	1	-	-	-	-	-
gALL_v3_c23539	3	5	-	-	-	-	-
gALL_v3_c2354	12	12	gnl|CDD|143591	cd07567, biotinidase_like, bio	gb|EFN74417.1|	Vanin-like protein 1 [Camponotus floridanus]	1,00E-12
gALL_v3_c23541	3	2	gnl|CDD|189997	pfam01465, GRIP, GRIP domai	gb|EFA03027.1|	hypothetical protein TcasGA2_TC010451 [Tribolium castaneum]	2,00E-35
gALL_v3_c23542	1	4	-	-	-	-	-
gALL_v3_c23543	1	5	-	-	-	-	-
gALL_v3_c23544	0	4	-	-	-	-	-
gALL_v3_c23545	1	4	-	-	ref|XP_975317.1|	PREDICTED: similar to CG13800 CG13800-PA [Tribolium castaneum]	1,00E-08
gALL_v3_c23546	3	2	-	-	gb|EFA09839.1|	hypothetical protein TcasGA2_TC011986 [Tribolium castaneum]	1,00E-58
gALL_v3_c23547	3	1	-	-	ref|XP_006811856.1|	PREDICTED: RNA-directed DNA polymerase from mobile element jockey-like [Saccoglossus kowalevskii]	5,00E-07
gALL_v3_c23548	2	6	-	-	-	-	-
gALL_v3_c2355	4	9	gnl|CDD|99970	cd03796, GT1_PIG-A_like, This	ref|XP_003425136.1|	PREDICTED: N-acetylglucosaminyl-phosphatidylinositol biosynthetic protein-like [Nasonia vitripennis]	1,00E-177
gALL_v3_c23550	1	2	-	-	-	-	-
gALL_v3_c23551	21	24	-	-	-	-	-
gALL_v3_c23552	1	4	-	-	-	-	-
gALL_v3_c23553	0	4	-	-	-	-	-
gALL_v3_c23555	1	3	-	-	-	-	-
gALL_v3_c23556	3	6	-	-	-	-	-
gALL_v3_c23557	2	3	-	-	ref|XP_321811.4|	AGAP001334-PA [Anopheles gambiae str. PEST] gb|EAA01150.5| AGAP001334-PA [Anopheles gambiae str. PEST]	3,00E-06
gALL_v3_c23558	2	2	-	-	-	-	-
gALL_v3_c2356	10	7	-	-	-	-	-
gALL_v3_c23560	0	3	-	-	-	-	-
gALL_v3_c23561	0	3	-	-	-	-	-
gALL_v3_c23562	8	8	-	-	-	-	-
gALL_v3_c23563	2	3	-	-	ref|XP_005106604.1|	PREDICTED: transforming growth factor-beta receptor-associated protein 1-like [Aplysia californica]	1,00E-34
gALL_v3_c23564	1	5	-	-	-	-	-

gALL_v3_c23565	3	1	-	-	-	-	-
gALL_v3_c23567	1	8	-	-	-	-	-
gALL_v3_c23568	3	2	gnl|CDD|29151	cd00189, TPR, Tetratricopeptic	gb|EUB56783.1|	UDP-N-acetylglucosamine--peptide N-acetylglucosaminyltransferase [Echinococcus granulosus]	1,00E-101
gALL_v3_c23569	3	4	-	-	-	-	-
gALL_v3_c2357	7	5	-	-	-	-	-
gALL_v3_c23570	2	3	-	-	ref|XP_004922065.1|	PREDICTED: uncharacterized protein LOC101745645 [Bombyx mori]	3,00E-11
gALL_v3_c23571	1	3	-	-	-	-	-
gALL_v3_c23572	2	2	-	-	-	-	-
gALL_v3_c23573	3	3	-	-	gb|EFN61389.1|	Importin-11 [Camponotus floridanus]	2,00E-25
gALL_v3_c23575	3	3	gnl|CDD|191607	pfam06775, Seipin, Putative ad	gb|EGI57185.1|	Seipin [Acromyrmex echinatior]	6,00E-32
gALL_v3_c23576	1	5	-	-	ref|XP_003248981.1|	PREDICTED: hypothetical protein LOC100570774, partial [Acyrthosiphon pisum]	4,00E-38
gALL_v3_c23577	0	4	-	-	ref|XP_003243557.1|	PREDICTED: zinc finger BED domain-containing protein 1-like [Acyrthosiphon pisum]	5,00E-15
gALL_v3_c23578	3	6	gnl|CDD|128621	smart00326, SH3, Src homolog	gb|EFN75434.1|	Intersectin-1 [Harpegnathos saltator]	5,00E-80
gALL_v3_c23580	1	7	-	-	-	-	-
gALL_v3_c23581	4	2	-	-	-	-	-
gALL_v3_c23582	3	3	-	-	-	-	-
gALL_v3_c23584	3	3	-	-	-	-	-
gALL_v3_c23585	1	3	-	-	-	-	-
gALL_v3_c23586	2	2	-	-	-	-	-
gALL_v3_c23587	8	4	-	-	ref|XP_968693.1|	PREDICTED: similar to missing oocyte CG7074-PA [Tribolium castaneum] gb|EFA10726.1| missing oocyte-like protein [Tribolium castaneum]	1,00E-118
gALL_v3_c23589	3	1	-	-	ref|XP_551859.3|	AGAP012330-PA [Anopheles gambiae str. PEST] gb|EAL38690.3| AGAP012330-PA [Anopheles gambiae str. PEST]	5,00E-12
gALL_v3_c2359	4	6	-	-	-	-	-
gALL_v3_c23590	1	6	-	-	-	-	-
gALL_v3_c23591	5	3	-	-	-	-	-
gALL_v3_c23593	7	6	-	-	ref|XP_006562338.1|	PREDICTED: uncharacterized protein LOC412326 isoform X2 [Apis mellifera] ref|XP_006562339.1| PREDICTED: uncharacterized protein LOC412326 isoform X3 [Apis mellife	1,00E-104
gALL_v3_c23595	4	2	-	-	gb|EMC78370.1|	Histone H4 transcription factor, partial [Columba livia]	4,00E-85
gALL_v3_c23597	3	1	-	-	-	-	-
gALL_v3_c23598	7	7	-	-	-	-	-
gALL_v3_c23599	3	2	gnl|CDD|119422	cd00896, PI3Kc_III, Phosphoin	gb|AGM32504.1|	phosphatidylinositol 3-kinase catalytic subunit type 3 [Coptotermes formosanus]	1,00E-108
gALL_v3_c236	20	15	-	-	-	-	-
gALL_v3_c2360	7	16	-	-	-	-	-
gALL_v3_c23601	2	1	-	-	-	-	-
gALL_v3_c23602	2	3	-	-	-	-	-
gALL_v3_c23603	1	4	-	-	-	-	-
gALL_v3_c23606	1	5	-	-	ref|XP_006817258.1|	PREDICTED: RNA-directed DNA polymerase from mobile element jockey-like [Saccoglossus kowalevskii]	2,00E-12
gALL_v3_c23607	3	2	-	-	gb|ADI61825.1|	endonuclease-reverse transcriptase [Bombyx mori]	9,00E-21
gALL_v3_c23608	0	3	-	-	gb|ELT89957.1|	hypothetical protein CAPTEDRAFT_198372 [Capitella teleta]	1,00E-16
gALL_v3_c23609	5	3	-	-	-	-	-
gALL_v3_c2361	15	25	gnl|CDD|190226	pfam02145, Rap_GAP, Rap/rar	ref|XP_002088033.1|	Rapgap1 [Drosophila yakuba] gb|EDW87745.1| Rapgap1 [Drosophila yakuba]	1,00E-179
gALL_v3_c23610	0	5	-	-	-	-	-
gALL_v3_c23611	3	1	-	-	-	-	-
gALL_v3_c23613	5	3	-	-	-	-	-
gALL_v3_c23614	6	4	-	-	-	-	-
gALL_v3_c23615	3	3	-	-	-	-	-
gALL_v3_c23616	3	3	-	-	-	-	-
gALL_v3_c23618	0	5	-	-	-	-	-
gALL_v3_c2362	6	5	-	-	gb|ELR53316.1|	hypothetical protein M91_20998, partial [Bos mutus]	1,00E-06
gALL_v3_c23620	1	2	-	-	-	-	-
gALL_v3_c23621	3	2	-	-	gb|EGI69421.1|	Calcineurin-binding protein cabin-1 [Acromyrmex echinatior]	5,00E-23
gALL_v3_c23622	0	3	-	-	-	-	-
gALL_v3_c23623	2	2	gnl|CDD|192374	pfam09770, PAT1, Topoisomer	-	-	-
gALL_v3_c23624	2	3	-	-	-	-	-
gALL_v3_c23625	3	4	gnl|CDD|144664	pfam01153, Glypican, Glypican	ref|XP_003488957.1|	PREDICTED: glypican-4-like [Bombus impatiens]	2,00E-63
gALL_v3_c23626	2	3	-	-	-	-	-
gALL_v3_c23627	7	17	-	-	ref|XP_002422963.1|	reverse transcriptase, putative [Pediculus humanus corporis] gb|EEB10225.1| reverse transcriptase, putative [Pediculus humanus corporis]	2,00E-94
gALL_v3_c23628	1	4	-	-	-	-	-
gALL_v3_c23629	1	3	-	-	ref|XP_006564866.1|	PREDICTED: zinc finger protein 160-like [Apis mellifera]	4,00E-31
gALL_v3_c2363	5	14	-	-	-	-	-
gALL_v3_c23630	1	3	-	-	-	-	-
gALL_v3_c23631	0	3	-	-	-	-	-
gALL_v3_c23632	2	1	-	-	-	-	-
gALL_v3_c23633	0	3	-	-	-	-	-
gALL_v3_c23634	1	4	-	-	-	-	-
gALL_v3_c23635	4	6	-	-	-	-	-
gALL_v3_c23636	0	3	-	-	-	-	-
gALL_v3_c23637	1	2	gnl|CDD|145371	pfam02181, FH2, Formin Hom	gb|EFA10512.1|	hypothetical protein TcasGA2_TC012763 [Tribolium castaneum]	3,00E-54
gALL_v3_c23638	1	3	-	-	-	-	-
gALL_v3_c23639	3	3	-	-	ref|XP_001602708.1|	PREDICTED: tetratricopeptide repeat protein 5-like [Nasonia vitripennis]	3,00E-63
gALL_v3_c2364	9	6	gnl|CDD|149029	pfam07738, Sad1_UNC, Sad1 /	ref|XP_002424807.1|	hect E3 ubiquitin ligase, putative [Pediculus humanus corporis] gb|EEB12069.1| hect E3 ubiquitin ligase, putative [Pediculus humanus corporis]	1,00E-159
gALL_v3_c23640	2	2	-	-	-	-	-
gALL_v3_c23642	4	3	gnl|CDD|145919	pfam03028, Dynein_heavy, Dy	gb|EFN61360.1|	Dynein beta chain, ciliary [Camponotus floridanus]	1,00E-106
gALL_v3_c23643	3	3	-	-	-	-	-
gALL_v3_c23646	3	6	-	-	ref|XP_001656094.1|	hypothetical protein AaeL_AAEL002871 [Aedes aegypti] gb|EAT45893.1| AAEL002871-PA, partial [Aedes aegypti]	5,00E-20
gALL_v3_c23647	0	3	-	-	-	-	-
gALL_v3_c23648	0	4	-	-	gb|EHJ67109.1|	transposase [Danaus plexippus]	6,00E-17
gALL_v3_c23649	4	3	-	-	ref|XP_974531.2|	PREDICTED: similar to conserved hypothetical protein [Tribolium castaneum]	1,00E-164
gALL_v3_c23652	2	3	-	-	-	-	-
gALL_v3_c23653	1	5	-	-	-	-	-
gALL_v3_c23654	1	3	gnl|CDD|166513	PLN02872, PLN02872, triacylg	ref|XP_001662499.1|	lipase 1 precursor [Aedes aegypti] gb|EAT35491.1| AAEL012350-PA [Aedes aegypti]	1,00E-42
gALL_v3_c23655	3	4	-	-	-	-	-
gALL_v3_c23656	2	3	-	-	-	-	-
gALL_v3_c23657	3	10	gnl|CDD|73156	cd01650, RT_nLTR_like, RT_nL	gb|AGM32707.1|	hypothetical protein, partial [Coptotermes formosanus]	1,00E-100
gALL_v3_c2366	17	22	gnl|CDD|165506	PHA03247, PHA03247, large te	ref|XP_003704803.1|	PREDICTED: transcription elongation regulator 1-like [Megachile rotundata]	1,00E-101
gALL_v3_c23660	3	4	-	-	-	-	-
gALL_v3_c23661	0	4	-	-	-	-	-
gALL_v3_c23662	3	4	-	-	-	-	-
gALL_v3_c23663	2	2	-	-	-	-	-
gALL_v3_c23664	1	4	-	-	-	-	-
gALL_v3_c23665	2	4	-	-	ref|XP_003697662.1|	PREDICTED: LOW QUALITY PROTEIN: ectopic P granules protein 5 homolog [Apis florea]	2,00E-56
gALL_v3_c23666	4	3	-	-	-	-	-
gALL_v3_c23667	1	3	-	-	-	-	-
gALL_v3_c23668	16	34	-	-	ref|XP_002431645.1|	hypothetical protein Phum_PHUM538590 [Pediculus humanus corporis] gb|EEB18907.1| hypothetical protein Phum_PHUM538590 [Pediculus humanus corporis]	2,00E-19
gALL_v3_c23669	2	1	-	-	-	-	-
gALL_v3_c2367	5	5	-	-	-	-	-
gALL_v3_c23670	3	1	-	-	-	-	-
gALL_v3_c23671	1	2	-	-	-	-	-
gALL_v3_c23672	2	3	-	-	-	-	-
gALL_v3_c23673	0	3	-	-	-	-	-
gALL_v3_c23674	2	3	-	-	-	-	-
gALL_v3_c23675	1	3	-	-	-	-	-
gALL_v3_c23676	0	3	-	-	-	-	-
gALL_v3_c23678	3	5	-	-	ref|XP_005671952.1|	PREDICTED: tetratricopeptide repeat protein 21B-like isoform X2 [Sus scrofa]	7,00E-88
gALL_v3_c23679	3	3	-	-	gb|EFZ17900.1|	hypothetical protein SINV_07555 [Solenopsis invicta]	1,00E-48
gALL_v3_c2368	9	12	-	-	ref|XP_002426190.1|	hypothetical protein Phum_PHUM240760 [Pediculus humanus corporis] gb|EEB13452.1| hypothetical protein Phum_PHUM240760 [Pediculus humanus corporis]	2,00E-38
gALL_v3_c23680	3	1	-	-	-	-	-
gALL_v3_c23681	0	3	-	-	-	-	-
gALL_v3_c23682	2	2	-	-	-	-	-
gALL_v3_c23683	1	2	-	-	-	-	-
gALL_v3_c23685	2	3	-	-	-	-	-
gALL_v3_c23686	2	10	-	-	-	-	-
gALL_v3_c23688	1	5	-	-	-	-	-
gALL_v3_c23689	1	5	-	-	-	-	-
gALL_v3_c2369	5	3	gnl|CDD|128919	smart00674, CENPB, Putative	ref|XP_001380458.2|	PREDICTED: major centromere autoantigen B-like [Monodelphis domestica]	5,00E-25
gALL_v3_c23690	2	5	-	-	-	-	-
gALL_v3_c23691	2	2	-	-	-	-	-
gALL_v3_c23692	3	2	-	-	gb|ETN63181.1|	sterol O-acyltransferase 2 [Anopheles darlingi]	8,00E-49
gALL_v3_c23693	4	8	-	-	-	-	-
gALL_v3_c23694	3	4	-	-	gb|EFN87441.1|	hypothetical protein EAI_00377 [Harpegnathos saltator]	2,00E-09
gALL_v3_c23695	0	6	-	-	gb|AEM44817.1|	unknown [Pectinophora gossypiella]	5,00E-11
gALL_v3_c23696	1	4	-	-	-	-	-
gALL_v3_c23697	1	3	-	-	gb|EFZ19146.1|	hypothetical protein SINV_06316 [Solenopsis invicta]	6,00E-07
gALL_v3_c23698	1	5	gnl|CDD|128829	smart00557, IG_FLMN, Filamin	gb|ERL95534.1|	hypothetical protein D910_12796 [Dendroctonus ponderosae]	4,00E-37
gALL_v3_c23700	3	2	-	-	-	-	-
gALL_v3_c23701	2	2	-	-	-	-	-
gALL_v3_c23703	2	2	-	-	-	-	-
gALL_v3_c23704	0	4	-	-	-	-	-
gALL_v3_c23705	3	2	-	-	-	-	-
gALL_v3_c23706	2	5	-	-	-	-	-
gALL_v3_c23707	5	5	-	-	gb|EFN83743.1|	Activating transcription factor 7-interacting protein 1 [Harpegnathos saltator]	1,00E-50
gALL_v3_c2371	2	7	-	-	ref|XP_005102572.1|	PREDICTED: tetratricopeptide repeat protein 17-like [Aplysia californica]	1,00E-10
gALL_v3_c23710	2	2	-	-	-	-	-
gALL_v3_c23712	0	5	-	-	-	-	-
gALL_v3_c23713	1	4	gnl|CDD|30099	cd01377, MYSc_type_II, Myos	gb|AAM97634.1|	myosin heavy chain type II [Ammophila sp. IRT-2002]	2,00E-10
gALL_v3_c23714	1	5	gnl|CDD|191169	pfam05028, PARG_cat, Poly (A	ref|XP_004561265.1|	PREDICTED: poly(ADP-ribose) glycohydrolase-like [Maylandia zebra]	4,00E-31
gALL_v3_c23715	1	3	-	-	-	-	-
gALL_v3_c23716	1	3	-	-	-	-	-
gALL_v3_c23717	1	3	-	-	-	-	-
gALL_v3_c23718	3	3	-	-	-	-	-
gALL_v3_c23719	4	6	-	-	ref|XP_001602535.2|	PREDICTED: hypothetical protein LOC100118603 [Nasonia vitripennis]	2,00E-75
gALL_v3_c2372	21	16	gnl|CDD|31442	COG1250, FadB, 3-hydroxyacyl	ref|NP_001155916.1|	L-3-hydroxyacyl-Coenzyme A dehydrogenase [Acyrthosiphon pisum]	1,00E-140
gALL_v3_c23720	2	4	-	-	-	-	-
gALL_v3_c23721	5	3	-	-	ref|XP_002428014.1|	mitochondrial glutamate carrier, putative [Pediculus humanus corporis] gb|EEB15276.1| mitochondrial glutamate carrier, putative [Pediculus humanus corporis]	5,00E-38
gALL_v3_c23722	0	4	-	-	gb|ETN62709.1|	structural maintenance of chromosomes smc3 [Anopheles darlingi]	2,00E-12
gALL_v3_c23723	1	5	-	-	ref|XP_003248262.1|	PREDICTED: putative nuclease HARBI1-like [Acyrthosiphon pisum]	2,00E-07

gALL_v3_c23724	1	3	-	-	-	-	-
gALL_v3_c23725	2	2	-	-	ref|XP_308673.4|	AGAP007085-PA [Anopheles gambiae str. PEST] gb|EAA03988.5| AGAP007085-PA [Anopheles gambiae str. PEST]	3,00E-06
gALL_v3_c23726	2	4	gnl|CDD|161940	TIGR00584, mug, mismatch-sp	ref|XP_002427210.1|	G/t mismatch-specific thymine DNA glycosylase, putative [Pediculus humanus corporis] gb|EEB14472.1| G/t mismatch-specific thymine DNA glycosylase, putative [Pediculı	6,00E-77
gALL_v3_c23727	1	3	-	-	-	-	-
gALL_v3_c23728	0	3	-	-	-	-	-
gALL_v3_c23729	2	2	-	-	-	-	-
gALL_v3_c2373	1	6	-	-	-	-	-
gALL_v3_c23730	2	10	-	-	gb|ERL95993.1|	hypothetical protein D910_00759, partial [Dendroctonus ponderosae]	1,00E-06
gALL_v3_c23732	1	3	-	-	-	-	-
gALL_v3_c23733	3	2	-	-	-	-	-
gALL_v3_c23734	3	4	gnl|CDD|73332	cd02868, PseudoU_synth_hTr	gb|ERL92096.1|	hypothetical protein D910_09417 [Dendroctonus ponderosae]	1,00E-51
gALL_v3_c23735	3	1	-	-	-	-	-
gALL_v3_c23736	1	3	-	-	ref|XP_973609.1|	PREDICTED: similar to GA11962-PA [Tribolium castaneum] gb|EFA08146.1| hypothetical protein TcasGA2_TC005763 [Tribolium castaneum]	4,00E-10
gALL_v3_c23737	5	3	gnl|CDD|188098	TIGR00957, MRP_assoc_pro, r	gb|EFN68626.1|	cGMP-dependent protein kinase, isozyme 1 [Camponotus floridanus]	2,00E-84
gALL_v3_c23739	2	4	gnl|CDD|133158	cd06094, RP_Saci_like, RP_Sac	ref|XP_001807263.1|	PREDICTED: similar to gag-pol polyprotein [Tribolium castaneum]	1,00E-61
gALL_v3_c2374	1	6	-	-	-	-	-
gALL_v3_c23740	3	2	-	-	ref|XP_001853641.1|	conserved hypothetical protein [Culex quinquefasciatus] gb|EDS34324.1| conserved hypothetical protein [Culex quinquefasciatus]	3,00E-11
gALL_v3_c23741	0	4	-	-	-	-	-
gALL_v3_c23742	1	6	-	-	-	-	-
gALL_v3_c23743	3	3	-	-	ref|XP_001599468.1|	PREDICTED: hypothetical protein LOC100114451 [Nasonia vitripennis]	4,00E-15
gALL_v3_c23744	1	3	-	-	-	-	-
gALL_v3_c23745	2	4	-	-	-	-	-
gALL_v3_c23746	2	1	-	-	-	-	-
gALL_v3_c23747	1	2	-	-	-	-	-
gALL_v3_c23748	0	5	gnl|CDD|150860	pfam10254, Pacs-1, PACS-1 cy	gb|EFZ20862.1|	hypothetical protein SINV_80540 [Solenopsis invicta]	2,00E-70
gALL_v3_c23749	3	1	-	-	-	-	-
gALL_v3_c2375	4	6	-	-	ref|XP_004924890.1|	PREDICTED: uncharacterized protein LOC101746893 [Bombyx mori]	3,00E-11
gALL_v3_c23750	0	6	-	-	ref|XP_002427087.1|	hypothetical protein Phum_PHUM294190 [Pediculus humanus corporis] gb|EEB14349.1| hypothetical protein Phum_PHUM294190 [Pediculus humanus corporis]	7,00E-18
gALL_v3_c23751	1	3	-	-	-	-	-
gALL_v3_c23754	3	1	-	-	-	-	-
gALL_v3_c23755	11	14	-	-	-	-	-
gALL_v3_c23756	3	5	-	-	ref|XP_003247363.1|	PREDICTED: hypothetical protein LOC100575801 [Acyrthosiphon pisum]	3,00E-10
gALL_v3_c23757	5	4	gnl|CDD|189372	pfam00067, p450, Cytochrom	dbj|BAD84176.1|	cytochrome P450 [Hodotermopsis sjostedti]	1,00E-86
gALL_v3_c23758	1	3	-	-	-	-	-
gALL_v3_c23759	1	3	-	-	gb|EFN63197.1|	Autophagy-related protein 2-like protein B [Camponotus floridanus]	2,00E-34
gALL_v3_c2376	4	6	-	-	gb|EFA08697.1|	hypothetical protein TcasGA2_TC006368 [Tribolium castaneum]	2,00E-44
gALL_v3_c23761	0	3	gnl|CDD|128471	smart00174, RHO, Rho (Ras ho	ref|XP_001319379.1|	small GTP-binding protein [Trichomonas vaginalis G3] gb|EAY07156.1| small GTP-binding protein, putative [Trichomonas vaginalis G3]	2,00E-32
gALL_v3_c23762	3	1	-	-	-	-	-
gALL_v3_c23763	0	5	-	-	-	-	-
gALL_v3_c23764	1	3	-	-	ref|XP_001601432.1|	PREDICTED: probable ATP-dependent RNA helicase DDX47-like [Nasonia vitripennis]	2,00E-22
gALL_v3_c23765	3	1	-	-	-	-	-
gALL_v3_c23766	2	2	-	-	-	-	-
gALL_v3_c23767	1	8	-	-	-	-	-
gALL_v3_c23768	0	4	-	-	-	-	-
gALL_v3_c23769	1	2	-	-	-	-	-
gALL_v3_c23770	4	7	-	-	ref|XP_003487086.1|	PREDICTED: hypothetical protein LOC100742623 [Bombus impatiens]	1,00E-18
gALL_v3_c23771	1	6	-	-	-	-	-
gALL_v3_c23772	0	5	-	-	-	-	-
gALL_v3_c23773	3	1	-	-	-	-	-
gALL_v3_c23774	0	5	-	-	ref|XP_003251327.1|	PREDICTED: UBA-like domain-containing protein 2-like isoform X1 [Apis mellifera] ref|XP_006612992.1| PREDICTED: UBA-like domain-containing protein 2-like isoform X2 |	2,00E-33
gALL_v3_c23776	0	4	-	-	-	-	-
gALL_v3_c23777	5	12	-	-	-	-	-
gALL_v3_c23778	2	2	-	-	ref|XP_003394362.1|	PREDICTED: e3 ubiquitin-protein ligase UBR4-like [Bombus terrestris]	7,00E-49
gALL_v3_c23779	2	5	gnl|CDD|178687	PLN03142, PLN03142, Probab	gb|EFN75028.1|	Chromatin-remodeling complex ATPase chain Iswi [Camponotus floridanus]	1,00E-112
gALL_v3_c2378	7	4	gnl|CDD|129044	smart00808, FABD, F-actin bin	ref|XP_968668.2|	PREDICTED: similar to AGAP004989-PB [Tribolium castaneum]	4,00E-45
gALL_v3_c23780	2	5	-	-	-	-	-
gALL_v3_c23781	3	4	-	-	-	-	-
gALL_v3_c23782	0	5	-	-	-	-	-
gALL_v3_c23783	3	1	-	-	-	-	-
gALL_v3_c23784	3	1	-	-	-	-	-
gALL_v3_c23785	0	3	-	-	-	-	-
gALL_v3_c23786	2	1	-	-	-	-	-
gALL_v3_c23787	3	2	-	-	-	-	-
gALL_v3_c23788	2	2	-	-	-	-	-
gALL_v3_c23789	1	3	-	-	-	-	-
gALL_v3_c2379	10	12	-	-	gb|EFA12330.1|	hypothetical protein TcasGA2_TC005158 [Tribolium castaneum]	6,00E-06
gALL_v3_c23790	0	4	-	-	gb|EGI66758.1|	Protein bric-a-brac 1 [Acromyrmex echinatior]	1,00E-13
gALL_v3_c23791	12	11	-	-	-	-	-
gALL_v3_c23792	3	2	-	-	gb|ADI61823.1|	endonuclease-reverse transcriptase [Bombyx mori]	5,00E-37
gALL_v3_c23794	1	3	-	-	-	-	-
gALL_v3_c23795	2	1	-	-	ref|XP_004934072.1|	PREDICTED: zinc finger protein 233-like, partial [Bombyx mori]	2,00E-57
gALL_v3_c23796	3	3	-	-	ref|XP_001943933.2|	PREDICTED: protein phosphatase 1 regulatory subunit 16A-like [Acyrthosiphon pisum]	1,00E-23
gALL_v3_c23797	1	3	-	-	-	-	-
gALL_v3_c23798	1	3	-	-	-	-	-
gALL_v3_c23799	3	2	-	-	-	-	-
gALL_v3_c238	20	24	-	-	-	-	-
gALL_v3_c23800	3	3	-	-	ref|XP_003426293.1|	PREDICTED: bleomycin hydrolase-like isoform 2 [Nasonia vitripennis]	4,00E-09
gALL_v3_c23801	3	2	gnl|CDD|113924	pfam05172, MPPN, MPPN (rrr	ref|XP_002427705.1|	Nucleoporin NUP53, putative [Pediculus humanus corporis] gb|EEB14967.1| Nucleoporin NUP53, putative [Pediculus humanus corporis]	2,00E-37
gALL_v3_c23802	2	1	-	-	-	-	-
gALL_v3_c23803	3	2	-	-	-	-	-
gALL_v3_c23804	4	5	-	-	-	-	-
gALL_v3_c23805	2	9	-	-	-	-	-
gALL_v3_c23806	4	3	-	-	-	-	-
gALL_v3_c23807	1	3	-	-	-	-	-
gALL_v3_c23808	2	4	-	-	-	-	-
gALL_v3_c23809	2	2	-	-	-	-	-
gALL_v3_c2381	3	6	-	-	-	-	-
gALL_v3_c23810	2	3	-	-	ref|XP_006029730.1|	PREDICTED: histone-lysine N-methyltransferase SETMAR-like [Alligator sinensis]	2,00E-08
gALL_v3_c23811	4	6	-	-	gb|EFN65619.1|	Putative uncharacterized transposon-derived protein F52C9.6 [Camponotus floridanus]	7,00E-16
gALL_v3_c23814	3	1	-	-	-	-	-
gALL_v3_c23815	3	3	-	-	gb|ABB00685.1|	suppressor of white-apricot [Drosophila orena]	8,00E-06
gALL_v3_c23816	1	3	-	-	-	-	-
gALL_v3_c23817	16	20	-	-	-	-	-
gALL_v3_c23818	4	2	-	-	-	-	-
gALL_v3_c23819	0	5	-	-	-	-	-
gALL_v3_c2382	13	20	gnl|CDD|32267	COG2084, MmsB, 3-hydroxyis	ref|XP_003702255.1|	PREDICTED: putative oxidoreductase GLYR1 homolog [Megachile rotundata]	1,00E-159
gALL_v3_c23820	2	2	-	-	-	-	-
gALL_v3_c23821	7	12	-	-	-	-	-
gALL_v3_c23822	6	5	gnl|CDD|128807	smart00534, MUTSac, ATPase	ref|XP_006811682.1|	PREDICTED: DNA mismatch repair protein Msh3-like [Saccoglossus kowalevskii]	5,00E-25
gALL_v3_c23824	4	5	-	-	-	-	-
gALL_v3_c23825	1	3	-	-	ref|XP_005464674.1|	PREDICTED: RNA-directed DNA polymerase from mobile element jockey-like [Oreochromis niloticus]	2,00E-07
gALL_v3_c23827	3	1	-	-	-	-	-
gALL_v3_c23828	3	1	gnl|CDD|190464	pfam02883, Alpha_adaptinC2,	gb|EFN81316.1|	ADP-ribosylation factor-binding protein GGA1 [Harpegnathos saltator]	2,00E-49
gALL_v3_c23829	2	2	-	-	-	-	-
gALL_v3_c2383	4	7	-	-	-	-	-
gALL_v3_c23830	2	2	-	-	ref|XP_002430050.1|	class A rhodopsin-like G-protein coupled receptor GPRadr1, putative [Pediculus humanus corporis] gb|EEB17312.1| class A rhodopsin-like G-protein coupled receptor GPR	7,00E-45
gALL_v3_c23832	2	4	-	-	-	-	-
gALL_v3_c23833	3	5	gnl|CDD|129951	TIGR00873, gnd, 6-phosphoglu	ref|NP_001083291.1|	6-phosphogluconate dehydrogenase, decarboxylating [Xenopus laevis] gb|AAH59958.1| Pgd protein [Xenopus laevis]	1,00E-57
gALL_v3_c23834	2	1	-	-	-	-	-
gALL_v3_c23835	1	2	-	-	-	-	-
gALL_v3_c23836	3	1	-	-	-	-	-
gALL_v3_c23837	2	2	-	-	-	-	-
gALL_v3_c23838	0	5	-	-	-	-	-
gALL_v3_c23839	4	2	gnl|CDD|187550	cd05239, GDP_FS_SDR_e, GDI	tpg|DAA34359.1|	TPA_inf: GDP-L-fucose synthetase [Amblyomma variegatum]	4,00E-57
gALL_v3_c2384	7	10	gnl|CDD|191470	pfam06201, PITH, C-terminal p	ref|XP_974503.1|	PREDICTED: similar to thioredoxin-like protein [Tribolium castaneum] gb|EFA06575.1| hypothetical protein TcasGA2_TC009487 [Tribolium castaneum]	1,00E-149
gALL_v3_c23840	5	2	-	-	-	-	-
gALL_v3_c23841	2	2	-	-	-	-	-
gALL_v3_c23842	1	2	-	-	-	-	-
gALL_v3_c23843	3	5	-	-	gb|EFN87210.1|	hypothetical protein EAI_12900 [Harpegnathos saltator]	6,00E-12
gALL_v3_c23844	1	3	-	-	-	-	-
gALL_v3_c23845	1	3	-	-	-	-	-
gALL_v3_c23846	3	2	-	-	-	-	-
gALL_v3_c23847	0	3	-	-	-	-	-
gALL_v3_c23848	4	3	-	-	-	-	-
gALL_v3_c23849	0	4	-	-	-	-	-
gALL_v3_c2385	7	15	-	-	-	-	-
gALL_v3_c23850	0	3	-	-	-	-	-
gALL_v3_c23851	0	5	-	-	-	-	-
gALL_v3_c23852	3	3	-	-	-	-	-
gALL_v3_c23853	2	4	-	-	-	-	-
gALL_v3_c23854	0	4	-	-	-	-	-
gALL_v3_c23855	0	4	-	-	-	-	-
gALL_v3_c23856	1	3	-	-	-	-	-
gALL_v3_c23857	0	3	-	-	-	-	-
gALL_v3_c23858	3	4	-	-	-	-	-
gALL_v3_c2386	10	6	-	-	-	-	-
gALL_v3_c23860	3	1	-	-	-	-	-
gALL_v3_c23861	2	2	-	-	-	-	-
gALL_v3_c23862	2	3	-	-	ref|XP_004929205.1|	PREDICTED: tetratricopeptide repeat protein 14 homolog isoform X1 [Bombyx mori]	9,00E-17
gALL_v3_c23863	1	6	-	-	ref|XP_973673.1|	PREDICTED: similar to vesicle associated protein, putative [Tribolium castaneum]	2,00E-17
gALL_v3_c23864	1	3	-	-	-	-	-
gALL_v3_c23865	4	4	-	-	-	-	-
gALL_v3_c23866	0	4	gnl|CDD|31445	COG1253, TlyC, Hemolysins ar	ref|XP_006563574.1|	PREDICTED: metal transporter CNNM2-like isoform X2 [Apis mellifera]	1,00E-101
gALL_v3_c23868	8	7	-	-	-	-	-

gALL_v3_c23869	0	5	-	-	-	-	-
gALL_v3_c23870	3	4	gnl|CDD|178163	PLN02548, PLN02548, adenos	gb|EFN72749.1|	Adenosine kinase 2 [Camponotus floridanus]	1,00E-32
gALL_v3_c23872	5	4	gnl|CDD|29331	cd00256, VATPase_H, VATPase	gb|AFN11665.1|	vacuolar ATPase subunit H [Locusta migratoria manilensis]	1,00E-38
gALL_v3_c23874	3	3	gnl|CDD|32062	COG1878, COG1878, Predicted	ref|NP_001156463.1|	uncharacterized protein LOC100302387 precursor [Acyrthosiphon pisum] dbj|BAH72639.1| ACYPI46563 [Acyrthosiphon pisum]	4,00E-19
gALL_v3_c23876	1	3	-	-	-	-	-
gALL_v3_c23877	1	3	-	-	-	-	-
gALL_v3_c23878	1	3	-	-	-	-	-
gALL_v3_c23879	2	1	-	-	-	-	-
gALL_v3_c23880	0	4	-	-	-	-	-
gALL_v3_c23881	2	3	-	-	-	-	-
gALL_v3_c23884	1	3	gnl|CDD|146960	pfam04572, Gb3_synth, Alpha	ref|XP_003708493.1|	PREDICTED: lactosylceramide 4-alpha-galactosyltransferase-like [Megachile rotundata]	2,00E-44
gALL_v3_c23885	1	5	-	-	gb|EFA00556.1|	hypothetical protein TcasGA2_TC003423 [Tribolium castaneum]	7,00E-14
gALL_v3_c23886	1	4	-	-	ref|XP_002049182.1|	GJ20889 [Drosophila virilis] gb|EDW60375.1| GJ20889 [Drosophila virilis]	2,00E-07
gALL_v3_c23887	3	2	-	-	-	-	-
gALL_v3_c23888	1	4	-	-	gb|ELU09604.1|	hypothetical protein CAPTEDRAFT_229371 [Capitella teleta]	5,00E-07
gALL_v3_c23889	1	2	-	-	-	-	-
gALL_v3_c23890	3	1	gnl|CDD|178495	PLN02907, PLN02907, glutama	gb|ENN77483.1|	hypothetical protein YQE_06011, partial [Dendroctonus ponderosae]	3,00E-63
gALL_v3_c23891	21	20	gnl|CDD|128473	smart00176, RAN, Ran (Ras-re	ref|XP_393761.1|	PREDICTED: GTP-binding nuclear protein Ran isoform X2 [Apis mellifera] ref|XP_006570503.1| PREDICTED: GTP-binding nuclear protein Ran isoform X1 [Apis mellifera] ref	3,00E-48
gALL_v3_c23892	1	5	-	-	-	-	-
gALL_v3_c23893	1	3	-	-	ref|XP_003701187.1|	PREDICTED: leucine-rich repeat and WD repeat-containing protein KIAA1239-like [Megachile rotundata]	1,00E-69
gALL_v3_c23894	3	1	-	-	-	-	-
gALL_v3_c23895	4	4	-	-	-	-	-
gALL_v3_c23896	2	2	-	-	-	-	-
gALL_v3_c23897	2	2	-	-	-	-	-
gALL_v3_c23898	0	5	-	-	-	-	-
gALL_v3_c23899	0	4	-	-	-	-	-
gALL_v3_c239	9	17	-	-	ref|XP_003692998.1|	PREDICTED: uncharacterized protein LOC100865023 isoform 2 [Apis florea]	2,00E-22
gALL_v3_c2390	25	19	-	-	-	-	-
gALL_v3_c23901	2	3	-	-	-	-	-
gALL_v3_c23902	2	1	-	-	-	-	-
gALL_v3_c23903	3	4	-	-	gb|AGM32681.1|	ecdysteroid UDP-glucosyltransferase protein, partial [Coptotermes formosanus]	2,00E-33
gALL_v3_c23905	1	3	-	-	sp|Q9XZ71.1|TNNT_PEF	RecName: Full=Troponin T; Short=TnT gb|AAD33603.1|AF133520_1 troponin T [Periplaneta americana]	9,00E-06
gALL_v3_c23906	2	6	gnl|CDD|190093	pfam01757, Acyl_transf_3, Ac	ref|XP_001605172.2|	PREDICTED: nose resistant to fluoxetine protein 6-like [Nasonia vitripennis]	1,00E-55
gALL_v3_c23907	2	1	-	-	-	-	-
gALL_v3_c23909	1	5	-	-	-	-	-
gALL_v3_c23910	0	3	-	-	-	-	-
gALL_v3_c23911	3	4	-	-	gb|ACD76747.1|	female neotenic-specific protein 8 [Cryptotermes secundus]	7,00E-16
gALL_v3_c23912	3	1	-	-	-	-	-
gALL_v3_c23913	3	1	gnl|CDD|190232	pfam02170, PAZ, PAZ domain.	pdb|1R4K|A	Chain A, Solution Structure Of The Drosophila Argonaute 1 Paz Domain	2,00E-86
gALL_v3_c23914	3	2	gnl|CDD|189334	pfam00001, 7tm_1, 7 transme	ref|XP_003487615.1|	PREDICTED: neuromedin-B receptor-like [Bombus impatiens]	7,00E-15
gALL_v3_c23915	3	1	-	-	-	-	-
gALL_v3_c23916	2	3	-	-	-	-	-
gALL_v3_c23917	4	9	-	-	-	-	-
gALL_v3_c23918	2	1	-	-	-	-	-
gALL_v3_c23919	0	5	-	-	-	-	-
gALL_v3_c2392	5	8	-	-	-	-	-
gALL_v3_c23920	0	5	-	-	-	-	-
gALL_v3_c23921	4	4	-	-	ref|XP_002427594.1|	conserved hypothetical protein [Pediculus humanus corporis] gb|EEB14856.1| conserved hypothetical protein [Pediculus humanus corporis]	1,00E-34
gALL_v3_c23922	1	4	-	-	-	-	-
gALL_v3_c23923	0	4	-	-	-	-	-
gALL_v3_c23924	3	7	-	-	-	-	-
gALL_v3_c23925	5	4	-	-	-	-	-
gALL_v3_c23926	1	2	-	-	-	-	-
gALL_v3_c23927	3	2	-	-	gb|EFZ21560.1|	hypothetical protein SINV_06372 [Solenopsis invicta]	2,00E-39
gALL_v3_c23928	5	4	-	-	-	-	-
gALL_v3_c2393	4	10	-	-	-	-	-
gALL_v3_c23931	4	5	-	-	-	-	-
gALL_v3_c23932	2	2	-	-	-	-	-
gALL_v3_c23933	1	3	-	-	-	-	-
gALL_v3_c23934	0	4	-	-	-	-	-
gALL_v3_c23935	1	3	-	-	-	-	-
gALL_v3_c23937	2	2	-	-	-	-	-
gALL_v3_c23939	4	12	gnl|CDD|29138	cd00176, SPEC, Spectrin repea	gb|EFA06228.1|	hypothetical protein TcasGA2_TC009077 [Tribolium castaneum]	2,00E-58
gALL_v3_c2394	38	26	gnl|CDD|192143	pfam08752, Gamma-COP, Coa	ref|XP_973414.1|	PREDICTED: similar to RE62270p [Tribolium castaneum] gb|EFA09678.1| hypothetical protein TcasGA2_TC011806 [Tribolium castaneum]	0
gALL_v3_c23940	2	1	-	-	gb|ESO07079.1|	hypothetical protein HELRODRAFT_184661 [Helobdella robusta]	7,00E-19
gALL_v3_c23942	3	4	gnl|CDD|193033	pfam12451, VPS11_C, Vacuola	gb|EGI61136.1|	Vacuolar protein sorting-associated protein 11-like protein [Acromyrmex echinatior]	4,00E-77
gALL_v3_c23943	2	2	-	-	-	-	-
gALL_v3_c23945	6	5	-	-	-	-	-
gALL_v3_c23946	0	3	-	-	-	-	-
gALL_v3_c23947	1	3	-	-	-	-	-
gALL_v3_c23948	2	1	-	-	-	-	-
gALL_v3_c23949	0	3	-	-	ref|XP_003516209.1|	PREDICTED: zinc finger protein 120-like, partial [Cricetulus griseus]	1,00E-51
gALL_v3_c2395	10	14	-	-	-	-	-
gALL_v3_c23950	3	4	gnl|CDD|73156	cd01650, RT_nLTR_like, RT_nL	ref|XP_004205527.1|	PREDICTED: RNA-directed DNA polymerase from mobile element jockey-like [Hydra vulgaris]	1,00E-32
gALL_v3_c23952	2	2	gnl|CDD|128386	smart00072, GuKc, Guanylate	gb|EFZ11533.1|	hypothetical protein SINV_10211 [Solenopsis invicta]	3,00E-63
gALL_v3_c23953	3	2	gnl|CDD|128516	smart00220, S_TKc, Serine/Th	ref|XP_001605282.1|	PREDICTED: serine/threonine-protein kinase 11-like isoform 1 [Nasonia vitripennis]	2,00E-52
gALL_v3_c23955	0	3	-	-	-	-	-
gALL_v3_c23957	0	5	-	-	-	-	-
gALL_v3_c23959	5	2	-	-	-	-	-
gALL_v3_c2396	9	6	-	-	-	-	-
gALL_v3_c23960	2	1	-	-	-	-	-
gALL_v3_c23962	1	3	-	-	-	-	-
gALL_v3_c23965	2	2	-	-	ref|XP_006639552.1|	PREDICTED: LOW QUALITY PROTEIN: engulfment and cell motility protein 2-like [Lepisosteus oculatus]	6,00E-12
gALL_v3_c23966	2	3	gnl|CDD|29257	cd00200, WD40, WD40 domai	ref|XP_974788.1|	PREDICTED: similar to GA20229-PA [Tribolium castaneum] gb|EEZ98265.1| hypothetical protein TcasGA2_TC000707 [Tribolium castaneum]	3,00E-90
gALL_v3_c23967	2	3	-	-	ref|XP_003699236.1|	PREDICTED: transcription elongation factor SPT6-like [Megachile rotundata]	4,00E-88
gALL_v3_c23968	2	3	-	-	-	-	-
gALL_v3_c23969	2	2	-	-	-	-	-
gALL_v3_c2397	38	33	-	-	-	-	-
gALL_v3_c23970	0	3	-	-	-	-	-
gALL_v3_c23971	1	3	-	-	-	-	-
gALL_v3_c23973	1	5	-	-	ref|XP_006562052.1|	PREDICTED: unconventional myosin-XVIIIa isoform X3 [Apis mellifera]	8,00E-49
gALL_v3_c23974	2	3	-	-	-	-	-
gALL_v3_c23975	2	2	-	-	gb|ESO83649.1|	hypothetical protein LOTGIDRAFT_222500 [Lottia gigantea]	2,00E-12
gALL_v3_c23976	1	4	-	-	-	-	-
gALL_v3_c23977	2	2	-	-	-	-	-
gALL_v3_c23978	4	10	-	-	-	-	-
gALL_v3_c2398	13	9	-	-	ref|XP_006132930.1|	PREDICTED: jerky protein-like [Pelodiscus sinensis]	2,00E-13
gALL_v3_c23980	1	3	-	-	-	-	-
gALL_v3_c23981	2	3	gnl|CDD|150430	pfam09755, DUF2046, Unchar	ref|XP_001599067.2|	PREDICTED: coiled-coil domain-containing protein 6-like [Nasonia vitripennis]	1,00E-111
gALL_v3_c23982	3	2	-	-	-	-	-
gALL_v3_c23984	1	6	-	-	-	-	-
gALL_v3_c23985	3	1	-	-	-	-	-
gALL_v3_c23986	1	2	-	-	-	-	-
gALL_v3_c23987	0	4	-	-	-	-	-
gALL_v3_c23988	1	5	gnl|CDD|133314	cd04114, Rab30, Rab30 subfar	dbj|BAN21044.1|	rab 19, 41 and [Riptortus pedestris]	1,00E-100
gALL_v3_c23989	2	4	-	-	-	-	-
gALL_v3_c2399	5	14	-	-	-	-	-
gALL_v3_c23990	0	5	-	-	-	-	-
gALL_v3_c23991	1	5	gnl|CDD|189741	pfam00850, Hist_deacetyl, His	gb|EFZ15318.1|	hypothetical protein SINV_11382 [Solenopsis invicta]	6,00E-33
gALL_v3_c23992	2	2	-	-	-	-	-
gALL_v3_c23993	3	1	gnl|CDD|176386	cd01791, Ubl5, UBL5 ubiquitin	ref|XP_003394299.1|	PREDICTED: ubiquitin-like protein 5-like [Bombus terrestris] ref|XP_003490890.1| PREDICTED: ubiquitin-like protein 5-like [Bombus impatiens] ref|XP_003699932.1| PRED	2,00E-31
gALL_v3_c23994	2	2	-	-	-	-	-
gALL_v3_c23997	3	2	gnl|CDD|29151	cd00189, TPR, Tetratricopeptic	emb|CAF91015.1|	unnamed protein product [Tetraodon nigroviridis]	1,00E-100
gALL_v3_c24	5	7	-	-	-	-	-
gALL_v3_c240	12	15	gnl|CDD|188655	cd09823, peroxinectin_like, pe	ref|XP_003397065.1|	PREDICTED: peroxidase-like isoform 2 [Bombus terrestris]	1,00E-36
gALL_v3_c2400	5	7	-	-	-	-	-
gALL_v3_c24000	1	4	-	-	-	-	-
gALL_v3_c24001	1	5	-	-	-	-	-
gALL_v3_c24003	1	4	-	-	ref|XP_003771124.1|	PREDICTED: N-acetylglucosamine-1-phosphotransferase subunits alpha/beta, partial [Sarcophilus harrisii]	1,00E-30
gALL_v3_c24004	0	3	-	-	-	-	-
gALL_v3_c24005	1	2	-	-	-	-	-
gALL_v3_c24006	1	3	-	-	-	-	-
gALL_v3_c24007	3	1	-	-	-	-	-
gALL_v3_c24008	2	1	gnl|CDD|133140	cd05473, beta_secretase_like,	ref|XP_002590109.1|	hypothetical protein BRAFLDRAFT_83387 [Branchiostoma floridae] gb|EEN46120.1| hypothetical protein BRAFLDRAFT_83387 [Branchiostoma floridae]	1,00E-24
gALL_v3_c24009	3	5	-	-	gb|ESO04366.1|	hypothetical protein HELRODRAFT_172731 [Helobdella robusta]	1,00E-32
gALL_v3_c2401	2	8	-	-	ref|XP_003492249.1|	PREDICTED: zinc finger SWIM domain-containing protein KIAA0913-like [Bombus impatiens]	3,00E-76
gALL_v3_c24010	2	3	-	-	-	-	-
gALL_v3_c24011	2	4	-	-	-	-	-
gALL_v3_c24012	3	1	-	-	-	-	-
gALL_v3_c24013	1	3	-	-	gb|EFN60815.1|	Pecanex-like protein 1 [Camponotus floridanus]	7,00E-27
gALL_v3_c24015	1	2	-	-	-	-	-
gALL_v3_c24016	3	1	-	-	ref|XP_002070410.1|	GK12038 [Drosophila willistoni] gb|EDW81396.1| GK12038 [Drosophila willistoni]	5,00E-07
gALL_v3_c24018	4	5	-	-	gb|EEZ99045.1|	hypothetical protein TcasGA2_TC004919 [Tribolium castaneum]	1,00E-19
gALL_v3_c24019	2	4	-	-	ref|XP_003485868.1|	PREDICTED: protein Daple-like [Bombus impatiens]	9,00E-50
gALL_v3_c2402	6	3	-	-	-	-	-
gALL_v3_c24020	2	5	-	-	-	-	-
gALL_v3_c24021	1	3	gnl|CDD|173506	PTZ00281, PTZ00281, actin; Pr	gb|AFR74960.1|	actin, partial [Rana clamitans]	1,00E-110
gALL_v3_c24022	0	5	-	-	-	-	-
gALL_v3_c24023	2	5	-	-	ref|XP_001861867.1|	tetratricopeptide repeat domain 21B [Culex quinquefasciatus] gb|EDS36206.1| tetratricopeptide repeat domain 21B [Culex quinquefasciatus]	2,00E-57
gALL_v3_c24024	2	5	gnl|CDD|190884	pfam04146, YTH, YT521-B-like	ref|XP_002609689.1|	hypothetical protein BRAFLDRAFT_123593 [Branchiostoma floridae] gb|EEN65699.1| hypothetical protein BRAFLDRAFT_123593 [Branchiostoma floridae]	1,00E-31
gALL_v3_c24025	2	1	-	-	-	-	-
gALL_v3_c24027	1	3	-	-	-	-	-
gALL_v3_c24028	3	4	-	-	-	-	-
gALL_v3_c24029	1	7	-	-	-	-	-

gALL_v3_c24030	2	2	-	-	gb|EGI70331.1|	Protein chiffon [Acromyrmex echinatior]	3,00E-15
gALL_v3_c24032	3	4	-	-	gb|EHJ77925.1|	50kDa lectin [Danaus plexippus]	5,00E-06
gALL_v3_c24033	2	2	-	-	-	-	-
gALL_v3_c24036	2	2	-	-	-	-	-
gALL_v3_c24037	3	2	-	-	ref|XP_004576595.1|	PREDICTED: zinc finger protein 880-like, partial [Maylandia zebra]	5,00E-23
gALL_v3_c24038	1	6	-	-	-	-	-
gALL_v3_c24039	5	5	-	-	gb|AAR84629.1|	putative alcohol dehydrogenase [Gryllotalpa orientalis]	4,00E-16
gALL_v3_c2404	4	4	gnl|CDD|31397	COG1204, COG1204, Superfan	ref|XP_003702521.1|	PREDICTED: putative U5 small nuclear ribonucleoprotein 200 kDa helicase-like [Megachile rotundata]	4,00E-89
gALL_v3_c24040	3	3	-	-	-	-	-
gALL_v3_c24041	0	3	-	-	-	-	-
gALL_v3_c24042	1	2	-	-	-	-	-
gALL_v3_c24043	2	3	-	-	-	-	-
gALL_v3_c24045	0	4	-	-	ref|XP_006567828.1|	PREDICTED: protein unc-79 homolog [Apis mellifera]	2,00E-73
gALL_v3_c24046	3	1	-	-	-	-	-
gALL_v3_c24048	2	2	-	-	-	-	-
gALL_v3_c24049	2	5	-	-	-	-	-
gALL_v3_c2405	8	18	-	-	ref|XP_975168.1|	PREDICTED: similar to Toll-interacting protein [Tribolium castaneum] gb|EFA01564.1| hypothetical protein TcasGA2_TC007125 [Tribolium castaneum]	2,00E-23
gALL_v3_c24050	1	3	-	-	gb|EFA06477.1|	hypothetical protein TcasGA2_TC009369 [Tribolium castaneum]	2,00E-49
gALL_v3_c24053	2	2	-	-	-	-	-
gALL_v3_c24054	2	4	-	-	gb|EFN67634.1|	Putative uncharacterized transposon-derived protein F52C9.6 [Camponotus floridanus]	5,00E-14
gALL_v3_c24055	3	5	gnl|CDD|63872	cd04403, RhoGAP_ARHGAP27	ref|XP_001944075.2|	PREDICTED: rho GTPase-activating protein 12-like [Acyrthosiphon pisum]	2,00E-93
gALL_v3_c24056	1	5	-	-	gb|EFZ12390.1|	hypothetical protein SINV_13354 [Solenopsis invicta]	3,00E-10
gALL_v3_c24057	5	3	-	-	-	-	-
gALL_v3_c24058	2	2	gnl|CDD|162122	TIGR00939, 2a57, Equilibrative	ref|XP_003397842.1|	PREDICTED: equilibrative nucleoside transporter 4-like [Bombus terrestris]	1,00E-55
gALL_v3_c24060	0	3	-	-	-	-	-
gALL_v3_c24061	0	4	-	-	ref|XP_006565802.1|	PREDICTED: uncharacterized protein LOC727150 isoform X1 [Apis mellifera]	1,00E-10
gALL_v3_c24062	2	3	-	-	-	-	-
gALL_v3_c24063	1	2	-	-	-	-	-
gALL_v3_c24064	2	3	-	-	-	-	-
gALL_v3_c24065	2	3	-	-	-	-	-
gALL_v3_c24066	1	3	-	-	-	-	-
gALL_v3_c24067	1	3	-	-	-	-	-
gALL_v3_c24068	0	4	-	-	-	-	-
gALL_v3_c2407	8	14	gnl|CDD|132995	cd00218, GlcAT-I, Beta1,3-gluc	ref|XP_001946469.1|	PREDICTED: galactosylgalactosylxylosylprotein 3-beta-glucuronosyltransferase I-like [Acyrthosiphon pisum]	1,00E-112
gALL_v3_c24070	1	3	-	-	-	-	-
gALL_v3_c24071	3	2	-	-	-	-	-
gALL_v3_c24072	1	8	-	-	-	-	-
gALL_v3_c24073	2	2	-	-	-	-	-
gALL_v3_c24074	2	3	-	-	-	-	-
gALL_v3_c24075	3	1	-	-	-	-	-
gALL_v3_c24076	2	3	-	-	-	-	-
gALL_v3_c24077	1	3	-	-	-	-	-
gALL_v3_c24078	3	1	-	-	-	-	-
gALL_v3_c24079	1	5	-	-	-	-	-
gALL_v3_c2408	6	13	-	-	-	-	-
gALL_v3_c24080	1	3	-	-	gb|ERL95097.1|	hypothetical protein D910_12367 [Dendroctonus ponderosae]	7,00E-16
gALL_v3_c24084	2	1	-	-	ref|XP_002426073.1|	exocyst complex component, putative [Pediculus humanus corporis] gb|EEB13335.1| exocyst complex component, putative [Pediculus humanus corporis]	1,00E-32
gALL_v3_c24085	14	23	-	-	gb|AGM32427.1|	soldier-caste specific protein [Coptotermes formosanus]	4,00E-58
gALL_v3_c24086	1	4	-	-	-	-	-
gALL_v3_c24087	9	16	-	-	ref|WP_017208447.1|	hypothetical protein [Rickettsia australis]	2,00E-07
gALL_v3_c24088	1	3	-	-	-	-	-
gALL_v3_c24089	1	2	-	-	gb|EGI70978.1|	Gamma-tubulin complex component 5 [Acromyrmex echinatior]	4,00E-15
gALL_v3_c2409	7	17	gnl|CDD|190053	pfam01608, I_LWEQ, I/LWEQ c	ref|XP_003493227.1|	PREDICTED: talin-2-like [Bombus impatiens]	1,00E-163
gALL_v3_c24090	3	1	-	-	-	-	-
gALL_v3_c24091	1	4	-	-	gb|EGI61494.1|	hypothetical protein G5I_10269 [Acromyrmex echinatior]	8,00E-36
gALL_v3_c24092	0	4	-	-	-	-	-
gALL_v3_c24093	0	4	-	-	-	-	-
gALL_v3_c24094	3	4	-	-	gb|EFA05026.1|	hypothetical protein TcasGA2_TC015113 [Tribolium castaneum]	5,00E-59
gALL_v3_c24095	2	3	-	-	gb|EFN75583.1|	Arsenite-resistance protein 2-like protein [Harpegnathos saltator]	1,00E-11
gALL_v3_c24096	1	2	-	-	-	-	-
gALL_v3_c24097	1	5	-	-	-	-	-
gALL_v3_c24098	0	3	-	-	-	-	-
gALL_v3_c24099	2	3	-	-	ref|XP_006618297.1|	PREDICTED: LOW QUALITY PROTEIN: twitchin-like [Apis dorsata]	7,00E-24
gALL_v3_c241	12	9	-	-	-	-	-
gALL_v3_c2410	8	12	gnl|CDD|192103	pfam08626, TRAPPC9-Trs120,	ref|XP_003704315.1|	PREDICTED: protein brunelleschi-like [Megachile rotundata]	1,00E-173
gALL_v3_c24100	1	4	gnl|CDD|128524	smart00228, PDZ, Domain pre	ref|XP_002429742.1|	conserved hypothetical protein [Pediculus humanus corporis] gb|EEB17004.1| conserved hypothetical protein [Pediculus humanus corporis]	8,00E-80
gALL_v3_c24101	3	6	-	-	-	-	-
gALL_v3_c24102	1	3	-	-	-	-	-
gALL_v3_c24103	2	3	-	-	ref|XP_002428042.1|	WD-repeat protein, putative [Pediculus humanus corporis] gb|EEB15304.1| WD-repeat protein, putative [Pediculus humanus corporis]	7,00E-53
gALL_v3_c24104	1	2	-	-	gb|EEZ97820.1|	hypothetical protein TcasGA2_TC000187 [Tribolium castaneum]	2,00E-66
gALL_v3_c24105	2	2	-	-	-	-	-
gALL_v3_c24107	1	4	gnl|CDD|189008	cd09601, M1_APN_2, Peptida	gb|EFN65601.1|	Aminopeptidase N [Camponotus floridanus]	5,00E-73
gALL_v3_c24108	2	3	-	-	-	-	-
gALL_v3_c24109	8	8	-	-	gb|AGM32131.1|	40S ribosomal protein S3a [Coptotermes formosanus]	3,00E-36
gALL_v3_c2411	6	7	-	-	-	-	-
gALL_v3_c24110	1	4	-	-	-	-	-
gALL_v3_c24111	3	1	-	-	-	-	-
gALL_v3_c24113	1	3	gnl|CDD|29838	cd00871, PI4Ka, Phosphoinosi	ref|XP_969785.1|	PREDICTED: similar to AGAP010801-PA [Tribolium castaneum]	4,00E-59
gALL_v3_c24114	0	4	-	-	-	-	-
gALL_v3_c24115	1	3	-	-	ref|XP_002426682.1|	UDP-GalNAc:polypeptide N-acetylgalactosaminyltransferase, putative [Pediculus humanus corporis] gb|EEB13944.1| UDP-GalNAc:polypeptide N-acetylgalactosaminyltrans	7,00E-20
gALL_v3_c24116	2	3	-	-	-	-	-
gALL_v3_c24117	2	2	-	-	ref|XP_002426118.1|	DNA damage-binding protein, putative [Pediculus humanus corporis] gb|EEB13380.1| DNA damage-binding protein, putative [Pediculus humanus corporis]	3,00E-08
gALL_v3_c24118	2	3	-	-	ref|XP_006610888.1|	PREDICTED: ras-associated and pleckstrin homology domains-containing protein 1-like, partial [Apis dorsata]	3,00E-15
gALL_v3_c24119	1	2	-	-	-	-	-
gALL_v3_c2412	7	5	-	-	-	-	-
gALL_v3_c24120	1	3	-	-	-	-	-
gALL_v3_c24121	7	6	-	-	gb|AGM32427.1|	soldier-caste specific protein [Coptotermes formosanus]	3,00E-69
gALL_v3_c24122	0	5	-	-	-	-	-
gALL_v3_c24124	0	3	-	-	-	-	-
gALL_v3_c24125	1	6	-	-	ref|XP_002430718.1|	conserved hypothetical protein [Pediculus humanus corporis] gb|EEB17980.1| conserved hypothetical protein [Pediculus humanus corporis]	2,00E-61
gALL_v3_c24126	3	1	-	-	-	-	-
gALL_v3_c24127	3	5	-	-	ref|XP_003486620.1|	PREDICTED: hypothetical protein LOC100746843 isoform 2 [Bombus impatiens]	7,00E-09
gALL_v3_c24128	4	2	-	-	-	-	-
gALL_v3_c24129	5	3	gnl|CDD|190466	pfam02889, Sec63, Sec63 Brl c	ref|XP_002429968.1|	U520, putative [Pediculus humanus corporis] gb|EEB17230.1| U520, putative [Pediculus humanus corporis]	0
gALL_v3_c24132	6	4	-	-	ref|XP_966592.1|	PREDICTED: similar to CG8034 CG8034-PA isoform 1 [Tribolium castaneum] gb|EFA02707.1| hypothetical protein TcasGA2_TC008430 [Tribolium castaneum]	5,00E-28
gALL_v3_c24133	1	3	-	-	gb|EGI65127.1|	Niemann-Pick C1 protein [Acromyrmex echinatior]	1,00E-11
gALL_v3_c24134	1	4	-	-	gb|ERL85599.1|	hypothetical protein D910_03018 [Dendroctonus ponderosae]	1,00E-24
gALL_v3_c24135	3	3	-	-	-	-	-
gALL_v3_c24136	2	2	-	-	-	-	-
gALL_v3_c24137	3	3	-	-	-	-	-
gALL_v3_c24138	3	1	-	-	-	-	-
gALL_v3_c24139	7	6	-	-	gb|ADI61823.1|	endonuclease-reverse transcriptase [Bombyx mori]	2,00E-15
gALL_v3_c2414	20	26	gnl|CDD|29261	cd00204, ANK, ankyrin repeat:	gb|EFZ16882.1|	hypothetical protein SINV_07109 [Solenopsis invicta]	9,00E-61
gALL_v3_c24140	3	2	-	-	-	-	-
gALL_v3_c24141	2	3	-	-	-	-	-
gALL_v3_c24143	2	2	-	-	ref|XP_001602115.2|	PREDICTED: hypothetical protein LOC100118032 [Nasonia vitripennis]	4,00E-14
gALL_v3_c24144	0	4	-	-	-	-	-
gALL_v3_c24146	3	4	-	-	-	-	-
gALL_v3_c24147	2	7	-	-	gb|EFN89848.1|	Integrin alpha-PS4 [Harpegnathos saltator]	9,00E-16
gALL_v3_c24148	2	1	-	-	-	-	-
gALL_v3_c2415	10	8	gnl|CDD|178167	PLN02552, PLN02552, isopent	gb|ETN62790.1|	isopentenyl-diphosphate delta isomerase [Anopheles darlingi]	2,00E-67
gALL_v3_c24150	6	4	-	-	gb|EFZ14377.1|	hypothetical protein SINV_11688 [Solenopsis invicta]	3,00E-18
gALL_v3_c24151	8	13	-	-	-	-	-
gALL_v3_c24152	2	3	-	-	-	-	-
gALL_v3_c24153	3	7	-	-	-	-	-
gALL_v3_c24154	1	5	-	-	-	-	-
gALL_v3_c24156	2	2	-	-	-	-	-
gALL_v3_c24157	2	5	-	-	gb|EFN66432.1|	Retrovirus-related Pol polyprotein from transposon 412 [Camponotus floridanus]	7,00E-11
gALL_v3_c24158	2	3	-	-	ref|XP_002432716.1|	Cyclin-G2, putative [Pediculus humanus corporis] gb|EEB19978.1| Cyclin-G2, putative [Pediculus humanus corporis]	1,00E-45
gALL_v3_c24159	0	5	-	-	-	-	-
gALL_v3_c2416	21	15	gnl|CDD|189335	pfam00002, 7tm_2, 7 transme	ref|XP_006571413.1|	PREDICTED: latrophilin Cirl-like isoform X5 [Apis mellifera]	1,00E-170
gALL_v3_c24160	3	3	-	-	-	-	-
gALL_v3_c24161	1	3	-	-	-	-	-
gALL_v3_c24163	2	2	-	-	-	-	-
gALL_v3_c24164	2	3	-	-	-	-	-
gALL_v3_c24166	1	5	-	-	gb|EFA13593.1|	hypothetical protein TcasGA2_TC001480 [Tribolium castaneum]	2,00E-23
gALL_v3_c24167	2	4	-	-	-	-	-
gALL_v3_c24168	1	4	-	-	-	-	-
gALL_v3_c24169	3	2	gnl|CDD|177628	PHA03392, egt, ecdysteroid U	gb|AGC84403.1|	glucosyl glucuronosyl transferases [Locusta migratoria]	2,00E-51
gALL_v3_c2417	6	6	-	-	ref|XP_003704340.1|	PREDICTED: peroxisomal targeting signal 1 receptor-like [Megachile rotundata]	8,00E-21
gALL_v3_c24170	13	17	gnl|CDD|185501	PTZ00178, PTZ00178, 60S ribo	sp|A8CAG3.1|RL17_PHL	RecName: Full=60S ribosomal protein L17 gb|ABV44752.1| 60S ribosomal protein L17-like protein [Phlebotomus papatasi]	2,00E-55
gALL_v3_c24171	2	2	gnl|CDD|29233	cd01460, vWA_midasin, VWA	ref|XP_972555.2|	PREDICTED: similar to CG13185 CG13185-PB [Tribolium castaneum]	4,00E-27
gALL_v3_c24172	2	2	-	-	-	-	-
gALL_v3_c24173	0	5	-	-	-	-	-
gALL_v3_c24174	2	4	-	-	-	-	-
gALL_v3_c24175	1	2	-	-	-	-	-
gALL_v3_c24176	0	4	-	-	-	-	-
gALL_v3_c24177	2	2	-	-	-	-	-
gALL_v3_c24179	2	2	-	-	-	-	-
gALL_v3_c24181	1	2	-	-	-	-	-
gALL_v3_c24182	3	1	-	-	-	-	-
gALL_v3_c24183	3	1	-	-	-	-	-
gALL_v3_c24184	3	2	gnl|CDD|34966	COG5407, SEC63, Preprotein t	gb|EFZ16351.1|	hypothetical protein SINV_08840 [Solenopsis invicta]	8,00E-79
gALL_v3_c24185	2	3	-	-	-	-	-

gALL_v3_c24186	4	4	-	-	-	-	-	
gALL_v3_c24187	2	1	gnl|CDD|189605	pfam00566, TBC, TBC domain.	gb|EFZ18742.1|	hypothetical protein SINV_07021 [Solenopsis invicta]		9,00E-93
gALL_v3_c24188	0	5	-	-	-	-	-	
gALL_v3_c24189	2	2	-	-	-	-	-	
gALL_v3_c2419	26	47	-	-	-	-	-	
gALL_v3_c24190	2	2	-	-	-	-	-	
gALL_v3_c24191	1	4	-	-	-	-	-	
gALL_v3_c24192	1	3	-	-	-	-	-	
gALL_v3_c24194	2	1	-	-	-	-	-	
gALL_v3_c24195	1	5	gnl|CDD|109297	pfam00233, PDEase_I, 3'5'-cyc	gb|EHJ78348.1|	hypothetical protein KGM_12836 [Danaus plexippus]		3,00E-90
gALL_v3_c24197	4	2	-	-	gb|EEZ99906.1|	hypothetical protein TcasGA2_TC002697 [Tribolium castaneum]		5,00E-60
gALL_v3_c24198	2	3	-	-	-	-	-	
gALL_v3_c24199	0	3	-	-	-	-	-	
gALL_v3_c242	21	21	gnl|CDD|193472	pfam12999, PRKCSH-like, Gluc	ref|XP_002428448.1|	glucosidase 2 subunit beta precursor, putative [Pediculus humanus corporis] gb|EEB15710.1| glucosidase 2 subunit beta precursor, putative [Pediculus humanus corporis]		1,00E-173
gALL_v3_c2420	1	5	-	-	-	-	-	
gALL_v3_c24200	1	3	-	-	-	-	-	
gALL_v3_c24201	2	2	gnl|CDD|153344	cd07660, BAR_Arfaptin, The B	gb|EGI67644.1|	Arfaptin-2 [Acromyrmex echinatior]		1,00E-80
gALL_v3_c24202	0	4	-	-	-	-	-	
gALL_v3_c24204	2	4	-	-	gb|EFN65855.1|	60S ribosomal protein L7 [Camponotus floridanus]		2,00E-21
gALL_v3_c24205	0	4	-	-	-	-	-	
gALL_v3_c24206	2	2	-	-	-	-	-	
gALL_v3_c24207	2	4	-	-	-	-	-	
gALL_v3_c24208	2	3	-	-	-	-	-	
gALL_v3_c24209	3	2	-	-	gb|ENN74891.1|	hypothetical protein YQE_08470, partial [Dendroctonus ponderosae]		2,00E-25
gALL_v3_c24210	1	2	-	-	-	-	-	
gALL_v3_c24211	1	3	-	-	-	-	-	
gALL_v3_c24212	5	4	-	-	-	-	-	
gALL_v3_c24213	1	8	-	-	-	-	-	
gALL_v3_c24214	4	5	gnl|CDD|190234	pfam02177, APP_N, Amyloid A	ref|XP_002426949.1|	conserved hypothetical protein [Pediculus humanus corporis] gb|EEB14211.1| conserved hypothetical protein [Pediculus humanus corporis]		4,00E-30
gALL_v3_c24215	3	1	-	-	-	-	-	
gALL_v3_c24216	1	3	-	-	gb|EFN65619.1|	Putative uncharacterized transposon-derived protein F52C9.6 [Camponotus floridanus]		5,00E-30
gALL_v3_c24217	4	3	gnl|CDD|178359	PLN02759, PLN02759, Format	ref|XP_005183705.1|	PREDICTED: c-1-tetrahydrofolate synthase, cytoplasmic-like isoform X1 [Musca domestica]		2,00E-87
gALL_v3_c24218	3	1	-	-	-	-	-	
gALL_v3_c24219	2	2	-	-	-	-	-	
gALL_v3_c2422	21	13	gnl|CDD|161951	TIGR00601, rad23, UV excision	ref|XP_002432231.1|	uv excision repair protein rad23, putative [Pediculus humanus corporis] gb|EEB19493.1| uv excision repair protein rad23, putative [Pediculus humanus corporis]		6,00E-95
gALL_v3_c24220	4	4	-	-	-	-	-	
gALL_v3_c24222	6	6	gnl|CDD|30378	COG0028, IlvB, Thiamine pyro	ref|XP_005095679.1|	PREDICTED: acetolactate synthase-like protein-like [Aplysia californica]		3,00E-71
gALL_v3_c24224	1	3	-	-	-	-	-	
gALL_v3_c24225	1	5	-	-	-	-	-	
gALL_v3_c24226	1	3	-	-	-	-	-	
gALL_v3_c24227	1	3	gnl|CDD|73156	cd01650, RT_nLTR_like, RT_nL	ref|XP_004213283.1|	PREDICTED: RNA-directed DNA polymerase from mobile element jockey-like, partial [Hydra vulgaris]		1,00E-31
gALL_v3_c24228	1	7	gnl|CDD|146595	pfam04048, Sec8_exocyst, Sec	ref|XP_001608195.1|	PREDICTED: exocyst complex component 4-like [Nasonia vitripennis]		1,00E-88
gALL_v3_c24229	2	2	-	-	-	-	-	
gALL_v3_c2423	17	11	gnl|CDD|100062	cd05824, LbH_M1P_guanylylT	ref|XP_002102150.1|	GD19750 [Drosophila simulans] gb|EDX11653.1| GD19750 [Drosophila simulans]		1,00E-40
gALL_v3_c24230	2	3	gnl|CDD|173753	cd07864, STKc_CDK12, Catalyt	gb|EFA09617.1|	hypothetical protein TcasGA2_TC011738 [Tribolium castaneum]		4,00E-89
gALL_v3_c24231	2	2	-	-	-	-	-	
gALL_v3_c24232	5	2	-	-	-	-	-	
gALL_v3_c24233	3	1	-	-	-	-	-	
gALL_v3_c24234	0	3	-	-	-	-	-	
gALL_v3_c24236	2	2	-	-	-	-	-	
gALL_v3_c24237	4	2	-	-	-	-	-	
gALL_v3_c24238	7	5	-	-	-	-	-	
gALL_v3_c24239	2	3	-	-	-	-	-	
gALL_v3_c2424	17	14	gnl|CDD|192374	pfam09770, PAT1, Topoisomer	ref|XP_002432701.1|	hypothetical protein Phum_PHUM597500 [Pediculus humanus corporis] gb|EEB19963.1| hypothetical protein Phum_PHUM597500 [Pediculus humanus corporis]		1,00E-39
gALL_v3_c24240	1	3	-	-	-	-	-	
gALL_v3_c24241	2	1	-	-	-	-	-	
gALL_v3_c24243	3	2	-	-	-	-	-	
gALL_v3_c24245	1	5	gnl|CDD|148495	pfam06905, FAIM1, Fas apopto	ref|XP_002426092.1|	Fas apoptotic inhibitory molecule, putative [Pediculus humanus corporis] gb|EEB13354.1| Fas apoptotic inhibitory molecule, putative [Pediculus humanus corporis]		1,00E-71
gALL_v3_c24246	4	2	-	-	-	-	-	
gALL_v3_c24248	16	18	-	-	-	-	-	
gALL_v3_c2425	7	10	gnl|CDD|190452	pfam02854, MIF4G, MIF4G do	ref|XP_003396125.1|	PREDICTED: eukaryotic translation initiation factor 4 gamma 2-like [Bombus terrestris]		1,00E-149
gALL_v3_c24251	2	1	-	-	-	-	-	
gALL_v3_c24252	2	2	-	-	-	-	-	
gALL_v3_c24254	4	8	-	-	-	-	-	
gALL_v3_c24256	2	2	-	-	-	-	-	
gALL_v3_c24257	0	4	-	-	-	-	-	
gALL_v3_c24258	2	4	-	-	-	-	-	
gALL_v3_c24259	5	3	-	-	-	-	-	
gALL_v3_c2426	5	2	gnl|CDD|131231	TIGR02176, pyruv_ox_red, pyr	gb|AAV74342.1|	pyruvate:ferredoxin oxidoreductase A-like protein [Trichomonas vaginalis]		2,00E-70
gALL_v3_c24260	1	5	-	-	-	-	-	
gALL_v3_c24261	0	4	-	-	ref|XP_002422898.1|	conserved hypothetical protein [Pediculus humanus corporis] gb|EEB10160.1| conserved hypothetical protein [Pediculus humanus corporis]		2,00E-14
gALL_v3_c24262	2	1	gnl|CDD|146001	pfam03159, XRN_N, XRN 5'-3'	ref|XP_002120271.2|	PREDICTED: 5'-3' exoribonuclease 1 [Ciona intestinalis]		9,00E-30
gALL_v3_c24263	1	2	-	-	-	-	-	
gALL_v3_c24264	3	2	gnl|CDD|187704	cd09280, RNase_HI_eukaryote	ref|XP_002609599.1|	hypothetical protein BRAFLDRAFT_125021 [Branchiostoma floridae] gb|EEN65609.1| hypothetical protein BRAFLDRAFT_125021 [Branchiostoma floridae]		6,00E-36
gALL_v3_c24265	3	1	-	-	-	-	-	
gALL_v3_c24266	4	5	gnl|CDD|34495	COG4886, COG4886, Leucine-r	ref|XP_002424562.1|	tartan protein, putative [Pediculus humanus corporis] gb|EEB11824.1| tartan protein, putative [Pediculus humanus corporis]		1,00E-156
gALL_v3_c24267	2	2	-	-	ref|XP_003701139.1|	PREDICTED: autophagy-related protein 9A-like [Megachile rotundata]		1,00E-25
gALL_v3_c24268	6	5	-	-	-	-	-	
gALL_v3_c24269	2	3	-	-	ref|XP_006618694.1|	PREDICTED: protein furry-like isoform X1 [Apis dorsata]		1,00E-89
gALL_v3_c2427	15	20	-	-	ref|XP_973041.2|	PREDICTED: hypothetical protein [Tribolium castaneum]		2,00E-09
gALL_v3_c24270	3	1	-	-	-	-	-	
gALL_v3_c24271	0	3	-	-	gb|EFA08913.1|	hypothetical protein TcasGA2_TC006618 [Tribolium castaneum]		5,00E-11
gALL_v3_c24272	1	3	-	-	-	-	-	
gALL_v3_c24275	1	3	-	-	-	-	-	
gALL_v3_c24276	2	7	gnl|CDD|189605	pfam00566, TBC, TBC domain.	ref|XP_003696260.1|	PREDICTED: growth hormone-regulated TBC protein 1-A-like [Apis florea]		1,00E-47
gALL_v3_c24277	3	3	-	-	-	-	-	
gALL_v3_c24279	2	4	gnl|CDD|72875	cd04513, Glycosylasparaginase	gb|EHJ64658.1|	aspartylglucosaminidase [Danaus plexippus]		3,00E-72
gALL_v3_c2428	14	19	-	-	-	-	-	
gALL_v3_c24280	0	6	-	-	-	-	-	
gALL_v3_c24281	1	2	-	-	-	-	-	
gALL_v3_c24282	0	5	-	-	-	-	-	
gALL_v3_c24283	0	3	-	-	-	-	-	
gALL_v3_c24284	2	3	-	-	-	-	-	
gALL_v3_c24285	1	3	-	-	ref|XP_002413197.1|	hypothetical protein IscW_ISCW012360 [Ixodes scapularis] gb|EEC16505.1| hypothetical protein IscW_ISCW012360 [Ixodes scapularis]		3,00E-33
gALL_v3_c24286	0	3	gnl|CDD|176471	cd03302, Adenylsuccinate_lya	ref|XP_003742661.1|	PREDICTED: adenylosuccinate lyase [Metaseiulus occidentalis]		9,00E-40
gALL_v3_c24287	1	3	gnl|CDD|128557	smart00261, FU, Furin-like rep	ref|XP_001662438.1|	hypothetical protein AaeL_AAEL002793 [Aedes aegypti] gb|EAT45948.1| AAEL002793-PA [Aedes aegypti]		1,00E-89
gALL_v3_c24289	3	1	-	-	-	-	-	
gALL_v3_c2429	12	12	gnl|CDD|192486	pfam10224, DUF2205, Predict	dbj|BAN20512.1|	conserved hypothetical protein [Riptortus pedestris]		2,00E-74
gALL_v3_c24290	0	4	-	-	-	-	-	
gALL_v3_c24291	1	4	-	-	-	-	-	
gALL_v3_c24292	1	3	gnl|CDD|189424	pfam00153, Mito_carr, Mitoch	ref|XP_002074979.1|	GK22863 [Drosophila willistoni] gb|EDW85965.1| GK22863 [Drosophila willistoni]		3,00E-39
gALL_v3_c24293	1	4	-	-	-	-	-	
gALL_v3_c24295	3	2	-	-	-	-	-	
gALL_v3_c24296	5	3	-	-	-	-	-	
gALL_v3_c24297	5	4	gnl|CDD|144508	pfam00931, NB-ARC, NB-ARC	gb|EFX74325.1|	hypothetical protein DAPPUDRAFT_226784 [Daphnia pulex]		2,00E-26
gALL_v3_c24299	2	4	gnl|CDD|143945	pfam00179, UQ_con, Ubiquitin	ref|XP_002432375.1|	NEDD8-conjugating enzyme Ubc12, putative [Pediculus humanus corporis] gb|EEB19637.1| NEDD8-conjugating enzyme Ubc12, putative [Pediculus humanus corporis]		1,00E-121
gALL_v3_c2430	9	13	-	-	gb|AGM32845.1|	alpha-endosulfine-like protein [Coptotermes formosanus]		4,00E-59
gALL_v3_c24301	6	14	-	-	-	-	-	
gALL_v3_c24302	2	3	gnl|CDD|28920	cd00038, CAP_ED, effector do	ref|XP_966775.1|	PREDICTED: similar to hyperpolarization-activated ion channel isoform 1 [Tribolium castaneum]		2,00E-89
gALL_v3_c24303	2	2	-	-	gb|EFA11400.1|	hypothetical protein TcasGA2_TC013573 [Tribolium castaneum]		1,00E-36
gALL_v3_c24304	0	5	-	-	-	-	-	
gALL_v3_c24306	0	4	-	-	-	-	-	
gALL_v3_c24308	2	2	-	-	-	-	-	
gALL_v3_c24309	1	4	gnl|CDD|143860	pfam00078, RVT_1, Reverse tr	ref|XP_003742488.1|	PREDICTED: RNA-directed DNA polymerase from mobile element jockey-like [Metaseiulus occidentalis]		1,00E-19
gALL_v3_c24310	4	4	-	-	ref|XP_002429661.1|	krueppel c2h2-type zinc finger protein, putative [Pediculus humanus corporis] gb|EEB16923.1| krueppel c2h2-type zinc finger protein, putative [Pediculus humanus corpor		4,00E-39
gALL_v3_c24311	2	4	-	-	ref|XP_003244610.1|	PREDICTED: slit homolog 1 protein-like [Acyrthosiphon pisum]		2,00E-13
gALL_v3_c24312	2	3	-	-	-	-	-	
gALL_v3_c24313	5	5	gnl|CDD|48404	cd02429, PTH2_like, Peptidyl-t	ref|XP_972516.2|	PREDICTED: similar to CG14903 CG14903-PA [Tribolium castaneum]		3,00E-51
gALL_v3_c24315	2	1	-	-	-	-	-	
gALL_v3_c24316	2	5	-	-	-	-	-	
gALL_v3_c24318	3	1	-	-	-	-	-	
gALL_v3_c2432	5	4	gnl|CDD|190555	pfam03184, DDE_1, DDE supe	ref|XP_006002184.1|	PREDICTED: tigger transposable element-derived protein 4-like [Latimeria chalumnae]		1,00E-36
gALL_v3_c24320	3	1	-	-	-	-	-	
gALL_v3_c24321	0	4	-	-	-	-	-	
gALL_v3_c24323	2	2	-	-	-	-	-	
gALL_v3_c24324	14	13	-	-	-	-	-	
gALL_v3_c24325	2	3	gnl|CDD|28928	cd00268, DEADc, DEAD-box he	gb|EFN61290.1|	Probable ATP-dependent RNA helicase DDX49 [Camponotus floridanus]		9,00E-92
gALL_v3_c24326	1	3	-	-	-	-	-	
gALL_v3_c24327	21	23	-	-	-	-	-	
gALL_v3_c24328	1	2	-	-	-	-	-	
gALL_v3_c2433	4	10	-	-	gb|EFA01910.1|	hypothetical protein TcasGA2_TC007520 [Tribolium castaneum]		8,00E-22
gALL_v3_c24330	2	2	-	-	-	-	-	
gALL_v3_c24331	3	3	-	-	-	-	-	
gALL_v3_c24332	2	4	-	-	ref|XP_003398133.1|	PREDICTED: beta-1,3-galactosyltransferase brn-like [Bombus terrestris]		1,00E-54
gALL_v3_c24333	0	4	-	-	-	-	-	
gALL_v3_c24335	3	1	-	-	-	-	-	
gALL_v3_c24336	4	7	-	-	-	-	-	
gALL_v3_c24337	2	3	-	-	-	-	-	
gALL_v3_c24339	1	5	-	-	ref|XP_002434404.1|	ubiquilin 1,2, putative [Ixodes scapularis] gb|EEC07228.1| ubiquilin 1,2, putative [Ixodes scapularis]		2,00E-27
gALL_v3_c2434	15	15	-	-	-	-	-	
gALL_v3_c24340	4	2	-	-	-	-	-	
gALL_v3_c24342	2	1	-	-	-	-	-	
gALL_v3_c24343	4	6	-	-	ref|XP_308919.3|	AGAP006825-PA [Anopheles gambiae str. PEST] gb|EAA04234.4| AGAP006825-PA [Anopheles gambiae str. PEST]		1,00E-116
gALL_v3_c24344	2	3	gnl|CDD|144522	pfam00955, HCO3_cotransp, H	ref|XP_003484711.1|	PREDICTED: anion exchange protein 2-like [Bombus impatiens]		7,00E-66

gALL_v3_c24345	2	2	-	-	-	-	-
gALL_v3_c24346	3	1	-	-	-	-	-
gALL_v3_c24347	1	4	-	-	-	-	-
gALL_v3_c24349	3	1	gnl|CDD|109787	pfam00743, FMO-like, Flavin-b	gb|EFZ18476.1|	hypothetical protein SINV_12134 [Solenopsis invicta]	4,00E-28
gALL_v3_c24350	1	5	-	-	-	-	-
gALL_v3_c24351	3	2	-	-	-	-	-
gALL_v3_c24353	2	2	-	-	-	-	-
gALL_v3_c24355	4	3	gnl|CDD|173561	PTZ00368, PTZ00368, universa	gb|AGM32414.1|	zinc finger protein [Coptotermes formosanus]	2,00E-26
gALL_v3_c24356	0	5	gnl|CDD|152704	pfam12269, zf-CpG_bind_C, C	ref|XP_002432996.1|	cpg binding protein, putative [Pediculus humanus corporis] gb|EEB20258.1| cpg binding protein, putative [Pediculus humanus corporis]	5,00E-52
gALL_v3_c24357	4	3	-	-	-	-	-
gALL_v3_c24358	2	2	gnl|CDD|181286	PRK08202, PRK08202, purine	gb|EFX76980.1|	hypothetical protein DAPPUDRAFT_306029 [Daphnia pulex]	7,00E-29
gALL_v3_c24359	0	3	-	-	-	-	-
gALL_v3_c2436	7	6	-	-	gb|ESO85828.1|	hypothetical protein LOTGIDRAFT_221280 [Lottia gigantea]	2,00E-08
gALL_v3_c24360	2	1	-	-	-	-	-
gALL_v3_c24361	3	8	-	-	dbj|BAN20910.1|	conserved hypothetical protein [Riptortus pedestris]	2,00E-22
gALL_v3_c24362	2	1	-	-	-	-	-
gALL_v3_c24363	1	6	-	-	-	-	-
gALL_v3_c24364	3	3	gnl|CDD|191810	pfam07679, I-set, Immunoglol	ref|XP_004927653.1|	PREDICTED: protein turtle-like [Bombyx mori]	1,00E-100
gALL_v3_c24366	3	1	-	-	gb|AGM32182.1|	hypothetical protein [Coptotermes formosanus]	8,00E-23
gALL_v3_c24367	1	3	-	-	gb|ERL85321.1|	hypothetical protein D910_02741, partial [Dendroctonus ponderosae]	2,00E-18
gALL_v3_c24368	0	3	-	-	-	-	-
gALL_v3_c24369	2	2	-	-	-	-	-
gALL_v3_c2437	8	8	gnl|CDD|128427	smart00119, HECTc, Domain H	gb|ETN64856.1|	ubiquitin-protein ligase [Anopheles darlingi]	2,00E-57
gALL_v3_c24370	1	3	-	-	-	-	-
gALL_v3_c24372	3	4	gnl|CDD|30021	cd02572, PseudoU_synth_hDy	ref|XP_002430157.1|	minifly protein, putative [Pediculus humanus corporis] gb|EEB17419.1| minifly protein, putative [Pediculus humanus corporis]	3,00E-88
gALL_v3_c24373	3	3	-	-	-	-	-
gALL_v3_c24374	2	1	-	-	-	-	-
gALL_v3_c24375	4	2	-	-	-	-	-
gALL_v3_c24376	0	3	-	-	-	-	-
gALL_v3_c24377	1	2	-	-	-	-	-
gALL_v3_c24378	1	3	-	-	-	-	-
gALL_v3_c24381	2	2	gnl|CDD|190340	pfam02535, Zip, ZIP Zinc trans	ref|XP_001603533.1|	PREDICTED: zinc transporter ZIP9-B-like [Nasonia vitripennis]	6,00E-36
gALL_v3_c24382	3	2	-	-	gb|EFN72328.1|	Histone-lysine N-methyltransferase SETMAR [Camponotus floridanus]	6,00E-29
gALL_v3_c24383	3	1	-	-	-	-	-
gALL_v3_c24385	2	2	-	-	-	-	-
gALL_v3_c24386	3	5	gnl|CDD|100083	cd06162, S2P-M50_PDZ_SREB	gb|EFZ23169.1|	hypothetical protein SINV_05718 [Solenopsis invicta]	2,00E-49
gALL_v3_c24387	3	1	-	-	-	-	-
gALL_v3_c24388	4	3	-	-	-	-	-
gALL_v3_c24389	1	2	-	-	-	-	-
gALL_v3_c2439	3	5	-	-	-	-	-
gALL_v3_c24391	2	2	-	-	-	-	-
gALL_v3_c24392	1	4	-	-	ref|XP_003396639.1|	PREDICTED: mitogen-activated protein kinase kinase kinase 13-like isoform 1 [Bombus terrestris] ref|XP_003487787.1| PREDICTED: mitogen-activated protein kinase kinas	3,00E-12
gALL_v3_c24393	2	1	-	-	ref|XP_002425877.1|	cutilin-1 precursor, putative [Pediculus humanus corporis] gb|EEB13139.1| cutilin-1 precursor, putative [Pediculus humanus corporis]	2,00E-24
gALL_v3_c24394	3	4	-	-	-	-	-
gALL_v3_c24396	0	4	-	-	-	-	-
gALL_v3_c24398	3	1	-	-	-	-	-
gALL_v3_c244	17	14	gnl|CDD|193173	pfam12697, Abhydrolase_6, A	ref|XP_001869650.1|	abhydrolase domain-containing protein 7 [Culex quinquefasciatus] gb|EDS29923.1| abhydrolase domain-containing protein 7 [Culex quinquefasciatus]	6,00E-62
gALL_v3_c2440	13	25	-	-	-	-	-
gALL_v3_c24400	2	2	gnl|CDD|148186	pfam06427, UDP-g_GGTase, U	gb|EFA03542.1|	hypothetical protein TcasGA2_TC013545 [Tribolium castaneum]	3,00E-72
gALL_v3_c24402	1	2	-	-	gb|EFX65788.1|	hypothetical protein DAPPUDRAFT_332854 [Daphnia pulex]	6,00E-15
gALL_v3_c24403	3	2	-	-	-	-	-
gALL_v3_c24404	4	2	-	-	-	-	-
gALL_v3_c24405	4	2	-	-	ref|XP_005180524.1|	PREDICTED: probable 28S ribosomal protein S26, mitochondrial-like [Musca domestica]	7,00E-32
gALL_v3_c24406	3	1	-	-	-	-	-
gALL_v3_c24407	3	1	-	-	-	-	-
gALL_v3_c24408	2	4	-	-	-	-	-
gALL_v3_c24409	1	4	-	-	-	-	-
gALL_v3_c2441	10	11	gnl|CDD|128405	smart00094, TR_FER, Transfer	ref|XP_003401471.1|	PREDICTED: transferrin-like [Bombus terrestris]	1,00E-48
gALL_v3_c24410	3	2	-	-	-	-	-
gALL_v3_c24411	2	1	-	-	-	-	-
gALL_v3_c24412	1	2	-	-	-	-	-
gALL_v3_c24413	3	6	-	-	-	-	-
gALL_v3_c24414	1	3	-	-	-	-	-
gALL_v3_c24415	2	3	-	-	-	-	-
gALL_v3_c24416	1	3	-	-	gb|AGM32527.1|	hemolymph juvenile hormone binding protein (JHBP) [Coptotermes formosanus]	5,00E-24
gALL_v3_c24418	2	4	-	-	-	-	-
gALL_v3_c24419	59	136	gnl|CDD|188788	cd09404, LIM1_MLP84B_like,	gb|AAV91410.1|	LIM protein 1 [Lonomia obliqua]	8,00E-43
gALL_v3_c2442	1	5	-	-	-	-	-
gALL_v3_c24420	1	3	-	-	gb|EFZ17617.1|	hypothetical protein SINV_06153 [Solenopsis invicta]	1,00E-11
gALL_v3_c24421	1	3	gnl|CDD|179368	PRK02106, PRK02106, choline	ref|XP_003247112.1|	PREDICTED: glucose dehydrogenase [acceptor]-like [Acyrthosiphon pisum]	4,00E-24
gALL_v3_c24422	3	1	-	-	-	-	-
gALL_v3_c24423	0	5	-	-	-	-	-
gALL_v3_c24424	1	3	gnl|CDD|31829	COG1643, HrpA, HrpA-like hel	ref|XP_004928390.1|	PREDICTED: putative ATP-dependent RNA helicase DHX57-like [Bombyx mori]	4,00E-26
gALL_v3_c24425	3	1	-	-	-	-	-
gALL_v3_c24428	2	3	-	-	-	-	-
gALL_v3_c24429	0	5	gnl|CDD|188657	cd09825, thyroid_peroxidase,	ref|XP_968570.1|	PREDICTED: similar to peroxidasin [Tribolium castaneum] gb|EFA01243.1| peroxidasin [Tribolium castaneum]	5,00E-88
gALL_v3_c2443	9	10	gnl|CDD|178917	PRK00179, pgi, glucose-6-pho:	gb|EFN82269.1|	Glucose-6-phosphate isomerase [Harpegnathos saltator]	1,00E-147
gALL_v3_c24430	7	9	-	-	-	-	-
gALL_v3_c24431	2	3	-	-	ref|XP_005307799.1|	PREDICTED: tigger transposable element-derived protein 1-like [Chrysemys picta bellii]	1,00E-13
gALL_v3_c24432	1	4	-	-	gb|EHJ74902.1|	hypothetical protein KGM_22644 [Danaus plexippus]	2,00E-63
gALL_v3_c24433	2	4	-	-	-	-	-
gALL_v3_c24434	2	2	gnl|CDD|193238	pfam12762, DDE_Tnp_IS1595,	gb|EFN89271.1|	hypothetical protein EAI_01097 [Harpegnathos saltator]	1,00E-15
gALL_v3_c24435	2	1	-	-	ref|XP_003701389.1|	PREDICTED: fumarylacetoacetate hydrolase domain-containing protein 2A-like isoform 2 [Megachile rotundata]	2,00E-08
gALL_v3_c24436	2	2	-	-	-	-	-
gALL_v3_c24438	0	4	-	-	-	-	-
gALL_v3_c24439	3	4	-	-	ref|XP_006616959.1|	PREDICTED: zinc finger protein 571-like isoform X2 [Apis dorsata]	2,00E-14
gALL_v3_c2444	8	14	-	-	-	-	-
gALL_v3_c24440	1	4	gnl|CDD|181802	PRK09367, PRK09367, histidin	gb|EFA07084.1|	hypothetical protein TcasGA2_TC010072 [Tribolium castaneum]	1,00E-104
gALL_v3_c24441	2	3	-	-	-	-	-
gALL_v3_c24442	3	3	-	-	-	-	-
gALL_v3_c24443	2	3	gnl|CDD|191810	pfam07679, I-set, Immunoglol	ref|XP_003486354.1|	PREDICTED: Down syndrome cell adhesion molecule-like protein CG42256-like [Bombus impatiens]	4,00E-40
gALL_v3_c24444	2	2	-	-	ref|XP_005178756.1|	PREDICTED: dentin sialophosphoprotein-like isoform X2 [Musca domestica]	2,00E-07
gALL_v3_c24445	3	3	-	-	-	-	-
gALL_v3_c24446	2	2	-	-	-	-	-
gALL_v3_c24447	2	1	-	-	-	-	-
gALL_v3_c24448	2	1	-	-	gb|EFN79725.1|	hypothetical protein EAI_16299 [Harpegnathos saltator]	4,00E-07
gALL_v3_c24449	1	5	-	-	-	-	-
gALL_v3_c2445	8	13	-	-	-	-	-
gALL_v3_c24450	0	4	-	-	-	-	-
gALL_v3_c24451	2	3	-	-	-	-	-
gALL_v3_c24452	1	3	gnl|CDD|148618	pfam07114, DUF1370, Protein	gb|ETN65187.1|	hypothetical protein AND_003054 [Anopheles darlingi]	1,00E-24
gALL_v3_c24454	2	3	-	-	-	-	-
gALL_v3_c24456	3	1	-	-	-	-	-
gALL_v3_c24457	4	4	-	-	gb|EFX86094.1|	hypothetical protein DAPPUDRAFT_313082 [Daphnia pulex]	5,00E-37
gALL_v3_c24459	3	1	-	-	-	-	-
gALL_v3_c2446	11	6	-	-	gb|ETN63984.1|	hypothetical protein AND_004288 [Anopheles darlingi]	3,00E-20
gALL_v3_c24460	2	4	-	-	gb|EFN65169.1|	Zinc finger CCCH domain-containing protein 3 [Camponotus floridanus]	2,00E-40
gALL_v3_c24461	2	1	-	-	-	-	-
gALL_v3_c24462	2	1	-	-	-	-	-
gALL_v3_c24463	3	1	-	-	-	-	-
gALL_v3_c24464	5	3	-	-	-	-	-
gALL_v3_c24465	2	1	-	-	-	-	-
gALL_v3_c24466	2	2	-	-	-	-	-
gALL_v3_c24467	2	3	gnl|CDD|148541	pfam06979, DUF1301, Protein	ref|XP_001848572.1|	transmembrane protein 70 [Culex quinquefasciatus] gb|EDS28615.1| transmembrane protein 70 [Culex quinquefasciatus]	1,00E-33
gALL_v3_c24468	1	6	-	-	gb|EFX80521.1|	hypothetical protein DAPPUDRAFT_304112 [Daphnia pulex]	8,00E-08
gALL_v3_c24469	2	2	-	-	-	-	-
gALL_v3_c24470	2	4	gnl|CDD|128856	smart00588, NEUZ, domain in	ref|XP_972157.1|	PREDICTED: similar to neuralized [Tribolium castaneum] gb|EEZ97846.1| hypothetical protein TcasGA2_TC000216 [Tribolium castaneum]	1,00E-110
gALL_v3_c24471	0	3	-	-	-	-	-
gALL_v3_c24472	3	1	-	-	-	-	-
gALL_v3_c24473	1	2	gnl|CDD|191315	pfam05608, DUF778, Protein	ref|XP_971702.2|	PREDICTED: similar to predicted protein [Tribolium castaneum]	8,00E-41
gALL_v3_c24474	3	2	-	-	-	-	-
gALL_v3_c24476	3	1	gnl|CDD|153344	cd07660, BAR_Arfaptin, The B	gb|EFZ19787.1|	hypothetical protein SINV_02162 [Solenopsis invicta]	2,00E-22
gALL_v3_c24477	2	5	-	-	-	-	-
gALL_v3_c24479	9	10	gnl|CDD|189635	pfam00621, RhoGEF, RhoGEF	ref|XP_002427478.1|	Rho/RAC guanine nucleotide exchange factor, putative [Pediculus humanus corporis] gb|EEB14740.1| Rho/RAC guanine nucleotide exchange factor, putative [Pediculus hu	6,00E-29
gALL_v3_c24480	4	2	-	-	-	-	-
gALL_v3_c24481	3	3	-	-	-	-	-
gALL_v3_c24482	5	3	-	-	-	-	-
gALL_v3_c24483	3	1	-	-	gb|AGM32845.1|	alpha-endosulfine-like protein [Coptotermes formosanus]	4,00E-33
gALL_v3_c24484	2	6	-	-	ref|XP_003242319.1|	PREDICTED: histone-lysine N-methyltransferase SETMAR-like [Acyrthosiphon pisum]	1,00E-70
gALL_v3_c24485	3	1	-	-	-	-	-
gALL_v3_c24486	2	1	-	-	-	-	-
gALL_v3_c24488	1	4	-	-	-	-	-
gALL_v3_c24489	2	3	-	-	-	-	-
gALL_v3_c2449	8	12	gnl|CDD|162093	TIGR00892, 2A0113, monocar	ref|XP_003707313.1|	PREDICTED: monocarboxylate transporter 3-like [Megachile rotundata]	0
gALL_v3_c24490	3	2	gnl|CDD|63873	cd04404, RhoGAP-p50rhoGAP	ref|XP_001950301.1|	PREDICTED: rho GTPase-activating protein 1-like [Acyrthosiphon pisum]	7,00E-13
gALL_v3_c24491	2	2	-	-	ref|XP_001663740.1|	hypothetical protein AaeL_AAEL013561 [Aedes aegypti] gb|EAT34174.1| AAEL013561-PA [Aedes aegypti]	1,00E-22
gALL_v3_c24493	0	4	-	-	ref|XP_002431379.1|	conserved hypothetical protein [Pediculus humanus corporis] gb|EEB18641.1| conserved hypothetical protein [Pediculus humanus corporis]	1,00E-41
gALL_v3_c24494	0	3	-	-	gb|AFH78148.1|	cytochrome P450 [Coptotermes formosanus]	3,00E-09
gALL_v3_c24495	2	5	-	-	gb|EFN80666.1|	E3 ubiquitin-protein ligase RNF123 [Harpegnathos saltator]	2,00E-35
gALL_v3_c24496	1	3	-	-	-	-	-
gALL_v3_c24497	2	4	-	-	-	-	-
gALL_v3_c24499	2	3	gnl|CDD|191451	pfam06102, DUF947, Domain	gb|EFN66578.1|	UPF0399 protein v1g245966 [Camponotus floridanus]	1,00E-42
gALL_v3_c245	8	11	-	-	gb|AGM32138.1|	ATP synthase lipid-binding protein [Coptotermes formosanus]	2,00E-10
gALL_v3_c2450	9	8	-	-	-	-	-

gALL_v3_c24500	1	6	-	-	-	-	-
gALL_v3_c24501	2	2	-	-	-	-	-
gALL_v3_c24502	1	2	gnl|CDD|162084	TIGR00879, SP, MFS transporte	ref|XP_002422622.1|	conserved hypothetical protein [Pediculus humanus corporis] gb|EEB09884.1| conserved hypothetical protein [Pediculus humanus corporis]	2,00E-18
gALL_v3_c24503	1	6	-	-	-	-	-
gALL_v3_c24504	2	3	-	-	-	-	-
gALL_v3_c24505	3	1	-	-	-	-	-
gALL_v3_c24506	1	3	-	-	-	-	-
gALL_v3_c24507	2	7	-	-	-	-	-
gALL_v3_c24508	2	4	-	-	-	-	-
gALL_v3_c24509	1	5	-	-	-	-	-
gALL_v3_c24510	1	7	-	-	-	-	-
gALL_v3_c24512	2	2	-	-	ref|XP_005726497.1|	PREDICTED: G patch domain-containing protein 1-like [Pundamilia nyererei]	5,00E-12
gALL_v3_c24513	1	2	-	-	-	-	-
gALL_v3_c24514	2	2	-	-	-	-	-
gALL_v3_c24515	0	3	-	-	gb|EFN65619.1|	Putative uncharacterized transposon-derived protein F52C9.6 [Camponotus floridanus]	6,00E-07
gALL_v3_c24516	1	4	-	-	-	-	-
gALL_v3_c24517	2	2	-	-	ref|XP_001948857.2|	PREDICTED: neurobeachin-like protein 1-like [Acyrthosiphon pisum]	6,00E-43
gALL_v3_c24518	1	3	-	-	-	-	-
gALL_v3_c24519	1	3	-	-	-	-	-
gALL_v3_c2452	9	13	gnl|CDD|128524	smart00228, PDZ, Domain pre	gb|EGI63441.1|	Membrane-associated guanylate kinase, WW and PDZ domain-containing protein 2 [Acromyrmex echinatior]	1,00E-124
gALL_v3_c24520	4	4	-	-	-	-	-
gALL_v3_c24522	0	4	-	-	-	-	-
gALL_v3_c24525	1	4	-	-	ref|XP_005180913.1|	PREDICTED: uncharacterized protein LOC101899226 [Musca domestica]	2,00E-15
gALL_v3_c24526	2	1	gnl|CDD|128921	smart00676, DM10, Domains	ref|XP_001298124.1|	MGC84469 protein [Trichomonas vaginalis G3] gb|EAX85194.1| MGC84469 protein, putative [Trichomonas vaginalis G3]	4,00E-61
gALL_v3_c24528	1	3	-	-	-	-	-
gALL_v3_c24529	3	2	-	-	ref|XP_966553.1|	PREDICTED: similar to phosphatidylcholine-sterol acyltransferase (lecithin-cholesterol acyltransferase) [Tribolium castaneum] gb|EFA04227.1| hypothetical protein TcasGA	1,00E-21
gALL_v3_c2453	16	22	-	-	-	-	-
gALL_v3_c24530	4	3	-	-	-	-	-
gALL_v3_c24531	3	2	-	-	-	-	-
gALL_v3_c24532	2	3	-	-	-	-	-
gALL_v3_c24533	0	4	-	-	gb|EFN63884.1|	hypothetical protein EAG_08086 [Camponotus floridanus]	2,00E-10
gALL_v3_c24534	2	1	-	-	-	-	-
gALL_v3_c24535	3	8	-	-	-	-	-
gALL_v3_c24536	1	4	-	-	-	-	-
gALL_v3_c24537	1	3	gnl|CDD|128349	smart00034, CLECT, C-type lec	gb|EFA04672.1|	hypothetical protein TcasGA2_TC014328 [Tribolium castaneum]	2,00E-50
gALL_v3_c24538	0	5	-	-	-	-	-
gALL_v3_c24540	2	2	-	-	-	-	-
gALL_v3_c24541	1	3	-	-	ref|XP_002428046.1|	dual 3',5'-cyclic-AMP and -GMP phosphodiesterase, putative [Pediculus humanus corporis] gb|EEB15308.1| dual 3',5'-cyclic-AMP and -GMP phosphodiesterase, putative [F	4,00E-68
gALL_v3_c24542	0	4	-	-	-	-	-
gALL_v3_c24543	0	4	-	-	-	-	-
gALL_v3_c24545	3	2	-	-	-	-	-
gALL_v3_c24546	3	1	-	-	-	-	-
gALL_v3_c2455	24	15	-	-	ref|XP_002428887.1|	Ras-GTPase-activating protein-binding protein, putative [Pediculus humanus corporis] gb|EEB16149.1| Ras-GTPase-activating protein-binding protein, putative [Pediculus I	2,00E-28
gALL_v3_c24550	3	2	-	-	-	-	-
gALL_v3_c24551	1	4	gnl|CDD|189488	pfam00289, CPSase_L_chain, (	ref|XP_003704972.1|	PREDICTED: acetyl-CoA carboxylase-like isoform 1 [Megachile rotundata]	1,00E-124
gALL_v3_c24552	1	5	-	-	ref|XP_006611947.1|	PREDICTED: protein-associating with the carboxyl-terminal domain of ezrin-like isoform X4 [Apis dorsata]	2,00E-47
gALL_v3_c24553	2	2	-	-	-	-	-
gALL_v3_c24554	2	3	-	-	-	-	-
gALL_v3_c24555	1	3	-	-	-	-	-
gALL_v3_c24556	2	1	-	-	-	-	-
gALL_v3_c24557	0	3	-	-	-	-	-
gALL_v3_c24558	0	3	-	-	-	-	-
gALL_v3_c24559	0	4	gnl|CDD|189839	pfam01094, ANF_receptor, Re	gb|EFN84958.1|	Retinal guanylyl cyclase 2 [Harpegnathos saltator]	5,00E-13
gALL_v3_c2456	10	6	-	-	ref|XP_003489033.1|	PREDICTED: integrator complex subunit 6-like [Bombus impatiens]	1,00E-101
gALL_v3_c24560	5	6	-	-	ref|XP_002060935.1|	GK10815 [Drosophila willistoni] gb|EDW71921.1| GK10815 [Drosophila willistoni]	2,00E-09
gALL_v3_c24561	3	2	-	-	-	-	-
gALL_v3_c24562	3	3	-	-	ref|XP_001604409.2|	PREDICTED: mps one binder kinase activator-like 1-like [Nasonia vitripennis]	3,00E-08
gALL_v3_c24563	1	4	-	-	-	-	-
gALL_v3_c24564	0	4	-	-	-	-	-
gALL_v3_c24565	2	4	-	-	-	-	-
gALL_v3_c24566	2	1	-	-	ref|XP_002427738.1|	vacuolar protein sorting-associated protein, putative [Pediculus humanus corporis] gb|EEB15000.1| vacuolar protein sorting-associated protein, putative [Pediculus humar	2,00E-16
gALL_v3_c24567	4	12	-	-	-	-	-
gALL_v3_c24568	5	3	-	-	ref|XP_003706195.1|	PREDICTED: uncharacterized protein LOC100876997 [Megachile rotundata]	2,00E-19
gALL_v3_c24569	4	2	-	-	-	-	-
gALL_v3_c2457	10	19	gnl|CDD|163652	cd07409, MPP_CD73_N, CD73	ref|XP_003400605.1|	PREDICTED: protein 5NUC-like isoform 1 [Bombus terrestris] ref|XP_003400606.1| PREDICTED: protein 5NUC-like isoform 2 [Bombus terrestris]	1,00E-145
gALL_v3_c24570	2	1	-	-	-	-	-
gALL_v3_c24571	0	3	-	-	-	-	-
gALL_v3_c24573	3	4	-	-	-	-	-
gALL_v3_c24574	2	2	-	-	-	-	-
gALL_v3_c24575	1	4	-	-	-	-	-
gALL_v3_c24576	2	2	gnl|CDD|189508	pfam00337, Gal-bind_lectin, G	dbj|BAM20137.1|	unknown unsecreted protein [Papilio xuthus]	2,00E-17
gALL_v3_c24577	1	3	-	-	-	-	-
gALL_v3_c24578	9	7	gnl|CDD|189445	pfam00199, Catalase, Catalase	gb|AFV36369.1|	catalase [Reticulitermes flavipes]	1,00E-75
gALL_v3_c24579	2	2	-	-	-	-	-
gALL_v3_c2458	10	13	gnl|CDD|176289	cd01213, tensin, Tensin Phosp	ref|XP_002424660.1|	tens, putative [Pediculus humanus corporis] gb|EEB11922.1| tens, putative [Pediculus humanus corporis]	2,00E-76
gALL_v3_c24580	3	3	-	-	-	-	-
gALL_v3_c24581	2	5	-	-	-	-	-
gALL_v3_c24582	2	1	-	-	-	-	-
gALL_v3_c24583	3	1	gnl|CDD|191075	pfam04739, AMPKBI, 5'-AMP-	ref|XP_003402479.1|	PREDICTED: 5'-AMP-activated protein kinase subunit beta-1-like [Bombus terrestris] ref|XP_003486737.1| PREDICTED: 5'-AMP-activated protein kinase subunit beta-1-like	4,00E-81
gALL_v3_c24585	3	2	-	-	-	-	-
gALL_v3_c24587	2	3	gnl|CDD|189369	pfam00061, Lipocalin, Lipocali	gb|AGM32122.1|	lipocalin / cytosolic fatty-acid binding protein [Coptotermes formosanus]	2,00E-65
gALL_v3_c24589	3	6	-	-	ref|XP_970868.1|	PREDICTED: similar to conserved hypothetical protein [Tribolium castaneum]	2,00E-51
gALL_v3_c2459	8	17	gnl|CDD|29151	cd00189, TPR, Tetratricopeptic	ref|XP_006825971.1|	PREDICTED: uncharacterized protein LOC100368918 [Saccoglossus kowalevskii]	4,00E-77
gALL_v3_c24590	1	3	-	-	ref|WP_004599161.1|	hypothetical protein [Rickettsia prowazekii] gb|EOB10793.1| hypothetical protein H377_2860 [Rickettsia prowazekii str. Cairo 3]	7,00E-60
gALL_v3_c24591	3	1	-	-	-	-	-
gALL_v3_c24592	1	2	-	-	gb|AGM32367.1|	syntaxin-12 [Coptotermes formosanus]	3,00E-14
gALL_v3_c24593	3	1	-	-	-	-	-
gALL_v3_c24595	0	4	-	-	-	-	-
gALL_v3_c24596	5	5	gnl|CDD|189954	pfam01359, Transposase_1, Tr	emb|CAP20051.1|	transposase [Portunus pelagicus]	6,00E-33
gALL_v3_c24597	1	2	-	-	-	-	-
gALL_v3_c24598	6	9	-	-	-	-	-
gALL_v3_c246	5	12	-	-	gb|EFA13373.1|	hypothetical protein TcasGA2_TC001390 [Tribolium castaneum]	1,00E-49
gALL_v3_c2460	9	12	-	-	ref|XP_321196.5|	AGAP001870-PA [Anopheles gambiae str. PEST] gb|EAA01594.6| AGAP001870-PA [Anopheles gambiae str. PEST]	3,00E-20
gALL_v3_c24600	0	4	-	-	-	-	-
gALL_v3_c24601	1	6	-	-	-	-	-
gALL_v3_c24602	3	11	-	-	-	-	-
gALL_v3_c24603	3	3	-	-	-	-	-
gALL_v3_c24604	1	3	-	-	-	-	-
gALL_v3_c24605	0	3	-	-	-	-	-
gALL_v3_c24606	3	5	-	-	-	-	-
gALL_v3_c24608	2	2	-	-	ref|XP_004930147.1|	PREDICTED: uncharacterized protein LOC101740603 [Bombyx mori]	9,00E-17
gALL_v3_c24609	2	3	-	-	-	-	-
gALL_v3_c2461	3	8	gnl|CDD|176377	cd01782, AF6_RA_repeat1, Ub	gb|EFZ22244.1|	hypothetical protein SINV_03689 [Solenopsis invicta]	1,00E-77
gALL_v3_c24610	3	1	gnl|CDD|144473	pfam00888, Cullin, Cullin fami	ref|XP_002432485.1|	Cullin-3, putative [Pediculus humanus corporis] gb|EEB19747.1| Cullin-3, putative [Pediculus humanus corporis]	1,00E-97
gALL_v3_c24611	0	3	-	-	-	-	-
gALL_v3_c24612	1	3	-	-	-	-	-
gALL_v3_c24613	3	6	-	-	ref|XP_006609999.1|	PREDICTED: peptidylprolyl isomerase domain and WD repeat-containing protein 1-like isoform X2 [Apis dorsata]	1,00E-47
gALL_v3_c24614	17	10	-	-	-	-	-
gALL_v3_c24615	0	5	-	-	-	-	-
gALL_v3_c24616	2	2	gnl|CDD|189634	pfam00620, RhoGAP, RhoGAP	ref|XP_970436.2|	PREDICTED: similar to breakpoint cluster region protein [Tribolium castaneum] gb|EFA00987.1| hypothetical protein TcasGA2_TC003900 [Tribolium castaneum]	1,00E-29
gALL_v3_c24617	2	2	-	-	-	-	-
gALL_v3_c24618	3	1	-	-	ref|XP_002427628.1|	conserved hypothetical protein [Pediculus humanus corporis] gb|EEB14890.1| conserved hypothetical protein [Pediculus humanus corporis]	3,00E-07
gALL_v3_c24619	1	2	-	-	-	-	-
gALL_v3_c2462	5	6	-	-	-	-	-
gALL_v3_c24620	3	3	-	-	gb|ESO07079.1|	hypothetical protein HELRODRAFT_184661 [Helobdella robusta]	8,00E-32
gALL_v3_c24621	1	5	gnl|CDD|173488	PTZ00239, PTZ00239, serine/t	dbj|BAB25919.1|	unnamed protein product [Mus musculus]	1,00E-32
gALL_v3_c24623	4	10	-	-	-	-	-
gALL_v3_c24624	1	3	-	-	-	-	-
gALL_v3_c24626	2	2	-	-	-	-	-
gALL_v3_c24627	2	3	-	-	-	-	-
gALL_v3_c24629	3	2	-	-	-	-	-
gALL_v3_c2463	32	22	-	-	gb|EFA05436.1|	hypothetical protein TcasGA2_TC015613 [Tribolium castaneum]	9,00E-50
gALL_v3_c24630	3	6	gnl|CDD|29115	cd00170, SEC14, Sec14p-like li	ref|XP_001602751.1|	PREDICTED: alpha-tocopherol transfer protein-like [Nasonia vitripennis]	1,00E-58
gALL_v3_c24631	3	1	-	-	-	-	-
gALL_v3_c24632	2	3	-	-	-	-	-
gALL_v3_c24634	5	2	-	-	-	-	-
gALL_v3_c24635	4	4	-	-	-	-	-
gALL_v3_c24636	3	1	gnl|CDD|143945	pfam00179, UQ_con, Ubiquiti	ref|XP_003707391.1|	PREDICTED: AKT-interacting protein-like [Megachile rotundata]	8,00E-55
gALL_v3_c24637	4	6	gnl|CDD|143235	cd05758, Ig5_KIRREL3-like, Fif	ref|XP_975294.1|	PREDICTED: similar to nephrin [Tribolium castaneum] gb|EFA00099.1| hypothetical protein TcasGA2_TC002914 [Tribolium castaneum]	0
gALL_v3_c24638	3	6	-	-	-	-	-
gALL_v3_c24639	3	2	-	-	-	-	-
gALL_v3_c2464	2	4	gnl|CDD|147050	pfam04697, Pinin_SDK_N, pin	gb|EFA07683.1|	hypothetical protein TcasGA2_TC030729 [Tribolium castaneum]	1,00E-23
gALL_v3_c24640	1	3	-	-	-	-	-
gALL_v3_c24641	1	2	-	-	-	-	-
gALL_v3_c24642	0	3	-	-	-	-	-
gALL_v3_c24643	5	4	-	-	-	-	-
gALL_v3_c24644	1	3	-	-	-	-	-
gALL_v3_c24645	1	3	gnl|CDD|173623	cd00180, PKc, Catalytic domai	ref|XP_001603402.2|	PREDICTED: TBC domain-containing protein kinase-like protein-like [Nasonia vitripennis]	5,00E-50
gALL_v3_c24646	2	3	gnl|CDD|188096	TIGR00930, 2a30, K-Cl cotrans	ref|XP_002428768.1|	sodium-potassium-chloride cotransporter, putative [Pediculus humanus corporis] gb|EEB16030.1| sodium-potassium-chloride cotransporter, putative [Pediculus humanus	4,00E-93
gALL_v3_c24647	3	1	-	-	-	-	-
gALL_v3_c24648	2	2	-	-	-	-	-
gALL_v3_c24649	1	3	-	-	-	-	-
gALL_v3_c2465	22	19	gnl|CDD|30625	COG0277, GlcD, FAD/FMN-cor	gb|AEE62432.1|	unknown [Dendroctonus ponderosae] gb|ERL86064.1| hypothetical protein D910_03478 [Dendroctonus ponderosae]	1,00E-144
gALL_v3_c24650	0	3	-	-	-	-	-

<table>
<tr><td>gALL_v3_c24651</td><td>2</td><td>7</td><td>-</td><td>-</td><td>-</td><td>-</td><td>-</td></tr>
<tr><td>gALL_v3_c24652</td><td>3</td><td>2</td><td>-</td><td>-</td><td>-</td><td>-</td><td>-</td></tr>
<tr><td>gALL_v3_c24655</td><td>3</td><td>1</td><td>-</td><td>-</td><td>-</td><td>-</td><td>-</td></tr>
<tr><td>gALL_v3_c24656</td><td>12</td><td>11</td><td>gnl|CDD|190129</td><td>pfam01835, A2M_N, MG2 dor</td><td>ref|XP_001944348.2|</td><td>PREDICTED: CD109 antigen-like [Acyrthosiphon pisum]</td><td>1,00E-118</td></tr>
<tr><td>gALL_v3_c24657</td><td>5</td><td>6</td><td>-</td><td>-</td><td>-</td><td>-</td><td>-</td></tr>
<tr><td>gALL_v3_c24659</td><td>2</td><td>3</td><td>-</td><td>-</td><td>-</td><td>-</td><td>-</td></tr>
<tr><td>gALL_v3_c2466</td><td>8</td><td>4</td><td>gnl|CDD|178495</td><td>PLN02907, PLN02907, glutama</td><td>ref|XP_006812505.1|</td><td>PREDICTED: bifunctional glutamate/proline--tRNA ligase-like [Saccoglossus kowalevskii]</td><td>2,00E-72</td></tr>
<tr><td>gALL_v3_c24660</td><td>1</td><td>3</td><td>-</td><td>-</td><td>-</td><td>-</td><td>-</td></tr>
<tr><td>gALL_v3_c24661</td><td>3</td><td>1</td><td>-</td><td>-</td><td>gb|EGI66262.1|</td><td>FLJ37770-like protein [Acromyrmex echinatior]</td><td>1,00E-09</td></tr>
<tr><td>gALL_v3_c24663</td><td>2</td><td>2</td><td>-</td><td>-</td><td>-</td><td>-</td><td>-</td></tr>
<tr><td>gALL_v3_c24664</td><td>0</td><td>2</td><td>-</td><td>-</td><td>-</td><td>-</td><td>-</td></tr>
<tr><td>gALL_v3_c24665</td><td>3</td><td>1</td><td>-</td><td>-</td><td>-</td><td>-</td><td>-</td></tr>
<tr><td>gALL_v3_c24666</td><td>3</td><td>3</td><td>-</td><td>-</td><td>-</td><td>-</td><td>-</td></tr>
<tr><td>gALL_v3_c24669</td><td>3</td><td>1</td><td>-</td><td>-</td><td>-</td><td>-</td><td>-</td></tr>
<tr><td>gALL_v3_c2467</td><td>6</td><td>3</td><td>-</td><td>-</td><td>-</td><td>-</td><td>-</td></tr>
<tr><td>gALL_v3_c24670</td><td>1</td><td>3</td><td>gnl|CDD|144473</td><td>pfam00888, Cullin, Cullin fami</td><td>ref|XP_002432485.1|</td><td>Cullin-3, putative [Pediculus humanus corporis] gb|EEB19747.1| Cullin-3, putative [Pediculus humanus corporis]</td><td>1,00E-129</td></tr>
<tr><td>gALL_v3_c24672</td><td>2</td><td>1</td><td>-</td><td>-</td><td>-</td><td>-</td><td>-</td></tr>
<tr><td>gALL_v3_c24673</td><td>1</td><td>6</td><td>-</td><td>-</td><td>-</td><td>-</td><td>-</td></tr>
<tr><td>gALL_v3_c24674</td><td>2</td><td>4</td><td>-</td><td>-</td><td>-</td><td>-</td><td>-</td></tr>
<tr><td>gALL_v3_c24675</td><td>5</td><td>4</td><td>gnl|CDD|30859</td><td>COG0513, SrmB, Superfamily I</td><td>gb|ESO94696.1|</td><td>hypothetical protein LOTGIDRAFT_176701 [Lottia gigantea]</td><td>7,00E-58</td></tr>
<tr><td>gALL_v3_c24676</td><td>7</td><td>4</td><td>gnl|CDD|178654</td><td>PLN03107, PLN03107, eukaryc</td><td>ref|XP_001607976.1|</td><td>PREDICTED: eukaryotic translation initiation factor 5A-like isoform 2 [Nasonia vitripennis] ref|XP_001607974.1| PREDICTED: eukaryotic translation initiation factor 5A-like i</td><td>4,00E-70</td></tr>
<tr><td>gALL_v3_c24677</td><td>1</td><td>3</td><td>-</td><td>-</td><td>-</td><td>-</td><td>-</td></tr>
<tr><td>gALL_v3_c24678</td><td>2</td><td>2</td><td>-</td><td>-</td><td>-</td><td>-</td><td>-</td></tr>
<tr><td>gALL_v3_c24679</td><td>1</td><td>3</td><td>-</td><td>-</td><td>-</td><td>-</td><td>-</td></tr>
<tr><td>gALL_v3_c2468</td><td>18</td><td>15</td><td>gnl|CDD|184937</td><td>PRK14974, PRK14974, cell divi</td><td>ref|XP_001651530.1|</td><td>signal recognition particle receptor alpha subunit (sr-alpha) [Aedes aegypti] gb|EAT42647.1| AAEL005856-PA [Aedes aegypti]</td><td>1,00E-165</td></tr>
<tr><td>gALL_v3_c24680</td><td>3</td><td>2</td><td>-</td><td>-</td><td>-</td><td>-</td><td>-</td></tr>
<tr><td>gALL_v3_c24681</td><td>1</td><td>3</td><td>-</td><td>-</td><td>-</td><td>-</td><td>-</td></tr>
<tr><td>gALL_v3_c24682</td><td>1</td><td>4</td><td>-</td><td>-</td><td>ref|XP_004925815.1|</td><td>PREDICTED: ubiquitin carboxyl-terminal hydrolase 8-like [Bombyx mori]</td><td>3,00E-40</td></tr>
<tr><td>gALL_v3_c24683</td><td>1</td><td>5</td><td>-</td><td>-</td><td>-</td><td>-</td><td>-</td></tr>
<tr><td>gALL_v3_c24684</td><td>0</td><td>4</td><td>-</td><td>-</td><td>-</td><td>-</td><td>-</td></tr>
<tr><td>gALL_v3_c24685</td><td>0</td><td>3</td><td>-</td><td>-</td><td>-</td><td>-</td><td>-</td></tr>
<tr><td>gALL_v3_c24686</td><td>2</td><td>2</td><td>-</td><td>-</td><td>-</td><td>-</td><td>-</td></tr>
<tr><td>gALL_v3_c24688</td><td>1</td><td>4</td><td>-</td><td>-</td><td>ref|XP_002412580.1|</td><td>lztr-1, putative [Ixodes scapularis] gb|EEC15876.1| lztr-1, putative [Ixodes scapularis]</td><td>1,00E-18</td></tr>
<tr><td>gALL_v3_c24689</td><td>2</td><td>1</td><td>-</td><td>-</td><td>-</td><td>-</td><td>-</td></tr>
<tr><td>gALL_v3_c2469</td><td>14</td><td>14</td><td>-</td><td>-</td><td>ref|XP_002429800.1|</td><td>hypothetical protein Phum_PHUM451730 [Pediculus humanus corporis] gb|EEB17062.1| hypothetical protein Phum_PHUM451730 [Pediculus humanus corporis]</td><td>4,00E-07</td></tr>
<tr><td>gALL_v3_c24690</td><td>1</td><td>2</td><td>-</td><td>-</td><td>-</td><td>-</td><td>-</td></tr>
<tr><td>gALL_v3_c24691</td><td>3</td><td>4</td><td>-</td><td>-</td><td>-</td><td>-</td><td>-</td></tr>
<tr><td>gALL_v3_c24692</td><td>4</td><td>4</td><td>-</td><td>-</td><td>-</td><td>-</td><td>-</td></tr>
<tr><td>gALL_v3_c24693</td><td>2</td><td>5</td><td>-</td><td>-</td><td>-</td><td>-</td><td>-</td></tr>
<tr><td>gALL_v3_c24694</td><td>2</td><td>2</td><td>-</td><td>-</td><td>-</td><td>-</td><td>-</td></tr>
<tr><td>gALL_v3_c24695</td><td>6</td><td>3</td><td>gnl|CDD|29261</td><td>cd00204, ANK, ankyrin repeat:</td><td>gb|AGE59088.1|</td><td>ankyrin repeat PH and SEC7 domain containing protein [Paramecium bursaria Chlorella virus OR0704.2.2]</td><td>1,00E-16</td></tr>
<tr><td>gALL_v3_c24696</td><td>1</td><td>4</td><td>gnl|CDD|162277</td><td>TIGR01271, CFTR_protein, cys</td><td>gb|EHJ68225.1|</td><td>putative ATP-dependent bile acid permease [Danaus plexippus]</td><td>1,00E-37</td></tr>
<tr><td>gALL_v3_c24697</td><td>1</td><td>4</td><td>-</td><td>-</td><td>-</td><td>-</td><td>-</td></tr>
<tr><td>gALL_v3_c24698</td><td>3</td><td>1</td><td>-</td><td>-</td><td>ref|XP_006564560.1|</td><td>PREDICTED: LOW QUALITY PROTEIN: supervillin-like [Apis mellifera]</td><td>2,00E-33</td></tr>
<tr><td>gALL_v3_c24699</td><td>0</td><td>3</td><td>-</td><td>-</td><td>-</td><td>-</td><td>-</td></tr>
<tr><td>gALL_v3_c2470</td><td>5</td><td>2</td><td>gnl|CDD|162084</td><td>TIGR00879, SP, MFS transporte</td><td>ref|XP_002431803.1|</td><td>sugar transporter, putative [Pediculus humanus corporis] gb|EEB19065.1| sugar transporter, putative [Pediculus humanus corporis]</td><td>3,00E-40</td></tr>
<tr><td>gALL_v3_c24700</td><td>0</td><td>4</td><td>-</td><td>-</td><td>-</td><td>-</td><td>-</td></tr>
<tr><td>gALL_v3_c24701</td><td>2</td><td>2</td><td>-</td><td>-</td><td>ref|XP_003697547.1|</td><td>PREDICTED: uncharacterized protein LOC100871163 [Apis florea]</td><td>2,00E-28</td></tr>
<tr><td>gALL_v3_c24702</td><td>4</td><td>4</td><td>gnl|CDD|145321</td><td>pfam02099, Josephin, Josephi</td><td>ref|XP_786733.3|</td><td>PREDICTED: ataxin-3-like [Strongylocentrotus purpuratus]</td><td>2,00E-41</td></tr>
<tr><td>gALL_v3_c24703</td><td>3</td><td>5</td><td>gnl|CDD|145379</td><td>pfam02192, PI3K_p85B, PI3-ki</td><td>ref|XP_003398686.1|</td><td>PREDICTED: phosphatidylinositol-4,5-bisphosphate 3-kinase catalytic subunit delta isoform-like [Bombus terrestris]</td><td>4,00E-43</td></tr>
<tr><td>gALL_v3_c24704</td><td>6</td><td>10</td><td>gnl|CDD|73156</td><td>cd01650, RT_nLTR_like, RT_nL</td><td>ref|XP_003243973.1|</td><td>PREDICTED: hypothetical protein LOC100570299 [Acyrthosiphon pisum]</td><td>8,00E-30</td></tr>
<tr><td>gALL_v3_c24705</td><td>1</td><td>2</td><td>gnl|CDD|133354</td><td>cd04154, Arl2, Arl2 subfamily.</td><td>gb|AEJ38209.1|</td><td>ADP-ribosylation factor-like protein 2 [Laodelphax striatella]</td><td>3,00E-17</td></tr>
<tr><td>gALL_v3_c24706</td><td>5</td><td>2</td><td>gnl|CDD|162739</td><td>TIGR02168, SMC_prok_B, chrc</td><td>gb|EGI66057.1|</td><td>Rho-associated protein kinase 2 [Acromyrmex echinatior]</td><td>8,00E-93</td></tr>
<tr><td>gALL_v3_c24707</td><td>0</td><td>3</td><td>-</td><td>-</td><td>-</td><td>-</td><td>-</td></tr>
<tr><td>gALL_v3_c24708</td><td>0</td><td>4</td><td>-</td><td>-</td><td>-</td><td>-</td><td>-</td></tr>
<tr><td>gALL_v3_c24709</td><td>3</td><td>1</td><td>-</td><td>-</td><td>gb|EFZ15761.1|</td><td>hypothetical protein SINV_08973 [Solenopsis invicta]</td><td>3,00E-35</td></tr>
<tr><td>gALL_v3_c2471</td><td>10</td><td>25</td><td>-</td><td>-</td><td>-</td><td>-</td><td>-</td></tr>
<tr><td>gALL_v3_c24710</td><td>0</td><td>6</td><td>-</td><td>-</td><td>ref|XP_002428038.1|</td><td>reverse transcriptase, putative [Pediculus humanus corporis] gb|EEB15300.1| reverse transcriptase, putative [Pediculus humanus corporis]</td><td>2,00E-24</td></tr>
<tr><td>gALL_v3_c24711</td><td>2</td><td>1</td><td>-</td><td>-</td><td>-</td><td>-</td><td>-</td></tr>
<tr><td>gALL_v3_c24712</td><td>2</td><td>4</td><td>-</td><td>-</td><td>ref|XP_002422823.1|</td><td>zinc finger protein, putative [Pediculus humanus corporis] gb|EEB10085.1| zinc finger protein, putative [Pediculus humanus corporis]</td><td>5,00E-18</td></tr>
<tr><td>gALL_v3_c24713</td><td>0</td><td>4</td><td>-</td><td>-</td><td>-</td><td>-</td><td>-</td></tr>
<tr><td>gALL_v3_c24714</td><td>1</td><td>3</td><td>-</td><td>-</td><td>-</td><td>-</td><td>-</td></tr>
<tr><td>gALL_v3_c24715</td><td>4</td><td>6</td><td>-</td><td>-</td><td>gb|ACG80812.1|</td><td>hypothetical protein, partial [Chlamydia pecorum]</td><td>2,00E-14</td></tr>
<tr><td>gALL_v3_c24716</td><td>3</td><td>2</td><td>-</td><td>-</td><td>-</td><td>-</td><td>-</td></tr>
<tr><td>gALL_v3_c24717</td><td>2</td><td>2</td><td>-</td><td>-</td><td>-</td><td>-</td><td>-</td></tr>
<tr><td>gALL_v3_c24718</td><td>5</td><td>3</td><td>gnl|CDD|190715</td><td>pfam03694, Erg28, Erg28 like</td><td>ref|XP_004536087.1|</td><td>PREDICTED: uncharacterized protein LOC101455651 [Ceratitis capitata]</td><td>2,00E-62</td></tr>
<tr><td>gALL_v3_c24719</td><td>2</td><td>2</td><td>-</td><td>-</td><td>-</td><td>-</td><td>-</td></tr>
<tr><td>gALL_v3_c24721</td><td>4</td><td>5</td><td>-</td><td>-</td><td>emb|CCO66533.1|</td><td>PREDICTED: similar to Zinc finger protein 271 (Zinc finger protein 7) (HZF7) (Zinc finger protein ZNFphex133) (Epstein-Barr virus-induced zinc finger protein) (ZNF-EB) (CT-ZF</td><td>2,00E-13</td></tr>
<tr><td>gALL_v3_c24722</td><td>1</td><td>6</td><td>gnl|CDD|128688</td><td>smart00410, IG_like, Immunog</td><td>ref|XP_003425150.1|</td><td>PREDICTED: tyrosine-protein kinase transmembrane receptor ROR2-like [Nasonia vitripennis]</td><td>1,00E-30</td></tr>
<tr><td>gALL_v3_c24724</td><td>1</td><td>3</td><td>-</td><td>-</td><td>-</td><td>-</td><td>-</td></tr>
<tr><td>gALL_v3_c24725</td><td>4</td><td>13</td><td>-</td><td>-</td><td>dbj|BAH72330.1|</td><td>ACYPI006530 [Acyrthosiphon pisum]</td><td>4,00E-54</td></tr>
<tr><td>gALL_v3_c24726</td><td>2</td><td>3</td><td>-</td><td>-</td><td>-</td><td>-</td><td>-</td></tr>
<tr><td>gALL_v3_c24727</td><td>4</td><td>5</td><td>-</td><td>-</td><td>ref|XP_001660592.1|</td><td>hypothetical protein AaeL_AAEL010042 [Aedes aegypti] gb|EAT38029.1| AAEL010042-PA [Aedes aegypti]</td><td>4,00E-26</td></tr>
<tr><td>gALL_v3_c24728</td><td>2</td><td>2</td><td>-</td><td>-</td><td>-</td><td>-</td><td>-</td></tr>
<tr><td>gALL_v3_c24729</td><td>0</td><td>3</td><td>-</td><td>-</td><td>-</td><td>-</td><td>-</td></tr>
<tr><td>gALL_v3_c2473</td><td>9</td><td>11</td><td>-</td><td>-</td><td>-</td><td>-</td><td>-</td></tr>
<tr><td>gALL_v3_c24730</td><td>1</td><td>2</td><td>-</td><td>-</td><td>-</td><td>-</td><td>-</td></tr>
<tr><td>gALL_v3_c24731</td><td>3</td><td>1</td><td>-</td><td>-</td><td>-</td><td>-</td><td>-</td></tr>
<tr><td>gALL_v3_c24732</td><td>1</td><td>3</td><td>-</td><td>-</td><td>-</td><td>-</td><td>-</td></tr>
<tr><td>gALL_v3_c24734</td><td>2</td><td>1</td><td>-</td><td>-</td><td>-</td><td>-</td><td>-</td></tr>
<tr><td>gALL_v3_c24735</td><td>1</td><td>2</td><td>-</td><td>-</td><td>-</td><td>-</td><td>-</td></tr>
<tr><td>gALL_v3_c24736</td><td>2</td><td>2</td><td>-</td><td>-</td><td>-</td><td>-</td><td>-</td></tr>
<tr><td>gALL_v3_c24737</td><td>2</td><td>2</td><td>-</td><td>-</td><td>ref|XP_006557771.1|</td><td>PREDICTED: LOW QUALITY PROTEIN: nitric oxide-associated protein 1-like [Apis mellifera]</td><td>2,00E-08</td></tr>
<tr><td>gALL_v3_c24738</td><td>1</td><td>4</td><td>-</td><td>-</td><td>-</td><td>-</td><td>-</td></tr>
<tr><td>gALL_v3_c24739</td><td>2</td><td>7</td><td>gnl|CDD|144080</td><td>pfam00350, Dynamin_N, Dyna</td><td>ref|XP_001657306.1|</td><td>dynamin [Aedes aegypti] gb|EAT44850.1| AAEL003829-PA [Aedes aegypti]</td><td>2,00E-84</td></tr>
<tr><td>gALL_v3_c2474</td><td>7</td><td>9</td><td>-</td><td>-</td><td>-</td><td>-</td><td>-</td></tr>
<tr><td>gALL_v3_c24740</td><td>1</td><td>3</td><td>-</td><td>-</td><td>-</td><td>-</td><td>-</td></tr>
<tr><td>gALL_v3_c24741</td><td>2</td><td>3</td><td>-</td><td>-</td><td>-</td><td>-</td><td>-</td></tr>
<tr><td>gALL_v3_c24742</td><td>2</td><td>4</td><td>-</td><td>-</td><td>-</td><td>-</td><td>-</td></tr>
<tr><td>gALL_v3_c24744</td><td>0</td><td>5</td><td>-</td><td>-</td><td>-</td><td>-</td><td>-</td></tr>
<tr><td>gALL_v3_c24745</td><td>4</td><td>2</td><td>-</td><td>-</td><td>-</td><td>-</td><td>-</td></tr>
<tr><td>gALL_v3_c24746</td><td>2</td><td>2</td><td>-</td><td>-</td><td>-</td><td>-</td><td>-</td></tr>
<tr><td>gALL_v3_c24747</td><td>3</td><td>3</td><td>-</td><td>-</td><td>ref|XP_002101042.1|</td><td>GE17398 [Drosophila yakuba] gb|EDX02150.1| GE17398 [Drosophila yakuba]</td><td>7,00E-35</td></tr>
<tr><td>gALL_v3_c24748</td><td>2</td><td>1</td><td>-</td><td>-</td><td>-</td><td>-</td><td>-</td></tr>
<tr><td>gALL_v3_c24749</td><td>2</td><td>1</td><td>-</td><td>-</td><td>-</td><td>-</td><td>-</td></tr>
<tr><td>gALL_v3_c24750</td><td>0</td><td>4</td><td>-</td><td>-</td><td>gb|EFZ22363.1|</td><td>hypothetical protein SINV_14405 [Solenopsis invicta]</td><td>5,00E-11</td></tr>
<tr><td>gALL_v3_c24751</td><td>1</td><td>2</td><td>-</td><td>-</td><td>-</td><td>-</td><td>-</td></tr>
<tr><td>gALL_v3_c24752</td><td>2</td><td>6</td><td>-</td><td>-</td><td>ref|XP_004924606.1|</td><td>PREDICTED: YTH domain family protein 3-like isoform X2 [Bombyx mori]</td><td>4,00E-34</td></tr>
<tr><td>gALL_v3_c24753</td><td>1</td><td>2</td><td>-</td><td>-</td><td>-</td><td>-</td><td>-</td></tr>
<tr><td>gALL_v3_c24754</td><td>7</td><td>5</td><td>gnl|CDD|189372</td><td>pfam00067, p450, Cytochrom</td><td>ref|XP_974014.1|</td><td>PREDICTED: similar to AGAP006082-PA [Tribolium castaneum] gb|EFA02906.1| cytochrome P450 301A1 [Tribolium castaneum]</td><td>4,00E-80</td></tr>
<tr><td>gALL_v3_c24756</td><td>4</td><td>3</td><td>-</td><td>-</td><td>-</td><td>-</td><td>-</td></tr>
<tr><td>gALL_v3_c24757</td><td>0</td><td>3</td><td>-</td><td>-</td><td>-</td><td>-</td><td>-</td></tr>
<tr><td>gALL_v3_c24758</td><td>2</td><td>2</td><td>gnl|CDD|192775</td><td>pfam11568, Med29, Mediator</td><td>ref|XP_002427140.1|</td><td>conserved hypothetical protein [Pediculus humanus corporis] gb|EEB14402.1| conserved hypothetical protein [Pediculus humanus corporis]</td><td>3,00E-36</td></tr>
<tr><td>gALL_v3_c24759</td><td>5</td><td>4</td><td>-</td><td>-</td><td>-</td><td>-</td><td>-</td></tr>
<tr><td>gALL_v3_c2476</td><td>4</td><td>10</td><td>-</td><td>-</td><td>-</td><td>-</td><td>-</td></tr>
<tr><td>gALL_v3_c24760</td><td>2</td><td>3</td><td>-</td><td>-</td><td>-</td><td>-</td><td>-</td></tr>
<tr><td>gALL_v3_c24761</td><td>2</td><td>2</td><td>-</td><td>-</td><td>-</td><td>-</td><td>-</td></tr>
<tr><td>gALL_v3_c24762</td><td>0</td><td>6</td><td>-</td><td>-</td><td>-</td><td>-</td><td>-</td></tr>
<tr><td>gALL_v3_c24764</td><td>3</td><td>4</td><td>-</td><td>-</td><td>-</td><td>-</td><td>-</td></tr>
<tr><td>gALL_v3_c24765</td><td>1</td><td>3</td><td>-</td><td>-</td><td>-</td><td>-</td><td>-</td></tr>
<tr><td>gALL_v3_c24766</td><td>1</td><td>5</td><td>-</td><td>-</td><td>-</td><td>-</td><td>-</td></tr>
<tr><td>gALL_v3_c24767</td><td>1</td><td>7</td><td>-</td><td>-</td><td>-</td><td>-</td><td>-</td></tr>
<tr><td>gALL_v3_c24768</td><td>2</td><td>2</td><td>-</td><td>-</td><td>-</td><td>-</td><td>-</td></tr>
<tr><td>gALL_v3_c24769</td><td>0</td><td>6</td><td>-</td><td>-</td><td>-</td><td>-</td><td>-</td></tr>
<tr><td>gALL_v3_c2477</td><td>7</td><td>13</td><td>gnl|CDD|132850</td><td>cd07211, Pat_PNPLA8, Patatin</td><td>ref|XP_001601278.2|</td><td>PREDICTED: calcium-independent phospholipase A2-gamma-like [Nasonia vitripennis]</td><td>0</td></tr>
<tr><td>gALL_v3_c24770</td><td>3</td><td>2</td><td>-</td><td>-</td><td>-</td><td>-</td><td>-</td></tr>
<tr><td>gALL_v3_c24771</td><td>1</td><td>8</td><td>-</td><td>-</td><td>-</td><td>-</td><td>-</td></tr>
<tr><td>gALL_v3_c24772</td><td>1</td><td>2</td><td>-</td><td>-</td><td>-</td><td>-</td><td>-</td></tr>
<tr><td>gALL_v3_c24773</td><td>3</td><td>4</td><td>-</td><td>-</td><td>-</td><td>-</td><td>-</td></tr>
<tr><td>gALL_v3_c24774</td><td>3</td><td>2</td><td>-</td><td>-</td><td>-</td><td>-</td><td>-</td></tr>
<tr><td>gALL_v3_c24775</td><td>0</td><td>5</td><td>-</td><td>-</td><td>-</td><td>-</td><td>-</td></tr>
<tr><td>gALL_v3_c24776</td><td>2</td><td>2</td><td>-</td><td>-</td><td>-</td><td>-</td><td>-</td></tr>
<tr><td>gALL_v3_c24777</td><td>0</td><td>5</td><td>-</td><td>-</td><td>-</td><td>-</td><td>-</td></tr>
<tr><td>gALL_v3_c24778</td><td>0</td><td>4</td><td>-</td><td>-</td><td>ref|XP_006614963.1|</td><td>PREDICTED: bromodomain adjacent to zinc finger domain protein 2B-like [Apis dorsata]</td><td>4,00E-35</td></tr>
<tr><td>gALL_v3_c24779</td><td>1</td><td>2</td><td>-</td><td>-</td><td>-</td><td>-</td><td>-</td></tr>
<tr><td>gALL_v3_c2478</td><td>18</td><td>12</td><td>-</td><td>-</td><td>ref|XP_006163116.1|</td><td>PREDICTED: pogo transposable element with KRAB domain [Tupaia chinensis] gb|ELV13317.1| Pogo transposable element with KRAB domain [Tupaia chinensis]</td><td>4,00E-20</td></tr>
<tr><td>gALL_v3_c24780</td><td>1</td><td>3</td><td>-</td><td>-</td><td>-</td><td>-</td><td>-</td></tr>
<tr><td>gALL_v3_c24781</td><td>2</td><td>2</td><td>-</td><td>-</td><td>-</td><td>-</td><td>-</td></tr>
<tr><td>gALL_v3_c24784</td><td>5</td><td>3</td><td>gnl|CDD|48457</td><td>cd03759, proteasome_beta_ty</td><td>ref|XP_003427914.1|</td><td>PREDICTED: proteasome subunit beta type-3-like isoform 2 [Nasonia vitripennis]</td><td>4,00E-72</td></tr>
<tr><td>gALL_v3_c24785</td><td>0</td><td>3</td><td>-</td><td>-</td><td>-</td><td>-</td><td>-</td></tr>
<tr><td>gALL_v3_c24786</td><td>5</td><td>6</td><td>gnl|CDD|144462</td><td>pfam00876, Innexin, Innexin.</td><td>ref|XP_968731.1|</td><td>PREDICTED: similar to innexin [Tribolium castaneum]</td><td>2,00E-16</td></tr>
<tr><td>gALL_v3_c24787</td><td>2</td><td>4</td><td>gnl|CDD|48666</td><td>cd03572, ENTH_epsin_related</td><td>ref|XP_006145725.1|</td><td>PREDICTED: AP-4 complex accessory subunit tepsin isoform X2 [Tupaia chinensis]</td><td>7,00E-11</td></tr>
<tr><td>gALL_v3_c24788</td><td>1</td><td>3</td><td>-</td><td>-</td><td>-</td><td>-</td><td>-</td></tr>
<tr><td>gALL_v3_c24789</td><td>2</td><td>2</td><td>-</td><td>-</td><td>-</td><td>-</td><td>-</td></tr>
<tr><td>gALL_v3_c2479</td><td>7</td><td>6</td><td>gnl|CDD|192379</td><td>pfam09790, Hyccin, Hyccin. M</td><td>ref|XP_001607177.2|</td><td>PREDICTED: hyccin-like [Nasonia vitripennis]</td><td>1,00E-121</td></tr>
<tr><td>gALL_v3_c24790</td><td>0</td><td>3</td><td>gnl|CDD|143386</td><td>cd00141, NT_POLXc, Nucleoti</td><td>ref|XP_005657709.1|</td><td>PREDICTED: DNA polymerase beta isoform X1 [Sus scrofa]</td><td>6,00E-43</td></tr>
<tr><td>gALL_v3_c24791</td><td>2</td><td>3</td><td>gnl|CDD|189834</td><td>pfam01084, Ribosomal_S18, R</td><td>ref|XP_971303.1|</td><td>PREDICTED: similar to ubiquitin fusion degradation protein 2 [Tribolium castaneum] gb|EFA10545.1| hypothetical protein TcasGA2_TC012801 [Tribolium castaneum]</td><td>1,00E-44</td></tr>
<tr><td>gALL_v3_c24792</td><td>2</td><td>3</td><td>-</td><td>-</td><td>-</td><td>-</td><td>-</td></tr>
<tr><td>gALL_v3_c24793</td><td>1</td><td>3</td><td>-</td><td>-</td><td>-</td><td>-</td><td>-</td></tr>
<tr><td>gALL_v3_c24794</td><td>2</td><td>4</td><td>-</td><td>-</td><td>-</td><td>-</td><td>-</td></tr>
<tr><td>gALL_v3_c24795</td><td>1</td><td>3</td><td>-</td><td>-</td><td>gb|ERL93178.1|</td><td>hypothetical protein D910_10475 [Dendroctonus ponderosae]</td><td>2,00E-39</td></tr>
<tr><td>gALL_v3_c24796</td><td>1</td><td>2</td><td>-</td><td>-</td><td>ref|XP_001867410.1|</td><td>conserved hypothetical protein [Culex quinquefasciatus] gb|EDS44934.1| conserved hypothetical protein [Culex quinquefasciatus]</td><td>5,00E-34</td></tr>
<tr><td>gALL_v3_c24797</td><td>2</td><td>3</td><td>-</td><td>-</td><td>gb|EFN87075.1|</td><td>Heparanase [Harpegnathos saltator]</td><td>1,00E-27</td></tr>
<tr><td>gALL_v3_c24799</td><td>3</td><td>1</td><td>-</td><td>-</td><td>ref|XP_001653797.1|</td><td>rab [Aedes aegypti] gb|EAT38811.1| AAEL009342-PA [Aedes aegypti]</td><td>5,00E-22</td></tr>
</table>

gALL_v3_c2480	1	6	-	-	-	-	-
gALL_v3_c24800	5	3	gnl|CDD|162097	TIGR00898, 2A0119, cation tra	ref|XP_001662579.1|	sugar transporter [Aedes aegypti] gb|EAT35388.1| AAEL012443-PA [Aedes aegypti]	1,00E-126
gALL_v3_c24801	3	3	-	-	-	-	-
gALL_v3_c24802	0	3	-	-	-	-	-
gALL_v3_c24804	3	3	-	-	-	-	-
gALL_v3_c24807	0	3	-	-	-	-	-
gALL_v3_c24808	1	2	-	-	-	-	-
gALL_v3_c24809	5	3	gnl|CDD|183390	PRK12268, PRK12268, methior	ref|XP_969201.1|	PREDICTED: similar to AAEL014709-PA [Tribolium castaneum] gb|EFA06771.1| hypothetical protein TcasGA2_TC009707 [Tribolium castaneum]	1,00E-104
gALL_v3_c24810	1	3	-	-	-	-	-
gALL_v3_c24811	3	6	-	-	-	-	-
gALL_v3_c24812	2	6	gnl|CDD|178635	PLN03086, PLN03086, PRLI-int	ref|XP_004929105.1|	PREDICTED: uncharacterized protein LOC101745142 [Bombyx mori]	6,00E-45
gALL_v3_c24814	3	4	-	-	-	-	-
gALL_v3_c24815	2	2	-	-	-	-	-
gALL_v3_c24816	2	6	-	-	-	-	-
gALL_v3_c24817	1	3	-	-	gb|EGI60342.1|	Transient receptor potential channel pyrexia [Acromyrmex echinatior]	4,00E-29
gALL_v3_c24818	2	7	gnl|CDD|146653	pfam04124, Dor1, Dor1-like fa	ref|XP_003701994.1|	PREDICTED: conserved oligomeric Golgi complex subunit 8-like [Megachile rotundata]	3,00E-99
gALL_v3_c24819	0	3	gnl|CDD|144735	pfam01251, Ribosomal_S7e, R	ref|XP_001582415.1|	40S ribosomal protein S7 [Trichomonas vaginalis G3] gb|EAY21429.1| 40S ribosomal protein S7, putative [Trichomonas vaginalis G3]	1,00E-27
gALL_v3_c2482	10	8	gnl|CDD|34495	COG4886, COG4886, Leucine-r	ref|XP_318952.3|	AGAP009839-PA [Anopheles gambiae str. PEST] gb|EAA14303.4| AGAP009839-PA [Anopheles gambiae str. PEST]	2,00E-84
gALL_v3_c24820	2	4	-	-	-	-	-
gALL_v3_c24821	2	1	-	-	-	-	-
gALL_v3_c24822	3	1	-	-	ref|XP_006610134.1|	PREDICTED: protein FAM8A1-like isoform X2 [Apis dorsata]	2,00E-11
gALL_v3_c24824	0	5	-	-	-	-	-
gALL_v3_c24825	0	3	-	-	-	-	-
gALL_v3_c24827	2	2	-	-	-	-	-
gALL_v3_c2483	7	8	gnl|CDD|146328	pfam03630, Fumble, Fumble.	ref|XP_006617108.1|	PREDICTED: pantothenate kinase 1-like isoform X2 [Apis dorsata]	2,00E-99
gALL_v3_c24832	0	4	-	-	-	-	-
gALL_v3_c24833	4	4	-	-	gb|EFA03165.1|	hypothetical protein TcasGA2_TC013085 [Tribolium castaneum]	4,00E-39
gALL_v3_c24834	1	2	-	-	-	-	-
gALL_v3_c24835	3	3	-	-	gb|AGH30328.1|	argonaute-3 [Nilaparvata lugens]	2,00E-08
gALL_v3_c24837	3	3	-	-	-	-	-
gALL_v3_c24838	4	5	-	-	-	-	-
gALL_v3_c24839	1	3	-	-	-	-	-
gALL_v3_c2484	11	14	gnl|CDD|130324	TIGR01257, rim_protein, retin	ref|XP_001947916.2|	PREDICTED: ATP-binding cassette sub-family A member 3-like [Acyrthosiphon pisum]	1,00E-145
gALL_v3_c24840	3	1	gnl|CDD|151191	pfam10695, Cw-hydrolase, Cel	ref|YP_004822531.1|	hypothetical protein PARA_08330 [Haemophilus parainfluenzae T3T1] ref|WP_005629862.1| hydrolase [Haemophilus] emb|CBW14553.1| 50s ribosomal protein l4 (prolip	1,00E-31
gALL_v3_c24841	2	1	-	-	-	-	-
gALL_v3_c24842	1	2	-	-	-	-	-
gALL_v3_c24843	1	2	-	-	-	-	-
gALL_v3_c24844	4	2	-	-	ref|XP_006748240.1|	PREDICTED: centrosomal protein of 104 kDa [Leptonychotes weddellii]	8,00E-47
gALL_v3_c24845	1	4	-	-	-	-	-
gALL_v3_c24846	0	3	-	-	-	-	-
gALL_v3_c24847	2	2	-	-	ref|XP_002426410.1|	conserved hypothetical protein [Pediculus humanus corporis] gb|EEB13672.1| conserved hypothetical protein [Pediculus humanus corporis]	6,00E-09
gALL_v3_c24849	1	2	-	-	-	-	-
gALL_v3_c2485	8	21	-	-	ref|XP_002431137.1|	hypothetical protein Phum_PHUM513220 [Pediculus humanus corporis] gb|EEB18399.1| hypothetical protein Phum_PHUM513220 [Pediculus humanus corporis]	6,00E-21
gALL_v3_c24850	1	2	-	-	-	-	-
gALL_v3_c24852	2	4	gnl|CDD|193590	cd09976, LOTUS_3_TDRD5, Th	gb|EKC34343.1|	Tudor domain-containing protein 5 [Crassostrea gigas]	7,00E-18
gALL_v3_c24853	2	2	-	-	-	-	-
gALL_v3_c24855	3	1	-	-	-	-	-
gALL_v3_c24856	2	2	-	-	-	-	-
gALL_v3_c24857	2	2	-	-	-	-	-
gALL_v3_c24858	2	4	gnl|CDD|144473	pfam00888, Cullin, Cullin fami	gb|EGI69720.1|	Cullin-3 [Acromyrmex echinatior]	2,00E-88
gALL_v3_c24859	3	3	-	-	ref|XP_002430688.1|	golgi-specific brefeldin A-resistance guanine nucleotide exchange factor, putative [Pediculus humanus corporis] gb|EEB17950.1| golgi-specific brefeldin A-resistance guanin	3,00E-69
gALL_v3_c24861	1	4	gnl|CDD|162097	TIGR00898, 2A0119, cation tra	gb|EFN86109.1|	Organic cation transporter protein [Harpegnathos saltator]	4,00E-20
gALL_v3_c24862	1	5	gnl|CDD|189635	pfam00621, RhoGEF, RhoGEF d	ref|XP_003706421.1|	PREDICTED: dynamin-binding protein-like [Megachile rotundata]	1,00E-54
gALL_v3_c24864	7	8	-	-	-	-	-
gALL_v3_c24865	7	6	-	-	ref|XP_005103941.1|	PREDICTED: piggyBac transposable element-derived protein 4-like [Aplysia californica]	2,00E-33
gALL_v3_c24866	2	5	-	-	gb|EFZ09476.1|	hypothetical protein SINV_09217 [Solenopsis invicta]	7,00E-91
gALL_v3_c24867	1	3	-	-	-	-	-
gALL_v3_c24868	1	3	-	-	-	-	-
gALL_v3_c2487	11	9	-	-	gb|EFN65942.1|	Afadin [Camponotus floridanus]	8,00E-09
gALL_v3_c24870	0	4	-	-	-	-	-
gALL_v3_c24871	1	3	-	-	ref|XP_004925181.1|	PREDICTED: LOW QUALITY PROTEIN: DNA-directed RNA polymerase I subunit RPA1-like [Bombyx mori]	1,00E-42
gALL_v3_c24872	1	3	-	-	-	-	-
gALL_v3_c24873	2	2	-	-	-	-	-
gALL_v3_c24874	1	3	-	-	ref|XP_968592.2|	PREDICTED: similar to AGAP005815-PA [Tribolium castaneum] gb|EEZ99837.1| hypothetical protein TcasGA2_TC002618 [Tribolium castaneum]	7,00E-45
gALL_v3_c24876	1	3	-	-	gb|EEZ97195.1|	hypothetical protein TcasGA2_TC004382 [Tribolium castaneum]	4,00E-44
gALL_v3_c24877	2	2	-	-	-	-	-
gALL_v3_c24878	0	5	-	-	ref|XP_006811772.1|	PREDICTED: phosphatidylinositide phosphatase SAC1-like [Saccoglossus kowalevskii]	2,00E-09
gALL_v3_c24879	4	2	gnl|CDD|176188	cd05285, sorbitol_DH, Sorbito	ref|XP_001655101.1|	alcohol dehydrogenase [Aedes aegypti] ref|XP_001655102.1| alcohol dehydrogenase [Aedes aegypti] ref|XP_001655103.1| alcohol dehydrogenase [Aedes aegypti] gb|EA	6,00E-64
gALL_v3_c2488	19	14	gnl|CDD|189372	pfam00067, p450, Cytochrome	ref|XP_002428676.1|	cytochrome P-450, putative [Pediculus humanus corporis] gb|EEB15938.1| cytochrome P-450, putative [Pediculus humanus corporis]	4,00E-24
gALL_v3_c24880	2	2	gnl|CDD|128895	smart00647, IBR, In Between I	ref|XP_002428785.1|	protein ariadne-1, putative [Pediculus humanus corporis] gb|EEB16047.1| protein ariadne-1, putative [Pediculus humanus corporis]	1,00E-138
gALL_v3_c24881	0	5	-	-	-	-	-
gALL_v3_c24883	1	4	-	-	gb|EFZ15431.1|	hypothetical protein SINV_04588 [Solenopsis invicta]	3,00E-48
gALL_v3_c24884	4	8	-	-	-	-	-
gALL_v3_c24885	5	9	gnl|CDD|162465	TIGR01652, ATPase-Plipid, pho	ref|XP_001650985.1|	phospholipid-transporting atpase 1 (aminophospholipid flippase 1) [Aedes aegypti] gb|EAT48161.1| AAEL000767-PA [Aedes aegypti]	0
gALL_v3_c24886	1	2	-	-	-	-	-
gALL_v3_c24887	3	5	-	-	-	-	-
gALL_v3_c24888	2	5	-	-	-	-	-
gALL_v3_c24889	4	2	-	-	gb|EFN89314.1|	hypothetical protein EAI_05291 [Harpegnathos saltator]	3,00E-11
gALL_v3_c2489	7	7	-	-	gb|EFN75044.1|	Uncharacterized protein KIAA1109 [Camponotus floridanus]	1,00E-121
gALL_v3_c24890	1	4	-	-	-	-	-
gALL_v3_c24892	2	7	-	-	-	-	-
gALL_v3_c24893	0	4	-	-	-	-	-
gALL_v3_c24894	1	4	-	-	-	-	-
gALL_v3_c24895	5	3	-	-	gb|EFN67634.1|	Putative uncharacterized transposon-derived protein F52C9.6 [Camponotus floridanus]	3,00E-15
gALL_v3_c24896	2	2	-	-	-	-	-
gALL_v3_c24898	3	1	-	-	-	-	-
gALL_v3_c24899	4	2	-	-	-	-	-
gALL_v3_c249	21	24	-	-	ref|XP_972543.2|	PREDICTED: similar to split ends CG18497-PA [Tribolium castaneum]	2,00E-26
gALL_v3_c2490	4	6	-	-	-	-	-
gALL_v3_c24900	0	4	-	-	gb|EFZ18342.1|	hypothetical protein SINV_03782 [Solenopsis invicta]	8,00E-10
gALL_v3_c24901	0	4	-	-	-	-	-
gALL_v3_c24902	1	2	-	-	-	-	-
gALL_v3_c24903	1	2	-	-	-	-	-
gALL_v3_c24905	2	1	-	-	ref|XP_001358640.2|	GA10156 [Drosophila pseudoobscura pseudoobscura] gb|EAL27781.2| GA10156 [Drosophila pseudoobscura pseudoobscura]	6,00E-14
gALL_v3_c24906	3	2	-	-	-	-	-
gALL_v3_c24907	0	4	-	-	-	-	-
gALL_v3_c24908	1	2	-	-	-	-	-
gALL_v3_c24909	8	7	-	-	-	-	-
gALL_v3_c2491	7	10	gnl|CDD|119351	cd02872, GH18_chitolectin_ch	ref|XP_970191.2|	PREDICTED: similar to AGAP005634-PA [Tribolium castaneum] gb|EFA06923.1| hypothetical protein TcasGA2_TC009872 [Tribolium castaneum]	1,00E-128
gALL_v3_c24910	2	1	-	-	-	-	-
gALL_v3_c24911	2	2	-	-	-	-	-
gALL_v3_c24914	1	4	-	-	-	-	-
gALL_v3_c24915	2	6	-	-	-	-	-
gALL_v3_c24916	2	6	-	-	-	-	-
gALL_v3_c24917	2	2	-	-	-	-	-
gALL_v3_c24918	2	3	-	-	-	-	-
gALL_v3_c24919	2	1	-	-	gb|ERL95993.1|	hypothetical protein D910_00759, partial [Dendroctonus ponderosae]	7,00E-13
gALL_v3_c24920	1	2	-	-	-	-	-
gALL_v3_c24921	3	1	-	-	-	-	-
gALL_v3_c24922	0	4	gnl|CDD|192782	pfam11597, Med13_N, Media	ref|XP_002425114.1|	Thyroid hormone receptor-associated protein, putative [Pediculus humanus corporis] gb|EEB12376.1| Thyroid hormone receptor-associated protein, putative [Pediculus h	1,00E-59
gALL_v3_c24923	3	1	-	-	ref|XP_002424309.1|	conserved hypothetical protein [Pediculus humanus corporis] gb|EEB11571.1| conserved hypothetical protein [Pediculus humanus corporis]	2,00E-51
gALL_v3_c24924	2	5	-	-	-	-	-
gALL_v3_c24928	1	3	-	-	-	-	-
gALL_v3_c2493	9	9	gnl|CDD|189654	pfam00651, BTB, BTB/POZ dor	ref|XP_003706425.1|	PREDICTED: BTB/POZ domain-containing protein 2-like [Megachile rotundata]	1,00E-138
gALL_v3_c24931	3	1	-	-	-	-	-
gALL_v3_c24932	2	4	-	-	dbj|BAN20625.1|	ABC transporter [Riptortus pedestris]	5,00E-23
gALL_v3_c24933	2	2	-	-	-	-	-
gALL_v3_c24934	2	2	-	-	ref|NP_001191879.1|	sodium channel modifier 1 [Acyrthosiphon pisum]	5,00E-25
gALL_v3_c24936	2	2	-	-	ref|XP_002431867.1|	reverse transcriptase, putative [Pediculus humanus corporis] gb|EEB19129.1| reverse transcriptase, putative [Pediculus humanus corporis]	4,00E-14
gALL_v3_c24937	1	2	-	-	-	-	-
gALL_v3_c24938	0	3	-	-	-	-	-
gALL_v3_c24939	7	11	-	-	-	-	-
gALL_v3_c24940	2	2	-	-	-	-	-
gALL_v3_c24941	1	3	-	-	-	-	-
gALL_v3_c24942	1	3	-	-	-	-	-
gALL_v3_c24943	3	2	-	-	-	-	-
gALL_v3_c24944	4	3	-	-	-	-	-
gALL_v3_c24945	1	3	-	-	-	-	-
gALL_v3_c24946	3	2	-	-	ref|XP_003700574.1|	PREDICTED: uncharacterized protein LOC100883763 [Megachile rotundata]	1,00E-15
gALL_v3_c24947	4	2	-	-	ref|XP_004578568.1|	PREDICTED: formin-2 [Ochotona princeps]	3,00E-17
gALL_v3_c24948	2	2	-	-	-	-	-
gALL_v3_c24949	0	5	-	-	-	-	-
gALL_v3_c24950	5	6	-	-	-	-	-
gALL_v3_c24951	0	3	-	-	-	-	-
gALL_v3_c24952	0	4	gnl|CDD|185453	PTZ00110, PTZ00110, helicase	gb|AAO15910.1|AF5100	helicase RM62-like protein A [Schistocerca gregaria]	3,00E-40
gALL_v3_c24953	4	2	gnl|CDD|88619	cd05156, ChoK_euk, Choline K	gb|EFN85093.1|	Choline/ethanolamine kinase [Harpegnathos saltator]	5,00E-42
gALL_v3_c24954	6	4	-	-	ref|XP_002432745.1|	conserved hypothetical protein [Pediculus humanus corporis] gb|EEB20007.1| conserved hypothetical protein [Pediculus humanus corporis]	3,00E-24
gALL_v3_c24955	3	2	-	-	-	-	-
gALL_v3_c24956	2	2	gnl|CDD|189008	cd09601, M1_APN_2, Peptida	ref|XP_003706475.1|	PREDICTED: aminopeptidase N-like [Megachile rotundata]	3,00E-17
gALL_v3_c24957	2	3	-	-	-	-	-
gALL_v3_c24958	2	5	gnl|CDD|189424	pfam00153, Mito_carr, Mitoch	gb|EHJ66181.1|	hypothetical protein KGM_13704 [Danaus plexippus]	1,00E-78
gALL_v3_c2496	9	5	gnl|CDD|129984	TIGR00906, 2A0303, cationic a	gb|EFZ22227.1|	hypothetical protein SINV_08605 [Solenopsis invicta]	2,00E-89
gALL_v3_c24961	2	4	-	-	-	-	-
gALL_v3_c24962	1	3	gnl|CDD|178162	PLN02547, PLN02547, dUTP p	gb|EHJ64263.1|	deoxyuridine 5'-triphosphate nucleotidohydrolase [Danaus plexippus]	2,00E-52

gALL_v3_c24963	2	2	-	-	-	-	-
gALL_v3_c24964	0	3	-	-	-	-	-
gALL_v3_c24966	4	2	-	-	-	-	-
gALL_v3_c24967	1	3	-	-	gb|ESO02093.1|	hypothetical protein HELRODRAFT_161324 [Helobdella robusta]	2,00E-07
gALL_v3_c24968	3	2	-	-	-	-	-
gALL_v3_c24969	2	2	-	-	-	-	-
gALL_v3_c24970	3	1	-	-	gb|EFN84348.1|	TBC1 domain family member 16 [Harpegnathos saltator]	6,00E-65
gALL_v3_c24973	1	3	-	-	-	-	-
gALL_v3_c24974	4	2	gnl|CDD|162078	TIGR00870, trp, transient-rece	ref|XP_002432382.1|	conserved hypothetical protein [Pediculus humanus corporis] gb|EEB19644.1| conserved hypothetical protein [Pediculus humanus corporis]	1,00E-125
gALL_v3_c24975	2	3	-	-	-	-	-
gALL_v3_c24977	4	4	-	-	ref|XP_003242865.1|	PREDICTED: hypothetical protein LOC100572069 [Acyrthosiphon pisum]	1,00E-08
gALL_v3_c24978	3	1	-	-	-	-	-
gALL_v3_c2498	23	39	gnl|CDD|34727	COG5126, FRQ1, Ca2+-binding	ref|XP_001648788.1|	hippocalcin [Aedes aegypti] ref|XP_001687984.1| AGAP007247-PC [Anopheles gambiae str. PEST] ref|XP_001687985.1| AGAP007247-PA [Anopheles gambiae str. PEST] re	1,00E-123
gALL_v3_c24980	2	3	-	-	-	-	-
gALL_v3_c24981	1	4	-	-	-	-	-
gALL_v3_c24982	0	4	-	-	-	-	-
gALL_v3_c24983	9	21	-	-	ref|XP_002424577.1|	protein unzipped precursor, putative [Pediculus humanus corporis] gb|EEB11839.1| protein unzipped precursor, putative [Pediculus humanus corporis]	2,00E-65
gALL_v3_c24984	2	3	-	-	ref|XP_003701352.1|	PREDICTED: probable G-protein coupled receptor 125-like [Megachile rotundata]	9,00E-43
gALL_v3_c24986	3	1	-	-	-	-	-
gALL_v3_c24987	2	2	-	-	-	-	-
gALL_v3_c24988	3	2	-	-	-	-	-
gALL_v3_c24989	1	2	-	-	-	-	-
gALL_v3_c24990	2	2	-	-	-	-	-
gALL_v3_c24991	1	4	-	-	ref|XP_001651399.1|	hypothetical protein AaeL_AAEL005735 [Aedes aegypti] gb|EAT42770.1| AAEL005735-PA [Aedes aegypti]	8,00E-06
gALL_v3_c24993	1	2	gnl|CDD|73154	cd01647, RT_LTR, RT_LTR: Rev	gb|EFA13180.1|	hypothetical protein TcasGA2_TC010314 [Tribolium castaneum]	5,00E-27
gALL_v3_c24994	1	2	-	-	-	-	-
gALL_v3_c24995	0	5	-	-	-	-	-
gALL_v3_c24996	2	2	-	-	ref|XP_003698749.1|	PREDICTED: UDP-GlcNAc:betaGal beta-1,3-N-acetylglucosaminyltransferase 5-like [Apis florea]	4,00E-17
gALL_v3_c24997	3	2	-	-	ref|XP_003701557.1|	PREDICTED: uncharacterized protein LOC100882911 [Megachile rotundata]	4,00E-16
gALL_v3_c24998	2	1	-	-	-	-	-
gALL_v3_c24999	6	15	-	-	-	-	-
gALL_v3_c25000	1	2	-	-	-	-	-
gALL_v3_c25002	2	1	-	-	-	-	-
gALL_v3_c25003	2	3	-	-	-	-	-
gALL_v3_c25004	1	3	-	-	-	-	-
gALL_v3_c25005	1	2	-	-	-	-	-
gALL_v3_c25006	0	4	-	-	ref|WP_002430991.1|	conserved hypothetical protein [Escherichia sp. 3_2_53FAA] gb|EEH89633.1| hypothetical protein ESAG_07158 [Escherichia sp. 3_2_53FAA]	4,00E-19
gALL_v3_c25007	2	1	gnl|CDD|189625	pfam00609, DAGK_acc, Diacyl	gb|EGI61141.1|	Diacylglycerol kinase epsilon [Acromyrmex echinatior]	2,00E-18
gALL_v3_c25009	3	3	-	-	-	-	-
gALL_v3_c2501	12	17	gnl|CDD|34929	COG5354, COG5354, Uncharac	ref|XP_972849.1|	PREDICTED: similar to eIF3-S9 [Tribolium castaneum] gb|EFA08366.1| hypothetical protein TcasGA2_TC006009 [Tribolium castaneum]	0
gALL_v3_c25010	2	5	-	-	gb|EFA13480.1|	hypothetical protein TcasGA2_TC002031 [Tribolium castaneum]	2,00E-15
gALL_v3_c25011	3	1	-	-	-	-	-
gALL_v3_c25012	3	1	-	-	-	-	-
gALL_v3_c25013	2	2	-	-	-	-	-
gALL_v3_c25014	0	3	-	-	-	-	-
gALL_v3_c25015	2	2	-	-	-	-	-
gALL_v3_c25016	3	1	-	-	-	-	-
gALL_v3_c25017	3	2	-	-	-	-	-
gALL_v3_c25018	0	3	-	-	-	-	-
gALL_v3_c25019	0	4	-	-	-	-	-
gALL_v3_c2502	11	17	-	-	-	-	-
gALL_v3_c25020	1	3	gnl|CDD|190208	pfam02096, 60KD_IMP, 60Kd i	ref|XP_001652200.1|	cytochrome oxidase biogenesis protein (oxa1 mitochondrial) [Aedes aegypti] gb|EAT41659.1| AAEL006734-PA [Aedes aegypti]	2,00E-48
gALL_v3_c25021	2	4	-	-	ref|XP_002425428.1|	conserved hypothetical protein [Pediculus humanus corporis] gb|EEB12690.1| conserved hypothetical protein [Pediculus humanus corporis]	2,00E-73
gALL_v3_c25022	3	4	-	-	gb|EFN75884.1|	Putative uncharacterized protein FLJ37770 [Harpegnathos saltator]	1,00E-09
gALL_v3_c25023	2	1	-	-	-	-	-
gALL_v3_c25024	0	3	-	-	gb|EJW78011.1|	hypothetical protein WUBG_11079, partial [Wuchereria bancrofti]	8,00E-57
gALL_v3_c25026	0	4	-	-	-	-	-
gALL_v3_c25027	1	3	-	-	ref|XP_003399252.1|	PREDICTED: hypothetical protein LOC100646958 [Bombus terrestris]	2,00E-34
gALL_v3_c25028	0	4	-	-	-	-	-
gALL_v3_c25029	3	1	-	-	-	-	-
gALL_v3_c2503	18	42	gnl|CDD|173688	cd05597, STKc_DMPK_like, Ca	ref|XP_002432458.1|	serine/threonine-protein kinase MRCK beta, putative [Pediculus humanus corporis] gb|EEB19720.1| serine/threonine-protein kinase MRCK beta, putative [Pediculus huma	0
gALL_v3_c25030	1	4	-	-	-	-	-
gALL_v3_c25031	2	2	-	-	-	-	-
gALL_v3_c25032	1	3	-	-	-	-	-
gALL_v3_c25034	3	1	-	-	-	-	-
gALL_v3_c25035	13	8	-	-	ref|XP_001600556.1|	PREDICTED: prohibitin-2-like [Nasonia vitripennis]	5,00E-18
gALL_v3_c25036	4	7	gnl|CDD|189795	pfam00995, Sec1, Sec1 family.	ref|XP_002430586.1|	protein ROP, putative [Pediculus humanus corporis] gb|EEB17848.1| protein ROP, putative [Pediculus humanus corporis]	7,00E-74
gALL_v3_c25037	0	4	-	-	-	-	-
gALL_v3_c25039	0	3	-	-	-	-	-
gALL_v3_c2504	4	12	-	-	ref|XP_006936921.1|	PREDICTED: putative upstream-binding factor 1-like protein 1 [Felis catus]	6,00E-08
gALL_v3_c25040	1	4	-	-	ref|XP_002425295.1|	discs large protein, putative [Pediculus humanus corporis] gb|EEB12557.1| discs large protein, putative [Pediculus humanus corporis]	6,00E-50
gALL_v3_c25041	2	8	gnl|CDD|133263	cd01860, Rab5_related, Rab5-	ref|XP_783878.1|	PREDICTED: ras-related protein Rab-5B-like [Strongylocentrotus purpuratus]	3,00E-45
gALL_v3_c25042	2	1	-	-	-	-	-
gALL_v3_c25044	1	2	-	-	-	-	-
gALL_v3_c25045	5	4	gnl|CDD|176369	cd01774, Faf1_like2_UBX, Faf1	gb|ERL87412.1|	hypothetical protein D910_04807 [Dendroctonus ponderosae]	3,00E-51
gALL_v3_c25046	5	6	-	-	-	-	-
gALL_v3_c25047	2	4	-	-	-	-	-
gALL_v3_c25048	1	2	-	-	ref|XP_006612913.1|	PREDICTED: dynamin-binding protein-like [Apis dorsata]	2,00E-20
gALL_v3_c25049	2	5	-	-	-	-	-
gALL_v3_c2505	4	5	-	-	-	-	-
gALL_v3_c25051	2	4	-	-	-	-	-
gALL_v3_c25052	0	5	-	-	-	-	-
gALL_v3_c25054	3	2	-	-	-	-	-
gALL_v3_c25056	0	3	-	-	-	-	-
gALL_v3_c25057	1	5	-	-	-	-	-
gALL_v3_c25058	4	7	-	-	-	-	-
gALL_v3_c25059	3	3	-	-	-	-	-
gALL_v3_c2506	6	6	-	-	-	-	-
gALL_v3_c25060	0	3	-	-	-	-	-
gALL_v3_c25061	2	1	-	-	-	-	-
gALL_v3_c25063	3	1	-	-	-	-	-
gALL_v3_c25064	3	1	-	-	-	-	-
gALL_v3_c25065	17	16	gnl|CDD|190777	pfam03868, Ribosomal_L6e_N	gb|AGM32491.1|	60S ribosomal protein L6 [Coptotermes formosanus]	9,00E-79
gALL_v3_c25066	3	1	-	-	-	-	-
gALL_v3_c25067	3	2	-	-	-	-	-
gALL_v3_c25068	0	4	-	-	gb|EFN86932.1|	A disintegrin and metalloproteinase with thrombospondin motifs 20 [Harpegnathos saltator]	4,00E-33
gALL_v3_c25069	1	6	-	-	-	-	-
gALL_v3_c2507	10	9	-	-	-	-	-
gALL_v3_c25070	0	3	-	-	-	-	-
gALL_v3_c25071	2	2	-	-	-	-	-
gALL_v3_c25072	5	5	gnl|CDD|28959	cd00078, HECTc, HECT domain	gb|EFZ18511.1|	hypothetical protein SINV_14428 [Solenopsis invicta]	1,00E-164
gALL_v3_c25073	1	6	-	-	-	-	-
gALL_v3_c25075	3	1	-	-	-	-	-
gALL_v3_c25076	2	4	-	-	-	-	-
gALL_v3_c25077	2	3	-	-	tpg|DAA00890.1|	TPA_exp: reverse transcriptase [Schistosoma mansoni]	3,00E-07
gALL_v3_c25078	1	3	-	-	ref|XP_001606433.2|	PREDICTED: hypothetical protein LOC100122828 [Nasonia vitripennis]	7,00E-09
gALL_v3_c25079	1	3	-	-	gb|EHJ79138.1|	hypothetical protein KGM_15461 [Danaus plexippus]	1,00E-109
gALL_v3_c2508	2	10	gnl|CDD|143441	cd07123, ALDH_F4-17_P5CDH	ref|XP_313649.4|	AGAP004366-PA [Anopheles gambiae str. PEST] gb|EAA09247.4| AGAP004366-PA [Anopheles gambiae str. PEST]	2,00E-87
gALL_v3_c25080	1	3	-	-	ref|XP_004930507.1|	PREDICTED: uncharacterized protein LOC101737962 [Bombyx mori]	2,00E-23
gALL_v3_c25081	1	3	gnl|CDD|173746	cd07850, STKc_JNK, Catalytic d	gb|EFZ18152.1|	hypothetical protein SINV_15711 [Solenopsis invicta]	2,00E-19
gALL_v3_c25082	3	1	-	-	-	-	-
gALL_v3_c25083	4	3	-	-	-	-	-
gALL_v3_c25085	4	5	-	-	-	-	-
gALL_v3_c25086	1	3	-	-	-	-	-
gALL_v3_c25088	1	3	-	-	dbj|BAC37177.1|	unnamed protein product [Mus musculus]	5,00E-41
gALL_v3_c25089	3	2	-	-	-	-	-
gALL_v3_c2509	6	7	-	-	-	-	-
gALL_v3_c25090	3	7	gnl|CDD|189783	pfam00956, NAP, Nucleosome	ref|XP_003484877.1|	PREDICTED: nucleosome assembly protein 1-like 1-like [Bombus impatiens]	2,00E-20
gALL_v3_c25091	1	4	-	-	-	-	-
gALL_v3_c25092	2	4	-	-	-	-	-
gALL_v3_c25094	3	1	-	-	-	-	-
gALL_v3_c25095	1	3	-	-	-	-	-
gALL_v3_c25096	2	2	-	-	ref|XP_003398141.1|	PREDICTED: hypothetical protein LOC100649776 [Bombus terrestris]	9,00E-10
gALL_v3_c25097	2	3	-	-	ref|XP_002429794.1|	histone-lysine N-methyltransferase SUVR5, putative [Pediculus humanus corporis] gb|EEB17056.1| histone-lysine N-methyltransferase SUVR5, putative [Pediculus humanu	3,00E-16
gALL_v3_c25098	2	2	-	-	-	-	-
gALL_v3_c25099	4	6	-	-	gb|EEZ99670.1|	hypothetical protein TcasGA2_TC002427 [Tribolium castaneum]	4,00E-09
gALL_v3_c251	5	4	-	-	-	-	-
gALL_v3_c2510	19	35	-	-	-	-	-
gALL_v3_c25102	2	4	-	-	-	-	-
gALL_v3_c25103	0	3	-	-	-	-	-
gALL_v3_c25104	2	4	-	-	gb|EFN67634.1|	Putative uncharacterized transposon-derived protein F52C9.6 [Camponotus floridanus]	1,00E-16
gALL_v3_c25105	3	2	-	-	-	-	-
gALL_v3_c25106	2	1	-	-	-	-	-
gALL_v3_c25107	2	5	-	-	-	-	-
gALL_v3_c25108	3	1	-	-	-	-	-
gALL_v3_c25109	2	1	-	-	-	-	-
gALL_v3_c2511	2	7	-	-	-	-	-
gALL_v3_c25112	3	4	-	-	-	-	-
gALL_v3_c25113	2	1	-	-	-	-	-
gALL_v3_c25114	0	4	-	-	-	-	-
gALL_v3_c25115	2	2	-	-	ref|XP_002427107.1|	hypothetical protein Phum_PHUM294490 [Pediculus humanus corporis] gb|EEB14369.1| hypothetical protein Phum_PHUM294490 [Pediculus humanus corporis]	1,00E-29
gALL_v3_c25116	5	3	gnl|CDD|190340	pfam02535, Zip, ZIP Zinc trans	ref|XP_001952790.2|	PREDICTED: zinc transporter ZIP8-like [Acyrthosiphon pisum]	1,00E-77
gALL_v3_c25117	2	1	-	-	-	-	-
gALL_v3_c25118	2	3	-	-	ref|XP_002425594.1|	conserved hypothetical protein [Pediculus humanus corporis] gb|EEB12856.1| conserved hypothetical protein [Pediculus humanus corporis]	5,00E-35

gALL_v3_c25119	4	8	-	-	-	-	-
gALL_v3_c2512	4	6	-	-	-	-	-
gALL_v3_c25120	2	1	-	-	-	-	-
gALL_v3_c25121	3	2	-	-	-	-	-
gALL_v3_c25122	2	2	-	-	-	-	-
gALL_v3_c25123	0	4	gnl|CDD|192350	pfam09730, BicD, Microtubule	ref|XP_970925.2|	PREDICTED: similar to AGAP010206-PA [Tribolium castaneum] gb|EFA07458.1| bicaudal D [Tribolium castaneum]	1,00E-30
gALL_v3_c25124	1	3	gnl|CDD|145641	pfam02602, HEM4, Uroporphy	gb|ESO97024.1|	hypothetical protein LOTGIDRAFT_231769 [Lottia gigantea]	1,00E-07
gALL_v3_c25126	6	13	-	-	-	-	-
gALL_v3_c25127	2	2	-	-	-	-	-
gALL_v3_c25128	0	5	gnl|CDD|149354	pfam08242, Methyltransf_12,	gb|ADV17350.1|	juvenile hormone acid methyltransferase [Schistocerca gregaria]	1,00E-62
gALL_v3_c2513	9	10	-	-	gb|EFN84291.1|	hypothetical protein EAI_10080 [Harpegnathos saltator]	5,00E-25
gALL_v3_c25130	1	3	-	-	-	-	-
gALL_v3_c25132	3	1	-	-	-	-	-
gALL_v3_c25133	5	3	-	-	-	-	-
gALL_v3_c25134	3	1	gnl|CDD|173506	PTZ00281, PTZ00281, actin; Pr	gb|AAM10445.1|	actin [Culicoides nubeculosus]	2,00E-86
gALL_v3_c25135	2	3	-	-	gb|EFN67634.1|	Putative uncharacterized transposon-derived protein F52C9.6 [Camponotus floridanus]	1,00E-06
gALL_v3_c25136	1	2	-	-	-	-	-
gALL_v3_c25137	2	2	-	-	-	-	-
gALL_v3_c25138	0	4	-	-	-	-	-
gALL_v3_c25139	3	10	-	-	-	-	-
gALL_v3_c2514	7	9	-	-	-	-	-
gALL_v3_c25140	3	1	-	-	-	-	-
gALL_v3_c25142	2	2	-	-	ref|XP_003954268.1|	PREDICTED: piggyBac transposable element-derived protein 3 isoform 1 [Pan troglodytes] ref|XP_003954269.1| PREDICTED: piggyBac transposable element-derived prote	4,00E-12
gALL_v3_c25144	4	8	gnl|CDD|162063	TIGR00840, b_cpa1, sodium/h	ref|XP_002430244.1|	sodium/hydrogen exchanger, putative [Pediculus humanus corporis] gb|EEB17506.1| sodium/hydrogen exchanger, putative [Pediculus humanus corporis]	0
gALL_v3_c25145	2	1	-	-	-	-	-
gALL_v3_c25146	1	2	-	-	ref|XP_973656.1|	PREDICTED: hypothetical protein [Tribolium castaneum] gb|EFA01608.1| hypothetical protein TcasGA2_TC007174 [Tribolium castaneum]	2,00E-06
gALL_v3_c25147	1	4	-	-	-	-	-
gALL_v3_c25149	2	2	-	-	dbj|BAN21528.1|	serine/threonine protein phosphatase 2a regulatory subunit a, partial [Riptortus pedestris]	4,00E-45
gALL_v3_c2515	17	11	gnl|CDD|31133	COG0790, COG0790, FOG: TPF	gb|EGI65960.1|	Protein SHC1 [Acromyrmex echinatior]	5,00E-33
gALL_v3_c25150	5	4	gnl|CDD|185439	PTZ00091, PTZ00091, 40S ribo	gb|ENN76674.1|	hypothetical protein YQE_06740, partial [Dendroctonus ponderosae] gb|ERL89292.1| hypothetical protein D910_06665 [Dendroctonus ponderosae]	1,00E-102
gALL_v3_c25151	2	2	gnl|CDD|29376	cd01408, SIRT1, SIRT1: Eukary	gb|EFZ09226.1|	hypothetical protein SINV_06882 [Solenopsis invicta]	6,00E-43
gALL_v3_c25152	2	2	-	-	-	-	-
gALL_v3_c25154	1	2	-	-	-	-	-
gALL_v3_c25157	3	1	-	-	-	-	-
gALL_v3_c25158	3	1	-	-	-	-	-
gALL_v3_c25159	0	4	-	-	-	-	-
gALL_v3_c25160	0	3	-	-	ref|XP_001602419.2|	PREDICTED: hypothetical protein LOC100118457 [Nasonia vitripennis]	2,00E-07
gALL_v3_c25162	0	3	-	-	ref|XP_006564758.1|	PREDICTED: uncharacterized protein DDB_G0271670-like [Apis mellifera]	6,00E-33
gALL_v3_c25163	3	6	-	-	-	-	-
gALL_v3_c25164	1	6	gnl|CDD|149859	pfam08923, MAPKK1_Int, Mit	ref|XP_003700749.1|	PREDICTED: ragulator complex protein LAMTOR3-A-like [Megachile rotundata]	2,00E-50
gALL_v3_c25165	0	3	-	-	-	-	-
gALL_v3_c25166	3	4	gnl|CDD|48338	cd00305, Cu-Zn_Superoxide_D	gb|AAD25400.1|AF1229	extracellular superoxide dismutase precursor [Pacifastacus leniusculus]	2,00E-31
gALL_v3_c25167	4	5	-	-	-	-	-
gALL_v3_c25168	3	3	-	-	-	-	-
gALL_v3_c25169	1	3	gnl|CDD|146692	pfam04184, ST7, ST7 protein.	ref|XP_001599139.2|	PREDICTED: protein ST7 homolog [Nasonia vitripennis]	3,00E-43
gALL_v3_c2517	10	13	gnl|CDD|153281	cd07597, BAR_SNX8, The Bin/.	gb|EFN81977.1|	Sorting nexin-8 [Harpegnathos saltator]	0
gALL_v3_c25170	0	3	-	-	-	-	-
gALL_v3_c25171	2	3	-	-	-	-	-
gALL_v3_c25172	0	4	-	-	-	-	-
gALL_v3_c25173	2	1	-	-	-	-	-
gALL_v3_c25175	2	2	-	-	-	-	-
gALL_v3_c25176	0	3	-	-	-	-	-
gALL_v3_c25177	2	1	-	-	ref|XP_002427482.1|	conserved hypothetical protein [Pediculus humanus corporis] gb|EEB14744.1| conserved hypothetical protein [Pediculus humanus corporis]	1,00E-26
gALL_v3_c25178	1	2	-	-	-	-	-
gALL_v3_c25179	2	1	-	-	-	-	-
gALL_v3_c2518	6	5	-	-	ref|XP_004920015.1|	PREDICTED: uncharacterized protein LOC101731432 [Xenopus (Silurana) tropicalis]	8,00E-08
gALL_v3_c25180	2	2	-	-	-	-	-
gALL_v3_c25181	3	1	-	-	-	-	-
gALL_v3_c25182	2	2	-	-	-	-	-
gALL_v3_c25183	3	1	-	-	ref|XP_006628595.1|	PREDICTED: zinc finger protein 76-like [Lepisosteus oculatus]	1,00E-100
gALL_v3_c25184	0	3	-	-	-	-	-
gALL_v3_c25185	0	5	-	-	-	-	-
gALL_v3_c25186	2	2	-	-	-	-	-
gALL_v3_c25188	2	3	-	-	-	-	-
gALL_v3_c25189	2	4	-	-	-	-	-
gALL_v3_c2519	16	20	gnl|CDD|31228	COG1025, Ptr, Secreted/peripl	ref|XP_971897.1|	PREDICTED: similar to metalloprotease [Tribolium castaneum] gb|EEZ97922.1| hypothetical protein TcasGA2_TC000308 [Tribolium castaneum]	0
gALL_v3_c25190	4	6	-	-	-	-	-
gALL_v3_c25191	4	4	-	-	-	-	-
gALL_v3_c25192	3	1	-	-	-	-	-
gALL_v3_c25193	3	3	-	-	dbj|BAN20840.1|	myelinprotein expression factor [Riptortus pedestris]	5,00E-11
gALL_v3_c25194	1	3	-	-	-	-	-
gALL_v3_c25196	0	3	-	-	-	-	-
gALL_v3_c25197	1	3	-	-	-	-	-
gALL_v3_c25198	2	2	-	-	-	-	-
gALL_v3_c25199	1	5	-	-	dbj|BAG71783.1|	fmr [Gryllus bimaculatus]	7,00E-58
gALL_v3_c252	6	6	-	-	gb|AGM32456.1|	zinc metalloprotease-like protein [Coptotermes formosanus]	2,00E-07
gALL_v3_c25200	7	9	gnl|CDD|128358	smart00043, CY, Cystatin-like c	gb|ERL92612.1|	hypothetical protein D910_09925 [Dendroctonus ponderosae]	4,00E-17
gALL_v3_c25201	2	2	-	-	-	-	-
gALL_v3_c25203	2	3	-	-	ref|XP_005112306.1|	PREDICTED: mitochondrial sodium/hydrogen exchanger 9B2-like isoform X1 [Aplysia californica]	1,00E-39
gALL_v3_c25204	7	4	-	-	-	-	-
gALL_v3_c25205	0	3	-	-	-	-	-
gALL_v3_c25206	2	1	-	-	-	-	-
gALL_v3_c25207	2	1	gnl|CDD|143833	pfam00046, Homeobox, Home	gb|AAW21973.1|	ventral nerve cord defective protein [Tribolium castaneum]	2,00E-52
gALL_v3_c25208	1	3	-	-	-	-	-
gALL_v3_c25209	2	3	-	-	-	-	-
gALL_v3_c25210	1	3	-	-	-	-	-
gALL_v3_c25211	2	3	-	-	ref|XP_001944491.1|	PREDICTED: jerky protein homolog-like [Acyrthosiphon pisum]	6,00E-10
gALL_v3_c25212	3	2	-	-	-	-	-
gALL_v3_c25213	3	3	gnl|CDD|148730	pfam07289, DUF1448, Protein	ref|XP_966616.1|	PREDICTED: similar to Bardet-Biedl syndrome 5 [Tribolium castaneum] gb|EFA11133.1| hypothetical protein TcasGA2_TC004734 [Tribolium castaneum]	7,00E-89
gALL_v3_c25214	3	2	-	-	gb|AAB65093.1|	Lian-Aa1 retrotransposon protein [Aedes aegypti]	5,00E-19
gALL_v3_c25216	1	6	gnl|CDD|189421	pfam00150, Cellulase, Cellulas	gb|AGM32347.1|	putative glycosyl hydrolase family5 [Coptotermes formosanus]	1,00E-100
gALL_v3_c25217	0	3	-	-	gb|EFN73134.1|	Uncharacterized WD repeat-containing protein alr3466 [Camponotus floridanus]	1,00E-25
gALL_v3_c25218	1	6	-	-	-	-	-
gALL_v3_c25219	3	4	-	-	ref|XP_001599239.2|	PREDICTED: eukaryotic translation initiation factor 2-alpha kinase 4-like [Nasonia vitripennis]	3,00E-41
gALL_v3_c2522	9	7	-	-	ref|XP_001842091.1|	conserved hypothetical protein [Culex quinquefasciatus] gb|EDS37629.1| conserved hypothetical protein [Culex quinquefasciatus]	5,00E-09
gALL_v3_c25220	5	2	-	-	-	-	-
gALL_v3_c25221	1	5	-	-	-	-	-
gALL_v3_c25222	2	3	-	-	-	-	-
gALL_v3_c25223	3	4	-	-	-	-	-
gALL_v3_c25224	2	2	-	-	ref|XP_002425606.1|	notch, putative [Pediculus humanus corporis] gb|EEB12868.1| notch, putative [Pediculus humanus corporis]	1,00E-21
gALL_v3_c25225	2	2	-	-	-	-	-
gALL_v3_c25226	2	2	-	-	-	-	-
gALL_v3_c25227	0	6	-	-	-	-	-
gALL_v3_c25228	2	2	-	-	-	-	-
gALL_v3_c25229	2	1	-	-	-	-	-
gALL_v3_c25230	4	3	-	-	-	-	-
gALL_v3_c25231	0	3	-	-	-	-	-
gALL_v3_c25232	3	2	-	-	gb|EFA03852.1|	hypothetical protein TcasGA2_TC013968 [Tribolium castaneum]	2,00E-12
gALL_v3_c25233	4	2	gnl|CDD|191386	pfam05843, Suf, Suppressor of	gb|EFN79573.1|	Protein suppressor of forked [Harpegnathos saltator]	1,00E-73
gALL_v3_c25235	2	2	-	-	gb|AGM32876.1|	hypothetical protein [Coptotermes formosanus]	1,00E-05
gALL_v3_c25236	3	3	-	-	-	-	-
gALL_v3_c25238	5	4	gnl|CDD|185538	PTZ00280, PTZ00280, Actin-re	ref|XP_001325937.1|	Actin-like protein 3 [Trichomonas vaginalis G3] gb|EAY13714.1| Actin-like protein 3, putative [Trichomonas vaginalis G3]	1,00E-147
gALL_v3_c25239	3	3	-	-	-	-	-
gALL_v3_c2524	13	10	gnl|CDD|31415	COG1222, RPT1, ATP-depende	ref|XP_003492182.1|	PREDICTED: 26S protease regulatory subunit 7-like [Bombus impatiens] ref|XP_006571886.1| PREDICTED: 26S protease regulatory subunit 7 [Apis mellifera] ref|XP_00662	0
gALL_v3_c25240	3	1	-	-	ref|XP_001323495.1|	selective LIM binding factor [Trichomonas vaginalis G3] gb|EAY11272.1| selective LIM binding factor, putative [Trichomonas vaginalis G3]	7,00E-73
gALL_v3_c25241	3	3	-	-	-	-	-
gALL_v3_c25243	5	4	-	-	-	-	-
gALL_v3_c25244	1	2	-	-	-	-	-
gALL_v3_c25245	0	3	-	-	-	-	-
gALL_v3_c25246	2	4	-	-	-	-	-
gALL_v3_c25247	1	2	-	-	-	-	-
gALL_v3_c25248	3	5	gnl|CDD|189977	pfam01412, ArfGap, Putative (	gb|EFN62661.1|	Centaurin-alpha-1 [Camponotus floridanus]	1,00E-143
gALL_v3_c25249	1	2	-	-	-	-	-
gALL_v3_c25250	3	1	-	-	-	-	-
gALL_v3_c25251	2	1	-	-	-	-	-
gALL_v3_c25252	0	4	-	-	ref|XP_002428082.1|	conserved hypothetical protein [Pediculus humanus corporis] gb|EEB15344.1| conserved hypothetical protein [Pediculus humanus corporis]	4,00E-13
gALL_v3_c25253	0	5	-	-	gb|EFN71501.1|	Ankyrin-1 [Camponotus floridanus]	2,00E-06
gALL_v3_c25254	0	4	-	-	-	-	-
gALL_v3_c25255	3	1	-	-	-	-	-
gALL_v3_c25256	1	4	-	-	-	-	-
gALL_v3_c25257	2	4	-	-	-	-	-
gALL_v3_c25259	1	2	-	-	-	-	-
gALL_v3_c2526	9	18	-	-	-	-	-
gALL_v3_c25260	2	2	-	-	-	-	-
gALL_v3_c25261	2	2	-	-	-	-	-
gALL_v3_c25262	2	2	-	-	gb|EFN84495.1|	Chondroitin sulfate proteoglycan 4 [Harpegnathos saltator]	9,00E-25
gALL_v3_c25263	2	2	-	-	-	-	-
gALL_v3_c25264	3	1	-	-	-	-	-
gALL_v3_c25265	5	6	gnl|CDD|190351	pfam02582, DUF155, Unchara	gb|EGI66426.1|	Required for meiotic nuclear division protein 1-like protein [Acromyrmex echinatior]	6,00E-72
gALL_v3_c25267	1	2	-	-	-	-	-
gALL_v3_c25268	5	2	-	-	-	-	-
gALL_v3_c25269	3	3	gnl|CDD|48179	cd02016, TPP_E1_OGDC_like,	ref|XP_967991.1|	PREDICTED: similar to dehydrogenase E1 and transketolase domain containing 1 [Tribolium castaneum]	1,00E-117
gALL_v3_c2527	1	8	-	-	-	-	-
gALL_v3_c25270	4	3	gnl|CDD|162130	TIGR00955, 3a01204, The Eye	gb|EGI70628.1|	ATP-binding cassette sub-family G member 1 [Acromyrmex echinatior]	1,00E-101

<table>
<tr><td>gALL_v3_c25271</td><td>0</td><td>5</td><td>-</td><td>-</td><td>ref|XP_005896196.1|</td><td>PREDICTED: GPALPP motifs-containing protein 1 [Bos mutus]</td><td>2,00E-21</td></tr>
<tr><td>gALL_v3_c25272</td><td>4</td><td>4</td><td>-</td><td>-</td><td>-</td><td>-</td><td>-</td></tr>
<tr><td>gALL_v3_c25273</td><td>2</td><td>1</td><td>-</td><td>-</td><td>-</td><td>-</td><td>-</td></tr>
<tr><td>gALL_v3_c25274</td><td>3</td><td>1</td><td>-</td><td>-</td><td>gb|EGI69460.1|</td><td>Integrator complex subunit 1 [Acromyrmex echinatior]</td><td>4,00E-45</td></tr>
<tr><td>gALL_v3_c25276</td><td>2</td><td>2</td><td>-</td><td>-</td><td>-</td><td>-</td><td>-</td></tr>
<tr><td>gALL_v3_c25277</td><td>0</td><td>6</td><td>-</td><td>-</td><td>-</td><td>-</td><td>-</td></tr>
<tr><td>gALL_v3_c25278</td><td>7</td><td>9</td><td>-</td><td>-</td><td>-</td><td>-</td><td>-</td></tr>
<tr><td>gALL_v3_c2528</td><td>8</td><td>14</td><td>-</td><td>-</td><td>ref|XP_003247777.1|</td><td>PREDICTED: hypothetical protein LOC100574987 isoform 5 [Acyrthosiphon pisum]</td><td>4,00E-27</td></tr>
<tr><td>gALL_v3_c25280</td><td>0</td><td>3</td><td>-</td><td>-</td><td>-</td><td>-</td><td>-</td></tr>
<tr><td>gALL_v3_c25281</td><td>2</td><td>1</td><td>-</td><td>-</td><td>-</td><td>-</td><td>-</td></tr>
<tr><td>gALL_v3_c25282</td><td>1</td><td>3</td><td>-</td><td>-</td><td>-</td><td>-</td><td>-</td></tr>
<tr><td>gALL_v3_c25283</td><td>5</td><td>4</td><td>-</td><td>-</td><td>-</td><td>-</td><td>-</td></tr>
<tr><td>gALL_v3_c25285</td><td>0</td><td>4</td><td>-</td><td>-</td><td>-</td><td>-</td><td>-</td></tr>
<tr><td>gALL_v3_c25287</td><td>2</td><td>2</td><td>-</td><td>-</td><td>-</td><td>-</td><td>-</td></tr>
<tr><td>gALL_v3_c25288</td><td>3</td><td>6</td><td>-</td><td>-</td><td>-</td><td>-</td><td>-</td></tr>
<tr><td>gALL_v3_c25289</td><td>0</td><td>4</td><td>-</td><td>-</td><td>ref|XP_004926277.1|</td><td>PREDICTED: uncharacterized protein LOC101745229 [Bombyx mori]</td><td>2,00E-10</td></tr>
<tr><td>gALL_v3_c2529</td><td>7</td><td>13</td><td>-</td><td>-</td><td>ref|NP_001259282.1|</td><td>CG34417, isoform T [Drosophila melanogaster] gb|AGB95127.1| CG34417, isoform T [Drosophila melanogaster]</td><td>4,00E-11</td></tr>
<tr><td>gALL_v3_c25290</td><td>2</td><td>1</td><td>gnl|CDD|189968</td><td>pfam01391, Collagen, Collage</td><td>ref|XP_002424552.1|</td><td>collagen alpha-1, IV, chain precursor, putative [Pediculus humanus corporis] gb|EEB11814.1| collagen alpha-1, IV, chain precursor, putative [Pediculus humanus corporis]</td><td>1,00E-13</td></tr>
<tr><td>gALL_v3_c25291</td><td>5</td><td>2</td><td>gnl|CDD|133040</td><td>cd04197, eIF-2B_epsilon_N, Tl</td><td>gb|EFX69078.1|</td><td>hypothetical protein DAPPUDRAFT_62668 [Daphnia pulex]</td><td>1,00E-96</td></tr>
<tr><td>gALL_v3_c25292</td><td>7</td><td>8</td><td>gnl|CDD|189372</td><td>pfam00067, p450, Cytochrom</td><td>gb|AAR97606.1|</td><td>cytochrome P450 9E1 [Diploptera punctata]</td><td>3,00E-36</td></tr>
<tr><td>gALL_v3_c25295</td><td>4</td><td>2</td><td>-</td><td>-</td><td>-</td><td>-</td><td>-</td></tr>
<tr><td>gALL_v3_c25296</td><td>2</td><td>5</td><td>gnl|CDD|29257</td><td>cd00200, WD40, WD40 domai</td><td>ref|XP_001944268.2|</td><td>PREDICTED: mRNA export factor-like [Acyrthosiphon pisum]</td><td>1,00E-101</td></tr>
<tr><td>gALL_v3_c25297</td><td>1</td><td>4</td><td>-</td><td>-</td><td>-</td><td>-</td><td>-</td></tr>
<tr><td>gALL_v3_c25298</td><td>4</td><td>2</td><td>-</td><td>-</td><td>ref|XP_002432605.1|</td><td>basic helix-loop-helix protein mist1, putative [Pediculus humanus corporis] gb|EEB19867.1| basic helix-loop-helix protein mist1, putative [Pediculus humanus corporis]</td><td>2,00E-10</td></tr>
<tr><td>gALL_v3_c25299</td><td>1</td><td>2</td><td>-</td><td>-</td><td>-</td><td>-</td><td>-</td></tr>
<tr><td>gALL_v3_c253</td><td>11</td><td>7</td><td>gnl|CDD|31309</td><td>COG1112, COG1112, Superfan</td><td>ref|XP_003487865.1|</td><td>PREDICTED: regulator of nonsense transcripts 1-like isoform 1 [Bombus impatiens]</td><td>1,00E-123</td></tr>
<tr><td>gALL_v3_c25300</td><td>2</td><td>2</td><td>-</td><td>-</td><td>gb|AGM32299.1|</td><td>serine-arginine protein 55-like protein, partial [Coptotermes formosanus]</td><td>1,00E-16</td></tr>
<tr><td>gALL_v3_c25301</td><td>2</td><td>2</td><td>-</td><td>-</td><td>-</td><td>-</td><td>-</td></tr>
<tr><td>gALL_v3_c25302</td><td>1</td><td>2</td><td>-</td><td>-</td><td>-</td><td>-</td><td>-</td></tr>
<tr><td>gALL_v3_c25303</td><td>1</td><td>3</td><td>-</td><td>-</td><td>-</td><td>-</td><td>-</td></tr>
<tr><td>gALL_v3_c25304</td><td>1</td><td>4</td><td>-</td><td>-</td><td>-</td><td>-</td><td>-</td></tr>
<tr><td>gALL_v3_c25305</td><td>6</td><td>9</td><td>-</td><td>-</td><td>gb|EFN74736.1|</td><td>PiggyBac transposable element-derived protein 4 [Camponotus floridanus]</td><td>1,00E-23</td></tr>
<tr><td>gALL_v3_c25306</td><td>4</td><td>2</td><td>gnl|CDD|73065</td><td>cd02659, peptidase_C19C, A s</td><td>ref|XP_002425902.1|</td><td>ubiquitin specific protease, putative [Pediculus humanus corporis] gb|EEB13164.1| ubiquitin specific protease, putative [Pediculus humanus corporis]</td><td>1,00E-85</td></tr>
<tr><td>gALL_v3_c25307</td><td>1</td><td>4</td><td>-</td><td>-</td><td>-</td><td>-</td><td>-</td></tr>
<tr><td>gALL_v3_c25308</td><td>1</td><td>6</td><td>-</td><td>-</td><td>ref|XP_001948239.2|</td><td>PREDICTED: tyrosine-protein kinase PR2-like [Acyrthosiphon pisum]</td><td>1,00E-36</td></tr>
<tr><td>gALL_v3_c2531</td><td>14</td><td>12</td><td>-</td><td>-</td><td>-</td><td>-</td><td>-</td></tr>
<tr><td>gALL_v3_c25310</td><td>1</td><td>2</td><td>-</td><td>-</td><td>-</td><td>-</td><td>-</td></tr>
<tr><td>gALL_v3_c25312</td><td>3</td><td>2</td><td>-</td><td>-</td><td>-</td><td>-</td><td>-</td></tr>
<tr><td>gALL_v3_c25313</td><td>0</td><td>3</td><td>gnl|CDD|191127</td><td>pfam04921, XAP5, XAP5 prote</td><td>ref|XP_002426155.1|</td><td>protein FAM50A, putative [Pediculus humanus corporis] gb|EEB13417.1| protein FAM50A, putative [Pediculus humanus corporis]</td><td>3,00E-73</td></tr>
<tr><td>gALL_v3_c25314</td><td>2</td><td>2</td><td>-</td><td>-</td><td>-</td><td>-</td><td>-</td></tr>
<tr><td>gALL_v3_c25315</td><td>0</td><td>3</td><td>-</td><td>-</td><td>-</td><td>-</td><td>-</td></tr>
<tr><td>gALL_v3_c25316</td><td>2</td><td>6</td><td>gnl|CDD|30962</td><td>COG0617, PcnB, tRNA nucleot</td><td>gb|AGM32735.1|</td><td>putative tRNA nucleotidyltransferase/poly(A) polymerase, partial [Coptotermes formosanus]</td><td>1,00E-139</td></tr>
<tr><td>gALL_v3_c25317</td><td>2</td><td>2</td><td>-</td><td>-</td><td>-</td><td>-</td><td>-</td></tr>
<tr><td>gALL_v3_c25318</td><td>0</td><td>2</td><td>-</td><td>-</td><td>-</td><td>-</td><td>-</td></tr>
<tr><td>gALL_v3_c25319</td><td>2</td><td>2</td><td>-</td><td>-</td><td>ref|XP_004930809.1|</td><td>PREDICTED: vacuolar protein sorting-associated protein 13A-like [Bombyx mori]</td><td>1,00E-50</td></tr>
<tr><td>gALL_v3_c2532</td><td>6</td><td>5</td><td>-</td><td>-</td><td>-</td><td>-</td><td>-</td></tr>
<tr><td>gALL_v3_c25320</td><td>1</td><td>3</td><td>-</td><td>-</td><td>gb|EFN83272.1|</td><td>Titin [Harpegnathos saltator]</td><td>9,00E-16</td></tr>
<tr><td>gALL_v3_c25321</td><td>2</td><td>1</td><td>-</td><td>-</td><td>-</td><td>-</td><td>-</td></tr>
<tr><td>gALL_v3_c25322</td><td>2</td><td>2</td><td>-</td><td>-</td><td>-</td><td>-</td><td>-</td></tr>
<tr><td>gALL_v3_c25323</td><td>3</td><td>1</td><td>-</td><td>-</td><td>-</td><td>-</td><td>-</td></tr>
<tr><td>gALL_v3_c25324</td><td>1</td><td>4</td><td>-</td><td>-</td><td>gb|EFN80520.1|</td><td>Multiple epidermal growth factor-like domains 8 [Harpegnathos saltator]</td><td>1,00E-113</td></tr>
<tr><td>gALL_v3_c25325</td><td>2</td><td>4</td><td>-</td><td>-</td><td>ref|XP_005188022.1|</td><td>PREDICTED: uncharacterized protein LOC101899879 isoform X1 [Musca domestica] ref|XP_005188023.1| PREDICTED: uncharacterized protein LOC101899879 isoform X2 [</td><td>1,00E-31</td></tr>
<tr><td>gALL_v3_c25326</td><td>0</td><td>3</td><td>gnl|CDD|163712</td><td>cd08156, catalase_clade_3, Cl</td><td>gb|AEV89764.1|</td><td>catalase, partial [Schistocerca gregaria]</td><td>9,00E-58</td></tr>
<tr><td>gALL_v3_c25328</td><td>1</td><td>2</td><td>-</td><td>-</td><td>-</td><td>-</td><td>-</td></tr>
<tr><td>gALL_v3_c2533</td><td>10</td><td>7</td><td>gnl|CDD|191856</td><td>pfam07807, RED_C, RED-like p</td><td>gb|EFN79871.1|</td><td>Protein Red [Harpegnathos saltator]</td><td>7,00E-60</td></tr>
<tr><td>gALL_v3_c25330</td><td>1</td><td>5</td><td>-</td><td>-</td><td>-</td><td>-</td><td>-</td></tr>
<tr><td>gALL_v3_c25331</td><td>2</td><td>4</td><td>gnl|CDD|191797</td><td>pfam07647, SAM_2, SAM dor</td><td>ref|XP_003494056.1|</td><td>PREDICTED: hypothetical protein LOC100743083 [Bombus impatiens]</td><td>1,00E-67</td></tr>
<tr><td>gALL_v3_c25332</td><td>3</td><td>3</td><td>gnl|CDD|162451</td><td>TIGR01622, SF-CC1, splicing fa</td><td>gb|EFN68744.1|</td><td>Probable RNA-binding protein 19 [Camponotus floridanus]</td><td>1,00E-61</td></tr>
<tr><td>gALL_v3_c25333</td><td>2</td><td>1</td><td>-</td><td>-</td><td>-</td><td>-</td><td>-</td></tr>
<tr><td>gALL_v3_c25334</td><td>0</td><td>5</td><td>-</td><td>-</td><td>-</td><td>-</td><td>-</td></tr>
<tr><td>gALL_v3_c25335</td><td>1</td><td>3</td><td>-</td><td>-</td><td>-</td><td>-</td><td>-</td></tr>
<tr><td>gALL_v3_c25336</td><td>2</td><td>2</td><td>gnl|CDD|189583</td><td>pfam00520, Ion_trans, Ion tra</td><td>dbj|BAA22091.1|</td><td>voltage-dependent calcium channel alpha-1 subunit [Blattella germanica]</td><td>9,00E-44</td></tr>
<tr><td>gALL_v3_c25337</td><td>2</td><td>3</td><td>-</td><td>-</td><td>-</td><td>-</td><td>-</td></tr>
<tr><td>gALL_v3_c25338</td><td>1</td><td>4</td><td>-</td><td>-</td><td>-</td><td>-</td><td>-</td></tr>
<tr><td>gALL_v3_c25339</td><td>0</td><td>3</td><td>-</td><td>-</td><td>-</td><td>-</td><td>-</td></tr>
<tr><td>gALL_v3_c25340</td><td>0</td><td>4</td><td>gnl|CDD|130360</td><td>TIGR01293, Kv_beta, voltage-c</td><td>ref|XP_002430766.1|</td><td>predicted protein [Pediculus humanus corporis] gb|EEB18028.1| predicted protein [Pediculus humanus corporis]</td><td>6,00E-71</td></tr>
<tr><td>gALL_v3_c25342</td><td>2</td><td>1</td><td>-</td><td>-</td><td>-</td><td>-</td><td>-</td></tr>
<tr><td>gALL_v3_c25343</td><td>2</td><td>1</td><td>-</td><td>-</td><td>-</td><td>-</td><td>-</td></tr>
<tr><td>gALL_v3_c25344</td><td>1</td><td>3</td><td>-</td><td>-</td><td>-</td><td>-</td><td>-</td></tr>
<tr><td>gALL_v3_c25345</td><td>0</td><td>3</td><td>-</td><td>-</td><td>ref|XP_005103941.1|</td><td>PREDICTED: piggyBac transposable element-derived protein 4-like [Aplysia californica]</td><td>3,00E-27</td></tr>
<tr><td>gALL_v3_c25346</td><td>0</td><td>5</td><td>-</td><td>-</td><td>ref|XP_971508.2|</td><td>PREDICTED: similar to MYC binding protein 2 [Tribolium castaneum]</td><td>2,00E-50</td></tr>
<tr><td>gALL_v3_c25347</td><td>1</td><td>3</td><td>-</td><td>-</td><td>-</td><td>-</td><td>-</td></tr>
<tr><td>gALL_v3_c25350</td><td>1</td><td>3</td><td>-</td><td>-</td><td>-</td><td>-</td><td>-</td></tr>
<tr><td>gALL_v3_c25351</td><td>1</td><td>4</td><td>-</td><td>-</td><td>ref|XP_001620907.1|</td><td>hypothetical protein NEMVEDRAFT_v1g248744 [Nematostella vectensis] gb|EDO28807.1| predicted protein [Nematostella vectensis]</td><td>7,00E-45</td></tr>
<tr><td>gALL_v3_c25352</td><td>0</td><td>5</td><td>gnl|CDD|128436</td><td>smart00131, KU, BPTI/Kunitz f</td><td>ref|XP_002431788.1|</td><td>papilin, putative [Pediculus humanus corporis] gb|EEB19050.1| papilin, putative [Pediculus humanus corporis]</td><td>4,00E-55</td></tr>
<tr><td>gALL_v3_c25353</td><td>3</td><td>2</td><td>-</td><td>-</td><td>ref|XP_003244015.1|</td><td>PREDICTED: hemicentin-1-like isoform 2 [Acyrthosiphon pisum]</td><td>8,00E-11</td></tr>
<tr><td>gALL_v3_c25354</td><td>1</td><td>3</td><td>-</td><td>-</td><td>-</td><td>-</td><td>-</td></tr>
<tr><td>gALL_v3_c25355</td><td>1</td><td>3</td><td>-</td><td>-</td><td>gb|EHJ76589.1|</td><td>putative leucine rich protein [Danaus plexippus]</td><td>2,00E-34</td></tr>
<tr><td>gALL_v3_c25356</td><td>2</td><td>4</td><td>-</td><td>-</td><td>ref|XP_003702958.1|</td><td>PREDICTED: transformation/transcription domain-associated protein isoform 1 [Megachile rotundata]</td><td>5,00E-62</td></tr>
<tr><td>gALL_v3_c25357</td><td>2</td><td>2</td><td>-</td><td>-</td><td>-</td><td>-</td><td>-</td></tr>
<tr><td>gALL_v3_c25358</td><td>1</td><td>5</td><td>-</td><td>-</td><td>-</td><td>-</td><td>-</td></tr>
<tr><td>gALL_v3_c2536</td><td>5</td><td>2</td><td>-</td><td>-</td><td>gb|EFA11568.1|</td><td>pyrokinin [Tribolium castaneum]</td><td>3,00E-15</td></tr>
<tr><td>gALL_v3_c25360</td><td>2</td><td>3</td><td>-</td><td>-</td><td>-</td><td>-</td><td>-</td></tr>
<tr><td>gALL_v3_c25361</td><td>2</td><td>2</td><td>gnl|CDD|189855</td><td>pfam01139, UPF0027, Unchar</td><td>ref|XP_002409445.1|</td><td>hypothetical protein IscW_ISCW024552 [Ixodes scapularis] gb|EEC12657.1| hypothetical protein IscW_ISCW024552 [Ixodes scapularis]</td><td>2,00E-67</td></tr>
<tr><td>gALL_v3_c25362</td><td>1</td><td>2</td><td>-</td><td>-</td><td>-</td><td>-</td><td>-</td></tr>
<tr><td>gALL_v3_c25364</td><td>1</td><td>3</td><td>-</td><td>-</td><td>-</td><td>-</td><td>-</td></tr>
<tr><td>gALL_v3_c25365</td><td>0</td><td>4</td><td>gnl|CDD|189784</td><td>pfam00957, Synaptobrevin, Sy</td><td>ref|XP_003427457.1|</td><td>PREDICTED: synaptobrevin-like isoform 2 [Nasonia vitripennis] ref|XP_003427458.1| PREDICTED: synaptobrevin-like isoform 3 [Nasonia vitripennis]</td><td>5,00E-36</td></tr>
<tr><td>gALL_v3_c25366</td><td>2</td><td>4</td><td>-</td><td>-</td><td>-</td><td>-</td><td>-</td></tr>
<tr><td>gALL_v3_c25367</td><td>4</td><td>3</td><td>-</td><td>-</td><td>ref|XP_003487224.1|</td><td>PREDICTED: hypothetical protein LOC100748921 isoform 1 [Bombus impatiens]</td><td>1,00E-21</td></tr>
<tr><td>gALL_v3_c25368</td><td>4</td><td>2</td><td>-</td><td>-</td><td>ref|XP_004932301.1|</td><td>PREDICTED: uncharacterized protein LOC101736992 [Bombyx mori]</td><td>1,00E-06</td></tr>
<tr><td>gALL_v3_c25369</td><td>11</td><td>7</td><td>gnl|CDD|118307</td><td>pfam09775, Keratin_assoc, Ke</td><td>gb|ELU17885.1|</td><td>hypothetical protein CAPTEDRAFT_220593 [Capitella teleta]</td><td>4,00E-16</td></tr>
<tr><td>gALL_v3_c2537</td><td>5</td><td>4</td><td>-</td><td>-</td><td>-</td><td>-</td><td>-</td></tr>
<tr><td>gALL_v3_c25370</td><td>4</td><td>4</td><td>-</td><td>-</td><td>-</td><td>-</td><td>-</td></tr>
<tr><td>gALL_v3_c25371</td><td>0</td><td>3</td><td>-</td><td>-</td><td>-</td><td>-</td><td>-</td></tr>
<tr><td>gALL_v3_c25372</td><td>1</td><td>3</td><td>-</td><td>-</td><td>-</td><td>-</td><td>-</td></tr>
<tr><td>gALL_v3_c25373</td><td>2</td><td>2</td><td>-</td><td>-</td><td>-</td><td>-</td><td>-</td></tr>
<tr><td>gALL_v3_c25374</td><td>1</td><td>3</td><td>-</td><td>-</td><td>gb|EFN86775.1|</td><td>hypothetical protein EAI_02636 [Harpegnathos saltator]</td><td>9,00E-07</td></tr>
<tr><td>gALL_v3_c25375</td><td>2</td><td>2</td><td>gnl|CDD|175974</td><td>cd00275, C2_PLC_like, C2 dor</td><td>ref|XP_001813325.1|</td><td>PREDICTED: similar to phospholipase C [Tribolium castaneum]</td><td>1,00E-53</td></tr>
<tr><td>gALL_v3_c25378</td><td>5</td><td>2</td><td>-</td><td>-</td><td>gb|EFA09018.1|</td><td>hypothetical protein TcasGA2_TC006727 [Tribolium castaneum]</td><td>2,00E-18</td></tr>
<tr><td>gALL_v3_c25379</td><td>0</td><td>3</td><td>-</td><td>-</td><td>-</td><td>-</td><td>-</td></tr>
<tr><td>gALL_v3_c2538</td><td>4</td><td>12</td><td>gnl|CDD|150427</td><td>pfam09752, DUF2048, Unchar</td><td>gb|EGI64879.1|</td><td>Uncharacterized protein C4orf29-like protein [Acromyrmex echinatior]</td><td>2,00E-72</td></tr>
<tr><td>gALL_v3_c25381</td><td>1</td><td>3</td><td>gnl|CDD|143220</td><td>cd05743, Ig_Perlecan_D2_like</td><td>ref|XP_003702533.1|</td><td>PREDICTED: LOW QUALITY PROTEIN: basement membrane-specific heparan sulfate proteoglycan core protein-like [Megachile rotundata]</td><td>4,00E-59</td></tr>
<tr><td>gALL_v3_c25382</td><td>2</td><td>7</td><td>gnl|CDD|35206</td><td>COG5647, COG5647, Cullin, a</td><td>dbj|BAM18767.1|</td><td>cullin-3 [Papilio xuthus]</td><td>1,00E-66</td></tr>
<tr><td>gALL_v3_c25383</td><td>0</td><td>3</td><td>-</td><td>-</td><td>-</td><td>-</td><td>-</td></tr>
<tr><td>gALL_v3_c25384</td><td>1</td><td>2</td><td>-</td><td>-</td><td>-</td><td>-</td><td>-</td></tr>
<tr><td>gALL_v3_c25385</td><td>0</td><td>3</td><td>-</td><td>-</td><td>-</td><td>-</td><td>-</td></tr>
<tr><td>gALL_v3_c25386</td><td>0</td><td>4</td><td>-</td><td>-</td><td>ref|XP_002430432.1|</td><td>conserved hypothetical protein [Pediculus humanus corporis] gb|EEB17694.1| conserved hypothetical protein [Pediculus humanus corporis]</td><td>3,00E-17</td></tr>
<tr><td>gALL_v3_c25387</td><td>0</td><td>3</td><td>-</td><td>-</td><td>-</td><td>-</td><td>-</td></tr>
<tr><td>gALL_v3_c25388</td><td>2</td><td>2</td><td>-</td><td>-</td><td>-</td><td>-</td><td>-</td></tr>
<tr><td>gALL_v3_c25389</td><td>2</td><td>3</td><td>gnl|CDD|162739</td><td>TIGR02168, SMC_prok_B, chro</td><td>ref|XP_002431483.1|</td><td>conserved hypothetical protein [Pediculus humanus corporis] gb|EEB18745.1| conserved hypothetical protein [Pediculus humanus corporis]</td><td>1,00E-41</td></tr>
<tr><td>gALL_v3_c2539</td><td>4</td><td>7</td><td>-</td><td>-</td><td>ref|XP_002424660.1|</td><td>tens, putative [Pediculus humanus corporis] gb|EEB11922.1| tens, putative [Pediculus humanus corporis]</td><td>8,00E-27</td></tr>
<tr><td>gALL_v3_c25390</td><td>2</td><td>1</td><td>-</td><td>-</td><td>-</td><td>-</td><td>-</td></tr>
<tr><td>gALL_v3_c25391</td><td>1</td><td>3</td><td>gnl|CDD|109266</td><td>pfam00201, UDPGT, UDP-gluc</td><td>ref|XP_003731177.1|</td><td>PREDICTED: UDP-glucuronosyltransferase 2B33-like [Strongylocentrotus purpuratus]</td><td>9,00E-26</td></tr>
<tr><td>gALL_v3_c25392</td><td>1</td><td>2</td><td>gnl|CDD|28913</td><td>cd00031, CA, Cadherin repeat</td><td>dbj|BAH36923.1|</td><td>fat tumor suppressor [Gryllus bimaculatus]</td><td>1,00E-80</td></tr>
<tr><td>gALL_v3_c25393</td><td>2</td><td>1</td><td>-</td><td>-</td><td>-</td><td>-</td><td>-</td></tr>
<tr><td>gALL_v3_c25394</td><td>1</td><td>3</td><td>-</td><td>-</td><td>-</td><td>-</td><td>-</td></tr>
<tr><td>gALL_v3_c25395</td><td>2</td><td>3</td><td>-</td><td>-</td><td>ref|XP_972562.1|</td><td>PREDICTED: similar to AGAP006992-PA [Tribolium castaneum] gb|EFA13400.1| hypothetical protein TcasGA2_TC015971 [Tribolium castaneum]</td><td>5,00E-61</td></tr>
<tr><td>gALL_v3_c25396</td><td>2</td><td>2</td><td>-</td><td>-</td><td>-</td><td>-</td><td>-</td></tr>
<tr><td>gALL_v3_c25397</td><td>5</td><td>2</td><td>-</td><td>-</td><td>gb|AGH30328.1|</td><td>argonaute-3 [Nilaparvata lugens]</td><td>7,00E-06</td></tr>
<tr><td>gALL_v3_c25398</td><td>2</td><td>2</td><td>-</td><td>-</td><td>-</td><td>-</td><td>-</td></tr>
<tr><td>gALL_v3_c25399</td><td>0</td><td>4</td><td>-</td><td>-</td><td>-</td><td>-</td><td>-</td></tr>
<tr><td>gALL_v3_c254</td><td>7</td><td>6</td><td>-</td><td>-</td><td>-</td><td>-</td><td>-</td></tr>
<tr><td>gALL_v3_c2540</td><td>25</td><td>18</td><td>-</td><td>-</td><td>-</td><td>-</td><td>-</td></tr>
<tr><td>gALL_v3_c25401</td><td>1</td><td>2</td><td>-</td><td>-</td><td>-</td><td>-</td><td>-</td></tr>
<tr><td>gALL_v3_c25402</td><td>3</td><td>4</td><td>-</td><td>-</td><td>-</td><td>-</td><td>-</td></tr>
<tr><td>gALL_v3_c25403</td><td>4</td><td>2</td><td>-</td><td>-</td><td>-</td><td>-</td><td>-</td></tr>
<tr><td>gALL_v3_c25404</td><td>2</td><td>1</td><td>-</td><td>-</td><td>-</td><td>-</td><td>-</td></tr>
<tr><td>gALL_v3_c25406</td><td>5</td><td>4</td><td>-</td><td>-</td><td>-</td><td>-</td><td>-</td></tr>
<tr><td>gALL_v3_c25407</td><td>2</td><td>1</td><td>-</td><td>-</td><td>-</td><td>-</td><td>-</td></tr>
<tr><td>gALL_v3_c25408</td><td>2</td><td>5</td><td>gnl|CDD|189496</td><td>pfam00307, CH, Calponin hon</td><td>ref|XP_002424941.1|</td><td>protein MLP1, putative [Pediculus humanus corporis] gb|EEB12203.1| protein MLP1, putative [Pediculus humanus corporis]</td><td>8,00E-72</td></tr>
<tr><td>gALL_v3_c25409</td><td>1</td><td>4</td><td>-</td><td>-</td><td>-</td><td>-</td><td>-</td></tr>
<tr><td>gALL_v3_c2541</td><td>6</td><td>5</td><td>-</td><td>-</td><td>-</td><td>-</td><td>-</td></tr>
<tr><td>gALL_v3_c25410</td><td>1</td><td>3</td><td>-</td><td>-</td><td>-</td><td>-</td><td>-</td></tr>
<tr><td>gALL_v3_c25411</td><td>3</td><td>2</td><td>gnl|CDD|129984</td><td>TIGR00906, 2A0303, cationic a</td><td>gb|ENN76165.1|</td><td>hypothetical protein YQE_07335, partial [Dendroctonus ponderosae] gb|ERL86806.1| hypothetical protein D910_04210 [Dendroctonus ponderosae]</td><td>4,00E-57</td></tr>
<tr><td>gALL_v3_c25412</td><td>1</td><td>3</td><td>-</td><td>-</td><td>ref|XP_003700052.1|</td><td>PREDICTED: uncharacterized protein LOC100880102 [Megachile rotundata]</td><td>5,00E-19</td></tr>
<tr><td>gALL_v3_c25413</td><td>1</td><td>3</td><td>-</td><td>-</td><td>-</td><td>-</td><td>-</td></tr>
<tr><td>gALL_v3_c25414</td><td>1</td><td>4</td><td>-</td><td>-</td><td>-</td><td>-</td><td>-</td></tr>
<tr><td>gALL_v3_c25415</td><td>1</td><td>5</td><td>-</td><td>-</td><td>-</td><td>-</td><td>-</td></tr>
<tr><td>gALL_v3_c25416</td><td>1</td><td>5</td><td>-</td><td>-</td><td>-</td><td>-</td><td>-</td></tr>
<tr><td>gALL_v3_c25417</td><td>1</td><td>3</td><td>-</td><td>-</td><td>-</td><td>-</td><td>-</td></tr>
<tr><td>gALL_v3_c25418</td><td>0</td><td>4</td><td>-</td><td>-</td><td>ref|XP_003428150.1|</td><td>PREDICTED: origin recognition complex subunit 1-like isoform 2 [Nasonia vitripennis] ref|XP_001604978.2| PREDICTED: origin recognition complex subunit 1-like isoform 1</td><td>3,00E-27</td></tr>
<tr><td>gALL_v3_c25419</td><td>0</td><td>3</td><td>-</td><td>-</td><td>-</td><td>-</td><td>-</td></tr>
<tr><td>gALL_v3_c2542</td><td>5</td><td>5</td><td>-</td><td>-</td><td>ref|XP_004929857.1|</td><td>PREDICTED: slit homolog 3 protein-like [Bombyx mori]</td><td>8,00E-59</td></tr>
<tr><td>gALL_v3_c25420</td><td>2</td><td>2</td><td>-</td><td>-</td><td>-</td><td>-</td><td>-</td></tr>
</table>

gALL_v3_c25421	2	2	-	-	-	-	-
gALL_v3_c25422	2	1	-	-	gb|EMC77429.1|	hypothetical protein A306_15251 [Columba livia]	8,00E-09
gALL_v3_c25423	3	1	-	-	-	-	-
gALL_v3_c25424	2	6	gnl|CDD|192041	pfam08449, UAA, UAA transpo	ref|XP_006621171.1|	PREDICTED: adenosine 3'-phospho 5'-phosphosulfate transporter 1-like [Apis dorsata]	7,00E-20
gALL_v3_c25426	3	2	-	-	ref|XP_006618301.1|	PREDICTED: uncharacterized protein LOC102674398 [Apis dorsata]	7,00E-06
gALL_v3_c25428	1	4	-	-	-	-	-
gALL_v3_c25429	5	4	-	-	gb|EFA09839.1|	hypothetical protein TcasGA2_TC011986 [Tribolium castaneum]	0
gALL_v3_c2543	10	11	-	-	-	-	-
gALL_v3_c25430	3	6	gnl|CDD|150790	pfam10160, Tmemb_40, Predi	ref|XP_006567508.1|	PREDICTED: transmembrane protein adipocyte-associated 1 homolog isoform X2 [Apis mellifera]	1,00E-26
gALL_v3_c25431	2	2	-	-	-	-	-
gALL_v3_c25432	2	2	-	-	gb|ADI61823.1|	endonuclease-reverse transcriptase [Bombyx mori]	1,00E-15
gALL_v3_c25433	2	3	gnl|CDD|128336	smart00020, Tryp_SPc, Trypsir	ref|XP_002430308.1|	transmembrane serine protease 9, putative [Pediculus humanus corporis] gb|EEB17570.1| transmembrane serine protease 9, putative [Pediculus humanus corporis]	2,00E-36
gALL_v3_c25434	4	2	gnl|CDD|146480	pfam03870, RNA_pol_Rpb8, R	gb|AEE63018.1|	unknown [Dendroctonus ponderosae]	9,00E-34
gALL_v3_c25435	1	5	-	-	ref|XP_002429107.1|	conserved hypothetical protein [Pediculus humanus corporis] gb|EEB16369.1| conserved hypothetical protein [Pediculus humanus corporis]	2,00E-65
gALL_v3_c25436	5	7	-	-	-	-	-
gALL_v3_c25438	3	1	-	-	ref|XP_002432753.1|	conserved hypothetical protein [Pediculus humanus corporis] gb|EEB20015.1| conserved hypothetical protein [Pediculus humanus corporis]	2,00E-36
gALL_v3_c25439	1	3	-	-	-	-	-
gALL_v3_c2544	7	14	gnl|CDD|188098	TIGR00957, MRP_assoc_pro, r	ref|XP_003488605.1|	PREDICTED: multidrug resistance-associated protein 1-like isoform 2 [Bombus impatiens]	0
gALL_v3_c25440	2	1	gnl|CDD|190189	pfam02008, zf-CXXC, CXXC zin	ref|XP_002427386.1|	JmjC domain-containing histone demethylation protein 1B, putative [Pediculus humanus corporis] gb|EEB14648.1| JmjC domain-containing histone demethylation protein	2,00E-65
gALL_v3_c25441	2	3	-	-	-	-	-
gALL_v3_c25442	0	3	-	-	-	-	-
gALL_v3_c25444	2	2	-	-	-	-	-
gALL_v3_c25446	4	3	-	-	-	-	-
gALL_v3_c25447	0	2	-	-	-	-	-
gALL_v3_c25448	1	3	-	-	-	-	-
gALL_v3_c25449	0	4	-	-	emb|CAR82249.1|	hypothetical protein [Cotesia congregata]	6,00E-50
gALL_v3_c25450	0	3	gnl|CDD|162469	TIGR01657, P-ATPase-V, P-type	gb|EFN68541.1|	Probable cation-transporting ATPase 13A3 [Camponotus floridanus]	1,00E-49
gALL_v3_c25451	2	2	-	-	-	-	-
gALL_v3_c25452	1	3	-	-	-	-	-
gALL_v3_c25453	1	2	-	-	-	-	-
gALL_v3_c25455	1	2	-	-	-	-	-
gALL_v3_c25456	2	1	-	-	-	-	-
gALL_v3_c25457	2	1	-	-	-	-	-
gALL_v3_c25458	1	4	gnl|CDD|191778	pfam07540, NOC3p, Nucleolar	gb|EFN69036.1|	Nucleolar complex protein 3-like protein [Camponotus floridanus]	1,00E-32
gALL_v3_c25459	1	2	-	-	-	-	-
gALL_v3_c2546	7	11	-	-	-	-	-
gALL_v3_c25460	2	3	-	-	gb|EFX66207.1|	hypothetical protein DAPPUDRAFT_332425 [Daphnia pulex]	1,00E-25
gALL_v3_c25461	1	4	-	-	-	-	-
gALL_v3_c25462	1	3	-	-	ref|XP_006083021.1|	PREDICTED: piggyBac transposable element-derived protein 3-like [Myotis lucifugus]	1,00E-14
gALL_v3_c25463	3	2	-	-	ref|XP_006623801.1|	PREDICTED: exocyst complex component 3-like [Apis dorsata]	4,00E-76
gALL_v3_c25464	3	1	-	-	-	-	-
gALL_v3_c25465	0	4	-	-	-	-	-
gALL_v3_c25466	1	3	-	-	-	-	-
gALL_v3_c25467	3	2	-	-	-	-	-
gALL_v3_c25468	4	2	-	-	-	-	-
gALL_v3_c25469	1	5	-	-	gb|EFN80767.1|	Uncharacterized protein KIAA1467-like protein [Harpegnathos saltator]	2,00E-15
gALL_v3_c2547	16	28	gnl|CDD|179088	PRK00696, sucC, succinyl-CoA	ref|XP_970725.1|	PREDICTED: similar to AGAP004744-PA [Tribolium castaneum]	3,00E-63
gALL_v3_c25470	2	2	-	-	-	-	-
gALL_v3_c25471	4	2	-	-	-	-	-
gALL_v3_c25472	1	4	-	-	-	-	-
gALL_v3_c25474	2	3	-	-	-	-	-
gALL_v3_c25475	2	1	-	-	-	-	-
gALL_v3_c25476	2	3	-	-	-	-	-
gALL_v3_c25477	2	2	-	-	-	-	-
gALL_v3_c25478	3	1	-	-	-	-	-
gALL_v3_c25480	3	1	-	-	-	-	-
gALL_v3_c25481	4	2	-	-	-	-	-
gALL_v3_c25482	3	1	-	-	-	-	-
gALL_v3_c25483	3	2	-	-	-	-	-
gALL_v3_c25484	1	1	-	-	-	-	-
gALL_v3_c25485	1	5	-	-	ref|XP_001846181.1|	UDP-galactose transporter [Culex quinquefasciatus] gb|EDS42877.1| UDP-galactose transporter [Culex quinquefasciatus]	3,00E-14
gALL_v3_c25486	3	3	gnl|CDD|114603	pfam05887, Trypan_PARP, Pro	-	-	-
gALL_v3_c25487	6	9	-	-	gb|EFN82224.1|	Probable 26S proteasome non-ATPase regulatory subunit 3 [Harpegnathos saltator]	5,00E-48
gALL_v3_c25488	1	2	-	-	-	-	-
gALL_v3_c25489	1	2	-	-	-	-	-
gALL_v3_c2549	12	9	gnl|CDD|133350	cd04150, Arf1_5_like, Arf1-Arl	ref|XP_001845067.1|	conserved hypothetical protein [Culex quinquefasciatus] gb|EDS39083.1| conserved hypothetical protein [Culex quinquefasciatus]	1,00E-116
gALL_v3_c25490	0	4	-	-	-	-	-
gALL_v3_c25491	2	2	-	-	-	-	-
gALL_v3_c25492	1	5	-	-	ref|XP_003241546.1|	PREDICTED: myosin-IXb isoform 2 [Acyrthosiphon pisum]	1,00E-74
gALL_v3_c25493	0	3	-	-	-	-	-
gALL_v3_c25494	3	2	-	-	gb|ENN71444.1|	hypothetical protein YQE_11863, partial [Dendroctonus ponderosae]	2,00E-42
gALL_v3_c25495	2	2	-	-	ref|XP_003708563.1|	PREDICTED: vacuolar protein sorting-associated protein 13B-like isoform 2 [Megachile rotundata]	8,00E-25
gALL_v3_c25496	3	1	-	-	ref|XP_003427073.1|	PREDICTED: transcription factor RFX3-like isoform 2 [Nasonia vitripennis]	4,00E-91
gALL_v3_c25498	4	7	-	-	-	-	-
gALL_v3_c25499	0	3	-	-	-	-	-
gALL_v3_c255	16	10	gnl|CDD|28845	cd02905, Macro_GDAP2_like,	ref|XP_001815509.1|	PREDICTED: similar to AGAP005091-PB [Tribolium castaneum]	0
gALL_v3_c2550	5	8	gnl|CDD|31389	COG1196, Smc, Chromosome	ref|XP_003486415.1|	PREDICTED: hypothetical protein LOC100742250 [Bombus impatiens]	1,00E-08
gALL_v3_c25500	3	6	-	-	gb|ADI61823.1|	endonuclease-reverse transcriptase [Bombyx mori]	9,00E-33
gALL_v3_c25501	2	1	-	-	-	-	-
gALL_v3_c25502	1	2	-	-	-	-	-
gALL_v3_c25503	4	5	gnl|CDD|34887	COG5296, COG5296, Transcrip	ref|XP_003701503.1|	PREDICTED: RNA polymerase-associated protein Rtf1-like [Megachile rotundata]	1,00E-142
gALL_v3_c25505	2	3	-	-	-	-	-
gALL_v3_c25506	2	2	gnl|CDD|192023	pfam08393, DHC_N2, Dynein I	ref|XP_001945551.2|	PREDICTED: dynein heavy chain 3, axonemal-like [Acyrthosiphon pisum]	3,00E-17
gALL_v3_c25507	4	2	-	-	-	-	-
gALL_v3_c25508	2	1	gnl|CDD|185741	cd09000, GH43_XYL_1, Glycos	dbj|GAE83498.1|	beta-xylosidase [Bacteroides reticulotermitis JCM 10512]	2,00E-51
gALL_v3_c25509	2	2	gnl|CDD|119392	cd06174, MFS, The Major Faci	ref|XP_002427227.1|	conserved hypothetical protein [Pediculus humanus corporis] gb|EEB14489.1| conserved hypothetical protein [Pediculus humanus corporis]	3,00E-40
gALL_v3_c25510	1	2	-	-	-	-	-
gALL_v3_c25511	1	2	-	-	ref|XP_002429582.1|	RalBP1-associated Eps domain-containing protein, putative [Pediculus humanus corporis] gb|EEB16844.1| RalBP1-associated Eps domain-containing protein, putative [Ped	3,00E-18
gALL_v3_c25512	4	3	-	-	-	-	-
gALL_v3_c25514	1	5	-	-	ref|XP_003702476.1|	PREDICTED: uncharacterized protein LOC100883693 [Megachile rotundata]	6,00E-27
gALL_v3_c25515	1	4	-	-	ref|XP_003247581.1|	PREDICTED: zinc finger protein 233-like [Acyrthosiphon pisum]	3,00E-15
gALL_v3_c25516	0	4	-	-	-	-	-
gALL_v3_c25517	3	1	-	-	-	-	-
gALL_v3_c25518	3	1	-	-	-	-	-
gALL_v3_c25519	2	2	-	-	-	-	-
gALL_v3_c2552	9	7	-	-	-	-	-
gALL_v3_c25520	3	3	-	-	ref|XP_002430433.1|	nuclear pore complex protein nup214, putative [Pediculus humanus corporis] gb|EEB17695.1| nuclear pore complex protein nup214, putative [Pediculus humanus corpori	6,00E-35
gALL_v3_c25521	0	4	-	-	-	-	-
gALL_v3_c25522	0	4	-	-	-	-	-
gALL_v3_c25523	4	2	-	-	-	-	-
gALL_v3_c25524	2	1	-	-	ref|XP_005746149.1|	PREDICTED: intraflagellar transport protein 81 homolog isoform X3 [Pundamilia nyererei] ref|XP_005940604.1| PREDICTED: intraflagellar transport protein 81 homolog iso	2,00E-15
gALL_v3_c25525	2	2	-	-	-	-	-
gALL_v3_c25527	1	6	-	-	ref|XP_003893844.1|	PREDICTED: uncharacterized protein C1orf96 homolog [Papio anubis]	3,00E-09
gALL_v3_c25528	0	5	-	-	-	-	-
gALL_v3_c25529	6	9	-	-	gb|AGM32476.1|	RAB geranylgeranyl transferase alpha like subunit [Coptotermes formosanus]	1,00E-154
gALL_v3_c2553	6	17	-	-	-	-	-
gALL_v3_c25530	2	1	-	-	-	-	-
gALL_v3_c25531	1	3	-	-	-	-	-
gALL_v3_c25532	2	2	-	-	-	-	-
gALL_v3_c25533	1	3	-	-	-	-	-
gALL_v3_c25534	3	3	gnl|CDD|173724	cd06606, STKc_MAPKKK, Catal	ref|XP_001599239.2|	PREDICTED: eukaryotic translation initiation factor 2-alpha kinase 4-like [Nasonia vitripennis]	3,00E-50
gALL_v3_c25535	4	2	-	-	-	-	-
gALL_v3_c25536	3	3	-	-	-	-	-
gALL_v3_c25537	0	4	-	-	-	-	-
gALL_v3_c25538	2	2	-	-	ref|XP_006034374.1|	PREDICTED: tigger transposable element-derived protein 1-like [Alligator sinensis]	3,00E-11
gALL_v3_c25539	1	5	gnl|CDD|190261	pfam02257, RFX_DNA_binding	gb|EFN78821.1|	Regulatory factor X domain-containing protein 2 [Harpegnathos saltator]	1,00E-84
gALL_v3_c2554	7	10	gnl|CDD|48211	cd03399, Band_7_flotillin, Bar	dbj|BAN20851.1|	flotillin-1 [Riptortus pedestris]	1,00E-131
gALL_v3_c25540	2	1	-	-	-	-	-
gALL_v3_c25541	0	4	-	-	-	-	-
gALL_v3_c25543	2	2	-	-	-	-	-
gALL_v3_c25546	0	3	-	-	-	-	-
gALL_v3_c25547	5	3	-	-	ref|XP_003243973.1|	PREDICTED: hypothetical protein LOC100570299 [Acyrthosiphon pisum]	1,00E-10
gALL_v3_c25548	3	3	-	-	-	-	-
gALL_v3_c25549	2	3	gnl|CDD|176031	cd08385, C2A_Synaptotagmin	ref|NP_001153672.1|	synaptotagmin I [Bombyx mori] gb|ACN21657.1| synaptotagmin I isoform A [Bombyx mori]	1,00E-115
gALL_v3_c2555	11	10	gnl|CDD|176012	cd04047, C2B_Copine, C2 dom	ref|XP_002425105.1|	Copine-8, putative [Pediculus humanus corporis] gb|EEB12367.1| Copine-8, putative [Pediculus humanus corporis]	1,00E-112
gALL_v3_c25551	1	2	-	-	-	-	-
gALL_v3_c25552	3	1	-	-	-	-	-
gALL_v3_c25553	2	1	gnl|CDD|133363	cd04163, Era, Era subfamily. E	gb|AGM32835.1|	putative GTP-binding protein Era [Coptotermes formosanus]	1,00E-52
gALL_v3_c25555	2	1	-	-	-	-	-
gALL_v3_c25556	2	2	-	-	-	-	-
gALL_v3_c25557	8	5	-	-	gb|EFZ09257.1|	hypothetical protein SINV_09572 [Solenopsis invicta]	1,00E-38
gALL_v3_c25558	0	3	-	-	-	-	-
gALL_v3_c2556	9	5	-	-	-	-	-
gALL_v3_c25560	1	4	-	-	gb|EFA09738.1|	hypothetical protein TcasGA2_TC011873 [Tribolium castaneum]	1,00E-12
gALL_v3_c25564	5	2	-	-	gb|EFA12829.1|	hypothetical protein TcasGA2_TC015881 [Tribolium castaneum]	6,00E-06
gALL_v3_c25565	8	4	-	-	-	-	-
gALL_v3_c25566	5	4	-	-	-	-	-
gALL_v3_c25567	2	2	-	-	-	-	-
gALL_v3_c25568	4	2	-	-	-	-	-
gALL_v3_c25569	0	3	-	-	-	-	-
gALL_v3_c2557	7	6	gnl|CDD|165506	PHA03247, PHA03247, large te	ref|XP_003699956.1|	PREDICTED: uncharacterized protein LOC100878395 [Megachile rotundata]	3,00E-34
gALL_v3_c25570	4	2	-	-	ref|XP_003248857.1|	PREDICTED: zinc finger protein 271-like [Acyrthosiphon pisum]	4,00E-76
gALL_v3_c25572	4	3	-	-	-	-	-
gALL_v3_c25573	7	4	-	-	-	-	-

gALL_v3_c25574	1	3	-	-	-	-	-
gALL_v3_c25576	0	4	-	-	-	-	-
gALL_v3_c25577	3	1	-	-	-	-	-
gALL_v3_c25580	0	3	-	-	-	-	-
gALL_v3_c25581	4	4	-	-	-	-	-
gALL_v3_c25582	1	3	gnl|CDD|132940	cd06609, STKc_MST3_like, Cat	ref|XP_002423585.1|	serine/threonine-protein kinase MST4, putative [Pediculus humanus corporis] gb|EEB10847.1| serine/threonine-protein kinase MST4, putative [Pediculus humanus corpor	1,00E-52
gALL_v3_c25583	3	7	-	-	-	-	-
gALL_v3_c25585	2	1	-	-	-	-	-
gALL_v3_c25587	2	2	-	-	-	-	-
gALL_v3_c2559	10	11	-	-	-	-	-
gALL_v3_c25590	2	2	-	-	-	-	-
gALL_v3_c25591	1	2	-	-	-	-	-
gALL_v3_c25592	2	2	-	-	ref|XP_001607596.2|	PREDICTED: spectrin beta chain-like [Nasonia vitripennis]	2,00E-07
gALL_v3_c25593	1	6	-	-	-	-	-
gALL_v3_c25594	1	4	gnl|CDD|147968	pfam06094, AIG2, AIG2-like fa	ref|XP_002427308.1|	conserved hypothetical protein [Pediculus humanus corporis] gb|EEB14570.1| conserved hypothetical protein [Pediculus humanus corporis]	1,00E-42
gALL_v3_c25595	3	3	gnl|CDD|29471	cd00371, HMA, Heavy-metal-a	ref|XP_003399727.1|	PREDICTED: LOW QUALITY PROTEIN: copper-transporting ATPase 1-like [Bombus terrestris]	5,00E-51
gALL_v3_c25596	5	4	gnl|CDD|178304	PLN02701, PLN02701, alpha-m	ref|XP_972030.1|	PREDICTED: similar to mannosidase alpha class 2a [Tribolium castaneum] gb|EFA06316.1| hypothetical protein TcasGA2_TC009186 [Tribolium castaneum]	1,00E-100
gALL_v3_c25597	4	2	-	-	-	-	-
gALL_v3_c25599	0	4	-	-	-	-	-
gALL_v3_c25600	2	6	gnl|CDD|34695	COG5092, NMT1, N-myristoyl	ref|XP_973156.2|	PREDICTED: similar to N-myristoyltransferase, putative [Tribolium castaneum]	0
gALL_v3_c25601	2	2	gnl|CDD|175973	cd00030, C2, C2 domain. The	gb|AGM32382.1|	C2 domain-containing protein [Coptotermes formosanus]	1,00E-55
gALL_v3_c25602	2	2	-	-	gb|EFN63189.1|	hypothetical protein EAG_04371 [Camponotus floridanus]	5,00E-08
gALL_v3_c25603	2	2	-	-	-	-	-
gALL_v3_c25604	2	2	-	-	-	-	-
gALL_v3_c25605	0	4	-	-	-	-	-
gALL_v3_c25606	1	2	-	-	-	-	-
gALL_v3_c25607	1	3	gnl|CDD|192024	pfam08395, 7tm_7, 7tm Chem	ref|XP_002426236.1|	Gustatory receptor PhGr6 [Pediculus humanus corporis] gb|EEB13498.1| Gustatory receptor PhGr6 [Pediculus humanus corporis]	3,00E-19
gALL_v3_c25609	3	1	-	-	gb|EFN74768.1|	Syntaxin-1A [Camponotus floridanus]	2,00E-07
gALL_v3_c2561	9	9	gnl|CDD|128491	smart00194, PTPc, Protein tyr	ref|XP_002430772.1|	tyrosine-protein phosphatase corkscrew, putative [Pediculus humanus corporis] gb|EEB18034.1| tyrosine-protein phosphatase corkscrew, putative [Pediculus humanus cor	0
gALL_v3_c25610	0	3	gnl|CDD|32366	COG2183, Tex, Transcriptional	ref|XP_002424176.1|	transcription elongation factor SPT6, putative [Pediculus humanus corporis] gb|EEB11438.1| transcription elongation factor SPT6, putative [Pediculus humanus corporis]	6,00E-76
gALL_v3_c25611	2	7	-	-	-	-	-
gALL_v3_c25612	1	4	-	-	-	-	-
gALL_v3_c25613	4	3	-	-	-	-	-
gALL_v3_c25614	2	2	-	-	-	-	-
gALL_v3_c25615	3	1	-	-	-	-	-
gALL_v3_c25616	2	2	gnl|CDD|152787	pfam12352, V-SNARE_C, Snare	ref|XP_002426178.1|	golgi SNAP receptor complex member, putative [Pediculus humanus corporis] gb|EEB13440.1| golgi SNAP receptor complex member, putative [Pediculus humanus corpori	1,00E-59
gALL_v3_c25617	32	35	-	-	dbj|BAM19359.1|	scarface, partial [Papilio xuthus]	1,00E-10
gALL_v3_c25618	0	3	-	-	-	-	-
gALL_v3_c25619	2	6	-	-	-	-	-
gALL_v3_c25620	1	2	-	-	-	-	-
gALL_v3_c25621	1	2	-	-	ref|XP_003730926.1|	PREDICTED: genome polyprotein-like, partial [Strongylocentrotus purpuratus]	1,00E-09
gALL_v3_c25622	2	4	gnl|CDD|190296	pfam02383, Syja_N, SacI homo	ref|XP_002429915.1|	synaptojanin-1, putative [Pediculus humanus corporis] gb|EEB17177.1| synaptojanin-1, putative [Pediculus humanus corporis]	1,00E-120
gALL_v3_c25623	2	2	-	-	-	-	-
gALL_v3_c25624	0	4	-	-	-	-	-
gALL_v3_c25625	3	2	-	-	-	-	-
gALL_v3_c25626	2	4	-	-	-	-	-
gALL_v3_c25627	1	2	-	-	-	-	-
gALL_v3_c25628	2	2	gnl|CDD|143611	cd07587, ML_beta-AS, mamm	ref|XP_005111515.1|	PREDICTED: beta-ureidopropionase-like [Aplysia californica]	2,00E-87
gALL_v3_c2563	12	7	-	-	-	-	-
gALL_v3_c25630	3	3	-	-	-	-	-
gALL_v3_c25631	1	4	-	-	-	-	-
gALL_v3_c25632	2	1	-	-	-	-	-
gALL_v3_c25633	4	9	-	-	-	-	-
gALL_v3_c25634	2	2	-	-	-	-	-
gALL_v3_c25635	3	1	-	-	-	-	-
gALL_v3_c25636	3	5	-	-	-	-	-
gALL_v3_c25637	2	3	-	-	-	-	-
gALL_v3_c25638	2	2	-	-	-	-	-
gALL_v3_c2564	14	14	-	-	-	-	-
gALL_v3_c25640	0	6	gnl|CDD|29257	cd00200, WD40, WD40 domai	ref|XP_002429632.1|	Regulatory-associated protein of mTOR, putative [Pediculus humanus corporis] gb|EEB16894.1| Regulatory-associated protein of mTOR, putative [Pediculus humanus corp	6,00E-68
gALL_v3_c25641	2	2	-	-	ref|XP_003695924.1|	PREDICTED: uncharacterized protein LOC100863302 [Apis florea]	7,00E-18
gALL_v3_c25642	4	2	-	-	-	-	-
gALL_v3_c25643	2	1	-	-	-	-	-
gALL_v3_c25644	3	4	-	-	-	-	-
gALL_v3_c25645	8	4	gnl|CDD|147569	pfam05456, eIF_4EBP, Eukaryo	ref|XP_001604271.1|	PREDICTED: eukaryotic translation initiation factor 4E-binding protein 1-like [Nasonia vitripennis]	3,00E-41
gALL_v3_c25646	2	2	-	-	-	-	-
gALL_v3_c25647	0	6	-	-	-	-	-
gALL_v3_c25648	3	2	-	-	-	-	-
gALL_v3_c2565	25	28	gnl|CDD|113680	pfam04916, Phospholip_B, Ph	ref|XP_001850556.1|	conserved hypothetical protein [Culex quinquefasciatus] gb|EDS32172.1| conserved hypothetical protein [Culex quinquefasciatus]	5,00E-06
gALL_v3_c25650	3	1	-	-	ref|XP_001604727.2|	PREDICTED: regulator of gene activity-like [Nasonia vitripennis]	3,00E-09
gALL_v3_c25651	0	3	-	-	-	-	-
gALL_v3_c25652	1	3	-	-	-	-	-
gALL_v3_c25653	7	6	-	-	-	-	-
gALL_v3_c25654	13	24	-	-	-	-	-
gALL_v3_c25656	1	3	-	-	-	-	-
gALL_v3_c25657	1	3	-	-	-	-	-
gALL_v3_c25658	1	3	-	-	-	-	-
gALL_v3_c25659	1	6	-	-	-	-	-
gALL_v3_c2566	4	5	gnl|CDD|153229	cd03135, GATase1_DJ-1, Type	gb|AGM32426.1|	DJ-1 family protein [Coptotermes formosanus]	1,00E-91
gALL_v3_c25660	2	2	-	-	gb|EFA10758.1|	starry night [Tribolium castaneum]	1,00E-10
gALL_v3_c25661	0	4	-	-	ref|XP_006619036.1|	PREDICTED: mitogen-activated protein kinase kinase kinase 4-like [Apis dorsata]	4,00E-06
gALL_v3_c25662	0	4	-	-	-	-	-
gALL_v3_c25663	2	5	-	-	-	-	-
gALL_v3_c25664	3	1	-	-	-	-	-
gALL_v3_c25665	0	3	-	-	-	-	-
gALL_v3_c25666	3	1	-	-	-	-	-
gALL_v3_c25668	7	6	gnl|CDD|48496	cd02947, TRX_family, TRX fam	gb|ETN65017.1|	thioredoxin [Anopheles darlingi]	1,00E-45
gALL_v3_c25669	4	3	-	-	-	-	-
gALL_v3_c2567	29	53	-	-	ref|XP_004921815.1|	PREDICTED: zinc finger C2HC domain-containing protein 1C-like isoform X1 [Bombyx mori]	2,00E-45
gALL_v3_c25670	2	4	-	-	-	-	-
gALL_v3_c25671	0	5	-	-	-	-	-
gALL_v3_c25672	2	1	-	-	-	-	-
gALL_v3_c25673	1	4	-	-	-	-	-
gALL_v3_c25674	3	2	-	-	-	-	-
gALL_v3_c25675	3	3	-	-	-	-	-
gALL_v3_c25676	3	2	-	-	-	-	-
gALL_v3_c25678	1	2	-	-	-	-	-
gALL_v3_c25679	0	4	-	-	-	-	-
gALL_v3_c2568	8	6	gnl|CDD|110998	pfam02055, Glyco_hydro_30,	gb|EGI59542.1|	Glucosylceramidase [Acromyrmex echinatior]	7,00E-88
gALL_v3_c25680	1	4	-	-	-	-	-
gALL_v3_c25682	4	2	gnl|CDD|128360	smart00045, DAGKa, Diacylgly	ref|XP_974283.2|	PREDICTED: similar to diacylglycerol kinase, beta 90kDa [Tribolium castaneum]	5,00E-60
gALL_v3_c25683	1	2	-	-	-	-	-
gALL_v3_c25684	5	4	-	-	ref|XP_005804292.1|	PREDICTED: constitutive coactivator of peroxisome proliferator-activated receptor gamma-like [Xiphophorus maculatus]	6,00E-24
gALL_v3_c25686	5	5	gnl|CDD|183391	PRK12270, kgd, alpha-ketoglu	-	-	-
gALL_v3_c25687	1	3	gnl|CDD|63844	cd04375, RhoGAP_DLC1, Rho(	ref|XP_002430653.1|	Rho-type GTPase activating protein, putative [Pediculus humanus corporis] gb|EEB17915.1| Rho-type GTPase activating protein, putative [Pediculus humanus corporis]	4,00E-86
gALL_v3_c25688	2	2	-	-	-	-	-
gALL_v3_c25689	7	7	-	-	-	-	-
gALL_v3_c25690	1	4	gnl|CDD|144338	pfam00698, Acyl_transf_1, Ac	ref|XP_006623860.1|	PREDICTED: fatty acid synthase-like [Apis dorsata]	3,00E-43
gALL_v3_c25691	3	5	-	-	-	-	-
gALL_v3_c25693	1	3	gnl|CDD|191507	pfam06377, Adipokin_hormo,	gb|ADB91364.1|	prepro-hypertrehalosemic hormone [Blattella germanica]	1,00E-22
gALL_v3_c25697	3	1	-	-	ref|XP_001942914.1|	PREDICTED: DNA polymerase subunit gamma-2, mitochondrial-like [Acyrthosiphon pisum]	2,00E-11
gALL_v3_c25698	0	5	gnl|CDD|178915	PRK00174, PRK00174, acetyl-(	ref|XP_002427742.1|	luciferase, putative [Pediculus humanus corporis] gb|EEB15004.1| luciferase, putative [Pediculus humanus corporis]	4,00E-49
gALL_v3_c25699	0	4	-	-	ref|XP_004928072.1|	PREDICTED: cytoplasmic dynein 2 heavy chain 1-like [Bombyx mori]	6,00E-06
gALL_v3_c257	8	13	-	-	-	-	-
gALL_v3_c2570	9	12	-	-	gb|ADI61823.1|	endonuclease-reverse transcriptase [Bombyx mori]	2,00E-45
gALL_v3_c25700	1	2	-	-	-	-	-
gALL_v3_c25702	2	3	-	-	-	-	-
gALL_v3_c25703	1	2	-	-	-	-	-
gALL_v3_c25705	5	7	-	-	gb|EFA04980.1|	hypothetical protein TcasGA2_TC015058 [Tribolium castaneum]	8,00E-92
gALL_v3_c25706	0	4	-	-	-	-	-
gALL_v3_c25707	3	3	-	-	-	-	-
gALL_v3_c25708	0	3	gnl|CDD|189825	pfam01062, Bestrophin, Bestr	ref|XP_006615436.1|	PREDICTED: bestrophin-4-like [Apis dorsata]	1,00E-72
gALL_v3_c25709	3	1	-	-	-	-	-
gALL_v3_c2571	6	3	-	-	-	-	-
gALL_v3_c25711	1	3	-	-	-	-	-
gALL_v3_c25712	2	1	gnl|CDD|190119	pfam01813, ATP-synt_D, ATP s	ref|XP_004523596.1|	PREDICTED: V-type proton ATPase subunit D 1-like [Ceratitis capitata]	1,00E-18
gALL_v3_c25713	1	3	-	-	-	-	-
gALL_v3_c25714	2	2	gnl|CDD|31397	COG1204, COG1204, Superfan	ref|XP_002429968.1|	U520, putative [Pediculus humanus corporis] gb|EEB17230.1| U520, putative [Pediculus humanus corporis]	6,00E-70
gALL_v3_c25715	0	3	-	-	-	-	-
gALL_v3_c25716	2	2	-	-	ref|XP_005657170.1|	PREDICTED: xyloside xylosyltransferase 1-like [Sus scrofa]	2,00E-30
gALL_v3_c25719	5	3	gnl|CDD|148141	pfam06350, HSL_N, Hormone-	ref|XP_975636.1|	PREDICTED: similar to hormone-sensitive lipase [Tribolium castaneum] gb|EFA02512.1| hypothetical protein TcasGA2_TC008216 [Tribolium castaneum]	4,00E-91
gALL_v3_c2572	11	22	gnl|CDD|189810	pfam01030, Recep_L_domain,	gb|EFN71461.1|	Insulin-like receptor [Camponotus floridanus]	1,00E-109
gALL_v3_c25720	3	1	-	-	-	-	-
gALL_v3_c25721	0	4	-	-	-	-	-
gALL_v3_c25722	2	3	gnl|CDD|189800	pfam01008, IF-2B, Initiation fa	ref|XP_001600995.1|	PREDICTED: translation initiation factor eIF-2B subunit beta [Nasonia vitripennis]	3,00E-74
gALL_v3_c25723	0	3	-	-	-	-	-
gALL_v3_c25725	4	3	-	-	-	-	-
gALL_v3_c25727	2	3	-	-	ref|XP_003695686.1|	PREDICTED: major facilitator superfamily domain-containing protein 6-like [Apis florea]	4,00E-27
gALL_v3_c25728	2	1	-	-	-	-	-
gALL_v3_c25729	0	5	-	-	-	-	-
gALL_v3_c2573	29	37	gnl|CDD|73156	cd01650, RT_nLTR_like, RT_nL	tpe|CAJ00236.1|	TPA: endonuclease-reverse transcriptase [Schistosoma mansoni]	1,00E-157
gALL_v3_c25730	0	3	-	-	-	-	-
gALL_v3_c25731	1	4	-	-	-	-	-
gALL_v3_c25732	1	3	-	-	-	-	-

gALL_v3_c25733	3	2	gnl|CDD|110834	pfam01869, BcrAD_BadFG, Ba	gb|EFX74032.1|	hypothetical protein DAPPUDRAFT_307522 [Daphnia pulex]	3,00E-54
gALL_v3_c25735	3	2	-	-	-	-	-
gALL_v3_c25736	2	1	-	-	-	-	-
gALL_v3_c25737	3	1	-	-	ref|XP_002114916.1|	hypothetical protein TRIADDRAFT_58909 [Trichoplax adhaerens] gb|EDV22372.1| hypothetical protein TRIADDRAFT_58909 [Trichoplax adhaerens]	3,00E-07
gALL_v3_c25738	2	2	-	-	-	-	-
gALL_v3_c25739	4	3	-	-	-	-	-
gALL_v3_c2574	13	11	gnl|CDD|189654	pfam00651, BTB, BTB/POZ dor	gb|AAP33158.1|	BTB/POZ domain-containing protein [Reticulitermes flavipes]	1,00E-180
gALL_v3_c25740	2	4	-	-	-	-	-
gALL_v3_c25742	2	1	-	-	gb|EGI70977.1|	RING finger protein 157 [Acromyrmex echinatior]	2,00E-08
gALL_v3_c25743	2	2	gnl|CDD|128516	smart00220, S_TKc, Serine/Th	ref|XP_002431024.1|	Ribosomal protein S6 kinase alpha-5, putative [Pediculus humanus corporis] gb|EEB18286.1| Ribosomal protein S6 kinase alpha-5, putative [Pediculus humanus corporis]	6,00E-48
gALL_v3_c25744	3	1	-	-	-	-	-
gALL_v3_c25745	2	2	gnl|CDD|162359	TIGR01438, TGR, thioredoxin a	gb|EKC27867.1|	Thioredoxin reductase 3 [Crassostrea gigas]	1,00E-65
gALL_v3_c25746	3	2	-	-	-	-	-
gALL_v3_c25747	2	2	-	-	ref|XP_002404979.1|	conserved hypothetical protein [Ixodes scapularis] gb|EEC15115.1| conserved hypothetical protein [Ixodes scapularis]	4,00E-46
gALL_v3_c25748	1	2	gnl|CDD|146259	pfam03520, KCNQ_channel, K	gb|EFA11047.1|	hypothetical protein TcasGA2_TC004640 [Tribolium castaneum]	6,00E-56
gALL_v3_c25749	0	4	-	-	-	-	-
gALL_v3_c2575	5	11	gnl|CDD|28913	cd00031, CA, Cadherin repeat	ref|XP_003241980.1|	PREDICTED: neural-cadherin-like [Acyrthosiphon pisum]	1,00E-56
gALL_v3_c25750	1	2	-	-	-	-	-
gALL_v3_c25751	3	3	gnl|CDD|128873	smart00611, SEC63, Domain o	gb|EFZ16351.1|	hypothetical protein SINV_08840 [Solenopsis invicta]	6,00E-91
gALL_v3_c25752	2	3	gnl|CDD|185566	PTZ00340, PTZ00340, O-sialog	ref|XP_006009929.1|	PREDICTED: probable tRNA threonylcarbamoyladenosine biosynthesis protein osgep, partial [Latimeria chalumnae]	1,00E-117
gALL_v3_c25753	1	2	-	-	-	-	-
gALL_v3_c25755	4	2	-	-	gb|EFN60282.1|	Nck-associated protein 5 [Camponotus floridanus]	5,00E-22
gALL_v3_c25756	0	3	-	-	-	-	-
gALL_v3_c25757	1	3	-	-	-	-	-
gALL_v3_c25758	3	5	-	-	-	-	-
gALL_v3_c25759	2	1	gnl|CDD|187584	cd05323, ADH_SDR_c_like, ins	gb|AAR84629.1|	putative alcohol dehydrogenase [Gryllotalpa orientalis]	6,00E-31
gALL_v3_c2576	6	11	gnl|CDD|191755	pfam07491, PPI_Ypi1, Protein	ref|XP_006561679.1|	PREDICTED: protein phosphatase 1 regulatory subunit 11 [Apis mellifera]	1,00E-25
gALL_v3_c25760	0	3	-	-	-	-	-
gALL_v3_c25761	4	2	-	-	-	-	-
gALL_v3_c25762	0	3	-	-	-	-	-
gALL_v3_c25763	1	3	gnl|CDD|189527	pfam00387, PI-PLC-Y, Phospha	ref|XP_001750272.1|	hypothetical protein [Monosiga brevicollis MX1] gb|EDQ84931.1| predicted protein [Monosiga brevicollis MX1]	5,00E-19
gALL_v3_c25764	2	2	-	-	-	-	-
gALL_v3_c25765	1	2	-	-	-	-	-
gALL_v3_c25767	3	2	-	-	-	-	-
gALL_v3_c25768	0	3	-	-	-	-	-
gALL_v3_c25769	2	1	gnl|CDD|179368	PRK02106, PRK02106, choline	ref|XP_003705711.1|	PREDICTED: glucose dehydrogenase [acceptor]-like [Megachile rotundata]	3,00E-91
gALL_v3_c2577	6	9	gnl|CDD|151044	pfam10479, FSA_C, Fragile site	gb|EFN86595.1|	Uncharacterized protein KIAA1109 [Harpegnathos saltator]	5,00E-64
gALL_v3_c25770	5	2	gnl|CDD|161668	TIGR00021, rpiA, ribose 5-pho	ref|XP_005185345.1|	PREDICTED: ribose-5-phosphate isomerase-like [Musca domestica]	1,00E-101
gALL_v3_c25771	0	3	-	-	-	-	-
gALL_v3_c25772	2	2	-	-	-	-	-
gALL_v3_c25773	1	4	-	-	-	-	-
gALL_v3_c25774	1	6	-	-	-	-	-
gALL_v3_c25777	1	3	-	-	ref|XP_001865394.1|	dumpy [Culex quinquefasciatus] gb|EDS41643.1| dumpy [Culex quinquefasciatus]	3,00E-92
gALL_v3_c25778	2	3	-	-	-	-	-
gALL_v3_c25779	5	12	-	-	ref|XP_973312.1|	PREDICTED: similar to eukaryotic translation initiation factor 3, theta subunit [Tribolium castaneum] gb|EFA10123.1| hypothetical protein TcasGA2_TC012303 [Tribolium ca	1,00E-154
gALL_v3_c2578	9	5	gnl|CDD|185425	PTZ00070, PTZ00070, 40S ribo	gb|ABZ04253.1|	ribosomal protein rps2 [Lineus viridis]	5,00E-44
gALL_v3_c25780	2	4	-	-	-	-	-
gALL_v3_c25781	1	3	-	-	-	-	-
gALL_v3_c25782	3	1	-	-	-	-	-
gALL_v3_c25785	0	4	gnl|CDD|31674	COG1485, COG1485, Predicte	ref|XP_003697330.1|	PREDICTED: putative ATPase N2B-like [Apis florea]	3,00E-17
gALL_v3_c25786	2	3	-	-	gb|ENN79716.1|	hypothetical protein YQE_03772, partial [Dendroctonus ponderosae]	7,00E-38
gALL_v3_c25787	2	2	-	-	-	-	-
gALL_v3_c25788	1	6	-	-	-	-	-
gALL_v3_c2579	6	10	-	-	gb|EFN86048.1|	Mitogen-activated protein kinase kinase kinase 15 [Harpegnathos saltator]	0
gALL_v3_c25790	2	4	-	-	-	-	-
gALL_v3_c25791	1	3	-	-	-	-	-
gALL_v3_c25792	2	2	gnl|CDD|192317	pfam09542, FFD_TFG, FFD anc	ref|XP_006560173.1|	PREDICTED: protein LSM14 homolog A isoform X1 [Apis mellifera] ref|XP_006620876.1| PREDICTED: protein LSM14 homolog A-like isoform X1 [Apis dorsata]	8,00E-37
gALL_v3_c25793	2	5	gnl|CDD|190547	pfam03153, TFIIA, Transcriptic	gb|AGM32187.1|	transcription initiation factor IIA subunit 1 [Coptotermes formosanus]	1,00E-27
gALL_v3_c25795	1	3	-	-	-	-	-
gALL_v3_c25796	2	2	-	-	-	-	-
gALL_v3_c25798	2	5	-	-	-	-	-
gALL_v3_c25799	3	1	-	-	-	-	-
gALL_v3_c258	7	6	-	-	ref|XP_006563915.1|	PREDICTED: EF-hand calcium-binding domain-containing protein 14-like isoform X5 [Apis mellifera]	9,00E-37
gALL_v3_c2580	7	6	-	-	-	-	-
gALL_v3_c25800	2	3	-	-	-	-	-
gALL_v3_c25801	2	1	-	-	gb|AHF24579.1|	hypothetical protein [uncultured bacterium Contig1529]	3,00E-43
gALL_v3_c25803	1	2	-	-	-	-	-
gALL_v3_c25804	2	3	-	-	ref|XP_970749.1|	PREDICTED: similar to ubiquitin-protein ligase [Tribolium castaneum] gb|EFA09707.1| hypothetical protein TcasGA2_TC011840 [Tribolium castaneum]	9,00E-47
gALL_v3_c25805	0	3	-	-	-	-	-
gALL_v3_c25806	2	3	-	-	ref|XP_003484524.1|	PREDICTED: mitogen-activated protein kinase kinase kinase 4-like [Bombus impatiens]	2,00E-24
gALL_v3_c25807	2	3	gnl|CDD|192358	pfam09742, Dymeclin, Dyggve	ref|XP_003484969.1|	PREDICTED: dymeclin-like [Bombus impatiens]	5,00E-38
gALL_v3_c25808	0	5	-	-	ref|XP_002424403.1|	conserved hypothetical protein [Pediculus humanus corporis] gb|EEB11665.1| conserved hypothetical protein [Pediculus humanus corporis]	4,00E-61
gALL_v3_c25809	3	2	gnl|CDD|191393	pfam05875, Ceramidase, Cera	ref|XP_003703753.1|	PREDICTED: alkaline ceramidase-like isoform 1 [Megachile rotundata]	3,00E-75
gALL_v3_c2581	4	12	-	-	ref|XP_002490879.1|	Mucin-like protein [Komagataella pastoris GS115] emb|CAY68599.1| Mucin-like protein [Komagataella pastoris GS115]	5,00E-07
gALL_v3_c25810	0	4	-	-	-	-	-
gALL_v3_c25811	1	2	-	-	-	-	-
gALL_v3_c25812	0	3	-	-	-	-	-
gALL_v3_c25813	1	4	-	-	-	-	-
gALL_v3_c25815	2	2	-	-	-	-	-
gALL_v3_c25816	6	5	-	-	ref|WP_007285192.1|	hypothetical protein [[Clostridium] bartlettii] gb|EDQ97287.1| hypothetical protein CLOBAR_00774 [Clostridium bartlettii DSM 16795] gb|EDQ97485.1| hypothetical prote	2,00E-19
gALL_v3_c25818	2	3	-	-	-	-	-
gALL_v3_c25819	3	5	-	-	ref|XP_006029730.1|	PREDICTED: histone-lysine N-methyltransferase SETMAR-like [Alligator sinensis]	2,00E-28
gALL_v3_c2582	8	11	gnl|CDD|162311	TIGR01349, PDHac_trf_mito, p	gb|EFX74372.1|	hypothetical protein DAPPUDRAFT_188759 [Daphnia pulex]	2,00E-85
gALL_v3_c25820	1	4	-	-	-	-	-
gALL_v3_c25822	2	3	-	-	-	-	-
gALL_v3_c25823	0	4	-	-	ref|XP_971977.1|	PREDICTED: similar to reserved [Tribolium castaneum]	9,00E-30
gALL_v3_c25824	2	3	-	-	gb|EEZ98574.1|	hypothetical protein TcasGA2_TC001087 [Tribolium castaneum]	5,00E-59
gALL_v3_c25825	2	1	-	-	-	-	-
gALL_v3_c25826	2	3	-	-	ref|XP_973440.2|	PREDICTED: similar to conserved hypothetical protein [Tribolium castaneum] gb|EFA08351.1| hypothetical protein TcasGA2_TC005994 [Tribolium castaneum]	8,00E-57
gALL_v3_c25827	3	1	gnl|CDD|58107	cd03776, MATH_TRAF6, Tumo	ref|XP_972955.1|	PREDICTED: similar to AGAP003004-PA [Tribolium castaneum] gb|EFA02851.1| TNF-receptor-associated factor 2 [Tribolium castaneum]	1,00E-60
gALL_v3_c25828	1	3	-	-	-	-	-
gALL_v3_c25829	7	5	-	-	-	-	-
gALL_v3_c25830	3	3	-	-	gb|AGM32707.1|	hypothetical protein, partial [Coptotermes formosanus]	5,00E-13
gALL_v3_c25831	2	7	gnl|CDD|188712	cd09326, LIM_CRP_like, The L	ref|XP_002430232.1|	muscle lim protein, putative [Pediculus humanus corporis] gb|EEB17494.1| muscle lim protein, putative [Pediculus humanus corporis]	1,00E-32
gALL_v3_c25832	3	3	gnl|CDD|63852	cd04383, RhoGAP_srGAP, Rho	ref|XP_002424891.1|	SLIT-ROBO Rho GTPase-activating protein, putative [Pediculus humanus corporis] gb|EEB12153.1| SLIT-ROBO Rho GTPase-activating protein, putative [Pediculus humanus	1,00E-121
gALL_v3_c25833	2	4	-	-	-	-	-
gALL_v3_c25834	6	6	-	-	-	-	-
gALL_v3_c25835	2	4	-	-	-	-	-
gALL_v3_c25836	0	4	-	-	-	-	-
gALL_v3_c25837	3	1	-	-	-	-	-
gALL_v3_c25838	3	1	-	-	-	-	-
gALL_v3_c25839	2	2	-	-	ref|XP_795032.3|	PREDICTED: multidrug resistance-associated protein 5-like [Strongylocentrotus purpuratus]	1,00E-10
gALL_v3_c2584	8	16	gnl|CDD|117878	pfam09336, Vps4_C, Vps4 C te	ref|XP_968121.1|	PREDICTED: similar to skd/vacuolar sorting [Tribolium castaneum] gb|EFA00923.1| hypothetical protein TcasGA2_TC003829 [Tribolium castaneum]	1,00E-128
gALL_v3_c25840	2	4	-	-	-	-	-
gALL_v3_c25841	0	5	-	-	-	-	-
gALL_v3_c25842	4	7	-	-	gb|EFN75248.1|	PiggyBac transposable element-derived protein 4 [Harpegnathos saltator]	7,00E-12
gALL_v3_c25843	3	7	-	-	-	-	-
gALL_v3_c25844	2	2	-	-	gb|EGI66561.1|	Protein zyg-11-like protein B [Acromyrmex echinatior]	2,00E-45
gALL_v3_c25845	2	3	gnl|CDD|29257	cd00200, WD40, WD40 domai	ref|XP_001601463.1|	PREDICTED: fizzy-related protein homolog [Nasonia vitripennis]	1,00E-165
gALL_v3_c25847	0	3	-	-	-	-	-
gALL_v3_c25848	3	2	-	-	-	-	-
gALL_v3_c25849	1	2	gnl|CDD|148168	pfam06400, Alpha-2-MRAP_N	ref|XP_002430317.1|	alpha-2-macroglobulin receptor-associated protein precursor, putative [Pediculus humanus corporis] gb|EEB17579.1| alpha-2-macroglobulin receptor-associated protein p	6,00E-41
gALL_v3_c2585	4	4	-	-	-	-	-
gALL_v3_c25850	0	3	-	-	ref|XP_002427879.1|	syntaxin-5, putative [Pediculus humanus corporis] gb|EEB15141.1| syntaxin-5, putative [Pediculus humanus corporis]	1,00E-09
gALL_v3_c25851	0	4	-	-	-	-	-
gALL_v3_c25852	2	1	-	-	gb|AGM32136.1|	hypothetical protein [Coptotermes formosanus]	2,00E-07
gALL_v3_c25853	4	3	-	-	gb|EEZ98550.1|	hypothetical protein TcasGA2_TC001057 [Tribolium castaneum]	2,00E-79
gALL_v3_c25854	3	1	-	-	-	-	-
gALL_v3_c25855	3	7	gnl|CDD|190224	pfam02141, DENN, DENN (AE	ref|XP_002423771.1|	Rab6-interacting protein, putative [Pediculus humanus corporis] gb|EEB11033.1| Rab6-interacting protein, putative [Pediculus humanus corporis]	1,00E-107
gALL_v3_c25856	2	2	-	-	-	-	-
gALL_v3_c25857	2	6	-	-	-	-	-
gALL_v3_c25858	3	3	gnl|CDD|146650	pfam04121, Nup84_Nup100, I	ref|XP_003703721.1|	PREDICTED: nuclear pore complex protein Nup107-like [Megachile rotundata]	4,00E-35
gALL_v3_c25859	3	2	gnl|CDD|130176	TIGR01106, ATPase-IIC_X-K, sc	ref|XP_001847413.1|	conserved hypothetical protein [Culex quinquefasciatus] gb|EDS26146.1| conserved hypothetical protein [Culex quinquefasciatus]	1,00E-16
gALL_v3_c2586	5	4	-	-	-	-	-
gALL_v3_c25860	1	4	-	-	-	-	-
gALL_v3_c25861	6	4	-	-	-	-	-
gALL_v3_c25862	2	2	-	-	-	-	-
gALL_v3_c25863	3	4	-	-	-	-	-
gALL_v3_c25864	1	3	gnl|CDD|148917	pfam07565, Band_3_cyto, Bar	ref|XP_006622710.1|	PREDICTED: sodium-driven chloride bicarbonate exchanger-like isoform X17 [Apis dorsata]	3,00E-57
gALL_v3_c25865	4	7	-	-	-	-	-
gALL_v3_c25866	2	4	gnl|CDD|162113	TIGR00927, 2A1904, K+-deper	ref|XP_001952678.2|	PREDICTED: sodium/potassium/calcium exchanger 2-like [Acyrthosiphon pisum]	1,00E-68
gALL_v3_c25867	2	1	-	-	-	-	-
gALL_v3_c25868	4	2	-	-	-	-	-
gALL_v3_c2587	30	25	-	-	gb|AGM32219.1|	hypothetical protein [Coptotermes formosanus]	9,00E-25
gALL_v3_c25870	0	6	-	-	-	-	-
gALL_v3_c25871	2	2	-	-	-	-	-
gALL_v3_c25872	3	2	-	-	gb|EFA13373.1|	hypothetical protein TcasGA2_TC001390 [Tribolium castaneum]	2,00E-38
gALL_v3_c25874	1	2	gnl|CDD|128989	smart00750, KIND, kinase non	ref|XP_001944998.2|	PREDICTED: protein spire-like [Acyrthosiphon pisum]	7,00E-31
gALL_v3_c25875	0	3	-	-	-	-	-
gALL_v3_c25876	2	1	-	-	-	-	-
gALL_v3_c25877	0	4	-	-	gb|EGI66503.1|	hypothetical protein G5I_04977 [Acromyrmex echinatior]	2,00E-07
gALL_v3_c25879	2	2	-	-	-	-	-
gALL_v3_c25880	10	14	-	-	gb|EFN67634.1|	Putative uncharacterized transposon-derived protein F52C9.6 [Camponotus floridanus]	6,00E-18
gALL_v3_c25881	0	6	gnl|CDD|189954	pfam01359, Transposase_1, Tr	ref|XP_003702583.1|	PREDICTED: histone-lysine N-methyltransferase SETMAR-like [Megachile rotundata]	1,00E-47

gALL_v3_c25882	1	2	-	-	-	-	-
gALL_v3_c25883	4	4	gnl|CDD|128895	smart00647, IBR, In Between I	ref|XP_006614825.1|	PREDICTED: E3 ubiquitin-protein ligase RNF14-like [Apis dorsata]	7,00E-62
gALL_v3_c25884	3	5	-	-	-	-	-
gALL_v3_c25885	0	3	-	-	-	-	-
gALL_v3_c25888	0	4	-	-	-	-	-
gALL_v3_c25889	1	2	-	-	-	-	-
gALL_v3_c25890	2	5	-	-	-	-	-
gALL_v3_c25891	3	2	-	-	-	-	-
gALL_v3_c25893	1	4	-	-	-	-	-
gALL_v3_c25894	3	2	-	-	-	-	-
gALL_v3_c25895	2	1	-	-	dbj|BAH71948.1|	ACYPI003239 [Acyrthosiphon pisum]	1,00E-15
gALL_v3_c25896	2	1	-	-	-	-	-
gALL_v3_c25897	0	3	-	-	-	-	-
gALL_v3_c25898	2	2	-	-	ref|XP_969896.2|	PREDICTED: similar to rolling pebbles [Tribolium castaneum]	6,00E-15
gALL_v3_c25899	2	2	gnl|CDD|145831	pfam02882, THF_DHG_CYH_C	gb|EFN87737.1|	C-1-tetrahydrofolate synthase, cytoplasmic [Harpegnathos saltator]	9,00E-49
gALL_v3_c259	4	12	-	-	-	-	-
gALL_v3_c25900	0	3	-	-	-	-	-
gALL_v3_c25901	3	3	-	-	ref|XP_003701283.1|	PREDICTED: HEAT repeat-containing protein 5B-like [Megachile rotundata]	2,00E-60
gALL_v3_c25902	2	2	-	-	-	-	-
gALL_v3_c25903	1	2	-	-	-	-	-
gALL_v3_c25904	2	6	-	-	gb|EFA09797.1|	hypothetical protein TcasGA2_TC011939 [Tribolium castaneum]	2,00E-55
gALL_v3_c25906	0	4	-	-	-	-	-
gALL_v3_c25907	0	4	-	-	-	-	-
gALL_v3_c25908	1	5	-	-	-	-	-
gALL_v3_c25909	0	5	-	-	-	-	-
gALL_v3_c2591	5	8	-	-	-	-	-
gALL_v3_c25911	3	2	-	-	ref|XP_004070552.1|	PREDICTED: protein AAR2 homolog [Oryzias latipes]	1,00E-15
gALL_v3_c25912	1	2	-	-	-	-	-
gALL_v3_c25913	3	6	-	-	gb|EHJ71699.1|	ubiquitin conjugating enzyme E2 [Danaus plexippus]	3,00E-07
gALL_v3_c25914	2	2	-	-	-	-	-
gALL_v3_c25915	5	2	-	-	dbj|BAN09206.1|	angiomotin [Gryllus bimaculatus]	2,00E-56
gALL_v3_c25917	1	2	-	-	-	-	-
gALL_v3_c25918	2	3	gnl|CDD|185447	PTZ00103, PTZ00103, 60S ribo	gb|AEU11840.1|	control protein HCTL036 [Heliconius burneyi] gb|AEU11841.1| control protein HCTL036 [Heliconius doris] gb|AEU11842.1| control protein HCTL036 [Heliconius xanthocles	1,00E-10
gALL_v3_c25919	0	5	-	-	ref|XP_003698099.1|	PREDICTED: Down syndrome cell adhesion molecule-like protein Dscam2-like, partial [Apis florea]	3,00E-52
gALL_v3_c2592	4	4	-	-	-	-	-
gALL_v3_c25920	0	4	-	-	-	-	-
gALL_v3_c25921	0	4	-	-	-	-	-
gALL_v3_c25922	1	3	-	-	ref|XP_002424783.1|	conserved hypothetical protein [Pediculus humanus corporis] gb|EEB12045.1| conserved hypothetical protein [Pediculus humanus corporis]	1,00E-120
gALL_v3_c25923	1	3	-	-	-	-	-
gALL_v3_c25924	2	3	-	-	-	-	-
gALL_v3_c25925	2	2	-	-	-	-	-
gALL_v3_c25926	0	5	-	-	-	-	-
gALL_v3_c25927	0	3	-	-	-	-	-
gALL_v3_c25928	1	4	-	-	ref|XP_001655845.1|	oxysterol-binding protein 1a [Aedes aegypti] gb|EAT35818.1| AAEL012056-PA [Aedes aegypti]	3,00E-28
gALL_v3_c25929	3	1	-	-	-	-	-
gALL_v3_c2593	12	24	-	-	-	-	-
gALL_v3_c25930	1	3	-	-	-	-	-
gALL_v3_c25931	6	4	-	-	-	-	-
gALL_v3_c25932	2	2	-	-	-	-	-
gALL_v3_c25933	3	2	-	-	-	-	-
gALL_v3_c25935	3	2	-	-	-	-	-
gALL_v3_c25936	0	3	-	-	-	-	-
gALL_v3_c25937	3	2	gnl|CDD|190081	pfam01728, FtsJ, FtsJ-like met	ref|XP_002427971.1|	conserved hypothetical protein [Pediculus humanus corporis] gb|EEB15233.1| conserved hypothetical protein [Pediculus humanus corporis]	1,00E-60
gALL_v3_c25938	1	2	-	-	-	-	-
gALL_v3_c25939	2	3	-	-	-	-	-
gALL_v3_c25940	1	3	-	-	-	-	-
gALL_v3_c25941	1	3	-	-	-	-	-
gALL_v3_c25942	5	2	-	-	-	-	-
gALL_v3_c25943	3	3	-	-	gb|EFA13518.1|	hypothetical protein TcasGA2_TC001491 [Tribolium castaneum]	3,00E-13
gALL_v3_c25944	4	3	-	-	-	-	-
gALL_v3_c25945	3	2	-	-	-	-	-
gALL_v3_c25946	7	11	gnl|CDD|191810	pfam07679, I-set, Immunoglol	gb|EFX83462.1|	hypothetical protein DAPPUDRAFT_315830 [Daphnia pulex]	5,00E-59
gALL_v3_c25947	1	2	-	-	-	-	-
gALL_v3_c25948	0	4	-	-	-	-	-
gALL_v3_c25949	1	1	-	-	-	-	-
gALL_v3_c2595	19	22	-	-	-	-	-
gALL_v3_c25950	2	1	-	-	-	-	-
gALL_v3_c25951	3	6	-	-	-	-	-
gALL_v3_c25952	3	1	-	-	-	-	-
gALL_v3_c25953	0	4	-	-	-	-	-
gALL_v3_c25954	2	4	-	-	-	-	-
gALL_v3_c25955	2	2	-	-	-	-	-
gALL_v3_c25956	0	4	-	-	-	-	-
gALL_v3_c25957	1	4	-	-	-	-	-
gALL_v3_c2596	7	13	-	-	ref|XP_003706353.1|	PREDICTED: WD repeat domain phosphoinositide-interacting protein 2-like [Megachile rotundata]	1,00E-178
gALL_v3_c25960	3	3	-	-	-	-	-
gALL_v3_c25961	3	2	-	-	-	-	-
gALL_v3_c25962	2	2	gnl|CDD|191178	pfam05051, COX17, Cytochror	ref|XP_967529.1|	PREDICTED: similar to Cytochrome c oxidase copper chaperone [Tribolium castaneum] gb|EFA08829.1| hypothetical protein TcasGA2_TC006526 [Tribolium castaneum]	2,00E-25
gALL_v3_c25963	1	4	-	-	ref|XP_002010671.1|	GI21670 [Drosophila mojavensis] gb|EDW06326.1| GI21670 [Drosophila mojavensis]	5,00E-26
gALL_v3_c25964	2	2	-	-	-	-	-
gALL_v3_c25965	0	4	gnl|CDD|175993	cd04027, C2B_Munc13, C2 do	ref|XP_002429405.1|	unc-13, putative [Pediculus humanus corporis] gb|EEB16667.1| unc-13, putative [Pediculus humanus corporis]	1,00E-100
gALL_v3_c25966	2	3	-	-	-	-	-
gALL_v3_c25967	3	3	-	-	-	-	-
gALL_v3_c25968	3	1	-	-	ref|XP_004919481.1|	PREDICTED: putative nuclease HARBI1-like [Xenopus (Silurana) tropicalis]	1,00E-14
gALL_v3_c25969	2	1	-	-	gb|EGI66221.1|	Protein turtle-like protein A [Acromyrmex echinatior]	7,00E-19
gALL_v3_c2597	5	4	gnl|CDD|149235	pfam08040, NADH_oxidored,	ref|XP_001656775.1|	NADH dehydrogenase, putative [Aedes aegypti] ref|XP_001656776.1| NADH dehydrogenase, putative [Aedes aegypti] gb|EAT45268.1| AAEL003423-PA [Aedes aegypti] gb	5,00E-22
gALL_v3_c25970	3	4	-	-	-	-	-
gALL_v3_c25971	0	4	-	-	ref|XP_001606503.2|	PREDICTED: neuroblastoma-amplified sequence [Nasonia vitripennis]	3,00E-37
gALL_v3_c25972	1	3	-	-	-	-	-
gALL_v3_c25973	2	1	-	-	-	-	-
gALL_v3_c25974	2	3	-	-	-	-	-
gALL_v3_c25975	2	1	gnl|CDD|188211	TIGR02342, chap_CCT_delta,	ref|XP_001322013.1|	chaperonin subunit delta CCTdelta [Trichomonas vaginalis G3] gb|AAG18497.1|AF226717_1 chaperonin subunit delta CCTdelta [Trichomonas vaginalis] gb|EAY09790.1| ch	6,00E-64
gALL_v3_c25977	6	13	-	-	-	-	-
gALL_v3_c25978	1	3	-	-	-	-	-
gALL_v3_c25979	2	2	gnl|CDD|183358	PRK11883, PRK11883, protopc	ref|XP_004931202.1|	PREDICTED: protoporphyrinogen oxidase-like [Bombyx mori]	6,00E-43
gALL_v3_c2598	22	31	-	-	gb|AGM32714.1|	hypothetical protein [Coptotermes formosanus]	3,00E-34
gALL_v3_c25980	0	3	-	-	emb|CDG15335.1|	Dachshund, partial [Thermobia domestica]	6,00E-17
gALL_v3_c25981	2	1	-	-	-	-	-
gALL_v3_c25982	1	2	-	-	-	-	-
gALL_v3_c25983	3	3	-	-	-	-	-
gALL_v3_c25984	1	3	-	-	-	-	-
gALL_v3_c25985	3	2	-	-	-	-	-
gALL_v3_c25986	1	2	-	-	-	-	-
gALL_v3_c25987	2	4	-	-	ref|XP_006741179.1|	PREDICTED: pogo transposable element with KRAB domain [Leptonychotes weddellii]	4,00E-11
gALL_v3_c25989	0	4	-	-	-	-	-
gALL_v3_c25990	3	2	-	-	-	-	-
gALL_v3_c25991	3	2	-	-	-	-	-
gALL_v3_c25992	9	16	-	-	-	-	-
gALL_v3_c25993	3	1	-	-	-	-	-
gALL_v3_c25994	1	2	-	-	-	-	-
gALL_v3_c25995	1	3	-	-	-	-	-
gALL_v3_c25996	0	4	gnl|CDD|189334	pfam00001, 7tm_1, 7 transme	gb|EFX64650.1|	hypothetical protein DAPPUDRAFT_66063 [Daphnia pulex]	3,00E-24
gALL_v3_c25997	2	1	-	-	-	-	-
gALL_v3_c25998	2	2	-	-	-	-	-
gALL_v3_c25999	0	3	gnl|CDD|178057	PLN02438, PLN02438, inositol	ref|XP_003291265.1|	inositol-3-phosphate synthase [Dictyostelium purpureum] gb|EGC32229.1| inositol-3-phosphate synthase [Dictyostelium purpureum]	2,00E-07
gALL_v3_c26	9	6	gnl|CDD|192287	pfam09420, Nop16, Ribosome	gb|AGM32093.1|	nucleolar protein 16-like protein [Coptotermes formosanus]	1,00E-107
gALL_v3_c2600	3	9	-	-	ref|XP_001807586.1|	PREDICTED: similar to camp-specific 3,5-cyclic phosphodiesterase [Tribolium castaneum]	1,00E-22
gALL_v3_c26000	0	5	gnl|CDD|58570	cd04269, ZnMc_adamalysin_II	gb|EFA09442.1|	hypothetical protein TcasGA2_TC010657 [Tribolium castaneum]	1,00E-103
gALL_v3_c26001	7	8	-	-	ref|XP_006615170.1|	PREDICTED: crossover junction endonuclease MUS81-like [Apis dorsata]	7,00E-07
gALL_v3_c26002	2	8	-	-	-	-	-
gALL_v3_c26003	1	2	-	-	-	-	-
gALL_v3_c26006	2	2	-	-	-	-	-
gALL_v3_c26007	3	4	-	-	-	-	-
gALL_v3_c26008	2	3	-	-	-	-	-
gALL_v3_c26009	2	2	-	-	ref|XP_004928501.1|	PREDICTED: uncharacterized protein LOC101746909 [Bombyx mori]	6,00E-07
gALL_v3_c2601	3	7	-	-	-	-	-
gALL_v3_c26010	2	2	gnl|CDD|144932	pfam01522, Polysacc_deac_1,	dbj|BAE53452.1|	predicted xylanase/chitin deacetylase [Reticulitermes speratus]	7,00E-21
gALL_v3_c26011	2	4	-	-	-	-	-
gALL_v3_c26012	0	4	-	-	-	-	-
gALL_v3_c26013	2	3	-	-	-	-	-
gALL_v3_c26014	2	2	-	-	ref|XP_003399362.1|	PREDICTED: niemann-Pick C1 protein-like [Bombus terrestris]	4,00E-12
gALL_v3_c26015	1	2	-	-	-	-	-
gALL_v3_c26017	1	3	-	-	-	-	-
gALL_v3_c26018	2	1	-	-	-	-	-
gALL_v3_c26019	4	12	-	-	-	-	-
gALL_v3_c2602	6	7	-	-	-	-	-
gALL_v3_c26020	4	7	-	-	-	-	-
gALL_v3_c26021	2	2	-	-	ref|XP_001950974.1|	PREDICTED: tRNA (uracil-5-)-methyltransferase homolog A-like [Acyrthosiphon pisum]	3,00E-21
gALL_v3_c26022	2	1	-	-	-	-	-
gALL_v3_c26023	0	6	-	-	ref|XP_003491505.1|	PREDICTED: hypothetical protein LOC100744735 [Bombus impatiens]	2,00E-25
gALL_v3_c26026	0	3	-	-	-	-	-
gALL_v3_c26027	2	3	gnl|CDD|128368	smart00053, DYNc, Dynamin,	ref|XP_003402356.1|	PREDICTED: dynamin-like [Bombus terrestris]	1,00E-81
gALL_v3_c26028	0	4	-	-	-	-	-
gALL_v3_c26029	6	4	-	-	ref|XP_006564251.1|	PREDICTED: myosin-I heavy chain isoform X2 [Apis mellifera]	7,00E-11

gALL_v3_c2603	7	4	-	-	ref|XP_002426582.1|	Cyclic AMP-dependent transcription factor ATF-6 beta, putative [Pediculus humanus corporis] gb|EEB13844.1| Cyclic AMP-dependent transcription factor ATF-6 beta, puta	2,00E-52
gALL_v3_c26030	3	2	-	-	-	-	-
gALL_v3_c26031	2	3	gnl|CDD|176323	cd01247, PH_GPBP, Goodpast	ref|XP_003703747.1|	PREDICTED: collagen type IV alpha-3-binding protein-like [Megachile rotundata]	2,00E-31
gALL_v3_c26032	0	3	-	-	-	-	-
gALL_v3_c26033	1	2	-	-	-	-	-
gALL_v3_c26035	1	6	-	-	gb|AGM32427.1|	soldier-caste specific protein [Coptotermes formosanus]	6,00E-71
gALL_v3_c26036	2	5	-	-	-	-	-
gALL_v3_c26037	2	1	-	-	-	-	-
gALL_v3_c26039	4	7	-	-	ref|XP_001601998.1|	PREDICTED: partner of Y14 and mago-like [Nasonia vitripennis]	3,00E-10
gALL_v3_c2604	14	19	gnl|CDD|34713	COG5110, RPN1, 26S proteaso	ref|XP_001604992.1|	PREDICTED: 26S proteasome non-ATPase regulatory subunit 2-like [Nasonia vitripennis]	0
gALL_v3_c26040	1	4	-	-	-	-	-
gALL_v3_c26041	1	3	-	-	ref|XP_003426012.1|	PREDICTED: hypothetical protein LOC100677801 [Nasonia vitripennis]	3,00E-16
gALL_v3_c26042	1	3	-	-	-	-	-
gALL_v3_c26044	1	3	-	-	-	-	-
gALL_v3_c26045	1	3	-	-	-	-	-
gALL_v3_c26046	2	2	-	-	-	-	-
gALL_v3_c26047	3	3	-	-	-	-	-
gALL_v3_c26048	0	4	-	-	-	-	-
gALL_v3_c26049	2	3	-	-	-	-	-
gALL_v3_c2605	23	20	gnl|CDD|129782	TIGR00699, GABAtrns_euk, 4-a	ref|XP_001656148.1|	4-aminobutyrate aminotransferase [Aedes aegypti] gb|EAT35209.1| AAEL012609-PA [Aedes aegypti]	0
gALL_v3_c26050	2	2	-	-	-	-	-
gALL_v3_c26052	2	4	-	-	-	-	-
gALL_v3_c26053	2	1	-	-	-	-	-
gALL_v3_c26054	4	2	-	-	-	-	-
gALL_v3_c26055	0	4	-	-	-	-	-
gALL_v3_c26056	3	3	-	-	-	-	-
gALL_v3_c26057	1	6	-	-	-	-	-
gALL_v3_c26058	1	3	-	-	-	-	-
gALL_v3_c26059	2	2	-	-	-	-	-
gALL_v3_c26060	4	5	gnl|CDD|189583	pfam00520, Ion_trans, Ion tra	ref|XP_001600530.2|	PREDICTED: sodium leak channel non-selective protein-like [Nasonia vitripennis]	1,00E-168
gALL_v3_c26061	2	2	-	-	-	-	-
gALL_v3_c26062	2	5	-	-	-	-	-
gALL_v3_c26063	3	1	-	-	-	-	-
gALL_v3_c26064	1	3	-	-	-	-	-
gALL_v3_c26065	3	1	-	-	-	-	-
gALL_v3_c26066	0	3	-	-	-	-	-
gALL_v3_c26068	1	3	-	-	-	-	-
gALL_v3_c2607	23	25	gnl|CDD|190879	pfam04137, ERO1, Endoplasm	ref|XP_003393715.1|	PREDICTED: ero1-like protein-like [Bombus terrestris]	9,00E-80
gALL_v3_c26070	3	2	-	-	-	-	-
gALL_v3_c26071	2	2	-	-	-	-	-
gALL_v3_c26072	1	3	-	-	-	-	-
gALL_v3_c26073	3	5	-	-	emb|CAJ14165.1|	BEL12_AG transposon polyprotein [Anopheles gambiae]	6,00E-44
gALL_v3_c26074	2	1	-	-	ref|WP_009232000.1|	conserved hypothetical protein, partial [Prevotella sp. oral taxon 317] gb|EFC67102.1| LOW QUALITY PROTEIN: hypothetical protein HMPREF0670_02906, partial [Prevotel	5,00E-06
gALL_v3_c26075	4	2	-	-	-	-	-
gALL_v3_c26076	2	1	-	-	-	-	-
gALL_v3_c26077	2	4	gnl|CDD|34965	COG5406, COG5406, Nucleosc	gb|EFZ18248.1|	hypothetical protein SINV_12622 [Solenopsis invicta]	1,00E-104
gALL_v3_c26078	2	1	-	-	-	-	-
gALL_v3_c2608	2	9	-	-	gb|EFN62957.1|	Protein chiffon [Camponotus floridanus]	2,00E-12
gALL_v3_c26080	3	2	-	-	-	-	-
gALL_v3_c26081	0	4	-	-	-	-	-
gALL_v3_c26083	3	2	-	-	-	-	-
gALL_v3_c26084	1	3	-	-	-	-	-
gALL_v3_c26085	1	3	-	-	-	-	-
gALL_v3_c26086	3	4	-	-	gb|EFN86545.1|	Autophagy-related protein 2-like protein A [Harpegnathos saltator]	9,00E-52
gALL_v3_c26087	3	2	-	-	-	-	-
gALL_v3_c26088	3	4	-	-	-	-	-
gALL_v3_c2609	6	4	-	-	-	-	-
gALL_v3_c26090	1	3	gnl|CDD|112833	pfam04034, DUF367, Domain	ref|XP_001654463.1|	hypothetical protein AaeL_AAEL010333 [Aedes aegypti] gb|EAT37698.1| AAEL010333-PA [Aedes aegypti]	6,00E-65
gALL_v3_c26091	0	3	-	-	-	-	-
gALL_v3_c26092	3	3	gnl|CDD|34751	COG5150, COG5150, Class 2 tr	gb|EFZ21833.1|	hypothetical protein SINV_03628 [Solenopsis invicta] gb|EGI68909.1| Protein Dr1 [Acromyrmex echinatior]	1,00E-64
gALL_v3_c26093	3	9	-	-	-	-	-
gALL_v3_c26094	0	3	-	-	ref|XP_002431606.1|	conserved hypothetical protein [Pediculus humanus corporis] gb|EEB18868.1| conserved hypothetical protein [Pediculus humanus corporis]	1,00E-45
gALL_v3_c26095	0	3	-	-	-	-	-
gALL_v3_c26096	2	2	-	-	-	-	-
gALL_v3_c26097	2	3	-	-	-	-	-
gALL_v3_c26098	5	6	-	-	-	-	-
gALL_v3_c26099	2	2	-	-	-	-	-
gALL_v3_c261	6	3	gnl|CDD|147006	pfam04636, PA26, PA26 p53-ir	ref|XP_002426903.1|	P53 regulated pa26 nuclear protein sestrin, putative [Pediculus humanus corporis] gb|EEB14165.1| P53 regulated pa26 nuclear protein sestrin, putative [Pediculus human	2,00E-21
gALL_v3_c2610	9	12	gnl|CDD|191390	pfam05856, ARPC4, ARP2/3 cc	gb|ETN59007.1|	ARP2/3 complex 20 kDa subunit [Anopheles darlingi]	2,00E-77
gALL_v3_c26100	2	1	-	-	ref|XP_553917.3|	AGAP010021-PA [Anopheles gambiae str. PEST] gb|EAL39252.3| AGAP010021-PA [Anopheles gambiae str. PEST]	4,00E-36
gALL_v3_c26102	0	5	-	-	-	-	-
gALL_v3_c26103	0	4	gnl|CDD|144764	pfam01285, TEA, TEA/ATTS do	gb|AEP43791.1|	scalloped [Biston betularia]	4,00E-44
gALL_v3_c26104	2	2	gnl|CDD|176644	cd00866, PEBP_euk, Phosphat	ref|XP_005176890.1|	PREDICTED: phosphatidylethanolamine-binding protein homolog F40A3.3-like [Musca domestica]	5,00E-20
gALL_v3_c26105	4	2	gnl|CDD|100116	cd06008, NF-X1-zinc-finger, Pr	ref|XP_970903.1|	PREDICTED: similar to ovarian zinc finger protein [Tribolium castaneum] gb|EEZ99870.1| hypothetical protein TcasGA2_TC002652 [Tribolium castaneum]	1,00E-81
gALL_v3_c26106	0	3	-	-	-	-	-
gALL_v3_c26107	3	3	-	-	-	-	-
gALL_v3_c26108	0	5	-	-	-	-	-
gALL_v3_c26109	2	2	-	-	-	-	-
gALL_v3_c2611	2	8	gnl|CDD|145898	pfam02990, EMP70, Endomen	ref|XP_314301.3|	AGAP004882-PA [Anopheles gambiae str. PEST] gb|EAA09712.3| AGAP004882-PA [Anopheles gambiae str. PEST]	7,00E-67
gALL_v3_c26110	1	3	-	-	-	-	-
gALL_v3_c26111	1	3	-	-	-	-	-
gALL_v3_c26112	2	1	-	-	-	-	-
gALL_v3_c26114	3	4	gnl|CDD|115148	pfam06472, ABC_membrane_	ref|XP_001943381.2|	PREDICTED: ATP-binding cassette sub-family D member 2-like [Acyrthosiphon pisum]	1,00E-113
gALL_v3_c26115	3	1	-	-	-	-	-
gALL_v3_c26116	4	4	-	-	-	-	-
gALL_v3_c26117	2	4	-	-	gb|EGI70182.1|	Uncharacterized protein C17orf85 [Acromyrmex echinatior]	2,00E-08
gALL_v3_c26118	0	3	-	-	-	-	-
gALL_v3_c26119	0	4	-	-	gb|EHJ77725.1|	putative suppressor protein [Danaus plexippus]	2,00E-07
gALL_v3_c2612	4	7	gnl|CDD|144704	pfam01204, Trehalase, Trehala	gb|AEW67359.1|	trehalase [Coptotermes formosanus]	7,00E-40
gALL_v3_c26120	2	1	-	-	ref|XP_001605545.2|	PREDICTED: WD repeat-containing protein 24-like [Nasonia vitripennis]	2,00E-40
gALL_v3_c26121	0	3	-	-	-	-	-
gALL_v3_c26124	0	3	-	-	-	-	-
gALL_v3_c26125	0	4	-	-	-	-	-
gALL_v3_c26126	1	4	-	-	-	-	-
gALL_v3_c26127	0	3	-	-	-	-	-
gALL_v3_c26129	3	3	-	-	-	-	-
gALL_v3_c2613	12	7	gnl|CDD|30753	COG0404, GcvT, Glycine cleava	ref|XP_968443.1|	PREDICTED: similar to AGAP007123-PA [Tribolium castaneum] gb|EFA02777.1| hypothetical protein TcasGA2_TC008514 [Tribolium castaneum]	1,00E-126
gALL_v3_c26130	2	2	-	-	-	-	-
gALL_v3_c26131	2	4	-	-	gb|EFN74736.1|	PiggyBac transposable element-derived protein 4 [Camponotus floridanus]	7,00E-19
gALL_v3_c26132	8	7	-	-	gb|AGM32191.1|	hypothetical protein [Coptotermes formosanus]	3,00E-34
gALL_v3_c26133	2	1	-	-	-	-	-
gALL_v3_c26134	3	1	gnl|CDD|190761	pfam03813, Nrap, Nrap protei	ref|XP_003226420.1|	PREDICTED: nucleolar protein 6-like [Anolis carolinensis]	5,00E-33
gALL_v3_c26135	4	3	-	-	-	-	-
gALL_v3_c26136	3	5	-	-	-	-	-
gALL_v3_c26138	2	2	-	-	-	-	-
gALL_v3_c26139	0	4	-	-	gb|EFA12622.1|	hypothetical protein TcasGA2_TC010249 [Tribolium castaneum]	1,00E-11
gALL_v3_c2614	27	32	-	-	ref|XP_002422963.1|	reverse transcriptase, putative [Pediculus humanus corporis] gb|EEB10225.1| reverse transcriptase, putative [Pediculus humanus corporis]	4,00E-25
gALL_v3_c26140	0	3	-	-	-	-	-
gALL_v3_c26141	3	1	-	-	-	-	-
gALL_v3_c26143	3	1	-	-	gb|EFZ20434.1|	hypothetical protein SINV_01500 [Solenopsis invicta]	8,00E-16
gALL_v3_c26144	3	2	gnl|CDD|190024	pfam01532, Glyco_hydro_47,	ref|XP_003395199.1|	PREDICTED: mannosyl-oligosaccharide alpha-1,2-mannosidase isoform A-like isoform 2 [Bombus terrestris]	7,00E-72
gALL_v3_c26145	4	3	gnl|CDD|190250	pfam02221, E1_DerP2_DerF2,	gb|AAX33733.1|	MPA2 allergen [Periplaneta americana]	4,00E-29
gALL_v3_c26146	4	5	-	-	ref|XP_002423078.1|	R3H domain-containing protein, putative [Pediculus humanus corporis] gb|EEB10340.1| R3H domain-containing protein, putative [Pediculus humanus corporis]	2,00E-20
gALL_v3_c26147	2	3	-	-	-	-	-
gALL_v3_c26148	3	2	-	-	-	-	-
gALL_v3_c26149	2	3	-	-	ref|XP_001618531.1|	hypothetical protein NEMVEDRAFT_v1g154278 [Nematostella vectensis] gb|EDO26431.1| predicted protein [Nematostella vectensis]	5,00E-09
gALL_v3_c2615	18	21	gnl|CDD|30768	COG0419, SbcC, ATPase involv	ref|XP_005483369.1|	PREDICTED: thyroid receptor-interacting protein 11 [Zonotrichia albicollis]	5,00E-19
gALL_v3_c26150	2	2	-	-	gb|EFZ16758.1|	hypothetical protein SINV_09282 [Solenopsis invicta]	8,00E-07
gALL_v3_c26152	3	4	-	-	-	-	-
gALL_v3_c26153	3	1	-	-	-	-	-
gALL_v3_c26154	5	3	-	-	-	-	-
gALL_v3_c26155	0	3	gnl|CDD|133283	cd01883, EF1_alpha, Eukaryot	gb|AAG28993.1|AF1572	translation elongation factor 1-alpha [Cunninghamella bertholletiae]	2,00E-74
gALL_v3_c26156	2	1	-	-	-	-	-
gALL_v3_c26157	4	3	-	-	-	-	-
gALL_v3_c26158	2	2	-	-	-	-	-
gALL_v3_c26159	2	3	-	-	-	-	-
gALL_v3_c2616	8	9	-	-	-	-	-
gALL_v3_c26160	0	3	-	-	-	-	-
gALL_v3_c26161	1	3	-	-	dbj|BAJ79290.1|	geranylgeranyl diphosphate synthase [Reticulitermes speratus]	8,00E-12
gALL_v3_c26162	1	3	-	-	-	-	-
gALL_v3_c26163	1	4	-	-	-	-	-
gALL_v3_c26164	1	3	-	-	-	-	-
gALL_v3_c26165	0	4	-	-	-	-	-
gALL_v3_c26166	3	2	-	-	-	-	-
gALL_v3_c26167	3	1	-	-	emb|CAA41925.1|	unnamed protein product [Drosophila miranda]	3,00E-20
gALL_v3_c26168	2	1	-	-	-	-	-
gALL_v3_c26169	3	1	-	-	-	-	-
gALL_v3_c2617	5	2	-	-	-	-	-
gALL_v3_c26170	1	3	-	-	gb|EFN79425.1|	hypothetical protein EAI_10473 [Harpegnathos saltator]	2,00E-10
gALL_v3_c26171	3	3	-	-	gb|EGI62652.1|	Nuclear receptor interaction protein [Acromyrmex echinatior]	4,00E-10
gALL_v3_c26172	2	1	-	-	gb|EFA01841.1|	hypothetical protein TcasGA2_TC007443 [Tribolium castaneum]	3,00E-19
gALL_v3_c26173	3	1	-	-	ref|XP_002429054.1|	grunge, putative [Pediculus humanus corporis] gb|EEB16316.1| grunge, putative [Pediculus humanus corporis]	8,00E-21
gALL_v3_c26174	1	3	-	-	-	-	-
gALL_v3_c26175	2	3	-	-	ref|XP_003707479.1|	PREDICTED: probable ATP-dependent RNA helicase DDX27 [Megachile rotundata]	2,00E-13

gALL_v3_c26178	2	4	-	-	-	-	-
gALL_v3_c26179	2	2	-	-	-	-	-
gALL_v3_c2618	25	20	gnl|CDD|31017	COG0673, MviM, Predicted de	gb|EFN61800.1|	Trans-1,2-dihydrobenzene-1,2-diol dehydrogenase [Camponotus floridanus]	1,00E-101
gALL_v3_c26180	0	4	-	-	-	-	-
gALL_v3_c26181	2	1	-	-	-	-	-
gALL_v3_c26182	2	3	-	-	-	-	-
gALL_v3_c26183	2	2	-	-	-	-	-
gALL_v3_c26184	3	2	-	-	-	-	-
gALL_v3_c26185	0	3	-	-	-	-	-
gALL_v3_c26186	2	5	-	-	gb|EGI59013.1|	hypothetical protein G5I_12868 [Acromyrmex echinatior]	8,00E-07
gALL_v3_c26187	2	2	gnl|CDD|192274	pfam09379, FERM_N, FERM N	dbj|BAN09207.1|	expanded protein [Gryllus bimaculatus]	3,00E-61
gALL_v3_c26188	2	3	-	-	-	-	-
gALL_v3_c26189	0	4	-	-	-	-	-
gALL_v3_c26191	3	4	-	-	-	-	-
gALL_v3_c26192	1	4	-	-	ref|XP_003241280.1|	PREDICTED: putative nuclease HARBI1-like [Acyrthosiphon pisum]	1,00E-66
gALL_v3_c26193	1	6	-	-	-	-	-
gALL_v3_c26194	3	2	-	-	-	-	-
gALL_v3_c26195	3	4	-	-	ref|XP_003492775.1|	PREDICTED: baculoviral IAP repeat-containing protein 6-like [Bombus impatiens]	3,00E-21
gALL_v3_c26196	2	2	-	-	-	-	-
gALL_v3_c26199	1	3	-	-	-	-	-
gALL_v3_c2620	5	7	gnl|CDD|188212	TIGR02349, DnaJ_bact, chaper	ref|XP_002430975.1|	protein tumorous imaginal discs, putative [Pediculus humanus corporis] gb|EEB18237.1| protein tumorous imaginal discs, putative [Pediculus humanus corporis]	2,00E-81
gALL_v3_c26200	4	3	-	-	-	-	-
gALL_v3_c26201	8	8	-	-	-	-	-
gALL_v3_c26202	4	3	gnl|CDD|190045	pfam01582, TIR, TIR domain.	gb|ACE63516.1|	Toll-9 receptor [Locusta migratoria manilensis]	3,00E-43
gALL_v3_c26203	1	3	-	-	-	-	-
gALL_v3_c26204	3	1	-	-	-	-	-
gALL_v3_c26206	8	10	gnl|CDD|29257	cd00200, WD40, WD40 domai	ref|XP_006825041.1|	PREDICTED: lissencephaly-1 homolog [Saccoglossus kowalevskii]	1,00E-129
gALL_v3_c26207	0	4	gnl|CDD|190555	pfam03184, DDE_1, DDE supe	ref|XP_002740600.1|	PREDICTED: tigger transposable element-derived protein 6-like [Saccoglossus kowalevskii]	2,00E-34
gALL_v3_c26208	1	5	-	-	gb|AAB92389.1|	non-LTR retrotransposon reverse transcriptase [Drosophila subobscura]	4,00E-20
gALL_v3_c26209	2	3	-	-	-	-	-
gALL_v3_c2621	6	10	gnl|CDD|133007	cd02514, GT13_GLCNAC-TI, G	ref|XP_002402316.1|	alpha-1,3-mannosyl-glycoprotein beta-1,2-N-acetylglucosaminyltransferase, putative [Ixodes scapularis] gb|EEC05957.1| alpha-1,3-mannosyl-glycoprotein beta-1,2-N-acet	6,00E-40
gALL_v3_c26210	3	4	-	-	-	-	-
gALL_v3_c26212	0	4	-	-	-	-	-
gALL_v3_c26213	2	1	-	-	-	-	-
gALL_v3_c26215	0	3	gnl|CDD|185577	PTZ00363, PTZ00363, rab-GDF	ref|XP_001298987.1|	GDP dissociation inhibitor family protein [Trichomonas vaginalis G3] gb|EAX86057.1| GDP dissociation inhibitor family protein [Trichomonas vaginalis G3]	1,00E-38
gALL_v3_c26216	1	3	-	-	-	-	-
gALL_v3_c26218	0	4	-	-	-	-	-
gALL_v3_c26219	1	3	gnl|CDD|191577	pfam06664, MIG-14_Wnt-bd,	ref|XP_001843799.1|	sprinter [Culex quinquefasciatus] gb|EDS34581.1| sprinter [Culex quinquefasciatus]	1,00E-41
gALL_v3_c2622	2	7	-	-	-	-	-
gALL_v3_c26220	3	4	-	-	-	-	-
gALL_v3_c26221	1	2	-	-	gb|ACE75384.1|	conserved hypothetical protein [Glyptapanteles indiensis]	3,00E-06
gALL_v3_c26222	3	4	-	-	ref|XP_968668.2|	PREDICTED: similar to AGAP004989-PB [Tribolium castaneum]	1,00E-27
gALL_v3_c26223	3	3	-	-	ref|XP_002427915.1|	RING finger protein, putative [Pediculus humanus corporis] gb|EEB15177.1| RING finger protein, putative [Pediculus humanus corporis]	3,00E-17
gALL_v3_c26224	0	4	-	-	-	-	-
gALL_v3_c26225	1	2	-	-	ref|XP_002422823.1|	zinc finger protein, putative [Pediculus humanus corporis] gb|EEB10085.1| zinc finger protein, putative [Pediculus humanus corporis]	2,00E-60
gALL_v3_c26226	1	3	-	-	-	-	-
gALL_v3_c26228	0	5	-	-	-	-	-
gALL_v3_c26229	0	3	-	-	-	-	-
gALL_v3_c2623	27	24	-	-	ref|XP_003485385.1|	PREDICTED: fatty acid-binding protein-like isoform 1 [Bombus impatiens]	3,00E-42
gALL_v3_c26230	2	1	-	-	-	-	-
gALL_v3_c26231	1	4	-	-	-	-	-
gALL_v3_c26232	1	2	-	-	-	-	-
gALL_v3_c26233	0	3	-	-	-	-	-
gALL_v3_c26234	3	3	-	-	-	-	-
gALL_v3_c26236	0	3	-	-	-	-	-
gALL_v3_c26237	4	5	-	-	-	-	-
gALL_v3_c26238	0	4	-	-	-	-	-
gALL_v3_c26239	2	3	-	-	-	-	-
gALL_v3_c2624	9	13	-	-	-	-	-
gALL_v3_c26241	2	2	-	-	-	-	-
gALL_v3_c26242	3	1	-	-	-	-	-
gALL_v3_c26243	3	1	gnl|CDD|128516	smart00220, S_TKc, Serine/Th	gb|EFX82812.1|	hypothetical protein DAPPUDRAFT_302355 [Daphnia pulex]	2,00E-58
gALL_v3_c26244	1	2	-	-	-	-	-
gALL_v3_c26245	1	3	-	-	-	-	-
gALL_v3_c26246	2	1	-	-	-	-	-
gALL_v3_c26247	0	5	-	-	-	-	-
gALL_v3_c26248	1	2	-	-	-	-	-
gALL_v3_c2625	3	6	-	-	ref|XP_002422797.1|	Fringe glycosyltransferase, putative [Pediculus humanus corporis] gb|EEB10059.1| Fringe glycosyltransferase, putative [Pediculus humanus corporis]	8,00E-12
gALL_v3_c26250	2	2	-	-	-	-	-
gALL_v3_c26251	0	4	-	-	-	-	-
gALL_v3_c26252	1	5	-	-	-	-	-
gALL_v3_c26253	2	1	gnl|CDD|162130	TIGR00955, 3a01204, The Eye	ref|XP_002428899.1|	ATP-binding cassette transporter, putative [Pediculus humanus corporis] gb|EEB16161.1| ATP-binding cassette transporter, putative [Pediculus humanus corporis]	4,00E-88
gALL_v3_c26254	2	2	-	-	-	-	-
gALL_v3_c26255	16	20	-	-	gb|EFN63523.1|	PKHD domain-containing transmembrane protein FLJ22222-like protein [Camponotus floridanus]	2,00E-37
gALL_v3_c26256	3	1	-	-	-	-	-
gALL_v3_c26257	2	5	gnl|CDD|143860	pfam00078, RVT_1, Reverse tr	ref|XP_004205527.1|	PREDICTED: RNA-directed DNA polymerase from mobile element jockey-like [Hydra vulgaris]	2,00E-26
gALL_v3_c26258	0	5	-	-	ref|XP_002428152.1|	ras GTP exchange factor, son of sevenless, putative [Pediculus humanus corporis] gb|EEB15414.1| ras GTP exchange factor, son of sevenless, putative [Pediculus humanus (	2,00E-10
gALL_v3_c26259	1	4	-	-	-	-	-
gALL_v3_c2626	10	15	gnl|CDD|73156	cd01650, RT_nLTR_like, RT_nL	gb|ADF18552.1|	endonuclease-reverse transcriptase [Bombyx mori]	8,00E-77
gALL_v3_c26260	1	3	-	-	-	-	-
gALL_v3_c26261	6	3	-	-	-	-	-
gALL_v3_c26262	3	1	-	-	-	-	-
gALL_v3_c26263	3	12	-	-	-	-	-
gALL_v3_c26264	0	4	-	-	-	-	-
gALL_v3_c26265	2	1	-	-	gb|EFN82978.1|	Beta-1,3-galactosyltransferase brn [Harpegnathos saltator]	4,00E-12
gALL_v3_c26266	2	2	-	-	-	-	-
gALL_v3_c26267	3	1	-	-	-	-	-
gALL_v3_c26268	1	3	-	-	-	-	-
gALL_v3_c26269	2	2	-	-	-	-	-
gALL_v3_c26270	2	1	-	-	-	-	-
gALL_v3_c26271	3	8	-	-	ref|XP_968557.1|	PREDICTED: similar to phospholipid scramblase 1, putative [Tribolium castaneum]	6,00E-13
gALL_v3_c26272	1	2	-	-	-	-	-
gALL_v3_c26273	0	3	-	-	-	-	-
gALL_v3_c26274	0	4	-	-	gb|EFZ13634.1|	hypothetical protein SINV_80235 [Solenopsis invicta]	7,00E-22
gALL_v3_c26275	1	3	-	-	-	-	-
gALL_v3_c26276	1	2	gnl|CDD|151101	pfam10561, UPF0565, Unchar	ref|XP_311074.5|	AGAP000078-PA [Anopheles gambiae str. PEST] gb|EAA06223.5| AGAP000078-PA [Anopheles gambiae str. PEST]	1,00E-24
gALL_v3_c26277	1	4	-	-	-	-	-
gALL_v3_c26278	1	1	-	-	-	-	-
gALL_v3_c26279	1	2	-	-	-	-	-
gALL_v3_c2628	4	9	-	-	ref|XP_001600654.1|	PREDICTED: somatostatin receptor type 2-like [Nasonia vitripennis]	8,00E-16
gALL_v3_c26281	1	2	-	-	-	-	-
gALL_v3_c26282	2	2	-	-	-	-	-
gALL_v3_c26283	2	2	-	-	-	-	-
gALL_v3_c26284	3	1	-	-	-	-	-
gALL_v3_c26285	2	1	gnl|CDD|28945	cd00063, FN3, Fibronectin typ	ref|XP_003694844.1|	PREDICTED: ephrin type-A receptor 4-A-like [Apis florea]	2,00E-40
gALL_v3_c26286	0	4	-	-	-	-	-
gALL_v3_c26287	3	2	-	-	-	-	-
gALL_v3_c2629	10	8	gnl|CDD|188217	TIGR02409, carnitine_bodg, ga	ref|XP_001991157.1|	GH12513 [Drosophila grimshawi] gb|EDV99781.1| GH12513 [Drosophila grimshawi]	1,00E-123
gALL_v3_c26290	2	2	gnl|CDD|29257	cd00200, WD40, WD40 domai	ref|XP_005186451.1|	PREDICTED: notchless protein homolog 1-like [Musca domestica]	1,00E-86
gALL_v3_c26291	1	2	-	-	-	-	-
gALL_v3_c26292	0	4	-	-	-	-	-
gALL_v3_c26293	2	1	-	-	-	-	-
gALL_v3_c26294	4	5	-	-	-	-	-
gALL_v3_c26295	2	1	gnl|CDD|190710	pfam03676, UPF0183, Unchar	gb|EFN79698.1|	UPF0183 protein CG7083 [Harpegnathos saltator]	2,00E-56
gALL_v3_c2630	28	38	-	-	-	-	-
gALL_v3_c26300	1	3	-	-	-	-	-
gALL_v3_c26301	5	3	-	-	-	-	-
gALL_v3_c26302	0	3	-	-	-	-	-
gALL_v3_c26303	7	8	gnl|CDD|178963	PRK00290, dnaK, molecular ch	gb|ACS75353.1|	ER protein gp78 [Locusta migratoria]	1,00E-115
gALL_v3_c26304	2	1	-	-	ref|XP_967794.1|	PREDICTED: similar to ADAM metallopeptidase with thrombospondin type 1 motif, 3 proprotein [Tribolium castaneum]	2,00E-15
gALL_v3_c26305	1	2	-	-	ref|XP_002424422.1|	conserved hypothetical protein [Pediculus humanus corporis] gb|EEB11684.1| conserved hypothetical protein [Pediculus humanus corporis]	4,00E-37
gALL_v3_c26306	2	1	-	-	-	-	-
gALL_v3_c26307	3	1	-	-	-	-	-
gALL_v3_c26309	3	1	-	-	-	-	-
gALL_v3_c2631	5	5	-	-	gb|EFZ14539.1|	hypothetical protein SINV_14642 [Solenopsis invicta]	6,00E-21
gALL_v3_c26310	2	2	-	-	-	-	-
gALL_v3_c26311	2	1	-	-	-	-	-
gALL_v3_c26312	3	1	-	-	ref|XP_971503.1|	PREDICTED: similar to synaptic vesicle protein [Tribolium castaneum] gb|EFA11011.1| hypothetical protein TcasGA2_TC004597 [Tribolium castaneum]	8,00E-36
gALL_v3_c26313	3	1	gnl|CDD|31690	COG1501, COG1501, Alpha-glu	ref|XP_969694.1|	PREDICTED: similar to acid alpha-glucosidase [Tribolium castaneum]	1,00E-83
gALL_v3_c26314	3	2	-	-	-	-	-
gALL_v3_c26315	3	1	-	-	-	-	-
gALL_v3_c26316	1	3	-	-	-	-	-
gALL_v3_c26317	2	1	-	-	-	-	-
gALL_v3_c26318	2	2	-	-	ref|XP_969540.1|	PREDICTED: similar to AGAP005736-PA [Tribolium castaneum] gb|EFA01050.1| hypothetical protein TcasGA2_TC003966 [Tribolium castaneum]	1,00E-23
gALL_v3_c26319	2	2	-	-	-	-	-
gALL_v3_c2632	6	5	gnl|CDD|132736	cd06938, NR_LBD_EcR, The lig	gb|AEY63780.1|	ecdysone receptor isoform A [Monochamus alternatus]	1,00E-116
gALL_v3_c26321	2	2	-	-	-	-	-
gALL_v3_c26322	1	2	-	-	-	-	-
gALL_v3_c26324	0	3	-	-	gb|EFN60993.1|	Dynein heavy chain 5, axonemal [Camponotus floridanus]	9,00E-58
gALL_v3_c26325	2	2	-	-	-	-	-
gALL_v3_c26326	2	2	-	-	gb|EGI63401.1|	Afadin- and alpha-actinin-binding protein [Acromyrmex echinatior]	7,00E-08
gALL_v3_c26327	2	2	gnl|CDD|191470	pfam06201, PITH, C-terminal p	dbj|BAN21134.1|	conserved hypothetical protein [Riptortus pedestris]	1,00E-29
gALL_v3_c26328	3	2	-	-	-	-	-
gALL_v3_c26329	9	8	-	-	-	-	-

gALL_v3_c26330	2	1	-	-	-	-	-
gALL_v3_c26331	2	3	-	-	gb|EHJ77680.1|	hypothetical protein KGM_14419 [Danaus plexippus]	6,00E-53
gALL_v3_c26332	3	1	-	-	-	-	-
gALL_v3_c26335	1	4	-	-	-	-	-
gALL_v3_c26336	2	4	-	-	-	-	-
gALL_v3_c26337	3	2	-	-	-	-	-
gALL_v3_c26338	3	1	-	-	-	-	-
gALL_v3_c26339	2	3	-	-	gb|EMJ04517.1|	hypothetical protein PRUPE_ppa019964mg, partial [Prunus persica]	8,00E-12
gALL_v3_c2634	20	20	-	-	-	-	-
gALL_v3_c26340	1	2	-	-	-	-	-
gALL_v3_c26341	0	3	-	-	-	-	-
gALL_v3_c26342	3	6	-	-	-	-	-
gALL_v3_c26343	1	2	-	-	-	-	-
gALL_v3_c26345	2	2	gnl|CDD|148354	pfam06699, PIG-F, GPI biosynt	ref|XP_003703040.1|	PREDICTED: phosphatidylinositol-glycan biosynthesis class F protein-like [Megachile rotundata]	1,00E-15
gALL_v3_c26347	4	2	-	-	-	-	-
gALL_v3_c26348	4	2	-	-	-	-	-
gALL_v3_c26349	0	5	-	-	-	-	-
gALL_v3_c26350	2	3	-	-	gb|ELU09476.1|	hypothetical protein CAPTEDRAFT_174837 [Capitella teleta]	1,00E-39
gALL_v3_c26351	3	5	-	-	-	-	-
gALL_v3_c26352	3	4	-	-	gb|EHJ71317.1|	hypothetical protein KGM_07282 [Danaus plexippus]	6,00E-06
gALL_v3_c26354	2	3	-	-	-	-	-
gALL_v3_c26355	2	2	gnl|CDD|189334	pfam00001, 7tm_1, 7 transme	dbj|BAJ83481.1|	serotonin receptor 2 alpha [Gryllus bimaculatus]	2,00E-83
gALL_v3_c26356	2	1	-	-	-	-	-
gALL_v3_c26357	3	3	-	-	-	-	-
gALL_v3_c26358	1	4	-	-	gb|EEZ99812.1|	hypothetical protein TcasGA2_TC002592 [Tribolium castaneum]	7,00E-13
gALL_v3_c26359	0	3	-	-	-	-	-
gALL_v3_c2636	5	10	-	-	-	-	-
gALL_v3_c26360	2	3	-	-	-	-	-
gALL_v3_c26361	1	1	-	-	-	-	-
gALL_v3_c26362	0	4	gnl|CDD|115674	pfam07035, Mic1, Colon canc	ref|XP_003397497.1|	PREDICTED: uncharacterized protein C18orf8-like [Bombus terrestris]	1,00E-64
gALL_v3_c26363	1	2	-	-	-	-	-
gALL_v3_c26364	1	2	-	-	-	-	-
gALL_v3_c26366	1	3	-	-	gb|EGI58065.1|	Histone-lysine N-methyltransferase SETMAR [Acromyrmex echinatior]	1,00E-06
gALL_v3_c26367	1	2	-	-	-	-	-
gALL_v3_c26368	3	1	-	-	-	-	-
gALL_v3_c26370	3	2	-	-	-	-	-
gALL_v3_c26371	1	3	-	-	-	-	-
gALL_v3_c26372	1	2	-	-	-	-	-
gALL_v3_c26373	0	6	-	-	-	-	-
gALL_v3_c26374	3	2	-	-	-	-	-
gALL_v3_c26375	2	3	-	-	ref|XP_006812321.1|	PREDICTED: c-Jun-amino-terminal kinase-interacting protein 4-like [Saccoglossus kowalevskii]	1,00E-21
gALL_v3_c26377	1	2	-	-	-	-	-
gALL_v3_c26378	2	2	-	-	-	-	-
gALL_v3_c26379	2	3	-	-	-	-	-
gALL_v3_c2638	2	6	-	-	-	-	-
gALL_v3_c26382	3	1	-	-	-	-	-
gALL_v3_c26383	1	2	-	-	-	-	-
gALL_v3_c26384	1	3	-	-	-	-	-
gALL_v3_c26385	1	2	-	-	-	-	-
gALL_v3_c26386	1	5	-	-	-	-	-
gALL_v3_c26387	3	3	-	-	-	-	-
gALL_v3_c26388	1	3	-	-	sp|Q25637.2|PER_PERA	RecName: Full=Period circadian protein gb|AAA64677.2| period clock protein homolog [Periplaneta americana]	6,00E-74
gALL_v3_c26389	1	6	-	-	-	-	-
gALL_v3_c2639	3	3	gnl|CDD|180426	PRK06148, PRK06148, hypothe	ref|XP_003703021.1|	PREDICTED: alanine--glyoxylate aminotransferase 2-like [Megachile rotundata]	2,00E-55
gALL_v3_c26390	1	2	-	-	-	-	-
gALL_v3_c26391	2	2	-	-	ref|XP_005989238.1|	PREDICTED: uncharacterized protein LOC102348870 [Latimeria chalumnae]	2,00E-20
gALL_v3_c26393	2	1	-	-	-	-	-
gALL_v3_c26394	3	4	gnl|CDD|181920	PRK09510, tolA, cell envelope	-	-	-
gALL_v3_c26395	2	3	-	-	ref|XP_003490164.1|	PREDICTED: gametogenetin-binding protein 2-like [Bombus impatiens]	9,00E-11
gALL_v3_c26396	0	3	-	-	-	-	-
gALL_v3_c26397	1	3	gnl|CDD|129984	TIGR00906, 2A0303, cationic a	gb|ETN62402.1|	cationic amino acid transporter [Anopheles darlingi]	2,00E-29
gALL_v3_c26399	5	2	-	-	-	-	-
gALL_v3_c2640	12	13	gnl|CDD|176644	cd00866, PEBP_euk, Phosphat	ref|XP_969591.1|	PREDICTED: similar to AGAP008909-PA [Tribolium castaneum] gb|EFA12819.1| hypothetical protein TcasGA2_TC001886 [Tribolium castaneum]	3,00E-26
gALL_v3_c26401	2	2	-	-	-	-	-
gALL_v3_c26402	0	4	-	-	ref|XP_003706431.1|	PREDICTED: uncharacterized protein LOC100876920 [Megachile rotundata]	4,00E-16
gALL_v3_c26403	2	3	-	-	-	-	-
gALL_v3_c26405	2	1	-	-	-	-	-
gALL_v3_c26406	2	3	-	-	-	-	-
gALL_v3_c26408	4	8	-	-	-	-	-
gALL_v3_c26409	0	4	-	-	-	-	-
gALL_v3_c26410	1	4	-	-	-	-	-
gALL_v3_c26411	3	7	-	-	-	-	-
gALL_v3_c26413	3	2	-	-	-	-	-
gALL_v3_c26414	1	2	-	-	-	-	-
gALL_v3_c26415	1	3	-	-	-	-	-
gALL_v3_c26416	0	4	-	-	-	-	-
gALL_v3_c26417	1	5	-	-	-	-	-
gALL_v3_c26418	0	3	-	-	-	-	-
gALL_v3_c2642	8	4	-	-	ref|XP_001604233.1|	PREDICTED: signal recognition particle 54 kDa protein-like [Nasonia vitripennis]	5,00E-07
gALL_v3_c26420	0	3	-	-	-	-	-
gALL_v3_c26421	2	5	-	-	-	-	-
gALL_v3_c26422	0	4	-	-	-	-	-
gALL_v3_c26423	2	1	-	-	-	-	-
gALL_v3_c26424	5	3	gnl|CDD|189954	pfam01359, Transposase_1, Tr	ref|XP_006029730.1|	PREDICTED: histone-lysine N-methyltransferase SETMAR-like [Alligator sinensis]	3,00E-78
gALL_v3_c26425	2	1	gnl|CDD|190008	pfam01490, Aa_trans, Transm	ref|XP_006624884.1|	PREDICTED: proton-coupled amino acid transporter 1-like [Apis dorsata]	1,00E-32
gALL_v3_c26426	1	2	-	-	-	-	-
gALL_v3_c26427	1	2	-	-	-	-	-
gALL_v3_c26428	2	2	-	-	gb|AGM32648.1|	cytochrome P450 [Coptotermes formosanus]	3,00E-17
gALL_v3_c26429	1	3	-	-	-	-	-
gALL_v3_c2643	8	12	-	-	ref|XP_001948919.1|	PREDICTED: mitogen-activated protein kinase kinase kinase 7-like [Acyrthosiphon pisum]	3,00E-24
gALL_v3_c26430	2	3	-	-	-	-	-
gALL_v3_c26431	2	8	-	-	dbj|BAN09206.1|	angiomotin [Gryllus bimaculatus]	8,00E-81
gALL_v3_c26432	1	3	-	-	ref|WP_005922760.1|	hypothetical protein [Faecalibacterium prausnitzii] gb|EDP22130.1| hypothetical protein FAEPRAM212_01166 [Faecalibacterium prausnitzii M21/2]	4,00E-45
gALL_v3_c26433	1	3	gnl|CDD|190625	pfam03399, SAC3_GANP, SAC3	ref|XP_003485634.1|	PREDICTED: 80 kDa MCM3-associated protein-like [Bombus impatiens]	2,00E-18
gALL_v3_c26434	2	1	-	-	-	-	-
gALL_v3_c26435	2	2	-	-	-	-	-
gALL_v3_c26436	5	2	gnl|CDD|188096	TIGR00930, 2a30, K-Cl cotrans	gb|EFN60266.1|	Bumetanide-sensitive sodium-(potassium)-chloride cotransporter [Camponotus floridanus]	1,00E-34
gALL_v3_c26437	1	2	-	-	-	-	-
gALL_v3_c26438	2	1	-	-	-	-	-
gALL_v3_c26439	10	6	gnl|CDD|100016	cd02187, beta_tubulin, The tu	dbj|BAF48691.1|	beta tubulin [Holomastigotoides mirabile]	1,00E-152
gALL_v3_c2644	5	4	gnl|CDD|128904	smart00657, RPOL4c, DNA-dir	ref|XP_006610861.1|	PREDICTED: DNA-directed RNA polymerase II 16 kDa polypeptide-like isoform X3 [Apis dorsata]	2,00E-53
gALL_v3_c26441	5	8	-	-	-	-	-
gALL_v3_c26442	2	2	-	-	-	-	-
gALL_v3_c26443	1	5	-	-	-	-	-
gALL_v3_c26444	1	3	-	-	dbj|BAD98519.1|	hypotheical protein [Nasutitermes takasagoensis]	2,00E-27
gALL_v3_c26445	1	3	-	-	-	-	-
gALL_v3_c26446	1	3	-	-	-	-	-
gALL_v3_c26447	1	6	-	-	-	-	-
gALL_v3_c26448	11	8	gnl|CDD|189411	pfam00135, COesterase, Carbo	ref|XP_003704957.1|	PREDICTED: venom carboxylesterase-6-like [Megachile rotundata]	5,00E-42
gALL_v3_c26449	1	3	gnl|CDD|31389	COG1196, Smc, Chromosome	-	-	-
gALL_v3_c2645	10	15	-	-	-	-	-
gALL_v3_c26450	1	3	-	-	ref|XP_002611770.1|	hypothetical protein BRAFLDRAFT_268910 [Branchiostoma floridae] gb|EEN67780.1| hypothetical protein BRAFLDRAFT_268910 [Branchiostoma floridae]	1,00E-08
gALL_v3_c26451	1	8	-	-	-	-	-
gALL_v3_c26453	1	2	-	-	-	-	-
gALL_v3_c26454	3	1	-	-	-	-	-
gALL_v3_c26455	0	3	-	-	-	-	-
gALL_v3_c26456	1	3	-	-	-	-	-
gALL_v3_c26457	1	4	-	-	-	-	-
gALL_v3_c26458	1	5	-	-	-	-	-
gALL_v3_c26459	2	2	-	-	-	-	-
gALL_v3_c2646	9	7	gnl|CDD|162459	TIGR01642, U2AF_lg, U2 snRN	ref|XP_002430045.1|	Splicing factor U2AF 50 kDa subunit, putative [Pediculus humanus corporis] gb|EEB17307.1| Splicing factor U2AF 50 kDa subunit, putative [Pediculus humanus corporis]	1,00E-117
gALL_v3_c26460	1	3	-	-	ref|XP_003244717.1|	PREDICTED: hypothetical protein LOC100574008 [Acyrthosiphon pisum]	2,00E-28
gALL_v3_c26461	3	2	-	-	-	-	-
gALL_v3_c26463	3	1	-	-	-	-	-
gALL_v3_c26464	5	2	gnl|CDD|189507	pfam00335, Tetraspannin, Tetr	gb|EFN65692.1|	Tetraspanin-11 [Camponotus floridanus]	4,00E-26
gALL_v3_c26465	2	3	-	-	gb|EFN86095.1|	Zinc finger SWIM domain-containing protein KIAA0913 [Harpegnathos saltator]	5,00E-38
gALL_v3_c26466	2	6	-	-	-	-	-
gALL_v3_c26467	4	2	-	-	-	-	-
gALL_v3_c26468	2	3	-	-	-	-	-
gALL_v3_c26469	0	4	-	-	ref|XP_004929225.1|	PREDICTED: zinc finger protein Xfin-like [Bombyx mori]	3,00E-37
gALL_v3_c2647	7	7	-	-	ref|XP_003399522.1|	PREDICTED: elongation of very long chain fatty acids protein AAEL008004-like [Bombus terrestris]	3,00E-26
gALL_v3_c26470	4	2	-	-	-	-	-
gALL_v3_c26471	0	3	gnl|CDD|175990	cd04024, C2A_Synaptotagmin	ref|XP_003424887.1|	PREDICTED: extended synaptotagmin-1 isoform 2 [Nasonia vitripennis]	1,00E-56
gALL_v3_c26472	4	2	-	-	-	-	-
gALL_v3_c26473	1	4	-	-	-	-	-
gALL_v3_c26474	15	31	gnl|CDD|28968	cd01390, HMGB-UBF_HMG-bo	ref|XP_973934.2|	PREDICTED: similar to High mobility group protein DSP1 (Protein dorsal switch 1) [Tribolium castaneum]	1,00E-108
gALL_v3_c26475	1	3	-	-	-	-	-
gALL_v3_c26476	1	3	-	-	gb|EGI63515.1|	Actin-interacting protein 1 [Acromyrmex echinatior]	5,00E-63
gALL_v3_c26477	3	1	-	-	-	-	-
gALL_v3_c26478	1	2	-	-	-	-	-
gALL_v3_c26479	1	2	-	-	ref|XP_003691161.1|	PREDICTED: uncharacterized protein LOC100865938 [Apis florea]	1,00E-09
gALL_v3_c2648	11	12	gnl|CDD|187547	cd05236, FAR-N_SDR_e, fatty	ref|XP_001950244.1|	PREDICTED: putative fatty acyl-CoA reductase CG5065-like [Acyrthosiphon pisum]	8,00E-11
gALL_v3_c26481	2	2	-	-	-	-	-
gALL_v3_c26483	1	3	-	-	-	-	-

gALL_v3_c26484	2	2	-	-	-	-	-
gALL_v3_c26485	3	5	gnl|CDD|148068	pfam06239, ECSIT, Evolutionar	ref|XP_001650325.1|	ecsit (evolutionarily conserved signaling intermediate in toll pathways) [Aedes aegypti] gb|EAT43515.1| AAEL005075-PA [Aedes aegypti]	1,00E-111
gALL_v3_c26486	2	1	-	-	ref|XP_001605115.1|	PREDICTED: poly(ADP-ribose) glycohydrolase-like [Nasonia vitripennis]	9,00E-23
gALL_v3_c26488	2	1	-	-	-	-	-
gALL_v3_c26489	7	13	-	-	-	-	-
gALL_v3_c2649	5	12	-	-	ref|XP_003392933.1|	PREDICTED: chromatin complexes subunit BAP18-like isoform 1 [Bombus terrestris]	2,00E-31
gALL_v3_c26491	3	1	gnl|CDD|149585	pfam08584, Ribonuc_P_40, Ri	ref|XP_003706864.1|	PREDICTED: ribonuclease P protein subunit p40-like [Megachile rotundata]	3,00E-40
gALL_v3_c26492	3	2	-	-	-	-	-
gALL_v3_c26493	4	2	-	-	ref|XP_006643481.1|	PREDICTED: zinc finger protein 180-like, partial [Lepisosteus oculatus]	1,00E-26
gALL_v3_c26494	1	2	gnl|CDD|190700	pfam03645, Tctex-1, Tctex-1 fa	ref|XP_003700731.1|	PREDICTED: tctex1 domain-containing protein 2-like [Megachile rotundata]	2,00E-34
gALL_v3_c26495	4	2	-	-	ref|XP_002801067.1|	PREDICTED: hypothetical protein LOC100426888 [Macaca mulatta]	3,00E-06
gALL_v3_c26496	1	4	-	-	-	-	-
gALL_v3_c26497	0	3	-	-	-	-	-
gALL_v3_c26498	1	3	gnl|CDD|100104	cd00590, RRM, RRM (RNA reco	ref|XP_002602165.1|	hypothetical protein BRAFLDRAFT_268357 [Branchiostoma floridae] gb|EEN58177.1| hypothetical protein BRAFLDRAFT_268357 [Branchiostoma floridae]	1,00E-75
gALL_v3_c26499	1	3	-	-	-	-	-
gALL_v3_c265	3	6	-	-	-	-	-
gALL_v3_c26500	1	2	gnl|CDD|179935	PRK05096, PRK05096, guanos	gb|EFA06520.1|	hypothetical protein TcasGA2_TC009423 [Tribolium castaneum]	6,00E-26
gALL_v3_c26501	2	2	-	-	-	-	-
gALL_v3_c26502	1	3	-	-	-	-	-
gALL_v3_c26503	2	1	-	-	-	-	-
gALL_v3_c26505	2	4	-	-	-	-	-
gALL_v3_c26506	0	3	-	-	-	-	-
gALL_v3_c26507	3	1	-	-	-	-	-
gALL_v3_c26508	2	2	-	-	-	-	-
gALL_v3_c2651	5	9	-	-	-	-	-
gALL_v3_c26510	5	4	gnl|CDD|178434	PLN02841, PLN02841, GPI ma	ref|XP_003425268.1|	PREDICTED: LOW QUALITY PROTEIN: GPI mannosyltransferase 1-like [Nasonia vitripennis]	5,00E-31
gALL_v3_c26511	3	1	-	-	-	-	-
gALL_v3_c26512	0	4	-	-	-	-	-
gALL_v3_c26513	0	3	-	-	-	-	-
gALL_v3_c26514	1	3	-	-	-	-	-
gALL_v3_c26515	3	2	-	-	ref|XP_004931200.1|	PREDICTED: uncharacterized protein YBL113C-like isoform X2 [Bombyx mori]	2,00E-14
gALL_v3_c26517	3	1	-	-	-	-	-
gALL_v3_c26518	1	7	-	-	-	-	-
gALL_v3_c26519	2	6	-	-	ref|XP_001961421.1|	GF14939 [Drosophila ananassae] gb|EDV30642.1| GF14939 [Drosophila ananassae]	7,00E-11
gALL_v3_c2652	3	9	-	-	-	-	-
gALL_v3_c26520	0	3	-	-	-	-	-
gALL_v3_c26521	2	1	gnl|CDD|189529	pfam00389, 2-Hacid_dh, D-iso	gb|EFZ21660.1|	hypothetical protein SINV_13482 [Solenopsis invicta]	1,00E-105
gALL_v3_c26522	2	2	-	-	-	-	-
gALL_v3_c26523	0	3	-	-	-	-	-
gALL_v3_c26524	1	3	-	-	-	-	-
gALL_v3_c26525	1	2	-	-	ref|XP_004925006.1|	PREDICTED: uncharacterized protein LOC101742051 [Bombyx mori]	1,00E-19
gALL_v3_c26526	2	1	-	-	-	-	-
gALL_v3_c26527	3	1	-	-	-	-	-
gALL_v3_c26528	2	1	-	-	emb|CAX75350.1|	O-glycosyltransferase [Schistosoma japonicum]	2,00E-06
gALL_v3_c26529	3	1	-	-	-	-	-
gALL_v3_c2653	5	6	-	-	-	-	-
gALL_v3_c26530	2	2	-	-	-	-	-
gALL_v3_c26531	1	3	-	-	-	-	-
gALL_v3_c26532	2	4	gnl|CDD|128336	smart00020, Tryp_SPc, Trypsir	gb|ENN75734.1|	hypothetical protein YQE_07694, partial [Dendroctonus ponderosae]	2,00E-68
gALL_v3_c26533	2	2	-	-	-	-	-
gALL_v3_c26534	2	3	-	-	-	-	-
gALL_v3_c26535	0	4	-	-	-	-	-
gALL_v3_c26536	2	2	-	-	-	-	-
gALL_v3_c26538	1	4	-	-	ref|XP_002427713.1|	dedicator of cytokinesis protein, putative [Pediculus humanus corporis] gb|EEB14975.1| dedicator of cytokinesis protein, putative [Pediculus humanus corporis]	7,00E-75
gALL_v3_c26539	2	5	-	-	-	-	-
gALL_v3_c2654	12	15	-	-	ref|XP_002430736.1|	conserved hypothetical protein [Pediculus humanus corporis] gb|EEB17998.1| conserved hypothetical protein [Pediculus humanus corporis]	4,00E-32
gALL_v3_c26540	3	4	-	-	-	-	-
gALL_v3_c26541	0	3	-	-	-	-	-
gALL_v3_c26542	1	2	gnl|CDD|192042	pfam08450, SGL, SMP-30/Gluc	ref|XP_002432441.1|	senescence marker protein-30, putative [Pediculus humanus corporis] gb|EEB19703.1| senescence marker protein-30, putative [Pediculus humanus corporis]	1,00E-14
gALL_v3_c26543	1	4	-	-	-	-	-
gALL_v3_c26544	0	3	-	-	-	-	-
gALL_v3_c26545	1	2	-	-	-	-	-
gALL_v3_c26546	2	1	-	-	-	-	-
gALL_v3_c26548	0	6	gnl|CDD|187584	cd05323, ADH_SDR_c_like, ins	ref|XP_966954.2|	PREDICTED: similar to putative alcohol dehydrogenase [Tribolium castaneum] gb|EFA05875.1| hypothetical protein TcasGA2_TC008684 [Tribolium castaneum]	2,00E-12
gALL_v3_c26549	2	6	-	-	-	-	-
gALL_v3_c2655	7	6	-	-	ref|XP_974307.1|	PREDICTED: similar to par-6 gamma [Tribolium castaneum]	3,00E-16
gALL_v3_c26550	0	5	-	-	-	-	-
gALL_v3_c26551	4	6	-	-	-	-	-
gALL_v3_c26552	2	1	gnl|CDD|193102	pfam12624, Chorein_N, N-terr	ref|XP_004930809.1|	PREDICTED: vacuolar protein sorting-associated protein 13A-like [Bombyx mori]	6,00E-25
gALL_v3_c26553	2	3	-	-	-	-	-
gALL_v3_c26554	2	2	-	-	-	-	-
gALL_v3_c26555	0	5	-	-	-	-	-
gALL_v3_c26556	1	6	-	-	-	-	-
gALL_v3_c26557	2	3	-	-	-	-	-
gALL_v3_c26558	0	4	-	-	-	-	-
gALL_v3_c26559	1	3	-	-	-	-	-
gALL_v3_c2656	6	6	gnl|CDD|128954	smart00714, LITAF, Possible m	ref|XP_003423956.1|	PREDICTED: lipopolysaccharide-induced tumor necrosis factor-alpha factor homolog [Nasonia vitripennis]	7,00E-12
gALL_v3_c26560	1	3	-	-	gb|ENN78341.1|	hypothetical protein YQE_05143, partial [Dendroctonus ponderosae] gb|ERL87924.1| hypothetical protein D910_05312 [Dendroctonus ponderosae]	2,00E-12
gALL_v3_c26564	5	2	-	-	gb|EFX64605.1|	hypothetical protein DAPPUDRAFT_334025 [Daphnia pulex]	5,00E-13
gALL_v3_c26568	0	3	-	-	-	-	-
gALL_v3_c2657	3	10	-	-	-	-	-
gALL_v3_c26570	2	3	gnl|CDD|133465	cd06855, GT_GPT_euk, UDP-G	ref|XP_968050.1|	PREDICTED: similar to CG5287 CG5287-PA [Tribolium castaneum] gb|EFA02198.1| hypothetical protein TcasGA2_TC007854 [Tribolium castaneum]	4,00E-43
gALL_v3_c26571	2	2	-	-	-	-	-
gALL_v3_c26572	2	6	-	-	-	-	-
gALL_v3_c26573	2	2	-	-	-	-	-
gALL_v3_c26574	3	2	gnl|CDD|191399	pfam05907, DUF866, Eukaryot	ref|XP_975460.1|	PREDICTED: similar to CG4646 CG4646-PA [Tribolium castaneum]	6,00E-30
gALL_v3_c26575	2	4	-	-	-	-	-
gALL_v3_c26576	2	3	gnl|CDD|29257	cd00200, WD40, WD40 domai	ref|XP_001607211.2|	PREDICTED: WD repeat-containing protein 91-like isoform 1 [Nasonia vitripennis]	2,00E-63
gALL_v3_c26577	1	2	-	-	-	-	-
gALL_v3_c26578	4	3	-	-	ref|XP_003401725.1|	PREDICTED: calcineurin-binding protein cabin-1-like [Bombus terrestris]	1,00E-11
gALL_v3_c26579	2	3	-	-	ref|XP_968552.2|	PREDICTED: similar to calmodulin-binding transcription activator [Tribolium castaneum]	7,00E-15
gALL_v3_c2658	9	5	gnl|CDD|29000	cd01328, FSL_SPARC, Follistati	gb|AAV88596.1|	Sparc [Schistocerca gregaria]	9,00E-91
gALL_v3_c26580	3	1	-	-	-	-	-
gALL_v3_c26581	4	7	-	-	ref|XP_001656629.1|	hypothetical protein AaeL_AAEL003229 [Aedes aegypti] gb|EAT45483.1| AAEL003229-PA [Aedes aegypti]	3,00E-11
gALL_v3_c26583	0	3	-	-	-	-	-
gALL_v3_c26584	2	1	-	-	-	-	-
gALL_v3_c26585	3	2	-	-	-	-	-
gALL_v3_c26586	3	3	-	-	gb|ELT99420.1|	hypothetical protein CAPTEDRAFT_88086, partial [Capitella teleta]	6,00E-56
gALL_v3_c26587	3	4	-	-	-	-	-
gALL_v3_c26588	2	3	-	-	ref|XP_002423279.1|	cAMP-specific 3',5'-cyclic phosphodiesterase, putative [Pediculus humanus corporis] gb|EEB10541.1| cAMP-specific 3',5'-cyclic phosphodiesterase, putative [Pediculus hun	6,00E-29
gALL_v3_c2659	7	8	gnl|CDD|189934	pfam01302, CAP_GLY, CAP-Gly	ref|XP_003699541.1|	PREDICTED: tubulin-folding cofactor B-like [Megachile rotundata]	2,00E-65
gALL_v3_c26590	3	1	-	-	-	-	-
gALL_v3_c26592	1	3	-	-	-	-	-
gALL_v3_c26593	1	3	-	-	-	-	-
gALL_v3_c26594	0	3	-	-	-	-	-
gALL_v3_c26595	1	2	-	-	-	-	-
gALL_v3_c26596	2	1	-	-	-	-	-
gALL_v3_c26597	3	1	-	-	-	-	-
gALL_v3_c26598	2	2	gnl|CDD|29257	cd00200, WD40, WD40 domai	gb|AAB49671.1|	beta-transducin repeat containing protein-2, partial [Xenopus laevis]	1,00E-122
gALL_v3_c26599	3	2	-	-	gb|AAX64155.1|	hypothetical protein SCH_0249 [Salmonella enterica subsp. enterica serovar Choleraesuis str. SC-B67]	3,00E-70
gALL_v3_c266	22	21	gnl|CDD|173625	cd05032, PTKc_InsR_like, Cata	gb|EGI67492.1|	Insulin-like receptor [Acromyrmex echinatior]	0
gALL_v3_c2660	11	10	gnl|CDD|177994	PLN02366, PLN02366, spermi	ref|XP_003400308.1|	PREDICTED: spermidine synthase-like [Bombus terrestris]	4,00E-51
gALL_v3_c26600	2	6	-	-	-	-	-
gALL_v3_c26602	2	1	-	-	-	-	-
gALL_v3_c26603	1	3	-	-	-	-	-
gALL_v3_c26604	1	4	-	-	-	-	-
gALL_v3_c26605	1	2	-	-	-	-	-
gALL_v3_c26606	2	2	-	-	-	-	-
gALL_v3_c26607	1	3	-	-	ref|XP_969527.1|	PREDICTED: similar to CG13409 CG13409-PA [Tribolium castaneum] gb|EEZ97376.1| hypothetical protein TcasGA2_TC011201 [Tribolium castaneum]	2,00E-23
gALL_v3_c26608	2	2	-	-	-	-	-
gALL_v3_c26609	3	2	-	-	gb|ESO06316.1|	hypothetical protein HELRODRAFT_160480 [Helobdella robusta]	3,00E-11
gALL_v3_c2661	7	8	-	-	ref|XP_004923734.1|	PREDICTED: heterogeneous nuclear ribonucleoprotein L-like [Bombyx mori]	7,00E-13
gALL_v3_c26611	3	6	gnl|CDD|63841	cd04372, RhoGAP_chimaerin,	ref|XP_001945123.1|	PREDICTED: beta-chimaerin-like [Acyrthosiphon pisum]	2,00E-12
gALL_v3_c26612	1	3	-	-	-	-	-
gALL_v3_c26613	2	3	gnl|CDD|34704	COG5101, CRM1, Importin bet	ref|XP_001604619.1|	PREDICTED: exportin-1-like [Nasonia vitripennis]	4,00E-92
gALL_v3_c26614	2	2	-	-	-	-	-
gALL_v3_c26615	3	3	gnl|CDD|34793	COG5194, APC11, Component	gb|ABR27849.1|	SCF ubiquitin ligase Rbx1 component [Triatoma infestans]	1,00E-69
gALL_v3_c26617	2	1	-	-	-	-	-
gALL_v3_c26618	0	5	-	-	gb|EHJ77170.1|	putative vacuolar protein sorting-associated protein [Danaus plexippus]	3,00E-36
gALL_v3_c26619	3	2	-	-	-	-	-
gALL_v3_c2662	6	9	-	-	-	-	-
gALL_v3_c26621	4	2	-	-	gb|ERL95993.1|	hypothetical protein D910_00759, partial [Dendroctonus ponderosae]	5,00E-12
gALL_v3_c26622	2	2	-	-	-	-	-
gALL_v3_c26623	2	2	-	-	-	-	-
gALL_v3_c26624	4	4	gnl|CDD|73192	cd00320, cpn10, Chaperonin 1	ref|XP_002428683.1|	10 kDa heat shock protein, putative [Pediculus humanus corporis] gb|EEB15945.1| 10 kDa heat shock protein, putative [Pediculus humanus corporis]	4,00E-48
gALL_v3_c26625	1	3	-	-	ref|XP_003427849.1|	PREDICTED: hypothetical protein LOC100115099 [Nasonia vitripennis]	5,00E-34
gALL_v3_c26626	1	2	-	-	gb|ESO91319.1|	hypothetical protein LOTGIDRAFT_105202 [Lottia gigantea]	1,00E-07
gALL_v3_c26627	2	1	-	-	-	-	-
gALL_v3_c26629	3	1	-	-	-	-	-
gALL_v3_c2663	4	6	-	-	-	-	-
gALL_v3_c26631	1	3	-	-	dbj|BAH70664.1|	ACYPI33427 [Acyrthosiphon pisum]	2,00E-41
gALL_v3_c26632	1	3	-	-	-	-	-
gALL_v3_c26633	2	1	-	-	-	-	-
gALL_v3_c26634	3	1	gnl|CDD|187696	cd09272, RNase_HI_RT_Ty1, T	ref|XP_004498716.1|	PREDICTED: uncharacterized protein LOC101504373 [Cicer arietinum]	2,00E-06

gALL_v3_c26635	1	3	-	-	-	-	-
gALL_v3_c26636	3	3	-	-	-	-	-
gALL_v3_c26637	2	3	-	-	ref|XP_001602334.1|	PREDICTED: sulfhydryl oxidase 1-like [Nasonia vitripennis]	9,00E-18
gALL_v3_c26638	1	3	-	-	-	-	-
gALL_v3_c26639	1	3	gnl|CDD|146634	pfam04101, Glyco_tran_28_C,	ref|XP_005799535.1|	PREDICTED: UDP-N-acetylglucosamine transferase subunit ALG13 homolog isoform X1 [Xiphophorus maculatus]	1,00E-56
gALL_v3_c2664	11	24	gnl|CDD|162072	TIGR00860, LIC, Cation transp	ref|NP_001103244.1|	glutamate-gated chloride channel isoform c precursor [Tribolium castaneum] gb|ABU63601.1| glutamate-gated chloride channel splice variant 3c [Tribolium castaneum]	1,00E-174
gALL_v3_c26640	4	2	gnl|CDD|73156	cd01650, RT_nLTR_like, RT_nL	ref|XP_003243437.1|	PREDICTED: hypothetical protein LOC100572891 [Acyrthosiphon pisum]	3,00E-38
gALL_v3_c26641	0	4	gnl|CDD|148286	pfam06585, JHBP, Haemolymp	ref|XP_001607045.1|	PREDICTED: hypothetical protein LOC100123404 [Nasonia vitripennis]	5,00E-28
gALL_v3_c26642	2	2	-	-	-	-	-
gALL_v3_c26643	4	3	-	-	-	-	-
gALL_v3_c26644	1	2	-	-	-	-	-
gALL_v3_c26646	3	1	-	-	-	-	-
gALL_v3_c26647	3	1	-	-	gb|AGZ15181.1|	phosphoesterase 29, partial [Laodelphax striatella]	4,00E-26
gALL_v3_c26648	2	3	-	-	-	-	-
gALL_v3_c26649	2	1	-	-	-	-	-
gALL_v3_c2665	13	11	gnl|CDD|34633	COG5028, COG5028, Vesicle c	gb|EFN81313.1|	Protein transport protein Sec24C [Harpegnathos saltator]	0
gALL_v3_c26650	2	1	-	-	-	-	-
gALL_v3_c26651	1	3	-	-	-	-	-
gALL_v3_c26654	4	3	-	-	gb|AGM32550.1|	hypothetical protein [Coptotermes formosanus]	6,00E-82
gALL_v3_c26655	1	2	-	-	-	-	-
gALL_v3_c26656	2	4	gnl|CDD|129972	TIGR00894, 2A0114euk, Na(+)	ref|XP_003398671.1|	PREDICTED: putative inorganic phosphate cotransporter-like isoform 3 [Bombus terrestris]	2,00E-56
gALL_v3_c26657	2	1	-	-	-	-	-
gALL_v3_c26658	3	1	-	-	gb|EKC30645.1|	Protection of telomeres protein 1 [Crassostrea gigas]	5,00E-07
gALL_v3_c26659	1	3	-	-	-	-	-
gALL_v3_c26661	3	3	gnl|CDD|73156	cd01650, RT_nLTR_like, RT_nL	pir||B56679	probable reverse transcriptase - house mosquito gb|AAA28291.1| reverse transcriptase [Culex pipiens]	1,00E-30
gALL_v3_c26662	1	3	-	-	-	-	-
gALL_v3_c26663	1	6	-	-	-	-	-
gALL_v3_c26665	2	4	-	-	-	-	-
gALL_v3_c26666	0	3	-	-	-	-	-
gALL_v3_c26668	0	4	-	-	-	-	-
gALL_v3_c26669	3	3	-	-	-	-	-
gALL_v3_c2667	8	13	gnl|CDD|30091	cd01369, KISc_KHC_KIF5, Kine	ref|XP_001606707.2|	PREDICTED: kinesin heavy chain [Nasonia vitripennis]	1,00E-163
gALL_v3_c26670	7	5	-	-	ref|XP_968372.2|	PREDICTED: similar to atrophin-1 like protein [Tribolium castaneum]	4,00E-70
gALL_v3_c26672	2	4	-	-	-	-	-
gALL_v3_c26673	2	1	-	-	-	-	-
gALL_v3_c26674	2	2	-	-	-	-	-
gALL_v3_c26675	3	4	-	-	ref|XP_002427021.1|	protein purity of essence, putative [Pediculus humanus corporis] gb|EEB14283.1| protein purity of essence, putative [Pediculus humanus corporis]	5,00E-74
gALL_v3_c26677	0	5	gnl|CDD|147061	pfam04710, Pellino, Pellino. P	ref|XP_967308.1|	PREDICTED: similar to AGAP004232-PA isoform 1 [Tribolium castaneum]	2,00E-27
gALL_v3_c26679	2	3	gnl|CDD|173623	cd00180, PKc, Catalytic domai	gb|AFI39850.1|	pelle, partial [Daphnia pulex] gb|AFI39851.1| pelle, partial [Daphnia pulex] gb|AFI39852.1| pelle, partial [Daphnia pulex] gb|AFI39853.1| pelle, partial [Daphnia pulex] gb	3,00E-49
gALL_v3_c26680	0	5	-	-	-	-	-
gALL_v3_c26681	2	4	-	-	ref|XP_002425243.1|	Sperm-associated antigen, putative [Pediculus humanus corporis] gb|EEB12505.1| Sperm-associated antigen, putative [Pediculus humanus corporis]	1,00E-76
gALL_v3_c26682	5	4	-	-	ref|XP_002425259.1|	protein N-terminal asparagine amidohydrolase, putative [Pediculus humanus corporis] gb|EEB12521.1| protein N-terminal asparagine amidohydrolase, putative [Pediculus	3,00E-37
gALL_v3_c26683	1	3	-	-	-	-	-
gALL_v3_c26684	1	3	-	-	-	-	-
gALL_v3_c26685	3	5	-	-	ref|WP_007302981.1|	Ankyrin repeat domain protein [Wolbachia endosymbiont of Culex quinquefasciatus] gb|EEB55313.1| Ankyrin repeat domain protein [Wolbachia endosymbiont of Culex q	2,00E-09
gALL_v3_c26686	8	11	gnl|CDD|173456	PTZ00169, PTZ00169, ADP/ATI	gb|AGM32826.1|	ADP/ATP carrier protein 2, partial [Coptotermes formosanus]	4,00E-26
gALL_v3_c26687	3	1	-	-	-	-	-
gALL_v3_c26688	3	5	-	-	-	-	-
gALL_v3_c26689	1	2	-	-	-	-	-
gALL_v3_c2669	23	21	-	-	-	-	-
gALL_v3_c26691	0	3	-	-	-	-	-
gALL_v3_c26692	2	1	-	-	-	-	-
gALL_v3_c26693	1	2	-	-	-	-	-
gALL_v3_c26694	0	3	-	-	-	-	-
gALL_v3_c26695	2	3	-	-	-	-	-
gALL_v3_c26696	2	2	-	-	-	-	-
gALL_v3_c26697	0	4	-	-	-	-	-
gALL_v3_c26698	2	1	-	-	-	-	-
gALL_v3_c26699	2	4	-	-	-	-	-
gALL_v3_c2670	7	5	-	-	-	-	-
gALL_v3_c26700	1	6	-	-	-	-	-
gALL_v3_c26701	2	1	-	-	-	-	-
gALL_v3_c26702	0	4	-	-	ref|XP_969733.1|	PREDICTED: similar to NHL (ring finger b-box coiled coil) domain containing family member (nhl-3) [Tribolium castaneum] gb|EFA09351.1| hypothetical protein TcasGA2_T(	7,00E-65
gALL_v3_c26703	0	5	-	-	-	-	-
gALL_v3_c26704	3	1	-	-	-	-	-
gALL_v3_c26705	1	2	-	-	-	-	-
gALL_v3_c26706	7	8	-	-	-	-	-
gALL_v3_c26707	2	3	-	-	-	-	-
gALL_v3_c26708	1	4	-	-	-	-	-
gALL_v3_c26709	0	3	-	-	-	-	-
gALL_v3_c2671	18	29	-	-	-	-	-
gALL_v3_c26710	3	4	-	-	gb|EGI68906.1|	Mariner Mos1 transposase [Acromyrmex echinatior]	2,00E-09
gALL_v3_c26711	0	4	-	-	-	-	-
gALL_v3_c26712	4	3	gnl|CDD|128427	smart00119, HECTc, Domain H	ref|XP_006621472.1|	PREDICTED: ubiquitin-protein ligase E3C-like [Apis dorsata]	4,00E-17
gALL_v3_c26713	1	6	-	-	gb|EFN71403.1|	hypothetical protein EAG_00286 [Camponotus floridanus]	4,00E-13
gALL_v3_c26714	5	4	-	-	-	-	-
gALL_v3_c26715	2	2	-	-	ref|XP_005189864.1|	PREDICTED: transmembrane protein 132B-like, partial [Musca domestica]	3,00E-66
gALL_v3_c26716	0	3	-	-	-	-	-
gALL_v3_c26717	2	4	gnl|CDD|29261	cd00204, ANK, ankyrin repeat:	dbj|BAL61211.1|	fem-1 homolog A-like protein [Locusta migratoria manilensis]	1,00E-116
gALL_v3_c26718	3	1	-	-	-	-	-
gALL_v3_c26719	2	1	-	-	ref|XP_003247581.1|	PREDICTED: zinc finger protein 233-like [Acyrthosiphon pisum]	4,00E-34
gALL_v3_c2672	6	13	gnl|CDD|192610	pfam10510, PIG-S, Phosphatic	gb|EFZ14930.1|	hypothetical protein SINV_13263 [Solenopsis invicta]	1,00E-96
gALL_v3_c26720	2	1	-	-	-	-	-
gALL_v3_c26722	2	2	-	-	-	-	-
gALL_v3_c26723	3	5	-	-	-	-	-
gALL_v3_c26724	3	2	gnl|CDD|147872	pfam05955, Herpes_gp2, Equi	-	-	-
gALL_v3_c26725	2	2	-	-	-	-	-
gALL_v3_c26726	4	6	-	-	-	-	-
gALL_v3_c26727	3	2	-	-	ref|XP_003700384.1|	PREDICTED: LOW QUALITY PROTEIN: protein unc-79 homolog [Megachile rotundata]	6,00E-33
gALL_v3_c26728	1	2	gnl|CDD|30528	COG0179, MhpD, 2-keto-4-per	ref|XP_003701388.1|	PREDICTED: fumarylacetoacetate hydrolase domain-containing protein 2A-like isoform 1 [Megachile rotundata]	2,00E-45
gALL_v3_c26729	2	2	-	-	-	-	-
gALL_v3_c2673	11	16	gnl|CDD|188098	TIGR00957, MRP_assoc_pro, r	ref|XP_001123016.2|	PREDICTED: multidrug resistance-associated protein 1-like [Apis mellifera]	1,00E-51
gALL_v3_c26730	1	3	gnl|CDD|29010	cd00110, LamG, Laminin G do	ref|XP_003701896.1|	PREDICTED: pikachurin-like [Megachile rotundata]	2,00E-67
gALL_v3_c26731	1	7	-	-	-	-	-
gALL_v3_c26732	2	3	-	-	-	-	-
gALL_v3_c26733	0	4	-	-	-	-	-
gALL_v3_c26734	4	5	-	-	-	-	-
gALL_v3_c26735	1	2	-	-	-	-	-
gALL_v3_c26737	2	1	-	-	-	-	-
gALL_v3_c26738	1	4	-	-	gb|EFA05569.1|	hypothetical protein TcasGA2_TC015758 [Tribolium castaneum]	7,00E-38
gALL_v3_c26739	0	3	-	-	-	-	-
gALL_v3_c2674	6	4	-	-	-	-	-
gALL_v3_c26740	6	6	-	-	-	-	-
gALL_v3_c26741	3	1	gnl|CDD|143803	pfam00012, HSP70, Hsp70 pro	dbj|BAN21201.1|	hypothetical protein [Riptortus pedestris]	1,00E-129
gALL_v3_c26742	2	1	-	-	-	-	-
gALL_v3_c26743	0	4	-	-	-	-	-
gALL_v3_c26744	1	3	gnl|CDD|63873	cd04404, RhoGAP-p50rhoGAP,	ref|XP_001604462.2|	PREDICTED: rho GTPase-activating protein 1-like [Nasonia vitripennis]	1,00E-62
gALL_v3_c26745	1	2	-	-	ref|XP_003492266.1|	PREDICTED: hypothetical protein LOC100745871 [Bombus impatiens]	5,00E-78
gALL_v3_c26746	1	2	-	-	-	-	-
gALL_v3_c26748	1	2	-	-	ref|XP_006775986.1|	PREDICTED: piggyBac transposable element-derived protein 3-like [Myotis davidii]	1,00E-35
gALL_v3_c26749	2	1	-	-	-	-	-
gALL_v3_c2675	12	10	-	-	gb|EFZ13522.1|	hypothetical protein SINV_04974 [Solenopsis invicta]	0
gALL_v3_c26750	2	2	-	-	-	-	-
gALL_v3_c26751	1	5	gnl|CDD|162147	TIGR00990, 3a0801s09, mitoc	ref|XP_002427796.1|	mitochondrial protein import receptor, putative [Pediculus humanus corporis] gb|EEB15058.1| mitochondrial protein import receptor, putative [Pediculus humanus corpo	5,00E-45
gALL_v3_c26753	2	1	-	-	-	-	-
gALL_v3_c26754	0	5	-	-	-	-	-
gALL_v3_c26755	2	4	-	-	gb|EFZ12814.1|	hypothetical protein SINV_13578 [Solenopsis invicta]	6,00E-11
gALL_v3_c26758	1	4	-	-	-	-	-
gALL_v3_c26759	4	2	-	-	-	-	-
gALL_v3_c2676	14	24	-	-	-	-	-
gALL_v3_c26760	2	1	gnl|CDD|73156	cd01650, RT_nLTR_like, RT_nL	gb|ERL93526.1|	hypothetical protein D910_10815 [Dendroctonus ponderosae]	9,00E-13
gALL_v3_c26761	0	4	-	-	-	-	-
gALL_v3_c26762	1	3	-	-	-	-	-
gALL_v3_c26763	1	3	-	-	-	-	-
gALL_v3_c26764	1	2	gnl|CDD|34495	COG4886, COG4886, Leucine-	gb|EFX71363.1|	hypothetical protein DAPPUDRAFT_60262 [Daphnia pulex]	2,00E-31
gALL_v3_c26765	3	4	-	-	-	-	-
gALL_v3_c26766	1	3	-	-	-	-	-
gALL_v3_c26768	3	3	-	-	-	-	-
gALL_v3_c26769	0	3	-	-	-	-	-
gALL_v3_c26770	0	3	-	-	-	-	-
gALL_v3_c26771	3	1	-	-	-	-	-
gALL_v3_c26772	0	3	-	-	-	-	-
gALL_v3_c26774	3	5	-	-	-	-	-
gALL_v3_c26775	2	2	-	-	-	-	-
gALL_v3_c26776	2	1	-	-	-	-	-
gALL_v3_c26777	7	4	-	-	-	-	-
gALL_v3_c26778	2	5	-	-	ref|XP_001506733.2|	PREDICTED: mitogen-activated protein kinase kinase kinase 1-like [Ornithorhynchus anatinus]	2,00E-21
gALL_v3_c26779	1	3	-	-	ref|XP_002426261.1|	conserved hypothetical protein [Pediculus humanus corporis] gb|EEB13523.1| conserved hypothetical protein [Pediculus humanus corporis]	2,00E-45
gALL_v3_c2678	12	25	gnl|CDD|34495	COG4886, COG4886, Leucine-	gb|EFN83431.1|	Protein flightless-1 [Harpegnathos saltator]	0
gALL_v3_c26781	2	1	-	-	-	-	-
gALL_v3_c26782	3	1	-	-	-	-	-
gALL_v3_c26783	3	4	-	-	-	-	-
gALL_v3_c26785	0	3	-	-	-	-	-
gALL_v3_c26786	2	3	gnl|CDD|99986	cd03816, GT1_ALG1_like, This	ref|XP_001601332.2|	PREDICTED: chitobiosyldiphosphodolichol beta-mannosyltransferase-like [Nasonia vitripennis]	3,00E-97

gALL_v3_c26787	2	5	-	-	ref|XP_003243437.1|	PREDICTED: hypothetical protein LOC100572891 [Acyrthosiphon pisum]	2,00E-11
gALL_v3_c26788	0	3	-	-	-	-	-
gALL_v3_c26789	2	2	-	-	-	-	-
gALL_v3_c2679	6	4	-	-	-	-	-
gALL_v3_c26790	2	2	gnl|CDD|190227	pfam02148, zf-UBP, Zn-finger i	ref|XP_002431644.1|	histone deacetylase hda2, putative [Pediculus humanus corporis] gb|EEB18906.1| histone deacetylase hda2, putative [Pediculus humanus corporis]	1,00E-59
gALL_v3_c26792	1	3	gnl|CDD|189502	pfam00326, Peptidase_S9, Pro	ref|XP_006558844.1|	PREDICTED: dipeptidyl aminopeptidase-like protein 6-like isoform X1 [Apis mellifera]	4,00E-98
gALL_v3_c26793	2	2	-	-	-	-	-
gALL_v3_c26794	1	2	-	-	ref|XP_002424409.1|	predicted protein [Pediculus humanus corporis] gb|EEB11671.1| predicted protein [Pediculus humanus corporis]	2,00E-11
gALL_v3_c26795	2	2	-	-	-	-	-
gALL_v3_c26796	3	1	-	-	-	-	-
gALL_v3_c26798	3	2	-	-	-	-	-
gALL_v3_c268	9	21	-	-	-	-	-
gALL_v3_c2680	5	2	gnl|CDD|143939	pfam00173, Cyt-b5, Cytochron	pdb|3OZZ|B	Chain B, Structure Of A Cytochrome B5 Core-Swap Mutant	3,00E-22
gALL_v3_c26800	3	1	-	-	-	-	-
gALL_v3_c26801	3	1	-	-	-	-	-
gALL_v3_c26802	0	3	-	-	-	-	-
gALL_v3_c26803	3	2	-	-	-	-	-
gALL_v3_c26804	3	5	gnl|CDD|189958	pfam01369, Sec7, Sec7 domai	ref|XP_002425320.1|	Brefeldin A-inhibited guanine nucleotide-exchange protein, putative [Pediculus humanus corporis] gb|EEB12582.1| Brefeldin A-inhibited guanine nucleotide-exchange prot	1,00E-124
gALL_v3_c26805	0	4	-	-	gb|EFN76211.1|	Mediator of RNA polymerase II transcription subunit 12 [Harpegnathos saltator]	6,00E-13
gALL_v3_c26806	0	3	-	-	-	-	-
gALL_v3_c26807	2	6	-	-	-	-	-
gALL_v3_c26808	1	2	-	-	-	-	-
gALL_v3_c26809	1	3	-	-	gb|EFN86018.1|	WD repeat-containing protein 32-like protein [Harpegnathos saltator]	2,00E-36
gALL_v3_c2681	15	19	-	-	-	-	-
gALL_v3_c26810	1	2	-	-	-	-	-
gALL_v3_c26811	5	10	-	-	-	-	-
gALL_v3_c26812	3	1	-	-	-	-	-
gALL_v3_c26813	2	4	gnl|CDD|128481	smart00184, RING, Ring finger	ref|XP_968664.1|	PREDICTED: similar to CG13605 CG13605-PA [Tribolium castaneum] gb|EEZ99887.1| hypothetical protein TcasGA2_TC002671 [Tribolium castaneum]	9,00E-65
gALL_v3_c26814	3	3	-	-	-	-	-
gALL_v3_c26815	2	1	-	-	-	-	-
gALL_v3_c26817	2	2	-	-	-	-	-
gALL_v3_c26818	3	1	gnl|CDD|145103	pfam01770, Folate_carrier, Re	gb|EFZ14695.1|	hypothetical protein SINV_01155 [Solenopsis invicta]	1,00E-37
gALL_v3_c26819	2	4	gnl|CDD|30181	cd01994, Alpha_ANH_like_IV,	ref|XP_001949226.2|	PREDICTED: meiotically up-regulated gene 71 protein-like [Acyrthosiphon pisum]	2,00E-64
gALL_v3_c26820	0	3	-	-	gb|EHJ68992.1|	putative beat protein [Danaus plexippus]	1,00E-16
gALL_v3_c26821	2	1	-	-	-	-	-
gALL_v3_c26822	2	4	-	-	-	-	-
gALL_v3_c26823	2	1	-	-	-	-	-
gALL_v3_c26824	2	1	-	-	-	-	-
gALL_v3_c26825	0	6	gnl|CDD|133006	cd02513, CMP-NeuAc_Syntha:	ref|NP_001156112.1|	cmp-n-acetylneuraminic acid synthase-like [Acyrthosiphon pisum] dbj|BAH72225.1| ACYPI002677 [Acyrthosiphon pisum]	1,00E-41
gALL_v3_c26826	3	2	-	-	-	-	-
gALL_v3_c26827	3	1	-	-	-	-	-
gALL_v3_c26828	1	7	-	-	-	-	-
gALL_v3_c26829	0	3	-	-	-	-	-
gALL_v3_c2683	5	7	-	-	-	-	-
gALL_v3_c26830	1	3	-	-	ref|XP_002425686.1|	hypothetical protein Phum_PHUM215460 [Pediculus humanus corporis] gb|EEB12948.1| hypothetical protein Phum_PHUM215460 [Pediculus humanus corporis]	2,00E-09
gALL_v3_c26831	1	3	-	-	-	-	-
gALL_v3_c26832	2	1	-	-	-	-	-
gALL_v3_c26833	2	1	-	-	-	-	-
gALL_v3_c26834	0	4	-	-	-	-	-
gALL_v3_c26835	0	2	gnl|CDD|153340	cd07656, F-BAR_srGAP, The F-	ref|XP_002424891.1|	SLIT-ROBO Rho GTPase-activating protein, putative [Pediculus humanus corporis] gb|EEB12153.1| SLIT-ROBO Rho GTPase-activating protein, putative [Pediculus humanus	5,00E-42
gALL_v3_c26836	2	6	-	-	-	-	-
gALL_v3_c26837	2	1	-	-	-	-	-
gALL_v3_c26838	2	1	-	-	-	-	-
gALL_v3_c26839	0	4	-	-	gb|ACZ26466.1|	Down syndrome cell adhesion molecule [Litopenaeus vannamei]	2,00E-21
gALL_v3_c2684	17	33	-	-	-	-	-
gALL_v3_c26840	2	1	-	-	-	-	-
gALL_v3_c26842	2	1	gnl|CDD|145882	pfam02958, DUF227, Domain	gb|EFN72316.1|	hypothetical protein EAG_02913 [Camponotus floridanus]	2,00E-19
gALL_v3_c26843	0	4	-	-	-	-	-
gALL_v3_c26844	1	3	-	-	-	-	-
gALL_v3_c26845	0	4	-	-	-	-	-
gALL_v3_c26846	1	3	-	-	-	-	-
gALL_v3_c26847	2	1	-	-	-	-	-
gALL_v3_c26849	2	3	gnl|CDD|48589	cd03040, GST_N_mPGES2, GS'	gb|ERL87353.1|	hypothetical protein D910_04748 [Dendroctonus ponderosae]	1,00E-55
gALL_v3_c26850	5	2	-	-	-	-	-
gALL_v3_c26851	2	2	-	-	-	-	-
gALL_v3_c26852	2	2	-	-	-	-	-
gALL_v3_c26853	0	4	-	-	-	-	-
gALL_v3_c26854	3	2	gnl|CDD|150848	pfam10237, N6-adenineMlase	ref|XP_002599123.1|	hypothetical protein BRAFLDRAFT_225067 [Branchiostoma floridae] gb|EEN55135.1| hypothetical protein BRAFLDRAFT_225067 [Branchiostoma floridae]	7,00E-22
gALL_v3_c26856	3	3	-	-	-	-	-
gALL_v3_c26857	2	1	gnl|CDD|128471	smart00174, RHO, Rho (Ras hc	gb|AGM32255.1|	Rac 1-related protein [Coptotermes formosanus]	1,00E-80
gALL_v3_c26858	2	6	-	-	gb|ESO08009.1|	hypothetical protein HELRODRAFT_75213 [Helobdella robusta]	1,00E-25
gALL_v3_c26859	0	3	-	-	ref|XP_966785.2|	PREDICTED: similar to munin CG33518-PA [Tribolium castaneum]	9,00E-09
gALL_v3_c2686	4	7	gnl|CDD|149033	pfam07742, BTG, BTG family.	ref|XP_006617577.1|	PREDICTED: protein Tob1-like [Apis dorsata]	1,00E-102
gALL_v3_c26860	2	2	-	-	-	-	-
gALL_v3_c26861	2	4	-	-	-	-	-
gALL_v3_c26862	4	11	-	-	ref|XP_006569403.1|	PREDICTED: ankyrin repeat and KH domain-containing protein mask [Apis mellifera]	9,00E-37
gALL_v3_c26863	2	1	-	-	-	-	-
gALL_v3_c26864	3	1	-	-	-	-	-
gALL_v3_c26865	2	1	gnl|CDD|162296	TIGR01311, glycerol_kin, glyce	gb|AGM32757.1|	putative glycerol kinase 3-like protein, partial [Coptotermes formosanus]	3,00E-50
gALL_v3_c26866	2	3	-	-	-	-	-
gALL_v3_c26867	2	1	-	-	-	-	-
gALL_v3_c26868	1	4	-	-	-	-	-
gALL_v3_c2687	4	4	-	-	-	-	-
gALL_v3_c26871	5	2	-	-	-	-	-
gALL_v3_c26872	2	2	-	-	-	-	-
gALL_v3_c26873	1	3	-	-	-	-	-
gALL_v3_c26874	3	2	-	-	-	-	-
gALL_v3_c26875	3	1	-	-	-	-	-
gALL_v3_c26876	2	2	-	-	-	-	-
gALL_v3_c26877	0	4	-	-	-	-	-
gALL_v3_c26878	0	3	-	-	-	-	-
gALL_v3_c26879	2	4	-	-	-	-	-
gALL_v3_c2688	22	15	gnl|CDD|179965	PRK05218, PRK05218, heat sh	gb|ACS75351.1|	endoplasmin [Locusta migratoria]	0
gALL_v3_c26880	4	4	-	-	emb|CAF88477.1|	unnamed protein product [Tetraodon nigroviridis]	1,00E-15
gALL_v3_c26881	2	2	-	-	-	-	-
gALL_v3_c26882	1	2	-	-	-	-	-
gALL_v3_c26883	2	1	-	-	gb|EFN60254.1|	hypothetical protein EAG_03219 [Camponotus floridanus]	2,00E-13
gALL_v3_c26884	0	3	-	-	ref|XP_001605769.2|	PREDICTED: echinoderm microtubule-associated protein-like CG42247-like [Nasonia vitripennis]	5,00E-21
gALL_v3_c26885	1	7	-	-	gb|EFZ12234.1|	hypothetical protein SINV_00931 [Solenopsis invicta]	3,00E-49
gALL_v3_c26886	2	3	-	-	-	-	-
gALL_v3_c26887	1	4	-	-	-	-	-
gALL_v3_c26888	1	3	-	-	-	-	-
gALL_v3_c26889	0	3	-	-	-	-	-
gALL_v3_c2689	3	7	-	-	-	-	-
gALL_v3_c26892	3	3	-	-	-	-	-
gALL_v3_c26893	2	2	-	-	-	-	-
gALL_v3_c26894	5	8	gnl|CDD|177628	PHA03392, egt, ecdysteroid UI	gb|AGM32626.1|	glucosyl/glucuronosyl transferase, partial [Coptotermes formosanus]	1,00E-63
gALL_v3_c26896	2	1	-	-	-	-	-
gALL_v3_c26897	3	2	gnl|CDD|129972	TIGR00894, 2A0114euk, Na(+)	ref|XP_003704060.1|	PREDICTED: putative inorganic phosphate cotransporter-like [Megachile rotundata]	1,00E-19
gALL_v3_c26898	3	1	-	-	ref|XP_001657920.1|	n-acetyllactosaminide beta-1,3-n-acetylglucosaminyltransferase [Aedes aegypti] gb|EAT41759.1| AAEL006638-PA [Aedes aegypti]	1,00E-121
gALL_v3_c26899	1	5	-	-	-	-	-
gALL_v3_c269	14	9	-	-	-	-	-
gALL_v3_c2690	5	11	-	-	gb|AGM32453.1|	heat shock protein-like protein [Coptotermes formosanus]	6,00E-31
gALL_v3_c26901	2	3	-	-	-	-	-
gALL_v3_c26902	3	3	-	-	-	-	-
gALL_v3_c26903	3	4	-	-	-	-	-
gALL_v3_c26904	1	2	gnl|CDD|178473	PLN02885, PLN02885, nicotina	ref|XP_005176326.1|	PREDICTED: nicotinate phosphoribosyltransferase-like isoform X2 [Musca domestica]	3,00E-42
gALL_v3_c26905	2	3	-	-	-	-	-
gALL_v3_c26906	2	2	-	-	-	-	-
gALL_v3_c26908	5	3	gnl|CDD|132729	cd06931, NR_LBD_HNF4_like,	ref|XP_968613.2|	PREDICTED: similar to hepatocyte nuclear factor 4 [Tribolium castaneum]	3,00E-93
gALL_v3_c26909	3	1	-	-	-	-	-
gALL_v3_c26910	4	6	-	-	-	-	-
gALL_v3_c26911	1	5	gnl|CDD|146069	pfam03250, Tropomodulin, Tro	ref|XP_006560804.1|	PREDICTED: tropomodulin isoform X10 [Apis mellifera] ref|XP_006560805.1| PREDICTED: tropomodulin isoform X11 [Apis mellifera]	1,00E-88
gALL_v3_c26912	2	1	-	-	-	-	-
gALL_v3_c26913	0	4	-	-	-	-	-
gALL_v3_c26914	0	4	-	-	ref|XP_002425902.1|	ubiquitin specific protease, putative [Pediculus humanus corporis] gb|EEB13164.1| ubiquitin specific protease, putative [Pediculus humanus corporis]	3,00E-42
gALL_v3_c26915	1	2	-	-	ref|XP_003427125.1|	PREDICTED: zinc finger protein Xfin-like [Nasonia vitripennis]	7,00E-13
gALL_v3_c26917	1	5	-	-	-	-	-
gALL_v3_c26918	1	4	-	-	ref|XP_001605068.2|	PREDICTED: pleckstrin homology domain-containing family F member 2-like [Nasonia vitripennis]	5,00E-06
gALL_v3_c26919	4	2	-	-	-	-	-
gALL_v3_c2692	3	7	gnl|CDD|188151	TIGR01517, ATPase-IIB_Ca, pla	ref|XP_002423093.1|	plasma membrane ca-ATPase B2, putative [Pediculus humanus corporis] gb|EEB10355.1| plasma membrane ca-ATPase B2, putative [Pediculus humanus corporis]	3,00E-51
gALL_v3_c26920	0	4	-	-	-	-	-
gALL_v3_c26921	2	3	-	-	-	-	-
gALL_v3_c26922	0	4	-	-	-	-	-
gALL_v3_c26923	2	4	-	-	-	-	-
gALL_v3_c26925	2	2	-	-	-	-	-
gALL_v3_c26927	0	3	-	-	-	-	-
gALL_v3_c26928	1	6	gnl|CDD|128491	smart00194, PTPc, Protein tyr	ref|XP_002424545.1|	conserved hypothetical protein [Pediculus humanus corporis] gb|EEB11807.1| conserved hypothetical protein [Pediculus humanus corporis]	7,00E-60
gALL_v3_c26929	5	2	-	-	ref|XP_003702088.1|	PREDICTED: uncharacterized protein LOC100877727 [Megachile rotundata]	3,00E-17
gALL_v3_c2693	5	3	-	-	-	-	-
gALL_v3_c26930	2	2	-	-	ref|XP_966699.2|	PREDICTED: similar to CG2747 CG2747-PB [Tribolium castaneum]	3,00E-64
gALL_v3_c26931	3	2	-	-	-	-	-
gALL_v3_c26932	0	5	-	-	ref|XP_004681516.1|	PREDICTED: transmembrane protease serine 11E [Condylura cristata]	2,00E-07
gALL_v3_c26933	3	1	gnl|CDD|28913	cd00031, CA, Cadherin repeat	ref|XP_971084.2|	PREDICTED: similar to AGAP007924-PA [Tribolium castaneum]	3,00E-69

gALL_v3_c26934	2	4	-	-	-	-	-
gALL_v3_c26935	1	3	-	-	ref|XP_005187220.1|	PREDICTED: protein tramtrack, beta isoform-like isoform X4 [Musca domestica]	4,00E-28
gALL_v3_c26936	5	14	-	-	-	-	-
gALL_v3_c26937	2	1	-	-	-	-	-
gALL_v3_c26939	2	4	-	-	gb|EFN67634.1|	Putative uncharacterized transposon-derived protein F52C9.6 [Camponotus floridanus]	4,00E-11
gALL_v3_c2694	9	10	gnl|CDD|147785	pfam05822, UMPH-1, Pyrimid	ref|XP_001603192.1|	PREDICTED: cytosolic 5'-nucleotidase 3-like [Nasonia vitripennis]	2,00E-63
gALL_v3_c26940	1	3	-	-	ref|XP_003401448.1|	PREDICTED: max-like protein X-like [Bombus terrestris] ref|XP_003487455.1| PREDICTED: max-like protein X-like [Bombus impatiens]	3,00E-30
gALL_v3_c26941	0	3	-	-	-	-	-
gALL_v3_c26942	1	5	-	-	-	-	-
gALL_v3_c26943	5	6	-	-	-	-	-
gALL_v3_c26944	3	2	-	-	-	-	-
gALL_v3_c26945	2	1	-	-	-	-	-
gALL_v3_c26946	2	2	-	-	-	-	-
gALL_v3_c26947	6	4	-	-	-	-	-
gALL_v3_c26948	1	2	-	-	-	-	-
gALL_v3_c26949	1	4	-	-	-	-	-
gALL_v3_c26950	2	2	-	-	ref|XP_003250312.1|	PREDICTED: alpha-tocopherol transfer protein-like [Apis mellifera]	3,00E-19
gALL_v3_c26951	1	4	gnl|CDD|153286	cd07602, BAR_RhoGAP_OPHN	ref|XP_003693893.1|	PREDICTED: LOW QUALITY PROTEIN: rho GTPase-activating protein 26-like [Apis florea]	2,00E-65
gALL_v3_c26952	4	3	-	-	-	-	-
gALL_v3_c26953	0	3	-	-	-	-	-
gALL_v3_c26954	1	4	-	-	-	-	-
gALL_v3_c26955	0	3	-	-	-	-	-
gALL_v3_c26956	0	3	gnl|CDD|29257	cd00200, WD40, WD40 domai	ref|XP_003485793.1|	PREDICTED: p21-activated protein kinase-interacting protein 1-like [Bombus impatiens]	2,00E-51
gALL_v3_c26958	2	1	-	-	gb|EGI57636.1|	Putative aminopeptidase W07G4.4 [Acromyrmex echinatior]	2,00E-17
gALL_v3_c2696	5	4	-	-	gb|ERL93178.1|	hypothetical protein D910_10475 [Dendroctonus ponderosae]	2,00E-20
gALL_v3_c26961	3	2	-	-	-	-	-
gALL_v3_c26962	2	2	-	-	-	-	-
gALL_v3_c26963	0	3	-	-	-	-	-
gALL_v3_c26964	2	7	-	-	-	-	-
gALL_v3_c26965	3	8	-	-	-	-	-
gALL_v3_c26966	0	4	gnl|CDD|162465	TIGR01652, ATPase-Plipid, pho	ref|XP_003485770.1|	PREDICTED: probable phospholipid-transporting ATPase IF-like [Bombus impatiens]	3,00E-29
gALL_v3_c26967	1	2	-	-	ref|XP_001605942.2|	PREDICTED: male-specific lethal 2 homolog [Nasonia vitripennis]	1,00E-14
gALL_v3_c26968	0	4	-	-	-	-	-
gALL_v3_c26969	2	2	-	-	-	-	-
gALL_v3_c2697	5	9	-	-	-	-	-
gALL_v3_c26970	1	2	-	-	-	-	-
gALL_v3_c26973	6	4	-	-	-	-	-
gALL_v3_c26974	0	4	-	-	-	-	-
gALL_v3_c26975	2	2	-	-	-	-	-
gALL_v3_c26976	0	5	-	-	-	-	-
gALL_v3_c26977	6	4	gnl|CDD|143945	pfam00179, UQ_con, Ubiquiti	ref|XP_002432721.1|	ubiquitin-conjugating enzyme E2-17 kDa, putative [Pediculus humanus corporis] gb|EEB19983.1| ubiquitin-conjugating enzyme E2-17 kDa, putative [Pediculus humanus cc	2,00E-41
gALL_v3_c26978	2	3	-	-	-	-	-
gALL_v3_c26979	2	2	-	-	-	-	-
gALL_v3_c26980	2	4	-	-	-	-	-
gALL_v3_c26981	1	2	-	-	-	-	-
gALL_v3_c26983	2	1	-	-	ref|XP_003705866.1|	PREDICTED: DNA-directed RNA polymerase I subunit RPA1-like [Megachile rotundata]	1,00E-18
gALL_v3_c26984	8	4	-	-	-	-	-
gALL_v3_c26985	1	2	-	-	-	-	-
gALL_v3_c26986	4	2	-	-	-	-	-
gALL_v3_c26987	1	3	-	-	-	-	-
gALL_v3_c26988	9	5	-	-	-	-	-
gALL_v3_c26989	8	4	-	-	-	-	-
gALL_v3_c2699	22	20	gnl|CDD|34853	COG5256, TEF1, Translation el	ref|XP_002425558.1|	eukaryotic peptide chain release factor GTP-binding subunit, putative [Pediculus humanus corporis] gb|EEB12820.1| eukaryotic peptide chain release factor GTP-binding su	1,00E-179
gALL_v3_c26990	3	1	-	-	gb|ESO03183.1|	hypothetical protein HELRODRAFT_174081 [Helobdella robusta]	1,00E-24
gALL_v3_c26991	1	2	-	-	-	-	-
gALL_v3_c26992	2	2	-	-	-	-	-
gALL_v3_c26993	1	2	-	-	-	-	-
gALL_v3_c26994	1	6	gnl|CDD|109812	pfam00769, ERM, Ezrin/radixi	ref|XP_006559571.1|	PREDICTED: merlin isoform X3 [Apis mellifera] ref|XP_006607367.1| PREDICTED: merlin-like isoform X3 [Apis dorsata]	1,00E-92
gALL_v3_c26995	3	4	-	-	-	-	-
gALL_v3_c26996	7	4	-	-	gb|AGM32817.1|	protein of unknown function (DUF423) [Coptotermes formosanus]	2,00E-55
gALL_v3_c26997	2	2	-	-	-	-	-
gALL_v3_c26998	0	4	-	-	-	-	-
gALL_v3_c26999	1	2	-	-	-	-	-
gALL_v3_c27	6	4	-	-	-	-	-
gALL_v3_c2700	6	6	-	-	-	-	-
gALL_v3_c27002	2	1	-	-	-	-	-
gALL_v3_c27003	1	3	-	-	-	-	-
gALL_v3_c27004	6	4	gnl|CDD|73156	cd01650, RT_nLTR_like, RT_nL	ref|XP_004205527.1|	PREDICTED: RNA-directed DNA polymerase from mobile element jockey-like [Hydra vulgaris]	1,00E-28
gALL_v3_c27005	1	3	-	-	-	-	-
gALL_v3_c27006	2	1	-	-	-	-	-
gALL_v3_c27007	5	2	-	-	-	-	-
gALL_v3_c27008	1	6	-	-	-	-	-
gALL_v3_c27009	4	11	-	-	-	-	-
gALL_v3_c2701	1	7	-	-	-	-	-
gALL_v3_c27010	23	21	gnl|CDD|153114	cd01056, Euk_Ferritin, eukary	gb|AGM32322.1|	ferritin-like precursor, partial [Coptotermes formosanus]	2,00E-59
gALL_v3_c27011	3	1	-	-	-	-	-
gALL_v3_c27012	2	5	gnl|CDD|128621	smart00326, SH3, Src homolo	ref|XP_006561990.1|	PREDICTED: cytoplasmic protein NCK1 isoform X2 [Apis mellifera] ref|XP_006561991.1| PREDICTED: cytoplasmic protein NCK1 isoform X3 [Apis mellifera] ref|XP_0066094	1,00E-118
gALL_v3_c27013	2	2	gnl|CDD|153286	cd07602, BAR_RhoGAP_OPHN	gb|EGI63097.1|	Rho GTPase-activating protein 26 [Acromyrmex echinatior]	2,00E-47
gALL_v3_c27014	1	4	-	-	-	-	-
gALL_v3_c27015	1	3	-	-	-	-	-
gALL_v3_c27016	2	1	-	-	-	-	-
gALL_v3_c27017	1	3	-	-	-	-	-
gALL_v3_c27018	1	3	-	-	-	-	-
gALL_v3_c2702	7	7	gnl|CDD|146187	pfam03417, AAT, Acyl-coenzyr	ref|XP_971848.1|	PREDICTED: similar to tan CG12120-PA [Tribolium castaneum] gb|EFA00578.1| hypothetical protein TcasGA2_TC003448 [Tribolium castaneum]	1,00E-125
gALL_v3_c27020	1	4	-	-	-	-	-
gALL_v3_c27021	2	2	-	-	dbj|BAL61212.1|	fem-1 homolog B-like protein [Locusta migratoria manilensis]	9,00E-36
gALL_v3_c27022	1	4	-	-	-	-	-
gALL_v3_c27023	1	3	-	-	-	-	-
gALL_v3_c27024	6	10	-	-	gb|ESP03464.1|	hypothetical protein LOTGIDRAFT_171401 [Lottia gigantea]	1,00E-09
gALL_v3_c27026	3	3	-	-	-	-	-
gALL_v3_c27027	3	1	-	-	gb|AGM32899.1|	Von Willebrand factor type A (vWA) domain containing protein [Coptotermes formosanus]	5,00E-09
gALL_v3_c27028	2	2	-	-	-	-	-
gALL_v3_c27029	1	3	-	-	ref|XP_006617828.1|	PREDICTED: protein still life, isoforms C/SIF type 2-like isoform X6 [Apis dorsata]	5,00E-33
gALL_v3_c2703	16	29	-	-	ref|XP_002425428.1|	conserved hypothetical protein [Pediculus humanus corporis] gb|EEB12690.1| conserved hypothetical protein [Pediculus humanus corporis]	5,00E-08
gALL_v3_c27030	2	2	-	-	-	-	-
gALL_v3_c27031	0	4	-	-	-	-	-
gALL_v3_c27032	0	3	-	-	gb|AFP58804.1|	dynactin subunit 4 [Apis cerana cerana] gb|AFP58805.1| dynactin subunit 4 [Apis cerana cerana]	9,00E-35
gALL_v3_c27033	7	5	gnl|CDD|34770	COG5170, CDC55, Serine/thre	ref|XP_003426482.1|	PREDICTED: protein phosphatase PP2A 55 kDa regulatory subunit-like isoform 2 [Nasonia vitripennis]	1,00E-105
gALL_v3_c27034	0	3	-	-	emb|CAR82249.1|	hypothetical protein [Cotesia congregata]	2,00E-31
gALL_v3_c27035	6	4	-	-	-	-	-
gALL_v3_c27036	0	6	-	-	-	-	-
gALL_v3_c27037	5	2	-	-	-	-	-
gALL_v3_c27038	1	4	-	-	gb|EFN79861.1|	hypothetical protein EAI_11067 [Harpegnathos saltator]	1,00E-36
gALL_v3_c27039	0	6	gnl|CDD|144648	pfam01130, CD36, CD36 famil	ref|XP_002428137.1|	scavenger receptor class B member, putative [Pediculus humanus corporis] gb|EEB15399.1| scavenger receptor class B member, putative [Pediculus humanus corporis]	3,00E-44
gALL_v3_c2704	5	5	-	-	gb|ETN62877.1|	SEC14 [Anopheles darlingi]	6,00E-26
gALL_v3_c27040	3	1	-	-	-	-	-
gALL_v3_c27041	1	2	-	-	gb|EFA11632.1|	hypothetical protein TcasGA2_TC000010 [Tribolium castaneum]	1,00E-05
gALL_v3_c27042	0	3	-	-	-	-	-
gALL_v3_c27043	1	2	-	-	-	-	-
gALL_v3_c27044	3	3	gnl|CDD|34716	COG5113, UFD2, Ubiquitin fus	ref|XP_002426709.1|	ubiquitin conjugation factor E4 B, putative [Pediculus humanus corporis] gb|EEB13971.1| ubiquitin conjugation factor E4 B, putative [Pediculus humanus corporis]	3,00E-92
gALL_v3_c27046	0	6	-	-	gb|EGI58156.1|	Mediator of RNA polymerase II transcription subunit 16 [Acromyrmex echinatior]	7,00E-58
gALL_v3_c27047	0	3	-	-	-	-	-
gALL_v3_c27048	2	3	-	-	-	-	-
gALL_v3_c27049	1	4	-	-	ref|XP_970784.1|	PREDICTED: similar to desert CG32025-PB [Tribolium castaneum]	1,00E-10
gALL_v3_c2705	3	7	-	-	-	-	-
gALL_v3_c27050	3	1	-	-	-	-	-
gALL_v3_c27051	0	4	-	-	-	-	-
gALL_v3_c27052	1	3	-	-	-	-	-
gALL_v3_c27054	0	4	-	-	-	-	-
gALL_v3_c27055	1	2	-	-	-	-	-
gALL_v3_c27056	1	3	-	-	-	-	-
gALL_v3_c27058	1	3	gnl|CDD|192544	pfam10347, Fmp27_GFWDK, I	gb|EFA07873.1|	hypothetical protein TcasGA2_TC005447 [Tribolium castaneum]	7,00E-63
gALL_v3_c27059	4	3	-	-	-	-	-
gALL_v3_c2706	7	4	gnl|CDD|128656	smart00362, RRM_2, RNA reco	ref|XP_001605411.1|	PREDICTED: serine/arginine-rich splicing factor 1-like [Nasonia vitripennis]	8,00E-99
gALL_v3_c27060	3	1	-	-	gb|AGH30328.1|	argonaute-3 [Nilaparvata lugens]	8,00E-08
gALL_v3_c27061	1	5	-	-	-	-	-
gALL_v3_c27063	3	5	-	-	-	-	-
gALL_v3_c27064	1	2	-	-	-	-	-
gALL_v3_c27065	0	3	-	-	-	-	-
gALL_v3_c27066	3	1	gnl|CDD|191250	pfam05282, AAR2, AAR2 prote	gb|EFN66286.1|	Uncharacterized protein C20orf4-like protein [Camponotus floridanus]	2,00E-34
gALL_v3_c27067	2	2	-	-	ref|XP_001659904.1|	mitochondrial ribosomal protein, L48, putative [Aedes aegypti] gb|EAT38858.1| AAEL009288-PA [Aedes aegypti]	7,00E-50
gALL_v3_c27068	2	2	-	-	-	-	-
gALL_v3_c27069	4	6	gnl|CDD|29257	cd00200, WD40, WD40 domai	ref|XP_002431665.1|	F-box/WD-repeat protein, putative [Pediculus humanus corporis] gb|EEB18927.1| F-box/WD-repeat protein, putative [Pediculus humanus corporis]	0
gALL_v3_c27070	4	4	-	-	-	-	-
gALL_v3_c27071	1	2	-	-	-	-	-
gALL_v3_c27072	1	2	-	-	-	-	-
gALL_v3_c27073	2	1	-	-	-	-	-
gALL_v3_c27074	3	2	gnl|CDD|72989	cd03230, ABC_DR_subfamily_	ref|XP_974932.1|	PREDICTED: similar to AGAP003680-PA [Tribolium castaneum]	8,00E-39
gALL_v3_c27075	2	1	-	-	-	-	-
gALL_v3_c27076	1	3	-	-	-	-	-
gALL_v3_c27077	3	5	-	-	-	-	-
gALL_v3_c27079	4	6	-	-	-	-	-
gALL_v3_c2708	11	6	-	-	-	-	-
gALL_v3_c27080	0	4	-	-	-	-	-

gALL_v3_c27081	4	2	-	-	-	-	-
gALL_v3_c27082	2	1	-	-	-	-	-
gALL_v3_c27083	0	4	-	-	-	-	-
gALL_v3_c27084	3	1	-	-	gb|EFN68057.1|	hypothetical protein EAG_05255 [Camponotus floridanus]	6,00E-06
gALL_v3_c27085	0	4	-	-	-	-	-
gALL_v3_c27086	1	5	-	-	-	-	-
gALL_v3_c27087	0	3	-	-	-	-	-
gALL_v3_c27088	0	4	-	-	gb|EGI62944.1|	FLJ37770-like protein [Acromyrmex echinatior]	9,00E-11
gALL_v3_c27089	1	2	-	-	-	-	-
gALL_v3_c2709	6	14	gnl|CDD|189372	pfam00067, p450, Cytochrom	sp|P29981.1|CP4C1_BL	RecName: Full=Cytochrome P450 4C1; AltName: Full=CYPIVC1 gb|AAA27819.1| cytochrome P450 [Blaberus discoidalis]	1,00E-122
gALL_v3_c27090	0	3	-	-	-	-	-
gALL_v3_c27091	3	3	gnl|CDD|189372	pfam00067, p450, Cytochrom	sp|Q964R0.1|CP6K1_BL	RecName: Full=Cytochrome P450 6k1; AltName: Full=CYPVIK1 gb|AAK57914.1|AF281328_1 cytochrome P450 CYP6K1 [Blattella germanica]	5,00E-75
gALL_v3_c27092	3	1	-	-	-	-	-
gALL_v3_c27093	0	4	-	-	-	-	-
gALL_v3_c27094	3	3	-	-	ref|XP_974638.1|	PREDICTED: similar to huntingtin-interacting protein HYPK [Tribolium castaneum] gb|EFA00449.1| hypothetical protein TcasGA2_TC003305 [Tribolium castaneum]	7,00E-29
gALL_v3_c27095	3	6	-	-	gb|EHJ77899.1|	hypothetical protein KGM_18671 [Danaus plexippus]	2,00E-26
gALL_v3_c27096	2	3	gnl|CDD|34648	COG5043, MRS6, Vacuolar pro	ref|XP_004925674.1|	PREDICTED: vacuolar protein sorting-associated protein 13C-like [Bombyx mori]	1,00E-34
gALL_v3_c27098	1	3	-	-	ref|XP_006615958.1|	PREDICTED: uncharacterized protein LOC102675370 [Apis dorsata]	2,00E-71
gALL_v3_c27099	1	2	-	-	-	-	-
gALL_v3_c27101	3	1	-	-	-	-	-
gALL_v3_c27102	1	2	-	-	-	-	-
gALL_v3_c27103	3	1	-	-	-	-	-
gALL_v3_c27104	2	3	-	-	-	-	-
gALL_v3_c27105	2	1	-	-	-	-	-
gALL_v3_c27106	1	2	-	-	-	-	-
gALL_v3_c27107	0	3	-	-	-	-	-
gALL_v3_c27108	3	3	-	-	-	-	-
gALL_v3_c27109	1	2	gnl|CDD|193272	pfam12796, Ank_2, Ankyrin re	gb|AGM32371.1|	ankyrin repeat protein [Coptotermes formosanus]	1,00E-53
gALL_v3_c27110	3	1	-	-	-	-	-
gALL_v3_c27111	3	5	gnl|CDD|128548	smart00252, SH2, Src homolog	ref|XP_003247559.1|	PREDICTED: SHC-transforming protein 1-like [Acyrthosiphon pisum]	6,00E-67
gALL_v3_c27112	2	4	-	-	gb|EFA10412.1|	hypothetical protein TcasGA2_TC012652 [Tribolium castaneum]	5,00E-78
gALL_v3_c27113	2	4	-	-	-	-	-
gALL_v3_c27114	3	3	-	-	gb|EFZ20457.1|	hypothetical protein SINV_06496 [Solenopsis invicta]	2,00E-38
gALL_v3_c27115	2	2	gnl|CDD|144418	pfam00812, Ephrin, Ephrin.	ref|XP_001942892.2|	PREDICTED: ephrin-B2-like [Acyrthosiphon pisum]	3,00E-83
gALL_v3_c27116	1	3	-	-	-	-	-
gALL_v3_c27117	2	1	-	-	gb|EEZ99770.1|	hypothetical protein TcasGA2_TC002545 [Tribolium castaneum]	5,00E-45
gALL_v3_c27119	0	3	-	-	-	-	-
gALL_v3_c2712	11	14	-	-	-	-	-
gALL_v3_c27120	2	1	-	-	-	-	-
gALL_v3_c27121	0	3	-	-	gb|EFN72328.1|	Histone-lysine N-methyltransferase SETMAR [Camponotus floridanus]	2,00E-08
gALL_v3_c27123	3	3	gnl|CDD|146288	pfam03571, Peptidase_M49, I	ref|XP_003736578.1|	GA20334, isoform B [Drosophila pseudoobscura pseudoobscura] gb|EIM52651.1| GA20334, isoform B [Drosophila pseudoobscura pseudoobscura]	5,00E-44
gALL_v3_c27124	3	1	-	-	-	-	-
gALL_v3_c27125	3	4	-	-	-	-	-
gALL_v3_c27126	0	3	-	-	-	-	-
gALL_v3_c27127	2	2	gnl|CDD|153373	cd07361, MEMO_like, Memo	gb|AAR09872.1|	similar to Drosophila melanogaster CG8031, partial [Drosophila yakuba]	9,00E-68
gALL_v3_c27128	0	3	-	-	-	-	-
gALL_v3_c27129	5	2	-	-	-	-	-
gALL_v3_c27130	1	3	-	-	-	-	-
gALL_v3_c27131	2	2	-	-	-	-	-
gALL_v3_c27132	4	2	-	-	-	-	-
gALL_v3_c27133	2	4	-	-	-	-	-
gALL_v3_c27134	3	1	-	-	-	-	-
gALL_v3_c27135	2	3	-	-	-	-	-
gALL_v3_c27136	3	1	-	-	-	-	-
gALL_v3_c27138	2	2	-	-	-	-	-
gALL_v3_c27139	4	7	gnl|CDD|128516	smart00220, S_TKc, Serine/Th	gb|EGI67847.1|	MAP kinase-interacting serine/threonine-protein kinase 1 [Acromyrmex echinatior]	5,00E-74
gALL_v3_c2714	2	8	-	-	-	-	-
gALL_v3_c27140	2	1	-	-	-	-	-
gALL_v3_c27141	1	3	-	-	gb|EMC90702.1|	Pogo transposable element with KRAB domain, partial [Columba livia]	4,00E-13
gALL_v3_c27142	0	3	-	-	-	-	-
gALL_v3_c27143	2	1	-	-	-	-	-
gALL_v3_c27144	1	3	-	-	-	-	-
gALL_v3_c27145	2	4	-	-	-	-	-
gALL_v3_c27147	3	1	-	-	-	-	-
gALL_v3_c27148	1	4	-	-	-	-	-
gALL_v3_c27149	3	1	-	-	-	-	-
gALL_v3_c2715	8	13	-	-	ref|XP_001603560.2|	PREDICTED: transmembrane protein 192-like [Nasonia vitripennis]	1,00E-52
gALL_v3_c27150	2	3	-	-	-	-	-
gALL_v3_c27151	2	2	-	-	-	-	-
gALL_v3_c27152	1	3	gnl|CDD|107377	cd06382, PBP1_iGluR_Kainate	ref|XP_002425881.1|	conserved hypothetical protein [Pediculus humanus corporis] gb|EEB13143.1| conserved hypothetical protein [Pediculus humanus corporis]	5,00E-25
gALL_v3_c27153	0	3	-	-	-	-	-
gALL_v3_c27154	2	8	-	-	-	-	-
gALL_v3_c27155	1	3	gnl|CDD|189008	cd09601, M1_APN_2, Peptida	ref|XP_006619944.1|	PREDICTED: glutamyl aminopeptidase-like isoform X3 [Apis dorsata]	2,00E-78
gALL_v3_c27156	2	3	-	-	-	-	-
gALL_v3_c27157	4	4	gnl|CDD|30138	cd01640, IPPase, IPPase; Inosi	ref|XP_001604882.1|	PREDICTED: 3'(2'),5'-bisphosphate nucleotidase 1-like isoform 1 [Nasonia vitripennis] ref|XP_003426141.1| PREDICTED: 3'(2'),5'-bisphosphate nucleotidase 1-like isoform 2	2,00E-55
gALL_v3_c27158	2	4	-	-	-	-	-
gALL_v3_c27159	0	3	-	-	-	-	-
gALL_v3_c2716	5	6	-	-	-	-	-
gALL_v3_c27160	0	3	-	-	-	-	-
gALL_v3_c27161	2	1	-	-	-	-	-
gALL_v3_c27162	0	3	-	-	-	-	-
gALL_v3_c27163	2	1	-	-	ref|XP_001843996.1|	cubilin [Culex quinquefasciatus] gb|EDS35495.1| cubilin [Culex quinquefasciatus]	8,00E-11
gALL_v3_c27164	0	3	-	-	-	-	-
gALL_v3_c27165	2	5	-	-	ref|XP_005942915.1|	PREDICTED: thrombospondin type-1 domain-containing protein 7A-like isoform X4 [Haplochromis burtoni]	1,00E-37
gALL_v3_c27166	0	4	gnl|CDD|189706	pfam00759, Glyco_hydro_9, G	ref|XP_002426465.1|	Endoglucanase E-4 precursor, putative [Pediculus humanus corporis] gb|EEB13727.1| Endoglucanase E-4 precursor, putative [Pediculus humanus corporis]	3,00E-63
gALL_v3_c27167	1	4	-	-	ref|XP_005302607.1|	PREDICTED: jerky protein-like [Chrysemys picta bellii]	2,00E-06
gALL_v3_c27168	0	3	-	-	-	-	-
gALL_v3_c2717	11	15	gnl|CDD|189390	pfam00102, Y_phosphatase, P	ref|XP_003703482.1|	PREDICTED: tyrosine-protein phosphatase non-receptor type 9-like [Megachile rotundata]	1,00E-139
gALL_v3_c27170	0	5	-	-	-	-	-
gALL_v3_c27171	0	5	-	-	ref|XP_002428082.1|	conserved hypothetical protein [Pediculus humanus corporis] gb|EEB15344.1| conserved hypothetical protein [Pediculus humanus corporis]	2,00E-46
gALL_v3_c27172	8	13	-	-	ref|XP_006564371.1|	PREDICTED: titin isoform X9 [Apis mellifera] ref|XP_006564372.1| PREDICTED: titin isoform X10 [Apis mellifera] ref|XP_006564373.1| PREDICTED: titin isoform X11 [Apis n	1,00E-13
gALL_v3_c27173	2	1	-	-	-	-	-
gALL_v3_c27175	2	6	-	-	-	-	-
gALL_v3_c27176	2	3	-	-	-	-	-
gALL_v3_c27177	3	5	-	-	gb|EFN75852.1|	Craniofacial development protein 2 [Harpegnathos saltator]	2,00E-46
gALL_v3_c27178	2	2	-	-	-	-	-
gALL_v3_c27179	2	1	-	-	-	-	-
gALL_v3_c2718	8	13	gnl|CDD|147462	pfam05276, SH3BP5, SH3 dor	gb|EFA09348.1|	hypothetical protein TcasGA2_TC001674 [Tribolium castaneum]	1,00E-111
gALL_v3_c27180	2	2	-	-	gb|EHJ72595.1|	sensitized chromosome inheritance modifier 19 [Danaus plexippus]	1,00E-24
gALL_v3_c27181	3	1	-	-	ref|XP_973309.1|	PREDICTED: similar to CG8128 CG8128-PA [Tribolium castaneum] gb|EFA08806.1| hypothetical protein TcasGA2_TC006500 [Tribolium castaneum]	9,00E-58
gALL_v3_c27182	2	8	gnl|CDD|73156	cd01650, RT_nLTR_like, RT_nL	gb|AAB65093.1|	Lian-Aa1 retrotransposon protein [Aedes aegypti]	1,00E-46
gALL_v3_c27183	2	3	-	-	gb|EFZ16115.1|	hypothetical protein SINV_02972 [Solenopsis invicta]	6,00E-10
gALL_v3_c27184	1	3	-	-	-	-	-
gALL_v3_c27185	1	2	-	-	-	-	-
gALL_v3_c27186	0	4	-	-	-	-	-
gALL_v3_c27187	2	2	-	-	-	-	-
gALL_v3_c27188	1	2	gnl|CDD|189337	pfam00004, AAA, ATPase fami	ref|XP_006608089.1|	PREDICTED: spastin-like isoform X2 [Apis dorsata]	1,00E-46
gALL_v3_c27189	0	3	-	-	-	-	-
gALL_v3_c2719	7	5	gnl|CDD|178605	PLN03042, PLN03042, Lactoyl	dbj|BAN21301.1|	lactoylglutathione lyase [Riptortus pedestris]	2,00E-97
gALL_v3_c27190	4	2	-	-	-	-	-
gALL_v3_c27191	3	4	-	-	-	-	-
gALL_v3_c27192	10	15	-	-	-	-	-
gALL_v3_c27194	2	2	-	-	ref|XP_002027106.1|	GL20061 [Drosophila persimilis] gb|EDW34962.1| GL20061 [Drosophila persimilis]	2,00E-63
gALL_v3_c27195	2	2	-	-	-	-	-
gALL_v3_c27196	0	4	-	-	-	-	-
gALL_v3_c27197	1	3	-	-	-	-	-
gALL_v3_c27198	1	4	-	-	-	-	-
gALL_v3_c27199	1	3	-	-	-	-	-
gALL_v3_c272	18	34	-	-	ref|XP_002401871.1|	conserved hypothetical protein [Ixodes scapularis] gb|EEC02867.1| conserved hypothetical protein [Ixodes scapularis]	4,00E-12
gALL_v3_c2720	8	10	gnl|CDD|109126	pfam00057, Ldl_recept_a, Lov	ref|XP_001946912.2|	PREDICTED: low-density lipoprotein receptor-related protein 1-like [Acyrthosiphon pisum]	1,00E-151
gALL_v3_c27200	2	2	-	-	-	-	-
gALL_v3_c27201	2	2	-	-	-	-	-
gALL_v3_c27202	2	1	-	-	-	-	-
gALL_v3_c27203	3	1	gnl|CDD|144473	pfam00888, Cullin, Cullin fami	ref|XP_974579.1|	PREDICTED: similar to cullin [Tribolium castaneum] gb|EFA05359.1| hypothetical protein TcasGA2_TC015524 [Tribolium castaneum]	2,00E-56
gALL_v3_c27204	2	1	gnl|CDD|173623	cd00180, PKc, Catalytic domai	ref|XP_002424288.1|	RAC protein kinase DRAC-PK85, putative [Pediculus humanus corporis] gb|EEB11550.1| RAC protein kinase DRAC-PK85, putative [Pediculus humanus corporis]	5,00E-63
gALL_v3_c27205	0	4	-	-	-	-	-
gALL_v3_c27208	1	4	-	-	-	-	-
gALL_v3_c27209	1	2	-	-	-	-	-
gALL_v3_c2721	20	37	gnl|CDD|189741	pfam00850, Hist_deacetyl, His	gb|EFN75801.1|	Histone deacetylase 4 [Harpegnathos saltator]	0
gALL_v3_c27210	1	2	-	-	gb|ENN81790.1|	hypothetical protein YQE_01821, partial [Dendroctonus ponderosae] gb|ERL93114.1| hypothetical protein D910_10416 [Dendroctonus ponderosae]	8,00E-16
gALL_v3_c27212	3	3	-	-	-	-	-
gALL_v3_c27213	0	4	-	-	-	-	-
gALL_v3_c27214	2	2	gnl|CDD|73022	cd03263, ABC_subfamily_A, Tl	ref|XP_001947916.2|	PREDICTED: ATP-binding cassette sub-family A member 3-like [Acyrthosiphon pisum]	1,00E-38
gALL_v3_c27216	2	1	-	-	gb|EFA10379.1|	hypothetical protein TcasGA2_TC012610 [Tribolium castaneum]	7,00E-10
gALL_v3_c27217	3	1	-	-	-	-	-
gALL_v3_c27218	2	3	-	-	-	-	-
gALL_v3_c27219	11	19	gnl|CDD|162451	TIGR01622, SF-CC1, splicing fa	ref|XP_003707644.1|	PREDICTED: RNA-binding protein 39-like isoform 2 [Megachile rotundata]	1,00E-135
gALL_v3_c2722	7	11	-	-	-	-	-
gALL_v3_c27220	1	2	-	-	-	-	-
gALL_v3_c27221	1	4	-	-	-	-	-
gALL_v3_c27222	0	3	-	-	-	-	-
gALL_v3_c27223	7	17	-	-	-	-	-
gALL_v3_c27224	2	2	-	-	-	-	-
gALL_v3_c27226	2	1	-	-	-	-	-

gALL_v3_c27227	0	3	-	-	-	-	-
gALL_v3_c2723	25	20	-	-	-	-	-
gALL_v3_c27230	2	2	-	-	gb|EFN75452.1|	Bifunctional 3'-phosphoadenosine 5'-phosphosulfate synthetase [Harpegnathos saltator]	1,00E-18
gALL_v3_c27231	2	2	-	-	ref|XP_003705118.1|	PREDICTED: uncharacterized protein LOC100882926 [Megachile rotundata]	7,00E-65
gALL_v3_c27232	1	4	-	-	-	-	-
gALL_v3_c27233	1	3	-	-	-	-	-
gALL_v3_c27234	2	2	-	-	-	-	-
gALL_v3_c27235	3	2	-	-	ref|NP_001039198.1|	TBC1 domain family member 2B [Xenopus (Silurana) tropicalis] sp|Q28CB1.1|TBD2B_XENTR RecName: Full=TBC1 domain family member 2B emb|CAJ81581.1| novel prot	5,00E-06
gALL_v3_c27236	0	3	-	-	-	-	-
gALL_v3_c27237	1	2	-	-	-	-	-
gALL_v3_c27238	2	5	-	-	-	-	-
gALL_v3_c27239	3	1	-	-	ref|XP_001813427.1|	PREDICTED: similar to arc CG6741-PB [Tribolium castaneum] gb|EFA10855.1| hypothetical protein TcasGA2_TC001632 [Tribolium castaneum]	1,00E-28
gALL_v3_c2724	4	6	gnl|CDD|114307	pfam05577, Peptidase_S28, Se	gb|EFX79132.1|	hypothetical protein DAPPUDRAFT_225197 [Daphnia pulex]	3,00E-64
gALL_v3_c27240	1	2	-	-	-	-	-
gALL_v3_c27241	0	3	-	-	-	-	-
gALL_v3_c27242	3	3	-	-	ref|XP_974509.1|	PREDICTED: similar to SP555 CG14041-PA [Tribolium castaneum] gb|EFA03472.1| hypothetical protein TcasGA2_TC013469 [Tribolium castaneum]	1,00E-87
gALL_v3_c27243	1	4	-	-	-	-	-
gALL_v3_c27244	2	3	-	-	ref|XP_005183710.1|	PREDICTED: serine/threonine-protein kinase Nek8-like [Musca domestica]	1,00E-15
gALL_v3_c27246	0	3	-	-	-	-	-
gALL_v3_c27247	0	3	-	-	-	-	-
gALL_v3_c27248	1	4	-	-	gb|EFZ10112.1|	hypothetical protein SINV_80283 [Solenopsis invicta]	1,00E-119
gALL_v3_c27249	6	3	gnl|CDD|128342	smart00027, EH, Eps15 homol	ref|XP_002429582.1|	RalBP1-associated Eps domain-containing protein, putative [Pediculus humanus corporis] gb|EEB16844.1| RalBP1-associated Eps domain-containing protein, putative [Ped	4,00E-87
gALL_v3_c2725	6	17	gnl|CDD|192374	pfam09770, PAT1, Topoisomer	ref|XP_006558002.1|	PREDICTED: histone-lysine N-methyltransferase 2C [Apis mellifera]	2,00E-13
gALL_v3_c27250	0	3	-	-	-	-	-
gALL_v3_c27252	2	2	-	-	-	-	-
gALL_v3_c27253	4	7	-	-	-	-	-
gALL_v3_c27254	4	3	-	-	-	-	-
gALL_v3_c27255	1	2	-	-	-	-	-
gALL_v3_c27256	2	1	gnl|CDD|176494	cd08764, Cyt_b561_CG1275_l	gb|EFA10387.1|	hypothetical protein TcasGA2_TC012618 [Tribolium castaneum]	2,00E-47
gALL_v3_c27257	2	1	-	-	-	-	-
gALL_v3_c27258	3	2	-	-	ref|XP_001849638.1|	39S ribosomal protein L15, mitochondrial [Culex quinquefasciatus] gb|EDS30619.1| 39S ribosomal protein L15, mitochondrial [Culex quinquefasciatus]	1,00E-113
gALL_v3_c27259	4	6	-	-	-	-	-
gALL_v3_c2726	11	7	gnl|CDD|48188	cd03313, enolase, Enolase: En	dbj|BAF48677.1|	enolase [Pseudotrichonympha grassii]	0
gALL_v3_c27260	1	3	-	-	-	-	-
gALL_v3_c27261	5	5	-	-	-	-	-
gALL_v3_c27262	2	2	-	-	ref|XP_006128720.1|	PREDICTED: tigger transposable element-derived protein 1-like [Pelodiscus sinensis]	4,00E-06
gALL_v3_c27264	2	1	-	-	gb|EFX63703.1|	hypothetical protein DAPPUDRAFT_306608 [Daphnia pulex]	1,00E-08
gALL_v3_c27265	1	4	-	-	gb|ENN77069.1|	hypothetical protein YQE_06404, partial [Dendroctonus ponderosae]	5,00E-06
gALL_v3_c27266	2	4	gnl|CDD|31397	COG1204, COG1204, Superfan	gb|EEZ99164.1|	hypothetical protein TcasGA2_TC016163 [Tribolium castaneum]	1,00E-99
gALL_v3_c27267	2	7	-	-	ref|XP_001356797.1|	GA19489 [Drosophila pseudoobscura pseudoobscura] ref|XP_002015132.1| GL18582 [Drosophila persimilis] gb|EAL33863.1| GA19489 [Drosophila pseudoobscura pseudo	2,00E-09
gALL_v3_c27269	3	2	-	-	-	-	-
gALL_v3_c27270	0	3	-	-	-	-	-
gALL_v3_c27272	1	2	-	-	-	-	-
gALL_v3_c27273	1	5	-	-	-	-	-
gALL_v3_c27274	2	2	-	-	-	-	-
gALL_v3_c27276	1	2	-	-	ref|XP_970073.2|	PREDICTED: similar to serine/threonine protein kinase [Tribolium castaneum]	6,00E-07
gALL_v3_c27277	1	3	-	-	-	-	-
gALL_v3_c27278	2	7	-	-	-	-	-
gALL_v3_c27279	2	1	-	-	-	-	-
gALL_v3_c27280	1	2	gnl|CDD|192389	pfam09808, SNAPc_SNAP43, S	ref|XP_006618424.1|	PREDICTED: snRNA-activating protein complex subunit 1-like, partial [Apis dorsata]	2,00E-13
gALL_v3_c27281	2	2	-	-	ref|XP_003244738.1|	PREDICTED: zinc finger protein 572-like [Acyrthosiphon pisum]	1,00E-49
gALL_v3_c27282	0	3	-	-	-	-	-
gALL_v3_c27283	1	4	-	-	ref|XP_002426969.1|	conserved hypothetical protein [Pediculus humanus corporis] gb|EEB14231.1| conserved hypothetical protein [Pediculus humanus corporis]	5,00E-40
gALL_v3_c27284	2	2	gnl|CDD|189450	pfam00209, SNF, Sodium:neur	ref|XP_005181015.1|	PREDICTED: sodium-dependent nutrient amino acid transporter 1-like [Musca domestica]	9,00E-60
gALL_v3_c27285	4	2	-	-	-	-	-
gALL_v3_c27286	2	2	-	-	gb|EFN63586.1|	hypothetical protein EAG_04860 [Camponotus floridanus]	2,00E-23
gALL_v3_c27288	0	4	-	-	-	-	-
gALL_v3_c27289	1	3	-	-	ref|XP_967380.2|	PREDICTED: similar to conserved hypothetical protein, partial [Tribolium castaneum]	2,00E-36
gALL_v3_c2729	14	18	-	-	dbj|BAJ79290.1|	geranylgeranyl diphosphate synthase [Reticulitermes speratus]	2,00E-28
gALL_v3_c27290	2	2	-	-	gb|ERL93681.1|	hypothetical protein D910_10969, partial [Dendroctonus ponderosae]	1,00E-66
gALL_v3_c27291	2	2	-	-	-	-	-
gALL_v3_c27292	1	3	-	-	-	-	-
gALL_v3_c27293	3	7	-	-	-	-	-
gALL_v3_c27294	2	3	gnl|CDD|190503	pfam03016, Exostosin, Exosto:	ref|XP_002429885.1|	Exostosin-1, putative [Pediculus humanus corporis] gb|EEB17147.1| Exostosin-1, putative [Pediculus humanus corporis]	3,00E-93
gALL_v3_c27295	1	3	-	-	-	-	-
gALL_v3_c27296	1	2	-	-	-	-	-
gALL_v3_c27297	2	1	-	-	-	-	-
gALL_v3_c27298	4	2	-	-	-	-	-
gALL_v3_c27299	1	1	-	-	gb|ERE86144.1|	clavesin-2-like protein [Cricetulus griseus]	2,00E-24
gALL_v3_c273	30	33	-	-	-	-	-
gALL_v3_c2730	11	12	-	-	-	-	-
gALL_v3_c27300	4	10	-	-	gb|EFN62436.1|	Troponin C [Camponotus floridanus]	1,00E-38
gALL_v3_c27301	0	4	gnl|CDD|128516	smart00220, S_TKc, Serine/Th	ref|XP_004929190.1|	PREDICTED: tribbles homolog 2-like [Bombyx mori]	2,00E-53
gALL_v3_c27302	3	1	gnl|CDD|162169	TIGR01040, V-ATPase_V1_B, V	gb|ABM05767.1|	vacuolar ATP synthase subunit B, partial [Peripatus sp. SJB-2006]	1,00E-23
gALL_v3_c27303	0	5	-	-	-	-	-
gALL_v3_c27304	2	2	-	-	gb|EGI68517.1|	Nck-associated protein 5 [Acromyrmex echinatior]	4,00E-18
gALL_v3_c27305	6	3	-	-	-	-	-
gALL_v3_c27306	1	3	-	-	-	-	-
gALL_v3_c27307	0	6	-	-	-	-	-
gALL_v3_c27309	3	2	-	-	-	-	-
gALL_v3_c2731	2	7	gnl|CDD|144191	pfam00503, G-alpha, G-protei	gb|EFN66163.1|	Guanine nucleotide-binding protein G(o) subunit alpha [Camponotus floridanus]	1,00E-57
gALL_v3_c27310	1	5	-	-	-	-	-
gALL_v3_c27311	5	2	-	-	-	-	-
gALL_v3_c27313	2	1	-	-	-	-	-
gALL_v3_c27314	1	3	gnl|CDD|143450	cd07132, ALDH_F3AB, Aldehy	ref|XP_001655924.1|	hypothetical protein AaeL_AAEL012161 [Aedes aegypti] gb|EAT35689.1| AAEL012161-PA [Aedes aegypti]	1,00E-30
gALL_v3_c27315	2	2	-	-	-	-	-
gALL_v3_c27316	3	2	-	-	-	-	-
gALL_v3_c27317	0	5	-	-	ref|XP_003242865.1|	PREDICTED: hypothetical protein LOC100572069 [Acyrthosiphon pisum]	1,00E-06
gALL_v3_c27318	3	2	-	-	-	-	-
gALL_v3_c27320	2	5	-	-	-	-	-
gALL_v3_c27321	1	2	-	-	-	-	-
gALL_v3_c27322	3	1	gnl|CDD|29257	cd00200, WD40, WD40 domai	ref|XP_003707510.1|	PREDICTED: phospholipase A-2-activating protein-like [Megachile rotundata]	5,00E-54
gALL_v3_c27323	1	2	-	-	-	-	-
gALL_v3_c27324	1	3	-	-	-	-	-
gALL_v3_c27325	1	3	-	-	-	-	-
gALL_v3_c27326	19	20	-	-	-	-	-
gALL_v3_c27327	1	4	gnl|CDD|189816	pfam01039, Carboxyl_trans, C	ref|XP_004522579.1|	PREDICTED: acetyl-CoA carboxylase-like isoform X4 [Ceratitis capitata]	1,00E-44
gALL_v3_c27328	1	2	-	-	-	-	-
gALL_v3_c27329	2	2	-	-	-	-	-
gALL_v3_c2733	4	6	-	-	-	-	-
gALL_v3_c27330	1	8	-	-	-	-	-
gALL_v3_c27331	2	2	-	-	-	-	-
gALL_v3_c27332	1	2	-	-	-	-	-
gALL_v3_c27333	2	2	-	-	ref|XP_005108480.1|	PREDICTED: DNA-dependent protein kinase catalytic subunit-like [Aplysia californica]	5,00E-12
gALL_v3_c27334	2	2	-	-	-	-	-
gALL_v3_c27336	1	1	-	-	-	-	-
gALL_v3_c27337	1	2	-	-	-	-	-
gALL_v3_c27338	2	3	gnl|CDD|119423	cd05163, TRRAP, TRansformat	ref|XP_003485007.1|	PREDICTED: transformation/transcription domain-associated protein-like isoform 1 [Bombus impatiens] ref|XP_003485008.1| PREDICTED: transformation/transcription dc	2,00E-67
gALL_v3_c27340	2	3	-	-	-	-	-
gALL_v3_c27341	1	4	-	-	-	-	-
gALL_v3_c27342	2	2	-	-	ref|NP_001229507.1|	uncharacterized protein LOC411721 [Apis mellifera]	2,00E-22
gALL_v3_c27343	1	3	-	-	-	-	-
gALL_v3_c27345	0	5	-	-	-	-	-
gALL_v3_c27346	1	3	-	-	ref|XP_969777.1|	PREDICTED: similar to CG33332 CG33332-PA [Tribolium castaneum]	8,00E-09
gALL_v3_c27347	2	1	-	-	-	-	-
gALL_v3_c27348	3	1	-	-	ref|XP_974098.2|	PREDICTED: similar to conserved hypothetical protein [Tribolium castaneum]	1,00E-35
gALL_v3_c27349	0	3	-	-	-	-	-
gALL_v3_c2735	23	31	-	-	ref|XP_001605028.2|	PREDICTED: hypothetical protein LOC100121415 [Nasonia vitripennis]	2,00E-33
gALL_v3_c27350	2	1	-	-	-	-	-
gALL_v3_c27351	2	3	-	-	-	-	-
gALL_v3_c27352	2	1	-	-	-	-	-
gALL_v3_c27353	2	2	-	-	-	-	-
gALL_v3_c27354	0	3	-	-	-	-	-
gALL_v3_c27355	1	2	-	-	-	-	-
gALL_v3_c27356	1	7	gnl|CDD|34783	COG5184, ATS1, Alpha-tubulin	ref|XP_003492127.1|	PREDICTED: LOW QUALITY PROTEIN: regulator of chromosome condensation-like [Bombus impatiens]	1,00E-93
gALL_v3_c27357	3	1	-	-	gb|EFA03042.1|	hypothetical protein TcasGA2_TC010468 [Tribolium castaneum]	4,00E-20
gALL_v3_c27358	0	4	-	-	gb|EFN89320.1|	Rap guanine nucleotide exchange factor 2 [Harpegnathos saltator]	2,00E-20
gALL_v3_c27359	0	3	-	-	-	-	-
gALL_v3_c2736	3	6	-	-	ref|XP_002426684.1|	hypothetical protein Phum_PHUM268290 [Pediculus humanus corporis] gb|EEB13946.1| hypothetical protein Phum_PHUM268290 [Pediculus humanus corporis]	6,00E-15
gALL_v3_c27360	4	2	gnl|CDD|190353	pfam02586, DUF159, Unchara	ref|XP_001947778.1|	PREDICTED: UPF0361 protein C3orf37 homolog [Acyrthosiphon pisum]	9,00E-79
gALL_v3_c27361	0	4	-	-	-	-	-
gALL_v3_c27362	0	4	-	-	-	-	-
gALL_v3_c27363	2	3	gnl|CDD|192455	pfam10075, PCI_Csn8, COP9 s	gb|EFA04765.1|	hypothetical protein TcasGA2_TC030655 [Tribolium castaneum]	2,00E-49
gALL_v3_c27364	0	4	gnl|CDD|185448	PTZ00104, PTZ00104, S-adeno	ref|XP_002087374.1|	GE16582 [Drosophila yakuba] gb|EDW87086.1| GE16582 [Drosophila yakuba]	2,00E-15
gALL_v3_c27365	3	1	-	-	-	-	-
gALL_v3_c27366	2	1	-	-	-	-	-
gALL_v3_c27367	1	4	gnl|CDD|185696	cd09019, galactose_mutarota:	ref|XP_001654671.1|	aldose-1-epimerase [Aedes aegypti] gb|EAT37419.1| AAEL010590-PA [Aedes aegypti]	3,00E-28
gALL_v3_c27368	0	6	-	-	-	-	-
gALL_v3_c27369	1	4	gnl|CDD|163672	cd08165, MPP_MPPE1, huma	gb|EGI65468.1|	Metallophosphoesterase 1 [Acromyrmex echinatior]	1,00E-60
gALL_v3_c27370	3	1	-	-	-	-	-
gALL_v3_c27371	2	2	-	-	-	-	-
gALL_v3_c27372	1	2	-	-	ref|XP_002426699.1|	conserved hypothetical protein [Pediculus humanus corporis] gb|EEB13961.1| conserved hypothetical protein [Pediculus humanus corporis]	3,00E-20
gALL_v3_c27374	4	5	-	-	-	-	-
gALL_v3_c27375	2	4	-	-	-	-	-

gALL_v3_c27376	2	2	-	-	-	-	-
gALL_v3_c27377	0	3	-	-	-	-	-
gALL_v3_c27379	2	2	-	-	gb|EFN86114.1|	Alpha-1,3-mannosyl-glycoprotein 2-beta-N-acetylglucosaminyltransferase [Harpegnathos saltator]	1,00E-06
gALL_v3_c2738	15	11	gnl|CDD|128489	smart00192, LDLa, Low-densit	gb|EFX73914.1|	hypothetical protein DAPPUDRAFT_307561 [Daphnia pulex]	1,00E-162
gALL_v3_c27380	1	3	-	-	-	-	-
gALL_v3_c27382	2	2	-	-	ref|NP_001164144.1|	sprouty-related protein with EVH-1 domain [Tribolium castaneum]	4,00E-41
gALL_v3_c27383	1	2	-	-	-	-	-
gALL_v3_c27384	1	2	-	-	-	-	-
gALL_v3_c27385	2	2	-	-	-	-	-
gALL_v3_c27387	0	3	-	-	-	-	-
gALL_v3_c27389	3	3	-	-	-	-	-
gALL_v3_c2739	8	5	gnl|CDD|128627	smart00332, PP2Cc, Serine/th	gb|EFZ21328.1|	hypothetical protein SINV_01503 [Solenopsis invicta]	3,00E-54
gALL_v3_c27390	1	3	-	-	-	-	-
gALL_v3_c27392	2	7	gnl|CDD|191824	pfam07717, OB_NTP_bind, Ol	gb|EGI70339.1|	Dosage compensation regulator [Acromyrmex echinatior]	2,00E-83
gALL_v3_c27393	0	3	-	-	-	-	-
gALL_v3_c27394	3	4	-	-	-	-	-
gALL_v3_c27395	0	3	gnl|CDD|128654	smart00360, RRM, RNA recog	gb|AGM32299.1|	serine-arginine protein 55-like protein, partial [Coptotermes formosanus]	4,00E-51
gALL_v3_c27396	4	4	-	-	-	-	-
gALL_v3_c27397	0	3	-	-	ref|XP_001945396.1|	PREDICTED: follistatin-related protein 5-like, partial [Acyrthosiphon pisum]	3,00E-69
gALL_v3_c27398	1	2	gnl|CDD|162378	TIGR01472, gmd, GDP-manno	ref|XP_001650108.1|	gdp mannose-4,6-dehydratase [Aedes aegypti] gb|EAT32804.1| AAEL014961-PA [Aedes aegypti]	1,00E-51
gALL_v3_c27399	1	3	-	-	-	-	-
gALL_v3_c274	3	7	-	-	-	-	-
gALL_v3_c2740	5	2	-	-	gb|AGM32893.1|	Kazal type serine protease inhibitor [Coptotermes formosanus]	1,00E-57
gALL_v3_c27400	1	4	-	-	-	-	-
gALL_v3_c27401	8	7	-	-	-	-	-
gALL_v3_c27402	1	2	-	-	-	-	-
gALL_v3_c27403	3	1	-	-	-	-	-
gALL_v3_c27404	1	3	-	-	gb|AAM77026.1|	olfactory binding protein [Rhyparobia maderae]	2,00E-08
gALL_v3_c27405	7	9	-	-	gb|AGM32333.1|	antioxidant enzyme (heavy metal associated) [Coptotermes formosanus]	1,00E-41
gALL_v3_c27406	3	2	-	-	gb|EFA13284.1|	hypothetical protein TcasGA2_TC010304 [Tribolium castaneum]	2,00E-13
gALL_v3_c27407	4	3	-	-	-	-	-
gALL_v3_c27408	0	3	-	-	-	-	-
gALL_v3_c27409	0	3	-	-	-	-	-
gALL_v3_c2741	13	8	-	-	-	-	-
gALL_v3_c27411	2	2	-	-	-	-	-
gALL_v3_c27412	1	5	-	-	-	-	-
gALL_v3_c27413	2	1	-	-	-	-	-
gALL_v3_c27414	2	4	-	-	-	-	-
gALL_v3_c27415	1	3	-	-	-	-	-
gALL_v3_c27417	1	2	-	-	-	-	-
gALL_v3_c27418	3	6	-	-	-	-	-
gALL_v3_c27419	6	6	gnl|CDD|179788	PRK04210, PRK04210, phosph	ref|YP_004699005.1|	phosphoenolpyruvate carboxykinase (GTP) [Treponema caldaria DSM 7334] ref|WP_013969778.1| phosphoenolpyruvate carboxykinase [Treponema caldaria] gb|AEJ2049	1,00E-130
gALL_v3_c27420	6	4	-	-	-	-	-
gALL_v3_c27421	1	4	-	-	ref|XP_006610983.1|	PREDICTED: uncharacterized protein LOC102673797 isoform X1 [Apis dorsata]	3,00E-08
gALL_v3_c27422	3	7	-	-	gb|AAB92394.1|	putative reverse transcriptase [Drosophila subobscura]	4,00E-24
gALL_v3_c27423	2	3	-	-	-	-	-
gALL_v3_c27424	2	3	-	-	-	-	-
gALL_v3_c27425	1	2	-	-	-	-	-
gALL_v3_c27426	1	2	gnl|CDD|193257	pfam12781, AAA_9, ATP-bindi	gb|ERL87387.1|	hypothetical protein D910_04782 [Dendroctonus ponderosae]	1,00E-67
gALL_v3_c27427	2	1	-	-	-	-	-
gALL_v3_c27429	1	2	-	-	-	-	-
gALL_v3_c2743	7	6	-	-	-	-	-
gALL_v3_c27430	0	2	-	-	-	-	-
gALL_v3_c27431	3	1	-	-	-	-	-
gALL_v3_c27432	1	4	-	-	ref|XP_003425117.1|	PREDICTED: hypothetical protein LOC100114433 isoform 2 [Nasonia vitripennis]	4,00E-35
gALL_v3_c27433	21	14	-	-	gb|AGT15837.1|	50MGP [Reticulitermes flavipes]	5,00E-28
gALL_v3_c27434	3	4	-	-	gb|EFN83345.1|	X-linked retinitis pigmentosa GTPase regulator-like protein [Harpegnathos saltator]	5,00E-10
gALL_v3_c27435	0	4	-	-	gb|ADI19371.1|	hypothetical protein [uncultured Spirochaetales bacterium HF0500_06B09]	2,00E-35
gALL_v3_c27436	0	3	-	-	-	-	-
gALL_v3_c27437	1	2	-	-	-	-	-
gALL_v3_c27438	3	2	-	-	-	-	-
gALL_v3_c27439	1	6	-	-	-	-	-
gALL_v3_c27440	0	4	-	-	-	-	-
gALL_v3_c27441	3	3	-	-	ref|XP_002402825.1|	u4/u6 small nuclear ribonucleoprotein, putative [Ixodes scapularis] gb|EEC14337.1| u4/u6 small nuclear ribonucleoprotein, putative [Ixodes scapularis]	1,00E-31
gALL_v3_c27442	4	2	-	-	gb|EFN84669.1|	hypothetical protein EAI_07799 [Harpegnathos saltator]	4,00E-06
gALL_v3_c27443	2	5	gnl|CDD|162739	TIGR02168, SMC_prok_B, chrc	gb|EFN83883.1|	Leucine-rich repeat-containing protein 45 [Harpegnathos saltator]	5,00E-16
gALL_v3_c27444	2	3	-	-	-	-	-
gALL_v3_c27445	3	1	-	-	-	-	-
gALL_v3_c27446	2	1	-	-	-	-	-
gALL_v3_c27448	2	2	-	-	-	-	-
gALL_v3_c27449	1	3	-	-	-	-	-
gALL_v3_c2745	8	14	-	-	dbj|GAA50252.1|	gap-Pol polyprotein [Clonorchis sinensis]	6,00E-06
gALL_v3_c27450	2	3	-	-	-	-	-
gALL_v3_c27451	1	2	-	-	gb|EFX69512.1|	hypothetical protein DAPPUDRAFT_329005 [Daphnia pulex]	8,00E-07
gALL_v3_c27452	1	2	-	-	-	-	-
gALL_v3_c27453	0	3	-	-	-	-	-
gALL_v3_c27454	3	1	gnl|CDD|112624	pfam03820, Mtc, Tricarboxylat	ref|XP_003696034.1|	PREDICTED: sideroflexin-2-like [Apis florea]	3,00E-30
gALL_v3_c27455	0	3	-	-	-	-	-
gALL_v3_c27456	1	4	-	-	-	-	-
gALL_v3_c27457	6	6	-	-	-	-	-
gALL_v3_c27458	0	5	-	-	-	-	-
gALL_v3_c27459	4	3	-	-	-	-	-
gALL_v3_c2746	2	7	-	-	-	-	-
gALL_v3_c27460	0	4	-	-	ref|XP_003244181.1|	PREDICTED: catenin delta-2-like isoform 2 [Acyrthosiphon pisum]	3,00E-69
gALL_v3_c27461	1	2	-	-	ref|XP_002428696.1|	Secernin-1, putative [Pediculus humanus corporis] gb|EEB15958.1| Secernin-1, putative [Pediculus humanus corporis]	1,00E-11
gALL_v3_c27462	2	1	-	-	ref|XP_003380555.1|	ankyrin repeat-containing domain protein [Trichinella spiralis] gb|EFV59825.1| ankyrin repeat-containing domain protein [Trichinella spiralis]	4,00E-06
gALL_v3_c27463	1	2	-	-	-	-	-
gALL_v3_c27464	0	3	-	-	-	-	-
gALL_v3_c27465	3	1	-	-	-	-	-
gALL_v3_c27467	5	3	-	-	-	-	-
gALL_v3_c27468	2	1	-	-	-	-	-
gALL_v3_c27469	1	4	gnl|CDD|146158	pfam03371, PRP38, PRP38 fan	ref|XP_967482.1|	PREDICTED: similar to PRP38 pre-mRNA processing factor 38 (yeast) domain containing B isoform 1 [Tribolium castaneum] gb|EEZ97201.1| hypothetical protein TcasGA2_1	1,00E-46
gALL_v3_c27470	1	2	-	-	-	-	-
gALL_v3_c27471	2	4	-	-	-	-	-
gALL_v3_c27472	1	3	-	-	-	-	-
gALL_v3_c27473	2	2	gnl|CDD|143939	pfam00173, Cyt-b5, Cytochror	gb|EHJ73294.1|	hypothetical protein KGM_20565 [Danaus plexippus]	4,00E-35
gALL_v3_c27474	5	3	-	-	gb|AGM32707.1|	hypothetical protein, partial [Coptotermes formosanus]	4,00E-42
gALL_v3_c27475	0	3	-	-	-	-	-
gALL_v3_c27476	2	1	-	-	ref|XP_003241595.1|	PREDICTED: zinc finger protein 572-like [Acyrthosiphon pisum]	3,00E-56
gALL_v3_c27477	1	2	-	-	-	-	-
gALL_v3_c27478	2	1	-	-	gb|ENN83385.1|	hypothetical protein YQE_00256, partial [Dendroctonus ponderosae]	1,00E-18
gALL_v3_c27479	3	7	-	-	ref|XP_973767.2|	PREDICTED: similar to kakapo [Tribolium castaneum]	1,00E-49
gALL_v3_c27481	2	2	-	-	-	-	-
gALL_v3_c27482	0	5	-	-	-	-	-
gALL_v3_c27483	3	5	-	-	-	-	-
gALL_v3_c27484	3	2	-	-	-	-	-
gALL_v3_c27485	2	4	-	-	-	-	-
gALL_v3_c27486	2	3	-	-	-	-	-
gALL_v3_c27487	2	2	-	-	-	-	-
gALL_v3_c27488	3	4	-	-	-	-	-
gALL_v3_c27489	1	4	-	-	-	-	-
gALL_v3_c2749	17	15	-	-	-	-	-
gALL_v3_c27490	4	5	-	-	gb|ERL87073.1|	hypothetical protein D910_04474 [Dendroctonus ponderosae]	1,00E-14
gALL_v3_c27491	1	3	-	-	-	-	-
gALL_v3_c27492	5	4	-	-	-	-	-
gALL_v3_c27493	1	2	-	-	-	-	-
gALL_v3_c27496	0	3	-	-	-	-	-
gALL_v3_c27497	6	5	gnl|CDD|128838	smart00568, GRAM, domain ir	gb|EFN82379.1|	GRAM domain-containing protein 1B [Harpegnathos saltator]	1,00E-116
gALL_v3_c27498	1	3	gnl|CDD|109266	pfam00201, UDPGT, UDP-gluc	gb|ABE60815.1|	cxpwmw04 [Periplaneta americana]	3,00E-27
gALL_v3_c27499	4	2	-	-	ref|XP_002427185.1|	hypothetical protein Phum_PHUM300620 [Pediculus humanus corporis] gb|EEB14447.1| hypothetical protein Phum_PHUM300620 [Pediculus humanus corporis]	3,00E-17
gALL_v3_c275	17	13	gnl|CDD|150877	pfam10271, Tmp39, Putative t	ref|XP_002432757.1|	conserved hypothetical protein [Pediculus humanus corporis] gb|EEB20019.1| conserved hypothetical protein [Pediculus humanus corporis]	0
gALL_v3_c2750	15	25	gnl|CDD|128516	smart00220, S_TKc, Serine/Th	gb|EHJ65793.1|	hypothetical protein KGM_07938 [Danaus plexippus]	1,00E-107
gALL_v3_c27501	3	4	-	-	-	-	-
gALL_v3_c27502	0	4	-	-	-	-	-
gALL_v3_c27503	3	2	gnl|CDD|128505	smart00209, TSP1, Thrombosp	ref|XP_002428039.1|	Semaphorin-5A precursor, putative [Pediculus humanus corporis] gb|EEB15301.1| Semaphorin-5A precursor, putative [Pediculus humanus corporis]	1,00E-105
gALL_v3_c27504	2	2	gnl|CDD|190008	pfam01490, Aa_trans, Transm	gb|EFZ20983.1|	hypothetical protein SINV_05703 [Solenopsis invicta]	7,00E-54
gALL_v3_c27505	2	1	-	-	-	-	-
gALL_v3_c27507	4	6	gnl|CDD|112537	pfam03727, Hexokinase_2, He	ref|XP_002041786.1|	GM11377 [Drosophila sechellia] gb|EDW45634.1| GM11377 [Drosophila sechellia]	1,00E-33
gALL_v3_c27508	0	4	-	-	-	-	-
gALL_v3_c27509	0	4	-	-	-	-	-
gALL_v3_c2751	6	10	-	-	-	-	-
gALL_v3_c27510	2	1	-	-	-	-	-
gALL_v3_c27511	1	2	-	-	-	-	-
gALL_v3_c27512	2	1	-	-	gb|EFN67634.1|	Putative uncharacterized transposon-derived protein F52C9.6 [Camponotus floridanus]	2,00E-11
gALL_v3_c27513	2	1	-	-	-	-	-
gALL_v3_c27514	0	5	-	-	gb|ERL89508.1|	hypothetical protein D910_06874 [Dendroctonus ponderosae]	1,00E-23
gALL_v3_c27515	0	3	-	-	-	-	-
gALL_v3_c27516	2	1	-	-	-	-	-
gALL_v3_c27517	1	4	-	-	-	-	-
gALL_v3_c27518	0	4	-	-	-	-	-
gALL_v3_c27519	1	3	-	-	-	-	-
gALL_v3_c2752	5	8	gnl|CDD|189816	pfam01039, Carboxyl_trans, C	ref|XP_002429216.1|	acetyl-CoA carboxylase, putative [Pediculus humanus corporis] gb|EEB16478.1| acetyl-CoA carboxylase, putative [Pediculus humanus corporis]	0
gALL_v3_c27520	0	3	-	-	-	-	-
gALL_v3_c27521	2	1	-	-	-	-	-

gALL_v3_c27522	2	1	-	-	gb|EFX86193.1|	hypothetical protein DAPPUDRAFT_44658 [Daphnia pulex]	1,00E-52
gALL_v3_c27523	0	3	-	-	-	-	-
gALL_v3_c27524	3	1	-	-	gb|EFX70495.1|	hypothetical protein DAPPUDRAFT_328037 [Daphnia pulex]	2,00E-13
gALL_v3_c27525	3	4	-	-	-	-	-
gALL_v3_c27527	3	2	-	-	-	-	-
gALL_v3_c27528	3	1	-	-	ref|XP_002431036.1|	serine/threonine-protein kinase MARK2, putative [Pediculus humanus corporis] gb|EEB18298.1| serine/threonine-protein kinase MARK2, putative [Pediculus humanus cor	1,00E-40
gALL_v3_c27529	5	10	-	-	-	-	-
gALL_v3_c27530	0	3	-	-	-	-	-
gALL_v3_c27532	2	4	-	-	gb|ESO90064.1|	hypothetical protein LOTGIDRAFT_123812, partial [Lottia gigantea]	2,00E-51
gALL_v3_c27534	7	6	gnl|CDD|150784	pfam10151, DUF2359, Unchar	gb|AGM32737.1|	uncharacterized conserved protein (DUF2359) [Coptotermes formosanus]	1,00E-106
gALL_v3_c27535	0	3	-	-	-	-	-
gALL_v3_c27537	2	1	-	-	ref|XP_799290.3|	PREDICTED: protein TFG-like [Strongylocentrotus purpuratus]	4,00E-07
gALL_v3_c27538	1	3	gnl|CDD|147266	pfam04998, RNA_pol_Rpb1_5	ref|XP_003706111.1|	PREDICTED: DNA-directed RNA polymerase III subunit RPC1-like [Megachile rotundata]	5,00E-51
gALL_v3_c2754	10	8	-	-	-	-	-
gALL_v3_c27540	2	1	-	-	-	-	-
gALL_v3_c27542	4	3	-	-	-	-	-
gALL_v3_c27543	1	3	-	-	ref|XP_002430824.1|	Ral guanine nucleotide exchange factor, putative [Pediculus humanus corporis] gb|EEB18086.1| Ral guanine nucleotide exchange factor, putative [Pediculus humanus corp	1,00E-38
gALL_v3_c27544	2	3	-	-	-	-	-
gALL_v3_c27545	3	2	-	-	-	-	-
gALL_v3_c27546	2	2	-	-	-	-	-
gALL_v3_c27547	2	3	-	-	-	-	-
gALL_v3_c27548	4	6	gnl|CDD|192510	pfam10277, Frag1, Frag1/DRA	ref|XP_974603.1|	PREDICTED: similar to CG3876 CG3876-PA [Tribolium castaneum] gb|EFA01947.1| hypothetical protein TcasGA2_TC007562 [Tribolium castaneum]	2,00E-37
gALL_v3_c27549	2	3	-	-	-	-	-
gALL_v3_c2755	5	6	gnl|CDD|192365	pfam09754, PAC2, PAC2 family	ref|XP_002427336.1|	conserved hypothetical protein [Pediculus humanus corporis] gb|EEB14598.1| conserved hypothetical protein [Pediculus humanus corporis]	3,00E-30
gALL_v3_c27550	1	5	-	-	-	-	-
gALL_v3_c27551	1	2	-	-	-	-	-
gALL_v3_c27552	0	3	-	-	ref|XP_006617629.1|	PREDICTED: serine/threonine-protein kinase tricorner-like isoform X6 [Apis dorsata]	8,00E-42
gALL_v3_c27553	1	4	gnl|CDD|193025	pfam12430, ABA_GPCR, Absci	ref|XP_001601598.2|	PREDICTED: Golgi pH regulator-like [Nasonia vitripennis]	1,00E-110
gALL_v3_c27555	5	4	-	-	-	-	-
gALL_v3_c27556	2	1	gnl|CDD|189955	pfam01363, FYVE, FYVE zinc fi	ref|XP_002429207.1|	zinc finger protein FYVE domain-containing protein, putative [Pediculus humanus corporis] gb|EEB16469.1| zinc finger protein FYVE domain-containing protein, putative [F	2,00E-42
gALL_v3_c27557	2	2	-	-	dbj|BAM18758.1|	sulfatase [Papilio xuthus]	8,00E-25
gALL_v3_c27558	0	3	-	-	-	-	-
gALL_v3_c27559	1	3	-	-	ref|XP_003423909.1|	PREDICTED: UPF0539 protein CG14977-like [Nasonia vitripennis]	1,00E-06
gALL_v3_c2756	6	13	-	-	gb|EFN83995.1|	Probable ubiquitin carboxyl-terminal hydrolase FAF-X [Harpegnathos saltator]	1,00E-148
gALL_v3_c27560	2	2	-	-	-	-	-
gALL_v3_c27561	2	2	-	-	-	-	-
gALL_v3_c27562	2	1	-	-	-	-	-
gALL_v3_c27564	2	1	-	-	-	-	-
gALL_v3_c27566	1	4	-	-	gb|EGI58842.1|	Nipped-B-like protein [Acromyrmex echinatior]	1,00E-15
gALL_v3_c27567	3	2	-	-	-	-	-
gALL_v3_c27568	1	5	gnl|CDD|189583	pfam00520, Ion_trans, Ion tra	dbj|BAA22091.1|	voltage-dependent calcium channel alpha-1 subunit [Blattella germanica]	7,00E-92
gALL_v3_c27569	2	2	-	-	-	-	-
gALL_v3_c2757	3	3	-	-	-	-	-
gALL_v3_c27570	2	3	-	-	ref|XP_002432511.1|	ubiquitin-protein ligase E3A, putative [Pediculus humanus corporis] gb|EEB19773.1| ubiquitin-protein ligase E3A, putative [Pediculus humanus corporis]	5,00E-43
gALL_v3_c27571	0	3	-	-	-	-	-
gALL_v3_c27572	5	3	-	-	-	-	-
gALL_v3_c27573	2	2	-	-	-	-	-
gALL_v3_c27574	2	1	-	-	-	-	-
gALL_v3_c27575	2	4	-	-	-	-	-
gALL_v3_c27576	2	4	-	-	-	-	-
gALL_v3_c27577	1	3	-	-	-	-	-
gALL_v3_c27578	0	3	-	-	-	-	-
gALL_v3_c27580	0	4	-	-	-	-	-
gALL_v3_c27581	2	3	-	-	-	-	-
gALL_v3_c27583	2	2	-	-	-	-	-
gALL_v3_c27584	0	3	-	-	-	-	-
gALL_v3_c27585	2	1	-	-	-	-	-
gALL_v3_c27586	2	2	-	-	-	-	-
gALL_v3_c27587	1	2	-	-	-	-	-
gALL_v3_c27589	3	1	-	-	-	-	-
gALL_v3_c2759	9	16	gnl|CDD|29077	cd01446, DSP_MapKP, N-term	ref|XP_002430571.1|	dual specificity protein phosphatase, putative [Pediculus humanus corporis] gb|EEB17833.1| dual specificity protein phosphatase, putative [Pediculus humanus corporis]	1,00E-142
gALL_v3_c27592	2	3	-	-	-	-	-
gALL_v3_c27594	3	3	-	-	emb|CAP20022.1|	transposase [Eriphia verrucosa]	5,00E-42
gALL_v3_c27595	2	3	-	-	-	-	-
gALL_v3_c27596	4	2	-	-	-	-	-
gALL_v3_c27597	2	1	-	-	-	-	-
gALL_v3_c27598	0	3	gnl|CDD|192080	pfam08585, DUF1767, Domair	ref|XP_004927766.1|	PREDICTED: tudor domain-containing protein 3-like [Bombyx mori]	3,00E-45
gALL_v3_c276	32	28	-	-	-	-	-
gALL_v3_c2760	4	6	-	-	ref|XP_002413021.1|	endonuclease/reverse transcriptase, putative [Ixodes scapularis] gb|EEC16329.1| endonuclease/reverse transcriptase, putative [Ixodes scapularis]	1,00E-14
gALL_v3_c27600	1	4	-	-	ref|XP_002430209.1|	Integrin alpha-PS2 precursor, putative [Pediculus humanus corporis] gb|EEB17471.1| Integrin alpha-PS2 precursor, putative [Pediculus humanus corporis]	6,00E-21
gALL_v3_c27601	1	3	-	-	ref|XP_006029730.1|	PREDICTED: histone-lysine N-methyltransferase SETMAR-like [Alligator sinensis]	2,00E-07
gALL_v3_c27602	1	2	-	-	-	-	-
gALL_v3_c27603	3	2	-	-	-	-	-
gALL_v3_c27604	1	4	-	-	ref|XP_003472586.2|	PREDICTED: CWF19-like protein 2 [Cavia porcellus]	9,00E-12
gALL_v3_c27607	2	3	-	-	-	-	-
gALL_v3_c27608	1	6	-	-	-	-	-
gALL_v3_c27609	2	1	-	-	ref|XP_970089.1|	PREDICTED: similar to CG3894 CG3894-PA [Tribolium castaneum] gb|EFA07350.1| hypothetical protein TcasGA2_TC015949 [Tribolium castaneum]	1,00E-10
gALL_v3_c27610	6	18	-	-	-	-	-
gALL_v3_c27611	3	1	-	-	-	-	-
gALL_v3_c27612	1	3	-	-	-	-	-
gALL_v3_c27613	3	1	-	-	-	-	-
gALL_v3_c27614	0	4	-	-	-	-	-
gALL_v3_c27615	0	3	-	-	ref|XP_002429661.1|	krueppel c2h2-type zinc finger protein, putative [Pediculus humanus corporis] gb|EEB16923.1| krueppel c2h2-type zinc finger protein, putative [Pediculus humanus corpoı	5,00E-34
gALL_v3_c27616	3	3	-	-	-	-	-
gALL_v3_c27617	1	3	-	-	-	-	-
gALL_v3_c27618	2	3	-	-	-	-	-
gALL_v3_c27619	4	2	-	-	-	-	-
gALL_v3_c2762	4	5	-	-	gb|EFA11632.1|	hypothetical protein TcasGA2_TC000010 [Tribolium castaneum]	2,00E-10
gALL_v3_c27620	3	1	-	-	-	-	-
gALL_v3_c27621	3	4	-	-	-	-	-
gALL_v3_c27622	2	1	-	-	-	-	-
gALL_v3_c27623	3	1	gnl|CDD|191369	pfam05769, DUF837, Protein (	gb|EFN82655.1|	FGFR1 oncogene partner 2-like protein [Harpegnathos saltator]	1,00E-48
gALL_v3_c27624	2	3	gnl|CDD|188047	TIGR00376, TIGR00376, DNA h	gb|EFZ21228.1|	hypothetical protein SINV_80088 [Solenopsis invicta]	5,00E-41
gALL_v3_c27625	6	3	gnl|CDD|178442	PLN02850, PLN02850, asparta	ref|XP_002423869.1|	aspartyl-tRNA synthetase, putative [Pediculus humanus corporis] gb|EEB11131.1| aspartyl-tRNA synthetase, putative [Pediculus humanus corporis]	6,00E-74
gALL_v3_c27626	3	2	-	-	-	-	-
gALL_v3_c27627	3	6	-	-	-	-	-
gALL_v3_c27628	0	3	-	-	-	-	-
gALL_v3_c2763	3	6	-	-	-	-	-
gALL_v3_c27631	3	3	-	-	-	-	-
gALL_v3_c27633	0	6	-	-	-	-	-
gALL_v3_c27634	1	3	-	-	-	-	-
gALL_v3_c27635	2	3	-	-	-	-	-
gALL_v3_c27636	2	3	-	-	-	-	-
gALL_v3_c27637	2	1	-	-	-	-	-
gALL_v3_c27638	1	2	-	-	-	-	-
gALL_v3_c27639	0	3	-	-	-	-	-
gALL_v3_c2764	6	4	gnl|CDD|144590	pfam01053, Cys_Met_Meta_P	ref|XP_002060103.1|	GJ15425 [Drosophila virilis] gb|EDW58188.1| GJ15425 [Drosophila virilis]	4,00E-78
gALL_v3_c27641	3	3	-	-	-	-	-
gALL_v3_c27642	2	6	-	-	-	-	-
gALL_v3_c27643	0	3	-	-	-	-	-
gALL_v3_c27644	0	5	-	-	-	-	-
gALL_v3_c27645	4	6	-	-	-	-	-
gALL_v3_c27646	3	1	-	-	-	-	-
gALL_v3_c27647	7	8	-	-	-	-	-
gALL_v3_c27648	1	3	-	-	-	-	-
gALL_v3_c27649	0	3	-	-	-	-	-
gALL_v3_c2765	5	6	gnl|CDD|189372	pfam00067, p450, Cytochrom	dbj|BAD84176.1|	cytochrome P450 [Hodotermopsis sjostedti]	4,00E-77
gALL_v3_c27650	1	4	-	-	-	-	-
gALL_v3_c27651	0	4	-	-	-	-	-
gALL_v3_c27652	1	3	-	-	-	-	-
gALL_v3_c27653	1	4	gnl|CDD|150043	pfam09230, DFF40, DNA fragn	ref|XP_003241871.1|	PREDICTED: DNA fragmentation factor subunit beta-like [Acyrthosiphon pisum]	2,00E-21
gALL_v3_c27654	3	4	-	-	-	-	-
gALL_v3_c27655	1	3	gnl|CDD|190554	pfam03178, CPSF_A, CPSF A su	ref|XP_002426118.1|	DNA damage-binding protein, putative [Pediculus humanus corporis] gb|EEB13380.1| DNA damage-binding protein, putative [Pediculus humanus corporis]	2,00E-96
gALL_v3_c27656	1	2	-	-	-	-	-
gALL_v3_c27657	1	2	-	-	-	-	-
gALL_v3_c27658	0	4	-	-	-	-	-
gALL_v3_c27659	3	5	-	-	-	-	-
gALL_v3_c2766	4	11	-	-	ref|XP_002430556.1|	rab3 interacting molecule, putative [Pediculus humanus corporis] gb|EEB17818.1| rab3 interacting molecule, putative [Pediculus humanus corporis]	1,00E-112
gALL_v3_c27660	0	4	-	-	-	-	-
gALL_v3_c27661	2	2	-	-	-	-	-
gALL_v3_c27662	1	5	-	-	-	-	-
gALL_v3_c27665	3	5	-	-	-	-	-
gALL_v3_c27666	1	2	-	-	-	-	-
gALL_v3_c27667	5	3	-	-	-	-	-
gALL_v3_c27668	1	3	gnl|CDD|178444	PLN02852, PLN02852, ferredo	dbj|BAN21618.1|	ferredoxin reductase, partial [Riptortus pedestris]	9,00E-45
gALL_v3_c27669	0	3	-	-	-	-	-
gALL_v3_c2767	7	4	-	-	-	-	-
gALL_v3_c27670	2	1	-	-	gb|EFN85317.1|	hypothetical protein EAI_16001 [Harpegnathos saltator]	1,00E-06
gALL_v3_c27671	2	1	-	-	-	-	-
gALL_v3_c27672	0	3	-	-	-	-	-
gALL_v3_c27673	0	3	-	-	-	-	-
gALL_v3_c27675	3	2	gnl|CDD|32699	COG2872, COG2872, Predicte	ref|NP_001133686.1|	Alanyl-tRNA synthetase domain-containing protein 1 [Salmo salar] gb|ACI33700.1| Alanyl-tRNA synthetase domain-containing protein 1 [Salmo salar]	2,00E-42
gALL_v3_c27676	2	2	-	-	-	-	-
gALL_v3_c27677	2	2	-	-	-	-	-

gALL_v3_c27678	2	2	-	-	gb|EFZ21804.1|	hypothetical protein SINV_13891 [Solenopsis invicta]	1,00E-07
gALL_v3_c27679	2	2	-	-	ref|XP_003251820.1|	PREDICTED: zinc finger protein 585A-like [Apis mellifera]	5,00E-49
gALL_v3_c2768	4	5	-	-	-	-	-
gALL_v3_c27680	0	3	-	-	-	-	-
gALL_v3_c27681	0	4	-	-	-	-	-
gALL_v3_c27682	2	4	-	-	-	-	-
gALL_v3_c27683	1	3	-	-	-	-	-
gALL_v3_c27684	3	3	-	-	-	-	-
gALL_v3_c27685	1	3	-	-	ref|XP_003244198.1|	PREDICTED: hypothetical protein LOC100573332 [Acyrthosiphon pisum]	6,00E-24
gALL_v3_c27687	4	6	-	-	ref|XP_002429830.1|	hypothetical protein Phum_PHUM452730 [Pediculus humanus corporis] gb|EEB17092.1| hypothetical protein Phum_PHUM452730 [Pediculus humanus corporis]	7,00E-06
gALL_v3_c27688	4	3	-	-	ref|XP_974881.1|	PREDICTED: similar to AGAP000114-PA [Tribolium castaneum]	2,00E-91
gALL_v3_c27689	2	2	-	-	-	-	-
gALL_v3_c27690	0	3	-	-	-	-	-
gALL_v3_c27691	2	2	-	-	-	-	-
gALL_v3_c27692	2	2	-	-	-	-	-
gALL_v3_c27694	4	3	-	-	ref|XP_001329422.1|	ankyrin repeat protein [Trichomonas vaginalis G3] gb|EAY17199.1| ankyrin repeat protein, putative [Trichomonas vaginalis G3]	5,00E-12
gALL_v3_c27696	2	2	-	-	-	-	-
gALL_v3_c27697	6	3	-	-	gb|AGM32626.1|	glucosyl/glucuronosyl transferase, partial [Coptotermes formosanus]	1,00E-47
gALL_v3_c27698	2	1	-	-	ref|XP_972184.1|	PREDICTED: similar to vesicle docking protein P115 [Tribolium castaneum] gb|EFA08682.1| hypothetical protein TcasGA2_TC006352 [Tribolium castaneum]	2,00E-44
gALL_v3_c27699	3	5	-	-	-	-	-
gALL_v3_c277	17	12	gnl|CDD|188148	TIGR01511, ATPase-IB1_Cu, cc	ref|XP_003493753.1|	PREDICTED: copper-transporting ATPase 1-like [Bombus impatiens]	2,00E-58
gALL_v3_c2770	19	30	gnl|CDD|128865	smart00602, VPS10, VPS10 do	ref|XP_002427164.1|	conserved hypothetical protein [Pediculus humanus corporis] gb|EEB14426.1| conserved hypothetical protein [Pediculus humanus corporis]	0
gALL_v3_c27700	0	4	-	-	ref|XP_004930158.1|	PREDICTED: protein naked cuticle homolog [Bombyx mori]	2,00E-13
gALL_v3_c27702	1	2	-	-	-	-	-
gALL_v3_c27703	0	4	-	-	-	-	-
gALL_v3_c27704	2	1	-	-	gb|EFN67634.1|	Putative uncharacterized transposon-derived protein F52C9.6 [Camponotus floridanus]	1,00E-11
gALL_v3_c27705	1	3	-	-	-	-	-
gALL_v3_c27706	4	5	-	-	-	-	-
gALL_v3_c27707	2	6	-	-	gb|EFA04104.1|	hypothetical protein TcasGA2_TC014339 [Tribolium castaneum]	2,00E-72
gALL_v3_c27708	1	3	gnl|CDD|176878	cd08869, START_RhoGAP, C-te	gb|EFN71418.1|	StAR-related lipid transfer protein 13 [Camponotus floridanus]	1,00E-87
gALL_v3_c27710	2	2	-	-	-	-	-
gALL_v3_c27711	3	4	-	-	-	-	-
gALL_v3_c27712	1	3	-	-	-	-	-
gALL_v3_c27713	3	5	-	-	-	-	-
gALL_v3_c27714	3	2	-	-	-	-	-
gALL_v3_c27715	3	11	-	-	-	-	-
gALL_v3_c27716	3	5	-	-	ref|XP_319054.4|	AGAP009932-PA [Anopheles gambiae str. PEST] gb|EAA14102.4| AGAP009932-PA [Anopheles gambiae str. PEST]	3,00E-16
gALL_v3_c27717	1	3	-	-	-	-	-
gALL_v3_c27718	2	2	-	-	-	-	-
gALL_v3_c2772	5	2	-	-	-	-	-
gALL_v3_c27720	1	4	-	-	-	-	-
gALL_v3_c27722	2	2	-	-	-	-	-
gALL_v3_c27723	1	3	-	-	-	-	-
gALL_v3_c27724	2	1	-	-	-	-	-
gALL_v3_c27725	0	4	-	-	ref|XP_001949080.2|	PREDICTED: protein PIEZO2-like [Acyrthosiphon pisum]	3,00E-39
gALL_v3_c27726	2	1	-	-	-	-	-
gALL_v3_c27727	1	3	-	-	-	-	-
gALL_v3_c27728	2	7	-	-	-	-	-
gALL_v3_c27729	2	1	-	-	-	-	-
gALL_v3_c2773	2	10	-	-	emb|CDI77044.1|	hypothetical protein EPH_0007420 [Eimeria praecox]	2,00E-06
gALL_v3_c27730	3	4	-	-	-	-	-
gALL_v3_c27731	1	3	-	-	-	-	-
gALL_v3_c27732	1	3	-	-	-	-	-
gALL_v3_c27733	3	1	-	-	gb|EFA13284.1|	hypothetical protein TcasGA2_TC010304 [Tribolium castaneum]	1,00E-15
gALL_v3_c27734	0	3	-	-	-	-	-
gALL_v3_c27735	3	2	-	-	-	-	-
gALL_v3_c27737	2	2	-	-	-	-	-
gALL_v3_c27738	2	1	-	-	-	-	-
gALL_v3_c27739	0	3	-	-	-	-	-
gALL_v3_c27740	8	9	-	-	-	-	-
gALL_v3_c27741	4	2	-	-	-	-	-
gALL_v3_c27742	2	1	gnl|CDD|191620	pfam06842, DUF1242, Protein	gb|ABM55427.1|	conserved secreted salivary protein [Xenopsylla cheopis]	3,00E-28
gALL_v3_c27743	3	3	-	-	-	-	-
gALL_v3_c27746	2	3	-	-	gb|EFN67634.1|	Putative uncharacterized transposon-derived protein F52C9.6 [Camponotus floridanus]	7,00E-12
gALL_v3_c27747	2	4	-	-	-	-	-
gALL_v3_c27748	2	2	-	-	-	-	-
gALL_v3_c27749	2	2	-	-	-	-	-
gALL_v3_c2775	8	11	-	-	ref|XP_002410496.1|	lysine-specific histone demethylase, putative [Ixodes scapularis] gb|EEC11164.1| lysine-specific histone demethylase, putative [Ixodes scapularis]	4,00E-13
gALL_v3_c27751	3	1	-	-	-	-	-
gALL_v3_c27752	0	4	gnl|CDD|189954	pfam01359, Transposase_1, Tr	ref|XP_003707187.1|	PREDICTED: histone-lysine N-methyltransferase SETMAR-like [Megachile rotundata]	7,00E-58
gALL_v3_c27753	1	4	-	-	-	-	-
gALL_v3_c27754	2	1	-	-	-	-	-
gALL_v3_c27755	1	2	-	-	-	-	-
gALL_v3_c27756	0	3	-	-	-	-	-
gALL_v3_c27757	0	3	-	-	gb|EEZ97790.1|	hypothetical protein TcasGA2_TC030578 [Tribolium castaneum]	3,00E-07
gALL_v3_c27758	2	3	-	-	-	-	-
gALL_v3_c2776	23	25	-	-	ref|XP_002431918.1|	reverse transcriptase, putative [Pediculus humanus corporis] gb|EEB19180.1| reverse transcriptase, putative [Pediculus humanus corporis]	3,00E-15
gALL_v3_c27760	3	1	-	-	emb|CAI26307.1|	beta-alanyl conjugating enzyme [Periplaneta americana]	1,00E-69
gALL_v3_c27762	2	1	gnl|CDD|162107	TIGR00918, 2A060602, The Eu	ref|XP_002428547.1|	patched 1, putative [Pediculus humanus corporis] gb|EEB15809.1| patched 1, putative [Pediculus humanus corporis]	2,00E-86
gALL_v3_c27763	1	4	-	-	gb|EFN70309.1|	Caskin-1 [Camponotus floridanus]	3,00E-26
gALL_v3_c27764	7	4	gnl|CDD|112934	pfam04142, Nuc_sug_transp,	gb|ERL94410.1|	hypothetical protein D910_11688 [Dendroctonus ponderosae]	9,00E-44
gALL_v3_c27766	3	1	-	-	-	-	-
gALL_v3_c27768	0	4	-	-	gb|AFZ78825.1|	putative C-type lectin [Coptotermes formosanus] gb|AFZ78855.1| C-type lectin [Coptotermes formosanus]	6,00E-28
gALL_v3_c27769	4	4	-	-	-	-	-
gALL_v3_c2777	7	10	gnl|CDD|143356	cd07851, STKc_p38, Catalytic (	gb|ADT91683.1|	p38 mitogen-activated protein kinase [Apis cerana cerana] gb|ADT91685.1| p38 mitogen-activated protein kinase [Apis cerana cerana]	0
gALL_v3_c27770	2	1	-	-	-	-	-
gALL_v3_c27771	3	1	-	-	ref|XP_003703275.1|	PREDICTED: protein SMG5-like [Megachile rotundata]	3,00E-22
gALL_v3_c27772	3	2	-	-	gb|AGM32928.1|	mitochondrial intermembrane space import and assembly protein [Coptotermes formosanus]	3,00E-80
gALL_v3_c27773	4	3	-	-	-	-	-
gALL_v3_c27774	2	2	-	-	gb|EFA09598.1|	hypothetical protein TcasGA2_TC011717 [Tribolium castaneum]	3,00E-16
gALL_v3_c27776	2	1	gnl|CDD|128980	smart00741, SapB, Saposin (B)	gb|AAY89926.1|	saposin-like protein 2 precursor [Trichomonas vaginalis]	3,00E-19
gALL_v3_c27777	2	2	-	-	-	-	-
gALL_v3_c27778	2	2	-	-	-	-	-
gALL_v3_c27779	2	3	-	-	gb|AGM32643.1|	cytochrome P450 [Coptotermes formosanus]	1,00E-08
gALL_v3_c2778	7	12	gnl|CDD|191070	pfam04722, Ssu72, Ssu72-like	ref|XP_002432227.1|	protein SSU72, putative [Pediculus humanus corporis] gb|EEB19489.1| protein SSU72, putative [Pediculus humanus corporis]	1,00E-112
gALL_v3_c27780	4	6	gnl|CDD|191169	pfam05028, PARG_cat, Poly (A	ref|XP_003706927.1|	PREDICTED: poly(ADP-ribose) glycohydrolase 1-like isoform 1 [Megachile rotundata]	1,00E-119
gALL_v3_c27781	3	2	-	-	-	-	-
gALL_v3_c27782	2	2	-	-	-	-	-
gALL_v3_c27783	3	5	-	-	dbj|BAJ79290.1|	geranylgeranyl diphosphate synthase [Reticulitermes speratus]	2,00E-53
gALL_v3_c27784	2	2	-	-	-	-	-
gALL_v3_c27785	4	3	-	-	-	-	-
gALL_v3_c27786	0	3	-	-	-	-	-
gALL_v3_c27787	2	2	-	-	-	-	-
gALL_v3_c27788	0	3	-	-	-	-	-
gALL_v3_c27789	2	3	-	-	-	-	-
gALL_v3_c2779	8	5	-	-	-	-	-
gALL_v3_c27790	0	3	-	-	-	-	-
gALL_v3_c27791	3	3	-	-	-	-	-
gALL_v3_c27792	2	2	gnl|CDD|146828	pfam04388, Hamartin, Hamar	gb|EGI62010.1|	Hamartin [Acromyrmex echinatior]	9,00E-26
gALL_v3_c27794	6	3	gnl|CDD|128516	smart00220, S_TKc, Serine/Th	ref|XP_003704355.1|	PREDICTED: serine/threonine-protein kinase 17A-like [Megachile rotundata]	1,00E-82
gALL_v3_c27795	2	2	-	-	-	-	-
gALL_v3_c27796	3	7	-	-	-	-	-
gALL_v3_c27797	3	9	-	-	-	-	-
gALL_v3_c27799	0	3	-	-	-	-	-
gALL_v3_c278	6	9	-	-	-	-	-
gALL_v3_c27800	3	2	gnl|CDD|149648	pfam08662, eIF2A, Eukaryotic	ref|XP_002427345.1|	eukaryotic translation initiation factor 3 subunit, putative [Pediculus humanus corporis] gb|EEB14607.1| eukaryotic translation initiation factor 3 subunit, putative [Pedicul	4,00E-55
gALL_v3_c27801	5	5	-	-	ref|XP_004850358.1|	PREDICTED: uncharacterized protein LOC101721125 [Heterocephalus glaber]	1,00E-35
gALL_v3_c27802	1	3	-	-	-	-	-
gALL_v3_c27804	3	1	gnl|CDD|191613	pfam06807, Clp1, Pre-mRNA c	ref|XP_004453557.1|	PREDICTED: polynucleotide 5'-hydroxyl-kinase NOL9 [Dasypus novemcinctus]	2,00E-27
gALL_v3_c27805	2	2	-	-	-	-	-
gALL_v3_c27806	0	6	gnl|CDD|148286	pfam06585, JHBP, Haemolymp	dbj|BAD91203.1|	soldier-specific protein-1 [Nasutitermes takasagoensis]	2,00E-40
gALL_v3_c27808	2	2	-	-	gb|EFA11305.1|	hypothetical protein TcasGA2_TC010842 [Tribolium castaneum]	6,00E-21
gALL_v3_c27809	2	2	-	-	ref|XP_002426255.1|	protein MLP1, putative [Pediculus humanus corporis] gb|EEB13517.1| protein MLP1, putative [Pediculus humanus corporis]	2,00E-96
gALL_v3_c2781	14	15	-	-	-	-	-
gALL_v3_c27810	2	2	-	-	-	-	-
gALL_v3_c27812	0	4	-	-	gb|EFA02604.1|	hypothetical protein TcasGA2_TC008323 [Tribolium castaneum]	3,00E-49
gALL_v3_c27813	2	2	-	-	-	-	-
gALL_v3_c27814	2	5	-	-	ref|XP_002061278.1|	GK20808 [Drosophila willistoni] gb|EDW72264.1| GK20808 [Drosophila willistoni]	2,00E-26
gALL_v3_c27815	2	3	-	-	-	-	-
gALL_v3_c27816	3	3	-	-	ref|XP_973087.1|	PREDICTED: similar to Inositol 1,4,5-triphosphate kinase 1 CG4026-PA [Tribolium castaneum]	3,00E-30
gALL_v3_c27817	1	4	gnl|CDD|34708	COG5105, MIH1, Mitotic induc	dbj|BAO51656.1|	cell division cycle 25 homologue [Athalia rosae]	3,00E-25
gALL_v3_c27818	0	3	-	-	-	-	-
gALL_v3_c2782	3	6	-	-	-	-	-
gALL_v3_c27820	3	1	-	-	gb|EFZ20434.1|	hypothetical protein SINV_01500 [Solenopsis invicta]	7,00E-86
gALL_v3_c27822	1	2	-	-	-	-	-
gALL_v3_c27823	2	1	gnl|CDD|193253	pfam12777, MT, Microtubule-l	ref|XP_001843519.1|	dynein heavy chain [Culex quinquefasciatus] gb|EDS32929.1| dynein heavy chain [Culex quinquefasciatus]	6,00E-29
gALL_v3_c27824	3	2	-	-	-	-	-
gALL_v3_c27825	2	4	-	-	-	-	-
gALL_v3_c27826	2	1	-	-	-	-	-
gALL_v3_c27828	2	1	gnl|CDD|176349	cd01273, CED-6, CED-6 Phospl	gb|EGI58300.1|	PTB domain-containing adapter protein ced-6 [Acromyrmex echinatior]	3,00E-42
gALL_v3_c2783	6	6	gnl|CDD|188356	TIGR03625, L3_bact, 50S ribos	gb|EFN76611.1|	39S ribosomal protein L3, mitochondrial [Harpegnathos saltator]	4,00E-95
gALL_v3_c27830	0	3	-	-	-	-	-
gALL_v3_c27831	1	2	-	-	-	-	-
gALL_v3_c27832	1	2	-	-	-	-	-

gALL_v3_c27833	1	2	-	-	-	-	-
gALL_v3_c27835	2	4	-	-	-	-	-
gALL_v3_c27836	2	1	-	-	-	-	-
gALL_v3_c27837	3	1	-	-	-	-	-
gALL_v3_c27838	2	2	-	-	-	-	-
gALL_v3_c2784	4	6	-	-	-	-	-
gALL_v3_c27840	1	3	-	-	-	-	-
gALL_v3_c27841	3	3	-	-	-	-	-
gALL_v3_c27843	2	1	-	-	-	-	-
gALL_v3_c27844	1	2	-	-	gb|EMP39244.1|	N-acetyllactosaminide beta-1,3-N-acetylglucosaminyltransferase [Chelonia mydas]	2,00E-47
gALL_v3_c27845	1	2	-	-	-	-	-
gALL_v3_c27846	4	4	-	-	-	-	-
gALL_v3_c27847	3	1	-	-	-	-	-
gALL_v3_c27848	0	4	-	-	-	-	-
gALL_v3_c27849	2	2	gnl|CDD|72875	cd04513, Glycosylasparaginas	gb|EFZ21943.1|	hypothetical protein SINV_04012 [Solenopsis invicta]	5,00E-20
gALL_v3_c2785	13	8	-	-	ref|XP_002612385.1|	hypothetical protein BRAFLDRAFT_280125 [Branchiostoma floridae] gb|EEN68394.1| hypothetical protein BRAFLDRAFT_280125 [Branchiostoma floridae]	3,00E-07
gALL_v3_c27851	1	2	gnl|CDD|143472	cd06559, Endonuclease_V, En	ref|XP_006812585.1|	PREDICTED: endonuclease V-like [Saccoglossus kowalevskii]	6,00E-13
gALL_v3_c27852	2	2	-	-	gb|AGM32958.1|	hypothetical protein [Coptotermes formosanus]	1,00E-46
gALL_v3_c27853	2	6	-	-	ref|XP_003700384.1|	PREDICTED: LOW QUALITY PROTEIN: protein unc-79 homolog [Megachile rotundata]	1,00E-114
gALL_v3_c27854	2	2	-	-	-	-	-
gALL_v3_c27856	2	2	-	-	-	-	-
gALL_v3_c27857	2	2	-	-	-	-	-
gALL_v3_c27858	4	5	-	-	-	-	-
gALL_v3_c27859	2	1	-	-	-	-	-
gALL_v3_c2786	15	17	gnl|CDD|185440	PTZ00092, PTZ00092, aconitat	ref|XP_003695791.1|	PREDICTED: cytoplasmic aconitate hydratase [Apis florea]	0
gALL_v3_c27860	3	2	-	-	-	-	-
gALL_v3_c27862	0	3	-	-	-	-	-
gALL_v3_c27863	1	2	-	-	-	-	-
gALL_v3_c27864	5	4	-	-	-	-	-
gALL_v3_c27865	0	3	-	-	-	-	-
gALL_v3_c27866	2	3	-	-	-	-	-
gALL_v3_c27867	0	3	-	-	-	-	-
gALL_v3_c2787	8	6	-	-	-	-	-
gALL_v3_c27870	2	2	-	-	-	-	-
gALL_v3_c27871	2	1	-	-	-	-	-
gALL_v3_c27872	3	4	gnl|CDD|189372	pfam00067, p450, Cytochrom	sp|Q964R1.1|CP6J1_BL	RecName: Full=Cytochrome P450 6j1; AltName: Full=CYPVIJ1 gb|AAK57913.1|AF281325_1 cytochrome P450 CYP6J1 [Blattella germanica]	1,00E-83
gALL_v3_c27873	2	1	-	-	gb|EFN60907.1|	Peroxidase [Camponotus floridanus]	1,00E-17
gALL_v3_c27874	2	1	-	-	gb|AAH07440.1|	HACL1 protein [Homo sapiens]	9,00E-16
gALL_v3_c27876	3	1	-	-	-	-	-
gALL_v3_c27878	3	4	-	-	ref|XP_002431382.1|	conserved hypothetical protein [Pediculus humanus corporis] gb|EEB18644.1| conserved hypothetical protein [Pediculus humanus corporis]	2,00E-34
gALL_v3_c27880	2	2	gnl|CDD|162267	TIGR01243, CDC48, AAA famil	gb|ERL86916.1|	hypothetical protein D910_04319, partial [Dendroctonus ponderosae]	2,00E-56
gALL_v3_c27881	1	3	-	-	-	-	-
gALL_v3_c27882	2	2	-	-	-	-	-
gALL_v3_c27884	0	3	-	-	-	-	-
gALL_v3_c27885	0	3	-	-	gb|EFN64434.1|	Myosin-IXa [Camponotus floridanus]	8,00E-46
gALL_v3_c27886	0	3	-	-	-	-	-
gALL_v3_c27887	3	3	-	-	-	-	-
gALL_v3_c27888	2	3	gnl|CDD|130753	TIGR01692, HIBADH, 3-hydrox	ref|XP_003731339.1|	PREDICTED: 3-hydroxyisobutyrate dehydrogenase, mitochondrial-like, partial [Strongylocentrotus purpuratus]	1,00E-30
gALL_v3_c2789	8	6	-	-	-	-	-
gALL_v3_c27890	1	4	-	-	gb|EHB15952.1|	hypothetical protein GW7_19510, partial [Heterocephalus glaber]	1,00E-08
gALL_v3_c27891	2	2	gnl|CDD|189642	pfam00629, MAM, MAM dom	gb|EGI59707.1|	MAM domain-containing glycosylphosphatidylinositol anchor protein 1 [Acromyrmex echinatior]	3,00E-30
gALL_v3_c27892	0	3	-	-	-	-	-
gALL_v3_c27893	0	3	-	-	-	-	-
gALL_v3_c27895	1	2	-	-	-	-	-
gALL_v3_c27896	2	1	-	-	-	-	-
gALL_v3_c27897	1	2	-	-	-	-	-
gALL_v3_c27898	1	2	-	-	-	-	-
gALL_v3_c27899	2	3	-	-	-	-	-
gALL_v3_c279	9	23	gnl|CDD|119392	cd06174, MFS, The Major Faci	ref|XP_006611240.1|	PREDICTED: uncharacterized protein LOC102671976 [Apis dorsata]	1,00E-108
gALL_v3_c27901	1	3	-	-	-	-	-
gALL_v3_c27902	4	7	-	-	-	-	-
gALL_v3_c27903	2	1	-	-	-	-	-
gALL_v3_c27905	2	2	gnl|CDD|128524	smart00228, PDZ, Domain pre	ref|XP_002427634.1|	whirlin, putative [Pediculus humanus corporis] gb|EEB14896.1| whirlin, putative [Pediculus humanus corporis]	8,00E-75
gALL_v3_c27906	2	2	-	-	ref|XP_003707759.1|	PREDICTED: alpha-tocopherol transfer protein-like [Megachile rotundata]	4,00E-09
gALL_v3_c27907	0	3	-	-	ref|XP_003707599.1|	PREDICTED: zinc finger protein 626-like [Megachile rotundata]	6,00E-11
gALL_v3_c27908	2	2	-	-	ref|XP_006816925.1|	PREDICTED: nucleolar protein 9-like [Saccoglossus kowalevskii]	2,00E-13
gALL_v3_c27909	3	1	-	-	-	-	-
gALL_v3_c27910	1	2	-	-	-	-	-
gALL_v3_c27911	5	15	-	-	ref|XP_003735074.1|	PREDICTED: uncharacterized protein LOC100895946 [Callithrix jacchus]	4,00E-06
gALL_v3_c27912	5	5	-	-	-	-	-
gALL_v3_c27913	0	3	-	-	-	-	-
gALL_v3_c27914	2	2	-	-	-	-	-
gALL_v3_c27915	0	3	-	-	-	-	-
gALL_v3_c27916	2	1	-	-	-	-	-
gALL_v3_c27917	2	2	-	-	-	-	-
gALL_v3_c27918	0	3	-	-	-	-	-
gALL_v3_c27919	2	1	-	-	-	-	-
gALL_v3_c2792	2	6	gnl|CDD|191072	pfam04727, ELMO_CED12, El	ref|XP_002609095.1|	hypothetical protein BRAFLDRAFT_91068 [Branchiostoma floridae] gb|EEN65105.1| hypothetical protein BRAFLDRAFT_91068 [Branchiostoma floridae]	1,00E-09
gALL_v3_c27920	2	1	-	-	-	-	-
gALL_v3_c27921	0	4	-	-	ref|XP_005102509.1|	PREDICTED: uncharacterized protein LOC101859112 [Aplysia californica]	4,00E-17
gALL_v3_c27922	3	2	-	-	-	-	-
gALL_v3_c27923	3	1	-	-	-	-	-
gALL_v3_c27925	2	3	-	-	-	-	-
gALL_v3_c27926	11	12	-	-	-	-	-
gALL_v3_c27927	2	3	-	-	-	-	-
gALL_v3_c27928	3	1	-	-	-	-	-
gALL_v3_c27929	2	3	-	-	-	-	-
gALL_v3_c2793	5	4	-	-	-	-	-
gALL_v3_c27930	2	3	-	-	gb|EGI69416.1|	HMG box-containing protein 4 [Acromyrmex echinatior]	3,00E-31
gALL_v3_c27931	2	6	-	-	ref|XP_001945420.2|	PREDICTED: transmembrane protein 63A-like [Acyrthosiphon pisum]	1,00E-10
gALL_v3_c27932	0	4	-	-	-	-	-
gALL_v3_c27933	1	2	-	-	ref|XP_973684.1|	PREDICTED: similar to conserved hypothetical protein [Tribolium castaneum] gb|EEZ99060.1| hypothetical protein TcasGA2_TC004935 [Tribolium castaneum]	1,00E-70
gALL_v3_c27934	2	5	-	-	gb|EFA11377.1|	hypothetical protein TcasGA2_TC011536 [Tribolium castaneum]	9,00E-19
gALL_v3_c27935	2	1	-	-	-	-	-
gALL_v3_c27936	2	2	gnl|CDD|144978	pfam01585, G-patch, G-patch	gb|EFN84165.1|	Tuftelin-interacting protein 11 [Harpegnathos saltator]	3,00E-42
gALL_v3_c27937	3	3	-	-	-	-	-
gALL_v3_c27938	1	5	gnl|CDD|189411	pfam00135, COesterase, Carb	gb|AGM32277.1|	carboxylesterase-6-like protein, partial [Coptotermes formosanus]	9,00E-98
gALL_v3_c27939	2	1	gnl|CDD|189411	pfam00135, COesterase, Carb	gb|AHJ81320.1|	carboxylesterase [Locusta migratoria]	1,00E-27
gALL_v3_c2794	5	6	-	-	-	-	-
gALL_v3_c27940	1	2	-	-	-	-	-
gALL_v3_c27941	2	3	-	-	-	-	-
gALL_v3_c27942	1	3	-	-	ref|XP_002434058.1|	transmembrane protein, putative [Ixodes scapularis] gb|EEC05458.1| transmembrane protein, putative [Ixodes scapularis]	2,00E-30
gALL_v3_c27943	1	3	-	-	-	-	-
gALL_v3_c27945	2	1	-	-	-	-	-
gALL_v3_c27946	0	3	-	-	-	-	-
gALL_v3_c27947	0	3	-	-	-	-	-
gALL_v3_c27948	1	3	gnl|CDD|193257	pfam12781, AAA_9, ATP-bindi	ref|XP_005099385.1|	PREDICTED: dynein heavy chain 2, axonemal-like [Aplysia californica]	7,00E-54
gALL_v3_c27949	2	1	-	-	-	-	-
gALL_v3_c27950	2	3	-	-	ref|XP_002430992.1|	E3 ubiquitin ligase triad3, putative [Pediculus humanus corporis] gb|EEB18254.1| E3 ubiquitin ligase triad3, putative [Pediculus humanus corporis]	4,00E-20
gALL_v3_c27951	2	2	-	-	-	-	-
gALL_v3_c27952	3	2	-	-	-	-	-
gALL_v3_c27953	3	1	-	-	-	-	-
gALL_v3_c27955	1	4	-	-	-	-	-
gALL_v3_c27956	2	2	-	-	-	-	-
gALL_v3_c27958	0	4	-	-	-	-	-
gALL_v3_c27959	3	1	-	-	-	-	-
gALL_v3_c27960	0	4	-	-	-	-	-
gALL_v3_c27962	1	2	-	-	-	-	-
gALL_v3_c27963	1	4	-	-	-	-	-
gALL_v3_c27964	3	2	-	-	-	-	-
gALL_v3_c27966	4	3	gnl|CDD|163230	TIGR03356, BGL, beta-galacto	gb|AGM32287.1|	beta-glucosidase, partial [Coptotermes formosanus]	2,00E-73
gALL_v3_c27967	5	2	-	-	-	-	-
gALL_v3_c27968	2	2	-	-	-	-	-
gALL_v3_c2797	6	10	-	-	-	-	-
gALL_v3_c27970	2	2	-	-	-	-	-
gALL_v3_c27972	1	6	-	-	-	-	-
gALL_v3_c27973	1	2	-	-	-	-	-
gALL_v3_c27974	3	3	-	-	ref|XP_003426634.1|	PREDICTED: hypoxia up-regulated protein 1-like isoform 2 [Nasonia vitripennis] ref|XP_001606217.2| PREDICTED: hypoxia up-regulated protein 1-like isoform 1 [Nasonia v	2,00E-57
gALL_v3_c27975	2	4	gnl|CDD|29257	cd00200, WD40, WD40 domai	gb|EFN86018.1|	WD repeat-containing protein 32-like protein [Harpegnathos saltator]	1,00E-106
gALL_v3_c27976	1	3	-	-	-	-	-
gALL_v3_c27977	3	1	-	-	ref|XP_006029730.1|	PREDICTED: histone-lysine N-methyltransferase SETMAR-like [Alligator sinensis]	2,00E-08
gALL_v3_c27978	2	2	-	-	-	-	-
gALL_v3_c2798	21	28	-	-	ref|XP_967427.2|	PREDICTED: similar to hypoxia-inducible factor 1 alpha [Tribolium castaneum]	3,00E-10
gALL_v3_c27980	2	2	-	-	-	-	-
gALL_v3_c27981	2	8	-	-	-	-	-
gALL_v3_c27982	0	3	-	-	-	-	-
gALL_v3_c27983	2	2	-	-	-	-	-
gALL_v3_c27984	2	2	-	-	-	-	-
gALL_v3_c27985	2	1	-	-	-	-	-
gALL_v3_c27987	0	4	-	-	-	-	-
gALL_v3_c27988	1	2	gnl|CDD|178622	PLN03073, PLN03073, ABC tra	ref|XP_972814.1|	PREDICTED: similar to ATP-dependent transporter [Tribolium castaneum] gb|EFA09785.1| hypothetical protein TcasGA2_TC011927 [Tribolium castaneum]	5,00E-52
gALL_v3_c2799	17	16	-	-	gb|ELW65852.1|	Brain-specific homeobox protein like protein [Tupaia chinensis]	4,00E-07
gALL_v3_c27991	2	2	-	-	-	-	-
gALL_v3_c27992	2	2	-	-	-	-	-

gALL_v3_c27993	2	4	gnl|CDD|161898	TIGR00476, selD, selenium do	ref|NP_725374.1|	Selenide,water dikinase, isoform A [Drosophila melanogaster] ref|NP_725375.1| Selenide,water dikinase, isoform B [Drosophila melanogaster] ref|XP_002033822.1| GM2	1,00E-73
gALL_v3_c27994	5	3	-	-	ref|NP_001161918.1|	cuticular protein analogous to peritrophins 1-H precursor [Tribolium castaneum] gb|ACY95473.1| cuticular protein analogous to peritrophins 1-H [Tribolium castaneum] gb	4,00E-08
gALL_v3_c27996	1	2	-	-	-	-	-
gALL_v3_c27999	1	3	-	-	-	-	-
gALL_v3_c28	9	7	gnl|CDD|178457	PLN02866, PLN02866, phosph	gb|EFA02113.1|	hypothetical protein TcasGA2_TC007759 [Tribolium castaneum]	1,00E-129
gALL_v3_c280	15	12	gnl|CDD|173671	cd05580, STKc_PKA, Catalytic	ref|XP_003240902.1|	PREDICTED: cAMP-dependent protein kinase catalytic subunit-like [Acyrthosiphon pisum]	0
gALL_v3_c2800	11	10	-	-	gb|EGI63032.1|	Mariner Mos1 transposase [Acromyrmex echinatior]	2,00E-06
gALL_v3_c28000	4	4	-	-	gb|EHJ66377.1|	hypothetical protein KGM_06440 [Danaus plexippus]	6,00E-36
gALL_v3_c28001	3	1	-	-	-	-	-
gALL_v3_c28002	1	3	-	-	-	-	-
gALL_v3_c28003	1	3	-	-	ref|XP_002431865.1|	conserved hypothetical protein [Pediculus humanus corporis] gb|EEB19127.1| conserved hypothetical protein [Pediculus humanus corporis]	4,00E-12
gALL_v3_c28004	2	2	-	-	ref|XP_001602452.2|	PREDICTED: integrator complex subunit 2-like isoform 1 [Nasonia vitripennis]	7,00E-54
gALL_v3_c28005	1	3	-	-	-	-	-
gALL_v3_c28006	3	2	-	-	-	-	-
gALL_v3_c28007	1	3	-	-	ref|XP_002432866.1|	1-phosphatidylinositol-4,5-bisphosphate phosphodiesterase, putative [Pediculus humanus corporis] gb|EEB20128.1| 1-phosphatidylinositol-4,5-bisphosphate phosphodies	1,00E-36
gALL_v3_c28008	4	2	-	-	ref|XP_006565820.1|	PREDICTED: transcription factor 12 isoform X7 [Apis mellifera]	8,00E-25
gALL_v3_c28009	1	2	-	-	-	-	-
gALL_v3_c28010	1	2	-	-	-	-	-
gALL_v3_c28011	2	1	-	-	-	-	-
gALL_v3_c28012	2	2	-	-	-	-	-
gALL_v3_c28013	4	5	gnl|CDD|153339	cd07655, F-BAR_PACSIN, The I	gb|AGM32199.1|	protein kinase C and casein kinase substrate in neurons protein, partial [Coptotermes formosanus]	1,00E-78
gALL_v3_c28014	29	60	gnl|CDD|153079	cd07932, arginine_kinase_like	gb|ACA00204.1|	arginine kinase [Periplaneta americana]	3,00E-85
gALL_v3_c28015	4	4	-	-	-	-	-
gALL_v3_c28016	2	3	gnl|CDD|128516	smart00220, S_TKc, Serine/Th	gb|EFN84150.1|	Phosphorylase b kinase gamma catalytic chain, skeletal muscle isoform [Harpegnathos saltator]	6,00E-97
gALL_v3_c28019	0	4	-	-	gb|ADI61823.1|	endonuclease-reverse transcriptase [Bombyx mori]	7,00E-08
gALL_v3_c2802	24	37	-	-	-	-	-
gALL_v3_c28020	2	2	-	-	-	-	-
gALL_v3_c28021	1	8	-	-	gb|EGI60047.1|	RanBP-type and C3HC4-type zinc finger-containing protein 1 [Acromyrmex echinatior]	3,00E-11
gALL_v3_c28022	2	1	-	-	-	-	-
gALL_v3_c28023	2	3	-	-	-	-	-
gALL_v3_c28024	3	2	-	-	-	-	-
gALL_v3_c28025	0	5	-	-	-	-	-
gALL_v3_c28027	0	4	-	-	-	-	-
gALL_v3_c28028	0	5	-	-	-	-	-
gALL_v3_c28029	0	4	-	-	-	-	-
gALL_v3_c28030	2	3	gnl|CDD|162785	TIGR02251, HIF-SF_euk, Dullar	gb|EFZ17505.1|	hypothetical protein SINV_15653 [Solenopsis invicta]	1,00E-100
gALL_v3_c28031	2	3	-	-	-	-	-
gALL_v3_c28032	1	2	-	-	-	-	-
gALL_v3_c28033	3	3	gnl|CDD|148965	pfam07651, ANTH, ANTH dom	ref|XP_006563901.1|	PREDICTED: phosphatidylinositol-binding clathrin assembly protein LAP isoform X21 [Apis mellifera]	1,00E-124
gALL_v3_c28034	3	1	-	-	-	-	-
gALL_v3_c28035	2	1	-	-	-	-	-
gALL_v3_c28036	0	6	-	-	-	-	-
gALL_v3_c28037	0	3	-	-	-	-	-
gALL_v3_c28038	0	3	-	-	-	-	-
gALL_v3_c2804	3	8	-	-	-	-	-
gALL_v3_c28040	2	1	-	-	-	-	-
gALL_v3_c28041	3	7	-	-	gb|EHJ68213.1|	hypothetical protein KGM_12625 [Danaus plexippus]	1,00E-10
gALL_v3_c28042	2	2	gnl|CDD|176262	cd08367, P53, P53 DNA-bindir	ref|XP_002422610.1|	Cellular tumor antigen p53, putative [Pediculus humanus corporis] gb|EEB09872.1| Cellular tumor antigen p53, putative [Pediculus humanus corporis]	3,00E-24
gALL_v3_c28043	0	3	-	-	ref|XP_002430872.1|	conserved hypothetical protein [Pediculus humanus corporis] gb|EEB18134.1| conserved hypothetical protein [Pediculus humanus corporis]	1,00E-10
gALL_v3_c28044	3	3	-	-	gb|EFA06653.1|	hypothetical protein TcasGA2_TC009577 [Tribolium castaneum]	2,00E-06
gALL_v3_c28045	4	8	-	-	-	-	-
gALL_v3_c28046	0	3	-	-	-	-	-
gALL_v3_c28047	0	4	-	-	-	-	-
gALL_v3_c28048	1	3	-	-	-	-	-
gALL_v3_c28049	0	3	-	-	-	-	-
gALL_v3_c28050	3	3	-	-	ref|XP_001949570.2|	PREDICTED: 39S ribosomal protein L44, mitochondrial-like [Acyrthosiphon pisum]	2,00E-54
gALL_v3_c28051	0	3	gnl|CDD|192200	pfam09058, L27_1, L27_1. Th	ref|XP_003399624.1|	PREDICTED: hypothetical protein LOC100644100 [Bombus terrestris]	2,00E-52
gALL_v3_c28052	2	5	gnl|CDD|189434	pfam00176, SNF2_N, SNF2 far	gb|ADE34301.1|	MIP05021p [Drosophila melanogaster]	1,00E-161
gALL_v3_c28053	1	2	-	-	-	-	-
gALL_v3_c28054	2	2	-	-	-	-	-
gALL_v3_c28055	2	2	-	-	-	-	-
gALL_v3_c28056	1	4	-	-	-	-	-
gALL_v3_c28057	3	3	-	-	ref|XP_004922428.1|	PREDICTED: inhibitor of Bruton tyrosine kinase-like [Bombyx mori]	2,00E-49
gALL_v3_c28058	1	2	-	-	-	-	-
gALL_v3_c2806	14	17	-	-	-	-	-
gALL_v3_c28062	2	2	-	-	-	-	-
gALL_v3_c28063	0	3	-	-	-	-	-
gALL_v3_c28064	4	4	gnl|CDD|29331	cd00256, VATPase_H, VATPase	gb|AFN11665.1|	vacuolar ATPase subunit H [Locusta migratoria manilensis]	3,00E-99
gALL_v3_c28065	2	2	-	-	-	-	-
gALL_v3_c28066	2	4	-	-	-	-	-
gALL_v3_c28067	1	3	-	-	-	-	-
gALL_v3_c28068	0	3	-	-	-	-	-
gALL_v3_c28069	1	3	-	-	-	-	-
gALL_v3_c28070	3	2	-	-	-	-	-
gALL_v3_c28071	2	5	gnl|CDD|48545	cd02996, PDI_a_ERp44, PDIa f	ref|XP_971182.1|	PREDICTED: similar to CG9911 CG9911-PA [Tribolium castaneum] gb|EFA09317.1| hypothetical protein TcasGA2_TC001643 [Tribolium castaneum]	1,00E-112
gALL_v3_c28072	2	2	-	-	-	-	-
gALL_v3_c28073	0	3	-	-	-	-	-
gALL_v3_c28074	0	3	gnl|CDD|189650	pfam00645, zf-PARP, Poly(ADP	ref|XP_001947212.1|	PREDICTED: poly [ADP-ribose] polymerase-like [Acyrthosiphon pisum]	7,00E-34
gALL_v3_c28077	1	2	-	-	-	-	-
gALL_v3_c28078	0	3	-	-	-	-	-
gALL_v3_c28079	2	2	-	-	-	-	-
gALL_v3_c2808	5	6	-	-	-	-	-
gALL_v3_c28080	2	3	-	-	ref|XP_004930809.1|	PREDICTED: vacuolar protein sorting-associated protein 13A-like [Bombyx mori]	6,00E-32
gALL_v3_c28081	1	3	-	-	-	-	-
gALL_v3_c28083	4	11	gnl|CDD|177210	MTH00153, COX1, cytochrome	gb|AAC52011.1|	mariner transposase [Homo sapiens]	4,00E-26
gALL_v3_c28084	2	1	-	-	-	-	-
gALL_v3_c28085	0	4	-	-	-	-	-
gALL_v3_c28086	1	3	-	-	-	-	-
gALL_v3_c28087	1	4	gnl|CDD|162643	TIGR01989, COQ6, Ubiquinone	gb|EFN64713.1|	Uncharacterized aarF domain-containing protein kinase 1 [Camponotus floridanus]	8,00E-39
gALL_v3_c28088	2	4	-	-	-	-	-
gALL_v3_c28089	0	5	-	-	-	-	-
gALL_v3_c2809	5	4	-	-	-	-	-
gALL_v3_c28090	2	2	-	-	-	-	-
gALL_v3_c28091	0	4	-	-	-	-	-
gALL_v3_c28092	1	2	-	-	-	-	-
gALL_v3_c28093	2	1	-	-	ref|XP_969244.1|	PREDICTED: similar to TWiK family of potassium channels family member (twk-46) [Tribolium castaneum]	1,00E-20
gALL_v3_c28094	11	10	-	-	gb|EFN75580.1|	hypothetical protein EAI_08636 [Harpegnathos saltator]	5,00E-14
gALL_v3_c28095	1	4	-	-	ref|XP_005302607.1|	PREDICTED: jerky protein-like [Chrysemys picta bellii]	3,00E-10
gALL_v3_c28096	2	1	-	-	-	-	-
gALL_v3_c28097	1	2	-	-	gb|AFI39863.1|	pelle, partial [Daphnia parvula]	2,00E-23
gALL_v3_c28099	0	3	-	-	-	-	-
gALL_v3_c28100	2	2	-	-	-	-	-
gALL_v3_c28101	1	3	-	-	ref|XP_004929330.1|	PREDICTED: LOW QUALITY PROTEIN: type II inositol 1,4,5-trisphosphate 5-phosphatase-like [Bombyx mori]	2,00E-08
gALL_v3_c28102	2	3	-	-	-	-	-
gALL_v3_c28104	2	4	-	-	-	-	-
gALL_v3_c28105	2	2	-	-	-	-	-
gALL_v3_c28106	0	6	gnl|CDD|128538	smart00242, MYSc, Myosin. La	ref|XP_006619236.1|	PREDICTED: unconventional myosin-XV-like isoform X2 [Apis dorsata]	3,00E-48
gALL_v3_c28107	4	4	gnl|CDD|128886	smart00638, LPD_N, Lipoprote	gb|EFX65940.1|	hypothetical protein DAPPUDRAFT_332702 [Daphnia pulex]	6,00E-41
gALL_v3_c28108	3	2	-	-	-	-	-
gALL_v3_c28109	2	2	-	-	-	-	-
gALL_v3_c2811	4	8	-	-	-	-	-
gALL_v3_c28110	2	7	-	-	-	-	-
gALL_v3_c28112	2	2	-	-	-	-	-
gALL_v3_c28113	5	6	gnl|CDD|144648	pfam01130, CD36, CD36 famil	ref|XP_002432097.1|	scavenger receptor class B member, putative [Pediculus humanus corporis] gb|EEB19359.1| scavenger receptor class B member, putative [Pediculus humanus corporis]	1,00E-108
gALL_v3_c28114	2	2	-	-	-	-	-
gALL_v3_c28115	1	4	-	-	-	-	-
gALL_v3_c28116	1	3	-	-	-	-	-
gALL_v3_c28118	1	2	-	-	-	-	-
gALL_v3_c28119	0	4	-	-	-	-	-
gALL_v3_c28120	1	1	-	-	ref|XP_006572166.1|	PREDICTED: TWiK family of potassium channels protein 7-like [Apis mellifera]	8,00E-28
gALL_v3_c28121	1	2	-	-	-	-	-
gALL_v3_c28122	1	2	gnl|CDD|190523	pfam03098, An_peroxidase, A	ref|XP_001807949.1|	PREDICTED: similar to oxidase/peroxidase [Tribolium castaneum]	6,00E-30
gALL_v3_c28123	2	1	-	-	gb|EFN75248.1|	PiggyBac transposable element-derived protein 4 [Harpegnathos saltator]	2,00E-12
gALL_v3_c28124	3	4	gnl|CDD|99927	cd05495, Bromo_cbp_like, Brc	ref|XP_003490706.1|	PREDICTED: hypothetical protein LOC100747609 [Bombus impatiens]	1,00E-144
gALL_v3_c28125	1	2	-	-	-	-	-
gALL_v3_c28127	2	1	-	-	-	-	-
gALL_v3_c28128	4	3	-	-	-	-	-
gALL_v3_c28129	1	2	-	-	-	-	-
gALL_v3_c2813	8	4	gnl|CDD|178447	PLN02856, PLN02856, fumary	ref|XP_002428283.1|	Fumarylacetoacetase, putative [Pediculus humanus corporis] gb|EEB15545.1| Fumarylacetoacetase, putative [Pediculus humanus corporis]	6,00E-54
gALL_v3_c28130	2	2	-	-	ref|XP_003699196.1|	PREDICTED: serine/threonine-protein kinase 3-like [Megachile rotundata]	5,00E-12
gALL_v3_c28131	1	2	-	-	-	-	-
gALL_v3_c28132	3	5	-	-	-	-	-
gALL_v3_c28133	1	2	-	-	-	-	-
gALL_v3_c28134	4	3	-	-	-	-	-
gALL_v3_c28135	2	4	-	-	-	-	-
gALL_v3_c28136	2	3	-	-	-	-	-
gALL_v3_c28137	0	3	-	-	-	-	-
gALL_v3_c28138	1	2	-	-	gb|EHJ78503.1|	endonuclease-reverse transcriptase [Danaus plexippus]	2,00E-22
gALL_v3_c28139	0	4	-	-	-	-	-
gALL_v3_c2814	5	5	-	-	gb|EFN78804.1|	Tetratricopeptide repeat protein 8 [Harpegnathos saltator]	1,00E-133
gALL_v3_c28140	2	5	-	-	-	-	-
gALL_v3_c28141	2	2	-	-	-	-	-
gALL_v3_c28142	0	3	gnl|CDD|189654	pfam00651, BTB, BTB/POZ dor	ref|XP_001945772.2|	PREDICTED: ankyrin repeat and FYVE domain-containing protein 1-like [Acyrthosiphon pisum]	4,00E-66
gALL_v3_c28143	6	12	-	-	ref|XP_006571091.1|	PREDICTED: calponin homology domain-containing protein DDB_G0272472-like isoform X9 [Apis mellifera]	3,00E-18

gALL_v3_c28144	0	3	-	-	ref|XP_002431064.1|	conserved hypothetical protein [Pediculus humanus corporis] gb|EEB18326.1| conserved hypothetical protein [Pediculus humanus corporis]	5,00E-07
gALL_v3_c28145	0	3	-	-	-	-	-
gALL_v3_c28147	3	1	-	-	ref|XP_004930217.1|	PREDICTED: facilitated trehalose transporter Tret1-like [Bombyx mori]	6,00E-20
gALL_v3_c28148	7	4	-	-	-	-	-
gALL_v3_c28150	3	2	-	-	-	-	-
gALL_v3_c28151	1	2	-	-	gb|EFN70827.1|	Vacuolar protein sorting-associated protein 52-like protein [Camponotus floridanus]	3,00E-28
gALL_v3_c28153	1	4	-	-	-	-	-
gALL_v3_c28154	0	3	-	-	-	-	-
gALL_v3_c28155	0	5	-	-	-	-	-
gALL_v3_c28156	2	3	-	-	ref|XP_003704776.1|	PREDICTED: colorectal mutant cancer protein-like [Megachile rotundata]	6,00E-27
gALL_v3_c28157	3	1	gnl|CDD|189981	pfam01425, Amidase, Amidase	ref|XP_001959388.1|	GF12071 [Drosophila ananassae] gb|EDV36210.1| GF12071 [Drosophila ananassae]	3,00E-38
gALL_v3_c28159	2	2	-	-	-	-	-
gALL_v3_c2816	19	32	-	-	-	-	-
gALL_v3_c28160	5	4	-	-	-	-	-
gALL_v3_c28161	0	4	-	-	-	-	-
gALL_v3_c28162	1	2	-	-	-	-	-
gALL_v3_c28163	3	2	-	-	-	-	-
gALL_v3_c28164	1	4	-	-	-	-	-
gALL_v3_c28165	3	6	-	-	-	-	-
gALL_v3_c28166	0	3	gnl|CDD|144126	pfam00413, Peptidase_M10, M	gb|EFZ15073.1|	hypothetical protein SINV_02809 [Solenopsis invicta]	4,00E-27
gALL_v3_c28167	1	4	-	-	ref|NP_001071859.1|	zinc finger protein [Ciona intestinalis] dbj|BAE06773.1| zinc finger protein [Ciona intestinalis]	5,00E-07
gALL_v3_c28168	1	6	-	-	ref|XP_003402427.1|	PREDICTED: protein turtle-like [Bombus terrestris]	9,00E-62
gALL_v3_c28169	2	1	-	-	-	-	-
gALL_v3_c2817	8	9	-	-	-	-	-
gALL_v3_c28170	1	4	-	-	ref|XP_001604189.2|	PREDICTED: protein MON2 homolog [Nasonia vitripennis]	6,00E-58
gALL_v3_c28171	3	4	gnl|CDD|143596	cd07572, nit, Nit1, Nit 2, and r	ref|XP_005113263.1|	PREDICTED: nitrilase and fragile histidine triad fusion protein NitFhit-like isoform X1 [Aplysia californica] ref|XP_005113264.1| PREDICTED: nitrilase and fragile histidine tria	3,00E-65
gALL_v3_c28172	0	3	-	-	-	-	-
gALL_v3_c28173	2	3	-	-	ref|XP_006615082.1|	PREDICTED: mitogen-activated protein kinase-binding protein 1-like isoform X8 [Apis dorsata]	3,00E-22
gALL_v3_c28174	2	4	-	-	-	-	-
gALL_v3_c28175	1	3	-	-	-	-	-
gALL_v3_c28176	3	1	-	-	ref|XP_006821712.1|	PREDICTED: uncharacterized protein LOC102806297 [Saccoglossus kowalevskii]	9,00E-13
gALL_v3_c28177	1	3	-	-	-	-	-
gALL_v3_c28178	3	1	-	-	-	-	-
gALL_v3_c28179	0	3	-	-	-	-	-
gALL_v3_c28180	2	2	-	-	-	-	-
gALL_v3_c28181	2	1	-	-	-	-	-
gALL_v3_c28182	2	7	-	-	-	-	-
gALL_v3_c28183	3	1	-	-	-	-	-
gALL_v3_c28184	3	6	-	-	ref|XP_004212946.1|	PREDICTED: piggyBac transposable element-derived protein 4-like [Hydra vulgaris]	6,00E-23
gALL_v3_c28185	1	3	-	-	-	-	-
gALL_v3_c28187	2	1	-	-	-	-	-
gALL_v3_c28188	1	4	-	-	-	-	-
gALL_v3_c2819	4	3	-	-	-	-	-
gALL_v3_c28190	1	1	gnl|CDD|58529	cd04414, NDPk6, Nucleoside c	gb|EFN73333.1|	Nucleoside diphosphate kinase 6 [Camponotus floridanus]	7,00E-48
gALL_v3_c28192	3	1	gnl|CDD|162048	TIGR00804, nupC, nucleoside t	gb|EGI62837.1|	Solute carrier family 28 member 3 [Acromyrmex echinatior]	3,00E-62
gALL_v3_c28193	0	3	-	-	-	-	-
gALL_v3_c28194	2	3	-	-	gb|ADI61823.1|	endonuclease-reverse transcriptase [Bombyx mori]	4,00E-47
gALL_v3_c28195	1	2	-	-	-	-	-
gALL_v3_c28196	1	4	-	-	gb|EEZ99246.1|	hypothetical protein TcasGA2_TC000871 [Tribolium castaneum]	2,00E-82
gALL_v3_c28197	1	3	-	-	-	-	-
gALL_v3_c282	14	9	-	-	-	-	-
gALL_v3_c2820	8	9	-	-	ref|XP_001862736.1|	conserved hypothetical protein [Culex quinquefasciatus] gb|EDS37428.1| conserved hypothetical protein [Culex quinquefasciatus]	1,00E-102
gALL_v3_c28200	3	1	-	-	-	-	-
gALL_v3_c28202	2	1	-	-	-	-	-
gALL_v3_c28203	1	3	gnl|CDD|192363	pfam09751, Es2, Nuclear prote	ref|XP_002432971.1|	es2 protein, putative [Pediculus humanus corporis] gb|EEB20233.1| es2 protein, putative [Pediculus humanus corporis]	6,00E-61
gALL_v3_c28204	3	4	-	-	ref|XP_001623794.1|	predicted protein [Nematostella vectensis] gb|EDO31694.1| predicted protein [Nematostella vectensis]	1,00E-07
gALL_v3_c28205	0	3	-	-	-	-	-
gALL_v3_c28206	2	2	-	-	-	-	-
gALL_v3_c28207	1	2	-	-	ref|XP_001892369.1|	hypothetical protein [Brugia malayi] gb|EDP38806.1| conserved hypothetical protein [Brugia malayi]	9,00E-09
gALL_v3_c28209	2	1	-	-	-	-	-
gALL_v3_c2821	11	14	gnl|CDD|192523	pfam10300, DUF3808, Protein	gb|EFN69425.1|	Tetratricopeptide repeat protein 39B [Camponotus floridanus]	1,00E-171
gALL_v3_c28211	1	2	-	-	-	-	-
gALL_v3_c28212	2	4	-	-	ref|XP_001605280.2|	PREDICTED: alpha-1,3-mannosyl-glycoprotein 4-beta-N-acetylglucosaminyltransferase B-like [Nasonia vitripennis]	5,00E-45
gALL_v3_c28213	2	1	-	-	-	-	-
gALL_v3_c28214	2	1	-	-	-	-	-
gALL_v3_c28215	2	1	-	-	-	-	-
gALL_v3_c28216	2	2	-	-	-	-	-
gALL_v3_c28217	2	1	-	-	ref|XP_003702638.1|	PREDICTED: ankyrin repeat and zinc finger domain-containing protein 1-like [Megachile rotundata]	4,00E-27
gALL_v3_c28218	1	3	-	-	-	-	-
gALL_v3_c28219	0	3	-	-	-	-	-
gALL_v3_c28220	2	3	-	-	gb|EFZ12186.1|	hypothetical protein SINV_00805 [Solenopsis invicta]	2,00E-24
gALL_v3_c28221	1	3	-	-	-	-	-
gALL_v3_c28224	3	3	-	-	-	-	-
gALL_v3_c28225	3	2	-	-	-	-	-
gALL_v3_c28226	1	4	gnl|CDD|189583	pfam00520, Ion_trans, Ion tra	gb|EFN63846.1|	Voltage-dependent calcium channel type D subunit alpha-1 [Camponotus floridanus]	3,00E-58
gALL_v3_c28227	2	2	-	-	-	-	-
gALL_v3_c28228	2	3	-	-	-	-	-
gALL_v3_c28229	0	4	-	-	-	-	-
gALL_v3_c2823	7	11	gnl|CDD|189368	pfam00060, Lig_chan, Ligand-g	ref|XP_002426455.1|	predicted protein [Pediculus humanus corporis] gb|EEB13717.1| predicted protein [Pediculus humanus corporis]	1,00E-135
gALL_v3_c28230	1	3	-	-	-	-	-
gALL_v3_c28231	5	2	-	-	-	-	-
gALL_v3_c28232	1	3	-	-	-	-	-
gALL_v3_c28233	1	2	gnl|CDD|149585	pfam08584, Ribonuc_P_40, Ri	ref|XP_003398472.1|	PREDICTED: LOW QUALITY PROTEIN: ribonuclease P protein subunit p40-like [Bombus terrestris]	2,00E-52
gALL_v3_c28234	0	3	-	-	-	-	-
gALL_v3_c28235	1	3	-	-	-	-	-
gALL_v3_c28236	3	1	-	-	-	-	-
gALL_v3_c28237	2	1	-	-	-	-	-
gALL_v3_c28238	3	1	gnl|CDD|190710	pfam03676, UPF0183, Unchar	ref|XP_003394534.1|	PREDICTED: UPF0183 protein CG7083-like [Bombus terrestris] ref|XP_003486521.1| PREDICTED: UPF0183 protein CG7083-like [Bombus impatiens]	3,00E-75
gALL_v3_c28239	1	2	-	-	-	-	-
gALL_v3_c28240	2	4	-	-	ref|XP_002424820.1|	conserved hypothetical protein [Pediculus humanus corporis] gb|EEB12082.1| conserved hypothetical protein [Pediculus humanus corporis]	6,00E-32
gALL_v3_c28241	3	4	gnl|CDD|34707	COG5104, PRP40, Splicing fact	ref|XP_006572735.1|	PREDICTED: pre-mRNA-processing factor 40 homolog A-like, partial [Apis mellifera]	1,00E-40
gALL_v3_c28242	3	3	gnl|CDD|145166	pfam01853, MOZ_SAS, MOZ/S	gb|EFN72673.1|	Probable histone acetyltransferase MYST1 [Camponotus floridanus]	3,00E-64
gALL_v3_c28244	0	3	-	-	-	-	-
gALL_v3_c28246	1	3	-	-	-	-	-
gALL_v3_c28247	0	3	-	-	-	-	-
gALL_v3_c28248	0	3	-	-	-	-	-
gALL_v3_c28249	2	1	-	-	-	-	-
gALL_v3_c2825	9	21	-	-	-	-	-
gALL_v3_c28250	1	2	-	-	-	-	-
gALL_v3_c28251	5	2	-	-	ref|XP_002433145.1|	conserved hypothetical protein [Pediculus humanus corporis] gb|EEB20407.1| conserved hypothetical protein [Pediculus humanus corporis]	1,00E-124
gALL_v3_c28253	1	2	-	-	-	-	-
gALL_v3_c28254	0	3	-	-	-	-	-
gALL_v3_c28255	2	1	-	-	-	-	-
gALL_v3_c28258	2	1	-	-	-	-	-
gALL_v3_c28259	1	3	-	-	-	-	-
gALL_v3_c2826	9	11	-	-	gb|EFN67634.1|	Putative uncharacterized transposon-derived protein F52C9.6 [Camponotus floridanus]	5,00E-14
gALL_v3_c28260	2	6	-	-	ref|XP_002424701.1|	conserved hypothetical protein [Pediculus humanus corporis] gb|EEB11963.1| conserved hypothetical protein [Pediculus humanus corporis]	3,00E-23
gALL_v3_c28261	3	8	gnl|CDD|162028	TIGR00763, lon, ATP-depender	gb|EFN79680.1|	Lon protease-like protein, mitochondrial [Harpegnathos saltator]	3,00E-76
gALL_v3_c28262	2	4	gnl|CDD|109161	pfam00093, VWC, von Willebr	ref|XP_003397177.1|	PREDICTED: protein kinase C-binding protein NELL1-like isoform 2 [Bombus terrestris]	1,00E-111
gALL_v3_c28263	0	3	-	-	-	-	-
gALL_v3_c28264	2	1	-	-	-	-	-
gALL_v3_c28265	0	3	-	-	-	-	-
gALL_v3_c28266	2	2	gnl|CDD|73009	cd03250, ABCC_MRP_domain	gb|EGI58005.1|	Putative multidrug resistance-associated protein lethal(2)03659 [Acromyrmex echinatior]	4,00E-32
gALL_v3_c28268	0	3	-	-	-	-	-
gALL_v3_c28269	3	1	-	-	-	-	-
gALL_v3_c2827	15	15	-	-	-	-	-
gALL_v3_c28271	5	4	-	-	-	-	-
gALL_v3_c28272	3	3	-	-	-	-	-
gALL_v3_c28274	1	3	-	-	-	-	-
gALL_v3_c28275	2	2	-	-	-	-	-
gALL_v3_c28276	2	4	-	-	-	-	-
gALL_v3_c28278	3	3	-	-	-	-	-
gALL_v3_c28279	2	2	-	-	ref|XP_003694021.1|	PREDICTED: neurobeachin-like [Apis florea]	5,00E-43
gALL_v3_c2828	6	9	-	-	dbj|BAL61212.1|	fem-1 homolog B-like protein [Locusta migratoria manilensis]	1,00E-129
gALL_v3_c28281	1	2	-	-	-	-	-
gALL_v3_c28282	3	3	-	-	gb|EFN65619.1|	Putative uncharacterized transposon-derived protein F52C9.6 [Camponotus floridanus]	8,00E-06
gALL_v3_c28283	1	6	-	-	-	-	-
gALL_v3_c28284	3	1	-	-	-	-	-
gALL_v3_c28285	2	1	-	-	-	-	-
gALL_v3_c28286	0	4	-	-	-	-	-
gALL_v3_c28287	2	3	-	-	-	-	-
gALL_v3_c28288	2	2	-	-	-	-	-
gALL_v3_c28289	1	3	-	-	-	-	-
gALL_v3_c28291	2	3	-	-	-	-	-
gALL_v3_c28292	3	1	-	-	-	-	-
gALL_v3_c28293	1	4	gnl|CDD|190807	pfam03952, Enolase_N, Enolas	dbj|BAF48677.1|	enolase [Pseudotrichonympha grassii]	1,00E-59
gALL_v3_c28294	1	2	-	-	gb|EFA06657.1|	hypothetical protein TcasGA2_TC009583 [Tribolium castaneum]	8,00E-49
gALL_v3_c28295	2	1	-	-	-	-	-
gALL_v3_c28296	2	3	-	-	-	-	-
gALL_v3_c28297	1	2	-	-	-	-	-
gALL_v3_c28298	2	1	-	-	-	-	-
gALL_v3_c28299	2	2	-	-	-	-	-
gALL_v3_c283	6	5	gnl|CDD|109297	pfam00233, PDEase_I, 3'5'-cyc	ref|XP_002432314.1|	cAMP-specific 3,5-cyclic phosphodiesterase, putative [Pediculus humanus corporis] gb|EEB19576.1| cAMP-specific 3,5-cyclic phosphodiesterase, putative [Pediculus huma	1,00E-103
gALL_v3_c2830	3	9	-	-	-	-	-

gALL_v3_c28300	2	2	-	-	-	-	-
gALL_v3_c28301	3	1	-	-	-	-	-
gALL_v3_c28302	3	1	-	-	-	-	-
gALL_v3_c28303	2	1	gnl|CDD|185480	PTZ00148, PTZ00148, 40S ribo	gb|AGM32337.1|	40S ribosomal protein S8 [Coptotermes formosanus]	2,00E-85
gALL_v3_c28304	0	3	-	-	-	-	-
gALL_v3_c28305	1	2	-	-	-	-	-
gALL_v3_c28306	0	3	-	-	ref|XP_001967512.1|	GF20702 [Drosophila ananassae] gb|EDV34046.1| GF20702 [Drosophila ananassae]	2,00E-31
gALL_v3_c28307	3	1	-	-	-	-	-
gALL_v3_c28308	4	3	-	-	ref|XP_004522830.1|	PREDICTED: multidrug resistance protein 1A-like isoform X2 [Ceratitis capitata]	2,00E-23
gALL_v3_c28309	1	6	-	-	-	-	-
gALL_v3_c28310	1	2	-	-	-	-	-
gALL_v3_c28311	1	2	-	-	-	-	-
gALL_v3_c28312	0	5	gnl|CDD|190884	pfam04146, YTH, YT521-B-like	ref|XP_003708670.1|	PREDICTED: YTH domain-containing protein 1-like, partial [Megachile rotundata]	4,00E-55
gALL_v3_c28313	1	3	-	-	-	-	-
gALL_v3_c28314	1	2	-	-	-	-	-
gALL_v3_c28315	2	2	-	-	-	-	-
gALL_v3_c28316	1	3	-	-	-	-	-
gALL_v3_c28317	0	3	-	-	-	-	-
gALL_v3_c28318	1	3	-	-	-	-	-
gALL_v3_c28319	2	1	-	-	-	-	-
gALL_v3_c2832	6	10	-	-	-	-	-
gALL_v3_c28320	0	3	-	-	-	-	-
gALL_v3_c28321	1	2	-	-	-	-	-
gALL_v3_c28322	3	3	-	-	-	-	-
gALL_v3_c28323	0	3	-	-	-	-	-
gALL_v3_c28325	2	4	-	-	gb|ENN80111.1|	hypothetical protein YQE_03470, partial [Dendroctonus ponderosae] gb|ERL87597.1| hypothetical protein D910_04988 [Dendroctonus ponderosae]	5,00E-13
gALL_v3_c28326	2	2	-	-	-	-	-
gALL_v3_c28327	3	5	-	-	-	-	-
gALL_v3_c28328	1	2	-	-	-	-	-
gALL_v3_c28329	1	3	-	-	-	-	-
gALL_v3_c28332	2	1	-	-	-	-	-
gALL_v3_c28333	3	3	-	-	gb|AGM32905.1|	hypothetical protein [Coptotermes formosanus]	8,00E-08
gALL_v3_c28334	0	3	-	-	-	-	-
gALL_v3_c28335	0	4	-	-	-	-	-
gALL_v3_c28336	2	1	-	-	-	-	-
gALL_v3_c28337	3	1	gnl|CDD|72906	cd04670, Nudix_Hydrolase_12	ref|XP_307498.3|	Anopheles gambiae str. PEST AGAP012590-PA [Anopheles gambiae str. PEST] gb|EAA03301.4| AGAP012590-PA [Anopheles gambiae str. PEST]	1,00E-21
gALL_v3_c28338	2	1	-	-	-	-	-
gALL_v3_c28339	3	2	gnl|CDD|146171	pfam03392, OS-D, Insect pher	emb|CAJ01476.1|	hypothetical protein [Locusta migratoria]	4,00E-38
gALL_v3_c2834	2	8	-	-	-	-	-
gALL_v3_c28340	0	3	-	-	-	-	-
gALL_v3_c28342	2	1	-	-	-	-	-
gALL_v3_c28345	1	3	gnl|CDD|189334	pfam00001, 7tm_1, 7 transme	emb|CAC36957.1|	tachykinin receptor like GPCR protein [Rhyparobia maderae]	2,00E-81
gALL_v3_c28346	2	1	-	-	-	-	-
gALL_v3_c28347	3	1	-	-	-	-	-
gALL_v3_c28348	0	3	-	-	-	-	-
gALL_v3_c28349	1	2	gnl|CDD|189395	pfam00110, wnt, wnt family.	gb|EFX82994.1|	putative secreted signaling factor WNT16 [Daphnia pulex]	4,00E-34
gALL_v3_c2835	6	6	-	-	-	-	-
gALL_v3_c28350	2	1	-	-	ref|XP_006567929.1|	PREDICTED: uncharacterized protein PF11_0213-like [Apis mellifera]	2,00E-08
gALL_v3_c28351	1	2	-	-	gb|EFZ13006.1|	hypothetical protein SINV_11056 [Solenopsis invicta]	1,00E-32
gALL_v3_c28352	1	2	-	-	-	-	-
gALL_v3_c28353	0	3	-	-	-	-	-
gALL_v3_c28354	0	4	-	-	-	-	-
gALL_v3_c28355	3	1	-	-	gb|AGM32900.1|	putative RNA-binding protein [Coptotermes formosanus]	1,00E-16
gALL_v3_c28357	2	4	-	-	gb|EFN67956.1|	Junctophilin-3 [Camponotus floridanus]	2,00E-39
gALL_v3_c28358	2	2	-	-	-	-	-
gALL_v3_c28359	2	4	-	-	gb|EFN81784.1|	Extracellular sulfatase SULF-1-like protein [Harpegnathos saltator]	1,00E-06
gALL_v3_c2836	6	4	-	-	gb|EFN83614.1|	RNA polymerase-associated protein Rtf1 [Harpegnathos saltator]	3,00E-27
gALL_v3_c28360	1	2	-	-	-	-	-
gALL_v3_c28361	2	4	-	-	-	-	-
gALL_v3_c28362	24	29	-	-	-	-	-
gALL_v3_c28363	3	4	-	-	-	-	-
gALL_v3_c28364	2	2	gnl|CDD|129984	TIGR00906, 2A0303, cationic a	ref|XP_003701179.1|	PREDICTED: probable cationic amino acid transporter-like [Megachile rotundata]	7,00E-58
gALL_v3_c28365	0	3	-	-	-	-	-
gALL_v3_c28367	4	4	-	-	ref|XP_001993993.1|	GH22499 [Drosophila grimshawi] gb|EDV94729.1| GH22499 [Drosophila grimshawi]	2,00E-33
gALL_v3_c28368	3	2	gnl|CDD|130820	TIGR01759, MalateDH-SF1, ma	gb|AGM32509.1|	malate dehydrogenase, partial [Coptotermes formosanus]	1,00E-126
gALL_v3_c28369	1	3	-	-	-	-	-
gALL_v3_c2837	4	11	gnl|CDD|162459	TIGR01642, U2AF_lg, U2 snRN	ref|XP_002613594.1|	hypothetical protein BRAFLDRAFT_226998 [Branchiostoma floridae] gb|EEN69603.1| hypothetical protein BRAFLDRAFT_226998 [Branchiostoma floridae]	2,00E-09
gALL_v3_c28370	1	5	-	-	-	-	-
gALL_v3_c28371	2	2	-	-	-	-	-
gALL_v3_c28372	0	3	gnl|CDD|31427	COG1234, ElaC, Metal-depend	ref|XP_968692.2|	PREDICTED: similar to AGAP009743-PA [Tribolium castaneum] gb|EFA09019.1| hypothetical protein TcasGA2_TC006728 [Tribolium castaneum]	4,00E-40
gALL_v3_c28373	2	1	gnl|CDD|180967	PRK07411, PRK07411, hypoth	gb|EFZ13226.1|	hypothetical protein SINV_06919 [Solenopsis invicta]	3,00E-41
gALL_v3_c28374	0	3	-	-	-	-	-
gALL_v3_c28375	3	4	gnl|CDD|133070	cd03858, M14_CP_N-E_like, C	ref|XP_002432672.1|	carboxypeptidase E precursor, putative [Pediculus humanus corporis] gb|EEB19934.1| carboxypeptidase E precursor, putative [Pediculus humanus corporis]	5,00E-19
gALL_v3_c28376	2	1	-	-	gb|EFN81509.1|	hypothetical protein EAI_11433 [Harpegnathos saltator]	1,00E-29
gALL_v3_c28377	1	2	-	-	-	-	-
gALL_v3_c28378	0	4	-	-	-	-	-
gALL_v3_c28379	5	2	gnl|CDD|57927	cd01856, YlqF, YlqF. Proteins c	ref|XP_003244970.1|	PREDICTED: mitochondrial GTPase 1-like [Acyrthosiphon pisum]	3,00E-90
gALL_v3_c2838	2	7	gnl|CDD|163644	cd07401, MPP_TMEM62_N, H	ref|XP_395342.4|	PREDICTED: transmembrane protein 62-like isoform X1 [Apis mellifera]	3,00E-29
gALL_v3_c28380	2	3	-	-	-	-	-
gALL_v3_c28381	3	1	-	-	-	-	-
gALL_v3_c28382	1	6	-	-	ref|XP_002429661.1|	krueppel c2h2-type zinc finger protein, putative [Pediculus humanus corporis] gb|EEB16923.1| krueppel c2h2-type zinc finger protein, putative [Pediculus humanus corpor	1,00E-111
gALL_v3_c28383	3	3	-	-	-	-	-
gALL_v3_c28384	1	2	-	-	-	-	-
gALL_v3_c28385	3	4	-	-	-	-	-
gALL_v3_c28386	0	4	gnl|CDD|192127	pfam08699, DUF1785, Domair	emb|CCD31669.1|	argonaute-1a, partial [Rhopalosiphum padi] emb|CCD31670.1| argonaute-1a, partial [Rhopalosiphum padi] emb|CCD31671.1| argonaute-1a, partial [Aphis gossypii] emb|	1,00E-62
gALL_v3_c28387	2	2	-	-	-	-	-
gALL_v3_c28389	1	2	-	-	-	-	-
gALL_v3_c2839	22	17	-	-	ref|XP_006137401.1|	PREDICTED: tigger transposable element-derived protein 1-like [Pelodiscus sinensis]	3,00E-07
gALL_v3_c28390	3	4	-	-	ref|XP_002422593.1|	smad4, putative [Pediculus humanus corporis] gb|EEB09855.1| smad4, putative [Pediculus humanus corporis]	1,00E-11
gALL_v3_c28391	2	3	-	-	dbj|BAN21654.1|	conserved hypothetical protein, partial [Riptortus pedestris]	8,00E-13
gALL_v3_c28392	1	3	-	-	-	-	-
gALL_v3_c28393	1	4	-	-	ref|XP_003465065.2|	PREDICTED: zinc finger protein 91-like [Cavia porcellus]	3,00E-14
gALL_v3_c28394	2	2	-	-	-	-	-
gALL_v3_c28395	2	3	-	-	ref|XP_004580233.1|	PREDICTED: transcription elongation regulator 1-like protein [Ochotona princeps]	1,00E-13
gALL_v3_c28396	2	1	-	-	-	-	-
gALL_v3_c28398	4	2	-	-	-	-	-
gALL_v3_c28399	3	2	-	-	-	-	-
gALL_v3_c284	2	8	-	-	-	-	-
gALL_v3_c28400	0	3	-	-	-	-	-
gALL_v3_c28402	5	3	-	-	-	-	-
gALL_v3_c28403	0	4	-	-	-	-	-
gALL_v3_c28404	3	1	-	-	-	-	-
gALL_v3_c28405	0	4	-	-	ref|XP_002426485.1|	conserved hypothetical protein [Pediculus humanus corporis] gb|EEB13747.1| conserved hypothetical protein [Pediculus humanus corporis]	2,00E-43
gALL_v3_c28406	1	5	gnl|CDD|150423	pfam09748, Med10, Transcrip	gb|ENN78401.1|	hypothetical protein YQE_05201, partial [Dendroctonus ponderosae] gb|ERL93280.1| hypothetical protein D910_10576 [Dendroctonus ponderosae]	6,00E-45
gALL_v3_c28407	0	3	-	-	-	-	-
gALL_v3_c28408	0	3	-	-	-	-	-
gALL_v3_c2841	6	3	-	-	-	-	-
gALL_v3_c28410	0	3	-	-	-	-	-
gALL_v3_c28412	2	2	-	-	-	-	-
gALL_v3_c28413	1	2	gnl|CDD|190890	pfam04157, EAP30, EAP30/Vp	gb|ABF51315.1|	ELL complex EAP30 subunit isoform 2 [Bombyx mori]	4,00E-24
gALL_v3_c28416	1	4	-	-	-	-	-
gALL_v3_c28417	3	7	-	-	ref|XP_006564377.1|	PREDICTED: titin-like [Apis mellifera]	2,00E-10
gALL_v3_c28418	2	3	-	-	ref|XP_006611306.1|	PREDICTED: dedicator of cytokinesis protein 3-like isoform X1 [Apis dorsata]	8,00E-55
gALL_v3_c28419	3	6	-	-	-	-	-
gALL_v3_c2842	6	5	-	-	-	-	-
gALL_v3_c28420	1	6	-	-	ref|XP_003492429.1|	PREDICTED: protein EFR3 homolog cmp44E-like [Bombus impatiens]	1,00E-118
gALL_v3_c28421	1	3	gnl|CDD|190340	pfam02535, Zip, ZIP Zinc trans	gb|ETN64781.1|	zinc transporter [Anopheles darlingi]	6,00E-22
gALL_v3_c28422	4	5	-	-	-	-	-
gALL_v3_c28423	5	3	-	-	-	-	-
gALL_v3_c28427	2	3	-	-	-	-	-
gALL_v3_c28428	0	3	-	-	-	-	-
gALL_v3_c28429	0	4	-	-	-	-	-
gALL_v3_c2843	4	10	-	-	-	-	-
gALL_v3_c28430	2	4	-	-	-	-	-
gALL_v3_c28431	2	2	gnl|CDD|145236	pfam01951, Archease, Archea	ref|NP_001155627.1|	archease [Acyrthosiphon pisum] dbj|BAH72625.1| ACYPI005448 [Acyrthosiphon pisum]	5,00E-23
gALL_v3_c28432	1	2	-	-	ref|XP_002431506.1|	conserved hypothetical protein [Pediculus humanus corporis] gb|EEB18768.1| conserved hypothetical protein [Pediculus humanus corporis]	2,00E-09
gALL_v3_c28434	0	4	-	-	ref|XP_006608420.1|	PREDICTED: pre-mRNA-splicing factor SYF1-like [Apis dorsata]	1,00E-26
gALL_v3_c28435	1	2	-	-	-	-	-
gALL_v3_c28436	1	3	-	-	-	-	-
gALL_v3_c28437	2	2	-	-	-	-	-
gALL_v3_c28438	0	3	-	-	-	-	-
gALL_v3_c28439	1	3	-	-	-	-	-
gALL_v3_c2844	13	8	gnl|CDD|191879	pfam07885, Ion_trans_2, Ion c	ref|XP_969244.1|	PREDICTED: similar to TWiK family of potassium channels family member (twk-46) [Tribolium castaneum]	1,00E-109
gALL_v3_c28440	0	3	-	-	-	-	-
gALL_v3_c28441	1	2	-	-	-	-	-
gALL_v3_c28442	3	2	-	-	ref|XP_002030206.1|	GM25311 [Drosophila sechellia] gb|EDW41192.1| GM25311 [Drosophila sechellia]	2,00E-08
gALL_v3_c28443	3	2	-	-	ref|XP_002423349.1|	protein outspread, putative [Pediculus humanus corporis] gb|EEB10611.1| protein outspread, putative [Pediculus humanus corporis]	5,00E-34
gALL_v3_c28444	6	3	gnl|CDD|144562	pfam01019, G_glu_transpept,	ref|XP_006621169.1|	PREDICTED: gamma-glutamyltranspeptidase 1-like isoform X3 [Apis dorsata] ref|XP_006621170.1| PREDICTED: gamma-glutamyltranspeptidase 1-like isoform X4 [Apis dors	9,00E-46
gALL_v3_c28445	1	2	gnl|CDD|73117	cd04618, CBS_pair_5, The CBS	ref|NP_001119720.1|	SNF4/AMP-activated protein kinase gamma subunit [Bombyx mori] gb|ACC62889.1| AMP-activated protein kinase gamma subunit [Bombyx mori]	6,00E-69
gALL_v3_c28446	1	3	-	-	-	-	-
gALL_v3_c28447	3	5	gnl|CDD|190590	pfam03281, Mab-21, Mab-21	ref|XP_969398.1|	PREDICTED: similar to GA11945-PA [Tribolium castaneum] gb|EFA01049.1| hypothetical protein TcasGA2_TC003965 [Tribolium castaneum]	1,00E-06
gALL_v3_c28448	1	2	-	-	-	-	-
gALL_v3_c28449	3	6	gnl|CDD|173456	PTZ00169, PTZ00169, ADP/ATI	gb|AGM32826.1|	ADP/ATP carrier protein 2, partial [Coptotermes formosanus]	7,00E-23
gALL_v3_c2845	17	25	gnl|CDD|133123	cd06592, GH31_glucosidase_I	gb|EFN84538.1|	Uncharacterized family 31 glucosidase KIAA1161 [Harpegnathos saltator]	1,00E-126

gALL_v3_c28450	2	1	-	-	-	-	-
gALL_v3_c28451	3	2	gnl|CDD|191169	pfam05028, PARG_cat, Poly (A	gb|EGI61214.1|	Poly(ADP-ribose) glycohydrolase [Acromyrmex echinatior]	9,00E-76
gALL_v3_c28452	2	2	-	-	-	-	-
gALL_v3_c28453	2	1	-	-	-	-	-
gALL_v3_c28454	1	2	-	-	-	-	-
gALL_v3_c28456	1	3	-	-	-	-	-
gALL_v3_c28458	0	3	-	-	-	-	-
gALL_v3_c28459	2	3	-	-	gb|EFN64480.1|	hypothetical protein EAG_01817 [Camponotus floridanus]	2,00E-08
gALL_v3_c2846	12	15	-	-	gb|EGI64614.1|	Protein strawberry notch [Acromyrmex echinatior]	1,00E-123
gALL_v3_c28460	2	1	-	-	-	-	-
gALL_v3_c28461	2	3	-	-	-	-	-
gALL_v3_c28462	4	6	gnl|CDD|28928	cd00268, DEADc, DEAD-box he	gb|AGM32791.1|	DEAD box ATP-dependent RNA helicase-like protein, partial [Coptotermes formosanus]	5,00E-47
gALL_v3_c28463	3	3	-	-	gb|EFN65619.1|	Putative uncharacterized transposon-derived protein F52C9.6 [Camponotus floridanus]	3,00E-07
gALL_v3_c28465	0	3	-	-	-	-	-
gALL_v3_c28466	3	2	-	-	-	-	-
gALL_v3_c28469	1	4	-	-	ref|XP_001948681.1|	PREDICTED: hypothetical protein LOC100159791 [Acyrthosiphon pisum]	3,00E-12
gALL_v3_c2847	6	11	-	-	-	-	-
gALL_v3_c28471	0	3	-	-	-	-	-
gALL_v3_c28472	2	2	-	-	-	-	-
gALL_v3_c28473	2	2	gnl|CDD|133052	cd06430, GT8_like_2, GT8_like	ref|XP_969798.1|	PREDICTED: similar to GLT8D3 protein [Tribolium castaneum]	4,00E-61
gALL_v3_c28474	2	2	-	-	-	-	-
gALL_v3_c28475	1	4	gnl|CDD|147473	pfam05303, DUF727, Protein c	gb|EFN83206.1|	UPF0279 protein C14orf129-like protein [Harpegnathos saltator]	1,00E-35
gALL_v3_c28476	2	3	-	-	ref|XP_001947608.2|	PREDICTED: peroxisomal multifunctional enzyme type 2-like isoform 1 [Acyrthosiphon pisum]	4,00E-11
gALL_v3_c28477	0	3	-	-	-	-	-
gALL_v3_c28478	6	12	gnl|CDD|165238	PHA02927, PHA02927, secrete	ref|XP_971920.2|	PREDICTED: similar to AGAP007761-PA [Tribolium castaneum]	0
gALL_v3_c28479	1	4	-	-	-	-	-
gALL_v3_c2848	26	34	gnl|CDD|29012	cd00112, LDLa, Low Density Li	gb|EFX73914.1|	hypothetical protein DAPPUDRAFT_307561 [Daphnia pulex]	0
gALL_v3_c28480	1	5	-	-	ref|XP_966659.1|	PREDICTED: similar to TATA-binding protein-associated factor 172 [Tribolium castaneum] gb|EFA08205.1| hypothetical protein TcasGA2_TC005832 [Tribolium castaneum]	3,00E-49
gALL_v3_c28481	1	2	-	-	-	-	-
gALL_v3_c28482	1	2	-	-	-	-	-
gALL_v3_c28483	8	15	-	-	-	-	-
gALL_v3_c28484	3	2	-	-	sp|Q964R0.1|CP6K1_BL	RecName: Full=Cytochrome P450 6k1; AltName: Full=CYPVIK1 gb|AAK57914.1|AF281328_1 cytochrome P450 CYP6K1 [Blattella germanica]	1,00E-16
gALL_v3_c28485	2	1	-	-	-	-	-
gALL_v3_c28486	1	2	-	-	-	-	-
gALL_v3_c28487	3	2	-	-	-	-	-
gALL_v3_c28488	0	4	gnl|CDD|190983	pfam04427, Brix, Brix domain.	ref|XP_974431.1|	PREDICTED: similar to peter pan CG5786-PA [Tribolium castaneum] gb|EEZ98288.1| hypothetical protein TcasGA2_TC000737 [Tribolium castaneum]	2,00E-46
gALL_v3_c28489	2	2	-	-	gb|EFN61169.1|	Uncharacterized protein KIAA1797 [Camponotus floridanus]	1,00E-24
gALL_v3_c28490	1	2	-	-	-	-	-
gALL_v3_c28491	0	3	-	-	-	-	-
gALL_v3_c28492	1	5	-	-	-	-	-
gALL_v3_c28493	0	4	-	-	-	-	-
gALL_v3_c28495	0	4	-	-	-	-	-
gALL_v3_c28498	3	4	gnl|CDD|192560	pfam10377, ATG11, Autophag	ref|XP_002429512.1|	protein NUF1, putative [Pediculus humanus corporis] gb|EEB16774.1| protein NUF1, putative [Pediculus humanus corporis]	4,00E-48
gALL_v3_c28499	1	2	-	-	-	-	-
gALL_v3_c285	17	18	-	-	-	-	-
gALL_v3_c2850	11	9	-	-	gb|EFN75184.1|	Epidermal growth factor receptor [Harpegnathos saltator]	6,00E-57
gALL_v3_c28501	2	2	-	-	-	-	-
gALL_v3_c28502	4	6	-	-	-	-	-
gALL_v3_c28503	2	2	-	-	ref|XP_002426163.1|	conserved hypothetical protein [Pediculus humanus corporis] gb|EEB13425.1| conserved hypothetical protein [Pediculus humanus corporis]	1,00E-24
gALL_v3_c28504	0	5	-	-	-	-	-
gALL_v3_c28505	1	6	-	-	ref|XP_003730218.1|	PREDICTED: uncharacterized protein LOC100889850 [Strongylocentrotus purpuratus]	2,00E-08
gALL_v3_c28506	0	3	-	-	-	-	-
gALL_v3_c28507	2	1	-	-	-	-	-
gALL_v3_c28508	2	2	-	-	gb|EFN71576.1|	Tankyrase-1 [Camponotus floridanus]	2,00E-12
gALL_v3_c28509	2	1	-	-	-	-	-
gALL_v3_c2851	5	5	-	-	-	-	-
gALL_v3_c28510	2	1	-	-	-	-	-
gALL_v3_c28511	6	4	-	-	gb|EFN63197.1|	Autophagy-related protein 2-like protein B [Camponotus floridanus]	2,00E-48
gALL_v3_c28512	1	2	-	-	-	-	-
gALL_v3_c28513	2	3	-	-	-	-	-
gALL_v3_c28515	2	2	gnl|CDD|189452	pfam00211, Guanylate_cyc, Ad	ref|XP_001948915.2|	PREDICTED: adenylate cyclase type 2-like [Acyrthosiphon pisum]	4,00E-57
gALL_v3_c28516	2	3	-	-	-	-	-
gALL_v3_c28517	2	1	-	-	-	-	-
gALL_v3_c28518	0	3	-	-	-	-	-
gALL_v3_c28519	1	3	-	-	ref|XP_002427400.1|	hypothetical protein Phum_PHUM317340 [Pediculus humanus corporis] gb|EEB14662.1| hypothetical protein Phum_PHUM317340 [Pediculus humanus corporis]	4,00E-21
gALL_v3_c2852	10	16	gnl|CDD|28897	cd00013, ADF, Actin depolyme	ref|XP_002430745.1|	protein kinase C and casein kinase substrate in neurons protein, putative [Pediculus humanus corporis] gb|EEB18007.1| protein kinase C and casein kinase substrate in neu	2,00E-79
gALL_v3_c28520	1	2	-	-	-	-	-
gALL_v3_c28521	3	5	-	-	-	-	-
gALL_v3_c28522	2	2	-	-	-	-	-
gALL_v3_c28523	2	4	gnl|CDD|115470	pfam06814, Lung_7-TM_R, Lu	ref|XP_001846376.1|	transmembrane protein 87A [Culex quinquefasciatus] gb|EDS43466.1| transmembrane protein 87A [Culex quinquefasciatus]	1,00E-126
gALL_v3_c28524	2	1	-	-	gb|EFZ21994.1|	hypothetical protein SINV_12711 [Solenopsis invicta]	4,00E-11
gALL_v3_c28525	3	1	-	-	ref|XP_001812682.1|	PREDICTED: similar to forked CG5424-PB [Tribolium castaneum]	3,00E-10
gALL_v3_c28526	1	2	-	-	-	-	-
gALL_v3_c28527	3	1	-	-	-	-	-
gALL_v3_c28530	2	2	-	-	-	-	-
gALL_v3_c28531	3	1	-	-	-	-	-
gALL_v3_c28532	2	1	-	-	-	-	-
gALL_v3_c28533	1	2	-	-	-	-	-
gALL_v3_c28534	4	2	-	-	-	-	-
gALL_v3_c28535	0	3	-	-	-	-	-
gALL_v3_c28536	3	4	gnl|CDD|189654	pfam00651, BTB, BTB/POZ dor	ref|XP_001809480.1|	PREDICTED: similar to AGAP000233-PA [Tribolium castaneum]	1,00E-79
gALL_v3_c28537	1	4	-	-	-	-	-
gALL_v3_c28538	2	2	gnl|CDD|145919	pfam03028, Dynein_heavy, Dy	ref|XP_001859558.1|	dynein beta chain [Culex quinquefasciatus] gb|EDS35091.1| dynein beta chain [Culex quinquefasciatus]	1,00E-89
gALL_v3_c28539	1	3	gnl|CDD|192376	pfam09783, Vac_ImportDeg, \	ref|XP_003699689.1|	PREDICTED: uncharacterized protein C17orf39-like [Megachile rotundata]	5,00E-72
gALL_v3_c2854	8	5	-	-	-	-	-
gALL_v3_c28540	0	3	-	-	-	-	-
gALL_v3_c28541	1	2	-	-	-	-	-
gALL_v3_c28542	2	2	-	-	-	-	-
gALL_v3_c28543	1	2	-	-	ref|XP_001330228.1|	TPR Domain containing protein [Trichomonas vaginalis G3] gb|EAY01380.1| TPR Domain containing protein [Trichomonas vaginalis G3]	1,00E-37
gALL_v3_c28544	3	3	-	-	-	-	-
gALL_v3_c28545	1	2	-	-	-	-	-
gALL_v3_c28546	2	4	-	-	ref|WP_005920083.1|	hypothetical protein [Faecalibacterium prausnitzii] gb|EDP22260.1| hypothetical protein FAEPRAM212_00883 [Faecalibacterium prausnitzii M21/2] gb|EDP22760.1| hypo	3,00E-38
gALL_v3_c28547	1	7	gnl|CDD|133004	cd02510, pp-GalNAc-T, pp-Gal	ref|XP_973543.1|	PREDICTED: similar to polypeptide GalNAc transferase 5 CG31651-PA [Tribolium castaneum] gb|EFA08271.1| hypothetical protein TcasGA2_TC005902 [Tribolium castaneur	1,00E-138
gALL_v3_c28548	2	1	-	-	-	-	-
gALL_v3_c28549	0	4	-	-	ref|XP_002429883.1|	zinc finger protein, putative [Pediculus humanus corporis] gb|EEB17145.1| zinc finger protein, putative [Pediculus humanus corporis]	2,00E-21
gALL_v3_c2855	19	27	-	-	gb|EFN75703.1|	Zinc finger protein 706 [Harpegnathos saltator]	1,00E-16
gALL_v3_c28550	3	2	-	-	-	-	-
gALL_v3_c28551	3	2	-	-	ref|XP_004614950.1|	PREDICTED: zinc finger protein 91-like [Sorex araneus]	2,00E-08
gALL_v3_c28552	0	3	-	-	-	-	-
gALL_v3_c28553	1	4	-	-	-	-	-
gALL_v3_c28554	1	2	-	-	-	-	-
gALL_v3_c28556	4	2	-	-	-	-	-
gALL_v3_c28557	1	2	gnl|CDD|188751	cd09365, LIM2_LIMK, The seco	gb|EFN66335.1|	LIM domain kinase 1 [Camponotus floridanus]	2,00E-91
gALL_v3_c28558	3	5	gnl|CDD|179778	PRK04195, PRK04195, replicat	ref|XP_001653061.1|	replication factor C large subunit, putative [Aedes aegypti] gb|EAT47550.1| AAEL001324-PA [Aedes aegypti]	5,00E-61
gALL_v3_c28559	4	2	-	-	-	-	-
gALL_v3_c28561	0	3	-	-	-	-	-
gALL_v3_c28562	3	1	-	-	-	-	-
gALL_v3_c28563	1	4	-	-	-	-	-
gALL_v3_c28564	0	4	-	-	-	-	-
gALL_v3_c28565	5	6	-	-	-	-	-
gALL_v3_c28566	1	5	-	-	-	-	-
gALL_v3_c28567	2	1	-	-	-	-	-
gALL_v3_c28568	1	3	-	-	-	-	-
gALL_v3_c28569	0	4	-	-	-	-	-
gALL_v3_c2857	3	4	-	-	gb|EHJ68530.1|	hypothetical protein KGM_00687 [Danaus plexippus]	7,00E-07
gALL_v3_c28570	1	2	-	-	-	-	-
gALL_v3_c28571	1	2	-	-	-	-	-
gALL_v3_c28572	1	2	-	-	-	-	-
gALL_v3_c28574	1	6	-	-	ref|XP_001606223.2|	PREDICTED: hypothetical protein LOC100122612 [Nasonia vitripennis]	2,00E-20
gALL_v3_c28575	1	2	-	-	-	-	-
gALL_v3_c28576	3	1	-	-	-	-	-
gALL_v3_c28577	5	5	gnl|CDD|153012	pfam12578, 3-PAP, Myotubula	ref|XP_001603556.2|	PREDICTED: myotubularin-related protein 10-like [Nasonia vitripennis]	2,00E-75
gALL_v3_c28578	1	3	-	-	-	-	-
gALL_v3_c2858	12	8	gnl|CDD|28968	cd01390, HMGB-UBF_HMG-bo	gb|EFN72639.1|	SWI/SNF-related matrix-associated actin-dependent regulator chromatin subfamily E member 1 [Camponotus floridanus]	1,00E-116
gALL_v3_c28580	0	3	-	-	-	-	-
gALL_v3_c28581	2	1	-	-	-	-	-
gALL_v3_c28582	2	4	-	-	gb|ADF18552.1|	endonuclease-reverse transcriptase [Bombyx mori]	5,00E-08
gALL_v3_c28583	0	4	-	-	ref|XP_001507978.2|	PREDICTED: tetratricopeptide repeat protein 26-like, partial [Ornithorhynchus anatinus]	4,00E-72
gALL_v3_c28585	2	3	gnl|CDD|29261	cd00204, ANK, ankyrin repeats	gb|AGM32371.1|	ankyrin repeat protein [Coptotermes formosanus]	4,00E-48
gALL_v3_c28586	3	1	-	-	-	-	-
gALL_v3_c28588	2	1	-	-	-	-	-
gALL_v3_c28589	2	5	-	-	gb|EFN67634.1|	Putative uncharacterized transposon-derived protein F52C9.6 [Camponotus floridanus]	9,00E-20
gALL_v3_c28590	2	2	-	-	-	-	-
gALL_v3_c28591	3	1	-	-	ref|XP_004369475.1|	PREDICTED: KAT8 regulatory NSL complex subunit 3 [Trichechus manatus latirostris]	2,00E-16
gALL_v3_c28592	0	3	-	-	-	-	-
gALL_v3_c28595	3	1	-	-	-	-	-
gALL_v3_c28596	3	8	-	-	-	-	-
gALL_v3_c28597	3	2	-	-	-	-	-
gALL_v3_c28598	1	5	-	-	-	-	-
gALL_v3_c28599	1	2	-	-	-	-	-
gALL_v3_c286	21	34	gnl|CDD|190616	pfam03370, CBM_21, Putative	ref|XP_003705716.1|	PREDICTED: uncharacterized protein LOC100878232 [Megachile rotundata]	2,00E-89
gALL_v3_c2860	11	12	gnl|CDD|185530	PTZ00258, PTZ00258, GTP-bin	ref|NP_001153863.1|	Obg-like ATPase 1 [Acyrthosiphon pisum] dbj|BAH70819.1| ACYPI52009 [Acyrthosiphon pisum] dbj|BAH70820.1| ACYPI52009 [Acyrthosiphon pisum] dbj|BAH70821.1| A(	0
gALL_v3_c28600	1	2	-	-	-	-	-
gALL_v3_c28601	2	2	-	-	-	-	-

gALL_v3_c28602	1	3	-	-	-	-	-
gALL_v3_c28603	3	1	-	-	-	-	-
gALL_v3_c28604	0	3	-	-	-	-	-
gALL_v3_c28605	2	3	-	-	-	-	-
gALL_v3_c28606	2	3	-	-	-	-	-
gALL_v3_c28607	2	2	-	-	ref|XP_003393928.1|	PREDICTED: protein lines-like [Bombus terrestris] ref|XP_003489763.1| PREDICTED: protein lines-like [Bombus impatiens]	1,00E-34
gALL_v3_c28608	3	2	-	-	ref|XP_002427503.1|	conserved hypothetical protein [Pediculus humanus corporis] gb|EEB14765.1| conserved hypothetical protein [Pediculus humanus corporis]	1,00E-31
gALL_v3_c28609	0	3	-	-	-	-	-
gALL_v3_c2861	6	13	-	-	-	-	-
gALL_v3_c28610	0	3	-	-	-	-	-
gALL_v3_c28611	2	2	-	-	ref|XP_002432336.1|	hypothetical protein Phum_PHUM580870 [Pediculus humanus corporis] gb|EEB19598.1| hypothetical protein Phum_PHUM580870 [Pediculus humanus corporis]	4,00E-24
gALL_v3_c28612	2	2	gnl|CDD|189411	pfam00135, COesterase, Carb	ref|XP_002425796.1|	conserved hypothetical protein [Pediculus humanus corporis] gb|EEB13058.1| conserved hypothetical protein [Pediculus humanus corporis]	3,00E-78
gALL_v3_c28614	2	2	-	-	-	-	-
gALL_v3_c28616	1	5	-	-	-	-	-
gALL_v3_c28617	1	3	-	-	-	-	-
gALL_v3_c28618	2	2	gnl|CDD|185479	PTZ00146, PTZ00146, fibrillari	ref|XP_968351.1|	PREDICTED: similar to Fibrillarin CG9888-PA [Tribolium castaneum] gb|EEZ99789.1| hypothetical protein TcasGA2_TC002569 [Tribolium castaneum]	1,00E-112
gALL_v3_c28619	2	2	gnl|CDD|162101	TIGR00911, 2A0308, L-type an	ref|XP_973463.1|	PREDICTED: similar to cationic amino acid transporter [Tribolium castaneum] gb|EFA03221.1| hypothetical protein TcasGA2_TC013142 [Tribolium castaneum]	1,00E-49
gALL_v3_c2862	14	13	-	-	-	-	-
gALL_v3_c28620	1	2	-	-	-	-	-
gALL_v3_c28621	1	3	-	-	-	-	-
gALL_v3_c28622	2	1	-	-	-	-	-
gALL_v3_c28623	3	3	gnl|CDD|190554	pfam03178, CPSF_A, CPSF A su	ref|XP_003490124.1|	PREDICTED: cleavage and polyadenylation specificity factor subunit 1-like [Bombus impatiens]	8,00E-70
gALL_v3_c28624	0	3	-	-	-	-	-
gALL_v3_c28625	1	3	gnl|CDD|145882	pfam02958, DUF227, Domain	ref|XP_001847798.1|	conserved hypothetical protein [Culex quinquefasciatus] gb|EDS26961.1| conserved hypothetical protein [Culex quinquefasciatus]	1,00E-23
gALL_v3_c28626	2	1	-	-	-	-	-
gALL_v3_c28627	2	2	-	-	-	-	-
gALL_v3_c28628	0	6	-	-	-	-	-
gALL_v3_c28629	6	5	-	-	-	-	-
gALL_v3_c2863	3	8	-	-	ref|XP_003426811.1|	PREDICTED: guanylate cyclase 32E-like isoform 2 [Nasonia vitripennis] ref|XP_001600593.2| PREDICTED: guanylate cyclase 32E-like isoform 1 [Nasonia vitripennis]	1,00E-06
gALL_v3_c28630	0	4	-	-	-	-	-
gALL_v3_c28631	1	3	gnl|CDD|146566	pfam03998, Utp11, Utp11 pro	ref|XP_003393839.1|	PREDICTED: probable U3 small nucleolar RNA-associated protein 11-like [Bombus terrestris]	3,00E-44
gALL_v3_c28632	2	2	-	-	-	-	-
gALL_v3_c28633	1	1	-	-	-	-	-
gALL_v3_c28634	3	1	-	-	-	-	-
gALL_v3_c28635	2	3	-	-	ref|XP_003742325.1|	PREDICTED: uncharacterized protein LOC100904897 [Metaseiulus occidentalis]	4,00E-42
gALL_v3_c28636	1	5	-	-	ref|XP_971508.2|	PREDICTED: similar to MYC binding protein 2 [Tribolium castaneum]	3,00E-72
gALL_v3_c28637	2	2	-	-	-	-	-
gALL_v3_c28638	3	3	-	-	-	-	-
gALL_v3_c28639	2	2	-	-	ref|XP_001946812.2|	PREDICTED: tubulin polyglutamylase TTLL4-like [Acyrthosiphon pisum]	5,00E-34
gALL_v3_c2864	7	4	gnl|CDD|147250	pfam04979, IPP-2, Protein pho	gb|EFA08390.1|	hypothetical protein TcasGA2_TC006037 [Tribolium castaneum]	2,00E-35
gALL_v3_c28640	2	1	-	-	ref|XP_001580803.1|	hypothetical protein [Trichomonas vaginalis G3] gb|EAY19817.1| hypothetical protein TVAG_178610 [Trichomonas vaginalis G3]	3,00E-43
gALL_v3_c28642	2	1	gnl|CDD|190050	pfam01602, Adaptin_N, Adapt	gb|ACY66380.1|	adaptor-related protein complex 2 beta 1 subunit [Scylla paramamosain]	3,00E-62
gALL_v3_c28643	2	1	-	-	-	-	-
gALL_v3_c28644	2	4	gnl|CDD|150476	pfam09812, MRP-L28, Mitocho	gb|ETN58122.1|	39S ribosomal protein L40, mitochondrial [Anopheles darlingi]	3,00E-45
gALL_v3_c28645	3	3	-	-	-	-	-
gALL_v3_c28646	0	4	gnl|CDD|128981	smart00742, Hr1, Rho effector	gb|ERL95280.1|	hypothetical protein D910_12546 [Dendroctonus ponderosae]	3,00E-18
gALL_v3_c28647	0	3	-	-	-	-	-
gALL_v3_c28648	2	2	-	-	ref|XP_002430541.1|	UDP-glucose:glycoprotein glucosyltransferase 2 precursor, putative [Pediculus humanus corporis] gb|EEB17803.1| UDP-glucose:glycoprotein glucosyltransferase 2 precursc	1,00E-41
gALL_v3_c28649	0	3	-	-	-	-	-
gALL_v3_c2865	15	9	-	-	ref|XP_623914.1|	PREDICTED: uncharacterized protein LOC551516 [Apis mellifera] ref|XP_006619187.1| PREDICTED: uncharacterized protein LOC102675414 [Apis dorsata]	1,00E-84
gALL_v3_c28650	0	3	-	-	-	-	-
gALL_v3_c28652	1	2	-	-	-	-	-
gALL_v3_c28653	0	4	-	-	-	-	-
gALL_v3_c28654	3	4	-	-	-	-	-
gALL_v3_c28655	0	3	-	-	-	-	-
gALL_v3_c28656	1	2	-	-	-	-	-
gALL_v3_c28657	5	2	gnl|CDD|185484	PTZ00155, PTZ00155, 40S ribo	gb|AEE62422.1|	unknown [Dendroctonus ponderosae]	5,00E-66
gALL_v3_c28658	1	3	gnl|CDD|192012	pfam08355, EF_assoc_1, EF ha	gb|EFA10155.1|	hypothetical protein TcasGA2_TC012343 [Tribolium castaneum]	3,00E-50
gALL_v3_c28659	3	1	-	-	-	-	-
gALL_v3_c2866	12	9	-	-	ref|XP_003694721.1|	PREDICTED: AP-1 complex subunit sigma-2-like isoform 1 [Apis florea] ref|XP_003705005.1| PREDICTED: AP-1 complex subunit sigma-2-like isoform 2 [Megachile rotundat	2,00E-21
gALL_v3_c28660	2	1	-	-	-	-	-
gALL_v3_c28661	0	3	-	-	-	-	-
gALL_v3_c28662	1	2	-	-	-	-	-
gALL_v3_c28663	1	6	gnl|CDD|132772	cd06862, PX_SNX9_18_like, Th	ref|XP_003699015.1|	PREDICTED: sorting nexin-9-like [Apis florea]	1,00E-128
gALL_v3_c28665	2	2	gnl|CDD|152592	pfam12157, DUF3591, Protein	gb|EEZ99738.1|	hypothetical protein TcasGA2_TC002507 [Tribolium castaneum]	3,00E-69
gALL_v3_c28666	3	3	-	-	ref|XP_002593623.1|	hypothetical protein BRAFLDRAFT_61218 [Branchiostoma floridae] gb|EEN49634.1| hypothetical protein BRAFLDRAFT_61218 [Branchiostoma floridae]	2,00E-07
gALL_v3_c28667	3	1	-	-	-	-	-
gALL_v3_c28668	3	1	-	-	-	-	-
gALL_v3_c28669	4	2	-	-	-	-	-
gALL_v3_c28670	3	5	-	-	-	-	-
gALL_v3_c28671	1	4	-	-	-	-	-
gALL_v3_c28672	5	9	-	-	ref|XP_002073687.1|	GK14239 [Drosophila willistoni] gb|EDW84673.1| GK14239 [Drosophila willistoni]	1,00E-23
gALL_v3_c28673	4	4	-	-	ref|XP_006622427.1|	PREDICTED: transforming growth factor-beta-induced protein ig-h3-like isoform X1 [Apis dorsata] ref|XP_006622428.1| PREDICTED: transforming growth factor-beta-induc	8,00E-11
gALL_v3_c28674	3	1	-	-	-	-	-
gALL_v3_c28676	1	3	-	-	-	-	-
gALL_v3_c28677	1	2	-	-	-	-	-
gALL_v3_c28678	2	3	-	-	-	-	-
gALL_v3_c28679	2	1	-	-	-	-	-
gALL_v3_c28680	0	3	-	-	-	-	-
gALL_v3_c28682	7	4	gnl|CDD|133036	cd04193, UDPGlcNAc_PPase, U	gb|AGN56418.1|	UDP N-acetylglucosamine pyrophosphorylases 1 [Locusta migratoria]	2,00E-82
gALL_v3_c28683	3	1	-	-	-	-	-
gALL_v3_c28684	1	2	-	-	-	-	-
gALL_v3_c28685	0	4	-	-	-	-	-
gALL_v3_c28686	2	1	-	-	-	-	-
gALL_v3_c28687	3	3	-	-	-	-	-
gALL_v3_c28688	0	3	-	-	-	-	-
gALL_v3_c28689	1	2	gnl|CDD|190873	pfam04118, Dopey_N, Dopey,	ref|XP_001943256.2|	PREDICTED: protein dopey-1 homolog isoform 1 [Acyrthosiphon pisum]	4,00E-42
gALL_v3_c2869	4	5	-	-	-	-	-
gALL_v3_c28690	2	1	-	-	ref|XP_006134159.1|	PREDICTED: histone-lysine N-methyltransferase SETMAR-like [Pelodiscus sinensis]	3,00E-11
gALL_v3_c28691	3	3	-	-	gb|EFA13462.1|	hypothetical protein TcasGA2_TC005062 [Tribolium castaneum]	6,00E-20
gALL_v3_c28692	1	3	-	-	ref|XP_002431171.1|	conserved hypothetical protein [Pediculus humanus corporis] gb|EEB18433.1| conserved hypothetical protein [Pediculus humanus corporis]	5,00E-50
gALL_v3_c28693	1	3	-	-	-	-	-
gALL_v3_c28694	2	1	gnl|CDD|28913	cd00031, CA, Cadherin repeat	ref|XP_002427847.1|	protocadherin-16 precursor, putative [Pediculus humanus corporis] gb|EEB15109.1| protocadherin-16 precursor, putative [Pediculus humanus corporis]	1,00E-56
gALL_v3_c28695	3	4	-	-	-	-	-
gALL_v3_c28697	1	2	-	-	-	-	-
gALL_v3_c28698	3	2	-	-	-	-	-
gALL_v3_c28699	3	2	-	-	-	-	-
gALL_v3_c287	5	4	-	-	ref|XP_002426827.1|	axoneme-associated protein mst101, putative [Pediculus humanus corporis] gb|EEB14089.1| axoneme-associated protein mst101, putative [Pediculus humanus corporis]	3,00E-15
gALL_v3_c2870	15	9	gnl|CDD|109126	pfam00057, Ldl_recept_a, Low	ref|XP_002429721.1|	low-density lipoprotein receptor, putative [Pediculus humanus corporis] gb|EEB16983.1| low-density lipoprotein receptor, putative [Pediculus humanus corporis]	0
gALL_v3_c28700	1	2	-	-	-	-	-
gALL_v3_c28701	5	3	gnl|CDD|189372	pfam00067, p450, Cytochrome	gb|AGM32645.1|	cytochrome P450, partial [Coptotermes formosanus]	2,00E-53
gALL_v3_c28702	4	2	gnl|CDD|193256	pfam12780, AAA_8, P-loop co	ref|XP_001320325.1|	Dynein heavy chain family protein [Trichomonas vaginalis G3] gb|EAY08102.1| Dynein heavy chain family protein [Trichomonas vaginalis G3]	1,00E-80
gALL_v3_c28704	1	3	-	-	-	-	-
gALL_v3_c28705	2	4	-	-	-	-	-
gALL_v3_c28706	1	4	-	-	-	-	-
gALL_v3_c28707	1	2	-	-	-	-	-
gALL_v3_c28708	0	3	-	-	ref|XP_003490706.1|	PREDICTED: hypothetical protein LOC100747609 [Bombus impatiens]	2,00E-19
gALL_v3_c28709	0	4	-	-	gb|EFN63692.1|	Putative uncharacterized protein FLJ37770 [Camponotus floridanus]	4,00E-23
gALL_v3_c2871	2	7	-	-	-	-	-
gALL_v3_c28712	1	2	-	-	-	-	-
gALL_v3_c28713	1	3	-	-	-	-	-
gALL_v3_c28714	2	4	-	-	-	-	-
gALL_v3_c28715	2	1	-	-	ref|XP_001326179.1|	hypothetical protein [Trichomonas vaginalis G3] gb|EAY13956.1| hypothetical protein TVAG_490940 [Trichomonas vaginalis G3]	5,00E-09
gALL_v3_c28716	0	6	-	-	-	-	-
gALL_v3_c28717	0	4	-	-	-	-	-
gALL_v3_c28718	2	1	-	-	-	-	-
gALL_v3_c2872	5	5	-	-	-	-	-
gALL_v3_c28720	2	3	-	-	-	-	-
gALL_v3_c28722	1	2	-	-	-	-	-
gALL_v3_c28723	0	5	-	-	-	-	-
gALL_v3_c28724	0	3	-	-	-	-	-
gALL_v3_c28725	4	2	-	-	ref|XP_001951020.2|	PREDICTED: protein RRP5 homolog [Acyrthosiphon pisum]	1,00E-73
gALL_v3_c28726	2	1	-	-	-	-	-
gALL_v3_c28727	1	2	-	-	-	-	-
gALL_v3_c28728	1	4	-	-	ref|XP_004929187.1|	PREDICTED: HEAT repeat-containing protein 1-like [Bombyx mori]	1,00E-44
gALL_v3_c28729	2	2	-	-	-	-	-
gALL_v3_c2873	8	12	gnl|CDD|128514	smart00218, ZU5, Domain pre	ref|XP_002432671.1|	ankyrin-1, putative [Pediculus humanus corporis] gb|EEB19933.1| ankyrin-1, putative [Pediculus humanus corporis]	1,00E-158
gALL_v3_c28730	2	4	-	-	ref|XP_001807591.1|	PREDICTED: similar to CG2938 CG2938-PB [Tribolium castaneum]	2,00E-15
gALL_v3_c28731	3	2	-	-	-	-	-
gALL_v3_c28733	2	1	-	-	-	-	-
gALL_v3_c28734	6	4	gnl|CDD|163712	cd08156, catalase_clade_3, Cl	gb|AFM73630.1|	catalase, partial [Bicyclus anynana]	1,00E-43
gALL_v3_c28735	2	2	-	-	-	-	-
gALL_v3_c28736	2	1	gnl|CDD|192508	pfam10274, ParcG, Parkin co-r	gb|AGM32422.1|	parkin co-regulated protein-like protein [Coptotermes formosanus]	7,00E-74
gALL_v3_c28738	0	4	-	-	-	-	-
gALL_v3_c28739	7	5	-	-	-	-	-
gALL_v3_c2874	5	9	-	-	-	-	-
gALL_v3_c28740	2	1	-	-	-	-	-
gALL_v3_c28742	2	2	-	-	-	-	-
gALL_v3_c28743	2	1	-	-	-	-	-
gALL_v3_c28744	3	1	-	-	-	-	-
gALL_v3_c28745	2	5	-	-	-	-	-
gALL_v3_c28747	3	3	-	-	ref|XP_004930111.1|	PREDICTED: microtubule-associated protein futsch-like [Bombyx mori]	3,00E-19
gALL_v3_c28748	2	5	gnl|CDD|173623	cd00180, PKc, Catalytic domai	ref|XP_001604278.2|	PREDICTED: eukaryotic translation initiation factor 2-alpha kinase-like [Nasonia vitripennis]	9,00E-67
gALL_v3_c28749	1	3	-	-	-	-	-

gALL_v3_c28750	1	2	-	-	-	-	-
gALL_v3_c28751	1	2	-	-	-	-	-
gALL_v3_c28752	2	1	-	-	-	-	-
gALL_v3_c28753	6	15	gnl|CDD|48119	cd03192, GST_C_Sigma_like,	gb|AEV23881.1|	glutathione S transferase class sigma [Blattella germanica]	1,00E-104
gALL_v3_c28754	2	1	-	-	-	-	-
gALL_v3_c28756	0	3	-	-	-	-	-
gALL_v3_c28757	2	2	-	-	-	-	-
gALL_v3_c28759	0	4	-	-	ref|XP_003242319.1|	PREDICTED: histone-lysine N-methyltransferase SETMAR-like [Acyrthosiphon pisum]	2,00E-15
gALL_v3_c2876	11	22	-	-	-	-	-
gALL_v3_c28760	0	3	-	-	-	-	-
gALL_v3_c28761	3	4	-	-	gb|EHJ76831.1|	hypothetical protein KGM_17362 [Danaus plexippus]	2,00E-15
gALL_v3_c28762	0	3	-	-	-	-	-
gALL_v3_c28763	2	1	-	-	-	-	-
gALL_v3_c28764	2	1	-	-	-	-	-
gALL_v3_c28765	1	2	-	-	-	-	-
gALL_v3_c28766	0	4	-	-	-	-	-
gALL_v3_c28767	0	3	-	-	-	-	-
gALL_v3_c28768	3	6	-	-	-	-	-
gALL_v3_c28769	2	1	-	-	-	-	-
gALL_v3_c2877	2	6	-	-	-	-	-
gALL_v3_c28770	5	5	-	-	-	-	-
gALL_v3_c28771	1	2	-	-	-	-	-
gALL_v3_c28772	1	2	-	-	-	-	-
gALL_v3_c28773	1	2	-	-	ref|XP_002432447.1|	homeobox protein arx, putative [Pediculus humanus corporis] gb|EEB19709.1| homeobox protein arx, putative [Pediculus humanus corporis]	8,00E-12
gALL_v3_c28774	2	3	-	-	-	-	-
gALL_v3_c28775	1	5	gnl|CDD|28913	cd00031, CA, Cadherin repeat	ref|XP_003703403.1|	PREDICTED: fat-like cadherin-related tumor suppressor homolog [Megachile rotundata]	2,00E-78
gALL_v3_c28776	2	3	-	-	ref|XP_001848911.1|	titin [Culex quinquefasciatus] gb|EDS29254.1| titin [Culex quinquefasciatus]	3,00E-19
gALL_v3_c28777	2	3	-	-	-	-	-
gALL_v3_c28778	4	3	-	-	-	-	-
gALL_v3_c28779	0	3	-	-	ref|XP_002433157.1|	hypothetical protein Phum_PHUM615940 [Pediculus humanus corporis] gb|EEB20419.1| hypothetical protein Phum_PHUM615940 [Pediculus humanus corporis]	1,00E-10
gALL_v3_c2878	8	7	gnl|CDD|178543	PLN02957, PLN02957, copper,	ref|NP_001191151.1|	copper chaperone for superoxide dismutase [Danio rerio]	1,00E-111
gALL_v3_c28780	8	6	gnl|CDD|178019	PLN02397, PLN02397, asparta	gb|AFZ78869.1|	aspartate aminotransferase [Coptotermes formosanus]	9,00E-97
gALL_v3_c28781	2	1	-	-	-	-	-
gALL_v3_c28782	2	1	-	-	-	-	-
gALL_v3_c28783	2	2	-	-	-	-	-
gALL_v3_c28784	0	3	-	-	-	-	-
gALL_v3_c28785	3	6	-	-	-	-	-
gALL_v3_c28786	2	3	-	-	-	-	-
gALL_v3_c28787	2	1	-	-	-	-	-
gALL_v3_c28788	2	1	-	-	-	-	-
gALL_v3_c2879	5	7	-	-	ref|XP_002431242.1|	hypothetical protein Phum_PHUM516380 [Pediculus humanus corporis] gb|EEB18504.1| hypothetical protein Phum_PHUM516380 [Pediculus humanus corporis]	1,00E-06
gALL_v3_c28790	2	3	-	-	-	-	-
gALL_v3_c28791	1	2	-	-	-	-	-
gALL_v3_c28792	1	3	-	-	-	-	-
gALL_v3_c28793	2	2	-	-	-	-	-
gALL_v3_c28794	2	8	-	-	-	-	-
gALL_v3_c28795	2	2	-	-	-	-	-
gALL_v3_c28796	2	1	-	-	-	-	-
gALL_v3_c28797	0	4	-	-	-	-	-
gALL_v3_c28798	3	1	gnl|CDD|73156	cd01650, RT_nLTR_like, RT_nL	gb|ADI61823.1|	endonuclease-reverse transcriptase [Bombyx mori]	2,00E-12
gALL_v3_c28799	9	8	-	-	-	-	-
gALL_v3_c2880	3	6	-	-	-	-	-
gALL_v3_c28800	4	3	-	-	-	-	-
gALL_v3_c28801	2	3	-	-	-	-	-
gALL_v3_c28802	1	2	-	-	-	-	-
gALL_v3_c28804	0	3	-	-	-	-	-
gALL_v3_c28805	0	4	-	-	-	-	-
gALL_v3_c28806	2	3	-	-	-	-	-
gALL_v3_c28807	3	2	-	-	-	-	-
gALL_v3_c28808	2	1	-	-	-	-	-
gALL_v3_c28809	0	4	-	-	ref|XP_003699494.1|	PREDICTED: uncharacterized protein LOC100882757 [Megachile rotundata]	1,00E-119
gALL_v3_c2881	2	8	-	-	-	-	-
gALL_v3_c28810	0	4	-	-	-	-	-
gALL_v3_c28811	2	2	-	-	-	-	-
gALL_v3_c28812	1	4	-	-	-	-	-
gALL_v3_c28813	3	8	gnl|CDD|177779	PLN00185, PLN00185, 60S rib	gb|AGM32494.1|	60S ribosomal protein L4-like protein, partial [Coptotermes formosanus]	4,00E-55
gALL_v3_c28815	0	6	-	-	-	-	-
gALL_v3_c28816	2	1	-	-	-	-	-
gALL_v3_c28817	1	2	-	-	-	-	-
gALL_v3_c28818	2	1	-	-	-	-	-
gALL_v3_c2882	9	10	-	-	-	-	-
gALL_v3_c28820	2	2	gnl|CDD|190340	pfam02535, Zip, ZIP Zinc trans	ref|XP_782100.1|	PREDICTED: zinc transporter ZIP1-like [Strongylocentrotus purpuratus]	4,00E-32
gALL_v3_c28821	2	1	-	-	-	-	-
gALL_v3_c28822	1	2	-	-	-	-	-
gALL_v3_c28823	1	2	-	-	-	-	-
gALL_v3_c28824	1	3	-	-	gb|AGM32916.1|	hypothetical protein [Coptotermes formosanus]	6,00E-74
gALL_v3_c28825	2	2	gnl|CDD|193445	pfam12972, NAGLU_C, Alpha-	ref|XP_003397173.1|	PREDICTED: alpha-N-acetylglucosaminidase-like [Bombus terrestris]	2,00E-37
gALL_v3_c28826	3	4	-	-	-	-	-
gALL_v3_c28827	0	4	-	-	-	-	-
gALL_v3_c28828	2	3	-	-	-	-	-
gALL_v3_c28829	2	1	-	-	-	-	-
gALL_v3_c2883	5	14	gnl|CDD|189522	pfam00375, SDF, Sodium:dica	ref|XP_003702289.1|	PREDICTED: excitatory amino acid transporter-like [Megachile rotundata]	1,00E-95
gALL_v3_c28830	0	4	-	-	-	-	-
gALL_v3_c28831	1	3	gnl|CDD|187754	cd09293, AMN1, Antagonist o	ref|XP_001812041.1|	PREDICTED: similar to AGAP007807-PA [Tribolium castaneum]	6,00E-91
gALL_v3_c28832	2	3	-	-	-	-	-
gALL_v3_c28833	2	1	-	-	-	-	-
gALL_v3_c28834	0	3	-	-	-	-	-
gALL_v3_c28835	3	4	-	-	gb|EFA11632.1|	hypothetical protein TcasGA2_TC000010 [Tribolium castaneum]	2,00E-10
gALL_v3_c28836	1	2	-	-	-	-	-
gALL_v3_c28838	2	1	gnl|CDD|192023	pfam08393, DHC_N2, Dynein	ref|XP_001323796.1|	Dynein heavy chain family protein [Trichomonas vaginalis G3] gb|EAY11573.1| Dynein heavy chain family protein [Trichomonas vaginalis G3]	2,00E-68
gALL_v3_c28839	5	2	-	-	-	-	-
gALL_v3_c2884	1	7	gnl|CDD|162469	TIGR01657, P-ATPase-V, P-typ	ref|XP_003708338.1|	PREDICTED: probable cation-transporting ATPase 13A3-like [Megachile rotundata]	5,00E-62
gALL_v3_c28840	2	4	-	-	-	-	-
gALL_v3_c28841	3	1	-	-	-	-	-
gALL_v3_c28842	0	4	-	-	-	-	-
gALL_v3_c28843	1	4	-	-	-	-	-
gALL_v3_c28844	2	2	-	-	-	-	-
gALL_v3_c28845	2	1	-	-	-	-	-
gALL_v3_c28846	0	3	-	-	-	-	-
gALL_v3_c28847	1	2	-	-	-	-	-
gALL_v3_c28848	0	3	-	-	-	-	-
gALL_v3_c28849	1	2	-	-	ref|XP_002423687.1|	hypothetical protein Phum_PHUM079840 [Pediculus humanus corporis] gb|EEB10949.1| hypothetical protein Phum_PHUM079840 [Pediculus humanus corporis]	3,00E-13
gALL_v3_c2885	6	13	gnl|CDD|162465	TIGR01652, ATPase-Plipid, ph	ref|XP_001650984.1|	phospholipid-transporting atpase 1 (aminophospholipid flippase 1) [Aedes aegypti] gb|EAT48160.1| AAEL000767-PB [Aedes aegypti]	0
gALL_v3_c28850	1	2	-	-	-	-	-
gALL_v3_c28851	0	4	-	-	-	-	-
gALL_v3_c28852	2	3	-	-	ref|XP_006611247.1|	PREDICTED: uncharacterized protein LOC102673144 [Apis dorsata]	1,00E-130
[truncated: 7,246,578 more chars]
